# Supplementary material for: Struct2Graph: a graph attention network for structure based predictions of protein–protein interactions
Source: BMC Bioinformatics. 2022 Sep 10;23:370. doi: 10.1186/s12859-022-04910-9 (PMC9464414; doi:10.1186/s12859-022-04910-9)
Supplement: Supplementary file 1 — Additional file 1: List of important residues as predicted by Struct2Graph. [file 12859_2022_4910_MOESM1_ESM.pdf]

Struct2Graph: A graph attention network for structure based predictions of protein-protein interactions

Mayank Baranwal, Abram Magner, Jacob Saldinger, Emine S. Turali-Emre, Shivani Kozarekar, Paolo Elvati, J. Scott VanEpps, Nicholas A. Kotov, Angela Violi, and Alfred O. Hero

Supplementary Material

Details Of Important Residues

A demonstration of the potential of Struct2Graph to identify specific interaction sites was performed on two example cases (neither part of the training set) with well-described interacting residues from protein pairs in the literature. Specifically, we studied two different interaction types: 1) A protein with multiple ligands competing for the same binding area [1]; and 2) A dynamic protein-protein adhesion interaction [4]. The reported interacting residues in these complexes are compared with the Struct2Graph's highest probability residues (top 20%) using standard 2x2 confusion matrices. In aggregate (i.e., two case examples with a total of three interacting pairs), Struct2Graph identifies interacting residues with 30% sensitivity, 89% specificity, and 87% accuracy. It should be noted that these protein pair examples are not in the training set, and Struct2Graph identifies these residues through its knowledge selection process in a completely unsupervised manner. Besides, as noted above, the identified residues could be critical for ensuring correct protein folding conformation and therefore *indirectly* important for predicting binding, but not captured by traditional analysis that focuses only on the specific interacting residues identified in the literature. Detailed results for each example are described:

1) **HMGB1 and PSMα_1 compete for binding TLR4**: Phenol soluble modulins (PSMs), short, amphipathic, helical peptides [2], play a crucial role in *Staphylococcus aureus* virulence, one of the most common causes of human bacterial infections worldwide [5]. *S. aureus* has seven PSMs (PSM$\alpha_1-\alpha_4$, PSM$\beta_1-\beta_2$, and δ-toxin) which have multiple functions including, cytolysis, biofilm structuring, and inflammatory activation via cytokine release and chemotaxis. PSMs specifically trigger the release of high mobility group box-1 protein (HMGB1). Toll-like receptor-4 (TLR4) interacts with HMGB1 activating nuclear factor NF-κB and proinflammatory cytokines production [6]. However, *S. aureus* PSMs$\alpha_1-\alpha_3$ significantly inhibit HMGB1-mediated phosphorylation of NF-κB by competing with HMGB1 via interactions with the same residues of TLR4 domain [1]. As such, the specific interacting residues for these pairs HMGB1:TLR4 (2LY4 : 3FXI) and PSMα_1:TLR4 (5KHB : 3FXI) have been well described [1].

Struct2Graph identifies interacting residues of the HMGB1:TLR4 pair with 90% accuracy in which the top 9 predicted residues for TLR4 fall within the reported active cavity (residues rank 336-477). In addition, among the top 20% predicted residues of HMGB1 were the specific interacting residues Tyr^{16} and Lys^{68}. For the PSMα_1:TLR4 pair, Struct2Graph identifies interacting residues with 92% accuracy. Again the top predicted residues fall within the previously identified TLR4 active cavity (336-477). For PSMα_1, interacting residues Gly^2 and Val^{10} were correctly identified. While the overall sensitivity for detecting an interacting residue is $\sim 20\%$ for this example, Struct2Graph was able to predict that PSMα_1 interacts with TLR4 in the same area as the HMGB1 binding site. More specifically, the predicted binding sites for both on TLR4 have 94% concordance. Figures 1a and 1b shows the residues predicted to be essential and highlights how Struct2Graph predicts a similar site for both interactions.

2) **SdrG-Fibrinogen Adhesion**: Microbial attachment to host tissues is a crucial step in most bacterial infections. Gram-positive pathogens such as Staphylococci, Streptococci, and Enterococci contain multiple cell wall-anchored proteins that act as an adhesin to mediate bacterial attachment to host tissues. These adhesin mediating interactions have been termed MSCRAMMs (microbial surface components recognizing adhesive matrix molecules) [3]. SdrG is an MSCRAMM of *Staphylococcus epidermidis* that binds to the $B\beta$ chain of human fibrinogen (Fg) via dynamic "dock, lock, and latch" mechanism [4].

Struct2Graph was used to evaluate the interaction between SdrG (PDB:r19A) and a synthetic peptide with homologous sequence to its binding site in Fg (PDB:r17C). Interacting residues between SdrG and the synthetic Fg peptide homolog were predicted with 75% accuracy. Among the high probability residues identified in SdrG

were 9 exact matches to those in the literature [4]. This included, Pro^{337}, Ser^{338}, Leu^{340}, Phe^{344}, Gln^{425}, Ser^{437}, Tyr^{577}, Asp^{578}, and Asn^{579}. Figure 1c shows the residues predicted to be essential for the interaction.

These results show that Struct2Graph provides insight into key residues involved in the protein-protein interaction without any training data on the specific nature of these interactions. A complete summary of the residues identified by Struct2Graph for the preceding examples is included in the supporting information. Any high probability residues identified but not confirmed as directly interacting may have indirect effects through maintaining appropriate 3D conformation of the protein.

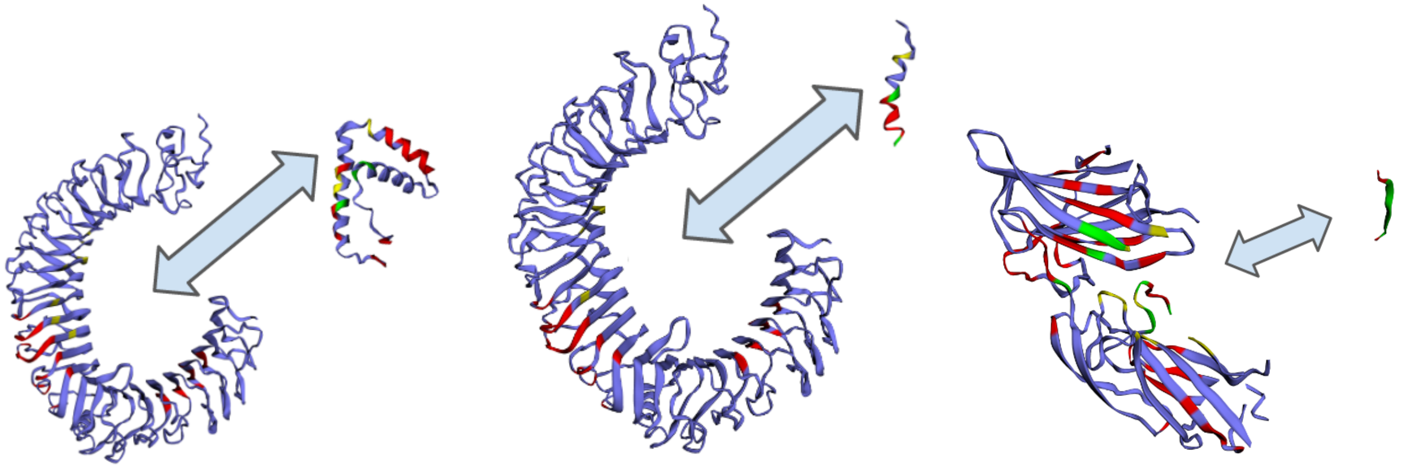

(a) (b) (c)

Figure 1: **Important residue prediction by Struct2Graph for three example scenarios.** (a) TLR4 with HMGB1, (b) TLR4 with PSMα, (c) SdrG and Fibrinogen adhesion. The different colored residues encode different information: (i) Red: Top-20% residues identified important by Struct2Graph, (ii) Yellow: Actual binding site not identified to be important by Struct2Graph, (iii) Green: True binding site overlapping with a residue identified important by Struct2Graph, (iV) Purple: neither important, nor actual interaction site. Recall that both HMGB1 and PSMα_1 are known to compete for the same binding sites on TLR4, and this gets reflected in the Struct2Graph predictive analysis as well.

Producing Mol2Vec-Like Embeddings Using Struct2Graph

Struct2Graph can be used to produce protein embeddings, similar to molecular embeddings produced by Mol2Vec. The embeddings are simply the output of the last layer of the graph convolutional layer averaged across the size of the graph. In the source code provided on Github (`https://github.com/baranwa2/Struct2Graph/blob/master/k-fold-CV.py`), the outputs $xs1$ and $xs2$ from the 'gnn' are averaged along the first-dimension to produce Mol2Vec-like embeddings.

References

[1] M. Chu, M. Zhou, C. Jiang, X. Chen, L. Guo, M. Zhang, Z. Chu, and Y. Wang. Staphylococcus aureus phenol-soluble modulins $\alpha1$–$\alpha3$ act as novel toll-like receptor (tlr) 4 antagonists to inhibit hmgb1/tlr4/nf-κb signaling pathway. *Frontiers in Immunology*, 9:862, 2018.

[2] C. Mehlin, C. M. Headley, and S. J. Klebanoff. An inflammatory polypeptide complex from staphylococcus epidermidis: isolation and characterization. *The Journal of experimental medicine*, 189(6):907–918, 1999.

[3] J. M. Patti and M. Höök. Microbial adhesins recognizing extracellular matrix macromolecules. *Current opinion in cell biology*, 6(5):752–758, 1994.

[4] K. Ponnuraj, M. G. Bowden, S. Davis, S. Gurusiddappa, D. Moore, D. Choe, Y. Xu, M. Hook, and S. V. Narayana. A "dock, lock, and latch" structural model for a staphylococcal adhesin binding to fibrinogen. *Cell*, 115(2):217–228, 2003.

[5] E. Tayeb-Fligelman, O. Tabachnikov, A. Moshe, O. Goldshmidt-Tran, M. R. Sawaya, N. Coquelle, J.-P. Colletier, and M. Landau. The cytotoxic staphylococcus aureus psmα3 reveals a cross-α amyloid-like fibril. *Science*, 355(6327):831–833, 2017.

[6] Y. Wang, H. Weng, J. F. Song, Y. H. Deng, S. Li, and H. B. Liu. Activation of the hmgb1-tlr4-nf-κb pathway may occur in patients with atopic eczema. *Molecular Medicine Reports*, 16(3):2714–2720, 2017.
