# Supplementary material for: Struct2Graph: a graph attention network for structure based predictions of protein–protein interactions
Source: BMC Bioinformatics. 2022 Sep 10;23:370. doi: 10.1186/s12859-022-04910-9 (PMC9464414; doi:10.1186/s12859-022-04910-9)
Supplement: Supplementary file 2 — Additional file 2: List of missense variants annotated in UniProtKB/Swiss-Prot human entries. [file 12859_2022_4910_MOESM2_ESM.pdf]


UniProt - Swiss-Prot Protein Knowledgebase

SIB Swiss Institute of Bioinformatics; Geneva, Switzerland

European Bioinformatics Institute (EBI); Hinxton, United Kingdom

Protein Information Resource (PIR); Washington DC, USA

Description: Index of human variants curated from literature reports
Name: humsavar.txt
Release: 2021_03 of 02-Jun-2021

This file lists all missense variants annotated in UniProtKB/Swiss-Prot human entries. It provides a variant classification which is intended for research purposes only, not for clinical and diagnostic use.

- The column 'Variant category' shows the classification of the variant using the American College of Medical Genetics and Genomics/Association for Molecular Pathology (ACMG/AMP) terminology (Richards et al. PubMed:25741868) into the following categories:

  LP/P = likely pathogenic or pathogenic
  LB/B = likely benign or benign
  US = uncertain significance

  These categories are assigned based on the variant annotation in the corresponding UniProtKB/Swiss-Prot entries that is curated from literature reports. The classification may change over time and must not be considered as a definitive statement about the pathogenic role of a variant.

- The column 'Disease name' shows the name of the disease or the disease sample in which variants have been found. Names are only provided for diseases catalogued in OMIM and for cancer samples.

Statistics for single amino acid variants:

LP/P: 31398
LB/B: 39584
US: 8763
Total: 79745

Main gene name	Swiss-Prot AC	FTId	AA change	Variant category	dbSNP	Disease name
A1BG	P04217	VAR_018369	p.His52Arg	LB/B	rs893184	-
A1BG	P04217	VAR_018370	p.His395Arg	LB/B	rs2241788	-
A1CF	Q9NQ94	VAR_052201	p.Val555Met	LB/B	rs9073	-
A1CF	Q9NQ94	VAR_059821	p.Ala558Ser	LB/B	rs11817448	-
A2M	P01023	VAR_000012	p.Arg704His	LB/B	rs1800434	-

A2M	P01023	VAR_000013	p.Cys972Tyr	LB/B	rs1800433	-
A2M	P01023	VAR_000014	p.Ile1000Val	LB/B	rs669	-
A2M	P01023	VAR_026820	p.Asn639Asp	LB/B	rs226405	-
A2M	P01023	VAR_026821	p.Leu815Gln	LB/B	rs3180392	-
A2ML1	A8K2U0	VAR_055463	p.Gly207Arg	LB/B	rs11047499	-
A2ML1	A8K2U0	VAR_055464	p.Cys970Tyr	LB/B	rs1558526	-
A2ML1	A8K2U0	VAR_055465	p.Thr1131Met	LB/B	rs7959680	-
A2ML1	A8K2U0	VAR_055466	p.Thr1412Ala	LB/B	rs7315591	-
A2ML1	A8K2U0	VAR_059083	p.Asp850Glu	LB/B	rs1860926	-
A2ML1	A8K2U0	VAR_059084	p.His1229Arg	LB/B	rs10219561	-
A2ML1	A8K2U0	VAR_071854	p.Arg1122Trp	LB/B	rs1860967	-
A2ML1	A8K2U0	VAR_071855	p.Met1257Val	LB/B	rs7308811	-
A2ML1	A8K2U0	VAR_071856	p.Thr1312Met	LB/B	rs201083574	-
A2ML1	A8K2U0	VAR_081010	p.Val296Ala	LB/B	rs192888493	-
A2ML1	A8K2U0	VAR_081011	p.Pro356Arg	LB/B	rs863224953	-
A2ML1	A8K2U0	VAR_081014	p.Arg1001Trp	LB/B	rs201725377	-
A2ML1	A8K2U0	VAR_081015	p.Ala1431Val	LB/B	rs863224955	-
A4GALT	Q9NPC4	VAR_014296	p.Met37Val	LB/B	rs11541159	-
A4GALT	Q9NPC4	VAR_014297	p.Met183Lys	LB/B	rs74315453	-
A4GALT	Q9NPC4	VAR_017508	p.Gly187Asp	LB/B	rs28940572	-
A4GALT	Q9NPC4	VAR_017509	p.Pro251Leu	LB/B	rs28940571	-
A4GALT	Q9NPC4	VAR_022320	p.Gln163Arg	LB/B	rs28915383	-
A4GALT	Q9NPC4	VAR_080910	p.Gln211Glu	LB/B	rs397514502	-
A4GNT	Q9UNA3	VAR_022096	p.Ala218Asp	LB/B	rs2246945	-
AAAS	Q9NRG9	VAR_012804	p.Gln15Lys	LP/P	rs121918549	Achalasia-addisonianism-alacrima syndrome (AAAS) [MIM:231550]
AAAS	Q9NRG9	VAR_012805	p.His160Arg	LP/P	rs1297831120	Achalasia-addisonianism-alacrima syndrome (AAAS) [MIM:231550]
AAAS	Q9NRG9	VAR_012806	p.Ser263Pro	LP/P	rs121918550	Achalasia-addisonianism-alacrima syndrome (AAAS) [MIM:231550]
AAAS	Q9NRG9	VAR_037060	p.Lys108Met	LB/B	rs13330	-
AACS	Q86V21	VAR_038303	p.Ile118Val	LB/B	rs11549081	-
AACS	Q86V21	VAR_060997	p.Ala470Val	LB/B	rs59883951	-
AADAC	P22760	VAR_014798	p.Val281Ile	LB/B	rs1803155	-
AADACL2	Q6P093	VAR_038140	p.Ala186Ser	LB/B	rs1972977	-
AADACL2	Q6P093	VAR_038141	p.Leu343Ile	LB/B	rs1052562	-
AADACL3	Q5VUY0	VAR_060665	p.Phe252Cys	LB/B	rs7513079	-
AADACL3	Q5VUY0	VAR_060666	p.Pro104Ser	LB/B	rs3010877	-
AADACL3	Q5VUY0	VAR_060667	p.Leu128Met	LB/B	rs3000859	-
AADACL3	Q5VUY0	VAR_060668	p.Arg186Trp	LB/B	rs17038445	-
AADACL3	Q5VUY0	VAR_060670	p.Met307Ile	LB/B	rs3000931	-
AADACL3	Q5VUY0	VAR_060671	p.Pro337Leu	LB/B	rs11121969	-
AADAT	Q8N5Z0	VAR_061005	p.Val243Ile	LB/B	rs56350236	-
AAGAB	Q6PD74	VAR_021533	p.Ile132Leu	LB/B	rs7173826	-
AAK1	Q2M2I8	VAR_031129	p.Lys509Gln	LB/B	rs6715776	-
AAK1	Q2M2I8	VAR_040348	p.Ile59Val	LB/B	rs34535244	-
AAK1	Q2M2I8	VAR_040349	p.Gln533His	LB/B	rs1263928487	-
AAK1	Q2M2I8	VAR_040350	p.Val603Ala	LB/B	rs56038532	-
AAK1	Q2M2I8	VAR_040351	p.Thr694Met	LB/B	rs55889248	-

AAK1 Q2M2I8 VAR_040352 p.Pro725Thr LB/B rs35285785 -
AAK1 Q2M2I8 VAR_040353 p.Pro771Arg LB/B rs34422616 -
AAK1 Q2M2I8 VAR_040354 p.Gly835Asp LB/B - -
AAMDC Q9H7C9 VAR_052696 p.Val92Met LB/B rs2186564 -
AAMP Q13685 VAR_037061 p.Ile250Val LB/B rs2305835 -
AANAT Q16613 VAR_048168 p.Arg15Cys LB/B rs34470791 -
AANAT Q16613 VAR_055086 p.Ala129Thr US rs28936679 -
AAR2 Q9Y312 VAR_048127 p.Pro124Thr LB/B rs6121183 -
AARD Q4LEZ3 VAR_043570 p.Gly96Arg LB/B rs16889283 -
AARS1 P49588 VAR_028204 p.Gly275Asp LB/B rs11537667 -
AARS1 P49588 VAR_063527 p.Arg329His LP/P rs267606621 Charcot-Marie-Tooth disease 2N (CMT2N) [MIM:613287]
AARS1 P49588 VAR_067084 p.Asn71Tyr LP/P rs387906792 Charcot-Marie-Tooth disease 2N (CMT2N) [MIM:613287]
AARS1 P49588 VAR_073293 p.Thr608Met US - -
AARS1 P49588 VAR_073719 p.Lys81Thr LP/P rs786205157 Developmental and epileptic encephalopathy 29 (DEE29) [MIM:616339]
AARS1 P49588 VAR_073720 p.Arg751Gly LP/P rs143370729 Developmental and epileptic encephalopathy 29 (DEE29) [MIM:616339]
AARS1 P49588 VAR_079703 p.Gly913Asp LP/P rs369774476 Developmental and epileptic encephalopathy 29 (DEE29) [MIM:616339]
AARS2 Q5JTZ9 VAR_027609 p.Ile339Val LB/B rs324136 -
AARS2 Q5JTZ9 VAR_027610 p.Ala484Asp LB/B rs495294 -
AARS2 Q5JTZ9 VAR_057357 p.Met850Val LB/B rs35783144 -
AARS2 Q5JTZ9 VAR_065956 p.Leu155Arg LP/P rs387907061 Combined oxidative phosphorylation deficiency 8 (COXPD8) [MIM:614096]
AARS2 Q5JTZ9 VAR_065957 p.Arg592Trp LP/P rs138119149 Combined oxidative phosphorylation deficiency 8 (COXPD8) [MIM:614096]
AARS2 Q5JTZ9 VAR_071837 p.Phe50Cys LP/P rs587777590 Leukoencephalopathy, progressive, with ovarian failure (LKENP) [MIM:615889]
AARS2 Q5JTZ9 VAR_071838 p.Ala77Val US rs375949891 -
AARS2 Q5JTZ9 VAR_071840 p.Arg199Cys US rs200105202 -
AARS2 Q5JTZ9 VAR_071841 p.Glu405Lys LP/P rs587777592 Leukoencephalopathy, progressive, with ovarian failure (LKENP) [MIM:615889]
AARS2 Q5JTZ9 VAR_071842 p.Val730Met LB/B rs35623954 -
AARS2 Q5JTZ9 VAR_071843 p.Gly965Arg LP/P rs543267101 Leukoencephalopathy, progressive, with ovarian failure (LKENP) [MIM:615889]
AASDH Q4L235 VAR_038309 p.Lys368Arg LB/B rs3796543 -
AASDH Q4L235 VAR_038310 p.Ala747Val LB/B rs3796544 -
AASDH Q4L235 VAR_038311 p.Val774Ile LB/B rs3796545 -
AASDH Q4L235 VAR_038312 p.Thr865Ala LB/B rs12498340 -
AASDH Q4L235 VAR_038313 p.Tyr1030Asp LB/B rs8340 -
AASDH Q4L235 VAR_061008 p.Ile61Val LB/B rs34543011 -
AASDH Q4L235 VAR_061009 p.Pro93Arg LB/B rs34228795 -
AATK Q6ZMQ8 VAR_027267 p.Thr118Met LB/B rs8082016 -
AATK Q6ZMQ8 VAR_032679 p.Ser81Phe US - An ovarian mucinous carcinoma sample
AATK Q6ZMQ8 VAR_032680 p.Leu97Val US - A lung adenocarcinoma sample
AATK Q6ZMQ8 VAR_032681 p.Met104Val US rs1337040042 An ovarian mucinous carcinoma sample
AATK Q6ZMQ8 VAR_032682 p.Gly703Cys LB/B rs7503604 -

AATK	Q6ZMQ8	VAR_032683	p.Ser815Arg	LB/B	rs56032966	-
AATK	Q6ZMQ8	VAR_032684	p.Ser923Leu	LB/B	rs56313973	-
AATK	Q6ZMQ8	VAR_032685	p.Glu1160Lys	LB/B	rs55793641	-
AATK	Q6ZMQ8	VAR_032686	p.Pro1192Ser	LB/B	rs55856613	-
AATK	Q6ZMQ8	VAR_032687	p.Phe1266Ser	LB/B	rs36000545	-
AATK	Q6ZMQ8	VAR_032688	p.Ala1332Thr	LB/B	rs55713566	-
ABAT	P80404	VAR_008883	p.Arg220Lys	LP/P	rs121434578	GABA transaminase deficiency (GABATD) [MIM:613163]
ABAT	P80404	VAR_018979	p.Gln56Arg	LB/B	rs1731017	-
ABCA1	O95477	VAR_009145	p.Val399Ala	LB/B	rs9282543	-
ABCA1	O95477	VAR_009146	p.Arg587Trp	LP/P	rs2853574	Tangier disease (TGD) [MIM:205400]
ABCA1	O95477	VAR_009147	p.Trp590Ser	LP/P	rs137854496	Tangier disease (TGD) [MIM:205400]
ABCA1	O95477	VAR_009148	p.Gln597Arg	LP/P	rs2853578	Tangier disease (TGD) [MIM:205400]
ABCA1	O95477	VAR_009150	p.Asn935Ser	LP/P	rs28937313	Tangier disease (TGD) [MIM:205400]
ABCA1	O95477	VAR_009151	p.Ala937Val	LP/P	rs137854495	Tangier disease (TGD) [MIM:205400]
ABCA1	O95477	VAR_009152	p.Asp1289Asn	LP/P	rs137854500	Tangier disease (TGD) [MIM:205400]
ABCA1	O95477	VAR_009153	p.Cys1477Arg	LP/P	rs137854494	Tangier disease (TGD) [MIM:205400]
ABCA1	O95477	VAR_009154	p.Ile1517Arg	LP/P	-	Tangier disease (TGD) [MIM:205400]
ABCA1	O95477	VAR_009155	p.Asn1800His	LP/P	rs146292819	Tangier disease (TGD) [MIM:205400]
ABCA1	O95477	VAR_012618	p.Arg219Lys	LB/B	rs2230806	-
ABCA1	O95477	VAR_012619	p.Arg230Cys	LB/B	rs9282541	-
ABCA1	O95477	VAR_012620	p.Ala255Thr	LP/P	rs758100110	Tangier disease (TGD) [MIM:205400]
ABCA1	O95477	VAR_012621	p.Val771Met	LB/B	rs2066718	-
ABCA1	O95477	VAR_012622	p.Thr774Pro	LB/B	rs35819696	-
ABCA1	O95477	VAR_012623	p.Lys776Asn	LB/B	rs138880920	-
ABCA1	O95477	VAR_012624	p.Val825Ile	LB/B	rs2066715	-
ABCA1	O95477	VAR_012625	p.Ile883Met	LB/B	rs2066714	-
ABCA1	O95477	VAR_012626	p.Thr929Ile	LP/P	-	Tangier disease (TGD) [MIM:205400]
ABCA1	O95477	VAR_012627	p.Ala1046Asp	LP/P	rs141021096	Tangier disease (TGD) [MIM:205400]
ABCA1	O95477	VAR_012628	p.Met1091Thr	LP/P	-	Hypoalphalipoproteinemia, primary, 1 (FHA1) [MIM:604091]
ABCA1	O95477	VAR_012629	p.Glu1172Asp	LB/B	rs33918808	-
ABCA1	O95477	VAR_012630	p.Ser1506Leu	LP/P	rs137854497	Tangier disease (TGD) [MIM:205400]
ABCA1	O95477	VAR_012631	p.Lys1587Arg	LB/B	rs2230808	-
ABCA1	O95477	VAR_012632	p.Asn1611Asp	LP/P	-	-
ABCA1	O95477	VAR_012633	p.Ser1731Cys	LB/B	rs760507032	-
ABCA1	O95477	VAR_012635	p.Arg2081Trp	LP/P	rs137854501	Tangier disease (TGD) [MIM:205400]
ABCA1	O95477	VAR_012636	p.Pro2150Leu	LP/P	rs369098049	Hypoalphalipoproteinemia, primary, 1 (FHA1) [MIM:604091]
ABCA1	O95477	VAR_012637	p.Leu2168Pro	LB/B	rs2853577	-
ABCA1	O95477	VAR_012638	p.Ile1555Thr	LB/B	rs1997618	-
ABCA1	O95477	VAR_012639	p.Leu1648Pro	LB/B	rs1883024	-
ABCA1	O95477	VAR_017016	p.Ser1181Phe	LB/B	rs76881554	-
ABCA1	O95477	VAR_017529	p.Pro85Leu	LP/P	rs145183203	Hypoalphalipoproteinemia, primary, 1 (FHA1) [MIM:604091]
ABCA1	O95477	VAR_017530	p.Asp1099Tyr	LP/P	rs28933692	Hypoalphalipoproteinemia, primary, 1 (FHA1) [MIM:604091]
ABCA1	O95477	VAR_035724	p.Glu210Asp	US	-	A colorectal cancer sample
ABCA1	O95477	VAR_035725	p.Asp917Tyr	US	-	A colorectal cancer sample

ABCA1 O95477 VAR_035726 p.Ala1407Thr US rs189206655 A colorectal cancer sample
ABCA1 O95477 VAR_035727 p.Ala2109Thr US - A colorectal cancer sample
ABCA1 O95477 VAR_037968 p.Asn935His LP/P rs28937314 Tangier disease (TGD) [MIM:205400]
ABCA1 O95477 VAR_037969 p.Val1054Ile LB/B rs13306072 -
ABCA1 O95477 VAR_037970 p.Arg1680Trp LP/P rs137854498 Tangier disease (TGD) [MIM:205400]
ABCA1 O95477 VAR_037971 p.Phe2009Ser LP/P rs137854499 Hypoalphalipoproteinemia, primary, 1 (FHA1) [MIM:604091]
ABCA1 O95477 VAR_062481 p.Pro248Ala LB/B rs142625938 -
ABCA1 O95477 VAR_062482 p.Glu284Lys LP/P - Tangier disease (TGD) [MIM:205400]
ABCA1 O95477 VAR_062483 p.Ser364Cys LB/B rs775035559 -
ABCA1 O95477 VAR_062484 p.Lys401Gln LB/B rs138487227 -
ABCA1 O95477 VAR_062485 p.Tyr482Cys LP/P - Tangier disease (TGD) [MIM:205400]
ABCA1 O95477 VAR_062486 p.Arg496Trp LB/B rs147675550 -
ABCA1 O95477 VAR_062487 p.Trp590Leu LP/P rs137854496 Tangier disease (TGD) [MIM:205400]
ABCA1 O95477 VAR_062488 p.Arg638Gln LB/B rs374190304 -
ABCA1 O95477 VAR_062489 p.Thr774Ser LB/B - -
ABCA1 O95477 VAR_062490 p.Glu815Gly LB/B rs145582736 -
ABCA1 O95477 VAR_062491 p.Trp840Arg LP/P rs1322998567 Tangier disease (TGD) [MIM:205400]
ABCA1 O95477 VAR_062492 p.Pro1065Ser LB/B - -
ABCA1 O95477 VAR_062493 p.Arg1068Cys LP/P rs745593394 Tangier disease (TGD) [MIM:205400]
ABCA1 O95477 VAR_062494 p.Gly1216Val LB/B rs562403512 -
ABCA1 O95477 VAR_062495 p.Arg1341Thr LB/B rs147743782 -
ABCA1 O95477 VAR_062496 p.Ser1376Gly LB/B rs145689805 -
ABCA1 O95477 VAR_062497 p.Leu1379Phe LP/P - Tangier disease (TGD) [MIM:205400]
ABCA1 O95477 VAR_062498 p.Arg1615Gln LB/B rs1251839800 -
ABCA1 O95477 VAR_062499 p.Ala1670Thr LB/B rs1203589782 -
ABCA1 O95477 VAR_062500 p.Arg1680Gln LB/B rs150125857 -
ABCA1 O95477 VAR_062501 p.Val1704Asp LP/P - Tangier disease (TGD) [MIM:205400]
ABCA1 O95477 VAR_062502 p.Arg1851Gln LP/P rs1055285452 Tangier disease (TGD) [MIM:205400]
ABCA1 O95477 VAR_062503 p.Arg1897Trp US rs760768125 Hypoalphalipoproteinemia, primary, 1 (FHA1) [MIM:604091]
ABCA1 O95477 VAR_062504 p.Arg1901Ser LP/P - Tangier disease (TGD) [MIM:205400]
ABCA1 O95477 VAR_062505 p.Arg1925Gln LB/B rs142688906 -
ABCA1 O95477 VAR_062506 p.Phe2163Ser US - -
ABCA1 O95477 VAR_062507 p.Gln2196His LP/P rs564764153 Tangier disease (TGD) [MIM:205400]
ABCA1 O95477 VAR_062508 p.Asp2243Glu LB/B rs34879708 -
ABCA1 O95477 VAR_062509 p.Val2244Ile LB/B rs144588452 -
ABCA10 Q8WWZ4 VAR_028384 p.Pro203Ser LB/B rs9909216 -
ABCA10 Q8WWZ4 VAR_028385 p.Ile287Val LB/B rs11657804 -
ABCA10 Q8WWZ4 VAR_028386 p.Met916Thr LB/B rs4968849 -
ABCA10 Q8WWZ4 VAR_055469 p.Arg1322Trp LB/B rs10491178 -
ABCA12 Q86UK0 VAR_019597 p.Ser459Thr LB/B rs113112835 -
ABCA12 Q86UK0 VAR_019598 p.Asn1380Ser LP/P rs28940269 Ichthyosis, congenital, autosomal recessive 4A (ARCI4A) [MIM:601277]
ABCA12 Q86UK0 VAR_019599 p.Gly1381Glu LP/P rs28940268 Ichthyosis, congenital, autosomal recessive 4A (ARCI4A) [MIM:601277]
ABCA12 Q86UK0 VAR_019600 p.Arg1514His LP/P rs28940270 Ichthyosis, congenital, autosomal recessive 4A (ARCI4A) [MIM:601277]

ABCA12 Q86UK0 VAR_019601 p.Glu1539Lys LP/P rs28940271 Ichthyosis, congenital, autosomal recessive 4A (ARCI4A) [MIM:601277]
ABCA12 Q86UK0 VAR_019602 p.Gly1651Ser LP/P rs28940568 Ichthyosis, congenital, autosomal recessive 4A (ARCI4A) [MIM:601277]
ABCA12 Q86UK0 VAR_027444 p.Glu550Gly LB/B rs16853149 -
ABCA12 Q86UK0 VAR_027445 p.Ser777Thr LB/B rs7560008 -
ABCA12 Q86UK0 VAR_027446 p.Gly1251Asp LB/B rs13414448 -
ABCA12 Q86UK0 VAR_027447 p.Arg1546Cys LB/B rs13401480 -
ABCA12 Q86UK0 VAR_027448 p.Glu2064Lys LB/B rs1213011 -
ABCA12 Q86UK0 VAR_027449 p.Asp2365Asn LB/B rs726070 -
ABCA12 Q86UK0 VAR_055473 p.Trp199Cys LB/B rs16853238 -
ABCA12 Q86UK0 VAR_055474 p.Asn237His LB/B rs11890512 -
ABCA12 Q86UK0 VAR_055475 p.Gln274Arg LB/B rs11890468 -
ABCA12 Q86UK0 VAR_055476 p.Arg287Gly LB/B rs11891778 -
ABCA12 Q86UK0 VAR_062663 p.Ala476Val US rs370640837 A pancreatic ductal adenocarcinoma sample
ABCA12 Q86UK0 VAR_067075 p.Thr345Pro LP/P rs1295935868 Ichthyosis, congenital, autosomal recessive 4A (ARCI4A) [MIM:601277]
ABCA12 Q86UK0 VAR_067076 p.Ser387Asn LP/P rs746315995 Ichthyosis, congenital, autosomal recessive 4B (ARCI4B) [MIM:242500]
ABCA12 Q86UK0 VAR_067077 p.Gly1136Asp LP/P - Ichthyosis, congenital, autosomal recessive 4A (ARCI4A) [MIM:601277]
ABCA12 Q86UK0 VAR_067078 p.Gly1179Arg LP/P rs267606622 Ichthyosis, congenital, autosomal recessive 4B (ARCI4B) [MIM:242500]
ABCA12 Q86UK0 VAR_067079 p.Trp1235Ser LP/P - Ichthyosis, congenital, autosomal recessive 4A (ARCI4A) [MIM:601277]
ABCA12 Q86UK0 VAR_067080 p.Ile1494Thr LP/P rs1263698595 Ichthyosis, congenital, autosomal recessive 4A (ARCI4A) [MIM:601277]
ABCA12 Q86UK0 VAR_067081 p.Gly1559Val LP/P rs1457513156 Ichthyosis, congenital, autosomal recessive 4A (ARCI4A) [MIM:601277]
ABCA12 Q86UK0 VAR_067082 p.Pro1798Leu LP/P rs181314573 Ichthyosis, congenital, autosomal recessive 4A (ARCI4A) [MIM:601277]
ABCA12 Q86UK0 VAR_067083 p.Thr1980Lys LP/P rs763858530 Ichthyosis, congenital, autosomal recessive 4A (ARCI4A) [MIM:601277]
ABCA13 Q86UQ4 VAR_055470 p.Thr1508Ile LB/B rs6583483 -
ABCA13 Q86UQ4 VAR_059087 p.Pro506Leu LB/B rs1880738 -
ABCA13 Q86UQ4 VAR_059088 p.Arg555His LB/B rs2361519 -
ABCA13 Q86UQ4 VAR_059089 p.Ile767Ser LB/B rs17712293 -
ABCA13 Q86UQ4 VAR_059090 p.Glu799Lys LB/B rs17547816 -
ABCA13 Q86UQ4 VAR_059091 p.Ile1434Val LB/B rs17132195 -
ABCA13 Q86UQ4 VAR_059092 p.Phe1540Leu LB/B rs17712299 -
ABCA13 Q86UQ4 VAR_059093 p.Ile1889Lys LB/B rs17132197 -
ABCA13 Q86UQ4 VAR_059094 p.Asn2033Asp LB/B rs17661364 -
ABCA13 Q86UQ4 VAR_059095 p.Ser2154Leu LB/B rs17092911 -
ABCA13 Q86UQ4 VAR_059096 p.Ala2178Glu LB/B rs1880736 -
ABCA13 Q86UQ4 VAR_059097 p.Leu2212Ser LB/B rs17132198 -
ABCA13 Q86UQ4 VAR_059098 p.Lys2436Arg LB/B rs17132206 -
ABCA13 Q86UQ4 VAR_059099 p.Ser2537Ala LB/B rs17132208 -
ABCA13 Q86UQ4 VAR_059100 p.Arg2674Trp LB/B rs2222648 -
ABCA13 Q86UQ4 VAR_059101 p.Ala3142Val LB/B rs3931814 -

ABCA13 Q86UQ4 VAR_059102 p.Tyr3851Phe LB/B rs17132289 -
ABCA13 Q86UQ4 VAR_059103 p.Asn4277Asp LB/B rs4917152 -
ABCA13 Q86UQ4 VAR_059104 p.Gln4302Arg LB/B rs4917153 -
ABCA13 Q86UQ4 VAR_059105 p.Pro4335Ala LB/B rs17132370 -
ABCA2 Q9BZC7 VAR_044526 p.Pro583His LB/B rs908828 -
ABCA2 Q9BZC7 VAR_044527 p.Phe674Val LB/B rs2090625 -
ABCA3 Q99758 VAR_023497 p.Leu101Pro LP/P rs121909182 Pulmonary surfactant metabolism dysfunction 3 (SMDP3) [MIM:610921]
ABCA3 Q99758 VAR_023498 p.Asn568Asp LP/P rs121909184 Pulmonary surfactant metabolism dysfunction 3 (SMDP3) [MIM:610921]
ABCA3 Q99758 VAR_023499 p.Leu1553Pro LP/P rs121909183 Pulmonary surfactant metabolism dysfunction 3 (SMDP3) [MIM:610921]
ABCA3 Q99758 VAR_023500 p.Gln1591Pro LP/P rs28936691 Pulmonary surfactant metabolism dysfunction 3 (SMDP3) [MIM:610921]
ABCA3 Q99758 VAR_025061 p.Asn140His LB/B rs45447801 -
ABCA3 Q99758 VAR_025062 p.Pro766Ser LB/B rs45592239 -
ABCA3 Q99758 VAR_035728 p.Leu290Met US - A breast cancer sample
ABCA3 Q99758 VAR_035729 p.Glu801Asp US - A breast cancer sample
ABCA3 Q99758 VAR_035730 p.His1069Gln US - A breast cancer sample
ABCA3 Q99758 VAR_084240 p.Arg43Leu US - Pulmonary surfactant metabolism dysfunction 3 (SMDP3) [MIM:610921]
ABCA3 Q99758 VAR_084241 p.Gln215Lys LP/P - Pulmonary surfactant metabolism dysfunction 3 (SMDP3) [MIM:610921]
ABCA3 Q99758 VAR_084242 p.Arg280Cys US - Pulmonary surfactant metabolism dysfunction 3 (SMDP3) [MIM:610921]
ABCA3 Q99758 VAR_084243 p.Arg288Lys US rs117603931 Pulmonary surfactant metabolism dysfunction 3 (SMDP3) [MIM:610921]
ABCA3 Q99758 VAR_084244 p.Glu292Val US rs149989682 Pulmonary surfactant metabolism dysfunction 3 (SMDP3) [MIM:610921]
ABCA3 Q99758 VAR_084245 p.Leu579Pro US - Pulmonary surfactant metabolism dysfunction 3 (SMDP3) [MIM:610921]
ABCA3 Q99758 VAR_084246 p.Arg605Gln US rs760006956 Pulmonary surfactant metabolism dysfunction 3 (SMDP3) [MIM:610921]
ABCA3 Q99758 VAR_084247 p.Glu690Lys LP/P - Pulmonary surfactant metabolism dysfunction 3 (SMDP3) [MIM:610921]
ABCA3 Q99758 VAR_084248 p.Leu982Pro LP/P rs1402761450 Pulmonary surfactant metabolism dysfunction 3 (SMDP3) [MIM:610921]
ABCA3 Q99758 VAR_084249 p.Asn1076Lys US - Pulmonary surfactant metabolism dysfunction 3 (SMDP3) [MIM:610921]
ABCA3 Q99758 VAR_084250 p.Thr1114Met LP/P rs891579143 Pulmonary surfactant metabolism dysfunction 3 (SMDP3) [MIM:610921]
ABCA3 Q99758 VAR_084251 p.Gly1221Ser LP/P - Pulmonary surfactant metabolism dysfunction 3 (SMDP3) [MIM:610921]
ABCA3 Q99758 VAR_084252 p.Pro1301Leu LP/P rs762699052 Pulmonary surfactant metabolism dysfunction 3 (SMDP3) [MIM:610921]
ABCA3 Q99758 VAR_084253 p.Gly1302Glu US - Pulmonary surfactant metabolism dysfunction 3 (SMDP3) [MIM:610921]
ABCA3 Q99758 VAR_084254 p.Lys1388Asn LP/P - Pulmonary surfactant metabolism dysfunction 3 (SMDP3) [MIM:610921]

ABCA3	Q99758	VAR_084255	p.Val1399Met	LP/P	rs763166660	Pulmonary surfactant metabolism dysfunction 3 (SMDP3) [MIM:610921]
ABCA3	Q99758	VAR_084257	p.Leu1580Pro	LP/P	-	Pulmonary surfactant metabolism dysfunction 3 (SMDP3) [MIM:610921]
ABCA4	P78363	VAR_008398	p.Arg18Trp	LP/P	rs121909205	Stargardt disease 1 (STGD1) [MIM:248200]
ABCA4	P78363	VAR_008399	p.Arg24His	LP/P	rs62645958	Stargardt disease 1 (STGD1) [MIM:248200]
ABCA4	P78363	VAR_008400	p.Cys54Tyr	LP/P	rs150774447	Stargardt disease 1 (STGD1) [MIM:248200]
ABCA4	P78363	VAR_008401	p.Gly65Glu	LP/P	rs62654395	Cone-rod dystrophy 3 (CORD3) [MIM:604116]
ABCA4	P78363	VAR_008401	p.Gly65Glu	LP/P	rs62654395	Stargardt disease 1 (STGD1) [MIM:248200]
ABCA4	P78363	VAR_008402	p.Cys75Gly	LP/P	rs61748526	Stargardt disease 1 (STGD1) [MIM:248200]
ABCA4	P78363	VAR_008403	p.Asn96Asp	LP/P	rs61748529	Stargardt disease 1 (STGD1) [MIM:248200]
ABCA4	P78363	VAR_008404	p.Asn96His	LP/P	rs61748529	Stargardt disease 1 (STGD1) [MIM:248200]
ABCA4	P78363	VAR_008405	p.Ala192Thr	LP/P	rs61748535	Stargardt disease 1 (STGD1) [MIM:248200]
ABCA4	P78363	VAR_008406	p.Arg212Cys	LP/P	rs61750200	Cone-rod dystrophy 3 (CORD3) [MIM:604116]
ABCA4	P78363	VAR_008406	p.Arg212Cys	LP/P	rs61750200	Stargardt disease 1 (STGD1) [MIM:248200]
ABCA4	P78363	VAR_008407	p.Asp249Gly	LP/P	rs62646865	Stargardt disease 1 (STGD1) [MIM:248200]
ABCA4	P78363	VAR_008408	p.Thr300Asn	LP/P	rs61748544	Stargardt disease 1 (STGD1) [MIM:248200]
ABCA4	P78363	VAR_008409	p.Ser336Cys	LP/P	rs61748547	Stargardt disease 1 (STGD1) [MIM:248200]
ABCA4	P78363	VAR_008410	p.Tyr340Asp	LP/P	rs61748548	Stargardt disease 1 (STGD1) [MIM:248200]
ABCA4	P78363	VAR_008411	p.Ala407Val	LP/P	rs61751264	Cone-rod dystrophy 3 (CORD3) [MIM:604116]
ABCA4	P78363	VAR_008411	p.Ala407Val	LP/P	rs61751264	Stargardt disease 1 (STGD1) [MIM:248200]
ABCA4	P78363	VAR_008412	p.Ser445Arg	LP/P	rs61748552	Stargardt disease 1 (STGD1) [MIM:248200]
ABCA4	P78363	VAR_008413	p.Glu471Lys	US	rs1800548	Macular degeneration, age-related, 2 (ARMD2) [MIM:153800]
ABCA4	P78363	VAR_008413	p.Glu471Lys	US	rs1800548	Stargardt disease 1 (STGD1) [MIM:248200]
ABCA4	P78363	VAR_008414	p.Asp523Glu	LP/P	rs62646868	Stargardt disease 1 (STGD1) [MIM:248200]
ABCA4	P78363	VAR_008415	p.Leu541Pro	LP/P	rs61751392	Cone-rod dystrophy 3 (CORD3) [MIM:604116]
ABCA4	P78363	VAR_008415	p.Leu541Pro	LP/P	rs61751392	Fundus flavimaculatus (FFM) [MIM:248200]
ABCA4	P78363	VAR_008415	p.Leu541Pro	LP/P	rs61751392	Stargardt disease 1 (STGD1) [MIM:248200]
ABCA4	P78363	VAR_008416	p.Arg572Pro	LP/P	rs61748559	Stargardt disease 1 (STGD1) [MIM:248200]
ABCA4	P78363	VAR_008417	p.Arg572Gln	LP/P	rs61748559	Stargardt disease 1 (STGD1) [MIM:248200]
ABCA4	P78363	VAR_008418	p.Arg602Trp	LP/P	rs61749409	Stargardt disease 1 (STGD1) [MIM:248200]
ABCA4	P78363	VAR_008419	p.Phe608Ile	LP/P	rs61752398	Stargardt disease 1 (STGD1) [MIM:248200]
ABCA4	P78363	VAR_008420	p.Val643Gly	LB/B	rs61754024	-
ABCA4	P78363	VAR_008421	p.Asp645Asn	LP/P	rs61749418	Stargardt disease 1 (STGD1) [MIM:248200]
ABCA4	P78363	VAR_008422	p.Gly818Glu	LP/P	rs61750202	Macular degeneration, age-related, 2 (ARMD2) [MIM:153800]
ABCA4	P78363	VAR_008422	p.Gly818Glu	LP/P	rs61750202	Stargardt disease 1 (STGD1) [MIM:248200]
ABCA4	P78363	VAR_008423	p.Trp821Arg	LP/P	rs61749433	Stargardt disease 1 (STGD1) [MIM:248200]
ABCA4	P78363	VAR_008424	p.Gly851Asp	LP/P	rs61749436	Stargardt disease 1 (STGD1) [MIM:248200]
ABCA4	P78363	VAR_008425	p.Gly863Ala	LP/P	rs76157638	Cone-rod dystrophy 3 (CORD3) [MIM:604116]
ABCA4	P78363	VAR_008425	p.Gly863Ala	LP/P	rs76157638	Fundus flavimaculatus (FFM) [MIM:248200]
ABCA4	P78363	VAR_008425	p.Gly863Ala	LP/P	rs76157638	Stargardt disease 1 (STGD1) [MIM:248200]
ABCA4	P78363	VAR_008426	p.Thr901Ala	LB/B	rs61754030	-
ABCA4	P78363	VAR_008427	p.Val931Met	LP/P	rs58331765	Stargardt disease 1 (STGD1) [MIM:248200]
ABCA4	P78363	VAR_008428	p.Arg943Gln	LB/B	rs1801581	-
ABCA4	P78363	VAR_008429	p.Gln957Arg	LP/P	rs61749448	Stargardt disease 1 (STGD1) [MIM:248200]
ABCA4	P78363	VAR_008430	p.Asn965Ser	LP/P	rs201471607	Stargardt disease 1 (STGD1) [MIM:248200]
ABCA4	P78363	VAR_008431	p.Gly978Cys	LP/P	rs61749452	Stargardt disease 1 (STGD1) [MIM:248200]

ABCA4	P78363	VAR_008432	p.Glu1036Lys	LP/P	rs61750061	Stargardt disease 1 (STGD1) [MIM:248200]
ABCA4	P78363	VAR_008433	p.Ala1038Val	LP/P	rs61751374	Cone-rod dystrophy 3 (CORD3) [MIM:604116]
ABCA4	P78363	VAR_008433	p.Ala1038Val	LP/P	rs61751374	Fundus flavimaculatus (FFM) [MIM:248200]
ABCA4	P78363	VAR_008433	p.Ala1038Val	LP/P	rs61751374	Stargardt disease 1 (STGD1) [MIM:248200]
ABCA4	P78363	VAR_008434	p.Ser1071Leu	LP/P	rs61750065	Stargardt disease 1 (STGD1) [MIM:248200]
ABCA4	P78363	VAR_008435	p.Val1072Ala	LP/P	-	Stargardt disease 1 (STGD1) [MIM:248200]
ABCA4	P78363	VAR_008436	p.Glu1087Lys	LP/P	rs61751398	Stargardt disease 1 (STGD1) [MIM:248200]
ABCA4	P78363	VAR_008437	p.Thr1112Asn	LP/P	rs61750122	Stargardt disease 1 (STGD1) [MIM:248200]
ABCA4	P78363	VAR_008438	p.Glu1122Lys	LP/P	rs61751399	Cone-rod dystrophy 3 (CORD3) [MIM:604116]
ABCA4	P78363	VAR_008438	p.Glu1122Lys	LP/P	rs61751399	Stargardt disease 1 (STGD1) [MIM:248200]
ABCA4	P78363	VAR_008439	p.Arg1129Leu	LP/P	rs1801269	Macular degeneration, age-related, 2 (ARMD2) [MIM:153800]
ABCA4	P78363	VAR_008439	p.Arg1129Leu	LP/P	rs1801269	Stargardt disease 1 (STGD1) [MIM:248200]
ABCA4	P78363	VAR_008440	p.Leu1201Arg	LP/P	rs61750126	Stargardt disease 1 (STGD1) [MIM:248200]
ABCA4	P78363	VAR_008441	p.Asp1204Asn	LP/P	rs61750127	Stargardt disease 1 (STGD1) [MIM:248200]
ABCA4	P78363	VAR_008442	p.Pro1314Thr	LB/B	rs61754041	-
ABCA4	P78363	VAR_008443	p.Pro1380Leu	LP/P	rs61750130	Stargardt disease 1 (STGD1) [MIM:248200]
ABCA4	P78363	VAR_008444	p.His1406Tyr	LP/P	rs61750133	Stargardt disease 1 (STGD1) [MIM:248200]
ABCA4	P78363	VAR_008445	p.Trp1408Leu	LP/P	rs61750134	Stargardt disease 1 (STGD1) [MIM:248200]
ABCA4	P78363	VAR_008446	p.Trp1408Arg	LP/P	rs61750135	Stargardt disease 1 (STGD1) [MIM:248200]
ABCA4	P78363	VAR_008447	p.Thr1428Met	LB/B	rs1800549	-
ABCA4	P78363	VAR_008448	p.Val1429Ala	LP/P	rs61752432	Stargardt disease 1 (STGD1) [MIM:248200]
ABCA4	P78363	VAR_008449	p.Val1433Ile	LP/P	rs56357060	Stargardt disease 1 (STGD1) [MIM:248200]
ABCA4	P78363	VAR_008450	p.Gly1439Asp	LP/P	rs61750140	Stargardt disease 1 (STGD1) [MIM:248200]
ABCA4	P78363	VAR_008451	p.Phe1440Ser	LP/P	rs61750141	Stargardt disease 1 (STGD1) [MIM:248200]
ABCA4	P78363	VAR_008452	p.Pro1486Leu	LP/P	rs61750145	Stargardt disease 1 (STGD1) [MIM:248200]
ABCA4	P78363	VAR_008453	p.Cys1488Arg	LP/P	rs61750146	Fundus flavimaculatus (FFM) [MIM:248200]
ABCA4	P78363	VAR_008453	p.Cys1488Arg	LP/P	rs61750146	Stargardt disease 1 (STGD1) [MIM:248200]
ABCA4	P78363	VAR_008454	p.Cys1490Tyr	LP/P	rs61751402	Cone-rod dystrophy 3 (CORD3) [MIM:604116]
ABCA4	P78363	VAR_008454	p.Cys1490Tyr	LP/P	rs61751402	Stargardt disease 1 (STGD1) [MIM:248200]
ABCA4	P78363	VAR_008455	p.Arg1517Ser	LP/P	rs1800550	Macular degeneration, age-related, 2 (ARMD2) [MIM:153800]
ABCA4	P78363	VAR_008456	p.Thr1526Met	LP/P	rs61750152	Stargardt disease 1 (STGD1) [MIM:248200]
ABCA4	P78363	VAR_008457	p.Asp1532Asn	LP/P	rs62642574	Stargardt disease 1 (STGD1) [MIM:248200]
ABCA4	P78363	VAR_008458	p.Ile1562Thr	LP/P	rs1762111	Cone-rod dystrophy 3 (CORD3) [MIM:604116]
ABCA4	P78363	VAR_008458	p.Ile1562Thr	LP/P	rs1762111	Fundus flavimaculatus (FFM) [MIM:248200]
ABCA4	P78363	VAR_008458	p.Ile1562Thr	LP/P	rs1762111	Macular degeneration, age-related, 2 (ARMD2) [MIM:153800]
ABCA4	P78363	VAR_008458	p.Ile1562Thr	LP/P	rs1762111	Stargardt disease 1 (STGD1) [MIM:248200]
ABCA4	P78363	VAR_008459	p.Gly1578Arg	LP/P	rs1800551	Macular degeneration, age-related, 2 (ARMD2) [MIM:153800]
ABCA4	P78363	VAR_008460	p.Leu1631Pro	LP/P	rs61750158	Stargardt disease 1 (STGD1) [MIM:248200]
ABCA4	P78363	VAR_008461	p.Arg1640Trp	LP/P	rs61751404	Cone-rod dystrophy 3 (CORD3) [MIM:604116]
ABCA4	P78363	VAR_008461	p.Arg1640Trp	LP/P	rs61751404	Stargardt disease 1 (STGD1) [MIM:248200]
ABCA4	P78363	VAR_008462	p.Tyr1652Asp	LP/P	rs61750560	Stargardt disease 1 (STGD1) [MIM:248200]
ABCA4	P78363	VAR_008463	p.Ser1696Asn	LP/P	rs61750564	Stargardt disease 1 (STGD1) [MIM:248200]
ABCA4	P78363	VAR_008464	p.Gln1703Lys	LP/P	-	Stargardt disease 1 (STGD1) [MIM:248200]
ABCA4	P78363	VAR_008465	p.Leu1729Pro	LP/P	rs61750567	Stargardt disease 1 (STGD1) [MIM:248200]
ABCA4	P78363	VAR_008466	p.Ala1794Asp	LP/P	rs61751406	Stargardt disease 1 (STGD1) [MIM:248200]

ABCA4	P78363	VAR_008467	p.Arg1820Pro	LP/P	rs62646875	Stargardt disease 1 (STGD1) [MIM:248200]
ABCA4	P78363	VAR_008468	p.His1838Tyr	LP/P	rs62642562	Stargardt disease 1 (STGD1) [MIM:248200]
ABCA4	P78363	VAR_008469	p.Arg1843Trp	LP/P	rs62642576	Stargardt disease 1 (STGD1) [MIM:248200]
ABCA4	P78363	VAR_008470	p.Asn1868Ile	LB/B	rs1801466	-
ABCA4	P78363	VAR_008471	p.Gly1886Glu	LP/P	rs62642579	Stargardt disease 1 (STGD1) [MIM:248200]
ABCA4	P78363	VAR_008473	p.Arg1898His	LP/P	rs1800552	Macular degeneration, age-related, 2 (ARMD2) [MIM:153800]
ABCA4	P78363	VAR_008473	p.Arg1898His	LP/P	rs1800552	Stargardt disease 1 (STGD1) [MIM:248200]
ABCA4	P78363	VAR_008474	p.Pro1948Leu	LB/B	rs56142141	-
ABCA4	P78363	VAR_008475	p.Gly1961Glu	LP/P	rs1800553	Fundus flavimaculatus (FFM) [MIM:248200]
ABCA4	P78363	VAR_008475	p.Gly1961Glu	LP/P	rs1800553	Stargardt disease 1 (STGD1) [MIM:248200]
ABCA4	P78363	VAR_008476	p.Leu1970Phe	LP/P	rs28938473	Fundus flavimaculatus (FFM) [MIM:248200]
ABCA4	P78363	VAR_008476	p.Leu1970Phe	LP/P	rs28938473	Macular degeneration, age-related, 2 (ARMD2) [MIM:153800]
ABCA4	P78363	VAR_008477	p.Gly1977Ser	LP/P	rs61750639	Macular degeneration, age-related, 2 (ARMD2) [MIM:153800]
ABCA4	P78363	VAR_008477	p.Gly1977Ser	LP/P	rs61750639	Stargardt disease 1 (STGD1) [MIM:248200]
ABCA4	P78363	VAR_008478	p.Leu2027Phe	LP/P	rs61751408	Fundus flavimaculatus (FFM) [MIM:248200]
ABCA4	P78363	VAR_008478	p.Leu2027Phe	LP/P	rs61751408	Stargardt disease 1 (STGD1) [MIM:248200]
ABCA4	P78363	VAR_008480	p.Arg2030Gln	LP/P	rs61750641	Fundus flavimaculatus (FFM) [MIM:248200]
ABCA4	P78363	VAR_008480	p.Arg2030Gln	LP/P	rs61750641	Stargardt disease 1 (STGD1) [MIM:248200]
ABCA4	P78363	VAR_008481	p.Val2050Leu	LP/P	rs41292677	Stargardt disease 1 (STGD1) [MIM:248200]
ABCA4	P78363	VAR_008482	p.Arg2077Trp	LP/P	rs61750645	Stargardt disease 1 (STGD1) [MIM:248200]
ABCA4	P78363	VAR_008483	p.Glu2096Lys	LP/P	rs61750646	Stargardt disease 1 (STGD1) [MIM:248200]
ABCA4	P78363	VAR_008484	p.Arg2106Cys	LP/P	rs61750648	Fundus flavimaculatus (FFM) [MIM:248200]
ABCA4	P78363	VAR_008484	p.Arg2106Cys	LP/P	rs61750648	Stargardt disease 1 (STGD1) [MIM:248200]
ABCA4	P78363	VAR_008485	p.Arg2107His	LP/P	rs62642564	Stargardt disease 1 (STGD1) [MIM:248200]
ABCA4	P78363	VAR_008486	p.His2128Arg	LP/P	rs61750651	Stargardt disease 1 (STGD1) [MIM:248200]
ABCA4	P78363	VAR_008487	p.Glu2131Lys	LP/P	rs61750652	Stargardt disease 1 (STGD1) [MIM:248200]
ABCA4	P78363	VAR_008488	p.Arg2139Trp	LP/P	rs61750653	Stargardt disease 1 (STGD1) [MIM:248200]
ABCA4	P78363	VAR_008489	p.Cys2150Tyr	LP/P	rs61751384	Cone-rod dystrophy 3 (CORD3) [MIM:604116]
ABCA4	P78363	VAR_008489	p.Cys2150Tyr	LP/P	rs61751384	Stargardt disease 1 (STGD1) [MIM:248200]
ABCA4	P78363	VAR_008490	p.Lys2160Arg	LP/P	rs281865405	Stargardt disease 1 (STGD1) [MIM:248200]
ABCA4	P78363	VAR_008491	p.Asp2177Asn	LB/B	rs1800555	-
ABCA4	P78363	VAR_008492	p.Ala60Val	LP/P	rs55732384	Stargardt disease 1 (STGD1) [MIM:248200]
ABCA4	P78363	VAR_008493	p.Asp846His	LB/B	rs61754027	-
ABCA4	P78363	VAR_008494	p.Ile1846Thr	LB/B	rs61750575	-
ABCA4	P78363	VAR_008495	p.Arg2038Trp	LP/P	rs61750643	Stargardt disease 1 (STGD1) [MIM:248200]
ABCA4	P78363	VAR_009157	p.Ser2255Ile	LB/B	rs6666652	-
ABCA4	P78363	VAR_012493	p.Leu11Pro	LP/P	rs62645946	Fundus flavimaculatus (FFM) [MIM:248200]
ABCA4	P78363	VAR_012495	p.Asn58Lys	LP/P	rs61748524	Stargardt disease 1 (STGD1) [MIM:248200]
ABCA4	P78363	VAR_012496	p.Ala60Glu	LP/P	-	Stargardt disease 1 (STGD1) [MIM:248200]
ABCA4	P78363	VAR_012497	p.Ala60Thr	LP/P	rs61751411	Stargardt disease 1 (STGD1) [MIM:248200]
ABCA4	P78363	VAR_012498	p.Pro68Leu	LP/P	rs62654397	Stargardt disease 1 (STGD1) [MIM:248200]
ABCA4	P78363	VAR_012499	p.Pro68Arg	LP/P	rs62654397	Stargardt disease 1 (STGD1) [MIM:248200]
ABCA4	P78363	VAR_012500	p.Gly72Arg	LP/P	rs61751412	Stargardt disease 1 (STGD1) [MIM:248200]
ABCA4	P78363	VAR_012501	p.Val77Glu	LP/P	rs61748527	Stargardt disease 1 (STGD1) [MIM:248200]
ABCA4	P78363	VAR_012502	p.Ser100Pro	LP/P	rs61748530	Stargardt disease 1 (STGD1) [MIM:248200]
ABCA4	P78363	VAR_012503	p.Arg152Gln	LB/B	rs62646862	-

ABCA4	P78363	VAR_012504	p.Ile156Val	LP/P	rs62646863	Stargardt disease 1 (STGD1) [MIM:248200]
ABCA4	P78363	VAR_012505	p.Gln190His	LP/P	rs281865397	Stargardt disease 1 (STGD1) [MIM:248200]
ABCA4	P78363	VAR_012506	p.Ser206Arg	LP/P	rs61748536	Stargardt disease 1 (STGD1) [MIM:248200]
ABCA4	P78363	VAR_012507	p.Arg212His	LB/B	rs6657239	-
ABCA4	P78363	VAR_012508	p.Arg220Cys	LP/P	rs61748538	Stargardt disease 1 (STGD1) [MIM:248200]
ABCA4	P78363	VAR_012509	p.Cys230Ser	LP/P	rs1057518767	Stargardt disease 1 (STGD1) [MIM:248200]
ABCA4	P78363	VAR_012510	p.Leu244Pro	LP/P	rs62646864	Stargardt disease 1 (STGD1) [MIM:248200]
ABCA4	P78363	VAR_012511	p.Asn247Ser	LP/P	rs62645950	Stargardt disease 1 (STGD1) [MIM:248200]
ABCA4	P78363	VAR_012512	p.Pro309Arg	LP/P	rs61748545	Stargardt disease 1 (STGD1) [MIM:248200]
ABCA4	P78363	VAR_012513	p.Glu328Val	LP/P	rs61751419	Stargardt disease 1 (STGD1) [MIM:248200]
ABCA4	P78363	VAR_012514	p.Arg333Trp	LP/P	rs61748546	Stargardt disease 1 (STGD1) [MIM:248200]
ABCA4	P78363	VAR_012515	p.Trp339Gly	LP/P	rs61751420	Fundus flavimaculatus (FFM) [MIM:248200]
ABCA4	P78363	VAR_012516	p.Asn380Lys	LP/P	rs61748549	Stargardt disease 1 (STGD1) [MIM:248200]
ABCA4	P78363	VAR_012517	p.His423Arg	LB/B	rs3112831	-
ABCA4	P78363	VAR_012518	p.Phe525Cys	LP/P	-	Stargardt disease 1 (STGD1) [MIM:248200]
ABCA4	P78363	VAR_012519	p.Arg537Cys	LP/P	rs61748556	Stargardt disease 1 (STGD1) [MIM:248200]
ABCA4	P78363	VAR_012520	p.Ala549Pro	LP/P	rs61748557	Stargardt disease 1 (STGD1) [MIM:248200]
ABCA4	P78363	VAR_012521	p.Gly550Arg	LP/P	rs61748558	Stargardt disease 1 (STGD1) [MIM:248200]
ABCA4	P78363	VAR_012522	p.Val552Ile	LB/B	rs145525174	-
ABCA4	P78363	VAR_012523	p.Arg602Gln	LP/P	rs61749410	Stargardt disease 1 (STGD1) [MIM:248200]
ABCA4	P78363	VAR_012524	p.Gly607Arg	LP/P	rs61749412	Stargardt disease 1 (STGD1) [MIM:248200]
ABCA4	P78363	VAR_012525	p.Gly607Trp	LP/P	rs61749412	Stargardt disease 1 (STGD1) [MIM:248200]
ABCA4	P78363	VAR_012526	p.Gln635Lys	LP/P	rs61749414	Stargardt disease 1 (STGD1) [MIM:248200]
ABCA4	P78363	VAR_012527	p.Gln636His	LP/P	rs61752400	Stargardt disease 1 (STGD1) [MIM:248200]
ABCA4	P78363	VAR_012528	p.Val643Met	LP/P	rs61749417	Stargardt disease 1 (STGD1) [MIM:248200]
ABCA4	P78363	VAR_012529	p.Arg653Cys	LP/P	rs61749420	Stargardt disease 1 (STGD1) [MIM:248200]
ABCA4	P78363	VAR_012530	p.Leu686Ser	LP/P	rs61752402	Stargardt disease 1 (STGD1) [MIM:248200]
ABCA4	P78363	VAR_012531	p.Thr716Met	LP/P	rs61749426	Stargardt disease 1 (STGD1) [MIM:248200]
ABCA4	P78363	VAR_012532	p.Cys764Tyr	LP/P	rs61749428	Stargardt disease 1 (STGD1) [MIM:248200]
ABCA4	P78363	VAR_012533	p.Ser765Arg	LP/P	rs61752404	Stargardt disease 1 (STGD1) [MIM:248200]
ABCA4	P78363	VAR_012534	p.Ser765Asn	LP/P	rs61749429	Stargardt disease 1 (STGD1) [MIM:248200]
ABCA4	P78363	VAR_012535	p.Val767Asp	LP/P	rs61751395	Stargardt disease 1 (STGD1) [MIM:248200]
ABCA4	P78363	VAR_012536	p.Leu797Pro	LP/P	rs61749432	Stargardt disease 1 (STGD1) [MIM:248200]
ABCA4	P78363	VAR_012537	p.Ile824Thr	LP/P	-	Stargardt disease 1 (STGD1) [MIM:248200]
ABCA4	P78363	VAR_012538	p.Val849Ala	LP/P	rs61749435	Stargardt disease 1 (STGD1) [MIM:248200]
ABCA4	P78363	VAR_012539	p.Ala854Thr	LP/P	rs61749437	Stargardt disease 1 (STGD1) [MIM:248200]
ABCA4	P78363	VAR_012541	p.Phe873Leu	LP/P	rs62642570	Stargardt disease 1 (STGD1) [MIM:248200]
ABCA4	P78363	VAR_012542	p.Thr897Ile	LP/P	rs61749440	Stargardt disease 1 (STGD1) [MIM:248200]
ABCA4	P78363	VAR_012543	p.His914Arg	LB/B	-	-
ABCA4	P78363	VAR_012544	p.Val935Ala	LP/P	rs61749444	Stargardt disease 1 (STGD1) [MIM:248200]
ABCA4	P78363	VAR_012545	p.Arg943Trp	LP/P	rs61749446	Fundus flavimaculatus (FFM) [MIM:248200]
ABCA4	P78363	VAR_012545	p.Arg943Trp	LP/P	rs61749446	Stargardt disease 1 (STGD1) [MIM:248200]
ABCA4	P78363	VAR_012546	p.Thr959Ile	LP/P	rs61752409	Stargardt disease 1 (STGD1) [MIM:248200]
ABCA4	P78363	VAR_012547	p.Thr971Asn	LP/P	rs61749450	Stargardt disease 1 (STGD1) [MIM:248200]
ABCA4	P78363	VAR_012548	p.Thr972Asn	US	rs61749451	Stargardt disease 1 (STGD1) [MIM:248200]
ABCA4	P78363	VAR_012549	p.Ser974Pro	LP/P	rs281865400	Stargardt disease 1 (STGD1) [MIM:248200]
ABCA4	P78363	VAR_012550	p.Val989Ala	LP/P	rs61749454	Stargardt disease 1 (STGD1) [MIM:248200]
ABCA4	P78363	VAR_012551	p.Gly991Arg	LP/P	rs61749455	Fundus flavimaculatus (FFM) [MIM:248200]
ABCA4	P78363	VAR_012552	p.Leu1014Arg	LP/P	rs61749456	Stargardt disease 1 (STGD1) [MIM:248200]

ABCA4	P78363	VAR_012553	p.Thr1019Ala	LP/P	rs61749457	Stargardt disease 1 (STGD1) [MIM:248200]
ABCA4	P78363	VAR_012554	p.Thr1019Met	LP/P	rs201855602	Stargardt disease 1 (STGD1) [MIM:248200]
ABCA4	P78363	VAR_012555	p.Glu1022Lys	LP/P	rs61749459	Stargardt disease 1 (STGD1) [MIM:248200]
ABCA4	P78363	VAR_012556	p.Lys1031Glu	LP/P	rs61750060	Stargardt disease 1 (STGD1) [MIM:248200]
ABCA4	P78363	VAR_012557	p.Arg1055Trp	LP/P	rs61752412	Stargardt disease 1 (STGD1) [MIM:248200]
ABCA4	P78363	VAR_012558	p.Ser1063Pro	LP/P	rs61752413	Stargardt disease 1 (STGD1) [MIM:248200]
ABCA4	P78363	VAR_012559	p.Glu1087Asp	LP/P	rs61752416	Stargardt disease 1 (STGD1) [MIM:248200]
ABCA4	P78363	VAR_012560	p.Gly1091Glu	LP/P	rs61752417	Fundus flavimaculatus (FFM) [MIM:248200]
ABCA4	P78363	VAR_012561	p.Arg1097Cys	LP/P	-	Stargardt disease 1 (STGD1) [MIM:248200]
ABCA4	P78363	VAR_012562	p.Arg1108Cys	LP/P	rs61750120	Fundus flavimaculatus (FFM) [MIM:248200]
ABCA4	P78363	VAR_012562	p.Arg1108Cys	LP/P	rs61750120	Stargardt disease 1 (STGD1) [MIM:248200]
ABCA4	P78363	VAR_012563	p.Arg1108His	LP/P	rs61750121	Stargardt disease 1 (STGD1) [MIM:248200]
ABCA4	P78363	VAR_012564	p.Arg1108Leu	LP/P	rs61750121	Stargardt disease 1 (STGD1) [MIM:248200]
ABCA4	P78363	VAR_012565	p.Arg1129Cys	LP/P	rs779426136	Stargardt disease 1 (STGD1) [MIM:248200]
ABCA4	P78363	VAR_012566	p.Lys1148Thr	LB/B	-	-
ABCA4	P78363	VAR_012567	p.Leu1250Pro	LP/P	rs61750128	Stargardt disease 1 (STGD1) [MIM:248200]
ABCA4	P78363	VAR_012568	p.Thr1253Met	US	rs61752424	Fundus flavimaculatus (FFM) [MIM:248200]
ABCA4	P78363	VAR_012569	p.Arg1300Gln	LP/P	rs61750129	Stargardt disease 1 (STGD1) [MIM:248200]
ABCA4	P78363	VAR_012570	p.Leu1388Pro	LP/P	rs61750131	Stargardt disease 1 (STGD1) [MIM:248200]
ABCA4	P78363	VAR_012571	p.Glu1399Lys	LP/P	rs62642573	Stargardt disease 1 (STGD1) [MIM:248200]
ABCA4	P78363	VAR_012572	p.Leu1430Pro	LP/P	-	Stargardt disease 1 (STGD1) [MIM:248200]
ABCA4	P78363	VAR_012573	p.Phe1440Val	LP/P	rs61752433	Stargardt disease 1 (STGD1) [MIM:248200]
ABCA4	P78363	VAR_012574	p.Arg1443His	LP/P	rs61750142	Stargardt disease 1 (STGD1) [MIM:248200]
ABCA4	P78363	VAR_012575	p.Cys1488Phe	LP/P	rs61750147	Stargardt disease 1 (STGD1) [MIM:248200]
ABCA4	P78363	VAR_012576	p.Cys1488Tyr	LP/P	rs61750147	Stargardt disease 1 (STGD1) [MIM:248200]
ABCA4	P78363	VAR_012577	p.Gly1508Cys	LP/P	-	Fundus flavimaculatus (FFM) [MIM:248200]
ABCA4	P78363	VAR_012578	p.Gln1513Arg	LP/P	rs281865402	Stargardt disease 1 (STGD1) [MIM:248200]
ABCA4	P78363	VAR_012579	p.Leu1525Pro	LP/P	rs61750151	Stargardt disease 1 (STGD1) [MIM:248200]
ABCA4	P78363	VAR_012580	p.Thr1537Met	LP/P	rs62642575	Stargardt disease 1 (STGD1) [MIM:248200]
ABCA4	P78363	VAR_012581	p.Ala1598Asp	LP/P	rs61750155	Cone-rod dystrophy 3 (CORD3) [MIM:604116]
ABCA4	P78363	VAR_012582	p.Ala1637Thr	LB/B	rs61754056	-
ABCA4	P78363	VAR_012583	p.Arg1640Gln	LP/P	rs61751403	Cone-rod dystrophy 3 (CORD3) [MIM:604116]
ABCA4	P78363	VAR_012583	p.Arg1640Gln	LP/P	rs61751403	Fundus flavimaculatus (FFM) [MIM:248200]
ABCA4	P78363	VAR_012583	p.Arg1640Gln	LP/P	rs61751403	Stargardt disease 1 (STGD1) [MIM:248200]
ABCA4	P78363	VAR_012585	p.Ser1689Pro	LP/P	rs61753020	Stargardt disease 1 (STGD1) [MIM:248200]
ABCA4	P78363	VAR_012586	p.Val1693Ile	LP/P	rs61750563	Stargardt disease 1 (STGD1) [MIM:248200]
ABCA4	P78363	VAR_012587	p.Arg1705Leu	LP/P	rs61753021	Stargardt disease 1 (STGD1) [MIM:248200]
ABCA4	P78363	VAR_012588	p.Met1733Thr	LP/P	rs765563320	Stargardt disease 1 (STGD1) [MIM:248200]
ABCA4	P78363	VAR_012589	p.Ser1736Pro	LP/P	rs61750568	Stargardt disease 1 (STGD1) [MIM:248200]
ABCA4	P78363	VAR_012590	p.Gly1748Arg	LP/P	rs61753025	Stargardt disease 1 (STGD1) [MIM:248200]
ABCA4	P78363	VAR_012592	p.Leu1763Pro	LP/P	rs61753028	Stargardt disease 1 (STGD1) [MIM:248200]
ABCA4	P78363	VAR_012593	p.Pro1776Leu	LP/P	rs281865404	Stargardt disease 1 (STGD1) [MIM:248200]
ABCA4	P78363	VAR_012594	p.Pro1780Ala	LP/P	rs121909207	Stargardt disease 1 (STGD1) [MIM:248200]
ABCA4	P78363	VAR_012595	p.Asn1799Asp	LP/P	rs61750574	Stargardt disease 1 (STGD1) [MIM:248200]
ABCA4	P78363	VAR_012596	p.Asn1805Asp	LP/P	rs61753029	Stargardt disease 1 (STGD1) [MIM:248200]
ABCA4	P78363	VAR_012597	p.Glu1817Asp	LB/B	-	-
ABCA4	P78363	VAR_012598	p.Val1884Glu	LP/P	rs62642578	Stargardt disease 1 (STGD1) [MIM:248200]
ABCA4	P78363	VAR_012599	p.Glu1885Lys	LP/P	rs62642563	Stargardt disease 1 (STGD1) [MIM:248200]
ABCA4	P78363	VAR_012600	p.Val1896Asp	LP/P	rs61750636	Stargardt disease 1 (STGD1) [MIM:248200]

ABCA4	P78363	VAR_012601	p.Val1921Met	LB/B	rs61753032	-
ABCA4	P78363	VAR_012602	p.Leu1940Pro	LP/P	rs61753033	Fundus flavimaculatus (FFM) [MIM:248200]
ABCA4	P78363	VAR_012602	p.Leu1940Pro	LP/P	rs61753033	Stargardt disease 1 (STGD1) [MIM:248200]
ABCA4	P78363	VAR_012603	p.Leu1971Arg	LP/P	rs61753034	Fundus flavimaculatus (FFM) [MIM:248200]
ABCA4	P78363	VAR_012604	p.Gly1975Arg	LP/P	rs61753036	Stargardt disease 1 (STGD1) [MIM:248200]
ABCA4	P78363	VAR_012605	p.Leu2035Pro	LP/P	rs61750642	Stargardt disease 1 (STGD1) [MIM:248200]
ABCA4	P78363	VAR_012606	p.Gly2059Ala	LB/B	-	-
ABCA4	P78363	VAR_012607	p.Leu2060Arg	LP/P	rs61753039	Cone-rod dystrophy 3 (CORD3) [MIM:604116]
ABCA4	P78363	VAR_012608	p.Tyr2071Phe	LP/P	-	Stargardt disease 1 (STGD1) [MIM:248200]
ABCA4	P78363	VAR_012609	p.Arg2077Gly	LP/P	rs61750645	Stargardt disease 1 (STGD1) [MIM:248200]
ABCA4	P78363	VAR_012610	p.Arg2107Cys	LP/P	rs2297669	Stargardt disease 1 (STGD1) [MIM:248200]
ABCA4	P78363	VAR_012611	p.Gly2146Asp	LP/P	rs61753044	Cone-rod dystrophy 3 (CORD3) [MIM:604116]
ABCA4	P78363	VAR_012612	p.Arg2149Leu	LP/P	rs61750655	Stargardt disease 1 (STGD1) [MIM:248200]
ABCA4	P78363	VAR_012613	p.Cys2150Arg	LP/P	rs61750656	Stargardt disease 1 (STGD1) [MIM:248200]
ABCA4	P78363	VAR_012614	p.Ala2216Val	LB/B	rs886044763	-
ABCA4	P78363	VAR_012615	p.Leu2229Pro	LP/P	rs61750659	Stargardt disease 1 (STGD1) [MIM:248200]
ABCA4	P78363	VAR_012616	p.Leu2241Val	LP/P	rs61748521	Stargardt disease 1 (STGD1) [MIM:248200]
ABCA4	P78363	VAR_012617	p.Arg2263Leu	LP/P	rs281865407	Stargardt disease 1 (STGD1) [MIM:248200]
ABCA4	P78363	VAR_014703	p.Ser752Ile	LB/B	rs1801369	-
ABCA4	P78363	VAR_035736	p.Thr224Met	US	rs373540612	A breast cancer sample
ABCA4	P78363	VAR_067427	p.Ala762Glu	LP/P	-	Macular degeneration, age-related, 2 (ARMD2) [MIM:153800]
ABCA4	P78363	VAR_067428	p.Trp1724Cys	LP/P	-	Macular degeneration, age-related, 2 (ARMD2) [MIM:153800]
ABCA4	P78363	VAR_067429	p.Ile2047Asn	LP/P	-	Macular degeneration, age-related, 2 (ARMD2) [MIM:153800]
ABCA4	P78363	VAR_067430	p.Cys2137Tyr	LP/P	-	Macular degeneration, age-related, 2 (ARMD2) [MIM:153800]
ABCA5	Q8WWZ7	VAR_027571	p.Gln93Lys	LB/B	rs12383	-
ABCA5	Q8WWZ7	VAR_027572	p.Gln484Arg	LB/B	rs17686569	-
ABCA5	Q8WWZ7	VAR_027573	p.Met753Val	LB/B	rs9898003	-
ABCA5	Q8WWZ7	VAR_027574	p.Ala832Ser	LB/B	rs536009	-
ABCA5	Q8WWZ7	VAR_027575	p.Met960Val	LB/B	rs557491	-
ABCA5	Q8WWZ7	VAR_048128	p.Ala178Thr	LB/B	rs11544715	-
ABCA5	Q8WWZ7	VAR_048129	p.Asp1260Gly	LB/B	rs11544716	-
ABCA6	Q8N139	VAR_027576	p.Val282Ile	LB/B	rs4968839	-
ABCA6	Q8N139	VAR_027577	p.Asn610Tyr	LB/B	rs9282554	-
ABCA6	Q8N139	VAR_027578	p.Met698Ile	LB/B	rs9282553	-
ABCA6	Q8N139	VAR_027579	p.Met875Ile	LB/B	rs7212506	-
ABCA6	Q8N139	VAR_027580	p.Asn1322Ser	LB/B	rs2302134	-
ABCA7	Q8IZY2	VAR_027581	p.Glu188Gly	LB/B	rs3764645	-
ABCA7	Q8IZY2	VAR_027582	p.Thr319Ala	LB/B	rs3752232	-
ABCA7	Q8IZY2	VAR_027583	p.His395Arg	LB/B	rs3764647	-
ABCA7	Q8IZY2	VAR_027584	p.Arg463His	LB/B	rs3752233	-
ABCA7	Q8IZY2	VAR_027585	p.Asn718Thr	LB/B	rs3752239	-
ABCA7	Q8IZY2	VAR_027586	p.Arg1349Gln	LB/B	rs3745842	-
ABCA7	Q8IZY2	VAR_027587	p.Gly1527Ala	LB/B	rs3752246	-
ABCA7	Q8IZY2	VAR_027588	p.Gln1686Arg	LB/B	rs4147918	-
ABCA7	Q8IZY2	VAR_027589	p.Ala2045Ser	LB/B	rs4147934	-

ABCA7 Q8IZY2 VAR_060985 p.Ala676Thr LB/B rs59851484 -
ABCA7 Q8IZY2 VAR_081204 p.Arg880Gln LP/P rs143718918 Alzheimer disease 9 (AD9) [MIM:608907]
ABCA8 O94911 VAR_027590 p.Thr256Ala LB/B rs16973446 -
ABCA8 O94911 VAR_027591 p.Gly331Ser LB/B rs4147979 -
ABCA8 O94911 VAR_027592 p.Tyr489Phe LB/B rs12150510 -
ABCA8 O94911 VAR_027593 p.Cys720Gly LB/B rs16973424 -
ABCA8 O94911 VAR_048130 p.Ala416Val LB/B rs35621847 -
ABCA8 O94911 VAR_048131 p.Leu659Arg LB/B rs35844316 -
ABCA8 O94911 VAR_048132 p.Gly1470Ser LB/B rs35403175 -
ABCA8 O94911 VAR_084139 p.Pro649Arg LB/B rs144777539 -
ABCA9 Q8IUA7 VAR_027594 p.Arg353His LB/B rs1860447 -
ABCA9 Q8IUA7 VAR_027595 p.Asn785Ser LB/B rs17684521 -
ABCA9 Q8IUA7 VAR_027596 p.Lys1306Thr LB/B rs2302294 -
ABCA9 Q8IUA7 VAR_027597 p.Gly1356Ser LB/B rs9916254 -
ABCB1 P08183 VAR_013361 p.Ser893Ala LB/B rs2032582 -
ABCB1 P08183 VAR_013362 p.Ser893Thr LB/B rs2032582 -
ABCB1 P08183 VAR_014704 p.Asn21Asp LB/B rs9282564 -
ABCB1 P08183 VAR_015001 p.Phe103Leu LB/B - -
ABCB1 P08183 VAR_015002 p.Gly185Val LB/B rs1128501 -
ABCB1 P08183 VAR_015003 p.Ser400Asn LB/B rs2229109 -
ABCB1 P08183 VAR_015004 p.Ala999Thr LB/B rs72552784 -
ABCB1 P08183 VAR_015005 p.Gln1107Pro LB/B rs55852620 -
ABCB1 P08183 VAR_018351 p.Glu108Lys LB/B - -
ABCB1 P08183 VAR_018352 p.Met986Val LB/B rs926081975 -
ABCB1 P08183 VAR_018353 p.Val1251Ile LB/B rs28364274 -
ABCB1 P08183 VAR_022276 p.Phe17Leu LB/B rs28381804 -
ABCB1 P08183 VAR_022277 p.Glu566Lys LB/B rs28381902 -
ABCB1 P08183 VAR_022278 p.Arg593Cys LB/B rs28381914 -
ABCB1 P08183 VAR_022279 p.Ile836Val LB/B rs28381967 -
ABCB1 P08183 VAR_022280 p.Pro1051Ala LB/B rs28401798 -
ABCB1 P08183 VAR_022281 p.Ser1141Thr LB/B rs2229107 -
ABCB1 P08183 VAR_035737 p.Lys887Asn US - A colorectal cancer sample
ABCB1 P08183 VAR_055423 p.Asn44Ser LB/B rs1202183 -
ABCB1 P08183 VAR_055424 p.Ala80Glu LB/B rs9282565 -
ABCB1 P08183 VAR_055425 p.Ile261Val LB/B rs36008564 -
ABCB1 P08183 VAR_055426 p.Ala599Thr LB/B rs2235036 -
ABCB1 P08183 VAR_055427 p.Arg669Cys LB/B rs35023033 -
ABCB1 P08183 VAR_055428 p.Val801Met LB/B rs2235039 -
ABCB1 P08183 VAR_055429 p.Ile829Val LB/B rs2032581 -
ABCB10 Q9NRK6 VAR_013702 p.Ala150Ser LB/B rs4148756 -
ABCB10 Q9NRK6 VAR_031435 p.Asp545Asn LB/B rs35698797 -
ABCB10 Q9NRK6 VAR_035735 p.Arg471Thr US - A breast cancer sample
ABCB10 Q9NRK6 VAR_048133 p.Arg242Gly LB/B rs17584642 -
ABCB11 O95342 VAR_010271 p.Glu297Gly LP/P rs11568372 Cholestasis, benign recurrent intrahepatic, 2 (BRIC2) [MIM:605479]
ABCB11 O95342 VAR_010271 p.Glu297Gly LP/P rs11568372 Cholestasis, progressive familial intrahepatic, 2 (PFIC2) [MIM:601847]
ABCB11 O95342 VAR_013332 p.Val284Leu LP/P - Cholestasis, progressive familial intrahepatic, 2 (PFIC2) [MIM:601847]

ABCB11	O95342	VAR_013333	p.Val444Ala	LB/B	rs2287622	-
ABCB11	O95342	VAR_013334	p.Lys461Glu	LP/P	rs1274558905	Cholestasis, progressive familial intrahepatic, 2 (PFIC2) [MIM:601847]
ABCB11	O95342	VAR_013335	p.Asp482Gly	LP/P	rs72549402	Cholestasis, progressive familial intrahepatic, 2 (PFIC2) [MIM:601847]
ABCB11	O95342	VAR_013336	p.Gly982Arg	LP/P	rs72549399	Cholestasis, progressive familial intrahepatic, 2 (PFIC2) [MIM:601847]
ABCB11	O95342	VAR_013337	p.Gly1004Asp	LP/P	-	Cholestasis, progressive familial intrahepatic, 2 (PFIC2) [MIM:601847]
ABCB11	O95342	VAR_013338	p.Arg1153Cys	LP/P	rs72549395	Cholestasis, progressive familial intrahepatic, 2 (PFIC2) [MIM:601847]
ABCB11	O95342	VAR_013339	p.Arg1268Gln	LP/P	rs72549394	Cholestasis, progressive familial intrahepatic, 2 (PFIC2) [MIM:601847]
ABCB11	O95342	VAR_030386	p.Glu186Gly	LP/P	rs72551307	Cholestasis, benign recurrent intrahepatic, 2 (BRIC2) [MIM:605479]
ABCB11	O95342	VAR_030387	p.Ile206Val	LB/B	rs11568357	-
ABCB11	O95342	VAR_030388	p.Gly238Val	LP/P	rs72551306	Cholestasis, progressive familial intrahepatic, 2 (PFIC2) [MIM:601847]
ABCB11	O95342	VAR_030389	p.Arg299Lys	LB/B	rs2287617	-
ABCB11	O95342	VAR_030390	p.Cys336Ser	LP/P	rs72551305	Cholestasis, progressive familial intrahepatic, 2 (PFIC2) [MIM:601847]
ABCB11	O95342	VAR_030391	p.Arg432Thr	LP/P	rs121908935	Cholestasis, benign recurrent intrahepatic, 2 (BRIC2) [MIM:605479]
ABCB11	O95342	VAR_030392	p.Ala570Thr	LP/P	rs886043807	Cholestasis, benign recurrent intrahepatic, 2 (BRIC2) [MIM:605479]
ABCB11	O95342	VAR_030393	p.Met677Val	LB/B	rs11568364	-
ABCB11	O95342	VAR_030394	p.Thr923Pro	LP/P	rs777469571	Cholestasis, benign recurrent intrahepatic, 2 (BRIC2) [MIM:605479]
ABCB11	O95342	VAR_030395	p.Ala926Pro	LP/P	rs72549400	Cholestasis, benign recurrent intrahepatic, 2 (BRIC2) [MIM:605479]
ABCB11	O95342	VAR_030396	p.Arg1050Cys	LP/P	rs72549398	Cholestasis, benign recurrent intrahepatic, 2 (BRIC2) [MIM:605479]
ABCB11	O95342	VAR_030397	p.Arg1128His	LP/P	rs756220860	Cholestasis, benign recurrent intrahepatic, 2 (BRIC2) [MIM:605479]
ABCB11	O95342	VAR_030398	p.Glu1186Lys	LB/B	rs1521808	-
ABCB11	O95342	VAR_035349	p.Val284Ala	LB/B	rs200739891	-
ABCB11	O95342	VAR_035350	p.Arg616Gly	LB/B	-	-
ABCB11	O95342	VAR_035351	p.Thr619Ala	LB/B	rs912519986	-
ABCB11	O95342	VAR_035352	p.Arg698His	LB/B	rs138642043	-
ABCB11	O95342	VAR_035353	p.Ala865Val	LB/B	rs118109635	-
ABCB11	O95342	VAR_035354	p.Arg958Gln	LB/B	rs761363245	-
ABCB11	O95342	VAR_043074	p.Arg415Gln	LB/B	rs371656014	-
ABCB11	O95342	VAR_043075	p.Asn591Ser	LB/B	rs11568367	-
ABCB11	O95342	VAR_043076	p.Asp676Tyr	US	-	-
ABCB11	O95342	VAR_043077	p.Gly855Arg	US	-	-
ABCB11	O95342	VAR_055472	p.Ser56Leu	LB/B	rs11568361	-
ABCB11	O95342	VAR_059106	p.Val444Asp	LB/B	rs2287622	-
ABCB11	O95342	VAR_059107	p.Val444Gly	LB/B	rs2287622	-

ABCB11	O95342	VAR_073967	p.Tyr337His	US	-	Cholestasis, progressive familial intrahepatic, 2 (PFIC2) [MIM:601847]
ABCB11	O95342	VAR_073968	p.Tyr472Cys	LP/P	rs369860506	Cholestasis, progressive familial intrahepatic, 2 (PFIC2) [MIM:601847]
ABCB11	O95342	VAR_073969	p.Arg696Trp	US	rs376216286	Cholestasis, progressive familial intrahepatic, 2 (PFIC2) [MIM:601847]
ABCB11	O95342	VAR_073970	p.Gln931Pro	US	-	Cholestasis, progressive familial intrahepatic, 2 (PFIC2) [MIM:601847]
ABCB11	O95342	VAR_073971	p.Asp1131Val	LP/P	-	Cholestasis, progressive familial intrahepatic, 2 (PFIC2) [MIM:601847]
ABCB11	O95342	VAR_073972	p.His1198Arg	US	-	Cholestasis, progressive familial intrahepatic, 2 (PFIC2) [MIM:601847]
ABCB11	O95342	VAR_083783	p.Cys129Tyr	LP/P	-	Cholestasis, progressive familial intrahepatic, 2 (PFIC2) [MIM:601847]
ABCB11	O95342	VAR_083784	p.Gln558His	LB/B	rs11568369	-
ABCB11	O95342	VAR_083785	p.Glu592Gln	LB/B	rs11568370	-
ABCB4	P21439	VAR_020223	p.Leu238Val	LB/B	rs45596335	-
ABCB4	P21439	VAR_020225	p.Arg652Gly	LB/B	rs2230028	-
ABCB4	P21439	VAR_023501	p.Thr175Ala	US	rs58238559	-
ABCB4	P21439	VAR_023502	p.Ser320Phe	US	rs72552778	Cholestasis of pregnancy, intrahepatic 3 (ICP3) [MIM:614972]
ABCB4	P21439	VAR_023502	p.Ser320Phe	US	rs72552778	Cholestasis, progressive familial intrahepatic, 3 (PFIC3) [MIM:602347]
ABCB4	P21439	VAR_023502	p.Ser320Phe	US	rs72552778	Gallbladder disease 1 (GBD1) [MIM:600803]
ABCB4	P21439	VAR_023503	p.Ala546Asp	LP/P	rs121918441	Cholestasis of pregnancy, intrahepatic 3 (ICP3) [MIM:614972]
ABCB4	P21439	VAR_023504	p.Pro1168Ser	LP/P	rs121918442	Gallbladder disease 1 (GBD1) [MIM:600803]
ABCB4	P21439	VAR_024359	p.Arg788Gln	LB/B	rs8187801	Gallbladder disease 1 (GBD1) [MIM:600803]
ABCB4	P21439	VAR_030763	p.Ile263Val	LB/B	rs45547936	-
ABCB4	P21439	VAR_030765	p.Thr651Asn	LB/B	rs45476795	-
ABCB4	P21439	VAR_043078	p.Asp87Glu	LB/B	-	-
ABCB4	P21439	VAR_043079	p.Pro95Ser	LB/B	rs377268767	-
ABCB4	P21439	VAR_043080	p.Trp138Arg	LP/P	rs72552781	Cholestasis, progressive familial intrahepatic, 3 (PFIC3) [MIM:602347]
ABCB4	P21439	VAR_043081	p.Arg150Lys	LP/P	rs757693457	Cholestasis of pregnancy, intrahepatic 3 (ICP3) [MIM:614972]
ABCB4	P21439	VAR_043082	p.Phe165Ile	LP/P	-	Gallbladder disease 1 (GBD1) [MIM:600803]
ABCB4	P21439	VAR_043083	p.Met301Thr	LP/P	rs72552779	Gallbladder disease 1 (GBD1) [MIM:600803]
ABCB4	P21439	VAR_043084	p.Ser346Ile	LP/P	rs67876345	Cholestasis, progressive familial intrahepatic, 3 (PFIC3) [MIM:602347]
ABCB4	P21439	VAR_043085	p.Ile367Val	LB/B	rs1168923653	-
ABCB4	P21439	VAR_043086	p.Glu395Gly	LP/P	rs72552777	Cholestasis, progressive familial intrahepatic, 3 (PFIC3) [MIM:602347]
ABCB4	P21439	VAR_043087	p.Thr424Ala	LP/P	rs1263565476	Cholestasis, progressive familial intrahepatic, 3 (PFIC3) [MIM:602347]
ABCB4	P21439	VAR_043088	p.Val425Met	LP/P	-	Cholestasis, progressive familial intrahepatic, 3 (PFIC3) [MIM:602347]
ABCB4	P21439	VAR_043089	p.Glu450Gly	LB/B	rs1189003716	-
ABCB4	P21439	VAR_043090	p.Glu528Asp	US	rs8187797	Gallbladder disease 1 (GBD1) [MIM:600803]

ABCB4 P21439 VAR_043091 p.Gly535Asp LP/P - Cholestasis, progressive familial intrahepatic, 3 (PFIC3) [MIM:602347]
ABCB4 P21439 VAR_043092 p.Ile541Phe LP/P rs66904256 Cholestasis, progressive familial intrahepatic, 3 (PFIC3) [MIM:602347]
ABCB4 P21439 VAR_043092 p.Ile541Phe LP/P rs66904256 Gallbladder disease 1 (GBD1) [MIM:600803]
ABCB4 P21439 VAR_043093 p.Leu556Arg LP/P - Cholestasis, progressive familial intrahepatic, 3 (PFIC3) [MIM:602347]
ABCB4 P21439 VAR_043094 p.Asp564Gly LP/P - Cholestasis, progressive familial intrahepatic, 3 (PFIC3) [MIM:602347]
ABCB4 P21439 VAR_043095 p.Arg590Gln US rs45575636 -
ABCB4 P21439 VAR_043096 p.Leu591Gln LP/P rs72552776 Gallbladder disease 1 (GBD1) [MIM:600803]
ABCB4 P21439 VAR_043097 p.Phe711Ser LP/P rs72552773 Cholestasis, progressive familial intrahepatic, 3 (PFIC3) [MIM:602347]
ABCB4 P21439 VAR_043098 p.Gly742Ser LB/B - -
ABCB4 P21439 VAR_043099 p.Gly762Glu LP/P - Cholestasis of pregnancy, intrahepatic 3 (ICP3) [MIM:614972]
ABCB4 P21439 VAR_043100 p.Ile764Leu US - -
ABCB4 P21439 VAR_043101 p.Thr775Met US rs148052192 -
ABCB4 P21439 VAR_043102 p.Ala934Thr US rs61730509 -
ABCB4 P21439 VAR_043103 p.Gly983Ser LP/P rs56187107 Cholestasis, progressive familial intrahepatic, 3 (PFIC3) [MIM:602347]
ABCB4 P21439 VAR_043104 p.Leu1082Gln LB/B rs1214110864 -
ABCB4 P21439 VAR_073728 p.Thr34Met LP/P rs142794414 Gallbladder disease 1 (GBD1) [MIM:600803]
ABCB4 P21439 VAR_073729 p.Arg47Gly LP/P - Gallbladder disease 1 (GBD1) [MIM:600803]
ABCB4 P21439 VAR_073730 p.Arg47Gln US rs372685632 -
ABCB4 P21439 VAR_073731 p.Gly68Arg LP/P rs1343667900 Cholestasis, progressive familial intrahepatic, 3 (PFIC3) [MIM:602347]
ABCB4 P21439 VAR_073732 p.Gly70Arg LP/P - Cholestasis, progressive familial intrahepatic, 3 (PFIC3) [MIM:602347]
ABCB4 P21439 VAR_073733 p.Leu71His LP/P rs780641693 Gallbladder disease 1 (GBD1) [MIM:600803]
ABCB4 P21439 VAR_073734 p.Leu73Val LP/P rs8187788 Cholestasis, progressive familial intrahepatic, 3 (PFIC3) [MIM:602347]
ABCB4 P21439 VAR_073734 p.Leu73Val LP/P rs8187788 Gallbladder disease 1 (GBD1) [MIM:600803]
ABCB4 P21439 VAR_073735 p.Phe78Cys LP/P rs1411970557 Gallbladder disease 1 (GBD1) [MIM:600803]
ABCB4 P21439 VAR_073736 p.Ser99Phe LP/P rs1408217402 Gallbladder disease 1 (GBD1) [MIM:600803]
ABCB4 P21439 VAR_073737 p.Gly124Ser LP/P - Gallbladder disease 1 (GBD1) [MIM:600803]
ABCB4 P21439 VAR_073738 p.Gly126Glu LP/P rs1021988376 Cholestasis, progressive familial intrahepatic, 3 (PFIC3) [MIM:602347]
ABCB4 P21439 VAR_073739 p.Phe154Ser LP/P - Gallbladder disease 1 (GBD1) [MIM:600803]
ABCB4 P21439 VAR_073740 p.Thr201Met LP/P rs753318087 Cholestasis, progressive familial intrahepatic, 3 (PFIC3) [MIM:602347]
ABCB4 P21439 VAR_073741 p.Ala250Pro LP/P - Cholestasis, progressive familial intrahepatic, 3 (PFIC3) [MIM:602347]
ABCB4 P21439 VAR_073742 p.Ala286Val LP/P rs765478923 Cholestasis, progressive familial intrahepatic, 3 (PFIC3) [MIM:602347]
ABCB4 P21439 VAR_073742 p.Ala286Val LP/P rs765478923 Gallbladder disease 1 (GBD1) [MIM:600803]
ABCB4 P21439 VAR_073743 p.Phe357Leu LP/P - Cholestasis, progressive familial intrahepatic, 3 (PFIC3) [MIM:602347]

ABCB4	P21439	VAR_073744	p.Ala364Val	LP/P	-	Cholestasis, progressive familial intrahepatic, 3 (PFIC3) [MIM:602347]
ABCB4	P21439	VAR_073745	p.Tyr403His	LP/P	rs121918443	Cholestasis, progressive familial intrahepatic, 3 (PFIC3) [MIM:602347]
ABCB4	P21439	VAR_073746	p.Arg406Gly	LP/P	-	Gallbladder disease 1 (GBD1) [MIM:600803]
ABCB4	P21439	VAR_073747	p.Arg406Gln	US	rs763807769	-
ABCB4	P21439	VAR_073748	p.Asp459His	LP/P	-	Cholestasis, progressive familial intrahepatic, 3 (PFIC3) [MIM:602347]
ABCB4	P21439	VAR_073749	p.Val475Ala	LP/P	-	Cholestasis, progressive familial intrahepatic, 3 (PFIC3) [MIM:602347]
ABCB4	P21439	VAR_073750	p.Pro479Leu	LP/P	rs748657435	Cholestasis, progressive familial intrahepatic, 3 (PFIC3) [MIM:602347]
ABCB4	P21439	VAR_073751	p.Leu481Arg	LP/P	-	Cholestasis, progressive familial intrahepatic, 3 (PFIC3) [MIM:602347]
ABCB4	P21439	VAR_073752	p.Asn510Ser	LP/P	rs375315619	Gallbladder disease 1 (GBD1) [MIM:600803]
ABCB4	P21439	VAR_073753	p.Ala511Thr	LP/P	rs1257887155	Cholestasis, progressive familial intrahepatic, 3 (PFIC3) [MIM:602347]
ABCB4	P21439	VAR_073753	p.Ala511Thr	LP/P	rs1257887155	Gallbladder disease 1 (GBD1) [MIM:600803]
ABCB4	P21439	VAR_073754	p.Glu513Lys	LP/P	-	Gallbladder disease 1 (GBD1) [MIM:600803]
ABCB4	P21439	VAR_073755	p.Arg545His	LP/P	-	Gallbladder disease 1 (GBD1) [MIM:600803]
ABCB4	P21439	VAR_073756	p.Arg549His	LP/P	rs761238221	Gallbladder disease 1 (GBD1) [MIM:600803]
ABCB4	P21439	VAR_073757	p.Glu558Lys	LP/P	rs1562975478	Cholestasis, progressive familial intrahepatic, 3 (PFIC3) [MIM:602347]
ABCB4	P21439	VAR_073758	p.His589Thr	LP/P	-	Gallbladder disease 1 (GBD1) [MIM:600803]
ABCB4	P21439	VAR_073759	p.Thr593Ala	LP/P	-	Cholestasis, progressive familial intrahepatic, 3 (PFIC3) [MIM:602347]
ABCB4	P21439	VAR_073760	p.Thr593Met	LP/P	rs571555115	Gallbladder disease 1 (GBD1) [MIM:600803]
ABCB4	P21439	VAR_073761	p.Met630Val	LP/P	rs372476723	Cholestasis, progressive familial intrahepatic, 3 (PFIC3) [MIM:602347]
ABCB4	P21439	VAR_073762	p.Glu647Lys	LP/P	rs972726699	Gallbladder disease 1 (GBD1) [MIM:600803]
ABCB4	P21439	VAR_073763	p.Leu701Pro	LP/P	rs988987669	Cholestasis, progressive familial intrahepatic, 3 (PFIC3) [MIM:602347]
ABCB4	P21439	VAR_073764	p.Thr715Ile	LP/P	rs138773456	Cholestasis, progressive familial intrahepatic, 3 (PFIC3) [MIM:602347]
ABCB4	P21439	VAR_073765	p.Gly723Glu	LP/P	-	Cholestasis, progressive familial intrahepatic, 3 (PFIC3) [MIM:602347]
ABCB4	P21439	VAR_073766	p.Pro726Leu	LP/P	rs141677867	Gallbladder disease 1 (GBD1) [MIM:600803]
ABCB4	P21439	VAR_073767	p.Pro726Thr	LP/P	-	Cholestasis, progressive familial intrahepatic, 3 (PFIC3) [MIM:602347]
ABCB4	P21439	VAR_073768	p.Ser729Leu	LP/P	rs970324585	Gallbladder disease 1 (GBD1) [MIM:600803]
ABCB4	P21439	VAR_073769	p.Ala737Val	LP/P	rs147134978	Cholestasis, progressive familial intrahepatic, 3 (PFIC3) [MIM:602347]
ABCB4	P21439	VAR_073770	p.Ala840Asp	LP/P	-	Cholestasis, progressive familial intrahepatic, 3 (PFIC3) [MIM:602347]
ABCB4	P21439	VAR_073771	p.Gly954Ser	LP/P	rs779829759	Cholestasis, progressive familial intrahepatic, 3 (PFIC3) [MIM:602347]
ABCB4	P21439	VAR_073772	p.Val964Thr	US	-	-
ABCB4	P21439	VAR_073773	p.Leu975Val	LP/P	rs759787957	Gallbladder disease 1 (GBD1) [MIM:600803]

ABCB4 P21439 VAR_073774 p.Ser978Pro LP/P rs1051861187 Cholestasis, progressive familial intrahepatic, 3 (PFIC3) [MIM:602347]
ABCB4 P21439 VAR_073775 p.Arg1084Trp LP/P rs1262922848 Gallbladder disease 1 (GBD1) [MIM:600803]
ABCB4 P21439 VAR_073776 p.Glu1125Lys LP/P - Cholestasis, progressive familial intrahepatic, 3 (PFIC3) [MIM:602347]
ABCB4 P21439 VAR_073777 p.Ala1193Thr LP/P - Cholestasis, progressive familial intrahepatic, 3 (PFIC3) [MIM:602347]
ABCB4 P21439 VAR_079611 p.Gly536Arg LP/P - Gallbladder disease 1 (GBD1) [MIM:600803]
ABCB4 P21439 VAR_079612 p.Ser1183Leu LP/P - Gallbladder disease 1 (GBD1) [MIM:600803]
ABCB4 P21439 VAR_079613 p.Gly1185Ser LP/P - Gallbladder disease 1 (GBD1) [MIM:600803]
ABCB5 Q2M3G0 VAR_028387 p.Lys560Glu LB/B rs2301641 -
ABCB5 Q2M3G0 VAR_028388 p.Lys669Arg LB/B rs13222448 -
ABCB5 Q2M3G0 VAR_028389 p.Ala915Thr LB/B rs17143304 -
ABCB5 Q2M3G0 VAR_028390 p.Glu970Lys LB/B rs6461515 -
ABCB5 Q2M3G0 VAR_033456 p.Gln905His LB/B rs35885925 -
ABCB5 Q2M3G0 VAR_035731 p.Glu675Val US - A colorectal cancer sample
ABCB5 Q2M3G0 VAR_062662 p.Ala880Thr US rs1187237313 A pancreatic ductal adenocarcinoma sample
ABCB6 Q9NP58 VAR_029749 p.Arg648Gln LB/B rs13402964 -
ABCB6 Q9NP58 VAR_035732 p.Arg69Gly US - A breast cancer sample
ABCB6 Q9NP58 VAR_047552 p.Leu293Val LB/B rs13018440 -
ABCB6 Q9NP58 VAR_060986 p.Arg343Gln LB/B rs60322991 -
ABCB6 Q9NP58 VAR_067394 p.Ala57Thr US rs387906911 Microphthalmia, isolated, with coloboma, 7 (MCOPCB7) [MIM:614497]
ABCB6 Q9NP58 VAR_067395 p.Leu811Val LP/P rs387906910 Microphthalmia, isolated, with coloboma, 7 (MCOPCB7) [MIM:614497]
ABCB6 Q9NP58 VAR_070602 p.Ser170Gly LP/P rs397514757 Dyschromatosis universalis hereditaria 3 (DUH3) [MIM:615402]
ABCB6 Q9NP58 VAR_070603 p.Leu356Pro LP/P rs397514756 Dyschromatosis universalis hereditaria 3 (DUH3) [MIM:615402]
ABCB6 Q9NP58 VAR_070604 p.Gly579Glu LP/P rs397514758 Dyschromatosis universalis hereditaria 3 (DUH3) [MIM:615402]
ABCB6 Q9NP58 VAR_071133 p.Arg375Gln LP/P rs754667801 Pseudohyperkalemia, familial, 2, due to red cell leak (PSHK2) [MIM:609153]
ABCB6 Q9NP58 VAR_071134 p.Arg375Trp LP/P rs764893806 Pseudohyperkalemia, familial, 2, due to red cell leak (PSHK2) [MIM:609153]
ABCB6 Q9NP58 VAR_071135 p.Ala453Val LP/P - Dyschromatosis universalis hereditaria 3 (DUH3) [MIM:615402]
ABCB6 Q9NP58 VAR_071136 p.Gln555Lys LP/P rs796065353 Dyschromatosis universalis hereditaria 3 (DUH3) [MIM:615402]
ABCB6 Q9NP58 VAR_073973 p.Ser322Arg LP/P rs1574815954 Dyschromatosis universalis hereditaria 3 (DUH3) [MIM:615402]
ABCB6 Q9NP58 VAR_073974 p.Tyr424His LP/P - Dyschromatosis universalis hereditaria 3 (DUH3) [MIM:615402]
ABCB6 Q9NP58 VAR_076206 p.Arg723Gln LP/P rs148211042 Pseudohyperkalemia, familial, 2, due to red cell leak (PSHK2) [MIM:609153]
ABCB6 Q9NP58 VAR_084494 p.Arg192Gln US - -
ABCB6 Q9NP58 VAR_084495 p.Arg276Trp US - -
ABCB6 Q9NP58 VAR_084496 p.Ala492Thr US - -
ABCB6 Q9NP58 VAR_084497 p.Thr521Ser US - -

ABCB6	Q9NP58	VAR_084498	p.Gly588Ser	US	-	-
ABCB6	Q9NP58	VAR_084499	p.Ala681Thr	US	-	-
ABCB7	O75027	VAR_009156	p.Ile400Met	LP/P	rs72554634	Anemia, sideroblastic, spinocerebellar ataxia (ASAT) [MIM:301310]
ABCB7	O75027	VAR_012640	p.Glu433Lys	LP/P	rs80356714	Anemia, sideroblastic, spinocerebellar ataxia (ASAT) [MIM:301310]
ABCB7	O75027	VAR_022872	p.Arg315Gly	LB/B	-	-
ABCB7	O75027	VAR_022873	p.Phe346Ile	LB/B	-	-
ABCB7	O75027	VAR_022874	p.Val411Leu	LP/P	rs80356713	Anemia, sideroblastic, spinocerebellar ataxia (ASAT) [MIM:301310]
ABCB7	O75027	VAR_037972	p.Val581Ala	LB/B	rs1340989	-
ABCB7	O75027	VAR_055471	p.Ala580Val	LB/B	rs1340989	-
ABCB7	O75027	VAR_067354	p.Glu208Asp	LP/P	rs515726147	Anemia, sideroblastic, spinocerebellar ataxia (ASAT) [MIM:301310]
ABCB8	Q9NUT2	VAR_013331	p.Val152Ile	LB/B	rs4148844	-
ABCB8	Q9NUT2	VAR_035733	p.Ile165Thr	US	-	A breast cancer sample
ABCB8	Q9NUT2	VAR_035734	p.Ala690Gly	US	-	A breast cancer sample
ABCB9	Q9NP78	VAR_013701	p.Val121Met	LB/B	rs3803002	-
ABCC1	P33527	VAR_011488	p.Arg633Gln	LB/B	rs112282109	-
ABCC1	P33527	VAR_011489	p.Gly671Val	LB/B	rs45511401	-
ABCC1	P33527	VAR_013317	p.Cys43Ser	LB/B	rs41395947	-
ABCC1	P33527	VAR_013318	p.Thr73Ile	LB/B	rs41494447	-
ABCC1	P33527	VAR_013319	p.Met117Thr	LB/B	-	-
ABCC1	P33527	VAR_013320	p.Arg433Ser	LB/B	rs60782127	-
ABCC1	P33527	VAR_013321	p.Arg723Gln	LB/B	rs4148356	-
ABCC1	P33527	VAR_013322	p.Arg1058Gln	LB/B	rs41410450	-
ABCC1	P33527	VAR_013323	p.Ser1512Leu	LB/B	rs369410659	-
ABCC1	P33527	VAR_055384	p.Ala861Thr	LB/B	rs45517537	-
ABCC1	P33527	VAR_055385	p.Cys1047Ser	LB/B	rs13337489	-
ABCC1	P33527	VAR_055386	p.Val1146Ile	LB/B	rs28706727	-
ABCC1	P33527	VAR_083988	p.Gly231Asp	US	-	Deafness, autosomal dominant, 77 (DFNA77) [MIM:618915]
ABCC1	P33527	VAR_083989	p.Trp242Cys	LB/B	-	-
ABCC1	P33527	VAR_083990	p.Glu296Val	US	-	Deafness, autosomal dominant, 77 (DFNA77) [MIM:618915]
ABCC1	P33527	VAR_083991	p.Asn590Ser	LP/P	-	Deafness, autosomal dominant, 77 (DFNA77) [MIM:618915]
ABCC1	P33527	VAR_083992	p.Val886Ile	LB/B	-	-
ABCC1	P33527	VAR_083993	p.Gly1007Arg	LB/B	-	-
ABCC1	P33527	VAR_083994	p.Met1086Thr	LB/B	-	-
ABCC10	Q5T3U5	VAR_028391	p.Ile948Thr	LB/B	rs2125739	-
ABCC11	Q96J66	VAR_025437	p.Arg19His	LB/B	rs16945988	-
ABCC11	Q96J66	VAR_025438	p.Gly180Arg	LB/B	rs17822931	-
ABCC11	Q96J66	VAR_048144	p.Ala317Glu	LB/B	rs11863236	-
ABCC11	Q96J66	VAR_048145	p.Thr546Met	LB/B	rs17822471	-
ABCC11	Q96J66	VAR_048146	p.Val648Ile	LB/B	rs16945930	-
ABCC11	Q96J66	VAR_048147	p.Val687Ile	LB/B	rs16945928	-
ABCC11	Q96J66	VAR_048148	p.Lys735Arg	LB/B	rs16945926	-
ABCC11	Q96J66	VAR_048149	p.His1344Arg	LB/B	rs16945916	-

ABCC11	Q96J66	VAR_077575	p.Arg630Trp	LB/B	rs41282045	-
ABCC11	Q96J66	VAR_077576	p.Met970Val	LB/B	rs41280943	-
ABCC12	Q96J65	VAR_028392	p.Ile9Leu	LB/B	rs16945901	-
ABCC12	Q96J65	VAR_028393	p.Ala102Glu	LB/B	rs16945874	-
ABCC12	Q96J65	VAR_028394	p.Asn587Tyr	LB/B	rs16945816	-
ABCC12	Q96J65	VAR_028395	p.Lys894Met	LB/B	rs8057474	-
ABCC12	Q96J65	VAR_028396	p.Thr989Ser	LB/B	rs6500305	-
ABCC12	Q96J65	VAR_028397	p.Tyr1013His	LB/B	rs6500304	-
ABCC12	Q96J65	VAR_028398	p.Arg1117Cys	LB/B	rs7193955	-
ABCC12	Q96J65	VAR_028399	p.Glu1191Ala	LB/B	rs16945787	-
ABCC12	Q96J65	VAR_028400	p.Phe1349Leu	LB/B	rs12373105	-
ABCC12	Q96J65	VAR_048139	p.Glu690Val	LB/B	rs34135219	-
ABCC12	Q96J65	VAR_048140	p.Ile1187Thr	LB/B	rs34106426	-
ABCC2	Q92887	VAR_000099	p.Arg768Trp	LP/P	rs56199535	Dubin-Johnson syndrome (DJS) [MIM:237500]
ABCC2	Q92887	VAR_010756	p.Gln1382Arg	LP/P	rs72558202	Dubin-Johnson syndrome (DJS) [MIM:237500]
ABCC2	Q92887	VAR_013324	p.Ser281Asn	LB/B	rs56131651	-
ABCC2	Q92887	VAR_013325	p.Val417Ile	LB/B	rs2273697	-
ABCC2	Q92887	VAR_013326	p.Ser789Phe	LB/B	rs56220353	-
ABCC2	Q92887	VAR_013327	p.Arg1150His	LP/P	rs72558200	Dubin-Johnson syndrome (DJS) [MIM:237500]
ABCC2	Q92887	VAR_013328	p.Ile1173Phe	LP/P	rs72558201	Dubin-Johnson syndrome (DJS) [MIM:237500]
ABCC2	Q92887	VAR_013330	p.Ala1450Thr	LB/B	rs56296335	-
ABCC2	Q92887	VAR_020226	p.Asp333Gly	LB/B	rs17222674	-
ABCC2	Q92887	VAR_020227	p.Arg353His	LB/B	rs7080681	-
ABCC2	Q92887	VAR_020228	p.Ile670Thr	LB/B	rs17222632	-
ABCC2	Q92887	VAR_020229	p.Leu849Arg	LB/B	rs17222617	-
ABCC2	Q92887	VAR_020230	p.Ile1036Thr	LB/B	rs45441199	-
ABCC2	Q92887	VAR_020231	p.Arg1181Leu	LB/B	rs8187692	-
ABCC2	Q92887	VAR_020232	p.Val1188Glu	LB/B	rs17222723	-
ABCC2	Q92887	VAR_020233	p.Pro1291Leu	LB/B	rs17216317	-
ABCC2	Q92887	VAR_020234	p.Cys1515Tyr	LB/B	rs8187710	-
ABCC2	Q92887	VAR_024360	p.Thr1273Ala	LB/B	rs8187699	-
ABCC2	Q92887	VAR_029113	p.Met246Leu	LB/B	rs17222744	-
ABCC2	Q92887	VAR_029115	p.Lys495Glu	LB/B	rs17222561	-
ABCC2	Q92887	VAR_029116	p.Phe562Leu	LB/B	rs17216233	-
ABCC2	Q92887	VAR_029117	p.Ile982Val	LB/B	rs17222554	-
ABCC2	Q92887	VAR_029118	p.Asn1063Ser	LB/B	rs17222540	-
ABCC2	Q92887	VAR_047152	p.Tyr39Phe	LB/B	rs927344	-
ABCC2	Q92887	VAR_070607	p.Thr486Ile	LB/B	rs17222589	-
ABCC2	Q92887	VAR_070608	p.Gly921Ser	LB/B	rs41318029	-
ABCC2	Q92887	VAR_070609	p.Arg1174His	LB/B	rs139188247	-
ABCC2	Q92887	VAR_070610	p.Asn1244Lys	LB/B	rs757141905	-
ABCC3	O15438	VAR_020235	p.Ser346Phe	LB/B	rs11568605	-
ABCC3	O15438	VAR_020237	p.Arg1297His	LB/B	rs11568591	-
ABCC3	O15438	VAR_020239	p.Gln1365Arg	LB/B	rs11568590	-
ABCC3	O15438	VAR_020240	p.Arg1381Ser	LB/B	rs45461799	-
ABCC3	O15438	VAR_029119	p.Gly11Asp	LB/B	rs11568609	-
ABCC3	O15438	VAR_029120	p.Arg1286Gly	LB/B	rs11568593	-
ABCC3	O15438	VAR_084161	p.Leu548Gln	LB/B	rs144520783	-
ABCC4	O15439	VAR_020241	p.Met184Thr	LB/B	rs45454092	-

ABCC4	O15439	VAR_020242	p.Gly187Trp	LB/B	rs11568658	-
ABCC4	O15439	VAR_020243	p.Lys498Glu	LB/B	rs11568669	-
ABCC4	O15439	VAR_020244	p.Met744Val	LB/B	rs9282570	-
ABCC4	O15439	VAR_020245	p.Val860Met	LB/B	rs45477596	-
ABCC4	O15439	VAR_020246	p.Val900Leu	LB/B	rs45504892	-
ABCC4	O15439	VAR_022072	p.Lys304Asn	LB/B	rs2274407	-
ABCC4	O15439	VAR_022073	p.Glu757Lys	LB/B	rs3765534	-
ABCC4	O15439	VAR_029121	p.Pro78Ala	LB/B	rs11568689	-
ABCC4	O15439	VAR_029122	p.Pro403Leu	LB/B	rs11568705	-
ABCC4	O15439	VAR_029123	p.Gly487Glu	LB/B	rs11568668	-
ABCC4	O15439	VAR_029124	p.Ile625Met	LB/B	rs11568699	-
ABCC4	O15439	VAR_029125	p.Pro667Leu	LB/B	rs11568697	-
ABCC4	O15439	VAR_029126	p.Thr1142Met	LB/B	rs11568644	-
ABCC4	O15439	VAR_045684	p.Tyr556Cys	LB/B	rs753414892	-
ABCC4	O15439	VAR_045685	p.Val776Ile	LB/B	rs146708960	-
ABCC4	O15439	VAR_045686	p.Arg820Ile	LB/B	rs11568659	-
ABCC4	O15439	VAR_045687	p.Val854Phe	LB/B	rs11568694	-
ABCC4	O15439	VAR_045688	p.Ile866Val	LB/B	rs139970608	-
ABCC4	O15439	VAR_046445	p.Leu18Ile	LB/B	rs11568681	-
ABCC4	O15439	VAR_046446	p.Cys171Gly	LB/B	rs4148460	-
ABCC4	O15439	VAR_046447	p.Lys293Glu	LB/B	rs11568684	-
ABCC4	O15439	VAR_046448	p.Thr356Met	LB/B	rs11568701	-
ABCC6	O95255	VAR_011490	p.Val614Ala	LB/B	rs12931472	-
ABCC6	O95255	VAR_011491	p.Arg1114Pro	LP/P	rs63750427	Pseudoxanthoma elasticum (PXE) [MIM:264800]
ABCC6	O95255	VAR_011492	p.Arg1138Gln	LP/P	rs60791294	Pseudoxanthoma elasticum (PXE) [MIM:264800]
ABCC6	O95255	VAR_011493	p.Arg1138Trp	LP/P	rs28939701	Pseudoxanthoma elasticum (PXE) [MIM:264800]
ABCC6	O95255	VAR_011494	p.Arg1268Gln	LB/B	rs2238472	-
ABCC6	O95255	VAR_011495	p.Arg1314Trp	LP/P	rs63750759	Arterial calcification of infancy, generalized, 2 (GACI2) [MIM:614473]
ABCC6	O95255	VAR_011495	p.Arg1314Trp	LP/P	rs63750759	Pseudoxanthoma elasticum (PXE) [MIM:264800]
ABCC6	O95255	VAR_013364	p.Gly61Asp	LB/B	rs72657696	-
ABCC6	O95255	VAR_013365	p.Arg64Trp	LB/B	rs557180313	-
ABCC6	O95255	VAR_013366	p.Gly207Arg	LB/B	rs72657697	-
ABCC6	O95255	VAR_013367	p.Arg265Gly	LB/B	rs72657698	-
ABCC6	O95255	VAR_013368	p.Lys281Glu	LB/B	rs4780606	-
ABCC6	O95255	VAR_013369	p.Ile319Val	LB/B	rs72657699	-
ABCC6	O95255	VAR_013370	p.Thr364Arg	LP/P	rs72653759	Pseudoxanthoma elasticum (PXE) [MIM:264800]
ABCC6	O95255	VAR_013371	p.Asn411Lys	LP/P	rs9930886	Pseudoxanthoma elasticum (PXE) [MIM:264800]
ABCC6	O95255	VAR_013372	p.Ala455Pro	LP/P	rs67996819	Pseudoxanthoma elasticum (PXE) [MIM:264800]
ABCC6	O95255	VAR_013373	p.Asn497Lys	LB/B	rs72653770	-
ABCC6	O95255	VAR_013374	p.Arg518Gln	LP/P	rs72653772	Pseudoxanthoma elasticum (PXE) [MIM:264800]
ABCC6	O95255	VAR_013375	p.Phe568Ser	LP/P	rs66864704	Pseudoxanthoma elasticum (PXE) [MIM:264800]
ABCC6	O95255	VAR_013376	p.His632Gln	LB/B	rs8058694	-
ABCC6	O95255	VAR_013377	p.Leu673Pro	LP/P	rs67470842	Pseudoxanthoma elasticum (PXE) [MIM:264800]
ABCC6	O95255	VAR_013378	p.Arg765Gln	LP/P	rs67561842	Pseudoxanthoma elasticum (PXE) [MIM:264800]
ABCC6	O95255	VAR_013379	p.Leu953His	LB/B	rs72657700	-
ABCC6	O95255	VAR_013380	p.Ser1121Trp	LP/P	rs63750987	Pseudoxanthoma elasticum (PXE) [MIM:264800]
ABCC6	O95255	VAR_013381	p.Arg1138Pro	LP/P	rs60791294	Pseudoxanthoma elasticum (PXE) [MIM:264800]
ABCC6	O95255	VAR_013382	p.Gly1203Asp	LP/P	rs63750607	Pseudoxanthoma elasticum (PXE) [MIM:264800]

ABCC6	O95255	VAR_013383	p.Trp1241Cys	LB/B	rs72657701	-
ABCC6	O95255	VAR_013384	p.Val1298Phe	LP/P	rs63751325	Pseudoxanthoma elasticum (PXE) [MIM:264800]
ABCC6	O95255	VAR_013385	p.Thr1301Ile	LP/P	rs63750494	Pseudoxanthoma elasticum (PXE) [MIM:264800]
ABCC6	O95255	VAR_013386	p.Gly1302Arg	LP/P	rs63749856	Pseudoxanthoma elasticum (PXE) [MIM:264800]
ABCC6	O95255	VAR_013387	p.Ala1303Pro	LP/P	rs63750410	Pseudoxanthoma elasticum (PXE) [MIM:264800]
ABCC6	O95255	VAR_013388	p.Arg1314Gln	LP/P	rs63751086	Pseudoxanthoma elasticum (PXE) [MIM:264800]
ABCC6	O95255	VAR_013389	p.Gly1321Ser	LP/P	rs63749823	Pseudoxanthoma elasticum (PXE) [MIM:264800]
ABCC6	O95255	VAR_013390	p.Arg1339Cys	LP/P	rs28939702	Pseudoxanthoma elasticum (PXE) [MIM:264800]
ABCC6	O95255	VAR_013391	p.Gln1347His	LP/P	rs63751111	Pseudoxanthoma elasticum (PXE) [MIM:264800]
ABCC6	O95255	VAR_013392	p.Gly1354Arg	LP/P	rs63750018	Pseudoxanthoma elasticum (PXE) [MIM:264800]
ABCC6	O95255	VAR_013393	p.Asp1361Asn	LP/P	rs58695352	Pseudoxanthoma elasticum (PXE) [MIM:264800]
ABCC6	O95255	VAR_013394	p.Ile1424Thr	LP/P	rs63750295	Pseudoxanthoma elasticum (PXE) [MIM:264800]
ABCC6	O95255	VAR_055477	p.Val665Ala	LB/B	rs4341770	-
ABCC6	O95255	VAR_059108	p.Met848Val	LB/B	rs6416668	-
ABCC6	O95255	VAR_060988	p.Leu1097Ile	LB/B	rs60707953	-
ABCC6	O95255	VAR_067841	p.Ala78Thr	LP/P	rs2856597	Pseudoxanthoma elasticum (PXE) [MIM:264800]
ABCC6	O95255	VAR_067842	p.Glu125Lys	LP/P	rs3853814	Pseudoxanthoma elasticum (PXE) [MIM:264800]
ABCC6	O95255	VAR_067843	p.Gly129Glu	LP/P	rs72653753	Pseudoxanthoma elasticum (PXE) [MIM:264800]
ABCC6	O95255	VAR_067844	p.Ala158Val	LB/B	rs2606921	-
ABCC6	O95255	VAR_067845	p.Ser317Arg	LP/P	rs78678589	Pseudoxanthoma elasticum (PXE) [MIM:264800]
ABCC6	O95255	VAR_067846	p.Leu355Arg	LP/P	rs72653758	Arterial calcification of infancy, generalized, 2 (GACI2) [MIM:614473]
ABCC6	O95255	VAR_067846	p.Leu355Arg	LP/P	rs72653758	Pseudoxanthoma elasticum (PXE) [MIM:264800]
ABCC6	O95255	VAR_067848	p.Asn370Asp	LP/P	rs72653760	Pseudoxanthoma elasticum (PXE) [MIM:264800]
ABCC6	O95255	VAR_067849	p.Arg382Trp	LP/P	rs72653761	Pseudoxanthoma elasticum (PXE) [MIM:264800]
ABCC6	O95255	VAR_067850	p.Arg391Gly	LP/P	rs72653762	Arterial calcification of infancy, generalized, 2 (GACI2) [MIM:614473]
ABCC6	O95255	VAR_067850	p.Arg391Gly	LP/P	rs72653762	Pseudoxanthoma elasticum (PXE) [MIM:264800]
ABCC6	O95255	VAR_067851	p.Lys392Asn	LP/P	rs72653763	Pseudoxanthoma elasticum (PXE) [MIM:264800]
ABCC6	O95255	VAR_067852	p.Ser398Gly	LP/P	rs72653764	Pseudoxanthoma elasticum (PXE) [MIM:264800]
ABCC6	O95255	VAR_067853	p.Val417Met	LB/B	rs768869262	-
ABCC6	O95255	VAR_067854	p.Cys440Gly	LP/P	rs72653766	Pseudoxanthoma elasticum (PXE) [MIM:264800]
ABCC6	O95255	VAR_067855	p.Leu463His	LP/P	rs72653767	Pseudoxanthoma elasticum (PXE) [MIM:264800]
ABCC6	O95255	VAR_067856	p.Leu495His	LP/P	rs72653769	Pseudoxanthoma elasticum (PXE) [MIM:264800]
ABCC6	O95255	VAR_067857	p.Val514Ile	LB/B	rs59157279	-
ABCC6	O95255	VAR_067858	p.Ser535Pro	LP/P	rs72653773	Pseudoxanthoma elasticum (PXE) [MIM:264800]
ABCC6	O95255	VAR_067859	p.Phe551Ser	LP/P	rs72653774	Pseudoxanthoma elasticum (PXE) [MIM:264800]
ABCC6	O95255	VAR_067860	p.Ser590Phe	LP/P	rs537233133	Arterial calcification of infancy, generalized, 2 (GACI2) [MIM:614473]
ABCC6	O95255	VAR_067861	p.Ala594Val	LP/P	rs72653776	Pseudoxanthoma elasticum (PXE) [MIM:264800]
ABCC6	O95255	VAR_067862	p.Arg600Cys	LP/P	rs72653777	Pseudoxanthoma elasticum (PXE) [MIM:264800]
ABCC6	O95255	VAR_067863	p.Gly663Cys	LP/P	rs72653780	Pseudoxanthoma elasticum (PXE) [MIM:264800]
ABCC6	O95255	VAR_067864	p.Leu677Pro	LP/P	rs72653782	Pseudoxanthoma elasticum (PXE) [MIM:264800]
ABCC6	O95255	VAR_067865	p.Gln698Pro	LP/P	rs72653783	Pseudoxanthoma elasticum (PXE) [MIM:264800]
ABCC6	O95255	VAR_067866	p.Glu699Asp	LP/P	rs72653784	Pseudoxanthoma elasticum (PXE) [MIM:264800]
ABCC6	O95255	VAR_067867	p.Arg724Lys	LB/B	rs58073789	-
ABCC6	O95255	VAR_067868	p.Arg724Leu	LB/B	-	-
ABCC6	O95255	VAR_067869	p.Leu726Pro	LP/P	rs72653785	Pseudoxanthoma elasticum (PXE) [MIM:264800]
ABCC6	O95255	VAR_067870	p.Ile742Val	LB/B	rs59593133	-

ABCC6	O95255	VAR_067871	p.Met751Lys	LP/P	rs72653786	Pseudoxanthoma elasticum (PXE) [MIM:264800]
ABCC6	O95255	VAR_067872	p.Gly755Arg	LP/P	rs72653787	Pseudoxanthoma elasticum (PXE) [MIM:264800]
ABCC6	O95255	VAR_067873	p.Arg760Trp	LP/P	rs72653788	Pseudoxanthoma elasticum (PXE) [MIM:264800]
ABCC6	O95255	VAR_067874	p.Ala766Asp	LP/P	rs72653789	Pseudoxanthoma elasticum (PXE) [MIM:264800]
ABCC6	O95255	VAR_067875	p.Asp777Asn	LP/P	rs72653790	Pseudoxanthoma elasticum (PXE) [MIM:264800]
ABCC6	O95255	VAR_067876	p.Arg807Gln	LP/P	rs72653794	Pseudoxanthoma elasticum (PXE) [MIM:264800]
ABCC6	O95255	VAR_067877	p.Arg807Trp	LP/P	rs72653793	Pseudoxanthoma elasticum (PXE) [MIM:264800]
ABCC6	O95255	VAR_067878	p.Val810Met	LP/P	rs72653795	Pseudoxanthoma elasticum (PXE) [MIM:264800]
ABCC6	O95255	VAR_067879	p.Thr811Met	LP/P	rs72653796	Pseudoxanthoma elasticum (PXE) [MIM:264800]
ABCC6	O95255	VAR_067880	p.Ala820Pro	LP/P	rs72653797	Pseudoxanthoma elasticum (PXE) [MIM:264800]
ABCC6	O95255	VAR_067881	p.Arg881Ser	LP/P	rs72653800	Pseudoxanthoma elasticum (PXE) [MIM:264800]
ABCC6	O95255	VAR_067882	p.Thr944Ile	LP/P	rs72653801	Pseudoxanthoma elasticum (PXE) [MIM:264800]
ABCC6	O95255	VAR_067883	p.Leu946Ile	LB/B	rs61340537	-
ABCC6	O95255	VAR_067884	p.Ala950Thr	LP/P	rs72657689	Pseudoxanthoma elasticum (PXE) [MIM:264800]
ABCC6	O95255	VAR_067885	p.Gly992Arg	LP/P	rs72657692	Pseudoxanthoma elasticum (PXE) [MIM:264800]
ABCC6	O95255	VAR_067888	p.Asp1056Glu	LP/P	rs72657694	Pseudoxanthoma elasticum (PXE) [MIM:264800]
ABCC6	O95255	VAR_067889	p.Arg1064Trp	LB/B	rs41278174	-
ABCC6	O95255	VAR_067890	p.Arg1114Cys	LP/P	rs63749794	Arterial calcification of infancy, generalized, 2 (GACI2) [MIM:614473]
ABCC6	O95255	VAR_067890	p.Arg1114Cys	LP/P	rs63749794	Pseudoxanthoma elasticum (PXE) [MIM:264800]
ABCC6	O95255	VAR_067891	p.Ser1121Leu	LP/P	rs63750987	Pseudoxanthoma elasticum (PXE) [MIM:264800]
ABCC6	O95255	VAR_067892	p.Thr1130Met	LP/P	rs63750459	Pseudoxanthoma elasticum (PXE) [MIM:264800]
ABCC6	O95255	VAR_067893	p.Gly1133Ala	LP/P	rs63750473	Pseudoxanthoma elasticum (PXE) [MIM:264800]
ABCC6	O95255	VAR_067894	p.Ala1139Thr	LP/P	rs63750146	Pseudoxanthoma elasticum (PXE) [MIM:264800]
ABCC6	O95255	VAR_067895	p.Arg1164Gln	LP/P	rs63750457	Pseudoxanthoma elasticum (PXE) [MIM:264800]
ABCC6	O95255	VAR_067896	p.Arg1221Cys	LP/P	rs63751215	Pseudoxanthoma elasticum (PXE) [MIM:264800]
ABCC6	O95255	VAR_067897	p.Arg1221His	LP/P	rs63751001	Arterial calcification of infancy, generalized, 2 (GACI2) [MIM:614473]
ABCC6	O95255	VAR_067898	p.Leu1226Ile	LP/P	rs63750125	Pseudoxanthoma elasticum (PXE) [MIM:264800]
ABCC6	O95255	VAR_067899	p.Arg1235Trp	LP/P	rs63750402	Pseudoxanthoma elasticum (PXE) [MIM:264800]
ABCC6	O95255	VAR_067900	p.Asp1238His	LP/P	rs63749796	Pseudoxanthoma elasticum (PXE) [MIM:264800]
ABCC6	O95255	VAR_067901	p.Leu1335Pro	LP/P	rs63750414	Pseudoxanthoma elasticum (PXE) [MIM:264800]
ABCC6	O95255	VAR_067902	p.Leu1335Gln	LP/P	rs63750414	Pseudoxanthoma elasticum (PXE) [MIM:264800]
ABCC6	O95255	VAR_067903	p.Arg1339Leu	LP/P	rs63750622	Pseudoxanthoma elasticum (PXE) [MIM:264800]
ABCC6	O95255	VAR_067904	p.Arg1339His	LP/P	rs63750622	Pseudoxanthoma elasticum (PXE) [MIM:264800]
ABCC6	O95255	VAR_067905	p.Pro1346Ser	LP/P	rs63751112	Pseudoxanthoma elasticum (PXE) [MIM:264800]
ABCC6	O95255	VAR_067906	p.Arg1357Trp	LP/P	rs63750428	Pseudoxanthoma elasticum (PXE) [MIM:264800]
ABCC6	O95255	VAR_067907	p.Glu1400Lys	LP/P	rs63751241	Pseudoxanthoma elasticum (PXE) [MIM:264800]
ABCC6	O95255	VAR_067908	p.Gln1406Lys	LP/P	rs387906859	Pseudoxanthoma elasticum (PXE) [MIM:264800]
ABCC6	O95255	VAR_067909	p.Arg1459Cys	LP/P	rs72547524	Pseudoxanthoma elasticum (PXE) [MIM:264800]
ABCC6	O95255	VAR_072803	p.Pro4His	US	rs1555523872	-
ABCC6	O95255	VAR_072804	p.Ala9Glu	US	rs1555523855	-
ABCC6	O95255	VAR_072805	p.Pro21Ser	US	rs1235912910	-
ABCC6	O95255	VAR_072806	p.Arg64Gln	US	rs777566074	-
ABCC6	O95255	VAR_072807	p.Ala90Thr	US	rs957828732	-
ABCC6	O95255	VAR_072808	p.Arg419Gln	US	rs772434460	-
ABCC6	O95255	VAR_072809	p.Leu605Pro	US	rs768271196	-
ABCC6	O95255	VAR_072810	p.Glu709Gly	US	rs1555513103	-
ABCC6	O95255	VAR_072811	p.Met834Thr	US	rs1355752953	-

ABCC6 O95255 VAR_072812 p.Leu948Pro US rs1555510407 -
ABCC6 O95255 VAR_072813 p.Ala1442Thr US rs1462269230 -
ABCC8 Q09428 VAR_000100 p.Gly716Val LP/P rs72559723 Familial hyperinsulinemic hypoglycemia 1 (HHF1) [MIM:256450]
ABCC8 Q09428 VAR_008537 p.Arg1352Pro LP/P rs28936370 Familial hyperinsulinemic hypoglycemia 1 (HHF1) [MIM:256450]
ABCC8 Q09428 VAR_008539 p.Arg1420Cys LP/P rs28938469 Familial hyperinsulinemic hypoglycemia 1 (HHF1) [MIM:256450]
ABCC8 Q09428 VAR_008540 p.Arg1493Trp LP/P rs28936371 Familial hyperinsulinemic hypoglycemia 1 (HHF1) [MIM:256450]
ABCC8 Q09428 VAR_008639 p.Arg74Gln LP/P rs72559734 Familial hyperinsulinemic hypoglycemia 1 (HHF1) [MIM:256450]
ABCC8 Q09428 VAR_008640 p.His125Gln LP/P rs60637558 Familial hyperinsulinemic hypoglycemia 1 (HHF1) [MIM:256450]
ABCC8 Q09428 VAR_008641 p.Val187Asp LP/P rs137852672 Familial hyperinsulinemic hypoglycemia 1 (HHF1) [MIM:256450]
ABCC8 Q09428 VAR_008642 p.Asn188Ser LP/P rs797045213 Familial hyperinsulinemic hypoglycemia 1 (HHF1) [MIM:256450]
ABCC8 Q09428 VAR_008643 p.Arg275Gln LB/B rs185040406 -
ABCC8 Q09428 VAR_008644 p.Asn406Asp LP/P rs72559728 Familial hyperinsulinemic hypoglycemia 1 (HHF1) [MIM:256450]
ABCC8 Q09428 VAR_008645 p.Val560Met LB/B rs4148619 -
ABCC8 Q09428 VAR_008646 p.Phe591Leu LP/P rs72559726 Familial hyperinsulinemic hypoglycemia 1 (HHF1) [MIM:256450]
ABCC8 Q09428 VAR_008647 p.Asp810Asn LB/B rs767572066 -
ABCC8 Q09428 VAR_008648 p.Arg834Cys LB/B rs140068774 -
ABCC8 Q09428 VAR_008649 p.Thr1138Met LP/P rs201351976 Familial hyperinsulinemic hypoglycemia 1 (HHF1) [MIM:256450]
ABCC8 Q09428 VAR_008650 p.Arg1214Gln LP/P rs367850779 Familial hyperinsulinemic hypoglycemia 1 (HHF1) [MIM:256450]
ABCC8 Q09428 VAR_008651 p.Val1360Gly LB/B - -
ABCC8 Q09428 VAR_008652 p.Ala1369Ser LB/B rs757110 -
ABCC8 Q09428 VAR_008653 p.Gly1378Arg LP/P rs925231098 Familial hyperinsulinemic hypoglycemia 1 (HHF1) [MIM:256450]
ABCC8 Q09428 VAR_008654 p.Gly1381Ser LP/P rs773448052 Familial hyperinsulinemic hypoglycemia 1 (HHF1) [MIM:256450]
ABCC8 Q09428 VAR_008655 p.Arg1393His LP/P rs769279368 Familial hyperinsulinemic hypoglycemia 1 (HHF1) [MIM:256450]
ABCC8 Q09428 VAR_008656 p.Gly1478Arg LP/P rs72559715 Familial hyperinsulinemic hypoglycemia 1 (HHF1) [MIM:256450]
ABCC8 Q09428 VAR_008658 p.Val1572Ile LB/B rs8192690 -
ABCC8 Q09428 VAR_015006 p.Asp673Asn LB/B rs777986828 -
ABCC8 Q09428 VAR_015007 p.Val1360Met LP/P - Familial hyperinsulinemic hypoglycemia 1 (HHF1) [MIM:256450]
ABCC8 Q09428 VAR_015008 p.Arg1436Gln LP/P rs387906407 Familial hyperinsulinemic hypoglycemia 1 (HHF1) [MIM:256450]
ABCC8 Q09428 VAR_015009 p.Glu1506Lys LP/P rs137852671 Familial hyperinsulinemic hypoglycemia 1 (HHF1) [MIM:256450]

ABCC8 Q09428 VAR_015010 p.Leu1543Pro LP/P rs72559713 Familial hyperinsulinemic hypoglycemia 1 (HHF1) [MIM:256450]
ABCC8 Q09428 VAR_029777 p.Leu104Val LB/B rs10400391 -
ABCC8 Q09428 VAR_029778 p.Phe132Leu LP/P rs80356637 Diabetes mellitus, permanent neonatal, 3 (PNDM3) [MIM:618857]
ABCC8 Q09428 VAR_029779 p.Leu213Arg LP/P rs80356642 Diabetes mellitus, permanent neonatal, 3 (PNDM3) [MIM:618857]
ABCC8 Q09428 VAR_029780 p.Cys435Arg LP/P - Transient neonatal diabetes mellitus 2 (TNDM2) [MIM:610374]
ABCC8 Q09428 VAR_029781 p.Leu582Val LP/P rs137852674 Transient neonatal diabetes mellitus 2 (TNDM2) [MIM:610374]
ABCC8 Q09428 VAR_029782 p.His1023Tyr LP/P - Transient neonatal diabetes mellitus 2 (TNDM2) [MIM:610374]
ABCC8 Q09428 VAR_029783 p.Arg1182Gln LP/P rs193922400 Transient neonatal diabetes mellitus 2 (TNDM2) [MIM:610374]
ABCC8 Q09428 VAR_029784 p.Arg1352His LP/P rs28936370 Leucine-induced hypoglycemia (LIH) [MIM:240800]
ABCC8 Q09428 VAR_029785 p.Arg1379Cys LP/P rs137852673 Transient neonatal diabetes mellitus 2 (TNDM2) [MIM:610374]
ABCC8 Q09428 VAR_029787 p.Ile1424Val LP/P rs80356653 Diabetes mellitus, permanent neonatal, 3 (PNDM3) [MIM:618857]
ABCC8 Q09428 VAR_031349 p.Gly7Arg LP/P rs781059815 Familial hyperinsulinemic hypoglycemia 1 (HHF1) [MIM:256450]
ABCC8 Q09428 VAR_031350 p.Val21Asp LP/P rs200670692 Familial hyperinsulinemic hypoglycemia 1 (HHF1) [MIM:256450]
ABCC8 Q09428 VAR_031351 p.Phe27Ser LP/P - Familial hyperinsulinemic hypoglycemia 1 (HHF1) [MIM:256450]
ABCC8 Q09428 VAR_031352 p.Gly70Glu LP/P - Familial hyperinsulinemic hypoglycemia 1 (HHF1) [MIM:256450]
ABCC8 Q09428 VAR_031353 p.Arg74Trp LP/P rs201682634 Familial hyperinsulinemic hypoglycemia 1 (HHF1) [MIM:256450]
ABCC8 Q09428 VAR_031354 p.Val86Ala LP/P rs193929360 Diabetes mellitus, permanent neonatal, 3 (PNDM3) [MIM:618857]
ABCC8 Q09428 VAR_031355 p.Gly111Arg LP/P rs761749884 Familial hyperinsulinemic hypoglycemia 1 (HHF1) [MIM:256450]
ABCC8 Q09428 VAR_031356 p.Ala116Pro LP/P rs72559731 Familial hyperinsulinemic hypoglycemia 1 (HHF1) [MIM:256450]
ABCC8 Q09428 VAR_031357 p.Met233Arg LP/P - Familial hyperinsulinemic hypoglycemia 1 (HHF1) [MIM:256450]
ABCC8 Q09428 VAR_031358 p.Asp310Asn LP/P rs769569410 Familial hyperinsulinemic hypoglycemia 1 (HHF1) [MIM:256450]
ABCC8 Q09428 VAR_031359 p.Cys418Arg LP/P rs67254669 Familial hyperinsulinemic hypoglycemia 1 (HHF1) [MIM:256450]
ABCC8 Q09428 VAR_031360 p.Arg495Gln LP/P rs1420601296 Familial hyperinsulinemic hypoglycemia 1 (HHF1) [MIM:256450]
ABCC8 Q09428 VAR_031361 p.Glu501Lys LP/P rs372307320 Familial hyperinsulinemic hypoglycemia 1 (HHF1) [MIM:256450]
ABCC8 Q09428 VAR_031362 p.Leu503Pro LP/P rs1554933168 Familial hyperinsulinemic hypoglycemia 1 (HHF1) [MIM:256450]

ABCC8 Q09428 VAR_031363 p.Leu508Pro LP/P rs72559727 Familial hyperinsulinemic hypoglycemia 1 (HHF1) [MIM:256450]
ABCC8 Q09428 VAR_031364 p.Pro551Arg LP/P - Familial hyperinsulinemic hypoglycemia 1 (HHF1) [MIM:256450]
ABCC8 Q09428 VAR_031365 p.Arg620Cys LP/P rs58241708 Familial hyperinsulinemic hypoglycemia 1 (HHF1) [MIM:256450]
ABCC8 Q09428 VAR_031366 p.Phe686Ser LP/P - Familial hyperinsulinemic hypoglycemia 1 (HHF1) [MIM:256450]
ABCC8 Q09428 VAR_031367 p.Lys719Thr LP/P - Familial hyperinsulinemic hypoglycemia 1 (HHF1) [MIM:256450]
ABCC8 Q09428 VAR_031368 p.Arg841Gly LP/P - Familial hyperinsulinemic hypoglycemia 1 (HHF1) [MIM:256450]
ABCC8 Q09428 VAR_031369 p.Lys889Thr LP/P rs761862121 Familial hyperinsulinemic hypoglycemia 1 (HHF1) [MIM:256450]
ABCC8 Q09428 VAR_031370 p.Ser956Phe LP/P rs72559721 Familial hyperinsulinemic hypoglycemia 1 (HHF1) [MIM:256450]
ABCC8 Q09428 VAR_031371 p.Thr1130Pro LP/P - Familial hyperinsulinemic hypoglycemia 1 (HHF1) [MIM:256450]
ABCC8 Q09428 VAR_031372 p.Leu1147Arg LP/P rs1262517518 Familial hyperinsulinemic hypoglycemia 1 (HHF1) [MIM:256450]
ABCC8 Q09428 VAR_031373 p.Arg1214Trp LP/P rs139964066 Familial hyperinsulinemic hypoglycemia 1 (HHF1) [MIM:256450]
ABCC8 Q09428 VAR_031374 p.Asn1295Lys LP/P rs542157938 Familial hyperinsulinemic hypoglycemia 1 (HHF1) [MIM:256450]
ABCC8 Q09428 VAR_031375 p.Lys1336Asn LP/P rs67767715 Familial hyperinsulinemic hypoglycemia 1 (HHF1) [MIM:256450]
ABCC8 Q09428 VAR_031376 p.Gly1342Glu LP/P - Familial hyperinsulinemic hypoglycemia 1 (HHF1) [MIM:256450]
ABCC8 Q09428 VAR_031377 p.Leu1349Gln LP/P - Familial hyperinsulinemic hypoglycemia 1 (HHF1) [MIM:256450]
ABCC8 Q09428 VAR_031378 p.Lys1384Gln LP/P - Familial hyperinsulinemic hypoglycemia 1 (HHF1) [MIM:256450]
ABCC8 Q09428 VAR_031379 p.Ser1386Phe LP/P rs72559718 Familial hyperinsulinemic hypoglycemia 1 (HHF1) [MIM:256450]
ABCC8 Q09428 VAR_031380 p.Gly1400Arg LP/P rs137852676 Diabetes mellitus, permanent neonatal, 3 (PNDM3) [MIM:618857]
ABCC8 Q09428 VAR_031380 p.Gly1400Arg LP/P rs137852676 Familial hyperinsulinemic hypoglycemia 1 (HHF1) [MIM:256450]
ABCC8 Q09428 VAR_031381 p.Arg1418His LP/P rs1446306735 Familial hyperinsulinemic hypoglycemia 1 (HHF1) [MIM:256450]
ABCC8 Q09428 VAR_031382 p.Leu1450Pro LP/P rs1554904565 Familial hyperinsulinemic hypoglycemia 1 (HHF1) [MIM:256450]
ABCC8 Q09428 VAR_031383 p.Ala1457Thr LP/P rs72559717 Familial hyperinsulinemic hypoglycemia 1 (HHF1) [MIM:256450]
ABCC8 Q09428 VAR_031384 p.Asp1471His LP/P - Familial hyperinsulinemic hypoglycemia 1 (HHF1) [MIM:256450]
ABCC8 Q09428 VAR_031385 p.Asp1471Asn LP/P rs72559716 Familial hyperinsulinemic hypoglycemia 1 (HHF1) [MIM:256450]

ABCC8 Q09428 VAR_031386 p.Arg1486Lys LP/P - Familial hyperinsulinemic hypoglycemia 1 (HHF1) [MIM:256450]
ABCC8 Q09428 VAR_031387 p.Arg1493Gln LP/P rs746480424 Familial hyperinsulinemic hypoglycemia 1 (HHF1) [MIM:256450]
ABCC8 Q09428 VAR_031388 p.Val1550Asp LP/P rs1221760584 Familial hyperinsulinemic hypoglycemia 1 (HHF1) [MIM:256450]
ABCC8 Q09428 VAR_031389 p.Leu1551Val LP/P rs1320740169 Familial hyperinsulinemic hypoglycemia 1 (HHF1) [MIM:256450]
ABCC8 Q09428 VAR_072928 p.Pro45Leu LP/P rs267606623 Diabetes mellitus, permanent neonatal, 3 (PNDM3) [MIM:618857]
ABCC8 Q09428 VAR_072929 p.Asn72Ser LP/P rs80356634 Diabetes mellitus, permanent neonatal, 3 (PNDM3) [MIM:618857]
ABCC8 Q09428 VAR_072930 p.Val86Gly LP/P rs193929360 Diabetes mellitus, permanent neonatal, 3 (PNDM3) [MIM:618857]
ABCC8 Q09428 VAR_072931 p.Phe132Val LP/P rs80356637 Diabetes mellitus, permanent neonatal, 3 (PNDM3) [MIM:618857]
ABCC8 Q09428 VAR_072932 p.Pro207Ser LP/P - Diabetes mellitus, permanent neonatal, 3 (PNDM3) [MIM:618857]
ABCC8 Q09428 VAR_072933 p.Glu208Lys LP/P - Diabetes mellitus, permanent neonatal, 3 (PNDM3) [MIM:618857]
ABCC8 Q09428 VAR_072934 p.Asp209Glu LP/P rs80356640 Diabetes mellitus, permanent neonatal, 3 (PNDM3) [MIM:618857]
ABCC8 Q09428 VAR_072935 p.Gln211Lys LP/P rs193929366 Diabetes mellitus, permanent neonatal, 3 (PNDM3) [MIM:618857]
ABCC8 Q09428 VAR_072936 p.Leu225Pro LP/P rs1048095 Diabetes mellitus, permanent neonatal, 3 (PNDM3) [MIM:618857]
ABCC8 Q09428 VAR_072937 p.Thr229Ile LP/P rs768017509 Diabetes mellitus, permanent neonatal, 3 (PNDM3) [MIM:618857]
ABCC8 Q09428 VAR_072938 p.Tyr263Asp LP/P rs778892038 Diabetes mellitus, permanent neonatal, 3 (PNDM3) [MIM:618857]
ABCC8 Q09428 VAR_072939 p.Glu382Lys LP/P rs80356651 Diabetes mellitus, permanent neonatal, 3 (PNDM3) [MIM:618857]
ABCC8 Q09428 VAR_072940 p.Leu511Met LP/P - Familial hyperinsulinemic hypoglycemia 1 (HHF1) [MIM:256450]
ABCC8 Q09428 VAR_072941 p.Gly716Asp LP/P rs72559723 Familial hyperinsulinemic hypoglycemia 1 (HHF1) [MIM:256450]
ABCC8 Q09428 VAR_072942 p.Glu824Lys LP/P - Familial hyperinsulinemic hypoglycemia 1 (HHF1) [MIM:256450]
ABCC8 Q09428 VAR_072943 p.Leu890Pro LP/P - Familial hyperinsulinemic hypoglycemia 1 (HHF1) [MIM:256450]
ABCC8 Q09428 VAR_072944 p.Ala1184Glu LP/P rs137852675 Diabetes mellitus, permanent neonatal, 3 (PNDM3) [MIM:618857]
ABCC8 Q09428 VAR_072945 p.Glu1326Lys LP/P rs200563930 Diabetes mellitus, permanent neonatal, 3 (PNDM3) [MIM:618857]
ABCC8 Q09428 VAR_072946 p.Gly1378Ser LP/P rs925231098 Familial hyperinsulinemic hypoglycemia 1 (HHF1) [MIM:256450]
ABCC8 Q09428 VAR_072947 p.Ser1388Tyr LP/P - Familial hyperinsulinemic hypoglycemia 1 (HHF1) [MIM:256450]

ABCC8	Q09428	VAR_072948	p.Leu1389Pro	LP/P	-	Familial hyperinsulinemic hypoglycemia 1 (HHF1) [MIM:256450]
ABCC8	Q09428	VAR_072949	p.Ala1457Val	LP/P	-	Familial hyperinsulinemic hypoglycemia 1 (HHF1) [MIM:256450]
ABCC8	Q09428	VAR_072950	p.Asn1480Ile	LP/P	-	Familial hyperinsulinemic hypoglycemia 1 (HHF1) [MIM:256450]
ABCC8	Q09428	VAR_072951	p.Asp1505Glu	LP/P	-	Familial hyperinsulinemic hypoglycemia 1 (HHF1) [MIM:256450]
ABCC8	Q09428	VAR_072952	p.Ile1511Ser	LP/P	-	Familial hyperinsulinemic hypoglycemia 1 (HHF1) [MIM:256450]
ABCC8	Q09428	VAR_072953	p.Val1522Leu	LP/P	-	Diabetes mellitus, permanent neonatal, 3 (PNDM3) [MIM:618857]
ABCC9	O60706	VAR_018483	p.Ala1513Thr	LP/P	rs72559751	Cardiomyopathy, dilated 1O (CMD1O) [MIM:608569]
ABCC9	O60706	VAR_048143	p.Pro1108Ser	LB/B	rs35404804	-
ABCC9	O60706	VAR_066210	p.Thr1547Ile	LP/P	rs387906805	Atrial fibrillation, familial, 12 (ATFB12) [MIM:614050]
ABCC9	O60706	VAR_068485	p.His60Tyr	LP/P	rs387907230	Hypertrichotic osteochondrodysplasia (HTOCD) [MIM:239850]
ABCC9	O60706	VAR_068486	p.Asp207Glu	LP/P	-	Hypertrichotic osteochondrodysplasia (HTOCD) [MIM:239850]
ABCC9	O60706	VAR_068487	p.Gly380Cys	LP/P	rs1165205076	Hypertrichotic osteochondrodysplasia (HTOCD) [MIM:239850]
ABCC9	O60706	VAR_068488	p.Pro432Leu	LP/P	-	Hypertrichotic osteochondrodysplasia (HTOCD) [MIM:239850]
ABCC9	O60706	VAR_068489	p.Ala478Val	LP/P	rs387907211	Hypertrichotic osteochondrodysplasia (HTOCD) [MIM:239850]
ABCC9	O60706	VAR_068490	p.Ser1020Pro	LP/P	rs387907229	Hypertrichotic osteochondrodysplasia (HTOCD) [MIM:239850]
ABCC9	O60706	VAR_068491	p.Phe1039Ser	LP/P	-	Hypertrichotic osteochondrodysplasia (HTOCD) [MIM:239850]
ABCC9	O60706	VAR_068492	p.Cys1043Tyr	LP/P	rs387907210	Hypertrichotic osteochondrodysplasia (HTOCD) [MIM:239850]
ABCC9	O60706	VAR_068493	p.Ser1054Tyr	LP/P	-	Hypertrichotic osteochondrodysplasia (HTOCD) [MIM:239850]
ABCC9	O60706	VAR_068494	p.Arg1116Cys	LP/P	rs387907228	Hypertrichotic osteochondrodysplasia (HTOCD) [MIM:239850]
ABCC9	O60706	VAR_068495	p.Arg1116His	LP/P	rs387907227	Hypertrichotic osteochondrodysplasia (HTOCD) [MIM:239850]
ABCC9	O60706	VAR_068496	p.Arg1154Gln	LP/P	rs387907209	Hypertrichotic osteochondrodysplasia (HTOCD) [MIM:239850]
ABCC9	O60706	VAR_068497	p.Arg1154Trp	LP/P	rs387907208	Hypertrichotic osteochondrodysplasia (HTOCD) [MIM:239850]
ABCC9	O60706	VAR_083082	p.Leu1160Arg	US	rs780799175	-
ABCD1	P33897	VAR_000024	p.Ser98Leu	LP/P	rs1557052294	Adrenoleukodystrophy (ALD) [MIM:300100]
ABCD1	P33897	VAR_000025	p.Arg104Cys	LP/P	-	Adrenoleukodystrophy (ALD) [MIM:300100]
ABCD1	P33897	VAR_000026	p.Arg104His	LP/P	rs1557052302	Adrenoleukodystrophy (ALD) [MIM:300100]
ABCD1	P33897	VAR_000027	p.Thr105Ile	LP/P	-	Adrenoleukodystrophy (ALD) [MIM:300100]
ABCD1	P33897	VAR_000028	p.Leu107Pro	LP/P	rs1569540688	Adrenoleukodystrophy (ALD) [MIM:300100]

ABCD1	P33897	VAR_000029	p.Ser108Trp	LP/P	-	Adrenoleukodystrophy (ALD) [MIM:300100]
ABCD1	P33897	VAR_000030	p.Gly116Arg	LP/P	rs398123110	Adrenoleukodystrophy (ALD) [MIM:300100]
ABCD1	P33897	VAR_000033	p.Ala141Thr	LP/P	rs193922097	Adrenoleukodystrophy (ALD) [MIM:300100]
ABCD1	P33897	VAR_000034	p.Asn148Ser	LP/P	rs128624216	Adrenoleukodystrophy (ALD) [MIM:300100]
ABCD1	P33897	VAR_000035	p.Ser149Asn	LP/P	-	Adrenoleukodystrophy (ALD) [MIM:300100]
ABCD1	P33897	VAR_000036	p.Arg152Cys	LP/P	rs1569540693	Adrenoleukodystrophy (ALD) [MIM:300100]
ABCD1	P33897	VAR_000037	p.Arg152Pro	LP/P	-	Adrenoleukodystrophy (ALD) [MIM:300100]
ABCD1	P33897	VAR_000038	p.Arg163His	LP/P	rs1057517954	Adrenoleukodystrophy (ALD) [MIM:300100]
ABCD1	P33897	VAR_000039	p.Tyr174Asp	LP/P	rs128624217	Adrenoleukodystrophy (ALD) [MIM:300100]
ABCD1	P33897	VAR_000040	p.Tyr174Ser	LP/P	rs1557052390	Adrenoleukodystrophy (ALD) [MIM:300100]
ABCD1	P33897	VAR_000041	p.Gln178Glu	LP/P	-	Adrenoleukodystrophy (ALD) [MIM:300100]
ABCD1	P33897	VAR_000042	p.Tyr181Cys	LP/P	-	Adrenoleukodystrophy (ALD) [MIM:300100]
ABCD1	P33897	VAR_000043	p.Arg182Pro	LP/P	-	Adrenoleukodystrophy (ALD) [MIM:300100]
ABCD1	P33897	VAR_000044	p.Asp194His	LP/P	-	Adrenoleukodystrophy (ALD) [MIM:300100]
ABCD1	P33897	VAR_000045	p.Asp200Val	LP/P	-	Adrenoleukodystrophy (ALD) [MIM:300100]
ABCD1	P33897	VAR_000046	p.Leu211Pro	LP/P	-	Adrenoleukodystrophy (ALD) [MIM:300100]
ABCD1	P33897	VAR_000047	p.Leu220Pro	LP/P	-	Adrenoleukodystrophy (ALD) [MIM:300100]
ABCD1	P33897	VAR_000048	p.Asp221Gly	LP/P	-	Adrenoleukodystrophy (ALD) [MIM:300100]
ABCD1	P33897	VAR_000049	p.Thr254Met	LP/P	rs1131691743	Adrenoleukodystrophy (ALD) [MIM:300100]
ABCD1	P33897	VAR_000050	p.Thr254Pro	LP/P	-	Adrenoleukodystrophy (ALD) [MIM:300100]
ABCD1	P33897	VAR_000051	p.Pro263Leu	LP/P	-	Adrenoleukodystrophy (ALD) [MIM:300100]
ABCD1	P33897	VAR_000052	p.Gly266Arg	LP/P	rs128624218	Adrenoleukodystrophy (ALD) [MIM:300100]
ABCD1	P33897	VAR_000053	p.Lys276Glu	LP/P	-	Adrenoleukodystrophy (ALD) [MIM:300100]
ABCD1	P33897	VAR_000054	p.Gly277Arg	LP/P	rs1603232195	Adrenoleukodystrophy (ALD) [MIM:300100]
ABCD1	P33897	VAR_000056	p.Gly277Trp	LP/P	-	Adrenoleukodystrophy (ALD) [MIM:300100]
ABCD1	P33897	VAR_000057	p.Glu291Asp	LP/P	-	Adrenoleukodystrophy (ALD) [MIM:300100]
ABCD1	P33897	VAR_000058	p.Glu291Lys	LP/P	rs128624213	Adrenoleukodystrophy (ALD) [MIM:300100]
ABCD1	P33897	VAR_000060	p.Ala294Thr	LP/P	rs1131691954	Adrenoleukodystrophy (ALD) [MIM:300100]
ABCD1	P33897	VAR_000061	p.Ser342Pro	LP/P	-	Adrenoleukodystrophy (ALD) [MIM:300100]
ABCD1	P33897	VAR_000062	p.Arg389Gly	LP/P	rs128624215	Adrenoleukodystrophy (ALD) [MIM:300100]
ABCD1	P33897	VAR_000063	p.Arg389His	LP/P	rs886044777	Adrenoleukodystrophy (ALD) [MIM:300100]
ABCD1	P33897	VAR_000064	p.Arg401Gln	LP/P	rs128624219	Adrenoleukodystrophy (ALD) [MIM:300100]
ABCD1	P33897	VAR_000065	p.Arg418Trp	LP/P	rs128624220	Adrenoleukodystrophy (ALD) [MIM:300100]
ABCD1	P33897	VAR_000066	p.Pro484Arg	LP/P	rs128624214	Adrenoleukodystrophy (ALD) [MIM:300100]
ABCD1	P33897	VAR_000067	p.Gly507Val	LP/P	-	Adrenoleukodystrophy (ALD) [MIM:300100]
ABCD1	P33897	VAR_000068	p.Gly512Ser	LP/P	rs1569541088	Adrenoleukodystrophy (ALD) [MIM:300100]
ABCD1	P33897	VAR_000069	p.Ser515Phe	LP/P	rs128624223	Adrenoleukodystrophy (ALD) [MIM:300100]
ABCD1	P33897	VAR_000070	p.Arg518Gln	LP/P	rs398123102	Adrenoleukodystrophy (ALD) [MIM:300100]
ABCD1	P33897	VAR_000071	p.Arg518Trp	LP/P	rs128624224	Adrenoleukodystrophy (ALD) [MIM:300100]
ABCD1	P33897	VAR_000072	p.Gly522Trp	LP/P	-	Adrenoleukodystrophy (ALD) [MIM:300100]
ABCD1	P33897	VAR_000074	p.Pro534Leu	LP/P	-	Adrenoleukodystrophy (ALD) [MIM:300100]
ABCD1	P33897	VAR_000075	p.Pro560Leu	LP/P	rs398123105	Adrenoleukodystrophy (ALD) [MIM:300100]
ABCD1	P33897	VAR_000076	p.Pro560Arg	LP/P	-	Adrenoleukodystrophy (ALD) [MIM:300100]
ABCD1	P33897	VAR_000077	p.Met566Lys	LP/P	-	Adrenoleukodystrophy (ALD) [MIM:300100]
ABCD1	P33897	VAR_000078	p.Arg591Gln	LP/P	rs1557054873	Adrenoleukodystrophy (ALD) [MIM:300100]
ABCD1	P33897	VAR_000079	p.Ser606Leu	LP/P	rs128624225	Adrenoleukodystrophy (ALD) [MIM:300100]
ABCD1	P33897	VAR_000080	p.Ser606Pro	LP/P	rs201774661	Adrenoleukodystrophy (ALD) [MIM:300100]
ABCD1	P33897	VAR_000081	p.Glu609Gly	LP/P	rs1557055260	Adrenoleukodystrophy (ALD) [MIM:300100]
ABCD1	P33897	VAR_000082	p.Glu609Lys	LP/P	rs150346282	Adrenoleukodystrophy (ALD) [MIM:300100]

ABCD1	P33897	VAR_000083	p.Arg617Cys	LP/P	rs4010613	Adrenoleukodystrophy (ALD) [MIM:300100]
ABCD1	P33897	VAR_000084	p.Arg617Gly	LP/P	-	Adrenoleukodystrophy (ALD) [MIM:300100]
ABCD1	P33897	VAR_000085	p.Arg617His	LP/P	rs11146842	Adrenoleukodystrophy (ALD) [MIM:300100]
ABCD1	P33897	VAR_000086	p.Ala626Thr	LP/P	rs1557055316	Adrenoleukodystrophy (ALD) [MIM:300100]
ABCD1	P33897	VAR_000087	p.Asp629His	LP/P	-	Adrenoleukodystrophy (ALD) [MIM:300100]
ABCD1	P33897	VAR_000089	p.Arg660Trp	LP/P	rs1569541203	Adrenoleukodystrophy (ALD) [MIM:300100]
ABCD1	P33897	VAR_000090	p.Trp679Arg	LP/P	rs1557055405	Adrenoleukodystrophy (ALD) [MIM:300100]
ABCD1	P33897	VAR_009349	p.Glu90Lys	LP/P	-	Adrenoleukodystrophy (ALD) [MIM:300100]
ABCD1	P33897	VAR_009350	p.Ser103Arg	LP/P	-	Adrenoleukodystrophy (ALD) [MIM:300100]
ABCD1	P33897	VAR_009351	p.Thr105Pro	LP/P	-	Adrenoleukodystrophy (ALD) [MIM:300100]
ABCD1	P33897	VAR_009352	p.Ser108Leu	LP/P	-	Adrenoleukodystrophy (ALD) [MIM:300100]
ABCD1	P33897	VAR_009353	p.Arg113Cys	LP/P	rs1557052306	Adrenoleukodystrophy (ALD) [MIM:300100]
ABCD1	P33897	VAR_009354	p.Pro143Ser	LP/P	-	Adrenoleukodystrophy (ALD) [MIM:300100]
ABCD1	P33897	VAR_009355	p.Arg152Leu	LP/P	-	Adrenoleukodystrophy (ALD) [MIM:300100]
ABCD1	P33897	VAR_009356	p.Arg152Ser	LP/P	-	Adrenoleukodystrophy (ALD) [MIM:300100]
ABCD1	P33897	VAR_009357	p.Ser161Pro	LP/P	-	Adrenoleukodystrophy (ALD) [MIM:300100]
ABCD1	P33897	VAR_009358	p.Arg163Pro	LP/P	-	Adrenoleukodystrophy (ALD) [MIM:300100]
ABCD1	P33897	VAR_009359	p.Tyr174Cys	LP/P	rs1557052390	Adrenoleukodystrophy (ALD) [MIM:300100]
ABCD1	P33897	VAR_009360	p.Arg189Trp	LP/P	rs1131691916	Adrenoleukodystrophy (ALD) [MIM:300100]
ABCD1	P33897	VAR_009361	p.Leu190Pro	LP/P	-	Adrenoleukodystrophy (ALD) [MIM:300100]
ABCD1	P33897	VAR_009362	p.Thr198Lys	LP/P	-	Adrenoleukodystrophy (ALD) [MIM:300100]
ABCD1	P33897	VAR_009363	p.Asp200Asn	LP/P	-	Adrenoleukodystrophy (ALD) [MIM:300100]
ABCD1	P33897	VAR_009364	p.Ser213Cys	LP/P	-	Adrenoleukodystrophy (ALD) [MIM:300100]
ABCD1	P33897	VAR_009365	p.Asn214Asp	LP/P	-	Adrenoleukodystrophy (ALD) [MIM:300100]
ABCD1	P33897	VAR_009366	p.Pro218Thr	LP/P	-	Adrenoleukodystrophy (ALD) [MIM:300100]
ABCD1	P33897	VAR_009367	p.Leu229Pro	LP/P	-	Adrenoleukodystrophy (ALD) [MIM:300100]
ABCD1	P33897	VAR_009368	p.Glu271Lys	LP/P	-	Adrenoleukodystrophy (ALD) [MIM:300100]
ABCD1	P33897	VAR_009369	p.Arg285Pro	LP/P	-	Adrenoleukodystrophy (ALD) [MIM:300100]
ABCD1	P33897	VAR_009370	p.Tyr296Cys	LP/P	rs797044610	Adrenoleukodystrophy (ALD) [MIM:300100]
ABCD1	P33897	VAR_009371	p.Gly298Asp	LP/P	-	Adrenoleukodystrophy (ALD) [MIM:300100]
ABCD1	P33897	VAR_009372	p.Glu302Lys	LP/P	-	Adrenoleukodystrophy (ALD) [MIM:300100]
ABCD1	P33897	VAR_009373	p.Leu322Pro	LP/P	-	Adrenoleukodystrophy (ALD) [MIM:300100]
ABCD1	P33897	VAR_009374	p.Lys336Met	LP/P	-	Adrenoleukodystrophy (ALD) [MIM:300100]
ABCD1	P33897	VAR_009375	p.Arg401Trp	LP/P	rs727503786	Adrenoleukodystrophy (ALD) [MIM:300100]
ABCD1	P33897	VAR_009376	p.Gly529Ser	LP/P	-	Adrenoleukodystrophy (ALD) [MIM:300100]
ABCD1	P33897	VAR_009377	p.Phe540Ser	LP/P	-	Adrenoleukodystrophy (ALD) [MIM:300100]
ABCD1	P33897	VAR_009378	p.Pro543Leu	LP/P	rs1557054776	Adrenoleukodystrophy (ALD) [MIM:300100]
ABCD1	P33897	VAR_009379	p.Gln544Arg	LP/P	-	Adrenoleukodystrophy (ALD) [MIM:300100]
ABCD1	P33897	VAR_009380	p.Ser552Pro	LP/P	-	Adrenoleukodystrophy (ALD) [MIM:300100]
ABCD1	P33897	VAR_009381	p.Arg554His	LP/P	rs201568579	Adrenoleukodystrophy (ALD) [MIM:300100]
ABCD1	P33897	VAR_009382	p.Arg591Trp	LP/P	rs398123106	Adrenoleukodystrophy (ALD) [MIM:300100]
ABCD1	P33897	VAR_009383	p.Ala616Val	LP/P	-	Adrenoleukodystrophy (ALD) [MIM:300100]
ABCD1	P33897	VAR_009384	p.Glu630Gly	LP/P	-	Adrenoleukodystrophy (ALD) [MIM:300100]
ABCD1	P33897	VAR_009385	p.Cys631Tyr	LP/P	-	Adrenoleukodystrophy (ALD) [MIM:300100]
ABCD1	P33897	VAR_009386	p.Ser633Arg	LP/P	-	Adrenoleukodystrophy (ALD) [MIM:300100]
ABCD1	P33897	VAR_009387	p.Ser636Ile	LP/P	-	Adrenoleukodystrophy (ALD) [MIM:300100]
ABCD1	P33897	VAR_009388	p.Asp638Tyr	LP/P	-	Adrenoleukodystrophy (ALD) [MIM:300100]
ABCD1	P33897	VAR_009389	p.Ala646Pro	LP/P	-	Adrenoleukodystrophy (ALD) [MIM:300100]
ABCD1	P33897	VAR_009390	p.Leu654Pro	LP/P	-	Adrenoleukodystrophy (ALD) [MIM:300100]

ABCD1	P33897	VAR_009391	p.His667Asp	LP/P	-	Adrenoleukodystrophy (ALD) [MIM:300100]
ABCD1	P33897	VAR_009392	p.Thr668Ile	LP/P	rs1557055398	Adrenoleukodystrophy (ALD) [MIM:300100]
ABCD1	P33897	VAR_009393	p.Thr693Met	LP/P	rs782311214	Adrenoleukodystrophy (ALD) [MIM:300100]
ABCD1	P33897	VAR_013340	p.Asn13Thr	LB/B	rs183021839	-
ABCD1	P33897	VAR_013341	p.Ala99Asp	LP/P	-	Adrenoleukodystrophy (ALD) [MIM:300100]
ABCD1	P33897	VAR_013342	p.Arg113Pro	LP/P	-	Adrenoleukodystrophy (ALD) [MIM:300100]
ABCD1	P33897	VAR_013344	p.Lys217Glu	LP/P	-	Adrenoleukodystrophy (ALD) [MIM:300100]
ABCD1	P33897	VAR_013345	p.Val224Glu	LP/P	-	Adrenoleukodystrophy (ALD) [MIM:300100]
ABCD1	P33897	VAR_013346	p.Arg274Trp	LP/P	rs782760033	Adrenoleukodystrophy (ALD) [MIM:300100]
ABCD1	P33897	VAR_013347	p.Arg280Cys	LP/P	rs193922098	Adrenoleukodystrophy (ALD) [MIM:300100]
ABCD1	P33897	VAR_013349	p.Trp339Arg	LP/P	rs1603233120	Adrenoleukodystrophy (ALD) [MIM:300100]
ABCD1	P33897	VAR_013350	p.Gly343Asp	LP/P	-	Adrenoleukodystrophy (ALD) [MIM:300100]
ABCD1	P33897	VAR_013352	p.Gln556Arg	LP/P	-	Adrenoleukodystrophy (ALD) [MIM:300100]
ABCD1	P33897	VAR_013353	p.Pro560Ser	LP/P	-	Adrenoleukodystrophy (ALD) [MIM:300100]
ABCD1	P33897	VAR_013354	p.Arg591Pro	LP/P	-	Adrenoleukodystrophy (ALD) [MIM:300100]
ABCD1	P33897	VAR_013355	p.Gly608Asp	LP/P	rs78993751	Adrenoleukodystrophy (ALD) [MIM:300100]
ABCD1	P33897	VAR_013356	p.Ala626Asp	LP/P	-	Adrenoleukodystrophy (ALD) [MIM:300100]
ABCD1	P33897	VAR_013357	p.Thr632Ile	LP/P	rs1064793877	Adrenoleukodystrophy (ALD) [MIM:300100]
ABCD1	P33897	VAR_013358	p.Ser633Ile	LP/P	-	Adrenoleukodystrophy (ALD) [MIM:300100]
ABCD1	P33897	VAR_013359	p.Val635Met	LP/P	rs201427153	Adrenoleukodystrophy (ALD) [MIM:300100]
ABCD1	P33897	VAR_013360	p.Arg660Pro	LP/P	-	Adrenoleukodystrophy (ALD) [MIM:300100]
ABCD1	P33897	VAR_023004	p.Cys88Trp	LP/P	-	Adrenoleukodystrophy (ALD) [MIM:300100]
ABCD1	P33897	VAR_023005	p.Gly343Ser	LP/P	-	Adrenoleukodystrophy (ALD) [MIM:300100]
ABCD1	P33897	VAR_023006	p.Leu503Pro	LP/P	-	Adrenoleukodystrophy (ALD) [MIM:300100]
ABCD1	P33897	VAR_023007	p.Ser514Arg	LP/P	-	Adrenoleukodystrophy (ALD) [MIM:300100]
ABCD1	P33897	VAR_067240	p.Thr198Arg	LP/P	-	Adrenoleukodystrophy (ALD) [MIM:300100]
ABCD1	P33897	VAR_067241	p.Gly266Glu	LP/P	-	Adrenoleukodystrophy (ALD) [MIM:300100]
ABCD1	P33897	VAR_067242	p.Leu523Phe	LP/P	rs1159943880	Adrenoleukodystrophy (ALD) [MIM:300100]
ABCD1	P33897	VAR_067243	p.Phe540Cys	LP/P	-	Adrenoleukodystrophy (ALD) [MIM:300100]
ABCD1	P33897	VAR_067244	p.Thr632Pro	LP/P	-	Adrenoleukodystrophy (ALD) [MIM:300100]
ABCD1	P33897	VAR_067245	p.Glu640Lys	LP/P	-	Adrenoleukodystrophy (ALD) [MIM:300100]
ABCD1	P33897	VAR_067246	p.Gly677Asp	LP/P	-	Adrenoleukodystrophy (ALD) [MIM:300100]
ABCD1	P33897	VAR_067328	p.Leu516Pro	LP/P	-	Adrenoleukodystrophy (ALD) [MIM:300100]
ABCD1	P33897	VAR_067329	p.Arg660Gln	LP/P	rs1557055340	Adrenoleukodystrophy (ALD) [MIM:300100]
ABCD1	P33897	VAR_075284	p.Ala95Asp	LP/P	-	Adrenoleukodystrophy (ALD) [MIM:300100]
ABCD1	P33897	VAR_075285	p.Gln316Pro	LP/P	-	Adrenoleukodystrophy (ALD) [MIM:300100]
ABCD2	Q9UBJ2	VAR_062664	p.Gln244Lys	US	-	A pancreatic ductal adenocarcinoma sample
ABCD3	P28288	VAR_000091	p.Gly17Asp	LB/B	rs121917999	-
ABCD4	O14678	VAR_020222	p.Glu368Lys	LB/B	rs3742801	-
ABCD4	O14678	VAR_020778	p.Ala304Thr	LB/B	rs4148077	-
ABCD4	O14678	VAR_048134	p.Val172Ile	LB/B	rs34992370	-
ABCD4	O14678	VAR_048135	p.Thr350Arg	LB/B	rs35073715	-
ABCD4	O14678	VAR_069097	p.Tyr319Cys	LP/P	rs201777056	Methylmalonic aciduria and homocystinuria type cblJ (MAHCJ) [MIM:614857]
ABCE1	P61221	VAR_068914	p.Ser489Cys	LB/B	rs3816497	-
ABCF1	Q8NE71	VAR_048136	p.Asn198Asp	LB/B	rs6902544	-
ABCF3	Q9NUQ8	VAR_027247	p.Pro503Leu	LB/B	rs11706273	-
ABCF3	Q9NUQ8	VAR_027248	p.Arg510His	LB/B	rs9811715	-
ABCG1	P45844	VAR_012279	p.Phe668Leu	LB/B	-	-

ABCG2	Q9UNQ0	VAR_018349	p.Phe431Leu	LB/B	-	-
ABCG2	Q9UNQ0	VAR_018350	p.Phe489Leu	LB/B	rs192169063	-
ABCG2	Q9UNQ0	VAR_020779	p.Val12Met	LB/B	rs2231137	-
ABCG2	Q9UNQ0	VAR_020780	p.Gln141Lys	LB/B	rs2231142	-
ABCG2	Q9UNQ0	VAR_022443	p.Thr316Pro	LB/B	-	-
ABCG2	Q9UNQ0	VAR_022704	p.Gln166Glu	LB/B	rs1061017	-
ABCG2	Q9UNQ0	VAR_022705	p.Ile206Leu	LB/B	rs12721643	-
ABCG2	Q9UNQ0	VAR_022706	p.Phe208Ser	LB/B	rs1061018	-
ABCG2	Q9UNQ0	VAR_022707	p.Ser248Pro	LB/B	rs3116448	-
ABCG2	Q9UNQ0	VAR_022708	p.Phe571Ile	LB/B	rs9282571	-
ABCG2	Q9UNQ0	VAR_022709	p.Asp620Asn	LB/B	rs34783571	-
ABCG2	Q9UNQ0	VAR_030357	p.Asp296His	LB/B	rs41282401	-
ABCG2	Q9UNQ0	VAR_030358	p.Ala528Thr	LB/B	rs45605536	-
ABCG2	Q9UNQ0	VAR_035355	p.Asn590Tyr	LB/B	rs34264773	-
ABCG2	Q9UNQ0	VAR_067363	p.Ser13Leu	LB/B	rs1319203095	-
ABCG2	Q9UNQ0	VAR_067364	p.Arg160Gln	LB/B	rs528655917	-
ABCG2	Q9UNQ0	VAR_067365	p.Gly354Arg	LB/B	rs138606116	-
ABCG2	Q9UNQ0	VAR_067366	p.Ser441Asn	LB/B	rs1354553769	-
ABCG2	Q9UNQ0	VAR_082302	p.Arg147Trp	LB/B	rs372192400	-
ABCG2	Q9UNQ0	VAR_082303	p.Thr153Met	LB/B	rs753759474	-
ABCG2	Q9UNQ0	VAR_082305	p.Phe373Cys	LB/B	rs752626614	-
ABCG2	Q9UNQ0	VAR_082306	p.Thr421Ala	LB/B	rs199854112	-
ABCG2	Q9UNQ0	VAR_082307	p.Thr434Met	LB/B	rs769734146	-
ABCG2	Q9UNQ0	VAR_082308	p.Ser476Pro	LB/B	rs1274428653	-
ABCG2	Q9UNQ0	VAR_082309	p.Ser572Arg	LB/B	rs200894058	-
ABCG4	Q9H172	VAR_048141	p.Pro352Leu	LB/B	rs35060365	-
ABCG5	Q9H222	VAR_012244	p.Glu146Gln	LP/P	rs758551848	Sitosterolemia 2 (STSL2) [MIM:618666]
ABCG5	Q9H222	VAR_012245	p.Arg389His	LP/P	rs119480069	Sitosterolemia 2 (STSL2) [MIM:618666]
ABCG5	Q9H222	VAR_012246	p.Arg419His	LP/P	rs119479067	Sitosterolemia 2 (STSL2) [MIM:618666]
ABCG5	Q9H222	VAR_012247	p.Arg419Pro	LP/P	rs119479067	Sitosterolemia 2 (STSL2) [MIM:618666]
ABCG5	Q9H222	VAR_012248	p.Arg550Ser	LP/P	-	Sitosterolemia 2 (STSL2) [MIM:618666]
ABCG5	Q9H222	VAR_012249	p.Gln604Glu	LB/B	rs6720173	-
ABCG5	Q9H222	VAR_020781	p.Asn437Lys	LP/P	rs575266356	Sitosterolemia 2 (STSL2) [MIM:618666]
ABCG5	Q9H222	VAR_020782	p.Ile523Val	LB/B	rs140899003	-
ABCG5	Q9H222	VAR_020783	p.Cys600Tyr	LB/B	rs779109455	-
ABCG5	Q9H222	VAR_020784	p.Met622Val	LB/B	rs140374206	-
ABCG5	Q9H222	VAR_033457	p.Thr517Ser	LB/B	rs17031672	-
ABCG5	Q9H222	VAR_048142	p.Arg50Cys	LB/B	rs6756629	-
ABCG8	Q9H221	VAR_012250	p.Asp19His	LB/B	rs11887534	-
ABCG8	Q9H221	VAR_012251	p.Tyr54Cys	LB/B	rs4148211	-
ABCG8	Q9H221	VAR_012252	p.Arg184His	LP/P	rs766212636	Sitosterolemia 1 (STSL1) [MIM:210250]
ABCG8	Q9H221	VAR_012253	p.Pro231Thr	LP/P	rs137852993	Sitosterolemia 1 (STSL1) [MIM:210250]
ABCG8	Q9H221	VAR_012254	p.Glu238Lys	US	rs34754243	Sitosterolemia 1 (STSL1) [MIM:210250]
ABCG8	Q9H221	VAR_012255	p.Ala259Val	LB/B	rs35518570	-
ABCG8	Q9H221	VAR_012256	p.Arg263Gln	LP/P	rs137852990	Sitosterolemia 1 (STSL1) [MIM:210250]
ABCG8	Q9H221	VAR_012257	p.Thr400Lys	LB/B	rs4148217	-
ABCG8	Q9H221	VAR_012258	p.Arg405His	LP/P	rs1177309800	Sitosterolemia 1 (STSL1) [MIM:210250]
ABCG8	Q9H221	VAR_012259	p.Leu501Pro	LP/P	rs1233989408	Sitosterolemia 1 (STSL1) [MIM:210250]
ABCG8	Q9H221	VAR_012260	p.Arg543Ser	LP/P	rs201690654	Sitosterolemia 1 (STSL1) [MIM:210250]

ABCG8	Q9H221	VAR_012262	p.Leu572Pro	LP/P	rs769576789	Sitosterolemia 1 (STSL1) [MIM:210250]
ABCG8	Q9H221	VAR_012263	p.Gly574Glu	LP/P	rs1325979386	Sitosterolemia 1 (STSL1) [MIM:210250]
ABCG8	Q9H221	VAR_012264	p.Gly574Arg	LP/P	rs137852988	Sitosterolemia 1 (STSL1) [MIM:210250]
ABCG8	Q9H221	VAR_012265	p.Gly575Arg	LB/B	-	-
ABCG8	Q9H221	VAR_012266	p.Leu596Arg	LP/P	rs137852992	Sitosterolemia 1 (STSL1) [MIM:210250]
ABCG8	Q9H221	VAR_012267	p.Val632Ala	LB/B	rs6544718	-
ABCG8	Q9H221	VAR_020785	p.Tyr641Phe	LB/B	rs145125968	-
ABCG8	Q9H221	VAR_022074	p.Val210Met	LB/B	rs9282574	-
ABCG8	Q9H221	VAR_022075	p.Met655Val	LB/B	rs9282573	-
ABHD1	Q96SE0	VAR_031087	p.Asp137Glu	LB/B	rs6715286	-
ABHD1	Q96SE0	VAR_031088	p.Trp371Cys	LB/B	rs2304678	-
ABHD1	Q96SE0	VAR_052484	p.Pro54Gln	LB/B	rs34127901	-
ABHD10	Q9NUJ1	VAR_031194	p.Ile251Val	LB/B	rs17429033	-
ABHD12	Q8N2K0	VAR_050630	p.Ala349Thr	LB/B	rs746748	-
ABHD12	Q8N2K0	VAR_081589	p.Arg186Pro	US	rs587777604	Polyneuropathy, hearing loss, ataxia, retinitis pigmentosa, and cataract (PHARC) [MIM:612674]
ABHD12	Q8N2K0	VAR_081590	p.Thr202Ile	US	-	Polyneuropathy, hearing loss, ataxia, retinitis pigmentosa, and cataract (PHARC) [MIM:612674]
ABHD12	Q8N2K0	VAR_081591	p.Thr253Arg	LP/P	rs772987424	Polyneuropathy, hearing loss, ataxia, retinitis pigmentosa, and cataract (PHARC) [MIM:612674]
ABHD12	Q8N2K0	VAR_081593	p.His372Gln	US	rs587777602	Polyneuropathy, hearing loss, ataxia, retinitis pigmentosa, and cataract (PHARC) [MIM:612674]
ABHD12B	Q7Z5M8	VAR_019100	p.Phe334Leu	LB/B	rs7154732	-
ABHD12B	Q7Z5M8	VAR_035676	p.Ile282Val	US	rs28564871	A breast cancer sample
ABHD14A	Q9BUJ0	VAR_031390	p.Arg32Gln	LB/B	rs17849626	-
ABHD14A	Q9BUJ0	VAR_031391	p.Cys61Trp	LB/B	rs404527	-
ABHD15	Q6UXT9	VAR_045821	p.Thr334Ala	LB/B	rs542939	-
ABHD16B	Q9H3Z7	VAR_050920	p.Leu10Gln	LB/B	rs2281534	-
ABHD17B	Q5VST6	VAR_031230	p.Gln169Lys	LB/B	rs17854317	-
ABHD17B	Q5VST6	VAR_054080	p.Arg154Lys	LB/B	rs12380380	-
ABHD2	P08910	VAR_031203	p.Arg253Gln	LB/B	rs17851730	-
ABHD3	Q8WU67	VAR_031089	p.Arg3Cys	LB/B	rs17851878	-
ABHD5	Q8WTS1	VAR_023387	p.Glu7Lys	LP/P	rs104893676	Chanarin-Dorfman syndrome (CDS) [MIM:275630]
ABHD5	Q8WTS1	VAR_023388	p.Gln130Pro	LP/P	rs28939077	Chanarin-Dorfman syndrome (CDS) [MIM:275630]
ABHD5	Q8WTS1	VAR_023389	p.Glu260Lys	LP/P	rs28939078	Chanarin-Dorfman syndrome (CDS) [MIM:275630]
ABHD5	Q8WTS1	VAR_037574	p.Ile72Thr	LB/B	rs2302349	-
ABHD5	Q8WTS1	VAR_057953	p.His82Arg	LB/B	rs145548259	-
ABHD5	Q8WTS1	VAR_057954	p.Ser115Gly	LP/P	-	Chanarin-Dorfman syndrome (CDS) [MIM:275630]
ABHD6	Q9BV23	VAR_081595	p.Arg113His	LB/B	rs200333190	-
ABHD6	Q9BV23	VAR_081596	p.Ser148Cys	LB/B	rs11544004	-
ABHD6	Q9BV23	VAR_081597	p.Pro204Leu	LB/B	rs199678322	-
ABHD6	Q9BV23	VAR_081598	p.Thr206Pro	LB/B	rs199696239	-
ABHD6	Q9BV23	VAR_081599	p.Gly231Val	LB/B	rs745824058	-
ABI1	Q8IZP0	VAR_048159	p.Gly331Ala	LB/B	rs2306236	-
ABI3	Q9P2A4	VAR_022030	p.Arg44Gln	LB/B	rs2233369	-
ABI3	Q9P2A4	VAR_060243	p.Ser203Phe	LB/B	rs616338	-

ABI3 Q9P2A4 VAR_060993 p.Phe209Ser LB/B rs616338 -
ABI3BP Q7Z7G0 VAR_079855 p.Asp670Gly US - -
ABL1 P00519 VAR_025043 p.Gly706Val LB/B rs34634745 -
ABL1 P00519 VAR_025044 p.Thr852Pro LB/B rs1588283506 -
ABL1 P00519 VAR_025045 p.Pro900Ser LB/B rs35266696 -
ABL1 P00519 VAR_025046 p.Ser972Leu LB/B rs2229067 -
ABL1 P00519 VAR_032676 p.Arg47Gly US - A lung large cell carcinoma sample
ABL1 P00519 VAR_032677 p.Arg166Lys US - A melanoma sample
ABL1 P00519 VAR_032678 p.Pro810Leu LB/B rs2229071 -
ABL1 P00519 VAR_051692 p.Leu140Pro LB/B rs1064152 -
ABL1 P00519 VAR_051693 p.Lys247Arg LB/B rs34549764 -
ABL1 P00519 VAR_051694 p.Ser968Pro LB/B rs1064165 -
ABL1 P00519 VAR_079482 p.Tyr226Cys LP/P rs1060499547 Congenital heart defects and skeletal malformations syndrome (CHDSKM) [MIM:617602]
ABL1 P00519 VAR_079483 p.Ala337Thr LP/P rs1060499548 Congenital heart defects and skeletal malformations syndrome (CHDSKM) [MIM:617602]
ABL2 P42684 VAR_029232 p.Lys930Arg LB/B rs17277288 -
ABL2 P42684 VAR_029233 p.Val946Met LB/B rs28913889 -
ABL2 P42684 VAR_029234 p.Pro996Arg LB/B rs28913890 -
ABL2 P42684 VAR_029235 p.Ser1085Asn LB/B rs28913891 -
ABL2 P42684 VAR_029236 p.Thr1101Ala LB/B rs28913892 -
ABL2 P42684 VAR_055411 p.Arg78His LB/B rs55655202 -
ABL2 P42684 VAR_055412 p.Glu99Gln US - -
ABL2 P42684 VAR_055413 p.Arg519Ile US - -
ABL2 P42684 VAR_055414 p.Thr769Ser LB/B rs55892721 -
ABLIM1 O14639 VAR_050141 p.Pro434Thr LB/B rs11593544 -
ABLIM1 O14639 VAR_050142 p.Arg637Gly LB/B rs7091419 -
ABLIM2 Q6H8Q1 VAR_062665 p.Gly227Arg US rs757430763 A pancreatic ductal adenocarcinoma sample
ABLIM2 Q6H8Q1 VAR_062666 p.Lys274Met US - A pancreatic ductal adenocarcinoma sample
ABLIM3 O94929 VAR_050143 p.Gly125Asp LB/B rs35907283 -
ABO P16442 VAR_003409 p.Pro156Leu LB/B rs1053878 -
ABO P16442 VAR_003410 p.Arg176Gly LB/B rs7853989 -
ABO P16442 VAR_003411 p.Gly235Ser LB/B rs8176743 -
ABO P16442 VAR_003412 p.Leu266Met LB/B rs8176746 -
ABO P16442 VAR_003413 p.Gly268Ala LB/B rs8176747 -
ABO P16442 VAR_003414 p.Arg352Trp LB/B - -
ABO P16442 VAR_019147 p.Gly35Arg LB/B rs8176696 -
ABO P16442 VAR_019148 p.Val36Phe LB/B rs688976 -
ABO P16442 VAR_019149 p.Arg63His LB/B rs549446 -
ABO P16442 VAR_019150 p.Pro74Ser LB/B rs512770 -
ABO P16442 VAR_019151 p.Arg161His LB/B rs8176738 -
ABO P16442 VAR_019152 p.Arg199Cys LB/B rs8176739 -
ABO P16442 VAR_019153 p.Phe216Ile LB/B rs8176740 -
ABO P16442 VAR_019154 p.Val277Met LB/B rs8176748 -
ABO P16442 VAR_033540 p.Pro257Leu LB/B rs8176745 -
ABO P16442 VAR_033541 p.Gly268Arg LB/B rs41302905 -
ABO P16442 VAR_036738 p.Thr163Met LB/B rs55756402 -
ABO P16442 VAR_036739 p.Arg198Trp LB/B - -
ABO P16442 VAR_036740 p.Met214Arg LB/B - -

ABO P16442 VAR_036741 p.Glu223Asp LB/B - -
ABO P16442 VAR_036742 p.Met288Arg LB/B - -
ABO P16442 VAR_036743 p.Asp291Asn LB/B - -
ABO P16442 VAR_036744 p.Lys346Met LB/B - -
ABO P16442 VAR_036745 p.Arg352Gly LB/B - -
ABO P16442 VAR_055227 p.Gly230Arg US - -
ABO P16442 VAR_072628 p.Pro234Ala LB/B - -
ABR Q12979 VAR_057186 p.Lys517Arg LB/B rs34169260 -
ABRAXAS1 Q6UWZ7 VAR_030790 p.Ala239Thr LB/B rs752929794 -
ABRAXAS1 Q6UWZ7 VAR_054054 p.Ala348Thr LB/B rs12642536 -
ABRAXAS1 Q6UWZ7 VAR_054055 p.Asp373Asn LB/B rs13125836 -
ABRAXAS1 Q6UWZ7 VAR_071865 p.Arg361Gln LP/P rs201627097 Breast cancer (BC) [MIM:114480]
ABTB2 Q8N961 VAR_022087 p.His250Gln LB/B rs1925368 -
ABTB2 Q8N961 VAR_024171 p.Thr918Ala LB/B rs2473928 -
ACAA1 P09110 VAR_011904 p.Glu172Asp LB/B rs156265 -
ACAA1 P09110 VAR_069148 p.Val387Ala LB/B rs2229528 -
ACAA2 P42765 VAR_052577 p.Met217Val LB/B rs11549285 -
ACACA Q13085 VAR_028929 p.Ala2271Val LB/B rs146351326 -
ACACA Q13085 VAR_036514 p.Arg1687Gln US rs1357271377 A colorectal cancer sample
ACACA Q13085 VAR_042941 p.Arg838Trp LB/B rs2287351 -
ACACB O00763 VAR_031255 p.Ile552Val LB/B rs16940029 -
ACACB O00763 VAR_031256 p.Ala651Thr LB/B rs2300455 -
ACACB O00763 VAR_031257 p.Val2141Ile LB/B rs2075260 -
ACACB O00763 VAR_062667 p.Arg193Leu US - A pancreatic ductal adenocarcinoma sample
ACAD10 Q6JQN1 VAR_031811 p.Gln200Arg LB/B rs35276160 -
ACAD10 Q6JQN1 VAR_031812 p.Thr216Pro LB/B rs35753710 -
ACAD10 Q6JQN1 VAR_031813 p.Asp463Asn LB/B rs36046440 -
ACAD10 Q6JQN1 VAR_031814 p.Ala880Val LB/B rs34245489 -
ACAD11 Q709F0 VAR_028826 p.Val362Leu LB/B rs6776576 -
ACAD8 Q9UKU7 VAR_035071 p.Met128Ile LP/P rs374317179 Isobutyryl-CoA dehydrogenase deficiency (IBDD) [MIM:611283]
ACAD8 Q9UKU7 VAR_035072 p.Asp134Tyr LP/P rs367857040 Isobutyryl-CoA dehydrogenase deficiency (IBDD) [MIM:611283]
ACAD8 Q9UKU7 VAR_035073 p.Gly137Arg LP/P rs371449613 Isobutyryl-CoA dehydrogenase deficiency (IBDD) [MIM:611283]
ACAD8 Q9UKU7 VAR_035074 p.Met152Thr LP/P rs121908418 Isobutyryl-CoA dehydrogenase deficiency (IBDD) [MIM:611283]
ACAD8 Q9UKU7 VAR_035075 p.Val203Ile LP/P rs759877257 Isobutyryl-CoA dehydrogenase deficiency (IBDD) [MIM:611283]
ACAD8 Q9UKU7 VAR_035076 p.Arg302Gln LP/P rs121908422 Isobutyryl-CoA dehydrogenase deficiency (IBDD) [MIM:611283]
ACAD8 Q9UKU7 VAR_035077 p.Ala320Thr LP/P rs200620279 Isobutyryl-CoA dehydrogenase deficiency (IBDD) [MIM:611283]
ACAD8 Q9UKU7 VAR_035078 p.Arg334Cys LP/P rs778823613 Isobutyryl-CoA dehydrogenase deficiency (IBDD) [MIM:611283]
ACAD8 Q9UKU7 VAR_035079 p.Gln385Arg LP/P rs367996531 Isobutyryl-CoA dehydrogenase deficiency (IBDD) [MIM:611283]
ACAD9 Q9H845 VAR_033459 p.Arg477Gln LB/B rs4494951 -

ACAD9 Q9H845 VAR_071892 p.Phe44Ile LP/P rs387907041 Mitochondrial complex I deficiency, nuclear type 20 (MC1DN20) [MIM:611126]
ACAD9 Q9H845 VAR_071893 p.Arg127Lys LP/P - Mitochondrial complex I deficiency, nuclear type 20 (MC1DN20) [MIM:611126]
ACAD9 Q9H845 VAR_071894 p.Arg193Trp US rs377547811 Mitochondrial complex I deficiency, nuclear type 20 (MC1DN20) [MIM:611126]
ACAD9 Q9H845 VAR_071895 p.Ala220Val LP/P - Mitochondrial complex I deficiency, nuclear type 20 (MC1DN20) [MIM:611126]
ACAD9 Q9H845 VAR_071896 p.Ser234Phe US - Mitochondrial complex I deficiency, nuclear type 20 (MC1DN20) [MIM:611126]
ACAD9 Q9H845 VAR_071897 p.Arg266Gln LP/P rs387907042 Mitochondrial complex I deficiency, nuclear type 20 (MC1DN20) [MIM:611126]
ACAD9 Q9H845 VAR_071898 p.Gly303Ser US rs143383023 Mitochondrial complex I deficiency, nuclear type 20 (MC1DN20) [MIM:611126]
ACAD9 Q9H845 VAR_071899 p.Ala326Thr US rs115532916 Mitochondrial complex I deficiency, nuclear type 20 (MC1DN20) [MIM:611126]
ACAD9 Q9H845 VAR_071900 p.Glu413Lys US rs149753643 Mitochondrial complex I deficiency, nuclear type 20 (MC1DN20) [MIM:611126]
ACAD9 Q9H845 VAR_071901 p.Arg414Cys LP/P rs777282696 Mitochondrial complex I deficiency, nuclear type 20 (MC1DN20) [MIM:611126]
ACAD9 Q9H845 VAR_071902 p.Arg417Cys LP/P rs368949613 Mitochondrial complex I deficiency, nuclear type 20 (MC1DN20) [MIM:611126]
ACAD9 Q9H845 VAR_071903 p.Arg469Trp LP/P rs139145143 Mitochondrial complex I deficiency, nuclear type 20 (MC1DN20) [MIM:611126]
ACAD9 Q9H845 VAR_071904 p.Arg518His LP/P rs781149699 Mitochondrial complex I deficiency, nuclear type 20 (MC1DN20) [MIM:611126]
ACAD9 Q9H845 VAR_071905 p.Arg532Trp LP/P rs377022708 Mitochondrial complex I deficiency, nuclear type 20 (MC1DN20) [MIM:611126]
ACAD9 Q9H845 VAR_076177 p.Cys271Gly LP/P - Mitochondrial complex I deficiency, nuclear type 20 (MC1DN20) [MIM:611126]
ACAD9 Q9H845 VAR_076178 p.Val384Met LP/P rs1447947184 Mitochondrial complex I deficiency, nuclear type 20 (MC1DN20) [MIM:611126]
ACAD9 Q9H845 VAR_076179 p.Leu606His LP/P - Mitochondrial complex I deficiency, nuclear type 20 (MC1DN20) [MIM:611126]
ACADL P28330 VAR_000328 p.Ser303Thr LB/B rs1801204 -
ACADL P28330 VAR_000329 p.Lys333Gln LB/B rs2286963 -
ACADM P11310 VAR_000317 p.Arg53Cys LP/P rs398123072 Acyl-CoA dehydrogenase medium-chain deficiency (ACADMD) [MIM:201450]
ACADM P11310 VAR_000319 p.Met149Ile LP/P rs121434277 Acyl-CoA dehydrogenase medium-chain deficiency (ACADMD) [MIM:201450]
ACADM P11310 VAR_000320 p.Thr193Ala LP/P rs121434279 Acyl-CoA dehydrogenase medium-chain deficiency (ACADMD) [MIM:201450]
ACADM P11310 VAR_000321 p.Gly195Arg LP/P rs121434278 Acyl-CoA dehydrogenase medium-chain deficiency (ACADMD) [MIM:201450]
ACADM P11310 VAR_000322 p.Cys244Arg LP/P rs121434276 Acyl-CoA dehydrogenase medium-chain deficiency (ACADMD) [MIM:201450]
ACADM P11310 VAR_000323 p.Gly267Arg LP/P rs121434274 Acyl-CoA dehydrogenase medium-chain deficiency (ACADMD) [MIM:201450]

ACADM P11310 VAR_000324 p.Met326Thr LP/P rs786204631 Acyl-CoA dehydrogenase medium-chain deficiency (ACADMD) [MIM:201450]
ACADM P11310 VAR_000325 p.Lys329Glu LP/P rs77931234 Acyl-CoA dehydrogenase medium-chain deficiency (ACADMD) [MIM:201450]
ACADM P11310 VAR_000326 p.Ser336Arg LP/P - Acyl-CoA dehydrogenase medium-chain deficiency (ACADMD) [MIM:201450]
ACADM P11310 VAR_000327 p.Ile375Thr LP/P rs121434275 Acyl-CoA dehydrogenase medium-chain deficiency (ACADMD) [MIM:201450]
ACADM P11310 VAR_013698 p.Tyr67His LP/P rs121434280 Acyl-CoA dehydrogenase medium-chain deficiency (ACADMD) [MIM:201450]
ACADM P11310 VAR_013699 p.Ser245Leu LP/P rs121434281 Acyl-CoA dehydrogenase medium-chain deficiency (ACADMD) [MIM:201450]
ACADM P11310 VAR_013700 p.Arg281Thr LP/P rs121434282 Acyl-CoA dehydrogenase medium-chain deficiency (ACADMD) [MIM:201450]
ACADM P11310 VAR_015954 p.Ile78Thr LP/P rs398123074 Acyl-CoA dehydrogenase medium-chain deficiency (ACADMD) [MIM:201450]
ACADM P11310 VAR_015955 p.Cys116Tyr LP/P rs875989859 Acyl-CoA dehydrogenase medium-chain deficiency (ACADMD) [MIM:201450]
ACADM P11310 VAR_015956 p.Thr121Ile LP/P rs121434283 Acyl-CoA dehydrogenase medium-chain deficiency (ACADMD) [MIM:201450]
ACADM P11310 VAR_015957 p.Arg206Leu LP/P rs200724875 Acyl-CoA dehydrogenase medium-chain deficiency (ACADMD) [MIM:201450]
ACADM P11310 VAR_015958 p.Gly310Arg LP/P rs747268471 Acyl-CoA dehydrogenase medium-chain deficiency (ACADMD) [MIM:201450]
ACADM P11310 VAR_015959 p.Tyr352Cys LP/P rs1227800781 Acyl-CoA dehydrogenase medium-chain deficiency (ACADMD) [MIM:201450]
ACADM P11310 VAR_035716 p.Pro132Arg US rs875989854 A breast cancer sample
ACADS P16219 VAR_000310 p.Arg46Trp LP/P rs121908003 Acyl-CoA dehydrogenase short-chain deficiency (ACADSD) [MIM:201470]
ACADS P16219 VAR_000311 p.Gly92Cys LP/P rs121908004 Acyl-CoA dehydrogenase short-chain deficiency (ACADSD) [MIM:201470]
ACADS P16219 VAR_000312 p.Arg107Cys LP/P rs61732144 Acyl-CoA dehydrogenase short-chain deficiency (ACADSD) [MIM:201470]
ACADS P16219 VAR_000314 p.Trp177Arg LP/P rs57443665 Acyl-CoA dehydrogenase short-chain deficiency (ACADSD) [MIM:201470]
ACADS P16219 VAR_000315 p.Gly209Ser LB/B rs1799958 -
ACADS P16219 VAR_000316 p.Arg383Cys LP/P rs28940872 Acyl-CoA dehydrogenase short-chain deficiency (ACADSD) [MIM:201470]
ACADS P16219 VAR_013565 p.Gly90Ser LP/P rs121908005 Acyl-CoA dehydrogenase short-chain deficiency (ACADSD) [MIM:201470]
ACADS P16219 VAR_013567 p.Arg171Trp LB/B rs1800556 -
ACADS P16219 VAR_013568 p.Ala192Val LP/P rs28940874 Acyl-CoA dehydrogenase short-chain deficiency (ACADSD) [MIM:201470]
ACADS P16219 VAR_013569 p.Arg325Trp LP/P rs121908006 Acyl-CoA dehydrogenase short-chain deficiency (ACADSD) [MIM:201470]
ACADS P16219 VAR_013570 p.Ser353Leu LP/P rs28941773 Acyl-CoA dehydrogenase short-chain deficiency (ACADSD) [MIM:201470]
ACADS P16219 VAR_013571 p.Arg380Trp LP/P rs28940875 Acyl-CoA dehydrogenase short-chain deficiency (ACADSD) [MIM:201470]

ACADS P16219 VAR_033458 p.Arg383His LB/B rs35233375 -
ACADSB P45954 VAR_013010 p.Leu255Phe LP/P rs137852649 Short/branched-chain acyl-CoA dehydrogenase deficiency (SBCADD) [MIM:610006]
ACADSB P45954 VAR_014749 p.Ser209Gly LB/B rs1799823 -
ACADSB P45954 VAR_048177 p.Arg13Lys LB/B rs12263012 -
ACADSB P45954 VAR_048178 p.Ile316Val LB/B rs1131430 -
ACADSB P45954 VAR_048179 p.Glu376Gly LB/B rs12357783 -
ACADVL P49748 VAR_000330 p.Gly43Asp LB/B rs2230178 -
ACADVL P49748 VAR_000332 p.Thr158Asn LP/P - Acyl-CoA dehydrogenase very long-chain deficiency (ACADVLD) [MIM:201475]
ACADVL P49748 VAR_000333 p.Gln159Arg LP/P rs746688190 Acyl-CoA dehydrogenase very long-chain deficiency (ACADVLD) [MIM:201475]
ACADVL P49748 VAR_000334 p.Val174Met LP/P rs369560930 Acyl-CoA dehydrogenase very long-chain deficiency (ACADVLD) [MIM:201475]
ACADVL P49748 VAR_000335 p.Gly185Ser LP/P rs545215807 Acyl-CoA dehydrogenase very long-chain deficiency (ACADVLD) [MIM:201475]
ACADVL P49748 VAR_000336 p.Glu218Lys LP/P rs1432183079 Acyl-CoA dehydrogenase very long-chain deficiency (ACADVLD) [MIM:201475]
ACADVL P49748 VAR_000337 p.Leu243Arg LP/P - Acyl-CoA dehydrogenase very long-chain deficiency (ACADVLD) [MIM:201475]
ACADVL P49748 VAR_000338 p.Lys247Thr LP/P - Acyl-CoA dehydrogenase very long-chain deficiency (ACADVLD) [MIM:201475]
ACADVL P49748 VAR_000339 p.Thr260Met LP/P rs113994168 Acyl-CoA dehydrogenase very long-chain deficiency (ACADVLD) [MIM:201475]
ACADVL P49748 VAR_000341 p.Ala281Asp LP/P - Acyl-CoA dehydrogenase very long-chain deficiency (ACADVLD) [MIM:201475]
ACADVL P49748 VAR_000342 p.Val283Ala LP/P rs113994167 Acyl-CoA dehydrogenase very long-chain deficiency (ACADVLD) [MIM:201475]
ACADVL P49748 VAR_000343 p.Gly290Asp LP/P rs866464446 Acyl-CoA dehydrogenase very long-chain deficiency (ACADVLD) [MIM:201475]
ACADVL P49748 VAR_000344 p.Gly294Glu LP/P rs200573371 Acyl-CoA dehydrogenase very long-chain deficiency (ACADVLD) [MIM:201475]
ACADVL P49748 VAR_000345 p.Lys299Asn LP/P rs774716484 Acyl-CoA dehydrogenase very long-chain deficiency (ACADVLD) [MIM:201475]
ACADVL P49748 VAR_000347 p.Val317Ala LP/P rs398123095 Acyl-CoA dehydrogenase very long-chain deficiency (ACADVLD) [MIM:201475]
ACADVL P49748 VAR_000348 p.Met352Val LP/P - Acyl-CoA dehydrogenase very long-chain deficiency (ACADVLD) [MIM:201475]
ACADVL P49748 VAR_000349 p.Arg366Cys LP/P rs771874163 Acyl-CoA dehydrogenase very long-chain deficiency (ACADVLD) [MIM:201475]
ACADVL P49748 VAR_000350 p.Arg366His LP/P rs112406105 Acyl-CoA dehydrogenase very long-chain deficiency (ACADVLD) [MIM:201475]
ACADVL P49748 VAR_000352 p.Lys382Gln LP/P rs118204015 Acyl-CoA dehydrogenase very long-chain deficiency (ACADVLD) [MIM:201475]
ACADVL P49748 VAR_000353 p.Asp405His LP/P - Acyl-CoA dehydrogenase very long-chain deficiency (ACADVLD) [MIM:201475]
ACADVL P49748 VAR_000354 p.Gly441Asp LP/P rs2309689 Acyl-CoA dehydrogenase very long-chain deficiency (ACADVLD) [MIM:201475]

ACADVL P49748 VAR_000355 p.Arg450His LP/P rs118204016 Acyl-CoA dehydrogenase very long-chain deficiency (ACADVLD) [MIM:201475]
ACADVL P49748 VAR_000356 p.Arg453Gln LP/P rs138058572 Acyl-CoA dehydrogenase very long-chain deficiency (ACADVLD) [MIM:201475]
ACADVL P49748 VAR_000357 p.Asp454Asn LP/P rs1419606204 Acyl-CoA dehydrogenase very long-chain deficiency (ACADVLD) [MIM:201475]
ACADVL P49748 VAR_000358 p.Arg456His LP/P rs794727112 Acyl-CoA dehydrogenase very long-chain deficiency (ACADVLD) [MIM:201475]
ACADVL P49748 VAR_000359 p.Arg459Trp LP/P rs766742117 Acyl-CoA dehydrogenase very long-chain deficiency (ACADVLD) [MIM:201475]
ACADVL P49748 VAR_000360 p.Gly463Glu LP/P rs200366828 Acyl-CoA dehydrogenase very long-chain deficiency (ACADVLD) [MIM:201475]
ACADVL P49748 VAR_000361 p.Arg469Gln LP/P rs398123083 Acyl-CoA dehydrogenase very long-chain deficiency (ACADVLD) [MIM:201475]
ACADVL P49748 VAR_000362 p.Arg469Trp LP/P rs113994170 Acyl-CoA dehydrogenase very long-chain deficiency (ACADVLD) [MIM:201475]
ACADVL P49748 VAR_000363 p.Leu502Pro LP/P - Acyl-CoA dehydrogenase very long-chain deficiency (ACADVLD) [MIM:201475]
ACADVL P49748 VAR_000364 p.Leu602Ile LP/P - Acyl-CoA dehydrogenase very long-chain deficiency (ACADVLD) [MIM:201475]
ACADVL P49748 VAR_000365 p.Arg613Trp LP/P rs118204014 Acyl-CoA dehydrogenase very long-chain deficiency (ACADVLD) [MIM:201475]
ACADVL P49748 VAR_010101 p.Ala213Pro LP/P rs140629318 Acyl-CoA dehydrogenase very long-chain deficiency (ACADVLD) [MIM:201475]
ACADVL P49748 VAR_010102 p.Lys247Glu LP/P rs387906253 Acyl-CoA dehydrogenase very long-chain deficiency (ACADVLD) [MIM:201475]
ACADVL P49748 VAR_010103 p.Phe458Leu LP/P rs118204017 Acyl-CoA dehydrogenase very long-chain deficiency (ACADVLD) [MIM:201475]
ACADVL P49748 VAR_010104 p.Ala490Pro LP/P rs759775666 Acyl-CoA dehydrogenase very long-chain deficiency (ACADVLD) [MIM:201475]
ACADVL P49748 VAR_010105 p.Glu534Lys US rs2230180 Acyl-CoA dehydrogenase very long-chain deficiency (ACADVLD) [MIM:201475]
ACADVL P49748 VAR_010106 p.Arg615Gln LP/P rs148584617 Acyl-CoA dehydrogenase very long-chain deficiency (ACADVLD) [MIM:201475]
ACADVL P49748 VAR_011990 p.Ala359Ser LB/B rs1051701 -
ACADVL P49748 VAR_011991 p.Ser623Phe LB/B rs13383 -
ACADVL P49748 VAR_029286 p.Leu17Phe LB/B rs2230179 -
ACADVL P49748 VAR_048176 p.Pro65Leu LB/B rs28934585 -
ACADVL P49748 VAR_083892 p.Ser583Trp LP/P rs1085307648 Acyl-CoA dehydrogenase very long-chain deficiency (ACADVLD) [MIM:201475]
ACAN P16112 VAR_056152 p.Asp102Glu LB/B rs16942318 -
ACAN P16112 VAR_056153 p.Arg275Gln LB/B rs34949187 -
ACAN P16112 VAR_056154 p.Pro2058Leu LB/B rs35061438 -
ACAN P16112 VAR_056155 p.Ser2120Arg LB/B rs34153007 -
ACAN P16112 VAR_063053 p.Asp2381Asn LP/P rs121913568 Spondyloepimetaphyseal dysplasia, aggrecan type (SEMDAG) [MIM:612813]
ACAN P16112 VAR_063765 p.Val2418Met LP/P rs779794758 Short stature and advanced bone age, with or without early-onset osteoarthritis and/or osteochondritis dissecans (SSOAOD) [MIM:165800]
ACAN P16112 VAR_080159 p.Pro864Leu LB/B rs3743398 -

ACAN	P16112	VAR_080160	p.Pro913Thr	LB/B	rs35430524	-
ACAN	P16112	VAR_080161	p.Ser930Ile	LB/B	rs938608	-
ACAN	P16112	VAR_080162	p.Ser939Thr	LB/B	rs938609	-
ACAN	P16112	VAR_080163	p.Thr1080Ala	LB/B	rs373544100	-
ACAN	P16112	VAR_080164	p.Thr1403Ala	LB/B	rs12899191	-
ACAN	P16112	VAR_080165	p.Glu1508Ala	LB/B	rs2882676	-
ACAN	P16112	VAR_080166	p.Ile1765Val	LB/B	rs4932439	-
ACAN	P16112	VAR_080167	p.Ile2079Val	LB/B	rs1042630	-
ACAN	P16112	VAR_080168	p.Asp2373Glu	LB/B	rs3817428	-
ACAN	P16112	VAR_080169	p.Gln2500Arg	LB/B	rs1126823	-
ACAP1	Q15027	VAR_036178	p.Lys114Arg	US	rs759855054	A breast cancer sample
ACAP1	Q15027	VAR_036179	p.Arg129Gln	US	rs754740225	A colorectal cancer sample
ACAP1	Q15027	VAR_048328	p.Arg68Cys	LB/B	rs35933585	-
ACAP1	Q15027	VAR_048329	p.Arg533Trp	LB/B	rs35019942	-
ACAT1	P24752	VAR_007496	p.Ala5Pro	LB/B	rs3741056	-
ACAT1	P24752	VAR_007498	p.Asn93Ser	LP/P	rs120074145	3-ketothiolase deficiency (3KTD) [MIM:203750]
ACAT1	P24752	VAR_007499	p.Gly152Ala	LP/P	rs762991875	3-ketothiolase deficiency (3KTD) [MIM:203750]
ACAT1	P24752	VAR_007500	p.Asn158Asp	LP/P	rs148639841	3-ketothiolase deficiency (3KTD) [MIM:203750]
ACAT1	P24752	VAR_007501	p.Gly183Arg	LP/P	rs120074141	3-ketothiolase deficiency (3KTD) [MIM:203750]
ACAT1	P24752	VAR_007502	p.Thr297Met	LP/P	rs886041122	3-ketothiolase deficiency (3KTD) [MIM:203750]
ACAT1	P24752	VAR_007503	p.Ala301Pro	LP/P	rs1420321267	3-ketothiolase deficiency (3KTD) [MIM:203750]
ACAT1	P24752	VAR_007504	p.Ile312Thr	LP/P	rs120074146	3-ketothiolase deficiency (3KTD) [MIM:203750]
ACAT1	P24752	VAR_007505	p.Ala333Pro	LP/P	rs120074147	3-ketothiolase deficiency (3KTD) [MIM:203750]
ACAT1	P24752	VAR_007506	p.Gly379Val	LP/P	rs120074143	3-ketothiolase deficiency (3KTD) [MIM:203750]
ACAT1	P24752	VAR_007507	p.Ala380Thr	LP/P	rs120074140	3-ketothiolase deficiency (3KTD) [MIM:203750]
ACAT2	Q9BWD1	VAR_019686	p.Lys211Arg	LB/B	rs25683	-
ACBD3	Q9H3P7	VAR_019615	p.Glu187Asp	LB/B	rs2306120	-
ACBD4	Q8NC06	VAR_055478	p.Pro118Leu	LB/B	rs901754	-
ACBD4	Q8NC06	VAR_059109	p.Arg242Gly	LB/B	rs16939879	-
ACBD5	Q5T8D3	VAR_032301	p.Thr472Met	LB/B	rs7918793	-
ACCS	Q96QU6	VAR_038685	p.Gly221Glu	US	rs35514614	A breast cancer sample
ACCS	Q96QU6	VAR_038686	p.Ser393Leu	US	-	A breast cancer sample
ACCS	Q96QU6	VAR_038687	p.Pro421Leu	LB/B	rs3107275	-
ACCS	Q96QU6	VAR_048227	p.Asp59Asn	LB/B	rs33952257	-
ACCS	Q96QU6	VAR_048228	p.Asp134Glu	LB/B	rs2018795	-
ACCSL	Q4AC99	VAR_060626	p.Cys529Arg	LB/B	rs2074051	-
ACD	Q96AP0	VAR_060224	p.Thr215Met	LB/B	rs72547495	-
ACD	Q96AP0	VAR_060225	p.Val432Ala	LB/B	rs6979	-
ACD	Q96AP0	VAR_075694	p.Pro405Thr	LP/P	rs201441120	Dyskeratosis congenita, autosomal recessive, 7 (DKCB7) [MIM:616553]
ACE	P12821	VAR_011707	p.Ala261Ser	LB/B	rs4303	-
ACE	P12821	VAR_011708	p.Arg561Trp	LB/B	rs4314	-
ACE	P12821	VAR_011709	p.Arg1286Ser	LB/B	rs4364	-
ACE	P12821	VAR_014189	p.Ile1018Thr	LB/B	rs4976	-
ACE	P12821	VAR_014190	p.Phe1051Val	LB/B	rs4977	-
ACE	P12821	VAR_014191	p.Arg1279Gln	LB/B	rs4980	-
ACE	P12821	VAR_014192	p.Gln1296Pro	LB/B	rs4981	-
ACE	P12821	VAR_020053	p.Asp592Gly	LB/B	rs12709426	-
ACE	P12821	VAR_023430	p.Tyr244Cys	LB/B	rs3730025	-

ACE	P12821	VAR_023431	p.Pro351Leu	LB/B	rs2229839	-
ACE	P12821	VAR_023432	p.Thr916Met	LB/B	rs3730043	-
ACE	P12821	VAR_023433	p.Thr1187Met	LB/B	rs12709442	-
ACE	P12821	VAR_023434	p.Pro1228Leu	LB/B	rs121912703	-
ACE	P12821	VAR_029139	p.Ala154Thr	LB/B	rs13306087	-
ACE	P12821	VAR_029140	p.Ala183Thr	LB/B	rs12720754	-
ACE	P12821	VAR_029141	p.Arg379Gln	LB/B	rs13306085	-
ACE	P12821	VAR_029142	p.Val524Ala	LB/B	rs12720746	-
ACE	P12821	VAR_034602	p.Met828Thr	LB/B	rs13306091	-
ACE	P12821	VAR_035434	p.Gly354Arg	LB/B	rs56394458	-
ACE	P12821	VAR_054000	p.Arg260Cys	LB/B	rs4302	-
ACE	P12821	VAR_054001	p.Arg260Leu	LB/B	rs4303	-
ACE	P12821	VAR_074173	p.Asp295Asn	LB/B	rs989500910	-
ACE2	Q9BYF1	VAR_023082	p.Lys26Arg	LB/B	rs4646116	-
ACE2	Q9BYF1	VAR_023083	p.Asn638Ser	LB/B	rs183135788	-
ACE2	Q9BYF1	VAR_083726	p.Ile468Val	LB/B	-	-
ACE2	Q9BYF1	VAR_083727	p.Asn720Asp	LB/B	-	-
ACER2	Q5QJU3	VAR_027150	p.Ala134Val	LB/B	rs10964136	-
ACER3	Q9NUN7	VAR_081205	p.Glu33Gly	LP/P	rs1554988032	Leukodystrophy, progressive, early childhood-onset (PLDECO) [MIM:617762]
ACHE	P22303	VAR_002359	p.His353Asn	LB/B	rs1799805	-
ACHE	P22303	VAR_011934	p.Val333Glu	LB/B	rs8286	-
ACHE	P22303	VAR_021325	p.Arg34Gln	LB/B	rs17881553	-
ACHE	P22303	VAR_021326	p.Pro135Ala	LB/B	rs17885778	-
ACIN1	Q9UKV3	VAR_022031	p.Ile311Met	LB/B	rs3811182	-
ACIN1	Q9UKV3	VAR_022032	p.Ser467Pro	LB/B	rs1885097	-
ACIN1	Q9UKV3	VAR_022033	p.Ser478Phe	LB/B	rs3751501	-
ACIN1	Q9UKV3	VAR_035777	p.Arg1160Gln	US	rs754494408	A colorectal cancer sample
ACIN1	Q9UKV3	VAR_050632	p.Arg257Lys	LB/B	rs11555803	-
ACIN1	Q9UKV3	VAR_061547	p.Ala447Pro	LB/B	rs941719	-
ACKR1	Q16570	VAR_003480	p.Gly42Asp	LB/B	rs12075	-
ACKR1	Q16570	VAR_015068	p.Arg89Cys	LB/B	rs34599082	-
ACKR1	Q16570	VAR_015069	p.Ala100Thr	LB/B	rs13962	-
ACKR1	Q16570	VAR_044116	p.Leu203Gln	LB/B	rs3027020	-
ACKR1	Q16570	VAR_044117	p.Ser326Phe	LB/B	rs17851570	-
ACKR2	O00590	VAR_024252	p.Tyr373Ser	LB/B	rs2228468	-
ACKR2	O00590	VAR_049379	p.Val41Ala	LB/B	rs2228467	-
ACKR2	O00590	VAR_049380	p.Ala248Val	LB/B	rs2228469	-
ACKR2	O00590	VAR_049381	p.Leu311Val	LB/B	rs6779520	-
ACKR3	P25106	VAR_027477	p.Leu219Trp	LB/B	rs10183641	-
ACLY	P53396	VAR_028230	p.Glu175Asp	LB/B	rs2304497	-
ACO1	P21399	VAR_048180	p.Ala395Asp	LB/B	rs3814519	-
ACO1	P21399	VAR_048181	p.Gly486Arg	LB/B	rs34630459	-
ACO1	P21399	VAR_069413	p.Thr318Met	LB/B	rs150373174	-
ACO2	Q99798	VAR_033297	p.Ala768Ser	LB/B	rs1804785	-
ACO2	Q99798	VAR_036572	p.Thr697Asn	US	-	A breast cancer sample
ACO2	Q99798	VAR_067543	p.Ser112Arg	LP/P	rs786200924	Infantile cerebellar-retinal degeneration (ICRD) [MIM:614559]
ACO2	Q99798	VAR_073435	p.Leu74Val	LP/P	rs141772938	Optic atrophy 9 (OPA9) [MIM:616289]

ACO2 Q99798 VAR_073436 p.Gly259Asp LP/P rs786204828 Infantile cerebellar-retinal degeneration (ICRD) [MIM:614559]
ACO2 Q99798 VAR_073437 p.Gly661Arg LP/P rs752034900 Optic atrophy 9 (OPA9) [MIM:616289]
ACO2 Q99798 VAR_073438 p.Lys736Asn LP/P rs786204829 Infantile cerebellar-retinal degeneration (ICRD) [MIM:614559]
ACOT1 Q86TX2 VAR_059830 p.Arg266His LB/B rs1049568 -
ACOT11 Q8WXI4 VAR_022119 p.Pro165Leu LB/B rs2304306 -
ACOT11 Q8WXI4 VAR_022120 p.Gly202Asp LB/B rs1702003 -
ACOT11 Q8WXI4 VAR_022121 p.Met212Ile LB/B rs2304305 -
ACOT11 Q8WXI4 VAR_048190 p.Arg11Trp LB/B rs34630746 -
ACOT11 Q8WXI4 VAR_048191 p.Arg536His LB/B rs12403630 -
ACOT12 Q8WYK0 VAR_048192 p.Val230Ile LB/B rs34607174 -
ACOT12 Q8WYK0 VAR_048193 p.Ala403Thr LB/B rs10371 -
ACOT12 Q8WYK0 VAR_064691 p.Leu190His US - -
ACOT2 P49753 VAR_016136 p.His475Arg LB/B rs7494 -
ACOT2 P49753 VAR_057271 p.Arg16Ser LB/B rs11545741 -
ACOT4 Q8N9L9 VAR_052300 p.Arg57Cys LB/B rs3742819 -
ACOT4 Q8N9L9 VAR_052301 p.Ala187Asp LB/B rs35724886 -
ACOT6 Q3I5F7 VAR_052302 p.Glu166Lys LB/B rs17782052 -
ACOT9 Q9Y305 VAR_062668 p.Asn305His US - A pancreatic ductal adenocarcinoma sample
ACOX1 Q15067 VAR_021529 p.Ile312Met LB/B rs1135640 -
ACOX1 Q15067 VAR_025789 p.Gly178Cys LP/P rs118204091 Adrenoleukodystrophy, pseudoneonatal (Pseudo-NALD) [MIM:264470]
ACOX1 Q15067 VAR_025790 p.Met278Val LP/P rs118204090 Adrenoleukodystrophy, pseudoneonatal (Pseudo-NALD) [MIM:264470]
ACOX1 Q15067 VAR_030619 p.Thr153Ile LB/B rs17855420 -
ACOX1 Q15067 VAR_048182 p.Gly101Ser LB/B rs3744032 -
ACOX1 Q15067 VAR_067041 p.Ser184Leu LP/P rs780887410 Adrenoleukodystrophy, pseudoneonatal (Pseudo-NALD) [MIM:264470]
ACOX1 Q15067 VAR_067042 p.Gly231Val LP/P - Adrenoleukodystrophy, pseudoneonatal (Pseudo-NALD) [MIM:264470]
ACOX1 Q15067 VAR_067043 p.Gln309Arg LP/P rs118204092 Adrenoleukodystrophy, pseudoneonatal (Pseudo-NALD) [MIM:264470]
ACOX1 Q15067 VAR_067044 p.Ser310Pro LP/P rs758962364 Adrenoleukodystrophy, pseudoneonatal (Pseudo-NALD) [MIM:264470]
ACOX1 Q15067 VAR_083893 p.Asn237Ser LP/P rs1567876984 Mitchell syndrome (MITCH) [MIM:618960]
ACOX2 Q99424 VAR_078765 p.Arg225Trp LP/P rs150832314 Congenital bile acid synthesis defect 6 (CBAS6) [MIM:617308]
ACOX3 O15254 VAR_030802 p.Glu34Ala LB/B rs12513296 -
ACOX3 O15254 VAR_030803 p.Asp497Asn LB/B rs13434465 -
ACOXL Q9NUZ1 VAR_035164 p.Thr255Met LB/B rs1554005 -
ACOXL Q9NUZ1 VAR_035165 p.Pro535Leu LB/B rs17041850 -
ACP1 P24666 VAR_006171 p.Gln106Arg LB/B rs79716074 -
ACP1 P24666 VAR_050526 p.Lys7Asn LB/B rs11691572 -
ACP1 P24666 VAR_050527 p.Ser137Phe LB/B rs35569198 -
ACP2 P11117 VAR_027801 p.Arg29Gln LB/B rs2167079 -
ACP2 P11117 VAR_034394 p.Ser320Phe LB/B rs34425282 -
ACP2 P11117 VAR_050519 p.Val402Ile LB/B rs4647764 -
ACP3 P15309 VAR_047960 p.Ser15Asn LB/B rs17850347 -

ACP3 P15309 VAR_047961 p.Phe124Val LB/B rs17856254 -
ACP3 P15309 VAR_047962 p.Trp226Arg LB/B rs17856253 -
ACP3 P15309 VAR_047963 p.Tyr330His LB/B rs17851392 -
ACP3 P15309 VAR_047964 p.Val360Ala LB/B rs17850198 -
ACP4 Q9BZG2 VAR_078014 p.Arg76Cys LP/P rs1057519277 Amelogenesis imperfecta 1J (AI1J) [MIM:617297]
ACP4 Q9BZG2 VAR_078015 p.Arg111Cys LP/P rs202073531 Amelogenesis imperfecta 1J (AI1J) [MIM:617297]
ACP4 Q9BZG2 VAR_078016 p.Ala128Pro US rs767907487 Amelogenesis imperfecta 1J (AI1J) [MIM:617297]
ACP4 Q9BZG2 VAR_078017 p.Glu133Lys US rs779823931 Amelogenesis imperfecta 1J (AI1J) [MIM:617297]
ACP4 Q9BZG2 VAR_078018 p.Ser238Leu LP/P rs763573828 Amelogenesis imperfecta 1J (AI1J) [MIM:617297]
ACP5 P13686 VAR_020602 p.Val148Met LB/B rs2305799 -
ACP5 P13686 VAR_020603 p.Val200Met LB/B rs2229531 -
ACP5 P13686 VAR_029288 p.Val221Ile LB/B rs2229532 -
ACP5 P13686 VAR_065920 p.Lys52Met LP/P - Spondyloenchondrodysplasia with immune dysregulation (SPENCDI) [MIM:607944]
ACP5 P13686 VAR_065921 p.Thr89Ile LP/P rs387906668 Spondyloenchondrodysplasia with immune dysregulation (SPENCDI) [MIM:607944]
ACP5 P13686 VAR_065922 p.Gly109Arg LP/P rs781050795 Spondyloenchondrodysplasia with immune dysregulation (SPENCDI) [MIM:607944]
ACP5 P13686 VAR_065923 p.Leu201Pro LP/P rs387906672 Spondyloenchondrodysplasia with immune dysregulation (SPENCDI) [MIM:607944]
ACP5 P13686 VAR_065924 p.Gly215Arg LP/P rs781199182 Spondyloenchondrodysplasia with immune dysregulation (SPENCDI) [MIM:607944]
ACP5 P13686 VAR_065925 p.Asp241Asn LP/P - Spondyloenchondrodysplasia with immune dysregulation (SPENCDI) [MIM:607944]
ACP5 P13686 VAR_065926 p.Asn262His LP/P rs1449857485 Spondyloenchondrodysplasia with immune dysregulation (SPENCDI) [MIM:607944]
ACP5 P13686 VAR_065927 p.Met264Lys LP/P rs387906670 Spondyloenchondrodysplasia with immune dysregulation (SPENCDI) [MIM:607944]
ACP6 Q9NPH0 VAR_022678 p.Met316Val LB/B rs6593795 -
ACR P10323 VAR_011650 p.Leu120Val LB/B rs1064734 -
ACR P10323 VAR_011651 p.Phe166Leu LB/B rs1064735 -
ACRBP Q8NEB7 VAR_050633 p.Thr336Ala LB/B rs3741923 -
ACRV1 P26436 VAR_050680 p.Gly126Arg LB/B rs34788353 -
ACSBG1 Q96GR2 VAR_038314 p.Glu194Val LB/B rs12899901 -
ACSBG1 Q96GR2 VAR_038315 p.Met633Val LB/B rs2304824 -
ACSBG1 Q96GR2 VAR_038316 p.Ala673Val LB/B rs11072735 -
ACSBG2 Q5FVE4 VAR_038317 p.Val143Ala LB/B rs4807840 -
ACSBG2 Q5FVE4 VAR_038318 p.Lys152Arg LB/B rs33937754 -
ACSBG2 Q5FVE4 VAR_038319 p.Gly584Asp LB/B rs17851959 -
ACSBG2 Q5FVE4 VAR_038320 p.Gly586Asp LB/B rs17851960 -
ACSBG2 Q5FVE4 VAR_038321 p.Pro601Arg LB/B rs35609668 -
ACSBG2 Q5FVE4 VAR_038322 p.Arg624Lys LB/B rs17856650 -
ACSBG2 Q5FVE4 VAR_038323 p.Glu626Gln LB/B rs17856651 -
ACSBG2 Q5FVE4 VAR_038324 p.Arg650Ser LB/B rs35605352 -
ACSF2 Q96CM8 VAR_038304 p.Gly75Val LB/B rs17856448 -
ACSF2 Q96CM8 VAR_038305 p.Val316Met LB/B rs3744523 -
ACSF3 Q4G176 VAR_038306 p.Leu2Pro LB/B rs7188200 -
ACSF3 Q4G176 VAR_038307 p.Ala17Pro LB/B rs11547019 -
ACSF3 Q4G176 VAR_038308 p.Val372Met LB/B rs3743979 -

ACSF3 Q4G176 VAR_066504 p.Met198Arg LP/P rs387907121 Combined malonic and methylmalonic aciduria (CMAMMA) [MIM:614265]
ACSF3 Q4G176 VAR_066505 p.Pro243Leu LP/P rs140986055 Combined malonic and methylmalonic aciduria (CMAMMA) [MIM:614265]
ACSF3 Q4G176 VAR_066506 p.Thr358Ile LP/P rs387907120 Combined malonic and methylmalonic aciduria (CMAMMA) [MIM:614265]
ACSF3 Q4G176 VAR_066507 p.Glu359Lys LP/P rs150487794 Combined malonic and methylmalonic aciduria (CMAMMA) [MIM:614265]
ACSF3 Q4G176 VAR_066508 p.Lys462Thr LP/P rs1362504214 Combined malonic and methylmalonic aciduria (CMAMMA) [MIM:614265]
ACSF3 Q4G176 VAR_066510 p.Arg471Gln LP/P rs387907119 Combined malonic and methylmalonic aciduria (CMAMMA) [MIM:614265]
ACSF3 Q4G176 VAR_066511 p.Arg471Trp LP/P rs138680796 Combined malonic and methylmalonic aciduria (CMAMMA) [MIM:614265]
ACSF3 Q4G176 VAR_066512 p.Gly480Ser LP/P - Combined malonic and methylmalonic aciduria (CMAMMA) [MIM:614265]
ACSF3 Q4G176 VAR_066513 p.Arg558Trp LP/P rs141090143 Combined malonic and methylmalonic aciduria (CMAMMA) [MIM:614265]
ACSL3 O95573 VAR_026716 p.Phe551Ser LB/B rs1046032 -
ACSL4 O60488 VAR_013180 p.Arg570Ser LP/P rs122458138 Mental retardation, X-linked 63 (MRX63) [MIM:300387]
ACSL4 O60488 VAR_036376 p.Arg133Cys US rs753267653 A colorectal cancer sample
ACSL4 O60488 VAR_083476 p.Asp379Asn LB/B - -
ACSL5 Q9ULC5 VAR_022117 p.Met182Val LB/B rs3736946 -
ACSL5 Q9ULC5 VAR_036377 p.Lys388Arg US - A colorectal cancer sample
ACSL5 Q9ULC5 VAR_036378 p.Gly466Asp US - A colorectal cancer sample
ACSL5 Q9ULC5 VAR_048240 p.Thr486Ala LB/B rs12254915 -
ACSM1 Q08AH1 VAR_035245 p.Ile479Val LB/B rs8056709 -
ACSM1 Q08AH1 VAR_035246 p.Ile515Thr LB/B rs16970453 -
ACSM1 Q08AH1 VAR_048238 p.Ile272Met LB/B rs16970511 -
ACSM2A Q08AH3 VAR_035247 p.Ser513Leu LB/B rs1133607 -
ACSM2A Q08AH3 VAR_035248 p.Ala561Thr LB/B rs1054977 -
ACSM2A Q08AH3 VAR_058692 p.Val335Leu LB/B rs4643305 -
ACSM2A Q08AH3 VAR_058694 p.Val337Gly LB/B rs4586421 -
ACSM3 Q53FZ2 VAR_035249 p.Leu100Pro LB/B rs5713 -
ACSM3 Q53FZ2 VAR_035250 p.Pro308Thr LB/B rs7196188 -
ACSM3 Q53FZ2 VAR_035251 p.Lys367Asn LB/B rs5716 -
ACSM3 Q53FZ2 VAR_048239 p.Asp270His LB/B rs13306603 -
ACSM4 P0C7M7 VAR_061010 p.Arg481His LB/B rs61584783 -
ACSM5 Q6NUN0 VAR_035252 p.Gln159His LB/B rs559741756 -
ACSM5 Q6NUN0 VAR_035253 p.Pro352Arg LB/B rs8062344 -
ACSM5 Q6NUN0 VAR_035254 p.His360Arg LB/B rs12931877 -
ACSM5 Q6NUN0 VAR_035255 p.Thr533Met LB/B rs554734865 -
ACSM5 Q6NUN0 VAR_055495 p.Arg65Gln LB/B rs9928053 -
ACSM5 Q6NUN0 VAR_055496 p.Glu182Lys LB/B rs7192210 -
ACSM5 Q6NUN0 VAR_061011 p.Met217Val LB/B rs59025904 -
ACSM6 Q6P461 VAR_043606 p.Lys227Arg LB/B rs7090248 -
ACSM6 Q6P461 VAR_063090 p.Glu19Gly LB/B rs591157 -
ACSM6 Q6P461 VAR_063091 p.Cys40Ser LB/B rs11188225 -

ACSS1 Q9NUB1 VAR_048184 p.Val488Met LB/B rs6050249 -
ACTA1 P68133 VAR_011680 p.Gly17Arg LP/P rs121909521 Myopathy, actin, congenital, with excess of thin myofilaments (MPCETM) [MIM:161800]
ACTA1 P68133 VAR_011681 p.Leu96Pro LP/P rs121909519 Nemaline myopathy 3 (NEM3) [MIM:161800]
ACTA1 P68133 VAR_011682 p.Asn117Ser LP/P rs121909520 Nemaline myopathy 3 (NEM3) [MIM:161800]
ACTA1 P68133 VAR_011683 p.Ile138Met LP/P rs121909526 Nemaline myopathy 3 (NEM3) [MIM:161800]
ACTA1 P68133 VAR_011684 p.Val165Leu LP/P rs121909522 Myopathy, actin, congenital, with excess of thin myofilaments (MPCETM) [MIM:161800]
ACTA1 P68133 VAR_011685 p.Glu261Val LP/P rs121909523 Nemaline myopathy 3 (NEM3) [MIM:161800]
ACTA1 P68133 VAR_011686 p.Gly270Cys LP/P rs121909525 Nemaline myopathy 3 (NEM3) [MIM:161800]
ACTA1 P68133 VAR_011687 p.Val372Phe LP/P - Nemaline myopathy 3 (NEM3) [MIM:161800]
ACTA1 P68133 VAR_013470 p.Met134Val LP/P - Nemaline myopathy 3 (NEM3) [MIM:161800]
ACTA1 P68133 VAR_013471 p.Met271Arg LP/P rs1553255360 Nemaline myopathy 3 (NEM3) [MIM:161800]
ACTA1 P68133 VAR_015579 p.His42Tyr LP/P - Nemaline myopathy 3 (NEM3) [MIM:161800]
ACTA1 P68133 VAR_015580 p.Gly184Asp LP/P - Nemaline myopathy 3 (NEM3) [MIM:161800]
ACTA1 P68133 VAR_015581 p.Arg185Gly LP/P - Nemaline myopathy 3 (NEM3) [MIM:161800]
ACTA1 P68133 VAR_015582 p.Arg185Cys LP/P rs1064794287 Nemaline myopathy 3 (NEM3) [MIM:161800]
ACTA1 P68133 VAR_015583 p.Arg258His LP/P - Nemaline myopathy 3 (NEM3) [MIM:161800]
ACTA1 P68133 VAR_015584 p.Gln265Leu LP/P - Nemaline myopathy 3 (NEM3) [MIM:161800]
ACTA1 P68133 VAR_015585 p.Asn282Lys LP/P - Nemaline myopathy 3 (NEM3) [MIM:161800]
ACTA1 P68133 VAR_015586 p.Asp288Gly LP/P - Nemaline myopathy 3 (NEM3) [MIM:161800]
ACTA1 P68133 VAR_015587 p.Ile359Leu LP/P rs121909524 Nemaline myopathy 3 (NEM3) [MIM:161800]
ACTA1 P68133 VAR_032917 p.Leu223Pro LP/P rs121909530 Myopathy, congenital, with fiber-type disproportion (CFTD) [MIM:255310]
ACTA1 P68133 VAR_032918 p.Asp294Val LP/P rs121909529 Myopathy, congenital, with fiber-type disproportion (CFTD) [MIM:255310]
ACTA1 P68133 VAR_032919 p.Pro334Ser LP/P rs121909531 Myopathy, congenital, with fiber-type disproportion (CFTD) [MIM:255310]
ACTA1 P68133 VAR_062424 p.Asp3Tyr LP/P rs121909527 Nemaline myopathy 3 (NEM3) [MIM:161800]
ACTA1 P68133 VAR_062425 p.Asp27Asn LP/P - Nemaline myopathy 3 (NEM3) [MIM:161800]
ACTA1 P68133 VAR_062426 p.Val37Leu LP/P rs1553255521 Nemaline myopathy 3 (NEM3) [MIM:161800]
ACTA1 P68133 VAR_062427 p.Pro40Leu LP/P - Nemaline myopathy 3 (NEM3) [MIM:161800]
ACTA1 P68133 VAR_062428 p.Gln43Arg LP/P - Nemaline myopathy 3 (NEM3) [MIM:161800]
ACTA1 P68133 VAR_062429 p.Gly44Val LP/P - Nemaline myopathy 3 (NEM3) [MIM:161800]
ACTA1 P68133 VAR_062430 p.Val45Phe LP/P rs398123562 Nemaline myopathy 3 (NEM3) [MIM:161800]
ACTA1 P68133 VAR_062431 p.Ile66Asn LP/P rs1553255502 Nemaline myopathy 3 (NEM3) [MIM:161800]
ACTA1 P68133 VAR_062432 p.Thr68Ile LP/P - Nemaline myopathy 3 (NEM3) [MIM:161800]
ACTA1 P68133 VAR_062433 p.Glu74Lys LP/P - Nemaline myopathy 3 (NEM3) [MIM:161800]
ACTA1 P68133 VAR_062434 p.His75Leu LP/P - Nemaline myopathy 3 (NEM3) [MIM:161800]
ACTA1 P68133 VAR_062435 p.His75Arg LP/P - Nemaline myopathy 3 (NEM3) [MIM:161800]
ACTA1 P68133 VAR_062436 p.Ile77Leu LP/P - Nemaline myopathy 3 (NEM3) [MIM:161800]
ACTA1 P68133 VAR_062437 p.Thr79Ala LP/P - Nemaline myopathy 3 (NEM3) [MIM:161800]
ACTA1 P68133 VAR_062438 p.Glu85Lys LP/P - Nemaline myopathy 3 (NEM3) [MIM:161800]
ACTA1 P68133 VAR_062439 p.Ala116Thr LP/P - Nemaline myopathy 3 (NEM3) [MIM:161800]
ACTA1 P68133 VAR_062440 p.Asn117Thr LP/P - Nemaline myopathy 3 (NEM3) [MIM:161800]
ACTA1 P68133 VAR_062441 p.Arg118His LP/P - Nemaline myopathy 3 (NEM3) [MIM:161800]
ACTA1 P68133 VAR_062442 p.Val136Ala LP/P - Nemaline myopathy 3 (NEM3) [MIM:161800]
ACTA1 P68133 VAR_062443 p.Ala140Pro LP/P - Nemaline myopathy 3 (NEM3) [MIM:161800]
ACTA1 P68133 VAR_062444 p.Leu142Pro LP/P rs1553255482 Nemaline myopathy 3 (NEM3) [MIM:161800]

ACTA1	P68133	VAR_062445	p.Gly148Asp	LP/P	-	Nemaline myopathy 3 (NEM3) [MIM:161800]
ACTA1	P68133	VAR_062446	p.Thr150Asn	LP/P	-	Nemaline myopathy 3 (NEM3) [MIM:161800]
ACTA1	P68133	VAR_062447	p.Asp156Asn	LP/P	-	Nemaline myopathy 3 (NEM3) [MIM:161800]
ACTA1	P68133	VAR_062448	p.Val165Met	LP/P	rs121909522	Nemaline myopathy 3 (NEM3) [MIM:161800]
ACTA1	P68133	VAR_062449	p.Ala172Gly	LP/P	-	Nemaline myopathy 3 (NEM3) [MIM:161800]
ACTA1	P68133	VAR_062450	p.Asp181Gly	LP/P	-	Nemaline myopathy 3 (NEM3) [MIM:161800]
ACTA1	P68133	VAR_062451	p.Asp181His	LP/P	-	Nemaline myopathy 3 (NEM3) [MIM:161800]
ACTA1	P68133	VAR_062452	p.Asp181Asn	LP/P	-	Nemaline myopathy 3 (NEM3) [MIM:161800]
ACTA1	P68133	VAR_062453	p.Arg185Asp	LP/P	-	Nemaline myopathy 3 (NEM3) [MIM:161800]
ACTA1	P68133	VAR_062454	p.Arg185Ser	LP/P	rs1064794287	Nemaline myopathy 3 (NEM3) [MIM:161800]
ACTA1	P68133	VAR_062455	p.Arg198Leu	LP/P	-	Nemaline myopathy 3 (NEM3) [MIM:161800]
ACTA1	P68133	VAR_062456	p.Gly199Ser	LP/P	-	Nemaline myopathy 3 (NEM3) [MIM:161800]
ACTA1	P68133	VAR_062457	p.Glu226Gly	LP/P	-	Nemaline myopathy 3 (NEM3) [MIM:161800]
ACTA1	P68133	VAR_062458	p.Glu226Gln	LP/P	rs1057521118	Nemaline myopathy 3 (NEM3) [MIM:161800]
ACTA1	P68133	VAR_062459	p.Asn227Val	LP/P	-	Nemaline myopathy 3 (NEM3) [MIM:161800]
ACTA1	P68133	VAR_062460	p.Met229Ile	LP/P	-	Nemaline myopathy 3 (NEM3) [MIM:161800]
ACTA1	P68133	VAR_062461	p.Met229Thr	LP/P	-	Nemaline myopathy 3 (NEM3) [MIM:161800]
ACTA1	P68133	VAR_062462	p.Met229Val	LP/P	rs794727714	Nemaline myopathy 3 (NEM3) [MIM:161800]
ACTA1	P68133	VAR_062463	p.Glu243Lys	LP/P	rs367543051	Nemaline myopathy 3 (NEM3) [MIM:161800]
ACTA1	P68133	VAR_062464	p.Gln248Lys	LP/P	-	Nemaline myopathy 3 (NEM3) [MIM:161800]
ACTA1	P68133	VAR_062465	p.Gln248Arg	LP/P	-	Nemaline myopathy 3 (NEM3) [MIM:161800]
ACTA1	P68133	VAR_062466	p.Gly253Asp	LP/P	-	Nemaline myopathy 3 (NEM3) [MIM:161800]
ACTA1	P68133	VAR_062467	p.Arg258Leu	LP/P	-	Nemaline myopathy 3 (NEM3) [MIM:161800]
ACTA1	P68133	VAR_062468	p.Gly270Asp	LP/P	-	Nemaline myopathy 3 (NEM3) [MIM:161800]
ACTA1	P68133	VAR_062469	p.Gly270Arg	LP/P	rs121909525	Nemaline myopathy 3 (NEM3) [MIM:161800]
ACTA1	P68133	VAR_062470	p.Ala274Glu	LP/P	rs1553255357	Nemaline myopathy 3 (NEM3) [MIM:161800]
ACTA1	P68133	VAR_062471	p.Tyr281His	LP/P	-	Nemaline myopathy 3 (NEM3) [MIM:161800]
ACTA1	P68133	VAR_062472	p.Met285Lys	LP/P	-	Nemaline myopathy 3 (NEM3) [MIM:161800]
ACTA1	P68133	VAR_062473	p.Glu336Ala	LP/P	rs121909528	Nemaline myopathy 3 (NEM3) [MIM:161800]
ACTA1	P68133	VAR_062474	p.Lys338Glu	LP/P	-	Nemaline myopathy 3 (NEM3) [MIM:161800]
ACTA1	P68133	VAR_062475	p.Lys338Ile	LP/P	-	Nemaline myopathy 3 (NEM3) [MIM:161800]
ACTA1	P68133	VAR_062476	p.Ser350Leu	LP/P	-	Nemaline myopathy 3 (NEM3) [MIM:161800]
ACTA1	P68133	VAR_062477	p.Arg374Ser	LP/P	-	Nemaline myopathy 3 (NEM3) [MIM:161800]
ACTA1	P68133	VAR_062478	p.Lys375Glu	LP/P	rs1571892209	Nemaline myopathy 3 (NEM3) [MIM:161800]
ACTA1	P68133	VAR_062479	p.Lys375Gln	LP/P	-	Nemaline myopathy 3 (NEM3) [MIM:161800]
ACTA1	P68133	VAR_076426	p.Glu197Asp	LP/P	rs869312739	Myopathy, scapulohumeroperoneal (SHPM) [MIM:616852]
ACTA1	P68133	VAR_076427	p.Lys328Asn	LP/P	rs398122936	Nemaline myopathy 3 (NEM3) [MIM:161800]
ACTA1	P68133	VAR_076428	p.Trp358Cys	LP/P	rs587777354	Nemaline myopathy 3 (NEM3) [MIM:161800]
ACTA1	P68133	VAR_083589	p.Pro72Arg	LP/P	-	Nemaline myopathy 3 (NEM3) [MIM:161800]
ACTA1	P68133	VAR_083590	p.Ala116Val	LP/P	-	Nemaline myopathy 3 (NEM3) [MIM:161800]
ACTA2	P62736	VAR_011944	p.Thr196Ser	LB/B	rs1803028	-
ACTA2	P62736	VAR_011945	p.Thr320Ala	LB/B	rs1803027	-
ACTA2	P62736	VAR_011946	p.His373Pro	LB/B	rs1062398	-
ACTA2	P62736	VAR_045915	p.Asn117Thr	LP/P	-	Aortic aneurysm, familial thoracic 6 (AAT6) [MIM:611788]
ACTA2	P62736	VAR_045916	p.Arg118Gln	LP/P	rs112602953	Aortic aneurysm, familial thoracic 6 (AAT6) [MIM:611788]

ACTA2 P62736 VAR_045917 p.Tyr135His LP/P rs751300489 Aortic aneurysm, familial thoracic 6 (AAT6) [MIM:611788]
ACTA2 P62736 VAR_045918 p.Arg149Cys LP/P rs121434526 Aortic aneurysm, familial thoracic 6 (AAT6) [MIM:611788]
ACTA2 P62736 VAR_045919 p.Val154Ala LP/P rs1554841298 Aortic aneurysm, familial thoracic 6 (AAT6) [MIM:611788]
ACTA2 P62736 VAR_045920 p.Arg258Cys LP/P rs121434528 Aortic aneurysm, familial thoracic 6 (AAT6) [MIM:611788]
ACTA2 P62736 VAR_045921 p.Arg258His LP/P rs121434527 Aortic aneurysm, familial thoracic 6 (AAT6) [MIM:611788]
ACTA2 P62736 VAR_045922 p.Arg292Gly LP/P - Aortic aneurysm, familial thoracic 6 (AAT6) [MIM:611788]
ACTA2 P62736 VAR_045923 p.Thr353Asn LP/P - Aortic aneurysm, familial thoracic 6 (AAT6) [MIM:611788]
ACTA2 P62736 VAR_062577 p.Arg39His LP/P rs794728021 Aortic aneurysm, familial thoracic 6 (AAT6) [MIM:611788]
ACTA2 P62736 VAR_062578 p.Tyr145Cys LP/P - Aortic aneurysm, familial thoracic 6 (AAT6) [MIM:611788]
ACTA2 P62736 VAR_062579 p.Arg185Gln LP/P rs1057521105 Aortic aneurysm, familial thoracic 6 (AAT6) [MIM:611788]
ACTA2 P62736 VAR_062580 p.Arg212Gln LP/P rs397516685 Aortic aneurysm, familial thoracic 6 (AAT6) [MIM:611788]
ACTA2 P62736 VAR_062581 p.Thr326Asn LP/P rs777832794 Aortic aneurysm, familial thoracic 6 (AAT6) [MIM:611788]
ACTA2 P62736 VAR_064516 p.Arg179His LP/P rs387906592 Moyamoya disease 5 (MYMY5) [MIM:614042]
ACTA2 P62736 VAR_064516 p.Arg179His LP/P rs387906592 Multisystemic smooth muscle dysfunction syndrome (MSMDS) [MIM:613834]
ACTB P60709 VAR_030026 p.Arg183Trp LP/P rs104894003 Dystonia, juvenile-onset (DJO) [MIM:607371]
ACTB P60709 VAR_048185 p.Pro243Leu LB/B rs11546899 -
ACTB P60709 VAR_067810 p.Asn12Asp LP/P rs281875331 Baraitser-Winter syndrome 1 (BRWS1) [MIM:243310]
ACTB P60709 VAR_067811 p.Leu65Val LP/P rs281875332 Baraitser-Winter syndrome 1 (BRWS1) [MIM:243310]
ACTB P60709 VAR_067812 p.Arg196Cys LP/P rs281875333 Baraitser-Winter syndrome 1 (BRWS1) [MIM:243310]
ACTB P60709 VAR_067813 p.Arg196His LP/P rs281875334 Baraitser-Winter syndrome 1 (BRWS1) [MIM:243310]
ACTC1 P68032 VAR_012857 p.Glu101Lys LP/P rs193922680 Cardiomyopathy, familial hypertrophic 11 (CMH11) [MIM:612098]
ACTC1 P68032 VAR_012858 p.Pro166Ala LP/P rs267606628 Cardiomyopathy, familial hypertrophic 11 (CMH11) [MIM:612098]
ACTC1 P68032 VAR_012859 p.Ala297Ser LP/P rs121912675 Cardiomyopathy, familial hypertrophic 11 (CMH11) [MIM:612098]
ACTC1 P68032 VAR_012860 p.Arg314His LP/P rs121912673 Cardiomyopathy, dilated 1R (CMD1R) [MIM:613424]
ACTC1 P68032 VAR_012861 p.Ala333Pro LP/P rs267606629 Cardiomyopathy, familial hypertrophic 11 (CMH11) [MIM:612098]
ACTC1 P68032 VAR_012862 p.Glu363Gly LP/P rs121912674 Cardiomyopathy, dilated 1R (CMD1R) [MIM:613424]

ACTC1	P68032	VAR_045924	p.His90Tyr	LP/P	rs121912676	Cardiomyopathy, familial hypertrophic 11 (CMH11) [MIM:612098]
ACTC1	P68032	VAR_045925	p.Arg97Cys	LP/P	rs759495229	Cardiomyopathy, familial hypertrophic 11 (CMH11) [MIM:612098]
ACTC1	P68032	VAR_046502	p.Met125Val	LP/P	rs121912677	Atrial septal defect 5 (ASD5) [MIM:612794]
ACTC1	P68032	VAR_046503	p.Tyr168Cys	LP/P	-	Cardiomyopathy, familial hypertrophic 11 (CMH11) [MIM:612098]
ACTC1	P68032	VAR_046504	p.Met307Leu	LP/P	-	Cardiomyopathy, familial hypertrophic 11 (CMH11) [MIM:612098]
ACTG1	P63261	VAR_032434	p.Thr89Ile	LP/P	rs28999111	Deafness, autosomal dominant, 20 (DFNA20) [MIM:604717]
ACTG1	P63261	VAR_032435	p.Lys118Met	LP/P	rs104894544	Deafness, autosomal dominant, 20 (DFNA20) [MIM:604717]
ACTG1	P63261	VAR_032436	p.Pro264Leu	LP/P	rs104894546	Deafness, autosomal dominant, 20 (DFNA20) [MIM:604717]
ACTG1	P63261	VAR_032437	p.Thr278Ile	LP/P	rs28999112	Deafness, autosomal dominant, 20 (DFNA20) [MIM:604717]
ACTG1	P63261	VAR_032438	p.Pro332Ala	LP/P	rs104894545	Deafness, autosomal dominant, 20 (DFNA20) [MIM:604717]
ACTG1	P63261	VAR_032439	p.Val370Ala	LP/P	rs104894547	Deafness, autosomal dominant, 20 (DFNA20) [MIM:604717]
ACTG1	P63261	VAR_048186	p.Thr160Ile	LB/B	rs11549206	-
ACTG1	P63261	VAR_067814	p.Thr120Ile	LP/P	rs281875325	Baraitser-Winter syndrome 2 (BRWS2) [MIM:614583]
ACTG1	P63261	VAR_067815	p.Ala135Val	LP/P	rs11549190	Baraitser-Winter syndrome 2 (BRWS2) [MIM:614583]
ACTG1	P63261	VAR_067816	p.Ser155Phe	LP/P	rs281875326	Baraitser-Winter syndrome 2 (BRWS2) [MIM:614583]
ACTG1	P63261	VAR_067817	p.Thr203Lys	LP/P	rs281875327	Baraitser-Winter syndrome 2 (BRWS2) [MIM:614583]
ACTG1	P63261	VAR_067818	p.Arg254Trp	LP/P	rs281875328	Baraitser-Winter syndrome 2 (BRWS2) [MIM:614583]
ACTG1	P63261	VAR_067819	p.Arg256Trp	LP/P	rs281875329	Baraitser-Winter syndrome 2 (BRWS2) [MIM:614583]
ACTG1	P63261	VAR_067824	p.Lys118Asn	LP/P	rs267606630	Deafness, autosomal dominant, 20 (DFNA20) [MIM:604717]
ACTG1	P63261	VAR_067825	p.Ile122Val	LP/P	rs281875330	Deafness, autosomal dominant, 20 (DFNA20) [MIM:604717]
ACTG1	P63261	VAR_067826	p.Glu241Lys	LP/P	rs267606631	Deafness, autosomal dominant, 20 (DFNA20) [MIM:604717]
ACTG1	P63261	VAR_079849	p.Pro70Leu	US	-	-
ACTG1	P63261	VAR_079878	p.Asp187His	LP/P	-	Deafness, autosomal dominant, 20 (DFNA20) [MIM:604717]
ACTG1	P63261	VAR_079879	p.Glu316Lys	US	-	Deafness, autosomal dominant, 20 (DFNA20) [MIM:604717]
ACTG2	P63267	VAR_071279	p.Arg40Cys	LP/P	rs587777385	Visceral myopathy (VSCM) [MIM:155310]
ACTG2	P63267	VAR_071280	p.Arg40His	LP/P	rs587777386	Visceral myopathy (VSCM) [MIM:155310]
ACTG2	P63267	VAR_071281	p.Met45Thr	LP/P	rs864309490	Visceral myopathy (VSCM) [MIM:155310]
ACTG2	P63267	VAR_071282	p.Arg63Gly	LP/P	rs864309491	Visceral myopathy (VSCM) [MIM:155310]

ACTG2 P63267 VAR_071283 p.Pro110Leu LP/P - Visceral myopathy (VSCM) [MIM:155310]
ACTG2 P63267 VAR_071284 p.Tyr134Asn LP/P rs587777388 Visceral myopathy (VSCM) [MIM:155310]
ACTG2 P63267 VAR_071285 p.Arg148Ser LP/P rs587777383 Visceral myopathy (VSCM) [MIM:155310]
ACTG2 P63267 VAR_071286 p.Arg178Cys LP/P rs78001248 Visceral myopathy (VSCM) [MIM:155310]
ACTG2 P63267 VAR_071287 p.Arg178His LP/P rs587777384 Visceral myopathy (VSCM) [MIM:155310]
ACTG2 P63267 VAR_071288 p.Arg178Leu LP/P rs587777384 Visceral myopathy (VSCM) [MIM:155310]
ACTG2 P63267 VAR_071289 p.Gly198Asp LP/P rs864309492 Visceral myopathy (VSCM) [MIM:155310]
ACTG2 P63267 VAR_071290 p.Arg257Cys LP/P rs587777387 Visceral myopathy (VSCM) [MIM:155310]
ACTL6A O96019 VAR_079728 p.Glu227Gln US rs1035631360 -
ACTL6A O96019 VAR_079729 p.Arg377Trp LP/P rs868064163 -
ACTL6B O94805 VAR_082123 p.Asp77Gly US rs1562851259 Intellectual developmental disorder with severe speech and ambulation defects (IDDSSAD) [MIM:618470]
ACTL6B O94805 VAR_082125 p.Arg130Gln US rs757603505 Developmental and epileptic encephalopathy 76 (DEE76) [MIM:618468]
ACTL6B O94805 VAR_082128 p.Leu206Pro LP/P rs1562848909 Developmental and epileptic encephalopathy 76 (DEE76) [MIM:618468]
ACTL6B O94805 VAR_082134 p.Gly343Arg LP/P rs1131692228 Intellectual developmental disorder with severe speech and ambulation defects (IDDSSAD) [MIM:618470]
ACTL6B O94805 VAR_082135 p.Gly349Ser LP/P rs955171017 Developmental and epileptic encephalopathy 76 (DEE76) [MIM:618468]
ACTL7A Q9Y615 VAR_024362 p.Val340Met LB/B rs7872077 -
ACTL7A Q9Y615 VAR_031425 p.Arg45Cys LB/B rs368653764 -
ACTL7A Q9Y615 VAR_033460 p.Ala161Pro LB/B rs35995497 -
ACTL7A Q9Y615 VAR_060998 p.Leu343Val LB/B rs56031956 -
ACTL8 Q9H568 VAR_032079 p.Ala3Ser LB/B rs694214 -
ACTL8 Q9H568 VAR_032080 p.Arg245Cys LB/B rs3795322 -
ACTL9 Q8TC94 VAR_043000 p.Phe37Ser LB/B rs2340550 -
ACTL9 Q8TC94 VAR_043001 p.Ala42Asp US - A colorectal cancer sample
ACTL9 Q8TC94 VAR_043002 p.Val51Ala LB/B rs10410943 -
ACTL9 Q8TC94 VAR_043003 p.His227Asn LB/B rs4804079 -
ACTL9 Q8TC94 VAR_043004 p.Ala332Thr US rs782721280 A colorectal cancer sample
ACTN1 P12814 VAR_053883 p.Asn707Thr LB/B rs7157661 -
ACTN1 P12814 VAR_053884 p.Thr868Ser LB/B rs11557769 -
ACTN1 P12814 VAR_069910 p.Gln32Lys LP/P rs387907346 Bleeding disorder, platelet-type 15 (BDPLT15) [MIM:615193]
ACTN1 P12814 VAR_069911 p.Arg46Gln LP/P rs387907348 Bleeding disorder, platelet-type 15 (BDPLT15) [MIM:615193]
ACTN1 P12814 VAR_069912 p.Val105Ile LP/P rs387907345 Bleeding disorder, platelet-type 15 (BDPLT15) [MIM:615193]
ACTN1 P12814 VAR_069913 p.Arg197Trp LB/B rs904887313 -
ACTN1 P12814 VAR_069914 p.Glu225Lys LP/P rs387907350 Bleeding disorder, platelet-type 15 (BDPLT15) [MIM:615193]
ACTN1 P12814 VAR_069915 p.Arg738Trp LP/P rs387907349 Bleeding disorder, platelet-type 15 (BDPLT15) [MIM:615193]
ACTN1 P12814 VAR_069916 p.Arg752Gln LP/P rs387907347 Bleeding disorder, platelet-type 15 (BDPLT15) [MIM:615193]
ACTN2 P35609 VAR_033487 p.Met604Val LB/B rs35997569 -
ACTN2 P35609 VAR_054628 p.Gln9Arg LP/P rs121434525 Cardiomyopathy, dilated 1AA, with or without left ventricular non-compaction (CMD1AA) [MIM:612158]

ACTN2 P35609 VAR_071970 p.Ala119Thr LP/P rs727502886 Cardiomyopathy, dilated 1AA, with or without left ventricular non-compaction (CMD1AA) [MIM:612158]
ACTN2 P35609 VAR_071970 p.Ala119Thr LP/P rs727502886 Cardiomyopathy, familial hypertrophic 23, with or without left ventricular non-compaction (CMH23) [MIM:612158]
ACTN2 P35609 VAR_071971 p.Thr495Met LP/P rs200248944 Cardiomyopathy, familial hypertrophic 23, with or without left ventricular non-compaction (CMH23) [MIM:612158]
ACTN2 P35609 VAR_071972 p.Glu583Ala LP/P rs200631005 Cardiomyopathy, familial hypertrophic 23, with or without left ventricular non-compaction (CMH23) [MIM:612158]
ACTN2 P35609 VAR_071973 p.Glu628Gly LP/P rs786204951 Cardiomyopathy, familial hypertrophic 23, with or without left ventricular non-compaction (CMH23) [MIM:612158]
ACTN2 P35609 VAR_074292 p.Met228Thr LP/P rs786205144 Cardiomyopathy, familial hypertrophic 23, with or without left ventricular non-compaction (CMH23) [MIM:612158]
ACTN2 P35609 VAR_083364 p.Leu131Pro US - Myopathy, distal, 6, adult onset, autosomal dominant (MPD6) [MIM:618655]
ACTN2 P35609 VAR_083365 p.Cys487Arg LP/P - Myopathy, distal, 6, adult onset, autosomal dominant (MPD6) [MIM:618655]
ACTN2 P35609 VAR_083366 p.Leu727Arg LP/P rs1572148902 Myopathy, congenital, with structured cores and Z-line abnormalities (MYOCOZ) [MIM:618654]
ACTN3 Q08043 VAR_012705 p.Arg523Gln LB/B rs1671064 -
ACTN3 Q08043 VAR_033488 p.Glu635Ala LB/B rs2229456 -
ACTN3 Q08043 VAR_047528 p.Cys628Arg LB/B rs618838 -
ACTN3 Q08043 VAR_047529 p.Gln776Arg LB/B rs540874 -
ACTN4 O43707 VAR_010378 p.Lys255Glu LP/P rs121908415 Focal segmental glomerulosclerosis 1 (FSGS1) [MIM:603278]
ACTN4 O43707 VAR_010379 p.Thr259Ile LP/P rs121908416 Focal segmental glomerulosclerosis 1 (FSGS1) [MIM:603278]
ACTN4 O43707 VAR_010380 p.Ser262Pro LP/P rs121908417 Focal segmental glomerulosclerosis 1 (FSGS1) [MIM:603278]
ACTN4 O43707 VAR_072115 p.Ser262Phe LP/P - Focal segmental glomerulosclerosis 1 (FSGS1) [MIM:603278]
ACTN4 O43707 VAR_072116 p.Ala427Thr LP/P rs201128110 Focal segmental glomerulosclerosis 1 (FSGS1) [MIM:603278]
ACTN4 O43707 VAR_072117 p.Asn748Asp LP/P - Focal segmental glomerulosclerosis 1 (FSGS1) [MIM:603278]
ACTN4 O43707 VAR_072118 p.Ala784Val US rs771421233 -
ACTN4 O43707 VAR_072119 p.Gly786Arg LB/B - -
ACTN4 O43707 VAR_072120 p.Pro787Leu LB/B - -
ACTN4 O43707 VAR_072121 p.Pro787Ser LB/B - -
ACTN4 O43707 VAR_072122 p.Cys793Tyr US - -
ACTN4 O43707 VAR_072123 p.Gly798Asp US - -
ACTN4 O43707 VAR_072124 p.Val801Met LB/B rs141727248 -
ACTN4 O43707 VAR_079797 p.Trp59Arg LP/P - Focal segmental glomerulosclerosis 1 (FSGS1) [MIM:603278]
ACTN4 O43707 VAR_079798 p.Glu72Gln LP/P - Focal segmental glomerulosclerosis 1 (FSGS1) [MIM:603278]
ACTN4 O43707 VAR_079799 p.Phe153Leu US - Focal segmental glomerulosclerosis 1 (FSGS1) [MIM:603278]
ACTN4 O43707 VAR_079800 p.Arg310Gln US rs112545413 Focal segmental glomerulosclerosis 1 (FSGS1) [MIM:603278]

ACTR1B P42025 VAR_025315 p.Val93Ala LB/B rs11547231 -
ACTR1B P42025 VAR_048187 p.Ala143Val LB/B rs11692435 -
ACTR3B Q9P1U1 VAR_048188 p.Arg250Gln LB/B rs2260545 -
ACTR5 Q9H9F9 VAR_027158 p.Arg298Leu LB/B rs17853829 -
ACTR5 Q9H9F9 VAR_027159 p.Ile483Val LB/B rs2245231 -
ACTR5 Q9H9F9 VAR_027160 p.Pro580Leu LB/B rs3752289 -
ACTR5 Q9H9F9 VAR_048189 p.Ile461Leu LB/B rs35805905 -
ACTR8 Q9H981 VAR_028033 p.Thr56Ile LB/B rs3733082 -
ACTRT1 Q8TDG2 VAR_080907 p.Glu124Lys US - -
ACTRT2 Q8TDY3 VAR_020416 p.Gly247Arg LB/B rs3795263 -
ACTRT3 Q9BYD9 VAR_055483 p.Glu234Lys LB/B rs2068178 -
ACVR1 Q04771 VAR_028444 p.Arg206His LP/P rs121912678 Fibrodysplasia ossificans progressiva (FOP) [MIM:135100]
ACVR1 Q04771 VAR_041392 p.Ala15Gly LB/B rs13406336 -
ACVR1 Q04771 VAR_041393 p.Ser41Phe LB/B rs55957214 -
ACVR1 Q04771 VAR_041394 p.His47Gln LB/B rs34056189 -
ACVR1 Q04771 VAR_041395 p.Pro115Ser US - A melanoma sample
ACVR1 Q04771 VAR_058419 p.Arg202Ile LP/P rs387906591 Fibrodysplasia ossificans progressiva (FOP) [MIM:135100]
ACVR1 Q04771 VAR_058420 p.Gln207Glu LP/P - Fibrodysplasia ossificans progressiva (FOP) [MIM:135100]
ACVR1 Q04771 VAR_058421 p.Gly328Glu LP/P rs387906589 Fibrodysplasia ossificans progressiva (FOP) [MIM:135100]
ACVR1 Q04771 VAR_058422 p.Gly328Arg LP/P rs387906588 Fibrodysplasia ossificans progressiva (FOP) [MIM:135100]
ACVR1 Q04771 VAR_058423 p.Gly328Trp LP/P rs387906588 Fibrodysplasia ossificans progressiva (FOP) [MIM:135100]
ACVR1 Q04771 VAR_058424 p.Gly356Asp LP/P rs121912679 Fibrodysplasia ossificans progressiva (FOP) [MIM:135100]
ACVR1 Q04771 VAR_058425 p.Arg375Pro LP/P rs387906590 Fibrodysplasia ossificans progressiva (FOP) [MIM:135100]
ACVR1B P36896 VAR_011716 p.Leu408Val LB/B rs928906 -
ACVR1B P36896 VAR_041406 p.Phe146Leu LB/B rs34488074 -
ACVR1C Q8NER5 VAR_041407 p.Ile195Thr LB/B rs56188432 -
ACVR1C Q8NER5 VAR_041408 p.Gly216Arg LB/B rs34742924 -
ACVR1C Q8NER5 VAR_041409 p.Trp267Arg US - A lung squamous cell carcinoma sample
ACVR1C Q8NER5 VAR_041410 p.Ile355Val LB/B rs35500979 -
ACVR1C Q8NER5 VAR_041411 p.Ile482Val LB/B rs7594480 -
ACVR2A P27037 VAR_032809 p.Ser258Arg LB/B rs34917571 -
ACVR2A P27037 VAR_032810 p.Asp306Asn US rs764255410 A gastric adenocarcinoma sample
ACVR2A P27037 VAR_064692 p.Ala367Thr US - -
ACVR2B Q13705 VAR_013281 p.Arg40His LP/P rs121434437 Heterotaxy, visceral, 4, autosomal (HTX4) [MIM:613751]
ACVR2B Q13705 VAR_013282 p.Val494Ile LP/P rs121434438 Heterotaxy, visceral, 4, autosomal (HTX4) [MIM:613751]
ACVR2B Q13705 VAR_041396 p.Pro176Arg LB/B rs35882617 -
ACVR2B Q13705 VAR_050594 p.Glu459Asp LB/B rs500611 -
ACVRL1 P37023 VAR_006204 p.Trp50Cys LP/P rs121909285 Telangiectasia, hereditary hemorrhagic, 2 (HHT2) [MIM:600376]

ACVRL1 P37023 VAR_006205 p.Cys51Tyr LP/P rs863223409 Telangiectasia, hereditary hemorrhagic, 2 (HHT2) [MIM:600376]
ACVRL1 P37023 VAR_006206 p.Arg67Gln LP/P rs863223414 Telangiectasia, hereditary hemorrhagic, 2 (HHT2) [MIM:600376]
ACVRL1 P37023 VAR_006207 p.Cys77Trp LP/P - Telangiectasia, hereditary hemorrhagic, 2 (HHT2) [MIM:600376]
ACVRL1 P37023 VAR_006208 p.Asn96Asp LP/P - Telangiectasia, hereditary hemorrhagic, 2 (HHT2) [MIM:600376]
ACVRL1 P37023 VAR_006210 p.Ser333Ile LP/P rs863223413 Telangiectasia, hereditary hemorrhagic, 2 (HHT2) [MIM:600376]
ACVRL1 P37023 VAR_006211 p.Arg374Trp LP/P rs28936401 Telangiectasia, hereditary hemorrhagic, 2 (HHT2) [MIM:600376]
ACVRL1 P37023 VAR_006212 p.Met376Arg LP/P rs28936399 Telangiectasia, hereditary hemorrhagic, 2 (HHT2) [MIM:600376]
ACVRL1 P37023 VAR_006213 p.Arg411Gln LP/P rs121909284 Telangiectasia, hereditary hemorrhagic, 2 (HHT2) [MIM:600376]
ACVRL1 P37023 VAR_006214 p.Pro424Thr LP/P rs1085307419 Telangiectasia, hereditary hemorrhagic, 2 (HHT2) [MIM:600376]
ACVRL1 P37023 VAR_011717 p.Ile245Asn LB/B rs1804508 -
ACVRL1 P37023 VAR_026785 p.Gly48Arg LP/P - Telangiectasia, hereditary hemorrhagic, 2 (HHT2) [MIM:600376]
ACVRL1 P37023 VAR_026786 p.Arg67Trp LP/P rs1085307405 Telangiectasia, hereditary hemorrhagic, 2 (HHT2) [MIM:600376]
ACVRL1 P37023 VAR_026787 p.Asp179Ala LP/P rs753792569 Telangiectasia, hereditary hemorrhagic, 2 (HHT2) [MIM:600376]
ACVRL1 P37023 VAR_026788 p.Gly211Asp LP/P rs28936687 Telangiectasia, hereditary hemorrhagic, 2 (HHT2) [MIM:600376]
ACVRL1 P37023 VAR_026789 p.Glu215Lys LP/P rs754283265 Telangiectasia, hereditary hemorrhagic, 2 (HHT2) [MIM:600376]
ACVRL1 P37023 VAR_026790 p.Gly223Arg LP/P - Telangiectasia, hereditary hemorrhagic, 2 (HHT2) [MIM:600376]
ACVRL1 P37023 VAR_026791 p.Lys229Arg LP/P - Telangiectasia, hereditary hemorrhagic, 2 (HHT2) [MIM:600376]
ACVRL1 P37023 VAR_026794 p.Leu285Phe LP/P rs1085307410 Telangiectasia, hereditary hemorrhagic, 2 (HHT2) [MIM:600376]
ACVRL1 P37023 VAR_026795 p.Ala306Pro LP/P - Telangiectasia, hereditary hemorrhagic, 2 (HHT2) [MIM:600376]
ACVRL1 P37023 VAR_026796 p.His314Tyr LP/P rs1565594311 Telangiectasia, hereditary hemorrhagic, 2 (HHT2) [MIM:600376]
ACVRL1 P37023 VAR_026797 p.Leu337Pro LP/P rs1592224349 Telangiectasia, hereditary hemorrhagic, 2 (HHT2) [MIM:600376]
ACVRL1 P37023 VAR_026798 p.Cys344Tyr LP/P rs28936688 Telangiectasia, hereditary hemorrhagic, 2 (HHT2) [MIM:600376]
ACVRL1 P37023 VAR_026799 p.Ala347Pro LP/P - Telangiectasia, hereditary hemorrhagic, 2 (HHT2) [MIM:600376]
ACVRL1 P37023 VAR_026800 p.Arg374Gln LP/P rs1060503248 Telangiectasia, hereditary hemorrhagic, 2 (HHT2) [MIM:600376]
ACVRL1 P37023 VAR_026801 p.Met376Val LP/P rs1555153277 Telangiectasia, hereditary hemorrhagic, 2 (HHT2) [MIM:600376]

ACVRL1 P37023 VAR_026802 p.Pro378Leu LP/P - Telangiectasia, hereditary hemorrhagic, 2 (HHT2) [MIM:600376]
ACVRL1 P37023 VAR_026803 p.Glu379Lys LP/P rs1131691686 Telangiectasia, hereditary hemorrhagic, 2 (HHT2) [MIM:600376]
ACVRL1 P37023 VAR_026804 p.Asp397Gly LP/P - Telangiectasia, hereditary hemorrhagic, 2 (HHT2) [MIM:600376]
ACVRL1 P37023 VAR_026805 p.Ile398Asn LP/P rs121909286 Telangiectasia, hereditary hemorrhagic, 2 (HHT2) [MIM:600376]
ACVRL1 P37023 VAR_026806 p.Trp399Ser LP/P rs121909289 Telangiectasia, hereditary hemorrhagic, 2 (HHT2) [MIM:600376]
ACVRL1 P37023 VAR_026807 p.Glu407Asp LP/P rs1565595129 Telangiectasia, hereditary hemorrhagic, 2 (HHT2) [MIM:600376]
ACVRL1 P37023 VAR_026808 p.Arg411Pro LP/P rs121909284 Telangiectasia, hereditary hemorrhagic, 2 (HHT2) [MIM:600376]
ACVRL1 P37023 VAR_026809 p.Arg411Trp LP/P rs121909287 Telangiectasia, hereditary hemorrhagic, 2 (HHT2) [MIM:600376]
ACVRL1 P37023 VAR_026810 p.Phe425Leu LP/P - Telangiectasia, hereditary hemorrhagic, 2 (HHT2) [MIM:600376]
ACVRL1 P37023 VAR_026811 p.Phe425Val LP/P - Telangiectasia, hereditary hemorrhagic, 2 (HHT2) [MIM:600376]
ACVRL1 P37023 VAR_026813 p.Arg479Leu LP/P - Telangiectasia, hereditary hemorrhagic, 2 (HHT2) [MIM:600376]
ACVRL1 P37023 VAR_026814 p.Ala482Val LP/P rs139142865 Telangiectasia, hereditary hemorrhagic, 2 (HHT2) [MIM:600376]
ACVRL1 P37023 VAR_026815 p.Arg484Trp LP/P rs121909288 Telangiectasia, hereditary hemorrhagic, 2 (HHT2) [MIM:600376]
ACVRL1 P37023 VAR_026816 p.Lys487Thr LP/P rs1085307428 Telangiectasia, hereditary hemorrhagic, 2 (HHT2) [MIM:600376]
ACVRL1 P37023 VAR_070308 p.Pro30Ser US rs149664056 -
ACVRL1 P37023 VAR_070309 p.Cys34Tyr LP/P - Telangiectasia, hereditary hemorrhagic, 2 (HHT2) [MIM:600376]
ACVRL1 P37023 VAR_070310 p.Ser38Cys LB/B - -
ACVRL1 P37023 VAR_070311 p.Trp50Gly LP/P - Telangiectasia, hereditary hemorrhagic, 2 (HHT2) [MIM:600376]
ACVRL1 P37023 VAR_070312 p.Thr52Ala LP/P rs1131691346 Telangiectasia, hereditary hemorrhagic, 2 (HHT2) [MIM:600376]
ACVRL1 P37023 VAR_070313 p.His66Pro LP/P - Telangiectasia, hereditary hemorrhagic, 2 (HHT2) [MIM:600376]
ACVRL1 P37023 VAR_070314 p.Cys69Arg LP/P - Telangiectasia, hereditary hemorrhagic, 2 (HHT2) [MIM:600376]
ACVRL1 P37023 VAR_070315 p.Asn96Ser LP/P - Telangiectasia, hereditary hemorrhagic, 2 (HHT2) [MIM:600376]
ACVRL1 P37023 VAR_070316 p.Leu138Pro LB/B - -
ACVRL1 P37023 VAR_070317 p.Asp176Tyr LP/P - Telangiectasia, hereditary hemorrhagic, 2 (HHT2) [MIM:600376]
ACVRL1 P37023 VAR_070318 p.Thr197Ile LP/P - Telangiectasia, hereditary hemorrhagic, 2 (HHT2) [MIM:600376]
ACVRL1 P37023 VAR_070319 p.Trp217Gly LP/P - Telangiectasia, hereditary hemorrhagic, 2 (HHT2) [MIM:600376]

ACVRL1 P37023 VAR_070320 p.Gly219Asp LP/P - Telangiectasia, hereditary hemorrhagic, 2 (HHT2) [MIM:600376]
ACVRL1 P37023 VAR_070321 p.Val226Glu LP/P rs1565593639 Telangiectasia, hereditary hemorrhagic, 2 (HHT2) [MIM:600376]
ACVRL1 P37023 VAR_070322 p.Ser233Leu LP/P rs762773076 Telangiectasia, hereditary hemorrhagic, 2 (HHT2) [MIM:600376]
ACVRL1 P37023 VAR_070323 p.Gln237Lys LP/P - Telangiectasia, hereditary hemorrhagic, 2 (HHT2) [MIM:600376]
ACVRL1 P37023 VAR_070324 p.Ile260Leu LP/P - Telangiectasia, hereditary hemorrhagic, 2 (HHT2) [MIM:600376]
ACVRL1 P37023 VAR_070325 p.Thr265Pro LP/P rs1592223978 Telangiectasia, hereditary hemorrhagic, 2 (HHT2) [MIM:600376]
ACVRL1 P37023 VAR_070326 p.Thr277Lys US - -
ACVRL1 P37023 VAR_070327 p.His280Arg LP/P - Telangiectasia, hereditary hemorrhagic, 2 (HHT2) [MIM:600376]
ACVRL1 P37023 VAR_070328 p.Leu289Pro LP/P - Telangiectasia, hereditary hemorrhagic, 2 (HHT2) [MIM:600376]
ACVRL1 P37023 VAR_070329 p.Leu294Arg LP/P - Telangiectasia, hereditary hemorrhagic, 2 (HHT2) [MIM:600376]
ACVRL1 P37023 VAR_070330 p.His328Gln LP/P rs1565594410 Telangiectasia, hereditary hemorrhagic, 2 (HHT2) [MIM:600376]
ACVRL1 P37023 VAR_070331 p.Asn335His LP/P - Telangiectasia, hereditary hemorrhagic, 2 (HHT2) [MIM:600376]
ACVRL1 P37023 VAR_070332 p.Leu342Pro LB/B rs762287966 -
ACVRL1 P37023 VAR_070333 p.Cys344Arg LP/P rs1592224412 Telangiectasia, hereditary hemorrhagic, 2 (HHT2) [MIM:600376]
ACVRL1 P37023 VAR_070334 p.Ala347Asp LP/P - Telangiectasia, hereditary hemorrhagic, 2 (HHT2) [MIM:600376]
ACVRL1 P37023 VAR_070335 p.Pro378Ser LP/P rs959973779 Telangiectasia, hereditary hemorrhagic, 2 (HHT2) [MIM:600376]
ACVRL1 P37023 VAR_070336 p.Ala400Thr US - -
ACVRL1 P37023 VAR_070337 p.Leu403Pro LP/P - Telangiectasia, hereditary hemorrhagic, 2 (HHT2) [MIM:600376]
ACVRL1 P37023 VAR_070338 p.Gly416Ser LP/P - Telangiectasia, hereditary hemorrhagic, 2 (HHT2) [MIM:600376]
ACVRL1 P37023 VAR_070339 p.Pro424Arg LP/P - Telangiectasia, hereditary hemorrhagic, 2 (HHT2) [MIM:600376]
ACVRL1 P37023 VAR_070340 p.Tyr426Cys LP/P - Telangiectasia, hereditary hemorrhagic, 2 (HHT2) [MIM:600376]
ACVRL1 P37023 VAR_070341 p.Pro433Arg LP/P - Telangiectasia, hereditary hemorrhagic, 2 (HHT2) [MIM:600376]
ACVRL1 P37023 VAR_070342 p.Pro449Ser LP/P - Telangiectasia, hereditary hemorrhagic, 2 (HHT2) [MIM:600376]
ACVRL1 P37023 VAR_070343 p.Arg479Pro LP/P rs1085307426 Telangiectasia, hereditary hemorrhagic, 2 (HHT2) [MIM:600376]
ACVRL1 P37023 VAR_070344 p.Lys486Glu US rs113700354 -
ACVRL1 P37023 VAR_075231 p.Cys41Gly LP/P - Telangiectasia, hereditary hemorrhagic, 2 (HHT2) [MIM:600376]

ACVRL1 P37023 VAR_075232 p.Cys41Tyr LP/P rs1184716348 Telangiectasia, hereditary hemorrhagic, 2 (HHT2) [MIM:600376]
ACVRL1 P37023 VAR_075233 p.Cys46Gly LP/P - Telangiectasia, hereditary hemorrhagic, 2 (HHT2) [MIM:600376]
ACVRL1 P37023 VAR_075234 p.Arg47Pro LP/P rs774389618 Telangiectasia, hereditary hemorrhagic, 2 (HHT2) [MIM:600376]
ACVRL1 P37023 VAR_075235 p.His66Tyr LP/P rs1480110873 Telangiectasia, hereditary hemorrhagic, 2 (HHT2) [MIM:600376]
ACVRL1 P37023 VAR_075236 p.Cys77Phe LP/P rs1330837892 Telangiectasia, hereditary hemorrhagic, 2 (HHT2) [MIM:600376]
ACVRL1 P37023 VAR_075237 p.Glu111Asp LB/B rs1481094868 -
ACVRL1 P37023 VAR_075238 p.Gly211Ser LP/P - Telangiectasia, hereditary hemorrhagic, 2 (HHT2) [MIM:600376]
ACVRL1 P37023 VAR_075239 p.Ile245Val LP/P - Telangiectasia, hereditary hemorrhagic, 2 (HHT2) [MIM:600376]
ACVRL1 P37023 VAR_075240 p.Leu313Val LP/P - Telangiectasia, hereditary hemorrhagic, 2 (HHT2) [MIM:600376]
ACVRL1 P37023 VAR_075241 p.Glu379Asp LP/P - Telangiectasia, hereditary hemorrhagic, 2 (HHT2) [MIM:600376]
ACVRL1 P37023 VAR_075242 p.Val404Gly LP/P - Telangiectasia, hereditary hemorrhagic, 2 (HHT2) [MIM:600376]
ACVRL1 P37023 VAR_075243 p.Ile417Phe LB/B rs141653630 -
ACVRL1 P37023 VAR_075244 p.Val441Met LP/P rs1565596498 Telangiectasia, hereditary hemorrhagic, 2 (HHT2) [MIM:600376]
ACVRL1 P37023 VAR_075245 p.Cys443Tyr LP/P - Telangiectasia, hereditary hemorrhagic, 2 (HHT2) [MIM:600376]
ACVRL1 P37023 VAR_079583 p.Lys8Asn LB/B - -
ACVRL1 P37023 VAR_079584 p.Glu59Val US rs1466116430 -
ACVRL1 P37023 VAR_079585 p.Glu159Val US - -
ACVRL1 P37023 VAR_079586 p.Ser225Cys US - -
ACVRL1 P37023 VAR_079587 p.Thr396Ala US - -
ACY1 Q03154 VAR_020452 p.Arg386Cys LP/P rs2229152 Aminoacylase-1 deficiency (ACY1D) [MIM:609924]
ACY1 Q03154 VAR_026104 p.Glu233Asp LP/P rs121912699 Aminoacylase-1 deficiency (ACY1D) [MIM:609924]
ACY1 Q03154 VAR_026105 p.Arg353Cys LP/P rs121912698 Aminoacylase-1 deficiency (ACY1D) [MIM:609924]
ACY1 Q03154 VAR_036076 p.Glu381Asp US - A breast cancer sample
ACY1 Q03154 VAR_043113 p.Arg197Trp LP/P rs121912700 Aminoacylase-1 deficiency (ACY1D) [MIM:609924]
ACY1 Q03154 VAR_043114 p.Arg393His LP/P rs121912701 Aminoacylase-1 deficiency (ACY1D) [MIM:609924]
ACY1 Q03154 VAR_051805 p.Asn179Ser LB/B rs887540 -
ACY1 Q03154 VAR_065562 p.Arg378Gln LP/P rs150480963 Aminoacylase-1 deficiency (ACY1D) [MIM:609924]
ACY1 Q03154 VAR_065563 p.Arg378Trp LP/P rs148346337 Aminoacylase-1 deficiency (ACY1D) [MIM:609924]
ACY3 Q96HD9 VAR_048341 p.Arg8Gln LB/B rs948445 -
ACY3 Q96HD9 VAR_048342 p.Val281Met LB/B rs2290959 -
ADA P00813 VAR_002209 p.Asp8Asn LB/B rs73598374 -
ADA P00813 VAR_002210 p.His15Asp LP/P rs121908725 Severe combined immunodeficiency autosomal recessive T-cell-negative/B-cell-negative/NK-cell-negative due to adenosine deaminase deficiency (ADASCID) [MIM:102700]

ADA P00813 VAR_002211 p.Gly20Arg LP/P rs121908724 Severe combined immunodeficiency autosomal recessive T-cell-negative/B-cell-negative/NK-cell-negative due to adenosine deaminase deficiency (ADASCID) [MIM:102700]
ADA P00813 VAR_002212 p.Gly74Cys LP/P rs121908730 Severe combined immunodeficiency autosomal recessive T-cell-negative/B-cell-negative/NK-cell-negative due to adenosine deaminase deficiency (ADASCID) [MIM:102700]
ADA P00813 VAR_002213 p.Arg76Trp LP/P rs121908736 Severe combined immunodeficiency autosomal recessive T-cell-negative/B-cell-negative/NK-cell-negative due to adenosine deaminase deficiency (ADASCID) [MIM:102700]
ADA P00813 VAR_002214 p.Lys80Arg LB/B rs11555566 -
ADA P00813 VAR_002215 p.Ala83Asp LP/P rs121908726 Severe combined immunodeficiency autosomal recessive T-cell-negative/B-cell-negative/NK-cell-negative due to adenosine deaminase deficiency (ADASCID) [MIM:102700]
ADA P00813 VAR_002216 p.Arg101Leu LP/P rs121908714 Severe combined immunodeficiency autosomal recessive T-cell-negative/B-cell-negative/NK-cell-negative due to adenosine deaminase deficiency (ADASCID) [MIM:102700]
ADA P00813 VAR_002217 p.Arg101Trp LP/P rs121908717 Severe combined immunodeficiency autosomal recessive T-cell-negative/B-cell-negative/NK-cell-negative due to adenosine deaminase deficiency (ADASCID) [MIM:102700]
ADA P00813 VAR_002218 p.Arg101Gln LP/P rs121908714 Severe combined immunodeficiency autosomal recessive T-cell-negative/B-cell-negative/NK-cell-negative due to adenosine deaminase deficiency (ADASCID) [MIM:102700]
ADA P00813 VAR_002219 p.Leu107Pro LP/P rs121908739 Severe combined immunodeficiency autosomal recessive T-cell-negative/B-cell-negative/NK-cell-negative due to adenosine deaminase deficiency (ADASCID) [MIM:102700]
ADA P00813 VAR_002220 p.Val129Met LP/P rs121908731 Severe combined immunodeficiency autosomal recessive T-cell-negative/B-cell-negative/NK-cell-negative due to adenosine deaminase deficiency (ADASCID) [MIM:102700]
ADA P00813 VAR_002221 p.Gly140Glu LP/P rs121908732 Severe combined immunodeficiency autosomal recessive T-cell-negative/B-cell-negative/NK-cell-negative due to adenosine deaminase deficiency (ADASCID) [MIM:102700]
ADA P00813 VAR_002222 p.Arg142Gln US rs61732239 A pancreatic ductal adenocarcinoma sample
ADA P00813 VAR_002223 p.Arg149Gln LP/P rs121908737 Severe combined immunodeficiency autosomal recessive T-cell-negative/B-cell-negative/NK-cell-negative due to adenosine deaminase deficiency (ADASCID) [MIM:102700]
ADA P00813 VAR_002224 p.Arg149Trp LP/P rs121908733 Severe combined immunodeficiency autosomal recessive T-cell-negative/B-cell-negative/NK-cell-negative due to adenosine deaminase deficiency (ADASCID) [MIM:102700]
ADA P00813 VAR_002225 p.Leu152Met LB/B rs121908728 -
ADA P00813 VAR_002226 p.Arg156Cys LP/P rs121908735 Severe combined immunodeficiency autosomal recessive T-cell-negative/B-cell-negative/NK-cell-negative due to adenosine deaminase deficiency (ADASCID) [MIM:102700]
ADA P00813 VAR_002227 p.Arg156His LP/P rs121908722 Severe combined immunodeficiency autosomal recessive T-cell-negative/B-cell-negative/NK-cell-negative due to adenosine deaminase deficiency (ADASCID) [MIM:102700]
ADA P00813 VAR_002228 p.Val177Met LP/P rs121908719 Severe combined immunodeficiency autosomal recessive T-cell-negative/B-cell-negative/NK-cell-negative due to adenosine deaminase deficiency (ADASCID) [MIM:102700]

ADA P00813 VAR_002229 p.Ala179Asp LP/P rs121908727 Severe combined immunodeficiency autosomal recessive T-cell-negative/B-cell-negative/NK-cell-negative due to adenosine deaminase deficiency (ADASCID) [MIM:102700]
ADA P00813 VAR_002230 p.Gln199Pro LP/P rs121908734 Severe combined immunodeficiency autosomal recessive T-cell-negative/B-cell-negative/NK-cell-negative due to adenosine deaminase deficiency (ADASCID) [MIM:102700]
ADA P00813 VAR_002231 p.Arg211Cys LP/P rs121908740 Severe combined immunodeficiency autosomal recessive T-cell-negative/B-cell-negative/NK-cell-negative due to adenosine deaminase deficiency (ADASCID) [MIM:102700]
ADA P00813 VAR_002232 p.Arg211His LP/P rs121908716 Severe combined immunodeficiency autosomal recessive T-cell-negative/B-cell-negative/NK-cell-negative due to adenosine deaminase deficiency (ADASCID) [MIM:102700]
ADA P00813 VAR_002233 p.Ala215Thr LP/P rs114025668 Severe combined immunodeficiency autosomal recessive T-cell-negative/B-cell-negative/NK-cell-negative due to adenosine deaminase deficiency (ADASCID) [MIM:102700]
ADA P00813 VAR_002234 p.Gly216Arg LP/P rs121908723 Severe combined immunodeficiency autosomal recessive T-cell-negative/B-cell-negative/NK-cell-negative due to adenosine deaminase deficiency (ADASCID) [MIM:102700]
ADA P00813 VAR_002235 p.Thr233Ile LB/B rs121908729 -
ADA P00813 VAR_002236 p.Pro274Leu LP/P rs121908738 Severe combined immunodeficiency autosomal recessive T-cell-negative/B-cell-negative/NK-cell-negative due to adenosine deaminase deficiency (ADASCID) [MIM:102700]
ADA P00813 VAR_002237 p.Ser291Leu LP/P rs121908721 Severe combined immunodeficiency autosomal recessive T-cell-negative/B-cell-negative/NK-cell-negative due to adenosine deaminase deficiency (ADASCID) [MIM:102700]
ADA P00813 VAR_002238 p.Pro297Gln LP/P rs121908718 Severe combined immunodeficiency autosomal recessive T-cell-negative/B-cell-negative/NK-cell-negative due to adenosine deaminase deficiency (ADASCID) [MIM:102700]
ADA P00813 VAR_002239 p.Leu304Arg LP/P rs199422327 Severe combined immunodeficiency autosomal recessive T-cell-negative/B-cell-negative/NK-cell-negative due to adenosine deaminase deficiency (ADASCID) [MIM:102700]
ADA P00813 VAR_002240 p.Ala329Val LP/P rs121908715 Severe combined immunodeficiency autosomal recessive T-cell-negative/B-cell-negative/NK-cell-negative due to adenosine deaminase deficiency (ADASCID) [MIM:102700]
ADA P00813 VAR_076954 p.Tyr97Cys US rs267606634 Severe combined immunodeficiency autosomal recessive T-cell-negative/B-cell-negative/NK-cell-negative due to adenosine deaminase deficiency (ADASCID) [MIM:102700]
ADA P00813 VAR_076955 p.Leu106Val US rs267606635 Severe combined immunodeficiency autosomal recessive T-cell-negative/B-cell-negative/NK-cell-negative due to adenosine deaminase deficiency (ADASCID) [MIM:102700]
ADA2 Q9NZK5 VAR_029802 p.His335Arg LB/B rs2231495 -
ADA2 Q9NZK5 VAR_071137 p.Gly47Arg LP/P rs202134424 Vasculitis, autoinflammation, immunodeficiency, and hematologic defects syndrome (VAIHS) [MIM:615688]
ADA2 Q9NZK5 VAR_071138 p.Gly47Val LP/P rs200930463 Vasculitis, autoinflammation, immunodeficiency, and hematologic defects syndrome (VAIHS) [MIM:615688]
ADA2 Q9NZK5 VAR_071139 p.Ala109Asp LP/P rs587777240 Vasculitis, autoinflammation, immunodeficiency, and hematologic defects syndrome (VAIHS) [MIM:615688]
ADA2 Q9NZK5 VAR_071140 p.His112Gln LP/P rs587777241 Vasculitis, autoinflammation, immunodeficiency, and hematologic defects syndrome (VAIHS) [MIM:615688]

ADA2 Q9NZK5 VAR_071141 p.Arg169Gln LP/P rs77563738 Vasculitis, autoinflammation, immunodeficiency, and hematologic defects syndrome (VAIHS) [MIM:615688]
ADA2 Q9NZK5 VAR_071142 p.Pro251Leu LP/P rs148936893 Vasculitis, autoinflammation, immunodeficiency, and hematologic defects syndrome (VAIHS) [MIM:615688]
ADA2 Q9NZK5 VAR_071143 p.Trp264Ser LP/P rs587777242 Vasculitis, autoinflammation, immunodeficiency, and hematologic defects syndrome (VAIHS) [MIM:615688]
ADA2 Q9NZK5 VAR_071144 p.Tyr453Cys LP/P rs376785840 Vasculitis, autoinflammation, immunodeficiency, and hematologic defects syndrome (VAIHS) [MIM:615688]
ADA2 Q9NZK5 VAR_072562 p.Val119Ala LP/P - Sneddon syndrome (SNDNS) [MIM:182410]
ADA2 Q9NZK5 VAR_072563 p.Gly142Ser LP/P - Sneddon syndrome (SNDNS) [MIM:182410]
ADAD2 Q8NCV1 VAR_036976 p.Gly44Glu LB/B rs8044695 -
ADAD2 Q8NCV1 VAR_055650 p.Gly235Arg LB/B rs11149631 -
ADAM10 O14672 VAR_066309 p.His176Tyr US rs267604273 A cutaneous metastatic melanoma sample
ADAM10 O14672 VAR_070907 p.Pro139Ser LP/P rs483352912 Reticulate acropigmentation of Kitamura (RAK) [MIM:615537]
ADAM10 O14672 VAR_070908 p.Gln170His LP/P rs61751103 Alzheimer disease 18 (AD18) [MIM:615590]
ADAM10 O14672 VAR_070909 p.Arg181Gly LP/P rs145518263 Alzheimer disease 18 (AD18) [MIM:615590]
ADAM10 O14672 VAR_070910 p.Cys524Tyr LP/P rs483352916 Reticulate acropigmentation of Kitamura (RAK) [MIM:615537]
ADAM11 O75078 VAR_062669 p.Ser693Arg US - A pancreatic ductal adenocarcinoma sample
ADAM12 O43184 VAR_036143 p.Asp301His US - A breast cancer sample
ADAM12 O43184 VAR_036144 p.Gly479Glu US rs1459457663 A breast cancer sample
ADAM12 O43184 VAR_036145 p.Leu792Phe US - A breast cancer sample
ADAM12 O43184 VAR_038542 p.Gly48Arg LB/B rs3740199 -
ADAM12 O43184 VAR_066310 p.Gly712Glu US - A cutaneous metastatic melanoma sample
ADAM12 O43184 VAR_066311 p.Pro893Ser US rs151030407 A cutaneous metastatic melanoma sample
ADAM15 Q13444 VAR_060315 p.Lys191Thr LB/B rs6427128 -
ADAM15 Q13444 VAR_060316 p.Pro294His LB/B rs2306122 -
ADAM15 Q13444 VAR_068970 p.Glu216Lys LB/B rs115753757 -
ADAM17 P78536 VAR_051586 p.Lys162Glu LB/B rs34431503 -
ADAM17 P78536 VAR_051587 p.Arg202Gly LB/B rs2230818 -
ADAM18 Q9Y3Q7 VAR_051588 p.Val212Phe LB/B rs10093794 -
ADAM18 Q9Y3Q7 VAR_066312 p.Pro170Ser US rs267601916 A cutaneous metastatic melanoma sample
ADAM18 Q9Y3Q7 VAR_066313 p.Val284Gly US - A cutaneous metastatic melanoma sample
ADAM18 Q9Y3Q7 VAR_066314 p.Met344Ile US rs267601918 A cutaneous metastatic melanoma sample
ADAM18 Q9Y3Q7 VAR_066315 p.Met362Lys US - A cutaneous metastatic melanoma sample
ADAM18 Q9Y3Q7 VAR_066316 p.Ser536Leu US - A cutaneous metastatic melanoma sample
ADAM19 Q9H013 VAR_036146 p.Arg133Gln US rs200894535 A colorectal cancer sample
ADAM19 Q9H013 VAR_036147 p.Ala298Thr US rs1178207005 A colorectal cancer sample
ADAM19 Q9H013 VAR_057066 p.Gly4Ser LB/B rs11465228 -
ADAM19 Q9H013 VAR_062670 p.His609Gln US - A pancreatic ductal adenocarcinoma sample
ADAM2 Q99965 VAR_035217 p.Gly10Trp LB/B rs34800519 -
ADAM20 O43506 VAR_047311 p.Phe19Leu LB/B rs1059166 -
ADAM22 Q9P0K1 VAR_020057 p.Pro81Arg LB/B rs2279542 -
ADAM22 Q9P0K1 VAR_051589 p.His119Tyr LB/B rs4728730 -
ADAM22 Q9P0K1 VAR_051590 p.Val207Ile LB/B rs17255978 -
ADAM22 Q9P0K1 VAR_080496 p.Cys401Tyr US rs747259064 Developmental and epileptic encephalopathy 61 (DEE61) [MIM:617933]
ADAM28 Q9UKQ2 VAR_024596 p.Val765Met LB/B rs7814768 -

ADAM28	Q9UKQ2	VAR_057067	p.Arg219Met	LB/B	rs9314282	-
ADAM28	Q9UKQ2	VAR_057068	p.Glu226Asp	LB/B	rs17736699	-
ADAM28	Q9UKQ2	VAR_057069	p.Asn493Ser	LB/B	rs7001647	-
ADAM28	Q9UKQ2	VAR_057070	p.Thr593Lys	LB/B	rs36041430	-
ADAM28	Q9UKQ2	VAR_057071	p.Thr604Pro	LB/B	rs35617826	-
ADAM28	Q9UKQ2	VAR_057072	p.Met684Ile	LB/B	rs7829965	-
ADAM28	Q9UKQ2	VAR_066317	p.Gly65Glu	US	-	A cutaneous metastatic melanoma sample
ADAM28	Q9UKQ2	VAR_066318	p.Gly134Glu	US	rs267601860	A cutaneous metastatic melanoma sample
ADAM28	Q9UKQ2	VAR_066319	p.Gly450Glu	US	rs267601862	A cutaneous metastatic melanoma sample
ADAM28	Q9UKQ2	VAR_066320	p.Ser482Phe	US	-	A cutaneous metastatic melanoma sample
ADAM28	Q9UKQ2	VAR_066321	p.Gly502Asp	US	rs267601864	A cutaneous metastatic melanoma sample
ADAM29	Q9UKF5	VAR_036148	p.Pro31Leu	US	rs544557652	A colorectal cancer sample
ADAM29	Q9UKF5	VAR_036149	p.Val205Ile	US	rs772388824	A colorectal cancer sample
ADAM29	Q9UKF5	VAR_066322	p.Leu72Phe	US	-	A melanoma cell line
ADAM29	Q9UKF5	VAR_066323	p.Ile89Met	US	-	A cutaneous metastatic melanoma sample
ADAM29	Q9UKF5	VAR_066324	p.Glu111Lys	US	rs267600087	A cutaneous metastatic melanoma sample
ADAM29	Q9UKF5	VAR_066325	p.Ser112Phe	US	-	A cutaneous metastatic melanoma sample
ADAM29	Q9UKF5	VAR_066326	p.Ser115Phe	US	-	A cutaneous metastatic melanoma sample
ADAM29	Q9UKF5	VAR_066327	p.Asp131Asn	US	-	A cutaneous metastatic melanoma sample
ADAM29	Q9UKF5	VAR_066328	p.Glu176Lys	US	rs899870236	A cutaneous metastatic melanoma sample
ADAM29	Q9UKF5	VAR_066329	p.Ser234Phe	US	rs866380131	A cutaneous metastatic melanoma sample
ADAM29	Q9UKF5	VAR_066330	p.Ile257Phe	US	rs140083180	A cutaneous metastatic melanoma sample
ADAM29	Q9UKF5	VAR_066331	p.Gly305Glu	US	rs267600089	A cutaneous metastatic melanoma sample
ADAM29	Q9UKF5	VAR_066332	p.Asp345Asn	US	rs267600090	A cutaneous metastatic melanoma sample
ADAM29	Q9UKF5	VAR_066333	p.Gly403Asp	US	rs150047888	A cutaneous metastatic melanoma sample
ADAM29	Q9UKF5	VAR_066334	p.Gly434Asp	US	rs267600091	A cutaneous metastatic melanoma sample
ADAM29	Q9UKF5	VAR_066335	p.Glu503Lys	US	-	A cutaneous metastatic melanoma sample
ADAM29	Q9UKF5	VAR_066336	p.His533Tyr	US	rs267600093	A cutaneous metastatic melanoma sample
ADAM30	Q9UKF2	VAR_024597	p.Leu359Pro	LB/B	rs2641348	-
ADAM30	Q9UKF2	VAR_061738	p.Thr737Ala	LB/B	rs35273427	-
ADAM32	Q8TC27	VAR_051591	p.Thr467Ser	LB/B	rs7845771	-
ADAM32	Q8TC27	VAR_055241	p.Gln98Arg	LB/B	rs17856744	-
ADAM32	Q8TC27	VAR_055242	p.Ser160Gly	LB/B	rs17852343	-
ADAM32	Q8TC27	VAR_055243	p.Leu327Val	LB/B	rs9643859	-
ADAM32	Q8TC27	VAR_055244	p.Lys658Asn	LB/B	rs13277386	-
ADAM32	Q8TC27	VAR_061739	p.Asp778Glu	LB/B	rs28705715	-
ADAM33	Q9BZ11	VAR_021847	p.Met764Thr	LB/B	rs2280091	-
ADAM33	Q9BZ11	VAR_029143	p.Thr178Ala	LB/B	rs3918392	-
ADAM33	Q9BZ11	VAR_029144	p.Pro774Ser	LB/B	rs2280090	-
ADAM33	Q9BZ11	VAR_030512	p.Asn109Ser	LB/B	rs41467948	-
ADAM33	Q9BZ11	VAR_030513	p.Thr272Met	LB/B	rs41534847	-
ADAM33	Q9BZ11	VAR_030514	p.Val316Ile	LB/B	rs41459049	-
ADAM33	Q9BZ11	VAR_030515	p.Pro336Ser	LB/B	rs41483049	-
ADAM33	Q9BZ11	VAR_030516	p.Ala365Ser	LB/B	rs41419248	-
ADAM33	Q9BZ11	VAR_030517	p.Asp441Glu	LB/B	rs41382144	-
ADAM33	Q9BZ11	VAR_030518	p.Trp515Arg	LB/B	rs615436	-
ADAM33	Q9BZ11	VAR_030519	p.Leu612His	LB/B	rs41453444	-
ADAM33	Q9BZ11	VAR_030520	p.Val710Ile	LB/B	rs3918396	-
ADAM33	Q9BZ11	VAR_030521	p.Cys739Gly	LB/B	rs41434648	-

ADAM33 Q9BZ11 VAR_030522 p.Asp742Tyr LB/B rs41462450 -
ADAM33 Q9BZ11 VAR_066337 p.Ala305Val US rs1169229302 A cutaneous metastatic melanoma sample
ADAM7 Q9H2U9 VAR_046728 p.Glu25Gln LB/B rs34852692 -
ADAM7 Q9H2U9 VAR_046729 p.Ile205Val LB/B rs7829386 -
ADAM7 Q9H2U9 VAR_046730 p.Val244Met LB/B rs13255694 -
ADAM7 Q9H2U9 VAR_046731 p.Ile453Thr LB/B rs3736281 -
ADAM7 Q9H2U9 VAR_046732 p.Leu570Val LB/B rs2307044 -
ADAM7 Q9H2U9 VAR_046733 p.Asn638His LB/B rs13259668 -
ADAM7 Q9H2U9 VAR_046734 p.Leu735Pro LB/B rs6980829 -
ADAM7 Q9H2U9 VAR_066296 p.Pro14Ser US rs150319320 A cutaneous metastatic melanoma sample
ADAM7 Q9H2U9 VAR_066297 p.Arg31Cys US rs137990671 A cutaneous metastatic melanoma sample
ADAM7 Q9H2U9 VAR_066298 p.Pro36Ser US rs267601866 A cutaneous metastatic melanoma sample
ADAM7 Q9H2U9 VAR_066299 p.His106Tyr US rs267601867 A cutaneous metastatic melanoma sample
ADAM7 Q9H2U9 VAR_066300 p.Leu173Pro US - -
ADAM7 Q9H2U9 VAR_066301 p.Val180Ala US - A cutaneous metastatic melanoma sample
ADAM7 Q9H2U9 VAR_066302 p.His243Tyr US - A cutaneous metastatic melanoma sample
ADAM7 Q9H2U9 VAR_066303 p.Gly302Glu US - A cutaneous metastatic melanoma sample
ADAM7 Q9H2U9 VAR_066304 p.Met359Ile US rs267601868 A cutaneous metastatic melanoma sample
ADAM7 Q9H2U9 VAR_066305 p.Gly533Glu US rs867202281 A cutaneous metastatic melanoma sample
ADAM7 Q9H2U9 VAR_066306 p.Phe593Leu US - -
ADAM7 Q9H2U9 VAR_066307 p.Glu639Lys US rs1034941983 A cutaneous metastatic melanoma sample
ADAM7 Q9H2U9 VAR_066308 p.Ser703Asn US - A cutaneous metastatic melanoma sample
ADAM8 P78325 VAR_059760 p.Gly101Arg LB/B rs11101675 -
ADAM8 P78325 VAR_061735 p.Arg189Trp LB/B rs45451297 -
ADAM8 P78325 VAR_061736 p.Arg433Cys LB/B rs12257830 -
ADAM8 P78325 VAR_061737 p.Ile775Thr LB/B rs3008319 -
ADAM8 P78325 VAR_069144 p.Trp35Arg LB/B rs2275725 -
ADAM8 P78325 VAR_069145 p.Phe657Leu LB/B rs2275720 -
ADAMDEC1 O15204 VAR_021848 p.Asn444Ser LB/B rs3765124 -
ADAMDEC1 O15204 VAR_024598 p.Met121Thr LB/B rs7007084 -
ADAMTS1 Q9UHI8 VAR_030001 p.Ala227Pro LB/B rs428785 -
ADAMTS10 Q9H324 VAR_054439 p.Ala25Thr LP/P - Weill-Marchesani syndrome 1 (WMS1) [MIM:277600]
ADAMTS10 Q9H324 VAR_054440 p.Arg119Gln LB/B rs3814291 -
ADAMTS10 Q9H324 VAR_054441 p.Thr134Ser LB/B rs7255721 -
ADAMTS12 P58397 VAR_057074 p.Gln110Glu LB/B rs16891862 -
ADAMTS12 P58397 VAR_057075 p.Arg1000Gln LB/B rs13362345 -
ADAMTS12 P58397 VAR_058972 p.Thr1495Ile LB/B rs25754 -
ADAMTS12 P58397 VAR_059761 p.Trp1177Arg LB/B rs3813474 -
ADAMTS12 P58397 VAR_059762 p.Ser1591Pro LB/B rs16891281 -
ADAMTS13 Q76LX8 VAR_027109 p.Arg7Trp LB/B rs34024143 -
ADAMTS13 Q76LX8 VAR_027110 p.Val88Met LP/P rs281875302 Thrombotic thrombocytopenic purpura, hereditary (TTP) [MIM:274150]
ADAMTS13 Q76LX8 VAR_027111 p.His96Asp LP/P rs121908467 Thrombotic thrombocytopenic purpura, hereditary (TTP) [MIM:274150]
ADAMTS13 Q76LX8 VAR_027112 p.Arg102Cys LP/P rs121908469 Thrombotic thrombocytopenic purpura, hereditary (TTP) [MIM:274150]
ADAMTS13 Q76LX8 VAR_027113 p.Arg193Trp LP/P rs281875287 Thrombotic thrombocytopenic purpura, hereditary (TTP) [MIM:274150]

ADAMTS13 Q76LX8 VAR_027114 p.Thr196Ile LP/P rs121908470 Thrombotic thrombocytopenic purpura, hereditary (TTP) [MIM:274150]
ADAMTS13 Q76LX8 VAR_027115 p.His234Gln LP/P rs281875304 Thrombotic thrombocytopenic purpura, hereditary (TTP) [MIM:274150]
ADAMTS13 Q76LX8 VAR_027116 p.Ala250Val LP/P rs121908478 Thrombotic thrombocytopenic purpura, hereditary (TTP) [MIM:274150]
ADAMTS13 Q76LX8 VAR_027117 p.Arg268Pro LP/P rs121908477 Thrombotic thrombocytopenic purpura, hereditary (TTP) [MIM:274150]
ADAMTS13 Q76LX8 VAR_027118 p.Trp390Cys LP/P rs281875306 Thrombotic thrombocytopenic purpura, hereditary (TTP) [MIM:274150]
ADAMTS13 Q76LX8 VAR_027119 p.Arg398His LP/P rs121908471 Thrombotic thrombocytopenic purpura, hereditary (TTP) [MIM:274150]
ADAMTS13 Q76LX8 VAR_027120 p.Gln448Glu LB/B rs2301612 -
ADAMTS13 Q76LX8 VAR_027121 p.Pro475Ser LB/B rs11575933 -
ADAMTS13 Q76LX8 VAR_027122 p.Cys508Tyr LP/P rs281875305 Thrombotic thrombocytopenic purpura, hereditary (TTP) [MIM:274150]
ADAMTS13 Q76LX8 VAR_027123 p.Arg528Gly LP/P rs121908473 Thrombotic thrombocytopenic purpura, hereditary (TTP) [MIM:274150]
ADAMTS13 Q76LX8 VAR_027124 p.Pro618Ala LB/B rs28647808 -
ADAMTS13 Q76LX8 VAR_027125 p.Arg625His LB/B rs36090624 -
ADAMTS13 Q76LX8 VAR_027126 p.Ile673Phe LP/P rs281875307 Thrombotic thrombocytopenic purpura, hereditary (TTP) [MIM:274150]
ADAMTS13 Q76LX8 VAR_027127 p.Arg692Cys LP/P rs121908475 Thrombotic thrombocytopenic purpura, hereditary (TTP) [MIM:274150]
ADAMTS13 Q76LX8 VAR_027128 p.Ala732Val LB/B rs41314453 -
ADAMTS13 Q76LX8 VAR_027129 p.Ala900Val LB/B rs685523 -
ADAMTS13 Q76LX8 VAR_027130 p.Ser903Leu LB/B rs78977446 -
ADAMTS13 Q76LX8 VAR_027131 p.Cys908Tyr LP/P rs281875301 Thrombotic thrombocytopenic purpura, hereditary (TTP) [MIM:274150]
ADAMTS13 Q76LX8 VAR_027132 p.Cys951Gly LP/P rs121908468 Thrombotic thrombocytopenic purpura, hereditary (TTP) [MIM:274150]
ADAMTS13 Q76LX8 VAR_027133 p.Cys1024Gly LP/P rs121908472 Thrombotic thrombocytopenic purpura, hereditary (TTP) [MIM:274150]
ADAMTS13 Q76LX8 VAR_027134 p.Ala1033Thr LB/B rs28503257 -
ADAMTS13 Q76LX8 VAR_027135 p.Arg1095Trp LB/B rs782383410 -
ADAMTS13 Q76LX8 VAR_027136 p.Arg1123Cys LP/P rs281875340 Thrombotic thrombocytopenic purpura, hereditary (TTP) [MIM:274150]
ADAMTS13 Q76LX8 VAR_027137 p.Cys1213Tyr LP/P rs121908474 Thrombotic thrombocytopenic purpura, hereditary (TTP) [MIM:274150]
ADAMTS13 Q76LX8 VAR_027138 p.Gly1239Val LP/P rs281875303 Thrombotic thrombocytopenic purpura, hereditary (TTP) [MIM:274150]
ADAMTS13 Q76LX8 VAR_027139 p.Arg1336Trp LP/P rs281875308 Thrombotic thrombocytopenic purpura, hereditary (TTP) [MIM:274150]
ADAMTS13 Q76LX8 VAR_027162 p.Gln456His LB/B rs36220239 -
ADAMTS13 Q76LX8 VAR_027163 p.Pro457Leu LB/B rs36220240 -
ADAMTS13 Q76LX8 VAR_027164 p.Glu740Lys LB/B rs36221451 -
ADAMTS13 Q76LX8 VAR_027165 p.Gly982Arg LB/B rs36222275 -
ADAMTS13 Q76LX8 VAR_027166 p.Thr1226Ile LB/B rs36222894 -

ADAMTS13 Q76LX8 VAR_067770 p.Ile79Met LP/P rs281875297 Thrombotic thrombocytopenic purpura, hereditary (TTP) [MIM:274150]
ADAMTS13 Q76LX8 VAR_067771 p.Ser119Phe LP/P rs281875291 Thrombotic thrombocytopenic purpura, hereditary (TTP) [MIM:274150]
ADAMTS13 Q76LX8 VAR_067772 p.Ile178Thr LP/P rs281875289 Thrombotic thrombocytopenic purpura, hereditary (TTP) [MIM:274150]
ADAMTS13 Q76LX8 VAR_067773 p.Ser203Pro LP/P rs281875298 Thrombotic thrombocytopenic purpura, hereditary (TTP) [MIM:274150]
ADAMTS13 Q76LX8 VAR_067774 p.Leu232Gln LP/P rs281875292 Thrombotic thrombocytopenic purpura, hereditary (TTP) [MIM:274150]
ADAMTS13 Q76LX8 VAR_067775 p.Asp235His LP/P rs281875337 Thrombotic thrombocytopenic purpura, hereditary (TTP) [MIM:274150]
ADAMTS13 Q76LX8 VAR_067776 p.Ser263Cys LP/P rs281875293 Thrombotic thrombocytopenic purpura, hereditary (TTP) [MIM:274150]
ADAMTS13 Q76LX8 VAR_067777 p.Tyr304Cys LP/P rs281875285 Thrombotic thrombocytopenic purpura, hereditary (TTP) [MIM:274150]
ADAMTS13 Q76LX8 VAR_067778 p.Cys311Tyr LP/P rs281875336 Thrombotic thrombocytopenic purpura, hereditary (TTP) [MIM:274150]
ADAMTS13 Q76LX8 VAR_067779 p.Thr339Arg LB/B rs149517360 -
ADAMTS13 Q76LX8 VAR_067780 p.Cys347Ser LP/P rs281875294 Thrombotic thrombocytopenic purpura, hereditary (TTP) [MIM:274150]
ADAMTS13 Q76LX8 VAR_067781 p.Arg349Cys LP/P rs281875288 Thrombotic thrombocytopenic purpura, hereditary (TTP) [MIM:274150]
ADAMTS13 Q76LX8 VAR_067782 p.Pro353Leu LP/P rs281875338 Thrombotic thrombocytopenic purpura, hereditary (TTP) [MIM:274150]
ADAMTS13 Q76LX8 VAR_067783 p.Arg507Gln LP/P rs281875296 Thrombotic thrombocytopenic purpura, hereditary (TTP) [MIM:274150]
ADAMTS13 Q76LX8 VAR_067784 p.Gly525Asp LP/P rs281875286 Thrombotic thrombocytopenic purpura, hereditary (TTP) [MIM:274150]
ADAMTS13 Q76LX8 VAR_067785 p.Ala596Val LP/P rs281875299 Thrombotic thrombocytopenic purpura, hereditary (TTP) [MIM:274150]
ADAMTS13 Q76LX8 VAR_067786 p.Ala606Pro LP/P rs281875290 Thrombotic thrombocytopenic purpura, hereditary (TTP) [MIM:274150]
ADAMTS13 Q76LX8 VAR_067787 p.Tyr658Cys LP/P rs281875335 Thrombotic thrombocytopenic purpura, hereditary (TTP) [MIM:274150]
ADAMTS13 Q76LX8 VAR_067788 p.Pro671Leu LP/P rs281875295 Thrombotic thrombocytopenic purpura, hereditary (TTP) [MIM:274150]
ADAMTS13 Q76LX8 VAR_067789 p.Cys758Arg LP/P rs281875300 Thrombotic thrombocytopenic purpura, hereditary (TTP) [MIM:274150]
ADAMTS13 Q76LX8 VAR_067790 p.Cys908Ser LP/P rs281875301 Thrombotic thrombocytopenic purpura, hereditary (TTP) [MIM:274150]
ADAMTS13 Q76LX8 VAR_067792 p.Arg1060Trp LP/P rs142572218 Thrombotic thrombocytopenic purpura, hereditary (TTP) [MIM:274150]
ADAMTS13 Q76LX8 VAR_067793 p.Arg1219Trp LP/P rs281875339 Thrombotic thrombocytopenic purpura, hereditary (TTP) [MIM:274150]
ADAMTS13 Q76LX8 VAR_067794 p.Ser1314Leu LB/B rs142060916 -
ADAMTS14 Q8WXS8 VAR_047837 p.Arg179Cys LB/B rs34022601 -
ADAMTS14 Q8WXS8 VAR_047838 p.Leu590Pro LB/B rs10823607 -
ADAMTS14 Q8WXS8 VAR_047839 p.Leu937Met LB/B rs12774070 -

ADAMTS14 Q8WXS8 VAR_047840 p.Ser1017Asn LB/B rs10999516 -
ADAMTS14 Q8WXS8 VAR_047841 p.Glu1049Gly LB/B rs4747096 -
ADAMTS15 Q8TE58 VAR_036150 p.Gln770Arg US rs776537988 A colorectal cancer sample
ADAMTS15 Q8TE58 VAR_036151 p.Cys878Gly US - A colorectal cancer sample
ADAMTS15 Q8TE58 VAR_051594 p.Asn623Ser LB/B rs11222114 -
ADAMTS16 Q8TE57 VAR_057076 p.Met110Val LB/B rs1863968 -
ADAMTS16 Q8TE57 VAR_057077 p.Ala486Thr LB/B rs16875054 -
ADAMTS16 Q8TE57 VAR_057078 p.Arg789Cys LB/B rs9313105 -
ADAMTS16 Q8TE57 VAR_057079 p.Arg859Leu LB/B rs16875122 -
ADAMTS16 Q8TE57 VAR_057080 p.Glu863Lys LB/B rs35394775 -
ADAMTS17 Q8TE56 VAR_057081 p.Ser216Leu LB/B rs7496668 -
ADAMTS17 Q8TE56 VAR_057082 p.Met482Thr LB/B rs28567966 -
ADAMTS17 Q8TE56 VAR_060317 p.Asn1094Ser LB/B rs2573652 -
ADAMTS17 Q8TE56 VAR_064041 p.Arg566Thr LB/B - -
ADAMTS18 Q8TE60 VAR_036152 p.Arg382Lys US rs368783738 A colorectal cancer sample
ADAMTS18 Q8TE60 VAR_036153 p.Lys455Thr US rs776584074 A colorectal cancer sample
ADAMTS18 Q8TE60 VAR_057083 p.Leu769Ile LB/B rs9930984 -
ADAMTS18 Q8TE60 VAR_057084 p.Ala946Ser LB/B rs12935394 -
ADAMTS18 Q8TE60 VAR_057085 p.Ser1080Arg LB/B rs35478105 -
ADAMTS18 Q8TE60 VAR_057086 p.Ser1159Thr LB/B rs3743749 -
ADAMTS18 Q8TE60 VAR_060231 p.Tyr191His LB/B rs11643211 -
ADAMTS18 Q8TE60 VAR_060232 p.Leu626Ile LB/B rs11640912 -
ADAMTS18 Q8TE60 VAR_066554 p.Ser179Leu LB/B rs387906972 -
ADAMTS18 Q8TE60 VAR_070849 p.Leu202Pro LP/P rs397515468 Microcornea, myopic chorioretinal atrophy, and telecanthus (MMCAT) [MIM:615458]
ADAMTS18 Q8TE60 VAR_070850 p.Cys577Trp LP/P rs148319220 Microcornea, myopic chorioretinal atrophy, and telecanthus (MMCAT) [MIM:615458]
ADAMTS19 Q8TE59 VAR_024599 p.Tyr1089Phe LB/B rs11749126 -
ADAMTS19 Q8TE59 VAR_036154 p.Leu360Ile US - A breast cancer sample
ADAMTS19 Q8TE59 VAR_057087 p.Glu582Gly LB/B rs10062501 -
ADAMTS2 O95450 VAR_020058 p.Val245Ile LB/B rs398829 -
ADAMTS2 O95450 VAR_020059 p.Pro1177Ser LB/B rs1054480 -
ADAMTS2 O95450 VAR_047927 p.Val74Met LB/B rs2271211 -
ADAMTS2 O95450 VAR_047928 p.Arg241His LB/B rs11750821 -
ADAMTS2 O95450 VAR_047929 p.Glu331Lys LB/B rs17667857 -
ADAMTS2 O95450 VAR_047930 p.Gly665Arg LB/B rs35372714 -
ADAMTS2 O95450 VAR_047931 p.Arg827Gln LB/B rs35445112 -
ADAMTS20 P59510 VAR_057088 p.Lys876Met LB/B rs7302446 -
ADAMTS20 P59510 VAR_057089 p.Arg1000His LB/B rs7297737 -
ADAMTS20 P59510 VAR_057090 p.Ser1273Phe LB/B rs7310011 -
ADAMTS3 O15072 VAR_055012 p.Arg138Lys LB/B rs788908 -
ADAMTS3 O15072 VAR_055013 p.Ser1074Pro LB/B rs35864003 -
ADAMTS3 O15072 VAR_081558 p.Leu168Pro LP/P rs1177851177 Hennekam lymphangiectasia-lymphedema syndrome 3 (HKLLS3) [MIM:618154]
ADAMTS3 O15072 VAR_081559 p.Ile291Thr LP/P rs61757480 Hennekam lymphangiectasia-lymphedema syndrome 3 (HKLLS3) [MIM:618154]
ADAMTS4 O75173 VAR_022450 p.Gln626Arg LB/B rs4233367 -
ADAMTS4 O75173 VAR_030636 p.Thr4Ile LB/B rs17855814 -
ADAMTS4 O75173 VAR_030637 p.Asp304Asn LB/B rs17855813 -

ADAMTS4	O75173	VAR_030638	p.Met369Val	LB/B	rs17855812	-
ADAMTS4	O75173	VAR_030639	p.Pro552Thr	LB/B	rs17855815	-
ADAMTS4	O75173	VAR_030640	p.Thr564Ala	LB/B	rs17855816	-
ADAMTS4	O75173	VAR_030641	p.Arg836Lys	LB/B	rs11807350	-
ADAMTS4	O75173	VAR_057073	p.Ala77Thr	LB/B	rs34448954	-
ADAMTS5	Q9UNA0	VAR_021849	p.Arg614His	LB/B	rs2830585	-
ADAMTS5	Q9UNA0	VAR_028199	p.Gly138Ala	LB/B	rs457947	-
ADAMTS5	Q9UNA0	VAR_028200	p.Leu692Pro	LB/B	rs226794	-
ADAMTS7	Q9UKP4	VAR_046112	p.Ser214Pro	LB/B	rs3825807	-
ADAMTS7	Q9UKP4	VAR_046113	p.Thr307Met	LB/B	rs2127898	-
ADAMTS7	Q9UKP4	VAR_046114	p.Thr1319Ala	LB/B	rs11630236	-
ADAMTS7	Q9UKP4	VAR_046115	p.Gly1414Ser	LB/B	rs2929155	-
ADAMTS7	Q9UKP4	VAR_046116	p.Gly1583Ala	LB/B	rs7495616	-
ADAMTS9	Q9P2N4	VAR_047081	p.Ser96Pro	LB/B	rs36115950	-
ADAMTS9	Q9P2N4	VAR_047082	p.Lys1579Glu	LB/B	rs17071010	-
ADAMTS9	Q9P2N4	VAR_047083	p.Asp1674Glu	LB/B	rs6787633	-
ADAMTS9	Q9P2N4	VAR_047084	p.Lys1740Arg	LB/B	rs17070967	-
ADAMTS9	Q9P2N4	VAR_047085	p.Glu1791Gln	LB/B	rs3796381	-
ADAMTS9	Q9P2N4	VAR_047086	p.Arg1933Gln	LB/B	rs17070905	-
ADAMTS9	Q9P2N4	VAR_051592	p.Ser96Thr	LB/B	rs36115950	-
ADAMTS9	Q9P2N4	VAR_051593	p.Lys1921Glu	LB/B	rs17070909	-
ADAMTSL1	Q8N6G6	VAR_017174	p.Ser242Asn	LB/B	rs776755	-
ADAMTSL2	Q86TH1	VAR_046011	p.Val364Ile	LB/B	rs35767802	-
ADAMTSL2	Q86TH1	VAR_054874	p.Arg113His	LP/P	rs113994122	Geleophysic dysplasia 1 (GPHYSD1) [MIM:231050]
ADAMTSL2	Q86TH1	VAR_054875	p.Glu114Lys	LP/P	rs113994123	Geleophysic dysplasia 1 (GPHYSD1) [MIM:231050]
ADAMTSL2	Q86TH1	VAR_054876	p.Pro147Leu	LP/P	rs113994121	Geleophysic dysplasia 1 (GPHYSD1) [MIM:231050]
ADAMTSL2	Q86TH1	VAR_054877	p.Gly811Arg	LP/P	rs113994124	Geleophysic dysplasia 1 (GPHYSD1) [MIM:231050]
ADAMTSL2	Q86TH1	VAR_066543	p.Trp50Cys	LP/P	rs1395219766	Geleophysic dysplasia 1 (GPHYSD1) [MIM:231050]
ADAMTSL2	Q86TH1	VAR_066544	p.Arg72Gln	LP/P	rs387907064	Geleophysic dysplasia 1 (GPHYSD1) [MIM:231050]
ADAMTSL2	Q86TH1	VAR_066545	p.Arg159Trp	LP/P	rs776178041	Geleophysic dysplasia 1 (GPHYSD1) [MIM:231050]
ADAMTSL2	Q86TH1	VAR_066546	p.Ala165Thr	LP/P	rs764516811	Geleophysic dysplasia 1 (GPHYSD1) [MIM:231050]
ADAMTSL2	Q86TH1	VAR_066547	p.Cys171Arg	LP/P	-	Geleophysic dysplasia 1 (GPHYSD1) [MIM:231050]
ADAMTSL2	Q86TH1	VAR_066548	p.Arg221Cys	LP/P	rs387907065	Geleophysic dysplasia 1 (GPHYSD1) [MIM:231050]
ADAMTSL2	Q86TH1	VAR_066549	p.Ala239Thr	LP/P	-	Geleophysic dysplasia 1 (GPHYSD1) [MIM:231050]
ADAMTSL2	Q86TH1	VAR_066551	p.Arg593Cys	LP/P	-	Geleophysic dysplasia 1 (GPHYSD1) [MIM:231050]
ADAMTSL2	Q86TH1	VAR_066552	p.Ser635Leu	LP/P	-	Geleophysic dysplasia 1 (GPHYSD1) [MIM:231050]
ADAMTSL2	Q86TH1	VAR_066553	p.Pro906Leu	LP/P	rs969732840	Geleophysic dysplasia 1 (GPHYSD1) [MIM:231050]
ADAMTSL3	P82987	VAR_027478	p.His146Arg	LB/B	rs4483821	-
ADAMTSL3	P82987	VAR_027479	p.Leu290Val	LB/B	rs4144691	-

ADAMTSL3 P82987 VAR_027480 p.Val661Leu LB/B rs4842838 -
ADAMTSL3 P82987 VAR_027481 p.Arg855His LB/B rs2277848 -
ADAMTSL3 P82987 VAR_027482 p.Leu869Phe LB/B rs2277849 -
ADAMTSL3 P82987 VAR_027483 p.Thr1370Ala LB/B rs17158450 -
ADAMTSL3 P82987 VAR_027484 p.Met1558Thr LB/B rs7175910 -
ADAMTSL3 P82987 VAR_027485 p.Thr1660Ile LB/B rs950169 -
ADAMTSL3 P82987 VAR_027486 p.Arg1679His LB/B rs11857906 -
ADAMTSL3 P82987 VAR_035809 p.Val330Met US rs1439091253 A colorectal cancer sample
ADAMTSL3 P82987 VAR_035810 p.Arg587His US rs142860011 A colorectal cancer sample
ADAMTSL3 P82987 VAR_035811 p.Arg855Cys US rs146769560 A colorectal cancer sample
ADAMTSL3 P82987 VAR_035812 p.Ala1315Glu US - A colorectal cancer sample
ADAMTSL3 P82987 VAR_057365 p.Gly713Arg LB/B rs34047645 -
ADAMTSL4 Q6UY14 VAR_061918 p.Ala193Pro LB/B rs41317515 -
ADAMTSL4 Q6UY14 VAR_061919 p.Arg1028His LB/B rs56411234 -
ADAP1 O75689 VAR_047470 p.Gly241Ser LB/B rs10256887 -
ADAR P55265 VAR_017240 p.Lys384Arg LB/B rs2229857 -
ADAR P55265 VAR_017604 p.Leu923Pro LP/P rs28936680 Dyschromatosis symmetrica hereditaria (DSH) [MIM:127400]
ADAR P55265 VAR_017605 p.Phe1165Ser LP/P rs28936681 Dyschromatosis symmetrica hereditaria (DSH) [MIM:127400]
ADAR P55265 VAR_021729 p.Cys966Phe LP/P - Dyschromatosis symmetrica hereditaria (DSH) [MIM:127400]
ADAR P55265 VAR_024407 p.Tyr587Cys LB/B rs17843865 -
ADAR P55265 VAR_026669 p.Arg1155Trp LP/P rs1044845711 Dyschromatosis symmetrica hereditaria (DSH) [MIM:127400]
ADAR P55265 VAR_035805 p.Glu806Val US rs144119808 A breast cancer sample
ADAR P55265 VAR_048725 p.Arg100Gly LB/B rs1466731 -
ADAR P55265 VAR_069535 p.Pro193Ala LP/P rs145588689 Aicardi-Goutieres syndrome 6 (AGS6) [MIM:615010]
ADAR P55265 VAR_069536 p.Ala870Thr LP/P rs398122893 Aicardi-Goutieres syndrome 6 (AGS6) [MIM:615010]
ADAR P55265 VAR_069537 p.Ile872Thr LP/P rs398122897 Aicardi-Goutieres syndrome 6 (AGS6) [MIM:615010]
ADAR P55265 VAR_069538 p.Arg892His LP/P rs398122892 Aicardi-Goutieres syndrome 6 (AGS6) [MIM:615010]
ADAR P55265 VAR_069539 p.Lys999Asn LP/P rs398122896 Aicardi-Goutieres syndrome 6 (AGS6) [MIM:615010]
ADAR P55265 VAR_069540 p.Gly1007Arg LP/P rs398122822 Aicardi-Goutieres syndrome 6 (AGS6) [MIM:615010]
ADAR P55265 VAR_069541 p.Tyr1112Phe LP/P rs398122895 Aicardi-Goutieres syndrome 6 (AGS6) [MIM:615010]
ADAR P55265 VAR_069542 p.Asp1113His LP/P rs398122894 Aicardi-Goutieres syndrome 6 (AGS6) [MIM:615010]
ADARB1 P78563 VAR_070931 p.Val224Ala LB/B rs199697177 -
ADARB1 P78563 VAR_083947 p.Lys127Glu US - Neurodevelopmental disorder with hypotonia, microcephaly, and seizures (NEDHYMS) [MIM:618862]
ADARB1 P78563 VAR_083948 p.Lys367Asn US - Neurodevelopmental disorder with hypotonia, microcephaly, and seizures (NEDHYMS) [MIM:618862]

ADARB1 P78563 VAR_083949 p.Thr498Ala US - Neurodevelopmental disorder with hypotonia, microcephaly, and seizures (NEDHYMS) [MIM:618862]
ADARB1 P78563 VAR_083950 p.Arg603Gln LP/P - Neurodevelopmental disorder with hypotonia, microcephaly, and seizures (NEDHYMS) [MIM:618862]
ADARB1 P78563 VAR_083951 p.Ala722Val US - Neurodevelopmental disorder with hypotonia, microcephaly, and seizures (NEDHYMS) [MIM:618862]
ADARB2 Q9NS39 VAR_020438 p.Ala44Thr LB/B rs3793733 -
ADARB2 Q9NS39 VAR_035806 p.Thr210Met US - A colorectal cancer sample
ADARB2 Q9NS39 VAR_035807 p.Val512Ile US rs138734198 A colorectal cancer sample
ADARB2 Q9NS39 VAR_048726 p.Ala626Thr LB/B rs2271275 -
ADAT1 Q9BUB4 VAR_032340 p.His167Asn LB/B rs3743598 -
ADAT1 Q9BUB4 VAR_032341 p.Thr203Asn LB/B rs3743599 -
ADAT1 Q9BUB4 VAR_055649 p.Thr242Pro LB/B rs3743600 -
ADAT1 Q9BUB4 VAR_061098 p.Ile226Val LB/B rs56029288 -
ADAT3 Q96EY9 VAR_035804 p.Arg332Cys US - A breast cancer sample
ADAT3 Q96EY9 VAR_069778 p.Val128Met LP/P - Mental retardation, autosomal recessive 36 (MRT36) [MIM:615286]
ADCK2 Q7Z695 VAR_029992 p.Ser307Pro LB/B rs1140034 -
ADCK2 Q7Z695 VAR_029993 p.Val418Met LB/B rs3748092 -
ADCK2 Q7Z695 VAR_029994 p.Pro622Leu LB/B rs1046515 -
ADCK2 Q7Z695 VAR_041418 p.Val418Leu LB/B rs3748092 -
ADCK2 Q7Z695 VAR_041419 p.Pro626Leu LB/B rs55922126 -
ADCK2 Q7Z695 VAR_060990 p.Ser66Gly LB/B rs2968558 -
ADCK5 Q3MIX3 VAR_029996 p.Ser17Arg LB/B rs6599528 -
ADCY1 Q08828 VAR_029186 p.Pro456Leu LB/B rs12721473 -
ADCY1 Q08828 VAR_029187 p.Ala940Thr LB/B rs45444695 -
ADCY1 Q08828 VAR_048246 p.Val984Met LB/B rs2293106 -
ADCY10 Q96PN6 VAR_038476 p.Thr234Met LB/B rs16859886 -
ADCY10 Q96PN6 VAR_038477 p.Ile697Val LB/B rs2071921 -
ADCY2 Q08462 VAR_029012 p.Val147Leu LB/B rs13166360 -
ADCY2 Q08462 VAR_048247 p.Val163Ile LB/B rs34043481 -
ADCY3 O60266 VAR_048248 p.Ser107Pro LB/B rs11676272 -
ADCY3 O60266 VAR_080791 p.Asn64Ile LB/B rs541941351 -
ADCY5 O95622 VAR_068821 p.Ala726Thr LP/P rs796065306 Dyskinesia, familial, with facial myokymia (FDFM) [MIM:606703]
ADCY5 O95622 VAR_073778 p.Arg418Trp LP/P rs864309483 Dyskinesia, familial, with facial myokymia (FDFM) [MIM:606703]
ADCY6 O43306 VAR_048249 p.Ala674Ser LB/B rs3730071 -
ADCY6 O43306 VAR_073434 p.Arg1116Cys LP/P rs786204798 Lethal congenital contracture syndrome 8 (LCCS8) [MIM:616287]
ADCY8 P40145 VAR_029188 p.Ala80Thr LB/B rs2228949 -
ADCY8 P40145 VAR_036328 p.Phe881Leu US - A colorectal cancer sample
ADCY9 O60503 VAR_023750 p.Ile772Met LB/B rs2230739 -
ADCY9 O60503 VAR_070887 p.Asn1154Ser LB/B rs61731445 -
ADCYAP1 P18509 VAR_014597 p.Asp54Gly LB/B rs2856966 -
ADD1 P35611 VAR_014184 p.Gly460Trp LB/B rs4961 -
ADD1 P35611 VAR_014185 p.Ser586Cys LB/B rs4963 -
ADD1 P35611 VAR_014863 p.Tyr270Asn LB/B rs4971 -
ADD1 P35611 VAR_014864 p.Glu376Asp LB/B rs4972 -

ADD1	P35611	VAR_014865	p.Asn510Ile	LB/B	rs4962	-
ADD1	P35611	VAR_022108	p.Arg6Cys	LB/B	rs2295497	-
ADD2	P35612	VAR_014866	p.Asp28Asn	LB/B	rs4986	-
ADD2	P35612	VAR_014867	p.Glu335Asp	LB/B	rs4982	-
ADD2	P35612	VAR_014868	p.Ser663Arg	LB/B	rs4985	-
ADD2	P35612	VAR_025318	p.Thr439Ala	LB/B	rs17855969	-
ADD2	P35612	VAR_048195	p.Ser98Cys	LB/B	rs4987	-
ADD3	Q9UEY8	VAR_076996	p.Gly367Asp	LP/P	rs564185858	Cerebral palsy, spastic quadriplegic 3 (CPSQ3) [MIM:617008]
ADGB	Q8N7X0	VAR_025948	p.Ile310Thr	LB/B	rs9497606	-
ADGB	Q8N7X0	VAR_063158	p.Thr1637Ala	LB/B	rs1052445	-
ADGRA1	Q86SQ6	VAR_055931	p.Ala183Ser	LB/B	rs34915504	-
ADGRA1	Q86SQ6	VAR_055932	p.Gln214Leu	LB/B	rs34679676	-
ADGRA2	Q96PE1	VAR_072079	p.Pro29Leu	US	-	-
ADGRA2	Q96PE1	VAR_072561	p.Thr375Lys	LB/B	-	-
ADGRA3	Q8IWK6	VAR_033971	p.Val1043Met	LB/B	rs9002	-
ADGRA3	Q8IWK6	VAR_033972	p.Val1166Gly	LB/B	rs3814416	-
ADGRB2	O60241	VAR_079840	p.Arg1465Trp	LP/P	rs778361520	-
ADGRB3	O60242	VAR_046525	p.Asn503Ser	LB/B	rs1932618	-
ADGRD1	Q6QNK2	VAR_049461	p.Val508Met	LB/B	rs11833801	-
ADGRD1	Q6QNK2	VAR_049462	p.Ser523Leu	LB/B	rs11061318	-
ADGRD1	Q6QNK2	VAR_077698	p.Tyr18Cys	LB/B	rs149181066	-
ADGRD1	Q6QNK2	VAR_077699	p.Tyr18His	LB/B	rs146611221	-
ADGRD1	Q6QNK2	VAR_077700	p.Asp32Asn	LB/B	rs376178471	-
ADGRD1	Q6QNK2	VAR_077701	p.Glu78Lys	LB/B	rs267603378	-
ADGRD1	Q6QNK2	VAR_077702	p.Thr82Met	LB/B	rs143395855	-
ADGRD1	Q6QNK2	VAR_077703	p.Tyr85Cys	LB/B	rs199848650	-
ADGRD1	Q6QNK2	VAR_077704	p.Tyr89Asp	LB/B	rs201546462	-
ADGRD1	Q6QNK2	VAR_077705	p.Phe110Leu	LB/B	rs148928637	-
ADGRD1	Q6QNK2	VAR_077706	p.Ser138Phe	LB/B	rs199526762	-
ADGRD1	Q6QNK2	VAR_077707	p.Gly140Ser	LB/B	rs374985420	-
ADGRD1	Q6QNK2	VAR_077708	p.Gly141Asp	LB/B	rs142759046	-
ADGRD1	Q6QNK2	VAR_077709	p.Val145Leu	LB/B	rs146945782	-
ADGRD1	Q6QNK2	VAR_077710	p.Arg150Trp	LB/B	rs144030317	-
ADGRD1	Q6QNK2	VAR_077711	p.Trp174Ser	LB/B	rs141606054	-
ADGRD1	Q6QNK2	VAR_077712	p.Glu178Lys	LB/B	rs148148477	-
ADGRD1	Q6QNK2	VAR_077713	p.Val184Ile	LB/B	rs61732860	-
ADGRD1	Q6QNK2	VAR_077714	p.Gly195Arg	LB/B	rs267603379	-
ADGRD1	Q6QNK2	VAR_077715	p.Arg199Cys	LB/B	rs146310036	-
ADGRD1	Q6QNK2	VAR_077716	p.Arg199His	LB/B	rs375084180	-
ADGRD1	Q6QNK2	VAR_077717	p.Glu203Asp	LB/B	rs201045213	-
ADGRD1	Q6QNK2	VAR_077718	p.Val209Met	LB/B	rs139478688	-
ADGRD1	Q6QNK2	VAR_077719	p.Asp226Asn	LB/B	rs377562590	-
ADGRD1	Q6QNK2	VAR_077720	p.Arg233Trp	LB/B	rs370807516	-
ADGRD1	Q6QNK2	VAR_077721	p.Ala241Thr	LB/B	rs368468703	-
ADGRD1	Q6QNK2	VAR_077722	p.Met242Thr	LB/B	rs201057341	-
ADGRD1	Q6QNK2	VAR_077723	p.Thr245Ile	LB/B	rs201053759	-
ADGRD1	Q6QNK2	VAR_077724	p.Thr257Ala	LB/B	rs146563785	-
ADGRD1	Q6QNK2	VAR_077725	p.Pro259Gln	LB/B	rs75096240	-

ADGRD1	Q6QNK2	VAR_077726	p.Ser265Tyr	LB/B	rs137909892	-
ADGRD1	Q6QNK2	VAR_077727	p.Ser268Asn	LB/B	rs149012578	-
ADGRD1	Q6QNK2	VAR_077728	p.Val270Ala	LB/B	rs147773154	-
ADGRD1	Q6QNK2	VAR_077729	p.Val270Met	LB/B	rs147105264	-
ADGRD1	Q6QNK2	VAR_077730	p.Pro293Ala	LB/B	rs146929634	-
ADGRD1	Q6QNK2	VAR_077731	p.Gly294Arg	LB/B	rs148017957	-
ADGRD1	Q6QNK2	VAR_077732	p.Pro308Ser	LB/B	rs201661954	-
ADGRD1	Q6QNK2	VAR_077733	p.Leu318Phe	LB/B	rs200641036	-
ADGRD1	Q6QNK2	VAR_077734	p.Ser349Asn	LB/B	rs370854685	-
ADGRD1	Q6QNK2	VAR_077735	p.Asn364Ser	LB/B	rs146050435	-
ADGRD1	Q6QNK2	VAR_077736	p.Thr369Met	LB/B	rs142314859	-
ADGRD1	Q6QNK2	VAR_077737	p.Phe383Ser	LB/B	rs200232576	-
ADGRD1	Q6QNK2	VAR_077738	p.Val393Met	LB/B	rs374606811	-
ADGRD1	Q6QNK2	VAR_077739	p.His397Gln	LB/B	rs201985264	-
ADGRD1	Q6QNK2	VAR_077740	p.Arg399Cys	LB/B	rs150882180	-
ADGRD1	Q6QNK2	VAR_077741	p.Gly404Ala	LB/B	rs371536090	-
ADGRD1	Q6QNK2	VAR_077742	p.Gln405Pro	LB/B	rs145663007	-
ADGRD1	Q6QNK2	VAR_077743	p.Ile410Val	LB/B	rs376489706	-
ADGRD1	Q6QNK2	VAR_077744	p.Pro411Ser	LB/B	rs147757048	-
ADGRD1	Q6QNK2	VAR_077745	p.Glu413Lys	LB/B	rs142628291	-
ADGRD1	Q6QNK2	VAR_077746	p.Ala419Val	LB/B	rs370959644	-
ADGRD1	Q6QNK2	VAR_077747	p.Gly425Ser	LB/B	rs374575404	-
ADGRD1	Q6QNK2	VAR_077748	p.Ala441Thr	LB/B	rs375271891	-
ADGRD1	Q6QNK2	VAR_077749	p.Ala448Asp	LB/B	rs200173874	-
ADGRD1	Q6QNK2	VAR_077750	p.His450Arg	LB/B	rs200060202	-
ADGRD1	Q6QNK2	VAR_077751	p.Asp453Asn	LB/B	rs149065791	-
ADGRD1	Q6QNK2	VAR_077752	p.Cys454Tyr	LB/B	rs143062748	-
ADGRD1	Q6QNK2	VAR_077753	p.Ala458Thr	LB/B	rs140426880	-
ADGRD1	Q6QNK2	VAR_077754	p.Ser464Ala	LB/B	rs78638447	-
ADGRD1	Q6QNK2	VAR_077755	p.Asn476Ser	LB/B	rs138163855	-
ADGRD1	Q6QNK2	VAR_077756	p.Ser478Leu	LB/B	rs372643228	-
ADGRD1	Q6QNK2	VAR_077757	p.Thr484Met	LB/B	rs149266247	-
ADGRD1	Q6QNK2	VAR_077758	p.Val485Ile	LB/B	rs369012277	-
ADGRD1	Q6QNK2	VAR_077759	p.Glu498Gly	LB/B	rs200576124	-
ADGRD1	Q6QNK2	VAR_077760	p.Ala499Ser	LB/B	rs150620459	-
ADGRD1	Q6QNK2	VAR_077761	p.Asn524Lys	LB/B	rs370443698	-
ADGRD1	Q6QNK2	VAR_077762	p.Val538Ala	LB/B	rs201849687	-
ADGRD1	Q6QNK2	VAR_077763	p.Val538Ile	LB/B	rs200971352	-
ADGRD1	Q6QNK2	VAR_077764	p.Arg540Cys	LB/B	rs147294464	-
ADGRD1	Q6QNK2	VAR_077765	p.Arg540His	LB/B	rs145630930	-
ADGRD1	Q6QNK2	VAR_077766	p.Arg560Cys	LB/B	rs371989819	-
ADGRD1	Q6QNK2	VAR_077767	p.Arg560His	LB/B	rs375179921	-
ADGRD1	Q6QNK2	VAR_077768	p.Ser567Leu	LB/B	rs192515185	-
ADGRD1	Q6QNK2	VAR_077769	p.Ile569Val	LB/B	rs139017446	-
ADGRD1	Q6QNK2	VAR_077770	p.Ala589Thr	LB/B	rs201161291	-
ADGRD1	Q6QNK2	VAR_077771	p.Val594Met	LB/B	rs189007948	-
ADGRD1	Q6QNK2	VAR_077772	p.Arg601His	LB/B	rs201524753	-
ADGRD1	Q6QNK2	VAR_077773	p.Leu608Met	LB/B	rs200052797	-
ADGRD1	Q6QNK2	VAR_077774	p.Arg624Cys	LB/B	rs117457351	-

ADGRD1	Q6QNK2	VAR_077775	p.Glu626Lys	LB/B	rs199778477	-
ADGRD1	Q6QNK2	VAR_077776	p.Thr630Ile	LB/B	rs145482553	-
ADGRD1	Q6QNK2	VAR_077777	p.Ser667Leu	LB/B	rs377401276	-
ADGRD1	Q6QNK2	VAR_077778	p.Arg673His	LB/B	rs141128784	-
ADGRD1	Q6QNK2	VAR_077779	p.Met695Thr	LB/B	rs143163307	-
ADGRD1	Q6QNK2	VAR_077780	p.Gly699Arg	LB/B	rs149859410	-
ADGRD1	Q6QNK2	VAR_077781	p.Ala720Val	LB/B	rs141439159	-
ADGRD1	Q6QNK2	VAR_077782	p.Ala743Thr	LB/B	rs143902981	-
ADGRD1	Q6QNK2	VAR_077783	p.His749Arg	LB/B	rs372207677	-
ADGRD1	Q6QNK2	VAR_077784	p.Asp751Glu	LB/B	rs147763331	-
ADGRD1	Q6QNK2	VAR_077785	p.Ala761Glu	LB/B	rs369201469	-
ADGRD1	Q6QNK2	VAR_077786	p.Val764Met	LB/B	rs149434203	-
ADGRD1	Q6QNK2	VAR_077787	p.Val777Met	LB/B	rs144814859	-
ADGRD1	Q6QNK2	VAR_077788	p.Ala779Val	LB/B	rs61746588	-
ADGRD1	Q6QNK2	VAR_077789	p.Thr793Met	LB/B	rs375488636	-
ADGRD1	Q6QNK2	VAR_077790	p.Asn795Lys	LB/B	rs369853823	-
ADGRD1	Q6QNK2	VAR_077791	p.Ala816Thr	LB/B	rs368828722	-
ADGRD1	Q6QNK2	VAR_077792	p.Thr827Met	LB/B	rs371135988	-
ADGRD1	Q6QNK2	VAR_077793	p.Ala831Thr	LB/B	rs146661482	-
ADGRD1	Q6QNK2	VAR_077794	p.Ala836Thr	LB/B	rs144023497	-
ADGRD1	Q6QNK2	VAR_077795	p.Ala836Val	LB/B	rs367789023	-
ADGRD1	Q6QNK2	VAR_077796	p.Met851Thr	LB/B	rs78058857	-
ADGRD1	Q6QNK2	VAR_077797	p.Arg868His	LB/B	rs61740366	-
ADGRD1	Q6QNK2	VAR_077798	p.Val869Ile	LB/B	rs377434309	-
ADGRD1	Q6QNK2	VAR_077799	p.Asp870Asn	LB/B	rs143670024	-
ADGRD1	Q6QNK2	VAR_077800	p.Val874Met	LB/B	rs148442158	-
ADGRD2	Q7Z7M1	VAR_083658	p.Pro170Ser	LB/B	-	-
ADGRD2	Q7Z7M1	VAR_083659	p.Ala630Thr	LB/B	-	-
ADGRE1	Q14246	VAR_027616	p.Ala57Thr	LB/B	rs330877	-
ADGRE1	Q14246	VAR_027617	p.Ser140Arg	LB/B	rs330880	-
ADGRE1	Q14246	VAR_027618	p.Asp174Asn	LB/B	rs897738	-
ADGRE1	Q14246	VAR_027619	p.Asn254Ser	LB/B	rs443658	-
ADGRE1	Q14246	VAR_027620	p.Ala298Val	LB/B	rs370094	-
ADGRE1	Q14246	VAR_027621	p.Thr389Met	LB/B	rs466876	-
ADGRE1	Q14246	VAR_027622	p.Ile424Val	LB/B	rs457857	-
ADGRE1	Q14246	VAR_027623	p.Lys496Gln	LB/B	rs373533	-
ADGRE1	Q14246	VAR_027624	p.Ile539Val	LB/B	rs461645	-
ADGRE1	Q14246	VAR_027625	p.Val589Ile	LB/B	rs7256147	-
ADGRE1	Q14246	VAR_027626	p.Phe691Cys	LB/B	rs2229769	-
ADGRE1	Q14246	VAR_027627	p.Val724Leu	LB/B	rs10406580	-
ADGRE1	Q14246	VAR_046976	p.Arg2Leu	LB/B	rs34176643	-
ADGRE1	Q14246	VAR_046977	p.Met663Thr	LB/B	rs2228539	-
ADGRE2	Q9UHX3	VAR_026719	p.Thr605Ile	LB/B	rs4410209	-
ADGRE2	Q9UHX3	VAR_026720	p.Leu614Phe	LB/B	rs2524383	-
ADGRE2	Q9UHX3	VAR_026721	p.Ser665Phe	LB/B	rs3752187	-
ADGRE2	Q9UHX3	VAR_061229	p.Ala314Val	LB/B	rs35612307	-
ADGRE2	Q9UHX3	VAR_061230	p.Glu720Asp	LB/B	rs57865820	-
ADGRE2	Q9UHX3	VAR_078578	p.Cys492Tyr	LP/P	rs199718602	Vibratory urticaria (VBU) [MIM:125630]
ADGRE3	Q9BY15	VAR_024472	p.Glu127Gln	LB/B	rs4606855	-

ADGRE3 Q9BY15 VAR_055926 p.Ala236Val LB/B rs34226397 -
ADGRE3 Q9BY15 VAR_060442 p.Arg385Gln LB/B rs45508602 -
ADGRE5 P48960 VAR_017760 p.Arg367Gln LB/B rs2230748 -
ADGRF1 Q5T601 VAR_055928 p.Ile787Val LB/B rs1226475 -
ADGRF2 Q8IZF7 VAR_024473 p.Gln148Arg LB/B rs6907125 -
ADGRF2 Q8IZF7 VAR_024474 p.Ile467Val LB/B rs9381594 -
ADGRF3 Q8IZF5 VAR_024475 p.Ala404Thr LB/B rs2052937 -
ADGRF4 Q8IZF3 VAR_024476 p.Lys541Asn LB/B rs9369738 -
ADGRF4 Q8IZF3 VAR_036224 p.Ser674Leu US rs572583506 A breast cancer sample
ADGRF4 Q8IZF3 VAR_055930 p.Arg507Cys LB/B rs12110938 -
ADGRF5 Q8IZF2 VAR_024477 p.Met856Thr LB/B rs547499 -
ADGRF5 Q8IZF2 VAR_025326 p.Thr604Met LB/B rs586024 -
ADGRF5 Q8IZF2 VAR_055291 p.Val801Ile LB/B rs9395218 -
ADGRG1 Q9Y653 VAR_017910 p.Ser281Arg LB/B rs1801257 -
ADGRG1 Q9Y653 VAR_017911 p.Gln306His LB/B rs1801255 -
ADGRG1 Q9Y653 VAR_026242 p.Arg38Trp LP/P rs121908462 Polymicrogyria, bilateral frontoparietal (BFPP) [MIM:606854]
ADGRG1 Q9Y653 VAR_026243 p.Tyr88Cys LP/P rs121908466 Polymicrogyria, bilateral frontoparietal (BFPP) [MIM:606854]
ADGRG1 Q9Y653 VAR_026244 p.Cys91Ser LP/P rs121908465 Polymicrogyria, bilateral frontoparietal (BFPP) [MIM:606854]
ADGRG1 Q9Y653 VAR_026245 p.Cys346Ser LP/P rs121908463 Polymicrogyria, bilateral frontoparietal (BFPP) [MIM:606854]
ADGRG1 Q9Y653 VAR_026246 p.Arg565Trp LP/P rs121908464 Polymicrogyria, bilateral frontoparietal (BFPP) [MIM:606854]
ADGRG1 Q9Y653 VAR_049457 p.Met493Thr LB/B rs17379472 -
ADGRG1 Q9Y653 VAR_049458 p.Pro527Leu LB/B rs16958679 -
ADGRG1 Q9Y653 VAR_069581 p.Arg38Gln LP/P rs764367185 Polymicrogyria, bilateral frontoparietal (BFPP) [MIM:606854]
ADGRG1 Q9Y653 VAR_069582 p.Trp349Ser LP/P - Polymicrogyria, bilateral frontoparietal (BFPP) [MIM:606854]
ADGRG1 Q9Y653 VAR_069583 p.Glu496Lys LP/P rs556518689 Polymicrogyria, bilateral frontoparietal (BFPP) [MIM:606854]
ADGRG1 Q9Y653 VAR_069584 p.Leu640Arg LP/P - Polymicrogyria, bilateral frontoparietal (BFPP) [MIM:606854]
ADGRG2 Q8IZP9 VAR_055289 p.His290Gln LB/B rs35974297 -
ADGRG2 Q8IZP9 VAR_055290 p.Asn771Ser LB/B rs3924227 -
ADGRG2 Q8IZP9 VAR_076259 p.Pro224Ser LB/B rs140334931 -
ADGRG2 Q8IZP9 VAR_080773 p.Asn64Ser US rs746638813 -
ADGRG3 Q86Y34 VAR_055927 p.Ala430Thr LB/B rs2290178 -
ADGRG4 Q8IZF6 VAR_055929 p.Ile276Met LB/B rs4829829 -
ADGRG4 Q8IZF6 VAR_059329 p.Pro368His LB/B rs5930931 -
ADGRG4 Q8IZF6 VAR_059330 p.Thr1213Asn LB/B rs4829830 -
ADGRG4 Q8IZF6 VAR_059331 p.Ser1540Pro LB/B rs912002 -
ADGRG4 Q8IZF6 VAR_059332 p.Phe1791Leu LB/B rs5930932 -
ADGRG6 Q86SQ4 VAR_024478 p.Lys230Gln LB/B rs11155242 -
ADGRG6 Q86SQ4 VAR_054128 p.Ser123Gly LB/B rs17280293 -
ADGRG6 Q86SQ4 VAR_054129 p.Gln1127Arg LB/B rs1262686 -

ADGRG6 Q86SQ4 VAR_075146 p.Val741Glu LP/P - Lethal congenital contracture syndrome 9 (LCCS9) [MIM:616503]
ADGRG6 Q86SQ4 VAR_075147 p.Val769Glu LP/P rs793888525 Lethal congenital contracture syndrome 9 (LCCS9) [MIM:616503]
ADGRG6 Q86SQ4 VAR_076965 p.Arg1057Gln LB/B rs536714306 -
ADGRG7 Q96K78 VAR_049459 p.Thr645Ser LB/B rs16842529 -
ADGRG7 Q96K78 VAR_049460 p.Arg756His LB/B rs9872512 -
ADGRG7 Q96K78 VAR_055138 p.Lys151Glu LB/B rs1144122 -
ADGRL1 O94910 VAR_049463 p.Glu595Gln LB/B rs34759320 -
ADGRL2 O95490 VAR_077836 p.Arg467Thr US - -
ADGRL3 Q9HAR2 VAR_055934 p.Arg465Gln LB/B rs35106420 -
ADGRL3 Q9HAR2 VAR_064477 p.Ala247Ser LB/B - -
ADGRL3 Q9HAR2 VAR_064478 p.Thr770Met LB/B - -
ADGRL3 Q9HAR2 VAR_064479 p.Leu915Val LB/B - -
ADGRL4 Q9HBW9 VAR_047072 p.Val300Leu LB/B rs12754818 -
ADGRL4 Q9HBW9 VAR_047073 p.His599Gln LB/B rs1968956 -
ADGRL4 Q9HBW9 VAR_047074 p.Ala620Gly LB/B rs2275902 -
ADGRV1 Q8WXG9 VAR_025995 p.Leu127Arg LB/B rs41311333 -
ADGRV1 Q8WXG9 VAR_025996 p.Arg249Lys LB/B rs41303344 -
ADGRV1 Q8WXG9 VAR_025997 p.Leu1093Phe LB/B rs2366777 -
ADGRV1 Q8WXG9 VAR_025998 p.Thr1927Met LB/B rs17544552 -
ADGRV1 Q8WXG9 VAR_025999 p.Val1951Ile LB/B rs4916684 -
ADGRV1 Q8WXG9 VAR_026000 p.Asn1985Asp LB/B rs41303352 -
ADGRV1 Q8WXG9 VAR_026001 p.Pro1987Leu LB/B rs4916685 -
ADGRV1 Q8WXG9 VAR_026002 p.Leu2004Phe LB/B rs16868972 -
ADGRV1 Q8WXG9 VAR_026003 p.Tyr2232Cys LB/B rs10037067 -
ADGRV1 Q8WXG9 VAR_026004 p.Asn2345Ser LB/B rs2366926 -
ADGRV1 Q8WXG9 VAR_026005 p.Gly2379Ala LB/B - -
ADGRV1 Q8WXG9 VAR_026006 p.Asn2584Ser LB/B rs1878878 -
ADGRV1 Q8WXG9 VAR_026007 p.Ser2764Leu LB/B rs16869016 -
ADGRV1 Q8WXG9 VAR_026008 p.Ala2803Thr LB/B rs111033530 -
ADGRV1 Q8WXG9 VAR_026009 p.Ala3217Val LB/B rs114137750 -
ADGRV1 Q8WXG9 VAR_026010 p.Gly3248Asp LB/B rs16869032 -
ADGRV1 Q8WXG9 VAR_026011 p.Glu3471Lys LB/B rs2366928 -
ADGRV1 Q8WXG9 VAR_026012 p.Glu5344Gly LB/B rs2438374 -
ADGRV1 Q8WXG9 VAR_026013 p.Thr5437Ala LB/B rs770471921 -
ADGRV1 Q8WXG9 VAR_046346 p.Val551Ala LB/B rs6889939 -
ADGRV1 Q8WXG9 VAR_046347 p.Ile1187Val LB/B rs16868935 -
ADGRV1 Q8WXG9 VAR_046348 p.Thr1916Ile LB/B rs35791889 -
ADGRV1 Q8WXG9 VAR_046349 p.Arg2097Cys LB/B rs16868974 -
ADGRV1 Q8WXG9 VAR_046350 p.Val3094Ile LB/B rs13157270 -
ADGRV1 Q8WXG9 VAR_046351 p.Phe3347Leu LB/B rs10067636 -
ADGRV1 Q8WXG9 VAR_046352 p.Glu3867Ala LB/B rs16869088 -
ADGRV1 Q8WXG9 VAR_055933 p.Glu3868Ala LB/B rs16869088 -
ADGRV1 Q8WXG9 VAR_068032 p.Arg4789Trp LP/P rs1131691924 Usher syndrome 2C (USH2C) [MIM:605472]
ADGRV1 Q8WXG9 VAR_068033 p.His5978Arg LP/P rs756460900 Usher syndrome 2C (USH2C) [MIM:605472]
ADH1B P00325 VAR_000426 p.Arg48His LB/B rs1229984 -
ADH1B P00325 VAR_000427 p.Arg370Cys LB/B rs2066702 -
ADH1B P00325 VAR_019322 p.Asn57Lys LB/B rs1041969 -

ADH1B P00325 VAR_019323 p.Thr60Ser LB/B rs6413413 -
ADH1C P00326 VAR_000428 p.Arg272Gln LB/B rs1693482 -
ADH1C P00326 VAR_000429 p.Ile350Val LB/B rs698 -
ADH1C P00326 VAR_023992 p.Arg48His LB/B rs35385902 -
ADH1C P00326 VAR_023993 p.Pro166Ser LB/B rs34195308 -
ADH1C P00326 VAR_023994 p.Pro352Thr LB/B rs35719513 -
ADH4 P08319 VAR_023461 p.Ile309Val LB/B rs1126671 -
ADH4 P08319 VAR_023462 p.Arg318His LB/B rs29001219 -
ADH4 P08319 VAR_023463 p.Val374Ile LB/B rs1126673 -
ADH5 P11766 VAR_025823 p.Leu163Ser LB/B rs28730623 -
ADH5 P11766 VAR_025824 p.Val309Ile LB/B rs28730628 -
ADH5 P11766 VAR_048199 p.Asp353Glu LB/B rs16996593 -
ADH6 P28332 VAR_022655 p.Cys102Gly LB/B rs28720152 -
ADH6 P28332 VAR_022656 p.Ile114Val LB/B rs28720153 -
ADH6 P28332 VAR_048198 p.Thr151Pro LB/B rs34582580 -
ADH7 P40394 VAR_024364 p.Gly92Ala LB/B rs1573496 -
ADHFE1 Q8IWW8 VAR_039470 p.Asp242Val US - A breast cancer sample
ADHFE1 Q8IWW8 VAR_054015 p.Cys449Arg LB/B rs1060242 -
ADIPOQ Q15848 VAR_013273 p.Gly84Arg LB/B rs199646033 -
ADIPOQ Q15848 VAR_013274 p.Arg112Cys LP/P rs121917815 Adiponectin deficiency (ADPND) [MIM:612556]
ADIPOQ Q15848 VAR_013275 p.Val117Met LB/B rs747223144 -
ADIPOQ Q15848 VAR_013276 p.Ile164Thr LB/B rs185847354 -
ADIPOQ Q15848 VAR_013277 p.Arg221Ser LB/B rs138773406 -
ADIPOQ Q15848 VAR_013278 p.His241Pro LB/B rs141205818 -
ADIPOQ Q15848 VAR_027395 p.Gly90Ser LB/B rs62625753 -
ADIPOQ Q15848 VAR_027396 p.Tyr111His LB/B rs17366743 -
ADIPOR2 Q86V24 VAR_048203 p.Gln39Arg LB/B rs12298275 -
ADK P55263 VAR_066640 p.Gly30Glu LP/P rs397514454 Hypermethioninemia due to adenosine kinase deficiency (HMAKD) [MIM:614300]
ADK P55263 VAR_066641 p.Asp235Ala LP/P rs397514453 Hypermethioninemia due to adenosine kinase deficiency (HMAKD) [MIM:614300]
ADK P55263 VAR_066642 p.Ala318Glu LP/P rs397514452 Hypermethioninemia due to adenosine kinase deficiency (HMAKD) [MIM:614300]
ADM P35318 VAR_014861 p.Ser50Arg LB/B rs5005 -
ADM P35318 VAR_048205 p.Pro85Arg LB/B rs2228573 -
ADNP Q9H2P0 VAR_078696 p.Gly1094Val US rs780706686 -
ADO Q96SZ5 VAR_025333 p.Gly25Trp LB/B rs2236295 -
ADO Q96SZ5 VAR_025334 p.Pro39Ala LB/B rs10995311 -
ADO Q96SZ5 VAR_033691 p.Pro266Ser LB/B rs569705 -
ADORA1 P30542 VAR_035754 p.Glu170Lys US rs899207013 A colorectal cancer sample
ADORA1 P30542 VAR_044138 p.Ala43Ser LB/B rs11547175 -
ADORA1 P30542 VAR_044139 p.Ser50Pro LB/B rs11547174 -
ADORA1 P30542 VAR_044140 p.Arg105His LB/B rs11547176 -
ADORA1 P30542 VAR_044141 p.Pro261Gln LB/B rs17852405 -
ADORA1 P30542 VAR_078549 p.Gly279Ser US rs748346254 -
ADORA2A P29274 VAR_003451 p.Gly392Arg LB/B rs1277013918 -
ADORA2A P29274 VAR_011835 p.Ala50Val LB/B rs4530 -
ADORA2A P29274 VAR_011836 p.Arg300His LB/B rs4990 -
ADORA3 P0DMS8 VAR_035755 p.Ala105Thr US rs746154553 A colorectal cancer sample

ADORA3	P0DMS8	VAR_049366	p.Ile248Leu	LB/B	rs35511654	-
ADORA3	P0DMS8	VAR_049367	p.Met266Lys	LB/B	rs2800889	-
ADPGK	Q9BRR6	VAR_060085	p.Lys184Arg	LB/B	rs8024644	-
ADPRHL1	Q8NDY3	VAR_048890	p.Ala7Val	LB/B	rs9577273	-
ADPRM	Q3LIE5	VAR_032125	p.Leu92Arg	LB/B	rs34940296	-
ADPRM	Q3LIE5	VAR_032126	p.Glu337Gly	LB/B	rs406446	-
ADPRS	Q9NX46	VAR_030579	p.Glu209Lys	LB/B	rs2236387	-
ADPRS	Q9NX46	VAR_081264	p.Asp34Asn	US	rs1557732234	Neurodegeneration, childhood-onset, stress-induced, with variable ataxia and seizures (CONDSIAS) [MIM:618170]
ADPRS	Q9NX46	VAR_081265	p.Thr79Pro	LP/P	rs1557733311	Neurodegeneration, childhood-onset, stress-induced, with variable ataxia and seizures (CONDSIAS) [MIM:618170]
ADPRS	Q9NX46	VAR_081267	p.Ser177Leu	US	rs200626873	Neurodegeneration, childhood-onset, stress-induced, with variable ataxia and seizures (CONDSIAS) [MIM:618170]
ADPRS	Q9NX46	VAR_081270	p.Val335Gly	LP/P	rs201735454	Neurodegeneration, childhood-onset, stress-induced, with variable ataxia and seizures (CONDSIAS) [MIM:618170]
ADRA1A	P35348	VAR_019509	p.Cys347Arg	LB/B	rs1048101	-
ADRA1A	P35348	VAR_035756	p.Gly40Trp	US	-	A breast cancer sample
ADRA1A	P35348	VAR_049370	p.Ile200Ser	LB/B	rs2229125	-
ADRA1A	P35348	VAR_049371	p.Lys414Arg	LB/B	rs3730247	-
ADRA1A	P35348	VAR_049372	p.Glu465Asp	LB/B	rs2229126	-
ADRA1B	P35368	VAR_019510	p.Val51Gly	LB/B	rs8192448	-
ADRA2A	P08913	VAR_014957	p.Asn266Lys	LB/B	rs1800035	-
ADRA2A	P08913	VAR_055908	p.Cys416Ser	LB/B	rs35658213	-
ADRA2B	P18089	VAR_025099	p.Gly211Ala	LB/B	rs9333568	-
ADRA2B	P18089	VAR_025100	p.Val379Gly	LB/B	rs527655811	-
ADRA2B	P18089	VAR_033462	p.Val376Ile	LB/B	rs1431850417	-
ADRA2B	P18089	VAR_033463	p.Val379Ile	LB/B	rs29000569	-
ADRB1	P08588	VAR_009879	p.Ser49Gly	LB/B	rs1801252	-
ADRB1	P08588	VAR_009880	p.Arg389Gly	LB/B	rs1801253	-
ADRB1	P08588	VAR_018742	p.Arg389Leu	LB/B	rs17875445	-
ADRB1	P08588	VAR_055909	p.Ala26Val	LB/B	rs34844626	-
ADRB1	P08588	VAR_055910	p.Ala29Thr	LB/B	rs35720093	-
ADRB1	P08588	VAR_055911	p.Arg31Gln	LB/B	rs35230616	-
ADRB1	P08588	VAR_055912	p.Arg399His	LB/B	rs36052953	-
ADRB1	P08588	VAR_055913	p.His405Tyr	LB/B	rs35705839	-
ADRB1	P08588	VAR_082587	p.Ala187Val	LB/B	rs776439595	-
ADRB2	P07550	VAR_003452	p.Gly16Arg	LB/B	rs1042713	-
ADRB2	P07550	VAR_003453	p.Glu27Gln	LB/B	rs1042714	-
ADRB2	P07550	VAR_003454	p.Val34Met	LB/B	rs990810566	-
ADRB2	P07550	VAR_003455	p.Thr164Ile	LB/B	rs1800888	-
ADRB2	P07550	VAR_009124	p.Ile159Leu	LB/B	-	-
ADRB2	P07550	VAR_009125	p.Ile159Phe	LB/B	-	-
ADRB2	P07550	VAR_009394	p.Lys375Arg	LB/B	rs771585355	-
ADRB2	P07550	VAR_025101	p.Ser220Cys	LB/B	rs3729943	-
ADRB2	P07550	VAR_049373	p.Asn15Ser	LB/B	rs33973603	-
ADRB3	P13945	VAR_003456	p.Trp64Arg	LB/B	rs4994	-
ADRB3	P13945	VAR_014166	p.Thr265Met	LB/B	rs4995	-
ADRB3	P13945	VAR_025102	p.Arg353Cys	LB/B	rs36031925	-
ADRB3	P13945	VAR_029205	p.Glu249Lys	LB/B	rs28364012	-

ADSL	P30566	VAR_000680	p.Ser438Pro	LP/P	rs119450940	Adenylosuccinase deficiency (ADSLD) [MIM:103050]
ADSL	P30566	VAR_007972	p.Ile72Val	LP/P	-	Adenylosuccinase deficiency (ADSLD) [MIM:103050]
ADSL	P30566	VAR_007973	p.Arg141Trp	LP/P	rs756210458	Adenylosuccinase deficiency (ADSLD) [MIM:103050]
ADSL	P30566	VAR_007974	p.Arg190Gln	LP/P	rs28941471	Adenylosuccinase deficiency (ADSLD) [MIM:103050]
ADSL	P30566	VAR_007975	p.Lys246Glu	LP/P	rs119450944	Adenylosuccinase deficiency (ADSLD) [MIM:103050]
ADSL	P30566	VAR_007976	p.Arg303Cys	LP/P	rs373458753	Adenylosuccinase deficiency (ADSLD) [MIM:103050]
ADSL	P30566	VAR_007977	p.Ser395Arg	LP/P	-	Adenylosuccinase deficiency (ADSLD) [MIM:103050]
ADSL	P30566	VAR_007978	p.Arg426His	LP/P	rs119450941	Adenylosuccinase deficiency (ADSLD) [MIM:103050]
ADSL	P30566	VAR_016930	p.Ala2Val	LP/P	rs143083947	Adenylosuccinase deficiency (ADSLD) [MIM:103050]
ADSL	P30566	VAR_016931	p.Met26Leu	LP/P	rs1311171245	Adenylosuccinase deficiency (ADSLD) [MIM:103050]
ADSL	P30566	VAR_016932	p.Thr450Ser	LP/P	rs372895468	Adenylosuccinase deficiency (ADSLD) [MIM:103050]
ADSL	P30566	VAR_017078	p.Ala3Val	LP/P	-	Adenylosuccinase deficiency (ADSLD) [MIM:103050]
ADSL	P30566	VAR_017079	p.Pro100Ala	LP/P	rs119450942	Adenylosuccinase deficiency (ADSLD) [MIM:103050]
ADSL	P30566	VAR_017080	p.Tyr114His	LP/P	rs374259530	Adenylosuccinase deficiency (ADSLD) [MIM:103050]
ADSL	P30566	VAR_017081	p.Arg194Cys	LP/P	rs1465152683	Adenylosuccinase deficiency (ADSLD) [MIM:103050]
ADSL	P30566	VAR_017082	p.Asp268Asn	LP/P	rs746501563	Adenylosuccinase deficiency (ADSLD) [MIM:103050]
ADSL	P30566	VAR_017083	p.Leu311Val	LP/P	-	Adenylosuccinase deficiency (ADSLD) [MIM:103050]
ADSL	P30566	VAR_017084	p.Pro318Leu	LP/P	rs202064195	Adenylosuccinase deficiency (ADSLD) [MIM:103050]
ADSL	P30566	VAR_017085	p.Val364Met	LP/P	rs370851726	Adenylosuccinase deficiency (ADSLD) [MIM:103050]
ADSL	P30566	VAR_017086	p.Arg374Trp	LP/P	rs376533026	Adenylosuccinase deficiency (ADSLD) [MIM:103050]
ADSL	P30566	VAR_017087	p.Arg396Cys	LP/P	rs755492501	Adenylosuccinase deficiency (ADSLD) [MIM:103050]
ADSL	P30566	VAR_017088	p.Arg396His	LP/P	rs763542069	Adenylosuccinase deficiency (ADSLD) [MIM:103050]
ADSL	P30566	VAR_017089	p.Asp422Tyr	LP/P	rs119450943	Adenylosuccinase deficiency (ADSLD) [MIM:103050]
ADSL	P30566	VAR_017090	p.Leu423Val	LP/P	-	Adenylosuccinase deficiency (ADSLD) [MIM:103050]
ADSL	P30566	VAR_017091	p.Asp430Asn	LP/P	rs554254383	Adenylosuccinase deficiency (ADSLD) [MIM:103050]
ADSL	P30566	VAR_017092	p.Ser447Pro	LP/P	rs777821034	Adenylosuccinase deficiency (ADSLD) [MIM:103050]
ADSL	P30566	VAR_017093	p.Arg452Pro	LP/P	-	Adenylosuccinase deficiency (ADSLD) [MIM:103050]
ADSL	P30566	VAR_037883	p.Ser31Asn	LB/B	rs5757921	-
ADSL	P30566	VAR_037884	p.Lys147Met	LB/B	rs11089991	-

ADSS1 Q8N142 VAR_076998 p.Asp261Asn LP/P rs140614802 Myopathy, distal, 5 (MPD5) [MIM:617030]
ADSS2 P30520 VAR_051881 p.Leu179Phe LB/B rs12134870 -
ADTRP Q96IZ2 VAR_024365 p.Val202Ile LB/B rs2076185 -
AEBP1 Q8IUX7 VAR_043118 p.Pro273Thr LB/B rs2537188 -
AEBP1 Q8IUX7 VAR_043119 p.Asp648Glu LB/B rs11770649 -
AEBP1 Q8IUX7 VAR_043120 p.Pro1001Leu LB/B rs4724285 -
AEBP1 Q8IUX7 VAR_043121 p.Lys1133Glu LB/B rs13928 -
AEBP1 Q8IUX7 VAR_043122 p.Val1148Ile LB/B rs13898 -
AEN Q8WTP8 VAR_039651 p.Pro15Leu LB/B rs3743477 -
AEN Q8WTP8 VAR_039652 p.Ser88Cys LB/B rs8026929 -
AEN Q8WTP8 VAR_039653 p.Asn140Asp LB/B rs8027765 -
AFAP1 Q8N556 VAR_038578 p.Ser403Cys LB/B rs28406288 -
AFAP1 Q8N556 VAR_038579 p.Val518Met LB/B rs41264705 -
AFAP1L2 Q8N4X5 VAR_050505 p.Gly138Arg LB/B rs11196689 -
AFAP1L2 Q8N4X5 VAR_050506 p.Ser366Arg LB/B rs7075067 -
AFAP1L2 Q8N4X5 VAR_050507 p.Thr522Ser LB/B rs2781806 -
AFAP1L2 Q8N4X5 VAR_054214 p.Glu726Lys LB/B rs11599051 -
AFF1 P51825 VAR_020370 p.Pro209Ala LB/B rs3733378 -
AFF1 P51825 VAR_036130 p.Gln1204Lys US - A breast cancer sample
AFF2 P51816 VAR_028217 p.Leu1185Met LB/B rs12858959 -
AFF3 P51826 VAR_030805 p.Asn358Ser LB/B rs4851223 -
AFF3 P51826 VAR_030806 p.Asn494Ser LB/B rs1047265 -
AFF3 P51826 VAR_080758 p.Gly1215Val US - -
AFF4 Q9UHB7 VAR_053003 p.Thr136Pro LB/B rs34527550 -
AFF4 Q9UHB7 VAR_064693 p.Ser757Thr US - -
AFF4 Q9UHB7 VAR_073790 p.Thr254Ala LP/P rs786205233 CHOPS syndrome (CHOPS) [MIM:616368]
AFF4 Q9UHB7 VAR_073791 p.Thr254Ser LP/P rs786205679 CHOPS syndrome (CHOPS) [MIM:616368]
AFF4 Q9UHB7 VAR_073792 p.Arg258Trp LP/P rs786205680 CHOPS syndrome (CHOPS) [MIM:616368]
AFG3L2 Q9Y4W6 VAR_063544 p.Asn432Thr LP/P rs151344512 Spinocerebellar ataxia 28 (SCA28) [MIM:610246]
AFG3L2 Q9Y4W6 VAR_063545 p.Glu691Lys LP/P rs151344520 Spinocerebellar ataxia 28 (SCA28) [MIM:610246]
AFG3L2 Q9Y4W6 VAR_063546 p.Ala694Glu LP/P rs151344521 Spinocerebellar ataxia 28 (SCA28) [MIM:610246]
AFG3L2 Q9Y4W6 VAR_063547 p.Arg702Gln LP/P rs151344523 Spinocerebellar ataxia 28 (SCA28) [MIM:610246]
AFG3L2 Q9Y4W6 VAR_064402 p.Thr654Ile LP/P rs151344513 Spinocerebellar ataxia 28 (SCA28) [MIM:610246]
AFG3L2 Q9Y4W6 VAR_064403 p.Met666Arg LP/P rs151344515 Spinocerebellar ataxia 28 (SCA28) [MIM:610246]
AFG3L2 Q9Y4W6 VAR_064404 p.Met666Thr LP/P rs151344515 Spinocerebellar ataxia 28 (SCA28) [MIM:610246]
AFG3L2 Q9Y4W6 VAR_064405 p.Met666Val LP/P rs151344514 Spinocerebellar ataxia 28 (SCA28) [MIM:610246]
AFG3L2 Q9Y4W6 VAR_064406 p.Gly671Glu LP/P rs151344518 Spinocerebellar ataxia 28 (SCA28) [MIM:610246]
AFG3L2 Q9Y4W6 VAR_064407 p.Gly671Arg LP/P rs151344517 Spinocerebellar ataxia 28 (SCA28) [MIM:610246]
AFG3L2 Q9Y4W6 VAR_064408 p.Glu700Lys LP/P rs151344522 Spinocerebellar ataxia 28 (SCA28) [MIM:610246]

AFG3L2 Q9Y4W6 VAR_067330 p.Tyr616Cys LP/P rs387906889 Spastic ataxia 5, autosomal recessive (SPAX5) [MIM:614487]
AFG3L2 Q9Y4W6 VAR_075198 p.Tyr689His LP/P - Spinocerebellar ataxia 28 (SCA28) [MIM:610246]
AFG3L2 Q9Y4W6 VAR_075199 p.Tyr689Asn LP/P - Spinocerebellar ataxia 28 (SCA28) [MIM:610246]
AFG3L2 Q9Y4W6 VAR_080736 p.Leu621Val US - Spinocerebellar ataxia 28 (SCA28) [MIM:610246]
AFG3L2 Q9Y4W6 VAR_084603 p.Glu74Ala US - Optic atrophy 12 (OPA12) [MIM:618977]
AFG3L2 Q9Y4W6 VAR_084604 p.Lys306Glu US - Spastic ataxia 5, autosomal recessive (SPAX5) [MIM:614487]
AFG3L2 Q9Y4W6 VAR_084605 p.Gly337Glu LP/P - Optic atrophy 12 (OPA12) [MIM:618977]
AFG3L2 Q9Y4W6 VAR_084606 p.Gly337Arg LP/P - Optic atrophy 12 (OPA12) [MIM:618977]
AFG3L2 Q9Y4W6 VAR_084607 p.Leu346Phe US - Optic atrophy 12 (OPA12) [MIM:618977]
AFG3L2 Q9Y4W6 VAR_084608 p.Glu376Lys US - Optic atrophy 12 (OPA12) [MIM:618977]
AFG3L2 Q9Y4W6 VAR_084609 p.Phe377Ser US - Optic atrophy 12 (OPA12) [MIM:618977]
AFG3L2 Q9Y4W6 VAR_084610 p.Asp407Gly US - Optic atrophy 12 (OPA12) [MIM:618977]
AFG3L2 Q9Y4W6 VAR_084611 p.Arg416Ser US - Optic atrophy 12 (OPA12) [MIM:618977]
AFG3L2 Q9Y4W6 VAR_084612 p.Thr430Ile US - Optic atrophy 12 (OPA12) [MIM:618977]
AFG3L2 Q9Y4W6 VAR_084613 p.Ala462Val LP/P - Optic atrophy 12 (OPA12) [MIM:618977]
AFG3L2 Q9Y4W6 VAR_084613 p.Ala462Val LP/P - Spastic ataxia 5, autosomal recessive (SPAX5) [MIM:614487]
AFG3L2 Q9Y4W6 VAR_084614 p.Arg465Lys LP/P - Optic atrophy 12 (OPA12) [MIM:618977]
AFG3L2 Q9Y4W6 VAR_084615 p.Arg468Cys LP/P - Optic atrophy 12 (OPA12) [MIM:618977]
AFG3L2 Q9Y4W6 VAR_084616 p.Pro514Leu LP/P - Optic atrophy 12 (OPA12) [MIM:618977]
AFG3L2 Q9Y4W6 VAR_084617 p.Tyr605Cys US - Optic atrophy 12 (OPA12) [MIM:618977]
AFG3L2 Q9Y4W6 VAR_084618 p.Gln620Lys LP/P - Spastic ataxia 5, autosomal recessive (SPAX5) [MIM:614487]
AFG3L2 Q9Y4W6 VAR_084619 p.Thr644Ser US - Optic atrophy 12 (OPA12) [MIM:618977]
AFM P43652 VAR_048218 p.Thr404Ser LB/B rs2276444 -
AFM P43652 VAR_061003 p.Arg395His LB/B rs41265665 -
AFP P02771 VAR_012049 p.Ala570Gly LB/B rs7790 -
AFP P02771 VAR_033928 p.Lys187Gln LB/B rs35765619 -
AFTPH Q6ULP2 VAR_056728 p.Asp233Gly LB/B rs35986567 -
AFTPH Q6ULP2 VAR_056729 p.Glu301Lys LB/B rs3770740 -
AFTPH Q6ULP2 VAR_056730 p.Asn550Ser LB/B rs3770739 -
AGA P20933 VAR_005069 p.Gly60Asp LP/P rs121964907 Aspartylglucosaminuria (AGU) [MIM:208400]
AGA P20933 VAR_005070 p.Ser72Pro LP/P rs121964909 Aspartylglucosaminuria (AGU) [MIM:208400]
AGA P20933 VAR_005071 p.Ala101Val LP/P rs121964908 Aspartylglucosaminuria (AGU) [MIM:208400]
AGA P20933 VAR_005072 p.Arg161Gln LP/P rs192195150 Aspartylglucosaminuria (AGU) [MIM:208400]
AGA P20933 VAR_005073 p.Cys163Ser LP/P rs121964904 Aspartylglucosaminuria (AGU) [MIM:208400]
AGA P20933 VAR_005074 p.Gly302Arg LP/P rs121964905 Aspartylglucosaminuria (AGU) [MIM:208400]
AGA P20933 VAR_005075 p.Cys306Arg LP/P rs121964906 Aspartylglucosaminuria (AGU) [MIM:208400]
AGA P20933 VAR_015427 p.Val12Leu US rs74626221 Aspartylglucosaminuria (AGU) [MIM:208400]
AGA P20933 VAR_015428 p.Gly100Glu LP/P rs386833421 Aspartylglucosaminuria (AGU) [MIM:208400]
AGA P20933 VAR_015429 p.Phe135Ser LP/P rs386833427 Aspartylglucosaminuria (AGU) [MIM:208400]
AGA P20933 VAR_015430 p.Gly252Glu LP/P rs386833433 Aspartylglucosaminuria (AGU) [MIM:208400]
AGA P20933 VAR_015431 p.Gly252Arg LP/P rs386833432 Aspartylglucosaminuria (AGU) [MIM:208400]
AGA P20933 VAR_015432 p.Thr257Ile LP/P rs386833434 Aspartylglucosaminuria (AGU) [MIM:208400]
AGA P20933 VAR_033533 p.Thr149Ser LB/B rs2228119 -
AGA P20933 VAR_061026 p.Thr322Ile LB/B rs56849061 -
AGAP1 Q9UPQ3 VAR_019550 p.Glu829Lys LB/B rs15718 -

AGAP1	Q9UPQ3	VAR_026446	p.Ser82Gly	US	-	-
AGAP1	Q9UPQ3	VAR_026447	p.Asp148Gly	LB/B	rs17855721	-
AGAP1	Q9UPQ3	VAR_026448	p.Val671Ile	LB/B	rs2034648	-
AGAP1	Q9UPQ3	VAR_026449	p.Arg798Gly	LB/B	rs762355360	-
AGAP1	Q9UPQ3	VAR_026450	p.Pro854Thr	LB/B	rs368301945	-
AGAP11	Q8TF27	VAR_042518	p.Ile82Val	LB/B	rs2641563	-
AGAP2	Q99490	VAR_022046	p.Gly507Ser	LB/B	rs2301553	-
AGAP2	Q99490	VAR_026438	p.Val455Ala	US	-	A glioblastoma cell line
AGAP2	Q99490	VAR_026439	p.Arg518Gly	US	-	A sarcoma cell line
AGAP2	Q99490	VAR_026440	p.Thr568Ile	US	-	-
AGAP2	Q99490	VAR_026441	p.Ala651Val	US	-	A glioblastoma cell line
AGAP2	Q99490	VAR_026442	p.Glu767Val	US	-	A glioblastoma cell line
AGAP2	Q99490	VAR_026443	p.Asn939Asp	US	-	A glioblastoma cell line
AGAP2	Q99490	VAR_026444	p.Val947Met	US	-	A sarcoma cell line
AGAP2	Q99490	VAR_026445	p.Ser1022Pro	US	-	A glioblastoma cell line
AGAP2	Q99490	VAR_036183	p.Thr339Ala	US	-	A breast cancer sample
AGAP2	Q99490	VAR_036184	p.Asp816Tyr	US	-	A breast cancer sample
AGAP2	Q99490	VAR_055532	p.Gly1124Val	LB/B	rs238521	-
AGAP4	Q96P64	VAR_031804	p.Lys661Glu	LB/B	rs15718	-
AGBL1	Q96MI9	VAR_048604	p.Pro463Leu	LB/B	rs8029810	-
AGBL1	Q96MI9	VAR_048605	p.Ser481Pro	LB/B	rs11857527	-
AGBL1	Q96MI9	VAR_059195	p.Gln1056Arg	LB/B	rs8028043	-
AGBL1	Q96MI9	VAR_070225	p.Cys1036Ser	LP/P	rs181958589	Corneal dystrophy, Fuchs endothelial, 8 (FECD8) [MIM:615523]
AGBL2	Q5U5Z8	VAR_031572	p.Arg349His	LB/B	rs7941404	-
AGBL2	Q5U5Z8	VAR_046637	p.Ile90Arg	LB/B	rs12795414	-
AGBL2	Q5U5Z8	VAR_046638	p.Thr333Pro	LB/B	rs35898124	-
AGBL2	Q5U5Z8	VAR_046639	p.Asp368Gly	LB/B	rs1870545	-
AGBL2	Q5U5Z8	VAR_046640	p.Met671Ile	LB/B	rs12286721	-
AGBL3	Q8NEM8	VAR_031573	p.Phe45Tyr	LB/B	rs2348049	-
AGBL3	Q8NEM8	VAR_031574	p.Glu122Gln	LB/B	rs4236655	-
AGBL3	Q8NEM8	VAR_031575	p.Thr360Ile	LB/B	rs17804854	-
AGBL4	Q5VU57	VAR_061078	p.Val443Met	LB/B	rs60977321	-
AGBL5	Q8NDL9	VAR_035231	p.Gly649Asp	LB/B	rs35804461	-
AGBL5	Q8NDL9	VAR_077018	p.Arg276Trp	LP/P	rs879253769	Retinitis pigmentosa 75 (RP75) [MIM:617023]
AGBL5	Q8NDL9	VAR_077019	p.Asp295Asn	LP/P	rs879253768	Retinitis pigmentosa 75 (RP75) [MIM:617023]
AGBL5	Q8NDL9	VAR_079522	p.Pro108Arg	US	-	Retinitis pigmentosa 75 (RP75) [MIM:617023]
AGBL5	Q8NDL9	VAR_079523	p.Val251Gly	US	-	Retinitis pigmentosa 75 (RP75) [MIM:617023]
AGBL5	Q8NDL9	VAR_079524	p.Arg281Cys	US	rs780394281	Retinitis pigmentosa 75 (RP75) [MIM:617023]
AGER	Q15109	VAR_011338	p.Gln100Arg	LB/B	-	-
AGER	Q15109	VAR_024500	p.Gly82Ser	LB/B	rs2070600	-
AGFG2	O95081	VAR_050566	p.Thr365Asn	LB/B	rs34731997	-
AGGF1	Q8N302	VAR_017901	p.Glu133Lys	LP/P	rs34203073	Klippel-Trenaunay syndrome (KTS) [MIM:149000]
AGGF1	Q8N302	VAR_017902	p.Pro698Thr	LB/B	rs34400049	-
AGGF1	Q8N302	VAR_037446	p.Thr180Ala	LB/B	rs9715897	-
AGGF1	Q8N302	VAR_037447	p.Leu471Pro	LB/B	rs17856835	-
AGK	Q53H12	VAR_027848	p.Val3Met	LB/B	rs10262855	-
AGL	P35573	VAR_009230	p.Gly1115Arg	LB/B	rs2230307	-
AGL	P35573	VAR_009231	p.Gly1448Arg	LP/P	rs118203964	Glycogen storage disease 3 (GSD3) [MIM:232400]

AGL P35573 VAR_009621 p.Arg387Gln LB/B rs17121464 -
AGL P35573 VAR_009622 p.Glu1343Lys LB/B rs112795811 -
AGL P35573 VAR_020389 p.Pro1067Ser LB/B rs3753494 -
AGL P35573 VAR_028051 p.Gln229Arg LB/B rs17121403 -
AGL P35573 VAR_028052 p.Ala701Ser LB/B rs3736297 -
AGL P35573 VAR_028053 p.Ile1144Asn LB/B rs2230308 -
AGL P35573 VAR_028054 p.Arg1253His LB/B rs12043139 -
AGL P35573 VAR_028055 p.Arg1487Gly LB/B rs12118058 -
AGL P35573 VAR_032084 p.Thr38Ala LB/B rs35278779 -
AGL P35573 VAR_032085 p.Ser962Cys LB/B rs34714252 -
AGL P35573 VAR_051010 p.Ala1207Thr LB/B rs11807956 -
AGMAT Q9BSE5 VAR_023485 p.Gly105Arg LB/B rs6429757 -
AGMAT Q9BSE5 VAR_048332 p.Arg140Gln LB/B rs11580170 -
AGMO Q6ZNB7 VAR_062201 p.Phe279Leu LB/B rs58564185 -
AGMO Q6ZNB7 VAR_062202 p.Ser280Tyr LB/B rs59160822 -
AGO1 Q9UL18 VAR_078651 p.Gly199Ser LP/P - -
AGPAT1 Q99943 VAR_050593 p.Pro30Ser LB/B rs11964847 -
AGPAT2 O15120 VAR_017325 p.Ala239Val LP/P rs145975461 Congenital generalized lipodystrophy 1 (CGL1) [MIM:608594]
AGPAT2 O15120 VAR_017327 p.Leu228Pro LP/P rs104894100 Congenital generalized lipodystrophy 1 (CGL1) [MIM:608594]
AGPAT2 O15120 VAR_017328 p.Gly136Arg LP/P rs797045222 Congenital generalized lipodystrophy 1 (CGL1) [MIM:608594]
AGPAT5 Q9NUQ2 VAR_022696 p.Tyr77Cys LB/B rs17077958 -
AGPS O00116 VAR_005002 p.Arg419His LP/P rs121434411 Rhizomelic chondrodysplasia punctata 3 (RCDP3) [MIM:600121]
AGPS O00116 VAR_025895 p.Thr309Ile LP/P rs121434412 Rhizomelic chondrodysplasia punctata 3 (RCDP3) [MIM:600121]
AGPS O00116 VAR_025896 p.Leu469Pro LP/P rs121434413 Rhizomelic chondrodysplasia punctata 3 (RCDP3) [MIM:600121]
AGPS O00116 VAR_066929 p.Arg182Gln LP/P - Rhizomelic chondrodysplasia punctata 3 (RCDP3) [MIM:600121]
AGPS O00116 VAR_066930 p.Glu471Lys LP/P - Rhizomelic chondrodysplasia punctata 3 (RCDP3) [MIM:600121]
AGPS O00116 VAR_066931 p.Thr568Met LP/P rs387907214 Rhizomelic chondrodysplasia punctata 3 (RCDP3) [MIM:600121]
AGRN O00468 VAR_048966 p.Val1666Ile LB/B rs17160775 -
AGRN O00468 VAR_068724 p.Val23Leu LB/B - -
AGRN O00468 VAR_068725 p.Asp58Asn LB/B - -
AGRN O00468 VAR_068726 p.Asn105Ile LP/P - Myasthenic syndrome, congenital, 8 (CMS8) [MIM:615120]
AGRN O00468 VAR_068727 p.Thr267Met LB/B - -
AGRN O00468 VAR_068728 p.Ala375Ser LB/B rs138031468 -
AGRN O00468 VAR_068729 p.Glu728Val LB/B rs113288277 -
AGRN O00468 VAR_068730 p.Gln852Arg LB/B rs9697293 -
AGRN O00468 VAR_068731 p.Val984Met LB/B - -
AGRN O00468 VAR_068732 p.Leu1088Phe LB/B rs150132566 -
AGRN O00468 VAR_068733 p.Thr1118Lys LB/B rs149159118 -
AGRN O00468 VAR_068734 p.Gln1135Arg LB/B rs142416636 -

AGRN O00468 VAR_068735 p.Pro1240Leu LB/B rs142620337 -
AGRN O00468 VAR_068736 p.Gly1341Arg LB/B - -
AGRN O00468 VAR_068737 p.Pro1451Leu LB/B - -
AGRN O00468 VAR_068738 p.Ala1514Thr LB/B rs111818381 -
AGRN O00468 VAR_068739 p.Gln1565His LB/B rs199876002 -
AGRN O00468 VAR_068740 p.Arg1671Gln LB/B - -
AGRN O00468 VAR_068741 p.Arg1698Pro LB/B - -
AGRN O00468 VAR_068742 p.Gly1709Arg LP/P - Myasthenic syndrome, congenital, 8 (CMS8) [MIM:615120]
AGRN O00468 VAR_068743 p.Arg1734His LB/B rs145444272 -
AGRN O00468 VAR_068744 p.Asp1789Asn LB/B - -
AGRN O00468 VAR_068745 p.Gly2046Val LB/B - -
AGRN O00468 VAR_069066 p.Val1727Phe LP/P - Myasthenic syndrome, congenital, 8 (CMS8) [MIM:615120]
AGRN O00468 VAR_071367 p.Gly76Ser LP/P - Myasthenic syndrome, congenital, 8 (CMS8) [MIM:615120]
AGRN O00468 VAR_071368 p.Ala745Val LB/B - -
AGRN O00468 VAR_071369 p.Gly1875Arg LP/P - Myasthenic syndrome, congenital, 8 (CMS8) [MIM:615120]
AGRP O00253 VAR_015385 p.Ala67Thr LB/B rs5030980 -
AGT P01019 VAR_007093 p.Thr207Met LB/B rs4762 -
AGT P01019 VAR_007094 p.Thr242Ile LB/B rs765678426 -
AGT P01019 VAR_007095 p.Leu244Arg LB/B rs5041 -
AGT P01019 VAR_007096 p.Met268Thr LB/B rs699 -
AGT P01019 VAR_007097 p.Tyr281Cys LB/B rs56073403 -
AGT P01019 VAR_014573 p.Leu392Met LB/B rs1805090 -
AGT P01019 VAR_022933 p.Leu43Phe LB/B rs41271499 -
AGT P01019 VAR_029166 p.Glu98Lys LB/B rs11568032 -
AGT P01019 VAR_029167 p.Met268Ile LB/B rs11568053 -
AGT P01019 VAR_035431 p.Thr137Met LB/B rs34829218 -
AGT P01019 VAR_035432 p.Pro335Ser LB/B rs17856352 -
AGT P01019 VAR_035433 p.Arg375Gln LP/P rs74315283 Renal tubular dysgenesis (RTD) [MIM:267430]
AGT P01019 VAR_051939 p.Gly114Cys LB/B rs2229389 -
AGTPBP1 Q9UPW5 VAR_036884 p.Glu423Lys US - A colorectal cancer sample
AGTPBP1 Q9UPW5 VAR_081909 p.Tyr694Asp LP/P rs1564071824 Neurodegeneration, childhood-onset, with cerebellar atrophy (CONDCA) [MIM:618276]
AGTPBP1 Q9UPW5 VAR_081911 p.Arg799Cys US - Neurodegeneration, childhood-onset, with cerebellar atrophy (CONDCA) [MIM:618276]
AGTPBP1 Q9UPW5 VAR_081912 p.Thr851Met LP/P rs760300826 Neurodegeneration, childhood-onset, with cerebellar atrophy (CONDCA) [MIM:618276]
AGTPBP1 Q9UPW5 VAR_081914 p.Arg910Cys US - Neurodegeneration, childhood-onset, with cerebellar atrophy (CONDCA) [MIM:618276]
AGTPBP1 Q9UPW5 VAR_081915 p.Arg918Trp LP/P rs1564035967 Neurodegeneration, childhood-onset, with cerebellar atrophy (CONDCA) [MIM:618276]
AGTPBP1 Q9UPW5 VAR_081916 p.His990Leu LP/P rs1564034077 Neurodegeneration, childhood-onset, with cerebellar atrophy (CONDCA) [MIM:618276]
AGTR1 P30556 VAR_011847 p.Cys289Trp LB/B rs1064533 -
AGTR1 P30556 VAR_011848 p.Thr336Pro LB/B rs1801021 -
AGTR1 P30556 VAR_029206 p.Ala163Thr LB/B rs12721226 -

AGTR1	P30556	VAR_029207	p.Ala244Ser	LB/B	rs12721225	-
AGTR1	P30556	VAR_035086	p.Thr282Met	LP/P	rs104893677	Renal tubular dysgenesis (RTD) [MIM:267430]
AGTR1	P30556	VAR_070375	p.Leu222Val	LB/B	rs17852013	-
AGTR1	P30556	VAR_070376	p.Pro341His	LB/B	rs17852012	-
AGTR2	P50052	VAR_011849	p.Arg248Lys	LB/B	rs5191	-
AGTR2	P50052	VAR_011850	p.Cys268Trp	LB/B	rs1042860	-
AGTR2	P50052	VAR_049374	p.Tyr231His	LB/B	rs3729977	-
AGTR2	P50052	VAR_065946	p.Gly21Val	US	rs121917810	-
AGTR2	P50052	VAR_065947	p.Arg324Gln	US	rs35474657	-
AGTR2	P50052	VAR_065948	p.Ile337Val	US	rs121917811	-
AGTRAP	Q6RW13	VAR_023075	p.Ala143Val	LB/B	rs17875960	-
AGXT	P21549	VAR_000587	p.Pro11Leu	LB/B	rs34116584	-
AGXT	P21549	VAR_000588	p.Gly41Arg	LP/P	rs121908523	Hyperoxaluria primary 1 (HP1) [MIM:259900]
AGXT	P21549	VAR_000589	p.Phe152Ile	LP/P	rs121908524	Hyperoxaluria primary 1 (HP1) [MIM:259900]
AGXT	P21549	VAR_000590	p.Gly170Arg	LP/P	rs121908529	Hyperoxaluria primary 1 (HP1) [MIM:259900]
AGXT	P21549	VAR_000591	p.Ser187Phe	LP/P	rs180177238	Hyperoxaluria primary 1 (HP1) [MIM:259900]
AGXT	P21549	VAR_000592	p.Ser205Pro	LP/P	rs121908520	Hyperoxaluria primary 1 (HP1) [MIM:259900]
AGXT	P21549	VAR_000593	p.Ile340Met	LB/B	rs4426527	-
AGXT	P21549	VAR_008878	p.Gly82Glu	LP/P	rs121908522	Hyperoxaluria primary 1 (HP1) [MIM:259900]
AGXT	P21549	VAR_008879	p.Arg233Cys	LP/P	rs121908526	Hyperoxaluria primary 1 (HP1) [MIM:259900]
AGXT	P21549	VAR_008880	p.Arg233His	LP/P	rs121908527	Hyperoxaluria primary 1 (HP1) [MIM:259900]
AGXT	P21549	VAR_008881	p.Ile244Thr	LP/P	rs121908525	Hyperoxaluria primary 1 (HP1) [MIM:259900]
AGXT	P21549	VAR_010969	p.Gly41Val	LP/P	rs180177168	Hyperoxaluria primary 1 (HP1) [MIM:259900]
AGXT	P21549	VAR_010971	p.Gly116Arg	LP/P	rs180177207	Hyperoxaluria primary 1 (HP1) [MIM:259900]
AGXT	P21549	VAR_010972	p.Gly156Arg	LP/P	rs121908530	Hyperoxaluria primary 1 (HP1) [MIM:259900]
AGXT	P21549	VAR_010973	p.Asp183Asn	LP/P	rs180177236	Hyperoxaluria primary 1 (HP1) [MIM:259900]
AGXT	P21549	VAR_048236	p.Asn22Ser	LB/B	rs34885252	-
AGXT	P21549	VAR_048237	p.Ala295Thr	LB/B	rs13408961	-
AGXT	P21549	VAR_060547	p.Thr9Asn	LB/B	rs115014558	-
AGXT	P21549	VAR_060548	p.Gly82Arg	LP/P	rs180177185	Hyperoxaluria primary 1 (HP1) [MIM:259900]
AGXT	P21549	VAR_060549	p.Trp108Arg	LP/P	rs180177197	Hyperoxaluria primary 1 (HP1) [MIM:259900]
AGXT	P21549	VAR_060550	p.Ala112Asp	LP/P	rs796052061	Hyperoxaluria primary 1 (HP1) [MIM:259900]
AGXT	P21549	VAR_060552	p.Leu153Val	LP/P	rs180177223	Hyperoxaluria primary 1 (HP1) [MIM:259900]
AGXT	P21549	VAR_060553	p.Ser158Leu	LP/P	rs180177225	Hyperoxaluria primary 1 (HP1) [MIM:259900]
AGXT	P21549	VAR_060554	p.Gly161Arg	LP/P	rs180177227	Hyperoxaluria primary 1 (HP1) [MIM:259900]
AGXT	P21549	VAR_060555	p.Cys173Tyr	LP/P	rs180177231	Hyperoxaluria primary 1 (HP1) [MIM:259900]
AGXT	P21549	VAR_060556	p.Gly190Arg	LP/P	rs180177239	Hyperoxaluria primary 1 (HP1) [MIM:259900]
AGXT	P21549	VAR_060557	p.Met195Arg	LP/P	rs180177244	Hyperoxaluria primary 1 (HP1) [MIM:259900]
AGXT	P21549	VAR_060558	p.Asp201Glu	LP/P	rs180177246	Hyperoxaluria primary 1 (HP1) [MIM:259900]
AGXT	P21549	VAR_060559	p.Ser218Leu	LP/P	rs180177253	Hyperoxaluria primary 1 (HP1) [MIM:259900]
AGXT	P21549	VAR_060560	p.Arg233Leu	LP/P	rs121908527	Hyperoxaluria primary 1 (HP1) [MIM:259900]
AGXT	P21549	VAR_060561	p.Asp243His	LP/P	rs180177258	Hyperoxaluria primary 1 (HP1) [MIM:259900]
AGXT	P21549	VAR_060562	p.Cys253Arg	LP/P	rs180177264	Hyperoxaluria primary 1 (HP1) [MIM:259900]
AGXT	P21549	VAR_060563	p.Ile279Met	LP/P	rs180177277	Hyperoxaluria primary 1 (HP1) [MIM:259900]
AGXT	P21549	VAR_060564	p.Ile279Thr	LB/B	rs140992177	-
AGXT	P21549	VAR_060565	p.Ala280Val	LB/B	rs73106685	-
AGXT	P21549	VAR_060566	p.Ser287Thr	LP/P	rs180177289	Hyperoxaluria primary 1 (HP1) [MIM:259900]
AGXT	P21549	VAR_060567	p.Arg289Cys	LP/P	rs180177290	Hyperoxaluria primary 1 (HP1) [MIM:259900]
AGXT	P21549	VAR_060569	p.Leu298Pro	LP/P	rs180177293	Hyperoxaluria primary 1 (HP1) [MIM:259900]

AGXT P21549 VAR_060570 p.Val326Ile LB/B rs115057148 -
AGXT P21549 VAR_060571 p.Val336Asp LP/P rs180177155 Hyperoxaluria primary 1 (HP1) [MIM:259900]
AGXT P21549 VAR_060572 p.Gly350Asp LP/P rs180177156 Hyperoxaluria primary 1 (HP1) [MIM:259900]
AGXT P21549 VAR_074582 p.Arg36Cys LP/P rs180177157 Hyperoxaluria primary 1 (HP1) [MIM:259900]
AGXT P21549 VAR_074583 p.Gly41Glu LP/P rs180177168 Hyperoxaluria primary 1 (HP1) [MIM:259900]
AGXT P21549 VAR_074584 p.Gly47Arg LP/P rs180177173 Hyperoxaluria primary 1 (HP1) [MIM:259900]
AGXT P21549 VAR_074585 p.Leu150Pro LP/P rs180177222 Hyperoxaluria primary 1 (HP1) [MIM:259900]
AGXT P21549 VAR_074586 p.Gly161Cys LP/P rs180177227 Hyperoxaluria primary 1 (HP1) [MIM:259900]
AGXT P21549 VAR_074587 p.Gly161Ser LP/P rs180177227 Hyperoxaluria primary 1 (HP1) [MIM:259900]
AGXT P21549 VAR_074588 p.Leu166Pro LP/P rs180177230 Hyperoxaluria primary 1 (HP1) [MIM:259900]
AGXT P21549 VAR_074589 p.Ile202Asn US rs536352238 Hyperoxaluria primary 1 (HP1) [MIM:259900]
AGXT2 Q9BYV1 VAR_022140 p.Val140Ile LB/B rs37369 -
AGXT2 Q9BYV1 VAR_022141 p.Thr212Ile LB/B rs180749 -
AGXT2 Q9BYV1 VAR_023483 p.Ser102Asn LB/B rs37370 -
AGXT2 Q9BYV1 VAR_029513 p.Val498Leu LB/B rs16899974 -
AGXT2 Q9BYV1 VAR_048231 p.Gly132Arg LB/B rs16870794 -
AGXT2 Q9BYV1 VAR_048232 p.Pro492Arg LB/B rs17245714 -
AGXT2 Q9BYV1 VAR_061006 p.Ser102Ile LB/B rs37370 -
AGXT2 Q9BYV1 VAR_061007 p.Ser102Thr LB/B rs37370 -
AHCTF1 Q8WYP5 VAR_027037 p.Asn874Ser LB/B rs2642990 -
AHCTF1 Q8WYP5 VAR_027038 p.Leu2185Val LB/B rs12410563 -
AHCY P23526 VAR_006934 p.Asp86Asn LB/B - -
AHCY P23526 VAR_052286 p.Arg38Trp LB/B rs13043752 -
AHCY P23526 VAR_058588 p.Arg49Cys LP/P rs369428934 Hypermethioninemia with S-adenosylhomocysteine hydrolase deficiency (HMAHCHD) [MIM:613752]
AHCY P23526 VAR_058589 p.Asp86Gly LP/P rs773162208 Hypermethioninemia with S-adenosylhomocysteine hydrolase deficiency (HMAHCHD) [MIM:613752]
AHCY P23526 VAR_058590 p.Ala89Val LP/P rs755222515 Hypermethioninemia with S-adenosylhomocysteine hydrolase deficiency (HMAHCHD) [MIM:613752]
AHCY P23526 VAR_058591 p.Tyr143Cys LP/P rs121918608 Hypermethioninemia with S-adenosylhomocysteine hydrolase deficiency (HMAHCHD) [MIM:613752]
AHDC1 Q5TGY3 VAR_037765 p.Ala935Thr LB/B rs4908364 -
AHI1 Q8N157 VAR_023391 p.Val443Asp LP/P rs121434350 Joubert syndrome 3 (JBTS3) [MIM:608629]
AHI1 Q8N157 VAR_037892 p.Ile49Asn LB/B - -
AHI1 Q8N157 VAR_037893 p.Arg548His LB/B rs35433555 -
AHI1 Q8N157 VAR_037894 p.Arg723Gln LP/P rs121434351 Joubert syndrome 3 (JBTS3) [MIM:608629]
AHI1 Q8N157 VAR_037895 p.Ser761Leu LB/B rs794727174 -
AHI1 Q8N157 VAR_037896 p.Arg830Trp LB/B rs13312995 -
AHI1 Q8N157 VAR_037897 p.Thr856Ser LB/B rs199736888 -
AHI1 Q8N157 VAR_037898 p.Tyr933Cys LB/B rs41288013 -
AHI1 Q8N157 VAR_037899 p.Gln1018Pro LB/B rs6940875 -
AHI1 Q8N157 VAR_037900 p.Ser1123Phe LB/B rs117447608 -
AHI1 Q8N157 VAR_037901 p.Pro1140Ser LB/B rs201148693 -
AHI1 Q8N157 VAR_068171 p.Glu1086Gly LB/B rs148000791 -
AHI1 Q8N157 VAR_071194 p.Arg351Leu LP/P rs397514726 Joubert syndrome 3 (JBTS3) [MIM:608629]
AHI1 Q8N157 VAR_076820 p.Thr671Ile LP/P rs772989270 Joubert syndrome 3 (JBTS3) [MIM:608629]
AHI1 Q8N157 VAR_076821 p.Asp719Gly LP/P rs863225134 Joubert syndrome 3 (JBTS3) [MIM:608629]
AHI1 Q8N157 VAR_076822 p.His896Arg LP/P rs863225135 Joubert syndrome 3 (JBTS3) [MIM:608629]
AHNAK Q09666 VAR_039058 p.Gly962Val LB/B rs664761 -

AHNAK	Q09666	VAR_039059	p.Ala2114Thr	LB/B	rs1298288	-
AHNAK	Q09666	VAR_039060	p.Pro2439Leu	LB/B	rs11824660	-
AHNAK	Q09666	VAR_039061	p.Gln3003Lys	LB/B	rs566144	-
AHNAK	Q09666	VAR_039062	p.Val3190Ile	LB/B	rs11231129	-
AHNAK	Q09666	VAR_039063	p.Ser3724Pro	LB/B	rs11231128	-
AHNAK	Q09666	VAR_039064	p.Gly4561Asp	LB/B	rs12795508	-
AHNAK	Q09666	VAR_039065	p.Met4611Val	LB/B	rs12801302	-
AHNAK	Q09666	VAR_039066	p.Ile4613Val	LB/B	rs12801153	-
AHNAK	Q09666	VAR_039067	p.Asp4631Gly	LB/B	rs12801123	-
AHNAK	Q09666	VAR_039068	p.Thr5415Ala	LB/B	rs11231126	-
AHNAK	Q09666	VAR_061551	p.Lys2247Thr	LB/B	rs61524789	-
AHNAK	Q09666	VAR_061552	p.Asp4304Gly	LB/B	rs11828907	-
AHNAK2	Q8IVF2	VAR_039069	p.Val1266Met	LB/B	rs748458962	-
AHNAK2	Q8IVF2	VAR_039070	p.Leu1470Val	LB/B	-	-
AHNAK2	Q8IVF2	VAR_039071	p.Ser2115Arg	LB/B	rs2582514	-
AHNAK2	Q8IVF2	VAR_039072	p.Asp2429Glu	LB/B	rs11160826	-
AHNAK2	Q8IVF2	VAR_039073	p.Leu2430Val	LB/B	rs2819426	-
AHNAK2	Q8IVF2	VAR_039074	p.Val2616Ala	LB/B	rs4264326	-
AHNAK2	Q8IVF2	VAR_039075	p.Leu5028Met	LB/B	rs9672139	-
AHNAK2	Q8IVF2	VAR_039076	p.Gly5564Arg	LB/B	rs2819420	-
AHNAK2	Q8IVF2	VAR_050635	p.Thr525Ala	LB/B	rs2278607	-
AHNAK2	Q8IVF2	VAR_050636	p.Met2107Val	LB/B	rs11846918	-
AHNAK2	Q8IVF2	VAR_050637	p.Lys2410Arg	LB/B	rs11845746	-
AHNAK2	Q8IVF2	VAR_050638	p.Val3796Leu	LB/B	-	-
AHNAK2	Q8IVF2	VAR_050639	p.Met3961Val	LB/B	rs10141053	-
AHNAK2	Q8IVF2	VAR_050640	p.Ile4071Met	LB/B	rs2582511	-
AHNAK2	Q8IVF2	VAR_050641	p.Phe4138Leu	LB/B	rs2582505	-
AHNAK2	Q8IVF2	VAR_050642	p.Lys4232Asn	LB/B	rs2819423	-
AHNAK2	Q8IVF2	VAR_050643	p.Val4278Ala	LB/B	rs2819422	-
AHNAK2	Q8IVF2	VAR_050644	p.Met4536Leu	LB/B	rs9672139	-
AHNAK2	Q8IVF2	VAR_050645	p.Thr4664Ala	LB/B	rs4465542	-
AHNAK2	Q8IVF2	VAR_050646	p.Gly5072Arg	LB/B	rs2819420	-
AHNAK2	Q8IVF2	VAR_050647	p.Tyr5184Asp	LB/B	rs2819419	-
AHNAK2	Q8IVF2	VAR_050648	p.Pro5397Ala	LB/B	rs3742935	-
AHNAK2	Q8IVF2	VAR_050649	p.Thr5732Met	LB/B	rs748358	-
AHNAK2	Q8IVF2	VAR_059560	p.Glu1856Asp	LB/B	rs2819435	-
AHNAK2	Q8IVF2	VAR_059561	p.Leu2146Val	LB/B	rs12890949	-
AHNAK2	Q8IVF2	VAR_059562	p.Glu2503Ala	LB/B	rs2819429	-
AHNAK2	Q8IVF2	VAR_059563	p.Arg2862Ser	LB/B	rs2582514	-
AHNAK2	Q8IVF2	VAR_059564	p.Asp3176Glu	LB/B	rs11160826	-
AHNAK2	Q8IVF2	VAR_059565	p.Leu3177Val	LB/B	rs2819426	-
AHNAK2	Q8IVF2	VAR_059566	p.Pro3336Leu	LB/B	rs10438247	-
AHNAK2	Q8IVF2	VAR_059567	p.Val3363Ala	LB/B	rs4264326	-
AHNAK2	Q8IVF2	VAR_059568	p.Asp3793Asn	LB/B	rs11160825	-
AHNAK2	Q8IVF2	VAR_059569	p.Met3869Val	LB/B	rs10438246	-
AHNAK2	Q8IVF2	VAR_059570	p.Lys3902Asn	LB/B	rs2819423	-
AHNAK2	Q8IVF2	VAR_059571	p.Ala4085Val	LB/B	rs372676751	-
AHNAK2	Q8IVF2	VAR_059572	p.Asp4198Asn	LB/B	rs534942818	-
AHNAK2	Q8IVF2	VAR_059573	p.Leu4326Pro	LB/B	rs2819421	-

AHNAK2 Q8IVF2 VAR_059574 p.Pro4478Leu LB/B rs763391124 -
AHNAK2 Q8IVF2 VAR_061548 p.Met1298Ile LB/B rs2819440 -
AHNAK2 Q8IVF2 VAR_061549 p.Gly3654Glu LB/B rs28380382 -
AHNAK2 Q8IVF2 VAR_061550 p.Gly5139Glu LB/B rs61421370 -
AHR P35869 VAR_009281 p.Arg554Lys LB/B rs2066853 -
AHR P35869 VAR_009282 p.Val570Ile LB/B rs4986826 -
AHR P35869 VAR_015516 p.Pro517Ser LB/B rs72552768 -
AHR P35869 VAR_015517 p.Met786Val LB/B rs72552769 -
AHRR A9YTQ3 VAR_043308 p.Leu114Pro LB/B rs35008248 -
AHRR A9YTQ3 VAR_043309 p.Pro189Ala LB/B rs2292596 -
AHRR A9YTQ3 VAR_043310 p.Gly373Val LB/B rs2303738 -
AHRR A9YTQ3 VAR_043311 p.Asp627His LB/B rs34453673 -
AHSA2P Q719I0 VAR_038256 p.Met248Thr US - A breast cancer sample
AHSG P02765 VAR_002388 p.Met248Thr LB/B rs4917 -
AHSG P02765 VAR_002389 p.Ser256Thr LB/B rs4918 -
AHSG P02765 VAR_012474 p.Asp276Asn LB/B rs70961709 -
AHSG P02765 VAR_012475 p.Arg317Cys LB/B rs35457250 -
AHSG P02765 VAR_055802 p.Val142Leu LB/B rs7633550 -
AHSG P02765 VAR_080645 p.Arg317His US rs201849460 Alopecia-mental retardation syndrome 1 (APMR1) [MIM:203650]
AHSP Q9NZD4 VAR_050650 p.Pro100Thr LB/B rs36018996 -
AICDA Q9GZX7 VAR_013774 p.Arg24Trp LP/P rs104894324 Immunodeficiency with hyper-IgM 2 (HIGM2) [MIM:605258]
AICDA Q9GZX7 VAR_013775 p.Trp80Arg LP/P rs104894320 Immunodeficiency with hyper-IgM 2 (HIGM2) [MIM:605258]
AICDA Q9GZX7 VAR_013776 p.Leu106Pro LP/P rs104894321 Immunodeficiency with hyper-IgM 2 (HIGM2) [MIM:605258]
AICDA Q9GZX7 VAR_013777 p.Met139Val LP/P rs104894322 Immunodeficiency with hyper-IgM 2 (HIGM2) [MIM:605258]
AICDA Q9GZX7 VAR_013778 p.Phe151Ser LP/P rs104894327 Immunodeficiency with hyper-IgM 2 (HIGM2) [MIM:605258]
AICDA Q9GZX7 VAR_014091 p.Arg25Cys LB/B rs1404944797 -
AICDA Q9GZX7 VAR_077563 p.Phe15Leu LP/P - Immunodeficiency with hyper-IgM 2 (HIGM2) [MIM:605258]
AICDA Q9GZX7 VAR_077564 p.Tyr31His LP/P - Immunodeficiency with hyper-IgM 2 (HIGM2) [MIM:605258]
AICDA Q9GZX7 VAR_077565 p.His56Tyr US - Immunodeficiency with hyper-IgM 2 (HIGM2) [MIM:605258]
AICDA Q9GZX7 VAR_077566 p.Cys87Arg US rs762590894 Immunodeficiency with hyper-IgM 2 (HIGM2) [MIM:605258]
AICDA Q9GZX7 VAR_077567 p.His130Pro LP/P - Immunodeficiency with hyper-IgM 2 (HIGM2) [MIM:605258]
AICDA Q9GZX7 VAR_077568 p.Arg174Ser US - Immunodeficiency with hyper-IgM 2 (HIGM2) [MIM:605258]
AIF1 P55008 VAR_048665 p.Gly14Arg LB/B rs2736182 -
AIFM1 O95831 VAR_067334 p.Gly308Glu LP/P rs1603224226 Combined oxidative phosphorylation deficiency 6 (COXPD6) [MIM:300816]
AIFM1 O95831 VAR_069468 p.Glu493Val LP/P rs281864468 Charcot-Marie-Tooth disease, X-linked recessive, 4, with or without cerebellar ataxia (CMTX4) [MIM:310490]

AIFM1 O95831 VAR_072791 p.Val243Leu LP/P rs1603225138 Combined oxidative phosphorylation deficiency 6 (COXPD6) [MIM:300816]
AIFM1 O95831 VAR_076211 p.Thr260Ala LP/P rs863225432 Deafness, X-linked, 5, with peripheral neuropathy (DFNX5) [MIM:300614]
AIFM1 O95831 VAR_076212 p.Leu344Phe US rs184474885 Deafness, X-linked, 5, with peripheral neuropathy (DFNX5) [MIM:300614]
AIFM1 O95831 VAR_076213 p.Gly360Arg US rs724160026 Deafness, X-linked, 5, with peripheral neuropathy (DFNX5) [MIM:300614]
AIFM1 O95831 VAR_076214 p.Arg422Gln LP/P rs724160021 Deafness, X-linked, 5, with peripheral neuropathy (DFNX5) [MIM:300614]
AIFM1 O95831 VAR_076215 p.Arg422Trp LP/P rs724160020 Deafness, X-linked, 5, with peripheral neuropathy (DFNX5) [MIM:300614]
AIFM1 O95831 VAR_076216 p.Arg430Cys US rs1223488720 Deafness, X-linked, 5, with peripheral neuropathy (DFNX5) [MIM:300614]
AIFM1 O95831 VAR_076217 p.Arg451Gln LP/P rs863225431 Deafness, X-linked, 5, with peripheral neuropathy (DFNX5) [MIM:300614]
AIFM1 O95831 VAR_076218 p.Ala472Val US - Deafness, X-linked, 5, with peripheral neuropathy (DFNX5) [MIM:300614]
AIFM1 O95831 VAR_076219 p.Pro475Leu US rs724160022 Deafness, X-linked, 5, with peripheral neuropathy (DFNX5) [MIM:300614]
AIFM1 O95831 VAR_076220 p.Val498Met US rs724160023 Deafness, X-linked, 5, with peripheral neuropathy (DFNX5) [MIM:300614]
AIFM1 O95831 VAR_076221 p.Ile591Met US - Deafness, X-linked, 5, with peripheral neuropathy (DFNX5) [MIM:300614]
AIFM1 O95831 VAR_083067 p.Gly262Ser LP/P rs1603224817 -
AIFM1 O95831 VAR_083068 p.Gly338Glu LP/P rs1603223152 Combined oxidative phosphorylation deficiency 6 (COXPD6) [MIM:300816]
AIFM1 O95831 VAR_083739 p.Gln235His LP/P rs377527583 Spondyloepimetaphyseal dysplasia, X-linked, with hypomyelinating leukodystrophy (SEMDHL) [MIM:300232]
AIFM1 O95831 VAR_083740 p.Asp237Gly LP/P rs1202786652 Spondyloepimetaphyseal dysplasia, X-linked, with hypomyelinating leukodystrophy (SEMDHL) [MIM:300232]
AIFM1 O95831 VAR_083741 p.Asp237Val LP/P rs1202786652 Spondyloepimetaphyseal dysplasia, X-linked, with hypomyelinating leukodystrophy (SEMDHL) [MIM:300232]
AIFM2 Q9BRQ8 VAR_050651 p.Met135Thr LB/B rs10999147 -
AIFM2 Q9BRQ8 VAR_050652 p.Asp288Asn LB/B rs2271694 -
AIFM3 Q96NN9 VAR_061553 p.Ser508Thr LB/B rs61356271 -
AIG1 Q9NVV5 VAR_057502 p.Gln151Glu LB/B rs1053193 -
AIM2 O14862 VAR_022022 p.Glu32Lys LB/B rs2276405 -
AIM2 O14862 VAR_043379 p.Cys304Tyr LB/B rs778047649 -
AIMP1 Q12904 VAR_025212 p.Pro79Ala LB/B rs1134648 -
AIMP1 Q12904 VAR_029156 p.Thr104Ala LB/B rs113844295 -
AIMP1 Q12904 VAR_050124 p.Thr117Ala LB/B rs2230255 -
AIMP2 Q13155 VAR_025521 p.Ala129Gly LB/B rs17855441 -
AIMP2 Q13155 VAR_050125 p.Leu166Ile LB/B rs34525431 -
AIMP2 Q13155 VAR_058392 p.Ile92Val US - A lung cancer cell line
AIMP2 Q13155 VAR_058394 p.Gly209Ser US rs982080297 A lung cancer cell line
AIP O00170 VAR_043908 p.Arg16His LB/B rs145047094 -
AIP O00170 VAR_043909 p.Gln228Lys LB/B rs641081 -

AIP O00170 VAR_043910 p.Lys241Glu US rs267606573 Pituitary adenoma 1, multiple types (PITA1) [MIM:102200]
AIP O00170 VAR_043912 p.Arg271Trp US rs267606579 Pituitary adenoma 1, multiple types (PITA1) [MIM:102200]
AIP O00170 VAR_043913 p.Arg304Gln LP/P rs104894190 Pituitary adenoma 1, multiple types (PITA1) [MIM:102200]
AIP O00170 VAR_061545 p.Gln307Arg LB/B rs4930199 -
AIPL1 Q9NZN9 VAR_010139 p.Cys239Arg LP/P rs62637012 Leber congenital amaurosis 4 (LCA4) [MIM:604393]
AIPL1 Q9NZN9 VAR_010140 p.Asp90His LB/B rs12449580 -
AIPL1 Q9NZN9 VAR_050626 p.Val33Ala LB/B rs16955859 -
AIPL1 Q9NZN9 VAR_050627 p.Tyr134Phe LB/B rs16955851 -
AIPL1 Q9NZN9 VAR_067165 p.Arg270His LP/P - Leber congenital amaurosis 4 (LCA4) [MIM:604393]
AIPL1 Q9NZN9 VAR_067166 p.Arg302Leu LB/B rs62637015 -
AIRE O43918 VAR_005004 p.Leu28Pro LP/P rs179363878 Autoimmune polyendocrine syndrome 1, with or without reversible metaphyseal dysplasia (APS1) [MIM:240300]
AIRE O43918 VAR_005005 p.Lys83Glu LP/P rs121434255 Autoimmune polyendocrine syndrome 1, with or without reversible metaphyseal dysplasia (APS1) [MIM:240300]
AIRE O43918 VAR_005006 p.Ser278Arg LB/B rs1800520 -
AIRE O43918 VAR_013713 p.Arg15Leu LP/P rs179363876 Autoimmune polyendocrine syndrome 1, with or without reversible metaphyseal dysplasia (APS1) [MIM:240300]
AIRE O43918 VAR_013714 p.Thr16Met LP/P rs179363877 Autoimmune polyendocrine syndrome 1, with or without reversible metaphyseal dysplasia (APS1) [MIM:240300]
AIRE O43918 VAR_013715 p.Leu29Pro LP/P rs179363879 Autoimmune polyendocrine syndrome 1, with or without reversible metaphyseal dysplasia (APS1) [MIM:240300]
AIRE O43918 VAR_013716 p.Trp78Arg LP/P rs179363880 Autoimmune polyendocrine syndrome 1, with or without reversible metaphyseal dysplasia (APS1) [MIM:240300]
AIRE O43918 VAR_013717 p.Val80Leu LP/P rs179363881 Autoimmune polyendocrine syndrome 1, with or without reversible metaphyseal dysplasia (APS1) [MIM:240300]
AIRE O43918 VAR_013718 p.Tyr85Cys LP/P rs179363882 Autoimmune polyendocrine syndrome 1, with or without reversible metaphyseal dysplasia (APS1) [MIM:240300]
AIRE O43918 VAR_013719 p.Tyr90Cys LP/P rs179363883 Autoimmune polyendocrine syndrome 1, with or without reversible metaphyseal dysplasia (APS1) [MIM:240300]
AIRE O43918 VAR_013720 p.Leu93Arg LP/P rs179363884 Autoimmune polyendocrine syndrome 1, with or without reversible metaphyseal dysplasia (APS1) [MIM:240300]
AIRE O43918 VAR_013721 p.Val301Met LP/P rs150634562 Autoimmune polyendocrine syndrome 1, with or without reversible metaphyseal dysplasia (APS1) [MIM:240300]
AIRE O43918 VAR_013722 p.Gly305Ser US - -
AIRE O43918 VAR_013723 p.Cys311Tyr LP/P rs386833674 Autoimmune polyendocrine syndrome 1, with or without reversible metaphyseal dysplasia (APS1) [MIM:240300]
AIRE O43918 VAR_013724 p.Pro326Gln LP/P rs179363885 Autoimmune polyendocrine syndrome 1, with or without reversible metaphyseal dysplasia (APS1) [MIM:240300]
AIRE O43918 VAR_014422 p.Gly228Trp LP/P rs121434257 Autoimmune polyendocrine syndrome 1, with or without reversible metaphyseal dysplasia (APS1) [MIM:240300]
AIRE O43918 VAR_026480 p.Arg15Cys LP/P rs179363875 Autoimmune polyendocrine syndrome 1, with or without reversible metaphyseal dysplasia (APS1) [MIM:240300]
AIRE O43918 VAR_026481 p.Ala21Val LP/P rs179363886 Autoimmune polyendocrine syndrome 1, with or without reversible metaphyseal dysplasia (APS1) [MIM:240300]

AIRE O43918 VAR_026483 p.Phe77Ser LP/P rs179363887 Autoimmune polyendocrine syndrome 1, with or without reversible metaphyseal dysplasia (APS1) [MIM:240300]
AIRE O43918 VAR_026484 p.Pro252Leu LB/B rs34397615 Autoimmune polyendocrine syndrome 1, with or without reversible metaphyseal dysplasia (APS1) [MIM:240300]
AIRE O43918 VAR_026485 p.Pro326Leu LP/P rs179363885 Autoimmune polyendocrine syndrome 1, with or without reversible metaphyseal dysplasia (APS1) [MIM:240300]
AIRE O43918 VAR_026486 p.Pro539Leu LP/P rs179363889 Autoimmune polyendocrine syndrome 1, with or without reversible metaphyseal dysplasia (APS1) [MIM:240300]
AIRE O43918 VAR_076940 p.Glu298Lys LB/B rs763636007 -
AIRE O43918 VAR_076941 p.Cys299Trp LB/B rs751066946 -
AIRE O43918 VAR_076942 p.Cys302Tyr US - -
AIRE O43918 VAR_076943 p.Arg303Gln LB/B rs139808903 -
AIRE O43918 VAR_076944 p.Arg303Trp LB/B rs778929451 -
AIRE O43918 VAR_076945 p.Gly306Arg LB/B rs754932526 -
AIRE O43918 VAR_076946 p.Ile309Met LB/B rs74162062 -
AIRE O43918 VAR_076947 p.Arg316Gln LB/B rs202027254 -
AIRE O43918 VAR_076948 p.Arg316Trp US rs139874934 -
AIRE O43918 VAR_076949 p.His319Pro LB/B rs776951380 -
AIRE O43918 VAR_076950 p.Arg328Gln LB/B rs775921321 -
AIRE O43918 VAR_076951 p.Arg328Trp LB/B rs74162063 -
AIRE O43918 VAR_076952 p.Ser332Arg LB/B rs766901260 -
AIRE O43918 VAR_076953 p.Val484Ala US rs769470638 -
AJAP1 Q9UKB5 VAR_031821 p.Gly263Arg LB/B rs242056 -
AK1 P00568 VAR_004021 p.Arg128Trp LP/P rs104894101 Hemolytic anemia due to adenylate kinase deficiency (HAAKD) [MIM:612631]
AK1 P00568 VAR_034046 p.Glu123Gln LB/B rs8192462 -
AK1 P00568 VAR_055337 p.Gly40Arg LP/P rs137853204 Hemolytic anemia due to adenylate kinase deficiency (HAAKD) [MIM:612631]
AK1 P00568 VAR_055338 p.Gly64Arg LP/P rs137853205 Hemolytic anemia due to adenylate kinase deficiency (HAAKD) [MIM:612631]
AK1 P00568 VAR_055340 p.Tyr164Cys LP/P rs137853203 Hemolytic anemia due to adenylate kinase deficiency (HAAKD) [MIM:612631]
AK2 P54819 VAR_050032 p.Ala209Thr LB/B rs12116440 -
AK2 P54819 VAR_054630 p.Arg103Trp LP/P rs267606648 Reticular dysgenesis (RDYS) [MIM:267500]
AK2 P54819 VAR_054631 p.Asp165Gly LP/P rs267606643 Reticular dysgenesis (RDYS) [MIM:267500]
AK5 Q9Y6K8 VAR_059435 p.Arg465Gln LB/B rs2803140 -
AK7 Q96M32 VAR_017059 p.Arg102Gln LB/B rs2275554 -
AK7 Q96M32 VAR_057950 p.Asn389Lys LB/B rs2369679 -
AK7 Q96M32 VAR_080917 p.Leu673Pro LP/P rs116298211 Spermatogenic failure 27 (SPGF27) [MIM:617965]
AK8 Q96MA6 VAR_030873 p.Ile5Thr LB/B rs2231400 -
AK8 Q96MA6 VAR_030874 p.Asp130Gly LB/B rs17407084 -
AKAP1 Q92667 VAR_024512 p.Val60Met LB/B rs2230770 -
AKAP1 Q92667 VAR_049676 p.Ala18Val LB/B rs17761023 -
AKAP1 Q92667 VAR_049677 p.Cys102Tyr LB/B rs2230771 -
AKAP1 Q92667 VAR_049678 p.Arg124Cys LB/B rs17833723 -
AKAP10 O43572 VAR_024607 p.Arg249His LB/B rs2108978 -
AKAP10 O43572 VAR_024608 p.Ile646Val LB/B rs203462 -
AKAP11 Q9UKA4 VAR_020131 p.Ser721Cys LB/B rs2236364 -
AKAP11 Q9UKA4 VAR_048207 p.His1070Arg LB/B rs17063163 -

AKAP11	Q9UKA4	VAR_048208	p.Leu1410Phe	LB/B	rs17063167	-
AKAP12	Q02952	VAR_035115	p.Lys117Glu	LB/B	rs10872670	-
AKAP12	Q02952	VAR_035116	p.Lys216Gln	LB/B	rs3734799	-
AKAP12	Q02952	VAR_035117	p.Glu920Gly	LB/B	rs13212161	-
AKAP12	Q02952	VAR_035118	p.Val1096Ile	LB/B	rs3734797	-
AKAP12	Q02952	VAR_035119	p.Arg1296Leu	LB/B	rs9478198	-
AKAP12	Q02952	VAR_035120	p.Glu1355Lys	LB/B	rs12201388	-
AKAP12	Q02952	VAR_035121	p.Glu1600Asp	LB/B	rs3823310	-
AKAP12	Q02952	VAR_035122	p.Glu1689Asp	LB/B	rs3734795	-
AKAP12	Q02952	VAR_035780	p.Glu240Lys	US	rs552053449	A colorectal cancer sample
AKAP12	Q02952	VAR_056731	p.Ala987Ser	LB/B	rs1042069	-
AKAP13	Q12802	VAR_030925	p.Met452Thr	LB/B	rs2061821	-
AKAP13	Q12802	VAR_030926	p.Trp494Arg	LB/B	rs2061822	-
AKAP13	Q12802	VAR_030927	p.Arg574Cys	LB/B	rs2061824	-
AKAP13	Q12802	VAR_030928	p.Gly624Val	LB/B	rs745191	-
AKAP13	Q12802	VAR_030929	p.Glu689Lys	LB/B	rs7177107	-
AKAP13	Q12802	VAR_030930	p.Val845Ala	LB/B	rs4075256	-
AKAP13	Q12802	VAR_030931	p.Val897Met	LB/B	rs4075254	-
AKAP13	Q12802	VAR_030932	p.Pro1062Ala	LB/B	rs4843074	-
AKAP13	Q12802	VAR_030933	p.Asp1086Asn	LB/B	rs4843075	-
AKAP13	Q12802	VAR_030934	p.Met1216Thr	LB/B	rs7162168	-
AKAP13	Q12802	VAR_030935	p.Gly2457Ser	LB/B	rs2241268	-
AKAP13	Q12802	VAR_030936	p.Ala2801Thr	LB/B	rs2614668	-
AKAP13	Q12802	VAR_051986	p.Lys526Gln	LB/B	rs34434221	-
AKAP13	Q12802	VAR_051987	p.Ser1525Gly	LB/B	rs35079107	-
AKAP17A	Q02040	VAR_055353	p.Pro194Ser	LB/B	rs17852504	-
AKAP2	Q9Y2D5	VAR_024248	p.Leu561Ser	LB/B	rs914358	-
AKAP3	O75969	VAR_036428	p.Arg831Cys	US	rs143517596	A colorectal cancer sample
AKAP3	O75969	VAR_055488	p.Gly118Glu	LB/B	rs2072355	-
AKAP3	O75969	VAR_055489	p.Ile500Thr	LB/B	rs12366671	-
AKAP3	O75969	VAR_055490	p.Ile661Thr	LB/B	rs1990313	-
AKAP3	O75969	VAR_055491	p.Ser700Phe	LB/B	rs2041291	-
AKAP3	O75969	VAR_055492	p.Ser725Leu	LB/B	rs2072357	-
AKAP3	O75969	VAR_059112	p.Ser700Pro	LB/B	rs2041290	-
AKAP3	O75969	VAR_060730	p.Thr464Ser	LB/B	rs11063266	-
AKAP3	O75969	VAR_061000	p.Glu525Lys	LB/B	rs1990312	-
AKAP4	Q5JQC9	VAR_027266	p.Ala673Gly	LB/B	rs12012704	-
AKAP4	Q5JQC9	VAR_048206	p.His233Arg	LB/B	rs17174078	-
AKAP5	P24588	VAR_056732	p.Pro100Leu	LB/B	rs2230491	-
AKAP5	P24588	VAR_056733	p.Glu314Lys	LB/B	rs34433837	-
AKAP5	P24588	VAR_060735	p.Thr203Ile	LB/B	rs1256149	-
AKAP6	Q13023	VAR_028171	p.Ala337Val	LB/B	rs3742926	-
AKAP6	Q13023	VAR_028172	p.Asn408Ser	LB/B	rs17099240	-
AKAP6	Q13023	VAR_028173	p.Ala1492Val	LB/B	rs11845640	-
AKAP6	Q13023	VAR_028174	p.Asn2035Asp	LB/B	rs1051695	-
AKAP6	Q13023	VAR_028175	p.Phe2171Tyr	LB/B	rs4647899	-
AKAP6	Q13023	VAR_028176	p.Asp2209His	LB/B	rs4402458	-
AKAP6	Q13023	VAR_035781	p.Lys910Met	US	-	A breast cancer sample
AKAP6	Q13023	VAR_035782	p.Met1192Ile	US	-	A breast cancer sample

AKAP6	Q13023	VAR_035783	p.Glu1702Gln	US	-	A breast cancer sample
AKAP6	Q13023	VAR_035784	p.Pro1839Thr	US	rs745389246	A colorectal cancer sample
AKAP6	Q13023	VAR_050653	p.Asn558Asp	LB/B	rs35210906	-
AKAP6	Q13023	VAR_050654	p.Glu892Lys	LB/B	rs34572259	-
AKAP6	Q13023	VAR_050655	p.Thr1516Ala	LB/B	rs17099587	-
AKAP6	Q13023	VAR_050656	p.Val1522Ile	LB/B	rs34711402	-
AKAP6	Q13023	VAR_050657	p.Glu2267Asp	LB/B	rs35977369	-
AKAP7	Q9P0M2	VAR_024246	p.Glu26Lys	LB/B	rs7771473	-
AKAP7	Q9P0M2	VAR_024247	p.Ser215Asn	LB/B	rs1190788	-
AKAP8	O43823	VAR_036534	p.Gln664His	US	-	A breast cancer sample
AKAP8L	Q9ULX6	VAR_068822	p.Gln458His	LB/B	rs2058322	-
AKAP9	Q99996	VAR_024249	p.Met463Ile	LB/B	rs6964587	-
AKAP9	Q99996	VAR_030162	p.Asn2792Ser	LB/B	rs6960867	-
AKAP9	Q99996	VAR_030163	p.Pro2979Ser	LB/B	rs1063242	-
AKAP9	Q99996	VAR_035785	p.Met2409Ile	US	-	A colorectal cancer sample
AKAP9	Q99996	VAR_035786	p.Glu3297Gln	US	rs756245027	A breast cancer sample
AKAP9	Q99996	VAR_043489	p.Ser1570Leu	LP/P	rs121908566	Long QT syndrome 11 (LQT11) [MIM:611820]
AKAP9	Q99996	VAR_043490	p.Lys2484Arg	LB/B	rs35759833	-
AKAP9	Q99996	VAR_043491	p.Gln3444Arg	LB/B	rs34956633	-
AKAP9	Q99996	VAR_043492	p.Met3614Val	LB/B	rs34327395	-
AKIP1	Q9NQ31	VAR_021565	p.Arg23Lys	LB/B	rs1133833	-
AKIP1	Q9NQ31	VAR_050688	p.Ile132Thr	LB/B	rs35131475	-
AKNA	Q7Z591	VAR_032586	p.Pro624Leu	LB/B	rs3748176	-
AKNA	Q7Z591	VAR_032587	p.Gln1097Arg	LB/B	rs1265891	-
AKNA	Q7Z591	VAR_032588	p.Arg1119Gln	LB/B	rs3748178	-
AKNA	Q7Z591	VAR_032589	p.Ser1303Pro	LB/B	rs2250242	-
AKNA	Q7Z591	VAR_032590	p.Tyr1327Cys	LB/B	rs2787344	-
AKNAD1	Q5T1N1	VAR_044198	p.Ser61Asn	LB/B	rs1277207	-
AKNAD1	Q5T1N1	VAR_044199	p.Ala104Val	LB/B	rs17621411	-
AKNAD1	Q5T1N1	VAR_044200	p.Glu167Gly	LB/B	rs17852793	-
AKNAD1	Q5T1N1	VAR_044201	p.His255Tyr	LB/B	rs9440631	-
AKNAD1	Q5T1N1	VAR_044202	p.Leu352Val	LB/B	rs11580913	-
AKNAD1	Q5T1N1	VAR_044203	p.Asn616Lys	LB/B	rs7551421	-
AKNAD1	Q5T1N1	VAR_044204	p.Cys654Tyr	LB/B	rs7522157	-
AKNAD1	Q5T1N1	VAR_061565	p.Gly582Val	LB/B	rs12060255	-
AKR1A1	P14550	VAR_048212	p.Asn52Ser	LB/B	rs2229540	-
AKR1A1	P14550	VAR_058909	p.Glu55Asp	LB/B	rs6690497	-
AKR1B1	P15121	VAR_014743	p.Ile15Phe	LB/B	rs5054	-
AKR1B1	P15121	VAR_014744	p.His42Leu	LB/B	rs5056	-
AKR1B1	P15121	VAR_014745	p.Leu73Val	LB/B	rs5057	-
AKR1B1	P15121	VAR_014746	p.Gly204Ser	LB/B	rs5061	-
AKR1B1	P15121	VAR_014747	p.Thr288Ile	LB/B	rs5062	-
AKR1B1	P15121	VAR_048213	p.Lys90Glu	LB/B	rs2229542	-
AKR1B10	O60218	VAR_013287	p.Asn313Asp	LB/B	rs4728329	-
AKR1B10	O60218	VAR_020077	p.Pro87Ser	LB/B	rs2303312	-
AKR1B10	O60218	VAR_020078	p.Met286Thr	LB/B	rs3735042	-
AKR1C1	Q04828	VAR_048214	p.Arg170His	LB/B	rs139588200	-
AKR1C1	Q04828	VAR_048215	p.Gln172Leu	LB/B	rs760575583	-
AKR1C2	P52895	VAR_014748	p.Leu172Gln	LB/B	rs11474	-

AKR1C2	P52895	VAR_048216	p.Phe46Tyr	LB/B	rs2854482	-
AKR1C2	P52895	VAR_066632	p.Ile79Val	LP/P	rs387906750	46,XY sex reversal 8 (SRXY8) [MIM:614279]
AKR1C2	P52895	VAR_066633	p.His90Gln	LP/P	rs797044460	46,XY sex reversal 8 (SRXY8) [MIM:614279]
AKR1C2	P52895	VAR_066634	p.His222Gln	LP/P	rs13222	46,XY sex reversal 8 (SRXY8) [MIM:614279]
AKR1C2	P52895	VAR_066635	p.Asn300Thr	LP/P	rs387906751	46,XY sex reversal 8 (SRXY8) [MIM:614279]
AKR1C3	P42330	VAR_013288	p.His5Gln	LB/B	rs12529	-
AKR1C3	P42330	VAR_013289	p.Met175Ile	LB/B	rs1131132	-
AKR1C3	P42330	VAR_032767	p.Arg66Gln	LB/B	rs35961894	-
AKR1C3	P42330	VAR_032768	p.Arg170Cys	LB/B	rs35575889	-
AKR1C3	P42330	VAR_032769	p.Pro180Ser	LB/B	rs34186955	-
AKR1C3	P42330	VAR_061001	p.Glu77Gly	LB/B	rs11551177	-
AKR1C4	P17516	VAR_013290	p.Ser145Cys	LB/B	rs3829125	-
AKR1C4	P17516	VAR_013291	p.Leu311Val	LB/B	rs17134592	-
AKR1C4	P17516	VAR_028240	p.Gly135Glu	LB/B	rs11253043	-
AKR1C4	P17516	VAR_028241	p.Cys170Tyr	LB/B	rs17851824	-
AKR1C4	P17516	VAR_028242	p.Gln250Arg	LB/B	rs4880718	-
AKR1C8P	Q5T2L2	VAR_032355	p.Arg50His	LB/B	rs7097295	-
AKR1D1	P51857	VAR_033007	p.Leu106Phe	LP/P	rs121918343	Congenital bile acid synthesis defect 2 (CBAS2) [MIM:235555]
AKR1D1	P51857	VAR_033008	p.Pro198Leu	LP/P	rs121918342	Congenital bile acid synthesis defect 2 (CBAS2) [MIM:235555]
AKR1D1	P51857	VAR_044430	p.Pro133Arg	LP/P	rs267606649	Congenital bile acid synthesis defect 2 (CBAS2) [MIM:235555]
AKR1D1	P51857	VAR_044431	p.Arg261Cys	LP/P	rs267606650	Congenital bile acid synthesis defect 2 (CBAS2) [MIM:235555]
AKR1D1	P51857	VAR_081756	p.Gly223Glu	LP/P	rs1228918719	Congenital bile acid synthesis defect 2 (CBAS2) [MIM:235555]
AKR1E2	Q96JD6	VAR_032356	p.Cys52Gly	LB/B	rs35429729	-
AKR1E2	Q96JD6	VAR_032357	p.Lys86Arg	LB/B	rs17133693	-
AKR7A2	O43488	VAR_017413	p.Ala142Thr	LB/B	rs1043657	-
AKR7A2	O43488	VAR_017414	p.Gln157His	LB/B	rs859208	-
AKR7A2	O43488	VAR_017415	p.Cys214Tyr	LB/B	rs2235794	-
AKR7A2	O43488	VAR_048209	p.Val135Met	LB/B	rs6670759	-
AKR7A2	O43488	VAR_048210	p.Gly198Ser	LB/B	rs2231200	-
AKR7A2	O43488	VAR_048211	p.Ser255Asn	LB/B	rs2231203	-
AKR7A2	O43488	VAR_060222	p.Glu180Lys	LB/B	rs859210	-
AKR7A3	O95154	VAR_017416	p.Val138Met	LB/B	rs2231198	-
AKR7A3	O95154	VAR_017417	p.Asn215Asp	LB/B	rs1738023	-
AKR7A3	O95154	VAR_017418	p.Thr323Ala	LB/B	rs1738025	-
AKR7L	Q8NHP1	VAR_046190	p.Ala255Thr	LB/B	rs2235795	-
AKR7L	Q8NHP1	VAR_046191	p.Phe322Val	LB/B	rs2982534	-
AKT1	P31749	VAR_051617	p.Val167Ala	LB/B	rs11555433	-
AKT1	P31749	VAR_055422	p.Glu17Lys	LP/P	rs121434592	Breast cancer (BC) [MIM:114480]
AKT1	P31749	VAR_055422	p.Glu17Lys	LP/P	rs121434592	Proteus syndrome (PROTEUSS) [MIM:176920]
AKT1	P31749	VAR_069791	p.Arg25Cys	LP/P	rs397514644	Cowden syndrome 6 (CWS6) [MIM:615109]
AKT1	P31749	VAR_069792	p.Thr435Pro	LP/P	rs397514645	Cowden syndrome 6 (CWS6) [MIM:615109]
AKT1S1	Q96B36	VAR_028239	p.Ala47Pro	LB/B	rs17850191	-
AKT2	P31751	VAR_040356	p.Ile188Val	LB/B	rs55859611	-
AKT2	P31751	VAR_040357	p.Arg208Lys	LB/B	rs35817154	-

AKT2 P31751 VAR_067309 p.Glu17Lys LP/P rs387906659 Hypoinsulinemic hypoglycemia with hemihypertrophy (HIHGHH) [MIM:240900]
AKT2 P31751 VAR_067310 p.Arg274His LP/P rs121434593 Diabetes mellitus, non-insulin-dependent (NIDDM) [MIM:125853]
AKT3 Q9Y243 VAR_040358 p.Gly171Arg US rs1402272180 A glioblastoma multiforme sample
AKT3 Q9Y243 VAR_065830 p.Glu17Lys US rs397514606 Melanoma
AKT3 Q9Y243 VAR_065830 p.Glu17Lys LP/P rs397514606 Megalencephaly-polymicrogyria-polydactyly-hydrocephalus syndrome 2 (MPPH2) [MIM:615937]
AKT3 Q9Y243 VAR_069260 p.Asn229Ser LP/P rs397514605 Megalencephaly-polymicrogyria-polydactyly-hydrocephalus syndrome 2 (MPPH2) [MIM:615937]
AKT3 Q9Y243 VAR_069261 p.Arg465Trp LP/P rs587776935 Megalencephaly-polymicrogyria-polydactyly-hydrocephalus syndrome 2 (MPPH2) [MIM:615937]
ALAD P13716 VAR_003633 p.Lys59Asn LB/B rs1800435 -
ALAD P13716 VAR_003634 p.Gly133Arg LP/P rs121912980 Acute hepatic porphyria (AHEPP) [MIM:612740]
ALAD P13716 VAR_003635 p.Arg240Trp LP/P rs121912982 Acute hepatic porphyria (AHEPP) [MIM:612740]
ALAD P13716 VAR_003636 p.Ala274Thr LP/P rs121912983 Acute hepatic porphyria (AHEPP) [MIM:612740]
ALAD P13716 VAR_003637 p.Val275Met LP/P rs121912981 Acute hepatic porphyria (AHEPP) [MIM:612740]
ALAD P13716 VAR_020973 p.Phe12Leu LB/B rs121912984 -
ALAD P13716 VAR_020974 p.Val153Met LP/P rs1554740221 Acute hepatic porphyria (AHEPP) [MIM:612740]
ALAS2 P22557 VAR_000562 p.Thr388Ser LP/P rs137852300 Anemia, sideroblastic, 1 (SIDBA1) [MIM:300751]
ALAS2 P22557 VAR_000563 p.Arg411Cys LP/P rs137852305 Anemia, sideroblastic, 1 (SIDBA1) [MIM:300751]
ALAS2 P22557 VAR_000564 p.Ile476Asn LP/P rs137852299 Anemia, sideroblastic, 1 (SIDBA1) [MIM:300751]
ALAS2 P22557 VAR_012334 p.Tyr199His LP/P rs137852310 Anemia, sideroblastic, 1 (SIDBA1) [MIM:300751]
ALAS2 P22557 VAR_012335 p.Arg204Gln LP/P rs1338391423 Anemia, sideroblastic, 1 (SIDBA1) [MIM:300751]
ALAS2 P22557 VAR_012336 p.Arg448Gln LP/P - Anemia, sideroblastic, 1 (SIDBA1) [MIM:300751]
ALAS2 P22557 VAR_012337 p.Arg452Cys LP/P rs137852311 Anemia, sideroblastic, 1 (SIDBA1) [MIM:300751]
ALAS2 P22557 VAR_018604 p.Asp159Tyr LP/P rs137852308 Anemia, sideroblastic, 1 (SIDBA1) [MIM:300751]
ALAS2 P22557 VAR_018605 p.Arg560His LP/P rs892041887 Anemia, sideroblastic, 1 (SIDBA1) [MIM:300751]
ALAS2 P22557 VAR_066232 p.Lys156Glu LP/P - Anemia, sideroblastic, 1 (SIDBA1) [MIM:300751]
ALAS2 P22557 VAR_066233 p.Arg170His LP/P - Anemia, sideroblastic, 1 (SIDBA1) [MIM:300751]
ALAS2 P22557 VAR_066234 p.Arg218His LP/P rs185504937 Anemia, sideroblastic, 1 (SIDBA1) [MIM:300751]
ALAS2 P22557 VAR_066235 p.Glu242Lys LP/P - Anemia, sideroblastic, 1 (SIDBA1) [MIM:300751]
ALAS2 P22557 VAR_066236 p.Asp263Asn LP/P - Anemia, sideroblastic, 1 (SIDBA1) [MIM:300751]
ALAS2 P22557 VAR_066237 p.Pro339Leu LP/P - Anemia, sideroblastic, 1 (SIDBA1) [MIM:300751]
ALAS2 P22557 VAR_066238 p.Arg375Cys LP/P - Anemia, sideroblastic, 1 (SIDBA1) [MIM:300751]
ALAS2 P22557 VAR_066239 p.Arg411His LP/P - Anemia, sideroblastic, 1 (SIDBA1) [MIM:300751]
ALAS2 P22557 VAR_066240 p.Arg452Gly LP/P - Anemia, sideroblastic, 1 (SIDBA1) [MIM:300751]
ALAS2 P22557 VAR_066241 p.Arg452His LP/P rs863223904 Anemia, sideroblastic, 1 (SIDBA1) [MIM:300751]
ALAS2 P22557 VAR_066242 p.Pro520Leu LB/B rs201062903 Anemia, sideroblastic, 1 (SIDBA1) [MIM:300751]
ALAS2 P22557 VAR_066243 p.Arg572His LP/P - Anemia, sideroblastic, 1 (SIDBA1) [MIM:300751]
ALAS2 P22557 VAR_066244 p.Tyr586Phe LB/B rs139596860 -
ALAS2 P22557 VAR_072328 p.Phe165Leu LP/P rs137852301 Anemia, sideroblastic, 1 (SIDBA1) [MIM:300751]
ALAS2 P22557 VAR_072329 p.Arg170Cys LP/P - Anemia, sideroblastic, 1 (SIDBA1) [MIM:300751]
ALAS2 P22557 VAR_072330 p.Val301Ala LP/P - Anemia, sideroblastic, 1 (SIDBA1) [MIM:300751]
ALAS2 P22557 VAR_072331 p.Arg517Gly LP/P - Anemia, sideroblastic, 1 (SIDBA1) [MIM:300751]
ALB P02768 VAR_000499 p.Arg23Cys LB/B rs80008208 -
ALB P02768 VAR_000500 p.Arg23His LB/B rs72552709 -
ALB P02768 VAR_000501 p.Arg24Leu LB/B rs74821926 -
ALB P02768 VAR_000502 p.Arg24Pro LB/B rs74821926 -

ALB	P02768	VAR_000503	p.Arg24Gln	LB/B	rs74821926	-
ALB	P02768	VAR_000504	p.Asp25Val	LB/B	rs75353611	-
ALB	P02768	VAR_000505	p.His27Gln	LB/B	rs76285851	-
ALB	P02768	VAR_000506	p.His27Tyr	LB/B	rs141733599	-
ALB	P02768	VAR_000507	p.Glu84Lys	LB/B	rs77050410	-
ALB	P02768	VAR_000508	p.Asp87Asn	LB/B	rs78574148	-
ALB	P02768	VAR_000509	p.Glu106Lys	LB/B	rs80296402	-
ALB	P02768	VAR_000510	p.Arg138Gly	LB/B	rs77238412	-
ALB	P02768	VAR_000511	p.Glu143Lys	LB/B	rs75522063	-
ALB	P02768	VAR_000512	p.His152Arg	LB/B	rs80095457	-
ALB	P02768	VAR_000513	p.Cys201Phe	LB/B	rs77656691	-
ALB	P02768	VAR_000514	p.Arg242His	LP/P	rs75002628	Hyperthyroxinemia, familial dysalbuminemic (FDAH) [MIM:615999]
ALB	P02768	VAR_000515	p.Lys249Gln	LB/B	rs79804069	-
ALB	P02768	VAR_000516	p.Lys264Glu	LB/B	rs79377490	-
ALB	P02768	VAR_000517	p.Gln292Arg	LB/B	rs80002911	-
ALB	P02768	VAR_000518	p.Asp293Gly	LB/B	rs79744198	-
ALB	P02768	VAR_000519	p.Lys300Asn	LB/B	rs74718349	-
ALB	P02768	VAR_000520	p.Lys337Asn	LB/B	rs72552710	-
ALB	P02768	VAR_000521	p.Asn342Lys	LB/B	rs77544362	-
ALB	P02768	VAR_000522	p.Ala344Thr	LB/B	rs78953271	-
ALB	P02768	VAR_000523	p.Glu345Lys	LB/B	rs72552711	-
ALB	P02768	VAR_000524	p.Glu357Lys	LB/B	rs77354753	-
ALB	P02768	VAR_000525	p.Glu378Lys	LB/B	rs76593094	-
ALB	P02768	VAR_000526	p.Glu382Lys	LB/B	rs75791663	-
ALB	P02768	VAR_000527	p.Asp389His	LB/B	rs77187142	-
ALB	P02768	VAR_000528	p.Asp389Val	LB/B	rs78538497	-
ALB	P02768	VAR_000529	p.Lys396Glu	LB/B	rs78166690	-
ALB	P02768	VAR_000530	p.Asp399Asn	LB/B	rs77514449	-
ALB	P02768	VAR_000531	p.Glu400Lys	LB/B	rs79047363	-
ALB	P02768	VAR_000532	p.Glu400Gln	LB/B	rs79047363	-
ALB	P02768	VAR_000533	p.Glu406Lys	LB/B	rs76483862	-
ALB	P02768	VAR_000534	p.Glu503Lys	LB/B	rs80259813	-
ALB	P02768	VAR_000535	p.Asp518Asn	LB/B	rs75920790	-
ALB	P02768	VAR_000536	p.Glu525Lys	LB/B	rs75523493	-
ALB	P02768	VAR_000537	p.Glu529Lys	LB/B	rs74826639	-
ALB	P02768	VAR_000538	p.Lys560Glu	LB/B	rs77645174	-
ALB	P02768	VAR_000539	p.Lys565Glu	LB/B	rs80345158	-
ALB	P02768	VAR_000540	p.Asp574Gly	LB/B	rs79738788	-
ALB	P02768	VAR_000541	p.Asp574Ala	LB/B	rs79738788	-
ALB	P02768	VAR_000542	p.Asp587Asn	LB/B	rs76587671	-
ALB	P02768	VAR_000543	p.Glu589Lys	LB/B	rs75709682	-
ALB	P02768	VAR_000544	p.Glu594Lys	LB/B	rs79228041	-
ALB	P02768	VAR_000545	p.Lys597Glu	LB/B	rs80106970	-
ALB	P02768	VAR_000546	p.Lys598Asn	LB/B	rs75738598	-
ALB	P02768	VAR_010657	p.Phe73Tyr	LB/B	-	-
ALB	P02768	VAR_013011	p.Leu90Pro	LP/P	rs77892378	Hyperthyroxinemia, familial dysalbuminemic (FDAH) [MIM:615999]
ALB	P02768	VAR_013012	p.Val146Glu	LB/B	rs77752336	-

ALB P02768 VAR_013013 p.Arg242Pro LP/P rs75002628 Hyperthyroxinemia, familial dysalbuminemic (FDAH) [MIM:615999]
ALB P02768 VAR_013014 p.Asp338Gly LB/B rs76242087 -
ALB P02768 VAR_013015 p.Asp338Val LB/B rs76242087 -
ALB P02768 VAR_013016 p.Lys383Asn LB/B rs75069738 -
ALB P02768 VAR_013017 p.Arg434Cys LB/B rs78575701 -
ALB P02768 VAR_013018 p.Val557Met LB/B rs78284052 -
ALB P02768 VAR_013019 p.Lys584Glu LB/B rs76671808 -
ALB P02768 VAR_014290 p.Glu121Gly LB/B - -
ALB P02768 VAR_014291 p.Ala215Thr LB/B rs3210154 -
ALB P02768 VAR_014292 p.Ala215Val LB/B rs3204504 -
ALB P02768 VAR_014293 p.Gln220Leu LB/B rs3210163 -
ALB P02768 VAR_014294 p.Glu420Lys LB/B - -
ALB P02768 VAR_014295 p.Lys490Glu LB/B rs1063469 -
ALCAM Q13740 VAR_003907 p.Asn258Ser LB/B rs1044240 -
ALCAM Q13740 VAR_003908 p.Thr301Met LB/B rs1044243 -
ALCAM Q13740 VAR_029514 p.Gly229Asp LB/B rs10933819 -
ALCAM Q13740 VAR_029515 p.Leu315Met LB/B rs12629872 -
ALCAM Q13740 VAR_029516 p.Val352Met LB/B rs2291375 -
ALCAM Q13740 VAR_049856 p.Met367Ile LB/B rs34926152 -
ALDH16A1 Q8IZ83 VAR_037638 p.Glu110Lys LB/B rs3745312 -
ALDH16A1 Q8IZ83 VAR_037639 p.Leu227Val LB/B rs1320303 -
ALDH18A1 P54886 VAR_038482 p.Arg84Gln LP/P rs121434582 Cutis laxa, autosomal recessive, 3A (ARCL3A) [MIM:219150]
ALDH18A1 P54886 VAR_051792 p.Thr299Ile LB/B rs2275272 Cutis laxa, autosomal recessive, 3A (ARCL3A) [MIM:219150]
ALDH18A1 P54886 VAR_051793 p.Ser372Tyr LB/B rs3765571 -
ALDH18A1 P54886 VAR_058006 p.His784Tyr LP/P rs121434583 Cutis laxa, autosomal recessive, 3A (ARCL3A) [MIM:219150]
ALDH18A1 P54886 VAR_075884 p.Gly93Arg LP/P - Cutis laxa, autosomal recessive, 3A (ARCL3A) [MIM:219150]
ALDH18A1 P54886 VAR_075885 p.Val120Ala LP/P rs863224945 Spastic paraplegia 9A, autosomal dominant (SPG9A) [MIM:601162]
ALDH18A1 P54886 VAR_075886 p.Arg128His LP/P rs768323248 Spastic paraplegia 9B, autosomal recessive (SPG9B) [MIM:616586]
ALDH18A1 P54886 VAR_075887 p.Arg138Leu LP/P rs863225045 Cutis laxa, autosomal dominant, 3 (ADCL3) [MIM:616603]
ALDH18A1 P54886 VAR_075888 p.Arg138Gln LP/P rs863225045 Cutis laxa, autosomal dominant, 3 (ADCL3) [MIM:616603]
ALDH18A1 P54886 VAR_075889 p.Arg138Trp LP/P rs863225044 Cutis laxa, autosomal dominant, 3 (ADCL3) [MIM:616603]
ALDH18A1 P54886 VAR_075890 p.Val243Leu LP/P rs864321669 Spastic paraplegia 9A, autosomal dominant (SPG9A) [MIM:601162]
ALDH18A1 P54886 VAR_075891 p.Arg252Gln LP/P rs864321670 Spastic paraplegia 9A, autosomal dominant (SPG9A) [MIM:601162]
ALDH18A1 P54886 VAR_075892 p.Leu637Pro LP/P rs869320690 Spastic paraplegia 9B, autosomal recessive (SPG9B) [MIM:616586]
ALDH18A1 P54886 VAR_075893 p.Ser652Phe LP/P - Spastic paraplegia 9A, autosomal dominant (SPG9A) [MIM:601162]

ALDH18A1 P54886 VAR_075894 p.Arg665Leu LP/P rs766264810 Spastic paraplegia 9A, autosomal dominant (SPG9A) [MIM:601162]
ALDH18A1 P54886 VAR_075895 p.Asp715His LP/P rs752669339 Spastic paraplegia 9B, autosomal recessive (SPG9B) [MIM:616586]
ALDH18A1 P54886 VAR_075896 p.Tyr782Cys LP/P rs774047299 Cutis laxa, autosomal recessive, 3A (ARCL3A) [MIM:219150]
ALDH1A1 P00352 VAR_017778 p.Ile177Phe LB/B rs8187929 -
ALDH1A1 P00352 VAR_048901 p.Asn121Ser LB/B rs1049981 -
ALDH1A1 P00352 VAR_048902 p.Gly125Arg LB/B rs11554423 -
ALDH1A2 O94788 VAR_025439 p.Glu50Gly LB/B rs34266719 -
ALDH1A2 O94788 VAR_025440 p.Ala110Val LB/B rs35365164 -
ALDH1A2 O94788 VAR_025441 p.Val348Ile LB/B rs4646626 -
ALDH1A2 O94788 VAR_025442 p.Glu436Lys LB/B rs34744827 -
ALDH1A3 P47895 VAR_019706 p.Met386Val LB/B rs3803430 -
ALDH1A3 P47895 VAR_069322 p.Arg89Cys LP/P rs397514652 Microphthalmia, isolated, 8 (MCOP8) [MIM:615113]
ALDH1A3 P47895 VAR_069323 p.Ala145Val LP/P rs754619607 Microphthalmia, isolated, 8 (MCOP8) [MIM:615113]
ALDH1A3 P47895 VAR_069324 p.Ile369Phe LP/P - Microphthalmia, isolated, 8 (MCOP8) [MIM:615113]
ALDH1A3 P47895 VAR_069325 p.Ala493Pro LP/P rs397514653 Microphthalmia, isolated, 8 (MCOP8) [MIM:615113]
ALDH1A3 P47895 VAR_072332 p.Val71Met LP/P rs386834230 Microphthalmia, isolated, 8 (MCOP8) [MIM:615113]
ALDH1A3 P47895 VAR_072333 p.Cys174Tyr LP/P - Microphthalmia, isolated, 8 (MCOP8) [MIM:615113]
ALDH1A3 P47895 VAR_072334 p.Pro355Arg LP/P - Microphthalmia, isolated, 8 (MCOP8) [MIM:615113]
ALDH1A3 P47895 VAR_072335 p.Gly382Arg LP/P - Microphthalmia, isolated, 8 (MCOP8) [MIM:615113]
ALDH1A3 P47895 VAR_072336 p.Glu411Lys LP/P - Microphthalmia, isolated, 8 (MCOP8) [MIM:615113]
ALDH1A3 P47895 VAR_072337 p.Asn466Lys LP/P - Microphthalmia, isolated, 8 (MCOP8) [MIM:615113]
ALDH1B1 P30837 VAR_002257 p.Ala86Val LB/B rs2228093 -
ALDH1B1 P30837 VAR_002258 p.Leu107Arg LB/B rs2073478 -
ALDH1B1 P30837 VAR_029891 p.Thr202Ile LB/B rs4646773 -
ALDH1B1 P30837 VAR_029892 p.Val253Met LB/B rs4878199 -
ALDH1L1 O75891 VAR_036101 p.Ala511Val US rs768309358 A colorectal cancer sample
ALDH1L1 O75891 VAR_052290 p.Leu254Pro LB/B rs3796191 -
ALDH1L1 O75891 VAR_052291 p.Val330Phe LB/B rs2886059 -
ALDH1L1 O75891 VAR_052292 p.Glu429Ala LB/B rs9282691 -
ALDH1L1 O75891 VAR_052293 p.Ala436Thr LB/B rs9282692 -
ALDH1L1 O75891 VAR_052295 p.Ser448Asn LB/B rs9282697 -
ALDH1L1 O75891 VAR_052296 p.Ser481Gly LB/B rs2276724 -
ALDH1L1 O75891 VAR_052297 p.Asp793Gly LB/B rs1127717 -
ALDH1L1 O75891 VAR_052298 p.Glu803Lys LB/B rs9282689 -
ALDH1L1 O75891 VAR_052299 p.Ile812Val LB/B rs4646750 -
ALDH2 P05091 VAR_002248 p.Glu504Lys LB/B rs671 -
ALDH2 P05091 VAR_011302 p.Glu496Lys LB/B rs769724893 -
ALDH2 P05091 VAR_011869 p.Glu337Val LB/B rs1062136 -
ALDH3A1 P30838 VAR_011303 p.Pro329Ala LB/B rs2228100 -
ALDH3A1 P30838 VAR_018981 p.Ser134Ala LB/B rs887241 -
ALDH3A1 P30838 VAR_018982 p.Gly309Glu LB/B rs3744692 -
ALDH3A2 P51648 VAR_002249 p.Leu106Arg LP/P rs72547558 Sjoegren-Larsson syndrome (SLS) [MIM:270200]

ALDH3A2 P51648 VAR_002250 p.Cys214Tyr LP/P rs72547564 Sjoegren-Larsson syndrome (SLS) [MIM:270200]
ALDH3A2 P51648 VAR_002251 p.Cys226Trp LP/P rs72547565 Sjoegren-Larsson syndrome (SLS) [MIM:270200]
ALDH3A2 P51648 VAR_002252 p.Asp245Asn LP/P rs72547568 Sjoegren-Larsson syndrome (SLS) [MIM:270200]
ALDH3A2 P51648 VAR_002254 p.Pro315Ser LP/P rs72547571 Sjoegren-Larsson syndrome (SLS) [MIM:270200]
ALDH3A2 P51648 VAR_002255 p.Ser365Leu LP/P rs72547573 Sjoegren-Larsson syndrome (SLS) [MIM:270200]
ALDH3A2 P51648 VAR_002256 p.Gly412Arg LP/P rs778115541 Sjoegren-Larsson syndrome (SLS) [MIM:270200]
ALDH3A2 P51648 VAR_017510 p.Ile45Phe LP/P - Sjoegren-Larsson syndrome (SLS) [MIM:270200]
ALDH3A2 P51648 VAR_017511 p.Val64Asp LP/P rs72547556 Sjoegren-Larsson syndrome (SLS) [MIM:270200]
ALDH3A2 P51648 VAR_017512 p.Pro114Leu LP/P rs72547559 Sjoegren-Larsson syndrome (SLS) [MIM:270200]
ALDH3A2 P51648 VAR_017513 p.Pro121Leu LP/P rs72547560 Sjoegren-Larsson syndrome (SLS) [MIM:270200]
ALDH3A2 P51648 VAR_017514 p.Thr184Met LP/P rs72547562 Sjoegren-Larsson syndrome (SLS) [MIM:270200]
ALDH3A2 P51648 VAR_017515 p.Thr184Arg LP/P - Sjoegren-Larsson syndrome (SLS) [MIM:270200]
ALDH3A2 P51648 VAR_017516 p.Gly185Ala LP/P rs72547563 Sjoegren-Larsson syndrome (SLS) [MIM:270200]
ALDH3A2 P51648 VAR_017517 p.Arg228Cys LP/P rs72547566 Sjoegren-Larsson syndrome (SLS) [MIM:270200]
ALDH3A2 P51648 VAR_017518 p.Cys237Tyr LP/P rs72547567 Sjoegren-Larsson syndrome (SLS) [MIM:270200]
ALDH3A2 P51648 VAR_017519 p.Lys266Asn LP/P rs72547569 Sjoegren-Larsson syndrome (SLS) [MIM:270200]
ALDH3A2 P51648 VAR_017520 p.Tyr279Asn LP/P rs72547570 Sjoegren-Larsson syndrome (SLS) [MIM:270200]
ALDH3A2 P51648 VAR_017521 p.Met328Ile LP/P rs72547572 Sjoegren-Larsson syndrome (SLS) [MIM:270200]
ALDH3A2 P51648 VAR_017522 p.Asn386Ser LP/P rs72547575 Sjoegren-Larsson syndrome (SLS) [MIM:270200]
ALDH3A2 P51648 VAR_017523 p.Gly406Arg LP/P - Sjoegren-Larsson syndrome (SLS) [MIM:270200]
ALDH3A2 P51648 VAR_017524 p.His411Tyr LP/P - Sjoegren-Larsson syndrome (SLS) [MIM:270200]
ALDH3A2 P51648 VAR_017525 p.Ser415Asn LP/P - Sjoegren-Larsson syndrome (SLS) [MIM:270200]
ALDH3A2 P51648 VAR_017526 p.Phe419Ser LP/P rs72547576 Sjoegren-Larsson syndrome (SLS) [MIM:270200]
ALDH3A2 P51648 VAR_017527 p.Arg423His LP/P rs768290318 Sjoegren-Larsson syndrome (SLS) [MIM:270200]
ALDH3A2 P51648 VAR_017528 p.Lys447Glu LP/P rs67939114 Sjoegren-Larsson syndrome (SLS) [MIM:270200]
ALDH3B2 P48448 VAR_022058 p.Ala50Thr LB/B rs3741178 -
ALDH3B2 P48448 VAR_055699 p.Ser302Arg LB/B rs4646826 -
ALDH3B2 P48448 VAR_058696 p.Ser52Asn LB/B rs1551888 -
ALDH3B2 P48448 VAR_058697 p.His203Arg LB/B rs6591270 -
ALDH3B2 P48448 VAR_058698 p.Ser220Gly LB/B rs2447571 -
ALDH3B2 P48448 VAR_058699 p.Arg276Trp LB/B rs17856219 -
ALDH3B2 P48448 VAR_058700 p.His361Arg LB/B rs1551886 -
ALDH4A1 P30038 VAR_002259 p.Pro16Leu LB/B rs146450609 -
ALDH4A1 P30038 VAR_002260 p.Ser352Leu LP/P rs137852937 Hyperprolinemia 2 (HYRPRO2) [MIM:239510]
ALDH4A1 P30038 VAR_029337 p.Val470Ile LB/B rs2230709 -
ALDH4A1 P30038 VAR_048903 p.Thr473Ala LB/B rs6695033 -
ALDH5A1 P51649 VAR_016758 p.His180Tyr LB/B rs2760118 -
ALDH5A1 P51649 VAR_016759 p.Pro182Leu LB/B rs3765310 -
ALDH5A1 P51649 VAR_026199 p.Cys93Phe LP/P rs765561257 Succinic semialdehyde dehydrogenase deficiency (SSADHD) [MIM:271980]
ALDH5A1 P51649 VAR_026200 p.Gly176Arg LP/P rs72552281 Succinic semialdehyde dehydrogenase deficiency (SSADHD) [MIM:271980]
ALDH5A1 P51649 VAR_026201 p.Cys223Tyr LP/P rs72552282 Succinic semialdehyde dehydrogenase deficiency (SSADHD) [MIM:271980]
ALDH5A1 P51649 VAR_026202 p.Thr233Met LP/P rs1326526453 Succinic semialdehyde dehydrogenase deficiency (SSADHD) [MIM:271980]
ALDH5A1 P51649 VAR_026203 p.Asn255Ser LP/P rs145087265 Succinic semialdehyde dehydrogenase deficiency (SSADHD) [MIM:271980]

ALDH5A1 P51649 VAR_026204 p.Gly268Glu LP/P rs375628463 Succinic semialdehyde dehydrogenase deficiency (SSADHD) [MIM:271980]
ALDH5A1 P51649 VAR_026205 p.Asn335Lys LP/P rs72552283 Succinic semialdehyde dehydrogenase deficiency (SSADHD) [MIM:271980]
ALDH5A1 P51649 VAR_026206 p.Pro382Leu LP/P - Succinic semialdehyde dehydrogenase deficiency (SSADHD) [MIM:271980]
ALDH5A1 P51649 VAR_026207 p.Pro382Gln LP/P - Succinic semialdehyde dehydrogenase deficiency (SSADHD) [MIM:271980]
ALDH5A1 P51649 VAR_026208 p.Gly409Asp LP/P rs118203984 Succinic semialdehyde dehydrogenase deficiency (SSADHD) [MIM:271980]
ALDH5A1 P51649 VAR_026209 p.Val487Glu LP/P - Succinic semialdehyde dehydrogenase deficiency (SSADHD) [MIM:271980]
ALDH5A1 P51649 VAR_026210 p.Gly533Arg LP/P rs72552284 Succinic semialdehyde dehydrogenase deficiency (SSADHD) [MIM:271980]
ALDH5A1 P51649 VAR_026227 p.Gly36Arg LB/B rs4646832 -
ALDH5A1 P51649 VAR_026228 p.Ala237Ser LB/B rs62621664 -
ALDH5A1 P51649 VAR_026229 p.Val406Ile LB/B rs143741652 -
ALDH5A1 P51649 VAR_069047 p.Asn372Ser LB/B - -
ALDH6A1 Q02252 VAR_010244 p.Gly446Arg LP/P rs72552258 Methylmalonate semialdehyde dehydrogenase deficiency (MMSDHD) [MIM:614105]
ALDH7A1 P49419 VAR_028202 p.Thr412Ala LB/B rs2306618 -
ALDH7A1 P49419 VAR_028203 p.Lys439Gln LB/B rs12514417 -
ALDH7A1 P49419 VAR_031718 p.Ala199Val LP/P rs121912709 Pyridoxine-dependent epilepsy (PDE) [MIM:266100]
ALDH7A1 P49419 VAR_031719 p.Glu427Gln LP/P rs121912707 Pyridoxine-dependent epilepsy (PDE) [MIM:266100]
ALDH7A1 P49419 VAR_069184 p.Gly202Val LP/P - Pyridoxine-dependent epilepsy (PDE) [MIM:266100]
ALDH7A1 P49419 VAR_069185 p.Gly291Glu LP/P - Pyridoxine-dependent epilepsy (PDE) [MIM:266100]
ALDH7A1 P49419 VAR_069186 p.Asn301Ile LP/P rs121912711 Pyridoxine-dependent epilepsy (PDE) [MIM:266100]
ALDH7A1 P49419 VAR_069187 p.Arg335Gln LP/P rs754449549 Pyridoxine-dependent epilepsy (PDE) [MIM:266100]
ALDH7A1 P49419 VAR_069188 p.Val395Gly LP/P - Pyridoxine-dependent epilepsy (PDE) [MIM:266100]
ALDH7A1 P49419 VAR_069189 p.Ser458Asn LP/P - Pyridoxine-dependent epilepsy (PDE) [MIM:266100]
ALDH8A1 Q9H2A2 VAR_037618 p.Phe402Ser LB/B rs2294315 -
ALDH9A1 P49189 VAR_011304 p.Cys116Ser LB/B - -
ALDOA P04075 VAR_000550 p.Asp129Gly LP/P rs121909533 Glycogen storage disease 12 (GSD12) [MIM:611881]
ALDOA P04075 VAR_044142 p.Glu207Lys LP/P rs121909534 Glycogen storage disease 12 (GSD12) [MIM:611881]
ALDOA P04075 VAR_044143 p.Cys339Tyr LP/P - Glycogen storage disease 12 (GSD12) [MIM:611881]
ALDOA P04075 VAR_044144 p.Gly347Ser LB/B rs138824667 Glycogen storage disease 12 (GSD12) [MIM:611881]
ALDOA P04075 VAR_048219 p.Glu82Gln LB/B rs11553107 -
ALDOA P04075 VAR_048220 p.Gly142Val LB/B rs11553108 -
ALDOB P05062 VAR_000551 p.Cys135Arg LP/P - Hereditary fructose intolerance (HFI) [MIM:229600]
ALDOB P05062 VAR_000552 p.Trp148Arg LB/B rs118204430 -
ALDOB P05062 VAR_000553 p.Ala150Pro LP/P rs1800546 Hereditary fructose intolerance (HFI) [MIM:229600]

ALDOB P05062 VAR_000554 p.Ala175Asp LP/P rs76917243 Hereditary fructose intolerance (HFI) [MIM:229600]
ALDOB P05062 VAR_000555 p.Leu257Pro LP/P rs764701775 Hereditary fructose intolerance (HFI) [MIM:229600]
ALDOB P05062 VAR_000556 p.Arg304Trp LP/P rs555935217 Hereditary fructose intolerance (HFI) [MIM:229600]
ALDOB P05062 VAR_000557 p.Asn335Lys LP/P rs78340951 Hereditary fructose intolerance (HFI) [MIM:229600]
ALDOB P05062 VAR_000558 p.Ala338Val LP/P rs77718928 Hereditary fructose intolerance (HFI) [MIM:229600]
ALDOB P05062 VAR_020822 p.Ile74Thr LP/P rs781023784 Hereditary fructose intolerance (HFI) [MIM:229600]
ALDOB P05062 VAR_020824 p.Pro185Arg LP/P - Hereditary fructose intolerance (HFI) [MIM:229600]
ALDOB P05062 VAR_020825 p.Glu207Gln LB/B rs3739721 -
ALDOB P05062 VAR_020826 p.Val222Phe LP/P rs1554702442 Hereditary fructose intolerance (HFI) [MIM:229600]
ALDOB P05062 VAR_020827 p.Leu229Pro LP/P rs1554702433 Hereditary fructose intolerance (HFI) [MIM:229600]
ALDOB P05062 VAR_020828 p.Arg304Gln LP/P rs145078268 Hereditary fructose intolerance (HFI) [MIM:229600]
ALDOB P05062 VAR_038429 p.Arg134Ser LB/B rs10123355 -
ALDOB P05062 VAR_038430 p.Ile268Asn LB/B rs10989495 -
ALDOB P05062 VAR_058211 p.Cys178Arg LP/P - Hereditary fructose intolerance (HFI) [MIM:229600]
ALDOB P05062 VAR_058212 p.Leu284Pro LP/P - Hereditary fructose intolerance (HFI) [MIM:229600]
ALDOB P05062 VAR_075348 p.Arg46Trp LP/P rs41281039 Hereditary fructose intolerance (HFI) [MIM:229600]
ALDOB P05062 VAR_075349 p.Tyr343His LP/P rs369586696 Hereditary fructose intolerance (HFI) [MIM:229600]
ALG1 Q9BT22 VAR_023364 p.Ser150Arg LP/P rs121908340 Congenital disorder of glycosylation 1K (CDG1K) [MIM:608540]
ALG1 Q9BT22 VAR_023365 p.Ser258Leu LP/P rs28939378 Congenital disorder of glycosylation 1K (CDG1K) [MIM:608540]
ALG1 Q9BT22 VAR_023366 p.Gln342Pro LP/P rs267606651 Congenital disorder of glycosylation 1K (CDG1K) [MIM:608540]
ALG1 Q9BT22 VAR_023367 p.Asp429Glu LB/B rs9745522 -
ALG1 Q9BT22 VAR_038425 p.Ser267Asn LB/B rs17849848 -
ALG1 Q9BT22 VAR_038426 p.Leu325Met LB/B rs17852920 -
ALG1 Q9BT22 VAR_038427 p.Gln455Arg LB/B rs17856919 -
ALG1 Q9BT22 VAR_049350 p.Arg438Trp LP/P rs16835020 Congenital disorder of glycosylation 1K (CDG1K) [MIM:608540]
ALG1 Q9BT22 VAR_077187 p.Gln50Arg LP/P rs794726944 Congenital disorder of glycosylation 1K (CDG1K) [MIM:608540]
ALG1 Q9BT22 VAR_077188 p.Ser71Phe LP/P rs200605408 Congenital disorder of glycosylation 1K (CDG1K) [MIM:608540]
ALG1 Q9BT22 VAR_077189 p.His74Leu LP/P rs201337379 Congenital disorder of glycosylation 1K (CDG1K) [MIM:608540]
ALG1 Q9BT22 VAR_077190 p.Leu88Val LP/P rs794727301 Congenital disorder of glycosylation 1K (CDG1K) [MIM:608540]

ALG1 Q9BT22 VAR_077191 p.Pro98Leu LP/P - Congenital disorder of glycosylation 1K (CDG1K) [MIM:608540]
ALG1 Q9BT22 VAR_077192 p.Leu114Phe LP/P - Congenital disorder of glycosylation 1K (CDG1K) [MIM:608540]
ALG1 Q9BT22 VAR_077193 p.Ile209Ser LP/P - Congenital disorder of glycosylation 1K (CDG1K) [MIM:608540]
ALG1 Q9BT22 VAR_077194 p.Arg276Trp LP/P rs151173406 Congenital disorder of glycosylation 1K (CDG1K) [MIM:608540]
ALG1 Q9BT22 VAR_077195 p.Val281Phe LP/P rs553396382 Congenital disorder of glycosylation 1K (CDG1K) [MIM:608540]
ALG1 Q9BT22 VAR_077196 p.Asp289Gly LP/P rs1180515976 Congenital disorder of glycosylation 1K (CDG1K) [MIM:608540]
ALG1 Q9BT22 VAR_077197 p.Asp291Val LP/P rs192564717 Congenital disorder of glycosylation 1K (CDG1K) [MIM:608540]
ALG1 Q9BT22 VAR_077198 p.Tyr353Asp LP/P - Congenital disorder of glycosylation 1K (CDG1K) [MIM:608540]
ALG1 Q9BT22 VAR_077199 p.Gly358Arg LP/P rs886042742 Congenital disorder of glycosylation 1K (CDG1K) [MIM:608540]
ALG1 Q9BT22 VAR_077200 p.Ser359Leu LP/P rs1299775990 Congenital disorder of glycosylation 1K (CDG1K) [MIM:608540]
ALG1 Q9BT22 VAR_077201 p.Ala360Val LP/P rs398124348 Congenital disorder of glycosylation 1K (CDG1K) [MIM:608540]
ALG1 Q9BT22 VAR_077202 p.Gly363Ala LP/P rs1596261161 Congenital disorder of glycosylation 1K (CDG1K) [MIM:608540]
ALG1 Q9BT22 VAR_077203 p.Leu366Gln LP/P - Congenital disorder of glycosylation 1K (CDG1K) [MIM:608540]
ALG1 Q9BT22 VAR_077204 p.His367Gln LP/P - Congenital disorder of glycosylation 1K (CDG1K) [MIM:608540]
ALG1 Q9BT22 VAR_077205 p.Met382Lys LP/P - Congenital disorder of glycosylation 1K (CDG1K) [MIM:608540]
ALG1 Q9BT22 VAR_077206 p.Gly384Arg LP/P rs1057520122 Congenital disorder of glycosylation 1K (CDG1K) [MIM:608540]
ALG1 Q9BT22 VAR_077207 p.Pro388Ser LP/P rs398124349 Congenital disorder of glycosylation 1K (CDG1K) [MIM:608540]
ALG10B Q5I7T1 VAR_023753 p.Ile446Val LB/B rs61730283 -
ALG10B Q5I7T1 VAR_048217 p.Ala84Gly LB/B rs6582584 -
ALG10B Q5I7T1 VAR_061002 p.Ser383Asn LB/B rs57963306 -
ALG11 Q2TAA5 VAR_055902 p.Asn108Ser LB/B rs17480245 -
ALG11 Q2TAA5 VAR_064908 p.Leu86Ser LP/P rs267606652 Congenital disorder of glycosylation 1P (CDG1P) [MIM:613661]
ALG11 Q2TAA5 VAR_068070 p.Tyr279Ser LP/P rs387907181 Congenital disorder of glycosylation 1P (CDG1P) [MIM:613661]
ALG11 Q2TAA5 VAR_068071 p.Gln318Pro LP/P rs387907184 Congenital disorder of glycosylation 1P (CDG1P) [MIM:613661]
ALG11 Q2TAA5 VAR_068072 p.Leu381Ser LP/P rs387907182 Congenital disorder of glycosylation 1P (CDG1P) [MIM:613661]
ALG11 Q2TAA5 VAR_068073 p.Glu398Lys LP/P rs387907183 Congenital disorder of glycosylation 1P (CDG1P) [MIM:613661]

ALG12 Q9BV10 VAR_017904 p.Thr67Met LP/P rs121907931 Congenital disorder of glycosylation 1G (CDG1G) [MIM:607143]
ALG12 Q9BV10 VAR_017905 p.Phe142Val LP/P rs28942090 Congenital disorder of glycosylation 1G (CDG1G) [MIM:607143]
ALG12 Q9BV10 VAR_017906 p.Arg146Gln LP/P rs121907932 Congenital disorder of glycosylation 1G (CDG1G) [MIM:607143]
ALG12 Q9BV10 VAR_017907 p.Leu158Pro LP/P rs121907934 Congenital disorder of glycosylation 1G (CDG1G) [MIM:607143]
ALG12 Q9BV10 VAR_024466 p.Ile393Val LB/B rs3922872 -
ALG12 Q9BV10 VAR_038428 p.Gly101Arg LP/P rs121907933 Congenital disorder of glycosylation 1G (CDG1G) [MIM:607143]
ALG13 Q9NP73 VAR_069218 p.Lys94Glu LP/P rs867599353 Developmental and epileptic encephalopathy 36 (DEE36) [MIM:300884]
ALG13 Q9NP73 VAR_069412 p.Asn107Ser LP/P rs398122394 Developmental and epileptic encephalopathy 36 (DEE36) [MIM:300884]
ALG14 Q96F25 VAR_029635 p.Val14Met LB/B rs11165298 -
ALG14 Q96F25 VAR_073331 p.Pro65Leu LP/P rs730882050 Myasthenic syndrome, congenital, 15 (CMS15) [MIM:616227]
ALG1L Q6GMV1 VAR_039962 p.Asn135Asp LB/B rs3828357 -
ALG1L Q6GMV1 VAR_039963 p.Ile159Thr LB/B rs3811679 -
ALG1L Q6GMV1 VAR_039964 p.Gly184Ser LB/B rs3187686 -
ALG2 Q9H553 VAR_049351 p.Ser11Pro LB/B rs11545137 -
ALG2 Q9H553 VAR_049352 p.Val367Ala LB/B rs35626507 -
ALG2 Q9H553 VAR_073332 p.Val68Gly LP/P rs730882051 Myasthenic syndrome, congenital, 14 (CMS14) [MIM:616228]
ALG3 Q92685 VAR_010306 p.Gly118Asp LP/P rs28940588 Congenital disorder of glycosylation 1D (CDG1D) [MIM:601110]
ALG3 Q92685 VAR_037805 p.Ile107Val LB/B rs2233463 -
ALG3 Q92685 VAR_037806 p.Arg171Gln LP/P rs119103236 Congenital disorder of glycosylation 1D (CDG1D) [MIM:601110]
ALG6 Q9Y672 VAR_013442 p.Ser304Phe LB/B rs4630153 -
ALG6 Q9Y672 VAR_013443 p.Ala333Val LP/P rs121908443 Congenital disorder of glycosylation 1C (CDG1C) [MIM:603147]
ALG6 Q9Y672 VAR_013444 p.Ser478Pro LP/P rs121908444 Congenital disorder of glycosylation 1C (CDG1C) [MIM:603147]
ALG6 Q9Y672 VAR_022511 p.Tyr131His LP/P rs35383149 Congenital disorder of glycosylation 1C (CDG1C) [MIM:603147]
ALG6 Q9Y672 VAR_022512 p.Ser170Ile LP/P - Congenital disorder of glycosylation 1C (CDG1C) [MIM:603147]
ALG6 Q9Y672 VAR_022513 p.Gly227Glu LP/P rs372079206 Congenital disorder of glycosylation 1C (CDG1C) [MIM:603147]
ALG6 Q9Y672 VAR_022514 p.Ser308Arg LP/P - Congenital disorder of glycosylation 1C (CDG1C) [MIM:603147]
ALG6 Q9Y672 VAR_055493 p.Lys226Asn LB/B rs35604168 -
ALG8 Q9BVK2 VAR_023480 p.Thr47Pro LP/P rs121908293 Congenital disorder of glycosylation 1H (CDG1H) [MIM:608104]
ALG8 Q9BVK2 VAR_023481 p.Asn222Ser LB/B rs665278 -
ALG8 Q9BVK2 VAR_023482 p.Gly275Asp LP/P rs121908294 Congenital disorder of glycosylation 1H (CDG1H) [MIM:608104]

ALG8	Q9BVK2	VAR_031596	p.Ile439Thr	LB/B	rs17825668	-
ALG9	Q9H6U8	VAR_023410	p.Tyr287Cys	LP/P	rs121908023	Congenital disorder of glycosylation 1L (CDG1L) [MIM:608776]
ALG9	Q9H6U8	VAR_023411	p.Val289Ile	LB/B	rs10502151	-
ALG9	Q9H6U8	VAR_023412	p.Pro506Leu	LB/B	rs185149177	-
ALG9	Q9H6U8	VAR_023413	p.Glu523Lys	LP/P	rs121908022	Congenital disorder of glycosylation 1L (CDG1L) [MIM:608776]
ALG9	Q9H6U8	VAR_049221	p.Ala232Pro	LB/B	rs36111204	-
ALG9	Q9H6U8	VAR_049222	p.Ser255Leu	LB/B	rs17113312	-
ALG9	Q9H6U8	VAR_049223	p.Ile528Ser	LB/B	rs12575909	-
ALK	Q9UM73	VAR_031042	p.Ile1461Val	LB/B	rs1670283	-
ALK	Q9UM73	VAR_031043	p.Lys1491Arg	LB/B	rs1881420	-
ALK	Q9UM73	VAR_031044	p.Asp1529Glu	LB/B	rs1881421	-
ALK	Q9UM73	VAR_041477	p.Ser90Leu	LB/B	rs34617074	-
ALK	Q9UM73	VAR_041478	p.Val163Leu	LB/B	rs55697431	-
ALK	Q9UM73	VAR_041479	p.Glu296Gln	LB/B	rs56077855	-
ALK	Q9UM73	VAR_041480	p.Val476Ala	LB/B	rs35093491	-
ALK	Q9UM73	VAR_041481	p.Leu560Phe	US	-	A breast pleomorphic lobular carcinoma sample
ALK	Q9UM73	VAR_041482	p.Thr680Ile	LB/B	rs35228363	-
ALK	Q9UM73	VAR_041483	p.Ala704Thr	LB/B	rs34829159	-
ALK	Q9UM73	VAR_041484	p.Ala877Ser	US	rs746442213	An ovarian serous carcinoma sample
ALK	Q9UM73	VAR_041485	p.Thr1012Met	LB/B	rs35073634	-
ALK	Q9UM73	VAR_041486	p.Gly1121Asp	LB/B	rs55760835	-
ALK	Q9UM73	VAR_041487	p.Ala1274Thr	LB/B	rs45502292	-
ALK	Q9UM73	VAR_041488	p.Met1328Leu	LB/B	rs56160491	-
ALK	Q9UM73	VAR_041489	p.Lys1416Asn	LB/B	rs55782189	-
ALK	Q9UM73	VAR_041490	p.Glu1419Lys	LB/B	rs56181542	-
ALK	Q9UM73	VAR_041491	p.Gln1429Arg	LB/B	rs55906201	-
ALK	Q9UM73	VAR_055987	p.Phe1376Ser	LB/B	rs17694720	-
ALK	Q9UM73	VAR_055988	p.Pro1599His	LB/B	rs1881423	-
ALK	Q9UM73	VAR_061288	p.Leu868Gln	LB/B	rs55941323	-
ALK	Q9UM73	VAR_063850	p.Asp1091Asn	LP/P	rs864309584	Neuroblastoma 3 (NBLST3) [MIM:613014]
ALK	Q9UM73	VAR_063851	p.Gly1128Ala	LP/P	rs113994088	Neuroblastoma 3 (NBLST3) [MIM:613014]
ALK	Q9UM73	VAR_063852	p.Thr1151Met	LP/P	rs113994091	Neuroblastoma 3 (NBLST3) [MIM:613014]
ALK	Q9UM73	VAR_063853	p.Met1166Arg	LP/P	rs1057520019	Neuroblastoma 3 (NBLST3) [MIM:613014]
ALK	Q9UM73	VAR_063854	p.Ile1171Asn	LP/P	rs1057519698	Neuroblastoma 3 (NBLST3) [MIM:613014]
ALK	Q9UM73	VAR_063855	p.Phe1174Cys	LP/P	rs1057519697	Neuroblastoma 3 (NBLST3) [MIM:613014]
ALK	Q9UM73	VAR_063856	p.Phe1174Ile	LP/P	rs281864719	Neuroblastoma 3 (NBLST3) [MIM:613014]
ALK	Q9UM73	VAR_063857	p.Phe1174Leu	LP/P	rs863225281	Neuroblastoma 3 (NBLST3) [MIM:613014]
ALK	Q9UM73	VAR_063858	p.Phe1174Val	LP/P	rs281864719	Neuroblastoma 3 (NBLST3) [MIM:613014]
ALK	Q9UM73	VAR_063859	p.Arg1192Pro	LP/P	rs113994089	Neuroblastoma 3 (NBLST3) [MIM:613014]
ALK	Q9UM73	VAR_063860	p.Ala1234Thr	LP/P	-	Neuroblastoma 3 (NBLST3) [MIM:613014]
ALK	Q9UM73	VAR_063861	p.Phe1245Cys	LP/P	rs863225283	Neuroblastoma 3 (NBLST3) [MIM:613014]
ALK	Q9UM73	VAR_063862	p.Phe1245Val	LP/P	rs281864720	Neuroblastoma 3 (NBLST3) [MIM:613014]
ALK	Q9UM73	VAR_063863	p.Ile1250Thr	LP/P	rs113994092	Neuroblastoma 3 (NBLST3) [MIM:613014]
ALK	Q9UM73	VAR_063864	p.Arg1275Leu	LB/B	rs113994087	-
ALK	Q9UM73	VAR_063865	p.Arg1275Gln	LP/P	rs113994087	Neuroblastoma 3 (NBLST3) [MIM:613014]
ALK	Q9UM73	VAR_063866	p.Tyr1278Ser	LP/P	rs863225285	Neuroblastoma 3 (NBLST3) [MIM:613014]
ALKBH1	Q13686	VAR_048221	p.Met135Ile	LB/B	rs17825440	-

ALKBH1 Q13686 VAR_048222 p.Met324Leu LB/B rs6494 -
ALKBH2 Q6NS38 VAR_048223 p.Arg203His LB/B rs33962311 -
ALKBH3 Q96Q83 VAR_026631 p.Asp228Glu LB/B rs1130290 -
ALKBH3 Q96Q83 VAR_026632 p.Arg164Cys LB/B rs2271815 -
ALKBH4 Q9NXW9 VAR_061004 p.Ala247Val LB/B rs41275227 -
ALKBH7 Q9BT30 VAR_048224 p.Arg191Gln LB/B rs7540 -
ALMS1 Q8TCU4 VAR_025433 p.Val672Gly LB/B rs2037814 -
ALMS1 Q8TCU4 VAR_025434 p.Ser2112Arg LB/B rs6724782 -
ALMS1 Q8TCU4 VAR_025435 p.Ser2575Asn LB/B rs3820700 -
ALMS1 Q8TCU4 VAR_025436 p.Asp2673His LB/B rs2017116 -
ALMS1 Q8TCU4 VAR_056734 p.Gly1413Ala LB/B rs886038612 -
ALMS1 Q8TCU4 VAR_059575 p.Ile1876Val LB/B rs6546838 -
ALMS1 Q8TCU4 VAR_059576 p.Arg2285Pro LB/B rs6546839 -
ALMS1 Q8TCU4 VAR_059577 p.Asn2857Ser LB/B rs10193972 -
ALMS1 Q8TCU4 VAR_059578 p.Lys3435Glu LB/B rs34071195 -
ALOX12 P18054 VAR_004279 p.Ala298Thr LB/B - -
ALOX12 P18054 VAR_018743 p.Gln261Arg LB/B rs1126667 -
ALOX12 P18054 VAR_018744 p.Asn322Ser LB/B rs434473 -
ALOX12 P18054 VAR_018745 p.Arg430His LB/B rs11571342 -
ALOX12 P18054 VAR_030471 p.Glu259Lys LB/B rs4987104 -
ALOX12 P18054 VAR_082032 p.Asp134His LB/B rs114985038 -
ALOX12B O75342 VAR_015173 p.Leu426Pro LP/P rs137853023 Ichthyosis, congenital, autosomal recessive 2 (ARCI2) [MIM:242100]
ALOX12B O75342 VAR_015174 p.His578Gln LP/P rs137853024 Ichthyosis, congenital, autosomal recessive 2 (ARCI2) [MIM:242100]
ALOX12B O75342 VAR_050000 p.Gly94Ser LB/B rs8077661 -
ALOX12B O75342 VAR_069545 p.Leu24Pro LP/P rs201575829 Ichthyosis, congenital, autosomal recessive 2 (ARCI2) [MIM:242100]
ALOX12B O75342 VAR_069546 p.Ile67Phe LP/P rs397514533 Ichthyosis, congenital, autosomal recessive 2 (ARCI2) [MIM:242100]
ALOX12B O75342 VAR_069547 p.Arg114Trp LP/P rs397514526 Ichthyosis, congenital, autosomal recessive 2 (ARCI2) [MIM:242100]
ALOX12B O75342 VAR_069548 p.Pro127Ser LP/P rs72842957 Ichthyosis, congenital, autosomal recessive 2 (ARCI2) [MIM:242100]
ALOX12B O75342 VAR_069549 p.Phe195Leu LP/P rs200516538 Ichthyosis, congenital, autosomal recessive 2 (ARCI2) [MIM:242100]
ALOX12B O75342 VAR_069550 p.Tyr318Cys LP/P - Ichthyosis, congenital, autosomal recessive 2 (ARCI2) [MIM:242100]
ALOX12B O75342 VAR_069551 p.Lys382Glu LP/P rs1567981916 Ichthyosis, congenital, autosomal recessive 2 (ARCI2) [MIM:242100]
ALOX12B O75342 VAR_069552 p.Thr383Met LP/P rs760428119 Ichthyosis, congenital, autosomal recessive 2 (ARCI2) [MIM:242100]
ALOX12B O75342 VAR_069553 p.Asn416Lys LP/P rs1039399607 Ichthyosis, congenital, autosomal recessive 2 (ARCI2) [MIM:242100]
ALOX12B O75342 VAR_069554 p.Gly462Asp LP/P rs774958790 Ichthyosis, congenital, autosomal recessive 2 (ARCI2) [MIM:242100]
ALOX12B O75342 VAR_069555 p.Arg488His LP/P rs763468558 Ichthyosis, congenital, autosomal recessive 2 (ARCI2) [MIM:242100]

ALOX12B O75342 VAR_069556 p.Tyr521Cys LP/P rs199766569 Ichthyosis, congenital, autosomal recessive 2 (ARCI2) [MIM:242100]
ALOX12B O75342 VAR_069557 p.Val527Met LP/P rs199545653 Ichthyosis, congenital, autosomal recessive 2 (ARCI2) [MIM:242100]
ALOX12B O75342 VAR_069558 p.Ala597Glu LP/P rs752509098 Ichthyosis, congenital, autosomal recessive 2 (ARCI2) [MIM:242100]
ALOX12B O75342 VAR_069559 p.Ala664Pro LP/P - Ichthyosis, congenital, autosomal recessive 2 (ARCI2) [MIM:242100]
ALOX12B O75342 VAR_069560 p.Arg679Leu LP/P rs397514528 Ichthyosis, congenital, autosomal recessive 2 (ARCI2) [MIM:242100]
ALOX15 P16050 VAR_018746 p.Asp90His LB/B rs11568142 -
ALOX15 P16050 VAR_018747 p.Asn103Lys LB/B rs11568099 -
ALOX15 P16050 VAR_018748 p.Arg205Gln LB/B rs11568101 -
ALOX15 P16050 VAR_035036 p.Gly102Val LB/B rs41439950 -
ALOX15 P16050 VAR_035037 p.Val239Met LB/B rs3892408 -
ALOX15 P16050 VAR_035038 p.Ala461Pro LB/B rs17852628 -
ALOX15 P16050 VAR_035039 p.Thr560Met LB/B rs34210653 -
ALOX15 P16050 VAR_083449 p.Arg402Trp US - -
ALOX15 P16050 VAR_083450 p.Gly422Glu US - -
ALOX15 P16050 VAR_083451 p.Gly422Arg US - -
ALOX15 P16050 VAR_083452 p.Pro617Ser US - -
ALOX15B O15296 VAR_024524 p.Gln656Arg LB/B rs4792147 -
ALOX15B O15296 VAR_024525 p.Ile676Val LB/B rs7225107 -
ALOX15B O15296 VAR_061334 p.Arg486His LB/B rs9895916 -
ALOX15B O15296 VAR_083544 p.Ala416Asp LB/B rs140152561 -
ALOX5 P09917 VAR_028018 p.Glu254Lys LB/B rs2228065 -
ALOX5 P09917 VAR_083301 p.Pro337Ser US - -
ALOX5 P09917 VAR_083302 p.Ala447Ser US - -
ALOX5 P09917 VAR_083303 p.Ala549Val US - -
ALOX5 P09917 VAR_083304 p.Pro577Leu US - -
ALOX5 P09917 VAR_083305 p.Thr591Met US - -
ALOX5 P09917 VAR_083306 p.Lys656Gln US - -
ALOXE3 Q9BYJ1 VAR_015175 p.Arg396Ser LP/P rs121434234 Ichthyosis, congenital, autosomal recessive 3 (ARCI3) [MIM:606545]
ALOXE3 Q9BYJ1 VAR_015176 p.Val500Phe LP/P rs121434232 Ichthyosis, congenital, autosomal recessive 3 (ARCI3) [MIM:606545]
ALOXE3 Q9BYJ1 VAR_069561 p.Leu237Met LP/P rs121434235 Ichthyosis, congenital, autosomal recessive 3 (ARCI3) [MIM:606545]
ALOXE3 Q9BYJ1 VAR_069562 p.Gly281Val LP/P rs786205120 Ichthyosis, congenital, autosomal recessive 3 (ARCI3) [MIM:606545]
ALOXE3 Q9BYJ1 VAR_069564 p.Leu427Pro LP/P rs1355284797 Ichthyosis, congenital, autosomal recessive 3 (ARCI3) [MIM:606545]
ALOXE3 Q9BYJ1 VAR_069565 p.Pro630Leu LP/P rs147149459 Ichthyosis, congenital, autosomal recessive 3 (ARCI3) [MIM:606545]
ALPG P10696 VAR_027553 p.Leu273Met LB/B rs17416141 -
ALPG P10696 VAR_027554 p.Leu316Arg LB/B rs183793479 -
ALPG P10696 VAR_027555 p.Gly527Glu LB/B rs1048999 -
ALPI P09923 VAR_011816 p.His298Leu LB/B rs1047223 -
ALPI P09923 VAR_050524 p.Arg144His LB/B rs7559279 -

ALPK1	Q96QP1	VAR_028982	p.Asn175Asp	LB/B	rs6533616	-
ALPK1	Q96QP1	VAR_028983	p.Gly565Asp	LB/B	rs2074388	-
ALPK1	Q96QP1	VAR_028984	p.His642Arg	LB/B	rs13148353	-
ALPK1	Q96QP1	VAR_028985	p.Met732Ile	LB/B	rs2074379	-
ALPK1	Q96QP1	VAR_028986	p.Met861Thr	LB/B	rs11726117	-
ALPK1	Q96QP1	VAR_028987	p.Gly870Ser	LB/B	rs2074380	-
ALPK1	Q96QP1	VAR_028988	p.Asn916Asp	LB/B	rs2074381	-
ALPK1	Q96QP1	VAR_041511	p.Gln67Arg	LB/B	rs33943680	-
ALPK1	Q96QP1	VAR_041512	p.Thr292Met	LB/B	rs34120296	-
ALPK1	Q96QP1	VAR_041513	p.Leu320Met	LB/B	rs757602009	-
ALPK1	Q96QP1	VAR_041514	p.Lys339Glu	US	-	An ovarian mucinous carcinoma sample
ALPK1	Q96QP1	VAR_041515	p.Lys383Glu	LB/B	rs147641444	-
ALPK1	Q96QP1	VAR_041516	p.Pro660Leu	LB/B	rs35389530	-
ALPK1	Q96QP1	VAR_041517	p.Gly681Asp	LB/B	rs35519493	-
ALPK1	Q96QP1	VAR_041518	p.Arg873Ile	LB/B	rs34946272	-
ALPK1	Q96QP1	VAR_041519	p.Glu910Asp	LB/B	rs35308602	-
ALPK1	Q96QP1	VAR_041520	p.Pro935Leu	LB/B	rs34780600	-
ALPK1	Q96QP1	VAR_041521	p.Arg1084Gln	LB/B	rs34677416	-
ALPK1	Q96QP1	VAR_041522	p.Leu1117Pro	LB/B	rs35756863	-
ALPK1	Q96QP1	VAR_041523	p.Ala1160Gly	LB/B	rs55696324	-
ALPK1	Q96QP1	VAR_057741	p.His1008Pro	LB/B	rs34079946	-
ALPK2	Q86TB3	VAR_045591	p.Glu942Lys	US	-	An ovarian undifferentiated carcinoma sample
ALPK2	Q86TB3	VAR_045593	p.Leu1296Val	LB/B	rs3809976	-
ALPK2	Q86TB3	VAR_045594	p.Lys1476Thr	US	-	A melanoma metastatic sample
ALPK2	Q86TB3	VAR_045595	p.Glu1969Lys	LB/B	rs1313881443	-
ALPK2	Q86TB3	VAR_054914	p.Lys2Thr	LB/B	rs6566987	-
ALPK2	Q86TB3	VAR_054915	p.Arg136Ser	LB/B	rs9944810	-
ALPK2	Q86TB3	VAR_054916	p.His719Gln	LB/B	rs12103986	-
ALPK2	Q86TB3	VAR_054917	p.Gly810Ser	LB/B	rs3809970	-
ALPK2	Q86TB3	VAR_054918	p.Arg825Thr	LB/B	rs3809972	-
ALPK2	Q86TB3	VAR_054919	p.Lys829Asn	LB/B	rs3809973	-
ALPK2	Q86TB3	VAR_054920	p.Ser884Leu	LB/B	rs3809974	-
ALPK2	Q86TB3	VAR_054921	p.Thr891Ile	LB/B	rs3826593	-
ALPK2	Q86TB3	VAR_054922	p.Asn916Lys	LB/B	rs4940404	-
ALPK2	Q86TB3	VAR_054923	p.Ser977Thr	LB/B	rs3809975	-
ALPK2	Q86TB3	VAR_054924	p.Leu1057Val	LB/B	rs3809976	-
ALPK2	Q86TB3	VAR_054925	p.Lys1134Asn	LB/B	rs35791514	-
ALPK2	Q86TB3	VAR_054926	p.His1174Pro	LB/B	rs3809977	-
ALPK2	Q86TB3	VAR_054927	p.Pro1449Ser	LB/B	rs3809982	-
ALPK2	Q86TB3	VAR_054928	p.Ala1551Ser	LB/B	rs3809983	-
ALPK2	Q86TB3	VAR_054929	p.Gln1579Arg	LB/B	rs33910491	-
ALPK2	Q86TB3	VAR_054930	p.Lys1729Glu	LB/B	rs34409558	-
ALPK2	Q86TB3	VAR_054931	p.Lys1730Glu	LB/B	rs17065127	-
ALPK2	Q86TB3	VAR_054932	p.His1767Tyr	LB/B	rs7234999	-
ALPK2	Q86TB3	VAR_054933	p.Ile2157Val	LB/B	rs7240666	-
ALPK2	Q86TB3	VAR_057742	p.Arg1884Cys	LB/B	rs33969768	-
ALPK2	Q86TB3	VAR_062168	p.Gly1063Val	LB/B	rs34347938	-
ALPK2	Q86TB3	VAR_082163	p.Ala29Val	LB/B	rs138405027	-
ALPK2	Q86TB3	VAR_082164	p.Ala202Thr	LB/B	rs115979836	-

ALPK2	Q86TB3	VAR_082165	p.Ser388Leu	LB/B	rs147887741	-
ALPK2	Q86TB3	VAR_082166	p.Thr397Ile	LB/B	rs79863383	-
ALPK2	Q86TB3	VAR_082167	p.Ala457Val	LB/B	rs199872766	-
ALPK2	Q86TB3	VAR_082168	p.Thr875Pro	LB/B	rs34109891	-
ALPK2	Q86TB3	VAR_082169	p.Ala969Thr	LB/B	rs371835741	-
ALPK2	Q86TB3	VAR_082170	p.Leu1288Ser	LB/B	rs35882005	-
ALPK2	Q86TB3	VAR_082171	p.Lys1745Asn	LB/B	rs56206581	-
ALPK2	Q86TB3	VAR_082172	p.Gln1853Glu	LB/B	rs55674018	-
ALPK2	Q86TB3	VAR_082173	p.Ala1919Thr	LB/B	rs374271622	-
ALPK2	Q86TB3	VAR_082174	p.Ala1978Thr	LB/B	rs146618330	-
ALPK3	Q96L96	VAR_028989	p.Thr414Ser	LB/B	rs3803403	-
ALPK3	Q96L96	VAR_028990	p.Gly579Glu	LB/B	rs3803405	-
ALPK3	Q96L96	VAR_028991	p.Thr761Met	LB/B	rs16974569	-
ALPK3	Q96L96	VAR_028992	p.Pro1299Leu	LB/B	rs306197	-
ALPK3	Q96L96	VAR_028993	p.Leu1622Pro	LB/B	rs187316	-
ALPK3	Q96L96	VAR_041524	p.Arg336His	LB/B	rs34407151	-
ALPK3	Q96L96	VAR_041525	p.Thr338Ile	LB/B	rs56015306	-
ALPK3	Q96L96	VAR_041526	p.Gln433Glu	US	-	A lung large cell carcinoma sample
ALPK3	Q96L96	VAR_041527	p.Gln602Arg	LB/B	rs55702300	-
ALPK3	Q96L96	VAR_041528	p.Gly663Asp	LB/B	rs34409363	-
ALPK3	Q96L96	VAR_041529	p.Arg836Leu	LB/B	rs34906636	-
ALPK3	Q96L96	VAR_041530	p.Glu929Asp	LB/B	rs56191073	-
ALPK3	Q96L96	VAR_041531	p.Gly1364Glu	US	-	A metastatic melanoma sample
ALPK3	Q96L96	VAR_041532	p.Arg1412Trp	LB/B	rs55752937	-
ALPK3	Q96L96	VAR_041533	p.Ala1557Asp	LB/B	rs34775428	-
ALPK3	Q96L96	VAR_057743	p.Glu1013Lys	LB/B	rs35633849	-
ALPK3	Q96L96	VAR_057744	p.Ala1137Gly	LB/B	rs34173528	-
ALPK3	Q96L96	VAR_057745	p.Ala1873Val	LB/B	rs36002219	-
ALPL	P05186	VAR_006147	p.Ala33Val	LP/P	rs121918005	Hypophosphatasia (HOPS) [MIM:146300]
ALPL	P05186	VAR_006148	p.Met62Leu	LP/P	-	Hypophosphatasia (HOPS) [MIM:146300]
ALPL	P05186	VAR_006149	p.Arg71Cys	LP/P	rs121918001	Hypophosphatasia (HOPS) [MIM:146300]
ALPL	P05186	VAR_006150	p.Arg71Pro	LP/P	rs121918003	Hypophosphatasia (HOPS) [MIM:146300]
ALPL	P05186	VAR_006151	p.Ala111Thr	LP/P	rs773257111	Hypophosphatasia (HOPS) [MIM:146300]
ALPL	P05186	VAR_006152	p.Arg136His	LP/P	rs121918011	Hypophosphatasia (HOPS) [MIM:146300]
ALPL	P05186	VAR_006153	p.Gly162Val	LP/P	rs121918012	Hypophosphatasia (HOPS) [MIM:146300]
ALPL	P05186	VAR_006154	p.His171Tyr	LP/P	-	Hypophosphatasia (HOPS) [MIM:146300]
ALPL	P05186	VAR_006155	p.Ala177Thr	LP/P	rs199669988	Hypophosphatasia (HOPS) [MIM:146300]
ALPL	P05186	VAR_006155	p.Ala177Thr	LP/P	rs199669988	Hypophosphatasia childhood type (HOPSC) [MIM:241510]
ALPL	P05186	VAR_006156	p.Ala179Thr	LP/P	rs121918000	Hypophosphatasia (HOPS) [MIM:146300]
ALPL	P05186	VAR_006157	p.Glu191Gly	LP/P	-	Hypophosphatasia (HOPS) [MIM:146300]
ALPL	P05186	VAR_006158	p.Glu191Lys	LP/P	rs121918007	Hypophosphatasia (HOPS) [MIM:146300]
ALPL	P05186	VAR_006159	p.Cys201Tyr	LP/P	-	Hypophosphatasia (HOPS) [MIM:146300]
ALPL	P05186	VAR_006160	p.Gln207Pro	LP/P	rs121918004	Hypophosphatasia (HOPS) [MIM:146300]
ALPL	P05186	VAR_006161	p.Tyr263His	LB/B	rs3200254	-
ALPL	P05186	VAR_006162	p.Leu289Phe	LP/P	-	Hypophosphatasia (HOPS) [MIM:146300]
ALPL	P05186	VAR_006163	p.Asp294Ala	LP/P	rs121918002	Hypophosphatasia (HOPS) [MIM:146300]
ALPL	P05186	VAR_006164	p.Asp306Val	LP/P	-	Hypophosphatasia (HOPS) [MIM:146300]
ALPL	P05186	VAR_006165	p.Phe327Leu	LP/P	rs121918010	Hypophosphatasia (HOPS) [MIM:146300]

ALPL	P05186	VAR_006165	p.Phe327Leu	LP/P	rs121918010	Hypophosphatasia infantile type (HOPSI) [MIM:241500]
ALPL	P05186	VAR_006166	p.Gly334Asp	LP/P	rs121918009	Hypophosphatasia (HOPS) [MIM:146300]
ALPL	P05186	VAR_006167	p.Asp378Val	LP/P	rs121918008	Hypophosphatasia (HOPS) [MIM:146300]
ALPL	P05186	VAR_006168	p.Val382Ile	LP/P	rs771540767	Hypophosphatasia (HOPS) [MIM:146300]
ALPL	P05186	VAR_006169	p.Tyr436His	LP/P	rs121918006	Hypophosphatasia (HOPS) [MIM:146300]
ALPL	P05186	VAR_006170	p.Glu476Lys	LP/P	-	Hypophosphatasia (HOPS) [MIM:146300]
ALPL	P05186	VAR_011081	p.Ala40Val	LP/P	rs770093969	Hypophosphatasia (HOPS) [MIM:146300]
ALPL	P05186	VAR_011082	p.Thr134Asn	LP/P	rs780583917	Hypophosphatasia (HOPS) [MIM:146300]
ALPL	P05186	VAR_011083	p.Ala176Thr	LP/P	rs121918019	Hypophosphatasia (HOPS) [MIM:146300]
ALPL	P05186	VAR_011084	p.Lys224Glu	LP/P	rs1226800998	Hypophosphatasia infantile type (HOPSI) [MIM:241500]
ALPL	P05186	VAR_011085	p.Arg246Ser	LP/P	rs1223142821	Hypophosphatasia (HOPS) [MIM:146300]
ALPL	P05186	VAR_011086	p.Ala348Thr	LP/P	rs1553414563	Hypophosphatasia (HOPS) [MIM:146300]
ALPL	P05186	VAR_011087	p.His381Arg	LP/P	-	Hypophosphatasia (HOPS) [MIM:146300]
ALPL	P05186	VAR_011088	p.Asp406Gly	LP/P	-	Hypophosphatasia (HOPS) [MIM:146300]
ALPL	P05186	VAR_011089	p.Gly426Cys	LP/P	-	Hypophosphatasia infantile type (HOPSI) [MIM:241500]
ALPL	P05186	VAR_011090	p.Arg450His	LP/P	rs150799088	Hypophosphatasia (HOPS) [MIM:146300]
ALPL	P05186	VAR_011091	p.Gly456Arg	LP/P	rs121918016	Hypophosphatasia infantile type (HOPSI) [MIM:241500]
ALPL	P05186	VAR_011092	p.Asn478Ile	LP/P	-	Hypophosphatasia (HOPS) [MIM:146300]
ALPL	P05186	VAR_011093	p.Cys489Ser	LP/P	-	Hypophosphatasia (HOPS) [MIM:146300]
ALPL	P05186	VAR_011094	p.Val522Ala	LB/B	rs34605986	-
ALPL	P05186	VAR_013146	p.Ala132Val	LP/P	-	Hypophosphatasia (HOPS) [MIM:146300]
ALPL	P05186	VAR_013972	p.Tyr28Cys	LP/P	-	Hypophosphatasia infantile type (HOPSI) [MIM:241500]
ALPL	P05186	VAR_013973	p.Ala51Val	LP/P	rs1470389268	Hypophosphatasia (HOPS) [MIM:146300]
ALPL	P05186	VAR_013974	p.Gly63Val	LP/P	-	Hypophosphatasia (HOPS) [MIM:146300]
ALPL	P05186	VAR_013975	p.Arg71His	LP/P	rs121918003	Hypophosphatasia (HOPS) [MIM:146300]
ALPL	P05186	VAR_013976	p.Gly75Ser	LP/P	rs1304394441	Hypophosphatasia (HOPS) [MIM:146300]
ALPL	P05186	VAR_013977	p.Ala116Thr	LP/P	rs121918013	Hypophosphatasia (HOPS) [MIM:146300]
ALPL	P05186	VAR_013978	p.Gly120Arg	LP/P	rs954135116	Hypophosphatasia (HOPS) [MIM:146300]
ALPL	P05186	VAR_013979	p.Gly129Arg	LP/P	-	Hypophosphatasia (HOPS) [MIM:146300]
ALPL	P05186	VAR_013980	p.Arg152His	LB/B	rs149344982	Hypophosphatasia (HOPS) [MIM:146300]
ALPL	P05186	VAR_013981	p.Asn170Asp	LP/P	-	Hypophosphatasia (HOPS) [MIM:146300]
ALPL	P05186	VAR_013982	p.Ser181Leu	LP/P	rs199590449	Hypophosphatasia (HOPS) [MIM:146300]
ALPL	P05186	VAR_013983	p.Arg184Trp	LP/P	rs763159520	Hypophosphatasia (HOPS) [MIM:146300]
ALPL	P05186	VAR_013984	p.Asn211Asp	LP/P	-	Hypophosphatasia (HOPS) [MIM:146300]
ALPL	P05186	VAR_013985	p.Gly220Val	LP/P	-	Hypophosphatasia (HOPS) [MIM:146300]
ALPL	P05186	VAR_013986	p.Arg223Trp	LP/P	rs766076920	Hypophosphatasia (HOPS) [MIM:146300]
ALPL	P05186	VAR_013986	p.Arg223Trp	LP/P	rs766076920	Hypophosphatasia childhood type (HOPSC) [MIM:241510]
ALPL	P05186	VAR_013987	p.Glu235Gly	LP/P	-	Hypophosphatasia (HOPS) [MIM:146300]
ALPL	P05186	VAR_013988	p.Gly249Val	LP/P	rs121918018	Hypophosphatasia (HOPS) [MIM:146300]
ALPL	P05186	VAR_013989	p.Glu291Lys	LP/P	rs786204473	Hypophosphatasia (HOPS) [MIM:146300]
ALPL	P05186	VAR_013990	p.Asp294Tyr	LP/P	rs1553414079	Hypophosphatasia (HOPS) [MIM:146300]
ALPL	P05186	VAR_013991	p.Gly326Arg	LP/P	-	Hypophosphatasia (HOPS) [MIM:146300]
ALPL	P05186	VAR_013992	p.Phe327Gly	LP/P	-	Hypophosphatasia (HOPS) [MIM:146300]
ALPL	P05186	VAR_013993	p.Arg391Cys	LP/P	rs371243939	Hypophosphatasia (HOPS) [MIM:146300]
ALPL	P05186	VAR_013994	p.Ala399Ser	LP/P	-	Hypophosphatasia (HOPS) [MIM:146300]

ALPL	P05186	VAR_013995	p.Val423Ala	LP/P	-	Hypophosphatasia (HOPS) [MIM:146300]
ALPL	P05186	VAR_013996	p.Ser445Pro	LP/P	rs1553415041	Hypophosphatasia (HOPS) [MIM:146300]
ALPL	P05186	VAR_013997	p.Arg450Cys	LP/P	rs138690664	Hypophosphatasia (HOPS) [MIM:146300]
ALPL	P05186	VAR_013998	p.Val459Met	LP/P	rs1054159992	Hypophosphatasia infantile type (HOPSI) [MIM:241500]
ALPL	P05186	VAR_013999	p.Gly473Ser	LP/P	-	Hypophosphatasia (HOPS) [MIM:146300]
ALPL	P05186	VAR_014000	p.Ile490Phe	LP/P	-	Hypophosphatasia (HOPS) [MIM:146300]
ALPL	P05186	VAR_014001	p.Gly491Arg	LP/P	rs1413274209	Hypophosphatasia (HOPS) [MIM:146300]
ALPL	P05186	VAR_025903	p.Ser17Phe	LP/P	-	Hypophosphatasia (HOPS) [MIM:146300]
ALPL	P05186	VAR_025904	p.Ala51Ser	LP/P	-	Hypophosphatasia (HOPS) [MIM:146300]
ALPL	P05186	VAR_025905	p.Met62Val	LP/P	-	Hypophosphatasia (HOPS) [MIM:146300]
ALPL	P05186	VAR_025906	p.Gly63Arg	LP/P	-	Hypophosphatasia (HOPS) [MIM:146300]
ALPL	P05186	VAR_025907	p.Thr68Met	LP/P	-	Hypophosphatasia childhood type (HOPSC) [MIM:241510]
ALPL	P05186	VAR_025908	p.Arg71Ser	LP/P	rs121918001	Hypophosphatasia childhood type (HOPSC) [MIM:241510]
ALPL	P05186	VAR_025909	p.Gln76Arg	LP/P	rs1057521085	Hypophosphatasia (HOPS) [MIM:146300]
ALPL	P05186	VAR_025910	p.Pro108Leu	LP/P	rs121918015	Hypophosphatasia (HOPS) [MIM:146300]
ALPL	P05186	VAR_025911	p.Ala114Gly	LP/P	-	Hypophosphatasia (HOPS) [MIM:146300]
ALPL	P05186	VAR_025912	p.Val128Met	LP/P	rs1159854007	Hypophosphatasia (HOPS) [MIM:146300]
ALPL	P05186	VAR_025913	p.Thr134His	LP/P	rs786204530	Hypophosphatasia (HOPS) [MIM:146300]
ALPL	P05186	VAR_025914	p.Thr148Ile	LP/P	rs1376937780	Hypophosphatasia (HOPS) [MIM:146300]
ALPL	P05186	VAR_025915	p.Gly162Ser	LP/P	rs760029254	Hypophosphatasia (HOPS) [MIM:146300]
ALPL	P05186	VAR_025916	p.His171Arg	LP/P	rs778232217	Hypophosphatasia (HOPS) [MIM:146300]
ALPL	P05186	VAR_025917	p.Asp189Glu	LP/P	-	Hypophosphatasia (HOPS) [MIM:146300]
ALPL	P05186	VAR_025918	p.Ile212Phe	LP/P	-	Hypophosphatasia (HOPS) [MIM:146300]
ALPL	P05186	VAR_025919	p.Gly220Ala	LP/P	-	Hypophosphatasia (HOPS) [MIM:146300]
ALPL	P05186	VAR_025920	p.Arg223Gln	LP/P	rs199665722	Hypophosphatasia (HOPS) [MIM:146300]
ALPL	P05186	VAR_025921	p.Arg272His	LP/P	rs781272386	Hypophosphatasia (HOPS) [MIM:146300]
ALPL	P05186	VAR_025922	p.Arg272Leu	LP/P	rs781272386	Hypophosphatasia (HOPS) [MIM:146300]
ALPL	P05186	VAR_025923	p.Leu275Pro	LP/P	rs1237252052	Hypophosphatasia childhood type (HOPSC) [MIM:241510]
ALPL	P05186	VAR_025924	p.Pro292Thr	LP/P	rs765458125	Hypophosphatasia (HOPS) [MIM:146300]
ALPL	P05186	VAR_025926	p.Met295Thr	LP/P	rs1220125702	Hypophosphatasia (HOPS) [MIM:146300]
ALPL	P05186	VAR_025927	p.Tyr297Asp	LP/P	-	Hypophosphatasia (HOPS) [MIM:146300]
ALPL	P05186	VAR_025928	p.Glu298Lys	LP/P	rs121918017	Hypophosphatasia infantile type (HOPSI) [MIM:241500]
ALPL	P05186	VAR_025929	p.Leu299Pro	LP/P	-	Hypophosphatasia (HOPS) [MIM:146300]
ALPL	P05186	VAR_025930	p.Glu311Lys	LP/P	rs763457259	Hypophosphatasia (HOPS) [MIM:146300]
ALPL	P05186	VAR_025932	p.Gly339Arg	LP/P	-	Hypophosphatasia (HOPS) [MIM:146300]
ALPL	P05186	VAR_025933	p.Glu354Asp	LP/P	rs1553414568	Hypophosphatasia (HOPS) [MIM:146300]
ALPL	P05186	VAR_025934	p.Arg391His	LP/P	rs1442918125	Hypophosphatasia (HOPS) [MIM:146300]
ALPL	P05186	VAR_025934	p.Arg391His	LP/P	rs1442918125	Hypophosphatasia childhood type (HOPSC) [MIM:241510]
ALPL	P05186	VAR_025935	p.Thr411Ala	LP/P	-	Hypophosphatasia (HOPS) [MIM:146300]
ALPL	P05186	VAR_025936	p.Leu414Met	LP/P	-	Hypophosphatasia (HOPS) [MIM:146300]
ALPL	P05186	VAR_025937	p.Asn417Ser	LP/P	rs121918014	Hypophosphatasia (HOPS) [MIM:146300]
ALPL	P05186	VAR_025938	p.Gly426Asp	LP/P	-	Hypophosphatasia (HOPS) [MIM:146300]
ALPL	P05186	VAR_025939	p.Glu452Lys	LP/P	rs966212736	Hypophosphatasia (HOPS) [MIM:146300]

ALPL	P05186	VAR_025940	p.Ala468Thr	LP/P	rs1196976671	Hypophosphatasia (HOPS) [MIM:146300]
ALPL	P05186	VAR_075557	p.Gly334Arg	LP/P	-	Hypophosphatasia (HOPS) [MIM:146300]
ALPL	P05186	VAR_075558	p.Gly420Ala	LP/P	-	Hypophosphatasia (HOPS) [MIM:146300]
ALPL	P05186	VAR_075559	p.Gly420Ser	LP/P	-	Hypophosphatasia (HOPS) [MIM:146300]
ALPL	P05186	VAR_075560	p.Val459Leu	LP/P	-	Hypophosphatasia (HOPS) [MIM:146300]
ALPL	P05186	VAR_075561	p.Glu476Ala	LP/P	-	Hypophosphatasia (HOPS) [MIM:146300]
ALPP	P05187	VAR_017419	p.Pro25Leu	LB/B	rs1130335	-
ALPP	P05187	VAR_050520	p.Ile89Leu	LB/B	rs13026692	-
ALPP	P05187	VAR_050521	p.Arg231Pro	LB/B	rs1048988	-
ALPP	P05187	VAR_050522	p.Arg263His	LB/B	rs2853378	-
ALPP	P05187	VAR_050523	p.Glu451Gly	LB/B	rs1048994	-
ALS2	Q96Q42	VAR_015655	p.His102Arg	LB/B	rs1416065347	-
ALS2	Q96Q42	VAR_015656	p.Val368Met	LB/B	rs3219156	-
ALS2	Q96Q42	VAR_015657	p.Arg1406Lys	LB/B	rs1559033861	-
ALS2	Q96Q42	VAR_036747	p.Ile94Val	LB/B	rs3219154	-
ALS2	Q96Q42	VAR_036748	p.Glu159Lys	LB/B	rs3219155	-
ALS2	Q96Q42	VAR_036749	p.Ser1255Phe	LB/B	rs10206276	-
ALS2CL	Q60I27	VAR_037791	p.Glu45Gln	LB/B	rs7642448	-
ALS2CL	Q60I27	VAR_037792	p.Gln280Glu	US	-	A breast cancer sample
ALS2CL	Q60I27	VAR_037793	p.Leu576Phe	US	-	A breast cancer sample
ALS2CL	Q60I27	VAR_061554	p.Gln29Arg	LB/B	rs59661801	-
ALX3	O95076	VAR_047475	p.Pro234Ala	LB/B	rs12749726	-
ALX3	O95076	VAR_063226	p.Leu168Val	LP/P	rs121908167	Frontonasal dysplasia 1 (FND1) [MIM:136760]
ALX3	O95076	VAR_063227	p.Arg183Trp	LP/P	rs121908168	Frontonasal dysplasia 1 (FND1) [MIM:136760]
ALX3	O95076	VAR_063228	p.Arg196Trp	LP/P	rs121908170	Frontonasal dysplasia 1 (FND1) [MIM:136760]
ALX3	O95076	VAR_063229	p.Asn203Ser	LP/P	rs121908166	Frontonasal dysplasia 1 (FND1) [MIM:136760]
ALX4	Q9H161	VAR_010783	p.Arg35Thr	LB/B	rs3824915	-
ALX4	Q9H161	VAR_010784	p.Pro102Ser	LB/B	rs12421995	-
ALX4	Q9H161	VAR_010785	p.Arg218Gln	LP/P	rs104894193	Parietal foramina 2 (PFM2) [MIM:609597]
ALX4	Q9H161	VAR_010897	p.Arg272Pro	LP/P	rs104894196	Parietal foramina 2 (PFM2) [MIM:609597]
ALX4	Q9H161	VAR_058413	p.Arg257Thr	LB/B	rs3824915	-
ALX4	Q9H161	VAR_069279	p.Val7Phe	LP/P	rs281865153	Craniosynostosis 5 (CRS5) [MIM:615529]
ALX4	Q9H161	VAR_069280	p.Lys211Glu	LP/P	rs281865154	Craniosynostosis 5 (CRS5) [MIM:615529]
ALX4	Q9H161	VAR_069281	p.Pro306Leu	LB/B	rs149897209	-
AMACR	Q9UHK6	VAR_010660	p.Val9Met	LB/B	rs3195676	-
AMACR	Q9UHK6	VAR_010661	p.Ser52Pro	LP/P	rs121917814	Alpha-methylacyl-CoA racemase deficiency (AMACRD) [MIM:614307]
AMACR	Q9UHK6	VAR_010661	p.Ser52Pro	LP/P	rs121917814	Congenital bile acid synthesis defect 4 (CBAS4) [MIM:214950]
AMACR	Q9UHK6	VAR_010662	p.Gly175Asp	LB/B	rs10941112	-
AMACR	Q9UHK6	VAR_010663	p.Leu201Ser	LB/B	rs2287939	-
AMACR	Q9UHK6	VAR_010664	p.Glu277Lys	LB/B	rs2278008	-
AMACR	Q9UHK6	VAR_010665	p.Leu107Pro	LP/P	rs121917816	Congenital bile acid synthesis defect 4 (CBAS4) [MIM:214950]
AMACR	Q9UHK6	VAR_055616	p.Arg118Gln	LB/B	rs16892150	-
AMACR	Q9UHK6	VAR_055617	p.Pro238Ser	LB/B	rs9282594	-
AMACR	Q9UHK6	VAR_055618	p.Gln239His	LB/B	rs34677	-
AMACR	Q9UHK6	VAR_055619	p.Met261Ile	LB/B	rs9282593	-
AMACR	Q9UHK6	VAR_055620	p.Met261Thr	LB/B	rs3195678	-

AMBN Q9NP70 VAR_014066 p.Met11Thr US - -
AMBN Q9NP70 VAR_014067 p.Leu354Pro LB/B rs72654387 -
AMBN Q9NP70 VAR_014069 p.His439Arg LB/B rs375426598 -
AMBN Q9NP70 VAR_048225 p.Ala255Val LB/B rs7439186 -
AMDHD1 Q96NU7 VAR_031419 p.Ser3Gly LB/B rs7955450 -
AMDHD1 Q96NU7 VAR_031420 p.Pro360His LB/B rs17024904 -
AMDHD2 Q9Y303 VAR_038301 p.Asp294Asn US rs1220386463 A colorectal cancer sample
AMELX Q99217 VAR_037581 p.Trp4Ser LP/P rs104894738 Amelogenesis imperfecta 1E (AI1E) [MIM:301200]
AMELX Q99217 VAR_037582 p.Thr37Ile LP/P rs104894733 Amelogenesis imperfecta 1E (AI1E) [MIM:301200]
AMELX Q99217 VAR_037583 p.Pro56Thr LP/P rs104894736 Amelogenesis imperfecta 1E (AI1E) [MIM:301200]
AMER1 Q5JTC6 VAR_031304 p.Lys292Asn LB/B rs138948924 -
AMER1 Q5JTC6 VAR_053870 p.Phe159Leu LB/B rs34677493 -
AMER1 Q5JTC6 VAR_053871 p.Ala278Ser LB/B rs35718712 -
AMER1 Q5JTC6 VAR_076268 p.Arg178Cys LB/B rs376626895 -
AMER2 Q8N7J2 VAR_031303 p.Ile659Met LB/B rs2282406 -
AMER2 Q8N7J2 VAR_036448 p.Ala457Thr US - A colorectal cancer sample
AMER3 Q8N944 VAR_039218 p.Ser340Pro LB/B rs1905235 -
AMFR Q9UKV5 VAR_035790 p.Asp605Val US rs373191257 A breast cancer sample
AMH P03971 VAR_007483 p.Val12Gly LP/P rs149082963 Persistent Muellerian duct syndrome 1 (PMDS1) [MIM:261550]
AMH P03971 VAR_007484 p.Ser49Ile LB/B rs10407022 -
AMH P03971 VAR_007485 p.Leu70Pro LP/P - Persistent Muellerian duct syndrome 1 (PMDS1) [MIM:261550]
AMH P03971 VAR_007486 p.Gly101Val LP/P - Persistent Muellerian duct syndrome 1 (PMDS1) [MIM:261550]
AMH P03971 VAR_007487 p.Arg123Trp LP/P rs569914235 Persistent Muellerian duct syndrome 1 (PMDS1) [MIM:261550]
AMH P03971 VAR_007488 p.Tyr167Cys LP/P rs371874189 Persistent Muellerian duct syndrome 1 (PMDS1) [MIM:261550]
AMH P03971 VAR_007489 p.Gln185Glu LB/B rs200523942 -
AMH P03971 VAR_007490 p.Arg194Cys LP/P rs777003373 Persistent Muellerian duct syndrome 1 (PMDS1) [MIM:261550]
AMH P03971 VAR_007491 p.Gln325Arg LB/B rs140765565 -
AMH P03971 VAR_007492 p.Val477Ala LP/P rs1358787117 Persistent Muellerian duct syndrome 1 (PMDS1) [MIM:261550]
AMH P03971 VAR_031027 p.His506Gln LP/P rs138571039 Persistent Muellerian duct syndrome 1 (PMDS1) [MIM:261550]
AMH P03971 VAR_031028 p.Cys525Tyr LP/P - Persistent Muellerian duct syndrome 1 (PMDS1) [MIM:261550]
AMH P03971 VAR_065100 p.Val515Ala LB/B rs10417628 -
AMHR2 Q16671 VAR_015525 p.Arg54Cys LP/P rs534999427 Persistent Muellerian duct syndrome 2 (PMDS2) [MIM:261550]
AMHR2 Q16671 VAR_015526 p.Gly142Val LP/P - Persistent Muellerian duct syndrome 2 (PMDS2) [MIM:261550]
AMHR2 Q16671 VAR_015527 p.His282Gln LP/P rs539695176 Persistent Muellerian duct syndrome 2 (PMDS2) [MIM:261550]
AMHR2 Q16671 VAR_015528 p.Arg406Gln LP/P rs137853104 Persistent Muellerian duct syndrome 2 (PMDS2) [MIM:261550]

AMHR2 Q16671 VAR_015529 p.Asp426Gly LP/P - Persistent Muellerian duct syndrome 2 (PMDS2) [MIM:261550]
AMHR2 Q16671 VAR_015530 p.Val458Ala LP/P rs775889926 Persistent Muellerian duct syndrome 2 (PMDS2) [MIM:261550]
AMHR2 Q16671 VAR_015531 p.Asp491His LP/P rs780680518 Persistent Muellerian duct syndrome 2 (PMDS2) [MIM:261550]
AMHR2 Q16671 VAR_015532 p.Arg504Cys LP/P rs772294564 Persistent Muellerian duct syndrome 2 (PMDS2) [MIM:261550]
AMHR2 Q16671 VAR_069048 p.Arg319His LB/B rs144236183 -
AMMECR1 Q9Y4X0 VAR_078027 p.Gly177Asp LP/P rs1057519337 Midface hypoplasia, hearing impairment, elliptocytosis, and nephrocalcinosis (MFHIEN) [MIM:300990]
AMN Q9BXJ7 VAR_015733 p.Thr41Ile LP/P rs119478058 Imerslund-Grasbeck syndrome 2 (IGS2) [MIM:618882]
AMN Q9BXJ7 VAR_081906 p.Met69Lys LP/P rs375774640 Imerslund-Grasbeck syndrome 2 (IGS2) [MIM:618882]
AMN Q9BXJ7 VAR_081907 p.Cys234Phe LP/P rs386834176 Imerslund-Grasbeck syndrome 2 (IGS2) [MIM:618882]
AMOTL1 Q8IY63 VAR_033498 p.Pro847Leu LB/B rs11020968 -
AMOTL2 Q9Y2J4 VAR_023535 p.Glu731Asp LB/B rs1353776 -
AMOTL2 Q9Y2J4 VAR_055497 p.Thr227Ile LB/B rs35377537 -
AMOTL2 Q9Y2J4 VAR_055498 p.Ala342Pro LB/B rs2303635 -
AMOTL2 Q9Y2J4 VAR_055499 p.Gly415Ser LB/B rs2241559 -
AMPD1 P23109 VAR_013270 p.Pro81Leu LB/B rs61752479 -
AMPD1 P23109 VAR_013271 p.Arg421Trp LP/P rs35859650 Myopathy due to myoadenylate deaminase deficiency (MMDD) [MIM:615511]
AMPD1 P23109 VAR_013272 p.Arg458His LP/P rs121912682 Myopathy due to myoadenylate deaminase deficiency (MMDD) [MIM:615511]
AMPD1 P23109 VAR_035801 p.Pro666His US - A colorectal cancer sample
AMPD1 P23109 VAR_048860 p.Glu55Lys LB/B rs2273268 -
AMPD2 Q01433 VAR_069105 p.Ile522Val LB/B rs201254826 -
AMPD2 Q01433 VAR_071158 p.Arg674His LP/P rs587777395 Pontocerebellar hypoplasia 9 (PCH9) [MIM:615809]
AMPD2 Q01433 VAR_071159 p.Asp793Tyr LP/P rs587777394 Pontocerebellar hypoplasia 9 (PCH9) [MIM:615809]
AMPD2 Q01433 VAR_071193 p.Glu778Asp LP/P rs587777392 Pontocerebellar hypoplasia 9 (PCH9) [MIM:615809]
AMPD3 Q01432 VAR_009881 p.Arg573Cys LP/P rs3741040 Adenosine monophosphate deaminase deficiency erythrocyte type (AMPDDE) [MIM:612874]
AMPD3 Q01432 VAR_033499 p.Arg185Trp LB/B rs11042836 -
AMPD3 Q01432 VAR_042606 p.Asn310Lys LP/P - Adenosine monophosphate deaminase deficiency erythrocyte type (AMPDDE) [MIM:612874]
AMPD3 Q01432 VAR_042607 p.Val311Leu LP/P rs117706710 Adenosine monophosphate deaminase deficiency erythrocyte type (AMPDDE) [MIM:612874]
AMPD3 Q01432 VAR_042608 p.Ala320Val LP/P rs147542803 Adenosine monophosphate deaminase deficiency erythrocyte type (AMPDDE) [MIM:612874]
AMPD3 Q01432 VAR_042609 p.Met324Thr LP/P rs750004231 Adenosine monophosphate deaminase deficiency erythrocyte type (AMPDDE) [MIM:612874]
AMPD3 Q01432 VAR_042610 p.Arg331Cys LP/P rs758038726 Adenosine monophosphate deaminase deficiency erythrocyte type (AMPDDE) [MIM:612874]

AMPD3 Q01432 VAR_042611 p.Arg402Cys LP/P rs766280048 Adenosine monophosphate deaminase deficiency erythrocyte type (AMPDDE) [MIM:612874]
AMPD3 Q01432 VAR_042612 p.Trp450Arg LP/P rs1273151844 Adenosine monophosphate deaminase deficiency erythrocyte type (AMPDDE) [MIM:612874]
AMPD3 Q01432 VAR_042613 p.Tyr455His LB/B rs36003153 -
AMPD3 Q01432 VAR_042614 p.Pro585Leu LP/P rs748852415 Adenosine monophosphate deaminase deficiency erythrocyte type (AMPDDE) [MIM:612874]
AMPD3 Q01432 VAR_042615 p.Gln712Pro LP/P - Adenosine monophosphate deaminase deficiency erythrocyte type (AMPDDE) [MIM:612874]
AMPH P49418 VAR_053004 p.Lys218Glu LB/B rs35166354 -
AMPH P49418 VAR_053005 p.Met376Ile LB/B rs17171345 -
AMPH P49418 VAR_053006 p.Lys496Thr LB/B rs35024632 -
AMT P48728 VAR_007951 p.His42Arg LP/P rs121964983 Non-ketotic hyperglycinemia (NKH) [MIM:605899]
AMT P48728 VAR_007952 p.Gly47Arg LP/P rs121964982 Non-ketotic hyperglycinemia (NKH) [MIM:605899]
AMT P48728 VAR_007953 p.Gly269Asp LP/P rs121964981 Non-ketotic hyperglycinemia (NKH) [MIM:605899]
AMT P48728 VAR_007954 p.Asp276His LP/P rs121964984 Non-ketotic hyperglycinemia (NKH) [MIM:605899]
AMT P48728 VAR_007955 p.Arg320His LP/P rs121964985 Non-ketotic hyperglycinemia (NKH) [MIM:605899]
AMT P48728 VAR_016847 p.Asn145Ile LP/P rs386833682 Non-ketotic hyperglycinemia (NKH) [MIM:605899]
AMT P48728 VAR_016848 p.Glu211Lys LP/P rs116192290 Non-ketotic hyperglycinemia (NKH) [MIM:605899]
AMT P48728 VAR_074107 p.Arg265Cys LP/P rs779483959 Non-ketotic hyperglycinemia (NKH) [MIM:605899]
AMT P48728 VAR_078794 p.Arg94Trp LP/P rs1126422 Non-ketotic hyperglycinemia (NKH) [MIM:605899]
AMT P48728 VAR_078795 p.Arg222Cys LP/P rs781466698 Non-ketotic hyperglycinemia (NKH) [MIM:605899]
AMT P48728 VAR_078796 p.Arg296Cys LP/P rs1056820947 Non-ketotic hyperglycinemia (NKH) [MIM:605899]
AMTN Q6UX39 VAR_035791 p.Gly78Ser US rs151041998 A colorectal cancer sample
AMTN Q6UX39 VAR_050661 p.Asn45Ser LB/B rs7660807 -
AMTN Q6UX39 VAR_050662 p.Ser50Pro LB/B rs34803339 -
AMZ1 Q400G9 VAR_024849 p.Arg491His LB/B rs7776970 -
AMZ2 Q86W34 VAR_024850 p.Asn30Asp LB/B rs3213690 -
AMZ2 Q86W34 VAR_047343 p.His146Gln LB/B rs3207194 -
ANAPC10 Q9UM13 VAR_025200 p.Arg46Gln LB/B rs35257136 -
ANAPC4 Q9UJX5 VAR_035792 p.Ile155Val US - A colorectal cancer sample
ANAPC4 Q9UJX5 VAR_054044 p.Arg465Gln LB/B rs34811474 -
ANAPC4 Q9UJX5 VAR_054045 p.Glu800Gly LB/B rs11550697 -
ANAPC5 Q9UJX4 VAR_035793 p.Gln617His US - A breast cancer sample
ANG P03950 VAR_013148 p.Lys84Glu LB/B rs17560 -
ANG P03950 VAR_044145 p.Phe12Ser LP/P - Amyotrophic lateral sclerosis 9 (ALS9) [MIM:611895]
ANG P03950 VAR_044146 p.Pro20Ser LP/P - Amyotrophic lateral sclerosis 9 (ALS9) [MIM:611895]
ANG P03950 VAR_044147 p.Gln36Leu LP/P rs121909535 Amyotrophic lateral sclerosis 9 (ALS9) [MIM:611895]
ANG P03950 VAR_044148 p.Lys41Glu LP/P rs121909537 Amyotrophic lateral sclerosis 9 (ALS9) [MIM:611895]
ANG P03950 VAR_044149 p.Lys41Ile LP/P rs121909536 Amyotrophic lateral sclerosis 9 (ALS9) [MIM:611895]
ANG P03950 VAR_044150 p.Ser52Asn LP/P rs121909542 Amyotrophic lateral sclerosis 9 (ALS9) [MIM:611895]
ANG P03950 VAR_044151 p.Arg55Lys LP/P rs121909538 Amyotrophic lateral sclerosis 9 (ALS9) [MIM:611895]
ANG P03950 VAR_044152 p.Cys63Trp LP/P rs121909539 Amyotrophic lateral sclerosis 9 (ALS9) [MIM:611895]

ANG P03950 VAR_044153 p.Lys64Ile LP/P rs121909540 Amyotrophic lateral sclerosis 9 (ALS9) [MIM:611895]
ANG P03950 VAR_044154 p.Ile70Val LB/B rs121909541 -
ANG P03950 VAR_044155 p.Pro136Leu LP/P rs121909543 Amyotrophic lateral sclerosis 9 (ALS9) [MIM:611895]
ANG P03950 VAR_044156 p.Val137Ile LP/P rs121909544 Amyotrophic lateral sclerosis 9 (ALS9) [MIM:611895]
ANG P03950 VAR_044157 p.His138Arg LP/P - Amyotrophic lateral sclerosis 9 (ALS9) [MIM:611895]
ANG P03950 VAR_073021 p.Tyr38His LP/P rs1032422334 Amyotrophic lateral sclerosis 9 (ALS9) [MIM:611895]
ANG P03950 VAR_073022 p.Asp46Gly LP/P rs1440927797 Amyotrophic lateral sclerosis 9 (ALS9) [MIM:611895]
ANGEL1 Q9UNK9 VAR_056239 p.Gly141Trp LB/B rs34270005 -
ANGEL1 Q9UNK9 VAR_056240 p.Phe515Cys LB/B rs2075773 -
ANGEL1 Q9UNK9 VAR_061374 p.Val383Leu LB/B rs45499197 -
ANGEL2 Q5VTE6 VAR_050290 p.Pro97Ser LB/B rs11542154 -
ANGPT1 Q15389 VAR_069165 p.Leu247Pro LB/B rs73701083 -
ANGPT2 O15123 VAR_049069 p.Val333Ile LB/B rs7813215 -
ANGPT4 Q9Y264 VAR_049070 p.Glu395Lys LB/B rs869171 -
ANGPTL3 Q9Y5C1 VAR_049071 p.Asn418Tyr LB/B rs4145257 -
ANGPTL3 Q9Y5C1 VAR_067283 p.Leu127Phe LB/B rs72649573 -
ANGPTL3 Q9Y5C1 VAR_075670 p.Lys63Thr LB/B rs146749211 -
ANGPTL3 Q9Y5C1 VAR_075671 p.Glu91Gly LB/B rs139334976 -
ANGPTL3 Q9Y5C1 VAR_075672 p.Leu164Phe LB/B rs775976787 -
ANGPTL3 Q9Y5C1 VAR_075673 p.Asn173Ser LB/B rs149624466 -
ANGPTL3 Q9Y5C1 VAR_075674 p.Met259Thr LB/B rs77871363 -
ANGPTL3 Q9Y5C1 VAR_075675 p.Arg288Gln LB/B rs763904695 -
ANGPTL3 Q9Y5C1 VAR_075677 p.Ser292Pro LB/B rs138899888 -
ANGPTL3 Q9Y5C1 VAR_075678 p.Tyr344Ser LB/B rs1334979946 -
ANGPTL3 Q9Y5C1 VAR_075679 p.Glu375Lys LB/B rs768802285 -
ANGPTL3 Q9Y5C1 VAR_075680 p.Tyr417Cys LB/B rs376210525 -
ANGPTL4 Q9BY76 VAR_020428 p.Thr266Met LB/B rs1044250 -
ANGPTL4 Q9BY76 VAR_032642 p.Pro5Leu LB/B rs761583091 -
ANGPTL4 Q9BY76 VAR_032643 p.Glu40Lys LB/B rs116843064 -
ANGPTL4 Q9BY76 VAR_032644 p.Met41Ile LB/B rs186754194 -
ANGPTL4 Q9BY76 VAR_032645 p.Ser67Arg LB/B rs538554190 -
ANGPTL4 Q9BY76 VAR_032646 p.Arg72Leu LB/B rs141831018 -
ANGPTL4 Q9BY76 VAR_032647 p.Gly77Arg LB/B rs568624939 -
ANGPTL4 Q9BY76 VAR_032648 p.Glu167Lys LB/B rs140640857 -
ANGPTL4 Q9BY76 VAR_032649 p.Pro174Ser LB/B rs1008363865 -
ANGPTL4 Q9BY76 VAR_032650 p.Glu190Gln LB/B rs77938377 -
ANGPTL4 Q9BY76 VAR_032651 p.Glu196Lys LB/B rs1192364228 -
ANGPTL4 Q9BY76 VAR_032652 p.Arg230Cys LB/B rs201026877 -
ANGPTL4 Q9BY76 VAR_032653 p.Gly233Arg LB/B - -
ANGPTL4 Q9BY76 VAR_032654 p.Phe237Val LB/B rs768374046 -
ANGPTL4 Q9BY76 VAR_032655 p.Pro251Thr LB/B rs376328756 -
ANGPTL4 Q9BY76 VAR_032656 p.Arg278Gln LB/B rs35061979 -
ANGPTL4 Q9BY76 VAR_032657 p.Val291Met LB/B rs150000287 -
ANGPTL4 Q9BY76 VAR_032658 p.Leu293Met LB/B rs775667046 -
ANGPTL4 Q9BY76 VAR_032659 p.Glu296Val LB/B - -

ANGPTL4	Q9BY76	VAR_032660	p.Pro307Ser	LB/B	rs751249880	-
ANGPTL4	Q9BY76	VAR_032661	p.Val308Met	LB/B	rs139998264	-
ANGPTL4	Q9BY76	VAR_032662	p.Arg336Cys	LB/B	rs140744493	-
ANGPTL4	Q9BY76	VAR_032663	p.Asp338Glu	LB/B	rs780121474	-
ANGPTL4	Q9BY76	VAR_032664	p.Trp349Cys	LB/B	rs1033145581	-
ANGPTL4	Q9BY76	VAR_032665	p.Gly361Arg	LB/B	rs755737249	-
ANGPTL4	Q9BY76	VAR_032666	p.Gly361Ser	LB/B	rs755737249	-
ANGPTL4	Q9BY76	VAR_032667	p.Arg371Gln	LB/B	rs779488814	-
ANGPTL4	Q9BY76	VAR_032668	p.Arg384Trp	LB/B	rs146942305	-
ANGPTL5	Q86XS5	VAR_055803	p.Ser175Pro	LB/B	rs7946238	-
ANGPTL7	O43827	VAR_025075	p.Glu51Asp	LB/B	rs28990992	-
ANGPTL7	O43827	VAR_025076	p.Arg140His	LB/B	rs28991002	-
ANGPTL7	O43827	VAR_025077	p.Gln175His	LB/B	rs28991009	-
ANGPTL8	Q6UXH0	VAR_039046	p.Arg59Trp	LB/B	rs2278426	-
ANGPTL8	Q6UXH0	VAR_039047	p.Arg147Gln	LB/B	rs34056604	-
ANK1	P16157	VAR_000595	p.Arg21Thr	LB/B	-	-
ANK1	P16157	VAR_000596	p.Val463Ile	LP/P	rs140085544	Spherocytosis 1 (SPH1) [MIM:182900]
ANK1	P16157	VAR_000597	p.Arg619His	LB/B	rs2304877	-
ANK1	P16157	VAR_000598	p.Val750Ala	LB/B	-	-
ANK1	P16157	VAR_000599	p.Asp845Glu	LB/B	-	-
ANK1	P16157	VAR_000600	p.Ser1392Thr	LB/B	-	-
ANK1	P16157	VAR_000601	p.Glu1286Asp	LB/B	-	-
ANK1	P16157	VAR_000602	p.Asp1592Asn	LB/B	rs1457291305	-
ANK1	P16157	VAR_026411	p.Val991Leu	LB/B	rs758454168	-
ANK1	P16157	VAR_028769	p.Leu733Ile	LB/B	rs11778936	-
ANK1	P16157	VAR_028770	p.Ala1126Pro	LB/B	rs504465	-
ANK1	P16157	VAR_028771	p.Thr1192Pro	LB/B	rs486770	-
ANK1	P16157	VAR_028772	p.Met1325Val	LB/B	rs10093583	-
ANK1	P16157	VAR_028773	p.Val1546Ile	LB/B	rs1060130	-
ANK1	P16157	VAR_035605	p.Asp332His	US	-	A breast cancer sample
ANK1	P16157	VAR_048263	p.Thr1075Ile	LB/B	rs35213384	-
ANK1	P16157	VAR_054991	p.Leu276Arg	LP/P	-	Spherocytosis 1 (SPH1) [MIM:182900]
ANK1	P16157	VAR_054992	p.Ile1054Thr	LP/P	-	Spherocytosis 1 (SPH1) [MIM:182900]
ANK1	P16157	VAR_061012	p.Arg832Gln	LB/B	rs34523608	-
ANK2	Q01484	VAR_022934	p.Glu1458Gly	US	rs72544141	Long QT syndrome 4 (LQT4) [MIM:600919]
ANK2	Q01484	VAR_022935	p.Leu3740Ile	LP/P	rs35530544	Long QT syndrome 4 (LQT4) [MIM:600919]
ANK2	Q01484	VAR_022936	p.Thr3744Asn	LP/P	rs121912705	Long QT syndrome 4 (LQT4) [MIM:600919]
ANK2	Q01484	VAR_022937	p.Arg3906Trp	LP/P	rs121912706	Long QT syndrome 4 (LQT4) [MIM:600919]
ANK2	Q01484	VAR_022938	p.Glu3931Lys	LP/P	rs45454496	Long QT syndrome 4 (LQT4) [MIM:600919]
ANK2	Q01484	VAR_035606	p.Gly685Glu	US	-	A breast cancer sample
ANK2	Q01484	VAR_035607	p.Gly1267Arg	US	-	A colorectal cancer sample
ANK2	Q01484	VAR_035608	p.Thr3653Lys	US	-	A colorectal cancer sample
ANK2	Q01484	VAR_055504	p.Asn687Ser	LB/B	rs29372	-
ANK2	Q01484	VAR_055505	p.Val2369Ala	LB/B	rs28377576	-
ANK2	Q01484	VAR_081135	p.Val1555Asp	LB/B	-	-
ANK3	Q12955	VAR_054333	p.Ile4257Val	LB/B	rs12261793	-
ANK3	Q12955	VAR_059115	p.His2885Gln	LB/B	rs11599164	-
ANK3	Q12955	VAR_059116	p.Ile3117Val	LB/B	rs28932171	-
ANK3	Q12955	VAR_059117	p.Lys3123Arg	LB/B	rs10821668	-

ANK3	Q12955	VAR_061013	p.Lys2318Arg	LB/B	rs59021407	-
ANK3	Q12955	VAR_061014	p.Gln2996His	LB/B	rs41274672	-
ANK3	Q12955	VAR_068702	p.Ser1569Ala	US	rs375050420	-
ANK3	Q12955	VAR_068703	p.Thr3720Met	US	rs201547988	-
ANK3	Q12955	VAR_068704	p.Thr4255Pro	US	rs769573528	-
ANK3	Q12955	VAR_077912	p.Asp968His	US	rs730882195	-
ANKAR	Q7Z5J8	VAR_032164	p.Ile675Val	LB/B	rs16831887	-
ANKAR	Q7Z5J8	VAR_032165	p.Leu1077Phe	LB/B	rs1225090	-
ANKDD1A	Q495B1	VAR_032358	p.Lys355Glu	LB/B	rs34988193	-
ANKEF1	Q9NU02	VAR_024172	p.Leu324Gln	LB/B	rs652633	-
ANKEF1	Q9NU02	VAR_033500	p.Pro74Thr	LB/B	rs7260784	-
ANKEF1	Q9NU02	VAR_033501	p.Gly412Glu	LB/B	rs524625	-
ANKEF1	Q9NU02	VAR_033502	p.Arg742Gln	LB/B	rs6087119	-
ANKEF1	Q9NU02	VAR_035609	p.Lys694Asn	US	-	A breast cancer sample
ANKFN1	Q8N957	VAR_060725	p.Val445Ile	LB/B	rs10852985	-
ANKH	Q9HCJ1	VAR_012192	p.Trp292Arg	LP/P	-	Craniometaphyseal dysplasia, autosomal dominant (CMDD) [MIM:123000]
ANKH	Q9HCJ1	VAR_012193	p.Cys331Arg	LP/P	-	Craniometaphyseal dysplasia, autosomal dominant (CMDD) [MIM:123000]
ANKH	Q9HCJ1	VAR_012198	p.Gly389Arg	LP/P	rs28939080	Craniometaphyseal dysplasia, autosomal dominant (CMDD) [MIM:123000]
ANKH	Q9HCJ1	VAR_017556	p.Met48Thr	LP/P	rs121908407	Chondrocalcinosis 2 (CCAL2) [MIM:118600]
ANKH	Q9HCJ1	VAR_022606	p.Pro5Leu	LP/P	rs121908409	Chondrocalcinosis 2 (CCAL2) [MIM:118600]
ANKH	Q9HCJ1	VAR_022607	p.Pro5Thr	LP/P	rs121908410	Chondrocalcinosis 2 (CCAL2) [MIM:118600]
ANKHD1	Q8IWZ3	VAR_035291	p.Leu175Met	LB/B	rs17850570	-
ANKHD1	Q8IWZ3	VAR_035292	p.Gly228Cys	LB/B	rs17850572	-
ANKHD1	Q8IWZ3	VAR_035293	p.Asn1760Ser	LB/B	rs3752704	-
ANKHD1	Q8IWZ3	VAR_048281	p.Gly1586Ser	LB/B	rs1051309	-
ANKIB1	Q9P2G1	VAR_030862	p.Leu1016Met	LB/B	rs38794	-
ANKK1	Q8NFD2	VAR_025010	p.Glu713Lys	LB/B	rs1800497	-
ANKK1	Q8NFD2	VAR_036784	p.Ala239Thr	LB/B	rs7118900	-
ANKK1	Q8NFD2	VAR_036785	p.Gly318Arg	LB/B	rs11604671	-
ANKK1	Q8NFD2	VAR_036786	p.Gly442Arg	LB/B	rs4938016	-
ANKK1	Q8NFD2	VAR_036787	p.His490Arg	LB/B	rs2734849	-
ANKK1	Q8NFD2	VAR_040359	p.Asp4Tyr	LB/B	rs35657708	-
ANKK1	Q8NFD2	VAR_040360	p.Arg122His	LB/B	rs35877321	-
ANKK1	Q8NFD2	VAR_040361	p.Pro276Leu	LB/B	rs35488601	-
ANKK1	Q8NFD2	VAR_040362	p.Lys347Thr	US	-	A breast infiltrating ductal carcinoma sample
ANKK1	Q8NFD2	VAR_040363	p.Leu366Phe	LB/B	rs56339158	-
ANKK1	Q8NFD2	VAR_040364	p.His367Gln	LB/B	rs34298987	-
ANKK1	Q8NFD2	VAR_040365	p.Glu376Lys	LB/B	rs56299709	-
ANKK1	Q8NFD2	VAR_040366	p.Glu426Lys	LB/B	rs55699907	-
ANKK1	Q8NFD2	VAR_040367	p.Gly451Arg	LB/B	rs34983219	-
ANKK1	Q8NFD2	VAR_040368	p.Thr595Ile	LB/B	rs55787008	-
ANKK1	Q8NFD2	VAR_040369	p.Pro596Leu	LB/B	rs7104979	-
ANKK1	Q8NFD2	VAR_040370	p.Asn653Ser	LB/B	rs55849504	-
ANKK1	Q8NFD2	VAR_040371	p.Ser670Gly	LB/B	rs56006094	-
ANKK1	Q8NFD2	VAR_040372	p.Gln717Leu	US	-	A lung large cell carcinoma sample
ANKK1	Q8NFD2	VAR_040373	p.Arg736Leu	US	-	A lung squamous cell carcinoma sample

ANKK1 Q8NFD2 VAR_040374 p.Glu764Lys US - A lung neuroendocrine carcinoma sample
ANKLE1 Q8NAG6 VAR_033507 p.Leu94Gln LB/B rs8108174 -
ANKLE1 Q8NAG6 VAR_033508 p.Leu184Trp LB/B rs2363956 -
ANKLE1 Q8NAG6 VAR_033509 p.Thr311Pro LB/B rs891017 -
ANKLE1 Q8NAG6 VAR_033510 p.Gln435Arg LB/B rs11086065 -
ANKLE1 Q8NAG6 VAR_033511 p.Val447Met LB/B rs34112069 -
ANKLE1 Q8NAG6 VAR_061015 p.Pro160Ser LB/B rs59119993 -
ANKLE1 Q8NAG6 VAR_063681 p.Ala31Thr LB/B rs8100241 -
ANKLE1 Q8NAG6 VAR_063682 p.Ala71Val LB/B rs1864116 -
ANKLE2 Q86XL3 VAR_031097 p.His122Tyr LB/B rs1132375 -
ANKLE2 Q86XL3 VAR_031098 p.Gln148Glu LB/B rs7968520 -
ANKLE2 Q86XL3 VAR_031099 p.Arg720His LB/B rs10781634 -
ANKLE2 Q86XL3 VAR_076205 p.Leu573Val US rs863225465 Microcephaly 16, primary, autosomal recessive (MCPH16) [MIM:616681]
ANKLE2 Q86XL3 VAR_083604 p.Ala8Val US rs986142623 Microcephaly 16, primary, autosomal recessive (MCPH16) [MIM:616681]
ANKLE2 Q86XL3 VAR_083605 p.Ala27Gly US - Microcephaly 16, primary, autosomal recessive (MCPH16) [MIM:616681]
ANKLE2 Q86XL3 VAR_083606 p.Ala109Pro LP/P - Microcephaly 16, primary, autosomal recessive (MCPH16) [MIM:616681]
ANKLE2 Q86XL3 VAR_083607 p.Gly201Trp LP/P rs1185537869 Microcephaly 16, primary, autosomal recessive (MCPH16) [MIM:616681]
ANKLE2 Q86XL3 VAR_083608 p.Val229Gly US - Microcephaly 16, primary, autosomal recessive (MCPH16) [MIM:616681]
ANKLE2 Q86XL3 VAR_083610 p.Arg536Cys US rs761627940 Microcephaly 16, primary, autosomal recessive (MCPH16) [MIM:616681]
ANKLE2 Q86XL3 VAR_083611 p.Gly585Val US - Microcephaly 16, primary, autosomal recessive (MCPH16) [MIM:616681]
ANKMY1 Q9P2S6 VAR_048264 p.Asp89Asn LB/B rs35278753 -
ANKMY1 Q9P2S6 VAR_048265 p.Ile424Met LB/B rs35996697 -
ANKMY1 Q9P2S6 VAR_048266 p.Asp451Val LB/B rs3796118 -
ANKMY1 Q9P2S6 VAR_048267 p.Val472Leu LB/B rs3821348 -
ANKMY1 Q9P2S6 VAR_048268 p.Thr649Met LB/B rs35044862 -
ANKRD1 Q15327 VAR_047112 p.Thr116Met US rs142354133 -
ANKRD10 Q9NXR5 VAR_020095 p.Pro320Leu LB/B rs3742185 -
ANKRD11 Q6UB99 VAR_075870 p.Arg2512Gln US - KBG syndrome (KBGS) [MIM:148050]
ANKRD12 Q6UB98 VAR_019425 p.Ser171Thr LB/B - -
ANKRD12 Q6UB98 VAR_022088 p.Ser818Asn LB/B rs2298546 -
ANKRD12 Q6UB98 VAR_024173 p.Lys906Arg LB/B rs4798791 -
ANKRD12 Q6UB98 VAR_026042 p.Pro277Ala LB/B rs2298548 -
ANKRD12 Q6UB98 VAR_048271 p.Glu390Asp LB/B rs35101529 -
ANKRD12 Q6UB98 VAR_048272 p.Thr507Ile LB/B rs17498752 -
ANKRD12 Q6UB98 VAR_048273 p.Thr531Ser LB/B rs7243088 -
ANKRD12 Q6UB98 VAR_048274 p.Leu998Ser LB/B rs34996750 -
ANKRD12 Q6UB98 VAR_048275 p.Ser1758Pro LB/B rs3744822 -
ANKRD12 Q6UB98 VAR_081528 p.Ser307Cys US - -
ANKRD13A Q8IZ07 VAR_048276 p.Leu505Pro LB/B rs2287174 -
ANKRD13C Q8N6S4 VAR_026825 p.Thr413Ser LB/B rs17852616 -
ANKRD16 Q6P6B7 VAR_026832 p.Gln353Arg LB/B rs1052420 -

ANKRD16	Q6P6B7	VAR_033503	p.Ala128Gly	LB/B	rs2296136	-
ANKRD17	O75179	VAR_036711	p.His2560Tyr	LB/B	rs2306059	-
ANKRD18A	Q8IVF6	VAR_055507	p.Glu130Lys	LB/B	rs1832313	-
ANKRD18A	Q8IVF6	VAR_055508	p.Ala277Glu	LB/B	rs632200	-
ANKRD18A	Q8IVF6	VAR_055509	p.Asn484Ser	LB/B	rs2996347	-
ANKRD18A	Q8IVF6	VAR_055510	p.Tyr688Cys	LB/B	rs2799163	-
ANKRD18A	Q8IVF6	VAR_055511	p.Thr942Ile	LB/B	rs11999308	-
ANKRD18A	Q8IVF6	VAR_055512	p.Glu945Asp	LB/B	rs12341435	-
ANKRD18A	Q8IVF6	VAR_059118	p.Glu838Asp	LB/B	rs12341435	-
ANKRD2	Q9GZV1	VAR_042498	p.Ala62Thr	LB/B	rs7094973	-
ANKRD22	Q5VYY1	VAR_027628	p.Glu73Gly	LB/B	rs17113412	-
ANKRD22	Q5VYY1	VAR_027629	p.Tyr79His	LB/B	rs17851907	-
ANKRD22	Q5VYY1	VAR_027630	p.Gln148Pro	LB/B	rs2304804	-
ANKRD22	Q5VYY1	VAR_027631	p.Arg177Ile	LB/B	rs7912706	-
ANKRD24	Q8TF21	VAR_042536	p.Ala111Thr	LB/B	rs2052191	-
ANKRD24	Q8TF21	VAR_042537	p.Arg349Gln	LB/B	rs12978469	-
ANKRD24	Q8TF21	VAR_042538	p.Glu585Lys	LB/B	rs10413818	-
ANKRD24	Q8TF21	VAR_042539	p.Ser684Ala	LB/B	rs353693	-
ANKRD26	Q9UPS8	VAR_026833	p.Gln20Arg	LB/B	rs7897309	-
ANKRD26	Q9UPS8	VAR_026834	p.Val1305Ile	LB/B	rs10829163	-
ANKRD26	Q9UPS8	VAR_026835	p.Phe1514Leu	LB/B	rs2274741	-
ANKRD26	Q9UPS8	VAR_055513	p.Ile425Val	LB/B	rs12359281	-
ANKRD26	Q9UPS8	VAR_055514	p.Val1220Leu	LB/B	rs12572862	-
ANKRD26P1	Q6NSI1	VAR_040003	p.Lys265Thr	LB/B	rs1436436	-
ANKRD27	Q96NW4	VAR_030317	p.Ser657Gly	LB/B	rs2287669	-
ANKRD27	Q96NW4	VAR_030318	p.Pro761Arg	LB/B	rs2302970	-
ANKRD29	Q8N6D5	VAR_026869	p.Gly112Glu	LB/B	rs17855552	-
ANKRD29	Q8N6D5	VAR_035610	p.Val95Met	US	rs767831283	A breast cancer sample
ANKRD30A	Q9BXX3	VAR_033504	p.Thr611Ala	LB/B	rs16937417	-
ANKRD30A	Q9BXX3	VAR_033505	p.Lys917Asn	LB/B	rs1209750	-
ANKRD30A	Q9BXX3	VAR_035611	p.Gln227Glu	US	-	A breast cancer sample
ANKRD30A	Q9BXX3	VAR_055515	p.Arg985Cys	LB/B	rs1200875	-
ANKRD30B	Q9BXX2	VAR_042540	p.Val375Met	LB/B	rs9748611	-
ANKRD30B	Q9BXX2	VAR_042541	p.Phe477Leu	LB/B	rs9675365	-
ANKRD31	Q8N7Z5	VAR_042542	p.Asp702Asn	LB/B	rs1422698	-
ANKRD31	Q8N7Z5	VAR_042543	p.Arg758Gly	LB/B	rs6893216	-
ANKRD31	Q8N7Z5	VAR_042544	p.Asp1609Glu	LB/B	rs961098	-
ANKRD31	Q8N7Z5	VAR_042545	p.Arg1777Lys	LB/B	rs4489037	-
ANKRD33	Q7Z3H0	VAR_059121	p.Tyr140Phe	LB/B	rs697636	-
ANKRD33	Q7Z3H0	VAR_059122	p.Gln257Arg	LB/B	rs34494292	-
ANKRD33	Q7Z3H0	VAR_059123	p.Thr313Asn	LB/B	rs12368048	-
ANKRD34B	A5PLL1	VAR_038952	p.Leu156Ser	LB/B	rs32857	-
ANKRD34C	P0C6C1	VAR_038953	p.Pro369Arg	LB/B	rs410400	-
ANKRD34C	P0C6C1	VAR_038954	p.Pro427His	LB/B	rs449340	-
ANKRD34C	P0C6C1	VAR_038955	p.Leu442Ile	LB/B	rs422777	-
ANKRD35	Q8N283	VAR_026870	p.Pro428Ser	LB/B	rs6670984	-
ANKRD35	Q8N283	VAR_033506	p.Asn978Asp	LB/B	rs16827032	-
ANKRD35	Q8N283	VAR_048278	p.Asn53Lys	LB/B	rs6658371	-
ANKRD35	Q8N283	VAR_061017	p.Arg592Gln	LB/B	rs41315701	-

ANKRD36B	Q8N2N9	VAR_057818	p.Glu496Asp	LB/B	rs13001728	-
ANKRD36B	Q8N2N9	VAR_060687	p.Glu483Gly	LB/B	rs1839230	-
ANKRD36B	Q8N2N9	VAR_060688	p.Ser534Ala	LB/B	rs6761299	-
ANKRD37	Q7Z713	VAR_048279	p.Thr152Ser	LB/B	rs4317244	-
ANKRD39	Q53RE8	VAR_026905	p.Ala113Thr	LB/B	rs17852947	-
ANKRD42	Q8N9B4	VAR_028366	p.Asn198Asp	LB/B	rs17515016	-
ANKRD53	Q8N9V6	VAR_030300	p.Leu153Ile	LB/B	rs17853403	-
ANKRD53	Q8N9V6	VAR_054427	p.Met243Thr	LB/B	rs36123544	-
ANKRD53	Q8N9V6	VAR_061018	p.Ala105Gly	LB/B	rs35096506	-
ANKRD53	Q8N9V6	VAR_067464	p.Arg349Leu	LB/B	rs11688921	-
ANKRD53	Q8N9V6	VAR_067465	p.His431Leu	LB/B	rs3796100	-
ANKRD55	Q3KP44	VAR_030283	p.Val345Met	LB/B	rs321776	-
ANKRD55	Q3KP44	VAR_055516	p.Arg594Gln	LB/B	rs34879141	-
ANKRD6	Q9Y2G4	VAR_039114	p.Gln122Glu	LB/B	rs16881983	-
ANKRD6	Q9Y2G4	VAR_039115	p.Ile128Val	LB/B	rs3748085	-
ANKRD6	Q9Y2G4	VAR_039116	p.Thr233Met	LB/B	rs2273238	-
ANKRD6	Q9Y2G4	VAR_039117	p.Thr545Ala	LB/B	rs9362667	-
ANKRD6	Q9Y2G4	VAR_055506	p.Ala550Thr	LB/B	rs9362667	-
ANKRD60	Q9BZ19	VAR_014400	p.Arg295Cys	LB/B	rs584855	-
ANKRD62	A6NC57	VAR_042527	p.Ala188Ser	LB/B	rs1986751	-
ANKRD62	A6NC57	VAR_042528	p.Cys265Arg	LB/B	rs6505715	-
ANKRD62	A6NC57	VAR_042529	p.Glu406Lys	LB/B	rs4519391	-
ANKRD62	A6NC57	VAR_042530	p.Ala613Thr	LB/B	rs7243248	-
ANKS1A	Q92625	VAR_021168	p.Leu694Ser	LB/B	rs820085	-
ANKS1A	Q92625	VAR_048282	p.Ala355Asp	LB/B	rs6930932	-
ANKS3	Q6ZW76	VAR_048283	p.Ala404Thr	LB/B	rs863980	-
ANKS3	Q6ZW76	VAR_048284	p.Ala593Thr	LB/B	rs9936675	-
ANKS6	Q68DC2	VAR_034794	p.Val644Ile	LB/B	rs6415847	-
ANKS6	Q68DC2	VAR_070105	p.Arg222Trp	LB/B	rs41283630	-
ANKS6	Q68DC2	VAR_070106	p.Ala312Pro	LP/P	-	Nephronophthisis 16 (NPHP16) [MIM:615382]
ANKS6	Q68DC2	VAR_070107	p.Arg440Gln	LB/B	rs763855876	-
ANKS6	Q68DC2	VAR_070108	p.Gln441Arg	LP/P	rs377750405	Nephronophthisis 16 (NPHP16) [MIM:615382]
ANKS6	Q68DC2	VAR_070109	p.Gly640Ser	LB/B	rs749102463	-
ANKS6	Q68DC2	VAR_070110	p.Pro735Ala	LB/B	rs79414550	-
ANKUB1	A6NFN9	VAR_045621	p.Arg217Trp	LB/B	rs7645720	-
ANKUB1	A6NFN9	VAR_045622	p.Ile306Met	LB/B	rs3821406	-
ANKUB1	A6NFN9	VAR_045623	p.Ser386Gly	LB/B	rs7610425	-
ANKZF1	Q9H8Y5	VAR_048269	p.Arg569Trp	LB/B	rs2293076	-
ANKZF1	Q9H8Y5	VAR_048270	p.Pro676Leu	LB/B	rs2293079	-
ANKZF1	Q9H8Y5	VAR_079137	p.Glu152Lys	US	rs149382949	-
ANKZF1	Q9H8Y5	VAR_079138	p.Arg585Gln	US	rs189875478	-
ANKZF1	Q9H8Y5	VAR_079139	p.Gln638Pro	LB/B	-	-
ANLN	Q9NQW6	VAR_025661	p.Ser65Trp	LB/B	rs3735400	-
ANLN	Q9NQW6	VAR_025662	p.Arg185Lys	LB/B	rs197367	-
ANLN	Q9NQW6	VAR_072418	p.Arg431Cys	LP/P	rs587777741	Focal segmental glomerulosclerosis 8 (FSGS8) [MIM:616032]
ANLN	Q9NQW6	VAR_072419	p.Gly618Cys	LP/P	rs1184529372	Focal segmental glomerulosclerosis 8 (FSGS8) [MIM:616032]
ANO1	Q5XXA6	VAR_032417	p.Phe608Ser	LB/B	rs2186797	-

ANO1 Q5XXA6 VAR_032418 p.Gly983Arg LB/B rs3740722 -
ANO10 Q9NW15 VAR_032638 p.Arg462Gln LB/B rs3772165 -
ANO10 Q9NW15 VAR_032639 p.Thr561Met LB/B rs17409162 -
ANO10 Q9NW15 VAR_032640 p.Val583Ala LB/B rs17853862 -
ANO10 Q9NW15 VAR_064888 p.Leu510Arg LP/P rs387907089 Spinocerebellar ataxia, autosomal recessive, 10 (SCAR10) [MIM:613728]
ANO2 Q9NQ90 VAR_020331 p.Ser505Ala LB/B rs1860961 -
ANO2 Q9NQ90 VAR_021932 p.Val112Ala LB/B rs3741903 -
ANO2 Q9NQ90 VAR_057286 p.Pro147Ser LB/B rs3741901 -
ANO2 Q9NQ90 VAR_061853 p.Met401Ile LB/B rs17788563 -
ANO3 Q9BYT9 VAR_057287 p.Leu781Val LB/B rs11825056 -
ANO3 Q9BYT9 VAR_069732 p.Trp490Cys LP/P rs1565132917 Dystonia 24 (DYT24) [MIM:615034]
ANO3 Q9BYT9 VAR_069733 p.Arg494Trp LP/P rs587776922 Dystonia 24 (DYT24) [MIM:615034]
ANO3 Q9BYT9 VAR_069734 p.Ser685Gly LP/P rs587776923 Dystonia 24 (DYT24) [MIM:615034]
ANO3 Q9BYT9 VAR_069735 p.Lys862Asn LP/P rs1277790116 Dystonia 24 (DYT24) [MIM:615034]
ANO4 Q32M45 VAR_032453 p.Gly115Ala LB/B rs34162417 -
ANO5 Q75V66 VAR_023524 p.Cys356Gly LP/P rs119103234 Gnathodiaphyseal dysplasia (GDD) [MIM:166260]
ANO5 Q75V66 VAR_023525 p.Cys356Arg LP/P rs119103234 Gnathodiaphyseal dysplasia (GDD) [MIM:166260]
ANO5 Q75V66 VAR_052339 p.Leu322Phe LB/B rs7481951 -
ANO5 Q75V66 VAR_052340 p.Asn882Lys LB/B rs34969327 -
ANO5 Q75V66 VAR_063582 p.Gly231Val US rs137854523 Muscular dystrophy, limb-girdle, autosomal recessive 12 (LGMDR12) [MIM:611307]
ANO5 Q75V66 VAR_063583 p.Arg758Cys US rs137854529 Miyoshi muscular dystrophy 3 (MMD3) [MIM:613319]
ANO5 Q75V66 VAR_063583 p.Arg758Cys US rs137854529 Muscular dystrophy, limb-girdle, autosomal recessive 12 (LGMDR12) [MIM:611307]
ANO5 Q75V66 VAR_068247 p.Arg58Trp US rs201725369 Muscular dystrophy, limb-girdle, autosomal recessive 12 (LGMDR12) [MIM:611307]
ANO5 Q75V66 VAR_068248 p.Trp655Cys US rs760137559 Miyoshi muscular dystrophy 3 (MMD3) [MIM:613319]
ANO5 Q75V66 VAR_076476 p.Cys356Tyr LP/P - Gnathodiaphyseal dysplasia (GDD) [MIM:166260]
ANO5 Q75V66 VAR_076477 p.Thr513Ile US rs281865467 Gnathodiaphyseal dysplasia (GDD) [MIM:166260]
ANO5 Q75V66 VAR_080271 p.Asn52Ser US rs143777403 Muscular dystrophy, limb-girdle, autosomal recessive 12 (LGMDR12) [MIM:611307]
ANO5 Q75V66 VAR_080272 p.Phe54Ser US rs886043577 Muscular dystrophy, limb-girdle, autosomal recessive 12 (LGMDR12) [MIM:611307]
ANO5 Q75V66 VAR_080273 p.Val87Ile US rs34994927 Muscular dystrophy, limb-girdle, autosomal recessive 12 (LGMDR12) [MIM:611307]
ANO5 Q75V66 VAR_080274 p.Asp93Glu US - Muscular dystrophy, limb-girdle, autosomal recessive 12 (LGMDR12) [MIM:611307]
ANO5 Q75V66 VAR_080275 p.Tyr143Cys US - Muscular dystrophy, limb-girdle, autosomal recessive 12 (LGMDR12) [MIM:611307]
ANO5 Q75V66 VAR_080276 p.Glu202Lys US rs115750596 Muscular dystrophy, limb-girdle, autosomal recessive 12 (LGMDR12) [MIM:611307]
ANO5 Q75V66 VAR_080277 p.Thr206Ala US rs78266558 Muscular dystrophy, limb-girdle, autosomal recessive 12 (LGMDR12) [MIM:611307]
ANO5 Q75V66 VAR_080278 p.Lys259Asn US - Muscular dystrophy, limb-girdle, autosomal recessive 12 (LGMDR12) [MIM:611307]

ANO5 Q75V66 VAR_080279 p.Asn265Ser US rs377553546 Muscular dystrophy, limb-girdle, autosomal recessive 12 (LGMDR12) [MIM:611307]
ANO5 Q75V66 VAR_080280 p.Pro266Leu US rs745908606 Muscular dystrophy, limb-girdle, autosomal recessive 12 (LGMDR12) [MIM:611307]
ANO5 Q75V66 VAR_080281 p.Thr267Ser US rs138144479 Muscular dystrophy, limb-girdle, autosomal recessive 12 (LGMDR12) [MIM:611307]
ANO5 Q75V66 VAR_080282 p.Arg404Leu US - Muscular dystrophy, limb-girdle, autosomal recessive 12 (LGMDR12) [MIM:611307]
ANO5 Q75V66 VAR_080284 p.Ser506Gly US rs141799673 Muscular dystrophy, limb-girdle, autosomal recessive 12 (LGMDR12) [MIM:611307]
ANO5 Q75V66 VAR_080286 p.Arg547Gln US rs139618850 Muscular dystrophy, limb-girdle, autosomal recessive 12 (LGMDR12) [MIM:611307]
ANO5 Q75V66 VAR_080287 p.Ser555Ile US rs375014127 Muscular dystrophy, limb-girdle, autosomal recessive 12 (LGMDR12) [MIM:611307]
ANO5 Q75V66 VAR_080288 p.Phe578Ser US rs137854526 Muscular dystrophy, limb-girdle, autosomal recessive 12 (LGMDR12) [MIM:611307]
ANO5 Q75V66 VAR_080289 p.Met618Ile US rs1422717390 Muscular dystrophy, limb-girdle, autosomal recessive 12 (LGMDR12) [MIM:611307]
ANO5 Q75V66 VAR_080291 p.Thr714Ser US rs200631556 Muscular dystrophy, limb-girdle, autosomal recessive 12 (LGMDR12) [MIM:611307]
ANO5 Q75V66 VAR_080292 p.Leu781Pro US - Muscular dystrophy, limb-girdle, autosomal recessive 12 (LGMDR12) [MIM:611307]
ANO5 Q75V66 VAR_080293 p.Ser796Leu US rs61910685 Muscular dystrophy, limb-girdle, autosomal recessive 12 (LGMDR12) [MIM:611307]
ANO5 Q75V66 VAR_080294 p.Cys804Ser US rs1233836740 Muscular dystrophy, limb-girdle, autosomal recessive 12 (LGMDR12) [MIM:611307]
ANO5 Q75V66 VAR_080295 p.Ala830Val US rs766853141 Muscular dystrophy, limb-girdle, autosomal recessive 12 (LGMDR12) [MIM:611307]
ANO5 Q75V66 VAR_080296 p.Met833Lys US rs142073798 Muscular dystrophy, limb-girdle, autosomal recessive 12 (LGMDR12) [MIM:611307]
ANO5 Q75V66 VAR_080297 p.Met839Arg US - Muscular dystrophy, limb-girdle, autosomal recessive 12 (LGMDR12) [MIM:611307]
ANO5 Q75V66 VAR_080298 p.Met900Leu US rs148293985 Muscular dystrophy, limb-girdle, autosomal recessive 12 (LGMDR12) [MIM:611307]
ANO6 Q4KMQ2 VAR_028109 p.Ala128Thr LB/B rs2162321 -
ANO7 Q6IWH7 VAR_032616 p.Val67Ile LB/B rs2302054 -
ANO7 Q6IWH7 VAR_065166 p.Glu912Lys LB/B rs7590653 -
ANO9 A1A5B4 VAR_032617 p.Ile391Val LB/B rs10794324 -
ANO9 A1A5B4 VAR_032618 p.Cys399Arg LB/B rs10794323 -
ANO9 A1A5B4 VAR_054621 p.Leu93Phe LB/B rs7395065 -
ANOS1 P23352 VAR_007720 p.Asn267Lys LP/P - Hypogonadotropic hypogonadism 1 with or without anosmia (HH1) [MIM:308700]
ANOS1 P23352 VAR_007721 p.Val534Ile LB/B rs808119 -
ANOS1 P23352 VAR_012742 p.Glu514Lys LP/P rs137852515 Hypogonadotropic hypogonadism 1 with or without anosmia (HH1) [MIM:308700]
ANOS1 P23352 VAR_031012 p.Cys163Tyr LP/P - Hypogonadotropic hypogonadism 1 with or without anosmia (HH1) [MIM:308700]
ANOS1 P23352 VAR_031013 p.Cys172Arg LP/P rs1394625082 Hypogonadotropic hypogonadism 1 with or without anosmia (HH1) [MIM:308700]

ANOS1 P23352 VAR_031014 p.Arg262Pro LP/P - Hypogonadotropic hypogonadism 1 with or without anosmia (HH1) [MIM:308700]
ANOS1 P23352 VAR_031015 p.Asn304Ser LP/P rs140812865 Hypogonadotropic hypogonadism 1 with or without anosmia (HH1) [MIM:308700]
ANOS1 P23352 VAR_031016 p.Ser396Leu LP/P rs137852517 Hypogonadotropic hypogonadism 1 with or without anosmia (HH1) [MIM:308700]
ANOS1 P23352 VAR_031017 p.Phe517Leu LP/P - Hypogonadotropic hypogonadism 1 with or without anosmia (HH1) [MIM:308700]
ANOS1 P23352 VAR_031018 p.Trp571Arg LP/P rs1170543613 Hypogonadotropic hypogonadism 1 with or without anosmia (HH1) [MIM:308700]
ANOS1 P23352 VAR_031019 p.Lys666Met LB/B - -
ANOS1 P23352 VAR_031020 p.Arg668His LB/B rs775708192 -
ANOS1 P23352 VAR_065362 p.Cys134Gly LP/P - Hypogonadotropic hypogonadism 1 with or without anosmia (HH1) [MIM:308700]
ANOS1 P23352 VAR_065363 p.Cys163Arg LP/P - Hypogonadotropic hypogonadism 1 with or without anosmia (HH1) [MIM:308700]
ANOS1 P23352 VAR_065364 p.Glu539Lys LP/P rs144586521 Hypogonadotropic hypogonadism 1 with or without anosmia (HH1) [MIM:308700]
ANOS1 P23352 VAR_069207 p.Tyr217Asp LP/P - -
ANOS1 P23352 VAR_069968 p.Val587Leu LP/P rs137900287 Hypogonadotropic hypogonadism 1 with or without anosmia (HH1) [MIM:308700]
ANOS1 P23352 VAR_072992 p.His672Arg LP/P rs199771303 Hypogonadotropic hypogonadism 1 with or without anosmia (HH1) [MIM:308700]
ANP32C O43423 VAR_020419 p.Arg71Lys LB/B rs2288675 -
ANP32C O43423 VAR_020420 p.Glu204Gly LB/B rs2288676 -
ANP32C O43423 VAR_026702 p.Ala23Val LB/B rs2288674 -
ANP32C O43423 VAR_026703 p.Tyr140His LB/B - -
ANP32C O43423 VAR_048314 p.Leu105Pro LB/B rs17008716 -
ANP32D O95626 VAR_027832 p.Leu46Phe LB/B rs7956679 -
ANPEP P15144 VAR_006727 p.Asp242Tyr LB/B - -
ANPEP P15144 VAR_006728 p.Leu243Pro LB/B - -
ANPEP P15144 VAR_014736 p.Arg86Gln LB/B rs25653 -
ANPEP P15144 VAR_014737 p.Ser752Asn LB/B rs25651 -
ANPEP P15144 VAR_031262 p.Val20Met LB/B rs10152474 -
ANPEP P15144 VAR_031263 p.Ala311Val LB/B rs17240268 -
ANPEP P15144 VAR_031264 p.Thr321Met LB/B rs8179199 -
ANPEP P15144 VAR_031265 p.Ile603Lys LB/B rs17240212 -
ANPEP P15144 VAR_031266 p.Ile603Met LB/B rs8192297 -
ANTXR1 Q9H6X2 VAR_053015 p.Arg7Lys LB/B rs28365986 -
ANTXR1 Q9H6X2 VAR_063146 p.Ala326Thr LB/B rs119475040 -
ANTXR2 P58335 VAR_022687 p.Leu45Pro LP/P rs886041401 Hyaline fibromatosis syndrome (HFS) [MIM:228600]
ANTXR2 P58335 VAR_022688 p.Gly105Asp LP/P rs137852902 Hyaline fibromatosis syndrome (HFS) [MIM:228600]
ANTXR2 P58335 VAR_022689 p.Ile189Thr LP/P rs137852905 Hyaline fibromatosis syndrome (HFS) [MIM:228600]
ANTXR2 P58335 VAR_022690 p.Cys218Arg LP/P rs781637328 Hyaline fibromatosis syndrome (HFS) [MIM:228600]

ANTXR2 P58335 VAR_022692 p.Leu329Arg LP/P rs137852903 Hyaline fibromatosis syndrome (HFS) [MIM:228600]
ANTXR2 P58335 VAR_022693 p.Ala357Pro LB/B rs12647691 -
ANTXR2 P58335 VAR_022694 p.Tyr381Cys LP/P rs137852901 Hyaline fibromatosis syndrome (HFS) [MIM:228600]
ANTXRL A6NF34 VAR_042963 p.Pro550Arg LB/B rs1554967247 -
ANXA10 Q9UJ72 VAR_030786 p.Met71Leu LB/B rs6836994 -
ANXA11 P50995 VAR_012006 p.Arg230Cys LB/B rs1049550 -
ANXA11 P50995 VAR_012007 p.Ile457Val LB/B rs1802932 -
ANXA11 P50995 VAR_048259 p.Arg191Gln LB/B rs2229554 -
ANXA11 P50995 VAR_080653 p.Gly38Arg US rs142083484 Amyotrophic lateral sclerosis 23 (ALS23) [MIM:617839]
ANXA11 P50995 VAR_080654 p.Asp40Gly LP/P rs1247392012 Amyotrophic lateral sclerosis 23 (ALS23) [MIM:617839]
ANXA11 P50995 VAR_080655 p.Gly175Arg US rs754594235 Amyotrophic lateral sclerosis 23 (ALS23) [MIM:617839]
ANXA11 P50995 VAR_080656 p.Gly189Glu US rs569546089 Amyotrophic lateral sclerosis 23 (ALS23) [MIM:617839]
ANXA11 P50995 VAR_080657 p.Arg235Gln US rs765489119 Amyotrophic lateral sclerosis 23 (ALS23) [MIM:617839]
ANXA11 P50995 VAR_080658 p.Arg346Cys US rs770574196 Amyotrophic lateral sclerosis 23 (ALS23) [MIM:617839]
ANXA13 P27216 VAR_055501 p.Arg86His LB/B rs2294013 -
ANXA13 P27216 VAR_055502 p.Val108Ile LB/B rs6995099 -
ANXA13 P27216 VAR_055503 p.Val272Ile LB/B rs2294015 -
ANXA2 P07355 VAR_012982 p.Val98Leu LB/B rs17845226 -
ANXA2R Q3ZCQ2 VAR_033348 p.Gln119Arg LB/B rs1054428 -
ANXA2R Q3ZCQ2 VAR_033349 p.Arg186Trp LB/B rs10971 -
ANXA3 P12429 VAR_013914 p.Ser19Asn LB/B rs5951 -
ANXA3 P12429 VAR_013915 p.Ile219Asn LB/B rs5948 -
ANXA3 P12429 VAR_013916 p.Pro251Leu LB/B rs5949 -
ANXA3 P12429 VAR_013917 p.Phe291Ser LB/B rs5941 -
ANXA4 P09525 VAR_055500 p.Thr85Met LB/B rs2228203 -
ANXA7 P20073 VAR_048253 p.Arg441Gln LB/B rs3750575 -
ANXA8 P13928 VAR_000604 p.Ser6Ala LB/B rs3870786 -
ANXA8 P13928 VAR_030630 p.Gly177Ala LB/B rs75345346 -
ANXA8L1 Q5VT79 VAR_048254 p.Ala177Gly LB/B rs1487921382 -
ANXA8L1 Q5VT79 VAR_067447 p.Ala134Val LB/B rs201596209 -
ANXA8L1 Q5VT79 VAR_067448 p.Lys192Asn LB/B rs1249096344 -
ANXA9 O76027 VAR_022814 p.Asp166Gly LB/B rs267733 -
ANXA9 O76027 VAR_031212 p.Arg225Gln LB/B rs775255778 -
ANXA9 O76027 VAR_048255 p.Ala28Thr LB/B rs16832595 -
ANXA9 O76027 VAR_048256 p.Thr114Ala LB/B rs7536645 -
ANXA9 O76027 VAR_048257 p.Ala119Thr LB/B rs16832602 -
ANXA9 O76027 VAR_048258 p.Arg232Gln LB/B rs7542365 -
AOAH P28039 VAR_020133 p.Ala166Thr LB/B rs3735384 -
AOAH P28039 VAR_033513 p.Ala266Gly LB/B rs3735386 -
AOAH P28039 VAR_050663 p.Asp28Asn LB/B rs2228410 -
AOC1 P19801 VAR_007542 p.His645Asp LB/B rs1049793 -

AOC1	P19801	VAR_025078	p.Thr16Met	LB/B	rs10156191	-
AOC1	P19801	VAR_025079	p.Ser332Phe	LB/B	rs1049742	-
AOC1	P19801	VAR_025080	p.Met479Ile	LB/B	rs45558339	-
AOC1	P19801	VAR_025081	p.Asn659His	LB/B	rs35070995	-
AOC2	O75106	VAR_025022	p.Ile5Val	LB/B	rs34230945	-
AOC2	O75106	VAR_025023	p.Tyr22Cys	LB/B	rs34435306	-
AOC2	O75106	VAR_025024	p.Pro141Leu	LB/B	rs35833794	-
AOC2	O75106	VAR_025025	p.Arg273Gln	LB/B	rs35508987	-
AOC2	O75106	VAR_025026	p.Glu427Asp	LB/B	rs34351794	-
AOC3	Q16853	VAR_012064	p.Tyr317His	LB/B	rs438287	-
AOC3	Q16853	VAR_024343	p.Arg329Gln	LB/B	rs2229595	-
AOC3	Q16853	VAR_025027	p.His167Tyr	LB/B	rs2228470	-
AOC3	Q16853	VAR_025028	p.Ile371Thr	LB/B	rs35097308	-
AOC3	Q16853	VAR_025029	p.Ala408Ser	LB/B	rs35643019	-
AOC3	Q16853	VAR_025030	p.Arg426His	LB/B	rs33986943	-
AOC3	Q16853	VAR_025031	p.Arg441Trp	LB/B	rs2229596	-
AOC3	Q16853	VAR_025032	p.Ala582Thr	LB/B	rs34987927	-
AOC3	Q16853	VAR_025033	p.Gly700Ser	LB/B	rs477207	-
AOC3	Q16853	VAR_025034	p.Ala749Val	LB/B	rs34012919	-
AOC3	Q16853	VAR_025035	p.Thr5Arg	LB/B	rs33954211	-
AOC3	Q16853	VAR_052603	p.Arg78Gln	LB/B	rs402680	-
AOC3	Q16853	VAR_052604	p.Val171Met	LB/B	rs408038	-
AOC3	Q16853	VAR_052605	p.His203Arg	LB/B	rs630079	-
AOPEP	Q8N6M6	VAR_021511	p.Val179Ala	LB/B	rs16911679	-
AOPEP	Q8N6M6	VAR_057053	p.Val179Ile	LB/B	rs16911679	-
AOPEP	Q8N6M6	VAR_057054	p.Arg255Gln	LB/B	rs16911681	-
AOPEP	Q8N6M6	VAR_057055	p.Arg386Cys	LB/B	rs34557833	-
AOX1	Q06278	VAR_047517	p.Arg802Cys	LB/B	rs41309768	-
AOX1	Q06278	VAR_047518	p.His1297Arg	LB/B	rs3731722	-
AOX1	Q06278	VAR_061136	p.Gln314Arg	LB/B	rs58185012	-
AOX1	Q06278	VAR_070256	p.Arg921His	LB/B	rs56199635	-
AOX1	Q06278	VAR_070257	p.Asn1135Ser	LB/B	rs55754655	-
AOX1	Q06278	VAR_070258	p.Ser1271Leu	LB/B	rs141786030	-
AP1AR	Q63HQ0	VAR_050769	p.Thr297Ile	LB/B	rs34900583	-
AP1B1	Q10567	VAR_062816	p.Thr777Ala	LB/B	rs2857465	-
AP1B1	Q10567	VAR_083524	p.Cys144Arg	LP/P	-	Keratitis-ichthyosis-deafness syndrome, autosomal recessive (KIDAR) [MIM:242150]
AP1G1	O43747	VAR_013572	p.Pro685His	LB/B	rs904763	-
AP1G1	O43747	VAR_048194	p.Val195Gly	LB/B	rs36037071	-
AP1G2	O75843	VAR_024363	p.Ser377Phe	LB/B	rs12897422	-
AP1M1	Q9BXS5	VAR_036536	p.Arg303Gln	US	rs999036825	A breast cancer sample
AP1S3	Q96PC3	VAR_072545	p.Phe4Cys	LP/P	rs116107386	Psoriasis 15, pustular (PSORS15) [MIM:616106]
AP1S3	Q96PC3	VAR_072546	p.Gln17Lys	LB/B	rs750870128	-
AP1S3	Q96PC3	VAR_072547	p.Thr22Ala	LB/B	rs149183052	-
AP1S3	Q96PC3	VAR_072548	p.Thr32Ile	LB/B	rs78536455	-
AP1S3	Q96PC3	VAR_072549	p.Arg33Trp	LP/P	rs138292988	Psoriasis 15, pustular (PSORS15) [MIM:616106]
AP1S3	Q96PC3	VAR_072550	p.Leu79Val	LB/B	rs34353588	-
AP1S3	Q96PC3	VAR_072551	p.Ile83Thr	LB/B	rs202157374	-
AP1S3	Q96PC3	VAR_072552	p.Gln124Glu	LB/B	-	-

AP2A1 O95782 VAR_060544 p.Pro270Leu LB/B rs17851121 -
AP2M1 Q96CW1 VAR_082954 p.Arg170Trp LP/P rs1577059692 Intellectual developmental disorder, autosomal dominant 60, with seizures (MRD60) [MIM:618587]
AP2S1 P53680 VAR_069570 p.Arg15Cys LP/P rs397514498 Hypocalciuric hypercalcemia, familial 3 (HHC3) [MIM:600740]
AP2S1 P53680 VAR_069571 p.Arg15His LP/P rs397514499 Hypocalciuric hypercalcemia, familial 3 (HHC3) [MIM:600740]
AP2S1 P53680 VAR_069572 p.Arg15Leu LP/P rs397514499 Hypocalciuric hypercalcemia, familial 3 (HHC3) [MIM:600740]
AP3B1 O00203 VAR_011596 p.Leu580Arg LP/P rs121908904 Hermansky-Pudlak syndrome 2 (HPS2) [MIM:608233]
AP3B1 O00203 VAR_058404 p.Val585Glu LB/B rs6453373 -
AP3D1 O14617 VAR_033517 p.Gly541Arg LB/B rs34569645 -
AP3D1 O14617 VAR_033518 p.Ile1072Val LB/B rs25673 -
AP3S1 Q92572 VAR_059111 p.Pro158Leu LB/B rs7733604 -
AP4B1 Q9Y6B7 VAR_030804 p.Leu480Ser LB/B rs1217401 -
AP4E1 Q9UPM8 VAR_031621 p.Cys163Arg LB/B rs2306331 -
AP4E1 Q9UPM8 VAR_076619 p.Ile85Thr LB/B rs147005786 -
AP4E1 Q9UPM8 VAR_076620 p.Phe96Val US rs566579877 Stuttering, familial persistent 1 (STUT1) [MIM:184450]
AP4E1 Q9UPM8 VAR_076621 p.Thr145Ser LB/B rs200034177 -
AP4E1 Q9UPM8 VAR_076622 p.His205Asn US rs148499164 Stuttering, familial persistent 1 (STUT1) [MIM:184450]
AP4E1 Q9UPM8 VAR_076623 p.Arg211Gln LB/B rs750328226 -
AP4E1 Q9UPM8 VAR_076624 p.Asn264Ser LB/B rs145541719 -
AP4E1 Q9UPM8 VAR_076625 p.Asn311Ser US rs536656846 Stuttering, familial persistent 1 (STUT1) [MIM:184450]
AP4E1 Q9UPM8 VAR_076626 p.Ser326Phe US rs372479885 Stuttering, familial persistent 1 (STUT1) [MIM:184450]
AP4E1 Q9UPM8 VAR_076627 p.His384Arg US rs866266998 Stuttering, familial persistent 1 (STUT1) [MIM:184450]
AP4E1 Q9UPM8 VAR_076628 p.Ile426Leu LB/B rs148817957 -
AP4E1 Q9UPM8 VAR_076629 p.Ala475Val US rs200678853 Stuttering, familial persistent 1 (STUT1) [MIM:184450]
AP4E1 Q9UPM8 VAR_076630 p.Val517Ile LP/P rs760021635 Stuttering, familial persistent 1 (STUT1) [MIM:184450]
AP4E1 Q9UPM8 VAR_076631 p.Met542Val US rs542940704 Stuttering, familial persistent 1 (STUT1) [MIM:184450]
AP4E1 Q9UPM8 VAR_076632 p.Val618Ile LB/B rs142215198 -
AP4E1 Q9UPM8 VAR_076633 p.Ser623Pro US rs766696884 Stuttering, familial persistent 1 (STUT1) [MIM:184450]
AP4E1 Q9UPM8 VAR_076634 p.Ile706Lys LB/B rs865868636 -
AP4E1 Q9UPM8 VAR_076635 p.Glu801Lys LP/P rs556450190 Stuttering, familial persistent 1 (STUT1) [MIM:184450]
AP4E1 Q9UPM8 VAR_076636 p.Met813Val LB/B rs779094838 -
AP4E1 Q9UPM8 VAR_076637 p.Ser905Pro US rs780520338 Stuttering, familial persistent 1 (STUT1) [MIM:184450]
AP4E1 Q9UPM8 VAR_076638 p.Pro978Ser US rs141278078 Stuttering, familial persistent 1 (STUT1) [MIM:184450]

AP4E1 Q9UPM8 VAR_076639 p.Ile1080Val US rs767423538 Stuttering, familial persistent 1 (STUT1) [MIM:184450]
AP4E1 Q9UPM8 VAR_076640 p.Leu1089Arg US rs550237440 Stuttering, familial persistent 1 (STUT1) [MIM:184450]
AP4E1 Q9UPM8 VAR_076641 p.Arg1105Gln US rs139640763 Stuttering, familial persistent 1 (STUT1) [MIM:184450]
AP4E1 Q9UPM8 VAR_079485 p.Lys719Glu US - -
AP5M1 Q9H0R1 VAR_055484 p.Gly226Arg LB/B rs10137359 -
AP5M1 Q9H0R1 VAR_055485 p.Glu379Asp LB/B rs10140245 -
AP5Z1 O43299 VAR_049511 p.Ser94Cys LB/B rs11549839 -
APBA1 Q02410 VAR_050664 p.Ser184Ala LB/B rs34788368 -
APBA2 Q99767 VAR_050665 p.Leu311Pro LB/B rs8040932 -
APBA3 O96018 VAR_011822 p.Ile527Phe LB/B rs1045236 -
APBA3 O96018 VAR_020134 p.Lys276Thr LB/B rs3746119 -
APBA3 O96018 VAR_047952 p.Cys376Arg LB/B rs8102086 -
APBA3 O96018 VAR_050666 p.Trp154Leu LB/B rs35932323 -
APBB1 O00213 VAR_014444 p.Met327Val LB/B rs1800423 -
APBB1 O00213 VAR_014445 p.Asn396Ser LB/B rs1800425 -
APBB1IP Q7Z5R6 VAR_050098 p.Thr404Ala LB/B rs34081356 -
APBB1IP Q7Z5R6 VAR_059447 p.Ala617Thr LB/B rs7903226 -
APBB2 Q92870 VAR_069029 p.Arg179Gln LB/B rs4861358 -
APBB3 O95704 VAR_024702 p.Cys231Arg LB/B rs250430 -
APBB3 O95704 VAR_029518 p.Gly165Arg LB/B rs7715021 -
APC P25054 VAR_005032 p.Ser171Ile LP/P - Familial adenomatous polyposis 1 (FAP1) [MIM:175100]
APC P25054 VAR_005033 p.Arg414Cys LP/P rs137854567 Familial adenomatous polyposis 1 (FAP1) [MIM:175100]
APC P25054 VAR_005034 p.Ser784Thr LP/P - Familial adenomatous polyposis 1 (FAP1) [MIM:175100]
APC P25054 VAR_005035 p.Gly817Cys LP/P - Gastric cancer (GASC) [MIM:613659]
APC P25054 VAR_005036 p.Ile880Thr US rs1400295986 Colorectal carcinoma
APC P25054 VAR_005036 p.Ile880Thr LP/P rs1400295986 Gastric cancer (GASC) [MIM:613659]
APC P25054 VAR_005037 p.Ser906Tyr US - Colorectal tumor
APC P25054 VAR_005038 p.Glu911Gly US - Colorectal tumor
APC P25054 VAR_005038 p.Glu911Gly LP/P - Familial adenomatous polyposis 1 (FAP1) [MIM:175100]
APC P25054 VAR_005039 p.Asn942Asp LP/P - Gastric cancer (GASC) [MIM:613659]
APC P25054 VAR_005040 p.Tyr1027Cys US rs869312784 Colorectal tumor
APC P25054 VAR_005041 p.Asn1118Asp LB/B rs140493115 -
APC P25054 VAR_005042 p.Gly1120Glu LP/P rs28933379 Gastric cancer (GASC) [MIM:613659]
APC P25054 VAR_005043 p.Arg1171His LP/P rs372481703 Gastric cancer (GASC) [MIM:613659]
APC P25054 VAR_005044 p.Pro1176Leu LP/P - Familial adenomatous polyposis 1 (FAP1) [MIM:175100]
APC P25054 VAR_005045 p.Phe1197Ser LP/P - Gastric cancer (GASC) [MIM:613659]
APC P25054 VAR_005046 p.Ile1259Thr LP/P - Gastric cancer (GASC) [MIM:613659]
APC P25054 VAR_005047 p.Thr1292Met US rs371113837 Familial adenomatous polyposis 1 (FAP1) [MIM:175100]
APC P25054 VAR_005048 p.Ile1304Val LB/B rs770157475 -
APC P25054 VAR_005049 p.Ile1307Lys LB/B rs1801155 -
APC P25054 VAR_005050 p.Gly1312Glu LP/P - Gastric cancer (GASC) [MIM:613659]
APC P25054 VAR_005051 p.Thr1313Ala US rs863225349 Colorectal tumor
APC P25054 VAR_005051 p.Thr1313Ala LP/P rs863225349 Familial adenomatous polyposis 1 (FAP1) [MIM:175100]

APC P25054 VAR_005052 p.Val1326Ala LP/P - Gastric cancer (GASC) [MIM:613659]
APC P25054 VAR_005053 p.Arg1348Trp LP/P - Familial adenomatous polyposis 1 (FAP1) [MIM:175100]
APC P25054 VAR_005054 p.Asp1422His US - Colorectal tumor
APC P25054 VAR_005055 p.Gly2502Ser LB/B rs2229995 -
APC P25054 VAR_005056 p.Ser2621Cys US rs72541816 Familial adenomatous polyposis 1 (FAP1) [MIM:175100]
APC P25054 VAR_005057 p.Leu2839Phe LP/P rs876658156 Familial adenomatous polyposis 1 (FAP1) [MIM:175100]
APC P25054 VAR_008992 p.Arg1171Cys LB/B rs201830995 -
APC P25054 VAR_008993 p.Val1822Asp LB/B rs459552 -
APC P25054 VAR_008994 p.Ile2738Thr LB/B rs863224552 -
APC P25054 VAR_009613 p.Arg99Trp US rs139196838 Familial adenomatous polyposis 1 (FAP1) [MIM:175100]
APC P25054 VAR_009614 p.Ser722Gly LP/P - Familial adenomatous polyposis 1 (FAP1) [MIM:175100]
APC P25054 VAR_009615 p.Glu1057Gly US - -
APC P25054 VAR_009616 p.Ala1184Pro LP/P - Familial adenomatous polyposis 1 (FAP1) [MIM:175100]
APC P25054 VAR_009617 p.Glu1317Gln LB/B rs1801166 -
APC P25054 VAR_012975 p.Val890Ile US rs779998847 Colorectal carcinoma
APC P25054 VAR_012976 p.Ala1508Val US - Colorectal carcinoma from a patient with MMRCS
APC P25054 VAR_017653 p.Ala1296Val LP/P rs1291513037 Medulloblastoma (MDB) [MIM:155255]
APC P25054 VAR_017654 p.Val1472Ile LP/P rs878853445 Medulloblastoma (MDB) [MIM:155255]
APC P25054 VAR_017655 p.Ser1495Gly LP/P - Medulloblastoma (MDB) [MIM:155255]
APC P25054 VAR_020141 p.Thr1496Ser LB/B rs2229996 -
APC P25054 VAR_020142 p.Ser1973Thr LB/B rs4987109 -
APC P25054 VAR_035794 p.Ile1254Phe US - A colorectal cancer sample
APC P25054 VAR_053976 p.Pro870Ser LB/B rs33974176 -
APC P25054 VAR_053977 p.Arg1882Thr LB/B rs34157245 -
APC P25054 VAR_053978 p.Val2499Leu LB/B rs33941929 -
APC P25054 VAR_065133 p.Ser1395Cys LB/B rs137854578 -
APC2 O95996 VAR_037703 p.Ala562Ser US - A breast cancer sample
APC2 O95996 VAR_037704 p.Gly2003Ser US rs1288757495 A breast cancer sample
APC2 O95996 VAR_037705 p.Ser2241Ala LB/B rs265277 -
APC2 O95996 VAR_081224 p.His1921Asn LB/B - -
APCDD1 Q8J025 VAR_050667 p.Val150Ile LB/B rs3748415 -
APCDD1 Q8J025 VAR_063497 p.Leu9Arg LP/P rs267606659 Hypotrichosis 1 (HYPT1) [MIM:605389]
APCDD1L Q8NCL9 VAR_029625 p.Cys30Arg LB/B rs3946003 -
APCDD1L Q8NCL9 VAR_029626 p.Tyr80His LB/B rs7265854 -
APCDD1L Q8NCL9 VAR_029627 p.Arg261Cys LB/B rs16981999 -
APCDD1L Q8NCL9 VAR_050668 p.Arg83Gln LB/B rs7265902 -
APCS P02743 VAR_006054 p.Glu155Gly LB/B - -
APCS P02743 VAR_006055 p.Ser158Gly LB/B - -
APCS P02743 VAR_035814 p.Gly141Ser US - A breast cancer sample
APEH P13798 VAR_051580 p.Thr541Met LB/B rs3816877 -
APEX1 P27695 VAR_013455 p.Gln51His LB/B rs1048945 -
APEX1 P27695 VAR_014823 p.Ile64Val LB/B rs2307486 -
APEX1 P27695 VAR_019790 p.Asp148Glu LB/B rs1130409 -
APEX2 Q9UBZ4 VAR_023390 p.Arg141Cys LB/B rs2301416 -
APEX2 Q9UBZ4 VAR_048261 p.Arg141Trp LB/B rs2301416 -
APEX2 Q9UBZ4 VAR_064033 p.His269Tyr LB/B rs145122391 -

APEX2	Q9UBZ4	VAR_064034	p.Asn392His	LB/B	rs201964062	-
APH1B	Q8WW43	VAR_048315	p.Phe217Leu	LB/B	rs1047552	-
API5	Q9BZZ5	VAR_021519	p.Pro276Ser	LB/B	-	-
API5	Q9BZZ5	VAR_021520	p.Met300Val	LB/B	rs5743240	-
API5	Q9BZZ5	VAR_021521	p.Gly493Ser	LB/B	rs2862934	-
APIP	Q96GX9	VAR_026575	p.Arg7Trp	LB/B	rs2956114	-
APIP	Q96GX9	VAR_026576	p.His23Arg	LB/B	rs17850326	-
APIP	Q96GX9	VAR_026577	p.Cys76Tyr	LB/B	rs1977420	-
APIP	Q96GX9	VAR_026578	p.Met181Val	LB/B	rs17850327	-
APLF	Q8IW19	VAR_032299	p.Ile100Val	LB/B	rs11902811	-
APLF	Q8IW19	VAR_032300	p.Leu336Phe	LB/B	rs13404469	-
APLF	Q8IW19	VAR_061557	p.Ser224Thr	LB/B	rs35002937	-
APLNR	P35414	VAR_049375	p.Val300Ile	LB/B	rs7943508	-
APLP2	Q06481	VAR_022039	p.Asp632Asn	LB/B	rs3740881	-
APMAP	Q9HDC9	VAR_014128	p.Ile65Val	LB/B	rs12242	-
APMAP	Q9HDC9	VAR_055039	p.Arg282Gln	LB/B	rs35097515	-
APMAP	Q9HDC9	VAR_055040	p.Arg374Trp	LB/B	rs28364786	-
APOA1	P02647	VAR_000605	p.Pro27His	LB/B	rs121912720	-
APOA1	P02647	VAR_000606	p.Pro27Arg	LB/B	rs121912720	-
APOA1	P02647	VAR_000607	p.Pro28Arg	LB/B	rs121912721	-
APOA1	P02647	VAR_000608	p.Arg34Leu	LB/B	rs28929476	-
APOA1	P02647	VAR_000609	p.Gly50Arg	LP/P	rs28931574	Amyloidosis 8 (AMYL8) [MIM:105200]
APOA1	P02647	VAR_000610	p.Leu84Arg	LP/P	rs121912724	Amyloidosis 8 (AMYL8) [MIM:105200]
APOA1	P02647	VAR_000611	p.Asp113Glu	LB/B	rs150243849	-
APOA1	P02647	VAR_000612	p.Ala119Asp	US	-	-
APOA1	P02647	VAR_000613	p.Asp127Asn	LB/B	rs921646982	-
APOA1	P02647	VAR_000615	p.Lys131Met	LB/B	rs4882	-
APOA1	P02647	VAR_000616	p.Trp132Arg	US	-	-
APOA1	P02647	VAR_000617	p.Glu134Lys	US	-	-
APOA1	P02647	VAR_000618	p.Glu160Lys	LB/B	rs121912718	-
APOA1	P02647	VAR_000619	p.Glu163Gly	LB/B	rs758509542	-
APOA1	P02647	VAR_000620	p.Pro167Arg	LB/B	rs121912719	-
APOA1	P02647	VAR_000621	p.Leu168Arg	US	-	-
APOA1	P02647	VAR_000622	p.Glu171Val	LB/B	rs1015066427	-
APOA1	P02647	VAR_000623	p.Pro189Arg	LB/B	rs121912722	-
APOA1	P02647	VAR_000624	p.Arg197Cys	LB/B	rs28931573	-
APOA1	P02647	VAR_000625	p.Glu222Lys	LB/B	rs121912717	-
APOA1	P02647	VAR_014609	p.Arg184Pro	LB/B	rs5078	-
APOA1	P02647	VAR_016189	p.Asp126His	LB/B	rs5077	-
APOA1	P02647	VAR_017017	p.Thr92Ile	LB/B	rs766422306	-
APOA1	P02647	VAR_021362	p.Val180Glu	LB/B	rs121912727	-
APOA1	P02647	VAR_025445	p.Ala61Thr	LB/B	rs12718465	-
APOA1	P02647	VAR_074073	p.Arg173Ser	US	-	-
APOA1	P02647	VAR_083310	p.Arg173Pro	US	-	Amyloidosis 8 (AMYL8) [MIM:105200]
APOA1	P02647	VAR_083311	p.Leu198Ser	LP/P	-	Amyloidosis 8 (AMYL8) [MIM:105200]
APOA1	P02647	VAR_083312	p.Ala199Pro	US	-	Amyloidosis 8 (AMYL8) [MIM:105200]
APOA4	P06727	VAR_000626	p.Val13Met	LB/B	rs12721041	-
APOA4	P06727	VAR_000627	p.Glu44Lys	US	-	-
APOA4	P06727	VAR_000628	p.Ser147Asn	LB/B	rs5104	-

APOA4	P06727	VAR_000629	p.Ala161Ser	LB/B	rs12721043	-
APOA4	P06727	VAR_000630	p.Ser178Leu	LB/B	rs1181852696	-
APOA4	P06727	VAR_000631	p.Glu185Lys	LB/B	rs201861136	-
APOA4	P06727	VAR_000632	p.Lys187Glu	LB/B	rs773492545	-
APOA4	P06727	VAR_000633	p.Glu250Lys	LB/B	rs121909576	-
APOA4	P06727	VAR_000634	p.Arg264Gln	LB/B	rs2238008	-
APOA4	P06727	VAR_000635	p.Arg305Cys	LB/B	rs150264487	-
APOA4	P06727	VAR_000636	p.Thr367Ser	LB/B	rs675	-
APOA4	P06727	VAR_000637	p.Gln380His	LB/B	rs5110	-
APOA4	P06727	VAR_014610	p.Gly74Ser	LB/B	rs5102	-
APOA4	P06727	VAR_014611	p.Val307Leu	LB/B	rs5108	-
APOA4	P06727	VAR_025443	p.Arg279Lys	LB/B	rs1042372	-
APOA4	P06727	VAR_025444	p.Gln77His	LB/B	rs12721042	-
APOA5	Q6Q788	VAR_021165	p.Ser19Trp	LB/B	rs3135506	-
APOA5	Q6Q788	VAR_021166	p.Val153Met	LB/B	rs3135507	-
APOA5	Q6Q788	VAR_021167	p.Gly185Cys	LB/B	rs2075291	-
APOA5	Q6Q788	VAR_035124	p.Asp37Glu	LB/B	rs34282181	-
APOB	P04114	VAR_005016	p.Phe1437Leu	LB/B	rs1801697	-
APOB	P04114	VAR_005017	p.Asn1914Ser	LB/B	rs1801699	-
APOB	P04114	VAR_005018	p.His1923Arg	LB/B	rs533617	-
APOB	P04114	VAR_005019	p.Glu2566Lys	LB/B	rs1801696	-
APOB	P04114	VAR_005020	p.Pro2739Leu	LB/B	rs676210	-
APOB	P04114	VAR_005021	p.Ala3121Thr	LB/B	rs1801694	-
APOB	P04114	VAR_005022	p.Asp3319His	LB/B	rs1042021	-
APOB	P04114	VAR_005023	p.Thr3427Lys	LB/B	rs1042022	-
APOB	P04114	VAR_005024	p.Gln3432Glu	LB/B	rs1042023	-
APOB	P04114	VAR_005025	p.Arg3527Gln	LP/P	rs5742904	Hypercholesterolemia, familial, 2 (FHCL2) [MIM:144010]
APOB	P04114	VAR_005026	p.Arg3558Cys	LP/P	rs12713559	Hypercholesterolemia, familial, 2 (FHCL2) [MIM:144010]
APOB	P04114	VAR_005027	p.Val3921Ile	LB/B	rs72654409	-
APOB	P04114	VAR_005028	p.Thr3945Ala	LB/B	rs1801698	-
APOB	P04114	VAR_005029	p.Val4128Met	LB/B	rs1801703	-
APOB	P04114	VAR_005030	p.Ser4338Asn	LB/B	rs1042034	-
APOB	P04114	VAR_005031	p.Ala4481Thr	LB/B	rs1801695	-
APOB	P04114	VAR_016184	p.Thr98Ile	LB/B	rs1367117	-
APOB	P04114	VAR_016185	p.Val733Ile	LB/B	rs1800476	-
APOB	P04114	VAR_016186	p.Arg3638Gln	LB/B	rs1801701	-
APOB	P04114	VAR_016187	p.Glu4181Lys	LB/B	rs1042031	-
APOB	P04114	VAR_016188	p.Arg4270Thr	LB/B	rs1801702	-
APOB	P04114	VAR_019827	p.Lys273Asn	LB/B	-	-
APOB	P04114	VAR_019828	p.Ala618Val	LB/B	rs679899	-
APOB	P04114	VAR_019829	p.Gln1218Glu	LB/B	rs1041956	-
APOB	P04114	VAR_019830	p.Leu2092Val	LB/B	rs1041960	-
APOB	P04114	VAR_019831	p.Ala2365Thr	LB/B	rs1041971	-
APOB	P04114	VAR_019832	p.Leu2680Gln	LB/B	rs1042013	-
APOB	P04114	VAR_019833	p.Ile3732Thr	LB/B	rs1042025	-
APOB	P04114	VAR_019834	p.Phe3949Leu	LB/B	rs1042027	-
APOB	P04114	VAR_019835	p.Tyr3964Phe	LB/B	rs1126468	-

APOB P04114 VAR_020135 p.Pro554Leu LB/B rs12714214 -
APOB P04114 VAR_020136 p.Val730Ile LB/B rs12691202 -
APOB P04114 VAR_020137 p.Thr741Asn LB/B rs12714192 -
APOB P04114 VAR_020138 p.Ala2456Asp LB/B rs12713675 -
APOB P04114 VAR_020139 p.Ser3294Pro LB/B rs12720855 -
APOB P04114 VAR_020140 p.Thr4484Met LB/B rs12713450 -
APOB P04114 VAR_022036 p.Tyr103His LB/B rs9282603 -
APOB P04114 VAR_022037 p.Pro145Ser LB/B rs6752026 -
APOB P04114 VAR_022038 p.Asn2785His LB/B rs2163204 -
APOB P04114 VAR_022610 p.Arg490Trp LP/P rs771541567 Hypobetalipoproteinemia, familial, 1 (FHBL1) [MIM:615558]
APOB P04114 VAR_022611 p.Arg1128His LB/B rs12713843 -
APOB P04114 VAR_029341 p.Ile408Thr LB/B rs12714225 -
APOB P04114 VAR_029342 p.Pro877Leu LB/B rs12714097 -
APOB P04114 VAR_029343 p.Gly1086Ser LB/B rs12720801 -
APOB P04114 VAR_029344 p.Asp1113His LB/B rs12713844 -
APOB P04114 VAR_029345 p.Arg1388His LB/B rs13306187 -
APOB P04114 VAR_029346 p.Asp2299His LB/B rs12713681 -
APOB P04114 VAR_029347 p.His3182Asn LB/B rs12720848 -
APOB P04114 VAR_029348 p.Ser3279Gly LB/B rs12720854 -
APOB P04114 VAR_029349 p.Ser3801Thr LB/B rs12713540 -
APOB P04114 VAR_029350 p.Val4394Ala LB/B rs12720843 -
APOB P04114 VAR_035795 p.Phe2564Cys US - A colorectal cancer sample
APOB P04114 VAR_056737 p.Thr194Met LB/B rs13306198 -
APOB P04114 VAR_056738 p.Pro955Ser LB/B rs13306206 -
APOB P04114 VAR_059582 p.Ile2313Val LB/B rs584542 -
APOB P04114 VAR_061558 p.Tyr1422Cys LB/B rs568413 -
APOB P04114 VAR_067278 p.Ser1613Thr LB/B rs61742247 -
APOB P04114 VAR_067279 p.Ile3835Leu LB/B rs776119459 -
APOB P04114 VAR_067280 p.Ile4314Val LB/B rs72654423 -
APOB P04114 VAR_067281 p.Ile4482Val LB/B rs142702699 -
APOB P04114 VAR_068911 p.Glu1670Asp LB/B rs773681906 -
APOB P04114 VAR_068912 p.Ile2037Asn LB/B - -
APOB P04114 VAR_076538 p.Ala251Thr LB/B rs61741625 -
APOB P04114 VAR_076539 p.Val952Leu US - Hypobetalipoproteinemia, familial, 1 (FHBL1) [MIM:615558]
APOBEC1 P41238 VAR_013779 p.Met80Ile LB/B rs2302515 -
APOBEC1 P41238 VAR_048720 p.Arg236Lys LB/B rs12820011 -
APOBEC2 Q9Y235 VAR_024406 p.Ile136Thr LB/B rs2076472 -
APOBEC3A P31941 VAR_048721 p.Thr19Ala LB/B rs17000556 -
APOBEC3B Q9UH17 VAR_018142 p.Lys62Glu LB/B rs2076109 -
APOBEC3B Q9UH17 VAR_018143 p.Pro98Leu LB/B rs2076110 -
APOBEC3B Q9UH17 VAR_018144 p.Thr146Lys LB/B rs5995649 -
APOBEC3B Q9UH17 VAR_033455 p.Ser109Ala LB/B rs17000697 -
APOBEC3B Q9UH17 VAR_048722 p.Arg351His LB/B rs1053813 -
APOBEC3F Q8IUX4 VAR_018145 p.Gln61Leu LB/B rs2076109 -
APOBEC3F Q8IUX4 VAR_018146 p.Pro97Leu LB/B rs201939303 -
APOBEC3F Q8IUX4 VAR_018147 p.Ala108Ser LB/B rs2020390 -
APOBEC3F Q8IUX4 VAR_018148 p.Val231Ile LB/B rs2076101 -

APOBEC3F	Q8IUX4	VAR_025058	p.Ala178Thr	LB/B	rs34182094	-
APOBEC3F	Q8IUX4	VAR_025059	p.Tyr307Cys	LB/B	rs12157816	-
APOBEC3F	Q8IUX4	VAR_038355	p.Arg48Pro	LB/B	rs35053197	-
APOBEC3G	Q9HC16	VAR_017837	p.His186Arg	LB/B	rs8177832	-
APOBEC3G	Q9HC16	VAR_025060	p.Gln275Glu	LB/B	rs17496046	-
APOBEC3G	Q9HC16	VAR_048723	p.Arg256His	LB/B	rs17000736	-
APOBEC3H	Q6NTF7	VAR_032835	p.Arg18Leu	LB/B	rs139293	-
APOBEC3H	Q6NTF7	VAR_032836	p.Gly105Arg	LB/B	rs139297	-
APOBEC3H	Q6NTF7	VAR_032837	p.Lys121Glu	LB/B	rs139298	-
APOBEC3H	Q6NTF7	VAR_032838	p.Lys121Asn	LB/B	rs139299	-
APOBEC3H	Q6NTF7	VAR_032839	p.Glu178Asp	LB/B	rs139302	-
APOBEC3H	Q6NTF7	VAR_067444	p.Glu140Lys	LB/B	rs139300	-
APOBEC4	Q8WW27	VAR_026639	p.Ser75Phe	LB/B	rs16861394	-
APOBEC4	Q8WW27	VAR_026640	p.Phe271Ser	LB/B	rs1174658	-
APOBEC4	Q8WW27	VAR_026641	p.Pro275Ser	LB/B	rs10911391	-
APOBEC4	Q8WW27	VAR_026642	p.Lys331Glu	LB/B	rs1174657	-
APOBEC4	Q8WW27	VAR_026643	p.Val345Met	LB/B	rs10911390	-
APOBEC4	Q8WW27	VAR_048724	p.Asp300Gly	LB/B	rs16861381	-
APOBR	Q0VD83	VAR_042432	p.Pro428Ala	LB/B	rs180743	-
APOC1	P02654	VAR_014183	p.Ile16Met	LB/B	rs5112	-
APOC1	P02654	VAR_029011	p.Thr71Ser	LB/B	rs142372275	-
APOC2	P02655	VAR_000639	p.Lys41Thr	LB/B	rs120074114	-
APOC2	P02655	VAR_000640	p.Trp48Arg	LP/P	rs120074115	Hyperlipoproteinemia 1B (HLPP1B) [MIM:207750]
APOC2	P02655	VAR_000641	p.Glu60Lys	LB/B	rs5122	-
APOC2	P02655	VAR_000642	p.Lys77Gln	LB/B	rs5126	-
APOC3	P02656	VAR_000643	p.Lys78Glu	LP/P	rs121918382	Hyperalphalipoproteinemia 2 (HALP2) [MIM:614028]
APOC3	P02656	VAR_000644	p.Thr94Ala	LB/B	rs121918381	-
APOC4	P55056	VAR_012068	p.Leu96Arg	LB/B	rs5167	-
APOC4	P55056	VAR_012069	p.Gln126Leu	LB/B	rs5168	-
APOC4	P55056	VAR_012081	p.Leu36Pro	LB/B	rs1132899	-
APOC4	P55056	VAR_012082	p.Gly52Asp	LB/B	rs12691089	-
APOC4	P55056	VAR_036540	p.Pro75Gln	US	-	A breast cancer sample
APOD	P05090	VAR_011931	p.Phe15Ser	LB/B	rs5952	-
APOD	P05090	VAR_011932	p.Ser115Leu	LB/B	rs5954	-
APOD	P05090	VAR_011933	p.Thr178Lys	LB/B	rs5955	-
APOE	P02649	VAR_000645	p.Glu21Lys	LB/B	rs121918392	-
APOE	P02649	VAR_000646	p.Glu31Lys	LP/P	rs201672011	Hyperlipoproteinemia 3 (HLPP3) [MIM:617347]
APOE	P02649	VAR_000647	p.Leu46Pro	US	rs769452	-
APOE	P02649	VAR_000648	p.Thr60Ala	LB/B	rs28931576	-
APOE	P02649	VAR_000649	p.Gln99Lys	LB/B	rs1180612218	-
APOE	P02649	VAR_000650	p.Pro102Arg	LB/B	rs11083750	-
APOE	P02649	VAR_000651	p.Ala117Thr	LB/B	rs28931577	-
APOE	P02649	VAR_000652	p.Cys130Arg	LP/P	rs429358	Alzheimer disease 2 (AD2) [MIM:104310]
APOE	P02649	VAR_000652	p.Cys130Arg	LP/P	rs429358	Hyperlipoproteinemia 3 (HLPP3) [MIM:617347]
APOE	P02649	VAR_000653	p.Gly145Asp	US	rs267606664	-
APOE	P02649	VAR_000655	p.Arg152Gln	LB/B	rs28931578	-
APOE	P02649	VAR_000656	p.Arg154Ser	LP/P	rs121918393	Hyperlipoproteinemia 3 (HLPP3) [MIM:617347]
APOE	P02649	VAR_000657	p.Arg154Cys	LP/P	rs121918393	Hyperlipoproteinemia 3 (HLPP3) [MIM:617347]

APOE	P02649	VAR_000658	p.Arg160Cys	LP/P	rs387906567	Hyperlipoproteinemia 3 (HLPP3) [MIM:617347]
APOE	P02649	VAR_000659	p.Arg163Cys	LP/P	rs769455	Hyperlipoproteinemia 3 (HLPP3) [MIM:617347]
APOE	P02649	VAR_000660	p.Arg163His	US	rs121918397	Hyperlipoproteinemia 3 (HLPP3) [MIM:617347]
APOE	P02649	VAR_000661	p.Lys164Gln	LP/P	rs121918394	Hyperlipoproteinemia 3 (HLPP3) [MIM:617347]
APOE	P02649	VAR_000662	p.Lys164Glu	LP/P	rs121918394	Hyperlipoproteinemia 3 (HLPP3) [MIM:617347]
APOE	P02649	VAR_000663	p.Ala170Pro	LB/B	rs267606662	-
APOE	P02649	VAR_000664	p.Arg176Cys	LP/P	rs7412	Hyperlipoproteinemia 3 (HLPP3) [MIM:617347]
APOE	P02649	VAR_000665	p.Arg242Gln	LB/B	rs267606663	-
APOE	P02649	VAR_000666	p.Arg246Cys	LB/B	rs121918395	-
APOE	P02649	VAR_000667	p.Val254Glu	LB/B	rs199768005	-
APOE	P02649	VAR_000669	p.Arg269Gly	LB/B	rs267606661	-
APOE	P02649	VAR_000670	p.Leu270Glu	US	-	-
APOE	P02649	VAR_000671	p.Arg292His	LB/B	rs121918398	-
APOE	P02649	VAR_000672	p.Ser314Arg	LB/B	rs28931579	-
APOE	P02649	VAR_014114	p.Gln64His	LB/B	rs370594287	-
APOE	P02649	VAR_016789	p.Ala124Val	LB/B	rs937063425	-
APOE	P02649	VAR_042734	p.Arg43Cys	LP/P	rs121918399	Lipoprotein glomerulopathy (LPG) [MIM:611771]
APOE	P02649	VAR_042735	p.Arg163Pro	LP/P	rs121918397	Lipoprotein glomerulopathy (LPG) [MIM:611771]
APOF	Q13790	VAR_055520	p.Ala178Gly	LB/B	rs11575216	-
APOH	P02749	VAR_000673	p.Val266Leu	LB/B	rs4581	-
APOH	P02749	VAR_008169	p.Ser107Asn	LB/B	rs1801692	-
APOH	P02749	VAR_008170	p.Cys325Gly	LB/B	rs1801689	-
APOH	P02749	VAR_008171	p.Trp335Ser	LB/B	rs1801690	-
APOH	P02749	VAR_019155	p.Arg154His	LB/B	rs8178847	-
APOH	P02749	VAR_048316	p.Val5Ala	LB/B	rs3826358	-
APOL1	O14791	VAR_011383	p.Glu150Lys	LB/B	rs2239785	-
APOL1	O14791	VAR_011384	p.Met228Ile	LB/B	rs136175	-
APOL1	O14791	VAR_011385	p.Arg255Lys	LB/B	rs136176	-
APOL1	O14791	VAR_036568	p.Ile188Thr	US	-	A breast cancer sample
APOL1	O14791	VAR_046641	p.Asp337Asn	LB/B	rs16996616	-
APOL1	O14791	VAR_061995	p.Ile384Met	LP/P	rs60910145	Focal segmental glomerulosclerosis 4 (FSGS4) [MIM:612551]
APOL1	O14791	VAR_063598	p.Ser342Gly	LP/P	rs73885319	Focal segmental glomerulosclerosis 4 (FSGS4) [MIM:612551]
APOL2	Q9BQE5	VAR_012978	p.Ile245Val	LB/B	rs132760	-
APOL2	Q9BQE5	VAR_024366	p.Arg182Cys	LB/B	rs7285167	-
APOL3	O95236	VAR_047488	p.Ala135Val	LB/B	rs6000152	-
APOL3	O95236	VAR_053007	p.Ser39Arg	LB/B	rs132653	-
APOL4	Q9BPW4	VAR_053008	p.Ile9Val	LB/B	rs132736	-
APOL4	Q9BPW4	VAR_053009	p.Val12Leu	LB/B	rs80587	-
APOL4	Q9BPW4	VAR_053010	p.Met159Val	LB/B	rs132700	-
APOL4	Q9BPW4	VAR_059966	p.Arg223His	LB/B	rs2227168	-
APOL4	Q9BPW4	VAR_059967	p.Ala319Glu	LB/B	rs6000173	-
APOL4	Q9BPW4	VAR_059968	p.Ser326Leu	LB/B	rs6000172	-
APOL5	Q9BWW9	VAR_020355	p.Thr272Met	LB/B	rs2076671	-
APOL5	Q9BWW9	VAR_020356	p.Thr323Met	LB/B	rs2076672	-
APOL5	Q9BWW9	VAR_020357	p.Ser406Cys	LB/B	rs2076673	-
APOL5	Q9BWW9	VAR_053012	p.Glu81Lys	LB/B	rs5999985	-
APOL6	Q9BWW8	VAR_053013	p.Asn173Lys	LB/B	rs5999923	-

APP P05067 VAR_000016 p.Ala692Gly LP/P rs63750671 Alzheimer disease 1 (AD1) [MIM:104300]
APP P05067 VAR_000017 p.Glu693Gln LP/P rs63750579 Cerebral amyloid angiopathy, APP-related (CAA-APP) [MIM:605714]
APP P05067 VAR_000018 p.Ala713Val US rs1800557 -
APP P05067 VAR_000019 p.Ala713Thr LP/P rs63750066 Alzheimer disease 1 (AD1) [MIM:104300]
APP P05067 VAR_000020 p.Ile716Val LP/P rs63750399 Alzheimer disease 1 (AD1) [MIM:104300]
APP P05067 VAR_000021 p.Val717Ile LP/P rs63750264 Alzheimer disease 1 (AD1) [MIM:104300]
APP P05067 VAR_000022 p.Val717Gly LP/P rs63749964 Alzheimer disease 1 (AD1) [MIM:104300]
APP P05067 VAR_000023 p.Val717Phe LP/P rs63750264 Alzheimer disease 1 (AD1) [MIM:104300]
APP P05067 VAR_010107 p.Glu665Asp LB/B rs63750363 -
APP P05067 VAR_010108 p.Val715Met LP/P rs63750734 Alzheimer disease 1 (AD1) [MIM:104300]
APP P05067 VAR_010109 p.Leu723Pro LP/P rs63751122 Alzheimer disease 1 (AD1) [MIM:104300]
APP P05067 VAR_014215 p.Glu693Gly LP/P rs63751039 Alzheimer disease 1 (AD1) [MIM:104300]
APP P05067 VAR_014216 p.Glu693Lys LP/P rs63750579 Cerebral amyloid angiopathy, APP-related (CAA-APP) [MIM:605714]
APP P05067 VAR_014217 p.Asp694Asn LP/P rs63749810 Cerebral amyloid angiopathy, APP-related (CAA-APP) [MIM:605714]
APP P05067 VAR_014218 p.Thr714Ile LP/P rs63750973 Alzheimer disease 1 (AD1) [MIM:104300]
APP P05067 VAR_014219 p.Val717Leu LP/P rs63750264 Alzheimer disease 1 (AD1) [MIM:104300]
APP P05067 VAR_022315 p.Glu501Lys LB/B rs45588932 -
APP P05067 VAR_032276 p.Leu705Val LP/P rs63750921 Cerebral amyloid angiopathy, APP-related (CAA-APP) [MIM:605714]
APP P05067 VAR_032277 p.Thr714Ala LP/P rs63750643 Alzheimer disease 1 (AD1) [MIM:104300]
APP P05067 VAR_044424 p.Asp678Asn LP/P rs63750064 Alzheimer disease 1 (AD1) [MIM:104300]
APPBP2 Q92624 VAR_052606 p.Ser561Asn LB/B rs34146848 -
APPL1 Q9UKG1 VAR_035909 p.Glu643Gln US - A breast cancer sample
APPL1 Q9UKG1 VAR_050958 p.Ala108Val LB/B rs4381906 -
APPL1 Q9UKG1 VAR_050959 p.Glu700Gly LB/B rs11544593 -
APPL1 Q9UKG1 VAR_075857 p.Asp94Asn LP/P rs796065047 Maturity-onset diabetes of the young 14 (MODY14) [MIM:616511]
APPL2 Q8NEU8 VAR_021505 p.Ala433Val LB/B rs2272495 -
APRG1 Q8IVJ8 VAR_032538 p.His107Tyr LB/B rs17266511 -
APRT P07741 VAR_006747 p.Asp65Val LP/P rs104894506 Adenine phosphoribosyltransferase deficiency (APRTD) [MIM:614723]
APRT P07741 VAR_006748 p.Leu110Pro LP/P rs104894508 Adenine phosphoribosyltransferase deficiency (APRTD) [MIM:614723]
APRT P07741 VAR_006749 p.Met136Thr LP/P rs28999113 Adenine phosphoribosyltransferase deficiency (APRTD) [MIM:614723]
APRT P07741 VAR_019055 p.Gln121Arg LB/B rs8191494 -
APRT P07741 VAR_022608 p.Val150Phe LP/P rs281860266 Adenine phosphoribosyltransferase deficiency (APRTD) [MIM:614723]
APRT P07741 VAR_022609 p.Cys153Arg LP/P - Adenine phosphoribosyltransferase deficiency (APRTD) [MIM:614723]
APRT P07741 VAR_069049 p.Leu33Pro LP/P - Adenine phosphoribosyltransferase deficiency (APRTD) [MIM:614723]
APRT P07741 VAR_069050 p.Val84Met LP/P rs200392753 Adenine phosphoribosyltransferase deficiency (APRTD) [MIM:614723]
APRT P07741 VAR_069051 p.Gly133Asp LP/P - Adenine phosphoribosyltransferase deficiency (APRTD) [MIM:614723]

APTX Q7Z2E3 VAR_018794 p.Lys211Gln LP/P - Ataxia-oculomotor apraxia syndrome (AOA) [MIM:208920]
APTX Q7Z2E3 VAR_018795 p.Ala212Val LP/P rs748165574 Ataxia-oculomotor apraxia syndrome (AOA) [MIM:208920]
APTX Q7Z2E3 VAR_018796 p.Arg213His LP/P rs150886026 Ataxia-oculomotor apraxia syndrome (AOA) [MIM:208920]
APTX Q7Z2E3 VAR_018797 p.His215Arg LP/P rs121908133 Ataxia-oculomotor apraxia syndrome (AOA) [MIM:208920]
APTX Q7Z2E3 VAR_018798 p.Pro220Leu LP/P rs121908131 Ataxia-oculomotor apraxia syndrome (AOA) [MIM:208920]
APTX Q7Z2E3 VAR_018799 p.Val277Gly LP/P rs121908132 Ataxia-oculomotor apraxia syndrome (AOA) [MIM:208920]
APTX Q7Z2E3 VAR_018800 p.Asp281Gly LP/P - Ataxia-oculomotor apraxia syndrome (AOA) [MIM:208920]
APTX Q7Z2E3 VAR_018801 p.Trp293Arg LP/P rs773393618 Ataxia-oculomotor apraxia syndrome (AOA) [MIM:208920]
APTX Q7Z2E3 VAR_025365 p.Leu237Pro LP/P rs267606665 Ataxia-oculomotor apraxia syndrome (AOA) [MIM:208920]
AQP1 P29972 VAR_004400 p.Ala45Val LB/B rs28362692 -
AQP1 P29972 VAR_013279 p.Pro38Leu LB/B rs104894004 -
AQP1 P29972 VAR_022318 p.Gly165Asp LB/B rs28362731 -
AQP10 Q96PS8 VAR_033519 p.Arg15Gln LB/B rs6668968 -
AQP10 Q96PS8 VAR_050063 p.His123Tyr LB/B rs6685323 -
AQP11 Q8NBQ7 VAR_020446 p.Gly102Ser LB/B rs2276415 -
AQP2 P41181 VAR_004401 p.Gly64Arg LP/P rs104894326 Diabetes insipidus, nephrogenic, autosomal (ANDI) [MIM:125800]
AQP2 P41181 VAR_004402 p.Arg187Cys LP/P rs104894328 Diabetes insipidus, nephrogenic, autosomal (ANDI) [MIM:125800]
AQP2 P41181 VAR_004403 p.Ser216Pro LP/P rs104894329 Diabetes insipidus, nephrogenic, autosomal (ANDI) [MIM:125800]
AQP2 P41181 VAR_015239 p.Leu22Val LP/P rs104894336 Diabetes insipidus, nephrogenic, autosomal (ANDI) [MIM:125800]
AQP2 P41181 VAR_015240 p.Leu28Pro LP/P - Diabetes insipidus, nephrogenic, autosomal (ANDI) [MIM:125800]
AQP2 P41181 VAR_015241 p.Ala47Val LP/P rs995684800 Diabetes insipidus, nephrogenic, autosomal (ANDI) [MIM:125800]
AQP2 P41181 VAR_015242 p.Asn68Ser LP/P rs104894331 Diabetes insipidus, nephrogenic, autosomal (ANDI) [MIM:125800]
AQP2 P41181 VAR_015243 p.Val71Met LP/P rs149659001 Diabetes insipidus, nephrogenic, autosomal (ANDI) [MIM:125800]
AQP2 P41181 VAR_015244 p.Thr125Met LP/P rs104894333 Diabetes insipidus, nephrogenic, autosomal (ANDI) [MIM:125800]
AQP2 P41181 VAR_015245 p.Thr126Met LP/P rs104894330 Diabetes insipidus, nephrogenic, autosomal (ANDI) [MIM:125800]
AQP2 P41181 VAR_015246 p.Ala147Thr LP/P rs104894334 Diabetes insipidus, nephrogenic, autosomal (ANDI) [MIM:125800]
AQP2 P41181 VAR_015247 p.Val168Met LP/P rs755694590 Diabetes insipidus, nephrogenic, autosomal (ANDI) [MIM:125800]

AQP2 P41181 VAR_015248 p.Gly175Arg LP/P rs104894335 Diabetes insipidus, nephrogenic, autosomal (ANDI) [MIM:125800]
AQP2 P41181 VAR_015249 p.Cys181Trp LP/P rs104894337 Diabetes insipidus, nephrogenic, autosomal (ANDI) [MIM:125800]
AQP2 P41181 VAR_015250 p.Pro185Ala LP/P rs761713751 Diabetes insipidus, nephrogenic, autosomal (ANDI) [MIM:125800]
AQP2 P41181 VAR_015251 p.Ala190Thr LP/P rs104894341 Diabetes insipidus, nephrogenic, autosomal (ANDI) [MIM:125800]
AQP2 P41181 VAR_015252 p.Val194Ile LB/B rs772051028 -
AQP2 P41181 VAR_015253 p.Trp202Cys LP/P - Diabetes insipidus, nephrogenic, autosomal (ANDI) [MIM:125800]
AQP2 P41181 VAR_015254 p.Glu258Lys LP/P rs104894332 Diabetes insipidus, nephrogenic, autosomal (ANDI) [MIM:125800]
AQP2 P41181 VAR_015255 p.Pro262Leu LP/P rs104894339 Diabetes insipidus, nephrogenic, autosomal (ANDI) [MIM:125800]
AQP2 P41181 VAR_015256 p.Gln57Pro LP/P rs28931580 Diabetes insipidus, nephrogenic, autosomal (ANDI) [MIM:125800]
AQP2 P41181 VAR_015257 p.Gly100Val LP/P rs104894338 Diabetes insipidus, nephrogenic, autosomal (ANDI) [MIM:125800]
AQP2 P41181 VAR_037577 p.Leu121Phe LB/B rs11169226 -
AQP2 P41181 VAR_062585 p.Ala70Asp LP/P - Diabetes insipidus, nephrogenic, autosomal (ANDI) [MIM:125800]
AQP2 P41181 VAR_062586 p.Gly100Arg LP/P rs1303076207 Diabetes insipidus, nephrogenic, autosomal (ANDI) [MIM:125800]
AQP2 P41181 VAR_062587 p.Gly180Ser LP/P rs147039983 Diabetes insipidus, nephrogenic, autosomal (ANDI) [MIM:125800]
AQP2 P41181 VAR_062588 p.Arg187His LP/P rs193922495 Diabetes insipidus, nephrogenic, autosomal (ANDI) [MIM:125800]
AQP2 P41181 VAR_062589 p.Arg254Leu LP/P - Diabetes insipidus, nephrogenic, autosomal (ANDI) [MIM:125800]
AQP2 P41181 VAR_062590 p.Arg254Gln LP/P - Diabetes insipidus, nephrogenic, autosomal (ANDI) [MIM:125800]
AQP2 P41181 VAR_071370 p.Thr108Met LP/P rs1468828294 Diabetes insipidus, nephrogenic, autosomal (ANDI) [MIM:125800]
AQP3 Q92482 VAR_025089 p.Val43Met LB/B rs34942735 -
AQP5 P55064 VAR_070442 p.Ala38Glu LP/P rs398123054 Keratoderma, palmoplantar, Bothnian type (PPKB) [MIM:600231]
AQP5 P55064 VAR_070443 p.Ile45Ser LP/P rs398123055 Keratoderma, palmoplantar, Bothnian type (PPKB) [MIM:600231]
AQP5 P55064 VAR_070444 p.Asn123Asp LP/P rs398123057 Keratoderma, palmoplantar, Bothnian type (PPKB) [MIM:600231]
AQP5 P55064 VAR_070445 p.Ile177Phe LP/P rs398123056 Keratoderma, palmoplantar, Bothnian type (PPKB) [MIM:600231]
AQP5 P55064 VAR_070446 p.Arg188Cys LP/P rs368292687 Keratoderma, palmoplantar, Bothnian type (PPKB) [MIM:600231]
AQP6 Q13520 VAR_047233 p.Val234Ile LB/B rs17124220 -
AQP7 O14520 VAR_061343 p.Leu38Val LB/B rs2381003 -
AQP7 O14520 VAR_061344 p.Lys63Thr LB/B rs4008658 -
AQP7 O14520 VAR_067253 p.Arg12Cys LB/B rs139297434 -

AQP7	O14520	VAR_067254	p.Val59Leu	LB/B	rs4008659	-
AQP7	O14520	VAR_067255	p.Gly264Val	LB/B	rs62542743	-
AQP8	O94778	VAR_021933	p.Ala260Pro	LB/B	rs2287798	-
AQP8	O94778	VAR_036484	p.Ile229Met	US	-	A breast cancer sample
AQP9	O43315	VAR_024538	p.Thr279Ala	LB/B	rs1867380	-
AR	P10275	VAR_004679	p.Glu2Lys	LP/P	rs104894742	Androgen insensitivity, partial (PAIS) [MIM:312300]
AR	P10275	VAR_004680	p.Leu54Ser	US	-	Prostate cancer
AR	P10275	VAR_004681	p.Leu57Gln	US	rs78686797	Prostate cancer
AR	P10275	VAR_004684	p.Arg608Gln	US	rs137852573	Breast cancer
AR	P10275	VAR_004684	p.Arg608Gln	LP/P	rs137852573	Androgen insensitivity, partial (PAIS) [MIM:312300]
AR	P10275	VAR_004685	p.Arg609Lys	US	rs137852576	Breast cancer
AR	P10275	VAR_004685	p.Arg609Lys	LP/P	rs137852576	Androgen insensitivity, partial (PAIS) [MIM:312300]
AR	P10275	VAR_004686	p.Ala646Asp	LB/B	rs1800053	-
AR	P10275	VAR_004687	p.Ile665Asn	LP/P	-	Androgen insensitivity syndrome (AIS) [MIM:300068]
AR	P10275	VAR_004687	p.Ile665Asn	LP/P	-	Androgen insensitivity, partial (PAIS) [MIM:312300]
AR	P10275	VAR_004688	p.Leu678Pro	LP/P	rs137852579	Androgen insensitivity syndrome (AIS) [MIM:300068]
AR	P10275	VAR_004690	p.Asp696His	LP/P	-	Androgen insensitivity syndrome (AIS) [MIM:300068]
AR	P10275	VAR_004691	p.Asp696Asn	LP/P	rs1555995840	Androgen insensitivity syndrome (AIS) [MIM:300068]
AR	P10275	VAR_004692	p.Asp696Val	LP/P	-	Androgen insensitivity syndrome (AIS) [MIM:300068]
AR	P10275	VAR_004693	p.Ser704Gly	LP/P	-	Androgen insensitivity syndrome (AIS) [MIM:300068]
AR	P10275	VAR_004693	p.Ser704Gly	LP/P	-	Androgen insensitivity, partial (PAIS) [MIM:312300]
AR	P10275	VAR_004694	p.Leu708Arg	LP/P	rs137852585	Androgen insensitivity syndrome (AIS) [MIM:300068]
AR	P10275	VAR_004695	p.Val731Met	US	rs137852571	Prostate cancer
AR	P10275	VAR_004696	p.Asp733Asn	LP/P	-	Androgen insensitivity syndrome (AIS) [MIM:300068]
AR	P10275	VAR_004697	p.Asp733Tyr	LP/P	-	Androgen insensitivity syndrome (AIS) [MIM:300068]
AR	P10275	VAR_004698	p.Met743Ile	LP/P	-	Androgen insensitivity, partial (PAIS) [MIM:312300]
AR	P10275	VAR_004699	p.Gly744Val	LP/P	rs137852600	Androgen insensitivity syndrome (AIS) [MIM:300068]
AR	P10275	VAR_004699	p.Gly744Val	LP/P	rs137852600	Androgen insensitivity, partial (PAIS) [MIM:312300]
AR	P10275	VAR_004700	p.Met750Val	LP/P	rs1085307685	Androgen insensitivity syndrome (AIS) [MIM:300068]
AR	P10275	VAR_004700	p.Met750Val	LP/P	rs1085307685	Androgen insensitivity, partial (PAIS) [MIM:312300]
AR	P10275	VAR_004701	p.Gly751Asp	LP/P	-	Androgen insensitivity syndrome (AIS) [MIM:300068]
AR	P10275	VAR_004702	p.Arg753Gln	LP/P	rs1057523747	Androgen insensitivity syndrome (AIS) [MIM:300068]
AR	P10275	VAR_004703	p.Phe755Val	LP/P	-	Androgen insensitivity syndrome (AIS) [MIM:300068]
AR	P10275	VAR_004704	p.Leu763Phe	LP/P	-	Androgen insensitivity syndrome (AIS) [MIM:300068]
AR	P10275	VAR_004705	p.Tyr764Cys	US	rs137852567	Prostate cancer
AR	P10275	VAR_004705	p.Tyr764Cys	LP/P	rs137852567	Androgen insensitivity, partial (PAIS) [MIM:312300]
AR	P10275	VAR_004707	p.Ala766Thr	LP/P	rs1555996863	Androgen insensitivity syndrome (AIS) [MIM:300068]
AR	P10275	VAR_004708	p.Arg775His	LP/P	rs137852572	Androgen insensitivity syndrome (AIS) [MIM:300068]
AR	P10275	VAR_004708	p.Arg775His	LP/P	rs137852572	Androgen insensitivity, partial (PAIS) [MIM:312300]

AR	P10275	VAR_004709	p.Arg775Cys	LP/P	rs137852562	Androgen insensitivity syndrome (AIS) [MIM:300068]
AR	P10275	VAR_004710	p.Arg780Trp	LP/P	-	Androgen insensitivity syndrome (AIS) [MIM:300068]
AR	P10275	VAR_004711	p.Met781Ile	LP/P	rs137852589	Androgen insensitivity syndrome (AIS) [MIM:300068]
AR	P10275	VAR_004711	p.Met781Ile	LP/P	rs137852589	Androgen insensitivity, partial (PAIS) [MIM:312300]
AR	P10275	VAR_004712	p.Cys785Tyr	LP/P	-	Androgen insensitivity syndrome (AIS) [MIM:300068]
AR	P10275	VAR_004713	p.Met788Val	LP/P	rs137852570	Androgen insensitivity syndrome (AIS) [MIM:300068]
AR	P10275	VAR_004714	p.Phe795Ser	LP/P	-	Androgen insensitivity syndrome (AIS) [MIM:300068]
AR	P10275	VAR_004715	p.Gln799Glu	US	rs137852591	Prostate cancer
AR	P10275	VAR_004715	p.Gln799Glu	LP/P	rs137852591	Androgen insensitivity syndrome (AIS) [MIM:300068]
AR	P10275	VAR_004715	p.Gln799Glu	LP/P	rs137852591	Androgen insensitivity, partial (PAIS) [MIM:312300]
AR	P10275	VAR_004716	p.Met808Arg	LP/P	-	Androgen insensitivity syndrome (AIS) [MIM:300068]
AR	P10275	VAR_004717	p.Met808Val	LP/P	-	Androgen insensitivity syndrome (AIS) [MIM:300068]
AR	P10275	VAR_004718	p.Ser815Asn	LP/P	-	Androgen insensitivity syndrome (AIS) [MIM:300068]
AR	P10275	VAR_004718	p.Ser815Asn	LP/P	-	Androgen insensitivity, partial (PAIS) [MIM:312300]
AR	P10275	VAR_004719	p.Arg832Leu	LP/P	-	Androgen insensitivity syndrome (AIS) [MIM:300068]
AR	P10275	VAR_004720	p.Arg832Gln	LP/P	rs1386577803	Androgen insensitivity syndrome (AIS) [MIM:300068]
AR	P10275	VAR_004721	p.Arg841Cys	LP/P	rs137852577	Androgen insensitivity syndrome (AIS) [MIM:300068]
AR	P10275	VAR_004722	p.Arg841Gly	LP/P	-	Androgen insensitivity, partial (PAIS) [MIM:312300]
AR	P10275	VAR_004723	p.Arg841His	LP/P	rs9332969	Androgen insensitivity syndrome (AIS) [MIM:300068]
AR	P10275	VAR_004724	p.Ile843Thr	LP/P	rs9332970	Androgen insensitivity syndrome (AIS) [MIM:300068]
AR	P10275	VAR_004725	p.Arg856Cys	LP/P	rs886041132	Androgen insensitivity syndrome (AIS) [MIM:300068]
AR	P10275	VAR_004726	p.Arg856His	LP/P	rs9332971	Androgen insensitivity syndrome (AIS) [MIM:300068]
AR	P10275	VAR_004727	p.Asp865Asn	LP/P	rs1555997810	Androgen insensitivity syndrome (AIS) [MIM:300068]
AR	P10275	VAR_004728	p.Val867Glu	LP/P	-	Androgen insensitivity syndrome (AIS) [MIM:300068]
AR	P10275	VAR_004729	p.Val867Leu	LP/P	rs137852564	Androgen insensitivity, partial (PAIS) [MIM:312300]
AR	P10275	VAR_004730	p.Val867Met	US	rs137852564	Prostate cancer
AR	P10275	VAR_004730	p.Val867Met	LP/P	rs137852564	Androgen insensitivity syndrome (AIS) [MIM:300068]
AR	P10275	VAR_004731	p.Ile870Met	LP/P	rs137852574	Androgen insensitivity, partial (PAIS) [MIM:312300]
AR	P10275	VAR_004732	p.Thr878Ala	US	rs137852578	Prostate cancer
AR	P10275	VAR_004733	p.Pro893Leu	LP/P	-	Androgen insensitivity syndrome (AIS) [MIM:300068]
AR	P10275	VAR_004734	p.Met896Thr	LP/P	-	Androgen insensitivity syndrome (AIS) [MIM:300068]
AR	P10275	VAR_004735	p.Leu908Phe	LP/P	-	Androgen insensitivity syndrome (AIS) [MIM:300068]
AR	P10275	VAR_004736	p.Pro914Ser	LP/P	-	Androgen insensitivity, partial (PAIS) [MIM:312300]
AR	P10275	VAR_009224	p.Gln196Arg	LP/P	-	Androgen insensitivity syndrome (AIS) [MIM:300068]
AR	P10275	VAR_009225	p.Leu257Pro	LP/P	-	Androgen insensitivity syndrome (AIS) [MIM:300068]
AR	P10275	VAR_009226	p.Pro392Arg	LP/P	rs773996740	Androgen insensitivity syndrome (AIS) [MIM:300068]
AR	P10275	VAR_009227	p.Pro392Ser	LP/P	rs201934623	Androgen insensitivity syndrome (AIS) [MIM:300068]

AR	P10275	VAR_009228	p.Gln445Arg	US	rs1355285524	Androgen insensitivity syndrome (AIS) [MIM:300068]
AR	P10275	VAR_009229	p.Arg841Ser	LP/P	-	Androgen insensitivity, partial (PAIS) [MIM:312300]
AR	P10275	VAR_009711	p.Gln64Arg	US	-	Prostate cancer
AR	P10275	VAR_009712	p.Gln114His	US	-	Prostate cancer
AR	P10275	VAR_009713	p.Lys182Arg	US	-	Prostate cancer
AR	P10275	VAR_009714	p.Ser207Arg	LB/B	rs374549047	-
AR	P10275	VAR_009715	p.Gly216Arg	LB/B	rs199554641	-
AR	P10275	VAR_009716	p.Met268Thr	US	-	Prostate cancer
AR	P10275	VAR_009717	p.Pro271Ser	US	-	Prostate cancer
AR	P10275	VAR_009718	p.Pro342Leu	US	rs138454018	Prostate cancer
AR	P10275	VAR_009719	p.Gly492Ser	LP/P	-	Androgen insensitivity syndrome (AIS) [MIM:300068]
AR	P10275	VAR_009720	p.Asp529Gly	US	-	Prostate cancer
AR	P10275	VAR_009721	p.Leu548Phe	LP/P	rs139524801	Androgen insensitivity, partial (PAIS) [MIM:312300]
AR	P10275	VAR_009722	p.Pro549Ser	LP/P	rs137852588	Androgen insensitivity syndrome (AIS) [MIM:300068]
AR	P10275	VAR_009723	p.Cys560Tyr	LP/P	-	Androgen insensitivity syndrome (AIS) [MIM:300068]
AR	P10275	VAR_009725	p.Gly569Val	US	-	-
AR	P10275	VAR_009726	p.Gly569Trp	LP/P	rs1555982864	Androgen insensitivity, partial (PAIS) [MIM:312300]
AR	P10275	VAR_009727	p.Tyr572Cys	LP/P	-	Androgen insensitivity syndrome (AIS) [MIM:300068]
AR	P10275	VAR_009728	p.Ala574Asp	LP/P	-	Androgen insensitivity syndrome (AIS) [MIM:300068]
AR	P10275	VAR_009729	p.Leu575Pro	US	-	Prostate cancer
AR	P10275	VAR_009730	p.Thr576Ala	US	-	Prostate cancer
AR	P10275	VAR_009731	p.Cys577Phe	LP/P	-	Androgen insensitivity syndrome (AIS) [MIM:300068]
AR	P10275	VAR_009732	p.Cys577Arg	LP/P	-	Androgen insensitivity syndrome (AIS) [MIM:300068]
AR	P10275	VAR_009733	p.Cys580Phe	LP/P	rs137852586	Androgen insensitivity syndrome (AIS) [MIM:300068]
AR	P10275	VAR_009734	p.Cys580Tyr	LP/P	rs137852586	Androgen insensitivity syndrome (AIS) [MIM:300068]
AR	P10275	VAR_009735	p.Lys581Arg	US	-	Prostate cancer
AR	P10275	VAR_009736	p.Val582Phe	LP/P	-	Androgen insensitivity syndrome (AIS) [MIM:300068]
AR	P10275	VAR_009737	p.Phe583Ser	LP/P	-	Androgen insensitivity, partial (PAIS) [MIM:312300]
AR	P10275	VAR_009738	p.Phe583Tyr	LP/P	rs137852587	Androgen insensitivity, partial (PAIS) [MIM:312300]
AR	P10275	VAR_009740	p.Arg586Lys	LP/P	-	Androgen insensitivity syndrome (AIS) [MIM:300068]
AR	P10275	VAR_009741	p.Ala587Val	US	-	Prostate cancer
AR	P10275	VAR_009742	p.Ala588Ser	US	-	Prostate cancer
AR	P10275	VAR_009743	p.Ala597Thr	LP/P	rs137852569	Androgen insensitivity syndrome (AIS) [MIM:300068]
AR	P10275	VAR_009744	p.Ser598Gly	LP/P	rs142280455	Androgen insensitivity, partial (PAIS) [MIM:312300]
AR	P10275	VAR_009745	p.Ser598Thr	US	-	-
AR	P10275	VAR_009746	p.Cys602Phe	LP/P	-	Androgen insensitivity syndrome (AIS) [MIM:300068]
AR	P10275	VAR_009747	p.Asp605Tyr	LP/P	-	Androgen insensitivity, partial (PAIS) [MIM:312300]
AR	P10275	VAR_009748	p.Asn611Thr	LP/P	-	Androgen insensitivity, partial (PAIS) [MIM:312300]
AR	P10275	VAR_009749	p.Cys612Tyr	LP/P	-	Androgen insensitivity syndrome (AIS) [MIM:300068]
AR	P10275	VAR_009751	p.Arg616His	LP/P	rs754201976	Androgen insensitivity syndrome (AIS) [MIM:300068]
AR	P10275	VAR_009751	p.Arg616His	LP/P	rs754201976	Androgen insensitivity, partial (PAIS) [MIM:312300]
AR	P10275	VAR_009752	p.Arg616Pro	LP/P	-	Androgen insensitivity syndrome (AIS) [MIM:300068]

AR P10275 VAR_009753 p.Leu617Pro LP/P rs1555990488 Androgen insensitivity syndrome (AIS)
[MIM:300068]
AR P10275 VAR_009754 p.Leu617Arg LP/P - Androgen insensitivity, partial (PAIS) [MIM:312300]
AR P10275 VAR_009755 p.Arg618Pro LP/P - Androgen insensitivity syndrome (AIS) [MIM:300068]
AR P10275 VAR_009755 p.Arg618Pro LP/P - Androgen insensitivity, partial (PAIS) [MIM:312300]
AR P10275 VAR_009756 p.Cys620Tyr US - Prostate cancer
AR P10275 VAR_009757 p.Arg630Gln US rs868669253 Prostate cancer
AR P10275 VAR_009758 p.Lys631Thr US - Prostate cancer
AR P10275 VAR_009760 p.Ser648Asn US rs137852584 Prostate cancer
AR P10275 VAR_009761 p.Gln671Arg US - Prostate cancer
AR P10275 VAR_009762 p.Pro672His LP/P - Androgen insensitivity, partial (PAIS) [MIM:312300]
AR P10275 VAR_009763 p.Ile673Thr US - Prostate cancer
AR P10275 VAR_009764 p.Glu682Lys LP/P rs1555995816 Androgen insensitivity syndrome (AIS)
[MIM:300068]
AR P10275 VAR_009765 p.Gly684Ala US - Prostate cancer
AR P10275 VAR_009766 p.Val685Ile LP/P rs1555995822 Androgen insensitivity syndrome (AIS)
[MIM:300068]
AR P10275 VAR_009767 p.Cys687Arg LP/P - Androgen insensitivity, partial (PAIS) [MIM:312300]
AR P10275 VAR_009768 p.Ala688Val LP/P - Androgen insensitivity, partial (PAIS) [MIM:312300]
AR P10275 VAR_009769 p.Gly689Glu LP/P - Androgen insensitivity syndrome (AIS) [MIM:300068]
AR P10275 VAR_009771 p.Leu701Met LP/P - Androgen insensitivity syndrome (AIS) [MIM:300068]
AR P10275 VAR_009772 p.Leu702Phe LP/P rs1555995851 Androgen insensitivity syndrome (AIS)
[MIM:300068]
AR P10275 VAR_009773 p.Leu702His US rs864622007 Prostate cancer
AR P10275 VAR_009773 p.Leu702His LP/P rs864622007 Androgen insensitivity syndrome (AIS)
[MIM:300068]
AR P10275 VAR_009774 p.Ser703Ala LP/P - Androgen insensitivity syndrome (AIS) [MIM:300068]
AR P10275 VAR_009775 p.Ser704Cys LP/P - Androgen insensitivity syndrome (AIS) [MIM:300068]
AR P10275 VAR_009776 p.Asn706Ser LP/P rs925822435 Androgen insensitivity syndrome (AIS)
[MIM:300068]
AR P10275 VAR_009777 p.Gly709Ala LP/P - Androgen insensitivity, partial (PAIS) [MIM:312300]
AR P10275 VAR_009778 p.Gly709Val LP/P - Androgen insensitivity syndrome (AIS) [MIM:300068]
AR P10275 VAR_009779 p.Arg711Thr LP/P - Androgen insensitivity syndrome (AIS) [MIM:300068]
AR P10275 VAR_009780 p.Leu713Phe LP/P rs137852595 Androgen insensitivity, partial (PAIS) [MIM:312300]
AR P10275 VAR_009781 p.Val716Met US rs1340026226 Prostate cancer
AR P10275 VAR_009782 p.Lys718Glu US - Prostate cancer
AR P10275 VAR_009783 p.Lys721Glu US - Prostate cancer
AR P10275 VAR_009784 p.Ala722Thr US rs137852583 Prostate cancer
AR P10275 VAR_009785 p.Leu723Phe LP/P - Androgen insensitivity syndrome (AIS) [MIM:300068]
AR P10275 VAR_009786 p.Pro724Ser LP/P - Androgen insensitivity syndrome (AIS) [MIM:300068]
AR P10275 VAR_009787 p.Gly725Asp US - Prostate cancer
AR P10275 VAR_009787 p.Gly725Asp LP/P - Androgen insensitivity syndrome (AIS) [MIM:300068]
AR P10275 VAR_009788 p.Phe726Leu LB/B rs1555996810 -
AR P10275 VAR_009789 p.Arg727Leu US rs137852593 Prostate cancer
AR P10275 VAR_009790 p.Asn728Lys LP/P rs768869912 Androgen insensitivity syndrome (AIS)
[MIM:300068]
AR P10275 VAR_009791 p.Leu729Ser LP/P - Androgen insensitivity, partial (PAIS) [MIM:312300]
AR P10275 VAR_009792 p.Gln734His LP/P - Androgen insensitivity, partial (PAIS) [MIM:312300]
AR P10275 VAR_009793 p.Ile738Thr LP/P - Androgen insensitivity, partial (PAIS) [MIM:312300]

AR	P10275	VAR_009794	p.Trp742Arg	LP/P	-	Androgen insensitivity syndrome (AIS) [MIM:300068]
AR	P10275	VAR_009795	p.Met743Val	LP/P	-	Androgen insensitivity, partial (PAIS) [MIM:312300]
AR	P10275	VAR_009796	p.Leu745Phe	US	-	Prostate cancer
AR	P10275	VAR_009796	p.Leu745Phe	LP/P	-	Androgen insensitivity syndrome (AIS) [MIM:300068]
AR	P10275	VAR_009797	p.Met746Thr	LP/P	-	Androgen insensitivity, partial (PAIS) [MIM:312300]
AR	P10275	VAR_009798	p.Val747Met	LP/P	-	Androgen insensitivity, partial (PAIS) [MIM:312300]
AR	P10275	VAR_009799	p.Ala749Asp	LP/P	-	Androgen insensitivity, partial (PAIS) [MIM:312300]
AR	P10275	VAR_009800	p.Ala749Thr	US	-	Prostate cancer
AR	P10275	VAR_009801	p.Ala749Val	US	-	Prostate cancer
AR	P10275	VAR_009802	p.Met750Ile	US	-	Prostate cancer
AR	P10275	VAR_009803	p.Gly751Ser	US	-	Prostate cancer
AR	P10275	VAR_009804	p.Trp752Arg	LP/P	-	Androgen insensitivity syndrome (AIS) [MIM:300068]
AR	P10275	VAR_009805	p.Phe755Leu	US	-	Prostate cancer
AR	P10275	VAR_009805	p.Phe755Leu	LP/P	-	Androgen insensitivity, partial (PAIS) [MIM:312300]
AR	P10275	VAR_009806	p.Thr756Ala	US	-	Prostate cancer
AR	P10275	VAR_009807	p.Asn757Ser	LP/P	rs141425171	Androgen insensitivity, partial (PAIS) [MIM:312300]
AR	P10275	VAR_009808	p.Val758Ala	US	-	Prostate cancer
AR	P10275	VAR_009809	p.Asn759Thr	LP/P	-	Androgen insensitivity, partial (PAIS) [MIM:312300]
AR	P10275	VAR_009810	p.Ser760Phe	LP/P	-	Androgen insensitivity syndrome (AIS) [MIM:300068]
AR	P10275	VAR_009811	p.Ser760Pro	US	-	Prostate cancer
AR	P10275	VAR_009812	p.Tyr764His	LP/P	-	Androgen insensitivity syndrome (AIS) [MIM:300068]
AR	P10275	VAR_009813	p.Phe765Leu	LP/P	-	Androgen insensitivity syndrome (AIS) [MIM:300068]
AR	P10275	VAR_009814	p.Ala766Val	LP/P	-	Androgen insensitivity syndrome (AIS) [MIM:300068]
AR	P10275	VAR_009815	p.Pro767Ser	LP/P	-	Androgen insensitivity syndrome (AIS) [MIM:300068]
AR	P10275	VAR_009816	p.Asp768Glu	LP/P	-	Androgen insensitivity syndrome (AIS) [MIM:300068]
AR	P10275	VAR_009817	p.Leu769Pro	LP/P	-	Androgen insensitivity syndrome (AIS) [MIM:300068]
AR	P10275	VAR_009818	p.Asn772His	LP/P	rs886041352	Androgen insensitivity, partial (PAIS) [MIM:312300]
AR	P10275	VAR_009819	p.Glu773Ala	LP/P	-	Androgen insensitivity, partial (PAIS) [MIM:312300]
AR	P10275	VAR_009820	p.Glu773Gly	LP/P	-	Androgen insensitivity, partial (PAIS) [MIM:312300]
AR	P10275	VAR_009821	p.Ser783Asn	US	-	Prostate cancer
AR	P10275	VAR_009822	p.Arg789Ser	LP/P	rs1254203917	Androgen insensitivity syndrome (AIS) [MIM:300068]
AR	P10275	VAR_009823	p.Leu791Phe	LP/P	-	Androgen insensitivity syndrome (AIS) [MIM:300068]
AR	P10275	VAR_009824	p.Ser792Pro	US	-	Prostate cancer
AR	P10275	VAR_009825	p.Glu794Asp	LB/B	rs1414341563	-
AR	P10275	VAR_009826	p.Cys807Tyr	LP/P	rs1064793480	Androgen insensitivity, partial (PAIS) [MIM:312300]
AR	P10275	VAR_009827	p.Met808Thr	LP/P	rs137852592	Androgen insensitivity, partial (PAIS) [MIM:312300]
AR	P10275	VAR_009828	p.Leu813Phe	LP/P	rs1555997625	Androgen insensitivity syndrome (AIS) [MIM:300068]
AR	P10275	VAR_009829	p.Gly821Ala	LP/P	-	Androgen insensitivity syndrome (AIS) [MIM:300068]
AR	P10275	VAR_009830	p.Leu822Val	LP/P	-	Androgen insensitivity, partial (PAIS) [MIM:312300]
AR	P10275	VAR_009831	p.Leu831Pro	US	-	Prostate cancer
AR	P10275	VAR_009832	p.Tyr835Cys	LP/P	rs1057521122	Androgen insensitivity syndrome (AIS) [MIM:300068]
AR	P10275	VAR_009833	p.Ile842Ser	LP/P	-	Androgen insensitivity, partial (PAIS) [MIM:312300]
AR	P10275	VAR_009834	p.Arg847Gly	US	-	Prostate cancer
AR	P10275	VAR_009835	p.Arg855Lys	LP/P	-	Androgen insensitivity, partial (PAIS) [MIM:312300]

AR P10275 VAR_009836 p.Phe857Leu LP/P rs137852598 Androgen insensitivity syndrome (AIS) [MIM:300068]
AR P10275 VAR_009837 p.Leu864Arg LP/P - Androgen insensitivity syndrome (AIS) [MIM:300068]
AR P10275 VAR_009838 p.Asp865Gly LP/P - Androgen insensitivity syndrome (AIS) [MIM:300068]
AR P10275 VAR_009839 p.Ser866Pro LP/P rs137852597 Androgen insensitivity syndrome (AIS) [MIM:300068]
AR P10275 VAR_009840 p.Ala871Gly LP/P - Androgen insensitivity, partial (PAIS) [MIM:312300]
AR P10275 VAR_009841 p.Ala871Val LP/P rs143040492 Androgen insensitivity, partial (PAIS) [MIM:312300]
AR P10275 VAR_009842 p.Arg872Gly LP/P - Androgen insensitivity syndrome (AIS) [MIM:300068]
AR P10275 VAR_009843 p.His875Tyr US rs137852581 Prostate cancer
AR P10275 VAR_009844 p.Thr878Ser US rs137852580 Prostate cancer
AR P10275 VAR_009845 p.Leu881Gln US - Prostate cancer
AR P10275 VAR_009846 p.Leu882Val LP/P - Androgen insensitivity syndrome (AIS) [MIM:300068]
AR P10275 VAR_009847 p.Met887Val LP/P rs755226547 Androgen insensitivity syndrome (AIS) [MIM:300068]
AR P10275 VAR_009848 p.Val890Met LP/P rs886041133 Androgen insensitivity syndrome (AIS) [MIM:300068]
AR P10275 VAR_009848 p.Val890Met LP/P rs886041133 Androgen insensitivity, partial (PAIS) [MIM:312300]
AR P10275 VAR_009849 p.Asp891Asn US - Prostate cancer
AR P10275 VAR_009850 p.Phe892Leu US - Prostate cancer
AR P10275 VAR_009851 p.Ala897Thr US - Prostate cancer
AR P10275 VAR_009852 p.Ile899Thr LP/P rs1555998105 Androgen insensitivity syndrome (AIS) [MIM:300068]
AR P10275 VAR_009853 p.Gln903Arg US rs137852582 Prostate cancer
AR P10275 VAR_009854 p.Val904Met LP/P - Androgen insensitivity, partial (PAIS) [MIM:312300]
AR P10275 VAR_009855 p.Pro905His LP/P - Androgen insensitivity syndrome (AIS) [MIM:300068]
AR P10275 VAR_009856 p.Pro905Ser LP/P - Androgen insensitivity syndrome (AIS) [MIM:300068]
AR P10275 VAR_009857 p.Gly910Glu US - Prostate cancer
AR P10275 VAR_009858 p.Gly910Arg LP/P - Androgen insensitivity, partial (PAIS) [MIM:312300]
AR P10275 VAR_009859 p.Lys911Arg US - Prostate cancer
AR P10275 VAR_009860 p.Val912Leu LP/P - Androgen insensitivity, partial (PAIS) [MIM:312300]
AR P10275 VAR_009861 p.Phe917Leu LP/P - Androgen insensitivity syndrome (AIS) [MIM:300068]
AR P10275 VAR_009862 p.His918Arg LP/P - Androgen insensitivity syndrome (AIS) [MIM:300068]
AR P10275 VAR_009863 p.Gln920Arg US - Prostate cancer
AR P10275 VAR_013474 p.Pro683Thr LP/P - Androgen insensitivity, partial (PAIS) [MIM:312300]
AR P10275 VAR_013475 p.Asn706Tyr LP/P - Androgen insensitivity syndrome (AIS) [MIM:300068]
AR P10275 VAR_013476 p.Gln712Glu LP/P - Androgen insensitivity, partial (PAIS) [MIM:312300]
AR P10275 VAR_013477 p.Gly744Glu LP/P rs137852600 Androgen insensitivity syndrome (AIS) [MIM:300068]
AR P10275 VAR_013478 p.Phe828Val LP/P - Androgen insensitivity, partial (PAIS) [MIM:312300]
AR P10275 VAR_013479 p.His875Arg LP/P - Androgen insensitivity syndrome (AIS) [MIM:300068]
AR P10275 VAR_013480 p.Asp880Tyr LP/P - Androgen insensitivity syndrome (AIS) [MIM:300068]
ARAF P10398 VAR_040375 p.Met98Thr LB/B rs56197559 -
ARAF P10398 VAR_040376 p.Gly331Cys US - A colorectal adenocarcinoma sample
ARAF P10398 VAR_040377 p.Glu578Asp LB/B rs55852926 -
ARAP1 Q96P48 VAR_055529 p.Arg358Gln LB/B rs34976830 -
ARAP1 Q96P48 VAR_061023 p.Gln1047Glu LB/B rs56200889 -
ARAP2 Q8WZ64 VAR_027952 p.Arg1523Gln LB/B rs4833069 -
ARAP2 Q8WZ64 VAR_055530 p.Lys384Asn LB/B rs35468501 -

ARAP2 Q8WZ64 VAR_055531 p.Phe1006Leu LB/B rs35218548 -
ARAP3 Q8WWN8 VAR_036180 p.Arg471Trp US rs1562420371 A colorectal cancer sample
ARAP3 Q8WWN8 VAR_036181 p.Ile1085Met US - A breast cancer sample
ARAP3 Q8WWN8 VAR_036182 p.Thr1428Pro US rs61749636 A breast cancer sample
ARAP3 Q8WWN8 VAR_048330 p.Asp218His LB/B rs1031904 -
ARCN1 P48444 VAR_011788 p.Phe186Leu LB/B rs682327 -
ARCN1 P48444 VAR_011789 p.Lys309Asn LB/B rs1063124 -
AREG P15514 VAR_018918 p.Asp80Val LB/B rs373527160 -
AREG P15514 VAR_018919 p.Tyr81Cys LB/B rs28364979 -
AREL1 O15033 VAR_083342 p.Pro779Leu LB/B rs371610162 -
ARF1 P84077 VAR_081272 p.Tyr35His LP/P rs879036238 Periventricular nodular heterotopia 8 (PVNH8) [MIM:618185]
ARF1 P84077 VAR_081273 p.Arg99His US - Periventricular nodular heterotopia 8 (PVNH8) [MIM:618185]
ARF1 P84077 VAR_081274 p.Lys127Glu LP/P - Periventricular nodular heterotopia 8 (PVNH8) [MIM:618185]
ARF4 P18085 VAR_048317 p.Val68Ala LB/B rs11550597 -
ARFGAP1 Q8N6T3 VAR_015187 p.Val184Met LB/B rs2273499 -
ARFGAP2 Q8N6H7 VAR_030780 p.Ser411Asn LB/B rs3740691 -
ARFGAP2 Q8N6H7 VAR_048321 p.Pro143Arg LB/B rs11542793 -
ARFGAP2 Q8N6H7 VAR_048322 p.Arg339His LB/B rs34662994 -
ARFGAP2 Q8N6H7 VAR_048323 p.Arg406Trp LB/B rs35950498 -
ARFGAP3 Q9NP61 VAR_013447 p.Ser355Arg LB/B rs1018448 -
ARFGAP3 Q9NP61 VAR_036177 p.Glu290Gly US - A breast cancer sample
ARFGAP3 Q9NP61 VAR_055523 p.Ser231Gly LB/B rs9607957 -
ARFGAP3 Q9NP61 VAR_055524 p.Ser370Gly LB/B rs16986123 -
ARFGAP3 Q9NP61 VAR_055525 p.Gln468His LB/B rs35498349 -
ARFGAP3 Q9NP61 VAR_055526 p.Ala482Thr LB/B rs36003980 -
ARFGAP3 Q9NP61 VAR_055527 p.Gln490Arg LB/B rs11551619 -
ARFGEF1 Q9Y6D6 VAR_028749 p.Asp273Tyr LB/B rs4321984 -
ARFGEF1 Q9Y6D6 VAR_036155 p.Gly316Glu US - A colorectal cancer sample
ARFGEF2 Q9Y6D5 VAR_028750 p.Ala527Val LB/B rs6063343 -
ARFGEF2 Q9Y6D5 VAR_036156 p.Lys794Glu US - A breast cancer sample
ARFGEF2 Q9Y6D5 VAR_037438 p.Glu209Lys US rs28937880 Periventricular nodular heterotopia 2 (PVNH2) [MIM:608097]
ARFGEF2 Q9Y6D5 VAR_069404 p.Arg802Gln LB/B rs748482139 -
ARFGEF3 Q5TH69 VAR_032154 p.Ser689Ala LB/B rs7764091 -
ARFGEF3 Q5TH69 VAR_032155 p.Ala1571Thr LB/B rs3736706 -
ARFGEF3 Q5TH69 VAR_032156 p.Lys2031Arg LB/B rs35964895 -
ARFGEF3 Q5TH69 VAR_051925 p.Glu413Asp LB/B rs9376338 -
ARFRP1 Q13795 VAR_036559 p.Leu108Val US - A breast cancer sample
ARG1 P05089 VAR_000674 p.Gly235Arg LP/P rs104893948 Argininemia (ARGIN) [MIM:207800]
ARG1 P05089 VAR_000675 p.Thr290Ser LB/B rs104893942 -
ARG1 P05089 VAR_015594 p.Ile11Thr LP/P rs28941474 Argininemia (ARGIN) [MIM:207800]
ARG1 P05089 VAR_015595 p.Gly138Val LP/P rs104893943 Argininemia (ARGIN) [MIM:207800]
ARG1 P05089 VAR_072164 p.Gly27Asp LP/P rs1326930389 Argininemia (ARGIN) [MIM:207800]
ARG1 P05089 VAR_072165 p.Gly74Val LP/P - Argininemia (ARGIN) [MIM:207800]
ARG1 P05089 VAR_072166 p.Ala125Val LP/P - Argininemia (ARGIN) [MIM:207800]
ARG1 P05089 VAR_072167 p.Thr134Ile LP/P - Argininemia (ARGIN) [MIM:207800]

ARG1 P05089 VAR_072168 p.Arg180Thr LP/P - Argininemia (ARGIN) [MIM:207800]
ARG1 P05089 VAR_072169 p.Arg308Gln LP/P rs377280518 Argininemia (ARGIN) [MIM:207800]
ARG2 P78540 VAR_033520 p.Gly240Arg LB/B rs17104534 -
ARGFX A6NJG6 VAR_037226 p.Arg145Gln LB/B rs9813391 -
ARHGAP1 Q07960 VAR_049137 p.Arg369Cys LB/B rs11822837 -
ARHGAP10 A1A4S6 VAR_035114 p.Met684Val LB/B rs2276932 -
ARHGAP10 A1A4S6 VAR_049141 p.Pro488Ser LB/B rs17024215 -
ARHGAP11A Q6P4F7 VAR_035546 p.Glu605Lys US - A breast cancer sample
ARHGAP12 Q8IWW6 VAR_024454 p.Phe442Ser LB/B rs2808096 -
ARHGAP18 Q8N392 VAR_060460 p.Thr23Ala LB/B rs3752536 -
ARHGAP18 Q8N392 VAR_060461 p.Asn91Ser LB/B rs11544371 -
ARHGAP18 Q8N392 VAR_060462 p.Gln165His LB/B rs11544372 -
ARHGAP19 Q14CB8 VAR_031152 p.Gln305Arg LB/B rs17112598 -
ARHGAP20 Q9P2F6 VAR_031489 p.Ala522Thr LB/B rs7936020 -
ARHGAP20 Q9P2F6 VAR_031490 p.Gly632Asp LB/B rs17853925 -
ARHGAP21 Q5T5U3 VAR_035187 p.Asn713Ser LB/B rs3748222 -
ARHGAP21 Q5T5U3 VAR_035188 p.Thr1594Ala LB/B rs1133897 -
ARHGAP21 Q5T5U3 VAR_035189 p.Val1611Ala LB/B rs1143051 -
ARHGAP21 Q5T5U3 VAR_035190 p.Glu1629Lys LB/B rs1143057 -
ARHGAP21 Q5T5U3 VAR_035191 p.Ala1727Thr LB/B rs1143075 -
ARHGAP21 Q5T5U3 VAR_035192 p.Ser1950Asn LB/B rs1127893 -
ARHGAP22 Q7Z5H3 VAR_031153 p.Thr410Lys LB/B rs1867586 -
ARHGAP22 Q7Z5H3 VAR_031154 p.Arg612Cys LB/B rs3747853 -
ARHGAP25 P42331 VAR_049142 p.Arg192Trp LB/B rs3749130 -
ARHGAP25 P42331 VAR_049143 p.Arg555Ser LB/B rs4241344 -
ARHGAP25 P42331 VAR_049144 p.Met556Thr LB/B rs10177248 -
ARHGAP26 Q9UNA1 VAR_013623 p.Asn417Ser LP/P rs121918546 Leukemia, juvenile myelomonocytic (JMML) [MIM:607785]
ARHGAP27 Q6ZUM4 VAR_038551 p.His889Gln LB/B rs117139057 -
ARHGAP28 Q9P2N2 VAR_031155 p.Thr190Ser LB/B rs6506448 -
ARHGAP28 Q9P2N2 VAR_031156 p.Gln727Pro LB/B rs1056408 -
ARHGAP28 Q9P2N2 VAR_055833 p.Ile168Val LB/B rs2303978 -
ARHGAP29 Q52LW3 VAR_038552 p.Ser552Cys US - A breast cancer sample
ARHGAP29 Q52LW3 VAR_038553 p.Gly1255Asp LB/B rs1999272 -
ARHGAP29 Q52LW3 VAR_049145 p.Pro1192Leu LB/B rs11165091 -
ARHGAP30 Q7Z6I6 VAR_031157 p.Phe70Leu LB/B rs17854839 -
ARHGAP30 Q7Z6I6 VAR_031158 p.Leu591Val LB/B rs3813609 -
ARHGAP31 Q2M1Z3 VAR_039122 p.Pro221Leu LB/B rs751793 -
ARHGAP31 Q2M1Z3 VAR_039123 p.Gly803Ser LB/B rs3732413 -
ARHGAP31 Q2M1Z3 VAR_039124 p.Ile1115Leu LB/B rs12107254 -
ARHGAP31 Q2M1Z3 VAR_039125 p.Val1366Met LB/B rs3796360 -
ARHGAP31 Q2M1Z3 VAR_039126 p.Thr1380Ile LB/B rs9852894 -
ARHGAP31 Q2M1Z3 VAR_065919 p.Thr727Ile LB/B rs539048828 -
ARHGAP4 P98171 VAR_028413 p.Ala104Val LB/B rs5987182 -
ARHGAP40 Q5TG30 VAR_042892 p.Gly133Arg LB/B rs6100455 -
ARHGAP40 Q5TG30 VAR_042893 p.Arg413Leu LB/B rs6070872 -
ARHGAP40 Q5TG30 VAR_042894 p.His463Asn LB/B rs16987460 -
ARHGAP44 Q17R89 VAR_031159 p.Val463Met LB/B rs3213688 -
ARHGAP45 Q92619 VAR_042693 p.Arg139His LB/B rs1801284 -

ARHGAP45 Q92619 VAR_042694 p.Glu259Asp LB/B rs2074442 -
ARHGAP45 Q92619 VAR_042695 p.Ser439Gly LB/B rs7251797 -
ARHGAP45 Q92619 VAR_042696 p.Met515Ile LB/B rs36084354 -
ARHGAP45 Q92619 VAR_042697 p.Ala886Pro LB/B rs34569196 -
ARHGAP5 Q13017 VAR_043980 p.Ile17Val LB/B rs17386818 -
ARHGAP6 O43182 VAR_024453 p.Asp791Glu LB/B rs1009758 -
ARHGAP8 P85298 VAR_049138 p.Tyr172Cys LB/B rs16992915 -
ARHGAP8 P85298 VAR_049139 p.Gly333Arg LB/B rs6007344 -
ARHGAP8 P85298 VAR_049140 p.Pro415Leu LB/B rs2071762 -
ARHGAP8 P85298 VAR_061182 p.Pro418Arg LB/B rs9614957 -
ARHGAP9 Q9BRR9 VAR_055830 p.Arg50Gly LB/B rs33927108 -
ARHGAP9 Q9BRR9 VAR_055831 p.Arg137Cys LB/B rs3802989 -
ARHGAP9 Q9BRR9 VAR_055832 p.Ser370Ala LB/B rs11544238 -
ARHGEF1 Q92888 VAR_033521 p.Pro375Leu LB/B rs2303797 -
ARHGEF1 Q92888 VAR_035969 p.Met165Val US - A colorectal cancer sample
ARHGEF10 O15013 VAR_019118 p.Thr357Ile LP/P rs28940281 Slowed nerve conduction velocity (SNCV) [MIM:608236]
ARHGEF10 O15013 VAR_038603 p.Val700Ile LB/B rs2294039 -
ARHGEF10 O15013 VAR_057188 p.Val725Ile LB/B rs2294039 -
ARHGEF10 O15013 VAR_073288 p.Asn227His US rs767902219 -
ARHGEF10L Q9HCE6 VAR_044183 p.Asp69Asn LB/B rs35497285 -
ARHGEF10L Q9HCE6 VAR_044184 p.Ala96Val US rs1421731181 A colorectal cancer sample
ARHGEF10L Q9HCE6 VAR_044185 p.Arg991Trp LB/B rs6695710 -
ARHGEF10L Q9HCE6 VAR_044186 p.Ile1219Val LB/B rs2270976 -
ARHGEF10L Q9HCE6 VAR_061794 p.Ser47Asn LB/B rs34417109 -
ARHGEF11 O15085 VAR_024285 p.His1427Arg LB/B rs945508 -
ARHGEF11 O15085 VAR_061795 p.Ser1416Gly LB/B rs868188 -
ARHGEF12 Q9NZN5 VAR_020191 p.Tyr973Phe LB/B rs2305013 -
ARHGEF15 O94989 VAR_054215 p.Gly155Val LB/B rs17857129 -
ARHGEF15 O94989 VAR_054216 p.Leu277Pro LB/B rs871841 -
ARHGEF15 O94989 VAR_054217 p.Ser831Pro LB/B rs3744647 -
ARHGEF15 O94989 VAR_057189 p.Pro73Ser LB/B rs9890841 -
ARHGEF15 O94989 VAR_077835 p.Arg604Cys US rs587777166 -
ARHGEF16 Q5VV41 VAR_059796 p.Val137Met LB/B rs3806164 -
ARHGEF16 Q5VV41 VAR_059797 p.His370Tyr LB/B rs2185639 -
ARHGEF16 Q5VV41 VAR_061796 p.Glu681Lys LB/B rs56309807 -
ARHGEF16 Q5VV41 VAR_083184 p.Arg24Trp LB/B rs562018000 -
ARHGEF17 Q96PE2 VAR_032132 p.Gly450Glu LB/B rs3741150 -
ARHGEF17 Q96PE2 VAR_032133 p.Ala1465Asp LB/B rs2298808 -
ARHGEF18 Q6ZSZ5 VAR_044066 p.Gln889Arg LB/B rs2287918 -
ARHGEF18 Q6ZSZ5 VAR_044067 p.Arg940Gln LB/B rs2287920 -
ARHGEF18 Q6ZSZ5 VAR_063099 p.Asn1207Ser LB/B rs9329368 -
ARHGEF18 Q6ZSZ5 VAR_078919 p.Thr458Ala LP/P rs987233144 Retinitis pigmentosa 78 (RP78) [MIM:617433]
ARHGEF19 Q8IW93 VAR_031950 p.Gly163Arg LB/B rs221058 -
ARHGEF19 Q8IW93 VAR_031951 p.Glu238Gln LB/B rs221057 -
ARHGEF25 Q86VW2 VAR_039402 p.Cys253Tyr LB/B rs17857333 -
ARHGEF25 Q86VW2 VAR_039403 p.Gly397Arg LB/B rs17854492 -
ARHGEF25 Q86VW2 VAR_039404 p.Gln506Arg LB/B rs1564374 -
ARHGEF26 Q96DR7 VAR_039425 p.Val29Leu LB/B rs12493885 -

ARHGEF26 Q96DR7 VAR_039426 p.Phe203Ser LB/B rs13096373 -
ARHGEF26 Q96DR7 VAR_058205 p.Leu60Pro LB/B rs12497267 -
ARHGEF28 Q8N1W1 VAR_039657 p.Arg98Met LB/B rs12659447 -
ARHGEF28 Q8N1W1 VAR_039658 p.Trp225Arg LB/B rs7714670 -
ARHGEF28 Q8N1W1 VAR_039659 p.Pro284Gln LB/B rs6453022 -
ARHGEF28 Q8N1W1 VAR_039660 p.Ser544Leu LB/B rs2973571 -
ARHGEF28 Q8N1W1 VAR_039661 p.Arg585Lys LB/B rs2973566 -
ARHGEF28 Q8N1W1 VAR_039662 p.His780Asn LB/B rs2973558 -
ARHGEF28 Q8N1W1 VAR_039663 p.Pro1548Ser LB/B rs17634865 -
ARHGEF28 Q8N1W1 VAR_039664 p.His1640Gln LB/B rs1478453 -
ARHGEF3 Q9NR81 VAR_021935 p.Lys13Arg LB/B rs3732507 -
ARHGEF3 Q9NR81 VAR_021936 p.Leu335Val LB/B rs3772219 -
ARHGEF37 A1IGU5 VAR_043579 p.Met421Leu LB/B rs4629585 -
ARHGEF37 A1IGU5 VAR_043580 p.Pro489Leu LB/B rs9324624 -
ARHGEF37 A1IGU5 VAR_043581 p.Ser518Arg LB/B rs7732714 -
ARHGEF37 A1IGU5 VAR_043582 p.Pro586Thr LB/B rs3733662 -
ARHGEF37 A1IGU5 VAR_043583 p.Met604Val LB/B rs1135093 -
ARHGEF38 Q9NXL2 VAR_038928 p.Lys67Asn US - A breast cancer sample
ARHGEF38 Q9NXL2 VAR_038929 p.Met88Val LB/B rs2276970 -
ARHGEF39 Q8N4T4 VAR_032876 p.His306Arg LB/B rs2297879 -
ARHGEF39 Q8N4T4 VAR_061801 p.Cys5Phe LB/B rs45567235 -
ARHGEF4 Q9NR80 VAR_035970 p.Lys100Arg US - A breast cancer sample
ARHGEF4 Q9NR80 VAR_035971 p.Thr441Arg US - A breast cancer sample
ARHGEF4 Q9NR80 VAR_057187 p.Asp33His LB/B rs10188052 -
ARHGEF40 Q8TER5 VAR_038061 p.Val956Leu LB/B rs7143633 -
ARHGEF40 Q8TER5 VAR_038062 p.Leu1189Ser LB/B rs1958396 -
ARHGEF40 Q8TER5 VAR_038063 p.Met1312Thr LB/B rs943992 -
ARHGEF40 Q8TER5 VAR_060541 p.Thr1418Pro LB/B rs17855344 -
ARHGEF6 Q15052 VAR_051981 p.Gln297His LB/B rs5974620 -
ARHGEF7 Q14155 VAR_064694 p.Thr790Ala US - -
ARHGEF9 O43307 VAR_028752 p.Gly55Ala LP/P rs121918361 Developmental and epileptic encephalopathy 8 (DEE8) [MIM:300607]
ARHGEF9 O43307 VAR_069370 p.Glu401Lys LP/P - -
ARHGEF9 O43307 VAR_072742 p.Arg290His LP/P - Developmental and epileptic encephalopathy 8 (DEE8) [MIM:300607]
ARID1A O14497 VAR_064695 p.Arg1020Lys US - -
ARID1A O14497 VAR_064696 p.Leu2089Pro US - -
ARID1A O14497 VAR_068021 p.Arg1658Trp LB/B rs1442666063 -
ARID1A O14497 VAR_068022 p.Ile1907Phe LB/B rs139230162 -
ARID1A O14497 VAR_068023 p.Gly2087Arg LB/B rs1553153748 -
ARID1A O14497 VAR_076938 p.Pro120Ser LB/B rs571264557 -
ARID1B Q8NFD5 VAR_036257 p.Gly814Ala US - A breast cancer sample
ARID1B Q8NFD5 VAR_067664 p.Gln82His LB/B - -
ARID1B Q8NFD5 VAR_067665 p.Gly246Ser LB/B rs375160616 -
ARID1B Q8NFD5 VAR_067670 p.Ala363Val LB/B rs748273011 -
ARID1B Q8NFD5 VAR_067671 p.Gly396Ala LB/B rs760718156 -
ARID1B Q8NFD5 VAR_067672 p.Met429Val LB/B rs199948752 -
ARID1B Q8NFD5 VAR_067674 p.Ser497Asn LB/B rs764716697 -
ARID1B Q8NFD5 VAR_067675 p.Met531Thr LB/B rs141260832 -

ARID1B	Q8NFD5	VAR_067676	p.Gln876Glu	LB/B	rs138254872	-
ARID1B	Q8NFD5	VAR_067677	p.Gln980Leu	LB/B	rs139620600	-
ARID1B	Q8NFD5	VAR_067678	p.Val1092Ile	LB/B	rs775526039	-
ARID1B	Q8NFD5	VAR_067679	p.Gln1249Pro	LB/B	rs768013849	-
ARID1B	Q8NFD5	VAR_067680	p.Gly1271Glu	LB/B	rs149389876	-
ARID1B	Q8NFD5	VAR_067681	p.Gly1303Arg	LB/B	rs199674889	-
ARID1B	Q8NFD5	VAR_067682	p.Ser1321Asn	LB/B	rs142808724	-
ARID1B	Q8NFD5	VAR_067683	p.Pro1411Ser	LB/B	-	-
ARID1B	Q8NFD5	VAR_067684	p.Gln1466Lys	LB/B	-	-
ARID1B	Q8NFD5	VAR_067685	p.Arg1506His	LB/B	rs1449208173	-
ARID1B	Q8NFD5	VAR_067686	p.Thr1573Met	LB/B	rs777745107	-
ARID1B	Q8NFD5	VAR_067687	p.Asn1659Ser	LB/B	rs140177120	-
ARID1B	Q8NFD5	VAR_067689	p.Lys1773Arg	LB/B	rs574141489	-
ARID1B	Q8NFD5	VAR_067690	p.Asp1851Asn	LB/B	rs200305796	-
ARID1B	Q8NFD5	VAR_067691	p.Lys1898Arg	LB/B	rs758204258	-
ARID1B	Q8NFD5	VAR_067692	p.Lys1954Arg	LB/B	rs756220726	-
ARID1B	Q8NFD5	VAR_067693	p.Gln2163Arg	LB/B	-	-
ARID1B	Q8NFD5	VAR_077456	p.Ser771Leu	US	-	-
ARID1B	Q8NFD5	VAR_077457	p.Gly1436Ala	LP/P	-	-
ARID1B	Q8NFD5	VAR_077458	p.Pro1563Leu	US	rs1455506883	-
ARID1B	Q8NFD5	VAR_077459	p.Asp1987Tyr	LP/P	-	-
ARID1B	Q8NFD5	VAR_078697	p.Pro488Arg	US	rs769085274	-
ARID1B	Q8NFD5	VAR_078698	p.Pro1413Ser	US	rs1051017338	-
ARID3A	Q99856	VAR_033203	p.Pro36His	LB/B	rs17857499	-
ARID3A	Q99856	VAR_033204	p.Lys320Glu	LB/B	rs17857501	-
ARID3A	Q99856	VAR_033205	p.Gly556Ser	LB/B	rs1051505	-
ARID3C	A6NKF2	VAR_043033	p.Arg310Gln	LB/B	rs12337871	-
ARID3C	A6NKF2	VAR_043034	p.Cys335Gly	LB/B	rs3808869	-
ARID4A	P29374	VAR_031566	p.His412Pro	LB/B	rs34982206	-
ARID4A	P29374	VAR_031567	p.Asn724Ser	LB/B	rs2230098	-
ARID4A	P29374	VAR_031568	p.Thr779Ala	LB/B	rs1051858	-
ARIH1	Q9Y4X5	VAR_082646	p.Glu15Gln	LP/P	rs1052050835	-
ARIH1	Q9Y4X5	VAR_082647	p.Glu44Gly	LP/P	-	-
ARIH2	O95376	VAR_054105	p.Glu24Lys	LB/B	rs11507	-
ARIH2	O95376	VAR_054106	p.Glu29Asp	LB/B	rs34221642	-
ARL11	Q969Q4	VAR_023742	p.Ser22Leu	LB/B	rs147389782	-
ARL11	Q969Q4	VAR_023743	p.Pro131Leu	LB/B	rs147120792	-
ARL11	Q969Q4	VAR_023744	p.Cys148Arg	LB/B	rs3803185	-
ARL11	Q969Q4	VAR_023745	p.Glu164Lys	LB/B	rs146850453	-
ARL11	Q969Q4	VAR_048318	p.Leu120Met	LB/B	rs35712316	-
ARL13B	Q3SXY8	VAR_048319	p.Thr348Ser	LB/B	rs33944211	-
ARL13B	Q3SXY8	VAR_054371	p.Arg79Gln	LP/P	rs121912606	Joubert syndrome 8 (JBTS8) [MIM:612291]
ARL13B	Q3SXY8	VAR_054372	p.Arg200Cys	LP/P	rs121912608	Joubert syndrome 8 (JBTS8) [MIM:612291]
ARL13B	Q3SXY8	VAR_069190	p.Arg390Leu	US	-	-
ARL13B	Q3SXY8	VAR_077496	p.Tyr86Cys	LP/P	rs863225430	Joubert syndrome 8 (JBTS8) [MIM:612291]
ARL14	Q8N4G2	VAR_055521	p.Asp83Asn	LB/B	rs35634980	-
ARL14	Q8N4G2	VAR_055522	p.Pro117Thr	LB/B	rs35633732	-
ARL14EP	Q8N8R7	VAR_033740	p.Thr180Pro	LB/B	rs7940297	-
ARL16	Q0P5N6	VAR_059129	p.Ser10Arg	LB/B	rs8066889	-

ARL17A Q8IVW1 VAR_017170 p.Leu170Ile LB/B - -
ARL2 P36404 VAR_028056 p.Val141Ala LB/B rs664226 -
ARL2BP Q9Y2Y0 VAR_053904 p.Glu87Lys LB/B rs7198865 -
ARL2BP Q9Y2Y0 VAR_070227 p.Met45Arg LP/P rs398123053 Retinitis pigmentosa 82 with or without situs inversus (RP82) [MIM:615434]
ARL3 P36405 VAR_014869 p.Leu34Met LB/B rs1141895 -
ARL3 P36405 VAR_081202 p.Arg149Cys LP/P rs776901858 Joubert syndrome 35 (JBTS35) [MIM:618161]
ARL3 P36405 VAR_081203 p.Arg149His LP/P rs770782663 Joubert syndrome 35 (JBTS35) [MIM:618161]
ARL3 P36405 VAR_081340 p.Tyr90Cys US rs1564730440 Retinitis pigmentosa 83 (RP83) [MIM:618173]
ARL4D P49703 VAR_028205 p.Thr91Asn LB/B rs1059968 -
ARL6 Q9H0F7 VAR_027643 p.Thr31Met LP/P rs104893680 Bardet-Biedl syndrome 3 (BBS3) [MIM:600151]
ARL6 Q9H0F7 VAR_027644 p.Thr31Arg LP/P rs104893680 Bardet-Biedl syndrome 3 (BBS3) [MIM:600151]
ARL6 Q9H0F7 VAR_027645 p.Gly169Ala LP/P rs104893679 Bardet-Biedl syndrome 3 (BBS3) [MIM:600151]
ARL6 Q9H0F7 VAR_027646 p.Leu170Trp LP/P rs104893681 Bardet-Biedl syndrome 3 (BBS3) [MIM:600151]
ARL6 Q9H0F7 VAR_064184 p.Ala89Val LP/P rs587777805 Retinitis pigmentosa 55 (RP55) [MIM:613575]
ARL6 Q9H0F7 VAR_071405 p.Ile94Thr LP/P rs771054395 Bardet-Biedl syndrome 3 (BBS3) [MIM:600151]
ARL6IP4 Q66PJ3 VAR_058333 p.Lys286Arg US - -
ARL6IP4 Q66PJ3 VAR_059583 p.Pro149Ala LB/B rs12825243 -
ARMC10 Q8N2F6 VAR_034681 p.Pro190Ser LB/B rs17849774 -
ARMC2 Q8NEN0 VAR_031948 p.Met166Thr LB/B rs9386758 -
ARMC2 Q8NEN0 VAR_031949 p.Asn433Asp LB/B rs17852775 -
ARMC2 Q8NEN0 VAR_082206 p.Ile760Asn US rs1562435988 Spermatogenic failure 38 (SPGF38) [MIM:618433]
ARMC3 Q5W041 VAR_050669 p.Glu345Gly LB/B rs16922864 -
ARMC3 Q5W041 VAR_050670 p.Ser608Pro LB/B rs11013233 -
ARMC3 Q5W041 VAR_050671 p.Arg626Gln LB/B rs10828395 -
ARMC5 Q96C12 VAR_050674 p.Ile170Val LB/B rs35923277 -
ARMC5 Q96C12 VAR_072352 p.Phe14Tyr LB/B rs151069962 -
ARMC5 Q96C12 VAR_072353 p.Ser115Pro LB/B rs199693319 -
ARMC5 Q96C12 VAR_072354 p.Leu156Phe LP/P rs114930262 ACTH-independent macronodular adrenal hyperplasia 2 (AIMAH2) [MIM:615954]
ARMC5 Q96C12 VAR_072355 p.Arg315Gln LP/P rs1415974570 ACTH-independent macronodular adrenal hyperplasia 2 (AIMAH2) [MIM:615954]
ARMC5 Q96C12 VAR_072356 p.Arg315Trp LP/P - ACTH-independent macronodular adrenal hyperplasia 2 (AIMAH2) [MIM:615954]
ARMC5 Q96C12 VAR_072357 p.Leu548Pro US rs587777661 ACTH-independent macronodular adrenal hyperplasia 2 (AIMAH2) [MIM:615954]
ARMC5 Q96C12 VAR_072358 p.Arg593Trp LP/P rs587777662 ACTH-independent macronodular adrenal hyperplasia 2 (AIMAH2) [MIM:615954]
ARMC5 Q96C12 VAR_072359 p.Gly798Ala LB/B rs115611533 -
ARMC5 Q96C12 VAR_072360 p.Arg898Trp LP/P rs587777659 ACTH-independent macronodular adrenal hyperplasia 2 (AIMAH2) [MIM:615954]
ARMC5 Q96C12 VAR_079097 p.Gly56Ala LB/B rs780112907 -
ARMC5 Q96C12 VAR_079100 p.Cys139Arg LP/P - ACTH-independent macronodular adrenal hyperplasia 2 (AIMAH2) [MIM:615954]
ARMC5 Q96C12 VAR_079102 p.Leu318Val US rs1293014259 ACTH-independent macronodular adrenal hyperplasia 2 (AIMAH2) [MIM:615954]
ARMC5 Q96C12 VAR_079103 p.Gly323Ala US rs35461188 ACTH-independent macronodular adrenal hyperplasia 2 (AIMAH2) [MIM:615954]

ARMC5 Q96C12 VAR_079104 p.Leu331Pro LP/P - ACTH-independent macronodular adrenal hyperplasia 2 (AIMAH2) [MIM:615954]
ARMC5 Q96C12 VAR_079105 p.Arg362Leu LP/P - ACTH-independent macronodular adrenal hyperplasia 2 (AIMAH2) [MIM:615954]
ARMC5 Q96C12 VAR_079106 p.Arg362Trp LP/P rs1385397608 ACTH-independent macronodular adrenal hyperplasia 2 (AIMAH2) [MIM:615954]
ARMC5 Q96C12 VAR_079108 p.Leu365Pro LP/P rs587777663 ACTH-independent macronodular adrenal hyperplasia 2 (AIMAH2) [MIM:615954]
ARMC5 Q96C12 VAR_079110 p.Leu394Pro US - ACTH-independent macronodular adrenal hyperplasia 2 (AIMAH2) [MIM:615954]
ARMC5 Q96C12 VAR_079112 p.Pro483Leu LB/B rs552657393 -
ARMC5 Q96C12 VAR_079113 p.Arg502His LB/B rs200054015 -
ARMC5 Q96C12 VAR_079114 p.Pro507Leu LB/B rs142376949 -
ARMC5 Q96C12 VAR_079115 p.Leu580Pro US - ACTH-independent macronodular adrenal hyperplasia 2 (AIMAH2) [MIM:615954]
ARMC5 Q96C12 VAR_079117 p.Thr643Met US rs370836071 ACTH-independent macronodular adrenal hyperplasia 2 (AIMAH2) [MIM:615954]
ARMC5 Q96C12 VAR_079118 p.Cys657Arg LP/P - ACTH-independent macronodular adrenal hyperplasia 2 (AIMAH2) [MIM:615954]
ARMC5 Q96C12 VAR_079119 p.Cys657Trp LP/P - ACTH-independent macronodular adrenal hyperplasia 2 (AIMAH2) [MIM:615954]
ARMC5 Q96C12 VAR_079120 p.Ile664Ser LP/P - ACTH-independent macronodular adrenal hyperplasia 2 (AIMAH2) [MIM:615954]
ARMC5 Q96C12 VAR_079123 p.Pro731Arg LP/P rs200951744 ACTH-independent macronodular adrenal hyperplasia 2 (AIMAH2) [MIM:615954]
ARMC5 Q96C12 VAR_079124 p.Tyr736Ser LP/P - ACTH-independent macronodular adrenal hyperplasia 2 (AIMAH2) [MIM:615954]
ARMC5 Q96C12 VAR_079125 p.Leu754Pro LP/P - ACTH-independent macronodular adrenal hyperplasia 2 (AIMAH2) [MIM:615954]
ARMC5 Q96C12 VAR_079128 p.His808Pro LP/P - ACTH-independent macronodular adrenal hyperplasia 2 (AIMAH2) [MIM:615954]
ARMC5 Q96C12 VAR_079129 p.Pro826Thr US - ACTH-independent macronodular adrenal hyperplasia 2 (AIMAH2) [MIM:615954]
ARMC9 Q7Z3E5 VAR_031170 p.Leu108Phe LB/B rs11558175 -
ARMC9 Q7Z3E5 VAR_031171 p.Ile209Thr LB/B rs16827883 -
ARMC9 Q7Z3E5 VAR_031172 p.Arg222His LB/B rs3752780 -
ARMC9 Q7Z3E5 VAR_056739 p.Ile180Val LB/B rs1626450 -
ARMC9 Q7Z3E5 VAR_069411 p.Asp330Asn LB/B - -
ARMC9 Q7Z3E5 VAR_080497 p.Gly69Arg US rs750247691 Joubert syndrome 30 (JBTS30) [MIM:617622]
ARMC9 Q7Z3E5 VAR_080499 p.Arg343Cys US rs759799287 Joubert syndrome 30 (JBTS30) [MIM:617622]
ARMC9 Q7Z3E5 VAR_080500 p.Arg446Cys US rs753432312 Joubert syndrome 30 (JBTS30) [MIM:617622]
ARMC9 Q7Z3E5 VAR_080501 p.Gly492Arg US rs780265931 Joubert syndrome 30 (JBTS30) [MIM:617622]
ARMC9 Q7Z3E5 VAR_080502 p.Pro520Leu US rs1114167449 Joubert syndrome 30 (JBTS30) [MIM:617622]
ARMCX4 Q5H9R4 VAR_024761 p.Arg84Gly LB/B rs5951332 -
ARMH4 Q86TY3 VAR_027782 p.Thr96Ile LB/B rs3829765 -
ARMH4 Q86TY3 VAR_027783 p.Ala391Val LB/B rs1018504 -
ARMH4 Q86TY3 VAR_027784 p.Val528Phe LB/B rs12886921 -
ARMH4 Q86TY3 VAR_027785 p.Gln613Glu LB/B rs2273442 -
ARMS2 P0C7Q2 VAR_044330 p.Arg3His LB/B rs10490923 -

ARMS2 P0C7Q2 VAR_044331 p.Ala69Ser LB/B rs10490924 -
ARMT1 Q9H993 VAR_025791 p.Pro77Arg LB/B rs17850732 -
ARMT1 Q9H993 VAR_053090 p.Lys73Asn LB/B rs35036943 -
ARMT1 Q9H993 VAR_053091 p.Gly150Glu LB/B rs35734927 -
ARMT1 Q9H993 VAR_053092 p.Ser154Ala LB/B rs34437617 -
ARMT1 Q9H993 VAR_053093 p.His161Pro LB/B rs36037706 -
ARMT1 Q9H993 VAR_053094 p.Ile264Val LB/B rs35989216 -
ARMT1 Q9H993 VAR_053095 p.Ala317Thr LB/B rs35972078 -
ARNT P27540 VAR_014819 p.Asp511Asn LB/B rs1805133 -
ARNT P27540 VAR_018906 p.Asp517Glu LB/B rs10305741 -
ARNT P27540 VAR_020189 p.Pro706Leu LB/B rs2275237 -
ARNT P27540 VAR_024280 p.Arg430Gln LB/B rs2229175 -
ARNT2 Q9HBZ2 VAR_049538 p.Gly679Ser LB/B rs4072568 -
ARNT2 Q9HBZ2 VAR_076841 p.Arg46Trp LB/B rs140468271 -
ARNT2 Q9HBZ2 VAR_076842 p.Arg107His LB/B rs371290912 -
ARNT2 Q9HBZ2 VAR_076843 p.Arg402Gln LB/B rs141193900 -
ARNT2 Q9HBZ2 VAR_076844 p.Trp410Arg LB/B rs150964641 -
ARNTL2 Q8WYA1 VAR_030158 p.Asn340Ser LB/B rs1037921 -
ARNTL2 Q8WYA1 VAR_030159 p.Ala574Val LB/B rs11049005 -
ARPC1B O15143 VAR_014477 p.Lys37Asn LB/B rs1045012 -
ARPC1B O15143 VAR_080353 p.Ala105Val US - Platelet abnormalities with eosinophilia and immune-mediated inflammatory disease (PLTEID) [MIM:617718]
ARPC1B O15143 VAR_080354 p.Ala238Thr US rs147238850 Platelet abnormalities with eosinophilia and immune-mediated inflammatory disease (PLTEID) [MIM:617718]
ARR3 P36575 VAR_025520 p.Leu44Phe LB/B rs11548182 -
ARR3 P36575 VAR_080595 p.Leu80Pro US rs1555941116 Myopia 26, X-linked, female-limited (MYP26) [MIM:301010]
ARR3 P36575 VAR_080597 p.Ala298Asp US rs765658563 Myopia 26, X-linked, female-limited (MYP26) [MIM:301010]
ARRDC1 Q8N5I2 VAR_048335 p.Gly363Cys LB/B rs35018943 -
ARRDC2 Q8TBH0 VAR_026895 p.Arg181His LB/B rs17852061 -
ARRDC2 Q8TBH0 VAR_026896 p.Arg192His LB/B rs17852062 -
ARRDC2 Q8TBH0 VAR_026897 p.Ala244Thr LB/B rs8110271 -
ARRDC2 Q8TBH0 VAR_026898 p.Leu396Pro LB/B rs7259041 -
ARRDC4 Q8NCT1 VAR_026899 p.Thr79Ala LB/B rs12101554 -
ARRDC4 Q8NCT1 VAR_026900 p.Pro347Ser LB/B rs17856817 -
ARRDC4 Q8NCT1 VAR_026901 p.Ser358Pro LB/B rs2130882 -
ARSA P15289 VAR_007243 p.Leu76Pro LB/B rs199476362 -
ARSA P15289 VAR_007244 p.Pro82Leu LP/P rs6151411 Metachromatic leukodystrophy (MLD) [MIM:250100]
ARSA P15289 VAR_007245 p.Arg84Gln LP/P rs74315458 Metachromatic leukodystrophy (MLD) [MIM:250100]
ARSA P15289 VAR_007246 p.Gly86Asp LP/P rs74315460 Metachromatic leukodystrophy (MLD) [MIM:250100]
ARSA P15289 VAR_007247 p.Ser95Asn LP/P rs199476363 Metachromatic leukodystrophy (MLD) [MIM:250100]
ARSA P15289 VAR_007248 p.Ser96Phe LP/P rs74315456 Metachromatic leukodystrophy (MLD) [MIM:250100]

ARSA P15289 VAR_007249 p.Ser96Leu LP/P rs199476371 Metachromatic leukodystrophy (MLD) [MIM:250100]
ARSA P15289 VAR_007250 p.Gly99Asp LP/P rs74315455 Metachromatic leukodystrophy (MLD) [MIM:250100]
ARSA P15289 VAR_007251 p.Gly119Arg LP/P rs199476364 Metachromatic leukodystrophy (MLD) [MIM:250100]
ARSA P15289 VAR_007252 p.Gly122Ser LP/P rs74315461 Metachromatic leukodystrophy (MLD) [MIM:250100]
ARSA P15289 VAR_007253 p.Leu135Pro LP/P rs121434215 Metachromatic leukodystrophy (MLD) [MIM:250100]
ARSA P15289 VAR_007254 p.Pro136Leu LP/P rs74315462 Metachromatic leukodystrophy (MLD) [MIM:250100]
ARSA P15289 VAR_007255 p.Asp152Tyr LP/P rs199476365 Metachromatic leukodystrophy (MLD) [MIM:250100]
ARSA P15289 VAR_007256 p.Gly154Asp LP/P rs74315463 Metachromatic leukodystrophy (MLD) [MIM:250100]
ARSA P15289 VAR_007257 p.Pro155Arg LP/P rs74315464 Metachromatic leukodystrophy (MLD) [MIM:250100]
ARSA P15289 VAR_007258 p.Pro167Arg LP/P rs74315465 Metachromatic leukodystrophy (MLD) [MIM:250100]
ARSA P15289 VAR_007259 p.Asp169Asn LP/P rs74315466 Metachromatic leukodystrophy (MLD) [MIM:250100]
ARSA P15289 VAR_007260 p.Cys172Tyr LP/P rs199476381 Metachromatic leukodystrophy (MLD) [MIM:250100]
ARSA P15289 VAR_007261 p.Ile179Ser LP/P rs74315457 Metachromatic leukodystrophy (MLD) [MIM:250100]
ARSA P15289 VAR_007262 p.Trp193Cys LB/B rs6151415 -
ARSA P15289 VAR_007263 p.Tyr201Cys LP/P rs199476345 Metachromatic leukodystrophy (MLD) [MIM:250100]
ARSA P15289 VAR_007264 p.Ala212Val LP/P rs74315467 Metachromatic leukodystrophy (MLD) [MIM:250100]
ARSA P15289 VAR_007265 p.Ala224Val LP/P rs74315468 Metachromatic leukodystrophy (MLD) [MIM:250100]
ARSA P15289 VAR_007266 p.Pro231Thr LP/P rs74315469 Metachromatic leukodystrophy (MLD) [MIM:250100]
ARSA P15289 VAR_007267 p.Arg244Cys LP/P rs74315470 Metachromatic leukodystrophy (MLD) [MIM:250100]
ARSA P15289 VAR_007268 p.Arg244His LP/P rs199476366 Metachromatic leukodystrophy (MLD) [MIM:250100]
ARSA P15289 VAR_007269 p.Gly245Arg LP/P rs74315471 Metachromatic leukodystrophy (MLD) [MIM:250100]
ARSA P15289 VAR_007270 p.Ser250Tyr LP/P rs199476367 Metachromatic leukodystrophy (MLD) [MIM:250100]
ARSA P15289 VAR_007271 p.Thr274Met LP/P rs74315472 Metachromatic leukodystrophy (MLD) [MIM:250100]
ARSA P15289 VAR_007272 p.Arg288Cys LP/P rs74315473 Metachromatic leukodystrophy (MLD) [MIM:250100]
ARSA P15289 VAR_007273 p.Ser295Tyr LP/P rs74315474 Metachromatic leukodystrophy (MLD) [MIM:250100]

ARSA P15289 VAR_007274 p.Gly309Ser LP/P rs74315459 Metachromatic leukodystrophy (MLD) [MIM:250100]
ARSA P15289 VAR_007275 p.Arg311Gln LP/P rs199476382 Metachromatic leukodystrophy (MLD) [MIM:250100]
ARSA P15289 VAR_007276 p.Ala314Thr LP/P rs199476368 Metachromatic leukodystrophy (MLD) [MIM:250100]
ARSA P15289 VAR_007277 p.Asp335Val LP/P rs74315475 Metachromatic leukodystrophy (MLD) [MIM:250100]
ARSA P15289 VAR_007278 p.Asn350Ser LB/B rs2071421 -
ARSA P15289 VAR_007279 p.Lys367Asn LP/P rs199476369 Metachromatic leukodystrophy (MLD) [MIM:250100]
ARSA P15289 VAR_007280 p.Arg370Gln LP/P rs74315477 Metachromatic leukodystrophy (MLD) [MIM:250100]
ARSA P15289 VAR_007281 p.Arg370Trp LP/P rs74315476 Metachromatic leukodystrophy (MLD) [MIM:250100]
ARSA P15289 VAR_007282 p.Pro377Leu LP/P rs74315478 Metachromatic leukodystrophy (MLD) [MIM:250100]
ARSA P15289 VAR_007283 p.Glu382Lys LP/P rs74315479 Metachromatic leukodystrophy (MLD) [MIM:250100]
ARSA P15289 VAR_007284 p.Arg384Cys LP/P rs199476370 Metachromatic leukodystrophy (MLD) [MIM:250100]
ARSA P15289 VAR_007285 p.Arg390Gln LP/P rs199476391 Metachromatic leukodystrophy (MLD) [MIM:250100]
ARSA P15289 VAR_007286 p.Arg390Trp LP/P rs74315480 Metachromatic leukodystrophy (MLD) [MIM:250100]
ARSA P15289 VAR_007287 p.Thr391Ser LB/B rs743616 -
ARSA P15289 VAR_007288 p.His397Tyr LP/P rs199476376 Metachromatic leukodystrophy (MLD) [MIM:250100]
ARSA P15289 VAR_007291 p.Pro426Leu LP/P rs28940893 Metachromatic leukodystrophy (MLD) [MIM:250100]
ARSA P15289 VAR_007292 p.Ala464Val LB/B - -
ARSA P15289 VAR_007293 p.Arg496His LB/B rs6151428 -
ARSA P15289 VAR_008132 p.Cys300Phe LP/P rs74315484 Metachromatic leukodystrophy (MLD) [MIM:250100]
ARSA P15289 VAR_008133 p.Pro425Thr LP/P rs74315485 Metachromatic leukodystrophy (MLD) [MIM:250100]
ARSA P15289 VAR_018838 p.Phe356Val LB/B rs6151422 -
ARSA P15289 VAR_018839 p.Asn440Ser LB/B rs6151427 -
ARSA P15289 VAR_054164 p.Ala18Asp LP/P rs199476339 Metachromatic leukodystrophy (MLD) [MIM:250100]
ARSA P15289 VAR_054165 p.Asp29Asn LP/P rs199476346 Metachromatic leukodystrophy (MLD) [MIM:250100]
ARSA P15289 VAR_054166 p.Asp30His LP/P rs199476340 Metachromatic leukodystrophy (MLD) [MIM:250100]
ARSA P15289 VAR_054167 p.Gly32Ser LP/P rs199476350 Metachromatic leukodystrophy (MLD) [MIM:250100]
ARSA P15289 VAR_054168 p.Leu68Pro LP/P rs199476351 Metachromatic leukodystrophy (MLD) [MIM:250100]

ARSA P15289 VAR_054169 p.Arg84Trp LP/P rs199476352 Metachromatic leukodystrophy (MLD) [MIM:250100]
ARSA P15289 VAR_054170 p.Pro94Ala LP/P rs199476353 Metachromatic leukodystrophy (MLD) [MIM:250100]
ARSA P15289 VAR_054171 p.Gly99Val LP/P rs74315455 Metachromatic leukodystrophy (MLD) [MIM:250100]
ARSA P15289 VAR_054172 p.Pro136Ser LP/P rs60504011 Metachromatic leukodystrophy (MLD) [MIM:250100]
ARSA P15289 VAR_054174 p.Arg143Gly LP/P rs199476373 Metachromatic leukodystrophy (MLD) [MIM:250100]
ARSA P15289 VAR_054175 p.Pro148Leu LP/P rs199476375 Metachromatic leukodystrophy (MLD) [MIM:250100]
ARSA P15289 VAR_054176 p.Gln153His LP/P rs199476377 Metachromatic leukodystrophy (MLD) [MIM:250100]
ARSA P15289 VAR_054177 p.Pro155Leu LP/P rs74315464 Metachromatic leukodystrophy (MLD) [MIM:250100]
ARSA P15289 VAR_054178 p.Cys156Arg LP/P rs199476348 Metachromatic leukodystrophy (MLD) [MIM:250100]
ARSA P15289 VAR_054179 p.Leu181Gln LP/P rs199476378 Metachromatic leukodystrophy (MLD) [MIM:250100]
ARSA P15289 VAR_054180 p.Gln190His LP/P rs199476372 Metachromatic leukodystrophy (MLD) [MIM:250100]
ARSA P15289 VAR_054181 p.Pro191Thr LP/P rs199476374 Metachromatic leukodystrophy (MLD) [MIM:250100]
ARSA P15289 VAR_054182 p.Ala212Pro LP/P rs199476341 Metachromatic leukodystrophy (MLD) [MIM:250100]
ARSA P15289 VAR_054183 p.Arg217His LP/P rs148403406 Metachromatic leukodystrophy (MLD) [MIM:250100]
ARSA P15289 VAR_054184 p.Phe219Val LP/P rs199476383 Metachromatic leukodystrophy (MLD) [MIM:250100]
ARSA P15289 VAR_054185 p.His227Tyr LP/P rs199476354 Metachromatic leukodystrophy (MLD) [MIM:250100]
ARSA P15289 VAR_054186 p.Phe247Ser LP/P rs199476384 Metachromatic leukodystrophy (MLD) [MIM:250100]
ARSA P15289 VAR_054187 p.Glu253Lys LP/P rs74315483 Metachromatic leukodystrophy (MLD) [MIM:250100]
ARSA P15289 VAR_054188 p.Asp255His LP/P rs80338819 Metachromatic leukodystrophy (MLD) [MIM:250100]
ARSA P15289 VAR_054189 p.Asp281Tyr LP/P rs199476386 Metachromatic leukodystrophy (MLD) [MIM:250100]
ARSA P15289 VAR_054190 p.Asn282Ser LP/P rs199476342 Metachromatic leukodystrophy (MLD) [MIM:250100]
ARSA P15289 VAR_054191 p.Thr286Pro LP/P rs28940894 Metachromatic leukodystrophy (MLD) [MIM:250100]
ARSA P15289 VAR_054192 p.Arg288His LP/P rs199476355 Metachromatic leukodystrophy (MLD) [MIM:250100]
ARSA P15289 VAR_054193 p.Gly293Asp LP/P rs199476387 Metachromatic leukodystrophy (MLD) [MIM:250100]

ARSA	P15289	VAR_054194	p.Gly293Ser	LP/P	rs199476349	Metachromatic leukodystrophy (MLD) [MIM:250100]
ARSA	P15289	VAR_054195	p.Cys294Tyr	LP/P	rs199476347	Metachromatic leukodystrophy (MLD) [MIM:250100]
ARSA	P15289	VAR_054196	p.Leu298Ser	LP/P	rs199476389	Metachromatic leukodystrophy (MLD) [MIM:250100]
ARSA	P15289	VAR_054197	p.Lys302Asn	LP/P	rs199476343	Metachromatic leukodystrophy (MLD) [MIM:250100]
ARSA	P15289	VAR_054198	p.Tyr306His	LP/P	rs199476379	Metachromatic leukodystrophy (MLD) [MIM:250100]
ARSA	P15289	VAR_054199	p.Gly308Asp	LP/P	rs199476356	Metachromatic leukodystrophy (MLD) [MIM:250100]
ARSA	P15289	VAR_054200	p.Gly308Val	LP/P	rs199476356	Metachromatic leukodystrophy (MLD) [MIM:250100]
ARSA	P15289	VAR_054201	p.Glu312Asp	LP/P	rs199476390	Metachromatic leukodystrophy (MLD) [MIM:250100]
ARSA	P15289	VAR_054202	p.Gly325Ser	LP/P	rs148092995	Metachromatic leukodystrophy (MLD) [MIM:250100]
ARSA	P15289	VAR_054203	p.Thr327Ile	LP/P	-	Metachromatic leukodystrophy (MLD) [MIM:250100]
ARSA	P15289	VAR_054204	p.Tyr376Asn	LP/P	rs199476344	Metachromatic leukodystrophy (MLD) [MIM:250100]
ARSA	P15289	VAR_054205	p.Asp381Glu	LP/P	rs6151425	Metachromatic leukodystrophy (MLD) [MIM:250100]
ARSA	P15289	VAR_054206	p.Thr408Ile	LP/P	rs28940895	Metachromatic leukodystrophy (MLD) [MIM:250100]
ARSA	P15289	VAR_054207	p.Thr409Ile	LP/P	rs74315481	Metachromatic leukodystrophy (MLD) [MIM:250100]
ARSA	P15289	VAR_054208	p.Leu428Pro	LP/P	rs199476392	Metachromatic leukodystrophy (MLD) [MIM:250100]
ARSA	P15289	VAR_054209	p.Tyr429Ser	LP/P	rs199476380	Metachromatic leukodystrophy (MLD) [MIM:250100]
ARSA	P15289	VAR_054210	p.Ala469Gly	LP/P	rs199476385	Metachromatic leukodystrophy (MLD) [MIM:250100]
ARSA	P15289	VAR_054211	p.Cys489Gly	LP/P	rs199476388	Metachromatic leukodystrophy (MLD) [MIM:250100]
ARSA	P15289	VAR_067414	p.Leu52Pro	LP/P	rs199476357	Metachromatic leukodystrophy (MLD) [MIM:250100]
ARSA	P15289	VAR_067415	p.His138Asp	LP/P	rs199476358	Metachromatic leukodystrophy (MLD) [MIM:250100]
ARSA	P15289	VAR_067416	p.Thr304Met	LP/P	rs199476359	Metachromatic leukodystrophy (MLD) [MIM:250100]
ARSA	P15289	VAR_067417	p.Glu307Lys	LP/P	rs199476360	Metachromatic leukodystrophy (MLD) [MIM:250100]
ARSA	P15289	VAR_067418	p.Ser406Gly	LP/P	rs199476361	Metachromatic leukodystrophy (MLD) [MIM:250100]
ARSB	P15848	VAR_007294	p.Thr92Met	LP/P	rs751010538	Mucopolysaccharidosis 6 (MPS6) [MIM:253200]
ARSB	P15848	VAR_007295	p.Arg95Gln	LP/P	rs118203942	Mucopolysaccharidosis 6 (MPS6) [MIM:253200]
ARSB	P15848	VAR_007296	p.Cys117Arg	LP/P	rs118203939	Mucopolysaccharidosis 6 (MPS6) [MIM:253200]
ARSB	P15848	VAR_007297	p.Gly137Val	LP/P	rs118203938	Mucopolysaccharidosis 6 (MPS6) [MIM:253200]

ARSB	P15848	VAR_007298	p.Arg152Trp	LP/P	rs991104525	Mucopolysaccharidosis 6 (MPS6) [MIM:253200]
ARSB	P15848	VAR_007299	p.Arg160Gln	LP/P	rs1196325597	Mucopolysaccharidosis 6 (MPS6) [MIM:253200]
ARSB	P15848	VAR_007300	p.Tyr210Cys	LP/P	rs118203943	Mucopolysaccharidosis 6 (MPS6) [MIM:253200]
ARSB	P15848	VAR_007301	p.Leu236Pro	LP/P	rs118203940	Mucopolysaccharidosis 6 (MPS6) [MIM:253200]
ARSB	P15848	VAR_007302	p.Gly302Arg	LP/P	rs779378413	Mucopolysaccharidosis 6 (MPS6) [MIM:253200]
ARSB	P15848	VAR_007303	p.Val376Met	LB/B	rs1071598	-
ARSB	P15848	VAR_007304	p.His393Pro	LP/P	rs118203944	Mucopolysaccharidosis 6 (MPS6) [MIM:253200]
ARSB	P15848	VAR_007305	p.Cys405Tyr	LP/P	rs118203941	Mucopolysaccharidosis 6 (MPS6) [MIM:253200]
ARSB	P15848	VAR_007306	p.Leu498Pro	LP/P	rs774358117	Mucopolysaccharidosis 6 (MPS6) [MIM:253200]
ARSB	P15848	VAR_016061	p.Val358Met	LB/B	rs1065757	-
ARSB	P15848	VAR_019017	p.Ser65Phe	LP/P	rs1233331806	Mucopolysaccharidosis 6 (MPS6) [MIM:253200]
ARSB	P15848	VAR_019019	p.Pro116His	LP/P	rs775780931	Mucopolysaccharidosis 6 (MPS6) [MIM:253200]
ARSB	P15848	VAR_019020	p.Met142Ile	LP/P	rs1554088053	Mucopolysaccharidosis 6 (MPS6) [MIM:253200]
ARSB	P15848	VAR_019021	p.Gly144Arg	LP/P	rs746206847	Mucopolysaccharidosis 6 (MPS6) [MIM:253200]
ARSB	P15848	VAR_019022	p.Trp146Leu	LP/P	rs1554088034	Mucopolysaccharidosis 6 (MPS6) [MIM:253200]
ARSB	P15848	VAR_019023	p.Trp146Arg	LP/P	rs1554088037	Mucopolysaccharidosis 6 (MPS6) [MIM:253200]
ARSB	P15848	VAR_019024	p.Trp146Ser	LP/P	rs1554088034	Mucopolysaccharidosis 6 (MPS6) [MIM:253200]
ARSB	P15848	VAR_019025	p.Cys192Arg	LP/P	rs1554087423	Mucopolysaccharidosis 6 (MPS6) [MIM:253200]
ARSB	P15848	VAR_019026	p.Gln239Arg	LP/P	rs1554086431	Mucopolysaccharidosis 6 (MPS6) [MIM:253200]
ARSB	P15848	VAR_019027	p.Trp312Cys	LP/P	rs759384989	Mucopolysaccharidosis 6 (MPS6) [MIM:253200]
ARSB	P15848	VAR_019028	p.Arg315Gln	LP/P	rs727503809	Mucopolysaccharidosis 6 (MPS6) [MIM:253200]
ARSB	P15848	VAR_019029	p.Leu321Pro	LP/P	rs1554079320	Mucopolysaccharidosis 6 (MPS6) [MIM:253200]
ARSB	P15848	VAR_019030	p.Ser384Asn	LB/B	rs25414	-
ARSB	P15848	VAR_019031	p.Phe399Leu	LP/P	rs200793396	Mucopolysaccharidosis 6 (MPS6) [MIM:253200]
ARSB	P15848	VAR_019032	p.Arg484Gly	LP/P	rs201101343	Mucopolysaccharidosis 6 (MPS6) [MIM:253200]
ARSB	P15848	VAR_019033	p.Cys521Tyr	LP/P	rs1554069661	Mucopolysaccharidosis 6 (MPS6) [MIM:253200]
ARSB	P15848	VAR_019034	p.Pro531Arg	LP/P	rs1554069659	Mucopolysaccharidosis 6 (MPS6) [MIM:253200]
ARSB	P15848	VAR_061883	p.Val358Leu	LB/B	rs1065757	-
ARSB	P15848	VAR_080270	p.Leu82Arg	LP/P	rs749465732	Mucopolysaccharidosis 6 (MPS6) [MIM:253200]
ARSD	P51689	VAR_052508	p.Ser224Cys	LB/B	rs211653	-
ARSD	P51689	VAR_052509	p.Val500Ile	LB/B	rs2229557	-
ARSD	P51689	VAR_052510	p.Met564Thr	LB/B	rs2228431	-
ARSF	P54793	VAR_058846	p.His527Tyr	LB/B	rs1052638	-
ARSG	Q96EG1	VAR_052511	p.Ala11Val	LB/B	rs8074806	-
ARSG	Q96EG1	VAR_052512	p.Thr236Ser	LB/B	rs1558876	-
ARSG	Q96EG1	VAR_052513	p.Trp274Arg	LB/B	rs1558878	-
ARSG	Q96EG1	VAR_052514	p.Arg385His	LB/B	rs9972951	-
ARSG	Q96EG1	VAR_074038	p.Arg326Gly	LB/B	rs144503106	-
ARSG	Q96EG1	VAR_074039	p.Arg398Trp	LB/B	rs11657051	-
ARSG	Q96EG1	VAR_074040	p.Thr444Met	LB/B	rs62000424	-
ARSG	Q96EG1	VAR_074041	p.Glu481Lys	LB/B	rs370852507	-
ARSG	Q96EG1	VAR_074042	p.Ile493Thr	LB/B	rs61999318	-
ARSG	Q96EG1	VAR_081577	p.Asp45Tyr	LP/P	-	Usher syndrome 4 (USH4) [MIM:618144]
ARSJ	Q5FYB0	VAR_052515	p.Ser565Arg	LB/B	rs17046588	-
ARSK	Q6UWY0	VAR_052516	p.Gln525Arg	LB/B	rs17084927	-
ARSL	P51690	VAR_007307	p.Arg12Ser	LP/P	rs122460151	Chondrodysplasia punctata 1, X-linked recessive (CDPX1) [MIM:302950]
ARSL	P51690	VAR_007308	p.Arg111Pro	LP/P	rs122460153	Chondrodysplasia punctata 1, X-linked recessive (CDPX1) [MIM:302950]

ARSL P51690 VAR_007309 p.Gly117Arg LP/P rs122460152 Chondrodysplasia punctata 1, X-linked recessive (CDPX1) [MIM:302950]
ARSL P51690 VAR_007310 p.Gly137Val LP/P rs80338711 Chondrodysplasia punctata 1, X-linked recessive (CDPX1) [MIM:302950]
ARSL P51690 VAR_007311 p.Gly245Arg LP/P rs122460154 Chondrodysplasia punctata 1, X-linked recessive (CDPX1) [MIM:302950]
ARSL P51690 VAR_007312 p.Cys492Tyr LP/P rs122460155 Chondrodysplasia punctata 1, X-linked recessive (CDPX1) [MIM:302950]
ARSL P51690 VAR_023570 p.Ile80Asn LP/P - Chondrodysplasia punctata 1, X-linked recessive (CDPX1) [MIM:302950]
ARSL P51690 VAR_023571 p.Thr481Met LP/P rs80338713 Chondrodysplasia punctata 1, X-linked recessive (CDPX1) [MIM:302950]
ARSL P51690 VAR_023572 p.Pro578Ser LP/P rs28935474 Chondrodysplasia punctata 1, X-linked recessive (CDPX1) [MIM:302950]
ARSL P51690 VAR_037974 p.Arg183His LB/B rs34412194 -
ARSL P51690 VAR_037975 p.Gly424Ser LB/B rs35143646 -
ART1 P52961 VAR_034125 p.Pro105Leu LB/B rs35123761 -
ART1 P52961 VAR_034126 p.Pro126Arg LB/B rs35619488 -
ART1 P52961 VAR_053526 p.Leu257Pro LB/B rs2280134 -
ART3 Q13508 VAR_060072 p.Ser363Leu LB/B rs1128864 -
ART3 Q13508 VAR_081146 p.Pro334Ser US rs143599971 -
ART4 Q93070 VAR_013707 p.Gly108Val LB/B rs28362797 -
ART4 Q93070 VAR_013708 p.Thr117Ile LB/B rs28362798 -
ART4 Q93070 VAR_013709 p.Asn265Asp LB/B rs11276 -
ART4 Q93070 VAR_013710 p.Leu300Val LB/B rs3088190 -
ART4 Q93070 VAR_022266 p.Asp135Glu LB/B rs28362799 -
ART4 Q93070 VAR_022267 p.Thr189Met LB/B rs28362800 -
ARTN Q5T4W7 VAR_026718 p.Gln19Arg LB/B rs2242637 -
ARV1 Q9H2C2 VAR_033525 p.Gly101Glu LB/B rs35764859 -
ARV1 Q9H2C2 VAR_077051 p.Gly189Arg LP/P rs730882241 Developmental and epileptic encephalopathy 38 (DEE38) [MIM:617020]
ARVCF O00192 VAR_020408 p.Val175Ala LB/B rs2240717 -
ARVCF O00192 VAR_024692 p.Arg906Gln LB/B rs165815 -
ARVCF O00192 VAR_033529 p.Pro220Leu LB/B rs2073748 -
ARVCF O00192 VAR_033530 p.Arg909Trp LB/B rs34687532 -
ARVCF O00192 VAR_033531 p.Arg909Gln LB/B rs34638476 -
ARVCF O00192 VAR_033532 p.Arg912Trp LB/B rs34445280 -
ARVCF O00192 VAR_053812 p.Arg539Gln LB/B rs16982871 -
ARX Q96QS3 VAR_015178 p.Arg332His LP/P rs111033612 Lissencephaly, X-linked 2 (LISX2) [MIM:300215]
ARX Q96QS3 VAR_015179 p.Leu343Gln LP/P rs104894741 Lissencephaly, X-linked 2 (LISX2) [MIM:300215]
ARX Q96QS3 VAR_015180 p.Pro353Leu LP/P rs104894743 Developmental and epileptic encephalopathy 1 (DEE1) [MIM:308350]
ARX Q96QS3 VAR_015669 p.Leu33Pro LP/P rs28936077 Mental retardation, X-linked, with or without seizures, ARX-related (MRXARX) [MIM:300419]
ARX Q96QS3 VAR_015671 p.Gly286Ser LP/P rs28935479 Mental retardation, X-linked, with or without seizures, ARX-related (MRXARX) [MIM:300419]
ARX Q96QS3 VAR_033260 p.Arg332Pro LP/P - Lissencephaly, X-linked 2 (LISX2) [MIM:300215]
ARX Q96QS3 VAR_033261 p.Thr333Asn LP/P rs104894745 Agenesis of the corpus callosum, with abnormal genitalia (ACCAG) [MIM:300004]

ARX Q96QS3 VAR_033262 p.Pro353Arg LP/P - Lissencephaly, X-linked 2 (LISX2) [MIM:300215]
ARX Q96QS3 VAR_033263 p.Ala521Thr LP/P rs746120093 Lissencephaly, X-linked 2 (LISX2) [MIM:300215]
AS3MT Q9HBK9 VAR_027392 p.Arg173Trp LB/B rs35232887 -
AS3MT Q9HBK9 VAR_027393 p.Met287Thr LB/B rs11191439 -
AS3MT Q9HBK9 VAR_027394 p.Thr306Ile LB/B rs34556438 -
ASAH1 Q13510 VAR_008860 p.Val72Met LB/B rs1071645 -
ASAH1 Q13510 VAR_008861 p.Ile93Val LB/B rs1049874 -
ASAH1 Q13510 VAR_008862 p.Thr222Lys LP/P rs137853593 Farber lipogranulomatosis (FRBRL) [MIM:228000]
ASAH1 Q13510 VAR_021579 p.Tyr36Cys LP/P rs137853595 Farber lipogranulomatosis (FRBRL) [MIM:228000]
ASAH1 Q13510 VAR_021581 p.Val97Glu LP/P - Farber lipogranulomatosis (FRBRL) [MIM:228000]
ASAH1 Q13510 VAR_021582 p.Glu138Val LP/P rs137853594 Farber lipogranulomatosis (FRBRL) [MIM:228000]
ASAH1 Q13510 VAR_021583 p.Gly235Arg LP/P rs1554808625 Farber lipogranulomatosis (FRBRL) [MIM:228000]
ASAH1 Q13510 VAR_021584 p.Arg254Gly US - Farber lipogranulomatosis (FRBRL) [MIM:228000]
ASAH1 Q13510 VAR_021585 p.Asn320Asp LP/P rs137853596 Farber lipogranulomatosis (FRBRL) [MIM:228000]
ASAH1 Q13510 VAR_021586 p.Pro362Arg LP/P - Farber lipogranulomatosis (FRBRL) [MIM:228000]
ASAH1 Q13510 VAR_021587 p.Val369Ile LB/B rs17636067 -
ASAH1 Q13510 VAR_038166 p.Gln22His LP/P - Farber lipogranulomatosis (FRBRL) [MIM:228000]
ASAH1 Q13510 VAR_038167 p.His23Asp LP/P - Farber lipogranulomatosis (FRBRL) [MIM:228000]
ASAH1 Q13510 VAR_038168 p.Asp124Glu LB/B rs2472205 -
ASAH1 Q13510 VAR_038169 p.Leu182Val LP/P rs137853597 Farber lipogranulomatosis (FRBRL) [MIM:228000]
ASAH1 Q13510 VAR_038170 p.Val246Ala LB/B rs10103355 -
ASAH1 Q13510 VAR_057979 p.Ala70Val LB/B rs10103355 -
ASAH1 Q13510 VAR_057980 p.Val88Met LB/B rs1071645 -
ASAH1 Q13510 VAR_068722 p.Thr42Met LP/P rs145873635 Spinal muscular atrophy with progressive myoclonic epilepsy (SMAPME) [MIM:159950]
ASAH1 Q13510 VAR_071994 p.Val97Gly LP/P - Farber lipogranulomatosis (FRBRL) [MIM:228000]
ASAH1 Q13510 VAR_071995 p.Gly168Trp LP/P - Farber lipogranulomatosis (FRBRL) [MIM:228000]
ASAH1 Q13510 VAR_072247 p.Lys152Asn LP/P rs200455852 Spinal muscular atrophy with progressive myoclonic epilepsy (SMAPME) [MIM:159950]
ASAH1 Q13510 VAR_081279 p.Thr42Ala US rs779888892 Spinal muscular atrophy with progressive myoclonic epilepsy (SMAPME) [MIM:159950]
ASAH1 Q13510 VAR_081280 p.Trp169Arg US rs756455049 Farber lipogranulomatosis (FRBRL) [MIM:228000]
ASAH1 Q13510 VAR_081282 p.Arg333Cys US rs543697946 Farber lipogranulomatosis (FRBRL) [MIM:228000]
ASAH2 Q9NR71 VAR_027064 p.Thr51Ala LB/B rs7067625 -
ASAH2 Q9NR71 VAR_027065 p.Ala346Ser LB/B rs1052952953 -
ASAP1 Q9ULH1 VAR_055528 p.Ile728Val LB/B rs966185 -
ASAP2 O43150 VAR_020307 p.Glu748Asp LB/B rs2715860 -
ASAP3 Q8TDY4 VAR_035612 p.Ala617Thr US - A colorectal cancer sample
ASAP3 Q8TDY4 VAR_048295 p.Glu377Ala LB/B rs16828486 -
ASB10 Q8WXI3 VAR_022090 p.Arg453Cys LB/B rs3800791 -
ASB10 Q8WXI3 VAR_069867 p.Thr48Ser US rs151344615 Glaucoma 1, open angle, F (GLC1F) [MIM:603383]
ASB10 Q8WXI3 VAR_069868 p.Gly65Glu US rs104886491 Glaucoma 1, open angle, F (GLC1F) [MIM:603383]
ASB10 Q8WXI3 VAR_069869 p.Val67Met US rs151344616 Glaucoma 1, open angle, F (GLC1F) [MIM:603383]
ASB10 Q8WXI3 VAR_069870 p.Arg72His US rs104886488 Glaucoma 1, open angle, F (GLC1F) [MIM:603383]
ASB10 Q8WXI3 VAR_069871 p.Asp88Val LB/B rs151344617 -
ASB10 Q8WXI3 VAR_069872 p.Asp91Tyr LB/B rs104886490 -

ASB10	Q8WXI3	VAR_069873	p.Arg94Gln	US	rs147737381	Glaucoma 1, open angle, F (GLC1F) [MIM:603383]
ASB10	Q8WXI3	VAR_069874	p.Asp97Glu	US	rs151344619	Glaucoma 1, open angle, F (GLC1F) [MIM:603383]
ASB10	Q8WXI3	VAR_069875	p.Ala172Val	LB/B	rs151344604	-
ASB10	Q8WXI3	VAR_069876	p.Arg183Cys	US	rs151344605	Glaucoma 1, open angle, F (GLC1F) [MIM:603383]
ASB10	Q8WXI3	VAR_069877	p.Arg189Trp	LB/B	rs104886473	-
ASB10	Q8WXI3	VAR_069878	p.Ala197Val	US	rs151344607	Glaucoma 1, open angle, F (GLC1F) [MIM:603383]
ASB10	Q8WXI3	VAR_069879	p.Val207Leu	US	rs104886474	Glaucoma 1, open angle, F (GLC1F) [MIM:603383]
ASB10	Q8WXI3	VAR_069880	p.Arg237Gly	LB/B	rs61735708	-
ASB10	Q8WXI3	VAR_069881	p.Arg272His	US	rs140602973	Glaucoma 1, open angle, F (GLC1F) [MIM:603383]
ASB10	Q8WXI3	VAR_069882	p.Gln295Leu	US	rs151344609	Glaucoma 1, open angle, F (GLC1F) [MIM:603383]
ASB10	Q8WXI3	VAR_069883	p.Arg304Cys	LB/B	rs61735130	-
ASB10	Q8WXI3	VAR_069884	p.Ala320Thr	US	rs151344610	Glaucoma 1, open angle, F (GLC1F) [MIM:603383]
ASB10	Q8WXI3	VAR_069885	p.Thr329Met	LB/B	rs104886481	-
ASB10	Q8WXI3	VAR_069886	p.His332Gln	US	rs104886482	Glaucoma 1, open angle, F (GLC1F) [MIM:603383]
ASB10	Q8WXI3	VAR_069887	p.His356Tyr	US	rs151344611	Glaucoma 1, open angle, F (GLC1F) [MIM:603383]
ASB10	Q8WXI3	VAR_069888	p.Arg360His	US	rs151344612	Glaucoma 1, open angle, F (GLC1F) [MIM:603383]
ASB10	Q8WXI3	VAR_069889	p.Arg372Cys	LB/B	rs62489646	-
ASB10	Q8WXI3	VAR_069890	p.Pro402Thr	LB/B	rs919533	-
ASB10	Q8WXI3	VAR_069891	p.Ser440Gly	US	rs104886487	Glaucoma 1, open angle, F (GLC1F) [MIM:603383]
ASB11	Q8WXH4	VAR_048286	p.Asp249Asn	LB/B	rs34025595	-
ASB11	Q8WXH4	VAR_048287	p.Ser263Gly	LB/B	rs35859007	-
ASB11	Q8WXH4	VAR_069428	p.Ser165Leu	LB/B	rs144572145	-
ASB15	Q8WXK1	VAR_060466	p.Pro57Leu	LB/B	rs6962756	-
ASB15	Q8WXK1	VAR_060467	p.Gly357Ala	LB/B	rs4731112	-
ASB16	Q96NS5	VAR_059127	p.Thr240Ile	LB/B	rs7224330	-
ASB16	Q96NS5	VAR_064697	p.Thr173Pro	US	-	-
ASB16-AS1	Q495Z4	VAR_032127	p.Ser32Arg	LB/B	rs7217858	-
ASB16-AS1	Q495Z4	VAR_061623	p.Cys114Arg	LB/B	rs7212573	-
ASB17	Q8WXJ9	VAR_024174	p.Ser2Asn	LB/B	rs3795251	-
ASB17	Q8WXJ9	VAR_048288	p.Val101Ala	LB/B	rs1796814	-
ASB18	Q6ZVZ8	VAR_037331	p.Ala127Pro	LB/B	rs7588748	-
ASB18	Q6ZVZ8	VAR_048289	p.Ala104Thr	LB/B	rs6756597	-
ASB18	Q6ZVZ8	VAR_048290	p.His407Asn	LB/B	rs10177957	-
ASB18	Q6ZVZ8	VAR_059128	p.Gly261Ser	LB/B	rs6431437	-
ASB2	Q96Q27	VAR_022089	p.Pro160Ser	LB/B	rs2295213	-
ASB4	Q9Y574	VAR_033512	p.Val17Leu	LB/B	rs35047380	-
ASCC1	Q8N9N2	VAR_061278	p.Asp34Asn	LB/B	rs11558719	-
ASCC1	Q8N9N2	VAR_066588	p.Asn318Ser	LB/B	rs146370051	-
ASCC2	Q9H1I8	VAR_019464	p.Arg509Gln	LB/B	rs4823054	-
ASCC2	Q9H1I8	VAR_025512	p.Asp407His	LB/B	rs28265	-
ASCC2	Q9H1I8	VAR_025513	p.Pro423Ser	LB/B	rs36571	-
ASCC2	Q9H1I8	VAR_025514	p.Arg639Leu	LB/B	rs6006259	-
ASCC2	Q9H1I8	VAR_050675	p.Arg96Cys	LB/B	rs1894473	-
ASCC2	Q9H1I8	VAR_050676	p.Val123Ile	LB/B	rs11549795	-
ASCC2	Q9H1I8	VAR_050677	p.Asp546Gly	LB/B	rs34833047	-
ASCC2	Q9H1I8	VAR_050678	p.Glu588Lys	LB/B	rs34062345	-
ASCC3	Q8N3C0	VAR_034859	p.Leu146Phe	LB/B	rs9390698	-
ASCC3	Q8N3C0	VAR_034860	p.Val1050Ile	LB/B	rs9497983	-
ASCC3	Q8N3C0	VAR_034861	p.Cys1800Trp	LB/B	rs35011147	-

ASCC3 Q8N3C0 VAR_034862 p.Val1930Met LB/B rs3213542 -
ASCC3 Q8N3C0 VAR_034863 p.Ser1995Cys LB/B rs240780 -
ASCC3 Q8N3C0 VAR_034864 p.Tyr2176Cys LB/B rs240768 -
ASCC3 Q8N3C0 VAR_049339 p.Glu344Lys LB/B rs6918004 -
ASCC3 Q8N3C0 VAR_049340 p.Asn478Ser LB/B rs7750940 -
ASCC3 Q8N3C0 VAR_049341 p.Val1425Ala LB/B rs17246013 -
ASCC3 Q8N3C0 VAR_049342 p.Arg1497Thr LB/B rs17305382 -
ASCC3 Q8N3C0 VAR_061212 p.Ser1016Cys LB/B rs57534235 -
ASCL1 P50553 VAR_013179 p.Glu158Gly LB/B rs1803157 -
ASCL3 Q9NQ33 VAR_055948 p.Arg54Leu LB/B rs4909951 -
ASGR2 P07307 VAR_068747 p.Gly85Arg LB/B rs2304978 -
ASH1L Q9NR48 VAR_028949 p.Thr1771Ala LB/B rs4971053 -
ASH1L Q9NR48 VAR_055905 p.Ser1416Pro LB/B rs13373934 -
ASH1L Q9NR48 VAR_069405 p.Ser277Gly LB/B rs186255422 -
ASH1L Q9NR48 VAR_069406 p.Ala724Ser US rs1293246328 Mental retardation, autosomal dominant 52 (MRD52) [MIM:617796]
ASH1L Q9NR48 VAR_069407 p.Lys972Arg US - Mental retardation, autosomal dominant 52 (MRD52) [MIM:617796]
ASH1L Q9NR48 VAR_069408 p.Tyr1276His US rs539982914 Mental retardation, autosomal dominant 52 (MRD52) [MIM:617796]
ASH1L Q9NR48 VAR_069409 p.Cys1775Trp US rs753734834 Mental retardation, autosomal dominant 52 (MRD52) [MIM:617796]
ASH1L Q9NR48 VAR_069410 p.Asp2853Gly US - Mental retardation, autosomal dominant 52 (MRD52) [MIM:617796]
ASH1L Q9NR48 VAR_080559 p.Val2085Ile US rs749494995 Mental retardation, autosomal dominant 52 (MRD52) [MIM:617796]
ASH1L Q9NR48 VAR_080561 p.Arg2396His US rs753029013 Mental retardation, autosomal dominant 52 (MRD52) [MIM:617796]
ASH1L Q9NR48 VAR_080562 p.Ala2791Pro LP/P rs1553241570 Mental retardation, autosomal dominant 52 (MRD52) [MIM:617796]
ASH2L Q9UBL3 VAR_050679 p.Ser478Phe LB/B rs34167006 -
ASIC2 Q16515 VAR_052036 p.Asp354Gly LB/B rs16967895 -
ASIC3 Q9UHC3 VAR_052037 p.Asn228Ser LB/B rs1864545 -
ASIC4 Q96FT7 VAR_052038 p.Pro614Gln LB/B rs6436153 -
ASIC4 Q96FT7 VAR_052039 p.Arg616Leu LB/B rs11689281 -
ASIC4 Q96FT7 VAR_059806 p.Val619Ala LB/B rs11695248 -
ASIP P42127 VAR_005003 p.Gln61Pro LB/B rs1129414 -
ASIP P42127 VAR_022125 p.Val13Ala LB/B rs2296151 -
ASL P04424 VAR_000676 p.Arg95Cys LP/P rs28940585 Argininosuccinic aciduria (ARGINSA) [MIM:207900]
ASL P04424 VAR_000677 p.Arg111Trp LP/P rs138310841 Argininosuccinic aciduria (ARGINSA) [MIM:207900]
ASL P04424 VAR_000678 p.Arg193Gln LP/P rs373697663 Argininosuccinic aciduria (ARGINSA) [MIM:207900]
ASL P04424 VAR_000679 p.Gln286Arg LP/P rs28941472 Argininosuccinic aciduria (ARGINSA) [MIM:207900]
ASL P04424 VAR_017572 p.Val178Met LP/P rs28941473 Argininosuccinic aciduria (ARGINSA) [MIM:207900]
ASL P04424 VAR_017573 p.Arg379Cys LP/P rs28940287 Argininosuccinic aciduria (ARGINSA) [MIM:207900]
ASL P04424 VAR_017574 p.Arg385Cys LP/P rs28940286 Argininosuccinic aciduria (ARGINSA) [MIM:207900]
ASL P04424 VAR_036281 p.Thr181Ser US - A breast cancer sample
ASL P04424 VAR_036282 p.Gly200Val US - A breast cancer sample
ASL P04424 VAR_043106 p.Asp31Asn LP/P rs754995756 Argininosuccinic aciduria (ARGINSA) [MIM:207900]
ASL P04424 VAR_043107 p.Arg113Gln LP/P rs752783461 Argininosuccinic aciduria (ARGINSA) [MIM:207900]

ASL	P04424	VAR_043108	p.Arg186Gln	LP/P	rs752397242	Argininosuccinic aciduria (ARGINSA) [MIM:207900]
ASL	P04424	VAR_043109	p.Arg236Trp	LP/P	rs761268464	Argininosuccinic aciduria (ARGINSA) [MIM:207900]
ASL	P04424	VAR_043110	p.Val335Leu	LP/P	-	Argininosuccinic aciduria (ARGINSA) [MIM:207900]
ASL	P04424	VAR_043111	p.Met382Arg	LP/P	-	Argininosuccinic aciduria (ARGINSA) [MIM:207900]
ASL	P04424	VAR_043112	p.Arg456Trp	LP/P	rs759396688	Argininosuccinic aciduria (ARGINSA) [MIM:207900]
ASL	P04424	VAR_072186	p.Val70Ala	LP/P	rs1027739421	Argininosuccinic aciduria (ARGINSA) [MIM:207900]
ASL	P04424	VAR_072187	p.Arg94Cys	LP/P	rs374304304	Argininosuccinic aciduria (ARGINSA) [MIM:207900]
ASL	P04424	VAR_072188	p.Arg94His	LP/P	rs777437569	Argininosuccinic aciduria (ARGINSA) [MIM:207900]
ASL	P04424	VAR_072189	p.Arg95His	LP/P	rs150244667	Argininosuccinic aciduria (ARGINSA) [MIM:207900]
ASL	P04424	VAR_072190	p.Ala104Val	LP/P	-	Argininosuccinic aciduria (ARGINSA) [MIM:207900]
ASL	P04424	VAR_072191	p.Asp120Glu	LP/P	-	Argininosuccinic aciduria (ARGINSA) [MIM:207900]
ASL	P04424	VAR_072192	p.Leu121His	LP/P	-	Argininosuccinic aciduria (ARGINSA) [MIM:207900]
ASL	P04424	VAR_072193	p.Arg126Trp	LP/P	rs201962738	Argininosuccinic aciduria (ARGINSA) [MIM:207900]
ASL	P04424	VAR_072194	p.Arg146Trp	LP/P	rs199938613	Argininosuccinic aciduria (ARGINSA) [MIM:207900]
ASL	P04424	VAR_072195	p.Pro156Arg	LP/P	rs769017508	Argininosuccinic aciduria (ARGINSA) [MIM:207900]
ASL	P04424	VAR_072196	p.His160Asn	LP/P	-	Argininosuccinic aciduria (ARGINSA) [MIM:207900]
ASL	P04424	VAR_072197	p.Pro166His	LP/P	-	Argininosuccinic aciduria (ARGINSA) [MIM:207900]
ASL	P04424	VAR_072198	p.Arg168His	LP/P	rs727503811	Argininosuccinic aciduria (ARGINSA) [MIM:207900]
ASL	P04424	VAR_072199	p.Ser170Asn	LP/P	rs1180650883	Argininosuccinic aciduria (ARGINSA) [MIM:207900]
ASL	P04424	VAR_072200	p.Leu180Arg	LP/P	rs1057141162	Argininosuccinic aciduria (ARGINSA) [MIM:207900]
ASL	P04424	VAR_072201	p.Arg182Gln	LP/P	rs751590073	Argininosuccinic aciduria (ARGINSA) [MIM:207900]
ASL	P04424	VAR_072202	p.Arg191Trp	LP/P	rs143508372	Argininosuccinic aciduria (ARGINSA) [MIM:207900]
ASL	P04424	VAR_072203	p.Arg193Trp	LP/P	rs1428029508	Argininosuccinic aciduria (ARGINSA) [MIM:207900]
ASL	P04424	VAR_072204	p.Ala205Val	LP/P	rs796051925	Argininosuccinic aciduria (ARGINSA) [MIM:207900]
ASL	P04424	VAR_072205	p.Arg213Gln	LP/P	rs1449589636	Argininosuccinic aciduria (ARGINSA) [MIM:207900]
ASL	P04424	VAR_072206	p.Leu227Pro	LP/P	-	Argininosuccinic aciduria (ARGINSA) [MIM:207900]
ASL	P04424	VAR_072207	p.Ser229Arg	LP/P	-	Argininosuccinic aciduria (ARGINSA) [MIM:207900]
ASL	P04424	VAR_072208	p.Ser229Thr	LP/P	rs1554327272	Argininosuccinic aciduria (ARGINSA) [MIM:207900]
ASL	P04424	VAR_072209	p.Asp231Glu	LP/P	-	Argininosuccinic aciduria (ARGINSA) [MIM:207900]
ASL	P04424	VAR_072210	p.Asp237Asn	LP/P	rs552951774	Argininosuccinic aciduria (ARGINSA) [MIM:207900]
ASL	P04424	VAR_072211	p.Met256Thr	LP/P	rs149057077	Argininosuccinic aciduria (ARGINSA) [MIM:207900]
ASL	P04424	VAR_072212	p.Leu262Pro	LP/P	rs1554327586	Argininosuccinic aciduria (ARGINSA) [MIM:207900]
ASL	P04424	VAR_072213	p.Leu295Pro	LP/P	rs1369337876	Argininosuccinic aciduria (ARGINSA) [MIM:207900]
ASL	P04424	VAR_072214	p.Gly301Arg	LP/P	rs1161412459	Argininosuccinic aciduria (ARGINSA) [MIM:207900]
ASL	P04424	VAR_072215	p.Arg306Trp	LP/P	rs868834862	Argininosuccinic aciduria (ARGINSA) [MIM:207900]
ASL	P04424	VAR_072216	p.Asp324Ala	LP/P	-	Argininosuccinic aciduria (ARGINSA) [MIM:207900]
ASL	P04424	VAR_072217	p.Gln326Leu	LP/P	-	Argininosuccinic aciduria (ARGINSA) [MIM:207900]
ASL	P04424	VAR_072218	p.Leu343Phe	LP/P	-	Argininosuccinic aciduria (ARGINSA) [MIM:207900]
ASL	P04424	VAR_072219	p.Leu343Pro	LP/P	-	Argininosuccinic aciduria (ARGINSA) [MIM:207900]
ASL	P04424	VAR_072220	p.Met368Val	LP/P	rs1554328202	Argininosuccinic aciduria (ARGINSA) [MIM:207900]
ASL	P04424	VAR_072221	p.Lys380Glu	LP/P	-	Argininosuccinic aciduria (ARGINSA) [MIM:207900]
ASL	P04424	VAR_072222	p.Arg385His	LP/P	rs746120802	Argininosuccinic aciduria (ARGINSA) [MIM:207900]
ASL	P04424	VAR_072223	p.Arg385Leu	LP/P	-	Argininosuccinic aciduria (ARGINSA) [MIM:207900]
ASL	P04424	VAR_072224	p.His388Gln	LP/P	-	Argininosuccinic aciduria (ARGINSA) [MIM:207900]
ASL	P04424	VAR_072225	p.Ser433Arg	LP/P	rs796051928	Argininosuccinic aciduria (ARGINSA) [MIM:207900]

ASL	P04424	VAR_072226	p.Val434Leu	LP/P	rs773071023	Argininosuccinic aciduria (ARGINSA) [MIM:207900]
ASL	P04424	VAR_072227	p.Ser447Asn	LP/P	rs373519615	Argininosuccinic aciduria (ARGINSA) [MIM:207900]
ASL	P04424	VAR_072228	p.Arg456Gln	LP/P	rs767271619	Argininosuccinic aciduria (ARGINSA) [MIM:207900]
ASL	P04424	VAR_075551	p.Glu73Lys	LP/P	-	Argininosuccinic aciduria (ARGINSA) [MIM:207900]
ASL	P04424	VAR_075552	p.Arg297Gln	LP/P	rs750431938	Argininosuccinic aciduria (ARGINSA) [MIM:207900]
ASMT	P46597	VAR_045991	p.Asn17Lys	LB/B	rs17149149	-
ASMT	P46597	VAR_069111	p.Asn13His	LB/B	rs121918819	-
ASMT	P46597	VAR_069112	p.Glu61Gln	LB/B	rs121918823	-
ASMT	P46597	VAR_069114	p.Lys81Glu	LB/B	rs117343570	-
ASMT	P46597	VAR_069115	p.Arg115Trp	LB/B	rs201053197	-
ASMT	P46597	VAR_069116	p.Gly151Ser	LB/B	rs192710293	-
ASMT	P46597	VAR_069117	p.Val166Ile	LB/B	rs373339042	-
ASMT	P46597	VAR_069118	p.Val171Met	LB/B	rs121918820	-
ASMT	P46597	VAR_069119	p.Val179Gly	LB/B	rs1215166326	-
ASMT	P46597	VAR_069120	p.Asp210Gly	LB/B	rs121918824	-
ASMT	P46597	VAR_069121	p.Ile211Met	LB/B	rs201316181	-
ASMT	P46597	VAR_069122	p.Thr217Met	LB/B	rs148036160	-
ASMT	P46597	VAR_069123	p.Lys219Arg	LB/B	rs121918825	-
ASMT	P46597	VAR_069125	p.Pro243Leu	LB/B	rs121918826	-
ASMT	P46597	VAR_069126	p.Ile269Met	LB/B	rs146121655	-
ASMT	P46597	VAR_069127	p.Cys273Ser	LB/B	rs121918827	-
ASMT	P46597	VAR_069128	p.Gly278Ala	LB/B	rs1188440875	-
ASMT	P46597	VAR_069129	p.Glu288Asp	LB/B	rs121918821	-
ASMT	P46597	VAR_069130	p.Arg291Gln	LB/B	rs121918828	-
ASMT	P46597	VAR_069131	p.Leu298Phe	US	rs121918822	-
ASMT	P46597	VAR_069132	p.Val305Met	US	-	-
ASMTL	O95671	VAR_054802	p.Val458Met	LB/B	rs4503285	-
ASMTL	O95671	VAR_054803	p.Arg541Lys	LB/B	rs1127297	-
ASNS	P08243	VAR_023443	p.Val210Glu	LB/B	rs1049674	-
ASNS	P08243	VAR_070896	p.Ala6Glu	LP/P	rs398122975	Asparagine synthetase deficiency (ASNSD) [MIM:615574]
ASNS	P08243	VAR_070897	p.Phe362Val	LP/P	rs398122973	Asparagine synthetase deficiency (ASNSD) [MIM:615574]
ASNS	P08243	VAR_070898	p.Arg550Cys	LP/P	rs398122974	Asparagine synthetase deficiency (ASNSD) [MIM:615574]
ASNSD1	Q9NWL6	VAR_039876	p.Gly190Arg	LB/B	rs1437880	-
ASNSD1	Q9NWL6	VAR_039877	p.Met434Thr	LB/B	rs35137531	-
ASPA	P45381	VAR_004995	p.Ile143Thr	LP/P	rs777936704	Canavan disease (CAND) [MIM:271900]
ASPA	P45381	VAR_004996	p.Cys152Arg	LP/P	rs104894548	Canavan disease (CAND) [MIM:271900]
ASPA	P45381	VAR_004998	p.Gly274Arg	LP/P	rs761064915	Canavan disease (CAND) [MIM:271900]
ASPA	P45381	VAR_004999	p.Glu285Ala	LP/P	rs28940279	Canavan disease (CAND) [MIM:271900]
ASPA	P45381	VAR_005000	p.Phe295Ser	LP/P	-	Canavan disease (CAND) [MIM:271900]
ASPA	P45381	VAR_005001	p.Ala305Glu	LP/P	rs28940574	Canavan disease (CAND) [MIM:271900]
ASPA	P45381	VAR_016778	p.His21Pro	LP/P	-	Canavan disease (CAND) [MIM:271900]
ASPA	P45381	VAR_016779	p.Ala57Thr	LP/P	-	Canavan disease (CAND) [MIM:271900]
ASPA	P45381	VAR_016780	p.Arg168His	LP/P	rs770706390	Canavan disease (CAND) [MIM:271900]
ASPA	P45381	VAR_016781	p.Pro181Thr	LP/P	rs786204572	Canavan disease (CAND) [MIM:271900]
ASPA	P45381	VAR_016782	p.Glu24Gly	LP/P	rs104894551	Canavan disease (CAND) [MIM:271900]
ASPA	P45381	VAR_016783	p.Asp68Ala	LP/P	-	Canavan disease (CAND) [MIM:271900]

ASPA P45381 VAR_016784 p.Asp114Tyr LP/P rs1446467099 Canavan disease (CAND) [MIM:271900]
ASPA P45381 VAR_016785 p.Cys152Trp LP/P - Canavan disease (CAND) [MIM:271900]
ASPA P45381 VAR_016786 p.Tyr231Cys LP/P rs104894550 Canavan disease (CAND) [MIM:271900]
ASPA P45381 VAR_016787 p.His244Arg LP/P rs1057516995 Canavan disease (CAND) [MIM:271900]
ASPA P45381 VAR_016788 p.Asp249Val LP/P rs104894552 Canavan disease (CAND) [MIM:271900]
ASPA P45381 VAR_039079 p.Ile16Thr LP/P rs769653717 Canavan disease (CAND) [MIM:271900]
ASPA P45381 VAR_039080 p.Gly27Arg LP/P rs766328537 Canavan disease (CAND) [MIM:271900]
ASPA P45381 VAR_039081 p.Asp114Glu LP/P - Canavan disease (CAND) [MIM:271900]
ASPA P45381 VAR_039082 p.Gly123Glu LP/P rs1057521115 Canavan disease (CAND) [MIM:271900]
ASPA P45381 VAR_039083 p.Cys152Tyr LP/P - Canavan disease (CAND) [MIM:271900]
ASPA P45381 VAR_039084 p.Arg168Cys LP/P rs937670540 Canavan disease (CAND) [MIM:271900]
ASPA P45381 VAR_039085 p.Pro183His LP/P rs1555539857 Canavan disease (CAND) [MIM:271900]
ASPA P45381 VAR_039086 p.Val186Phe LP/P - Canavan disease (CAND) [MIM:271900]
ASPA P45381 VAR_039087 p.Met195Arg LP/P - Canavan disease (CAND) [MIM:271900]
ASPA P45381 VAR_039088 p.Pro280Leu LP/P rs1555541310 Canavan disease (CAND) [MIM:271900]
ASPA P45381 VAR_039089 p.Pro280Ser LP/P rs750505963 Canavan disease (CAND) [MIM:271900]
ASPA P45381 VAR_039090 p.Ala287Thr LP/P rs774323189 Canavan disease (CAND) [MIM:271900]
ASPA P45381 VAR_039091 p.Cys310Gly LB/B rs376854191 -
ASPA P45381 VAR_078086 p.Glu24Lys LP/P - Canavan disease (CAND) [MIM:271900]
ASPA P45381 VAR_078087 p.Leu30Pro LP/P rs1555538144 Canavan disease (CAND) [MIM:271900]
ASPA P45381 VAR_078088 p.Ala57Val LP/P rs1555538148 Canavan disease (CAND) [MIM:271900]
ASPA P45381 VAR_078089 p.Arg63Thr LP/P rs1555538151 Canavan disease (CAND) [MIM:271900]
ASPA P45381 VAR_078090 p.Leu69Arg LP/P rs776777887 Canavan disease (CAND) [MIM:271900]
ASPA P45381 VAR_078091 p.Gly101Val LP/P - Canavan disease (CAND) [MIM:271900]
ASPA P45381 VAR_078092 p.Glu129Lys LP/P rs773049803 Canavan disease (CAND) [MIM:271900]
ASPA P45381 VAR_078093 p.Ile170Thr LP/P rs144321760 Canavan disease (CAND) [MIM:271900]
ASPA P45381 VAR_078094 p.Ile177Thr LP/P - Canavan disease (CAND) [MIM:271900]
ASPA P45381 VAR_078095 p.Gly180Val LP/P rs1014551540 Canavan disease (CAND) [MIM:271900]
ASPA P45381 VAR_078096 p.Asp204His LP/P - Canavan disease (CAND) [MIM:271900]
ASPA P45381 VAR_078097 p.Gln248Arg LP/P - Canavan disease (CAND) [MIM:271900]
ASPA P45381 VAR_078098 p.Ala286Asp LP/P rs1414684396 Canavan disease (CAND) [MIM:271900]
ASPDH A6ND91 VAR_062676 p.Gln266Arg LB/B rs12977172 -
ASPG Q86U10 VAR_059131 p.Cys95Arg LB/B rs1770984 -
ASPG Q86U10 VAR_059132 p.Leu96Val LB/B rs1744284 -
ASPG Q86U10 VAR_059133 p.Ser344Arg LB/B rs8012505 -
ASPH Q12797 VAR_053781 p.Arg354Met LB/B rs6995412 -
ASPH Q12797 VAR_071821 p.Arg735Trp LP/P rs374385878 Facial dysmorphism, lens dislocation, anterior segment abnormalities, and spontaneous filtering blebs (FDLAB) [MIM:601552]
ASPHD2 Q6ICH7 VAR_060123 p.Asn235Ser LB/B rs34902186 -
ASPM Q8IZT6 VAR_019084 p.Ser2562Gly LB/B rs41310927 -
ASPM Q8IZT6 VAR_019085 p.Leu2647Ile LB/B rs3762271 -
ASPM Q8IZT6 VAR_019086 p.Leu3132Arg LB/B rs36004306 -
ASPM Q8IZT6 VAR_024369 p.Arg430Gly LB/B rs6428388 -
ASPM Q8IZT6 VAR_046758 p.Thr869Ser LB/B rs7551108 -
ASPM Q8IZT6 VAR_046759 p.Ser1090Phe LB/B rs16841081 -
ASPM Q8IZT6 VAR_046760 p.Tyr2494His LB/B rs964201 -
ASPM Q8IZT6 VAR_046761 p.Gln2620His LB/B rs12138336 -
ASPM Q8IZT6 VAR_046762 p.His3258Arg LB/B rs7528827 -
ASPM Q8IZT6 VAR_047263 p.Ile313Val LB/B rs12025066 -

ASPM Q8IZT6 VAR_071930 p.Asn2526Asp LB/B rs587783267 -
ASPM Q8IZT6 VAR_071931 p.Gln3180Pro LB/B rs193251130 -
ASPRV1 Q53RT3 VAR_051508 p.Thr49Ala LB/B rs3796097 -
ASPRV1 Q53RT3 VAR_084554 p.Lys199Glu LP/P - Ichthyosis, lamellar, autosomal dominant (ADLI) [MIM:146750]
ASPRV1 Q53RT3 VAR_084555 p.Arg311Pro LP/P - Ichthyosis, lamellar, autosomal dominant (ADLI) [MIM:146750]
ASPRV1 Q53RT3 VAR_084556 p.Pro314Thr LP/P - Ichthyosis, lamellar, autosomal dominant (ADLI) [MIM:146750]
ASPSCR1 Q9BZE9 VAR_027503 p.Leu252Gln LB/B rs8074498 -
ASPSCR1 Q9BZE9 VAR_027504 p.Asp487Glu LB/B rs13087 -
ASPSCR1 Q9BZE9 VAR_034745 p.Val318Met LB/B rs34085048 -
ASRGL1 Q7L266 VAR_081118 p.Gly178Arg US - -
ASS1 P00966 VAR_000681 p.Gly14Ser LP/P rs121908636 Citrullinemia 1 (CTLN1) [MIM:215700]
ASS1 P00966 VAR_000682 p.Ser18Leu LP/P rs121908643 Citrullinemia 1 (CTLN1) [MIM:215700]
ASS1 P00966 VAR_000683 p.Arg86Cys LP/P rs121908644 Citrullinemia 1 (CTLN1) [MIM:215700]
ASS1 P00966 VAR_000684 p.Ala118Thr LP/P rs775305020 Citrullinemia 1 (CTLN1) [MIM:215700]
ASS1 P00966 VAR_000685 p.Arg157His LP/P rs121908637 Citrullinemia 1 (CTLN1) [MIM:215700]
ASS1 P00966 VAR_000686 p.Ser180Asn LP/P rs121908638 Citrullinemia 1 (CTLN1) [MIM:215700]
ASS1 P00966 VAR_000687 p.Ala192Val LP/P - Citrullinemia 1 (CTLN1) [MIM:215700]
ASS1 P00966 VAR_000688 p.Arg272Cys LP/P rs762387914 Citrullinemia 1 (CTLN1) [MIM:215700]
ASS1 P00966 VAR_000689 p.Gly280Arg LP/P - Citrullinemia 1 (CTLN1) [MIM:215700]
ASS1 P00966 VAR_000690 p.Arg304Trp LP/P rs121908642 Citrullinemia 1 (CTLN1) [MIM:215700]
ASS1 P00966 VAR_000691 p.Gly324Ser LP/P rs121908639 Citrullinemia 1 (CTLN1) [MIM:215700]
ASS1 P00966 VAR_000692 p.Arg363Leu LP/P - Citrullinemia 1 (CTLN1) [MIM:215700]
ASS1 P00966 VAR_000693 p.Arg363Trp LP/P rs121908640 Citrullinemia 1 (CTLN1) [MIM:215700]
ASS1 P00966 VAR_000694 p.Gly390Arg LP/P rs121908641 Citrullinemia 1 (CTLN1) [MIM:215700]
ASS1 P00966 VAR_015891 p.Cys19Arg LP/P - Citrullinemia 1 (CTLN1) [MIM:215700]
ASS1 P00966 VAR_015892 p.Arg86His LP/P rs575001023 Citrullinemia 1 (CTLN1) [MIM:215700]
ASS1 P00966 VAR_015893 p.Arg95Ser LP/P - Citrullinemia 1 (CTLN1) [MIM:215700]
ASS1 P00966 VAR_015894 p.Pro96Ser LP/P - Citrullinemia 1 (CTLN1) [MIM:215700]
ASS1 P00966 VAR_015895 p.Gly117Ser LP/P rs770944877 Citrullinemia 1 (CTLN1) [MIM:215700]
ASS1 P00966 VAR_015896 p.Gly117Asp LP/P - Citrullinemia 1 (CTLN1) [MIM:215700]
ASS1 P00966 VAR_015897 p.Arg157Cys LP/P rs770585183 Citrullinemia 1 (CTLN1) [MIM:215700]
ASS1 P00966 VAR_015898 p.Trp179Arg LP/P rs121908646 Citrullinemia 1 (CTLN1) [MIM:215700]
ASS1 P00966 VAR_015899 p.Glu191Lys LP/P rs777828000 Citrullinemia 1 (CTLN1) [MIM:215700]
ASS1 P00966 VAR_015900 p.Arg265His LP/P rs398123131 Citrullinemia 1 (CTLN1) [MIM:215700]
ASS1 P00966 VAR_015901 p.Val269Met LP/P rs370595480 Citrullinemia 1 (CTLN1) [MIM:215700]
ASS1 P00966 VAR_015902 p.Glu283Lys LP/P rs765338121 Citrullinemia 1 (CTLN1) [MIM:215700]
ASS1 P00966 VAR_015903 p.Lys310Arg LP/P rs199751308 Citrullinemia 1 (CTLN1) [MIM:215700]
ASS1 P00966 VAR_015904 p.Gly362Val LP/P rs121908647 Citrullinemia 1 (CTLN1) [MIM:215700]
ASS1 P00966 VAR_016007 p.Glu270Gln LP/P rs775163147 Citrullinemia 1 (CTLN1) [MIM:215700]
ASS1 P00966 VAR_016008 p.Arg279Gln LP/P rs371265106 Citrullinemia 1 (CTLN1) [MIM:215700]
ASS1 P00966 VAR_016009 p.Lys310Gln LP/P rs121908648 Citrullinemia 1 (CTLN1) [MIM:215700]
ASS1 P00966 VAR_016010 p.Arg363Gly LP/P - Citrullinemia 1 (CTLN1) [MIM:215700]
ASS1 P00966 VAR_016011 p.Arg363Gln LP/P rs771937610 Citrullinemia 1 (CTLN1) [MIM:215700]
ASS1 P00966 VAR_016012 p.Thr389Ile LP/P rs1474017319 Citrullinemia 1 (CTLN1) [MIM:215700]
ASS1 P00966 VAR_016013 p.Val69Ala LP/P rs771594651 Citrullinemia 1 (CTLN1) [MIM:215700]
ASS1 P00966 VAR_016014 p.Arg108Leu LP/P rs35269064 Citrullinemia 1 (CTLN1) [MIM:215700]

ASS1	P00966	VAR_016015	p.Thr119Ile	LP/P	-	Citrullinemia 1 (CTLN1) [MIM:215700]
ASS1	P00966	VAR_058337	p.Gln40Leu	LP/P	-	Citrullinemia 1 (CTLN1) [MIM:215700]
ASS1	P00966	VAR_058338	p.Ser79Pro	LP/P	-	Citrullinemia 1 (CTLN1) [MIM:215700]
ASS1	P00966	VAR_058339	p.Pro96His	LP/P	-	Citrullinemia 1 (CTLN1) [MIM:215700]
ASS1	P00966	VAR_058340	p.Asp124Asn	LP/P	rs936192871	Citrullinemia 1 (CTLN1) [MIM:215700]
ASS1	P00966	VAR_058341	p.Arg127Gln	LP/P	rs201623252	Citrullinemia 1 (CTLN1) [MIM:215700]
ASS1	P00966	VAR_058342	p.Arg127Trp	LP/P	rs771794639	Citrullinemia 1 (CTLN1) [MIM:215700]
ASS1	P00966	VAR_058343	p.Leu160Pro	LP/P	rs969835605	Citrullinemia 1 (CTLN1) [MIM:215700]
ASS1	P00966	VAR_058344	p.Tyr190Asp	LP/P	-	Citrullinemia 1 (CTLN1) [MIM:215700]
ASS1	P00966	VAR_058345	p.Glu191Gln	LP/P	-	Citrullinemia 1 (CTLN1) [MIM:215700]
ASS1	P00966	VAR_058346	p.Ala202Glu	LP/P	rs376371866	Citrullinemia 1 (CTLN1) [MIM:215700]
ASS1	P00966	VAR_058347	p.Leu206Pro	LP/P	-	Citrullinemia 1 (CTLN1) [MIM:215700]
ASS1	P00966	VAR_058348	p.Val263Met	LP/P	rs192838388	Citrullinemia 1 (CTLN1) [MIM:215700]
ASS1	P00966	VAR_058349	p.Arg265Cys	LP/P	rs148918985	Citrullinemia 1 (CTLN1) [MIM:215700]
ASS1	P00966	VAR_058350	p.Lys277Thr	LP/P	-	Citrullinemia 1 (CTLN1) [MIM:215700]
ASS1	P00966	VAR_058351	p.Thr284Ile	LP/P	rs886039853	Citrullinemia 1 (CTLN1) [MIM:215700]
ASS1	P00966	VAR_058352	p.Tyr291Ser	LP/P	-	Citrullinemia 1 (CTLN1) [MIM:215700]
ASS1	P00966	VAR_058353	p.Asp296Gly	LP/P	-	Citrullinemia 1 (CTLN1) [MIM:215700]
ASS1	P00966	VAR_058354	p.Met302Val	LP/P	-	Citrullinemia 1 (CTLN1) [MIM:215700]
ASS1	P00966	VAR_058355	p.Arg307Cys	LP/P	rs183276875	Citrullinemia 1 (CTLN1) [MIM:215700]
ASS1	P00966	VAR_058356	p.Gly324Val	LP/P	-	Citrullinemia 1 (CTLN1) [MIM:215700]
ASS1	P00966	VAR_058357	p.Ser341Phe	LP/P	-	Citrullinemia 1 (CTLN1) [MIM:215700]
ASS1	P00966	VAR_058358	p.Val345Gly	LP/P	-	Citrullinemia 1 (CTLN1) [MIM:215700]
ASS1	P00966	VAR_058359	p.Gly347Arg	LP/P	-	Citrullinemia 1 (CTLN1) [MIM:215700]
ASS1	P00966	VAR_058360	p.Tyr359Asp	LP/P	-	Citrullinemia 1 (CTLN1) [MIM:215700]
ASS1	P00966	VAR_072792	p.Val141Gly	LP/P	rs1184442048	Citrullinemia 1 (CTLN1) [MIM:215700]
ASS1	P00966	VAR_078388	p.Val64Ile	US	rs556297791	Citrullinemia 1 (CTLN1) [MIM:215700]
ASS1	P00966	VAR_078389	p.Thr91Pro	LP/P	rs769018733	Citrullinemia 1 (CTLN1) [MIM:215700]
ASS1	P00966	VAR_078390	p.Pro96Leu	LP/P	-	Citrullinemia 1 (CTLN1) [MIM:215700]
ASS1	P00966	VAR_078392	p.Arg100Cys	LP/P	rs370695114	Citrullinemia 1 (CTLN1) [MIM:215700]
ASS1	P00966	VAR_078393	p.Arg100His	LP/P	rs138279074	Citrullinemia 1 (CTLN1) [MIM:215700]
ASS1	P00966	VAR_078394	p.Ala111Asp	LP/P	-	Citrullinemia 1 (CTLN1) [MIM:215700]
ASS1	P00966	VAR_078395	p.Gly117Cys	LP/P	-	Citrullinemia 1 (CTLN1) [MIM:215700]
ASS1	P00966	VAR_078396	p.Arg127Leu	LB/B	rs201623252	-
ASS1	P00966	VAR_078398	p.Arg157Ser	LP/P	-	Citrullinemia 1 (CTLN1) [MIM:215700]
ASS1	P00966	VAR_078400	p.Ala164Pro	LP/P	-	Citrullinemia 1 (CTLN1) [MIM:215700]
ASS1	P00966	VAR_078401	p.Ser180Ile	LP/P	rs121908638	Citrullinemia 1 (CTLN1) [MIM:215700]
ASS1	P00966	VAR_078402	p.Asn184Lys	LP/P	rs368192467	Citrullinemia 1 (CTLN1) [MIM:215700]
ASS1	P00966	VAR_078403	p.Gly230Arg	LP/P	-	Citrullinemia 1 (CTLN1) [MIM:215700]
ASS1	P00966	VAR_078404	p.Asn237Ile	LP/P	-	Citrullinemia 1 (CTLN1) [MIM:215700]
ASS1	P00966	VAR_078405	p.Ala258Pro	LP/P	-	Citrullinemia 1 (CTLN1) [MIM:215700]
ASS1	P00966	VAR_078406	p.Ala258Val	LP/P	rs753078725	Citrullinemia 1 (CTLN1) [MIM:215700]
ASS1	P00966	VAR_078407	p.Arg272His	LP/P	rs768215008	Citrullinemia 1 (CTLN1) [MIM:215700]
ASS1	P00966	VAR_078408	p.Arg272Leu	LP/P	rs768215008	Citrullinemia 1 (CTLN1) [MIM:215700]
ASS1	P00966	VAR_078411	p.Leu290Pro	LP/P	-	Citrullinemia 1 (CTLN1) [MIM:215700]
ASS1	P00966	VAR_078412	p.Ala299Asp	LP/P	rs768394647	Citrullinemia 1 (CTLN1) [MIM:215700]
ASS1	P00966	VAR_078413	p.Val306Gly	LP/P	-	Citrullinemia 1 (CTLN1) [MIM:215700]
ASS1	P00966	VAR_078415	p.Val321Met	LP/P	rs727503813	Citrullinemia 1 (CTLN1) [MIM:215700]
ASS1	P00966	VAR_078416	p.Arg335His	LP/P	rs555388438	Citrullinemia 1 (CTLN1) [MIM:215700]

ASS1 P00966 VAR_078417 p.Cys337Arg LP/P - Citrullinemia 1 (CTLN1) [MIM:215700]
ASS1 P00966 VAR_078419 p.Gly356Val LP/P - Citrullinemia 1 (CTLN1) [MIM:215700]
ASS1 P00966 VAR_078422 p.Thr389Pro LP/P - Citrullinemia 1 (CTLN1) [MIM:215700]
ASTL Q6HA08 VAR_033491 p.Gln222Arg LB/B rs749458 -
ASTL Q6HA08 VAR_057063 p.Lys277Gln LB/B rs1657502 -
ASTL Q6HA08 VAR_061734 p.Arg204His LB/B rs41320144 -
ASTN1 O14525 VAR_036764 p.Gly1270Arg LB/B rs12118933 -
ASTN1 O14525 VAR_055713 p.Arg1278Gly LB/B rs12118933 -
ASTN1 O14525 VAR_069030 p.His932Arg LB/B rs2228956 -
ASTN1 O14525 VAR_069031 p.His942Gln LB/B rs2281180 -
ASTN2 O75129 VAR_036765 p.Val70Ile LB/B rs16933591 -
ASTN2 O75129 VAR_036766 p.Arg865His LB/B rs3818503 -
ASTN2 O75129 VAR_036767 p.Val1149Ile LB/B rs16933591 -
ASTN2 O75129 VAR_036768 p.Val1293Leu US - A breast cancer sample
ASTN2 O75129 VAR_064699 p.Ala229Val US - -
ASTN2 O75129 VAR_084650 p.Asp298His US - -
ASXL1 Q8IXJ9 VAR_028157 p.Pro815Leu LB/B rs6058694 -
ASXL1 Q8IXJ9 VAR_028158 p.Leu1325Phe LB/B rs6057581 -
ASXL1 Q8IXJ9 VAR_051602 p.Val751Ile LB/B rs6058693 -
ASXL1 Q8IXJ9 VAR_051603 p.Leu983Arg LB/B rs34359205 -
ASXL2 Q76L83 VAR_037773 p.Leu731Pro LB/B rs13385963 -
ASXL2 Q76L83 VAR_037774 p.Ala796Val LB/B rs17854251 -
ASXL2 Q76L83 VAR_037775 p.Thr1210Pro LB/B rs12991178 -
ASXL2 Q76L83 VAR_037776 p.Thr1242Pro LB/B rs12990978 -
ASXL3 Q9C0F0 VAR_039267 p.Asn954Ser LB/B rs2282632 -
ASXL3 Q9C0F0 VAR_039268 p.Met1415Arg LB/B rs16964887 -
ASXL3 Q9C0F0 VAR_039269 p.Val1652Met LB/B rs17746949 -
ASXL3 Q9C0F0 VAR_039270 p.Met1708Val LB/B rs7232237 -
ASZ1 Q8WWH4 VAR_024175 p.Lys216Thr LB/B rs1029396 -
ATAD1 Q8NBU5 VAR_035903 p.Val107Ile US - A colorectal cancer sample
ATAD1 Q8NBU5 VAR_080830 p.Gln54His US rs1554884979 Hyperekplexia 4 (HKPX4) [MIM:618011]
ATAD2 Q6PL18 VAR_047625 p.Ile1280Thr LB/B rs3758122 -
ATAD2B Q9ULI0 VAR_055467 p.Ser118Pro LB/B rs10210982 -
ATAD3A Q9NVI7 VAR_023526 p.Gly15Asp LB/B rs2274435 -
ATAD3A Q9NVI7 VAR_055468 p.Ser101Asn LB/B rs1619896 -
ATAD3A Q9NVI7 VAR_083867 p.Leu77Arg LP/P rs1570319915 Pontocerebellar hypoplasia, hypotonia, and respiratory insufficiency syndrome, neonatal lethal (PHRINL) [MIM:618810]
ATAD3A Q9NVI7 VAR_083869 p.Leu454Arg LP/P rs1570345942 Pontocerebellar hypoplasia, hypotonia, and respiratory insufficiency syndrome, neonatal lethal (PHRINL) [MIM:618810]
ATAD3B Q5T9A4 VAR_048120 p.Val7Ile LB/B rs1240504 -
ATAD5 Q96QE3 VAR_038572 p.Thr35Ser LB/B rs9910051 -
ATAD5 Q96QE3 VAR_038573 p.Pro87Ser LB/B rs3816780 -
ATAD5 Q96QE3 VAR_038574 p.Glu135Gly LB/B rs11080134 -
ATAD5 Q96QE3 VAR_038575 p.Arg249Lys LB/B rs17826219 -
ATAD5 Q96QE3 VAR_038576 p.Asn699His LB/B rs3764421 -
ATAD5 Q96QE3 VAR_038577 p.Tyr1419His LB/B rs11657270 -
ATAD5 Q96QE3 VAR_079496 p.Asp215Asn LB/B rs367699401 -
ATCAY Q86WG3 VAR_017164 p.Ser301Arg LP/P - Cerebellar ataxia, cayman type (ATCAY) [MIM:601238]
ATF1 P18846 VAR_024382 p.Pro191Ala LB/B rs2230674 -

ATF2 P15336 VAR_035999 p.Asp352His US - A breast cancer sample
ATF3 P18847 VAR_048442 p.Thr38Met LB/B rs11571541 -
ATF3 P18847 VAR_081532 p.Asn168Thr US - -
ATF4 P18848 VAR_014768 p.Glu322Asp LB/B rs1803324 -
ATF4 P18848 VAR_028253 p.Gln22Pro LB/B rs4894 -
ATF4 P18848 VAR_029259 p.Pro258Ala LB/B rs1803323 -
ATF5 Q9Y2D1 VAR_022786 p.Pro121Leu LB/B rs283526 -
ATF6 P18850 VAR_022455 p.Met67Leu LB/B rs1058405 -
ATF6 P18850 VAR_022456 p.Met67Val LB/B rs1058405 -
ATF6 P18850 VAR_022457 p.Ala145Pro LB/B rs2070150 -
ATF6 P18850 VAR_022458 p.Pro157Ser LB/B rs1135983 -
ATF6 P18850 VAR_075681 p.Arg324Cys LP/P rs761357250 Achromatopsia 7 (ACHM7) [MIM:616517]
ATF6 P18850 VAR_075682 p.Tyr567Asn LP/P rs796065053 Achromatopsia 7 (ACHM7) [MIM:616517]
ATF7IP Q6VMQ6 VAR_031283 p.Glu278Lys LB/B rs2231908 -
ATF7IP Q6VMQ6 VAR_031284 p.Asn348Ile LB/B rs2231909 -
ATF7IP Q6VMQ6 VAR_031285 p.Lys530Arg LB/B rs3213764 -
ATF7IP2 Q5U623 VAR_031286 p.Thr537Ile LB/B rs9932051 -
ATF7IP2 Q5U623 VAR_031287 p.Ala543Thr LB/B rs9931441 -
ATF7IP2 Q5U623 VAR_053872 p.Ser527Leu LB/B rs34834862 -
ATG10 Q9H0Y0 VAR_021562 p.Thr212Met LB/B rs1864183 -
ATG10 Q9H0Y0 VAR_021563 p.Pro220His LB/B rs1864182 -
ATG10 Q9H0Y0 VAR_024370 p.Ser62Pro LB/B rs3734114 -
ATG14 Q6ZNE5 VAR_049514 p.Asn131Lys LB/B rs17675076 -
ATG14 Q6ZNE5 VAR_061240 p.Val59Ile LB/B rs57295720 -
ATG16L1 Q676U5 VAR_021834 p.Thr300Ala LP/P rs2241880 Inflammatory bowel disease 10 (IBD10) [MIM:611081]
ATG16L1 Q676U5 VAR_053386 p.Glu307Lys LB/B rs1866878 -
ATG16L2 Q8NAA4 VAR_043605 p.Arg220Trp LB/B rs11235604 -
ATG2A Q2TAZ0 VAR_038158 p.Val175Ile LB/B rs12293826 -
ATG2A Q2TAZ0 VAR_038159 p.Ala627Val LB/B rs2285347 -
ATG2A Q2TAZ0 VAR_038160 p.Gly948Arg LB/B rs11827140 -
ATG2A Q2TAZ0 VAR_061027 p.Arg394Cys LB/B rs35115827 -
ATG2A Q2TAZ0 VAR_061028 p.Ala404Val LB/B rs60711419 -
ATG2A Q2TAZ0 VAR_061029 p.Pro656Arg LB/B rs656195 -
ATG2B Q96BY7 VAR_021523 p.Gln1383Glu LB/B rs3759601 -
ATG2B Q96BY7 VAR_023096 p.Ile1567Thr LB/B rs2289622 -
ATG2B Q96BY7 VAR_045956 p.Asn1124Asp LB/B rs9323945 -
ATG4B Q9Y4P1 VAR_021486 p.Leu354Gln LB/B rs7601000 -
ATG5 Q9H1Y0 VAR_036243 p.Lys58Met US - A colorectal cancer sample
ATG5 Q9H1Y0 VAR_079274 p.Glu122Asp LP/P rs1131692265 Spinocerebellar ataxia, autosomal recessive, 25 (SCAR25) [MIM:617584]
ATG7 O95352 VAR_053014 p.Val471Ala LB/B rs36117895 -
ATG9A Q7Z3C6 VAR_021835 p.Ser592Gly LB/B rs2276635 -
ATG9A Q7Z3C6 VAR_055534 p.Gln659His LB/B rs2276634 -
ATG9B Q674R7 VAR_061030 p.Pro166Leu LB/B rs61078191 -
ATIC P31939 VAR_019306 p.Thr116Ser LB/B rs2372536 -
ATIC P31939 VAR_019307 p.Lys426Arg LP/P rs121434478 AICAR transformylase/IMP cyclohydrolase deficiency (AICAR) [MIM:608688]

ATL1	Q8WXF7	VAR_017146	p.Arg217Gln	LP/P	rs119476049	Spastic paraplegia 3, autosomal dominant (SPG3) [MIM:182600]
ATL1	Q8WXF7	VAR_017147	p.Arg239Cys	LP/P	rs119476046	Spastic paraplegia 3, autosomal dominant (SPG3) [MIM:182600]
ATL1	Q8WXF7	VAR_017148	p.His258Arg	LP/P	rs119476048	Spastic paraplegia 3, autosomal dominant (SPG3) [MIM:182600]
ATL1	Q8WXF7	VAR_017149	p.Ser259Tyr	LP/P	rs119476047	Spastic paraplegia 3, autosomal dominant (SPG3) [MIM:182600]
ATL1	Q8WXF7	VAR_019446	p.Ala161Pro	LP/P	-	Spastic paraplegia 3, autosomal dominant (SPG3) [MIM:182600]
ATL1	Q8WXF7	VAR_019447	p.His247Pro	LP/P	-	Spastic paraplegia 3, autosomal dominant (SPG3) [MIM:182600]
ATL1	Q8WXF7	VAR_058963	p.Asp43Glu	LB/B	rs17850684	-
ATL1	Q8WXF7	VAR_058964	p.Phe193Cys	LB/B	rs17850683	-
ATL1	Q8WXF7	VAR_065508	p.Glu66Gln	LP/P	rs200314808	Neuropathy, hereditary sensory, 1D (HSN1D) [MIM:613708]
ATL1	Q8WXF7	VAR_065509	p.Leu157Trp	LP/P	rs119476051	Spastic paraplegia 3, autosomal dominant (SPG3) [MIM:182600]
ATL1	Q8WXF7	VAR_065510	p.Asn355Lys	LP/P	rs1555365597	Neuropathy, hereditary sensory, 1D (HSN1D) [MIM:613708]
ATL1	Q8WXF7	VAR_065511	p.Met408Val	LP/P	rs28939094	Spastic paraplegia 3, autosomal dominant (SPG3) [MIM:182600]
ATL1	Q8WXF7	VAR_065512	p.Arg415Trp	LP/P	rs119476050	Spastic paraplegia 3, autosomal dominant (SPG3) [MIM:182600]
ATL1	Q8WXF7	VAR_067655	p.Gln154Glu	LP/P	-	Spastic paraplegia 3, autosomal dominant (SPG3) [MIM:182600]
ATL1	Q8WXF7	VAR_067656	p.Tyr196Cys	US	rs1555364246	-
ATL1	Q8WXF7	VAR_067657	p.Val253Ile	LP/P	rs864622520	Spastic paraplegia 3, autosomal dominant (SPG3) [MIM:182600]
ATL1	Q8WXF7	VAR_067658	p.Phe413Val	LP/P	-	Spastic paraplegia 3, autosomal dominant (SPG3) [MIM:182600]
ATL1	Q8WXF7	VAR_067659	p.Asn440Thr	LP/P	-	Spastic paraplegia 3, autosomal dominant (SPG3) [MIM:182600]
ATL1	Q8WXF7	VAR_067660	p.Arg495Trp	LP/P	rs864622269	Spastic paraplegia 3, autosomal dominant (SPG3) [MIM:182600]
ATL1	Q8WXF7	VAR_071708	p.Arg415Gln	LP/P	rs397514712	Spastic paraplegia 3, autosomal dominant (SPG3) [MIM:182600]
ATL1	Q8WXF7	VAR_071709	p.Arg416Cys	LP/P	rs387906941	Spastic paraplegia 3, autosomal dominant (SPG3) [MIM:182600]
ATL1	Q8WXF7	VAR_071874	p.Arg118Gln	LP/P	rs606231265	Spastic paraplegia 3, autosomal dominant (SPG3) [MIM:182600]
ATL2	Q8NHH9	VAR_032265	p.Trp18Arg	LB/B	rs3731847	-
ATL2	Q8NHH9	VAR_032266	p.Asn272Ser	LB/B	rs34873284	-
ATL2	Q8NHH9	VAR_032267	p.Asp420His	LB/B	rs7582826	-
ATL3	Q6DD88	VAR_070973	p.Tyr192Cys	LP/P	rs587777108	Neuropathy, hereditary sensory, 1F (HSN1F) [MIM:615632]
ATM	Q13315	VAR_010798	p.Ser49Cys	LB/B	rs1800054	-
ATM	Q13315	VAR_010799	p.Asp126Glu	LB/B	rs2234997	-
ATM	Q13315	VAR_010800	p.Val182Leu	LB/B	rs3218707	-

ATM	Q13315	VAR_010801	p.Lys224Glu	US	rs145053092	Ataxia telangiectasia (AT) [MIM:208900]
ATM	Q13315	VAR_010802	p.Pro292Leu	LP/P	rs747727055	Ataxia telangiectasia (AT) [MIM:208900]
ATM	Q13315	VAR_010803	p.Ile323Val	LP/P	rs587781511	Ataxia telangiectasia (AT) [MIM:208900]
ATM	Q13315	VAR_010804	p.Tyr332Cys	US	-	B-cell chronic lymphocytic leukemia
ATM	Q13315	VAR_010805	p.Ala350Thr	US	rs371713984	B-cell chronic lymphocytic leukemia
ATM	Q13315	VAR_010806	p.Ile352Thr	US	rs369203092	B-cell chronic lymphocytic leukemia
ATM	Q13315	VAR_010807	p.Gly514Asp	LB/B	rs2235000	-
ATM	Q13315	VAR_010808	p.Phe570Ser	LP/P	rs777301065	Ataxia telangiectasia (AT) [MIM:208900]
ATM	Q13315	VAR_010810	p.Ser707Pro	LB/B	rs4986761	-
ATM	Q13315	VAR_010812	p.Asn768Asp	LP/P	-	Ataxia telangiectasia (AT) [MIM:208900]
ATM	Q13315	VAR_010813	p.Arg785Cys	LP/P	rs587778065	Ataxia telangiectasia (AT) [MIM:208900]
ATM	Q13315	VAR_010814	p.Phe858Leu	LB/B	rs1800056	-
ATM	Q13315	VAR_010815	p.Leu950Arg	LP/P	rs786203054	Ataxia telangiectasia (AT) [MIM:208900]
ATM	Q13315	VAR_010816	p.Leu1001Gln	LP/P	-	Ataxia telangiectasia (AT) [MIM:208900]
ATM	Q13315	VAR_010817	p.Met1040Val	US	rs3092857	-
ATM	Q13315	VAR_010818	p.Pro1054Arg	LB/B	rs1800057	-
ATM	Q13315	VAR_010819	p.His1082Leu	LP/P	-	Ataxia telangiectasia (AT) [MIM:208900]
ATM	Q13315	VAR_010820	p.Glu1091Asp	LP/P	-	Ataxia telangiectasia (AT) [MIM:208900]
ATM	Q13315	VAR_010821	p.Ile1407Thr	US	rs1234250980	T-prolymphocytic leukemia
ATM	Q13315	VAR_010822	p.Leu1420Phe	LB/B	rs1800058	-
ATM	Q13315	VAR_010823	p.Leu1420Pro	LP/P	-	Ataxia telangiectasia (AT) [MIM:208900]
ATM	Q13315	VAR_010824	p.Lys1454Asn	LB/B	rs148993589	-
ATM	Q13315	VAR_010825	p.Phe1463Ser	US	-	-
ATM	Q13315	VAR_010826	p.Leu1465Pro	LP/P	rs730881391	Ataxia telangiectasia (AT) [MIM:208900]
ATM	Q13315	VAR_010827	p.Pro1566Arg	LP/P	-	Ataxia telangiectasia (AT) [MIM:208900]
ATM	Q13315	VAR_010828	p.Val1570Ala	LB/B	rs140856217	-
ATM	Q13315	VAR_010829	p.Asp1682His	US	rs121434217	T-prolymphocytic leukemia
ATM	Q13315	VAR_010830	p.Ser1691Arg	LB/B	rs1800059	-
ATM	Q13315	VAR_010831	p.Thr1743Ile	LP/P	rs587779844	Ataxia telangiectasia (AT) [MIM:208900]
ATM	Q13315	VAR_010833	p.Asp1853Asn	LB/B	rs1801516	-
ATM	Q13315	VAR_010834	p.Asp1853Val	LB/B	rs1801673	-
ATM	Q13315	VAR_010835	p.Leu1910His	US	-	T-prolymphocytic leukemia
ATM	Q13315	VAR_010836	p.Val1913Gly	LP/P	rs1060501688	Ataxia telangiectasia (AT) [MIM:208900]
ATM	Q13315	VAR_010837	p.Thr1953Arg	US	-	B-cell chronic lymphocytic leukemia
ATM	Q13315	VAR_010838	p.Asp2016Gly	LP/P	rs587781302	Ataxia telangiectasia (AT) [MIM:208900]
ATM	Q13315	VAR_010839	p.Gly2063Glu	LP/P	rs866290641	Ataxia telangiectasia (AT) [MIM:208900]
ATM	Q13315	VAR_010840	p.Ala2067Asp	LP/P	rs397514577	Ataxia telangiectasia (AT) [MIM:208900]
ATM	Q13315	VAR_010841	p.Val2079Ile	LB/B	rs1800060	-
ATM	Q13315	VAR_010842	p.Glu2139Gly	US	-	T-prolymphocytic leukemia
ATM	Q13315	VAR_010843	p.Glu2164Lys	US	rs1317619286	T-prolymphocytic leukemia
ATM	Q13315	VAR_010844	p.Ser2218Cys	LP/P	-	Ataxia telangiectasia (AT) [MIM:208900]
ATM	Q13315	VAR_010846	p.Arg2227Cys	LP/P	rs564652222	Ataxia telangiectasia (AT) [MIM:208900]
ATM	Q13315	VAR_010848	p.Ala2274Thr	US	rs567060474	B-cell chronic lymphocytic leukemia
ATM	Q13315	VAR_010849	p.Gly2287Ala	US	rs1800061	-
ATM	Q13315	VAR_010850	p.Thr2396Ser	US	rs370559102	T-prolymphocytic leukemia
ATM	Q13315	VAR_010852	p.Ala2420Pro	US	-	B-cell chronic lymphocytic leukemia
ATM	Q13315	VAR_010853	p.Glu2423Gly	US	rs121434221	Mantle cell lymphoma
ATM	Q13315	VAR_010854	p.Val2424Gly	LP/P	rs28904921	Ataxia telangiectasia (AT) [MIM:208900]
ATM	Q13315	VAR_010856	p.Thr2438Ile	LB/B	rs147604227	-

ATM	Q13315	VAR_010857	p.Gln2442Pro	US	-	T-prolymphocytic leukemia
ATM	Q13315	VAR_010858	p.Tyr2470Asp	LP/P	rs876659365	Ataxia telangiectasia (AT) [MIM:208900]
ATM	Q13315	VAR_010859	p.Arg2486Gly	US	-	T-prolymphocytic leukemia
ATM	Q13315	VAR_010860	p.Trp2491Arg	LP/P	-	Ataxia telangiectasia (AT) [MIM:208900]
ATM	Q13315	VAR_010862	p.His2554Asp	LP/P	-	Ataxia telangiectasia (AT) [MIM:208900]
ATM	Q13315	VAR_010863	p.Asp2625Gln	LP/P	-	Ataxia telangiectasia (AT) [MIM:208900]
ATM	Q13315	VAR_010865	p.Leu2656Pro	LP/P	rs121434218	Ataxia telangiectasia (AT) [MIM:208900]
ATM	Q13315	VAR_010868	p.Glu2668Gly	US	-	Ataxia telangiectasia (AT) [MIM:208900]
ATM	Q13315	VAR_010869	p.Gly2695Ala	US	-	B-cell chronic lymphocytic leukemia
ATM	Q13315	VAR_010869	p.Gly2695Ala	US	-	T-prolymphocytic leukemia
ATM	Q13315	VAR_010870	p.Ile2702Arg	LP/P	rs876659735	Ataxia telangiectasia (AT) [MIM:208900]
ATM	Q13315	VAR_010871	p.Leu2722Arg	US	-	T-prolymphocytic leukemia
ATM	Q13315	VAR_010872	p.Asp2725Gly	US	rs1555128314	-
ATM	Q13315	VAR_010873	p.Asp2725Val	US	-	T-prolymphocytic leukemia
ATM	Q13315	VAR_010874	p.Ala2726Val	LP/P	-	Ataxia telangiectasia (AT) [MIM:208900]
ATM	Q13315	VAR_010875	p.Phe2732Leu	US	rs876659619	T-prolymphocytic leukemia
ATM	Q13315	VAR_010876	p.Gly2765Ser	LB/B	rs748634900	-
ATM	Q13315	VAR_010878	p.Cys2824Tyr	LP/P	rs876660927	Ataxia telangiectasia (AT) [MIM:208900]
ATM	Q13315	VAR_010879	p.Phe2827Cys	LP/P	rs121434216	Ataxia telangiectasia (AT) [MIM:208900]
ATM	Q13315	VAR_010880	p.Pro2829Leu	LP/P	rs938431501	Ataxia telangiectasia (AT) [MIM:208900]
ATM	Q13315	VAR_010881	p.Arg2832Cys	LP/P	rs587779872	Ataxia telangiectasia (AT) [MIM:208900]
ATM	Q13315	VAR_010882	p.Arg2849Pro	LP/P	rs587782202	Ataxia telangiectasia (AT) [MIM:208900]
ATM	Q13315	VAR_010883	p.Ser2855Arg	LP/P	rs780905851	Ataxia telangiectasia (AT) [MIM:208900]
ATM	Q13315	VAR_010886	p.Gly2867Arg	LP/P	-	Ataxia telangiectasia (AT) [MIM:208900]
ATM	Q13315	VAR_010888	p.Leu2890Val	US	rs587779874	T-prolymphocytic leukemia
ATM	Q13315	VAR_010889	p.Glu2904Gly	LP/P	rs786202826	Ataxia telangiectasia (AT) [MIM:208900]
ATM	Q13315	VAR_010890	p.Arg2909Gly	LP/P	-	Ataxia telangiectasia (AT) [MIM:208900]
ATM	Q13315	VAR_010892	p.Ala3006Pro	US	rs876658767	-
ATM	Q13315	VAR_010893	p.Arg3008Cys	LP/P	rs587782292	Ataxia telangiectasia (AT) [MIM:208900]
ATM	Q13315	VAR_010894	p.Arg3008His	US	rs587781894	B-cell chronic lymphocytic leukemia
ATM	Q13315	VAR_010895	p.Lys3018Asn	US	-	B-cell chronic lymphocytic leukemia
ATM	Q13315	VAR_041545	p.Arg23Gln	US	rs587779858	A colorectal adenocarcinoma sample
ATM	Q13315	VAR_041546	p.Asp140His	LB/B	rs55633650	-
ATM	Q13315	VAR_041547	p.Arg250Gln	LB/B	rs56123940	-
ATM	Q13315	VAR_041548	p.Ser333Phe	LB/B	rs28904919	-
ATM	Q13315	VAR_041549	p.Arg337Cys	US	rs138398778	A colorectal adenocarcinoma sample
ATM	Q13315	VAR_041550	p.Arg337His	US	rs202160435	A colorectal adenocarcinoma sample
ATM	Q13315	VAR_041551	p.Val410Ala	LB/B	rs56128736	-
ATM	Q13315	VAR_041552	p.Asn504Ser	LB/B	rs56365018	-
ATM	Q13315	VAR_041553	p.Cys540Tyr	US	-	A colorectal adenocarcinoma sample
ATM	Q13315	VAR_041554	p.Leu546Val	LB/B	rs2227924	-
ATM	Q13315	VAR_041555	p.Phe582Leu	LB/B	rs2235006	-
ATM	Q13315	VAR_041556	p.Glu848Gln	US	rs879254046	A lung adenocarcinoma sample
ATM	Q13315	VAR_041557	p.Pro872Ser	LB/B	rs3218673	-
ATM	Q13315	VAR_041558	p.Arg924Trp	US	rs55723361	-
ATM	Q13315	VAR_041559	p.Thr935Ala	LB/B	rs35813135	-
ATM	Q13315	VAR_041560	p.Ser1179Phe	US	-	A gastric adenocarcinoma sample
ATM	Q13315	VAR_041561	p.Met1321Ile	LB/B	rs35184530	-
ATM	Q13315	VAR_041562	p.His1380Tyr	LB/B	rs3092856	-

ATM	Q13315	VAR_041563	p.Pro1382Ser	LB/B	rs55859590	-
ATM	Q13315	VAR_041564	p.Ile1469Met	US	rs775047783	A renal papillary cancer sample
ATM	Q13315	VAR_041565	p.Tyr1475Cys	LB/B	rs34640941	-
ATM	Q13315	VAR_041566	p.Asn1650Ser	LB/B	rs55870064	-
ATM	Q13315	VAR_041567	p.Asn1739Thr	US	-	A colorectal adenocarcinoma sample
ATM	Q13315	VAR_041568	p.Met1916Ile	US	-	A breast pleomorphic lobular carcinoma sample
ATM	Q13315	VAR_041569	p.Ala1945Thr	US	-	A colorectal adenocarcinoma sample
ATM	Q13315	VAR_041570	p.Tyr1961Cys	US	rs56399311	-
ATM	Q13315	VAR_041571	p.Ser1983Asn	LB/B	rs659243	-
ATM	Q13315	VAR_041572	p.Glu1991Asp	US	rs587782274	A renal clear cell carcinoma sample
ATM	Q13315	VAR_041573	p.Leu2307Phe	US	rs56009889	-
ATM	Q13315	VAR_041574	p.Leu2332Pro	LB/B	rs4988111	-
ATM	Q13315	VAR_041575	p.Ile2356Phe	US	rs876658517	A renal clear cell carcinoma sample
ATM	Q13315	VAR_041576	p.Ser2408Leu	US	rs730881315	A colorectal adenocarcinoma sample
ATM	Q13315	VAR_041577	p.Arg2443Gln	US	rs587782310	A colorectal adenocarcinoma sample
ATM	Q13315	VAR_041578	p.Cys2464Arg	US	rs55801750	-
ATM	Q13315	VAR_041579	p.Leu2492Arg	LB/B	rs56399857	-
ATM	Q13315	VAR_041580	p.Thr2666Ala	US	rs745775382	A lung adenocarcinoma sample
ATM	Q13315	VAR_041581	p.Arg2719His	US	rs55982963	-
ATM	Q13315	VAR_041582	p.Pro2842Arg	US	rs879254065	A lung adenocarcinoma sample
ATM	Q13315	VAR_041583	p.Asp2870Asn	LB/B	rs55798854	-
ATM	Q13315	VAR_056678	p.Arg45Trp	US	rs3218684	-
ATM	Q13315	VAR_056679	p.Thr761Ser	LB/B	rs2235011	-
ATM	Q13315	VAR_056680	p.Ser788Arg	LB/B	rs641252	-
ATM	Q13315	VAR_056681	p.Asp814Glu	LB/B	rs3218695	-
ATM	Q13315	VAR_056682	p.Thr935Met	LB/B	rs3218708	-
ATM	Q13315	VAR_056683	p.Leu942Phe	LB/B	rs3218688	-
ATM	Q13315	VAR_056684	p.Glu1313Gln	LB/B	rs3092841	-
ATM	Q13315	VAR_056685	p.Ala1427Thr	LB/B	rs2229021	-
ATM	Q13315	VAR_056686	p.Leu1541Phe	LB/B	rs3092849	-
ATM	Q13315	VAR_056687	p.Val1729Leu	LB/B	rs3092907	-
ATM	Q13315	VAR_056688	p.Arg2034Gln	LB/B	rs3218670	-
ATM	Q13315	VAR_056689	p.Thr2335Lys	LB/B	rs3092831	-
ATM	Q13315	VAR_056690	p.Glu2570Gly	LB/B	rs28904920	-
ATM	Q13315	VAR_056691	p.Thr2640Ile	LB/B	rs4988125	-
ATM	Q13315	VAR_056692	p.Gly2709Ser	LB/B	rs3218680	-
ATM	Q13315	VAR_077237	p.Leu1046Pro	LP/P	rs568461905	Ataxia telangiectasia (AT) [MIM:208900]
ATM	Q13315	VAR_077238	p.Gly2023Arg	LP/P	rs11212587	Ataxia telangiectasia (AT) [MIM:208900]
ATM	Q13315	VAR_077239	p.Leu2068Ser	LP/P	rs1555114558	Ataxia telangiectasia (AT) [MIM:208900]
ATM	Q13315	VAR_077240	p.Tyr2080Asp	LP/P	rs1064795467	Ataxia telangiectasia (AT) [MIM:208900]
ATM	Q13315	VAR_077241	p.Tyr2627His	LP/P	-	Ataxia telangiectasia (AT) [MIM:208900]
ATM	Q13315	VAR_077242	p.Phe2834Leu	LP/P	-	Ataxia telangiectasia (AT) [MIM:208900]
ATM	Q13315	VAR_077243	p.Asn3003Asp	LP/P	rs1137889	Ataxia telangiectasia (AT) [MIM:208900]
ATM	Q13315	VAR_080300	p.Leu1322Ile	LB/B	rs144535256	-
ATM	Q13315	VAR_083373	p.Ile68Val	US	-	-
ATM	Q13315	VAR_083374	p.Val341Ile	US	-	-
ATM	Q13315	VAR_083375	p.Pro597Leu	US	-	-
ATM	Q13315	VAR_083376	p.Asp661Ala	US	-	-
ATM	Q13315	VAR_083377	p.Glu699Gly	US	-	-

ATM Q13315 VAR_083378 p.Ser759Gly US - -
ATM Q13315 VAR_083379 p.Asn813Ser US - -
ATM Q13315 VAR_083380 p.Ala869Gly US - -
ATM Q13315 VAR_083381 p.Phe897Ile US - -
ATM Q13315 VAR_083382 p.Leu1255Val US - -
ATM Q13315 VAR_083383 p.His1474Asp US - -
ATM Q13315 VAR_083384 p.Leu1488Val US - -
ATM Q13315 VAR_083385 p.Arg2105Thr US - -
ATM Q13315 VAR_083386 p.Ala2524Pro US - -
ATM Q13315 VAR_083387 p.Met2531Thr US - -
ATM Q13315 VAR_083388 p.Lys2810Gln US - -
ATM Q13315 VAR_083389 p.Arg2832His US - -
ATM Q13315 VAR_083390 p.Pro2974Leu US - -
ATM Q13315 VAR_083391 p.Gly3029Asp US - -
ATM Q13315 VAR_083392 p.Val3056Leu US - -
ATMIN O43313 VAR_050681 p.Ser240Pro LB/B rs2278022 -
ATMIN O43313 VAR_050682 p.Lys305Glu LB/B rs2278023 -
ATN1 P54259 VAR_030937 p.Met339Ile LB/B rs1058045 -
ATN1 P54259 VAR_083058 p.His1054Asn LP/P - Congenital hypotonia, epilepsy, developmental delay, and digital anomalies (CHEDDA) [MIM:618494]
ATN1 P54259 VAR_083059 p.His1058Tyr LP/P - Congenital hypotonia, epilepsy, developmental delay, and digital anomalies (CHEDDA) [MIM:618494]
ATN1 P54259 VAR_083062 p.His1060Tyr LP/P - Congenital hypotonia, epilepsy, developmental delay, and digital anomalies (CHEDDA) [MIM:618494]
ATN1 P54259 VAR_083063 p.His1062Asp LP/P - Congenital hypotonia, epilepsy, developmental delay, and digital anomalies (CHEDDA) [MIM:618494]
ATN1 P54259 VAR_083064 p.His1062Arg LP/P - Congenital hypotonia, epilepsy, developmental delay, and digital anomalies (CHEDDA) [MIM:618494]
ATN1 P54259 VAR_083065 p.Leu1063Arg LP/P - Congenital hypotonia, epilepsy, developmental delay, and digital anomalies (CHEDDA) [MIM:618494]
ATOH1 Q92858 VAR_049539 p.His237Gln LB/B rs35182771 -
ATOH7 Q8N100 VAR_072398 p.Asn46His LP/P rs587777666 Persistent hyperplastic primary vitreous, autosomal recessive (PHPVAR) [MIM:221900]
ATOH7 Q8N100 VAR_072399 p.Ala47Thr US - -
ATOH7 Q8N100 VAR_072400 p.Glu49Val LP/P rs587777664 Persistent hyperplastic primary vitreous, autosomal recessive (PHPVAR) [MIM:221900]
ATOH7 Q8N100 VAR_072401 p.Arg65Gly LB/B rs111699024 -
ATOH8 Q96SQ7 VAR_039582 p.Leu150Pro LB/B rs17851881 -
ATP10A O60312 VAR_022004 p.Thr532Met LB/B rs2066703 -
ATP10A O60312 VAR_022005 p.Ala784Thr LB/B rs2066704 -
ATP10A O60312 VAR_022006 p.Trp1172Cys LB/B rs2076742 -
ATP10A O60312 VAR_022007 p.Ala1179Thr LB/B rs2076744 -
ATP10A O60312 VAR_022008 p.Ile1188Val LB/B rs2076745 -
ATP10A O60312 VAR_022009 p.Arg1298Ser LB/B rs3816800 -
ATP10A O60312 VAR_048380 p.Ser353Tyr LB/B rs17116056 -
ATP10A O60312 VAR_048381 p.Glu834Lys LB/B rs17555920 -
ATP10A O60312 VAR_048382 p.Val1198Met LB/B rs2076746 -
ATP10A O60312 VAR_048383 p.Ala1397Val LB/B rs9324127 -
ATP10A O60312 VAR_061038 p.Arg504His LB/B rs56724944 -

ATP10A	O60312	VAR_078700	p.Arg208Trp	US	rs763246380	-
ATP10B	O94823	VAR_048384	p.Cys217Arg	LB/B	rs958912	-
ATP10B	O94823	VAR_084141	p.Asn105Ser	US	rs184217288	-
ATP10B	O94823	VAR_084143	p.Thr161Asn	US	rs73306687	-
ATP10B	O94823	VAR_084144	p.Gly393Trp	LB/B	rs149397148	-
ATP10B	O94823	VAR_084145	p.Ala558Val	US	rs770267845	-
ATP10B	O94823	VAR_084146	p.Gly648Arg	US	rs188580726	-
ATP10B	O94823	VAR_084147	p.Gly671Arg	US	rs61734666	-
ATP10B	O94823	VAR_084148	p.Val748Leu	US	rs192890224	-
ATP10B	O94823	VAR_084149	p.Asn865Lys	US	rs61734665	-
ATP10B	O94823	VAR_084150	p.Glu993Ala	US	rs761562566	-
ATP10B	O94823	VAR_084151	p.Ile1038Thr	US	-	-
ATP10B	O94823	VAR_084152	p.Ile1222Thr	US	rs144497343	-
ATP10B	O94823	VAR_084153	p.Leu1421Phe	US	rs61734664	-
ATP10B	O94823	VAR_084190	p.Val219Met	US	rs139187738	-
ATP10B	O94823	VAR_084191	p.Ile540Thr	US	rs56340994	-
ATP10D	Q9P241	VAR_020187	p.Val1240Ile	LB/B	rs1058793	-
ATP10D	Q9P241	VAR_024371	p.Ser1389Thr	LB/B	rs4145944	-
ATP10D	Q9P241	VAR_048385	p.Thr43Ile	LB/B	rs33995001	-
ATP10D	Q9P241	VAR_048386	p.Cys171Arg	LB/B	rs7683838	-
ATP10D	Q9P241	VAR_048387	p.Thr320Ile	LB/B	rs35596623	-
ATP10D	Q9P241	VAR_048388	p.Ala337Thr	LB/B	rs35012290	-
ATP10D	Q9P241	VAR_048389	p.Asn511Ser	LB/B	rs10003238	-
ATP10D	Q9P241	VAR_048390	p.Phe522Leu	LB/B	rs6843325	-
ATP10D	Q9P241	VAR_048391	p.Pro716Thr	LB/B	rs34208443	-
ATP10D	Q9P241	VAR_048392	p.Asn720Ser	LB/B	rs34169638	-
ATP10D	Q9P241	VAR_048393	p.Ser959Asn	LB/B	rs17462252	-
ATP10D	Q9P241	VAR_048394	p.Arg1183Lys	LB/B	rs16851681	-
ATP10D	Q9P241	VAR_048395	p.Ala1392Gly	LB/B	rs35375547	-
ATP11A	P98196	VAR_048379	p.Val1091Ile	LB/B	rs11616795	-
ATP11A	P98196	VAR_059139	p.Met317Val	LB/B	rs368865	-
ATP11C	Q8NB49	VAR_021827	p.Cys114Trp	LB/B	rs2491014	-
ATP11C	Q8NB49	VAR_036501	p.Thr157Ile	US	-	A colorectal cancer sample
ATP11C	Q8NB49	VAR_036502	p.Gln931Pro	US	-	A colorectal cancer sample
ATP11C	Q8NB49	VAR_055546	p.Tyr522Cys	LB/B	rs17281983	-
ATP11C	Q8NB49	VAR_061036	p.Val972Met	LB/B	rs55724992	-
ATP11C	Q8NB49	VAR_081016	p.Thr418Asn	LP/P	rs1556323334	Hemolytic anemia, congenital, X-linked (HAXL) [MIM:301015]
ATP12A	P54707	VAR_020186	p.Pro863Leu	LB/B	rs2289909	-
ATP13A1	Q9HD20	VAR_084651	p.Glu349Lys	US	-	-
ATP13A2	Q9NQ11	VAR_058451	p.Thr12Met	LP/P	rs151117874	Kufor-Rakeb syndrome (KRS) [MIM:606693]
ATP13A2	Q9NQ11	VAR_058452	p.Gly49Ser	LB/B	rs56379718	-
ATP13A2	Q9NQ11	VAR_058453	p.Arg294Gln	LB/B	rs56367069	-
ATP13A2	Q9NQ11	VAR_058454	p.Pro389Leu	LB/B	rs56275621	-
ATP13A2	Q9NQ11	VAR_058455	p.Gly504Arg	LP/P	rs121918227	Kufor-Rakeb syndrome (KRS) [MIM:606693]
ATP13A2	Q9NQ11	VAR_058456	p.Gly533Arg	LP/P	-	Kufor-Rakeb syndrome (KRS) [MIM:606693]
ATP13A2	Q9NQ11	VAR_058457	p.Val578Gly	LB/B	rs56186751	-
ATP13A2	Q9NQ11	VAR_058458	p.Ala746Thr	LP/P	rs147277743	Kufor-Rakeb syndrome (KRS) [MIM:606693]
ATP13A2	Q9NQ11	VAR_058459	p.Arg762Trp	LB/B	rs55635527	-

ATP13A2 Q9NQ11 VAR_058460 p.Val776Ile LB/B rs56170027 -
ATP13A2 Q9NQ11 VAR_058461 p.Ile946Phe LB/B rs55708915 -
ATP13A2 Q9NQ11 VAR_066019 p.Phe182Leu LP/P - Kufor-Rakeb syndrome (KRS) [MIM:606693]
ATP13A2 Q9NQ11 VAR_066020 p.Gly877Arg LP/P rs144701072 Kufor-Rakeb syndrome (KRS) [MIM:606693]
ATP13A2 Q9NQ11 VAR_066021 p.Leu1059Arg LP/P rs137853967 Kufor-Rakeb syndrome (KRS) [MIM:606693]
ATP13A2 Q9NQ11 VAR_070194 p.Met854Arg LP/P rs587777053 Kufor-Rakeb syndrome (KRS) [MIM:606693]
ATP13A2 Q9NQ11 VAR_078055 p.Thr517Ile LP/P rs1057519291 Spastic paraplegia 78, autosomal recessive (SPG78) [MIM:617225]
ATP13A2 Q9NQ11 VAR_078056 p.Gly522Val US - Kufor-Rakeb syndrome (KRS) [MIM:606693]
ATP13A2 Q9NQ11 VAR_083537 p.Ile441Phe US rs772446950 Kufor-Rakeb syndrome (KRS) [MIM:606693]
ATP13A2 Q9NQ11 VAR_083538 p.Ala1069Thr US rs774238872 Kufor-Rakeb syndrome (KRS) [MIM:606693]
ATP13A4 Q4VNC1 VAR_038849 p.Ile181Met LB/B rs6788448 -
ATP13A4 Q4VNC1 VAR_038850 p.Val353Ala LB/B - -
ATP13A4 Q4VNC1 VAR_038851 p.Glu646Asp LB/B rs35424709 -
ATP13A5 Q4VNC0 VAR_043614 p.Glu133Gln LB/B rs6797429 -
ATP13A5 Q4VNC0 VAR_043615 p.Gly739Ser LB/B rs2280268 -
ATP13A5 Q4VNC0 VAR_043616 p.Ile1053Val LB/B rs6787746 -
ATP13A5 Q4VNC0 VAR_043617 p.Val1131Ala LB/B rs2271791 -
ATP13A5 Q4VNC0 VAR_043618 p.Lys1204Gln LB/B rs7428010 -
ATP13A5 Q4VNC0 VAR_061039 p.Ser96Tyr LB/B rs12637558 -
ATP1A1 P05023 VAR_048374 p.Ser47Ile LB/B rs12564026 -
ATP1A1 P05023 VAR_081039 p.Leu48Arg LP/P rs1553190285 Charcot-Marie-Tooth disease 2DD (CMT2DD) [MIM:618036]
ATP1A1 P05023 VAR_081040 p.Ile592Thr US rs1553192086 Charcot-Marie-Tooth disease 2DD (CMT2DD) [MIM:618036]
ATP1A1 P05023 VAR_081041 p.Ala597Thr US - Charcot-Marie-Tooth disease 2DD (CMT2DD) [MIM:618036]
ATP1A1 P05023 VAR_081042 p.Pro600Ala LP/P rs1553192091 Charcot-Marie-Tooth disease 2DD (CMT2DD) [MIM:618036]
ATP1A1 P05023 VAR_081043 p.Pro600Thr US rs1553192091 Charcot-Marie-Tooth disease 2DD (CMT2DD) [MIM:618036]
ATP1A1 P05023 VAR_081044 p.Asp601Phe US - Charcot-Marie-Tooth disease 2DD (CMT2DD) [MIM:618036]
ATP1A1 P05023 VAR_081045 p.Asp811Ala LP/P rs1553192783 Charcot-Marie-Tooth disease 2DD (CMT2DD) [MIM:618036]
ATP1A1 P05023 VAR_081937 p.Leu302Arg LP/P - Hypomagnesemia, seizures, and mental retardation 2 (HOMGSMR2) [MIM:618314]
ATP1A1 P05023 VAR_081938 p.Gly303Arg LP/P rs1557785503 Hypomagnesemia, seizures, and mental retardation 2 (HOMGSMR2) [MIM:618314]
ATP1A1 P05023 VAR_081939 p.Met859Arg LP/P rs781629728 Hypomagnesemia, seizures, and mental retardation 2 (HOMGSMR2) [MIM:618314]
ATP1A2 P50993 VAR_019934 p.Thr378Asn LP/P rs28934002 Alternating hemiplegia of childhood 1 (AHC1) [MIM:104290]
ATP1A2 P50993 VAR_019935 p.Arg689Gln LP/P rs28933401 Migraine, familial hemiplegic, 2 (FHM2) [MIM:602481]
ATP1A2 P50993 VAR_019936 p.Met731Thr LP/P rs28933400 Migraine, familial hemiplegic, 2 (FHM2) [MIM:602481]
ATP1A2 P50993 VAR_019937 p.Leu764Pro LP/P rs28933398 Migraine, familial hemiplegic, 2 (FHM2) [MIM:602481]

ATP1A2 P50993 VAR_019938 p.Trp887Arg LP/P rs28933399 Migraine, familial hemiplegic, 2 (FHM2) [MIM:602481]
ATP1A2 P50993 VAR_065685 p.Gly715Arg LP/P rs1553245771 Migraine, familial hemiplegic, 2 (FHM2) [MIM:602481]
ATP1A2 P50993 VAR_069991 p.Gly874Ser LP/P - Migraine, familial hemiplegic, 2 (FHM2) [MIM:602481]
ATP1A2 P50993 VAR_069992 p.Arg1007Trp LP/P rs746795369 Migraine, familial hemiplegic, 2 (FHM2) [MIM:602481]
ATP1A2 P50993 VAR_078231 p.Gly366Ala LP/P rs1057518514 -
ATP1A2 P50993 VAR_078232 p.Arg593Trp US rs886039530 -
ATP1A3 P13637 VAR_026735 p.Ile274Thr LP/P rs80356532 Dystonia 12 (DYT12) [MIM:128235]
ATP1A3 P13637 VAR_026736 p.Glu277Lys LP/P rs80356533 Dystonia 12 (DYT12) [MIM:128235]
ATP1A3 P13637 VAR_026737 p.Thr613Met LP/P rs80356534 Dystonia 12 (DYT12) [MIM:128235]
ATP1A3 P13637 VAR_026738 p.Ile758Ser LP/P rs80356535 Dystonia 12 (DYT12) [MIM:128235]
ATP1A3 P13637 VAR_026739 p.Phe780Leu LP/P rs80356536 Dystonia 12 (DYT12) [MIM:128235]
ATP1A3 P13637 VAR_026740 p.Asp801Tyr LP/P rs80356537 Dystonia 12 (DYT12) [MIM:128235]
ATP1A3 P13637 VAR_068935 p.Ser137Phe LP/P rs542652468 Alternating hemiplegia of childhood 2 (AHC2) [MIM:614820]
ATP1A3 P13637 VAR_068936 p.Ser137Tyr LP/P rs542652468 Alternating hemiplegia of childhood 2 (AHC2) [MIM:614820]
ATP1A3 P13637 VAR_068937 p.Gln140Leu LP/P rs606231427 Alternating hemiplegia of childhood 2 (AHC2) [MIM:614820]
ATP1A3 P13637 VAR_068938 p.Asp220Asn LP/P rs1396898460 Alternating hemiplegia of childhood 2 (AHC2) [MIM:614820]
ATP1A3 P13637 VAR_068939 p.Ile274Asn LP/P rs80356532 Alternating hemiplegia of childhood 2 (AHC2) [MIM:614820]
ATP1A3 P13637 VAR_068940 p.Cys333Phe LP/P rs606231430 Alternating hemiplegia of childhood 2 (AHC2) [MIM:614820]
ATP1A3 P13637 VAR_068941 p.Gly755Ser LP/P rs557052809 Alternating hemiplegia of childhood 2 (AHC2) [MIM:614820]
ATP1A3 P13637 VAR_068942 p.Asn773Ser LP/P rs606231437 Alternating hemiplegia of childhood 2 (AHC2) [MIM:614820]
ATP1A3 P13637 VAR_068943 p.Asp801Asn LP/P rs80356537 Alternating hemiplegia of childhood 2 (AHC2) [MIM:614820]
ATP1A3 P13637 VAR_068944 p.Met806Arg LP/P rs549006436 Alternating hemiplegia of childhood 2 (AHC2) [MIM:614820]
ATP1A3 P13637 VAR_068945 p.Ile810Ser LP/P rs536681257 Alternating hemiplegia of childhood 2 (AHC2) [MIM:614820]
ATP1A3 P13637 VAR_068946 p.Ser811Pro LP/P rs387907282 Alternating hemiplegia of childhood 2 (AHC2) [MIM:614820]
ATP1A3 P13637 VAR_068947 p.Glu815Lys LP/P rs387907281 Alternating hemiplegia of childhood 2 (AHC2) [MIM:614820]
ATP1A3 P13637 VAR_068949 p.Asp923Asn LP/P rs267606670 Dystonia 12 (DYT12) [MIM:128235]
ATP1A3 P13637 VAR_068950 p.Gly947Arg LP/P rs398122887 Alternating hemiplegia of childhood 2 (AHC2) [MIM:614820]
ATP1A3 P13637 VAR_068951 p.Ala955Asp LP/P rs606231446 Alternating hemiplegia of childhood 2 (AHC2) [MIM:614820]
ATP1A3 P13637 VAR_068952 p.Asp992Tyr LP/P rs606231447 Alternating hemiplegia of childhood 2 (AHC2) [MIM:614820]

ATP1A3 P13637 VAR_070767 p.Val322Asp LP/P rs606231428 Alternating hemiplegia of childhood 2 (AHC2) [MIM:614820]
ATP1A3 P13637 VAR_070768 p.Leu371Pro LP/P rs606231433 Alternating hemiplegia of childhood 2 (AHC2) [MIM:614820]
ATP1A3 P13637 VAR_070769 p.Gly755Cys LP/P rs557052809 Alternating hemiplegia of childhood 2 (AHC2) [MIM:614820]
ATP1A3 P13637 VAR_070770 p.Ser772Arg LP/P rs534926223 Alternating hemiplegia of childhood 2 (AHC2) [MIM:614820]
ATP1A3 P13637 VAR_070771 p.Asn773Ile LP/P rs606231437 Alternating hemiplegia of childhood 2 (AHC2) [MIM:614820]
ATP1A3 P13637 VAR_070772 p.Glu818Lys LP/P rs587777771 Cerebellar ataxia, areflexia, pes cavus, optic atrophy, and sensorineural hearing loss (CAPOS) [MIM:601338]
ATP1A3 P13637 VAR_070773 p.Asp923Tyr LP/P rs267606670 Alternating hemiplegia of childhood 2 (AHC2) [MIM:614820]
ATP1A3 P13637 VAR_070774 p.Cys927Tyr LP/P rs606231444 Alternating hemiplegia of childhood 2 (AHC2) [MIM:614820]
ATP1A3 P13637 VAR_078699 p.Ala320Thr LP/P rs879255368 -
ATP1A4 Q13733 VAR_048375 p.Gly83Asp LB/B rs6427504 -
ATP1A4 Q13733 VAR_048376 p.Glu297Lys LB/B rs17368402 -
ATP1A4 Q13733 VAR_048377 p.Met541Arg LB/B rs16831482 -
ATP1A4 Q13733 VAR_048378 p.Met586Ile LB/B rs7528360 -
ATP1B2 P14415 VAR_030339 p.Thr199Ala LB/B rs2227866 -
ATP1B2 P14415 VAR_061031 p.Gln124Leu LB/B rs34745087 -
ATP1B4 Q9UN42 VAR_055535 p.Val48Ala LB/B rs2072452 -
ATP23 Q9Y6H3 VAR_054489 p.Ser48Cys LB/B rs3751325 -
ATP2A1 O14983 VAR_015588 p.Pro789Leu LP/P rs121918115 Brody myopathy (BRM) [MIM:601003]
ATP2A2 P16615 VAR_008608 p.Gly23Glu LP/P rs28929478 Darier disease (DD) [MIM:124200]
ATP2A2 P16615 VAR_008609 p.Asn39Thr LP/P - Darier disease (DD) [MIM:124200]
ATP2A2 P16615 VAR_008611 p.Leu65Ser LP/P - Darier disease (DD) [MIM:124200]
ATP2A2 P16615 VAR_008612 p.Arg131Gln LP/P rs121912738 Darier disease (DD) [MIM:124200]
ATP2A2 P16615 VAR_008613 p.Pro160Leu LP/P - Darier disease (DD) [MIM:124200]
ATP2A2 P16615 VAR_008614 p.Ser186Pro LP/P - Darier disease (DD) [MIM:124200]
ATP2A2 P16615 VAR_008615 p.Gly211Asp LP/P - Darier disease (DD) [MIM:124200]
ATP2A2 P16615 VAR_008616 p.Val223Met LP/P - Darier disease (DD) [MIM:124200]
ATP2A2 P16615 VAR_008617 p.Cys268Phe LP/P rs121912733 Darier disease (DD) [MIM:124200]
ATP2A2 P16615 VAR_008618 p.Gly310Val LP/P - Darier disease (DD) [MIM:124200]
ATP2A2 P16615 VAR_008619 p.Cys318Arg LP/P - Darier disease (DD) [MIM:124200]
ATP2A2 P16615 VAR_008620 p.Ile348Thr LP/P - Darier disease (DD) [MIM:124200]
ATP2A2 P16615 VAR_008621 p.Glu412Gly LP/P - Darier disease (DD) [MIM:124200]
ATP2A2 P16615 VAR_008622 p.Ser495Phe LP/P - Darier disease (DD) [MIM:124200]
ATP2A2 P16615 VAR_008623 p.Cys560Arg LP/P rs121912734 Darier disease (DD) [MIM:124200]
ATP2A2 P16615 VAR_008624 p.Phe675Ser LP/P - Darier disease (DD) [MIM:124200]
ATP2A2 P16615 VAR_008625 p.Lys683Glu LP/P - Darier disease (DD) [MIM:124200]
ATP2A2 P16615 VAR_008626 p.Asp702Asn LP/P - Darier disease (DD) [MIM:124200]
ATP2A2 P16615 VAR_008627 p.Ala745Asp LP/P - Darier disease (DD) [MIM:124200]
ATP2A2 P16615 VAR_008629 p.Ser765Leu LP/P - Darier disease (DD) [MIM:124200]
ATP2A2 P16615 VAR_008630 p.Asn767Ser LP/P rs121912732 Darier disease (DD) [MIM:124200]
ATP2A2 P16615 VAR_008631 p.Gly769Arg LP/P rs121912736 Darier disease (DD) [MIM:124200]
ATP2A2 P16615 VAR_008632 p.Ala803Thr LP/P - Darier disease (DD) [MIM:124200]

ATP2A2	P16615	VAR_008633	p.Ala838Pro	LP/P	-	Darier disease (DD) [MIM:124200]
ATP2A2	P16615	VAR_008634	p.Val843Phe	LP/P	-	Darier disease (DD) [MIM:124200]
ATP2A2	P16615	VAR_008635	p.Cys875Gly	LP/P	-	Darier disease (DD) [MIM:124200]
ATP2A2	P16615	VAR_008636	p.Ser920Tyr	LP/P	-	Darier disease (DD) [MIM:124200]
ATP2A2	P16615	VAR_008637	p.His943Arg	LP/P	-	Darier disease (DD) [MIM:124200]
ATP2A2	P16615	VAR_008638	p.Pro975Arg	LP/P	-	Darier disease (DD) [MIM:124200]
ATP2A2	P16615	VAR_009508	p.Thr357Lys	LP/P	-	Darier disease (DD) [MIM:124200]
ATP2A2	P16615	VAR_009509	p.Gly749Arg	LP/P	-	Darier disease (DD) [MIM:124200]
ATP2A2	P16615	VAR_017532	p.Pro602Leu	LP/P	rs121912737	Acrokeratosis verruciformis (AKV) [MIM:101900]
ATP2A2	P16615	VAR_079686	p.Asn101Ser	LP/P	-	Darier disease (DD) [MIM:124200]
ATP2A2	P16615	VAR_079688	p.Leu590Pro	LP/P	-	Darier disease (DD) [MIM:124200]
ATP2A2	P16615	VAR_079689	p.Gly625Ala	LP/P	-	Darier disease (DD) [MIM:124200]
ATP2A2	P16615	VAR_079690	p.Asp626Glu	LP/P	-	Darier disease (DD) [MIM:124200]
ATP2A2	P16615	VAR_079692	p.Ala672Pro	LP/P	-	Darier disease (DD) [MIM:124200]
ATP2A2	P16615	VAR_079693	p.Gln691Pro	LP/P	-	Darier disease (DD) [MIM:124200]
ATP2A2	P16615	VAR_079694	p.Arg750Trp	LP/P	-	Darier disease (DD) [MIM:124200]
ATP2A2	P16615	VAR_079695	p.Ser765Trp	LP/P	-	Darier disease (DD) [MIM:124200]
ATP2A2	P16615	VAR_079697	p.Leu900Pro	LP/P	-	Darier disease (DD) [MIM:124200]
ATP2A3	Q93084	VAR_036498	p.Arg674His	US	rs144535413	A breast cancer sample
ATP2A3	Q93084	VAR_048372	p.Gln869His	LB/B	rs11654827	-
ATP2B1	P20020	VAR_000698	p.Met267Arg	LB/B	-	-
ATP2B2	Q01814	VAR_084464	p.Gly293Ser	US	-	-
ATP2B2	Q01814	VAR_084465	p.Val631Met	US	-	-
ATP2B3	Q16720	VAR_027928	p.Ile198Met	LB/B	rs2269409	-
ATP2B3	Q16720	VAR_069308	p.Gly1107Asp	LP/P	rs397514619	Spinocerebellar ataxia, X-linked 1 (SCAX1) [MIM:302500]
ATP2B3	Q16720	VAR_084698	p.Arg482His	LP/P	-	Spinocerebellar ataxia, X-linked 1 (SCAX1) [MIM:302500]
ATP2C1	P98194	VAR_008803	p.Ala304Thr	LP/P	rs137853012	Hailey-Hailey disease (HHD) [MIM:169600]
ATP2C1	P98194	VAR_008804	p.Leu318Pro	LP/P	-	Hailey-Hailey disease (HHD) [MIM:169600]
ATP2C1	P98194	VAR_008805	p.Met641Arg	LP/P	-	Hailey-Hailey disease (HHD) [MIM:169600]
ATP2C1	P98194	VAR_008806	p.Gly645Arg	LP/P	-	Hailey-Hailey disease (HHD) [MIM:169600]
ATP2C1	P98194	VAR_008807	p.Thr709Met	LP/P	rs778865612	Hailey-Hailey disease (HHD) [MIM:169600]
ATP2C1	P98194	VAR_008808	p.Pro744Arg	LP/P	-	Hailey-Hailey disease (HHD) [MIM:169600]
ATP2C1	P98194	VAR_010130	p.Pro201Leu	LP/P	-	Hailey-Hailey disease (HHD) [MIM:169600]
ATP2C1	P98194	VAR_010131	p.Cys344Tyr	LP/P	-	Hailey-Hailey disease (HHD) [MIM:169600]
ATP2C1	P98194	VAR_010132	p.Thr570Ile	LP/P	-	Hailey-Hailey disease (HHD) [MIM:169600]
ATP2C1	P98194	VAR_019523	p.Cys490Phe	LP/P	rs137853014	Hailey-Hailey disease (HHD) [MIM:169600]
ATP2C1	P98194	VAR_019524	p.Leu584Pro	LP/P	rs137853015	Hailey-Hailey disease (HHD) [MIM:169600]
ATP2C1	P98194	VAR_022672	p.Gly309Cys	LP/P	-	Hailey-Hailey disease (HHD) [MIM:169600]
ATP2C1	P98194	VAR_022673	p.Leu341Pro	LP/P	-	Hailey-Hailey disease (HHD) [MIM:169600]
ATP2C1	P98194	VAR_022674	p.Cys411Arg	LP/P	-	Hailey-Hailey disease (HHD) [MIM:169600]
ATP2C1	P98194	VAR_022675	p.Ile580Val	LP/P	rs1282232888	Hailey-Hailey disease (HHD) [MIM:169600]
ATP2C1	P98194	VAR_022676	p.Asp742Tyr	LP/P	-	Hailey-Hailey disease (HHD) [MIM:169600]
ATP2C1	P98194	VAR_022677	p.Gly789Arg	LP/P	-	Hailey-Hailey disease (HHD) [MIM:169600]
ATP2C1	P98194	VAR_048373	p.Ala450Thr	LB/B	rs41434650	-
ATP2C1	P98194	VAR_079698	p.Gly220Glu	LP/P	-	Hailey-Hailey disease (HHD) [MIM:169600]
ATP2C1	P98194	VAR_079699	p.Gly309Val	LP/P	rs1393570830	Hailey-Hailey disease (HHD) [MIM:169600]
ATP2C1	P98194	VAR_079702	p.Ala731Asp	LP/P	-	Hailey-Hailey disease (HHD) [MIM:169600]

ATP2C2 O75185 VAR_047935 p.Gly411Ser LB/B rs2303853 -
ATP2C2 O75185 VAR_047936 p.Met466Leu LB/B rs247897 -
ATP2C2 O75185 VAR_047937 p.Leu907Pro LB/B rs16973859 -
ATP2C2 O75185 VAR_059137 p.Leu165Met LB/B rs247818 -
ATP2C2 O75185 VAR_070929 p.Leu604Gln LB/B rs62640926 -
ATP4A P20648 VAR_019428 p.Val265Ala LB/B rs2733743 -
ATP5F1A P25705 VAR_048369 p.Ala32Ser LB/B rs2228437 -
ATP5F1A P25705 VAR_069769 p.Arg329Cys LP/P rs587776960 Mitochondrial complex V deficiency, nuclear type 4 (MC5DN4) [MIM:615228]
ATP5F1A P25705 VAR_071982 p.Tyr321Cys LP/P rs587777788 Combined oxidative phosphorylation deficiency 22 (COXPD22) [MIM:616045]
ATP5F1B P06576 VAR_048371 p.Glu274Gln LB/B rs1042001 -
ATP5F1B P06576 VAR_074188 p.Ala130Val LB/B - -
ATP5F1D P30049 VAR_081452 p.Pro82Leu LP/P rs867410737 Mitochondrial complex V deficiency, nuclear type 5 (MC5DN5) [MIM:618120]
ATP5F1D P30049 VAR_081453 p.Val106Gly LP/P rs1555745989 Mitochondrial complex V deficiency, nuclear type 5 (MC5DN5) [MIM:618120]
ATP5F1E P56381 VAR_066211 p.Tyr12Cys LP/P rs387906929 Mitochondrial complex V deficiency, nuclear type 3 (MC5DN3) [MIM:614053]
ATP5MC2 Q06055 VAR_011920 p.Ser58Ile LB/B rs13819 -
ATP5MC2 Q06055 VAR_011921 p.Met141Lys LB/B rs1803177 -
ATP5MC3 P48201 VAR_011922 p.Gly93Glu LB/B rs1802622 -
ATP5MJ P56378 VAR_014526 p.Ile9Val LB/B rs1053419 -
ATP5PB P24539 VAR_013176 p.Thr152Asn LB/B rs1264895 -
ATP5PB P24539 VAR_033534 p.Thr152Met LB/B rs1264895 -
ATP5PF P18859 VAR_083477 p.Ile39Thr LB/B - -
ATP5PO P48047 VAR_011930 p.Lys98Arg LB/B rs4842 -
ATP6AP1 Q15904 VAR_077021 p.Leu144Pro LP/P rs878853276 Immunodeficiency 47 (IMD47) [MIM:300972]
ATP6AP1 Q15904 VAR_077022 p.Tyr313Cys LP/P rs878853278 Immunodeficiency 47 (IMD47) [MIM:300972]
ATP6AP1 Q15904 VAR_077023 p.Glu346Lys LP/P rs878853277 Immunodeficiency 47 (IMD47) [MIM:300972]
ATP6AP1 Q15904 VAR_077024 p.Met428Ile LP/P rs878853275 Immunodeficiency 47 (IMD47) [MIM:300972]
ATP6AP2 O75787 VAR_051313 p.Pro90Ala LB/B rs9014 -
ATP6AP2 O75787 VAR_051314 p.Ala290Pro LB/B rs35798522 -
ATP6AP2 O75787 VAR_082052 p.Arg71His LP/P rs1057523485 Congenital disorder of glycosylation 2R (CDG2R) [MIM:301045]
ATP6AP2 O75787 VAR_082053 p.Leu98Ser LP/P - Congenital disorder of glycosylation 2R (CDG2R) [MIM:301045]
ATP6V0A2 Q9Y487 VAR_042730 p.Arg685Gln LB/B rs7969410 -
ATP6V0A2 Q9Y487 VAR_042731 p.Ala813Val LB/B rs17883456 -
ATP6V0A4 Q9HBG4 VAR_017255 p.Pro524Leu LP/P rs121908368 Renal tubular acidosis, distal, autosomal recessive (RTADR) [MIM:602722]
ATP6V0A4 Q9HBG4 VAR_017256 p.Met580Thr LP/P rs3807153 Renal tubular acidosis, distal, autosomal recessive (RTADR) [MIM:602722]
ATP6V0A4 Q9HBG4 VAR_017257 p.Gly820Arg LP/P rs267606671 Renal tubular acidosis, distal, autosomal recessive (RTADR) [MIM:602722]
ATP6V0A4 Q9HBG4 VAR_020992 p.Val2Ala LB/B rs10258719 -
ATP6V0A4 Q9HBG4 VAR_020993 p.Gly175Asp LP/P - Renal tubular acidosis, distal, autosomal recessive (RTADR) [MIM:602722]

ATP6V0A4 Q9HBG4 VAR_020995 p.Arg449His LP/P rs1443883930 Renal tubular acidosis, distal, autosomal recessive (RTADR) [MIM:602722]
ATP6V0A4 Q9HBG4 VAR_020996 p.Arg807Gln LP/P rs28939081 Renal tubular acidosis, distal, autosomal recessive (RTADR) [MIM:602722]
ATP6V0A4 Q9HBG4 VAR_066612 p.Phe554Leu LB/B rs1026435 -
ATP6V0A4 Q9HBG4 VAR_066613 p.His604Gln LB/B rs3807154 -
ATP6V0B Q99437 VAR_035703 p.Val155Met US rs373883976 A breast cancer sample
ATP6V0D2 Q8N8Y2 VAR_032039 p.Gly272Arg LB/B rs10094744 -
ATP6V0D2 Q8N8Y2 VAR_032040 p.Glu295Lys LB/B rs4263741 -
ATP6V1A P38606 VAR_078606 p.Gly72Asp LP/P rs1060505037 Cutis laxa, autosomal recessive, 2D (ARCL2D) [MIM:617403]
ATP6V1A P38606 VAR_078607 p.Arg338Cys LP/P rs1060505036 Cutis laxa, autosomal recessive, 2D (ARCL2D) [MIM:617403]
ATP6V1A P38606 VAR_080994 p.Asp11Asn US rs746407800 -
ATP6V1A P38606 VAR_080995 p.Pro27Arg US rs1553709380 Epileptic encephalopathy, infantile or early childhood, 3 (IECEE3) [MIM:618012]
ATP6V1A P38606 VAR_080996 p.Asp100Tyr LP/P rs1553709855 Epileptic encephalopathy, infantile or early childhood, 3 (IECEE3) [MIM:618012]
ATP6V1A P38606 VAR_080997 p.Pro249Arg US - -
ATP6V1A P38606 VAR_080998 p.Asp349Asn LP/P rs1553710664 Epileptic encephalopathy, infantile or early childhood, 3 (IECEE3) [MIM:618012]
ATP6V1A P38606 VAR_080999 p.Asp371Gly US - Epileptic encephalopathy, infantile or early childhood, 3 (IECEE3) [MIM:618012]
ATP6V1B1 P15313 VAR_007866 p.Leu81Pro LP/P rs121964880 Renal tubular acidosis, distal, with progressive nerve deafness (dRTA-D) [MIM:267300]
ATP6V1B1 P15313 VAR_007867 p.Arg124Trp LP/P rs727505222 Renal tubular acidosis, distal, with progressive nerve deafness (dRTA-D) [MIM:267300]
ATP6V1B1 P15313 VAR_007868 p.Met174Arg LP/P - Renal tubular acidosis, distal, with progressive nerve deafness (dRTA-D) [MIM:267300]
ATP6V1B1 P15313 VAR_007869 p.Thr275Pro LP/P rs1161604514 Renal tubular acidosis, distal, with progressive nerve deafness (dRTA-D) [MIM:267300]
ATP6V1B1 P15313 VAR_007870 p.Gly316Glu LP/P rs1553420413 Renal tubular acidosis, distal, with progressive nerve deafness (dRTA-D) [MIM:267300]
ATP6V1B1 P15313 VAR_007871 p.Pro346Arg LP/P rs781838938 Renal tubular acidosis, distal, with progressive nerve deafness (dRTA-D) [MIM:267300]
ATP6V1B1 P15313 VAR_007872 p.Gly364Ser LP/P - Renal tubular acidosis, distal, with progressive nerve deafness (dRTA-D) [MIM:267300]
ATP6V1B1 P15313 VAR_021011 p.Thr30Ile LB/B rs17720303 -
ATP6V1B1 P15313 VAR_021012 p.Gly123Val LP/P rs1343871627 Renal tubular acidosis, distal, with progressive nerve deafness (dRTA-D) [MIM:267300]
ATP6V1B1 P15313 VAR_021013 p.Arg157Cys LP/P rs782500780 Renal tubular acidosis, distal, with progressive nerve deafness (dRTA-D) [MIM:267300]
ATP6V1B1 P15313 VAR_021014 p.Glu161Lys LB/B rs114234874 -
ATP6V1B1 P15313 VAR_021015 p.Arg465His LB/B rs142905621 -
ATP6V1B2 P21281 VAR_073962 p.Arg485Pro LP/P rs730882177 Zimmermann-Laband syndrome 2 (ZLS2) [MIM:616455]
ATP6V1C2 Q8NEY4 VAR_032041 p.Asn143Asp LB/B rs1198849 -
ATP6V1E1 P36543 VAR_036565 p.Arg50Gly US - A colorectal cancer sample

ATP6V1E1 P36543 VAR_078604 p.Leu128Pro LP/P rs1060505031 Cutis laxa, autosomal recessive, 2C (ARCL2C) [MIM:617402]
ATP6V1E1 P36543 VAR_078605 p.Arg212Trp LP/P rs1028534806 Cutis laxa, autosomal recessive, 2C (ARCL2C) [MIM:617402]
ATP6V1F Q16864 VAR_048348 p.Gly24Val LB/B rs10958 -
ATP6V1G3 Q96LB4 VAR_048343 p.Glu54Gln LB/B rs16843254 -
ATP7A Q04656 VAR_000699 p.Ala629Pro LP/P rs72554639 Menkes disease (MNK) [MIM:309400]
ATP7A Q04656 VAR_000700 p.Gly727Arg LP/P rs72554644 Menkes disease (MNK) [MIM:309400]
ATP7A Q04656 VAR_000701 p.Leu1006Pro LP/P rs72554651 Menkes disease (MNK) [MIM:309400]
ATP7A Q04656 VAR_000702 p.Gly1019Asp LP/P rs72554652 Menkes disease (MNK) [MIM:309400]
ATP7A Q04656 VAR_009999 p.Ser637Leu LP/P rs151340631 Occipital horn syndrome (OHS) [MIM:304150]
ATP7A Q04656 VAR_010000 p.Val767Leu LB/B rs2227291 -
ATP7A Q04656 VAR_010001 p.Leu873Arg LP/P rs72554646 Menkes disease (MNK) [MIM:309400]
ATP7A Q04656 VAR_010002 p.Gly876Glu LP/P - Menkes disease (MNK) [MIM:309400]
ATP7A Q04656 VAR_010003 p.Cys1000Arg LP/P - Menkes disease (MNK) [MIM:309400]
ATP7A Q04656 VAR_010004 p.Gly1300Glu LP/P - Menkes disease (MNK) [MIM:309400]
ATP7A Q04656 VAR_010005 p.Gly1302Arg LP/P rs72554657 Menkes disease (MNK) [MIM:309400]
ATP7A Q04656 VAR_010006 p.Gly1302Val LP/P - Menkes disease (MNK) [MIM:309400]
ATP7A Q04656 VAR_010007 p.Asp1305Ala LP/P - Menkes disease (MNK) [MIM:309400]
ATP7A Q04656 VAR_010008 p.Ala1362Val LP/P - Menkes disease (MNK) [MIM:309400]
ATP7A Q04656 VAR_016119 p.Ile669Thr LB/B rs2234935 -
ATP7A Q04656 VAR_016120 p.Arg703His LB/B rs2234936 -
ATP7A Q04656 VAR_016121 p.Ile1464Val LB/B rs2234938 -
ATP7A Q04656 VAR_023261 p.Leu706Arg LP/P rs72554642 Menkes disease (MNK) [MIM:309400]
ATP7A Q04656 VAR_023262 p.Arg844His LP/P rs367775730 Menkes disease (MNK) [MIM:309400]
ATP7A Q04656 VAR_023263 p.Gly853Arg LP/P - Menkes disease (MNK) [MIM:309400]
ATP7A Q04656 VAR_023264 p.Gly860Val LP/P - Menkes disease (MNK) [MIM:309400]
ATP7A Q04656 VAR_023265 p.Gly876Arg LP/P - Menkes disease (MNK) [MIM:309400]
ATP7A Q04656 VAR_023266 p.Gln924Arg LP/P - Occipital horn syndrome (OHS) [MIM:304150]
ATP7A Q04656 VAR_023267 p.Ala1007Val LP/P - Menkes disease (MNK) [MIM:309400]
ATP7A Q04656 VAR_023268 p.Gly1015Asp LP/P - Menkes disease (MNK) [MIM:309400]
ATP7A Q04656 VAR_023269 p.Asp1044Gly LP/P - Menkes disease (MNK) [MIM:309400]
ATP7A Q04656 VAR_023270 p.Leu1100Pro LP/P - Menkes disease (MNK) [MIM:309400]
ATP7A Q04656 VAR_023271 p.Gly1118Asp LP/P rs72554654 Menkes disease (MNK) [MIM:309400]
ATP7A Q04656 VAR_023272 p.Gly1255Arg LP/P rs72554655 Menkes disease (MNK) [MIM:309400]
ATP7A Q04656 VAR_023273 p.Lys1282Glu LP/P - Menkes disease (MNK) [MIM:309400]
ATP7A Q04656 VAR_023274 p.Asn1304Lys LP/P - Menkes disease (MNK) [MIM:309400]
ATP7A Q04656 VAR_023275 p.Gly1315Arg LP/P rs797045390 Menkes disease (MNK) [MIM:309400]
ATP7A Q04656 VAR_023276 p.Ala1325Val LP/P - Menkes disease (MNK) [MIM:309400]
ATP7A Q04656 VAR_023277 p.Ser1344Arg LP/P - Menkes disease (MNK) [MIM:309400]
ATP7A Q04656 VAR_023278 p.Ile1345Phe LP/P - Menkes disease (MNK) [MIM:309400]
ATP7A Q04656 VAR_023279 p.Gly1369Arg LP/P - Menkes disease (MNK) [MIM:309400]
ATP7A Q04656 VAR_023280 p.Ser1397Phe LP/P - Menkes disease (MNK) [MIM:309400]
ATP7A Q04656 VAR_063882 p.Thr994Ile LP/P rs267606673 Distal spinal muscular atrophy, X-linked, 3 (DSMAX3) [MIM:300489]
ATP7A Q04656 VAR_063883 p.Asn1304Ser LP/P rs151340632 Occipital horn syndrome (OHS) [MIM:304150]
ATP7A Q04656 VAR_063884 p.Pro1386Ser LP/P rs267606672 Distal spinal muscular atrophy, X-linked, 3 (DSMAX3) [MIM:300489]
ATP7A Q04656 VAR_068831 p.Thr1048Ile LP/P - Menkes disease (MNK) [MIM:309400]

ATP7A	Q04656	VAR_080663	p.Lys1350Glu	LB/B	rs4826245	-
ATP7A	Q04656	VAR_084344	p.Glu628Val	LP/P	-	Menkes disease (MNK) [MIM:309400]
ATP7A	Q04656	VAR_084345	p.Lys633Arg	LP/P	-	Menkes disease (MNK) [MIM:309400]
ATP7A	Q04656	VAR_084346	p.Ser653Tyr	LP/P	-	Menkes disease (MNK) [MIM:309400]
ATP7A	Q04656	VAR_084347	p.Gly666Arg	LP/P	rs797045344	Menkes disease (MNK) [MIM:309400]
ATP7A	Q04656	VAR_084348	p.Gly728Asp	LP/P	rs797045350	Menkes disease (MNK) [MIM:309400]
ATP7A	Q04656	VAR_084349	p.Ser761Pro	LP/P	-	Menkes disease (MNK) [MIM:309400]
ATP7A	Q04656	VAR_084350	p.Lys802Asn	LP/P	-	Menkes disease (MNK) [MIM:309400]
ATP7A	Q04656	VAR_084351	p.Gly1005Arg	LP/P	rs1569550143	Menkes disease (MNK) [MIM:309400]
ATP7A	Q04656	VAR_084352	p.Lys1037Asn	LP/P	-	Menkes disease (MNK) [MIM:309400]
ATP7A	Q04656	VAR_084353	p.Asp1301Gly	LP/P	rs1557238588	Menkes disease (MNK) [MIM:309400]
ATP7A	Q04656	VAR_084354	p.Gly1302Glu	LP/P	-	Menkes disease (MNK) [MIM:309400]
ATP7A	Q04656	VAR_084355	p.Asp1305Gly	LP/P	-	Menkes disease (MNK) [MIM:309400]
ATP7A	Q04656	VAR_084356	p.Ala1308Asp	LP/P	-	Menkes disease (MNK) [MIM:309400]
ATP7A	Q04656	VAR_084357	p.Ala1373Pro	LP/P	-	Menkes disease (MNK) [MIM:309400]
ATP7A	Q04656	VAR_084358	p.Met1393Thr	LP/P	-	Menkes disease (MNK) [MIM:309400]
ATP7B	P35670	VAR_000703	p.Gly85Val	LP/P	rs786204643	Wilson disease (WD) [MIM:277900]
ATP7B	P35670	VAR_000704	p.Gly96Asp	LB/B	rs1429553821	-
ATP7B	P35670	VAR_000705	p.Ile390Val	LB/B	rs770903362	-
ATP7B	P35670	VAR_000706	p.Ser406Ala	LB/B	rs1801243	-
ATP7B	P35670	VAR_000707	p.Val446Leu	LB/B	rs587783298	-
ATP7B	P35670	VAR_000708	p.Val456Leu	LB/B	rs1801244	-
ATP7B	P35670	VAR_000709	p.Leu466Val	LB/B	-	-
ATP7B	P35670	VAR_000710	p.Leu492Ser	LP/P	rs1566580253	Wilson disease (WD) [MIM:277900]
ATP7B	P35670	VAR_000711	p.Asn565Ser	LB/B	rs778475094	-
ATP7B	P35670	VAR_000712	p.Gly626Ala	US	rs587783299	Wilson disease (WD) [MIM:277900]
ATP7B	P35670	VAR_000713	p.Asp642His	US	rs72552285	Wilson disease (WD) [MIM:277900]
ATP7B	P35670	VAR_000714	p.Met645Arg	LP/P	rs121907998	Wilson disease (WD) [MIM:277900]
ATP7B	P35670	VAR_000715	p.Met665Ile	US	rs72552259	Wilson disease (WD) [MIM:277900]
ATP7B	P35670	VAR_000716	p.Gly691Arg	LP/P	rs121908001	Wilson disease (WD) [MIM:277900]
ATP7B	P35670	VAR_000717	p.Leu708Pro	LP/P	rs121908000	Wilson disease (WD) [MIM:277900]
ATP7B	P35670	VAR_000718	p.Gly710Arg	LP/P	-	Wilson disease (WD) [MIM:277900]
ATP7B	P35670	VAR_000719	p.Gly710Ser	LP/P	rs137853285	Wilson disease (WD) [MIM:277900]
ATP7B	P35670	VAR_000720	p.Gly711Glu	LP/P	-	Wilson disease (WD) [MIM:277900]
ATP7B	P35670	VAR_000721	p.Tyr713Cys	LP/P	rs756883878	Wilson disease (WD) [MIM:277900]
ATP7B	P35670	VAR_000722	p.Arg723Gly	LB/B	-	-
ATP7B	P35670	VAR_000723	p.Ile747Phe	LP/P	-	Wilson disease (WD) [MIM:277900]
ATP7B	P35670	VAR_000724	p.Asp765Asn	LP/P	rs28942075	Wilson disease (WD) [MIM:277900]
ATP7B	P35670	VAR_000725	p.Met769Val	LP/P	rs193922103	Wilson disease (WD) [MIM:277900]
ATP7B	P35670	VAR_000726	p.Leu776Val	US	rs1217463955	Wilson disease (WD) [MIM:277900]
ATP7B	P35670	VAR_000727	p.Arg778Gly	LP/P	rs137853284	Wilson disease (WD) [MIM:277900]
ATP7B	P35670	VAR_000728	p.Arg778Leu	LP/P	rs28942074	Wilson disease (WD) [MIM:277900]
ATP7B	P35670	VAR_000729	p.Arg778Gln	LP/P	rs28942074	Wilson disease (WD) [MIM:277900]
ATP7B	P35670	VAR_000730	p.Arg778Trp	LP/P	rs137853284	Wilson disease (WD) [MIM:277900]
ATP7B	P35670	VAR_000731	p.Leu795Phe	US	rs751710854	Wilson disease (WD) [MIM:277900]
ATP7B	P35670	VAR_000732	p.Lys832Arg	LB/B	rs1061472	-
ATP7B	P35670	VAR_000733	p.Pro840Leu	LP/P	rs768671894	Wilson disease (WD) [MIM:277900]
ATP7B	P35670	VAR_000734	p.Ile857Thr	LP/P	rs1057520235	Wilson disease (WD) [MIM:277900]
ATP7B	P35670	VAR_000735	p.Val864Ile	LB/B	-	-

ATP7B	P35670	VAR_000736	p.Gly869Arg	LP/P	rs191312027	Wilson disease (WD) [MIM:277900]
ATP7B	P35670	VAR_000737	p.Ala874Val	LP/P	rs121907994	Wilson disease (WD) [MIM:277900]
ATP7B	P35670	VAR_000738	p.Asp918Asn	LP/P	rs540935874	Wilson disease (WD) [MIM:277900]
ATP7B	P35670	VAR_000739	p.Arg919Gly	LP/P	rs121907993	Wilson disease (WD) [MIM:277900]
ATP7B	P35670	VAR_000740	p.Arg919Trp	LP/P	rs121907993	Wilson disease (WD) [MIM:277900]
ATP7B	P35670	VAR_000741	p.Ser921Asn	LP/P	rs1230241288	Wilson disease (WD) [MIM:277900]
ATP7B	P35670	VAR_000742	p.Thr933Pro	US	rs1555288410	Wilson disease (WD) [MIM:277900]
ATP7B	P35670	VAR_000743	p.Thr935Met	LP/P	rs750019452	Wilson disease (WD) [MIM:277900]
ATP7B	P35670	VAR_000744	p.Gly943Asp	LP/P	rs779323689	Wilson disease (WD) [MIM:277900]
ATP7B	P35670	VAR_000745	p.Gly943Ser	LP/P	rs28942076	Wilson disease (WD) [MIM:277900]
ATP7B	P35670	VAR_000746	p.Arg952Lys	LB/B	rs732774	-
ATP7B	P35670	VAR_000747	p.Arg969Gln	LP/P	rs121907996	Wilson disease (WD) [MIM:277900]
ATP7B	P35670	VAR_000748	p.Thr977Met	LP/P	rs72552255	Wilson disease (WD) [MIM:277900]
ATP7B	P35670	VAR_000749	p.Pro992Leu	LP/P	rs201038679	Wilson disease (WD) [MIM:277900]
ATP7B	P35670	VAR_000750	p.Val995Ala	US	rs777791532	Wilson disease (WD) [MIM:277900]
ATP7B	P35670	VAR_000751	p.Ala1003Thr	LP/P	rs201497300	Wilson disease (WD) [MIM:277900]
ATP7B	P35670	VAR_000752	p.Ala1018Val	LP/P	rs371840514	Wilson disease (WD) [MIM:277900]
ATP7B	P35670	VAR_000753	p.Gly1035Val	LP/P	rs753594031	Wilson disease (WD) [MIM:277900]
ATP7B	P35670	VAR_000754	p.Arg1041Trp	US	rs746485916	Wilson disease (WD) [MIM:277900]
ATP7B	P35670	VAR_000755	p.Leu1043Pro	LP/P	rs1412025509	Wilson disease (WD) [MIM:277900]
ATP7B	P35670	VAR_000756	p.Glu1064Ala	LP/P	rs374094065	Wilson disease (WD) [MIM:277900]
ATP7B	P35670	VAR_000757	p.Glu1064Lys	LP/P	rs376910645	Wilson disease (WD) [MIM:277900]
ATP7B	P35670	VAR_000758	p.His1069Gln	LP/P	rs76151636	Wilson disease (WD) [MIM:277900]
ATP7B	P35670	VAR_000759	p.Leu1083Phe	LP/P	rs1286080173	Wilson disease (WD) [MIM:277900]
ATP7B	P35670	VAR_000760	p.Gly1089Glu	LP/P	rs1555285911	Wilson disease (WD) [MIM:277900]
ATP7B	P35670	VAR_000761	p.Gly1089Val	LP/P	-	Wilson disease (WD) [MIM:277900]
ATP7B	P35670	VAR_000762	p.Gly1101Arg	LP/P	rs786204483	Wilson disease (WD) [MIM:277900]
ATP7B	P35670	VAR_000763	p.Ile1102Thr	LP/P	rs560952220	Wilson disease (WD) [MIM:277900]
ATP7B	P35670	VAR_000764	p.Val1109Met	LB/B	rs759109027	-
ATP7B	P35670	VAR_000765	p.Val1140Ala	LB/B	rs1801249	-
ATP7B	P35670	VAR_000766	p.Gln1142His	LP/P	rs778749563	Wilson disease (WD) [MIM:277900]
ATP7B	P35670	VAR_000767	p.Val1146Met	LP/P	rs1213481140	Wilson disease (WD) [MIM:277900]
ATP7B	P35670	VAR_000768	p.Ile1148Thr	LP/P	rs60431989	Wilson disease (WD) [MIM:277900]
ATP7B	P35670	VAR_000769	p.Trp1153Cys	LP/P	rs1330620114	Wilson disease (WD) [MIM:277900]
ATP7B	P35670	VAR_000770	p.Met1169Val	LP/P	rs749085322	Wilson disease (WD) [MIM:277900]
ATP7B	P35670	VAR_000771	p.Ala1183Gly	LP/P	rs587783315	Wilson disease (WD) [MIM:277900]
ATP7B	P35670	VAR_000772	p.Ala1183Thr	US	-	Wilson disease (WD) [MIM:277900]
ATP7B	P35670	VAR_000773	p.Gly1186Cys	LP/P	-	Wilson disease (WD) [MIM:277900]
ATP7B	P35670	VAR_000774	p.Gly1186Ser	US	rs786204547	Wilson disease (WD) [MIM:277900]
ATP7B	P35670	VAR_000775	p.Gly1213Val	LP/P	rs1555284582	Wilson disease (WD) [MIM:277900]
ATP7B	P35670	VAR_000776	p.Val1216Met	LP/P	rs776280797	Wilson disease (WD) [MIM:277900]
ATP7B	P35670	VAR_000778	p.Thr1220Met	LP/P	rs193922107	Wilson disease (WD) [MIM:277900]
ATP7B	P35670	VAR_000779	p.Asp1222Tyr	LP/P	-	Wilson disease (WD) [MIM:277900]
ATP7B	P35670	VAR_000781	p.Gly1266Val	LP/P	-	Wilson disease (WD) [MIM:277900]
ATP7B	P35670	VAR_000782	p.Asp1267Ala	LP/P	rs1555283916	Wilson disease (WD) [MIM:277900]
ATP7B	P35670	VAR_000783	p.Asn1270Ser	LP/P	rs121907990	Wilson disease (WD) [MIM:277900]
ATP7B	P35670	VAR_000784	p.Pro1273Leu	LP/P	rs758355520	Wilson disease (WD) [MIM:277900]
ATP7B	P35670	VAR_000785	p.Ala1278Val	US	-	Wilson disease (WD) [MIM:277900]
ATP7B	P35670	VAR_000787	p.Ser1310Arg	LP/P	rs749380700	Wilson disease (WD) [MIM:277900]

ATP7B	P35670	VAR_000788	p.Arg1322Pro	LP/P	rs753330854	Wilson disease (WD) [MIM:277900]
ATP7B	P35670	VAR_000789	p.Gly1341Asp	LP/P	rs779494870	Wilson disease (WD) [MIM:277900]
ATP7B	P35670	VAR_000790	p.Trp1353Arg	LP/P	rs1160679283	Wilson disease (WD) [MIM:277900]
ATP7B	P35670	VAR_000791	p.Ala1358Ser	LP/P	-	Wilson disease (WD) [MIM:277900]
ATP7B	P35670	VAR_009004	p.Arg616Gln	US	rs752850609	Wilson disease (WD) [MIM:277900]
ATP7B	P35670	VAR_009006	p.Gly711Arg	LP/P	rs1394999756	Wilson disease (WD) [MIM:277900]
ATP7B	P35670	VAR_009007	p.Gly711Trp	LP/P	rs1394999756	Wilson disease (WD) [MIM:277900]
ATP7B	P35670	VAR_009008	p.Ser744Pro	LP/P	rs1593726081	Wilson disease (WD) [MIM:277900]
ATP7B	P35670	VAR_009009	p.Met769Arg	LP/P	rs772595172	Wilson disease (WD) [MIM:277900]
ATP7B	P35670	VAR_009010	p.Leu795Arg	LP/P	-	Wilson disease (WD) [MIM:277900]
ATP7B	P35670	VAR_009011	p.Gly869Val	LP/P	-	Wilson disease (WD) [MIM:277900]
ATP7B	P35670	VAR_009012	p.Cys985Tyr	LP/P	-	Wilson disease (WD) [MIM:277900]
ATP7B	P35670	VAR_009013	p.Ala1003Val	LP/P	rs775055397	Wilson disease (WD) [MIM:277900]
ATP7B	P35670	VAR_009014	p.Thr1033Ala	LP/P	rs1555286620	Wilson disease (WD) [MIM:277900]
ATP7B	P35670	VAR_009015	p.Arg1041Pro	LP/P	-	Wilson disease (WD) [MIM:277900]
ATP7B	P35670	VAR_009016	p.Pro1052Leu	LP/P	rs778543794	Wilson disease (WD) [MIM:277900]
ATP7B	P35670	VAR_009017	p.Gly1061Glu	LP/P	rs764131178	Wilson disease (WD) [MIM:277900]
ATP7B	P35670	VAR_009018	p.Ala1063Val	LP/P	rs587783309	Wilson disease (WD) [MIM:277900]
ATP7B	P35670	VAR_009019	p.Glu1068Gly	LP/P	rs1555286478	Wilson disease (WD) [MIM:277900]
ATP7B	P35670	VAR_009020	p.Gln1095Pro	LP/P	rs1555285891	Wilson disease (WD) [MIM:277900]
ATP7B	P35670	VAR_009021	p.Cys1104Phe	LP/P	-	Wilson disease (WD) [MIM:277900]
ATP7B	P35670	VAR_009022	p.Arg1151His	LP/P	rs377297166	Wilson disease (WD) [MIM:277900]
ATP7B	P35670	VAR_009023	p.Met1169Thr	LP/P	rs1555285311	Wilson disease (WD) [MIM:277900]
ATP7B	P35670	VAR_009024	p.Glu1173Lys	LP/P	rs756029120	Wilson disease (WD) [MIM:277900]
ATP7B	P35670	VAR_009025	p.His1207Arg	LB/B	rs7334118	-
ATP7B	P35670	VAR_009026	p.Val1239Gly	LP/P	rs374628199	Wilson disease (WD) [MIM:277900]
ATP7B	P35670	VAR_009027	p.Val1262Phe	LP/P	rs769484789	Wilson disease (WD) [MIM:277900]
ATP7B	P35670	VAR_009028	p.Gly1266Arg	LP/P	rs121907992	Wilson disease (WD) [MIM:277900]
ATP7B	P35670	VAR_009029	p.Val1297Ile	LB/B	rs148399850	-
ATP7B	P35670	VAR_009030	p.Leu1327Val	LP/P	-	Wilson disease (WD) [MIM:277900]
ATP7B	P35670	VAR_009031	p.Ser1363Phe	LP/P	rs776848753	Wilson disease (WD) [MIM:277900]
ATP7B	P35670	VAR_009032	p.Thr1434Met	US	rs60986317	Wilson disease (WD) [MIM:277900]
ATP7B	P35670	VAR_010010	p.Gly710Ala	LP/P	rs1555291285	Wilson disease (WD) [MIM:277900]
ATP7B	P35670	VAR_010011	p.Tyr741Cys	LP/P	rs770533110	Wilson disease (WD) [MIM:277900]
ATP7B	P35670	VAR_010012	p.Gly891Val	LP/P	-	Wilson disease (WD) [MIM:277900]
ATP7B	P35670	VAR_010013	p.Ile967Phe	LP/P	rs60003608	Wilson disease (WD) [MIM:277900]
ATP7B	P35670	VAR_010014	p.Thr1031Ile	LP/P	-	Wilson disease (WD) [MIM:277900]
ATP7B	P35670	VAR_010015	p.Arg1038Lys	LP/P	rs59959366	Wilson disease (WD) [MIM:277900]
ATP7B	P35670	VAR_010017	p.Val1106Asp	LP/P	rs775541743	Wilson disease (WD) [MIM:277900]
ATP7B	P35670	VAR_010018	p.Trp1153Arg	LP/P	-	Wilson disease (WD) [MIM:277900]
ATP7B	P35670	VAR_010019	p.Gly1176Arg	LP/P	rs137853279	Wilson disease (WD) [MIM:277900]
ATP7B	P35670	VAR_010020	p.Asp1222Val	LP/P	-	Wilson disease (WD) [MIM:277900]
ATP7B	P35670	VAR_010021	p.Gly1355Ser	LP/P	rs1555282751	Wilson disease (WD) [MIM:277900]
ATP7B	P35670	VAR_023010	p.Ala14Asp	LB/B	rs587783319	-
ATP7B	P35670	VAR_023011	p.Asn41Ser	LP/P	rs201738967	Wilson disease (WD) [MIM:277900]
ATP7B	P35670	VAR_023012	p.Arg616Trp	LP/P	rs374172791	Wilson disease (WD) [MIM:277900]
ATP7B	P35670	VAR_023013	p.Leu641Ser	US	rs186924074	Wilson disease (WD) [MIM:277900]
ATP7B	P35670	VAR_023014	p.Pro690Leu	LP/P	rs1555291809	Wilson disease (WD) [MIM:277900]
ATP7B	P35670	VAR_023015	p.Ser693Cys	LP/P	rs1212479289	Wilson disease (WD) [MIM:277900]

ATP7B	P35670	VAR_023016	p.Ser721Pro	LP/P	rs765667658	Wilson disease (WD) [MIM:277900]
ATP7B	P35670	VAR_023017	p.Thr737Arg	US	-	Wilson disease (WD) [MIM:277900]
ATP7B	P35670	VAR_023018	p.Pro760Leu	LP/P	rs766907687	Wilson disease (WD) [MIM:277900]
ATP7B	P35670	VAR_023019	p.Asp765Gly	LP/P	rs1555291147	Wilson disease (WD) [MIM:277900]
ATP7B	P35670	VAR_023020	p.Pro768His	LP/P	-	Wilson disease (WD) [MIM:277900]
ATP7B	P35670	VAR_023021	p.Met769Ile	LP/P	-	Wilson disease (WD) [MIM:277900]
ATP7B	P35670	VAR_023022	p.Gly875Arg	LB/B	rs587783304	-
ATP7B	P35670	VAR_023023	p.Val890Met	LP/P	rs786204718	Wilson disease (WD) [MIM:277900]
ATP7B	P35670	VAR_023024	p.Gln898Arg	LP/P	-	Wilson disease (WD) [MIM:277900]
ATP7B	P35670	VAR_023025	p.Asp918Glu	LP/P	-	Wilson disease (WD) [MIM:277900]
ATP7B	P35670	VAR_023026	p.Val949Gly	LP/P	rs1169959260	Wilson disease (WD) [MIM:277900]
ATP7B	P35670	VAR_023027	p.Ser975Tyr	LP/P	rs778163447	Wilson disease (WD) [MIM:277900]
ATP7B	P35670	VAR_023028	p.Thr1033Ser	LP/P	-	Wilson disease (WD) [MIM:277900]
ATP7B	P35670	VAR_023029	p.Phe1094Leu	LP/P	rs1397083296	Wilson disease (WD) [MIM:277900]
ATP7B	P35670	VAR_023030	p.Pro1098Arg	LP/P	-	Wilson disease (WD) [MIM:277900]
ATP7B	P35670	VAR_023031	p.Gly1099Ser	LP/P	rs761632029	Wilson disease (WD) [MIM:277900]
ATP7B	P35670	VAR_023032	p.Gly1111Asp	LP/P	rs182659444	Wilson disease (WD) [MIM:277900]
ATP7B	P35670	VAR_023033	p.Thr1143Asn	LB/B	rs587783313	-
ATP7B	P35670	VAR_023034	p.Ala1168Ser	LP/P	rs777879359	Wilson disease (WD) [MIM:277900]
ATP7B	P35670	VAR_023035	p.Thr1232Pro	LP/P	rs568009639	Wilson disease (WD) [MIM:277900]
ATP7B	P35670	VAR_023036	p.Pro1245Ser	LB/B	rs587783316	-
ATP7B	P35670	VAR_023037	p.Lys1248Asn	LP/P	-	Wilson disease (WD) [MIM:277900]
ATP7B	P35670	VAR_023038	p.Leu1255Ile	LP/P	-	Wilson disease (WD) [MIM:277900]
ATP7B	P35670	VAR_023039	p.Asp1271Asn	LP/P	-	Wilson disease (WD) [MIM:277900]
ATP7B	P35670	VAR_023040	p.Asp1279Gly	LP/P	rs778914828	Wilson disease (WD) [MIM:277900]
ATP7B	P35670	VAR_023041	p.Leu1305Pro	LP/P	rs377144951	Wilson disease (WD) [MIM:277900]
ATP7B	P35670	VAR_023042	p.Ile1336Thr	LP/P	-	Wilson disease (WD) [MIM:277900]
ATP7B	P35670	VAR_023043	p.Gly1355Cys	LP/P	-	Wilson disease (WD) [MIM:277900]
ATP7B	P35670	VAR_023044	p.Leu1373Pro	LP/P	rs780811477	Wilson disease (WD) [MIM:277900]
ATP7B	P35670	VAR_023045	p.Leu1373Arg	LP/P	rs780811477	Wilson disease (WD) [MIM:277900]
ATP7B	P35670	VAR_044453	p.Val290Leu	LB/B	-	-
ATP7B	P35670	VAR_044454	p.Ala486Ser	US	rs1282624946	Wilson disease (WD) [MIM:277900]
ATP7B	P35670	VAR_044455	p.Tyr532His	US	-	Wilson disease (WD) [MIM:277900]
ATP7B	P35670	VAR_044456	p.Gly591Asp	LP/P	rs797045402	Wilson disease (WD) [MIM:277900]
ATP7B	P35670	VAR_044457	p.Ala604Pro	US	-	Wilson disease (WD) [MIM:277900]
ATP7B	P35670	VAR_044458	p.His639Tyr	US	rs200728096	Wilson disease (WD) [MIM:277900]
ATP7B	P35670	VAR_044459	p.Ser653Tyr	US	-	Wilson disease (WD) [MIM:277900]
ATP7B	P35670	VAR_044460	p.Cys703Tyr	LP/P	rs767218895	Wilson disease (WD) [MIM:277900]
ATP7B	P35670	VAR_044461	p.Gly710Val	LP/P	-	Wilson disease (WD) [MIM:277900]
ATP7B	P35670	VAR_044462	p.Ala756Gly	LP/P	-	Wilson disease (WD) [MIM:277900]
ATP7B	P35670	VAR_044463	p.Thr766Met	LP/P	rs121907997	Wilson disease (WD) [MIM:277900]
ATP7B	P35670	VAR_044464	p.Thr766Arg	LP/P	rs121907997	Wilson disease (WD) [MIM:277900]
ATP7B	P35670	VAR_044465	p.Leu776Pro	LP/P	-	Wilson disease (WD) [MIM:277900]
ATP7B	P35670	VAR_044466	p.Ala861Thr	LP/P	-	Wilson disease (WD) [MIM:277900]
ATP7B	P35670	VAR_044467	p.Gly875Val	LP/P	-	Wilson disease (WD) [MIM:277900]
ATP7B	P35670	VAR_044468	p.Gly943Cys	LP/P	rs28942076	Wilson disease (WD) [MIM:277900]
ATP7B	P35670	VAR_044469	p.Gly988Arg	LP/P	rs199623434	Wilson disease (WD) [MIM:277900]
ATP7B	P35670	VAR_044470	p.Thr991Met	LP/P	rs41292782	Wilson disease (WD) [MIM:277900]
ATP7B	P35670	VAR_044471	p.Pro992His	LP/P	rs201038679	Wilson disease (WD) [MIM:277900]

ATP7B	P35670	VAR_044472	p.Met996Thr	LP/P	rs770782111	Wilson disease (WD) [MIM:277900]
ATP7B	P35670	VAR_044473	p.Gly1000Arg	LP/P	rs751078884	Wilson disease (WD) [MIM:277900]
ATP7B	P35670	VAR_044474	p.Thr1029Ile	LP/P	rs1555286628	Wilson disease (WD) [MIM:277900]
ATP7B	P35670	VAR_044475	p.Ala1065Pro	LP/P	-	Wilson disease (WD) [MIM:277900]
ATP7B	P35670	VAR_044476	p.Cys1104Tyr	LP/P	rs764041557	Wilson disease (WD) [MIM:277900]
ATP7B	P35670	VAR_044477	p.Val1106Ile	US	rs541208827	Wilson disease (WD) [MIM:277900]
ATP7B	P35670	VAR_044478	p.Gly1176Glu	LP/P	rs1318758433	Wilson disease (WD) [MIM:277900]
ATP7B	P35670	VAR_044480	p.Gly1221Glu	LP/P	rs1486594906	Wilson disease (WD) [MIM:277900]
ATP7B	P35670	VAR_044481	p.Asp1222Asn	LP/P	-	Wilson disease (WD) [MIM:277900]
ATP7B	P35670	VAR_044482	p.Val1252Ile	LP/P	-	Wilson disease (WD) [MIM:277900]
ATP7B	P35670	VAR_044483	p.Gln1256Arg	LP/P	rs1555283946	Wilson disease (WD) [MIM:277900]
ATP7B	P35670	VAR_044484	p.Asp1279Tyr	LP/P	-	Wilson disease (WD) [MIM:277900]
ATP7B	P35670	VAR_044485	p.Gly1287Ser	LP/P	rs762866453	Wilson disease (WD) [MIM:277900]
ATP7B	P35670	VAR_044486	p.Asp1296Asn	LP/P	rs199821556	Wilson disease (WD) [MIM:277900]
ATP7B	P35670	VAR_044488	p.Tyr1331Ser	LP/P	rs1131691741	Wilson disease (WD) [MIM:277900]
ATP7B	P35670	VAR_044489	p.Gly1341Ser	LP/P	rs587783317	Wilson disease (WD) [MIM:277900]
ATP7B	P35670	VAR_044490	p.Gly1341Val	LP/P	-	Wilson disease (WD) [MIM:277900]
ATP7B	P35670	VAR_044491	p.Pro1352Ser	LP/P	rs1388795855	Wilson disease (WD) [MIM:277900]
ATP7B	P35670	VAR_044492	p.Leu1368Pro	LP/P	rs749171049	Wilson disease (WD) [MIM:277900]
ATP7B	P35670	VAR_044493	p.Cys1375Ser	US	rs1365425480	Wilson disease (WD) [MIM:277900]
ATP7B	P35670	VAR_044494	p.Pro1379Ser	US	rs181250704	Wilson disease (WD) [MIM:277900]
ATP7B	P35670	VAR_044495	p.Asp1407Glu	LB/B	rs587783320	-
ATP7B	P35670	VAR_058925	p.Val536Ala	LB/B	rs138427376	Wilson disease (WD) [MIM:277900]
ATP7B	P35670	VAR_058926	p.Ser657Arg	US	rs372436901	Wilson disease (WD) [MIM:277900]
ATP7B	P35670	VAR_058927	p.Ala971Val	LP/P	rs770340441	Wilson disease (WD) [MIM:277900]
ATP7B	P35670	VAR_058928	p.Thr974Met	LP/P	rs201061621	Wilson disease (WD) [MIM:277900]
ATP7B	P35670	VAR_058929	p.Gln1004Pro	LP/P	rs587783307	Wilson disease (WD) [MIM:277900]
ATP7B	P35670	VAR_058930	p.Gly1149Ala	LP/P	rs1566462533	Wilson disease (WD) [MIM:277900]
ATP7B	P35670	VAR_058931	p.Asp1164Asn	LP/P	rs867107727	Wilson disease (WD) [MIM:277900]
ATP7B	P35670	VAR_058932	p.Glu1173Gly	LP/P	-	Wilson disease (WD) [MIM:277900]
ATP7B	P35670	VAR_058933	p.Arg1228Thr	LP/P	-	Wilson disease (WD) [MIM:277900]
ATP7B	P35670	VAR_058934	p.Ile1230Val	LP/P	rs200911496	Wilson disease (WD) [MIM:277900]
ATP7B	P35670	VAR_058935	p.Asp1267Val	LP/P	-	Wilson disease (WD) [MIM:277900]
ATP7B	P35670	VAR_058936	p.Ala1328Thr	LP/P	rs1333619338	Wilson disease (WD) [MIM:277900]
ATP7B	P35670	VAR_058937	p.Met1359Ile	LP/P	rs759551693	Wilson disease (WD) [MIM:277900]
ATP7B	P35670	VAR_067335	p.Leu549Pro	US	-	Wilson disease (WD) [MIM:277900]
ATP7B	P35670	VAR_067336	p.Gly998Asp	LP/P	-	Wilson disease (WD) [MIM:277900]
ATP7B	P35670	VAR_067337	p.Asn1332Asp	LP/P	-	Wilson disease (WD) [MIM:277900]
ATP7B	P35670	VAR_067338	p.Gly1341Arg	LP/P	rs587783317	Wilson disease (WD) [MIM:277900]
ATP7B	P35670	VAR_075336	p.Thr788Ile	LP/P	rs541408630	Wilson disease (WD) [MIM:277900]
ATP7B	P35670	VAR_075337	p.Val1036Ile	LP/P	rs761147984	Wilson disease (WD) [MIM:277900]
ATP7B	P35670	VAR_075338	p.Arg1151Cys	LP/P	rs755554442	Wilson disease (WD) [MIM:277900]
ATP7B	P35670	VAR_075339	p.Val1243Leu	LB/B	rs1277243795	-
ATP7B	P35670	VAR_075340	p.Pro1245Thr	LP/P	-	Wilson disease (WD) [MIM:277900]
ATP7B	P35670	VAR_076693	p.Arg136Trp	US	rs557577836	Wilson disease (WD) [MIM:277900]
ATP7B	P35670	VAR_076694	p.Arg148Trp	US	rs373762572	Wilson disease (WD) [MIM:277900]
ATP7B	P35670	VAR_076695	p.Ser382Cys	US	rs774102085	Wilson disease (WD) [MIM:277900]
ATP7B	P35670	VAR_076696	p.Glu541Lys	US	rs187046823	Wilson disease (WD) [MIM:277900]
ATP7B	P35670	VAR_076697	p.Val597Ile	US	rs760501309	Wilson disease (WD) [MIM:277900]

ATP7B	P35670	VAR_076698	p.Gly614Cys	US	rs376565432	Wilson disease (WD) [MIM:277900]
ATP7B	P35670	VAR_076699	p.Val731Ala	US	-	Wilson disease (WD) [MIM:277900]
ATP7B	P35670	VAR_076700	p.Leu745Pro	US	rs1362773192	Wilson disease (WD) [MIM:277900]
ATP7B	P35670	VAR_076701	p.Leu936Val	US	rs367855110	Wilson disease (WD) [MIM:277900]
ATP7B	P35670	VAR_076702	p.Met1017Ile	US	rs755851188	Wilson disease (WD) [MIM:277900]
ATP7B	P35670	VAR_076703	p.Ile1021Val	US	rs776490710	Wilson disease (WD) [MIM:277900]
ATP7B	P35670	VAR_076704	p.Ala1058Val	US	-	Wilson disease (WD) [MIM:277900]
ATP7B	P35670	VAR_076705	p.Pro1070Ser	US	rs1423701688	Wilson disease (WD) [MIM:277900]
ATP7B	P35670	VAR_076706	p.Ala1074Val	US	rs1206016866	Wilson disease (WD) [MIM:277900]
ATP7B	P35670	VAR_076707	p.Ala1250Gly	US	rs372042739	Wilson disease (WD) [MIM:277900]
ATP7B	P35670	VAR_076708	p.Val1298Ile	US	rs753044473	Wilson disease (WD) [MIM:277900]
ATP7B	P35670	VAR_076709	p.Val1298Leu	US	-	Wilson disease (WD) [MIM:277900]
ATP7B	P35670	VAR_076710	p.Ser1431Tyr	US	-	Wilson disease (WD) [MIM:277900]
ATP7B	P35670	VAR_076711	p.Ser1432Phe	US	rs375692175	Wilson disease (WD) [MIM:277900]
ATP7B	P35670	VAR_076729	p.Tyr44Asn	US	rs1566605396	Wilson disease (WD) [MIM:277900]
ATP7B	P35670	VAR_076730	p.Cys108Arg	US	rs1566603189	Wilson disease (WD) [MIM:277900]
ATP7B	P35670	VAR_076731	p.Cys157Phe	US	rs551275663	Wilson disease (WD) [MIM:277900]
ATP7B	P35670	VAR_076732	p.Val606Gly	US	rs1173050016	Wilson disease (WD) [MIM:277900]
ATP7B	P35670	VAR_076733	p.Met729Val	US	rs773447981	Wilson disease (WD) [MIM:277900]
ATP7B	P35670	VAR_076734	p.Leu732His	US	-	Wilson disease (WD) [MIM:277900]
ATP7B	P35670	VAR_076735	p.Leu732Pro	US	-	Wilson disease (WD) [MIM:277900]
ATP7B	P35670	VAR_076736	p.Arg827Trp	LP/P	rs539585071	Wilson disease (WD) [MIM:277900]
ATP7B	P35670	VAR_076737	p.Ala874Pro	US	rs376355660	Wilson disease (WD) [MIM:277900]
ATP7B	P35670	VAR_076738	p.Gly891Asp	US	rs483352684	Wilson disease (WD) [MIM:277900]
ATP7B	P35670	VAR_076740	p.Ser921Arg	US	rs1052485948	Wilson disease (WD) [MIM:277900]
ATP7B	P35670	VAR_076741	p.Ile929Val	LB/B	rs534960245	-
ATP7B	P35670	VAR_076742	p.Cys980Tyr	US	rs1038582488	Wilson disease (WD) [MIM:277900]
ATP7B	P35670	VAR_076743	p.Leu987Pro	US	-	Wilson disease (WD) [MIM:277900]
ATP7B	P35670	VAR_076744	p.Lys1010Thr	LP/P	rs747584649	Wilson disease (WD) [MIM:277900]
ATP7B	P35670	VAR_076745	p.Val1024Ala	US	rs1416453532	Wilson disease (WD) [MIM:277900]
ATP7B	P35670	VAR_076746	p.Thr1031Ala	US	-	Wilson disease (WD) [MIM:277900]
ATP7B	P35670	VAR_076747	p.Cys1091Tyr	US	rs778825095	Wilson disease (WD) [MIM:277900]
ATP7B	P35670	VAR_076748	p.Thr1178Ala	US	rs1387431334	Wilson disease (WD) [MIM:277900]
ATP7B	P35670	VAR_076749	p.Ala1202Gly	US	-	Wilson disease (WD) [MIM:277900]
ATP7B	P35670	VAR_076750	p.Gly1266Glu	US	rs1566444586	Wilson disease (WD) [MIM:277900]
ATP7B	P35670	VAR_076751	p.Gly1268Arg	US	rs1314712150	Wilson disease (WD) [MIM:277900]
ATP7B	P35670	VAR_076752	p.Ala1295Asp	LP/P	rs1340942427	Wilson disease (WD) [MIM:277900]
ATP7B	P35670	VAR_076765	p.Arg827Pro	US	rs368589213	Wilson disease (WD) [MIM:277900]
ATP7B	P35670	VAR_076766	p.Thr858Ala	US	-	Wilson disease (WD) [MIM:277900]
ATP7B	P35670	VAR_076767	p.Ile899Phe	US	-	Wilson disease (WD) [MIM:277900]
ATP7B	P35670	VAR_076768	p.Arg969Trp	US	rs774028495	Wilson disease (WD) [MIM:277900]
ATP7B	P35670	VAR_076769	p.Ala982Val	US	rs1487547257	Wilson disease (WD) [MIM:277900]
ATP7B	P35670	VAR_076770	p.Gly1012Arg	US	-	Wilson disease (WD) [MIM:277900]
ATP7B	P35670	VAR_076771	p.Gly1012Val	US	rs772089544	Wilson disease (WD) [MIM:277900]
ATP7B	P35670	VAR_076772	p.Thr1288Met	US	rs373748155	Wilson disease (WD) [MIM:277900]
ATP7B	P35670	VAR_076773	p.Asn1332Lys	US	-	Wilson disease (WD) [MIM:277900]
ATP7B	P35670	VAR_076774	p.Ser1369Leu	US	rs1555282678	Wilson disease (WD) [MIM:277900]
ATP7B	P35670	VAR_076793	p.Val149Leu	LB/B	-	-
ATP7B	P35670	VAR_076794	p.Gly591Ser	US	rs1566559224	Wilson disease (WD) [MIM:277900]

ATP7B P35670 VAR_076795 p.Val825Leu LB/B - -
ATP7B P35670 VAR_076810 p.Gly170Val US - Wilson disease (WD) [MIM:277900]
ATP7B P35670 VAR_076811 p.Asp765His US rs28942075 Wilson disease (WD) [MIM:277900]
ATP7B P35670 VAR_076812 p.Gly836Glu US rs773809011 Wilson disease (WD) [MIM:277900]
ATP7B P35670 VAR_076813 p.Trp939Cys LP/P rs1057517310 Wilson disease (WD) [MIM:277900]
ATP7B P35670 VAR_076814 p.Ala990Pro US - Wilson disease (WD) [MIM:277900]
ATP7B P35670 VAR_076815 p.Lys1010Arg US rs747584649 Wilson disease (WD) [MIM:277900]
ATP7B P35670 VAR_076816 p.Cys1104Ser LP/P - Wilson disease (WD) [MIM:277900]
ATP7B P35670 VAR_076817 p.Leu1113Met US - Wilson disease (WD) [MIM:277900]
ATP7B P35670 VAR_076818 p.Gly1281Asp US rs755202606 Wilson disease (WD) [MIM:277900]
ATP7B P35670 VAR_076819 p.Glu1293Lys US rs776300396 Wilson disease (WD) [MIM:277900]
ATP7B P35670 VAR_076970 p.Pro539Leu LP/P rs572122562 Wilson disease (WD) [MIM:277900]
ATP7B P35670 VAR_076971 p.Trp779Gly LP/P rs751798708 Wilson disease (WD) [MIM:277900]
ATP7B P35670 VAR_076972 p.Arg816Ser LP/P - Wilson disease (WD) [MIM:277900]
ATP7B P35670 VAR_076973 p.Gly1347Ser US rs587783318 Wilson disease (WD) [MIM:277900]
ATP7B P35670 VAR_077616 p.Ala982Thr US rs750407121 Wilson disease (WD) [MIM:277900]
ATP7B P35670 VAR_077617 p.Glu1136Lys US - Wilson disease (WD) [MIM:277900]
ATP7B P35670 VAR_077618 p.Gly1149Glu US - Wilson disease (WD) [MIM:277900]
ATP7B P35670 VAR_079550 p.Lys1010Glu US rs1414727042 Wilson disease (WD) [MIM:277900]
ATP8A1 Q9Y2Q0 VAR_022003 p.Thr673Met LB/B rs3792687 -
ATP8A2 Q9NTI2 VAR_055543 p.Ala1069Thr LB/B rs2296242 -
ATP8A2 Q9NTI2 VAR_069928 p.Ile376Met LP/P rs546968533 Cerebellar ataxia, mental retardation, and dysequilibrium syndrome 4 (CAMRQ4) [MIM:615268]
ATP8A2 Q9NTI2 VAR_084370 p.Lys429Asn US - Cerebellar ataxia, mental retardation, and dysequilibrium syndrome 4 (CAMRQ4) [MIM:615268]
ATP8A2 Q9NTI2 VAR_084371 p.Lys429Met US - Cerebellar ataxia, mental retardation, and dysequilibrium syndrome 4 (CAMRQ4) [MIM:615268]
ATP8A2 Q9NTI2 VAR_084372 p.Ala544Pro US - Cerebellar ataxia, mental retardation, and dysequilibrium syndrome 4 (CAMRQ4) [MIM:615268]
ATP8A2 Q9NTI2 VAR_084375 p.Arg625Trp US rs764911379 Cerebellar ataxia, mental retardation, and dysequilibrium syndrome 4 (CAMRQ4) [MIM:615268]
ATP8A2 Q9NTI2 VAR_084376 p.Trp702Arg US - Cerebellar ataxia, mental retardation, and dysequilibrium syndrome 4 (CAMRQ4) [MIM:615268]
ATP8A2 Q9NTI2 VAR_084377 p.Asn917Asp US - Cerebellar ataxia, mental retardation, and dysequilibrium syndrome 4 (CAMRQ4) [MIM:615268]
ATP8B1 O43520 VAR_008809 p.Leu288Ser LP/P rs121909099 Cholestasis, progressive familial intrahepatic, 1 (PFIC1) [MIM:211600]
ATP8B1 O43520 VAR_008810 p.Gly308Val LP/P rs111033609 Cholestasis, progressive familial intrahepatic, 1 (PFIC1) [MIM:211600]
ATP8B1 O43520 VAR_008812 p.Ile661Thr LP/P rs121909100 Cholestasis, benign recurrent intrahepatic, 1 (BRIC1) [MIM:243300]
ATP8B1 O43520 VAR_008812 p.Ile661Thr LP/P rs121909100 Cholestasis, progressive familial intrahepatic, 1 (PFIC1) [MIM:211600]
ATP8B1 O43520 VAR_008813 p.Gly892Arg LP/P rs121909098 Cholestasis, benign recurrent intrahepatic, 1 (BRIC1) [MIM:243300]
ATP8B1 O43520 VAR_008813 p.Gly892Arg LP/P rs121909098 Cholestasis, progressive familial intrahepatic, 1 (PFIC1) [MIM:211600]
ATP8B1 O43520 VAR_015423 p.Asp554Asn LP/P rs121909101 Cholestasis, progressive familial intrahepatic, 1 (PFIC1) [MIM:211600]

ATP8B1 O43520 VAR_029271 p.His78Gln LB/B rs3745079 -
ATP8B1 O43520 VAR_029272 p.Ile577Val LB/B rs3745078 -
ATP8B1 O43520 VAR_029273 p.Arg952Gln LB/B rs12968116 -
ATP8B1 O43520 VAR_036499 p.Ala886Val US rs767398921 A breast cancer sample
ATP8B1 O43520 VAR_036500 p.Ile1178Met US - A breast cancer sample
ATP8B1 O43520 VAR_043044 p.Asn45Thr LP/P rs146599962 Cholestasis of pregnancy, intrahepatic 1 (ICP1) [MIM:147480]
ATP8B1 O43520 VAR_043045 p.Asp70Asn US rs34719006 Cholestasis, benign recurrent intrahepatic, 1 (BRIC1) [MIM:243300]
ATP8B1 O43520 VAR_043046 p.Leu127Pro LP/P - Cholestasis, progressive familial intrahepatic, 1 (PFIC1) [MIM:211600]
ATP8B1 O43520 VAR_043047 p.Lys203Glu US rs56355310 Cholestasis of pregnancy, intrahepatic 1 (ICP1) [MIM:147480]
ATP8B1 O43520 VAR_043048 p.Phe305Ile LB/B rs150860808 -
ATP8B1 O43520 VAR_043049 p.Gly308Asp LP/P rs111033609 Cholestasis, benign recurrent intrahepatic, 1 (BRIC1) [MIM:243300]
ATP8B1 O43520 VAR_043050 p.Ile344Phe LP/P rs140665115 Cholestasis, benign recurrent intrahepatic, 1 (BRIC1) [MIM:243300]
ATP8B1 O43520 VAR_043051 p.Arg384His LB/B rs2271260 -
ATP8B1 O43520 VAR_043052 p.Ile393Val LB/B rs34315917 -
ATP8B1 O43520 VAR_043053 p.Ser403Tyr LP/P - Cholestasis, progressive familial intrahepatic, 1 (PFIC1) [MIM:211600]
ATP8B1 O43520 VAR_043054 p.Arg412Pro LP/P - Cholestasis, progressive familial intrahepatic, 1 (PFIC1) [MIM:211600]
ATP8B1 O43520 VAR_043055 p.Glu429Ala LB/B rs34018205 -
ATP8B1 O43520 VAR_043056 p.Ser453Tyr LP/P - Cholestasis, benign recurrent intrahepatic, 1 (BRIC1) [MIM:243300]
ATP8B1 O43520 VAR_043057 p.Asp454Gly LP/P - Cholestasis, benign recurrent intrahepatic, 1 (BRIC1) [MIM:243300]
ATP8B1 O43520 VAR_043058 p.Thr456Met LP/P rs121909104 Cholestasis, progressive familial intrahepatic, 1 (PFIC1) [MIM:211600]
ATP8B1 O43520 VAR_043059 p.Tyr500His LP/P rs147642236 Cholestasis, progressive familial intrahepatic, 1 (PFIC1) [MIM:211600]
ATP8B1 O43520 VAR_043061 p.His535Leu LP/P - Cholestasis, progressive familial intrahepatic, 1 (PFIC1) [MIM:211600]
ATP8B1 O43520 VAR_043062 p.Ser580Asn LB/B rs33963153 -
ATP8B1 O43520 VAR_043063 p.Arg600Gln LP/P rs1202682161 Cholestasis, benign recurrent intrahepatic, 1 (BRIC1) [MIM:243300]
ATP8B1 O43520 VAR_043064 p.Arg600Trp LP/P rs780186596 Cholestasis, benign recurrent intrahepatic, 1 (BRIC1) [MIM:243300]
ATP8B1 O43520 VAR_043065 p.Arg628Trp LP/P rs752045131 Cholestasis, benign recurrent intrahepatic, 1 (BRIC1) [MIM:243300]
ATP8B1 O43520 VAR_043066 p.Met674Thr LB/B rs35470719 -
ATP8B1 O43520 VAR_043067 p.Asp688Gly LP/P rs1337978497 Cholestasis, progressive familial intrahepatic, 1 (PFIC1) [MIM:211600]
ATP8B1 O43520 VAR_043068 p.Ile694Thr LP/P rs541474497 Cholestasis, benign recurrent intrahepatic, 1 (BRIC1) [MIM:243300]
ATP8B1 O43520 VAR_043069 p.Gly733Arg LP/P rs1350369369 Cholestasis, progressive familial intrahepatic, 1 (PFIC1) [MIM:211600]

ATP8B1 O43520 VAR_043070 p.Lys814Asn LB/B rs34018300 -
ATP8B1 O43520 VAR_043071 p.Phe853Ser LP/P rs773092889 Cholestasis, progressive familial intrahepatic, 1 (PFIC1) [MIM:211600]
ATP8B1 O43520 VAR_043072 p.Arg867Cys LP/P rs121909103 Cholestasis of pregnancy, intrahepatic 1 (ICP1) [MIM:147480]
ATP8B1 O43520 VAR_043073 p.Gly1040Arg LP/P rs1438249656 Cholestasis, progressive familial intrahepatic, 1 (PFIC1) [MIM:211600]
ATP8B1 O43520 VAR_055045 p.Ala1152Thr LB/B rs222581 -
ATP8B1 O43520 VAR_071045 p.Pro209Thr LP/P rs515726138 Cholestasis, progressive familial intrahepatic, 1 (PFIC1) [MIM:211600]
ATP8B1 O43520 VAR_071046 p.Ser1012Ile LP/P - Cholestasis, progressive familial intrahepatic, 1 (PFIC1) [MIM:211600]
ATP8B3 O60423 VAR_055544 p.Gly45Arg LB/B rs7250872 -
ATP8B3 O60423 VAR_055545 p.Val618Ile LB/B rs8100856 -
ATP8B4 Q8TF62 VAR_046962 p.Asn225Ser LB/B rs16963151 -
ATP8B4 Q8TF62 VAR_046963 p.His452Asn LB/B rs2452524 -
ATP8B4 Q8TF62 VAR_046964 p.Asn1165Lys LB/B rs16962989 -
ATP8B4 Q8TF62 VAR_046965 p.Val1190Gly LB/B rs16962987 -
ATP9B O43861 VAR_047557 p.Ser39Gly LB/B rs4078115 -
ATP9B O43861 VAR_047558 p.Asp504Asn LB/B rs36034863 -
ATP9B O43861 VAR_047559 p.Met732Leu LB/B rs585033 -
ATP9B O43861 VAR_061037 p.Arg108Gln LB/B rs34938281 -
ATPAF1 Q5TC12 VAR_038906 p.Ser62Gly LB/B rs11211337 -
ATPAF2 Q8N5M1 VAR_023386 p.Trp94Arg LP/P rs104894554 Mitochondrial complex V deficiency, nuclear type 1 (MC5DN1) [MIM:604273]
ATPSCKMT Q6P4H8 VAR_039345 p.Thr75Met LB/B rs2438652 -
ATPSCKMT Q6P4H8 VAR_039346 p.Ala105Val LB/B rs16884350 -
ATPSCKMT Q6P4H8 VAR_039347 p.Val114Ala LB/B rs17360625 -
ATPSCKMT Q6P4H8 VAR_039348 p.Leu229Met LB/B rs15757 -
ATR Q13535 VAR_041584 p.Thr64Ala LB/B rs35306038 -
ATR Q13535 VAR_041585 p.His90Tyr LB/B rs28897763 -
ATR Q13535 VAR_041586 p.Lys297Asn LB/B rs2229033 -
ATR Q13535 VAR_041587 p.Val316Ile LB/B rs28897764 -
ATR Q13535 VAR_041588 p.Val959Met LB/B rs28910271 -
ATR Q13535 VAR_041589 p.Tyr1087His LB/B rs34253059 -
ATR Q13535 VAR_041590 p.Ser1213Gly LB/B rs34766606 -
ATR Q13535 VAR_041591 p.Ala1488Pro US - A lung squamous cell carcinoma sample
ATR Q13535 VAR_041592 p.Ser1607Asn LB/B rs55724025 -
ATR Q13535 VAR_041593 p.Asn1612Ser LB/B rs55894265 -
ATR Q13535 VAR_041594 p.Ala2002Gly US - A lung adenocarcinoma sample
ATR Q13535 VAR_041595 p.Gly2120Ala LB/B rs35134774 -
ATR Q13535 VAR_041596 p.Tyr2132Asp LB/B rs28910273 -
ATR Q13535 VAR_041597 p.Ser2233Ile US - A lung large cell carcinoma sample
ATR Q13535 VAR_041598 p.Arg2425Gln LB/B rs2229032 -
ATR Q13535 VAR_041599 p.Pro2434Ala LB/B rs33972295 -
ATR Q13535 VAR_041600 p.Glu2438Lys US - A breast pleomorphic lobular carcinoma sample
ATR Q13535 VAR_041601 p.Glu2537Gln US - A breast infiltrating ductal carcinoma sample
ATR Q13535 VAR_050532 p.Met211Thr LB/B rs2227928 -
ATR Q13535 VAR_050533 p.Ile1526Val LB/B rs34124242 -

ATR Q13535 VAR_067919 p.Gln2144Arg LP/P rs387906797 Cutaneous telangiectasia and cancer syndrome, familial (FCTCS) [MIM:614564]
ATRAID Q6UW56 VAR_057991 p.Ala209Ser LB/B rs7437 -
ATRIP Q8WXE1 VAR_050683 p.Lys125Gln LB/B rs11925638 -
ATRIP Q8WXE1 VAR_050684 p.Pro240Leu LB/B rs35240314 -
ATRN O75882 VAR_048967 p.Asp303Ala LB/B rs6107308 -
ATRN O75882 VAR_048968 p.Ile426Thr LB/B rs17782078 -
ATRN O75882 VAR_048969 p.Arg1152Lys LB/B rs3886999 -
ATRN O75882 VAR_048970 p.Val1226Ile LB/B rs12329487 -
ATRNL1 Q5VV63 VAR_043446 p.Ser989Asn LB/B rs1953758 -
ATRX P46100 VAR_001226 p.Pro190Ala LP/P rs122445103 Alpha-thalassemia mental retardation syndrome, X-linked (ATRX) [MIM:301040]
ATRX P46100 VAR_001227 p.Leu192Phe LP/P - Alpha-thalassemia mental retardation syndrome, X-linked (ATRX) [MIM:301040]
ATRX P46100 VAR_001228 p.Cys200Ser LP/P - Alpha-thalassemia mental retardation syndrome, X-linked (ATRX) [MIM:301040]
ATRX P46100 VAR_001229 p.Cys220Arg LP/P - Alpha-thalassemia mental retardation syndrome, X-linked (ATRX) [MIM:301040]
ATRX P46100 VAR_001230 p.Trp222Ser LP/P - Alpha-thalassemia mental retardation syndrome, X-linked (ATRX) [MIM:301040]
ATRX P46100 VAR_001231 p.Cys243Phe LP/P - Alpha-thalassemia mental retardation syndrome, X-linked (ATRX) [MIM:301040]
ATRX P46100 VAR_001232 p.Arg246Cys LP/P rs122445105 Alpha-thalassemia mental retardation syndrome, X-linked (ATRX) [MIM:301040]
ATRX P46100 VAR_001233 p.Gly249Asp LP/P - Alpha-thalassemia mental retardation syndrome, X-linked (ATRX) [MIM:301040]
ATRX P46100 VAR_001234 p.His1609Arg LP/P rs122445093 Alpha-thalassemia mental retardation syndrome, X-linked (ATRX) [MIM:301040]
ATRX P46100 VAR_001235 p.Cys1614Arg LP/P rs122445094 Alpha-thalassemia mental retardation syndrome, X-linked (ATRX) [MIM:301040]
ATRX P46100 VAR_001236 p.Lys1650Asn LP/P rs122445095 Alpha-thalassemia mental retardation syndrome, X-linked (ATRX) [MIM:301040]
ATRX P46100 VAR_001237 p.Asn1860Ser LB/B rs45439799 -
ATRX P46100 VAR_001238 p.Asp2035Val LP/P rs122445096 Alpha-thalassemia mental retardation syndrome, X-linked (ATRX) [MIM:301040]
ATRX P46100 VAR_001239 p.Tyr2084His LP/P rs122445097 Alpha-thalassemia mental retardation syndrome, X-linked (ATRX) [MIM:301040]
ATRX P46100 VAR_001240 p.Arg2131Gln LP/P rs122445101 Mental retardation-hypotonic facies syndrome, X-linked, 1 (MRXHF1) [MIM:309580]
ATRX P46100 VAR_001241 p.Tyr2163Cys LP/P rs122445098 Alpha-thalassemia mental retardation syndrome, X-linked (ATRX) [MIM:301040]
ATRX P46100 VAR_010914 p.Arg246Leu LP/P - Alpha-thalassemia mental retardation syndrome, X-linked (ATRX) [MIM:301040]
ATRX P46100 VAR_012113 p.Gly175Glu LP/P - Alpha-thalassemia mental retardation syndrome, X-linked (ATRX) [MIM:301040]
ATRX P46100 VAR_012115 p.Asn179Ser LP/P rs398123425 Alpha-thalassemia mental retardation syndrome, X-linked (ATRX) [MIM:301040]
ATRX P46100 VAR_012116 p.Pro190Leu LP/P rs1057518708 Alpha-thalassemia mental retardation syndrome, X-linked (ATRX) [MIM:301040]

ATRX P46100 VAR_012117 p.Pro190Ser LP/P - Alpha-thalassemia mental retardation syndrome, X-linked (ATRX) [MIM:301040]
ATRX P46100 VAR_012118 p.Val194Ile LP/P - Alpha-thalassemia mental retardation syndrome, X-linked (ATRX) [MIM:301040]
ATRX P46100 VAR_012119 p.Gln219Pro LP/P - Alpha-thalassemia mental retardation syndrome, X-linked (ATRX) [MIM:301040]
ATRX P46100 VAR_012120 p.Gly249Cys LP/P - Alpha-thalassemia mental retardation syndrome, X-linked (ATRX) [MIM:301040]
ATRX P46100 VAR_012121 p.Val1538Gly US - Alpha-thalassemia mental retardation syndrome, X-linked (ATRX) [MIM:301040]
ATRX P46100 VAR_012122 p.Val1552Phe LP/P - Alpha-thalassemia mental retardation syndrome, X-linked (ATRX) [MIM:301040]
ATRX P46100 VAR_012123 p.Leu1645Ser LP/P - Alpha-thalassemia mental retardation syndrome, X-linked (ATRX) [MIM:301040]
ATRX P46100 VAR_012124 p.Pro1713Ser LP/P - Alpha-thalassemia mental retardation syndrome, X-linked (ATRX) [MIM:301040]
ATRX P46100 VAR_012125 p.Arg1742Lys LP/P rs122445104 Alpha-thalassemia mental retardation syndrome, X-linked (ATRX) [MIM:301040]
ATRX P46100 VAR_012126 p.Tyr1847Cys LP/P - Alpha-thalassemia mental retardation syndrome, X-linked (ATRX) [MIM:301040]
ATRX P46100 VAR_012127 p.Ile2050Thr LP/P rs122445110 Mental retardation-hypotonic facies syndrome, X-linked, 1 (MRXHF1) [MIM:309580]
ATRX P46100 VAR_016914 p.Ser596Pro LB/B rs1051678 -
ATRX P46100 VAR_016915 p.Glu740Gly LB/B rs1051680 -
ATRX P46100 VAR_016916 p.Thr1621Met LP/P rs122445106 Alpha-thalassemia mental retardation syndrome, X-linked (ATRX) [MIM:301040]
ATRX P46100 VAR_023438 p.Gln929Glu LB/B rs3088074 -
ATRX P46100 VAR_032625 p.Cys220Tyr LP/P rs122445111 Mental retardation-hypotonic facies syndrome, X-linked, 1 (MRXHF1) [MIM:309580]
ATRX P46100 VAR_032626 p.Leu409Ser LP/P rs122445109 Mental retardation-hypotonic facies syndrome, X-linked, 1 (MRXHF1) [MIM:309580]
ATRX P46100 VAR_032627 p.Arg2271Gly LP/P rs122445112 Mental retardation-hypotonic facies syndrome, X-linked, 1 (MRXHF1) [MIM:309580]
ATRX P46100 VAR_055939 p.Gln545Glu LB/B rs35738915 -
ATXN1 P54253 VAR_046616 p.His209Gln LB/B rs11969612 -
ATXN1 P54253 VAR_046617 p.Pro753Ser LB/B rs16885 -
ATXN1L P0C7T5 VAR_044496 p.Ser313Pro LB/B rs7194407 -
ATXN2 Q99700 VAR_047629 p.Leu107Val LB/B rs695871 -
ATXN2 Q99700 VAR_047630 p.Ser248Asn LB/B rs7969300 -
ATXN3 P54252 VAR_013688 p.Val212Met LB/B rs1048755 -
ATXN3L Q9H3M9 VAR_029861 p.Leu266Phe LB/B rs16999010 -
ATXN3L Q9H3M9 VAR_029862 p.Gly332Asp LB/B rs4830842 -
ATXN7 O15265 VAR_011823 p.Lys264Arg LB/B rs1053338 -
ATXN7 O15265 VAR_011824 p.Pro663Ser LB/B rs1053340 -
ATXN7 O15265 VAR_020143 p.Val862Met LB/B rs3774729 -
ATXN7 O15265 VAR_053779 p.Ile573Val LB/B rs3733124 -
ATXN7L2 Q5T6C5 VAR_053780 p.Pro495Ser LB/B rs1149172 -
AUH Q13825 VAR_016911 p.Ala240Val LP/P rs769894315 3-methylglutaconic aciduria 1 (MGCA1) [MIM:250950]

AUNIP Q9H7T9 VAR_031776 p.Lys82Thr LB/B rs34449716 -
AURKA O14965 VAR_030840 p.Gly11Arg LB/B rs6069717 -
AURKA O14965 VAR_030841 p.Phe31Ile LB/B rs2273535 -
AURKA O14965 VAR_030842 p.Val57Ile LB/B rs1047972 -
AURKA O14965 VAR_041127 p.Pro50Leu LB/B rs34572020 -
AURKA O14965 VAR_041128 p.Ser155Arg US - A colorectal adenocarcinoma sample
AURKA O14965 VAR_041129 p.Val174Met US - A metastatic melanoma sample
AURKA O14965 VAR_041130 p.Met373Val LB/B rs33923703 -
AURKA O14965 VAR_061745 p.Ser104Leu LB/B rs2230743 -
AURKAIP1 Q9NWT8 VAR_020132 p.Gln107His LB/B rs3736374 -
AURKB Q96GD4 VAR_027970 p.His100Gln LB/B rs3027254 -
AURKB Q96GD4 VAR_027971 p.Met298Thr LB/B rs1059476 -
AURKB Q96GD4 VAR_040383 p.Ala52Val LB/B rs55878091 -
AURKB Q96GD4 VAR_040384 p.Thr179Met LB/B rs55871613 -
AURKC Q9UQB9 VAR_040385 p.Gly52Glu US - A lung adenocarcinoma sample
AURKC Q9UQB9 VAR_040386 p.Glu148Gln US - A lung squamous cell carcinoma sample
AURKC Q9UQB9 VAR_040387 p.His244Gln US - A lung adenocarcinoma sample
AUTS2 Q8WXX7 VAR_013864 p.Ala303Ser LB/B rs2293507 -
AVEN Q9NQS1 VAR_020144 p.Gln228Arg LB/B rs2241647 -
AVIL O75366 VAR_054974 p.Lys204Glu LB/B rs2172521 -
AVIL O75366 VAR_083229 p.Arg135Gln LP/P rs138047529 Nephrotic syndrome 21 (NPHS21) [MIM:618594]
AVIL O75366 VAR_083230 p.Leu425Met LP/P rs763782471 Nephrotic syndrome 21 (NPHS21) [MIM:618594]
AVIL O75366 VAR_083231 p.Arg446His US rs1334894971 Nephrotic syndrome 21 (NPHS21) [MIM:618594]
AVL9 Q8NBF6 VAR_027083 p.Cys257Ser LB/B rs2290213 -
AVP P01185 VAR_004980 p.Ser17Phe LP/P - Diabetes insipidus, neurohypophyseal (NDI)
[MIM:125700]
AVP P01185 VAR_004981 p.Ala19Thr LP/P rs387906511 Diabetes insipidus, neurohypophyseal (NDI)
[MIM:125700]
AVP P01185 VAR_004982 p.Ala19Val LP/P rs387906512 Diabetes insipidus, neurohypophyseal (NDI)
[MIM:125700]
AVP P01185 VAR_004983 p.Gly45Arg LP/P - Diabetes insipidus, neurohypophyseal (NDI) [MIM:125700]
AVP P01185 VAR_004984 p.Gly48Val LP/P rs121964883 Diabetes insipidus, neurohypophyseal (NDI)
[MIM:125700]
AVP P01185 VAR_004985 p.Arg51Cys LP/P - Diabetes insipidus, neurohypophyseal (NDI)
[MIM:125700]
AVP P01185 VAR_004986 p.Pro55Leu LP/P - Diabetes insipidus, neurohypophyseal (NDI)
[MIM:125700]
AVP P01185 VAR_004988 p.Glu78Gly LP/P - Diabetes insipidus, neurohypophyseal (NDI) [MIM:125700]
AVP P01185 VAR_004989 p.Leu81Pro LP/P - Diabetes insipidus, neurohypophyseal (NDI)
[MIM:125700]
AVP P01185 VAR_004990 p.Gly88Arg LP/P - Diabetes insipidus, neurohypophyseal (NDI) [MIM:125700]
AVP P01185 VAR_004991 p.Gly88Ser LP/P rs121964882 Diabetes insipidus, neurohypophyseal (NDI)
[MIM:125700]
AVP P01185 VAR_004992 p.Cys92Ser LP/P - Diabetes insipidus, neurohypophyseal (NDI) [MIM:125700]
AVP P01185 VAR_004993 p.Gly93Trp LP/P rs121964885 Diabetes insipidus, neurohypophyseal (NDI)
[MIM:125700]
AVP P01185 VAR_004994 p.Gly96Cys LP/P - Diabetes insipidus, neurohypophyseal (NDI) [MIM:125700]
AVP P01185 VAR_011894 p.Pro82Leu LB/B rs5195 -
AVP P01185 VAR_011895 p.Gly119Val LB/B rs1051744 -

AVP	P01185	VAR_015262	p.Tyr21His	LP/P	rs121964893	Diabetes insipidus, neurohypophyseal (NDI) [MIM:125700]
AVP	P01185	VAR_015263	p.Pro26Leu	LP/P	rs142886338	Diabetes insipidus, neurohypophyseal (NDI) [MIM:125700]
AVP	P01185	VAR_015264	p.Cys52Arg	LP/P	-	Diabetes insipidus, neurohypophyseal (NDI) [MIM:125700]
AVP	P01185	VAR_015265	p.Gly54Arg	LP/P	rs121964888	Diabetes insipidus, neurohypophyseal (NDI) [MIM:125700]
AVP	P01185	VAR_015266	p.Gly54Val	LP/P	rs121964887	Diabetes insipidus, neurohypophyseal (NDI) [MIM:125700]
AVP	P01185	VAR_015267	p.Cys59Arg	LP/P	-	Diabetes insipidus, neurohypophyseal (NDI) [MIM:125700]
AVP	P01185	VAR_015268	p.Cys59Tyr	LP/P	-	Diabetes insipidus, neurohypophyseal (NDI) [MIM:125700]
AVP	P01185	VAR_015269	p.Ser87Phe	LP/P	rs121964890	Diabetes insipidus, neurohypophyseal (NDI) [MIM:125700]
AVP	P01185	VAR_015270	p.Cys92Tyr	LP/P	rs121964891	Diabetes insipidus, neurohypophyseal (NDI) [MIM:125700]
AVP	P01185	VAR_015271	p.Gly96Val	LP/P	rs121964886	Diabetes insipidus, neurohypophyseal (NDI) [MIM:125700]
AVP	P01185	VAR_015272	p.Arg97Cys	LP/P	-	Diabetes insipidus, neurohypophyseal (NDI) [MIM:125700]
AVP	P01185	VAR_015273	p.Arg97Pro	LP/P	-	Diabetes insipidus, neurohypophyseal (NDI) [MIM:125700]
AVP	P01185	VAR_015274	p.Cys98Gly	LP/P	-	Diabetes insipidus, neurohypophyseal (NDI) [MIM:125700]
AVP	P01185	VAR_015275	p.Cys104Phe	LP/P	-	Diabetes insipidus, neurohypophyseal (NDI) [MIM:125700]
AVP	P01185	VAR_015276	p.Cys105Arg	LP/P	-	Diabetes insipidus, neurohypophyseal (NDI) [MIM:125700]
AVP	P01185	VAR_015277	p.Cys116Gly	LP/P	rs74315383	Diabetes insipidus, neurohypophyseal (NDI) [MIM:125700]
AVP	P01185	VAR_015278	p.Cys116Arg	LP/P	-	Diabetes insipidus, neurohypophyseal (NDI) [MIM:125700]
AVP	P01185	VAR_015279	p.Cys105Tyr	LP/P	-	Diabetes insipidus, neurohypophyseal (NDI) [MIM:125700]
AVP	P01185	VAR_019273	p.Val67Ala	LP/P	rs28934878	Diabetes insipidus, neurohypophyseal (NDI) [MIM:125700]
AVP	P01185	VAR_019274	p.Gly96Asp	LP/P	-	Diabetes insipidus, neurohypophyseal (NDI) [MIM:125700]
AVP	P01185	VAR_019275	p.Cys104Gly	LP/P	-	Diabetes insipidus, neurohypophyseal (NDI) [MIM:125700]
AVP	P01185	VAR_019276	p.Cys116Trp	LP/P	-	Diabetes insipidus, neurohypophyseal (NDI) [MIM:125700]
AVP	P01185	VAR_029997	p.Cys58Phe	LP/P	-	Diabetes insipidus, neurohypophyseal (NDI) [MIM:125700]
AVP	P01185	VAR_029998	p.Cys98Ser	LP/P	-	Diabetes insipidus, neurohypophyseal (NDI) [MIM:125700]
AVP	P01185	VAR_029999	p.Ala99Pro	LP/P	-	Diabetes insipidus, neurohypophyseal (NDI) [MIM:125700]
AVPI1	Q5T686	VAR_031405	p.Ala41Gly	LB/B	rs2275047	-
AVPR1A	P37288	VAR_022065	p.Gly6Ser	LB/B	rs2228154	-
AVPR1B	P47901	VAR_025159	p.Lys65Asn	LB/B	rs35369693	-

AVPR1B	P47901	VAR_025160	p.Gly191Arg	LB/B	rs33990840	-
AVPR1B	P47901	VAR_025161	p.Ser267Gly	LB/B	rs36030374	-
AVPR1B	P47901	VAR_025162	p.Arg364His	LB/B	rs28632197	-
AVPR1B	P47901	VAR_061228	p.His224Gln	LB/B	rs3891058	-
AVPR2	P30518	VAR_003516	p.Thr7Ser	LB/B	rs5196	-
AVPR2	P30518	VAR_003517	p.Leu44Pro	LP/P	-	Diabetes insipidus, nephrogenic, X-linked (XNDI) [MIM:304800]
AVPR2	P30518	VAR_003519	p.Arg64Trp	LB/B	rs150351033	-
AVPR2	P30518	VAR_003520	p.His80Arg	LP/P	-	Diabetes insipidus, nephrogenic, X-linked (XNDI) [MIM:304800]
AVPR2	P30518	VAR_003521	p.Val88Met	LP/P	-	Diabetes insipidus, nephrogenic, X-linked (XNDI) [MIM:304800]
AVPR2	P30518	VAR_003522	p.Arg106Cys	LP/P	-	Diabetes insipidus, nephrogenic, X-linked (XNDI) [MIM:304800]
AVPR2	P30518	VAR_003523	p.Cys112Arg	LP/P	-	Diabetes insipidus, nephrogenic, X-linked (XNDI) [MIM:304800]
AVPR2	P30518	VAR_003524	p.Arg113Trp	LP/P	rs28935496	Diabetes insipidus, nephrogenic, X-linked (XNDI) [MIM:304800]
AVPR2	P30518	VAR_003525	p.Ser126Phe	LP/P	-	Diabetes insipidus, nephrogenic, X-linked (XNDI) [MIM:304800]
AVPR2	P30518	VAR_003526	p.Tyr128Ser	LP/P	-	Diabetes insipidus, nephrogenic, X-linked (XNDI) [MIM:304800]
AVPR2	P30518	VAR_003527	p.Ala132Asp	LP/P	rs104894747	Diabetes insipidus, nephrogenic, X-linked (XNDI) [MIM:304800]
AVPR2	P30518	VAR_003528	p.Arg137His	LP/P	rs104894756	Diabetes insipidus, nephrogenic, X-linked (XNDI) [MIM:304800]
AVPR2	P30518	VAR_003529	p.Arg143Pro	LP/P	-	Diabetes insipidus, nephrogenic, X-linked (XNDI) [MIM:304800]
AVPR2	P30518	VAR_003530	p.Ala147Val	LB/B	rs5200	-
AVPR2	P30518	VAR_003531	p.Trp164Ser	LP/P	-	Diabetes insipidus, nephrogenic, X-linked (XNDI) [MIM:304800]
AVPR2	P30518	VAR_003532	p.Ser167Leu	LP/P	-	Diabetes insipidus, nephrogenic, X-linked (XNDI) [MIM:304800]
AVPR2	P30518	VAR_003533	p.Ser167Thr	LP/P	-	Diabetes insipidus, nephrogenic, X-linked (XNDI) [MIM:304800]
AVPR2	P30518	VAR_003534	p.Arg181Cys	LP/P	rs104894757	Diabetes insipidus, nephrogenic, X-linked (XNDI) [MIM:304800]
AVPR2	P30518	VAR_003535	p.Gly185Cys	LP/P	rs104894748	Diabetes insipidus, nephrogenic, X-linked (XNDI) [MIM:304800]
AVPR2	P30518	VAR_003536	p.Arg202Cys	LP/P	rs782806507	Diabetes insipidus, nephrogenic, X-linked (XNDI) [MIM:304800]
AVPR2	P30518	VAR_003537	p.Arg203Cys	LP/P	rs104894750	Diabetes insipidus, nephrogenic, X-linked (XNDI) [MIM:304800]
AVPR2	P30518	VAR_003538	p.Tyr205Cys	LP/P	rs104894749	Diabetes insipidus, nephrogenic, X-linked (XNDI) [MIM:304800]
AVPR2	P30518	VAR_003541	p.Tyr280Cys	LP/P	rs104894752	Diabetes insipidus, nephrogenic, X-linked (XNDI) [MIM:304800]
AVPR2	P30518	VAR_003542	p.Ala285Pro	LP/P	rs193922122	Diabetes insipidus, nephrogenic, X-linked (XNDI) [MIM:304800]

AVPR2 P30518 VAR_003543 p.Pro286Leu LP/P rs1557100917 Diabetes insipidus, nephrogenic, X-linked (XNDI) [MIM:304800]
AVPR2 P30518 VAR_003544 p.Pro286Arg LP/P - Diabetes insipidus, nephrogenic, X-linked (XNDI) [MIM:304800]
AVPR2 P30518 VAR_003545 p.Leu292Pro LP/P - Diabetes insipidus, nephrogenic, X-linked (XNDI) [MIM:304800]
AVPR2 P30518 VAR_003546 p.Leu309Pro LP/P - Diabetes insipidus, nephrogenic, X-linked (XNDI) [MIM:304800]
AVPR2 P30518 VAR_003547 p.Asn317Lys LP/P - Diabetes insipidus, nephrogenic, X-linked (XNDI) [MIM:304800]
AVPR2 P30518 VAR_003548 p.Trp323Ser LP/P - Diabetes insipidus, nephrogenic, X-linked (XNDI) [MIM:304800]
AVPR2 P30518 VAR_011858 p.Ala42Val LB/B rs5198 -
AVPR2 P30518 VAR_015296 p.Gly12Glu LB/B rs2071126 -
AVPR2 P30518 VAR_015297 p.Leu43Pro LP/P - Diabetes insipidus, nephrogenic, X-linked (XNDI) [MIM:304800]
AVPR2 P30518 VAR_015298 p.Ile46Lys LP/P rs104894759 Diabetes insipidus, nephrogenic, X-linked (XNDI) [MIM:304800]
AVPR2 P30518 VAR_015299 p.Leu53Arg LP/P - Diabetes insipidus, nephrogenic, X-linked (XNDI) [MIM:304800]
AVPR2 P30518 VAR_015300 p.Asn55Asp LP/P - Diabetes insipidus, nephrogenic, X-linked (XNDI) [MIM:304800]
AVPR2 P30518 VAR_015301 p.Asn55His LP/P - Diabetes insipidus, nephrogenic, X-linked (XNDI) [MIM:304800]
AVPR2 P30518 VAR_015302 p.Leu59Pro LP/P rs193922112 Diabetes insipidus, nephrogenic, X-linked (XNDI) [MIM:304800]
AVPR2 P30518 VAR_015303 p.Ala61Val LB/B - -
AVPR2 P30518 VAR_015304 p.Leu62Pro LP/P - Diabetes insipidus, nephrogenic, X-linked (XNDI) [MIM:304800]
AVPR2 P30518 VAR_015305 p.Leu81Phe LP/P - Diabetes insipidus, nephrogenic, X-linked (XNDI) [MIM:304800]
AVPR2 P30518 VAR_015306 p.Leu83Pro LP/P - Diabetes insipidus, nephrogenic, X-linked (XNDI) [MIM:304800]
AVPR2 P30518 VAR_015307 p.Leu83Gln LP/P - Diabetes insipidus, nephrogenic, X-linked (XNDI) [MIM:304800]
AVPR2 P30518 VAR_015308 p.Ala84Asp LP/P - Diabetes insipidus, nephrogenic, X-linked (XNDI) [MIM:304800]
AVPR2 P30518 VAR_015309 p.Asp85Asn LP/P rs104894754 Diabetes insipidus, nephrogenic, X-linked (XNDI) [MIM:304800]
AVPR2 P30518 VAR_015310 p.Gln92Arg LP/P - Diabetes insipidus, nephrogenic, X-linked (XNDI) [MIM:304800]
AVPR2 P30518 VAR_015311 p.Leu94Gln LP/P - Diabetes insipidus, nephrogenic, X-linked (XNDI) [MIM:304800]
AVPR2 P30518 VAR_015312 p.Pro95Leu LP/P - Diabetes insipidus, nephrogenic, X-linked (XNDI) [MIM:304800]
AVPR2 P30518 VAR_015313 p.Trp99Arg LP/P - Diabetes insipidus, nephrogenic, X-linked (XNDI) [MIM:304800]
AVPR2 P30518 VAR_015314 p.Arg104Cys LP/P rs104894760 Diabetes insipidus, nephrogenic, X-linked (XNDI) [MIM:304800]

AVPR2 P30518 VAR_015315 p.Phe105Val LP/P rs104894758 Diabetes insipidus, nephrogenic, X-linked (XNDI) [MIM:304800]
AVPR2 P30518 VAR_015316 p.Gly107Glu LP/P - Diabetes insipidus, nephrogenic, X-linked (XNDI) [MIM:304800]
AVPR2 P30518 VAR_015317 p.Cys112Tyr LP/P - Diabetes insipidus, nephrogenic, X-linked (XNDI) [MIM:304800]
AVPR2 P30518 VAR_015318 p.Gly122Arg LP/P - Diabetes insipidus, nephrogenic, X-linked (XNDI) [MIM:304800]
AVPR2 P30518 VAR_015319 p.Met123Lys LP/P - Diabetes insipidus, nephrogenic, X-linked (XNDI) [MIM:304800]
AVPR2 P30518 VAR_015320 p.Ser127Phe LP/P - Diabetes insipidus, nephrogenic, X-linked (XNDI) [MIM:304800]
AVPR2 P30518 VAR_015321 p.Ile130Phe LP/P rs796052096 Diabetes insipidus, nephrogenic, X-linked (XNDI) [MIM:304800]
AVPR2 P30518 VAR_015322 p.Leu135Pro LP/P rs1557100610 Diabetes insipidus, nephrogenic, X-linked (XNDI) [MIM:304800]
AVPR2 P30518 VAR_015323 p.Arg139Ser LB/B - -
AVPR2 P30518 VAR_015324 p.Ala163Pro LP/P - Diabetes insipidus, nephrogenic, X-linked (XNDI) [MIM:304800]
AVPR2 P30518 VAR_015325 p.Pro173Ser LP/P - Diabetes insipidus, nephrogenic, X-linked (XNDI) [MIM:304800]
AVPR2 P30518 VAR_015326 p.Gln174Leu LP/P - Diabetes insipidus, nephrogenic, X-linked (XNDI) [MIM:304800]
AVPR2 P30518 VAR_015327 p.Asp191Gly LP/P - Diabetes insipidus, nephrogenic, X-linked (XNDI) [MIM:304800]
AVPR2 P30518 VAR_015328 p.Gly201Asp LP/P rs104894755 Diabetes insipidus, nephrogenic, X-linked (XNDI) [MIM:304800]
AVPR2 P30518 VAR_015329 p.Thr204Asn LP/P - Diabetes insipidus, nephrogenic, X-linked (XNDI) [MIM:304800]
AVPR2 P30518 VAR_015330 p.Val206Asp LP/P - Diabetes insipidus, nephrogenic, X-linked (XNDI) [MIM:304800]
AVPR2 P30518 VAR_015331 p.Thr207Asn LP/P - Diabetes insipidus, nephrogenic, X-linked (XNDI) [MIM:304800]
AVPR2 P30518 VAR_015332 p.Ile209Phe LP/P - Diabetes insipidus, nephrogenic, X-linked (XNDI) [MIM:304800]
AVPR2 P30518 VAR_015333 p.Phe214Ser LP/P - Diabetes insipidus, nephrogenic, X-linked (XNDI) [MIM:304800]
AVPR2 P30518 VAR_015334 p.Val215Met LB/B rs112109182 -
AVPR2 P30518 VAR_015335 p.Pro217Thr LP/P - Diabetes insipidus, nephrogenic, X-linked (XNDI) [MIM:304800]
AVPR2 P30518 VAR_015336 p.Leu219Pro LP/P - Diabetes insipidus, nephrogenic, X-linked (XNDI) [MIM:304800]
AVPR2 P30518 VAR_015337 p.Leu219Arg LP/P - Diabetes insipidus, nephrogenic, X-linked (XNDI) [MIM:304800]
AVPR2 P30518 VAR_015338 p.Arg252Trp LB/B rs61733407 -
AVPR2 P30518 VAR_015339 p.Met272Lys LP/P - Diabetes insipidus, nephrogenic, X-linked (XNDI) [MIM:304800]
AVPR2 P30518 VAR_015340 p.Val277Ala LP/P - Diabetes insipidus, nephrogenic, X-linked (XNDI) [MIM:304800]

AVPR2 P30518 VAR_015341 p.Leu282Pro LP/P - Diabetes insipidus, nephrogenic, X-linked (XNDI) [MIM:304800]
AVPR2 P30518 VAR_015342 p.Pro286Ser LP/P - Diabetes insipidus, nephrogenic, X-linked (XNDI) [MIM:304800]
AVPR2 P30518 VAR_015343 p.Phe287Leu LP/P - Diabetes insipidus, nephrogenic, X-linked (XNDI) [MIM:304800]
AVPR2 P30518 VAR_015344 p.Leu289Pro LP/P - Diabetes insipidus, nephrogenic, X-linked (XNDI) [MIM:304800]
AVPR2 P30518 VAR_015345 p.Ala294Pro LP/P - Diabetes insipidus, nephrogenic, X-linked (XNDI) [MIM:304800]
AVPR2 P30518 VAR_015346 p.Leu309Arg LP/P - Diabetes insipidus, nephrogenic, X-linked (XNDI) [MIM:304800]
AVPR2 P30518 VAR_015347 p.Ser315Arg LP/P - Diabetes insipidus, nephrogenic, X-linked (XNDI) [MIM:304800]
AVPR2 P30518 VAR_015348 p.Ser318Thr LB/B - -
AVPR2 P30518 VAR_015349 p.Cys319Arg LP/P - Diabetes insipidus, nephrogenic, X-linked (XNDI) [MIM:304800]
AVPR2 P30518 VAR_015350 p.Asn321Asp LP/P - Diabetes insipidus, nephrogenic, X-linked (XNDI) [MIM:304800]
AVPR2 P30518 VAR_015351 p.Asn321Lys LP/P rs193922123 Diabetes insipidus, nephrogenic, X-linked (XNDI) [MIM:304800]
AVPR2 P30518 VAR_015352 p.Asn321Tyr LP/P - Diabetes insipidus, nephrogenic, X-linked (XNDI) [MIM:304800]
AVPR2 P30518 VAR_015353 p.Pro322His LP/P - Diabetes insipidus, nephrogenic, X-linked (XNDI) [MIM:304800]
AVPR2 P30518 VAR_015354 p.Pro322Ser LP/P - Diabetes insipidus, nephrogenic, X-linked (XNDI) [MIM:304800]
AVPR2 P30518 VAR_015355 p.Trp323Arg LP/P - Diabetes insipidus, nephrogenic, X-linked (XNDI) [MIM:304800]
AVPR2 P30518 VAR_015356 p.Gly352Asp LB/B rs146350208 -
AVPR2 P30518 VAR_025901 p.Arg137Cys LP/P rs104894761 Nephrogenic syndrome of inappropriate antidiuresis (NSIAD) [MIM:300539]
AVPR2 P30518 VAR_025902 p.Arg137Leu LP/P rs104894756 Nephrogenic syndrome of inappropriate antidiuresis (NSIAD) [MIM:300539]
AVPR2 P30518 VAR_035769 p.Arg247His US rs149668713 A breast cancer sample
AVPR2 P30518 VAR_062591 p.Gly122Asp LP/P - Diabetes insipidus, nephrogenic, X-linked (XNDI) [MIM:304800]
AXDND1 Q5T1B0 VAR_031841 p.Ala742Val LB/B rs17369441 -
AXDND1 Q5T1B0 VAR_031842 p.Glu991Gln LB/B rs6425573 -
AXDND1 Q5T1B0 VAR_050705 p.Ile522Leu LB/B rs11577579 -
AXDND1 Q5T1B0 VAR_050706 p.Gln993Glu LB/B rs6658180 -
AXIN1 O15169 VAR_015589 p.Leu106Arg LP/P - Hepatocellular carcinoma (HCC) [MIM:114550]
AXIN1 O15169 VAR_015590 p.Pro345Leu LP/P rs779951904 Hepatocellular carcinoma (HCC) [MIM:114550]
AXIN1 O15169 VAR_015591 p.Gly425Ser LP/P rs116350678 Hepatocellular carcinoma (HCC) [MIM:114550]
AXIN1 O15169 VAR_015592 p.Gly650Ser LP/P rs117208012 Hepatocellular carcinoma (HCC) [MIM:114550]
AXIN1 O15169 VAR_015593 p.Arg841Gln LB/B rs34015754 -
AXIN2 Q9Y2T1 VAR_054860 p.Ser50Pro LB/B rs2240308 -
AXL P30530 VAR_041877 p.Arg499Cys US rs747576071 A gastric adenocarcinoma sample
AXL P30530 VAR_041878 p.Ser515Gly LB/B rs1240393707 -

AXL P30530 VAR_045596 p.Thr112Met LB/B rs35202236 -
AXL P30530 VAR_045597 p.Arg295Trp US rs751738506 A lung neuroendocrine carcinoma sample
AXL P30530 VAR_057990 p.Asp266Asn LB/B rs7249222 -
AZIN2 Q96A70 VAR_050611 p.Ala288Ser LB/B rs16835244 -
B2M P61769 VAR_030660 p.Ala11Pro LP/P rs104894481 Immunodeficiency 43 (IMD43) [MIM:241600]
B2M P61769 VAR_076691 p.Asp96Asn LP/P rs398122820 Amyloidosis 8 (AMYL8) [MIM:105200]
B3GALNT1 O75752 VAR_019646 p.Glu266Ala LB/B rs28937582 -
B3GALNT1 O75752 VAR_019647 p.Gly271Arg LB/B rs104893683 -
B3GALNT1 O75752 VAR_025091 p.Asp126Asn LB/B rs2231257 -
B3GALNT2 Q8NCR0 VAR_035860 p.Asn203Ser US - A breast cancer sample
B3GALNT2 Q8NCR0 VAR_069638 p.Gly247Glu LP/P rs367543072 Muscular dystrophy-dystroglycanopathy congenital with brain and eye anomalies A11 (MDDGA11) [MIM:615181]
B3GALNT2 Q8NCR0 VAR_069639 p.Val252Gly LP/P rs367543073 Muscular dystrophy-dystroglycanopathy congenital with brain and eye anomalies A11 (MDDGA11) [MIM:615181]
B3GALNT2 Q8NCR0 VAR_069640 p.Val268Met LP/P rs367543074 Muscular dystrophy-dystroglycanopathy congenital with brain and eye anomalies A11 (MDDGA11) [MIM:615181]
B3GALNT2 Q8NCR0 VAR_069641 p.Arg292Pro LP/P rs367543076 Muscular dystrophy-dystroglycanopathy congenital with brain and eye anomalies A11 (MDDGA11) [MIM:615181]
B3GALT5 Q9Y2C3 VAR_020460 p.Met85Thr LB/B rs3746887 -
B3GALT5 Q9Y2C3 VAR_033536 p.Arg144His LB/B rs734411 -
B3GALT5 Q9Y2C3 VAR_049347 p.Ser27Arg LB/B rs12627708 -
B3GALT6 Q96L58 VAR_059317 p.Glu174Asp LB/B rs12085009 -
B3GALT6 Q96L58 VAR_070132 p.Arg6Trp LP/P rs397514722 Ehlers-Danlos syndrome, spondylodysplastic type, 2 (EDSSPD2) [MIM:615349]
B3GALT6 Q96L58 VAR_070133 p.Ser65Gly LP/P rs397514719 Spondyloepimetaphyseal dysplasia with joint laxity, 1, with or without fractures (SEMDJL1) [MIM:271640]
B3GALT6 Q96L58 VAR_070134 p.Pro67Leu LP/P rs397514720 Spondyloepimetaphyseal dysplasia with joint laxity, 1, with or without fractures (SEMDJL1) [MIM:271640]
B3GALT6 Q96L58 VAR_070136 p.Asp156Asn LP/P rs397514718 Spondyloepimetaphyseal dysplasia with joint laxity, 1, with or without fractures (SEMDJL1) [MIM:271640]
B3GALT6 Q96L58 VAR_070137 p.Asp207His LP/P rs397514723 Spondyloepimetaphyseal dysplasia with joint laxity, 1, with or without fractures (SEMDJL1) [MIM:271640]
B3GALT6 Q96L58 VAR_070138 p.Gly217Ser LP/P rs397514724 Spondyloepimetaphyseal dysplasia with joint laxity, 1, with or without fractures (SEMDJL1) [MIM:271640]
B3GALT6 Q96L58 VAR_070139 p.Arg232Cys LP/P rs397514717 Spondyloepimetaphyseal dysplasia with joint laxity, 1, with or without fractures (SEMDJL1) [MIM:271640]
B3GALT6 Q96L58 VAR_070140 p.Cys300Ser LP/P rs786200939 Spondyloepimetaphyseal dysplasia with joint laxity, 1, with or without fractures (SEMDJL1) [MIM:271640]
B3GALT6 Q96L58 VAR_070141 p.Ser309Thr LP/P rs397514721 Ehlers-Danlos syndrome, spondylodysplastic type, 2 (EDSSPD2) [MIM:615349]
B3GALT6 Q96L58 VAR_084154 p.Thr79Ala LP/P - Spondyloepimetaphyseal dysplasia with joint laxity, 1, with or without fractures (SEMDJL1) [MIM:271640]
B3GALT6 Q96L58 VAR_084155 p.Ser159Tyr US - Al-Gazali syndrome (ALGAZ) [MIM:609465]
B3GALT6 Q96L58 VAR_084156 p.Tyr182Cys US rs1314046622 Ehlers-Danlos syndrome, spondylodysplastic type, 2 (EDSSPD2) [MIM:615349]
B3GALT6 Q96L58 VAR_084157 p.Phe186Leu LP/P rs1553151294 Spondyloepimetaphyseal dysplasia with joint laxity, 1, with or without fractures (SEMDJL1) [MIM:271640]
B3GALT6 Q96L58 VAR_084158 p.Cys206Trp LP/P rs763080896 Al-Gazali syndrome (ALGAZ) [MIM:609465]

B3GALT6 Q96L58 VAR_084159 p.Arg256Trp LP/P - Spondyloepimetaphyseal dysplasia with joint laxity, 1, with or without fractures (SEMDJL1) [MIM:271640]
B3GALT6 Q96L58 VAR_084160 p.Glu265Asp US rs374677519 Al-Gazali syndrome (ALGAZ) [MIM:609465]
B3GAT1 Q9P2W7 VAR_044538 p.Ala131Thr LB/B rs35434644 -
B3GAT3 O94766 VAR_066624 p.Arg277Gln LP/P rs387906937 Multiple joint dislocations, short stature, and craniofacial dysmorphism with or without congenital heart defects (JDSCD) [MIM:245600]
B3GAT3 O94766 VAR_075370 p.Pro140Leu LP/P rs879255269 Multiple joint dislocations, short stature, and craniofacial dysmorphism with or without congenital heart defects (JDSCD) [MIM:245600]
B3GAT3 O94766 VAR_075371 p.Gly223Ser US rs372487178 Multiple joint dislocations, short stature, and craniofacial dysmorphism with or without congenital heart defects (JDSCD) [MIM:245600]
B3GLCT Q6Y288 VAR_027849 p.Glu370Lys LB/B rs1041073 -
B3GNT3 Q9Y2A9 VAR_022644 p.Arg328His LB/B rs36686 -
B3GNT3 Q9Y2A9 VAR_074178 p.Ile317Phe LB/B - -
B3GNT4 Q9C0J1 VAR_032600 p.Pro6Ala LB/B rs7136356 -
B3GNT4 Q9C0J1 VAR_032601 p.Ser83Thr LB/B rs1001178 -
B3GNT4 Q9C0J1 VAR_032602 p.Leu87Pro LB/B rs35203505 -
B3GNT7 Q8NFL0 VAR_029633 p.Val233Ile LB/B rs2290130 -
B3GNT8 Q7Z7M8 VAR_049348 p.Ser137Gly LB/B rs284662 -
B3GNTL1 Q67FW5 VAR_032603 p.Ala341Thr LB/B rs7225887 -
B4GALNT1 Q00973 VAR_012052 p.Leu35Val LB/B rs774896 -
B4GALNT1 Q00973 VAR_012053 p.Gly172Arg LB/B rs810205 -
B4GALNT1 Q00973 VAR_049237 p.Ala516Val LB/B rs17454674 -
B4GALNT1 Q00973 VAR_070235 p.Arg300Cys LP/P rs756710480 Spastic paraplegia 26, autosomal recessive (SPG26) [MIM:609195]
B4GALNT1 Q00973 VAR_070236 p.Asp433Ala LP/P rs879255242 Spastic paraplegia 26, autosomal recessive (SPG26) [MIM:609195]
B4GALNT2 Q8NHY0 VAR_035990 p.Pro459His US - A colorectal cancer sample
B4GALNT2 Q8NHY0 VAR_049238 p.Ala40Asp LB/B rs7207403 -
B4GALNT2 Q8NHY0 VAR_049239 p.Cys466Arg LB/B rs7224888 -
B4GALNT3 Q6L9W6 VAR_027842 p.Gly59Ser LB/B rs2075033 -
B4GALNT3 Q6L9W6 VAR_027843 p.Arg265Gln LB/B rs11063529 -
B4GALNT3 Q6L9W6 VAR_027844 p.Lys411Arg LB/B rs7298766 -
B4GALNT3 Q6L9W6 VAR_048717 p.Arg768Gln LB/B rs11063570 -
B4GALNT3 Q6L9W6 VAR_048718 p.Arg992His LB/B rs36078145 -
B4GALNT4 Q76KP1 VAR_061096 p.Ala67Val LB/B rs34063493 -
B4GALNT4 Q76KP1 VAR_061097 p.Gly88Val LB/B rs11822725 -
B4GALT1 P15291 VAR_054019 p.Arg21Trp LB/B rs1065764 -
B4GALT1 P15291 VAR_054020 p.His257Arg LB/B rs9169 -
B4GALT2 O60909 VAR_020487 p.Gln122His LB/B rs1859728 -
B4GALT2 O60909 VAR_054021 p.Gly338Arg LB/B rs35904809 -
B4GALT4 O60513 VAR_022697 p.Gln116Glu LB/B rs3764779 -
B4GALT5 O43286 VAR_024468 p.Gly61Ser LB/B rs2273086 -
B4GALT5 O43286 VAR_033538 p.Tyr371Asp LB/B rs35195217 -
B4GALT5 O43286 VAR_054022 p.Asp368Asn LB/B rs235035 -
B4GALT6 Q9UBX8 VAR_054023 p.Ile379Val LB/B rs34683195 -
B4GALT7 Q9UBV7 VAR_010293 p.Ala186Asp LP/P rs121917817 Ehlers-Danlos syndrome, spondylodysplastic type, 1 (EDSSPD1) [MIM:130070]
B4GALT7 Q9UBV7 VAR_010294 p.Leu206Pro LP/P rs121917818 Ehlers-Danlos syndrome, spondylodysplastic type, 1 (EDSSPD1) [MIM:130070]

B4GAT1 O43505 VAR_025019 p.Thr253Ser LB/B rs35429253 -
B4GAT1 O43505 VAR_069989 p.Asn390Asp LP/P rs397509397 Muscular dystrophy-dystroglycanopathy congenital with brain and eye anomalies A13 (MDDGA13) [MIM:615287]
B4GAT1 O43505 VAR_069990 p.Ala406Val LP/P rs397509396 Muscular dystrophy-dystroglycanopathy congenital with brain and eye anomalies A13 (MDDGA13) [MIM:615287]
B9D1 Q9UPM9 VAR_066995 p.Arg61Trp LB/B rs73980038 -
B9D1 Q9UPM9 VAR_075700 p.Ser51Pro LB/B rs546359789 -
B9D1 Q9UPM9 VAR_076974 p.Tyr32Cys LP/P rs771170000 Joubert syndrome 27 (JBTS27) [MIM:617120]
B9D1 Q9UPM9 VAR_076975 p.Arg156Gln LP/P rs886038205 Joubert syndrome 27 (JBTS27) [MIM:617120]
B9D2 Q9BPU9 VAR_036626 p.Ser101Arg LP/P rs1487082103 Meckel syndrome 10 (MKS10) [MIM:614175]
B9D2 Q9BPU9 VAR_066996 p.Ile11Met LB/B rs2241714 -
B9D2 Q9BPU9 VAR_080463 p.Leu36Pro US rs757863670 Joubert syndrome 34 (JBTS34) [MIM:614175]
B9D2 Q9BPU9 VAR_080464 p.Pro74Ser US rs863225150 Joubert syndrome 34 (JBTS34) [MIM:614175]
B9D2 Q9BPU9 VAR_080465 p.Gly155Ser US rs750436680 Joubert syndrome 34 (JBTS34) [MIM:614175]
BAALC Q8WXS3 VAR_056741 p.Ser129Thr LB/B rs34542607 -
BAAT Q14032 VAR_023737 p.Met76Val LP/P rs28937579 Familial hypercholanemia (FHCA) [MIM:607748]
BAAT Q14032 VAR_052303 p.Arg20Gln LB/B rs1572983 -
BACE1 P56817 VAR_051509 p.Arg481Cys LB/B rs539765 -
BACE1 P56817 VAR_060692 p.Val265Ala LB/B rs28989503 -
BACH1 O14867 VAR_048441 p.Ser314Pro LB/B rs35474725 -
BACH2 Q9BYV9 VAR_033535 p.Ala418Thr LB/B rs34335140 -
BACH2 Q9BYV9 VAR_082216 p.Leu24Pro LP/P - Immunodeficiency 60 (IMD60) [MIM:618394]
BACH2 Q9BYV9 VAR_082217 p.Glu788Lys US rs757652995 Immunodeficiency 60 (IMD60) [MIM:618394]
BAD Q92934 VAR_015380 p.Ala107Ser LB/B rs3729933 -
BAG3 O95817 VAR_048344 p.Arg71Gln LB/B rs35434411 -
BAG3 O95817 VAR_048345 p.Cys151Arg LB/B rs2234962 -
BAG3 O95817 VAR_048346 p.Ala405Val LB/B rs11199064 -
BAG3 O95817 VAR_048347 p.Pro407Leu LB/B rs3858340 -
BAG3 O95817 VAR_063089 p.Pro209Leu LP/P rs121918312 Myopathy, myofibrillar, 6 (MFM6) [MIM:612954]
BAG3 O95817 VAR_065479 p.Arg71Trp LP/P rs387906874 Cardiomyopathy, dilated 1HH (CMD1HH) [MIM:613881]
BAG3 O95817 VAR_065480 p.Arg477His LP/P rs387906876 Cardiomyopathy, dilated 1HH (CMD1HH) [MIM:613881]
BAG3 O95817 VAR_066777 p.Pro77Leu LB/B rs141355480 -
BAG3 O95817 VAR_066778 p.Ile94Phe LB/B rs145393807 -
BAG3 O95817 VAR_066779 p.Pro115Ser LB/B rs774241343 -
BAG3 O95817 VAR_066780 p.Ala155Thr LB/B rs61756328 -
BAG3 O95817 VAR_066781 p.Arg218Trp LP/P rs397514506 Cardiomyopathy, dilated 1HH (CMD1HH) [MIM:613881]
BAG3 O95817 VAR_066782 p.Arg258Trp LB/B rs117671123 -
BAG3 O95817 VAR_066783 p.Asp300Asn LB/B rs78439745 -
BAG3 O95817 VAR_066784 p.Pro380Ser LB/B rs144692954 -
BAG3 O95817 VAR_066785 p.Glu455Lys LP/P rs397516881 Cardiomyopathy, dilated 1HH (CMD1HH) [MIM:613881]
BAG3 O95817 VAR_066786 p.Leu462Pro LP/P rs397514507 Cardiomyopathy, dilated 1HH (CMD1HH) [MIM:613881]
BAG3 O95817 VAR_066787 p.Val468Met LP/P - Cardiomyopathy, dilated 1HH (CMD1HH) [MIM:613881]
BAG3 O95817 VAR_066788 p.Glu553Asp LB/B rs763530097 -
BAG5 Q9UL15 VAR_058712 p.Cys157Trp LB/B rs17854644 -

BAG6	P46379	VAR_023531	p.Ser625Pro	LB/B	rs1052486	-
BAG6	P46379	VAR_037150	p.Ala728Val	LB/B	rs11548856	-
BAGE2	Q86Y30	VAR_059585	p.Arg95Thr	LB/B	rs2740327	-
BAGE2	Q86Y30	VAR_059586	p.Arg106Gln	LB/B	rs9808647	-
BAGE3	Q86Y29	VAR_059587	p.Arg95Thr	LB/B	rs2740327	-
BAGE3	Q86Y29	VAR_059588	p.Gln106Arg	LB/B	rs9808647	-
BAHCC1	Q9P281	VAR_050685	p.Arg2510Cys	LB/B	rs8746	-
BAHCC1	Q9P281	VAR_059589	p.Thr1168Ala	LB/B	rs7213444	-
BAHCC1	Q9P281	VAR_061559	p.Ala272Thr	LB/B	rs12952981	-
BAHCC1	Q9P281	VAR_061560	p.Arg1434Gln	LB/B	rs35572189	-
BAHCC1	Q9P281	VAR_061561	p.Thr1678Ala	LB/B	rs12601317	-
BAHCC1	Q9P281	VAR_061562	p.Val2029Ile	LB/B	rs34680524	-
BAHD1	Q8TBE0	VAR_032359	p.Glu26Gly	LB/B	rs3743143	-
BAHD1	Q8TBE0	VAR_032360	p.Asp182His	LB/B	rs17856679	-
BAHD1	Q8TBE0	VAR_032361	p.Gln298Lys	LB/B	rs3803357	-
BAIAP2	Q9UQB8	VAR_050686	p.Gln519Arg	LB/B	rs4969391	-
BAIAP2L1	Q9UHR4	VAR_033515	p.Ser460Thr	LB/B	rs2269966	-
BAIAP3	O94812	VAR_026667	p.Asp582Ala	LB/B	rs1132356	-
BAIAP3	O94812	VAR_050687	p.Ser879Ile	LB/B	rs36074509	-
BAK1	Q16611	VAR_018829	p.Ala28Val	LB/B	rs4987115	-
BAK1	Q16611	VAR_018830	p.Ser69Arg	LB/B	rs5745592	-
BAK1	Q16611	VAR_048417	p.Arg42His	LB/B	rs1051911	-
BANF1	O75531	VAR_065954	p.Ala12Thr	LP/P	rs387906871	Nestor-Guillermo progeria syndrome (NGPS) [MIM:614008]
BANF2	Q9H503	VAR_013693	p.Thr78Ser	LB/B	rs1053993	-
BANF2	Q9H503	VAR_024372	p.Asn3Asp	LB/B	rs4814640	-
BANK1	Q8NDB2	VAR_027729	p.Arg61His	LB/B	rs10516487	-
BANK1	Q8NDB2	VAR_027730	p.Ala383Thr	LB/B	rs3733197	-
BANK1	Q8NDB2	VAR_027731	p.Cys650Arg	LB/B	rs3113676	-
BAP1	Q92560	VAR_051517	p.Val616Glu	LB/B	rs35353781	-
BAP1	Q92560	VAR_063498	p.Ala95Asp	US	-	A lung cancer sample
BAP1	Q92560	VAR_063499	p.Gly178Val	US	-	A lung cancer sample
BAP1	Q92560	VAR_065976	p.Ser63Cys	LB/B	rs747311942	-
BAP1	Q92560	VAR_065977	p.Phe81Val	US	-	-
BAP1	Q92560	VAR_065978	p.Cys91Trp	US	-	-
BAP1	Q92560	VAR_065979	p.Glu315Ala	LB/B	rs149974450	-
BAP1	Q92560	VAR_065980	p.Glu685Val	US	-	-
BAP1	Q92560	VAR_075251	p.Ile47Phe	US	-	-
BARD1	Q99728	VAR_010354	p.Pro24Ser	LB/B	rs1048108	-
BARD1	Q99728	VAR_010355	p.Lys153Glu	LB/B	rs753377280	-
BARD1	Q99728	VAR_010356	p.Val507Met	LB/B	rs2070094	-
BARD1	Q99728	VAR_010357	p.Cys557Ser	LB/B	rs28997576	-
BARD1	Q99728	VAR_010358	p.Gln564His	US	-	An ovarian clear cell adenocarcinoma
BARD1	Q99728	VAR_010359	p.Arg658Cys	LB/B	rs3738888	-
BARD1	Q99728	VAR_010360	p.Val695Leu	US	rs111367604	A breast cancer sample
BARD1	Q99728	VAR_010361	p.Ser761Asn	US	rs142155101	An uterine cancer sample
BARD1	Q99728	VAR_020109	p.Ser241Cys	LB/B	rs3738885	-
BARD1	Q99728	VAR_024611	p.Arg378Ser	LB/B	rs2229571	-
BARD1	Q99728	VAR_028309	p.Ser728Phe	LB/B	rs13389423	-

BARD1	Q99728	VAR_038371	p.Ser186Gly	LB/B	rs16852741	-
BARD1	Q99728	VAR_038372	p.Cys645Arg	LB/B	rs2228456	-
BARX1	Q9HBU1	VAR_010927	p.Ala48Thr	LB/B	rs191789925	-
BASP1	P80723	VAR_048396	p.Ala76Val	LB/B	rs3733748	-
BATF2	Q8N1L9	VAR_048443	p.Gly6Ser	LB/B	rs12419103	-
BATF3	Q9NR55	VAR_039988	p.Val11Ile	LB/B	rs2202683	-
BAX	Q07812	VAR_007809	p.Gly67Arg	US	rs398122513	A T-cell acute lymphoblastic leukemia cell line
BAX	Q07812	VAR_013575	p.Gly11Glu	LB/B	rs555248599	-
BAX	Q07812	VAR_013576	p.Gly108Val	US	-	A Burkitt lymphoma
BAX	Q07812	VAR_047053	p.Gly39Arg	LB/B	rs36017265	-
BAZ1A	Q9NRL2	VAR_028049	p.Asp344Glu	LB/B	rs1133285	-
BAZ1A	Q9NRL2	VAR_048423	p.Asn1366Lys	LB/B	rs1044140	-
BAZ2A	Q9UIF9	VAR_055548	p.Val498Glu	LB/B	rs2230579	-
BAZ2B	Q9UIF8	VAR_055549	p.Met71Thr	LB/B	rs10202670	-
BAZ2B	Q9UIF8	VAR_055550	p.Leu422Ser	LB/B	rs3213790	-
BAZ2B	Q9UIF8	VAR_055551	p.Pro530Leu	LB/B	rs3732287	-
BAZ2B	Q9UIF8	VAR_055552	p.Gly702Val	LB/B	rs2302924	-
BAZ2B	Q9UIF8	VAR_055553	p.Ser2024Asn	LB/B	rs415793	-
BBOF1	Q8ND07	VAR_027815	p.Lys496Glu	LB/B	rs3742809	-
BBOF1	Q8ND07	VAR_059618	p.Arg58Gln	LB/B	rs3784039	-
BBOF1	Q8ND07	VAR_059619	p.Arg183Lys	LB/B	rs17182762	-
BBOF1	Q8ND07	VAR_059620	p.Asp230Val	LB/B	rs3784038	-
BBS1	Q8NFJ9	VAR_017215	p.Glu234Lys	LP/P	rs35520756	Bardet-Biedl syndrome 1 (BBS1) [MIM:209900]
BBS1	Q8NFJ9	VAR_017216	p.Met390Arg	LP/P	rs113624356	Bardet-Biedl syndrome 1 (BBS1) [MIM:209900]
BBS1	Q8NFJ9	VAR_017217	p.Leu518Pro	LP/P	rs121917778	Bardet-Biedl syndrome 1 (BBS1) [MIM:209900]
BBS1	Q8NFJ9	VAR_038880	p.His35Arg	LP/P	rs775990952	Bardet-Biedl syndrome 1 (BBS1) [MIM:209900]
BBS1	Q8NFJ9	VAR_038881	p.Lys53Glu	LP/P	rs766602837	Bardet-Biedl syndrome 1 (BBS1) [MIM:209900]
BBS1	Q8NFJ9	VAR_038882	p.Asp148Asn	LP/P	rs200688985	Bardet-Biedl syndrome 1 (BBS1) [MIM:209900]
BBS1	Q8NFJ9	VAR_038883	p.Arg160Gln	LP/P	rs376894444	Bardet-Biedl syndrome 1 (BBS1) [MIM:209900]
BBS1	Q8NFJ9	VAR_038884	p.Gly305Ser	LP/P	rs942862410	Bardet-Biedl syndrome 1 (BBS1) [MIM:209900]
BBS1	Q8NFJ9	VAR_038886	p.Tyr434Ser	LP/P	-	Bardet-Biedl syndrome 1 (BBS1) [MIM:209900]
BBS1	Q8NFJ9	VAR_038887	p.Leu503His	LP/P	rs778225393	Bardet-Biedl syndrome 1 (BBS1) [MIM:209900]
BBS1	Q8NFJ9	VAR_038888	p.Leu518Gln	LP/P	-	Bardet-Biedl syndrome 1 (BBS1) [MIM:209900]
BBS1	Q8NFJ9	VAR_065554	p.Gly559Asp	LB/B	rs544767888	-
BBS1	Q8NFJ9	VAR_066278	p.Ile330Thr	LP/P	-	Bardet-Biedl syndrome 1 (BBS1) [MIM:209900]
BBS1	Q8NFJ9	VAR_066485	p.Leu206Val	LB/B	rs146052054	-
BBS1	Q8NFJ9	VAR_066486	p.Pro245Leu	LB/B	rs151203205	-
BBS10	Q8TAM1	VAR_026391	p.Arg34Pro	LP/P	rs137852836	Bardet-Biedl syndrome 10 (BBS10) [MIM:615987]
BBS10	Q8TAM1	VAR_026392	p.Arg49Trp	LP/P	rs768933093	Bardet-Biedl syndrome 10 (BBS10) [MIM:615987]
BBS10	Q8TAM1	VAR_026393	p.Cys91Trp	LP/P	rs148374859	Bardet-Biedl syndrome 10 (BBS10) [MIM:615987]
BBS10	Q8TAM1	VAR_026394	p.Leu170Ser	LP/P	rs780916348	Bardet-Biedl syndrome 10 (BBS10) [MIM:615987]
BBS10	Q8TAM1	VAR_026395	p.Cys195Trp	LP/P	-	Bardet-Biedl syndrome 10 (BBS10) [MIM:615987]
BBS10	Q8TAM1	VAR_026396	p.Tyr197Cys	LP/P	rs756632517	Bardet-Biedl syndrome 10 (BBS10) [MIM:615987]
BBS10	Q8TAM1	VAR_026397	p.Val240Gly	LP/P	-	Bardet-Biedl syndrome 10 (BBS10) [MIM:615987]
BBS10	Q8TAM1	VAR_026398	p.Leu308Phe	LP/P	-	Bardet-Biedl syndrome 10 (BBS10) [MIM:615987]

BBS10	Q8TAM1	VAR_026399	p.Ser311Ala	LP/P	rs137852837	Bardet-Biedl syndrome 10 (BBS10) [MIM:615987]
BBS10	Q8TAM1	VAR_026400	p.Ser329Leu	LP/P	rs1000990130	Bardet-Biedl syndrome 10 (BBS10) [MIM:615987]
BBS10	Q8TAM1	VAR_026401	p.Pro363Leu	LP/P	rs938066133	Bardet-Biedl syndrome 10 (BBS10) [MIM:615987]
BBS10	Q8TAM1	VAR_026402	p.Leu376Phe	LB/B	rs11109474	-
BBS10	Q8TAM1	VAR_026403	p.Leu414Ser	LP/P	rs786204575	Bardet-Biedl syndrome 10 (BBS10) [MIM:615987]
BBS10	Q8TAM1	VAR_026404	p.Lys579Arg	LP/P	rs141521925	Bardet-Biedl syndrome 10 (BBS10) [MIM:615987]
BBS10	Q8TAM1	VAR_026405	p.Tyr613Cys	LP/P	rs575957641	Bardet-Biedl syndrome 10 (BBS10) [MIM:615987]
BBS10	Q8TAM1	VAR_026406	p.Tyr613His	LP/P	rs141647931	Bardet-Biedl syndrome 10 (BBS10) [MIM:615987]
BBS10	Q8TAM1	VAR_026407	p.Gly677Val	LP/P	rs1555202553	Bardet-Biedl syndrome 10 (BBS10) [MIM:615987]
BBS10	Q8TAM1	VAR_026408	p.Thr689Pro	LP/P	rs759387000	Bardet-Biedl syndrome 10 (BBS10) [MIM:615987]
BBS10	Q8TAM1	VAR_052272	p.Pro539Leu	LB/B	rs35676114	-
BBS10	Q8TAM1	VAR_066252	p.Leu55Pro	LP/P	rs1460517643	Bardet-Biedl syndrome 10 (BBS10) [MIM:615987]
BBS10	Q8TAM1	VAR_066253	p.Asp142Asn	LB/B	rs142863601	-
BBS10	Q8TAM1	VAR_066254	p.Lys188Thr	LP/P	-	Bardet-Biedl syndrome 10 (BBS10) [MIM:615987]
BBS10	Q8TAM1	VAR_066255	p.Met255Ile	US	rs139658279	-
BBS10	Q8TAM1	VAR_066256	p.Ala296Thr	US	rs150587582	-
BBS10	Q8TAM1	VAR_066257	p.His410Gln	LP/P	rs1447555059	Bardet-Biedl syndrome 10 (BBS10) [MIM:615987]
BBS10	Q8TAM1	VAR_066258	p.Leu600Ser	LP/P	-	Bardet-Biedl syndrome 10 (BBS10) [MIM:615987]
BBS10	Q8TAM1	VAR_066259	p.Ala636Val	LP/P	rs113224628	Bardet-Biedl syndrome 10 (BBS10) [MIM:615987]
BBS10	Q8TAM1	VAR_066260	p.Leu687Pro	LP/P	-	Bardet-Biedl syndrome 10 (BBS10) [MIM:615987]
BBS10	Q8TAM1	VAR_066261	p.His715Arg	LB/B	rs769179905	-
BBS10	Q8TAM1	VAR_075722	p.Val11Gly	LP/P	rs137852838	Bardet-Biedl syndrome 10 (BBS10) [MIM:615987]
BBS12	Q6ZW61	VAR_034919	p.Ile39Thr	LB/B	rs138036823	-
BBS12	Q6ZW61	VAR_034921	p.Glu126Asp	LB/B	rs309369	-
BBS12	Q6ZW61	VAR_034922	p.Pro159Leu	US	rs1450190654	Bardet-Biedl syndrome 12 (BBS12) [MIM:615989]
BBS12	Q6ZW61	VAR_034923	p.Ile170Val	LB/B	rs1218692709	-
BBS12	Q6ZW61	VAR_034924	p.Lys195Arg	LB/B	rs17854892	-
BBS12	Q6ZW61	VAR_034925	p.Asn238Lys	LB/B	rs17006082	-
BBS12	Q6ZW61	VAR_034926	p.Ala289Pro	LP/P	rs121918328	Bardet-Biedl syndrome 12 (BBS12) [MIM:615989]
BBS12	Q6ZW61	VAR_034927	p.Arg386Gln	LB/B	rs309370	-
BBS12	Q6ZW61	VAR_034928	p.Ser429Thr	LB/B	rs7665271	-
BBS12	Q6ZW61	VAR_034929	p.Asn461His	LB/B	rs10027479	-
BBS12	Q6ZW61	VAR_034930	p.Asp467Asn	LB/B	rs13135778	-
BBS12	Q6ZW61	VAR_034931	p.Arg484Lys	LB/B	rs35690634	-

BBS12 Q6ZW61 VAR_034932 p.Gly540Val LP/P rs1010403072 Bardet-Biedl syndrome 12 (BBS12) [MIM:615989]
BBS12 Q6ZW61 VAR_034933 p.Ala615Val LB/B rs17857451 -
BBS12 Q6ZW61 VAR_062964 p.Ile346Thr LP/P rs1553941373 Bardet-Biedl syndrome 12 (BBS12) [MIM:615989]
BBS12 Q6ZW61 VAR_062965 p.Thr501Met LP/P rs138011813 Bardet-Biedl syndrome 12 (BBS12) [MIM:615989]
BBS12 Q6ZW61 VAR_066266 p.Leu88Arg LP/P rs746271266 Bardet-Biedl syndrome 12 (BBS12) [MIM:615989]
BBS12 Q6ZW61 VAR_066267 p.Gly119Ser LB/B rs77731085 -
BBS12 Q6ZW61 VAR_066268 p.Tyr263His LB/B rs150040166 -
BBS12 Q6ZW61 VAR_066269 p.Gln293Glu LP/P - Bardet-Biedl syndrome 12 (BBS12) [MIM:615989]
BBS12 Q6ZW61 VAR_066270 p.Arg355Gln LP/P rs1474900361 Bardet-Biedl syndrome 12 (BBS12) [MIM:615989]
BBS12 Q6ZW61 VAR_066271 p.Val400Met LP/P rs771136797 Bardet-Biedl syndrome 12 (BBS12) [MIM:615989]
BBS12 Q6ZW61 VAR_066272 p.Lys408Arg US - -
BBS12 Q6ZW61 VAR_066274 p.Tyr524Cys US rs770746493 -
BBS12 Q6ZW61 VAR_066275 p.Arg525His US rs776730549 Bardet-Biedl syndrome 12 (BBS12) [MIM:615989]
BBS12 Q6ZW61 VAR_066276 p.Gly539Asp LP/P - Bardet-Biedl syndrome 12 (BBS12) [MIM:615989]
BBS12 Q6ZW61 VAR_066277 p.Arg674Cys LP/P rs759088490 Bardet-Biedl syndrome 12 (BBS12) [MIM:615989]
BBS2 Q9BXC9 VAR_013162 p.Asn70Ser LP/P rs4784677 Bardet-Biedl syndrome 2 (BBS2) [MIM:615981]
BBS2 Q9BXC9 VAR_013163 p.Val75Gly LP/P rs121908174 Bardet-Biedl syndrome 2 (BBS2) [MIM:615981]
BBS2 Q9BXC9 VAR_013164 p.Asp104Ala LP/P rs121908179 Bardet-Biedl syndrome 2 (BBS2) [MIM:615981]
BBS2 Q9BXC9 VAR_013164 p.Asp104Ala LP/P rs121908179 Retinitis pigmentosa 74 (RP74) [MIM:616562]
BBS2 Q9BXC9 VAR_013165 p.Ile123Val LB/B rs11373 -
BBS2 Q9BXC9 VAR_013166 p.Arg315Gln LP/P rs544773389 Bardet-Biedl syndrome 2 (BBS2) [MIM:615981]
BBS2 Q9BXC9 VAR_013167 p.Arg315Trp LP/P rs121908178 Bardet-Biedl syndrome 2 (BBS2) [MIM:615981]
BBS2 Q9BXC9 VAR_013168 p.Thr558Ile LP/P rs370581600 Bardet-Biedl syndrome 2 (BBS2) [MIM:615981]
BBS2 Q9BXC9 VAR_013169 p.Arg632Pro LP/P rs138043021 Bardet-Biedl syndrome 2 (BBS2) [MIM:615981]
BBS2 Q9BXC9 VAR_013169 p.Arg632Pro LP/P rs138043021 Retinitis pigmentosa 74 (RP74) [MIM:616562]
BBS2 Q9BXC9 VAR_029747 p.Ala122Val LB/B rs17856449 -
BBS2 Q9BXC9 VAR_029748 p.Ala504Val LB/B rs16957538 -
BBS2 Q9BXC9 VAR_038889 p.Arg23Pro LP/P - Bardet-Biedl syndrome 2 (BBS2) [MIM:615981]
BBS2 Q9BXC9 VAR_038890 p.Asp174Glu LP/P rs767373822 Bardet-Biedl syndrome 2 (BBS2) [MIM:615981]
BBS2 Q9BXC9 VAR_038891 p.Leu349Trp LP/P rs752280639 Bardet-Biedl syndrome 2 (BBS2) [MIM:615981]
BBS2 Q9BXC9 VAR_038892 p.Arg643His LP/P rs532361142 Bardet-Biedl syndrome 2 (BBS2) [MIM:615981]
BBS2 Q9BXC9 VAR_066280 p.Gly81Cys LP/P rs750506474 Bardet-Biedl syndrome 2 (BBS2) [MIM:615981]
BBS2 Q9BXC9 VAR_066281 p.Leu125Arg LP/P - Bardet-Biedl syndrome 2 (BBS2) [MIM:615981]
BBS2 Q9BXC9 VAR_066282 p.Ala136Pro LP/P - Bardet-Biedl syndrome 2 (BBS2) [MIM:615981]
BBS2 Q9BXC9 VAR_066283 p.Cys307Trp LP/P - Bardet-Biedl syndrome 2 (BBS2) [MIM:615981]
BBS2 Q9BXC9 VAR_066284 p.Tyr317Cys LP/P - Bardet-Biedl syndrome 2 (BBS2) [MIM:615981]
BBS2 Q9BXC9 VAR_066285 p.Glu629Lys LB/B rs746505864 -
BBS2 Q9BXC9 VAR_075726 p.Ala33Asp LP/P rs797045155 Retinitis pigmentosa 74 (RP74) [MIM:616562]
BBS2 Q9BXC9 VAR_075727 p.Pro134Arg LP/P rs376306240 Retinitis pigmentosa 74 (RP74) [MIM:616562]
BBS2 Q9BXC9 VAR_075728 p.Gly139Val LP/P rs121908181 Bardet-Biedl syndrome 2 (BBS2) [MIM:615981]
BBS4 Q96RK4 VAR_013170 p.Arg295Pro LP/P rs121434632 Bardet-Biedl syndrome 4 (BBS4) [MIM:615982]
BBS4 Q96RK4 VAR_017049 p.Asn165His LP/P - Bardet-Biedl syndrome 4 (BBS4) [MIM:615982]
BBS4 Q96RK4 VAR_017050 p.Leu327Pro LP/P - Bardet-Biedl syndrome 4 (BBS4) [MIM:615982]

BBS4	Q96RK4	VAR_017051	p.Ala364Glu	LP/P	rs28938468	Bardet-Biedl syndrome 4 (BBS4) [MIM:615982]
BBS4	Q96RK4	VAR_017052	p.Ser457Ile	LP/P	-	Bardet-Biedl syndrome 4 (BBS4) [MIM:615982]
BBS4	Q96RK4	VAR_017053	p.Met472Val	LP/P	rs2277596	Bardet-Biedl syndrome 4 (BBS4) [MIM:615982]
BBS4	Q96RK4	VAR_017054	p.Ile354Thr	LB/B	rs2277598	-
BBS4	Q96RK4	VAR_028722	p.Glu268Lys	LB/B	rs11638283	-
BBS4	Q96RK4	VAR_028723	p.Ala393Val	LB/B	rs17852452	-
BBS4	Q96RK4	VAR_038894	p.Lys46Arg	LB/B	rs75295839	-
BBS4	Q96RK4	VAR_038895	p.Leu351Arg	LP/P	-	Bardet-Biedl syndrome 4 (BBS4) [MIM:615982]
BBS4	Q96RK4	VAR_038896	p.Asp368Gly	LP/P	rs772548770	Bardet-Biedl syndrome 4 (BBS4) [MIM:615982]
BBS4	Q96RK4	VAR_038897	p.Pro503Leu	LP/P	rs756419611	Bardet-Biedl syndrome 4 (BBS4) [MIM:615982]
BBS4	Q96RK4	VAR_066287	p.Glu61Lys	LB/B	rs1251827333	-
BBS4	Q96RK4	VAR_066288	p.Glu412Asp	LB/B	rs147202164	-
BBS4	Q96RK4	VAR_066289	p.Thr488Lys	LB/B	rs561284402	-
BBS5	Q8N3I7	VAR_025316	p.Asn184Ser	LB/B	rs137853921	-
BBS5	Q8N3I7	VAR_025317	p.Arg207His	LB/B	rs35487251	-
BBS5	Q8N3I7	VAR_066290	p.Gly72Ser	LP/P	rs121908581	Bardet-Biedl syndrome 5 (BBS5) [MIM:615983]
BBS5	Q8N3I7	VAR_066291	p.Asn251Asp	LB/B	rs143113298	-
BBS5	Q8N3I7	VAR_072380	p.Thr183Ala	LP/P	rs121908582	Bardet-Biedl syndrome 5 (BBS5) [MIM:615983]
BBS7	Q8IWZ6	VAR_017212	p.Thr211Ile	LP/P	rs119466002	Bardet-Biedl syndrome 7 (BBS7) [MIM:615984]
BBS7	Q8IWZ6	VAR_017213	p.His323Arg	LP/P	rs119466001	Bardet-Biedl syndrome 7 (BBS7) [MIM:615984]
BBS7	Q8IWZ6	VAR_038893	p.Ile66Phe	LP/P	rs1553934343	Bardet-Biedl syndrome 7 (BBS7) [MIM:615984]
BBS7	Q8IWZ6	VAR_065555	p.Tyr671Cys	LB/B	rs1013002037	-
BBS7	Q8IWZ6	VAR_066286	p.Gly63Arg	LP/P	rs754579374	Bardet-Biedl syndrome 7 (BBS7) [MIM:615984]
BBS7	Q8IWZ6	VAR_066459	p.Gln293Pro	LB/B	rs889417696	-
BBS9	Q3SYG4	VAR_026389	p.Gly141Arg	LP/P	rs137852857	Bardet-Biedl syndrome 9 (BBS9) [MIM:615986]
BBS9	Q3SYG4	VAR_026390	p.Ala455Val	LB/B	rs764873070	-
BBS9	Q3SYG4	VAR_051289	p.Thr12Ala	LB/B	rs4498440	-
BBS9	Q3SYG4	VAR_051290	p.Ala455Thr	LB/B	rs11773504	-
BBS9	Q3SYG4	VAR_051291	p.Arg521Gln	LB/B	rs34218557	-
BBS9	Q3SYG4	VAR_066292	p.Thr549Ile	LB/B	rs59252892	-
BBS9	Q3SYG4	VAR_066293	p.Leu665Phe	LB/B	rs116262072	-
BBS9	Q3SYG4	VAR_066294	p.Leu779Gln	LB/B	rs142434516	-
BBX	Q8WY36	VAR_061264	p.Pro576Ser	LB/B	rs59781647	-
BCAM	P50895	VAR_021348	p.Arg77His	LB/B	rs28399653	-
BCAM	P50895	VAR_021349	p.Val196Ile	LB/B	rs28399654	-
BCAM	P50895	VAR_021350	p.Met204Lys	LB/B	rs28399656	-
BCAM	P50895	VAR_021351	p.Arg282His	LB/B	rs9967601	-
BCAM	P50895	VAR_021352	p.Val381Ile	LB/B	rs28399626	-
BCAM	P50895	VAR_021353	p.Lys451Gln	LB/B	rs28399630	-
BCAM	P50895	VAR_021354	p.Thr539Ala	LB/B	rs1135062	-
BCAM	P50895	VAR_021355	p.Gln581Leu	LB/B	rs28399659	-
BCAN	Q96GW7	VAR_019551	p.Glu504Lys	LB/B	rs1056695	-
BCAN	Q96GW7	VAR_050123	p.Ser356Leu	LB/B	rs12065791	-
BCAR1	P56945	VAR_035798	p.Ser407Thr	US	rs144989936	A breast cancer sample
BCAR1	P56945	VAR_057820	p.Arg491Leu	LB/B	rs16957558	-
BCAR1	P56945	VAR_057821	p.His558Arg	LB/B	rs16957552	-
BCAR1	P56945	VAR_058970	p.Pro76Ser	LB/B	rs1035539	-
BCAR3	O75815	VAR_050689	p.Glu464Gly	LB/B	rs12062278	-
BCAR3	O75815	VAR_050690	p.Gln593His	LB/B	rs17110107	-

BCAS1 O75363 VAR_024251 p.Gly255Glu LB/B rs6022903 -
BCAS1 O75363 VAR_026674 p.Gln24Lys LB/B rs394732 -
BCAS1 O75363 VAR_026675 p.Val163Ala LB/B rs158551 -
BCAS1 O75363 VAR_026676 p.Ser583Pro LB/B rs1055246 -
BCAS1 O75363 VAR_050691 p.Gln472His LB/B rs35575210 -
BCAS2 O75934 VAR_035799 p.Asn139Ser US rs1197668726 A colorectal cancer sample
BCAS3 Q9H6U6 VAR_057583 p.Ile106Val LB/B rs34712615 -
BCAS3 Q9H6U6 VAR_065093 p.Asn87Ser LB/B rs2643103 -
BCAS4 Q8TDM0 VAR_016031 p.Glu56Asp LB/B rs2272962 -
BCAS4 Q8TDM0 VAR_059590 p.Arg173His LB/B rs7273412 -
BCAT1 P54687 VAR_019614 p.Glu321Lys LB/B rs7313020 -
BCAT1 P54687 VAR_047681 p.Thr59Met LB/B rs17374285 -
BCAT1 P54687 VAR_047682 p.Gly330Ser LB/B rs1057204 -
BCAT2 O15382 VAR_048234 p.Thr186Arg LB/B rs11548193 -
BCAT2 O15382 VAR_084533 p.Arg170Gln LP/P - Hypervalinemia and hyperleucine-isoleucinemia (HVLI) [MIM:618850]
BCAT2 O15382 VAR_084534 p.Glu264Lys LP/P - Hypervalinemia and hyperleucine-isoleucinemia (HVLI) [MIM:618850]
BCCIP Q9P287 VAR_046642 p.Glu254Gln LB/B rs17153610 -
BCDIN3D Q7Z5W3 VAR_032614 p.Ser288Arg LB/B rs11169172 -
BCHE P06276 VAR_002360 p.Asp98Gly LP/P rs1799807 Butyrylcholinesterase deficiency (BCHED) [MIM:617936]
BCHE P06276 VAR_002362 p.Leu358Ile LP/P rs121918557 Butyrylcholinesterase deficiency (BCHED) [MIM:617936]
BCHE P06276 VAR_002364 p.Ala567Thr LP/P rs1803274 Butyrylcholinesterase deficiency (BCHED) [MIM:617936]
BCHE P06276 VAR_040012 p.Thr52Met LP/P rs56309853 Butyrylcholinesterase deficiency (BCHED) [MIM:617936]
BCHE P06276 VAR_040013 p.Phe56Ile LP/P rs531738678 Butyrylcholinesterase deficiency (BCHED) [MIM:617936]
BCHE P06276 VAR_040014 p.Tyr61Cys LP/P rs116097205 Butyrylcholinesterase deficiency (BCHED) [MIM:617936]
BCHE P06276 VAR_040015 p.Pro65Ser LP/P rs148170012 Butyrylcholinesterase deficiency (BCHED) [MIM:617936]
BCHE P06276 VAR_040016 p.Asp98His LP/P - Butyrylcholinesterase deficiency (BCHED) [MIM:617936]
BCHE P06276 VAR_040017 p.Asn124Tyr LP/P rs1339128583 Butyrylcholinesterase deficiency (BCHED) [MIM:617936]
BCHE P06276 VAR_040018 p.Pro128Ser LP/P rs3732880 Butyrylcholinesterase deficiency (BCHED) [MIM:617936]
BCHE P06276 VAR_040019 p.Gly143Asp LP/P rs201820739 Butyrylcholinesterase deficiency (BCHED) [MIM:617936]
BCHE P06276 VAR_040020 p.Leu153Phe LP/P rs747598704 Butyrylcholinesterase deficiency (BCHED) [MIM:617936]
BCHE P06276 VAR_040021 p.Tyr156Cys LP/P rs121918558 Butyrylcholinesterase deficiency (BCHED) [MIM:617936]
BCHE P06276 VAR_040022 p.Val170Met LP/P rs527843566 Butyrylcholinesterase deficiency (BCHED) [MIM:617936]
BCHE P06276 VAR_040023 p.Asp198Glu LP/P rs781368801 Butyrylcholinesterase deficiency (BCHED) [MIM:617936]

BCHE	P06276	VAR_040024	p.Ser226Gly	LP/P	rs370077923	Butyrylcholinesterase deficiency (BCHED) [MIM:617936]
BCHE	P06276	VAR_040025	p.Ala227Val	LP/P	-	Butyrylcholinesterase deficiency (BCHED) [MIM:617936]
BCHE	P06276	VAR_040026	p.Ala229Thr	LP/P	-	Butyrylcholinesterase deficiency (BCHED) [MIM:617936]
BCHE	P06276	VAR_040027	p.Thr271Met	LP/P	rs28933389	Butyrylcholinesterase deficiency (BCHED) [MIM:617936]
BCHE	P06276	VAR_040028	p.Thr278Pro	LP/P	rs892642457	Butyrylcholinesterase deficiency (BCHED) [MIM:617936]
BCHE	P06276	VAR_040029	p.Glu283Asp	LB/B	rs16849700	-
BCHE	P06276	VAR_040030	p.Lys295Arg	LP/P	rs115624085	Butyrylcholinesterase deficiency (BCHED) [MIM:617936]
BCHE	P06276	VAR_040031	p.Leu335Pro	LP/P	rs104893684	Butyrylcholinesterase deficiency (BCHED) [MIM:617936]
BCHE	P06276	VAR_040032	p.Ala356Asp	LP/P	rs770337031	Butyrylcholinesterase deficiency (BCHED) [MIM:617936]
BCHE	P06276	VAR_040033	p.Gly393Arg	LP/P	rs115129687	Butyrylcholinesterase deficiency (BCHED) [MIM:617936]
BCHE	P06276	VAR_040034	p.Arg414Cys	LP/P	rs745364489	Butyrylcholinesterase deficiency (BCHED) [MIM:617936]
BCHE	P06276	VAR_040035	p.Gly418Val	LP/P	rs28933390	Butyrylcholinesterase deficiency (BCHED) [MIM:617936]
BCHE	P06276	VAR_040036	p.Phe446Ser	LP/P	-	Butyrylcholinesterase deficiency (BCHED) [MIM:617936]
BCHE	P06276	VAR_040037	p.Glu488Lys	LP/P	rs200998515	Butyrylcholinesterase deficiency (BCHED) [MIM:617936]
BCHE	P06276	VAR_040038	p.Trp499Arg	LP/P	-	Butyrylcholinesterase deficiency (BCHED) [MIM:617936]
BCHE	P06276	VAR_040039	p.Phe502Leu	LP/P	rs769316835	Butyrylcholinesterase deficiency (BCHED) [MIM:617936]
BCHE	P06276	VAR_040040	p.Glu525Val	LP/P	rs121918556	Butyrylcholinesterase deficiency (BCHED) [MIM:617936]
BCHE	P06276	VAR_040041	p.Arg543Cys	LP/P	rs199660374	Butyrylcholinesterase deficiency (BCHED) [MIM:617936]
BCHE	P06276	VAR_040042	p.Gln546Leu	LP/P	-	Butyrylcholinesterase deficiency (BCHED) [MIM:617936]
BCHE	P06276	VAR_072094	p.Lys40Arg	LB/B	rs116047990	-
BCHE	P06276	VAR_072095	p.Gly103Arg	LP/P	rs979653503	Butyrylcholinesterase deficiency (BCHED) [MIM:617936]
BCHE	P06276	VAR_072096	p.Glu118Asp	LP/P	-	Butyrylcholinesterase deficiency (BCHED) [MIM:617936]
BCHE	P06276	VAR_072097	p.Ile127Met	LB/B	rs755600722	-
BCHE	P06276	VAR_072098	p.Val232Asp	LP/P	-	Butyrylcholinesterase deficiency (BCHED) [MIM:617936]
BCHE	P06276	VAR_072099	p.Val322Met	LB/B	rs754644618	-
BCHE	P06276	VAR_072100	p.Gly361Cys	LP/P	-	Butyrylcholinesterase deficiency (BCHED) [MIM:617936]
BCHE	P06276	VAR_072101	p.Arg498Trp	LB/B	rs115017300	-
BCHE	P06276	VAR_072730	p.Ala62Val	LP/P	rs1553778274	Butyrylcholinesterase deficiency (BCHED) [MIM:617936]
BCKDHA	P12694	VAR_004968	p.Arg159Trp	LP/P	rs769688327	Maple syrup urine disease 1A (MSUD1A) [MIM:248600]
BCKDHA	P12694	VAR_004969	p.Gln190Lys	LP/P	-	Maple syrup urine disease 1A (MSUD1A) [MIM:248600]
BCKDHA	P12694	VAR_004970	p.Ala253Thr	LP/P	rs199599175	Maple syrup urine disease 1A (MSUD1A) [MIM:248600]
BCKDHA	P12694	VAR_004971	p.Ile326Thr	LP/P	-	Maple syrup urine disease 1A (MSUD1A) [MIM:248600]

BCKDHA P12694 VAR_004972 p.Tyr413Cys LP/P - Maple syrup urine disease 1A (MSUD1A) [MIM:248600]
BCKDHA P12694 VAR_004973 p.Tyr438Asn LP/P rs137852870 Maple syrup urine disease 1A (MSUD1A) [MIM:248600]
BCKDHA P12694 VAR_015101 p.Gly290Arg LP/P rs137852871 Maple syrup urine disease 1A (MSUD1A) [MIM:248600]
BCKDHA P12694 VAR_015102 p.Phe409Cys LP/P rs137852872 Maple syrup urine disease 1A (MSUD1A) [MIM:248600]
BCKDHA P12694 VAR_034360 p.Pro39His LB/B rs11549936 -
BCKDHA P12694 VAR_034361 p.Thr151Met LB/B rs34442879 -
BCKDHA P12694 VAR_069748 p.Thr211Met LP/P rs398123503 Maple syrup urine disease 1A (MSUD1A) [MIM:248600]
BCKDHA P12694 VAR_069749 p.Ala220Val LP/P rs375785084 Maple syrup urine disease 1A (MSUD1A) [MIM:248600]
BCKDHA P12694 VAR_069750 p.Arg346Cys LP/P rs182923857 Maple syrup urine disease 1A (MSUD1A) [MIM:248600]
BCKDHB P21953 VAR_004974 p.His206Arg LP/P - Maple syrup urine disease 1B (MSUD1B) [MIM:248600]
BCKDHB P21953 VAR_024851 p.Arg183Pro LP/P rs79761867 Maple syrup urine disease 1B (MSUD1B) [MIM:248600]
BCKDHB P21953 VAR_024852 p.Gly278Ser LP/P rs386834233 Maple syrup urine disease 1B (MSUD1B) [MIM:248600]
BCKDHB P21953 VAR_050437 p.Thr41Ile LB/B rs35470366 -
BCKDHB P21953 VAR_068348 p.Arg170His LP/P rs371518124 Maple syrup urine disease 1B (MSUD1B) [MIM:248600]
BCKDHB P21953 VAR_068349 p.Gln346Arg LP/P - Maple syrup urine disease 1B (MSUD1B) [MIM:248600]
BCKDK O14874 VAR_069037 p.Arg224Pro LP/P rs147210405 Branched-chain ketoacid dehydrogenase kinase deficiency (BCKDKD) [MIM:614923]
BCKDK O14874 VAR_072184 p.Arg174Gly LP/P - Branched-chain ketoacid dehydrogenase kinase deficiency (BCKDKD) [MIM:614923]
BCKDK O14874 VAR_072185 p.Leu389Pro LP/P - Branched-chain ketoacid dehydrogenase kinase deficiency (BCKDKD) [MIM:614923]
BCL10 O95999 VAR_013208 p.Ala5Ser US rs12037217 -
BCL10 O95999 VAR_013209 p.Val16Glu US - -
BCL10 O95999 VAR_013210 p.Lys31Glu US - -
BCL10 O95999 VAR_013211 p.Lys45Gln LB/B - -
BCL10 O95999 VAR_013212 p.Thr52Ile US - -
BCL10 O95999 VAR_013213 p.Cys57Arg US - -
BCL10 O95999 VAR_013214 p.Arg58Gly US rs121918314 -
BCL10 O95999 VAR_013215 p.Arg58Gln LB/B - -
BCL10 O95999 VAR_013216 p.Arg64Lys US - -
BCL10 O95999 VAR_013217 p.Asn93Ser LB/B - -
BCL10 O95999 VAR_013218 p.Asp101Glu US - -
BCL10 O95999 VAR_013219 p.Ser134Pro US - -
BCL10 O95999 VAR_013220 p.Met153Val LB/B - -
BCL10 O95999 VAR_013221 p.Thr168Ala US rs1384278393 -
BCL10 O95999 VAR_013222 p.Leu174Ser US - -
BCL10 O95999 VAR_013224 p.Gly213Glu US rs3768235 -
BCL10 O95999 VAR_013225 p.Ser218Phe US - -
BCL10 O95999 VAR_013226 p.Val230Ile US - -
BCL10 O95999 VAR_077898 p.Thr162Met LB/B rs200837308 -

BCL11A Q9H165 VAR_035553 p.Ser142Phe US - A breast cancer sample
BCL11A Q9H165 VAR_076921 p.Thr47Pro LP/P rs886037864 Intellectual developmental disorder with persistence of fetal hemoglobin (IDPFH) [MIM:617101]
BCL11A Q9H165 VAR_076922 p.Cys48Phe LP/P rs886037865 Intellectual developmental disorder with persistence of fetal hemoglobin (IDPFH) [MIM:617101]
BCL11A Q9H165 VAR_076923 p.His66Gln LP/P rs886037866 Intellectual developmental disorder with persistence of fetal hemoglobin (IDPFH) [MIM:617101]
BCL11B Q9C0K0 VAR_035554 p.Ser331Pro US - A colorectal cancer sample
BCL11B Q9C0K0 VAR_065741 p.Glu32Val US - -
BCL11B Q9C0K0 VAR_065742 p.Pro229Ser LB/B rs749837100 -
BCL11B Q9C0K0 VAR_078423 p.Asn441Lys LP/P rs750610248 Immunodeficiency 49 (IMD49) [MIM:617237]
BCL11B Q9C0K0 VAR_081175 p.Asn807Lys LP/P rs888230251 Immunodeficiency 49 (IMD49) [MIM:617237]
BCL2 P10415 VAR_000827 p.Thr7Ser LB/B - -
BCL2 P10415 VAR_000828 p.Pro59Ser US - Non-Hodgkin lymphoma
BCL2 P10415 VAR_000829 p.Val93Ile US - Non-Hodgkin lymphoma
BCL2 P10415 VAR_014716 p.Ala43Thr LB/B rs1800477 -
BCL2A1 Q16548 VAR_020342 p.Cys19Tyr LB/B rs1138357 -
BCL2A1 Q16548 VAR_020343 p.Asn39Lys LB/B rs1138358 -
BCL2A1 Q16548 VAR_020344 p.Gly82Asp LB/B rs3826007 -
BCL2A1 Q16548 VAR_044059 p.Glu117Asp LB/B rs34080999 -
BCL2L10 Q9HD36 VAR_047113 p.Leu21Arg LB/B rs2231292 -
BCL2L12 Q9HB09 VAR_048419 p.Gly47Val LB/B rs2060263 -
BCL2L13 Q9BXK5 VAR_024377 p.Ile55Val LB/B rs16981016 -
BCL2L13 Q9BXK5 VAR_024378 p.Pro360Ser LB/B rs9306198 -
BCL2L13 Q9BXK5 VAR_059141 p.Ile46Arg LB/B rs2587070 -
BCL2L13 Q9BXK5 VAR_059142 p.Ile46Thr LB/B rs2587070 -
BCL2L15 Q5TBC7 VAR_031475 p.Asp90Asn LB/B rs1217381 -
BCL2L2 Q92843 VAR_048418 p.Gln133Arg LB/B rs910332 -
BCL6 P41182 VAR_014825 p.His676Tyr LB/B rs1056936 -
BCL6 P41182 VAR_019970 p.Ala493Thr LB/B rs2229362 -
BCL6 P41182 VAR_052709 p.Asn252Ser LB/B rs34463990 -
BCL7A Q4VC05 VAR_033539 p.Asn120Thr LB/B rs34821485 -
BCL9 O00512 VAR_046545 p.Pro671Ser LB/B rs3820129 -
BCL9 O00512 VAR_046546 p.Arg782Lys LB/B rs34002844 -
BCLAF1 Q9NYF8 VAR_050692 p.Ser209Cys LB/B rs6940018 -
BCLAF1 Q9NYF8 VAR_050693 p.Tyr459Asp LB/B rs1967446 -
BCLAF1 Q9NYF8 VAR_050694 p.Leu461His LB/B rs1967445 -
BCLAF1 Q9NYF8 VAR_050695 p.Asn629Ser LB/B rs7381749 -
BCLAF1 Q9NYF8 VAR_050696 p.Arg875Cys LB/B rs34541670 -
BCLAF1 Q9NYF8 VAR_059591 p.Gly66Ala LB/B rs9942517 -
BCO1 Q9HAY6 VAR_048406 p.Arg267Ser LB/B rs12934922 -
BCO1 Q9HAY6 VAR_048407 p.Ala379Val LB/B rs7501331 -
BCO1 Q9HAY6 VAR_058112 p.Thr170Met LP/P rs119478057 Hypercarotenemia and vitamin A deficiency, autosomal dominant (HCVAD) [MIM:115300]
BCO2 Q9BYV7 VAR_047047 p.Leu231Pro LB/B rs10891338 -
BCO2 Q9BYV7 VAR_047048 p.Gly289Glu LB/B rs17113607 -
BCO2 Q9BYV7 VAR_047049 p.Ile548Leu LB/B rs2217401 -
BCOR Q6W2J9 VAR_020921 p.Pro85Leu LP/P rs121434618 Microphthalmia, syndromic, 2 (MCOPS2) [MIM:300166]

BCORL1 Q5H9F3 VAR_037467 p.Gly209Ser LB/B rs5932715 -
BCORL1 Q5H9F3 VAR_037468 p.Gly832Asp US - A breast cancer sample
BCORL1 Q5H9F3 VAR_061020 p.Phe111Leu LB/B rs4830173 -
BCORL1 Q5H9F3 VAR_070559 p.Asn820Ser US rs398123004 Shukla-Vernon syndrome (SHUVER) [MIM:301029]
BCORL1 Q5H9F3 VAR_080909 p.Thr327Ile US - -
BCORL1 Q5H9F3 VAR_082288 p.Pro32Leu US - Shukla-Vernon syndrome (SHUVER) [MIM:301029]
BCORL1 Q5H9F3 VAR_082289 p.Ser496Phe US rs1057521638 Shukla-Vernon syndrome (SHUVER) [MIM:301029]
BCORL1 Q5H9F3 VAR_082290 p.Val782Glu US rs1488781894 Shukla-Vernon syndrome (SHUVER) [MIM:301029]
BCR P11274 VAR_031552 p.Asn796Ser LB/B rs140504 -
BCR P11274 VAR_031553 p.Glu1037Lys LB/B rs776552570 -
BCR P11274 VAR_031554 p.Thr1127Met LB/B rs35812689 -
BCR P11274 VAR_041883 p.Ser400Pro US - A bladder transitional cell carcinoma sample
BCR P11274 VAR_041884 p.Ile413Met LB/B rs56321828 -
BCR P11274 VAR_041885 p.Asp752Glu LB/B rs12484731 -
BCR P11274 VAR_041886 p.Tyr910Cys LB/B rs35537221 -
BCR P11274 VAR_041887 p.Val949Ile LB/B rs2229038 -
BCR P11274 VAR_041888 p.Val1091Met LB/B rs778229520 -
BCR P11274 VAR_041889 p.Thr1096Ala LB/B rs745459086 -
BCR P11274 VAR_041890 p.Ala1104Gly LB/B rs11558696 -
BCR P11274 VAR_041891 p.Asp1106Asn LB/B rs879255379 -
BCR P11274 VAR_041892 p.Ala1149Thr LB/B rs200099830 -
BCR P11274 VAR_041893 p.Glu1161Lys LB/B - -
BCR P11274 VAR_041894 p.Lys1187Glu LB/B rs1195127922 -
BCR P11274 VAR_041895 p.Val1189Met LB/B rs55816482 -
BCR P11274 VAR_041896 p.Ala1204Gly LB/B rs56265970 -
BCR P11274 VAR_041897 p.Trp1235Arg LB/B rs55719322 -
BCR P11274 VAR_051983 p.Lys558Thr LB/B rs4437065 -
BCS1L Q9Y276 VAR_018149 p.Ser78Gly LP/P rs28937590 GRACILE syndrome (GRACILE) [MIM:603358]
BCS1L Q9Y276 VAR_018159 p.Pro99Leu LP/P rs121908572 Mitochondrial complex III deficiency, nuclear 1 (MC3DN1) [MIM:124000]
BCS1L Q9Y276 VAR_018160 p.Arg144Gln LP/P rs386833857 GRACILE syndrome (GRACILE) [MIM:603358]
BCS1L Q9Y276 VAR_018161 p.Arg155Pro LP/P rs121908573 Mitochondrial complex III deficiency, nuclear 1 (MC3DN1) [MIM:124000]
BCS1L Q9Y276 VAR_018162 p.Ser277Asn LP/P rs121908571 Mitochondrial complex III deficiency, nuclear 1 (MC3DN1) [MIM:124000]
BCS1L Q9Y276 VAR_018163 p.Val327Ala LP/P rs386833858 GRACILE syndrome (GRACILE) [MIM:603358]
BCS1L Q9Y276 VAR_018164 p.Val353Met LP/P rs121908574 Mitochondrial complex III deficiency, nuclear 1 (MC3DN1) [MIM:124000]
BCS1L Q9Y276 VAR_032086 p.Gly35Arg LP/P rs121908579 Bjoernstad syndrome (BJS) [MIM:262000]
BCS1L Q9Y276 VAR_032087 p.Arg45Cys LP/P rs121908575 Mitochondrial complex III deficiency, nuclear 1 (MC3DN1) [MIM:124000]
BCS1L Q9Y276 VAR_032088 p.Arg114Trp LP/P rs778769841 Bjoernstad syndrome (BJS) [MIM:262000]
BCS1L Q9Y276 VAR_032089 p.Arg183His LP/P rs121908577 Bjoernstad syndrome (BJS) [MIM:262000]
BCS1L Q9Y276 VAR_032090 p.Arg184Cys LP/P rs121908578 Bjoernstad syndrome (BJS) [MIM:262000]
BCS1L Q9Y276 VAR_032090 p.Arg184Cys LP/P rs121908578 Mitochondrial complex III deficiency, nuclear 1 (MC3DN1) [MIM:124000]

BCS1L Q9Y276 VAR_032091 p.Gln302Glu LP/P rs1457171169 Bjoernstad syndrome (BJS) [MIM:262000]
BCS1L Q9Y276 VAR_032092 p.Arg306His LP/P rs1280810181 Bjoernstad syndrome (BJS) [MIM:262000]
BCS1L Q9Y276 VAR_064615 p.Thr50Ala LP/P rs121908580 Mitochondrial complex III deficiency, nuclear 1 (MC3DN1) [MIM:124000]
BCS1L Q9Y276 VAR_064616 p.Arg73Cys LP/P rs140812286 Mitochondrial complex III deficiency, nuclear 1 (MC3DN1) [MIM:124000]
BCS1L Q9Y276 VAR_064617 p.Arg183Cys LP/P rs144885874 Mitochondrial complex III deficiency, nuclear 1 (MC3DN1) [MIM:124000]
BCS1L Q9Y276 VAR_064618 p.Phe368Ile LP/P - Mitochondrial complex III deficiency, nuclear 1 (MC3DN1) [MIM:124000]
BCS1L Q9Y276 VAR_072243 p.Gly129Arg LP/P rs1057521059 Mitochondrial complex III deficiency, nuclear 1 (MC3DN1) [MIM:124000]
BCS1L Q9Y276 VAR_072244 p.Tyr301Asn LP/P rs587777278 Bjoernstad syndrome (BJS) [MIM:262000]
BDH2 Q9BUT1 VAR_023602 p.Asn70Ser LB/B rs1054707 -
BDKRB1 P46663 VAR_014359 p.Ala250Val LB/B rs2229459 -
BDKRB1 P46663 VAR_049376 p.Arg317Gln LB/B rs8004609 -
BDKRB2 P30411 VAR_003457 p.Arg14Cys LB/B rs1046248 -
BDKRB2 P30411 VAR_012284 p.Gly354Glu LB/B rs2227279 -
BDNF P23560 VAR_004626 p.Val66Met LB/B rs6265 -
BDNF P23560 VAR_011797 p.Gln75His LB/B rs1048218 -
BDNF P23560 VAR_011798 p.Arg125Met LB/B rs1048220 -
BDNF P23560 VAR_011799 p.Arg127Leu LB/B rs1048221 -
BDNF P23560 VAR_018260 p.Thr2Ile LP/P rs8192466 Congenital central hypoventilation syndrome (CCHS) [MIM:209880]
BDNF P23560 VAR_080766 p.Met122Thr US rs765594245 -
BDP1 A6H8Y1 VAR_043312 p.Asp38Glu LB/B rs3748043 -
BDP1 A6H8Y1 VAR_043313 p.Arg757Cys LB/B rs3761966 -
BDP1 A6H8Y1 VAR_043314 p.Val778Met LB/B rs3761967 -
BDP1 A6H8Y1 VAR_043315 p.Ile1264Met LB/B rs715747 -
BDP1 A6H8Y1 VAR_043316 p.Val1347Met LB/B rs6886336 -
BDP1 A6H8Y1 VAR_043317 p.Lys1469Glu LB/B rs1698063 -
BDP1 A6H8Y1 VAR_043318 p.Ile2013Leu LB/B rs6453014 -
BDP1 A6H8Y1 VAR_056743 p.Asn26Ser LB/B rs3748042 -
BDP1 A6H8Y1 VAR_056744 p.Ala125Val LB/B rs9687593 -
BDP1 A6H8Y1 VAR_056745 p.Lys722Glu LB/B rs36009281 -
BDP1 A6H8Y1 VAR_056746 p.Gly1180Ser LB/B rs715748 -
BDP1 A6H8Y1 VAR_056747 p.Phe1244Ile LB/B rs1961760 -
BDP1 A6H8Y1 VAR_056748 p.Gln1676Glu LB/B rs12187098 -
BDP1 A6H8Y1 VAR_056749 p.Asn2555Ser LB/B rs17276250 -
BECN1 Q14457 VAR_005236 p.Ile403Thr LB/B - -
BECN1 Q14457 VAR_010384 p.Ala103Val LB/B - -
BEND2 Q8NDZ0 VAR_022885 p.Phe154Leu LB/B rs17274127 -
BEND2 Q8NDZ0 VAR_050935 p.Ser188Phe LB/B rs12859329 -
BEND7 Q8N7W2 VAR_035501 p.Arg313Thr US - A breast cancer sample
BEND7 Q8N7W2 VAR_057830 p.Asn341Ser LB/B rs12247033 -
BEST1 O76090 VAR_000830 p.Thr6Pro LP/P rs28940275 Macular dystrophy, vitelliform, 2 (VMD2) [MIM:153700]
BEST1 O76090 VAR_000831 p.Val9Ala LP/P rs281865205 Macular dystrophy, vitelliform, 2 (VMD2) [MIM:153700]

BEST1 O76090 VAR_000832 p.Val9Met LP/P rs28940276 Macular dystrophy, vitelliform, 2 (VMD2) [MIM:153700]
BEST1 O76090 VAR_000833 p.Ala10Thr LP/P rs281865206 Macular dystrophy, vitelliform, 2 (VMD2) [MIM:153700]
BEST1 O76090 VAR_000834 p.Leu21Val LP/P rs281865212 Macular dystrophy, vitelliform, 2 (VMD2) [MIM:153700]
BEST1 O76090 VAR_000835 p.Trp24Cys LP/P rs281865213 Macular dystrophy, vitelliform, 2 (VMD2) [MIM:153700]
BEST1 O76090 VAR_000836 p.Arg25Gln LP/P rs281865215 Macular dystrophy, vitelliform, 2 (VMD2) [MIM:153700]
BEST1 O76090 VAR_000837 p.Arg25Trp LP/P rs281865214 Macular dystrophy, vitelliform, 2 (VMD2) [MIM:153700]
BEST1 O76090 VAR_000838 p.Ser27Arg LP/P rs281865216 Macular dystrophy, vitelliform, 2 (VMD2) [MIM:153700]
BEST1 O76090 VAR_000839 p.Gln58Leu LP/P rs281865529 Macular dystrophy, vitelliform, 2 (VMD2) [MIM:153700]
BEST1 O76090 VAR_000840 p.Leu67Val LB/B - -
BEST1 O76090 VAR_000841 p.Tyr85His LP/P rs28940274 Macular dystrophy, vitelliform, 2 (VMD2) [MIM:153700]
BEST1 O76090 VAR_000842 p.Arg92Ser LP/P rs281865224 Macular dystrophy, vitelliform, 2 (VMD2) [MIM:153700]
BEST1 O76090 VAR_000843 p.Trp93Cys LP/P rs28940273 Macular dystrophy, vitelliform, 2 (VMD2) [MIM:153700]
BEST1 O76090 VAR_000844 p.Asn99Lys LP/P rs281865227 Macular dystrophy, vitelliform, 2 (VMD2) [MIM:153700]
BEST1 O76090 VAR_000845 p.Leu100Arg LP/P rs281865228 Macular dystrophy, vitelliform, 2 (VMD2) [MIM:153700]
BEST1 O76090 VAR_000846 p.Asp104Glu LP/P rs281865232 Macular dystrophy, vitelliform, 2 (VMD2) [MIM:153700]
BEST1 O76090 VAR_000847 p.Arg141His LP/P rs121918284 Bestrophinopathy, autosomal recessive (ARB) [MIM:611809]
BEST1 O76090 VAR_000847 p.Arg141His LP/P rs121918284 Macular dystrophy, vitelliform, 2 (VMD2) [MIM:153700]
BEST1 O76090 VAR_000848 p.Ser209Asn LP/P rs281865237 Macular dystrophy, vitelliform, 2 (VMD2) [MIM:153700]
BEST1 O76090 VAR_000849 p.Arg218Cys LP/P rs281865238 Macular dystrophy, vitelliform, 2 (VMD2) [MIM:153700]
BEST1 O76090 VAR_000850 p.Arg218Gln LP/P - Macular dystrophy, vitelliform, 2 (VMD2) [MIM:153700]
BEST1 O76090 VAR_000851 p.Arg218Ser LP/P rs281865238 Macular dystrophy, vitelliform, 2 (VMD2) [MIM:153700]
BEST1 O76090 VAR_000852 p.Leu224Met LP/P rs281865242 Macular dystrophy, vitelliform, 2 (VMD2) [MIM:153700]
BEST1 O76090 VAR_000853 p.Tyr227Cys LP/P rs267606677 Retinitis pigmentosa 50 (RP50) [MIM:613194]
BEST1 O76090 VAR_000854 p.Tyr227Asn LP/P rs28941469 Macular dystrophy, vitelliform, 2 (VMD2) [MIM:153700]
BEST1 O76090 VAR_000855 p.Ser231Arg LP/P rs281865244 Macular dystrophy, vitelliform, 2 (VMD2) [MIM:153700]
BEST1 O76090 VAR_000856 p.Val235Met LP/P rs281865245 Macular dystrophy, vitelliform, 2 (VMD2) [MIM:153700]

BEST1 O76090 VAR_000857 p.Thr237Arg LP/P rs281865246 Macular dystrophy, vitelliform, 2 (VMD2) [MIM:153700]
BEST1 O76090 VAR_000858 p.Ala243Val LP/P rs28940570 Macular dystrophy, vitelliform, 2 (VMD2) [MIM:153700]
BEST1 O76090 VAR_000860 p.Pro297Ala LP/P rs1805143 Macular dystrophy, vitelliform, 2 (VMD2) [MIM:153700]
BEST1 O76090 VAR_000861 p.Gly299Glu LP/P rs28941468 Macular dystrophy, vitelliform, 2 (VMD2) [MIM:153700]
BEST1 O76090 VAR_000862 p.Glu300Lys LP/P rs281865258 Macular dystrophy, vitelliform, 2 (VMD2) [MIM:153700]
BEST1 O76090 VAR_000863 p.Asp301Glu LP/P rs281865261 Macular dystrophy, vitelliform, 2 (VMD2) [MIM:153700]
BEST1 O76090 VAR_000864 p.Asp301Asn LP/P rs281865259 Macular dystrophy, vitelliform, 2 (VMD2) [MIM:153700]
BEST1 O76090 VAR_000865 p.Phe305Ser LP/P rs281865265 Macular dystrophy, vitelliform, 2 (VMD2) [MIM:153700]
BEST1 O76090 VAR_000866 p.Ile310Thr LP/P rs281865271 Macular dystrophy, vitelliform, 2 (VMD2) [MIM:153700]
BEST1 O76090 VAR_000867 p.Val311Gly LP/P - Macular dystrophy, vitelliform, 2 (VMD2) [MIM:153700]
BEST1 O76090 VAR_000868 p.Asp312Asn LP/P rs281865277 Bestrophinopathy, autosomal recessive (ARB) [MIM:611809]
BEST1 O76090 VAR_000868 p.Asp312Asn LP/P rs281865277 Macular dystrophy, vitelliform, 2 (VMD2) [MIM:153700]
BEST1 O76090 VAR_009278 p.Glu578Val LB/B rs1800010 -
BEST1 O76090 VAR_010468 p.Ala10Val LP/P rs281865207 Macular dystrophy, vitelliform, 2 (VMD2) [MIM:153700]
BEST1 O76090 VAR_010469 p.Arg13His LP/P rs281865209 Macular dystrophy, vitelliform, 2 (VMD2) [MIM:153700]
BEST1 O76090 VAR_010470 p.Ser16Phe LP/P rs281865210 Macular dystrophy, vitelliform, 2 (VMD2) [MIM:153700]
BEST1 O76090 VAR_010471 p.Phe17Cys LP/P rs281865211 Macular dystrophy, vitelliform, 2 (VMD2) [MIM:153700]
BEST1 O76090 VAR_010472 p.Ile73Asn LP/P - Macular dystrophy, vitelliform, 2 (VMD2) [MIM:153700]
BEST1 O76090 VAR_010473 p.Leu82Val LP/P rs281865530 Macular dystrophy, vitelliform, 2 (VMD2) [MIM:153700]
BEST1 O76090 VAR_010474 p.Arg92Cys LP/P rs281865224 Macular dystrophy, vitelliform, 2 (VMD2) [MIM:153700]
BEST1 O76090 VAR_010475 p.Arg92His LP/P rs281865225 Macular dystrophy, vitelliform, 2 (VMD2) [MIM:153700]
BEST1 O76090 VAR_010476 p.Gln96His LP/P rs281865226 Macular dystrophy, vitelliform, 2 (VMD2) [MIM:153700]
BEST1 O76090 VAR_010477 p.Glu119Gln LB/B rs1805142 -
BEST1 O76090 VAR_010478 p.Gly135Ser LP/P rs281865234 Macular dystrophy, vitelliform, 2 (VMD2) [MIM:153700]
BEST1 O76090 VAR_010479 p.Ala146Lys LP/P rs1800995 Macular dystrophy, vitelliform, 2 (VMD2) [MIM:153700]
BEST1 O76090 VAR_010480 p.Thr216Ile LB/B rs281865275 -
BEST1 O76090 VAR_010481 p.Arg218His LP/P rs281865239 Macular dystrophy, vitelliform, 2 (VMD2) [MIM:153700]

BEST1 O76090 VAR_010482 p.Val235Leu LP/P rs281865245 Macular dystrophy, vitelliform, 2 (VMD2) [MIM:153700]
BEST1 O76090 VAR_010483 p.Gln293Lys LP/P rs281865250 Macular dystrophy, vitelliform, 2 (VMD2) [MIM:153700]
BEST1 O76090 VAR_010484 p.Asn296Ser LP/P rs281865255 Macular dystrophy, vitelliform, 2 (VMD2) [MIM:153700]
BEST1 O76090 VAR_010485 p.Pro297Ser LP/P rs1805143 Macular dystrophy, vitelliform, 2 (VMD2) [MIM:153700]
BEST1 O76090 VAR_010486 p.Glu300Asp LP/P rs1805144 Macular dystrophy, vitelliform, 2 (VMD2) [MIM:153700]
BEST1 O76090 VAR_010487 p.Thr307Ile LP/P rs281865269 Macular dystrophy, vitelliform, 2 (VMD2) [MIM:153700]
BEST1 O76090 VAR_010488 p.Glu525Ala LB/B rs200582915 -
BEST1 O76090 VAR_010489 p.Glu557Lys LB/B rs147192139 -
BEST1 O76090 VAR_010490 p.Thr561Ala LB/B rs281865283 -
BEST1 O76090 VAR_010491 p.Leu567Phe US rs148060787 -
BEST1 O76090 VAR_017366 p.Thr6Arg LP/P rs281865204 Macular dystrophy, vitelliform, 2 (VMD2) [MIM:153700]
BEST1 O76090 VAR_017367 p.Asn11Ile LP/P rs281865208 Macular dystrophy, vitelliform, 2 (VMD2) [MIM:153700]
BEST1 O76090 VAR_017368 p.Gly26Arg LP/P - Macular dystrophy, vitelliform, 2 (VMD2) [MIM:153700]
BEST1 O76090 VAR_017369 p.Tyr29His LP/P rs281865217 Macular dystrophy, vitelliform, 2 (VMD2) [MIM:153700]
BEST1 O76090 VAR_017370 p.Lys30Arg LP/P rs281865218 Macular dystrophy, vitelliform, 2 (VMD2) [MIM:153700]
BEST1 O76090 VAR_017371 p.Leu41Pro LP/P rs121918288 Bestrophinopathy, autosomal recessive (ARB) [MIM:611809]
BEST1 O76090 VAR_017371 p.Leu41Pro LP/P rs121918288 Macular dystrophy, vitelliform, 2 (VMD2) [MIM:153700]
BEST1 O76090 VAR_017372 p.Arg47His LP/P rs28940278 Macular dystrophy, vitelliform, 2 (VMD2) [MIM:153700]
BEST1 O76090 VAR_017373 p.Phe80Leu LP/P rs281865221 Macular dystrophy, vitelliform, 2 (VMD2) [MIM:153700]
BEST1 O76090 VAR_017374 p.Val89Ala LP/P - Macular dystrophy, vitelliform, 2 (VMD2) [MIM:153700]
BEST1 O76090 VAR_017375 p.Thr91Ile LP/P rs281865223 Macular dystrophy, vitelliform, 2 (VMD2) [MIM:153700]
BEST1 O76090 VAR_017376 p.Pro101Thr LP/P rs281865229 Macular dystrophy, vitelliform, 2 (VMD2) [MIM:153700]
BEST1 O76090 VAR_017377 p.Trp102Arg LP/P rs281865230 Macular dystrophy, vitelliform, 2 (VMD2) [MIM:153700]
BEST1 O76090 VAR_017378 p.Asp104His LP/P rs281865231 Macular dystrophy, vitelliform, 2 (VMD2) [MIM:153700]
BEST1 O76090 VAR_017379 p.Asn133Lys LP/P rs281865233 Macular dystrophy, vitelliform, 2 (VMD2) [MIM:153700]
BEST1 O76090 VAR_017380 p.Leu140Arg LP/P rs281865235 Macular dystrophy, vitelliform, 2 (VMD2) [MIM:153700]
BEST1 O76090 VAR_017381 p.Ala195Val LP/P rs200277476 Bestrophinopathy, autosomal recessive (ARB) [MIM:611809]

BEST1 O76090 VAR_017381 p.Ala195Val LP/P rs200277476 Macular dystrophy, vitelliform, 2 (VMD2) [MIM:153700]
BEST1 O76090 VAR_025731 p.Arg105Cys LB/B rs281865273 -
BEST1 O76090 VAR_025732 p.Phe113Leu LP/P rs1445469923 Macular dystrophy, vitelliform, 2 (VMD2) [MIM:153700]
BEST1 O76090 VAR_025733 p.Ile201Thr LP/P rs199529046 Macular dystrophy, vitelliform, 2 (VMD2) [MIM:153700]
BEST1 O76090 VAR_025734 p.Leu207Ile LB/B rs74653691 Macular dystrophy, vitelliform, 2 (VMD2) [MIM:153700]
BEST1 O76090 VAR_025735 p.Cys221Trp LP/P rs281865240 Macular dystrophy, vitelliform, 2 (VMD2) [MIM:153700]
BEST1 O76090 VAR_025736 p.Gly222Val LB/B rs281865241 -
BEST1 O76090 VAR_025737 p.Leu224Pro LP/P rs281865243 Macular dystrophy, vitelliform, 2 (VMD2) [MIM:153700]
BEST1 O76090 VAR_025738 p.Thr241Asn LP/P rs281865247 Macular dystrophy, vitelliform, 2 (VMD2) [MIM:153700]
BEST1 O76090 VAR_025739 p.Ala243Thr LP/P rs137853905 Macular dystrophy, vitelliform, 2 (VMD2) [MIM:153700]
BEST1 O76090 VAR_025740 p.Val275Ile LB/B rs281865276 -
BEST1 O76090 VAR_025741 p.Phe276Leu LP/P rs281865248 Macular dystrophy, vitelliform, 2 (VMD2) [MIM:153700]
BEST1 O76090 VAR_025742 p.Leu294Val LP/P rs281865251 Macular dystrophy, vitelliform, 2 (VMD2) [MIM:153700]
BEST1 O76090 VAR_025743 p.Ile295Thr LP/P rs281865253 Macular dystrophy, vitelliform, 2 (VMD2) [MIM:153700]
BEST1 O76090 VAR_025744 p.Asn296His LP/P rs281865254 Macular dystrophy, vitelliform, 2 (VMD2) [MIM:153700]
BEST1 O76090 VAR_025745 p.Phe298Ser LP/P rs281865257 Macular dystrophy, vitelliform, 2 (VMD2) [MIM:153700]
BEST1 O76090 VAR_025746 p.Asp302Gly LP/P rs281865263 Macular dystrophy, vitelliform, 2 (VMD2) [MIM:153700]
BEST1 O76090 VAR_025747 p.Asp302His LP/P rs281865262 Macular dystrophy, vitelliform, 2 (VMD2) [MIM:153700]
BEST1 O76090 VAR_025748 p.Asp302Val LP/P rs281865263 Macular dystrophy, vitelliform, 2 (VMD2) [MIM:153700]
BEST1 O76090 VAR_025749 p.Asp303Glu LP/P rs281865264 Macular dystrophy, vitelliform, 2 (VMD2) [MIM:153700]
BEST1 O76090 VAR_025750 p.Glu306Asp LP/P rs281865267 Macular dystrophy, vitelliform, 2 (VMD2) [MIM:153700]
BEST1 O76090 VAR_025751 p.Glu306Gly LP/P rs281865266 Macular dystrophy, vitelliform, 2 (VMD2) [MIM:153700]
BEST1 O76090 VAR_025752 p.Thr307Ala LP/P rs281865268 Macular dystrophy, vitelliform, 2 (VMD2) [MIM:153700]
BEST1 O76090 VAR_025753 p.Asn308Ser LP/P rs281865270 Macular dystrophy, vitelliform, 2 (VMD2) [MIM:153700]
BEST1 O76090 VAR_043493 p.Pro152Ala LP/P rs1417478879 Bestrophinopathy, autosomal recessive (ARB) [MIM:611809]
BEST1 O76090 VAR_043494 p.Val317Met LP/P rs121918287 Bestrophinopathy, autosomal recessive (ARB) [MIM:611809]

BEST1 O76090 VAR_043495 p.Met325Thr LP/P rs368387447 Bestrophinopathy, autosomal recessive (ARB) [MIM:611809]
BEST1 O76090 VAR_043496 p.Ala357Val LB/B rs17854138 -
BEST1 O76090 VAR_058273 p.Ile3Thr LP/P - Macular dystrophy, vitelliform, 2 (VMD2) [MIM:153700]
BEST1 O76090 VAR_058274 p.Val86Met LP/P rs121918289 Vitreoretinochoroidopathy, autosomal dominant (ADVIRC) [MIM:193220]
BEST1 O76090 VAR_058275 p.Tyr236Cys LP/P rs121918291 Vitreoretinochoroidopathy, autosomal dominant (ADVIRC) [MIM:193220]
BEST1 O76090 VAR_058276 p.Val239Met LP/P rs121918290 Vitreoretinochoroidopathy, autosomal dominant (ADVIRC) [MIM:193220]
BEST1 O76090 VAR_058277 p.Val242Met LP/P - Macular dystrophy, vitelliform, 2 (VMD2) [MIM:153700]
BEST1 O76090 VAR_058313 p.Gly299Ala LP/P - Macular dystrophy, vitelliform, 2 (VMD2) [MIM:153700]
BEST1 O76090 VAR_063169 p.Leu140Val LP/P rs267606678 Bestrophinopathy, autosomal recessive (ARB) [MIM:611809]
BEST1 O76090 VAR_063169 p.Leu140Val LP/P rs267606678 Retinitis pigmentosa 50 (RP50) [MIM:613194]
BEST1 O76090 VAR_063170 p.Ile205Thr LP/P rs267606680 Retinitis pigmentosa 50 (RP50) [MIM:613194]
BEST1 O76090 VAR_063171 p.Asp228Asn LP/P rs267606676 Retinitis pigmentosa 50 (RP50) [MIM:613194]
BEST1 O76090 VAR_075346 p.Leu40Pro US - Bestrophinopathy, autosomal recessive (ARB) [MIM:611809]
BEST1 O76090 VAR_075347 p.Arg202Trp LP/P rs765998048 Bestrophinopathy, autosomal recessive (ARB) [MIM:611809]
BEST3 Q8N1M1 VAR_048409 p.Tyr43His LB/B rs1025016 -
BEST3 Q8N1M1 VAR_048410 p.Glu622Gly LB/B rs17106884 -
BEST4 Q8NFU0 VAR_048411 p.Tyr62Cys LB/B rs16832245 -
BEST4 Q8NFU0 VAR_048412 p.Tyr217Ser LB/B rs16832242 -
BEST4 Q8NFU0 VAR_048413 p.Gln331Glu LB/B rs16832241 -
BEST4 Q8NFU0 VAR_048414 p.Arg402Leu LB/B rs16832239 -
BEX1 Q9HBH7 VAR_025756 p.Ala40Val LB/B rs709036 -
BEX1 Q9HBH7 VAR_025757 p.Met66Ile LB/B rs1045082 -
BEX1 Q9HBH7 VAR_042667 p.Val9Leu LB/B rs3174500 -
BEX1 Q9HBH7 VAR_042668 p.Ser11Asn LB/B rs1045058 -
BEX1 Q9HBH7 VAR_042669 p.Ser13Ile LB/B rs1045061 -
BEX1 Q9HBH7 VAR_042670 p.Met14Val LB/B rs1045063 -
BEX1 Q9HBH7 VAR_042671 p.Ala17Val LB/B rs1045065 -
BFAR Q9NZS9 VAR_052075 p.Met140Arg LB/B rs11546303 -
BFAR Q9NZS9 VAR_052076 p.Arg245His LB/B rs35377618 -
BFSP1 Q12934 VAR_024492 p.Gly345Ser LB/B rs6080719 -
BFSP1 Q12934 VAR_036683 p.Asp656Glu LB/B rs16999317 -
BFSP1 Q12934 VAR_078861 p.Asp348Asn LP/P rs1085307126 Cataract 33, multiple types (CTRCT33) [MIM:611391]
BFSP2 Q13515 VAR_012164 p.Arg287Trp LP/P rs104893685 Cataract 12, multiple types (CTRCT12) [MIM:611597]
BGLAP P02818 VAR_038743 p.Arg94Gln LB/B rs34702397 -
BGN P21810 VAR_036605 p.Arg266Thr US - A breast cancer sample
BGN P21810 VAR_036606 p.Lys288Asn US - A breast cancer sample
BGN P21810 VAR_076590 p.Lys147Glu LP/P rs879255604 Spondyloepimetaphyseal dysplasia, X-linked (SEMDX) [MIM:300106]
BGN P21810 VAR_076591 p.Gly259Val LP/P rs879255605 Spondyloepimetaphyseal dysplasia, X-linked (SEMDX) [MIM:300106]

BGN P21810 VAR_078028 p.Gly80Ser US rs886037825 Meester-Loeys syndrome (MRLS) [MIM:300989]
BGN P21810 VAR_078029 p.Gln303Pro US rs886037824 Meester-Loeys syndrome (MRLS) [MIM:300989]
BHLHA9 Q7RTU4 VAR_073333 p.Asn71Asp LP/P rs672601337 Syndactyly, mesoaxial synostotic, with phalangeal reduction (MSSD) [MIM:609432]
BHLHA9 Q7RTU4 VAR_073334 p.Arg73Pro LP/P rs672601338 Syndactyly, mesoaxial synostotic, with phalangeal reduction (MSSD) [MIM:609432]
BHLHA9 Q7RTU4 VAR_073335 p.Arg75Leu LP/P rs672601339 Syndactyly, mesoaxial synostotic, with phalangeal reduction (MSSD) [MIM:609432]
BHLHA9 Q7RTU4 VAR_077086 p.Glu74Leu US rs886037856 Camptosynpolydactyly, complex (CCSPD) [MIM:607539]
BHLHB9 Q6PI77 VAR_049266 p.Ser132Gly LB/B rs2179675 -
BHLHB9 Q6PI77 VAR_049267 p.Cys318Arg LB/B rs4514179 -
BHLHE22 Q8NFJ8 VAR_061255 p.Ser28Ala LB/B rs7016250 -
BHLHE41 Q9C0J9 VAR_063259 p.Pro384Arg LB/B rs121912617 -
BHLHE41 Q9C0J9 VAR_082585 p.Tyr362His LB/B rs1591838266 -
BHLHE41 Q9C0J9 VAR_082586 p.Pro384Gln LB/B rs121912617 -
BHMT Q93088 VAR_015886 p.Arg239Gln LB/B rs3733890 -
BHMT Q93088 VAR_061345 p.Gly199Ser LB/B rs59866108 -
BICC1 Q9H694 VAR_029658 p.Gly8Asp LB/B rs7905025 -
BICC1 Q9H694 VAR_033542 p.Ser943Pro LB/B rs4948550 -
BICC1 Q9H694 VAR_060133 p.Asn945Ser LB/B rs7895817 -
BICC1 Q9H694 VAR_066759 p.Asn711Thr LB/B rs138916713 -
BICC1 Q9H694 VAR_066760 p.Glu932Gly LP/P rs387907124 Renal dysplasia, cystic (CYSRD) [MIM:601331]
BICC1 Q9H694 VAR_072077 p.Met270Val US rs753582128 -
BICD1 Q96G01 VAR_069060 p.Thr778Ala LB/B rs200845476 -
BICD2 Q8TD16 VAR_070111 p.Lys90Arg LB/B rs61754130 -
BICD2 Q8TD16 VAR_070112 p.Ser107Leu LP/P rs398123028 Spinal muscular atrophy, lower extremity-predominant 2A, childhood onset, autosomal dominant (SMALED2A) [MIM:615290]
BICD2 Q8TD16 VAR_070113 p.Asn188Thr LP/P rs398123029 Spinal muscular atrophy, lower extremity-predominant 2A, childhood onset, autosomal dominant (SMALED2A) [MIM:615290]
BICD2 Q8TD16 VAR_070114 p.Ile189Phe LP/P - Spinal muscular atrophy, lower extremity-predominant 2A, childhood onset, autosomal dominant (SMALED2A) [MIM:615290]
BICD2 Q8TD16 VAR_070115 p.Arg501Pro LP/P rs398123032 Spinal muscular atrophy, lower extremity-predominant 2A, childhood onset, autosomal dominant (SMALED2A) [MIM:615290]
BICD2 Q8TD16 VAR_070116 p.Lys508Thr LP/P rs398123031 Spinal muscular atrophy, lower extremity-predominant 2A, childhood onset, autosomal dominant (SMALED2A) [MIM:615290]
BICD2 Q8TD16 VAR_070117 p.Thr703Met LP/P rs371707778 Spinal muscular atrophy, lower extremity-predominant 2A, childhood onset, autosomal dominant (SMALED2A) [MIM:615290]
BICD2 Q8TD16 VAR_070118 p.Glu774Gly LP/P rs398123030 Spinal muscular atrophy, lower extremity-predominant 2A, childhood onset, autosomal dominant (SMALED2A) [MIM:615290]
BICD2 Q8TD16 VAR_081854 p.Gln194Arg LP/P rs1564061982 Spinal muscular atrophy, lower extremity-predominant, 2B, prenatal onset, autosomal dominant (SMALED2B) [MIM:618291]
BICD2 Q8TD16 VAR_081855 p.Cys542Trp LP/P - Spinal muscular atrophy, lower extremity-predominant, 2B, prenatal onset, autosomal dominant (SMALED2B) [MIM:618291]
BICD2 Q8TD16 VAR_081857 p.Arg694Cys LP/P rs797045412 Spinal muscular atrophy, lower extremity-predominant, 2B, prenatal onset, autosomal dominant (SMALED2B) [MIM:618291]
BICDL2 A1A5D9 VAR_034978 p.Gln273Arg LB/B rs2244494 -
BICDL2 A1A5D9 VAR_060543 p.Gln55Glu LB/B rs7204908 -
BICRA Q9NZM4 VAR_059665 p.Thr1044Ala LB/B rs13346368 -

BICRA Q9NZM4 VAR_061663 p.Pro683Ser LB/B rs3745762 -
BID P55957 VAR_018845 p.Ser10Gly LB/B rs8190315 -
BID P55957 VAR_025332 p.His162Gln LB/B rs17853595 -
BID P55957 VAR_061041 p.Met194Thr LB/B rs59225839 -
BIK Q13323 VAR_029179 p.Glu19Lys LB/B rs4988415 -
BIK Q13323 VAR_029180 p.Leu148Pro LB/B rs11574527 -
BIK Q13323 VAR_048420 p.Thr26Ile LB/B rs11090143 -
BIN1 O00499 VAR_037425 p.Lys35Asn LP/P rs121909273 Myopathy, centronuclear, 2 (CNM2) [MIM:255200]
BIN1 O00499 VAR_037426 p.Asp151Asn LP/P rs121909274 Myopathy, centronuclear, 2 (CNM2) [MIM:255200]
BIN1 O00499 VAR_081081 p.Arg24Cys LP/P - -
BIN1 O00499 VAR_081082 p.Arg145Cys LP/P rs1249621033 Myopathy, centronuclear, 2 (CNM2) [MIM:255200]
BIN1 O00499 VAR_081083 p.Arg154Gln LP/P rs267606681 Myopathy, centronuclear, 2 (CNM2) [MIM:255200]
BIN1 O00499 VAR_081084 p.Arg234Cys LP/P rs777176261 Myopathy, centronuclear, 2 (CNM2) [MIM:255200]
BIN2 Q9UBW5 VAR_028883 p.Ser48Asn LB/B rs7312857 -
BIN2 Q9UBW5 VAR_028884 p.Asn529Asp LB/B rs7954976 -
BIRC2 Q13490 VAR_025016 p.Met453Val LB/B rs370745983 -
BIRC2 Q13490 VAR_025017 p.Ala506Val LB/B rs34510872 -
BIRC2 Q13490 VAR_025018 p.Pro549Ser LB/B rs35494784 -
BIRC2 Q13490 VAR_049535 p.Met453Ile LB/B rs34749508 -
BIRC3 Q13489 VAR_021069 p.Lys260Arg LB/B rs2276113 -
BIRC3 Q13489 VAR_021070 p.Arg401Lys LB/B rs17881197 -
BIRC3 Q13489 VAR_049536 p.Val386Met LB/B rs12222256 -
BIRC5 O15392 VAR_021071 p.Lys129Glu LB/B rs2071214 -
BIRC7 Q96CA5 VAR_020253 p.Glu223Gln LB/B rs1077019 -
BIRC8 Q96P09 VAR_028282 p.His196Tyr LB/B rs8109165 -
BIRC8 Q96P09 VAR_055944 p.Ala156Thr LB/B rs35880972 -
BIRC8 Q96P09 VAR_055945 p.Leu165Ser LB/B rs34092035 -
BIRC8 Q96P09 VAR_055946 p.Ala225Thr LB/B rs35700345 -
BIRC8 Q96P09 VAR_055947 p.Ala225Val LB/B rs34683072 -
BLCAP P62952 VAR_068908 p.Tyr2Cys LB/B rs11557677 -
BLCAP P62952 VAR_068909 p.Gln5Arg US - -
BLCAP P62952 VAR_068910 p.Lys15Arg LB/B rs11557676 -
BLID Q8IZY5 VAR_056750 p.Ala76Asp LB/B rs7116084 -
BLK P51451 VAR_041672 p.Thr48Ile LB/B rs35339715 -
BLK P51451 VAR_041673 p.Ala71Thr LB/B rs55758736 -
BLM P54132 VAR_006901 p.Gln672Arg LP/P rs747281324 Bloom syndrome (BLM) [MIM:210900]
BLM P54132 VAR_006902 p.Thr843Ile LP/P rs137853152 Bloom syndrome (BLM) [MIM:210900]
BLM P54132 VAR_006903 p.Cys1055Ser LP/P rs367543029 Bloom syndrome (BLM) [MIM:210900]
BLM P54132 VAR_009138 p.Gly891Glu LP/P rs763471784 Bloom syndrome (BLM) [MIM:210900]
BLM P54132 VAR_009139 p.Cys901Tyr LP/P rs758311406 Bloom syndrome (BLM) [MIM:210900]
BLM P54132 VAR_009140 p.Cys1036Phe LP/P rs137853153 Bloom syndrome (BLM) [MIM:210900]
BLM P54132 VAR_014912 p.Ser1209Thr LB/B rs1801256 -
BLM P54132 VAR_016032 p.Ile841Thr LP/P rs767086502 Bloom syndrome (BLM) [MIM:210900]
BLM P54132 VAR_016033 p.Cys878Arg LP/P - Bloom syndrome (BLM) [MIM:210900]
BLM P54132 VAR_022295 p.Lys137Arg LB/B rs28384988 -

BLM	P54132	VAR_022296	p.Thr298Met	LB/B	rs28384991	-
BLM	P54132	VAR_022297	p.Arg591Gln	LB/B	rs28385012	-
BLM	P54132	VAR_022298	p.Pro868Leu	LB/B	rs2227935	-
BLM	P54132	VAR_022299	p.Val1205Ile	LB/B	rs28385141	-
BLM	P54132	VAR_022300	p.Glu1213Lys	LB/B	rs28385142	-
BLM	P54132	VAR_022301	p.Val1321Ile	LB/B	rs7167216	-
BLM	P54132	VAR_051731	p.Ala1043Asp	LB/B	rs2229035	-
BLMH	Q13867	VAR_010896	p.Ile443Val	LB/B	rs1050565	-
BLOC1S2	Q6QNY1	VAR_054068	p.Ser13Arg	LB/B	rs33965491	-
BLVRA	P53004	VAR_014851	p.Gln56Arg	LB/B	rs1050916	-
BLVRA	P53004	VAR_019230	p.Ala3Thr	LB/B	rs699512	-
BLVRA	P53004	VAR_019231	p.Leu37Val	LB/B	rs17245918	-
BLVRB	P30043	VAR_019168	p.Arg46Gln	LB/B	rs11547746	-
BLZF1	Q9H2G9	VAR_028142	p.Gln40Arg	LB/B	rs1028180	-
BLZF1	Q9H2G9	VAR_028143	p.Arg196Gln	LB/B	rs1064274	-
BMI1	P35226	VAR_052087	p.Cys18Tyr	LB/B	rs1042059	-
BMP1	P13497	VAR_036141	p.Asp45His	US	-	A breast cancer sample
BMP1	P13497	VAR_051584	p.Val719Ile	LB/B	rs11996036	-
BMP1	P13497	VAR_067224	p.Phe249Leu	LP/P	rs398122891	Osteogenesis imperfecta 13 (OI13) [MIM:614856]
BMP1	P13497	VAR_069096	p.Gly12Arg	LP/P	rs318240762	Osteogenesis imperfecta 13 (OI13) [MIM:614856]
BMP1	P13497	VAR_072248	p.Met270Val	LP/P	rs786205219	Osteogenesis imperfecta 13 (OI13) [MIM:614856]
BMP10	O95393	VAR_052572	p.Thr200Ser	LB/B	rs2231342	-
BMP10	O95393	VAR_052573	p.Asn250Lys	LB/B	rs2231345	-
BMP15	O95972	VAR_021195	p.Tyr235Cys	LP/P	rs104894765	Ovarian dysgenesis 2 (ODG2) [MIM:300510]
BMP15	O95972	VAR_058974	p.Ser5Arg	LB/B	rs113099187	-
BMP15	O95972	VAR_058975	p.Arg61Gln	LP/P	-	Premature ovarian failure 4 (POF4) [MIM:300510]
BMP15	O95972	VAR_058976	p.Arg61Trp	LP/P	rs144392417	Premature ovarian failure 4 (POF4) [MIM:300510]
BMP15	O95972	VAR_058977	p.Arg68Trp	LP/P	rs104894763	Premature ovarian failure 4 (POF4) [MIM:300510]
BMP15	O95972	VAR_058978	p.Arg76Cys	LP/P	rs104894766	Premature ovarian failure 4 (POF4) [MIM:300510]
BMP15	O95972	VAR_058979	p.Arg76His	LP/P	rs1557279925	Premature ovarian failure 4 (POF4) [MIM:300510]
BMP15	O95972	VAR_058980	p.Asn103Ser	LB/B	rs41308602	-
BMP15	O95972	VAR_058981	p.Arg138His	LP/P	rs371418883	Premature ovarian failure 4 (POF4) [MIM:300510]
BMP15	O95972	VAR_058982	p.Leu148Pro	LP/P	rs114823607	Premature ovarian failure 4 (POF4) [MIM:300510]
BMP15	O95972	VAR_058983	p.Ala180Phe	US	-	-
BMP15	O95972	VAR_058984	p.Ala180Thr	US	rs104894767	Premature ovarian failure 4 (POF4) [MIM:300510]
BMP15	O95972	VAR_058985	p.Asn196Lys	LP/P	-	Premature ovarian failure 4 (POF4) [MIM:300510]
BMP15	O95972	VAR_058986	p.Arg206His	LP/P	rs782516193	Premature ovarian failure 4 (POF4) [MIM:300510]
BMP15	O95972	VAR_058987	p.Trp221Arg	LP/P	rs375284458	Premature ovarian failure 4 (POF4) [MIM:300510]
BMP15	O95972	VAR_058988	p.Ile243Val	LP/P	rs782379521	Premature ovarian failure 4 (POF4) [MIM:300510]

BMP15 O95972 VAR_066932 p.His200Tyr LB/B rs202165852 -
BMP15 O95972 VAR_066933 p.Arg329Cys LP/P rs782375794 Premature ovarian failure 4 (POF4) [MIM:300510]
BMP2 P12643 VAR_020061 p.Ser37Ala LB/B rs2273073 -
BMP2 P12643 VAR_020062 p.Ala106Thr LB/B rs2273074 -
BMP2 P12643 VAR_024232 p.Arg190Ser LB/B rs235768 -
BMP2 P12643 VAR_052568 p.Pro77Ser LB/B rs36105541 -
BMP2 P12643 VAR_052569 p.Leu161Ser LB/B rs34183594 -
BMP2 P12643 VAR_052570 p.Asp387Gly LB/B rs11545591 -
BMP2K Q9NSY1 VAR_040388 p.Val68Met US rs770167074 A lung squamous cell carcinoma sample
BMP2K Q9NSY1 VAR_040389 p.Asp212Val LB/B rs56143363 -
BMP2K Q9NSY1 VAR_040390 p.Arg288His LB/B rs55782848 -
BMP2K Q9NSY1 VAR_051618 p.Gly405Ser LB/B rs2288255 -
BMP2K Q9NSY1 VAR_051619 p.Thr1002Ser LB/B rs12507099 -
BMP2K Q9NSY1 VAR_059765 p.Gln486His LB/B rs2114202 -
BMP3 P12645 VAR_020063 p.Arg192Gln LB/B rs3733549 -
BMP3 P12645 VAR_047418 p.Gln176Lys LB/B rs34213771 -
BMP3 P12645 VAR_047419 p.Gln176Leu LB/B rs34847147 -
BMP3 P12645 VAR_047420 p.Leu205Phe LB/B rs6831040 -
BMP3 P12645 VAR_047421 p.Thr222Met LB/B rs34505126 -
BMP4 P12644 VAR_016174 p.Val152Ala LB/B rs17563 -
BMP4 P12644 VAR_043531 p.Ser91Cys LP/P rs121912767 Non-syndromic orofacial cleft 11 (OFC11) [MIM:600625]
BMP4 P12644 VAR_043532 p.Glu93Gly LP/P rs121912765 Microphthalmia, syndromic, 6 (MCOPS6) [MIM:607932]
BMP4 P12644 VAR_043533 p.Thr116Ser LB/B rs750427266 -
BMP4 P12644 VAR_043534 p.Asn150Lys LB/B rs767216159 -
BMP4 P12644 VAR_043535 p.Thr225Ala LB/B rs144556455 -
BMP4 P12644 VAR_043536 p.Arg226Trp LB/B rs140590144 -
BMP4 P12644 VAR_043537 p.Ser367Thr LB/B rs1320581580 -
BMP4 P12644 VAR_058314 p.Thr102Ala LB/B rs202159001 -
BMP4 P12644 VAR_058315 p.Arg162Gln LP/P rs770493925 Non-syndromic orofacial cleft 11 (OFC11) [MIM:600625]
BMP4 P12644 VAR_058316 p.Gly168Ala LB/B - -
BMP4 P12644 VAR_058317 p.Arg287His LP/P rs121912768 Non-syndromic orofacial cleft 11 (OFC11) [MIM:600625]
BMP4 P12644 VAR_058318 p.Ala346Val LP/P rs121912766 Non-syndromic orofacial cleft 11 (OFC11) [MIM:600625]
BMP5 P22003 VAR_047054 p.His2Tyr LB/B rs9475437 -
BMP5 P22003 VAR_061896 p.Asn121Ser LB/B rs35124644 -
BMP6 P22004 VAR_036200 p.Ala343Asp US - A colorectal cancer sample
BMP6 P22004 VAR_036201 p.Pro476Leu US rs909733732 A colorectal cancer sample
BMP6 P22004 VAR_047055 p.Arg257Cys LB/B rs10458105 -
BMP7 P18075 VAR_064058 p.Leu198Pro LB/B rs376798352 -
BMP7 P18075 VAR_064059 p.Asn321Ser LB/B rs61733438 -
BMP8A Q7Z5Y6 VAR_052571 p.Arg293His LB/B rs6525 -
BMP8A Q7Z5Y6 VAR_059859 p.Met84Val LB/B rs4660269 -
BMP8B P34820 VAR_014474 p.His293Arg LB/B rs17403490 -
BMPER Q8N8U9 VAR_028166 p.Arg555Trp LB/B rs10249320 -

BMPER Q8N8U9 VAR_065823 p.Pro370Leu LP/P rs387906993 Diaphanospondylodysostosis (DSD) [MIM:608022]
BMPR1A P36894 VAR_015533 p.Cys124Arg LP/P rs199476087 Juvenile polyposis syndrome (JPS) [MIM:174900]
BMPR1A P36894 VAR_015534 p.Ala338Asp LP/P rs199476086 Juvenile polyposis syndrome (JPS) [MIM:174900]
BMPR1A P36894 VAR_015535 p.Cys376Tyr LP/P rs199476088 Juvenile polyposis syndrome (JPS) [MIM:174900]
BMPR1A P36894 VAR_022828 p.Tyr62Asp LP/P - Juvenile polyposis syndrome (JPS) [MIM:174900]
BMPR1A P36894 VAR_022829 p.Cys82Tyr LP/P - Juvenile polyposis syndrome (JPS) [MIM:174900]
BMPR1A P36894 VAR_022830 p.Cys130Arg LP/P rs1131691168 Juvenile polyposis syndrome (JPS) [MIM:174900]
BMPR1A P36894 VAR_022831 p.Arg443Cys LP/P rs35619497 Juvenile polyposis syndrome (JPS) [MIM:174900]
BMPR1A P36894 VAR_022832 p.Met470Thr LP/P rs199476089 Juvenile polyposis syndrome (JPS) [MIM:174900]
BMPR1A P36894 VAR_041397 p.Pro2Thr LB/B rs11528010 -
BMPR1A P36894 VAR_041398 p.Phe58Tyr US - A renal clear cell carcinoma sample
BMPR1A P36894 VAR_041399 p.Val450Met LB/B rs55932635 -
BMPR1A P36894 VAR_041400 p.Arg486Gln US rs752802257 A gastric adenocarcinoma sample
BMPR1A P36894 VAR_077353 p.Met460Thr US rs758309022 -
BMPR1B O00238 VAR_023819 p.Ile200Lys LP/P rs121434417 Brachydactyly A2 (BDA2) [MIM:112600]
BMPR1B O00238 VAR_023820 p.Arg486Trp LP/P rs121434418 Brachydactyly A2 (BDA2) [MIM:112600]
BMPR1B O00238 VAR_037967 p.Arg486Gln LP/P rs121434419 Brachydactyly A2 (BDA2) [MIM:112600]
BMPR1B O00238 VAR_041401 p.Arg31His US rs200035802 A gastric adenocarcinoma sample
BMPR1B O00238 VAR_041402 p.Arg149Trp LB/B rs34231464 -
BMPR1B O00238 VAR_041403 p.Arg224His LB/B rs35973133 -
BMPR1B O00238 VAR_041404 p.Asp297Asn US - A metastatic melanoma sample
BMPR1B O00238 VAR_041405 p.Arg371Gln LB/B rs34970181 -
BMPR1B O00238 VAR_075520 p.Arg31Cys US rs745854387 Acromesomelic dysplasia, Demirhan type (AMDD) [MIM:609441]
BMPR1B O00238 VAR_075521 p.Cys53Arg LP/P rs863225041 Acromesomelic dysplasia, Demirhan type (AMDD) [MIM:609441]
BMPR1B O00238 VAR_076406 p.Lys325Asn LP/P rs869025614 Brachydactyly A1, D (BDA1D) [MIM:616849]
BMPR2 Q13873 VAR_013670 p.Cys60Tyr LP/P rs1085307172 Pulmonary hypertension, primary, 1 (PPH1) [MIM:178600]
BMPR2 Q13873 VAR_013671 p.Cys117Tyr LP/P rs1085307215 Pulmonary hypertension, primary, 1 (PPH1) [MIM:178600]
BMPR2 Q13873 VAR_013672 p.Cys118Trp LP/P rs137852743 Pulmonary hypertension, primary, 1 (PPH1) [MIM:178600]
BMPR2 Q13873 VAR_013673 p.Cys123Arg LP/P rs137852750 Pulmonary hypertension, primary, 1 (PPH1) [MIM:178600]
BMPR2 Q13873 VAR_013674 p.Cys123Ser LP/P rs137852750 Pulmonary hypertension, primary, 1 (PPH1) [MIM:178600]
BMPR2 Q13873 VAR_013675 p.Glu224Asp LB/B rs754343081 -
BMPR2 Q13873 VAR_013676 p.Cys347Tyr LP/P rs137852744 Pulmonary hypertension, primary, 1 (PPH1) [MIM:178600]
BMPR2 Q13873 VAR_013677 p.Cys420Arg LP/P rs1085307324 Pulmonary hypertension, primary, 1 (PPH1) [MIM:178600]

BMPR2 Q13873 VAR_013678 p.Cys483Arg LP/P rs1085307354 Pulmonary hypertension, primary, 1 (PPH1) [MIM:178600]
BMPR2 Q13873 VAR_013679 p.Asp485Gly LP/P rs137852745 Pulmonary hypertension, primary, 1 (PPH1) [MIM:178600]
BMPR2 Q13873 VAR_013680 p.Arg491Gln LP/P rs137852749 Pulmonary hypertension, primary, 1 (PPH1) [MIM:178600]
BMPR2 Q13873 VAR_013681 p.Arg491Trp LP/P rs137852746 Pulmonary hypertension, primary, 1 (PPH1) [MIM:178600]
BMPR2 Q13873 VAR_013682 p.Lys512Thr LP/P rs1085307364 Pulmonary hypertension, primary, 1 (PPH1) [MIM:178600]
BMPR2 Q13873 VAR_013683 p.Asn519Lys LP/P rs1085307365 Pulmonary hypertension, primary, 1 (PPH1) [MIM:178600]
BMPR2 Q13873 VAR_019996 p.Ser775Asn LB/B rs2228545 -
BMPR2 Q13873 VAR_033109 p.Gln82His LP/P rs1085307185 Pulmonary hypertension, primary, 1 (PPH1) [MIM:178600]
BMPR2 Q13873 VAR_033110 p.Gly182Asp LP/P rs137852754 Pulmonary hypertension, primary, 1 (PPH1) [MIM:178600]
BMPR2 Q13873 VAR_033111 p.Arg899Pro LP/P rs137852752 Pulmonary hypertension, primary, 1 (PPH1) [MIM:178600]
BMPR2 Q13873 VAR_073041 p.Tyr67Cys LP/P rs1085307177 Pulmonary hypertension, primary, 1 (PPH1) [MIM:178600]
BMPR2 Q13873 VAR_073042 p.Ser863Asn LP/P rs1006246556 Pulmonary hypertension, primary, 1 (PPH1) [MIM:178600]
BMPR2 Q13873 VAR_079588 p.Ser64Arg US - Pulmonary hypertension, primary, 1 (PPH1) [MIM:178600]
BMPR2 Q13873 VAR_079589 p.Ile77Leu US - Pulmonary hypertension, primary, 1 (PPH1) [MIM:178600]
BMPR2 Q13873 VAR_079590 p.Cys84Phe LP/P rs1085307197 Pulmonary hypertension, primary, 1 (PPH1) [MIM:178600]
BMPR2 Q13873 VAR_079591 p.His87Tyr US - Pulmonary hypertension, primary, 1 (PPH1) [MIM:178600]
BMPR2 Q13873 VAR_079592 p.Gln92Leu US - Pulmonary hypertension, primary, 1 (PPH1) [MIM:178600]
BMPR2 Q13873 VAR_079593 p.Gln109His US - Pulmonary hypertension, primary, 1 (PPH1) [MIM:178600]
BMPR2 Q13873 VAR_079594 p.Pro138Ala US - Pulmonary hypertension, primary, 1 (PPH1) [MIM:178600]
BMPR2 Q13873 VAR_079595 p.Ala162Pro US - Pulmonary hypertension, primary, 1 (PPH1) [MIM:178600]
BMPR2 Q13873 VAR_079597 p.Arg248Gly US - Pulmonary hypertension, primary, 1 (PPH1) [MIM:178600]
BMPR2 Q13873 VAR_079598 p.Asp264Asn US - Pulmonary hypertension, primary, 1 (PPH1) [MIM:178600]
BMPR2 Q13873 VAR_079600 p.Val341Met US rs767882551 Pulmonary hypertension, primary, 1 (PPH1) [MIM:178600]
BMPR2 Q13873 VAR_079601 p.Lys467Arg US - Pulmonary hypertension, primary, 1 (PPH1) [MIM:178600]
BMS1 Q14692 VAR_057503 p.Arg237His LB/B rs2272881 -
BMS1 Q14692 VAR_057504 p.Ser552Pro LB/B rs3814621 -

BMS1 Q14692 VAR_057505 p.Lys652Arg LB/B rs787795 -
BMS1 Q14692 VAR_057506 p.Met884Val LB/B rs2419109 -
BMS1 Q14692 VAR_057507 p.Val1141Ile LB/B rs12764004 -
BMS1 Q14692 VAR_072539 p.Arg930His LP/P rs587777706 Aplasia cutis congenita, non-syndromic (ACC) [MIM:107600]
BMX P51813 VAR_041674 p.Ser284Leu LB/B rs35353387 -
BMX P51813 VAR_041675 p.Arg670Trp US rs1029888196 A lung large cell carcinoma sample
BNC1 Q01954 VAR_083487 p.Leu532Pro US rs149100994 Premature ovarian failure 16 (POF16) [MIM:618723]
BNC2 Q6ZN30 VAR_033543 p.Thr782Ala LB/B rs3739714 -
BNC2 Q6ZN30 VAR_052707 p.Leu550Val LB/B rs4961490 -
BNC2 Q6ZN30 VAR_083492 p.Thr158Ile US rs144242525 Lower urinary tract obstruction, congenital (LUTO) [MIM:618612]
BNC2 Q6ZN30 VAR_083493 p.Glu346Gln US rs945575406 Lower urinary tract obstruction, congenital (LUTO) [MIM:618612]
BNC2 Q6ZN30 VAR_083495 p.His888Arg LP/P rs1563774686 Lower urinary tract obstruction, congenital (LUTO) [MIM:618612]
BNIP1 Q12981 VAR_019169 p.Gln14His LB/B rs5745100 -
BNIP2 Q12982 VAR_018837 p.Ser24Thr LB/B rs6151509 -
BNIP5 P0C671 VAR_056808 p.Leu582Val LB/B rs743852 -
BNIPL Q7Z465 VAR_051917 p.Ser65Asn LB/B rs12068365 -
BNIPL Q7Z465 VAR_051918 p.Ser226Asn LB/B rs12068365 -
BOC Q9BWV1 VAR_033600 p.Lys883Asn LB/B rs35536878 -
BOC Q9BWV1 VAR_033601 p.Gln915His LB/B rs3814405 -
BOC Q9BWV1 VAR_035503 p.Val713Met US rs367589886 A breast cancer sample
BOD1L1 Q8NFC6 VAR_035220 p.Thr429Met LB/B rs2035820 -
BOD1L1 Q8NFC6 VAR_035221 p.Leu650Ile LB/B rs1971278 -
BOD1L1 Q8NFC6 VAR_035222 p.Ala1369Gly LB/B rs17745712 -
BOD1L1 Q8NFC6 VAR_035223 p.Thr1448Ala LB/B rs17745676 -
BOD1L1 Q8NFC6 VAR_035224 p.Thr1515Ala LB/B rs16888885 -
BOD1L1 Q8NFC6 VAR_035225 p.Val1645Ile LB/B rs17807493 -
BOD1L1 Q8NFC6 VAR_035226 p.Gly2361Ser LB/B rs3822227 -
BOD1L1 Q8NFC6 VAR_035227 p.Pro2396Leu LB/B rs3733557 -
BOD1L1 Q8NFC6 VAR_036124 p.Ser246Ile US - A breast cancer sample
BOD1L1 Q8NFC6 VAR_061166 p.Val2944Met LB/B rs28538279 -
BOLA1 Q9Y3E2 VAR_033630 p.Gly98Ala LB/B rs1044808 -
BOLA3 Q53S33 VAR_076180 p.His96Arg LP/P rs148674363 Multiple mitochondrial dysfunctions syndrome 2 with hyperglycinemia (MMDS2) [MIM:614299]
BOLA3 Q53S33 VAR_077911 p.Ile67Asn LP/P rs550855238 Multiple mitochondrial dysfunctions syndrome 2 with hyperglycinemia (MMDS2) [MIM:614299]
BORA Q6PGQ7 VAR_030110 p.Ser210Leu LB/B rs9543107 -
BORA Q6PGQ7 VAR_030111 p.Ser308Phe LB/B rs1146858 -
BORCS5 Q969J3 VAR_038840 p.Ser41Cys LB/B rs3741795 -
BORCS5 Q969J3 VAR_038841 p.Asp191Asn LB/B rs3751262 -
BPGM P07738 VAR_065367 p.Arg62Gln LP/P rs751972865 Erythrocytosis, familial, 8 (ECYT8) [MIM:222800]
BPGM P07738 VAR_065368 p.Arg90Cys LP/P rs121964925 Erythrocytosis, familial, 8 (ECYT8) [MIM:222800]
BPI P17213 VAR_018401 p.Ala16Val LB/B rs1341023 -
BPI P17213 VAR_018402 p.Ala196Val LB/B rs5743509 -
BPI P17213 VAR_018403 p.Glu216Lys LB/B rs4358188 -

BPI	P17213	VAR_049728	p.Ala12Thr	LB/B	rs5743497	-
BPI	P17213	VAR_049729	p.Ala12Val	LB/B	rs5743498	-
BPI	P17213	VAR_049730	p.Arg90Cys	LB/B	rs5743500	-
BPI	P17213	VAR_049732	p.Glu140Gln	LB/B	rs5743506	-
BPI	P17213	VAR_049733	p.Ala280Val	LB/B	rs5741804	-
BPI	P17213	VAR_049734	p.Val377Ile	LB/B	rs5743524	-
BPI	P17213	VAR_049735	p.Asn404Asp	LB/B	rs5741809	-
BPI	P17213	VAR_049736	p.Lys451Glu	LB/B	rs5743542	-
BPIFA2	Q96DR5	VAR_049751	p.Gly43Arg	LB/B	rs6059139	-
BPIFA2	Q96DR5	VAR_049752	p.Lys113Glu	LB/B	rs17304572	-
BPIFA2	Q96DR5	VAR_049753	p.Arg221Cys	LB/B	rs6120140	-
BPIFA3	Q9BQP9	VAR_049754	p.Ala41Glu	LB/B	rs17124391	-
BPIFA3	Q9BQP9	VAR_049755	p.Val136Ile	LB/B	rs3818222	-
BPIFB1	Q8TDL5	VAR_016756	p.Ile84Val	LB/B	rs1078761	-
BPIFB1	Q8TDL5	VAR_016757	p.Ser479Thr	LB/B	rs1999663	-
BPIFB1	Q8TDL5	VAR_023361	p.Ser298Pro	LB/B	rs6120221	-
BPIFB1	Q8TDL5	VAR_023362	p.Ser313Ile	LB/B	rs6120222	-
BPIFB1	Q8TDL5	VAR_049747	p.Thr140Ala	LB/B	rs34578060	-
BPIFB1	Q8TDL5	VAR_049748	p.Val284Met	LB/B	rs6141383	-
BPIFB1	Q8TDL5	VAR_049749	p.Asp287His	LB/B	rs34548457	-
BPIFB1	Q8TDL5	VAR_049750	p.Thr464Ser	LB/B	rs17856249	-
BPIFB2	Q8N4F0	VAR_024515	p.Lys31Met	LB/B	rs6088066	-
BPIFB2	Q8N4F0	VAR_036550	p.Ala20Val	US	-	A colorectal cancer sample
BPIFB2	Q8N4F0	VAR_049740	p.Ala63Val	LB/B	rs34128772	-
BPIFB3	P59826	VAR_049742	p.Val228Met	LB/B	rs4911290	-
BPIFB3	P59826	VAR_049743	p.Thr290Met	LB/B	rs2093066	-
BPIFB3	P59826	VAR_049744	p.His334Gln	LB/B	rs6057717	-
BPIFB3	P59826	VAR_049745	p.Tyr369Cys	LB/B	rs6059063	-
BPIFB3	P59826	VAR_049746	p.Pro449Ser	LB/B	rs378098	-
BPIFB4	P59827	VAR_055998	p.Asp199Gly	LB/B	rs4339026	-
BPIFB4	P59827	VAR_055999	p.Gly206Trp	LB/B	rs2424943	-
BPIFB4	P59827	VAR_056000	p.Ile268Val	LB/B	rs2070325	-
BPIFB4	P59827	VAR_056001	p.Asn320Thr	LB/B	rs2889732	-
BPIFB4	P59827	VAR_059372	p.Gly167Trp	LB/B	rs2424943	-
BPIFB4	P59827	VAR_059373	p.Ile229Val	LB/B	rs2070325	-
BPIFB4	P59827	VAR_069042	p.Phe527Leu	LB/B	rs11699009	-
BPIFB4	P59827	VAR_069043	p.Thr533Ile	LB/B	rs11696307	-
BPIFB6	Q8NFQ5	VAR_024518	p.Val97Ile	LB/B	rs2070317	-
BPIFB6	Q8NFQ5	VAR_033632	p.Thr16Met	LB/B	rs17301126	-
BPIFB6	Q8NFQ5	VAR_033633	p.Pro149Thr	LB/B	rs11907355	-
BPIFB6	Q8NFQ5	VAR_033634	p.Ser347Gly	LB/B	rs4911287	-
BPIFB6	Q8NFQ5	VAR_065088	p.Arg296His	LB/B	rs79809934	-
BPIFC	Q8NFQ6	VAR_024516	p.Val269Ala	LB/B	rs2076051	-
BPIFC	Q8NFQ6	VAR_024517	p.Ser451Pro	LB/B	rs5998478	-
BPIFC	Q8NFQ6	VAR_033631	p.Val302Leu	LB/B	rs5994570	-
BPIFC	Q8NFQ6	VAR_049741	p.Glu479Ala	LB/B	rs35856742	-
BPNT2	Q9NX62	VAR_065847	p.Asp177Asn	LP/P	rs387907101	Chondrodysplasia with joint dislocations, GPAPP type (CDP-GPAPP) [MIM:614078]

BPNT2 Q9NX62 VAR_065848 p.Thr183Pro LP/P rs387907102 Chondrodysplasia with joint dislocations, GPAPP type (CDP-GPAPP) [MIM:614078]
BPTF Q12830 VAR_080531 p.Ala1924Thr LP/P rs1425998598 Neurodevelopmental disorder with dysmorphic facies and distal limb anomalies (NEDDFL) [MIM:617755]
BPTF Q12830 VAR_080532 p.Met2996Arg LP/P rs782736894 Neurodevelopmental disorder with dysmorphic facies and distal limb anomalies (NEDDFL) [MIM:617755]
BRAF P15056 VAR_018512 p.Gly466Val LP/P rs121913351 Lung cancer (LNCR) [MIM:211980]
BRAF P15056 VAR_018513 p.Leu597Arg LP/P rs121913366 Lung cancer (LNCR) [MIM:211980]
BRAF P15056 VAR_018613 p.Arg462Ile LP/P rs180177032 Colorectal cancer (CRC) [MIM:114500]
BRAF P15056 VAR_018614 p.Ile463Ser LP/P rs180177033 Colorectal cancer (CRC) [MIM:114500]
BRAF P15056 VAR_018615 p.Gly464Glu LP/P rs121913348 Colorectal cancer (CRC) [MIM:114500]
BRAF P15056 VAR_018616 p.Gly464Val US rs121913348 A colorectal cancer cell line
BRAF P15056 VAR_018617 p.Gly466Ala US rs121913351 Melanoma
BRAF P15056 VAR_018618 p.Gly466Glu US rs121913351 Melanoma
BRAF P15056 VAR_018620 p.Gly469Ala LP/P rs121913355 Familial non-Hodgkin lymphoma (NHL) [MIM:605027]
BRAF P15056 VAR_018621 p.Gly469Glu US rs121913355 Colon cancer
BRAF P15056 VAR_018621 p.Gly469Glu LP/P rs121913355 Cardiofaciocutaneous syndrome 1 (CFC1) [MIM:115150]
BRAF P15056 VAR_018622 p.Gly469Arg LP/P rs121913357 Familial non-Hodgkin lymphoma (NHL) [MIM:605027]
BRAF P15056 VAR_018623 p.Glu586Lys US rs121913340 Ovarian cancer
BRAF P15056 VAR_018624 p.Asp594Gly LP/P rs121913338 Familial non-Hodgkin lymphoma (NHL) [MIM:605027]
BRAF P15056 VAR_018625 p.Phe595Leu LP/P rs121913341 Cardiofaciocutaneous syndrome 1 (CFC1) [MIM:115150]
BRAF P15056 VAR_018626 p.Gly596Arg US rs121913361 A colorectal adenocarcinoma sample
BRAF P15056 VAR_018627 p.Leu597Val LP/P rs121913369 Noonan syndrome 7 (NS7) [MIM:613706]
BRAF P15056 VAR_018628 p.Val600Asp US rs121913377 A melanoma cell line
BRAF P15056 VAR_018629 p.Val600Glu LP/P rs113488022 Colorectal cancer (CRC) [MIM:114500]
BRAF P15056 VAR_018630 p.Lys601Glu LP/P rs121913364 Colorectal cancer (CRC) [MIM:114500]
BRAF P15056 VAR_026113 p.Ala246Pro LP/P rs180177034 Cardiofaciocutaneous syndrome 1 (CFC1) [MIM:115150]
BRAF P15056 VAR_026114 p.Gln257Arg LP/P rs180177035 Cardiofaciocutaneous syndrome 1 (CFC1) [MIM:115150]
BRAF P15056 VAR_026115 p.Leu485Phe LP/P rs180177036 Cardiofaciocutaneous syndrome 1 (CFC1) [MIM:115150]
BRAF P15056 VAR_026116 p.Lys499Glu LP/P rs180177037 Cardiofaciocutaneous syndrome 1 (CFC1) [MIM:115150]
BRAF P15056 VAR_026117 p.Glu501Gly LP/P rs180177039 Cardiofaciocutaneous syndrome 1 (CFC1) [MIM:115150]
BRAF P15056 VAR_026118 p.Glu501Lys LP/P rs180177038 Cardiofaciocutaneous syndrome 1 (CFC1) [MIM:115150]
BRAF P15056 VAR_026119 p.Asn581Asp LP/P rs180177040 Cardiofaciocutaneous syndrome 1 (CFC1) [MIM:115150]
BRAF P15056 VAR_035096 p.Ser467Ala LP/P rs869025606 Cardiofaciocutaneous syndrome 1 (CFC1) [MIM:115150]
BRAF P15056 VAR_035097 p.Phe468Ser LP/P rs397507473 Cardiofaciocutaneous syndrome 1 (CFC1) [MIM:115150]

BRAF P15056 VAR_035098 p.Gly596Val LP/P rs397507483 Cardiofaciocutaneous syndrome 1 (CFC1) [MIM:115150]
BRAF P15056 VAR_040391 p.Pro301Ser LB/B rs34776339 -
BRAF P15056 VAR_040392 p.Gly469Val US rs121913355 A colorectal adenocarcinoma sample
BRAF P15056 VAR_040393 p.Asn581Ser US rs121913370 A colorectal adenocarcinoma sample
BRAF P15056 VAR_058620 p.Thr241Met LP/P rs387906660 Noonan syndrome 7 (NS7) [MIM:613706]
BRAF P15056 VAR_058621 p.Thr241Pro LP/P rs387906661 Cardiofaciocutaneous syndrome 1 (CFC1) [MIM:115150]
BRAF P15056 VAR_058621 p.Thr241Pro LP/P rs387906661 LEOPARD syndrome 3 (LPRD3) [MIM:613707]
BRAF P15056 VAR_058622 p.Thr241Arg LP/P rs387906660 Noonan syndrome 7 (NS7) [MIM:613706]
BRAF P15056 VAR_058623 p.Leu245Phe LP/P rs397507466 Cardiofaciocutaneous syndrome 1 (CFC1) [MIM:115150]
BRAF P15056 VAR_058624 p.Glu275Lys LP/P - Cardiofaciocutaneous syndrome 1 (CFC1) [MIM:115150]
BRAF P15056 VAR_058625 p.Lys499Asn LP/P rs397507476 Cardiofaciocutaneous syndrome 1 (CFC1) [MIM:115150]
BRAF P15056 VAR_058626 p.Leu525Pro LP/P rs869025340 Cardiofaciocutaneous syndrome 1 (CFC1) [MIM:115150]
BRAF P15056 VAR_058627 p.Trp531Cys LP/P rs606231228 Noonan syndrome 7 (NS7) [MIM:613706]
BRAF P15056 VAR_058628 p.Thr599Arg LP/P rs121913375 Cardiofaciocutaneous syndrome 1 (CFC1) [MIM:115150]
BRAF P15056 VAR_058629 p.Lys601Gln LP/P rs121913364 Cardiofaciocutaneous syndrome 1 (CFC1) [MIM:115150]
BRAF P15056 VAR_058630 p.Asp638Glu LP/P rs180177042 Cardiofaciocutaneous syndrome 1 (CFC1) [MIM:115150]
BRAF P15056 VAR_058631 p.Gln709Arg LP/P rs397507486 Cardiofaciocutaneous syndrome 1 (CFC1) [MIM:115150]
BRAF P15056 VAR_065171 p.Thr244Pro LP/P rs397507465 Cardiofaciocutaneous syndrome 1 (CFC1) [MIM:115150]
BRAF P15056 VAR_065172 p.Gln262Lys LP/P rs397507470 Cardiofaciocutaneous syndrome 1 (CFC1) [MIM:115150]
BRAF P15056 VAR_065173 p.Asn580Asp LP/P - Cardiofaciocutaneous syndrome 1 (CFC1) [MIM:115150]
BRAT1 Q6PJG6 VAR_031202 p.Arg20Gly LB/B rs17856488 -
BRAT1 Q6PJG6 VAR_061594 p.Arg737Trp LB/B rs60152725 -
BRAT1 Q6PJG6 VAR_081168 p.Leu140Pro US rs1085307958 Neurodevelopmental disorder with cerebellar atrophy and with or without seizures (NEDCAS) [MIM:618056]
BRAT1 Q6PJG6 VAR_081168 p.Leu140Pro US rs1085307958 Rigidity and multifocal seizure syndrome, lethal neonatal (RMFSL) [MIM:614498]
BRAT1 Q6PJG6 VAR_081169 p.Arg609Trp US rs886039312 Neurodevelopmental disorder with cerebellar atrophy and with or without seizures (NEDCAS) [MIM:618056]
BRAT1 Q6PJG6 VAR_081170 p.Ala642Glu US rs200502048 Neurodevelopmental disorder with cerebellar atrophy and with or without seizures (NEDCAS) [MIM:618056]
BRAWNIN Q69YU5 VAR_062284 p.Arg45Cys LB/B rs2293624 -
BRCA1 P38398 VAR_007754 p.Val11Ala US rs80357017 -
BRCA1 P38398 VAR_007755 p.Ile21Val US rs80357406 -
BRCA1 P38398 VAR_007756 p.Leu22Ser LP/P rs80357438 Breast cancer (BC) [MIM:114480]
BRCA1 P38398 VAR_007757 p.Cys61Gly LP/P rs28897672 Breast cancer (BC) [MIM:114480]
BRCA1 P38398 VAR_007757 p.Cys61Gly LP/P rs28897672 Ovarian cancer (OC) [MIM:167000]
BRCA1 P38398 VAR_007758 p.Cys64Gly LP/P rs80357064 Breast cancer (BC) [MIM:114480]
BRCA1 P38398 VAR_007759 p.Cys64Tyr LB/B rs55851803 -

BRCA1	P38398	VAR_007760	p.His239Arg	LB/B	rs80357396	-
BRCA1	P38398	VAR_007761	p.Val271Met	LP/P	rs80357244	Breast cancer (BC) [MIM:114480]
BRCA1	P38398	VAR_007762	p.Gln356Arg	LB/B	rs1799950	-
BRCA1	P38398	VAR_007764	p.Ile379Met	LB/B	rs56128296	-
BRCA1	P38398	VAR_007765	p.Phe461Leu	LP/P	rs56046357	Breast cancer (BC) [MIM:114480]
BRCA1	P38398	VAR_007766	p.Tyr465Asp	LP/P	rs397508869	Breast cancer (BC) [MIM:114480]
BRCA1	P38398	VAR_007767	p.Arg507Ile	US	rs80357224	-
BRCA1	P38398	VAR_007768	p.Gly552Val	LP/P	rs397508893	Breast cancer (BC) [MIM:114480]
BRCA1	P38398	VAR_007769	p.Asp693Asn	LB/B	rs4986850	-
BRCA1	P38398	VAR_007770	p.Val772Ala	LB/B	rs80357467	-
BRCA1	P38398	VAR_007771	p.Lys820Glu	LB/B	rs56082113	-
BRCA1	P38398	VAR_007772	p.Thr826Lys	US	rs28897683	Breast cancer (BC) [MIM:114480]
BRCA1	P38398	VAR_007773	p.Arg841Trp	US	rs1800709	Breast-ovarian cancer, familial, 1 (BROVCA1) [MIM:604370]
BRCA1	P38398	VAR_007774	p.Pro871Leu	LB/B	rs799917	-
BRCA1	P38398	VAR_007775	p.Leu892Ser	LP/P	rs397508994	Breast cancer (BC) [MIM:114480]
BRCA1	P38398	VAR_007776	p.Gly960Asp	LP/P	rs397509022	Breast cancer (BC) [MIM:114480]
BRCA1	P38398	VAR_007777	p.Met1008Ile	LB/B	rs1800704	-
BRCA1	P38398	VAR_007778	p.Thr1025Ile	LP/P	rs397509034	Breast cancer (BC) [MIM:114480]
BRCA1	P38398	VAR_007779	p.Glu1038Gly	LB/B	rs16941	-
BRCA1	P38398	VAR_007780	p.Ser1040Asn	LB/B	rs4986852	-
BRCA1	P38398	VAR_007781	p.Val1047Ala	LP/P	rs397509037	Breast cancer (BC) [MIM:114480]
BRCA1	P38398	VAR_007782	p.Pro1150Ser	LP/P	rs80357272	Breast cancer (BC) [MIM:114480]
BRCA1	P38398	VAR_007783	p.Lys1183Arg	LB/B	rs16942	-
BRCA1	P38398	VAR_007784	p.Glu1219Asp	US	rs80356876	-
BRCA1	P38398	VAR_007785	p.Arg1347Gly	LB/B	rs28897689	-
BRCA1	P38398	VAR_007786	p.Ser1431Pro	LB/B	-	-
BRCA1	P38398	VAR_007787	p.Arg1443Gly	US	rs41293455	Breast cancer (BC) [MIM:114480]
BRCA1	P38398	VAR_007788	p.Ser1512Ile	LB/B	rs1800744	-
BRCA1	P38398	VAR_007789	p.Thr1561Ile	US	rs56158747	-
BRCA1	P38398	VAR_007790	p.Lys1606Glu	US	rs80356943	-
BRCA1	P38398	VAR_007791	p.Ser1613Gly	LB/B	rs1799966	-
BRCA1	P38398	VAR_007792	p.Met1628Val	US	rs80357465	-
BRCA1	P38398	VAR_007793	p.Met1628Thr	US	rs4986854	Some patients with sporadic breast cancer
BRCA1	P38398	VAR_007794	p.Pro1637Leu	LB/B	rs80357048	-
BRCA1	P38398	VAR_007795	p.Met1652Ile	LB/B	rs1799967	-
BRCA1	P38398	VAR_007796	p.Ala1708Glu	LP/P	rs28897696	Breast cancer (BC) [MIM:114480]
BRCA1	P38398	VAR_007797	p.Val1713Gly	LB/B	-	-
BRCA1	P38398	VAR_007798	p.Pro1749Arg	US	rs80357462	Ovarian cancer (OC) [MIM:167000]
BRCA1	P38398	VAR_007799	p.Met1775Arg	LP/P	rs41293463	Breast cancer (BC) [MIM:114480]
BRCA1	P38398	VAR_008759	p.Glu227Lys	US	-	Ovarian cancer (OC) [MIM:167000]
BRCA1	P38398	VAR_008760	p.Pro346Ser	US	rs80357015	Breast cancer (BC) [MIM:114480]
BRCA1	P38398	VAR_008761	p.Lys1406Asn	LB/B	rs1800707	-
BRCA1	P38398	VAR_008762	p.Ala1641Pro	US	rs1800726	Ovarian cancer (OC) [MIM:167000]
BRCA1	P38398	VAR_008763	p.Asp1692Asn	US	rs80187739	Ovarian cancer (OC) [MIM:167000]
BRCA1	P38398	VAR_008764	p.Pro1776Ser	US	rs1800757	Ovarian cancer (OC) [MIM:167000]
BRCA1	P38398	VAR_008765	p.Pro1812Ser	US	rs1800751	Ovarian cancer (OC) [MIM:167000]
BRCA1	P38398	VAR_019944	p.Gly275Ser	LB/B	rs8176153	-
BRCA1	P38398	VAR_019945	p.Ser1140Gly	US	rs2227945	Breast cancer (BC) [MIM:114480]

BRCA1	P38398	VAR_019946	p.Thr1620Ala	LB/B	rs8176219	-
BRCA1	P38398	VAR_020110	p.Asn723Asp	LB/B	rs4986845	-
BRCA1	P38398	VAR_020111	p.Phe989Ser	LB/B	rs4986848	-
BRCA1	P38398	VAR_020112	p.Arg1443Gln	LB/B	rs4986849	-
BRCA1	P38398	VAR_020679	p.Glu10Lys	LP/P	-	Breast cancer (BC) [MIM:114480]
BRCA1	P38398	VAR_020679	p.Glu10Lys	LP/P	-	Breast-ovarian cancer, familial, 1 (BROVCA1) [MIM:604370]
BRCA1	P38398	VAR_020680	p.Glu23Lys	LP/P	-	Breast cancer (BC) [MIM:114480]
BRCA1	P38398	VAR_020680	p.Glu23Lys	LP/P	-	Breast-ovarian cancer, familial, 1 (BROVCA1) [MIM:604370]
BRCA1	P38398	VAR_020681	p.Arg71Lys	US	rs80356913	Breast cancer (BC) [MIM:114480]
BRCA1	P38398	VAR_020682	p.Asn656Ile	LB/B	-	-
BRCA1	P38398	VAR_020683	p.Asp749Tyr	LP/P	rs80357114	Breast cancer (BC) [MIM:114480]
BRCA1	P38398	VAR_020684	p.His835Tyr	US	rs751656678	Breast-ovarian cancer, familial, 1 (BROVCA1) [MIM:604370]
BRCA1	P38398	VAR_020685	p.Tyr856His	US	rs80356892	A patient with sporadic breast cancer
BRCA1	P38398	VAR_020686	p.Arg866Gln	US	-	Breast cancer (BC) [MIM:114480]
BRCA1	P38398	VAR_020687	p.His888Tyr	US	rs80357480	Breast cancer (BC) [MIM:114480]
BRCA1	P38398	VAR_020688	p.Glu1060Ala	LB/B	rs80357184	-
BRCA1	P38398	VAR_020689	p.Ser1139Ile	US	rs80357228	Breast cancer (BC) [MIM:114480]
BRCA1	P38398	VAR_020690	p.Ser1187Ile	LP/P	-	Breast cancer (BC) [MIM:114480]
BRCA1	P38398	VAR_020690	p.Ser1187Ile	LP/P	-	Breast-ovarian cancer, familial, 1 (BROVCA1) [MIM:604370]
BRCA1	P38398	VAR_020691	p.Gln1200His	LP/P	rs56214134	Breast cancer (BC) [MIM:114480]
BRCA1	P38398	VAR_020691	p.Gln1200His	LP/P	rs56214134	Breast-ovarian cancer, familial, 1 (BROVCA1) [MIM:604370]
BRCA1	P38398	VAR_020692	p.Arg1204Ile	LP/P	-	Breast cancer (BC) [MIM:114480]
BRCA1	P38398	VAR_020693	p.Lys1207Asn	LP/P	-	Breast cancer (BC) [MIM:114480]
BRCA1	P38398	VAR_020694	p.Glu1210Gly	US	rs1060502347	Breast cancer (BC) [MIM:114480]
BRCA1	P38398	VAR_020695	p.Ser1217Tyr	LP/P	-	Breast cancer (BC) [MIM:114480]
BRCA1	P38398	VAR_020695	p.Ser1217Tyr	LP/P	-	Breast-ovarian cancer, familial, 1 (BROVCA1) [MIM:604370]
BRCA1	P38398	VAR_020696	p.Phe1226Leu	LP/P	-	Breast-ovarian cancer, familial, 1 (BROVCA1) [MIM:604370]
BRCA1	P38398	VAR_020697	p.Arg1243Gly	LP/P	-	Breast-ovarian cancer, familial, 1 (BROVCA1) [MIM:604370]
BRCA1	P38398	VAR_020698	p.Ser1297Pro	US	rs1450793674	Breast cancer (BC) [MIM:114480]
BRCA1	P38398	VAR_020699	p.Met1411Thr	US	rs273900729	Breast cancer (BC) [MIM:114480]
BRCA1	P38398	VAR_020699	p.Met1411Thr	US	rs273900729	Ovarian cancer (OC) [MIM:167000]
BRCA1	P38398	VAR_020700	p.Val1665Met	LB/B	rs80357169	-
BRCA1	P38398	VAR_020701	p.Lys1690Gln	US	rs397507239	Some patients with sporadic breast cancer
BRCA1	P38398	VAR_020702	p.Cys1697Arg	LP/P	rs80356993	Ovarian cancer (OC) [MIM:167000]
BRCA1	P38398	VAR_020704	p.Leu1786Pro	US	rs398122697	Breast-ovarian cancer, familial, 1 (BROVCA1) [MIM:604370]
BRCA1	P38398	VAR_021913	p.Ile925Leu	LB/B	rs4986847	-
BRCA1	P38398	VAR_035947	p.Leu30Phe	US	-	A breast cancer sample
BRCA1	P38398	VAR_035948	p.Leu758Phe	US	-	A breast cancer sample
BRCA1	P38398	VAR_035949	p.Gly778Cys	US	-	A breast cancer sample
BRCA1	P38398	VAR_052077	p.Ser153Arg	LB/B	rs28897674	-

BRCA1	P38398	VAR_052078	p.Asn1236Lys	US	rs28897687	Breast cancer (BC) [MIM:114480]
BRCA1	P38398	VAR_052079	p.Glu1250Lys	LB/B	rs28897686	-
BRCA1	P38398	VAR_052080	p.Phe1662Cys	LB/B	rs28897695	-
BRCA1	P38398	VAR_063212	p.Met1775Lys	LP/P	rs41293463	Breast cancer (BC) [MIM:114480]
BRCA1	P38398	VAR_063899	p.Met18Thr	US	rs80356929	Breast cancer (BC) [MIM:114480]
BRCA1	P38398	VAR_063900	p.Arg1495Met	US	rs80357389	Breast cancer (BC) [MIM:114480]
BRCA1	P38398	VAR_063901	p.Ala1623Gly	LB/B	rs80356862	-
BRCA1	P38398	VAR_063902	p.Thr1685Ala	US	rs80356890	Breast cancer (BC) [MIM:114480]
BRCA1	P38398	VAR_063903	p.Thr1685Ile	LB/B	rs80357043	-
BRCA1	P38398	VAR_063904	p.Met1689Arg	US	rs80357061	Breast cancer (BC) [MIM:114480]
BRCA1	P38398	VAR_063905	p.Gly1706Glu	US	rs80356860	Breast cancer (BC) [MIM:114480]
BRCA1	P38398	VAR_063906	p.Ser1715Arg	US	rs80357094	Breast cancer (BC) [MIM:114480]
BRCA1	P38398	VAR_063907	p.Gly1738Arg	US	rs80356937	Breast cancer (BC) [MIM:114480]
BRCA1	P38398	VAR_063908	p.Leu1764Pro	US	rs80357281	Breast cancer (BC) [MIM:114480]
BRCA1	P38398	VAR_063909	p.Ile1766Ser	US	rs80357463	Breast cancer (BC) [MIM:114480]
BRCA1	P38398	VAR_063910	p.Gly1788Val	US	rs80357069	Breast cancer (BC) [MIM:114480]
BRCA1	P38398	VAR_070458	p.Ser4Phe	US	rs786203152	Breast cancer (BC) [MIM:114480]
BRCA1	P38398	VAR_070459	p.Lys45Gln	US	rs769650474	Breast cancer (BC) [MIM:114480]
BRCA1	P38398	VAR_070460	p.Asp67Tyr	US	rs80357102	Breast cancer (BC) [MIM:114480]
BRCA1	P38398	VAR_070461	p.Tyr105Cys	LB/B	rs28897673	-
BRCA1	P38398	VAR_070462	p.Asn132Lys	US	rs80357413	Breast cancer (BC) [MIM:114480]
BRCA1	P38398	VAR_070463	p.Pro142His	US	rs55971303	Breast cancer (BC) [MIM:114480]
BRCA1	P38398	VAR_070464	p.Leu147Phe	US	rs748876625	Breast cancer (BC) [MIM:114480]
BRCA1	P38398	VAR_070465	p.Leu165Pro	US	-	Breast cancer (BC) [MIM:114480]
BRCA1	P38398	VAR_070466	p.Arg170Trp	US	rs80357325	Breast cancer (BC) [MIM:114480]
BRCA1	P38398	VAR_070467	p.Ser186Tyr	US	rs55688530	Breast cancer (BC) [MIM:114480]
BRCA1	P38398	VAR_070468	p.Val191Ile	US	rs80357090	Breast cancer (BC) [MIM:114480]
BRCA1	P38398	VAR_070469	p.Thr231Met	US	rs80357001	Breast cancer (BC) [MIM:114480]
BRCA1	P38398	VAR_070470	p.Asp245Val	US	rs80356865	Breast cancer (BC) [MIM:114480]
BRCA1	P38398	VAR_070471	p.Leu246Val	US	rs28897675	Breast cancer (BC) [MIM:114480]
BRCA1	P38398	VAR_070472	p.Val271Leu	US	rs80357244	Breast cancer (BC) [MIM:114480]
BRCA1	P38398	VAR_070473	p.Leu668Phe	US	rs80357250	Breast cancer (BC) [MIM:114480]
BRCA1	P38398	VAR_070474	p.Asp695Asn	US	rs28897681	Breast cancer (BC) [MIM:114480]
BRCA1	P38398	VAR_070475	p.Pro798Leu	US	rs876660005	Breast cancer (BC) [MIM:114480]
BRCA1	P38398	VAR_070476	p.Asn810Tyr	US	rs28897682	Breast cancer (BC) [MIM:114480]
BRCA1	P38398	VAR_070477	p.Arg841Gln	US	rs80357337	Breast cancer (BC) [MIM:114480]
BRCA1	P38398	VAR_070478	p.Arg866Cys	LB/B	rs41286300	-
BRCA1	P38398	VAR_070479	p.Ser1101Asn	US	rs41293447	Breast cancer (BC) [MIM:114480]
BRCA1	P38398	VAR_070480	p.Ser1140Asn	US	-	Breast cancer (BC) [MIM:114480]
BRCA1	P38398	VAR_070481	p.Glu1214Lys	US	rs80356923	Breast cancer (BC) [MIM:114480]
BRCA1	P38398	VAR_070482	p.Leu1267Ser	US	rs587782190	Breast cancer (BC) [MIM:114480]
BRCA1	P38398	VAR_070483	p.Glu1282Val	US	rs80357217	Breast cancer (BC) [MIM:114480]
BRCA1	P38398	VAR_070485	p.Ser1301Arg	US	rs273900719	Breast cancer (BC) [MIM:114480]
BRCA1	P38398	VAR_070486	p.Glu1346Lys	US	rs80357407	Breast cancer (BC) [MIM:114480]
BRCA1	P38398	VAR_070487	p.Val1378Ile	US	rs28897690	Breast cancer (BC) [MIM:114480]
BRCA1	P38398	VAR_070488	p.Met1400Val	US	rs80357306	Breast cancer (BC) [MIM:114480]
BRCA1	P38398	VAR_070489	p.Leu1407Pro	US	rs80357492	Breast cancer (BC) [MIM:114480]
BRCA1	P38398	VAR_070490	p.Ser1448Gly	US	rs80357486	Breast cancer (BC) [MIM:114480]
BRCA1	P38398	VAR_070491	p.Ser1486Cys	US	rs397507232	Breast cancer (BC) [MIM:114480]

BRCA1	P38398	VAR_070492	p.Val1534Met	US	rs55815649	Breast cancer (BC) [MIM:114480]
BRCA1	P38398	VAR_070493	p.Arg1589Pro	US	-	Breast cancer (BC) [MIM:114480]
BRCA1	P38398	VAR_070494	p.Ser1651Phe	US	rs80356938	Breast cancer (BC) [MIM:114480]
BRCA1	P38398	VAR_070495	p.Ser1651Pro	US	rs879254042	Breast cancer (BC) [MIM:114480]
BRCA1	P38398	VAR_070496	p.Ser1655Phe	US	rs80357390	Breast cancer (BC) [MIM:114480]
BRCA1	P38398	VAR_070497	p.His1686Gln	US	rs397509218	Breast cancer (BC) [MIM:114480]
BRCA1	P38398	VAR_070498	p.His1686Arg	US	rs730882166	Breast cancer (BC) [MIM:114480]
BRCA1	P38398	VAR_070500	p.Thr1691Ile	US	rs80357034	Breast cancer (BC) [MIM:114480]
BRCA1	P38398	VAR_070501	p.Arg1699Gln	US	rs41293459	Breast cancer (BC) [MIM:114480]
BRCA1	P38398	VAR_070502	p.Gly1706Ala	US	rs80356860	Breast cancer (BC) [MIM:114480]
BRCA1	P38398	VAR_070503	p.Trp1718Cys	US	rs80357239	Breast cancer (BC) [MIM:114480]
BRCA1	P38398	VAR_070504	p.Thr1720Ala	US	rs56195342	Breast cancer (BC) [MIM:114480]
BRCA1	P38398	VAR_070505	p.Glu1735Lys	US	rs397509238	Breast cancer (BC) [MIM:114480]
BRCA1	P38398	VAR_070506	p.Val1736Ala	US	rs45553935	Breast cancer (BC) [MIM:114480]
BRCA1	P38398	VAR_070506	p.Val1736Ala	US	rs45553935	Fanconi anemia, complementation group S (FANCS) [MIM:617883]
BRCA1	P38398	VAR_070507	p.Asp1739Gly	US	rs80357227	Breast cancer (BC) [MIM:114480]
BRCA1	P38398	VAR_070508	p.Asp1739Val	US	rs80357227	Breast cancer (BC) [MIM:114480]
BRCA1	P38398	VAR_070509	p.His1746Gln	US	rs786202389	Breast cancer (BC) [MIM:114480]
BRCA1	P38398	VAR_070510	p.Arg1753Thr	US	rs397509246	Breast cancer (BC) [MIM:114480]
BRCA1	P38398	VAR_070511	p.Cys1767Ser	US	rs1597804426	Breast cancer (BC) [MIM:114480]
BRCA1	P38398	VAR_070512	p.Gly1770Val	US	rs863224765	Breast cancer (BC) [MIM:114480]
BRCA1	P38398	VAR_070513	p.Trp1782Cys	US	-	Breast cancer (BC) [MIM:114480]
BRCA1	P38398	VAR_070514	p.Ala1789Thr	US	rs80357078	Breast cancer (BC) [MIM:114480]
BRCA1	P38398	VAR_070515	p.Glu1794Asp	US	rs397509275	Breast cancer (BC) [MIM:114480]
BRCA1	P38398	VAR_070516	p.Val1804Asp	US	rs80356920	Breast cancer (BC) [MIM:114480]
BRCA1	P38398	VAR_070517	p.Pro1812Arg	US	-	Breast cancer (BC) [MIM:114480]
BRCA1	P38398	VAR_070518	p.Trp1837Arg	US	rs80356959	Breast cancer (BC) [MIM:114480]
BRCA1	P38398	VAR_070519	p.His1862Leu	US	rs80357183	Breast cancer (BC) [MIM:114480]
BRCA1	P38398	VAR_075666	p.Arg1699Trp	LP/P	rs55770810	Breast cancer (BC) [MIM:114480]
BRCA1	P38398	VAR_075666	p.Arg1699Trp	LP/P	rs55770810	Fanconi anemia, complementation group S (FANCS) [MIM:617883]
BRCA1	P38398	VAR_075666	p.Arg1699Trp	LP/P	rs55770810	Ovarian cancer (OC) [MIM:167000]
BRCA1	P38398	VAR_079607	p.Leu1780Pro	US	rs80357474	Breast cancer (BC) [MIM:114480]
BRCA1	P38398	VAR_079607	p.Leu1780Pro	US	rs80357474	Breast-ovarian cancer, familial, 1 (BROVCA1) [MIM:604370]
BRCA1	P38398	VAR_079607	p.Leu1780Pro	US	rs80357474	Ovarian cancer (OC) [MIM:167000]
BRCA2	P51587	VAR_005085	p.Phe32Leu	LP/P	rs397508057	Breast cancer (BC) [MIM:114480]
BRCA2	P51587	VAR_005086	p.Lys53Arg	LP/P	rs397507595	Breast cancer (BC) [MIM:114480]
BRCA2	P51587	VAR_005087	p.Ala75Pro	US	rs28897701	Ovarian cancer
BRCA2	P51587	VAR_005087	p.Ala75Pro	US	rs28897701	Renal cancer
BRCA2	P51587	VAR_005088	p.Phe81Leu	LP/P	rs80358507	Breast cancer (BC) [MIM:114480]
BRCA2	P51587	VAR_005089	p.Pro201Arg	LP/P	rs397507822	Breast cancer (BC) [MIM:114480]
BRCA2	P51587	VAR_005090	p.Val211Ala	LP/P	-	Breast cancer (BC) [MIM:114480]
BRCA2	P51587	VAR_005091	p.Pro222Ser	LP/P	rs397507873	Breast cancer (BC) [MIM:114480]
BRCA2	P51587	VAR_005092	p.Asn289His	LB/B	rs766173	-
BRCA2	P51587	VAR_005093	p.Val355Leu	US	-	Lung cancer
BRCA2	P51587	VAR_005094	p.Asn372His	LB/B	rs144848	-
BRCA2	P51587	VAR_005095	p.Cys554Trp	US	rs80358451	Pancreas cancer

BRCA2	P51587	VAR_005095	p.Cys554Trp	LP/P	rs80358451	Breast cancer (BC) [MIM:114480]
BRCA2	P51587	VAR_005096	p.Thr630Ile	US	rs80358479	Ovarian cancer
BRCA2	P51587	VAR_005097	p.Asp728Ala	LP/P	rs757577670	Breast cancer (BC) [MIM:114480]
BRCA2	P51587	VAR_005098	p.Asn991Asp	LB/B	rs1799944	-
BRCA2	P51587	VAR_005099	p.Asn1147Ser	LB/B	rs1799951	-
BRCA2	P51587	VAR_005101	p.Gly1529Arg	US	rs28897728	Bladder cancer
BRCA2	P51587	VAR_005102	p.Asn1880Lys	LB/B	rs11571657	-
BRCA2	P51587	VAR_005103	p.Thr1915Met	LB/B	rs4987117	-
BRCA2	P51587	VAR_005104	p.Arg2034Cys	LB/B	rs1799954	-
BRCA2	P51587	VAR_005105	p.Gly2274Val	LP/P	rs55712212	Breast cancer (BC) [MIM:114480]
BRCA2	P51587	VAR_005106	p.His2415Asn	LP/P	-	Breast cancer (BC) [MIM:114480]
BRCA2	P51587	VAR_005107	p.Gln2421His	LP/P	-	Breast cancer (BC) [MIM:114480]
BRCA2	P51587	VAR_005108	p.Asp3095Glu	US	rs80359198	Breast cancer (BC) [MIM:114480]
BRCA2	P51587	VAR_005109	p.Ile3103Met	US	rs80359204	Melanoma
BRCA2	P51587	VAR_005110	p.Met3118Thr	LP/P	rs56204128	Breast cancer (BC) [MIM:114480]
BRCA2	P51587	VAR_005111	p.Thr3357Arg	LP/P	rs80358388	Breast cancer (BC) [MIM:114480]
BRCA2	P51587	VAR_005112	p.Ile3412Val	LB/B	rs1801426	-
BRCA2	P51587	VAR_008766	p.Asn108His	LB/B	rs80358567	-
BRCA2	P51587	VAR_008767	p.Lys327Glu	US	rs80359242	Breast cancer (BC) [MIM:114480]
BRCA2	P51587	VAR_008768	p.Thr582Pro	LB/B	rs80358457	-
BRCA2	P51587	VAR_008769	p.Asp707Tyr	LB/B	rs80358487	-
BRCA2	P51587	VAR_008770	p.Met784Val	LB/B	rs11571653	-
BRCA2	P51587	VAR_008771	p.Asn886Ile	LB/B	rs80358526	-
BRCA2	P51587	VAR_008772	p.Asp935Asn	US	rs28897716	Breast cancer (BC) [MIM:114480]
BRCA2	P51587	VAR_008774	p.Cys1290Tyr	LB/B	rs41293485	-
BRCA2	P51587	VAR_008775	p.Thr1414Met	LB/B	rs70953664	-
BRCA2	P51587	VAR_008776	p.Asp1420Tyr	LB/B	rs28897727	-
BRCA2	P51587	VAR_008777	p.Asp1513Asn	LB/B	rs80358687	-
BRCA2	P51587	VAR_008778	p.Glu1593Asp	LB/B	rs80358703	-
BRCA2	P51587	VAR_008779	p.Gly1771Asp	US	rs80358755	Breast cancer (BC) [MIM:114480]
BRCA2	P51587	VAR_008780	p.Asn1805Ser	LB/B	rs80358765	-
BRCA2	P51587	VAR_008781	p.Asp1902Asn	LB/B	rs4987048	-
BRCA2	P51587	VAR_008782	p.His2074Asn	LB/B	rs34309943	-
BRCA2	P51587	VAR_008783	p.Glu2089Asp	LP/P	-	Breast cancer (BC) [MIM:114480]
BRCA2	P51587	VAR_008784	p.Val2138Phe	LB/B	rs11571659	-
BRCA2	P51587	VAR_008785	p.Ala2466Val	LB/B	rs169547	-
BRCA2	P51587	VAR_008786	p.Leu2480Val	LB/B	rs80358965	-
BRCA2	P51587	VAR_008787	p.Ile2490Thr	LB/B	rs11571707	-
BRCA2	P51587	VAR_008788	p.Arg2502His	US	rs56070345	Ovarian cancer
BRCA2	P51587	VAR_008789	p.Thr2515Ile	US	rs28897744	Breast cancer (BC) [MIM:114480]
BRCA2	P51587	VAR_008790	p.Arg2787His	US	rs80359078	Breast cancer (BC) [MIM:114480]
BRCA2	P51587	VAR_008790	p.Arg2787His	US	rs80359078	Ovarian cancer
BRCA2	P51587	VAR_008791	p.Ile2944Phe	LB/B	rs4987047	-
BRCA2	P51587	VAR_008792	p.Ala2951Thr	US	rs11571769	Breast cancer (BC) [MIM:114480]
BRCA2	P51587	VAR_008793	p.Val2969Met	LB/B	rs59004709	-
BRCA2	P51587	VAR_008794	p.Tyr3098His	US	rs41293521	Breast cancer (BC) [MIM:114480]
BRCA2	P51587	VAR_008794	p.Tyr3098His	US	rs41293521	Ovarian cancer
BRCA2	P51587	VAR_008795	p.Lys3257Arg	LB/B	rs55847618	-
BRCA2	P51587	VAR_008796	p.Arg3276Ser	LB/B	rs80359245	-

BRCA2	P51587	VAR_018661	p.Thr2722Arg	LP/P	rs80359062	Breast cancer (BC) [MIM:114480]
BRCA2	P51587	VAR_018908	p.Lys322Gln	LB/B	rs11571640	-
BRCA2	P51587	VAR_018909	p.Leu929Ser	LB/B	rs2227943	-
BRCA2	P51587	VAR_018910	p.Ser976Phe	LB/B	rs11571656	-
BRCA2	P51587	VAR_018911	p.Asn987Ile	LB/B	rs2227944	-
BRCA2	P51587	VAR_018912	p.His1561Asn	LB/B	rs2219594	-
BRCA2	P51587	VAR_018913	p.Lys2162Arg	LB/B	rs11571660	-
BRCA2	P51587	VAR_018914	p.His2440Arg	LB/B	rs4986860	-
BRCA2	P51587	VAR_018915	p.Ser2835Pro	LB/B	rs11571746	-
BRCA2	P51587	VAR_018916	p.Glu2856Ala	US	rs11571747	Breast cancer (BC) [MIM:114480]
BRCA2	P51587	VAR_018917	p.Val3244Ile	LB/B	rs11571831	-
BRCA2	P51587	VAR_020705	p.Tyr42Cys	US	rs4987046	Breast cancer (BC) [MIM:114480]
BRCA2	P51587	VAR_020705	p.Tyr42Cys	US	rs4987046	Ovarian cancer
BRCA2	P51587	VAR_020706	p.Asn60Ser	US	rs80358463	Breast cancer (BC) [MIM:114480]
BRCA2	P51587	VAR_020707	p.Gly405Arg	US	-	Breast cancer (BC) [MIM:114480]
BRCA2	P51587	VAR_020708	p.Thr431Ile	US	rs876660828	Breast cancer (BC) [MIM:114480]
BRCA2	P51587	VAR_020709	p.Arg448His	US	rs80358423	Breast cancer (BC) [MIM:114480]
BRCA2	P51587	VAR_020710	p.Glu462Gly	US	rs56403624	Breast cancer (BC) [MIM:114480]
BRCA2	P51587	VAR_020711	p.Thr598Ala	LB/B	rs28897710	-
BRCA2	P51587	VAR_020712	p.Leu613Arg	US	rs587780646	Breast cancer (BC) [MIM:114480]
BRCA2	P51587	VAR_020713	p.Glu1036Lys	US	-	Breast cancer (BC) [MIM:114480]
BRCA2	P51587	VAR_020714	p.Ser1106Arg	US	rs1298550035	Breast cancer (BC) [MIM:114480]
BRCA2	P51587	VAR_020715	p.Ser1179Asn	LP/P	rs397507674	Breast cancer (BC) [MIM:114480]
BRCA2	P51587	VAR_020716	p.Asn1279Ser	LB/B	rs1060502384	-
BRCA2	P51587	VAR_020717	p.Lys1445Thr	US	-	Breast cancer (BC) [MIM:114480]
BRCA2	P51587	VAR_020718	p.Phe1524Val	US	rs56386506	Breast cancer (BC) [MIM:114480]
BRCA2	P51587	VAR_020719	p.Cys1580Tyr	LP/P	rs398122784	Breast cancer (BC) [MIM:114480]
BRCA2	P51587	VAR_020720	p.Thr1679Ile	LP/P	-	Breast cancer (BC) [MIM:114480]
BRCA2	P51587	VAR_020721	p.Val1804Ala	LP/P	rs370252983	Breast cancer (BC) [MIM:114480]
BRCA2	P51587	VAR_020722	p.Glu1901Lys	LP/P	-	Breast cancer (BC) [MIM:114480]
BRCA2	P51587	VAR_020723	p.Ile1929Val	US	rs79538375	Breast cancer (BC) [MIM:114480]
BRCA2	P51587	VAR_020724	p.Thr2031Ala	US	-	Breast cancer (BC) [MIM:114480]
BRCA2	P51587	VAR_020725	p.Ser2072Cys	LP/P	rs80358862	Breast cancer (BC) [MIM:114480]
BRCA2	P51587	VAR_020726	p.Tyr2094Cys	LP/P	rs397507838	Breast cancer (BC) [MIM:114480]
BRCA2	P51587	VAR_020727	p.Pro2096Leu	LP/P	-	Breast cancer (BC) [MIM:114480]
BRCA2	P51587	VAR_020728	p.Val2118Leu	US	-	Breast cancer (BC) [MIM:114480]
BRCA2	P51587	VAR_020729	p.Lys2128Asn	LP/P	rs397507847	Breast cancer (BC) [MIM:114480]
BRCA2	P51587	VAR_020730	p.Glu2275Gly	US	-	Breast cancer (BC) [MIM:114480]
BRCA2	P51587	VAR_020731	p.Phe2293Leu	US	rs80358912	Breast cancer (BC) [MIM:114480]
BRCA2	P51587	VAR_020732	p.Gly2353Arg	US	rs80358935	Breast cancer (BC) [MIM:114480]
BRCA2	P51587	VAR_020733	p.Arg2488Lys	US	rs80358968	Breast cancer (BC) [MIM:114480]
BRCA2	P51587	VAR_020734	p.Asn2706Ser	LB/B	rs80359055	-
BRCA2	P51587	VAR_020735	p.Asp2723His	US	rs41293511	Breast cancer (BC) [MIM:114480]
BRCA2	P51587	VAR_020736	p.Val2728Ile	LP/P	rs28897749	Breast cancer (BC) [MIM:114480]
BRCA2	P51587	VAR_020737	p.Lys2729Asn	US	rs80359065	Breast cancer (BC) [MIM:114480]
BRCA2	P51587	VAR_020738	p.Gly2793Arg	US	rs80359082	Breast cancer (BC) [MIM:114480]
BRCA2	P51587	VAR_020739	p.Lys2950Asn	US	rs28897754	Breast cancer (BC) [MIM:114480]
BRCA2	P51587	VAR_020740	p.Thr3013Ile	US	rs28897755	Breast cancer (BC) [MIM:114480]
BRCA2	P51587	VAR_020741	p.Pro3063Ser	US	rs80359176	A patient with ovarian cancer

BRCA2	P51587	VAR_020742	p.Gly3076Glu	LP/P	rs80359187	Breast cancer (BC) [MIM:114480]
BRCA2	P51587	VAR_020743	p.Asn3124Ile	LP/P	rs28897759	Breast cancer (BC) [MIM:114480]
BRCA2	P51587	VAR_020744	p.Lys3196Glu	LP/P	rs80359228	Breast cancer (BC) [MIM:114480]
BRCA2	P51587	VAR_020745	p.Thr3374Ile	LB/B	rs56309455	-
BRCA2	P51587	VAR_028167	p.Gly25Arg	LP/P	rs80358961	Breast cancer (BC) [MIM:114480]
BRCA2	P51587	VAR_028168	p.Trp31Cys	LP/P	rs80359214	Breast cancer (BC) [MIM:114480]
BRCA2	P51587	VAR_028169	p.Trp31Arg	LP/P	rs80359182	Breast cancer (BC) [MIM:114480]
BRCA2	P51587	VAR_032712	p.Thr64Ile	LP/P	rs397507615	Breast cancer (BC) [MIM:114480]
BRCA2	P51587	VAR_032713	p.Arg118His	US	rs80358603	One patient with esophageal carcinoma
BRCA2	P51587	VAR_032714	p.Met192Thr	US	rs80358805	One patient with pancreatic cancer
BRCA2	P51587	VAR_032715	p.Thr225Ala	LB/B	rs80358897	-
BRCA2	P51587	VAR_032716	p.Cys315Ser	US	rs79483201	One patient with esophageal carcinoma
BRCA2	P51587	VAR_032717	p.Ser326Arg	LP/P	rs28897706	Breast cancer (BC) [MIM:114480]
BRCA2	P51587	VAR_032718	p.Ile505Thr	LP/P	rs28897708	Breast cancer (BC) [MIM:114480]
BRCA2	P51587	VAR_032719	p.Ile729Met	LP/P	rs397507620	Breast cancer (BC) [MIM:114480]
BRCA2	P51587	VAR_032720	p.Ser1172Leu	US	rs80358600	Breast cancer (BC) [MIM:114480]
BRCA2	P51587	VAR_032721	p.Leu1522Phe	LB/B	rs397507729	-
BRCA2	P51587	VAR_032722	p.Lys1690Asn	LP/P	rs56087561	Breast cancer (BC) [MIM:114480]
BRCA2	P51587	VAR_032723	p.Asn1730Tyr	LP/P	rs397507770	Breast cancer (BC) [MIM:114480]
BRCA2	P51587	VAR_032724	p.Thr1887Met	LP/P	rs397507795	Breast cancer (BC) [MIM:114480]
BRCA2	P51587	VAR_032725	p.Val1988Ile	US	rs28897739	One patient with esophageal carcinoma
BRCA2	P51587	VAR_032726	p.Gly2044Val	LB/B	rs56191579	-
BRCA2	P51587	VAR_032727	p.Arg2108Cys	LB/B	rs55794205	-
BRCA2	P51587	VAR_032728	p.Asn2135His	LP/P	rs80358876	Breast cancer (BC) [MIM:114480]
BRCA2	P51587	VAR_032729	p.Tyr2222Cys	LP/P	rs397507875	Breast cancer (BC) [MIM:114480]
BRCA2	P51587	VAR_032730	p.Arg2336His	LP/P	rs28897743	Fanconi anemia complementation group D1 (FANCD1) [MIM:605724]
BRCA2	P51587	VAR_032731	p.Gln2456Glu	LP/P	rs397507912	Breast cancer (BC) [MIM:114480]
BRCA2	P51587	VAR_032732	p.Leu2510Pro	LP/P	rs80358979	Fanconi anemia complementation group D1 (FANCD1) [MIM:605724]
BRCA2	P51587	VAR_032733	p.Trp2626Cys	LP/P	rs80359013	Fanconi anemia complementation group D1 (FANCD1) [MIM:605724]
BRCA2	P51587	VAR_032734	p.Arg2842Cys	US	rs80359104	One patient with esophageal carcinoma
BRCA2	P51587	VAR_032735	p.Pro3300Ser	US	rs770868371	One patient with esophageal carcinoma
BRCA2	P51587	VAR_035436	p.Ser599Phe	LB/B	rs1046984	-
BRCA2	P51587	VAR_056751	p.Lys513Arg	LB/B	rs28897709	-
BRCA2	P51587	VAR_056752	p.Ile982Leu	LB/B	rs28897717	-
BRCA2	P51587	VAR_056753	p.Val1542Met	LB/B	rs28897729	-
BRCA2	P51587	VAR_056754	p.Val1643Ala	LB/B	rs28897731	-
BRCA2	P51587	VAR_056755	p.Ser1979Arg	LB/B	rs28897737	-
BRCA2	P51587	VAR_056756	p.Asp2238Glu	LB/B	rs28897742	-
BRCA2	P51587	VAR_056757	p.Arg2336Gln	LB/B	rs28897743	-
BRCA2	P51587	VAR_056758	p.Asn2447Asp	LB/B	rs4986859	-
BRCA2	P51587	VAR_056759	p.Leu2686Pro	LB/B	rs28897746	-
BRCA2	P51587	VAR_056760	p.Leu2792Pro	US	rs28897751	Breast cancer (BC) [MIM:114480]
BRCA2	P51587	VAR_056761	p.Leu3101Arg	LB/B	rs28897758	-
BRCA2	P51587	VAR_061563	p.His2116Arg	LB/B	rs55953736	-
BRCA2	P51587	VAR_063911	p.Arg2502Cys	US	rs55716624	Breast cancer (BC) [MIM:114480]
BRCA2	P51587	VAR_063912	p.Ile2627Phe	US	rs80359014	Breast cancer (BC) [MIM:114480]

BRCA2	P51587	VAR_063913	p.Leu2653Pro	US	rs80359022	Breast cancer (BC) [MIM:114480]
BRCA2	P51587	VAR_063914	p.Arg2659Lys	US	rs80359027	Breast cancer (BC) [MIM:114480]
BRCA2	P51587	VAR_063915	p.Glu2663Val	LP/P	rs80359031	-
BRCA2	P51587	VAR_063916	p.Asp2723Gly	LP/P	rs41293513	Breast cancer (BC) [MIM:114480]
BRCA2	P51587	VAR_063917	p.Gly2748Asp	US	rs80359071	Breast cancer (BC) [MIM:114480]
BRCA2	P51587	VAR_063918	p.Arg3052Trp	LP/P	rs45580035	Breast cancer (BC) [MIM:114480]
BRCA2	P51587	VAR_076440	p.Pro606Leu	LB/B	rs80358469	-
BRCC3	P46736	VAR_050097	p.Ile74Val	LB/B	rs28997578	-
BRD1	O95696	VAR_048424	p.Arg38Gly	LB/B	rs11549978	-
BRD1	O95696	VAR_048425	p.Ala321Ser	LB/B	rs12157714	-
BRD1	O95696	VAR_048426	p.Ala730Thr	LB/B	rs35331092	-
BRD1	O95696	VAR_079184	p.Val230Leu	LB/B	-	-
BRD2	P25440	VAR_022132	p.Leu238Phe	LB/B	rs176250	-
BRD2	P25440	VAR_029300	p.Ala474Val	LB/B	rs3918143	-
BRD2	P25440	VAR_029301	p.Arg547Lys	LB/B	rs1049369	-
BRD2	P25440	VAR_041904	p.Gly30Glu	US	-	A glioblastoma multiforme sample
BRD2	P25440	VAR_041905	p.Ala49Gly	LB/B	rs3918144	-
BRD2	P25440	VAR_041906	p.Ala49Ser	LB/B	rs55669504	-
BRD2	P25440	VAR_041907	p.Ala212Pro	LB/B	rs35952031	-
BRD2	P25440	VAR_041908	p.Pro260Gln	LB/B	rs35294809	-
BRD2	P25440	VAR_041909	p.Arg558Gly	US	-	A gastric adenocarcinoma sample
BRD2	P25440	VAR_041910	p.Ala569Thr	LB/B	rs34530779	-
BRD2	P25440	VAR_041911	p.Ala599Pro	LB/B	rs55952113	-
BRD2	P25440	VAR_041912	p.Pro714Leu	US	-	A glioblastoma multiforme sample
BRD3	Q15059	VAR_041913	p.Thr36Asn	US	-	A renal clear cell carcinoma sample
BRD3	Q15059	VAR_041914	p.Ala161Thr	US	-	A gastric adenocarcinoma sample
BRD3	Q15059	VAR_041915	p.Ala172Val	LB/B	rs34609592	-
BRD3	Q15059	VAR_041916	p.Lys435Gln	LB/B	rs36093130	-
BRD3	Q15059	VAR_041917	p.Arg441His	LB/B	rs56017928	-
BRD3	Q15059	VAR_041918	p.Ser447Pro	LB/B	rs55754444	-
BRD4	O60885	VAR_041919	p.Pro37Ser	LB/B	rs35177876	-
BRD4	O60885	VAR_041920	p.Ala371Gly	LB/B	rs55805532	-
BRD4	O60885	VAR_041921	p.Ser563Asn	LB/B	rs55970906	-
BRD4	O60885	VAR_041922	p.Thr598Ser	LB/B	rs34362023	-
BRD4	O60885	VAR_041923	p.Arg669His	LB/B	rs35824241	-
BRD4	O60885	VAR_048427	p.Arg1097His	LB/B	rs35676845	-
BRD8	Q9H0E9	VAR_030695	p.Thr490Met	LB/B	rs11750814	-
BRD8	Q9H0E9	VAR_048428	p.Leu896Pro	LB/B	rs6883021	-
BRD8	Q9H0E9	VAR_048429	p.Gln1198Arg	LB/B	rs412051	-
BRD9	Q9H8M2	VAR_033635	p.Ala266Thr	LB/B	rs34292369	-
BRD9	Q9H8M2	VAR_033636	p.Ala389Thr	LB/B	rs414349	-
BRD9	Q9H8M2	VAR_059143	p.Ala170Thr	LB/B	rs34292369	-
BRDT	Q58F21	VAR_026584	p.Gln62Lys	LB/B	rs10783071	-
BRDT	Q58F21	VAR_026585	p.Lys238Asn	LB/B	rs1156281	-
BRDT	Q58F21	VAR_026586	p.Asn410Lys	LB/B	rs3088232	-
BRDT	Q58F21	VAR_041924	p.Ser2Phe	LB/B	rs55806733	-
BRDT	Q58F21	VAR_041925	p.Arg6Gln	LB/B	rs56273490	-
BRDT	Q58F21	VAR_041926	p.Ala89Val	US	rs781375003	A gastric adenocarcinoma sample
BRDT	Q58F21	VAR_041927	p.His288Tyr	US	-	A lung neuroendocrine carcinoma sample

BRDT Q58F21 VAR_041928 p.Glu357Lys LB/B rs34674879 -
BRDT Q58F21 VAR_041929 p.Pro542Ala LB/B rs55912588 -
BRDT Q58F21 VAR_047327 p.Lys336Thr LB/B rs1064567 -
BRDT Q58F21 VAR_047328 p.Arg605Gln LB/B rs35327986 -
BRDT Q58F21 VAR_047329 p.Pro696Leu LB/B rs10747493 -
BRDT Q58F21 VAR_079306 p.Gly928Asp US rs754258809 Spermatogenic failure 21 (SPGF21) [MIM:617644]
BRF1 Q92994 VAR_035723 p.Val542Met US rs371981699 A colorectal cancer sample
BRF1 Q92994 VAR_072710 p.Arg223Trp LP/P rs370270828 Cerebellofaciodental syndrome (CFDS) [MIM:616202]
BRF1 Q92994 VAR_072711 p.Ser226Leu LP/P rs606231416 Cerebellofaciodental syndrome (CFDS) [MIM:616202]
BRF1 Q92994 VAR_072712 p.Thr259Met LP/P rs373957300 Cerebellofaciodental syndrome (CFDS) [MIM:616202]
BRF1 Q92994 VAR_072713 p.Pro292His LP/P rs606231450 Cerebellofaciodental syndrome (CFDS) [MIM:616202]
BRI3 O95415 VAR_033516 p.Thr123Ala LB/B rs12865 -
BRICD5 Q6PL45 VAR_031629 p.Val46Ile LB/B rs26857 -
BRICD5 Q6PL45 VAR_061620 p.Ser31Gly LB/B rs26856 -
BRICD5 Q6PL45 VAR_061621 p.His122Tyr LB/B rs58292351 -
BRINP1 O60477 VAR_024930 p.Ala437Thr LB/B rs1043377 -
BRINP1 O60477 VAR_029989 p.Ser347Arg LB/B - -
BRINP1 O60477 VAR_029990 p.Arg358His LB/B rs17476783 -
BRINP1 O60477 VAR_036336 p.Pro712Thr US - A colorectal cancer sample
BRINP2 Q9C0B6 VAR_049022 p.Tyr71Cys LB/B rs16850984 -
BRINP2 Q9C0B6 VAR_049023 p.Leu390Val LB/B rs3176443 -
BRIP1 Q9BX63 VAR_020896 p.Pro47Ala LP/P rs28903098 Breast cancer (BC) [MIM:114480]
BRIP1 Q9BX63 VAR_020897 p.Arg173Cys LB/B rs4988345 -
BRIP1 Q9BX63 VAR_020898 p.Val193Ile LB/B rs4988346 -
BRIP1 Q9BX63 VAR_020899 p.Leu195Pro LB/B rs4988347 -
BRIP1 Q9BX63 VAR_020900 p.Met299Ile LP/P rs137852985 Breast cancer (BC) [MIM:114480]
BRIP1 Q9BX63 VAR_020901 p.Arg419Trp LB/B rs150624408 -
BRIP1 Q9BX63 VAR_020902 p.Phe531Val LB/B rs4988350 -
BRIP1 Q9BX63 VAR_020903 p.Gln540Leu LB/B rs4988349 -
BRIP1 Q9BX63 VAR_020904 p.Cys832Tyr LB/B rs4988355 -
BRIP1 Q9BX63 VAR_020905 p.Ser919Pro LB/B rs4986764 -
BRIP1 Q9BX63 VAR_020906 p.Val935Gly LB/B rs4988356 -
BRIP1 Q9BX63 VAR_020907 p.Pro1034Leu US rs1199923024 A patient with ovarian cancer
BRIP1 Q9BX63 VAR_023700 p.Gln255His LP/P - Fanconi anemia complementation group J (FANCJ) [MIM:609054]
BRIP1 Q9BX63 VAR_023701 p.Arg264Trp LB/B rs28997569 -
BRIP1 Q9BX63 VAR_023702 p.Ala349Pro LP/P rs149364097 Fanconi anemia complementation group J (FANCJ) [MIM:609054]
BRIP1 Q9BX63 VAR_023703 p.Trp647Cys LP/P rs786202760 Fanconi anemia complementation group J (FANCJ) [MIM:609054]
BRIP1 Q9BX63 VAR_023704 p.Arg707Cys LP/P rs764803896 Fanconi anemia complementation group J (FANCJ) [MIM:609054]
BRIP1 Q9BX63 VAR_052192 p.Ile633Met LB/B rs28997572 -
BRIP1 Q9BX63 VAR_052193 p.Asp1148Glu LB/B rs28997573 -
BRME1 Q0VDD7 VAR_033356 p.Gly267Arg LB/B rs2305775 -

BRME1 Q0VDD7 VAR_033357 p.Gln500Arg LB/B rs3803892 -
BRPF1 P55201 VAR_028232 p.Gly1117Glu LB/B rs1042294 -
BRPF1 P55201 VAR_048430 p.His1193Gln LB/B rs36081837 -
BRPF1 P55201 VAR_078077 p.Pro370Ser LP/P rs1057519509 Intellectual developmental disorder with dysmorphic facies and ptosis (IDDDFP) [MIM:617333]
BRPF1 P55201 VAR_078078 p.Cys389Arg LP/P rs1057519515 Intellectual developmental disorder with dysmorphic facies and ptosis (IDDDFP) [MIM:617333]
BRPF3 Q9ULD4 VAR_048431 p.Ala278Gly LB/B rs17658935 -
BRPF3 Q9ULD4 VAR_061042 p.Ser177Gly LB/B rs45504893 -
BRS3 P32247 VAR_011844 p.Thr53Pro LB/B rs5232 -
BRS3 P32247 VAR_011845 p.Leu162Gln LB/B rs5234 -
BRSK1 Q8TDC3 VAR_040394 p.Arg303Trp US rs144130246 A gastric adenocarcinoma sample
BRSK1 Q8TDC3 VAR_040395 p.Val319Ile US - A lung large cell carcinoma sample
BRSK1 Q8TDC3 VAR_040396 p.Gly391Glu US - A metastatic melanoma sample
BRSK1 Q8TDC3 VAR_040397 p.Thr531Asn LB/B rs55892637 -
BRSK1 Q8TDC3 VAR_040398 p.Gly749Ser LB/B rs12978445 -
BRSK1 Q8TDC3 VAR_040399 p.Pro764Ala LB/B rs55796422 -
BRWD1 Q9NSI6 VAR_026435 p.Gln83Glu LB/B rs2056844 -
BRWD1 Q9NSI6 VAR_026436 p.Ser1511Pro LB/B rs2183573 -
BRWD1 Q9NSI6 VAR_026437 p.Leu1699Pro LB/B rs1041439 -
BRWD1 Q9NSI6 VAR_057584 p.Lys2156Arg LB/B rs2234548 -
BRWD3 Q6RI45 VAR_031491 p.Lys1288Arg LB/B rs3122407 -
BRWD3 Q6RI45 VAR_036940 p.Lys1596Glu US rs137853272 Mental retardation, X-linked 93 (MRX93) [MIM:300659]
BSCL2 Q96G97 VAR_022375 p.Asn88Ser LP/P rs137852972 Neuronopathy, distal hereditary motor, 5C (HMN5C) [MIM:619112]
BSCL2 Q96G97 VAR_022375 p.Asn88Ser LP/P rs137852972 Spastic paraplegia 17, autosomal dominant (SPG17) [MIM:270685]
BSCL2 Q96G97 VAR_022376 p.Ser90Leu LP/P rs137852973 Neuronopathy, distal hereditary motor, 5C (HMN5C) [MIM:619112]
BSCL2 Q96G97 VAR_022376 p.Ser90Leu LP/P rs137852973 Spastic paraplegia 17, autosomal dominant (SPG17) [MIM:270685]
BSCL2 Q96G97 VAR_022377 p.Ala212Pro LP/P rs137852971 Congenital generalized lipodystrophy 2 (CGL2) [MIM:269700]
BSG P35613 VAR_011720 p.Gly269Val LB/B rs1803203 -
BSG P35613 VAR_013574 p.Glu208Lys LB/B rs104894669 -
BSG P35613 VAR_082637 p.Lys152Asn LB/B rs14704 -
BSG P35613 VAR_082638 p.Val176Leu LB/B rs2229662 -
BSG P35613 VAR_082639 p.Leu206Pro LB/B rs55911144 -
BSG P35613 VAR_084546 p.Leu13Val LB/B - -
BSG P35613 VAR_084547 p.Thr16Ala LB/B - -
BSG P35613 VAR_084548 p.Val26Phe LB/B - -
BSN Q9UPA5 VAR_055105 p.Ala741Thr LB/B rs34762726 -
BSN Q9UPA5 VAR_055106 p.Gly1213Asp LB/B rs35762866 -
BSN Q9UPA5 VAR_055107 p.Ala3863Thr LB/B rs2005557 -
BSND Q8WZ55 VAR_019783 p.Arg8Leu LP/P rs74315288 Bartter syndrome 4A, neonatal, with sensorineural deafness (BARTS4A) [MIM:602522]
BSND Q8WZ55 VAR_019784 p.Arg8Trp LP/P rs74315285 Bartter syndrome 4A, neonatal, with sensorineural deafness (BARTS4A) [MIM:602522]

BSND Q8WZ55 VAR_019785 p.Gly10Ser LP/P rs74315287 Bartter syndrome 4A, neonatal, with sensorineural deafness (BARTS4A) [MIM:602522]
BSND Q8WZ55 VAR_019786 p.Gly47Arg LP/P rs74315289 Bartter syndrome 4A, neonatal, with sensorineural deafness (BARTS4A) [MIM:602522]
BSND Q8WZ55 VAR_061564 p.Val43Ile LB/B rs34561376 -
BSPRY Q5W0U4 VAR_026882 p.Gln293His LB/B rs818711 -
BSPRY Q5W0U4 VAR_026883 p.Thr374Ile LB/B rs3088235 -
BSPRY Q5W0U4 VAR_048397 p.Ala261Pro LB/B rs34089316 -
BST1 Q10588 VAR_021964 p.Arg125His LB/B rs2302465 -
BST1 Q10588 VAR_021965 p.Arg145Gln LB/B rs2302464 -
BST1 Q10588 VAR_028438 p.Ala77Val LB/B rs2302466 -
BST1 Q10588 VAR_028439 p.Ile101Val LB/B rs6840615 -
BST2 Q10589 VAR_012067 p.Val143Phe LB/B rs1804402 -
BTBD10 Q9BSF8 VAR_033638 p.Thr145Ala LB/B rs34185489 -
BTBD11 A6QL63 VAR_042534 p.Ala1002Asp LB/B rs11610050 -
BTBD11 A6QL63 VAR_042535 p.Gly1076Ser LB/B rs12303478 -
BTBD11 A6QL63 VAR_055560 p.Gly448Ser LB/B rs1558781 -
BTBD16 Q32M84 VAR_027070 p.Arg318Gln LB/B rs2421013 -
BTBD16 Q32M84 VAR_027071 p.Gly331Asp LB/B rs986178 -
BTBD16 Q32M84 VAR_027072 p.His439Pro LB/B rs1048347 -
BTBD16 Q32M84 VAR_027073 p.Gln472Arg LB/B rs10510108 -
BTBD18 B2RXH4 VAR_063155 p.Ser302Thr LB/B rs78162678 -
BTBD18 B2RXH4 VAR_063156 p.Glu352Gly LB/B rs201912857 -
BTBD8 Q5XKL5 VAR_033637 p.Lys136Arg LB/B rs17131602 -
BTBD8 Q5XKL5 VAR_048436 p.Val60Ile LB/B rs34856868 -
BTC P35070 VAR_029307 p.Cys7Gly LB/B rs28549760 -
BTC P35070 VAR_029308 p.Leu124Met LB/B rs11938093 -
BTC P35070 VAR_061151 p.Leu44Phe LB/B rs56320257 -
BTD P43251 VAR_005113 p.Phe128Val LP/P rs397514355 Biotinidase deficiency (BTD deficiency) [MIM:253260]
BTD P43251 VAR_005114 p.Ala171Thr LP/P rs13073139 Biotinidase deficiency (BTD deficiency) [MIM:253260]
BTD P43251 VAR_005115 p.Asp228Tyr LP/P rs397514380 Biotinidase deficiency (BTD deficiency) [MIM:253260]
BTD P43251 VAR_005116 p.His323Arg LP/P rs397507176 Biotinidase deficiency (BTD deficiency) [MIM:253260]
BTD P43251 VAR_005117 p.Asp444His LP/P rs13078881 Biotinidase deficiency (BTD deficiency) [MIM:253260]
BTD P43251 VAR_005118 p.Gly451Asp LP/P rs397514419 Biotinidase deficiency (BTD deficiency) [MIM:253260]
BTD P43251 VAR_005119 p.Gln456His LP/P rs80338685 Biotinidase deficiency (BTD deficiency) [MIM:253260]
BTD P43251 VAR_005120 p.Thr532Met LP/P rs104893688 Biotinidase deficiency (BTD deficiency) [MIM:253260]
BTD P43251 VAR_005121 p.Arg538Cys LP/P rs80338686 Biotinidase deficiency (BTD deficiency) [MIM:253260]
BTD P43251 VAR_056238 p.Pro391Ser LB/B rs35034250 -
BTG1 P62324 VAR_021345 p.Asn139Ser LB/B rs28399541 -
BTG1 P62324 VAR_021346 p.Gln141Glu LB/B rs28399542 -

BTG2	P78543	VAR_048437	p.Val153Met	LB/B	rs12039961	-
BTK	Q06187	VAR_006216	p.Leu11Pro	LP/P	rs1603020228	X-linked agammaglobulinemia (XLA) [MIM:300755]
BTK	Q06187	VAR_006217	p.Lys12Arg	LP/P	-	X-linked agammaglobulinemia (XLA) [MIM:300755]
BTK	Q06187	VAR_006218	p.Ser14Phe	LP/P	rs1057520682	X-linked agammaglobulinemia (XLA) [MIM:300755]
BTK	Q06187	VAR_006219	p.Phe25Ser	LP/P	-	X-linked agammaglobulinemia (XLA) [MIM:300755]
BTK	Q06187	VAR_006220	p.Arg28His	LP/P	rs128620185	X-linked agammaglobulinemia (XLA) [MIM:300755]
BTK	Q06187	VAR_006221	p.Arg28Pro	LP/P	-	X-linked agammaglobulinemia (XLA) [MIM:300755]
BTK	Q06187	VAR_006222	p.Thr33Pro	LP/P	rs128620189	X-linked agammaglobulinemia (XLA) [MIM:300755]
BTK	Q06187	VAR_006223	p.Val64Phe	LP/P	-	X-linked agammaglobulinemia (XLA) [MIM:300755]
BTK	Q06187	VAR_006225	p.Val113Asp	LP/P	rs128621190	X-linked agammaglobulinemia (XLA) [MIM:300755]
BTK	Q06187	VAR_006227	p.Arg288Trp	LP/P	rs128621194	X-linked agammaglobulinemia (XLA) [MIM:300755]
BTK	Q06187	VAR_006228	p.Leu295Pro	LP/P	-	X-linked agammaglobulinemia (XLA) [MIM:300755]
BTK	Q06187	VAR_006230	p.Gly302Glu	LP/P	-	X-linked agammaglobulinemia (XLA) [MIM:300755]
BTK	Q06187	VAR_006231	p.Arg307Gly	LP/P	rs128621195	X-linked agammaglobulinemia (XLA) [MIM:300755]
BTK	Q06187	VAR_006232	p.Tyr334Ser	LP/P	rs128621196	X-linked agammaglobulinemia (XLA) [MIM:300755]
BTK	Q06187	VAR_006233	p.Leu358Phe	LP/P	-	X-linked agammaglobulinemia (XLA) [MIM:300755]
BTK	Q06187	VAR_006234	p.Tyr361Cys	LP/P	rs28935478	X-linked agammaglobulinemia (XLA) [MIM:300755]
BTK	Q06187	VAR_006235	p.His362Gln	LP/P	-	X-linked agammaglobulinemia (XLA) [MIM:300755]
BTK	Q06187	VAR_006236	p.His364Pro	LP/P	-	X-linked agammaglobulinemia (XLA) [MIM:300755]
BTK	Q06187	VAR_006237	p.Asn365Tyr	LP/P	-	X-linked agammaglobulinemia (XLA) [MIM:300755]
BTK	Q06187	VAR_006238	p.Ile370Met	LP/P	-	X-linked agammaglobulinemia (XLA) [MIM:300755]
BTK	Q06187	VAR_006239	p.Leu408Pro	LP/P	rs128621198	X-linked agammaglobulinemia (XLA) [MIM:300755]
BTK	Q06187	VAR_006240	p.Tyr418His	LP/P	rs144079566	X-linked agammaglobulinemia (XLA) [MIM:300755]
BTK	Q06187	VAR_006241	p.Ile429Asn	LP/P	-	X-linked agammaglobulinemia (XLA) [MIM:300755]
BTK	Q06187	VAR_006242	p.Lys430Glu	LP/P	rs128620184	X-linked agammaglobulinemia (XLA) [MIM:300755]
BTK	Q06187	VAR_006243	p.Tyr476Asp	LP/P	-	X-linked agammaglobulinemia (XLA) [MIM:300755]
BTK	Q06187	VAR_006244	p.Met477Arg	LP/P	-	X-linked agammaglobulinemia (XLA) [MIM:300755]
BTK	Q06187	VAR_006245	p.Cys502Phe	LP/P	-	X-linked agammaglobulinemia (XLA) [MIM:300755]
BTK	Q06187	VAR_006246	p.Cys502Trp	LP/P	rs41310709	X-linked agammaglobulinemia (XLA) [MIM:300755]
BTK	Q06187	VAR_006247	p.Cys506Arg	LP/P	rs128621200	X-linked agammaglobulinemia (XLA) [MIM:300755]
BTK	Q06187	VAR_006248	p.Cys506Tyr	LP/P	-	X-linked agammaglobulinemia (XLA) [MIM:300755]
BTK	Q06187	VAR_006249	p.Met509Val	LP/P	-	X-linked agammaglobulinemia (XLA) [MIM:300755]
BTK	Q06187	VAR_006251	p.Arg520Gln	LP/P	rs128621202	X-linked agammaglobulinemia (XLA) [MIM:300755]
BTK	Q06187	VAR_006252	p.Asp521His	LP/P	-	X-linked agammaglobulinemia (XLA) [MIM:300755]
BTK	Q06187	VAR_006253	p.Asp521Asn	LP/P	-	X-linked agammaglobulinemia (XLA) [MIM:300755]
BTK	Q06187	VAR_006254	p.Arg525Pro	LP/P	-	X-linked agammaglobulinemia (XLA) [MIM:300755]
BTK	Q06187	VAR_006255	p.Arg525Gln	LP/P	rs128620183	X-linked agammaglobulinemia (XLA) [MIM:300755]
BTK	Q06187	VAR_006256	p.Asn526Lys	LP/P	rs1569291237	X-linked agammaglobulinemia (XLA) [MIM:300755]
BTK	Q06187	VAR_006257	p.Leu542Pro	LP/P	rs128621203	X-linked agammaglobulinemia (XLA) [MIM:300755]
BTK	Q06187	VAR_006258	p.Arg544Lys	LP/P	-	X-linked agammaglobulinemia (XLA) [MIM:300755]
BTK	Q06187	VAR_006259	p.Arg562Pro	LP/P	rs104894770	X-linked agammaglobulinemia (XLA) [MIM:300755]
BTK	Q06187	VAR_006260	p.Arg562Trp	LP/P	rs128621204	X-linked agammaglobulinemia (XLA) [MIM:300755]
BTK	Q06187	VAR_006261	p.Glu567Lys	LP/P	-	X-linked agammaglobulinemia (XLA) [MIM:300755]
BTK	Q06187	VAR_006262	p.Trp581Arg	LP/P	rs128621205	X-linked agammaglobulinemia (XLA) [MIM:300755]

BTK	Q06187	VAR_006263	p.Ala582Val	LP/P	-	X-linked agammaglobulinemia (XLA) [MIM:300755]
BTK	Q06187	VAR_006264	p.Met587Leu	LP/P	rs1603001822	X-linked agammaglobulinemia (XLA) [MIM:300755]
BTK	Q06187	VAR_006265	p.Glu589Gly	LP/P	rs128621206	X-linked agammaglobulinemia (XLA) [MIM:300755]
BTK	Q06187	VAR_006267	p.Ser592Pro	LP/P	rs1603001783	X-linked agammaglobulinemia (XLA) [MIM:300755]
BTK	Q06187	VAR_006268	p.Gly594Glu	LP/P	-	X-linked agammaglobulinemia (XLA) [MIM:300755]
BTK	Q06187	VAR_006269	p.Gly594Arg	LP/P	rs1555977339	X-linked agammaglobulinemia (XLA) [MIM:300755]
BTK	Q06187	VAR_006270	p.Tyr598Cys	LP/P	-	X-linked agammaglobulinemia (XLA) [MIM:300755]
BTK	Q06187	VAR_006271	p.Ala607Asp	LP/P	rs128621208	X-linked agammaglobulinemia (XLA) [MIM:300755]
BTK	Q06187	VAR_006272	p.Gly613Asp	LP/P	rs128621209	X-linked agammaglobulinemia (XLA) [MIM:300755]
BTK	Q06187	VAR_006273	p.Pro619Ser	LP/P	-	X-linked agammaglobulinemia (XLA) [MIM:300755]
BTK	Q06187	VAR_006274	p.Met630Ile	LB/B	-	-
BTK	Q06187	VAR_006275	p.Met630Lys	LP/P	rs128621210	X-linked agammaglobulinemia (XLA) [MIM:300755]
BTK	Q06187	VAR_006276	p.Cys633Tyr	LP/P	-	X-linked agammaglobulinemia (XLA) [MIM:300755]
BTK	Q06187	VAR_006277	p.Arg641Cys	LP/P	-	X-linked agammaglobulinemia (XLA) [MIM:300755]
BTK	Q06187	VAR_006278	p.Arg641His	LP/P	-	X-linked agammaglobulinemia (XLA) [MIM:300755]
BTK	Q06187	VAR_006279	p.Phe644Ser	LP/P	-	X-linked agammaglobulinemia (XLA) [MIM:300755]
BTK	Q06187	VAR_006280	p.Leu647Pro	LP/P	-	X-linked agammaglobulinemia (XLA) [MIM:300755]
BTK	Q06187	VAR_006281	p.Leu652Pro	LP/P	rs128622212	X-linked agammaglobulinemia (XLA) [MIM:300755]
BTK	Q06187	VAR_008291	p.Lys19Glu	LP/P	-	X-linked agammaglobulinemia (XLA) [MIM:300755]
BTK	Q06187	VAR_008292	p.Lys27Arg	LP/P	-	X-linked agammaglobulinemia (XLA) [MIM:300755]
BTK	Q06187	VAR_008293	p.Arg28Cys	LP/P	-	X-linked agammaglobulinemia (XLA) [MIM:300755]
BTK	Q06187	VAR_008294	p.Tyr40Cys	LP/P	rs1555980875	X-linked agammaglobulinemia (XLA) [MIM:300755]
BTK	Q06187	VAR_008295	p.Tyr40Asn	LP/P	-	X-linked agammaglobulinemia (XLA) [MIM:300755]
BTK	Q06187	VAR_008296	p.Ile61Asn	LP/P	-	X-linked agammaglobulinemia (XLA) [MIM:300755]
BTK	Q06187	VAR_008297	p.Val64Asp	LP/P	-	X-linked agammaglobulinemia (XLA) [MIM:300755]
BTK	Q06187	VAR_008298	p.Ser115Phe	LP/P	-	X-linked agammaglobulinemia (XLA) [MIM:300755]
BTK	Q06187	VAR_008299	p.Thr117Pro	LP/P	-	X-linked agammaglobulinemia (XLA) [MIM:300755]
BTK	Q06187	VAR_008300	p.Gln127His	LP/P	-	X-linked agammaglobulinemia (XLA) [MIM:300755]
BTK	Q06187	VAR_008301	p.Cys154Ser	LP/P	-	X-linked agammaglobulinemia (XLA) [MIM:300755]
BTK	Q06187	VAR_008302	p.Cys155Gly	LP/P	-	X-linked agammaglobulinemia (XLA) [MIM:300755]
BTK	Q06187	VAR_008303	p.Cys155Arg	LP/P	-	X-linked agammaglobulinemia (XLA) [MIM:300755]
BTK	Q06187	VAR_008304	p.Thr184Pro	LP/P	-	X-linked agammaglobulinemia (XLA) [MIM:300755]
BTK	Q06187	VAR_008305	p.Arg288Gln	LP/P	rs1555978277	X-linked agammaglobulinemia (XLA) [MIM:300755]
BTK	Q06187	VAR_008306	p.Gly302Arg	LP/P	-	X-linked agammaglobulinemia (XLA) [MIM:300755]
BTK	Q06187	VAR_008307	p.Arg307Thr	LP/P	-	X-linked agammaglobulinemia (XLA) [MIM:300755]
BTK	Q06187	VAR_008308	p.Asp308Glu	LP/P	-	X-linked agammaglobulinemia (XLA) [MIM:300755]
BTK	Q06187	VAR_008309	p.Val319Ala	LP/P	-	X-linked agammaglobulinemia (XLA) [MIM:300755]
BTK	Q06187	VAR_008310	p.Ser366Phe	LP/P	-	X-linked agammaglobulinemia (XLA) [MIM:300755]
BTK	Q06187	VAR_008311	p.Leu369Phe	LP/P	-	X-linked agammaglobulinemia (XLA) [MIM:300755]
BTK	Q06187	VAR_008312	p.Arg372Gly	LP/P	-	X-linked agammaglobulinemia (XLA) [MIM:300755]
BTK	Q06187	VAR_008313	p.Gly414Arg	LP/P	-	X-linked agammaglobulinemia (XLA) [MIM:300755]
BTK	Q06187	VAR_008314	p.Lys430Arg	LP/P	-	X-linked agammaglobulinemia (XLA) [MIM:300755]
BTK	Q06187	VAR_008315	p.Glu445Asp	LP/P	-	X-linked agammaglobulinemia (XLA) [MIM:300755]

BTK	Q06187	VAR_008316	p.Gly462Asp	LP/P	-	X-linked agammaglobulinemia (XLA) [MIM:300755]
BTK	Q06187	VAR_008317	p.Gly462Val	LP/P	-	X-linked agammaglobulinemia (XLA) [MIM:300755]
BTK	Q06187	VAR_008318	p.Ala508Asp	LP/P	-	X-linked agammaglobulinemia (XLA) [MIM:300755]
BTK	Q06187	VAR_008319	p.Met509Ile	LP/P	-	X-linked agammaglobulinemia (XLA) [MIM:300755]
BTK	Q06187	VAR_008320	p.Leu518Arg	LP/P	-	X-linked agammaglobulinemia (XLA) [MIM:300755]
BTK	Q06187	VAR_008321	p.Asp521Gly	LP/P	-	X-linked agammaglobulinemia (XLA) [MIM:300755]
BTK	Q06187	VAR_008322	p.Ala523Glu	LP/P	-	X-linked agammaglobulinemia (XLA) [MIM:300755]
BTK	Q06187	VAR_008323	p.Arg525Gly	LP/P	rs886041149	X-linked agammaglobulinemia (XLA) [MIM:300755]
BTK	Q06187	VAR_008324	p.Val535Phe	LP/P	-	X-linked agammaglobulinemia (XLA) [MIM:300755]
BTK	Q06187	VAR_008325	p.Phe559Ser	LP/P	-	X-linked agammaglobulinemia (XLA) [MIM:300755]
BTK	Q06187	VAR_008326	p.Trp563Leu	LP/P	rs1555977474	X-linked agammaglobulinemia (XLA) [MIM:300755]
BTK	Q06187	VAR_008327	p.Phe583Ser	LP/P	-	X-linked agammaglobulinemia (XLA) [MIM:300755]
BTK	Q06187	VAR_008328	p.Glu589Asp	LP/P	-	X-linked agammaglobulinemia (XLA) [MIM:300755]
BTK	Q06187	VAR_008330	p.Pro619Ala	LP/P	-	X-linked agammaglobulinemia (XLA) [MIM:300755]
BTK	Q06187	VAR_008331	p.Pro619Thr	LP/P	-	X-linked agammaglobulinemia (XLA) [MIM:300755]
BTK	Q06187	VAR_008332	p.Ala622Pro	LP/P	-	X-linked agammaglobulinemia (XLA) [MIM:300755]
BTK	Q06187	VAR_008333	p.Val626Gly	LP/P	-	X-linked agammaglobulinemia (XLA) [MIM:300755]
BTK	Q06187	VAR_008334	p.Met630Thr	LP/P	-	X-linked agammaglobulinemia (XLA) [MIM:300755]
BTK	Q06187	VAR_008335	p.Phe644Leu	LP/P	-	X-linked agammaglobulinemia (XLA) [MIM:300755]
BTK	Q06187	VAR_008960	p.Tyr39Ser	LP/P	-	X-linked agammaglobulinemia (XLA) [MIM:300755]
BTK	Q06187	VAR_008961	p.Leu512Pro	LP/P	-	X-linked agammaglobulinemia (XLA) [MIM:300755]
BTK	Q06187	VAR_008962	p.Leu512Gln	LP/P	-	X-linked agammaglobulinemia (XLA) [MIM:300755]
BTK	Q06187	VAR_008963	p.Arg544Gly	LP/P	-	X-linked agammaglobulinemia (XLA) [MIM:300755]
BTK	Q06187	VAR_008964	p.Ser578Tyr	LP/P	-	X-linked agammaglobulinemia (XLA) [MIM:300755]
BTK	Q06187	VAR_008965	p.Glu589Lys	LP/P	-	X-linked agammaglobulinemia (XLA) [MIM:300755]
BTK	Q06187	VAR_041676	p.Arg82Lys	LB/B	rs56035945	-
BTK	Q06187	VAR_041677	p.Pro190Lys	US	-	A lung large cell carcinoma sample
BTK	Q06187	VAR_074309	p.Cys481Ser	US	rs1057519825	-
BTLA	Q7Z6A9	VAR_027607	p.Arg157Ser	LB/B	rs2931761	-
BTLA	Q7Z6A9	VAR_027608	p.Pro267Leu	LB/B	rs9288952	-
BTLA	Q7Z6A9	VAR_056027	p.Ile124Val	LB/B	rs16859633	-
BTN1A1	Q13410	VAR_021169	p.Ala213Thr	LB/B	rs3736781	-
BTN1A1	Q13410	VAR_026546	p.Asp503Glu	LB/B	rs9393728	-
BTN1A1	Q13410	VAR_030770	p.Val303Ala	LB/B	rs1980600	-
BTN1A1	Q13410	VAR_061302	p.Pro521Ser	LB/B	rs35555795	-
BTN2A1	Q7KYR7	VAR_049825	p.Arg124Cys	LB/B	rs3734539	-
BTN2A1	Q7KYR7	VAR_049826	p.Val207Met	LB/B	rs13195509	-
BTN2A1	Q7KYR7	VAR_049827	p.Arg378Gln	LB/B	rs3734542	-
BTN2A1	Q7KYR7	VAR_049828	p.Gly451Ala	LB/B	rs3734543	-
BTN2A1	Q7KYR7	VAR_061303	p.Trp178Cys	LB/B	rs13195402	-
BTN2A1	Q7KYR7	VAR_061304	p.Trp178Leu	LB/B	rs13195401	-
BTN2A2	Q8WVV5	VAR_049829	p.Pro479Ser	LB/B	rs16891646	-
BTN2A3P	Q96KV6	VAR_049830	p.Gly79Asp	LB/B	rs7745238	-
BTN2A3P	Q96KV6	VAR_049831	p.Val148Ile	LB/B	rs10946829	-
BTN2A3P	Q96KV6	VAR_049832	p.Asn300Ser	LB/B	rs2893848	-
BTN3A1	O00481	VAR_021170	p.Ser224Asn	LB/B	rs1057933	-
BTN3A1	O00481	VAR_028788	p.Pro456Thr	LB/B	rs4712990	-
BTN3A1	O00481	VAR_061305	p.Arg15His	LB/B	rs56161420	-

BTN3A1 O00481 VAR_061306 p.Arg282Thr LB/B rs41266839 -
BTN3A2 P78410 VAR_026211 p.Asn181Asp LB/B rs9358936 -
BTN3A2 P78410 VAR_049833 p.Arg167Thr LB/B rs9379861 -
BTN3A2 P78410 VAR_049834 p.Ala182Thr LB/B rs12205731 -
BTN3A2 P78410 VAR_049835 p.Arg211Lys LB/B rs35183513 -
BTN3A2 P78410 VAR_049836 p.Ser307Asn LB/B rs13216828 -
BTNL2 Q9UIR0 VAR_021171 p.Lys196Glu LB/B rs2076523 -
BTNL2 Q9UIR0 VAR_029128 p.Ser334Leu LB/B rs28362679 -
BTNL2 Q9UIR0 VAR_033602 p.Trp94Arg LB/B rs28362682 -
BTNL2 Q9UIR0 VAR_033603 p.Asp283Val LB/B rs34423804 -
BTNL2 Q9UIR0 VAR_033604 p.Ala352Thr LB/B rs35037492 -
BTNL2 Q9UIR0 VAR_033605 p.Pro379Leu LB/B rs28362678 -
BTNL2 Q9UIR0 VAR_033606 p.Met380Ile LB/B rs28362677 -
BTNL2 Q9UIR0 VAR_033607 p.Pro393Gln LB/B rs41521946 -
BTNL2 Q9UIR0 VAR_049837 p.Val188Met LB/B rs9461742 -
BTNL2 Q9UIR0 VAR_049838 p.Ser360Gly LB/B rs2076530 -
BTNL2 Q9UIR0 VAR_061307 p.Arg181Gln LB/B rs28362681 -
BTNL2 Q9UIR0 VAR_061308 p.Ala202Val LB/B rs28362680 -
BTNL8 Q6UX41 VAR_049839 p.Thr143Ala LB/B rs2276995 -
BTNL8 Q6UX41 VAR_049840 p.Glu229Lys LB/B rs7724813 -
BTNL8 Q6UX41 VAR_069135 p.His311Gln LB/B rs146303629 -
BTNL8 Q6UX41 VAR_069136 p.Thr326Lys LB/B rs7703365 -
BTNL8 Q6UX41 VAR_069137 p.Ala477Thr LB/B rs113820381 -
BTNL9 Q6UXG8 VAR_049841 p.Gly511Arg LB/B rs10068763 -
BTRC Q9Y297 VAR_020119 p.Pro592His LB/B rs2270439 -
BTRC Q9Y297 VAR_022027 p.Ala543Ser LB/B rs4151060 -
BUB1 O43683 VAR_008849 p.Glu36Asp US rs1801328 Colorectal cancer
BUB1 O43683 VAR_008850 p.Ser492Tyr US rs121909055 Colorectal cancer
BUB1 O43683 VAR_008851 p.Pro648Arg US rs376649190 Colorectal cancer
BUB1 O43683 VAR_015687 p.Tyr259Cys US - Pancreatic cancer
BUB1 O43683 VAR_015688 p.His265Asn US - Pancreatic cancer
BUB1 O43683 VAR_040400 p.Gly20Asp LB/B rs35890336 -
BUB1 O43683 VAR_040401 p.Asn534Asp LB/B rs36109304 -
BUB1B O60566 VAR_008852 p.Met15Thr US rs1392369693 A colorectal cancer cell line
BUB1B O60566 VAR_008853 p.Arg349Gln LB/B rs1801376 -
BUB1B O60566 VAR_008854 p.Val618Ala LB/B rs1801528 -
BUB1B O60566 VAR_028921 p.Arg36Gln LP/P rs534297115 Premature chromatid separation trait (PCS) [MIM:176430]
BUB1B O60566 VAR_028922 p.Glu390Asp LB/B rs1017842 -
BUB1B O60566 VAR_028923 p.Arg550Gln LP/P rs28989187 Mosaic variegated aneuploidy syndrome 1 (MVA1) [MIM:257300]
BUB1B O60566 VAR_028924 p.Arg814His LP/P rs28989182 Mosaic variegated aneuploidy syndrome 1 (MVA1) [MIM:257300]
BUB1B O60566 VAR_028925 p.Leu844Phe LP/P rs28989181 Mosaic variegated aneuploidy syndrome 1 (MVA1) [MIM:257300]
BUB1B O60566 VAR_028926 p.Ile909Thr LP/P rs28989184 Mosaic variegated aneuploidy syndrome 1 (MVA1) [MIM:257300]
BUB1B O60566 VAR_028927 p.Gln921His LP/P rs28989183 Mosaic variegated aneuploidy syndrome 1 (MVA1) [MIM:257300]

BUB1B O60566 VAR_028928 p.Leu1012Pro LP/P rs28989185 Mosaic variegated aneuploidy syndrome 1 (MVA1) [MIM:257300]
BUB1B O60566 VAR_040402 p.Thr40Met LB/B rs56079734 -
BUB1B O60566 VAR_054549 p.Pro378Ser LB/B rs17851677 -
BUD13 Q9BRD0 VAR_032343 p.Arg120Cys LB/B rs10488698 -
BUD13 Q9BRD0 VAR_032344 p.Pro148Leu LB/B rs11820589 -
BUD13 Q9BRD0 VAR_032345 p.Arg242Ile LB/B rs11216131 -
BUD13 Q9BRD0 VAR_053908 p.Ser388Cys LB/B rs35004487 -
BVES Q8NE79 VAR_017155 p.Arg129Trp LB/B rs2275289 -
BVES Q8NE79 VAR_053600 p.Met127Ile LB/B rs9486039 -
BVES Q8NE79 VAR_075625 p.Ser201Phe LP/P rs869025337 Muscular dystrophy, limb-girdle, autosomal recessive 25 (LGMDR25) [MIM:616812]
BYSL Q13895 VAR_033641 p.Glu103Lys LB/B rs2296916 -
BYSL Q13895 VAR_048439 p.Pro426Ser LB/B rs3828855 -
BZW2 Q9Y6E2 VAR_033642 p.Asp44Ala LB/B rs35233079 -
C10orf113 Q5VZT2 VAR_028801 p.Asp110His LB/B rs625223 -
C10orf113 Q5VZT2 VAR_050858 p.Arg72Gly LB/B rs11591355 -
C10orf120 Q5SQS8 VAR_050859 p.Thr20Lys LB/B rs41448048 -
C10orf120 Q5SQS8 VAR_050860 p.Ala269Thr LB/B rs2947594 -
C10orf62 Q5T681 VAR_030216 p.Glu121Asp LB/B rs7093840 -
C10orf82 Q8WW14 VAR_050854 p.Thr124Met LB/B rs11551267 -
C10orf90 Q96M02 VAR_030908 p.Arg134His LB/B rs11245008 -
C10orf90 Q96M02 VAR_030909 p.Asp262Asn LB/B rs11245007 -
C10orf90 Q96M02 VAR_050855 p.Met57Ile LB/B rs11558415 -
C10orf90 Q96M02 VAR_050856 p.Asp531Glu LB/B rs12412320 -
C11orf1 Q9H5F2 VAR_050861 p.Lys49Gln LB/B rs11540721 -
C11orf1 Q9H5F2 VAR_050862 p.Gln85His LB/B rs3180820 -
C11orf1 Q9H5F2 VAR_054163 p.Val40Ala LB/B rs9280 -
C11orf16 Q9NQ32 VAR_056827 p.Pro144Leu LB/B rs2568076 -
C11orf16 Q9NQ32 VAR_056828 p.Val210Leu LB/B rs11042127 -
C11orf24 Q96F05 VAR_027713 p.Gly97Val LB/B rs3802746 -
C11orf24 Q96F05 VAR_027714 p.Ala150Thr LB/B rs901827 -
C11orf40 Q8WZ69 VAR_029581 p.Phe100Ser LB/B rs12795289 -
C11orf42 Q8N5U0 VAR_026831 p.Pro242Ser LB/B rs10769671 -
C11orf52 Q96A22 VAR_029587 p.Thr23Arg LB/B rs7124407 -
C11orf68 Q9H3H3 VAR_060319 p.Gln195Arg LB/B rs7947504 -
C11orf72 Q8NBR9 VAR_029584 p.Pro22Thr LB/B rs12421329 -
C11orf94 C9JXX5 VAR_063154 p.Gln80His LB/B rs2271849 -
C11orf96 Q7Z7L8 VAR_039242 p.Thr26Ala LB/B rs1973717 -
C11orf96 Q7Z7L8 VAR_039243 p.Ser47Pro LB/B rs12797684 -
C11orf96 Q7Z7L8 VAR_039244 p.Arg110Ser LB/B rs12796438 -
C11orf96 Q7Z7L8 VAR_039245 p.Arg144Cys LB/B rs12796667 -
C11orf96 Q7Z7L8 VAR_039246 p.Phe151Ser LB/B rs12798337 -
C11orf96 Q7Z7L8 VAR_039247 p.Pro352Ser LB/B rs2434483 -
C12orf29 Q8N999 VAR_035197 p.Pro23Leu LB/B rs11541954 -
C12orf29 Q8N999 VAR_035198 p.Val238Leu LB/B rs9262 -
C12orf4 Q9NQ89 VAR_081778 p.Leu328Pro LP/P rs1468772495 Mental retardation, autosomal recessive 66 (MRT66) [MIM:618221]
C12orf40 Q86WS4 VAR_061610 p.Ile13Leu LB/B rs58302581 -

C12orf42	Q96LP6	VAR_030229	p.Glu11Asp	LB/B	rs10778257	-	
C12orf42	Q96LP6	VAR_030230	p.Pro182Arg	LB/B	rs7484376	-	
C12orf50	Q8NA57	VAR_033266	p.Gln306Arg	LB/B	rs10777084	-	
C12orf50	Q8NA57	VAR_033267	p.Arg322His	LB/B	rs11104703	-	
C12orf54	Q6X4T0	VAR_030252	p.Pro86Ser	LB/B	rs11458	-	
C12orf57	Q99622	VAR_069774	p.Leu51Gln	LP/P	rs587776955	Temtamy syndrome (TEMTYS) [MIM:218340]	
C12orf60	Q5U649	VAR_030226	p.Lys51Arg	LB/B	rs17853860	-	
C12orf60	Q5U649	VAR_030227	p.Lys65Arg	LB/B	rs7304054	-	
C12orf60	Q5U649	VAR_030228	p.Asn103Lys	LB/B	rs7307438	-	
C12orf71	A8MTZ7	VAR_056837	p.Ile140Val	LB/B	rs708167	-	
C12orf76	Q8N812	VAR_039916	p.Ala26Thr	US	-	A colorectal cancer sample	
C14orf119	Q9NWQ9	VAR_033744	p.Leu16Val	LB/B	rs35065609	-	
C14orf177	Q52M58	VAR_030224	p.Arg3Trp	LB/B	rs17097718	-	
C14orf177	Q52M58	VAR_030225	p.Val33Ala	LB/B	rs4905757	-	
C14orf178	Q8N769	VAR_030248	p.His26Tyr	LB/B	rs8015313	-	
C14orf93	Q9H972	VAR_050875	p.Ala190Val	LB/B	rs3829409	-	
C15orf32	Q32M92	VAR_050884	p.Ala17Thr	LB/B	rs1455773	-	
C15orf39	Q6ZRI6	VAR_026891	p.Ala119Pro	LB/B	rs1873379	-	
C15orf39	Q6ZRI6	VAR_026892	p.Gly491Asp	LB/B	rs11072532	-	
C15orf39	Q6ZRI6	VAR_026893	p.Ser536Ala	LB/B	rs28509789	-	
C15orf39	Q6ZRI6	VAR_026894	p.Gly945Asp	LB/B	rs3743211	-	
C15orf40	Q8WUR7	VAR_069397	p.Ala89Thr	LB/B	rs376937425	-	
C15orf54	Q8N8G6	VAR_044189	p.Thr59Met	LB/B	rs11853050	-	
C15orf54	Q8N8G6	VAR_044190	p.Glu77Lys	LB/B	rs16968547	-	
C16orf46	Q6P387	VAR_030893	p.Thr77Ser	LB/B	rs17855893	-	
C16orf46	Q6P387	VAR_030894	p.Ile288Thr	LB/B	rs7198494	-	
C16orf46	Q6P387	VAR_030895	p.Tyr335His	LB/B	rs10459872	-	
C16orf46	Q6P387	VAR_030896	p.Lys357Glu	LB/B	rs9930623	-	
C16orf46	Q6P387	VAR_030897	p.Pro388Ser	LB/B	rs12929250	-	
C16orf72	Q14CZ0	VAR_034657	p.Asn221Ser	LB/B	rs34869458	-	
C16orf78	Q8WTQ4	VAR_034632	p.Arg152Gln	LB/B	rs16947350	-	
C16orf89	Q6UX73	VAR_040004	p.Leu296Ser	LB/B	rs1127920	-	
C16orf89	Q6UX73	VAR_040005	p.Tyr363His	LB/B	rs11642955	-	
C16orf89	Q6UX73	VAR_057992	p.Gly215Ala	LB/B	rs17853191	-	
C17orf50	Q8WW18	VAR_050899	p.Asp22Glu	LB/B	rs4795087	-	
C17orf58	Q2M2W7	VAR_030902	p.Ile92Val	LB/B	rs9891146	-	
C17orf77	Q96MU5	VAR_029628	p.Arg47Ser	LB/B	rs493430	-	
C17orf78	Q8N4C9	VAR_034877	p.Thr152Ser	LB/B	rs1714987	-	
C17orf80	Q9BSJ5	VAR_031779	p.Gly226Ser	LB/B	rs9902726	-	
C17orf80	Q9BSJ5	VAR_031780	p.Lys322Gln	LB/B	rs34784472	-	
C17orf80	Q9BSJ5	VAR_031781	p.Phe356Leu	LB/B	rs745143	-	
C17orf80	Q9BSJ5	VAR_031782	p.His395Asn	LB/B	rs904384	-	
C17orf80	Q9BSJ5	VAR_031783	p.Cys396Arg	LB/B	rs904383	-	
C17orf80	Q9BSJ5	VAR_031784	p.Gln420His	LB/B	rs745142	-	
C17orf80	Q9BSJ5	VAR_031785	p.Ala522Thr	LB/B	rs1566286	-	
C17orf98	A8MV24	VAR_044022	p.Pro61Leu	LB/B	rs7210156	-	
C17orf99	Q6UX52	VAR_067466	p.Trp101Arg	LB/B	rs4789017	-	
C18orf21	Q32NC0	VAR_030903	p.Thr132Ala	LB/B	rs2276314	-	
C18orf32	Q8TCD1	VAR_039374	p.Pro37His	LB/B	rs11537626	-	

C19orf12 Q9NSK7 VAR_066617 p.Thr11Met LP/P rs397514477 Neurodegeneration with brain iron accumulation 4 (NBIA4) [MIM:614298]
C19orf12 Q9NSK7 VAR_066618 p.Gly53Arg LP/P rs200133991 Neurodegeneration with brain iron accumulation 4 (NBIA4) [MIM:614298]
C19orf12 Q9NSK7 VAR_066619 p.Gly65Glu LP/P rs752450983 Neurodegeneration with brain iron accumulation 4 (NBIA4) [MIM:614298]
C19orf12 Q9NSK7 VAR_066620 p.Gly69Arg LP/P rs515726205 Neurodegeneration with brain iron accumulation 4 (NBIA4) [MIM:614298]
C19orf12 Q9NSK7 VAR_066621 p.Lys142Glu US rs146170087 -
C19orf12 Q9NSK7 VAR_066622 p.Lys142Thr LB/B rs79915936 -
C19orf12 Q9NSK7 VAR_069756 p.Ser39Phe LP/P rs1204865094 Neurodegeneration with brain iron accumulation 4 (NBIA4) [MIM:614298]
C19orf12 Q9NSK7 VAR_069757 p.Ala48Pro LP/P - Neurodegeneration with brain iron accumulation 4 (NBIA4) [MIM:614298]
C19orf12 Q9NSK7 VAR_069758 p.Pro60Leu LP/P rs1424999393 Neurodegeneration with brain iron accumulation 4 (NBIA4) [MIM:614298]
C19orf12 Q9NSK7 VAR_069759 p.Gly65Val LP/P rs752450983 Neurodegeneration with brain iron accumulation 4 (NBIA4) [MIM:614298]
C19orf12 Q9NSK7 VAR_069760 p.Pro83Leu LP/P rs201987973 Neurodegeneration with brain iron accumulation 4 (NBIA4) [MIM:614298]
C19orf12 Q9NSK7 VAR_069761 p.Arg98Ser LP/P rs1384930997 Neurodegeneration with brain iron accumulation 4 (NBIA4) [MIM:614298]
C19orf12 Q9NSK7 VAR_069762 p.Leu121Gln LP/P - Neurodegeneration with brain iron accumulation 4 (NBIA4) [MIM:614298]
C19orf12 Q9NSK7 VAR_069763 p.Ala134Pro US rs1264612218 Neurodegeneration with brain iron accumulation 4 (NBIA4) [MIM:614298]
C19orf12 Q9NSK7 VAR_069764 p.Gln149Arg LB/B rs73023451 -
C19orf12 Q9NSK7 VAR_070668 p.Ala63Pro LP/P rs376103979 Neurodegeneration with brain iron accumulation 4 (NBIA4) [MIM:614298]
C19orf12 Q9NSK7 VAR_070668 p.Ala63Pro LP/P rs376103979 Spastic paraplegia 43, autosomal recessive (SPG43) [MIM:615043]
C19orf12 Q9NSK7 VAR_076803 p.Gly58Ser LP/P rs1358503478 Neurodegeneration with brain iron accumulation 4 (NBIA4) [MIM:614298]
C19orf12 Q9NSK7 VAR_076804 p.Gln96Pro LP/P - Neurodegeneration with brain iron accumulation 4 (NBIA4) [MIM:614298]
C19orf18 Q8NEA5 VAR_050909 p.His208Tyr LB/B rs8110831 -
C19orf48 Q6RUI8 VAR_029852 p.Val7Ile LB/B rs7251816 -
C19orf48 Q6RUI8 VAR_029853 p.Arg84Cys LB/B rs4802741 -
C19orf48 Q6RUI8 VAR_029854 p.Gly104Glu LB/B rs4801853 -
C19orf53 Q9UNZ5 VAR_053783 p.Lys39Arg LB/B rs10104 -
C19orf71 A6NCJ1 VAR_061253 p.Pro83Leu LB/B rs12608919 -
C19orf71 A6NCJ1 VAR_061254 p.Ala137Val LB/B rs6510758 -
C19orf73 Q9NVV2 VAR_046164 p.Ser106Gly LB/B rs2232003 -
C1D Q13901 VAR_053990 p.Ser127Pro LB/B rs10444 -
C1GALT1C1 Q96EU7 VAR_031910 p.Asp131Glu LB/B rs17261572 -
C1GALT1C1 Q96EU7 VAR_031911 p.Glu152Lys LP/P rs137853599 Tn polyagglutination syndrome (TNPS) [MIM:300622]
C1GALT1C1 Q96EU7 VAR_069274 p.Ala143Val LB/B rs45557031 -

C1GALT1C1	Q96EU7	VAR_069275	p.Ser193Pro	LP/P	rs397514537	Tn polyagglutination syndrome (TNPS) [MIM:300622]
C1GALT1C1	Q96EU7	VAR_069276	p.Gln222His	LB/B	rs200973382	-
C1orf105	O95561	VAR_027710	p.Met97Val	LB/B	rs16844498	-
C1orf105	O95561	VAR_027711	p.Ser137Asn	LB/B	rs1129942	-
C1orf112	Q9NSG2	VAR_030906	p.Ala481Ser	LB/B	rs2272920	-
C1orf127	Q8N9H9	VAR_031750	p.Ala530Val	LB/B	rs1281018	-
C1orf127	Q8N9H9	VAR_056766	p.Arg256Gln	LB/B	rs1281012	-
C1orf127	Q8N9H9	VAR_056767	p.Val366Asp	LB/B	rs1281016	-
C1orf131	Q8NDD1	VAR_031906	p.Leu28Val	LB/B	rs2274067	-
C1orf141	Q5JVX7	VAR_033186	p.Glu85Gly	LB/B	rs2273682	-
C1orf141	Q5JVX7	VAR_033187	p.Val189Ile	LB/B	rs11208997	-
C1orf147	Q96MC9	VAR_027074	p.Gly213Ala	LB/B	rs2336940	-
C1orf147	Q96MC9	VAR_027075	p.Pro233Ser	LB/B	rs11118256	-
C1orf147	Q96MC9	VAR_027076	p.Thr259Met	LB/B	rs17024805	-
C1orf158	Q8N1D5	VAR_027091	p.Phe191Leu	LB/B	rs1132185	-
C1orf162	Q8NEQ5	VAR_035106	p.Gly3Ser	LB/B	rs6703267	-
C1orf167	Q5SNV9	VAR_035266	p.Pro453Ser	LB/B	rs6668699	-
C1orf167	Q5SNV9	VAR_035267	p.Arg544Gln	LB/B	rs4845880	-
C1orf167	Q5SNV9	VAR_035268	p.Arg602Trp	LB/B	rs6667720	-
C1orf167	Q5SNV9	VAR_035269	p.Trp782Arg	LB/B	rs6699881	-
C1orf167	Q5SNV9	VAR_035270	p.Arg800Cys	LB/B	rs7538516	-
C1orf167	Q5SNV9	VAR_035271	p.Ser848Ile	LB/B	rs6697244	-
C1orf167	Q5SNV9	VAR_035272	p.Arg944His	LB/B	rs4846043	-
C1orf167	Q5SNV9	VAR_035273	p.Trp955Arg	LB/B	rs4846044	-
C1orf167	Q5SNV9	VAR_035274	p.Arg1215His	LB/B	rs3737967	-
C1orf167	Q5SNV9	VAR_035275	p.Cys1295Ser	LB/B	rs1537514	-
C1orf167	Q5SNV9	VAR_035276	p.Arg1451Gly	LB/B	rs868014	-
C1orf174	Q8IYL3	VAR_033152	p.Thr53Arg	LB/B	rs4274008	-
C1orf174	Q8IYL3	VAR_033153	p.Ala101Ser	LB/B	rs10909820	-
C1orf174	Q8IYL3	VAR_057829	p.Pro165Ala	LB/B	rs12036962	-
C1orf194	Q5T5A4	VAR_082033	p.Lys28Ile	LB/B	rs1553246861	-
C1orf194	Q5T5A4	VAR_082034	p.Ile122Asn	LP/P	rs1553246716	-
C1orf198	Q9H425	VAR_050707	p.Ala274Ser	LB/B	rs34864456	-
C1orf198	Q9H425	VAR_050708	p.Lys306Arg	LB/B	rs35115679	-
C1orf210	Q8IVY1	VAR_033655	p.Ser12Leu	LB/B	rs35465732	-
C1orf220	Q5T0J3	VAR_056768	p.Pro3Leu	LB/B	rs12568310	-
C1orf50	Q9BV19	VAR_054409	p.Thr178Met	LB/B	rs11548275	-
C1orf74	Q96LT6	VAR_050701	p.Leu146Phe	LB/B	rs7550857	-
C1orf87	Q8N0U7	VAR_031745	p.Leu185Val	LB/B	rs12737449	-
C1orf87	Q8N0U7	VAR_031746	p.Asn301Asp	LB/B	rs17120025	-
C1orf87	Q8N0U7	VAR_031747	p.Lys403Glu	LB/B	rs626251	-
C1orf87	Q8N0U7	VAR_031748	p.Ala406Pro	LB/B	rs35260089	-
C1orf87	Q8N0U7	VAR_035493	p.Gln151Glu	US	-	A breast cancer sample
C1orf94	Q6P1W5	VAR_031051	p.Gln235Glu	LB/B	rs1382602	-
C1orf94	Q6P1W5	VAR_031052	p.Asp302Glu	LB/B	rs1414474	-
C1orf94	Q6P1W5	VAR_050702	p.Tyr438His	LB/B	rs17556981	-
C1QA	P02745	VAR_021090	p.Glu23Lys	LB/B	rs17887074	-

C1QB P02746 VAR_008541 p.Gly42Asp LP/P - Complement component C1q deficiency (C1QD) [MIM:613652]
C1QB P02746 VAR_035551 p.Ala123Thr US rs776292843 A breast cancer sample
C1QBP Q07021 VAR_080391 p.Cys186Ser US rs748497469 Combined oxidative phosphorylation deficiency 33 (COXPD33) [MIM:617713]
C1QBP Q07021 VAR_080393 p.Phe204Leu US rs767427194 Combined oxidative phosphorylation deficiency 33 (COXPD33) [MIM:617713]
C1QBP Q07021 VAR_080394 p.Gly247Trp LP/P rs1394499137 Combined oxidative phosphorylation deficiency 33 (COXPD33) [MIM:617713]
C1QBP Q07021 VAR_080395 p.Leu275Phe US rs1555532484 Combined oxidative phosphorylation deficiency 33 (COXPD33) [MIM:617713]
C1QBP Q07021 VAR_080396 p.Leu275Pro LP/P rs1555532483 Combined oxidative phosphorylation deficiency 33 (COXPD33) [MIM:617713]
C1QC P02747 VAR_008542 p.Gly34Arg LP/P rs200206736 Complement component C1q deficiency (C1QD) [MIM:613652]
C1QTNF1 Q9BXJ1 VAR_068885 p.Arg241Gln LB/B rs35035542 -
C1QTNF12 Q5T7M4 VAR_054065 p.Gly14Arg LB/B rs7539412 -
C1QTNF4 Q9BXJ3 VAR_081086 p.His198Gln US - -
C1QTNF4 Q9BXJ3 VAR_081087 p.Gly301Asp US rs1418830230 -
C1QTNF5 Q9BXJ0 VAR_032628 p.Gln44Arg LB/B rs11538245 -
C1QTNF5 Q9BXJ0 VAR_032629 p.Ser163Arg LP/P rs111033578 Late-onset retinal degeneration (LORD) [MIM:605670]
C1QTNF6 Q9BXI9 VAR_046624 p.Gly21Val LB/B rs229527 -
C1QTNF6 Q9BXI9 VAR_046625 p.Pro42Arg LB/B rs229526 -
C1QTNF6 Q9BXI9 VAR_046626 p.Gly55Asp LB/B rs7290488 -
C1QTNF6 Q9BXI9 VAR_046627 p.Pro138Leu LB/B rs17812699 -
C1QTNF6 Q9BXI9 VAR_046628 p.Arg226His LB/B rs17812681 -
C1QTNF9 P0C862 VAR_032840 p.Met219Val LB/B rs3751357 -
C1QTNF9 P0C862 VAR_032841 p.Val301Met LB/B rs4589405 -
C1QTNF9 P0C862 VAR_059148 p.Leu6Phe LB/B rs1974332 -
C1R P00736 VAR_016103 p.Ser152Leu LB/B rs1801046 -
C1R P00736 VAR_018667 p.Tyr131His LB/B rs1278295523 -
C1R P00736 VAR_018668 p.His163Tyr LB/B rs144141261 -
C1R P00736 VAR_018669 p.Glu184Lys LB/B rs1126605 -
C1R P00736 VAR_018670 p.Gly261Arg LB/B rs3813728 -
C1R P00736 VAR_047933 p.Thr186Arg LB/B rs4519167 -
C1R P00736 VAR_077106 p.Val50Asp LP/P - Ehlers-Danlos syndrome, periodontal type, 1 (EDSPD1) [MIM:130080]
C1R P00736 VAR_077107 p.Asp290Gly US rs1057518643 Ehlers-Danlos syndrome, periodontal type, 1 (EDSPD1) [MIM:130080]
C1R P00736 VAR_077108 p.Gly297Asp US rs1057519026 Ehlers-Danlos syndrome, periodontal type, 1 (EDSPD1) [MIM:130080]
C1R P00736 VAR_077109 p.Leu300Pro US rs1057515579 Ehlers-Danlos syndrome, periodontal type, 1 (EDSPD1) [MIM:130080]
C1R P00736 VAR_077110 p.Arg301Pro US rs760277934 Ehlers-Danlos syndrome, periodontal type, 1 (EDSPD1) [MIM:130080]
C1R P00736 VAR_077111 p.Tyr302Cys LP/P rs1057519576 Ehlers-Danlos syndrome, periodontal type, 1 (EDSPD1) [MIM:130080]

C1R P00736 VAR_077113 p.Cys309Trp LP/P rs769707492 Ehlers-Danlos syndrome, periodontal type, 1 (EDSPD1) [MIM:130080]
C1R P00736 VAR_077114 p.Cys338Arg LP/P rs1057519577 Ehlers-Danlos syndrome, periodontal type, 1 (EDSPD1) [MIM:130080]
C1R P00736 VAR_077115 p.Cys358Phe LP/P rs1057518645 Ehlers-Danlos syndrome, periodontal type, 1 (EDSPD1) [MIM:130080]
C1R P00736 VAR_077116 p.Trp364Cys US rs1057519578 Ehlers-Danlos syndrome, periodontal type, 1 (EDSPD1) [MIM:130080]
C1R P00736 VAR_077117 p.Cys371Trp LP/P rs1057519579 Ehlers-Danlos syndrome, periodontal type, 1 (EDSPD1) [MIM:130080]
C1R P00736 VAR_077119 p.Trp435Arg US rs1060499554 Ehlers-Danlos syndrome, periodontal type, 1 (EDSPD1) [MIM:130080]
C1RL Q9NZP8 VAR_038852 p.Ile285Val LB/B rs3742089 -
C1S P09871 VAR_014565 p.Arg383His LB/B rs20573 -
C1S P09871 VAR_033643 p.Arg119His LB/B rs12146727 -
C1S P09871 VAR_033644 p.Val327Leu LB/B rs2239170 -
C1S P09871 VAR_077120 p.Cys294Arg LP/P rs886040975 Ehlers-Danlos syndrome, periodontal type, 2 (EDSPD2) [MIM:617174]
C2 P06681 VAR_008544 p.Cys131Tyr LP/P rs760744400 Complement component 2 deficiency (C2D) [MIM:217000]
C2 P06681 VAR_008545 p.Ser209Phe LP/P rs28934590 Complement component 2 deficiency (C2D) [MIM:217000]
C2 P06681 VAR_008546 p.Gly464Arg LP/P rs151340617 Complement component 2 deficiency (C2D) [MIM:217000]
C2 P06681 VAR_011772 p.Phe533Leu LB/B rs1042664 -
C2 P06681 VAR_019158 p.Glu318Asp LB/B rs9332739 -
C2 P06681 VAR_019159 p.Arg734Cys LB/B rs4151648 -
C20orf144 Q9BQM9 VAR_050921 p.Gln62Arg LB/B rs7260921 -
C20orf173 Q96LM9 VAR_024334 p.Lys141Glu LB/B rs7261862 -
C20orf85 Q9H1P6 VAR_033759 p.Arg26His LB/B rs16984945 -
C20orf85 Q9H1P6 VAR_033760 p.Ile99Val LB/B rs17440813 -
C20orf85 Q9H1P6 VAR_061632 p.Arg34Gln LB/B rs45576936 -
C20orf96 Q9NUD7 VAR_056847 p.Ile305Phe LB/B rs3827147 -
C22orf23 Q9BZE7 VAR_057346 p.Met136Leu LB/B rs35562630 -
C22orf31 O95567 VAR_028808 p.Cys46Arg LB/B rs9625679 -
C22orf31 O95567 VAR_028809 p.Thr210Arg LB/B rs714136 -
C22orf42 Q6IC83 VAR_045606 p.Leu73Pro LB/B rs5998267 -
C2CD2 Q9Y426 VAR_050928 p.Val211Ala LB/B rs2839421 -
C2CD2 Q9Y426 VAR_050929 p.Thr618Ala LB/B rs9981024 -
C2CD2L O14523 VAR_028797 p.Arg413Trp LB/B rs2239896 -
C2CD3 Q4AC94 VAR_037181 p.Pro773Arg LB/B rs34050666 -
C2CD3 Q4AC94 VAR_037182 p.Arg997Gln LB/B rs11235995 -
C2CD3 Q4AC94 VAR_037183 p.Arg1219Gln LB/B rs826058 -
C2CD3 Q4AC94 VAR_037184 p.Tyr1297Cys LB/B rs1095423 -
C2CD3 Q4AC94 VAR_037185 p.Ser1663Asn LB/B rs12419308 -
C2CD3 Q4AC94 VAR_037186 p.Gly1831Trp LB/B rs1632245 -
C2CD3 Q4AC94 VAR_037187 p.Arg1832Gly LB/B rs1632242 -
C2CD3 Q4AC94 VAR_071196 p.Cys1029Gly LP/P rs587777654 Orofaciodigital syndrome 14 (OFD14) [MIM:615948]

C2CD3 Q4AC94 VAR_075697 p.Gly1743Cys LB/B rs1064793399 -
C2CD4B A6NLJ0 VAR_039696 p.Phe276Val LB/B rs8040712 -
C2CD4B A6NLJ0 VAR_039697 p.Asp346Glu LB/B rs1055090 -
C2CD6 Q53TS8 VAR_024769 p.His376Gln LB/B rs10804117 -
C2CD6 Q53TS8 VAR_035787 p.Lys123Met US - A colorectal cancer sample
C2orf16 Q68DN1 VAR_031313 p.Arg357Gly LB/B rs17006143 -
C2orf16 Q68DN1 VAR_031314 p.Asp505Val LB/B rs13410886 -
C2orf16 Q68DN1 VAR_031315 p.Thr655Ser LB/B rs1919125 -
C2orf16 Q68DN1 VAR_031316 p.Ala660Glu LB/B rs1919126 -
C2orf16 Q68DN1 VAR_031317 p.Val685Ala LB/B rs1919127 -
C2orf16 Q68DN1 VAR_031318 p.Ile767Thr LB/B rs13416968 -
C2orf16 Q68DN1 VAR_031319 p.Ile774Val LB/B rs1919128 -
C2orf16 Q68DN1 VAR_031320 p.Ser1500Phe LB/B rs12618071 -
C2orf16 Q68DN1 VAR_031321 p.His1559Tyr LB/B rs13392197 -
C2orf16 Q68DN1 VAR_031322 p.Asn1613Ser LB/B rs13031957 -
C2orf16 Q68DN1 VAR_031323 p.Ser1665Pro LB/B rs28381983 -
C2orf42 Q9NWW7 VAR_050712 p.Gln314Pro LB/B rs3213941 -
C2orf48 Q96LS8 VAR_029619 p.Pro90Leu LB/B rs13406078 -
C2orf48 Q96LS8 VAR_050713 p.Trp119Ser LB/B rs7423163 -
C2orf49 Q9BVC5 VAR_029759 p.Gly185Asp LB/B rs28930676 -
C2orf73 Q8N5S3 VAR_042973 p.Pro254Leu LB/B rs2280718 -
C2orf73 Q8N5S3 VAR_042974 p.Arg275Thr LB/B rs13184 -
C2orf73 Q8N5S3 VAR_061571 p.His29Asn LB/B rs55714450 -
C2orf76 Q3KRA6 VAR_039931 p.Ile46Val LB/B rs1132267 -
C2orf76 Q3KRA6 VAR_039932 p.Lys116Arg LB/B rs1052500 -
C2orf80 Q0P641 VAR_050717 p.Arg82His LB/B rs11898181 -
C2orf80 Q0P641 VAR_050718 p.Cys130Arg LB/B rs6435421 -
C2orf80 Q0P641 VAR_050719 p.Ser152Gly LB/B rs10804166 -
C2orf83 Q53S99 VAR_039201 p.Glu104Gln LB/B rs2138402 -
C2orf83 Q53S99 VAR_061865 p.Ser45Leu LB/B rs28739019 -
C2orf88 Q9BSF0 VAR_046151 p.Thr56Ile LB/B rs6753459 -
C3 P01024 VAR_001983 p.Arg102Gly LB/B rs2230199 -
C3 P01024 VAR_001984 p.Pro314Leu LB/B rs1047286 -
C3 P01024 VAR_001985 p.Asp549Asn LP/P rs1449441916 Complement component 3 deficiency (C3D) [MIM:613779]
C3 P01024 VAR_019206 p.Arg863Lys LB/B rs11569472 -
C3 P01024 VAR_019207 p.Gly1224Asp LB/B rs11569534 -
C3 P01024 VAR_019208 p.Ile1367Thr LB/B rs11569541 -
C3 P01024 VAR_020262 p.Glu469Asp LB/B rs11569422 -
C3 P01024 VAR_029326 p.Ser1619Arg LB/B rs2230210 -
C3 P01024 VAR_029792 p.Gln1521Arg LB/B rs7256789 -
C3 P01024 VAR_029793 p.His1601Asn LB/B rs1803225 -
C3 P01024 VAR_063213 p.Arg592Gln LP/P rs121909583 Hemolytic uremic syndrome atypical 5 (AHUS5) [MIM:612925]
C3 P01024 VAR_063214 p.Arg592Trp LP/P rs771353792 Hemolytic uremic syndrome atypical 5 (AHUS5) [MIM:612925]
C3 P01024 VAR_063215 p.Arg735Trp LP/P rs117793540 Hemolytic uremic syndrome atypical 5 (AHUS5) [MIM:612925]

C3	P01024	VAR_063216	p.Ala1094Val	LP/P	rs121909584	Hemolytic uremic syndrome atypical 5 (AHUS5) [MIM:612925]
C3	P01024	VAR_063217	p.Asp1115Asn	LP/P	rs121909585	Hemolytic uremic syndrome atypical 5 (AHUS5) [MIM:612925]
C3	P01024	VAR_063218	p.Cys1158Trp	LP/P	-	Hemolytic uremic syndrome atypical 5 (AHUS5) [MIM:612925]
C3	P01024	VAR_063219	p.Gln1161Lys	LP/P	-	Hemolytic uremic syndrome atypical 5 (AHUS5) [MIM:612925]
C3	P01024	VAR_063220	p.His1464Asp	LP/P	-	Hemolytic uremic syndrome atypical 5 (AHUS5) [MIM:612925]
C3	P01024	VAR_063654	p.Phe603Val	LP/P	-	Hemolytic uremic syndrome atypical 5 (AHUS5) [MIM:612925]
C3	P01024	VAR_063655	p.Arg1042Leu	LP/P	-	Hemolytic uremic syndrome atypical 5 (AHUS5) [MIM:612925]
C3	P01024	VAR_070941	p.Lys155Gln	LP/P	rs147859257	Macular degeneration, age-related, 9 (ARMD9) [MIM:611378]
C3AR1	Q16581	VAR_019164	p.Val136Ala	LB/B	rs11567806	-
C3orf18	Q9UK00	VAR_025720	p.Ala8Asp	LB/B	rs386598	-
C3orf18	Q9UK00	VAR_025721	p.Ala162Val	LB/B	rs1034405	-
C3orf20	Q8ND61	VAR_025722	p.Ala176Thr	LB/B	rs17040196	-
C3orf20	Q8ND61	VAR_027886	p.Gly42Asp	LB/B	rs17040154	-
C3orf20	Q8ND61	VAR_027887	p.Asp65Asn	LB/B	rs9821143	-
C3orf20	Q8ND61	VAR_027888	p.Ser230Tyr	LB/B	rs17852774	-
C3orf20	Q8ND61	VAR_027889	p.Ala298Thr	LB/B	rs17040196	-
C3orf20	Q8ND61	VAR_027890	p.Ile407Val	LB/B	rs6765537	-
C3orf20	Q8ND61	VAR_027891	p.Leu422Val	LB/B	rs6790129	-
C3orf20	Q8ND61	VAR_056770	p.Gln205Arg	LB/B	rs34230332	-
C3orf20	Q8ND61	VAR_056771	p.Ala689Val	LB/B	rs34045813	-
C3orf33	Q6P1S2	VAR_028846	p.Ala47Thr	LB/B	rs9853408	-
C3orf33	Q6P1S2	VAR_028847	p.Ser160Asn	LB/B	rs358733	-
C3orf62	Q6ZUJ4	VAR_050724	p.Glu110Lys	LB/B	rs13077498	-
C3P1	Q6ZMU1	VAR_044412	p.Gln172Arg	LB/B	rs8113341	-
C3P1	Q6ZMU1	VAR_044413	p.Asp230Asn	LB/B	rs10403787	-
C3P1	Q6ZMU1	VAR_044414	p.Asp251Gly	LB/B	rs1993466	-
C3P1	Q6ZMU1	VAR_044415	p.Leu262Arg	LB/B	rs4552116	-
C4A	P0C0L4	VAR_001987	p.Arg477Trp	LB/B	-	-
C4A	P0C0L4	VAR_001988	p.Pro726Leu	LB/B	rs1215093373	-
C4A	P0C0L4	VAR_001992	p.Thr1201Ser	LB/B	-	-
C4A	P0C0L4	VAR_001993	p.Val1207Ala	LB/B	rs28357075	-
C4A	P0C0L4	VAR_001994	p.Leu1210Arg	LB/B	rs28357076	-
C4A	P0C0L4	VAR_001995	p.Ser1286Ala	LB/B	rs201016130	-
C4A	P0C0L4	VAR_019778	p.Ser347Tyr	LB/B	rs392610	-
C4A	P0C0L4	VAR_019779	p.Asp727Asn	LB/B	-	-
C4A	P0C0L4	VAR_019780	p.Ala907Thr	LB/B	rs429329	-
C4A	P0C0L4	VAR_069154	p.Leu141Val	LB/B	rs9296005	-
C4A	P0C0L4	VAR_069155	p.Val418Ala	LB/B	-	-
C4A	P0C0L4	VAR_069156	p.His549Pro	LB/B	rs2229405	-
C4A	P0C0L4	VAR_069158	p.Asp1073Gly	LB/B	rs147162052	-
C4A	P0C0L4	VAR_069159	p.Asn1176Ser	LB/B	rs17874654	-

C4B	P0C0L5	VAR_023729	p.Ser347Tyr	LB/B	rs139889867	-
C4B	P0C0L5	VAR_023730	p.Thr907Ala	LB/B	rs796750528	-
C4B	P0C0L5	VAR_023731	p.Gly1073Asp	LB/B	rs2258218	-
C4B	P0C0L5	VAR_023732	p.Ser1176Asn	LB/B	rs2746414	-
C4B	P0C0L5	VAR_023734	p.Ala1207Val	LB/B	rs2229403	-
C4B	P0C0L5	VAR_023735	p.Arg1210Leu	LB/B	rs2229409	-
C4B	P0C0L5	VAR_069160	p.Pro478Leu	LB/B	-	-
C4B	P0C0L5	VAR_069161	p.Ile1317Phe	LB/B	rs2023616	-
C4BPA	P04003	VAR_001978	p.Tyr357His	LB/B	-	-
C4BPA	P04003	VAR_012038	p.Trp473Leu	LB/B	rs1801341	-
C4BPA	P04003	VAR_024420	p.Ile300Thr	LB/B	rs4844573	-
C4BPA	P04003	VAR_048815	p.Ala60Val	LB/B	rs17020956	-
C4BPA	P04003	VAR_061123	p.Pro4Gln	LB/B	rs55867570	-
C4BPA	P04003	VAR_061124	p.Arg240His	LB/B	rs45574833	-
C4BPB	P20851	VAR_012039	p.Pro198Ser	LB/B	rs1803226	-
C4BPB	P20851	VAR_038734	p.Lys102Gln	LB/B	rs56258224	-
C4orf17	Q53FE4	VAR_028110	p.Gly64Glu	LB/B	rs13143848	-
C4orf17	Q53FE4	VAR_028111	p.Ser85Pro	LB/B	rs13119384	-
C4orf17	Q53FE4	VAR_028112	p.Glu91Lys	LB/B	rs17029087	-
C4orf19	Q8IY42	VAR_032118	p.Ala151Thr	LB/B	rs2973275	-
C4orf19	Q8IY42	VAR_032119	p.Glu274Gly	LB/B	rs3733500	-
C4orf19	Q8IY42	VAR_050770	p.Asp39Glu	LB/B	rs6852908	-
C4orf3	Q8WVX3	VAR_039911	p.Arg17Gln	LB/B	rs17851522	-
C4orf3	Q8WVX3	VAR_039912	p.Pro32His	LB/B	rs11544530	-
C4orf33	Q8N1A6	VAR_033334	p.Arg40Met	LB/B	rs35199409	-
C4orf33	Q8N1A6	VAR_033335	p.Ser104Leu	LB/B	rs2271570	-
C4orf33	Q8N1A6	VAR_033336	p.Met107Val	LB/B	rs337277	-
C4orf33	Q8N1A6	VAR_033337	p.His166Arg	LB/B	rs17351999	-
C4orf36	Q96KX1	VAR_029755	p.Ser36Tyr	LB/B	rs1550931	-
C4orf36	Q96KX1	VAR_056784	p.Ser18Asn	LB/B	rs11938345	-
C4orf45	Q96LM5	VAR_039901	p.Ala46Val	LB/B	rs17037864	-
C4orf45	Q96LM5	VAR_039902	p.Ser75Gly	LB/B	rs662473	-
C4orf45	Q96LM5	VAR_039903	p.Tyr84Cys	LB/B	rs17037858	-
C4orf45	Q96LM5	VAR_039904	p.Gly93Glu	LB/B	rs619128	-
C4orf45	Q96LM5	VAR_039905	p.Arg134His	LB/B	rs10517695	-
C4orf50	Q6ZRC1	VAR_042680	p.Arg86Trp	LB/B	rs16837960	-
C4orf50	Q6ZRC1	VAR_042681	p.Ala150Pro	US	-	A breast cancer sample
C4orf50	Q6ZRC1	VAR_042682	p.Ile177Val	LB/B	rs6839295	-
C4orf50	Q6ZRC1	VAR_042683	p.Val199Met	LB/B	rs7695618	-
C4orf54	D6RIA3	VAR_080344	p.Gly595Arg	US	rs1034287647	-
C4orf54	D6RIA3	VAR_080345	p.Phe1415Ser	US	rs555103041	-
C4orf54	D6RIA3	VAR_080346	p.Thr1632Ile	US	-	-
C5	P01031	VAR_001996	p.Phe518Ser	LB/B	-	-
C5	P01031	VAR_014574	p.Val802Ile	LB/B	rs17611	-
C5	P01031	VAR_014575	p.Met1053Leu	LB/B	rs17609	-
C5	P01031	VAR_014576	p.Ser1310Asn	LB/B	rs17610	-
C5	P01031	VAR_014577	p.Glu1437Asp	LB/B	rs17612	-
C5	P01031	VAR_023946	p.Thr389Ile	LB/B	-	-
C5	P01031	VAR_038735	p.Val145Ile	LB/B	rs17216529	-

C5	P01031	VAR_038736	p.Arg449Gly	LB/B	rs2230213	-
C5	P01031	VAR_038737	p.Arg928Gln	LB/B	rs41309892	-
C5	P01031	VAR_038738	p.Gly933Val	LB/B	rs41309902	-
C5	P01031	VAR_038739	p.Ile1033Thr	LB/B	rs41311881	-
C5	P01031	VAR_038740	p.Asp1037Asn	LB/B	rs41311883	-
C5	P01031	VAR_038741	p.Gln1043Lys	LB/B	rs41311887	-
C5	P01031	VAR_048822	p.Leu354Met	LB/B	rs34552775	-
C5	P01031	VAR_048823	p.Asp966Tyr	LB/B	rs2230212	-
C5	P01031	VAR_048824	p.Val1365Ala	LB/B	rs16910245	-
C5	P01031	VAR_071067	p.Arg885Cys	LB/B	rs373359894	-
C5	P01031	VAR_071068	p.Arg885His	LB/B	rs56040400	-
C5AR1	P21730	VAR_049377	p.Asp2Asn	LB/B	rs4467185	-
C5AR1	P21730	VAR_049378	p.Lys279Asn	LB/B	rs11880097	-
C5AR2	Q9P296	VAR_068748	p.Pro233Leu	LB/B	rs149572881	-
C5AR2	Q9P296	VAR_068749	p.Ser323Ile	US	rs150599989	-
C5orf22	Q49AR2	VAR_035149	p.Thr235Pro	LB/B	rs17410000	-
C5orf22	Q49AR2	VAR_035150	p.Asp405Glu	LB/B	rs16901277	-
C5orf34	Q96MH7	VAR_035618	p.Ser266Arg	US	-	A breast cancer sample
C5orf34	Q96MH7	VAR_061589	p.Thr32Ser	LB/B	rs6872851	-
C5orf46	Q6UWT4	VAR_056787	p.Phe18Ile	LB/B	rs7722926	-
C5orf49	A4QMS7	VAR_043546	p.Gln68His	LB/B	rs6883562	-
C5orf49	A4QMS7	VAR_043547	p.Glu87Lys	LB/B	rs16879215	-
C5orf49	A4QMS7	VAR_043548	p.Pro139Ser	LB/B	rs326181	-
C5orf51	A6NDU8	VAR_044032	p.Gln20His	LB/B	rs12520325	-
C5orf60	A6NFR6	VAR_044323	p.Arg55Cys	LB/B	rs1319931	-
C5orf60	A6NFR6	VAR_044324	p.Leu56Pro	LB/B	rs13168357	-
C5orf64	Q2M2E5	VAR_043940	p.Ala57Thr	LB/B	rs16893687	-
C5orf64	Q2M2E5	VAR_043941	p.Arg101Trp	LB/B	rs436696	-
C6	P13671	VAR_006056	p.Ala119Glu	LB/B	rs1801033	-
C6	P13671	VAR_027647	p.Lys397Glu	LB/B	rs6896011	-
C6	P13671	VAR_027648	p.Ser470Phe	LB/B	rs10462014	-
C6orf118	Q5T5N4	VAR_022887	p.Ile256Met	LB/B	rs510579	-
C6orf118	Q5T5N4	VAR_022888	p.Val363Leu	LB/B	rs9459350	-
C6orf118	Q5T5N4	VAR_050807	p.Arg166Leu	LB/B	rs36007498	-
C6orf118	Q5T5N4	VAR_050808	p.Gly271Glu	LB/B	rs17852379	-
C6orf118	Q5T5N4	VAR_050809	p.Thr301Met	LB/B	rs540751	-
C6orf118	Q5T5N4	VAR_050810	p.Arg385Gln	LB/B	rs17856754	-
C6orf132	Q5T0Z8	VAR_056799	p.Lys4Asn	LB/B	rs9688934	-
C6orf141	Q5SZD1	VAR_022939	p.Gln137Glu	LB/B	rs6919674	-
C6orf141	Q5SZD1	VAR_030643	p.Pro235Leu	LB/B	rs9473588	-
C6orf15	Q6UXA7	VAR_022907	p.Leu40Phe	LB/B	rs2233974	-
C6orf15	Q6UXA7	VAR_022908	p.Asn43Asp	LB/B	rs2233975	-
C6orf15	Q6UXA7	VAR_022909	p.Ala83Pro	LB/B	rs1265053	-
C6orf15	Q6UXA7	VAR_022910	p.Lys165Glu	LB/B	rs1265054	-
C6orf15	Q6UXA7	VAR_028732	p.Val81Ala	LB/B	rs2233977	-
C6orf15	Q6UXA7	VAR_028733	p.Ala145Pro	LB/B	rs2233978	-
C6orf15	Q6UXA7	VAR_028734	p.Gly291Asp	LB/B	rs2233984	-
C6orf15	Q6UXA7	VAR_050801	p.Gly48Arg	LB/B	rs2233976	-
C6orf15	Q6UXA7	VAR_050802	p.Met232Ile	LB/B	rs2233982	-

C6orf15	Q6UXA7	VAR_054399	p.Val5Met	LB/B	rs2270191	-
C6orf201	Q7Z4U5	VAR_056805	p.Arg20Pro	LB/B	rs619483	-
C6orf201	Q7Z4U5	VAR_056806	p.Asp25Tyr	LB/B	rs9503910	-
C6orf201	Q7Z4U5	VAR_064769	p.Asn117Lys	LB/B	rs643232	-
C6orf223	Q8N319	VAR_050816	p.Lys48Glu	LB/B	rs2295333	-
C6orf223	Q8N319	VAR_061592	p.Val107Met	LB/B	rs60336135	-
C6orf47	O95873	VAR_022911	p.Gly68Arg	LB/B	rs3130617	-
C6orf47	O95873	VAR_056797	p.Lys92Asn	LB/B	rs2242655	-
C6orf52	Q5T4I8	VAR_033318	p.Ala13Asp	LB/B	rs7749306	-
C6orf62	Q9GZU0	VAR_050803	p.Trp116Cys	LB/B	rs34238213	-
C6orf62	Q9GZU0	VAR_050804	p.Arg140Ser	LB/B	rs35050510	-
C7	P10643	VAR_012643	p.Arg220Gln	LP/P	rs369349760	Complement component 7 deficiency (C7D) [MIM:610102]
C7	P10643	VAR_012644	p.Gly379Arg	LP/P	rs121964921	Complement component 7 deficiency (C7D) [MIM:610102]
C7	P10643	VAR_012645	p.Arg521Ser	LP/P	rs121964920	Complement component 7 deficiency (C7D) [MIM:610102]
C7	P10643	VAR_012646	p.Glu682Gln	LP/P	rs541873000	Complement component 7 deficiency (C7D) [MIM:610102]
C7	P10643	VAR_012647	p.Arg687His	LP/P	rs113187203	Complement component 7 deficiency (C7D) [MIM:610102]
C7	P10643	VAR_022023	p.Lys420Gln	LB/B	rs3792646	-
C7	P10643	VAR_033798	p.Ser389Thr	LB/B	rs1063499	-
C7	P10643	VAR_033799	p.Thr587Pro	LB/B	rs13157656	-
C7	P10643	VAR_050480	p.Cys128Arg	LB/B	rs2271708	-
C7	P10643	VAR_081726	p.Arg222His	LB/B	rs75345202	-
C7orf25	Q9BPX7	VAR_053848	p.Gly265Glu	LB/B	rs3735471	-
C7orf26	Q96N11	VAR_061593	p.Arg245Cys	LB/B	rs35534502	-
C7orf31	Q8N865	VAR_022781	p.Tyr82Cys	LB/B	rs2717858	-
C7orf31	Q8N865	VAR_022782	p.Ala158Thr	LB/B	rs12535348	-
C7orf31	Q8N865	VAR_022783	p.Thr187Ser	LB/B	rs2523072	-
C7orf31	Q8N865	VAR_022784	p.His300Arg	LB/B	rs2285738	-
C7orf31	Q8N865	VAR_056809	p.Glu13Lys	LB/B	rs11980216	-
C7orf31	Q8N865	VAR_056810	p.Arg86Cys	LB/B	rs3213642	-
C7orf31	Q8N865	VAR_056811	p.Pro238Gln	LB/B	rs11984293	-
C7orf31	Q8N865	VAR_056812	p.Ala249Thr	LB/B	rs17150982	-
C7orf31	Q8N865	VAR_060608	p.Pro143Gln	LB/B	rs17855102	-
C7orf31	Q8N865	VAR_060609	p.Asn518Asp	LB/B	rs17857512	-
C7orf57	Q8NEG2	VAR_056813	p.Ala74Ser	LB/B	rs10951942	-
C8A	P07357	VAR_011889	p.Gln93Lys	LB/B	rs652785	-
C8A	P07357	VAR_011890	p.Thr407Ile	LB/B	rs706479	-
C8A	P07357	VAR_011891	p.Arg485Leu	LB/B	rs1620075	-
C8A	P07357	VAR_011892	p.Glu561Gln	LB/B	rs1342440	-
C8A	P07357	VAR_033800	p.Asp458Asn	LB/B	rs17114555	-
C8A	P07357	VAR_033801	p.Pro575Leu	LB/B	rs17300936	-
C8B	P07358	VAR_012642	p.Arg117Gly	LB/B	rs1013579	-
C8B	P07358	VAR_027649	p.Glu108Lys	LB/B	rs12067507	-
C8B	P07358	VAR_027650	p.Pro261Leu	LB/B	rs12085435	-
C8G	P07360	VAR_014668	p.Arg69Gln	LB/B	rs17614	-

C8G	P07360	VAR_014669	p.His124Asn	LB/B	rs17613	-
C8G	P07360	VAR_044319	p.Asp118Gly	LB/B	rs7850844	-
C8orf17	Q9NRJ1	VAR_042878	p.Thr19Met	LB/B	rs2233233	-
C8orf17	Q9NRJ1	VAR_042879	p.Lys49Arg	LB/B	rs2233235	-
C8orf17	Q9NRJ1	VAR_042880	p.Thr57Ile	LB/B	rs2233236	-
C8orf34	Q49A92	VAR_042690	p.Lys161Asn	US	-	A colorectal cancer sample
C8orf34	Q49A92	VAR_042691	p.Ala512Thr	LB/B	rs16935065	-
C8orf37	Q96NL8	VAR_033683	p.Pro19Ala	LB/B	rs36096184	-
C8orf37	Q96NL8	VAR_067305	p.Arg177Trp	LP/P	rs387907136	Bardet-Biedl syndrome 21 (BBS21) [MIM:617406]
C8orf37	Q96NL8	VAR_067305	p.Arg177Trp	LP/P	rs387907136	Cone-rod dystrophy 16 (CORD16) [MIM:614500]
C8orf37	Q96NL8	VAR_067306	p.Gln182Arg	LP/P	rs387907137	Retinitis pigmentosa 64 (RP64) [MIM:614500]
C8orf44	Q96CB5	VAR_030888	p.Gln131Arg	LB/B	rs1909534	-
C8orf44	Q96CB5	VAR_030889	p.Phe148Ser	LB/B	rs1057463	-
C8orf48	Q96LL4	VAR_063134	p.Ser28Phe	LB/B	rs13273355	-
C8orf48	Q96LL4	VAR_063135	p.Leu285Met	LB/B	rs11203497	-
C8orf74	Q6P047	VAR_033684	p.Leu15Phe	LB/B	rs11250058	-
C8orf74	Q6P047	VAR_061596	p.Ile222Val	LB/B	rs57041981	-
C8orf86	Q6ZUL3	VAR_046186	p.Arg81His	LB/B	rs1378332	-
C9	P02748	VAR_012648	p.Cys119Gly	LP/P	rs121909593	Complement component 9 deficiency (C9D) [MIM:613825]
C9	P02748	VAR_022024	p.Arg5Trp	LB/B	rs700233	-
C9	P02748	VAR_027651	p.Ile203Val	LB/B	rs13361416	-
C9	P02748	VAR_033802	p.Thr279Ser	LB/B	rs34625111	-
C9	P02748	VAR_050481	p.Asp127Tyr	LB/B	rs696763	-
C9	P02748	VAR_061503	p.Ser427Thr	LB/B	rs34421659	-
C9	P02748	VAR_070940	p.Pro167Ser	LP/P	rs34882957	Macular degeneration, age-related, 15 (ARMD15) [MIM:615591]
C9orf129	Q5T035	VAR_061600	p.Arg191His	LB/B	rs36081907	-
C9orf131	Q5VYM1	VAR_047239	p.Trp222Leu	LB/B	rs615474	-
C9orf131	Q5VYM1	VAR_047240	p.Leu285Phe	LB/B	rs10117097	-
C9orf131	Q5VYM1	VAR_047241	p.Leu437Val	LB/B	rs35523761	-
C9orf131	Q5VYM1	VAR_047242	p.Ser623Thr	LB/B	rs2298312	-
C9orf131	Q5VYM1	VAR_047243	p.Pro916Ser	LB/B	rs3739871	-
C9orf163	Q8N9P6	VAR_032526	p.Leu5Pro	LB/B	rs34376913	-
C9orf43	Q8TAL5	VAR_061597	p.Asp252Gly	LB/B	rs41313331	-
C9orf50	Q5SZB4	VAR_033220	p.Arg248Lys	LB/B	rs918165	-
C9orf50	Q5SZB4	VAR_033221	p.Arg312Gln	LB/B	rs3213763	-
C9orf50	Q5SZB4	VAR_033222	p.Arg381Gln	LB/B	rs2302779	-
C9orf50	Q5SZB4	VAR_033223	p.Lys415Arg	LB/B	rs3087721	-
C9orf72	Q96LT7	VAR_050827	p.Asn207Ser	LB/B	rs17769294	-
C9orf78	Q9NZ63	VAR_050828	p.Gln70His	LB/B	rs1237745	-
CA1	P00915	VAR_001378	p.His68Arg	LB/B	rs990757234	-
CA1	P00915	VAR_001379	p.Gly254Arg	LB/B	rs121909577	-
CA1	P00915	VAR_048679	p.Ala143Val	LB/B	rs7821248	-
CA12	O43570	VAR_065292	p.Glu143Lys	LP/P	rs267606694	Hyperchlorhidrosis, isolated (HYCHL) [MIM:143860]
CA12	O43570	VAR_081182	p.His121Gln	LP/P	rs775067652	Hyperchlorhidrosis, isolated (HYCHL) [MIM:143860]

CA13 Q8N1Q1 VAR_059207 p.Asn68Ser LB/B rs4740046 -
CA2 P00918 VAR_001380 p.Lys18Glu LB/B rs118203931 -
CA2 P00918 VAR_001381 p.Gln92Pro LP/P rs1304160279 Osteopetrosis, autosomal recessive 3 (OPTB3) [MIM:259730]
CA2 P00918 VAR_001382 p.His107Tyr LP/P rs118203933 Osteopetrosis, autosomal recessive 3 (OPTB3) [MIM:259730]
CA2 P00918 VAR_001383 p.Pro236His LB/B rs118203932 -
CA2 P00918 VAR_001384 p.Asn252Asp LB/B rs2228063 -
CA2 P00918 VAR_021009 p.His94Tyr LP/P - Osteopetrosis, autosomal recessive 3 (OPTB3) [MIM:259730]
CA2 P00918 VAR_021010 p.Gly144Arg LP/P - Osteopetrosis, autosomal recessive 3 (OPTB3) [MIM:259730]
CA3 P07451 VAR_016180 p.Val31Ile LB/B rs20571 -
CA4 P22748 VAR_024749 p.Arg14Trp LP/P rs104894559 Retinitis pigmentosa 17 (RP17) [MIM:600852]
CA4 P22748 VAR_024750 p.Arg219Ser LP/P rs121434551 Retinitis pigmentosa 17 (RP17) [MIM:600852]
CA4 P22748 VAR_048680 p.Val237Leu LB/B rs2229178 -
CA4 P22748 VAR_071430 p.Ala12Thr LP/P rs1245199379 Retinitis pigmentosa 17 (RP17) [MIM:600852]
CA4 P22748 VAR_071431 p.Arg69His LP/P rs121434552 Retinitis pigmentosa 17 (RP17) [MIM:600852]
CA4 P22748 VAR_071432 p.Asn177Lys LB/B rs185942554 -
CA5A P35218 VAR_071188 p.Ser233Pro LP/P rs587777316 Hyperammonemia due to carbonic anhydrase VA deficiency (CA5AD) [MIM:615751]
CA6 P23280 VAR_028268 p.Thr55Met LB/B rs2274327 -
CA6 P23280 VAR_028269 p.Met68Leu LB/B rs2274328 -
CA6 P23280 VAR_028270 p.Gly70Ala LB/B rs2274329 -
CA6 P23280 VAR_028271 p.Ser90Gly LB/B rs2274333 -
CA6 P23280 VAR_033712 p.Gln37Leu LB/B rs34265054 -
CA6 P23280 VAR_061093 p.Arg58Trp LB/B rs58800854 -
CA8 P35219 VAR_063634 p.Ser100Pro LP/P rs267606695 Cerebellar ataxia, mental retardation, and dysequilibrium syndrome 3 (CMARQ3) [MIM:613227]
CA9 Q16790 VAR_010787 p.Val33Met LB/B rs2071676 -
CA9 Q16790 VAR_020049 p.Gln326Arg LB/B rs3829078 -
CAAP1 Q9H8G2 VAR_056818 p.Val233Met LB/B rs12342214 -
CABIN1 Q9Y6J0 VAR_052607 p.Ala56Thr LB/B rs5760185 -
CABIN1 Q9Y6J0 VAR_052608 p.Asp225Asn LB/B rs17004823 -
CABIN1 Q9Y6J0 VAR_052609 p.Ser517Arg LB/B rs9624393 -
CABIN1 Q9Y6J0 VAR_052610 p.Arg660Ser LB/B rs9624395 -
CABIN1 Q9Y6J0 VAR_052611 p.Arg853Gln LB/B rs17854874 -
CABIN1 Q9Y6J0 VAR_052612 p.Gln921Glu LB/B rs12166151 -
CABLES2 Q9BTV7 VAR_026532 p.Thr428Lys LB/B rs6089219 -
CABP2 Q9NPB3 VAR_063087 p.Arg94Gln LB/B rs2276118 -
CABP4 P57796 VAR_029375 p.Arg124Cys LP/P rs121917828 Cone-rod synaptic disorder, congenital non-progressive (CRSD) [MIM:610427]
CABP5 Q9NP86 VAR_020020 p.Val128Ala LB/B rs3745746 -
CABP5 Q9NP86 VAR_033695 p.Thr65Arg LB/B rs34862923 -
CABP5 Q9NP86 VAR_048633 p.Leu80Pro LB/B rs8105198 -
CABP5 Q9NP86 VAR_048634 p.Glu140Lys LB/B rs34681062 -
CABP5 Q9NP86 VAR_048635 p.Ile147Ser LB/B rs10425606 -
CABS1 Q96KC9 VAR_043924 p.Lys76Gln LB/B rs3796706 -
CABS1 Q96KC9 VAR_043925 p.Thr211Ile LB/B rs1351419 -

CABS1 Q96KC9 VAR_043926 p.Trp298Gly LB/B rs2291182 -
CABYR O75952 VAR_023818 p.Ser490Ala LB/B rs1049683 -
CABYR O75952 VAR_030040 p.Thr74Met LB/B rs3786417 -
CABYR O75952 VAR_030041 p.Lys448Arg LB/B rs1049682 -
CABYR O75952 VAR_050709 p.Ile186Val LB/B rs35118855 -
CACFD1 Q9UGQ2 VAR_069103 p.Ile58Met LB/B rs3124765 -
CACHD1 Q5VU97 VAR_035052 p.Met414Thr LB/B rs6588100 -
CACNA1A O00555 VAR_001491 p.Arg192Gln LP/P rs121908211 Migraine, familial hemiplegic, 1 (FHM1) [MIM:141500]
CACNA1A O00555 VAR_001492 p.Thr665Met LP/P rs121908212 Episodic ataxia 2 (EA2) [MIM:108500]
CACNA1A O00555 VAR_001492 p.Thr665Met LP/P rs121908212 Migraine, familial hemiplegic, 1 (FHM1) [MIM:141500]
CACNA1A O00555 VAR_001493 p.Val713Ala LP/P rs121908213 Migraine, familial hemiplegic, 1 (FHM1) [MIM:141500]
CACNA1A O00555 VAR_001494 p.Ile1809Leu LP/P rs121908214 Migraine, familial hemiplegic, 1 (FHM1) [MIM:141500]
CACNA1A O00555 VAR_014456 p.Ala21Val LB/B rs15999 -
CACNA1A O00555 VAR_014458 p.Pro913Ser LB/B rs16020 -
CACNA1A O00555 VAR_014459 p.Glu917Asp LB/B rs16022 -
CACNA1A O00555 VAR_014461 p.Glu1014Lys LB/B rs16024 -
CACNA1A O00555 VAR_014462 p.Gly1104Ser LB/B rs16027 -
CACNA1A O00555 VAR_014463 p.Pro2395Ser LB/B rs16056 -
CACNA1A O00555 VAR_043820 p.Arg195Lys LP/P rs121908222 Migraine, familial hemiplegic, 1 (FHM1) [MIM:141500]
CACNA1A O00555 VAR_043821 p.Ser218Leu LP/P rs121908225 Migraine, familial hemiplegic, 1 (FHM1) [MIM:141500]
CACNA1A O00555 VAR_043822 p.His253Tyr LP/P rs121908228 Episodic ataxia 2 (EA2) [MIM:108500]
CACNA1A O00555 VAR_043823 p.Cys256Arg LP/P rs121908231 Episodic ataxia 2 (EA2) [MIM:108500]
CACNA1A O00555 VAR_043824 p.Cys287Tyr LP/P rs121908236 Episodic ataxia 2 (EA2) [MIM:108500]
CACNA1A O00555 VAR_043825 p.Gly293Arg LP/P rs121908215 Episodic ataxia 2 (EA2) [MIM:108500]
CACNA1A O00555 VAR_043825 p.Gly293Arg LP/P rs121908215 Spinocerebellar ataxia 6 (SCA6) [MIM:183086]
CACNA1A O00555 VAR_043826 p.Arg582Gln LP/P rs121908217 Migraine, familial hemiplegic, 1 (FHM1) [MIM:141500]
CACNA1A O00555 VAR_043826 p.Arg582Gln LP/P rs121908217 Spinocerebellar ataxia 6 (SCA6) [MIM:183086]
CACNA1A O00555 VAR_043827 p.Asp714Glu LP/P rs121908218 Migraine, familial hemiplegic, 1 (FHM1) [MIM:141500]
CACNA1A O00555 VAR_043828 p.Glu992Val LB/B rs16023 -
CACNA1A O00555 VAR_043829 p.Lys1334Glu LP/P rs121908223 Migraine, familial hemiplegic, 1 (FHM1) [MIM:141500]
CACNA1A O00555 VAR_043830 p.Arg1345Gln LP/P rs121908230 Migraine, familial hemiplegic, 1 (FHM1) [MIM:141500]
CACNA1A O00555 VAR_043831 p.Tyr1383Cys LP/P rs121908219 Migraine, familial hemiplegic, 1 (FHM1) [MIM:141500]
CACNA1A O00555 VAR_043832 p.Phe1402Cys LP/P rs121908227 Episodic ataxia 2 (EA2) [MIM:108500]
CACNA1A O00555 VAR_043833 p.Val1455Leu LP/P rs121908237 Migraine, familial hemiplegic, 1 (FHM1) [MIM:141500]
CACNA1A O00555 VAR_043834 p.Gly1481Arg LP/P rs121908232 Episodic ataxia 2 (EA2) [MIM:108500]
CACNA1A O00555 VAR_043835 p.Phe1489Ser LP/P rs121908233 Episodic ataxia 2 (EA2) [MIM:108500]
CACNA1A O00555 VAR_043836 p.Val1492Ile LP/P rs121908234 Episodic ataxia 2 (EA2) [MIM:108500]

CACNA1A O00555 VAR_043837 p.Arg1660His LP/P rs121908216 Episodic ataxia 2 (EA2) [MIM:108500]
CACNA1A O00555 VAR_043838 p.Arg1666Trp LP/P rs121908220 Migraine, familial hemiplegic, 1 (FHM1) [MIM:141500]
CACNA1A O00555 VAR_043839 p.Trp1682Arg LP/P rs121908221 Migraine, familial hemiplegic, 1 (FHM1) [MIM:141500]
CACNA1A O00555 VAR_043840 p.His1735Leu LP/P rs121908229 Episodic ataxia 2 (EA2) [MIM:108500]
CACNA1A O00555 VAR_043841 p.Glu1755Lys LP/P rs121908226 Episodic ataxia 2 (EA2) [MIM:108500]
CACNA1A O00555 VAR_043842 p.Arg2134Cys LP/P rs121908235 Episodic ataxia 2 (EA2) [MIM:108500]
CACNA1A O00555 VAR_059221 p.Glu731Ala LB/B rs16019 -
CACNA1A O00555 VAR_059222 p.Pro1172Leu LB/B rs16028 -
CACNA1A O00555 VAR_063683 p.Tyr248Cys LP/P rs121908238 Episodic ataxia 2 (EA2) [MIM:108500]
CACNA1A O00555 VAR_063684 p.Leu389Phe LP/P rs121908239 Episodic ataxia 2 (EA2) [MIM:108500]
CACNA1A O00555 VAR_063685 p.Ala405Thr LP/P rs121908245 Spinocerebellar ataxia 6 (SCA6) [MIM:183086]
CACNA1A O00555 VAR_063686 p.Ala453Thr LB/B rs41276886 -
CACNA1A O00555 VAR_063687 p.Thr500Met LP/P rs121908240 Episodic ataxia 2 (EA2) [MIM:108500]
CACNA1A O00555 VAR_063688 p.Gly637Asp LP/P rs121908246 Episodic ataxia 2 (EA2) [MIM:108500]
CACNA1A O00555 VAR_063689 p.Met797Thr LP/P rs121908241 Episodic ataxia 2 (EA2) [MIM:108500]
CACNA1A O00555 VAR_063690 p.Pro896Arg LP/P rs121908242 Episodic ataxia 2 (EA2) [MIM:108500]
CACNA1A O00555 VAR_063691 p.Arg1663Gln LP/P rs121908247 Spinocerebellar ataxia 6 (SCA6) [MIM:183086]
CACNA1A O00555 VAR_063692 p.Arg1678Cys LP/P rs121908243 Episodic ataxia 2 (EA2) [MIM:108500]
CACNA1A O00555 VAR_063693 p.Cys1868Arg LP/P rs121908244 Episodic ataxia 2 (EA2) [MIM:108500]
CACNA1A O00555 VAR_063706 p.Val1694Ile LP/P rs121908224 Migraine, familial hemiplegic, 1 (FHM1) [MIM:141500]
CACNA1A O00555 VAR_067342 p.Glu388Lys LP/P - Episodic ataxia 2 (EA2) [MIM:108500]
CACNA1A O00555 VAR_077071 p.Glu101Gln LP/P rs886037944 Developmental and epileptic encephalopathy 42 (DEE42) [MIM:617106]
CACNA1A O00555 VAR_077072 p.Ala712Thr LP/P rs886037945 Developmental and epileptic encephalopathy 42 (DEE42) [MIM:617106]
CACNA1A O00555 VAR_077073 p.Trp1435Arg LP/P - Developmental and epileptic encephalopathy 42 (DEE42) [MIM:617106]
CACNA1A O00555 VAR_077075 p.Ala1507Ser LP/P rs886037946 Developmental and epileptic encephalopathy 42 (DEE42) [MIM:617106]
CACNA1A O00555 VAR_079826 p.Arg1672Pro LB/B rs1057519429 -
CACNA1A O00555 VAR_080738 p.Asp1337Tyr LP/P rs1568473283 Spinocerebellar ataxia 6 (SCA6) [MIM:183086]
CACNA1B Q00975 VAR_048741 p.Asn167Lys LB/B rs4422842 -
CACNA1B Q00975 VAR_048742 p.Glu1436Lys LB/B rs1322525317 -
CACNA1B Q00975 VAR_048743 p.Glu1500Lys LB/B rs12377346 -
CACNA1B Q00975 VAR_061100 p.Ala862Ser LB/B rs7873074 -
CACNA1B Q00975 VAR_061101 p.Thr996Ala LB/B rs11137342 -
CACNA1B Q00975 VAR_073432 p.Arg1389His US rs184841813 -
CACNA1C Q13936 VAR_001495 p.Ala752Thr LB/B - -
CACNA1C Q13936 VAR_001496 p.Ala2169Thr LB/B - -
CACNA1C Q13936 VAR_026741 p.Gly402Ser LP/P - Timothy syndrome (TS) [MIM:601005]
CACNA1C Q13936 VAR_026742 p.Gly406Arg LP/P - Timothy syndrome (TS) [MIM:601005]
CACNA1C Q13936 VAR_044039 p.Ala39Val US - Brugada syndrome 3 (BRGDA3) [MIM:611875]
CACNA1C Q13936 VAR_044040 p.Gly490Arg US - Brugada syndrome 3 (BRGDA3) [MIM:611875]
CACNA1C Q13936 VAR_045987 p.Gln84Arg LB/B rs1051345 -
CACNA1C Q13936 VAR_045988 p.Ile391Leu LB/B rs1051356 -

CACNA1C	Q13936	VAR_059223	p.Pro1868Leu	LB/B	rs10848683	-
CACNA1C	Q13936	VAR_059224	p.Met1869Val	LB/B	rs10774053	-
CACNA1C	Q13936	VAR_061102	p.Lys1893Arg	LB/B	rs10774054	-
CACNA1C	Q13936	VAR_064700	p.Ser878Arg	US	-	-
CACNA1C	Q13936	VAR_072381	p.Ile1186Thr	LP/P	-	Long QT syndrome 8 (LQT8) [MIM:618447]
CACNA1C	Q13936	VAR_072381	p.Ile1186Thr	LP/P	-	Timothy syndrome (TS) [MIM:601005]
CACNA1C	Q13936	VAR_075148	p.Ala28Thr	US	-	Long QT syndrome 8 (LQT8) [MIM:618447]
CACNA1C	Q13936	VAR_075149	p.Gly37Arg	LB/B	-	-
CACNA1C	Q13936	VAR_075150	p.Ile304Thr	LB/B	-	-
CACNA1C	Q13936	VAR_075151	p.Pro381Ser	US	-	Long QT syndrome 8 (LQT8) [MIM:618447]
CACNA1C	Q13936	VAR_075152	p.Met456Ile	US	-	Long QT syndrome 8 (LQT8) [MIM:618447]
CACNA1C	Q13936	VAR_075153	p.Glu477Lys	US	-	Long QT syndrome 8 (LQT8) [MIM:618447]
CACNA1C	Q13936	VAR_075154	p.Arg518Cys	LP/P	-	Timothy syndrome (TS) [MIM:601005]
CACNA1C	Q13936	VAR_075155	p.Arg518His	LP/P	-	Timothy syndrome (TS) [MIM:601005]
CACNA1C	Q13936	VAR_075156	p.Ala582Asp	LP/P	-	Long QT syndrome 8 (LQT8) [MIM:618447]
CACNA1C	Q13936	VAR_075157	p.Pro817Ser	LB/B	-	-
CACNA1C	Q13936	VAR_075158	p.Arg858His	LP/P	-	Long QT syndrome 8 (LQT8) [MIM:618447]
CACNA1C	Q13936	VAR_075159	p.Arg860Gly	LP/P	-	Long QT syndrome 8 (LQT8) [MIM:618447]
CACNA1C	Q13936	VAR_075160	p.Ile1186Val	LP/P	-	Long QT syndrome 8 (LQT8) [MIM:618447]
CACNA1C	Q13936	VAR_075161	p.Ala1365Thr	US	-	Long QT syndrome 8 (LQT8) [MIM:618447]
CACNA1C	Q13936	VAR_075162	p.Ile1523Met	LP/P	-	Long QT syndrome 8 (LQT8) [MIM:618447]
CACNA1C	Q13936	VAR_075163	p.Glu1544Lys	LP/P	-	Long QT syndrome 8 (LQT8) [MIM:618447]
CACNA1C	Q13936	VAR_075164	p.Val1755Ile	LB/B	-	-
CACNA1C	Q13936	VAR_075165	p.Ala1765Gly	LB/B	-	-
CACNA1C	Q13936	VAR_075166	p.Asp1787Asn	US	-	Long QT syndrome 8 (LQT8) [MIM:618447]
CACNA1C	Q13936	VAR_075167	p.Thr1800Ile	US	-	Long QT syndrome 8 (LQT8) [MIM:618447]
CACNA1C	Q13936	VAR_075168	p.Gly1831Cys	US	-	Long QT syndrome 8 (LQT8) [MIM:618447]
CACNA1C	Q13936	VAR_075169	p.Thr1835Met	LB/B	-	-
CACNA1C	Q13936	VAR_075170	p.Gly1843Arg	LB/B	-	-
CACNA1C	Q13936	VAR_075171	p.Glu1948Lys	US	-	Long QT syndrome 8 (LQT8) [MIM:618447]
CACNA1C	Q13936	VAR_075172	p.Thr1953Met	US	-	Long QT syndrome 8 (LQT8) [MIM:618447]
CACNA1C	Q13936	VAR_075173	p.Arg1972Cys	LB/B	-	-
CACNA1C	Q13936	VAR_075174	p.Arg2056Gln	LB/B	rs112414325	-
CACNA1C	Q13936	VAR_075175	p.Thr2081Asn	US	rs771424529	Long QT syndrome 8 (LQT8) [MIM:618447]
CACNA1C	Q13936	VAR_075176	p.Val2097Ile	US	-	Long QT syndrome 8 (LQT8) [MIM:618447]
CACNA1C	Q13936	VAR_075177	p.Ala2122Gly	US	rs549476254	Long QT syndrome 8 (LQT8) [MIM:618447]
CACNA1C	Q13936	VAR_075178	p.Asn2174Ser	LB/B	-	-
CACNA1C	Q13936	VAR_076415	p.Ala2091Ser	US	-	-
CACNA1C	Q13936	VAR_078701	p.Arg1159His	US	-	-
CACNA1C	Q13936	VAR_082632	p.Lys834Glu	US	-	Long QT syndrome 8 (LQT8) [MIM:618447]
CACNA1C	Q13936	VAR_082633	p.Pro857Leu	LP/P	-	Long QT syndrome 8 (LQT8) [MIM:618447]
CACNA1C	Q13936	VAR_082634	p.Pro857Arg	LP/P	-	Long QT syndrome 8 (LQT8) [MIM:618447]
CACNA1C	Q13936	VAR_082635	p.Arg1989Gln	US	-	Long QT syndrome 8 (LQT8) [MIM:618447]
CACNA1D	Q01668	VAR_061103	p.Asp2097Asn	LB/B	rs41276455	-
CACNA1D	Q01668	VAR_070868	p.Gly403Asp	LP/P	-	Primary aldosteronism, seizures, and neurologic abnormalities (PASNA) [MIM:615474]
CACNA1D	Q01668	VAR_070869	p.Ile750Met	LP/P	rs41276445	Primary aldosteronism, seizures, and neurologic abnormalities (PASNA) [MIM:615474]
CACNA1D	Q01668	VAR_079531	p.Val401Leu	LP/P	-	-

CACNA1D Q01668 VAR_079532 p.Gly407Arg LP/P rs1163276899 -
CACNA1D Q01668 VAR_079533 p.Ala749Gly LP/P - -
CACNA1E Q15878 VAR_031912 p.Asp859Glu LB/B rs35737760 -
CACNA1E Q15878 VAR_046996 p.Ala1955Thr LB/B rs704326 -
CACNA1E Q15878 VAR_081838 p.Leu228Pro LP/P rs1553286282 Developmental and epileptic encephalopathy 69 (DEE69) [MIM:618285]
CACNA1E Q15878 VAR_081839 p.Gly348Arg LP/P - Developmental and epileptic encephalopathy 69 (DEE69) [MIM:618285]
CACNA1E Q15878 VAR_081840 p.Gly352Arg LP/P rs886039323 Developmental and epileptic encephalopathy 69 (DEE69) [MIM:618285]
CACNA1E Q15878 VAR_081841 p.Ile603Leu LP/P rs778291283 Developmental and epileptic encephalopathy 69 (DEE69) [MIM:618285]
CACNA1E Q15878 VAR_081842 p.Gly690Asp LP/P rs1361083258 Developmental and epileptic encephalopathy 69 (DEE69) [MIM:618285]
CACNA1E Q15878 VAR_081843 p.Phe698Ser LP/P rs869312920 Developmental and epileptic encephalopathy 69 (DEE69) [MIM:618285]
CACNA1E Q15878 VAR_081844 p.Ala700Thr LP/P - Developmental and epileptic encephalopathy 69 (DEE69) [MIM:618285]
CACNA1E Q15878 VAR_081845 p.Ile701Val LP/P - Developmental and epileptic encephalopathy 69 (DEE69) [MIM:618285]
CACNA1E Q15878 VAR_081846 p.Ala702Pro LP/P rs12131800 Developmental and epileptic encephalopathy 69 (DEE69) [MIM:618285]
CACNA1E Q15878 VAR_081847 p.Ala702Thr LP/P rs12131800 Developmental and epileptic encephalopathy 69 (DEE69) [MIM:618285]
CACNA1E Q15878 VAR_081850 p.Ile1422Phe LP/P - Developmental and epileptic encephalopathy 69 (DEE69) [MIM:618285]
CACNA1E Q15878 VAR_081851 p.Thr1425Asn LP/P - Developmental and epileptic encephalopathy 69 (DEE69) [MIM:618285]
CACNA1E Q15878 VAR_081852 p.Gly1430Arg LP/P rs1553345844 Developmental and epileptic encephalopathy 69 (DEE69) [MIM:618285]
CACNA1E Q15878 VAR_081853 p.Ala1720Gly LP/P - Developmental and epileptic encephalopathy 69 (DEE69) [MIM:618285]
CACNA1F O60840 VAR_001504 p.Gly369Asp LP/P rs122456133 Night blindness, congenital stationary, 2A (CSNB2A) [MIM:300071]
CACNA1F O60840 VAR_001505 p.Arg519Gln LP/P rs34162630 Night blindness, congenital stationary, 2A (CSNB2A) [MIM:300071]
CACNA1F O60840 VAR_001506 p.Arg1060Trp LP/P - Night blindness, congenital stationary, 2A (CSNB2A) [MIM:300071]
CACNA1F O60840 VAR_001507 p.Leu1375His LP/P - Night blindness, congenital stationary, 2A (CSNB2A) [MIM:300071]
CACNA1F O60840 VAR_029376 p.Asn746Thr LB/B rs141159097 -
CACNA1F O60840 VAR_030807 p.Pro14Leu LB/B rs6520408 -
CACNA1F O60840 VAR_030808 p.Cys74Arg LP/P - Night blindness, congenital stationary, 2A (CSNB2A) [MIM:300071]
CACNA1F O60840 VAR_030809 p.Gly150Arg LP/P - Night blindness, congenital stationary, 2A (CSNB2A) [MIM:300071]
CACNA1F O60840 VAR_030810 p.Ser229Pro LP/P - Night blindness, congenital stationary, 2A (CSNB2A) [MIM:300071]

CACNA1F O60840 VAR_030811 p.Gly261Arg LP/P - Night blindness, congenital stationary, 2A (CSNB2A) [MIM:300071]
CACNA1F O60840 VAR_030812 p.Val635Ile US rs141010716 Night blindness, congenital stationary, 2A (CSNB2A) [MIM:300071]
CACNA1F O60840 VAR_030813 p.Gly674Asp LP/P - Night blindness, congenital stationary, 2A (CSNB2A) [MIM:300071]
CACNA1F O60840 VAR_030814 p.Phe753Cys LP/P rs1602644716 Night blindness, congenital stationary, 2A (CSNB2A) [MIM:300071]
CACNA1F O60840 VAR_030815 p.Ile756Thr LP/P rs122456136 Night blindness, congenital stationary, 2A (CSNB2A) [MIM:300071]
CACNA1F O60840 VAR_030816 p.Leu860Pro LP/P - Night blindness, congenital stationary, 2A (CSNB2A) [MIM:300071]
CACNA1F O60840 VAR_030817 p.Ala928Asp LP/P - Night blindness, congenital stationary, 2A (CSNB2A) [MIM:300071]
CACNA1F O60840 VAR_030818 p.Gly1018Arg LP/P rs1249437161 Night blindness, congenital stationary, 2A (CSNB2A) [MIM:300071]
CACNA1F O60840 VAR_030819 p.Leu1079Pro LP/P - Night blindness, congenital stationary, 2A (CSNB2A) [MIM:300071]
CACNA1F O60840 VAR_030820 p.Cys1499Arg LP/P - Night blindness, congenital stationary, 2A (CSNB2A) [MIM:300071]
CACNA1F O60840 VAR_030821 p.Pro1500Arg LP/P - Night blindness, congenital stationary, 2A (CSNB2A) [MIM:300071]
CACNA1F O60840 VAR_030822 p.Leu1508Pro LP/P - Night blindness, congenital stationary, 2A (CSNB2A) [MIM:300071]
CACNA1F O60840 VAR_031822 p.Ala1270Thr LB/B rs34308720 -
CACNA1F O60840 VAR_054818 p.Arg1930His LB/B rs33910054 -
CACNA1F O60840 VAR_055662 p.Ala1259Thr LB/B rs34308720 -
CACNA1F O60840 VAR_071433 p.Gly603Arg LP/P rs201654095 Aaland island eye disease (AIED) [MIM:300600]
CACNA1F O60840 VAR_071433 p.Gly603Arg LP/P rs201654095 Night blindness, congenital stationary, 2A (CSNB2A) [MIM:300071]
CACNA1G O43497 VAR_076292 p.Arg1715His LP/P rs755221106 Spinocerebellar ataxia 42 (SCA42) [MIM:616795]
CACNA1G O43497 VAR_081177 p.Ala961Thr LP/P rs886041505 Spinocerebellar ataxia 42, early-onset, severe, with neurodevelopmental deficits (SCA42ND) [MIM:618087]
CACNA1G O43497 VAR_081178 p.Met1531Val LP/P rs1555558553 Spinocerebellar ataxia 42, early-onset, severe, with neurodevelopmental deficits (SCA42ND) [MIM:618087]
CACNA1H O95180 VAR_033698 p.Glu1974Gly LB/B rs3751886 -
CACNA1H O95180 VAR_045935 p.Phe161Leu LP/P rs119454947 Epilepsy, childhood absence 6 (ECA6) [MIM:611942]
CACNA1H O95180 VAR_045936 p.Glu282Lys LP/P rs119454948 Epilepsy, childhood absence 6 (ECA6) [MIM:611942]
CACNA1H O95180 VAR_045937 p.Met313Val LB/B rs36117280 -
CACNA1H O95180 VAR_045938 p.Cys456Ser LP/P - Epilepsy, childhood absence 6 (ECA6) [MIM:611942]
CACNA1H O95180 VAR_045939 p.Gly499Ser LP/P rs560915333 Epilepsy, childhood absence 6 (ECA6) [MIM:611942]
CACNA1H O95180 VAR_045940 p.Pro640Leu LB/B rs61734410 -
CACNA1H O95180 VAR_045941 p.Pro648Leu LP/P rs1288484976 Epilepsy, childhood absence 6 (ECA6) [MIM:611942]
CACNA1H O95180 VAR_045942 p.Val664Ala LB/B rs4984636 -

CACNA1H O95180 VAR_045943 p.Pro684Ser LB/B rs762185083 -
CACNA1H O95180 VAR_045944 p.Arg744Gln LP/P rs373764821 Epilepsy, childhood absence 6 (ECA6) [MIM:611942]
CACNA1H O95180 VAR_045945 p.Ala748Val LP/P rs770371468 Epilepsy, childhood absence 6 (ECA6) [MIM:611942]
CACNA1H O95180 VAR_045946 p.Gly773Asp LP/P rs267606697 Epilepsy, childhood absence 6 (ECA6) [MIM:611942]
CACNA1H O95180 VAR_045947 p.Gly784Ser LP/P rs779526640 Epilepsy, childhood absence 6 (ECA6) [MIM:611942]
CACNA1H O95180 VAR_045948 p.Arg788Cys LB/B rs3751664 -
CACNA1H O95180 VAR_045949 p.Val812Met LB/B rs28365119 -
CACNA1H O95180 VAR_045950 p.Val831Met LP/P rs119454949 Epilepsy, childhood absence 6 (ECA6) [MIM:611942]
CACNA1H O95180 VAR_045951 p.Gly848Ser LP/P rs374272094 Epilepsy, childhood absence 6 (ECA6) [MIM:611942]
CACNA1H O95180 VAR_045952 p.Asp1463Asn LP/P rs542245543 Epilepsy, childhood absence 6 (ECA6) [MIM:611942]
CACNA1H O95180 VAR_045953 p.Arg2060His LB/B rs1054644 -
CACNA1H O95180 VAR_045954 p.Arg2077His LB/B rs1054645 -
CACNA1H O95180 VAR_045955 p.Pro2173Ser LB/B rs200675829 -
CACNA1H O95180 VAR_061104 p.Arg1871Gln LB/B rs58124832 -
CACNA1H O95180 VAR_066986 p.Pro618Leu LP/P rs60734921 Epilepsy, idiopathic generalized 6 (EIG6) [MIM:611942]
CACNA1H O95180 VAR_066987 p.Gly755Asp LP/P rs142306293 Epilepsy, idiopathic generalized 6 (EIG6) [MIM:611942]
CACNA1H O95180 VAR_077064 p.Ser196Leu LP/P rs780596901 Hyperaldosteronism, familial, 4 (HALD4) [MIM:617027]
CACNA1H O95180 VAR_077065 p.Met1549Ile LP/P - Hyperaldosteronism, familial, 4 (HALD4) [MIM:617027]
CACNA1H O95180 VAR_077066 p.Met1549Val LP/P rs786205050 Hyperaldosteronism, familial, 4 (HALD4) [MIM:617027]
CACNA1H O95180 VAR_077067 p.Val1951Glu LP/P rs746967306 -
CACNA1H O95180 VAR_077068 p.Pro2083Leu LP/P rs759924732 Hyperaldosteronism, familial, 4 (HALD4) [MIM:617027]
CACNA1H O95180 VAR_078237 p.His516Tyr US rs1057519554 -
CACNA1H O95180 VAR_078702 p.Ser1970Cys US rs1267377730 -
CACNA1I Q9P0X4 VAR_013883 p.Ile1040Val LB/B rs136853 -
CACNA1I Q9P0X4 VAR_013884 p.Gly1782Ala LB/B rs2294369 -
CACNA1I Q9P0X4 VAR_020050 p.Gly1782Arg LB/B rs2294369 -
CACNA1I Q9P0X4 VAR_048745 p.Thr1513Met LB/B rs8141262 -
CACNA1S Q13698 VAR_001498 p.Leu458His LB/B rs12742169 -
CACNA1S Q13698 VAR_001499 p.Arg528His LP/P rs80338777 Periodic paralysis hypokalemic 1 (HOKPP1) [MIM:170400]
CACNA1S Q13698 VAR_001500 p.Arg1086His LP/P rs1800559 Malignant hyperthermia 5 (MHS5) [MIM:601887]
CACNA1S Q13698 VAR_001501 p.Arg1239Gly LP/P rs28930069 Periodic paralysis hypokalemic 1 (HOKPP1) [MIM:170400]
CACNA1S Q13698 VAR_001502 p.Arg1239His LP/P rs28930068 Periodic paralysis hypokalemic 1 (HOKPP1) [MIM:170400]

CACNA1S Q13698 VAR_001503 p.Arg1539Cys LB/B rs3850625 -
CACNA1S Q13698 VAR_046970 p.Ala69Gly LB/B rs12406479 -
CACNA1S Q13698 VAR_046971 p.Arg1658His LB/B rs13374149 -
CACNA1S Q13698 VAR_046972 p.Leu1800Ser LB/B rs12139527 -
CACNA1S Q13698 VAR_046973 p.Glu1840Asp LB/B rs1042379 -
CACNA1S Q13698 VAR_054953 p.Arg528Gly LP/P rs80338778 Periodic paralysis hypokalemic 1 (HOKPP1) [MIM:170400]
CACNA1S Q13698 VAR_054954 p.Arg900Ser LP/P - Periodic paralysis hypokalemic 1 (HOKPP1) [MIM:170400]
CACNA2D1 P54289 VAR_035047 p.Asp1057Ala LB/B rs35131433 -
CACNA2D1 P54289 VAR_053960 p.Glu1019Asp LB/B rs9886043 -
CACNA2D2 Q9NY47 VAR_035048 p.Ala138Val LB/B rs35497591 -
CACNA2D2 Q9NY47 VAR_057782 p.Glu334Lys LB/B rs743855 -
CACNA2D2 Q9NY47 VAR_083105 p.Pro261Leu US rs1211603072 Cerebellar atrophy with seizures and variable developmental delay (CASVDD) [MIM:618501]
CACNA2D2 Q9NY47 VAR_083106 p.Leu1047Pro LP/P rs587776948 Cerebellar atrophy with seizures and variable developmental delay (CASVDD) [MIM:618501]
CACNA2D2 Q9NY47 VAR_083107 p.Leu1053Pro US rs1575578837 Cerebellar atrophy with seizures and variable developmental delay (CASVDD) [MIM:618501]
CACNA2D4 Q7Z3S7 VAR_035049 p.Ile327Val LB/B rs10735005 -
CACNA2D4 Q7Z3S7 VAR_035050 p.Arg863His LB/B rs36077411 -
CACNA2D4 Q7Z3S7 VAR_035051 p.Thr869Met LB/B rs35331095 -
CACNB1 Q02641 VAR_036349 p.Pro339Leu US rs1266205915 A colorectal cancer sample
CACNB2 Q08289 VAR_036350 p.Ala99Gly US rs745502425 A colorectal cancer sample
CACNB2 Q08289 VAR_044041 p.Ser535Leu US rs121917812 Brugada syndrome 4 (BRGDA4) [MIM:611876]
CACNB3 P54284 VAR_024384 p.Arg423His LB/B rs2229954 -
CACNB4 O00305 VAR_013669 p.Cys104Phe LP/P rs1805031 Episodic ataxia 5 (EA5) [MIM:613855]
CACNG1 Q06432 VAR_012063 p.Gly196Ser LB/B rs1799938 -
CACNG2 Q9Y698 VAR_066599 p.Val143Leu LP/P - Mental retardation, autosomal dominant 10 (MRD10) [MIM:614256]
CACNG6 Q9BXT2 VAR_061540 p.Cys252Ser LB/B rs12980121 -
CAD P27708 VAR_035897 p.Arg177Gln US rs374122292 A colorectal cancer sample
CAD P27708 VAR_035898 p.Tyr735Cys US - A colorectal cancer sample
CAD P27708 VAR_073955 p.Arg2024Gln LP/P rs763410987 Developmental and epileptic encephalopathy 50 (DEE50) [MIM:616457]
CAD P27708 VAR_078289 p.Met33Arg US rs751610198 Developmental and epileptic encephalopathy 50 (DEE50) [MIM:616457]
CADM1 Q9BY67 VAR_061309 p.Asp285Glu LB/B rs45525440 -
CADM3 Q8N126 VAR_059383 p.Arg162Trp LB/B rs3026987 -
CADM4 Q8NFZ8 VAR_032906 p.Thr225Ala LB/B rs34246023 -
CADPS2 Q86UW7 VAR_024786 p.Ala298Thr LB/B rs17144625 -
CAGE1 Q8TC20 VAR_031200 p.Thr169Ile LB/B rs10223538 -
CAGE1 Q8TC20 VAR_031201 p.Glu282Ala LB/B rs2876098 -
CALCA P01258 VAR_014592 p.Asp57Asn LB/B rs5239 -
CALCA P01258 VAR_014593 p.Ser76Arg LB/B rs5241 -
CALCA P01258 VAR_025271 p.Gly2Arg LB/B rs34587547 -
CALCA P01258 VAR_025272 p.Glu67Lys LB/B rs34164367 -
CALCA P01258 VAR_025273 p.Ser123Thr LB/B rs34414857 -
CALCA P01258 VAR_025274 p.Gln138Pro LB/B rs13306224 -

CALCA P06881 VAR_048584 p.Asp57Asn LB/B rs5239 -
CALCOCO1 Q9P1Z2 VAR_036881 p.Arg393Lys LB/B rs3741659 -
CALCOCO2 Q13137 VAR_037489 p.Gly140Glu LB/B rs550510 -
CALCOCO2 Q13137 VAR_037490 p.Gly227Arg LB/B rs2303016 -
CALCOCO2 Q13137 VAR_037491 p.Val248Ala LB/B rs2303015 -
CALCOCO2 Q13137 VAR_037492 p.Thr273Ala LB/B rs17849804 -
CALCOCO2 Q13137 VAR_037493 p.Pro389Ala LB/B rs10278 -
CALCR P30988 VAR_003580 p.Pro447Leu LB/B rs1801197 -
CALCRL Q16602 VAR_049453 p.Phe16Leu LB/B rs13391909 -
CALCRL Q16602 VAR_049454 p.Arg274Ile LB/B rs34010553 -
CALCRL Q16602 VAR_054822 p.Asn8Tyr LB/B rs698577 -
CALD1 Q05682 VAR_065254 p.His397Arg LB/B rs6973420 -
CALHM1 Q8IU99 VAR_023095 p.Leu86Pro LB/B rs2986017 -
CALHM2 Q9HA72 VAR_033924 p.Val194Met LB/B rs2232662 -
CALHM2 Q9HA72 VAR_053084 p.Val136Gly LB/B rs2232660 -
CALHM6 Q5R3K3 VAR_053085 p.Gly80Arg LB/B rs1057192 -
CALHM6 Q5R3K3 VAR_053086 p.Glu293Lys LB/B rs11544160 -
CALHM6 Q5R3K3 VAR_071081 p.Thr100Ala LB/B rs10784 -
CALM1 P0DP23 VAR_069222 p.Asn54Ile LP/P rs267607276 Ventricular tachycardia, catecholaminergic polymorphic, 4 (CPVT4) [MIM:614916]
CALM1 P0DP23 VAR_073275 p.Phe90Leu LP/P rs730882253 Long QT syndrome 14 (LQT14) [MIM:616247]
CALM1 P0DP23 VAR_073282 p.Phe142Leu LP/P rs1085307479 Long QT syndrome 14 (LQT14) [MIM:616247]
CALM1 P0DP23 VAR_078263 p.Glu141Gly LP/P - Long QT syndrome 14 (LQT14) [MIM:616247]
CALM1 P0DP23 VAR_078541 p.Asn98Ser LP/P rs267607277 Ventricular tachycardia, catecholaminergic polymorphic, 4 (CPVT4) [MIM:614916]
CALM1 P0DP23 VAR_078542 p.Asp130Gly LP/P rs730882252 Long QT syndrome 14 (LQT14) [MIM:616247]
CALM1 P0DP23 VAR_083814 p.Glu141Val LP/P - Long QT syndrome 14 (LQT14) [MIM:616247]
CALM2 P0DP24 VAR_073276 p.Asp96Val LP/P rs730882254 Long QT syndrome 15 (LQT15) [MIM:616249]
CALM2 P0DP24 VAR_073277 p.Asn98Ile LP/P rs398124647 Long QT syndrome 15 (LQT15) [MIM:616249]
CALM2 P0DP24 VAR_073279 p.Asp132Glu LP/P rs398124648 Long QT syndrome 15 (LQT15) [MIM:616249]
CALM2 P0DP24 VAR_073280 p.Asp134His LP/P rs398124650 Long QT syndrome 15 (LQT15) [MIM:616249]
CALM2 P0DP24 VAR_073281 p.Gln136Pro LP/P rs398124649 Long QT syndrome 15 (LQT15) [MIM:616249]
CALM2 P0DP24 VAR_078262 p.Asp130Val LP/P - Long QT syndrome 15 (LQT15) [MIM:616249]
CALM2 P0DP24 VAR_078543 p.Asn98Ser LP/P rs398124647 Long QT syndrome 15 (LQT15) [MIM:616249]
CALM2 P0DP24 VAR_078544 p.Asp130Gly LP/P rs730882252 Long QT syndrome 15 (LQT15) [MIM:616249]
CALM3 P0DP25 VAR_078261 p.Ala103Val LP/P - Ventricular tachycardia, catecholaminergic polymorphic, 6 (CPVT6) [MIM:618782]
CALM3 P0DP25 VAR_083815 p.Asp130Gly LP/P - Long QT syndrome 16 (LQT16) [MIM:618782]
CALM3 P0DP25 VAR_083816 p.Glu141Lys LP/P - Long QT syndrome 16 (LQT16) [MIM:618782]
CALML4 Q96GE6 VAR_048586 p.Arg28Cys LB/B rs3803381 -
CALML4 Q96GE6 VAR_048587 p.Thr154Lys LB/B rs2280217 -
CALML5 Q9NZT1 VAR_047545 p.Ser58Gly LB/B rs11546426 -
CALML5 Q9NZT1 VAR_047546 p.Lys74Arg LB/B rs10904516 -
CALML6 Q8TD86 VAR_048588 p.Trp60Arg LB/B rs28581776 -
CALR3 Q96L12 VAR_027944 p.Leu8Phe LB/B rs11544148 -
CALR3 Q96L12 VAR_027945 p.Asp248Gly LB/B rs10411092 -
CALR3 Q96L12 VAR_027946 p.Val274Ile LB/B rs12459238 -
CALR3 Q96L12 VAR_048589 p.Asp284Asn LB/B rs10404156 -
CALR3 Q96L12 VAR_065476 p.Lys82Arg US rs142951029 -

CALU O43852 VAR_022051 p.Arg4Gln LB/B rs2290228 -
CAMK1 Q14012 VAR_040596 p.Pro217Ser US rs907102077 A metastatic melanoma sample
CAMK1 Q14012 VAR_040597 p.Glu361Lys LB/B rs56033923 -
CAMK1D Q8IU85 VAR_040599 p.Ile66Met LB/B rs34194224 -
CAMK1G Q96NX5 VAR_020530 p.Val329Ile LB/B rs11119315 -
CAMK1G Q96NX5 VAR_040600 p.Glu259Gln LB/B rs35561962 -
CAMK1G Q96NX5 VAR_040601 p.Ala443Thr US rs1228276529 A breast infiltrating ductal carcinoma sample
CAMK2A Q9UQM7 VAR_080579 p.Phe98Ser LP/P rs1554122526 Mental retardation, autosomal dominant 53 (MRD53) [MIM:617798]
CAMK2A Q9UQM7 VAR_080580 p.Glu109Asp LP/P - Mental retardation, autosomal dominant 53 (MRD53) [MIM:617798]
CAMK2A Q9UQM7 VAR_080581 p.Ala112Val US - Mental retardation, autosomal dominant 53 (MRD53) [MIM:617798]
CAMK2A Q9UQM7 VAR_080582 p.Pro138Ala US - Mental retardation, autosomal dominant 53 (MRD53) [MIM:617798]
CAMK2A Q9UQM7 VAR_080583 p.Glu183Val LP/P rs1554122129 Mental retardation, autosomal dominant 53 (MRD53) [MIM:617798]
CAMK2A Q9UQM7 VAR_080584 p.Pro212Leu US rs926027867 Mental retardation, autosomal dominant 53 (MRD53) [MIM:617798]
CAMK2A Q9UQM7 VAR_080585 p.Pro235Leu US rs864309606 Mental retardation, autosomal dominant 53 (MRD53) [MIM:617798]
CAMK2A Q9UQM7 VAR_080586 p.His282Arg LP/P rs1554121875 Mental retardation, autosomal dominant 53 (MRD53) [MIM:617798]
CAMK2A Q9UQM7 VAR_080587 p.Thr286Pro LP/P rs1554121872 Mental retardation, autosomal dominant 53 (MRD53) [MIM:617798]
CAMK2A Q9UQM7 VAR_081160 p.Pro212Gln LP/P - Mental retardation, autosomal dominant 53 (MRD53) [MIM:617798]
CAMK2A Q9UQM7 VAR_081161 p.His466Tyr LP/P rs1554119274 Mental retardation, autosomal recessive 63 (MRT63) [MIM:618095]
CAMK2B Q13554 VAR_045581 p.Pro489Leu US rs555460132 A colorectal adenocarcinoma sample
CAMK2B Q13554 VAR_045582 p.Glu510Lys LB/B rs35452727 -
CAMK2B Q13554 VAR_080589 p.Glu110Lys LP/P rs1554402092 Mental retardation, autosomal dominant 54 (MRD54) [MIM:617799]
CAMK2B Q13554 VAR_080590 p.Pro139Leu LP/P rs1554389088 Mental retardation, autosomal dominant 54 (MRD54) [MIM:617799]
CAMK2B Q13554 VAR_080591 p.Glu237Lys LP/P rs1554386687 Mental retardation, autosomal dominant 54 (MRD54) [MIM:617799]
CAMK2B Q13554 VAR_080592 p.Lys301Glu LP/P rs1554385111 Mental retardation, autosomal dominant 54 (MRD54) [MIM:617799]
CAMK2B Q13554 VAR_081162 p.Pro213Leu LP/P rs1554387293 Mental retardation, autosomal dominant 54 (MRD54) [MIM:617799]
CAMK2B Q13554 VAR_081163 p.Arg284Ser LP/P rs1554385203 Mental retardation, autosomal dominant 54 (MRD54) [MIM:617799]
CAMK2D Q13557 VAR_028196 p.Gln463Glu LB/B rs1053668 -
CAMK2D Q13557 VAR_040602 p.Asp167Glu LB/B rs35367671 -
CAMK2D Q13557 VAR_040603 p.Thr493Ile LB/B rs35765784 -
CAMK2G Q13555 VAR_042430 p.Ser36Pro LB/B rs17853266 -
CAMK2G Q13555 VAR_069390 p.Arg292Pro LP/P rs397514627 Intellectual developmental disorder, autosomal dominant 59 (MRD59) [MIM:618522]

CAMK4	Q16566	VAR_040604	p.Glu150Gly	US	-	A lung adenocarcinoma sample
CAMK4	Q16566	VAR_040605	p.Asp178Asn	LB/B	rs35548075	-
CAMK4	Q16566	VAR_040606	p.Gln465Arg	LB/B	rs56360861	-
CAMK4	Q16566	VAR_040607	p.Ile469Met	US	rs1239950009	A lung large cell carcinoma sample
CAMKK1	Q8N5S9	VAR_020531	p.Glu375Gly	LB/B	rs7214723	-
CAMKK2	Q96RR4	VAR_020532	p.Thr85Ser	LB/B	rs3817190	-
CAMKK2	Q96RR4	VAR_020533	p.Arg363Cys	LB/B	rs1132780	-
CAMKK2	Q96RR4	VAR_032788	p.Ser10Asn	LB/B	rs28360477	-
CAMKK2	Q96RR4	VAR_040610	p.Cys123Tyr	LB/B	rs35403710	-
CAMKK2	Q96RR4	VAR_040611	p.Pro127Leu	US	-	A lung neuroendocrine carcinoma sample
CAMKK2	Q96RR4	VAR_040612	p.Ala182Thr	US	rs1207121718	A colorectal adenocarcinoma sample
CAMKK2	Q96RR4	VAR_040613	p.Arg492His	LB/B	rs34129994	-
CAMKV	Q8NCB2	VAR_027539	p.Tyr491Cys	LB/B	rs17849325	-
CAMKV	Q8NCB2	VAR_041337	p.Arg40Trp	US	rs1407808379	A colorectal adenocarcinoma sample
CAMKV	Q8NCB2	VAR_041338	p.Gly60Ser	US	-	An ovarian serous carcinoma sample
CAMKV	Q8NCB2	VAR_041339	p.Arg274Trp	US	-	A colorectal adenocarcinoma sample
CAMKV	Q8NCB2	VAR_041340	p.Glu279Asp	LB/B	rs56071455	-
CAMKV	Q8NCB2	VAR_041341	p.Pro472Leu	LB/B	rs56307047	-
CAMLG	P49069	VAR_024297	p.Val78Ile	LB/B	rs12657663	-
CAMLG	P49069	VAR_050710	p.Gly100Ser	LB/B	rs11552197	-
CAMSAP1	Q5T5Y3	VAR_038398	p.Ala476Val	LB/B	rs35639321	-
CAMSAP2	Q08AD1	VAR_038399	p.Ile361Leu	US	-	A colorectal cancer sample
CAMSAP2	Q08AD1	VAR_038400	p.Pro958Leu	LB/B	rs3753952	-
CAMSAP2	Q08AD1	VAR_038401	p.Arg1028Pro	LB/B	rs6674599	-
CAMSAP2	Q08AD1	VAR_057796	p.Pro969Leu	LB/B	rs3753952	-
CAMSAP2	Q08AD1	VAR_057797	p.Pro1039Arg	LB/B	rs6674599	-
CAMSAP3	Q9P1Y5	VAR_053991	p.Pro335Ser	LB/B	rs3745358	-
CAMTA1	Q9Y6Y1	VAR_047824	p.Asn1177Lys	LB/B	rs41278952	-
CAMTA1	Q9Y6Y1	VAR_047825	p.Asn1218Thr	LB/B	rs41278954	-
CAMTA1	Q9Y6Y1	VAR_047826	p.Thr1336Ile	LB/B	rs137974312	-
CAMTA2	O94983	VAR_026417	p.Ala267Pro	LB/B	rs238234	-
CAMTA2	O94983	VAR_026418	p.Ser903Pro	LB/B	rs16942615	-
CAND1	Q86VP6	VAR_025327	p.Ala952Val	LB/B	rs17854618	-
CAND1	Q86VP6	VAR_054041	p.Val803Ala	LB/B	rs12580996	-
CAND2	O75155	VAR_055023	p.Gln408Arg	LB/B	rs2305398	-
CAND2	O75155	VAR_055024	p.Pro476Leu	LB/B	rs2305397	-
CAND2	O75155	VAR_055025	p.Ser533Pro	LB/B	rs3732675	-
CAND2	O75155	VAR_055026	p.His655Arg	LB/B	rs9838943	-
CAND2	O75155	VAR_055027	p.Leu845Phe	LB/B	rs17037287	-
CAND2	O75155	VAR_055028	p.His858Pro	LB/B	rs3732678	-
CAND2	O75155	VAR_055029	p.Val990Ile	LB/B	rs3817121	-
CAND2	O75155	VAR_055030	p.Ala1225Thr	LB/B	rs12629133	-
CANT1	Q8WVQ1	VAR_062980	p.Pro299Leu	LP/P	rs267606700	Desbuquois dysplasia 1 (DBQD1) [MIM:251450]
CANT1	Q8WVQ1	VAR_062981	p.Arg300Cys	LP/P	rs267606701	Desbuquois dysplasia 1 (DBQD1) [MIM:251450]
CANT1	Q8WVQ1	VAR_062982	p.Arg300His	LP/P	rs267606699	Desbuquois dysplasia 1 (DBQD1) [MIM:251450]
CANT1	Q8WVQ1	VAR_068655	p.Asp112Glu	LP/P	rs749246739	Desbuquois dysplasia 1 (DBQD1) [MIM:251450]
CANT1	Q8WVQ1	VAR_068656	p.Trp125Cys	LP/P	rs587776898	Desbuquois dysplasia 1 (DBQD1) [MIM:251450]
CANT1	Q8WVQ1	VAR_068657	p.Met165Thr	LP/P	-	Desbuquois dysplasia 1 (DBQD1) [MIM:251450]
CANT1	Q8WVQ1	VAR_068658	p.Leu224Pro	LP/P	rs150181226	Desbuquois dysplasia 1 (DBQD1) [MIM:251450]

CANT1 Q8WVQ1 VAR_068659 p.Val226Met LP/P rs377546036 Desbuquois dysplasia 1 (DBQD1)
[MIM:251450]
CANT1 Q8WVQ1 VAR_068659 p.Val226Met LP/P rs377546036 Epiphyseal dysplasia, multiple, 7 (EDM7)
[MIM:617719]
CANT1 Q8WVQ1 VAR_068660 p.Ser303Arg LP/P - Desbuquois dysplasia 1 (DBQD1) [MIM:251450]
CANT1 Q8WVQ1 VAR_068661 p.Ala323Thr LB/B rs9903215 -
CANT1 Q8WVQ1 VAR_068662 p.Ala360Asp LP/P rs387907081 Desbuquois dysplasia 1 (DBQD1) [MIM:251450]
CANT1 Q8WVQ1 VAR_068663 p.Ile374Asn LP/P - Desbuquois dysplasia 1 (DBQD1) [MIM:251450]
CANT1 Q8WVQ1 VAR_068664 p.Gly391Glu LB/B rs34082669 -
CANT1 Q8WVQ1 VAR_080400 p.Ile171Phe US rs1014317450 Epiphyseal dysplasia, multiple, 7 (EDM7)
[MIM:617719]
CAP1 Q01518 VAR_028419 p.Cys229Gly LB/B rs11207440 -
CAP1 Q01518 VAR_028420 p.Cys236Gly LB/B rs6665926 -
CAP1 Q01518 VAR_028421 p.Ile245Ser LB/B rs6665933 -
CAP1 Q01518 VAR_028422 p.Cys247Gly LB/B rs6665936 -
CAP1 Q01518 VAR_028423 p.Tyr249Asp LB/B rs6665937 -
CAP1 Q01518 VAR_028424 p.Ser256Ala LB/B rs6665944 -
CAP2 P40123 VAR_033717 p.Thr311Ala LB/B rs34620829 -
CAP2 P40123 VAR_033718 p.Tyr316Cys LB/B rs34206659 -
CAPG P40121 VAR_047776 p.Val41Ile LB/B rs2229668 -
CAPG P40121 VAR_047777 p.Arg198Trp LB/B rs11539103 -
CAPG P40121 VAR_047778 p.His335Arg LB/B rs6886 -
CAPN1 P07384 VAR_021085 p.Thr103Ala LB/B rs17885718 -
CAPN1 P07384 VAR_021086 p.Arg433Pro LB/B rs10895991 -
CAPN1 P07384 VAR_021087 p.Gly492Arg LB/B rs17883283 -
CAPN1 P07384 VAR_021088 p.Val676Ile LB/B rs17884773 -
CAPN1 P07384 VAR_077899 p.Arg295Pro LP/P rs756205995 Spastic paraplegia 76, autosomal recessive
(SPG76) [MIM:616907]
CAPN10 Q9HC96 VAR_014437 p.Pro200Thr LB/B rs3792268 -
CAPN10 Q9HC96 VAR_014438 p.Arg202His LB/B rs768407925 -
CAPN10 Q9HC96 VAR_014439 p.Ala341Val LB/B rs776848131 -
CAPN10 Q9HC96 VAR_014440 p.Thr504Ala LB/B rs7607759 -
CAPN10 Q9HC96 VAR_014441 p.Ala529Ser LB/B - -
CAPN10 Q9HC96 VAR_014442 p.Ser613Asn LB/B rs146148004 -
CAPN10 Q9HC96 VAR_014443 p.Ile666Val LB/B rs2975766 -
CAPN10 Q9HC96 VAR_036049 p.Glu276Gly US - A colorectal cancer sample
CAPN11 Q9UMQ6 VAR_024587 p.Ser728Asn LB/B rs7761137 -
CAPN11 Q9UMQ6 VAR_033713 p.Val266Met LB/B rs6938938 -
CAPN11 Q9UMQ6 VAR_033714 p.Val441Ala LB/B rs16871612 -
CAPN11 Q9UMQ6 VAR_033715 p.Ile521Val LB/B rs34710081 -
CAPN11 Q9UMQ6 VAR_033716 p.Ser544Arg LB/B rs35527493 -
CAPN12 Q6ZSI9 VAR_051516 p.Cys287Arg LB/B rs12983010 -
CAPN13 Q6MZZ7 VAR_028964 p.His249Tyr LB/B rs17010210 -
CAPN13 Q6MZZ7 VAR_028965 p.Ala280Thr LB/B rs508405 -
CAPN13 Q6MZZ7 VAR_028966 p.Ile596Thr LB/B rs2276568 -
CAPN2 P17655 VAR_014435 p.Asp22Glu LB/B rs25655 -
CAPN2 P17655 VAR_014436 p.Lys568Gln LB/B rs17599 -
CAPN2 P17655 VAR_021404 p.Ser68Gly LB/B rs2230083 -
CAPN2 P17655 VAR_021405 p.Lys476Arg LB/B rs9804140 -

CAPN2 P17655 VAR_021406 p.Glu521Gln LB/B rs28370127 -
CAPN2 P17655 VAR_021407 p.Lys677Gln LB/B rs2230082 -
CAPN3 P20807 VAR_001363 p.Leu182Gln LP/P - Muscular dystrophy, limb-girdle, autosomal recessive 1 (LGMDR1) [MIM:253600]
CAPN3 P20807 VAR_001365 p.Gly234Glu LP/P rs1555420634 Muscular dystrophy, limb-girdle, autosomal recessive 1 (LGMDR1) [MIM:253600]
CAPN3 P20807 VAR_001366 p.Val354Gly LP/P rs1555421271 Muscular dystrophy, limb-girdle, autosomal recessive 1 (LGMDR1) [MIM:253600]
CAPN3 P20807 VAR_001367 p.Arg490Trp LP/P rs141656719 Muscular dystrophy, limb-girdle, autosomal recessive 1 (LGMDR1) [MIM:253600]
CAPN3 P20807 VAR_001368 p.Arg572Gln LP/P rs121434544 Muscular dystrophy, limb-girdle, autosomal recessive 1 (LGMDR1) [MIM:253600]
CAPN3 P20807 VAR_001369 p.Ser744Gly LP/P rs750083132 Muscular dystrophy, limb-girdle, autosomal recessive 1 (LGMDR1) [MIM:253600]
CAPN3 P20807 VAR_001370 p.Arg769Gln LP/P rs80338802 Muscular dystrophy, limb-girdle, autosomal recessive 1 (LGMDR1) [MIM:253600]
CAPN3 P20807 VAR_009548 p.Val4Ile LP/P rs140660066 Muscular dystrophy, limb-girdle, autosomal recessive 1 (LGMDR1) [MIM:253600]
CAPN3 P20807 VAR_009549 p.Pro26Leu LP/P rs762020512 Muscular dystrophy, limb-girdle, autosomal recessive 1 (LGMDR1) [MIM:253600]
CAPN3 P20807 VAR_009550 p.Asp77Asn LP/P - Muscular dystrophy, limb-girdle, autosomal recessive 1 (LGMDR1) [MIM:253600]
CAPN3 P20807 VAR_009551 p.Ser86Phe LP/P rs121434546 Muscular dystrophy, limb-girdle, autosomal recessive 1 (LGMDR1) [MIM:253600]
CAPN3 P20807 VAR_009553 p.Glu107Lys LB/B rs1801505 -
CAPN3 P20807 VAR_009554 p.Arg118Gly LP/P rs1566973583 Muscular dystrophy, limb-girdle, autosomal recessive 1 (LGMDR1) [MIM:253600]
CAPN3 P20807 VAR_009555 p.Cys137Arg LP/P - Muscular dystrophy, limb-girdle, autosomal recessive 1 (LGMDR1) [MIM:253600]
CAPN3 P20807 VAR_009556 p.Ile162Leu LP/P - Muscular dystrophy, limb-girdle, autosomal recessive 1 (LGMDR1) [MIM:253600]
CAPN3 P20807 VAR_009557 p.Pro183Leu LP/P - Muscular dystrophy, limb-girdle, autosomal recessive 1 (LGMDR1) [MIM:253600]
CAPN3 P20807 VAR_009558 p.Thr184Met LB/B rs35889956 -
CAPN3 P20807 VAR_009559 p.Leu189Pro LP/P rs758795961 Muscular dystrophy, limb-girdle, autosomal recessive 1 (LGMDR1) [MIM:253600]
CAPN3 P20807 VAR_009560 p.Gly214Ser LP/P rs369784333 Muscular dystrophy, limb-girdle, autosomal recessive 1 (LGMDR1) [MIM:253600]
CAPN3 P20807 VAR_009561 p.Ser215Pro LP/P - Muscular dystrophy, limb-girdle, autosomal recessive 1 (LGMDR1) [MIM:253600]
CAPN3 P20807 VAR_009563 p.Glu217Lys LP/P rs773001194 Muscular dystrophy, limb-girdle, autosomal recessive 1 (LGMDR1) [MIM:253600]
CAPN3 P20807 VAR_009564 p.Gly222Arg LP/P rs1345121557 Muscular dystrophy, limb-girdle, autosomal recessive 1 (LGMDR1) [MIM:253600]
CAPN3 P20807 VAR_009565 p.Glu226Lys LP/P - Muscular dystrophy, limb-girdle, autosomal recessive 1 (LGMDR1) [MIM:253600]
CAPN3 P20807 VAR_009566 p.Thr232Ile LP/P - Muscular dystrophy, limb-girdle, autosomal recessive 1 (LGMDR1) [MIM:253600]
CAPN3 P20807 VAR_009567 p.Ala236Thr LB/B rs1801449 -

CAPN3 P20807 VAR_009569 p.Pro319Leu LP/P rs121434547 Muscular dystrophy, limb-girdle, autosomal recessive 1 (LGMDR1) [MIM:253600]
CAPN3 P20807 VAR_009570 p.His334Gln LP/P - Muscular dystrophy, limb-girdle, autosomal recessive 1 (LGMDR1) [MIM:253600]
CAPN3 P20807 VAR_009571 p.Tyr336Asn LP/P - Muscular dystrophy, limb-girdle, autosomal recessive 1 (LGMDR1) [MIM:253600]
CAPN3 P20807 VAR_009572 p.Trp360Cys LP/P rs267606703 Muscular dystrophy, limb-girdle, autosomal recessive 1 (LGMDR1) [MIM:253600]
CAPN3 P20807 VAR_009573 p.Arg437Cys LP/P rs777483913 Muscular dystrophy, limb-girdle, autosomal recessive 1 (LGMDR1) [MIM:253600]
CAPN3 P20807 VAR_009574 p.Arg440Trp LP/P rs777323132 Muscular dystrophy, limb-girdle, autosomal recessive 1 (LGMDR1) [MIM:253600]
CAPN3 P20807 VAR_009575 p.Gly441Asp LP/P - Muscular dystrophy, limb-girdle, autosomal recessive 1 (LGMDR1) [MIM:253600]
CAPN3 P20807 VAR_009576 p.Gly445Arg LP/P rs773827877 Muscular dystrophy, limb-girdle, autosomal recessive 1 (LGMDR1) [MIM:253600]
CAPN3 P20807 VAR_009577 p.Arg448Cys LP/P rs776043976 Muscular dystrophy, limb-girdle, autosomal recessive 1 (LGMDR1) [MIM:253600]
CAPN3 P20807 VAR_009578 p.Arg448Gly LP/P rs776043976 Muscular dystrophy, limb-girdle, autosomal recessive 1 (LGMDR1) [MIM:253600]
CAPN3 P20807 VAR_009579 p.Arg448His LP/P rs863224956 Muscular dystrophy, limb-girdle, autosomal recessive 1 (LGMDR1) [MIM:253600]
CAPN3 P20807 VAR_009580 p.Ser479Gly LP/P rs201736037 Muscular dystrophy, limb-girdle, autosomal recessive 1 (LGMDR1) [MIM:253600]
CAPN3 P20807 VAR_009581 p.Gln486Glu LP/P - Muscular dystrophy, limb-girdle, autosomal recessive 1 (LGMDR1) [MIM:253600]
CAPN3 P20807 VAR_009582 p.Arg489Gln LP/P rs147764579 Muscular dystrophy, limb-girdle, autosomal recessive 1 (LGMDR1) [MIM:253600]
CAPN3 P20807 VAR_009583 p.Arg489Trp LP/P rs863224957 Muscular dystrophy, limb-girdle, autosomal recessive 1 (LGMDR1) [MIM:253600]
CAPN3 P20807 VAR_009584 p.Arg490Gln LP/P rs121434548 Muscular dystrophy, limb-girdle, autosomal recessive 1 (LGMDR1) [MIM:253600]
CAPN3 P20807 VAR_009585 p.Arg493Trp LP/P rs557164942 Muscular dystrophy, limb-girdle, autosomal recessive 1 (LGMDR1) [MIM:253600]
CAPN3 P20807 VAR_009586 p.Gly496Arg LP/P rs761637940 Muscular dystrophy, limb-girdle, autosomal recessive 1 (LGMDR1) [MIM:253600]
CAPN3 P20807 VAR_009587 p.Ile502Thr LP/P rs148044781 Muscular dystrophy, limb-girdle, autosomal recessive 1 (LGMDR1) [MIM:253600]
CAPN3 P20807 VAR_009588 p.Arg541Gln LP/P rs398123143 Muscular dystrophy, limb-girdle, autosomal recessive 1 (LGMDR1) [MIM:253600]
CAPN3 P20807 VAR_009589 p.Gly567Trp LP/P rs727503839 Muscular dystrophy, limb-girdle, autosomal recessive 1 (LGMDR1) [MIM:253600]
CAPN3 P20807 VAR_009590 p.Arg572Trp LP/P rs863224959 Muscular dystrophy, limb-girdle, autosomal recessive 1 (LGMDR1) [MIM:253600]
CAPN3 P20807 VAR_009591 p.Ser606Leu LP/P rs199806879 Muscular dystrophy, limb-girdle, autosomal recessive 1 (LGMDR1) [MIM:253600]
CAPN3 P20807 VAR_009592 p.Gln638Pro LP/P - Muscular dystrophy, limb-girdle, autosomal recessive 1 (LGMDR1) [MIM:253600]

CAPN3 P20807 VAR_009593 p.Arg698Pro LP/P - Muscular dystrophy, limb-girdle, autosomal recessive 1 (LGMDR1) [MIM:253600]
CAPN3 P20807 VAR_009594 p.Ala702Val LP/P rs886042557 Muscular dystrophy, limb-girdle, autosomal recessive 1 (LGMDR1) [MIM:253600]
CAPN3 P20807 VAR_009595 p.Asp705Gly LP/P - Muscular dystrophy, limb-girdle, autosomal recessive 1 (LGMDR1) [MIM:253600]
CAPN3 P20807 VAR_009596 p.Asp705His LP/P - Muscular dystrophy, limb-girdle, autosomal recessive 1 (LGMDR1) [MIM:253600]
CAPN3 P20807 VAR_009597 p.Phe731Ser LP/P - Muscular dystrophy, limb-girdle, autosomal recessive 1 (LGMDR1) [MIM:253600]
CAPN3 P20807 VAR_009598 p.Arg748Gln LP/P rs587780290 Muscular dystrophy, limb-girdle, autosomal recessive 1 (LGMDR1) [MIM:253600]
CAPN3 P20807 VAR_009599 p.His774Asp US - Muscular dystrophy, limb-girdle, autosomal recessive 1 (LGMDR1) [MIM:253600]
CAPN3 P20807 VAR_009600 p.Ala798Glu US rs149095128 Muscular dystrophy, limb-girdle, autosomal recessive 1 (LGMDR1) [MIM:253600]
CAPN3 P20807 VAR_015389 p.Ala160Gly LB/B rs17592 -
CAPN3 P20807 VAR_022272 p.Gly21Glu LB/B rs28364364 -
CAPN3 P20807 VAR_047691 p.Glu622Ala LB/B rs11557723 -
CAPN5 O15484 VAR_069277 p.Arg243Leu LP/P rs397514601 Vitreoretinopathy, neovascular inflammatory (VRNI) [MIM:193235]
CAPN5 O15484 VAR_069278 p.Leu244Pro LP/P rs397514602 Vitreoretinopathy, neovascular inflammatory (VRNI) [MIM:193235]
CAPN6 Q9Y6Q1 VAR_021084 p.Val277Leu LB/B rs12013711 -
CAPN6 Q9Y6Q1 VAR_036048 p.Gly358Arg US - A colorectal cancer sample
CAPN6 Q9Y6Q1 VAR_051515 p.Thr518Ile LB/B rs12851517 -
CAPN9 O14815 VAR_022188 p.Ala102Val LB/B rs12562749 -
CAPN9 O14815 VAR_022189 p.Ser122Arg LB/B rs28359608 -
CAPN9 O14815 VAR_022190 p.Asp164Asn LB/B rs28359632 -
CAPN9 O14815 VAR_022191 p.Ile234Thr LB/B rs28359644 -
CAPN9 O14815 VAR_022192 p.Ala239Thr LB/B rs28359647 -
CAPN9 O14815 VAR_022193 p.Arg277Trp LB/B rs28359655 -
CAPN9 O14815 VAR_022194 p.Lys322Gln LB/B rs1933631 -
CAPN9 O14815 VAR_022195 p.His327Gln LB/B rs28359684 -
CAPN9 O14815 VAR_022196 p.Glu342Lys LB/B rs16852652 -
CAPN9 O14815 VAR_022197 p.Arg458Trp LB/B rs28359688 -
CAPN9 O14815 VAR_022198 p.Arg522Trp LB/B rs12731961 -
CAPN9 O14815 VAR_022199 p.Met611Ile LB/B rs16852683 -
CAPNS1 P04632 VAR_021089 p.Met224Val LB/B rs17878750 -
CAPRIN1 Q14444 VAR_042425 p.Ala263Asp LB/B rs1132973 -
CAPRIN1 Q14444 VAR_042426 p.Gln588His LB/B rs12282627 -
CAPRIN1 Q14444 VAR_042427 p.Arg616His LB/B rs11552285 -
CAPRIN2 Q6IMN6 VAR_034939 p.Met519Val LB/B rs2304630 -
CAPRIN2 Q6IMN6 VAR_048445 p.Pro114Ser LB/B rs17688567 -
CAPRIN2 Q6IMN6 VAR_048446 p.Lys237Arg LB/B rs12146709 -
CAPRIN2 Q6IMN6 VAR_048447 p.Ser655Leu LB/B rs2304628 -
CAPS Q13938 VAR_048638 p.Arg125Gly LB/B rs7249419 -
CAPS Q13938 VAR_080772 p.Arg180Gln US rs375700493 -
CAPS2 Q9BXY5 VAR_061085 p.Leu125Phe LB/B rs10879901 -

CAPSL Q8WWF8 VAR_029631 p.Arg85Gln LB/B rs1445898 -
CAPSL Q8WWF8 VAR_029632 p.Met201Val LB/B rs1345826 -
CAPZA1 P52907 VAR_073834 p.Ser219Leu LB/B rs555597264 -
CARD10 Q9BWT7 VAR_028116 p.Arg289Gln LB/B rs9610775 -
CARD11 Q9BXL7 VAR_028117 p.Thr670Met LB/B rs3735134 -
CARD11 Q9BXL7 VAR_028118 p.Ser694Leu LB/B rs3735133 -
CARD11 Q9BXL7 VAR_069710 p.Gly123Ser LP/P rs387907352 B-cell expansion with NFKB and T-cell anergy (BENTA) [MIM:616452]
CARD11 Q9BXL7 VAR_069711 p.Glu134Gly LP/P rs387907351 B-cell expansion with NFKB and T-cell anergy (BENTA) [MIM:616452]
CARD11 Q9BXL7 VAR_079284 p.Glu57Asp LP/P - Immunodeficiency 11B with atopic dermatitis (IMD11B) [MIM:617638]
CARD11 Q9BXL7 VAR_079285 p.Leu194Pro LP/P - Immunodeficiency 11B with atopic dermatitis (IMD11B) [MIM:617638]
CARD11 Q9BXL7 VAR_079286 p.Arg975Trp LP/P rs1064795307 Immunodeficiency 11B with atopic dermatitis (IMD11B) [MIM:617638]
CARD14 Q9BXL6 VAR_022043 p.Arg883His LB/B rs2289541 -
CARD14 Q9BXL6 VAR_024401 p.Arg547Ser LB/B rs2066964 -
CARD14 Q9BXL6 VAR_048608 p.Val585Ile LB/B rs34367357 -
CARD14 Q9BXL6 VAR_059196 p.Arg820Trp LB/B rs11652075 -
CARD14 Q9BXL6 VAR_061080 p.Arg962Gln LB/B rs34850974 -
CARD14 Q9BXL6 VAR_068222 p.Arg38Cys LB/B rs281875217 -
CARD14 Q9BXL6 VAR_068223 p.Arg62Gln LB/B rs115582620 -
CARD14 Q9BXL6 VAR_068224 p.Gly117Ser LP/P rs281875215 Pityriasis rubra pilaris (PRP) [MIM:173200]
CARD14 Q9BXL6 VAR_068224 p.Gly117Ser LP/P rs281875215 Psoriasis 2 (PSORS2) [MIM:602723]
CARD14 Q9BXL6 VAR_068225 p.Glu138Ala LP/P rs281875214 Psoriasis 2 (PSORS2) [MIM:602723]
CARD14 Q9BXL6 VAR_068226 p.Glu142Gly LP/P rs281875213 Psoriasis 2 (PSORS2) [MIM:602723]
CARD14 Q9BXL6 VAR_068227 p.Glu142Lys LP/P rs281875212 Psoriasis 2 (PSORS2) [MIM:602723]
CARD14 Q9BXL6 VAR_068228 p.Leu150Arg LP/P rs146214639 Psoriasis 2 (PSORS2) [MIM:602723]
CARD14 Q9BXL6 VAR_068229 p.His171Asn LB/B rs281875216 -
CARD14 Q9BXL6 VAR_068230 p.Asp176His LB/B rs144475004 -
CARD14 Q9BXL6 VAR_068231 p.Arg179His LB/B rs199517469 -
CARD14 Q9BXL6 VAR_068232 p.Val191Leu LB/B rs281875218 -
CARD14 Q9BXL6 VAR_068233 p.Ser200Asn LB/B rs114688446 -
CARD14 Q9BXL6 VAR_068234 p.Asp285Gly LB/B rs281875219 -
CARD14 Q9BXL6 VAR_068235 p.Ile593Asn LB/B rs281875220 -
CARD14 Q9BXL6 VAR_068236 p.Arg682Trp LB/B rs117918077 -
CARD14 Q9BXL6 VAR_068237 p.Gly714Ser LB/B rs151150961 -
CARD14 Q9BXL6 VAR_068238 p.Asp973Glu LB/B rs144285237 -
CARD14 Q9BXL6 VAR_068820 p.Leu156Pro LP/P rs387907240 Pityriasis rubra pilaris (PRP) [MIM:173200]
CARD14 Q9BXL6 VAR_078583 p.Arg69Trp LP/P rs375624435 Psoriasis 2 (PSORS2) [MIM:602723]
CARD14 Q9BXL6 VAR_078584 p.Cys127Ser LP/P - Pityriasis rubra pilaris (PRP) [MIM:173200]
CARD14 Q9BXL6 VAR_078585 p.Gln136Leu LP/P - Pityriasis rubra pilaris (PRP) [MIM:173200]
CARD14 Q9BXL6 VAR_078586 p.Arg151Gln US rs200731780 Psoriasis 2 (PSORS2) [MIM:602723]
CARD14 Q9BXL6 VAR_078587 p.Arg151Trp US rs777305616 Psoriasis 2 (PSORS2) [MIM:602723]
CARD14 Q9BXL6 VAR_078588 p.Glu197Lys LP/P rs200790561 Psoriasis 2 (PSORS2) [MIM:602723]
CARD14 Q9BXL6 VAR_078589 p.Leu209Pro US - Psoriasis 2 (PSORS2) [MIM:602723]
CARD14 Q9BXL6 VAR_078590 p.Ala216Thr US rs574982768 Psoriasis 2 (PSORS2) [MIM:602723]
CARD14 Q9BXL6 VAR_078591 p.Arg218Cys US rs747854314 -

CARD14 Q9BXL6 VAR_078592 p.Met338Val US rs200132496 -
CARD14 Q9BXL6 VAR_078593 p.Leu350Pro LB/B rs1412261979 -
CARD14 Q9BXL6 VAR_078594 p.Leu357Pro LB/B - -
CARD14 Q9BXL6 VAR_078595 p.Thr420Ala US rs762364495 Psoriasis 2 (PSORS2) [MIM:602723]
CARD14 Q9BXL6 VAR_078596 p.Ser602Leu US rs201285077 Psoriasis 2 (PSORS2) [MIM:602723]
CARD14 Q9BXL6 VAR_078597 p.Ala639Gly US - Psoriasis 2 (PSORS2) [MIM:602723]
CARD16 Q5EG05 VAR_046279 p.Arg33Ser LB/B rs35966314 -
CARD16 Q5EG05 VAR_046280 p.Gln37Lys LB/B rs1042744 -
CARD16 Q5EG05 VAR_046281 p.Ala56Asp LB/B rs34534919 -
CARD16 Q5EG05 VAR_046282 p.Asn167Ile LB/B rs542571 -
CARD6 Q9BX69 VAR_046689 p.Ser86Leu LB/B rs10512747 -
CARD6 Q9BX69 VAR_046690 p.Arg93Lys LB/B rs7715491 -
CARD6 Q9BX69 VAR_046691 p.Met395Lys LB/B rs3812030 -
CARD6 Q9BX69 VAR_046692 p.Asp426Val LB/B rs3812031 -
CARD6 Q9BX69 VAR_046693 p.Ala575Asp LB/B rs36085996 -
CARD6 Q9BX69 VAR_046694 p.Ile576Val LB/B rs323562 -
CARD8 Q9Y2G2 VAR_048606 p.Ile173Val LB/B rs11881179 -
CARD8 Q9Y2G2 VAR_061079 p.Glu204Ala LB/B rs59878320 -
CARD8 Q9Y2G2 VAR_084560 p.Val44Ile US - Inflammatory bowel disease 30 (IBD30) [MIM:619079]
CARD8 Q9Y2G2 VAR_084561 p.Phe102Ile LP/P rs2043211 Inflammatory bowel disease 30 (IBD30) [MIM:619079]
CARD9 Q9H257 VAR_048607 p.Ser12Asn LB/B rs4077515 -
CARD9 Q9H257 VAR_070828 p.Gly72Ser LP/P rs398122362 Candidiasis, familial, 2 (CANDF2) [MIM:212050]
CARD9 Q9H257 VAR_070829 p.Arg101Cys LP/P rs398122364 Candidiasis, familial, 2 (CANDF2) [MIM:212050]
CARD9 Q9H257 VAR_070830 p.Arg373Pro LP/P rs149712114 Candidiasis, familial, 2 (CANDF2) [MIM:212050]
CARD9 Q9H257 VAR_084630 p.Arg18Trp LP/P - Candidiasis, familial, 2 (CANDF2) [MIM:212050]
CARD9 Q9H257 VAR_084632 p.Arg35Gln LP/P - Candidiasis, familial, 2 (CANDF2) [MIM:212050]
CARD9 Q9H257 VAR_084633 p.Arg57His LP/P - Candidiasis, familial, 2 (CANDF2) [MIM:212050]
CARD9 Q9H257 VAR_084634 p.Arg70Trp LP/P - Candidiasis, familial, 2 (CANDF2) [MIM:212050]
CARD9 Q9H257 VAR_084635 p.Tyr91His LP/P - Candidiasis, familial, 2 (CANDF2) [MIM:212050]
CARD9 Q9H257 VAR_084636 p.Arg101Leu LP/P - Candidiasis, familial, 2 (CANDF2) [MIM:212050]
CARD9 Q9H257 VAR_084638 p.Lys196Glu LP/P - Candidiasis, familial, 2 (CANDF2) [MIM:212050]
CARD9 Q9H257 VAR_084642 p.Ala380Pro LP/P - Candidiasis, familial, 2 (CANDF2) [MIM:212050]
CARMIL1 Q5VZK9 VAR_039923 p.Val77Ile LB/B rs9358856 -
CARMIL1 Q5VZK9 VAR_039924 p.Pro545Leu LB/B rs12207840 -
CARMIL1 Q5VZK9 VAR_039925 p.Ala639Gly LB/B rs7454756 -
CARMIL1 Q5VZK9 VAR_039926 p.Asn1117Ser LB/B rs9885914 -
CARMIL2 Q6F5E8 VAR_081149 p.Arg50Thr LP/P rs1567626023 Immunodeficiency 58 (IMD58) [MIM:618131]
CARMIL2 Q6F5E8 VAR_081150 p.Leu372Arg LP/P rs1567628757 Immunodeficiency 58 (IMD58) [MIM:618131]
CARMIL2 Q6F5E8 VAR_081151 p.Leu525Gln LP/P rs1567629943 Immunodeficiency 58 (IMD58) [MIM:618131]
CARMIL2 Q6F5E8 VAR_081152 p.Leu639His LP/P rs775061512 Immunodeficiency 58 (IMD58) [MIM:618131]
CARMIL3 Q8ND23 VAR_039845 p.Leu1022Met LB/B rs10146906 -
CARNS1 A5YM72 VAR_042625 p.Pro14Thr LB/B rs868167 -
CARNS1 A5YM72 VAR_060320 p.Met498Leu LB/B rs17853668 -
CARS1 P49589 VAR_084305 p.Arg341His LP/P - Microcephaly, developmental delay, and brittle hair syndrome (MDBH) [MIM:618891]
CARS1 P49589 VAR_084306 p.Ser359Leu LP/P - Microcephaly, developmental delay, and brittle hair syndrome (MDBH) [MIM:618891]

CARS1 P49589 VAR_084308 p.Leu400Gln LP/P - Microcephaly, developmental delay, and brittle hair syndrome (MDBH) [MIM:618891]
CARS2 Q9HA77 VAR_034523 p.Glu440Lys LB/B rs965189 -
CARS2 Q9HA77 VAR_034524 p.Gln555Pro LB/B rs1043886 -
CARS2 Q9HA77 VAR_075669 p.Pro251Leu LP/P rs557671802 Combined oxidative phosphorylation deficiency 27 (COXPD27) [MIM:616672]
CARTPT Q16568 VAR_012199 p.Leu61Phe LB/B rs121909065 -
CARTPT Q16568 VAR_012200 p.Ser66Thr LB/B rs78242624 -
CARTPT Q16568 VAR_053022 p.Leu113Met LB/B rs12517689 -
CASC2 Q6XLA1 VAR_039469 p.Pro8Ser US - Some patients with endometrial cancer
CASD1 Q96PB1 VAR_035383 p.Arg386Ser LB/B rs17855797 -
CASK O14936 VAR_041956 p.Gly96Val US - A lung large cell carcinoma sample
CASK O14936 VAR_058719 p.Arg28Leu LP/P rs137852816 FG syndrome 4 (FGS4) [MIM:300422]
CASK O14936 VAR_062996 p.Tyr268His LP/P rs137852817 Mental retardation and microcephaly with pontine and cerebellar hypoplasia (MICPCH) [MIM:300749]
CASK O14936 VAR_062997 p.Pro396Ser LP/P rs137852820 Mental retardation and microcephaly with pontine and cerebellar hypoplasia (MICPCH) [MIM:300749]
CASK O14936 VAR_062998 p.Asp710Gly LP/P rs137852818 Mental retardation and microcephaly with pontine and cerebellar hypoplasia (MICPCH) [MIM:300749]
CASKIN2 Q8WXE0 VAR_060244 p.Glu891Gly LB/B rs7503373 -
CASP1 P29466 VAR_048615 p.Arg15His LB/B rs1042743 -
CASP10 Q92851 VAR_014071 p.Leu285Phe LP/P rs17860403 Autoimmune lymphoproliferative syndrome 2A (ALPS2A) [MIM:603909]
CASP10 Q92851 VAR_014072 p.Val410Ile LB/B rs13010627 -
CASP10 Q92851 VAR_037428 p.Met147Thr LP/P rs121909776 Gastric cancer (GASC) [MIM:613659]
CASP10 Q92851 VAR_037429 p.Ile406Leu LB/B rs80358239 -
CASP10 Q92851 VAR_037430 p.Ala414Val LP/P rs28936699 Familial non-Hodgkin lymphoma (NHL) [MIM:605027]
CASP10 Q92851 VAR_037431 p.Tyr446Cys LB/B rs17860405 -
CASP10 Q92851 VAR_055361 p.Ser239Cys LB/B rs41473647 -
CASP10 Q92851 VAR_055362 p.Pro444Ser LB/B rs41513147 -
CASP10 Q92851 VAR_065233 p.Lys14Thr US - -
CASP10 Q92851 VAR_065234 p.Arg21Cys LB/B rs559979934 -
CASP10 Q92851 VAR_065235 p.Leu285Pro US - -
CASP12 Q6UXS9 VAR_080638 p.Ile68Thr LB/B rs693001 -
CASP12 Q6UXS9 VAR_080640 p.Ser238Gly LB/B rs647039 -
CASP2 P42575 VAR_016334 p.Val172Leu LB/B rs4647297 -
CASP2 P42575 VAR_016335 p.Pro178Ala LB/B rs4647298 -
CASP2 P42575 VAR_016336 p.Arg441Gly LB/B rs4647338 -
CASP2 P42575 VAR_055621 p.Ala105Gly LB/B rs762263774 -
CASP3 P42574 VAR_001401 p.Glu190Asp LB/B rs1049210 -
CASP3 P42574 VAR_048616 p.His22Arg LB/B rs35578277 -
CASP4 P49662 VAR_061081 p.Asp47Asn LB/B rs56226603 -
CASP4 P49662 VAR_061082 p.Glu284Asp LB/B rs55901059 -
CASP4 P49662 VAR_075654 p.Arg134Cys LB/B rs181090259 -
CASP5 P51878 VAR_024403 p.Phe29Leu LB/B rs3181320 -
CASP5 P51878 VAR_024404 p.Arg168His LB/B rs3181179 -
CASP5 P51878 VAR_024405 p.Val217Leu LB/B rs3181326 -
CASP5 P51878 VAR_047216 p.Lys19Asn LB/B rs45483102 -

CASP5 P51878 VAR_047217 p.Leu26Trp LB/B rs1792778 -
CASP5 P51878 VAR_047218 p.Thr106Ala LB/B rs507879 -
CASP5 P51878 VAR_047219 p.Leu334Val LB/B rs523104 -
CASP5 P51878 VAR_047220 p.Glu353Lys LB/B rs45619739 -
CASP5 P51878 VAR_054480 p.Leu75Arg LB/B rs45585331 -
CASP5 P51878 VAR_054481 p.Arg298His LB/B rs45464699 -
CASP5 P51878 VAR_054482 p.Glu382Gln LB/B rs45458695 -
CASP6 P55212 VAR_016130 p.Ala109Thr LB/B rs5030674 -
CASP6 P55212 VAR_020126 p.Thr182Ser LB/B rs5030593 -
CASP6 P55212 VAR_029242 p.Glu35Lys LB/B rs11574697 -
CASP7 P55210 VAR_048617 p.Asp4Glu LB/B rs11555408 -
CASP7 P55210 VAR_048618 p.Asp255Glu LB/B rs2227310 -
CASP8 Q14790 VAR_014204 p.Arg248Trp LP/P rs17860424 Caspase-8 deficiency (CASP8D) [MIM:607271]
CASP8 Q14790 VAR_020127 p.Asp285His LB/B rs1045485 -
CASP8 Q14790 VAR_025816 p.Ser219Thr LB/B rs35976359 -
CASP8AP2 Q9UKL3 VAR_050700 p.Pro1659Ser LB/B rs3799896 -
CASP9 P55211 VAR_015415 p.Ala28Val LB/B rs1052571 -
CASP9 P55211 VAR_015416 p.Ser99Leu LB/B rs4646008 -
CASP9 P55211 VAR_015417 p.Thr102Ile LB/B rs2308941 -
CASP9 P55211 VAR_015418 p.Leu106Val LB/B rs2308938 -
CASP9 P55211 VAR_015419 p.Glu114Asp LB/B rs2020897 -
CASP9 P55211 VAR_015420 p.Arg173His LB/B rs2308950 -
CASP9 P55211 VAR_015421 p.Gln221Arg LB/B rs1052576 -
CASP9 P55211 VAR_016131 p.Gly176Arg LB/B rs2308949 -
CASP9 P55211 VAR_016132 p.Arg192Cys LB/B rs2308939 -
CASP9 P55211 VAR_022053 p.Ile185Met LB/B rs9282624 -
CASP9 P55211 VAR_059198 p.Phe136Leu LB/B rs1132312 -
CASQ1 P31415 VAR_053021 p.Tyr140Phe LB/B rs34489853 -
CASQ1 P31415 VAR_073337 p.Asp244Gly LP/P rs730882052 Myopathy, vacuolar, with CASQ1 aggregates (VMCQA) [MIM:616231]
CASQ1 P31415 VAR_079704 p.Asp44Asn LP/P rs140253806 Myopathy, tubular aggregate, 1 (TAM1) [MIM:160565]
CASQ1 P31415 VAR_079705 p.Gly103Asp LP/P - Myopathy, tubular aggregate, 1 (TAM1) [MIM:160565]
CASQ1 P31415 VAR_079706 p.Ile385Thr LP/P rs371278891 Myopathy, tubular aggregate, 1 (TAM1) [MIM:160565]
CASQ2 O14958 VAR_016075 p.Asp307His LP/P rs121434549 Ventricular tachycardia, catecholaminergic polymorphic, 2 (CPVT2) [MIM:611938]
CASQ2 O14958 VAR_023692 p.Thr66Ala LB/B rs4074536 -
CASQ2 O14958 VAR_023693 p.Val76Met LB/B rs10801999 -
CASQ2 O14958 VAR_044118 p.Leu167His LP/P rs121434550 Ventricular tachycardia, catecholaminergic polymorphic, 2 (CPVT2) [MIM:611938]
CASQ2 O14958 VAR_055234 p.Arg33Gln LP/P rs749547712 Ventricular tachycardia, catecholaminergic polymorphic, 2 (CPVT2) [MIM:611938]
CASQ2 O14958 VAR_067036 p.His244Arg LB/B rs28730716 -
CASQ2 O14958 VAR_067037 p.Asn335Lys LB/B rs28730712 -
CASQ2 O14958 VAR_076546 p.Lys180Arg LP/P rs886039816 Ventricular tachycardia, catecholaminergic polymorphic, 2 (CPVT2) [MIM:611938]
CASR P41180 VAR_003585 p.Pro39Ala LP/P rs121909262 Hypocalciuric hypercalcemia, familial 1 (HHC1) [MIM:145980]

CASR P41180 VAR_003586 p.Arg62Met LP/P rs121909265 Hypocalciuric hypercalcemia, familial 1 (HHC1) [MIM:145980]
CASR P41180 VAR_003587 p.Arg66Cys LP/P rs121909266 Hypocalciuric hypercalcemia, familial 1 (HHC1) [MIM:145980]
CASR P41180 VAR_003588 p.Ala116Thr LP/P rs104893691 Hypocalcemia, autosomal dominant 1 (HYPOC1) [MIM:601198]
CASR P41180 VAR_003589 p.Glu127Ala LP/P rs121909260 Hypocalcemia, autosomal dominant 1 (HYPOC1) [MIM:601198]
CASR P41180 VAR_003590 p.Thr138Met LP/P rs121909263 Hypocalciuric hypercalcemia, familial 1 (HHC1) [MIM:145980]
CASR P41180 VAR_003591 p.Gly143Glu LP/P rs121909264 Hypocalciuric hypercalcemia, familial 1 (HHC1) [MIM:145980]
CASR P41180 VAR_003592 p.Leu174Arg LP/P - Hypocalciuric hypercalcemia, familial 1 (HHC1) [MIM:145980]
CASR P41180 VAR_003593 p.Arg185Gln LP/P rs104893689 Hypocalciuric hypercalcemia, familial 1 (HHC1) [MIM:145980]
CASR P41180 VAR_003594 p.Arg227Leu LP/P rs28936684 Hyperparathyroidism, neonatal severe (NSHPT) [MIM:239200]
CASR P41180 VAR_003595 p.Arg227Gln LP/P rs28936684 Hypocalciuric hypercalcemia, familial 1 (HHC1) [MIM:145980]
CASR P41180 VAR_003596 p.Glu297Lys LP/P rs121909259 Hypocalciuric hypercalcemia, familial 1 (HHC1) [MIM:145980]
CASR P41180 VAR_003597 p.Cys582Tyr LP/P rs104893690 Hyperparathyroidism, neonatal severe (NSHPT) [MIM:239200]
CASR P41180 VAR_003597 p.Cys582Tyr LP/P rs104893690 Hypocalciuric hypercalcemia, familial 1 (HHC1) [MIM:145980]
CASR P41180 VAR_003598 p.Gln681His LP/P rs121909261 Hypocalcemia, autosomal dominant 1 (HYPOC1) [MIM:601198]
CASR P41180 VAR_003599 p.Arg795Trp LP/P rs121909258 Hypocalciuric hypercalcemia, familial 1 (HHC1) [MIM:145980]
CASR P41180 VAR_003600 p.Phe806Ser LP/P rs104893693 Hypocalcemia, autosomal dominant 1 (HYPOC1) [MIM:601198]
CASR P41180 VAR_003601 p.Cys851Ser LB/B rs200777304 -
CASR P41180 VAR_012649 p.Gly557Glu LP/P - Hypocalciuric hypercalcemia, familial 1 (HHC1) [MIM:145980]
CASR P41180 VAR_014450 p.Ala986Ser LB/B rs1801725 -
CASR P41180 VAR_014451 p.Glu1011Gln LB/B rs1801726 -
CASR P41180 VAR_015414 p.Leu616Val LP/P rs104893703 Hypocalcemia, autosomal dominant 1 (HYPOC1) [MIM:601198]
CASR P41180 VAR_020220 p.Pro951Thr LB/B rs4987051 -
CASR P41180 VAR_020221 p.Arg990Gly LB/B rs1042636 -
CASR P41180 VAR_021019 p.Glu767Lys LP/P - Hypocalcemia, autosomal dominant 1 (HYPOC1) [MIM:601198]
CASR P41180 VAR_058046 p.Leu11Ser LP/P rs200673016 Hypocalciuric hypercalcemia, familial 1 (HHC1) [MIM:145980]
CASR P41180 VAR_058047 p.Leu13Pro LP/P rs104893717 Hypocalciuric hypercalcemia, familial 1 (HHC1) [MIM:145980]
CASR P41180 VAR_058048 p.Thr14Ala LB/B rs199515839 -

CASR P41180 VAR_058049 p.Gly21Arg LP/P rs1064794290 Hypocalciuric hypercalcemia, familial 1 (HHC1) [MIM:145980]
CASR P41180 VAR_058050 p.Lys47Asn LP/P rs104893702 Hypocalcemia, autosomal dominant 1 (HYPOC1) [MIM:601198]
CASR P41180 VAR_058051 p.Asn118Lys LP/P rs104893695 Hypocalcemia, autosomal dominant 1 (HYPOC1) [MIM:601198]
CASR P41180 VAR_058052 p.Leu125Pro LP/P rs104893708 Hypocalcemia, autosomal dominant 1 (HYPOC1) [MIM:601198]
CASR P41180 VAR_058053 p.Phe128Leu LP/P rs104893696 Hypocalcemia, autosomal dominant 1 (HYPOC1) [MIM:601198]
CASR P41180 VAR_058054 p.Cys131Trp LP/P rs121909267 Hypocalcemia, autosomal dominant 1 (HYPOC1) [MIM:601198]
CASR P41180 VAR_058055 p.Thr151Met LP/P rs104893694 Hypocalcemia, autosomal dominant 1 (HYPOC1) [MIM:601198]
CASR P41180 VAR_058056 p.Ser171Asn LP/P - Hypocalciuric hypercalcemia, familial 1 (HHC1) [MIM:145980]
CASR P41180 VAR_058057 p.Phe180Cys LP/P rs121909268 Hypocalciuric hypercalcemia, familial 1 (HHC1) [MIM:145980]
CASR P41180 VAR_058058 p.Glu191Lys LP/P rs104893697 Hypocalcemia, autosomal dominant 1 (HYPOC1) [MIM:601198]
CASR P41180 VAR_058059 p.Pro221Gln LP/P rs397514728 Hypocalciuric hypercalcemia, familial 1 (HHC1) [MIM:145980]
CASR P41180 VAR_058060 p.Lys225Thr LP/P - Hypocalciuric hypercalcemia, familial 1 (HHC1) [MIM:145980]
CASR P41180 VAR_058061 p.Glu250Lys LB/B rs62269092 -
CASR P41180 VAR_058062 p.Ser271Phe LP/P - Hypocalciuric hypercalcemia, familial 1 (HHC1) [MIM:145980]
CASR P41180 VAR_058063 p.Gly397Arg LP/P rs1064794291 Hypocalciuric hypercalcemia, familial 1 (HHC1) [MIM:145980]
CASR P41180 VAR_058064 p.Arg465Gln LP/P rs104893716 Hypocalciuric hypercalcemia, familial 1 (HHC1) [MIM:145980]
CASR P41180 VAR_058065 p.Gly509Arg LP/P rs193922423 Hypocalciuric hypercalcemia, familial 1 (HHC1) [MIM:145980]
CASR P41180 VAR_058066 p.Gly553Arg LP/P rs104893719 Hypocalciuric hypercalcemia, familial 1 (HHC1) [MIM:145980]
CASR P41180 VAR_058067 p.Ile555Val LP/P rs777646067 Hypocalciuric hypercalcemia, familial 1 (HHC1) [MIM:145980]
CASR P41180 VAR_058068 p.Cys562Tyr LP/P - Hypocalciuric hypercalcemia, familial 1 (HHC1) [MIM:145980]
CASR P41180 VAR_058069 p.Cys582Phe LP/P rs104893690 Hypocalciuric hypercalcemia, familial 1 (HHC1) [MIM:145980]
CASR P41180 VAR_058070 p.Glu604Lys LP/P rs104893712 Hypocalcemia, autosomal dominant 1 (HYPOC1) [MIM:601198]
CASR P41180 VAR_058071 p.Phe612Ser LP/P rs104893698 Hypocalcemia, autosomal dominant 1 (HYPOC1) [MIM:601198]
CASR P41180 VAR_058072 p.Gly623Asp LP/P - Hypocalciuric hypercalcemia, familial 1 (HHC1) [MIM:145980]
CASR P41180 VAR_058073 p.Gly670Glu LP/P rs104893700 Hyperparathyroidism, neonatal severe (NSHPT) [MIM:239200]

CASR P41180 VAR_058074 p.Gly670Arg LP/P - Hypocalciuric hypercalcemia, familial 1 (HHC1) [MIM:145980]
CASR P41180 VAR_058075 p.Leu727Gln LP/P rs104893718 Hypocalcemia, autosomal dominant 1 (HYPOC1) [MIM:601198]
CASR P41180 VAR_058076 p.Val728Phe LP/P - Hypocalciuric hypercalcemia, familial 1 (HHC1) [MIM:145980]
CASR P41180 VAR_058077 p.Trp742Arg LP/P - Hypocalciuric hypercalcemia, familial 1 (HHC1) [MIM:145980]
CASR P41180 VAR_058078 p.Leu773Arg LP/P rs104893699 Hypocalcemia, autosomal dominant 1 (HYPOC1) [MIM:601198]
CASR P41180 VAR_058079 p.Phe788Cys LP/P rs104893701 Hypocalcemia, autosomal dominant 1 (HYPOC1) [MIM:601198]
CASR P41180 VAR_058080 p.Phe788Leu LP/P rs886041537 Hypocalcemia, autosomal dominant 1 (HYPOC1) [MIM:601198]
CASR P41180 VAR_058081 p.Ser820Phe LP/P rs104893710 Hypocalcemia, autosomal dominant 1 (HYPOC1) [MIM:601198]
CASR P41180 VAR_058082 p.Ala843Glu LP/P rs104893706 Hypocalcemia, autosomal dominant 1 (HYPOC1) [MIM:601198]
CASR P41180 VAR_058083 p.Phe881Leu LP/P rs104893704 -
CASR P41180 VAR_058084 p.Arg886Trp LP/P - Hypocalciuric hypercalcemia, familial 1 (HHC1) [MIM:145980]
CASR P41180 VAR_060206 p.Glu354Ala LP/P - Epilepsy, idiopathic generalized 8 (EIG8) [MIM:612899]
CASR P41180 VAR_060207 p.Ile686Val LP/P rs753013993 Epilepsy, idiopathic generalized 8 (EIG8) [MIM:612899]
CASR P41180 VAR_060208 p.Arg898Gln LP/P rs121909269 Epilepsy, idiopathic generalized 8 (EIG8) [MIM:612899]
CASR P41180 VAR_060209 p.Ala988Gly LP/P - Epilepsy, idiopathic generalized 8 (EIG8) [MIM:612899]
CASR P41180 VAR_060210 p.Ala988Val LP/P rs759027000 Epilepsy, idiopathic generalized 8 (EIG8) [MIM:612899]
CASR P41180 VAR_065198 p.Gln27Arg US - -
CASR P41180 VAR_065199 p.Thr100Ile LP/P - Hyperparathyroidism, neonatal severe (NSHPT) [MIM:239200]
CASR P41180 VAR_065201 p.Pro339Thr US - -
CASR P41180 VAR_065202 p.Leu650Pro LP/P - Hyperparathyroidism, neonatal severe (NSHPT) [MIM:239200]
CASR P41180 VAR_065203 p.Val689Met LP/P - Hyperparathyroidism, neonatal severe (NSHPT) [MIM:239200]
CASR P41180 VAR_065494 p.Val697Met LP/P - Hypocalciuric hypercalcemia, familial 1 (HHC1) [MIM:145980]
CASR P41180 VAR_078139 p.Phe42Ser LP/P rs1553765909 Hypocalciuric hypercalcemia, familial 1 (HHC1) [MIM:145980]
CASR P41180 VAR_078140 p.Ser53Pro LP/P - Hypocalciuric hypercalcemia, familial 1 (HHC1) [MIM:145980]
CASR P41180 VAR_078141 p.Pro55Leu LP/P rs886041154 Hypocalciuric hypercalcemia, familial 1 (HHC1) [MIM:145980]
CASR P41180 VAR_078142 p.Arg66His LP/P rs1276839362 Hypocalciuric hypercalcemia, familial 1 (HHC1) [MIM:145980]
CASR P41180 VAR_078143 p.Ile81Met LP/P - Hypocalciuric hypercalcemia, familial 1 (HHC1) [MIM:145980]

CASR P41180 VAR_078144 p.Ala110Thr LP/P - Hypocalciuric hypercalcemia, familial 1 (HHC1) [MIM:145980]
CASR P41180 VAR_078145 p.Ser122Cys LP/P - Hypocalcemia, autosomal dominant 1 (HYPOC1) [MIM:601198]
CASR P41180 VAR_078146 p.Leu125Phe LP/P - Hypocalcemia, autosomal dominant 1 (HYPOC1) [MIM:601198]
CASR P41180 VAR_078147 p.Cys129Arg LP/P - Hypocalcemia, autosomal dominant 1 (HYPOC1) [MIM:601198]
CASR P41180 VAR_078148 p.Pro136Leu LP/P - Hypocalcemia, autosomal dominant 1 (HYPOC1) [MIM:601198]
CASR P41180 VAR_078149 p.Gly143Arg LP/P rs769256610 Hypocalciuric hypercalcemia, familial 1 (HHC1) [MIM:145980]
CASR P41180 VAR_078150 p.Gly158Arg LP/P - Hypocalciuric hypercalcemia, familial 1 (HHC1) [MIM:145980]
CASR P41180 VAR_078151 p.Leu159Pro LP/P - Hypocalciuric hypercalcemia, familial 1 (HHC1) [MIM:145980]
CASR P41180 VAR_078152 p.Ser166Gly LP/P rs193922441 Hypocalciuric hypercalcemia, familial 1 (HHC1) [MIM:145980]
CASR P41180 VAR_078153 p.Arg172Gly LP/P rs201851934 Hypocalciuric hypercalcemia, familial 1 (HHC1) [MIM:145980]
CASR P41180 VAR_078154 p.Asn178Asp LP/P rs1060502855 Hypocalciuric hypercalcemia, familial 1 (HHC1) [MIM:145980]
CASR P41180 VAR_078155 p.Asp215Gly LP/P - Hypocalciuric hypercalcemia, familial 1 (HHC1) [MIM:145980]
CASR P41180 VAR_078156 p.Arg220Trp LP/P rs1482119762 Hypocalciuric hypercalcemia, familial 1 (HHC1) [MIM:145980]
CASR P41180 VAR_078157 p.Pro221Leu LP/P rs397514728 Hypocalcemia, autosomal dominant 1 (HYPOC1) [MIM:601198]
CASR P41180 VAR_078158 p.Pro221Ser LP/P - Hypocalciuric hypercalcemia, familial 1 (HHC1) [MIM:145980]
CASR P41180 VAR_078159 p.Glu228Lys LP/P - Hypocalcemia, autosomal dominant 1 (HYPOC1) [MIM:601198]
CASR P41180 VAR_078160 p.Gln459Arg LP/P - Hypocalciuric hypercalcemia, familial 1 (HHC1) [MIM:145980]
CASR P41180 VAR_078161 p.Gly549Arg LP/P - Hypocalciuric hypercalcemia, familial 1 (HHC1) [MIM:145980]
CASR P41180 VAR_078162 p.Thr550Ile LP/P - Hypocalciuric hypercalcemia, familial 1 (HHC1) [MIM:145980]
CASR P41180 VAR_078163 p.Arg551Lys LP/P rs1060502861 Hyperparathyroidism, neonatal severe (NSHPT) [MIM:239200]
CASR P41180 VAR_078164 p.Cys565Gly LP/P - Hypocalciuric hypercalcemia, familial 1 (HHC1) [MIM:145980]
CASR P41180 VAR_078165 p.Pro569His LP/P - Hypocalcemia, autosomal dominant 1 (HYPOC1) [MIM:601198]
CASR P41180 VAR_078166 p.Gly571Trp LP/P - Hypocalciuric hypercalcemia, familial 1 (HHC1) [MIM:145980]
CASR P41180 VAR_078168 p.Ser657Tyr LP/P - Hypocalciuric hypercalcemia, familial 1 (HHC1) [MIM:145980]

CASR P41180 VAR_078169 p.Cys661Tyr LP/P - Hypocalciuric hypercalcemia, familial 1 (HHC1) [MIM:145980]
CASR P41180 VAR_078170 p.Arg680His LP/P rs773146939 Hypocalciuric hypercalcemia, familial 1 (HHC1) [MIM:145980]
CASR P41180 VAR_078171 p.Gln681Arg LP/P - Hypocalcemia, autosomal dominant 1 (HYPOC1) [MIM:601198]
CASR P41180 VAR_078172 p.Glu707Val LP/P - Hypocalciuric hypercalcemia, familial 1 (HHC1) [MIM:145980]
CASR P41180 VAR_078173 p.Pro748Arg LP/P - Hypocalciuric hypercalcemia, familial 1 (HHC1) [MIM:145980]
CASR P41180 VAR_078175 p.Gly774Ser LP/P - Hypocalciuric hypercalcemia, familial 1 (HHC1) [MIM:145980]
CASR P41180 VAR_078176 p.Asn802Ile LP/P - Hypocalcemia, autosomal dominant 1 (HYPOC1) [MIM:601198]
CASR P41180 VAR_078177 p.Asn802Ser LP/P rs140022350 Hypocalciuric hypercalcemia, familial 1 (HHC1) [MIM:145980]
CASR P41180 VAR_078178 p.Val817Ile LP/P rs1057518933 Hypocalciuric hypercalcemia, familial 1 (HHC1) [MIM:145980]
CASR P41180 VAR_078179 p.Gly830Ser LP/P - Hypocalcemia, autosomal dominant 1 (HYPOC1) [MIM:601198]
CASR P41180 VAR_078180 p.Phe832Leu LP/P - Hypocalcemia, autosomal dominant 1 (HYPOC1) [MIM:601198]
CASR P41180 VAR_078181 p.Phe832Ser LP/P - Hypocalcemia, autosomal dominant 1 (HYPOC1) [MIM:601198]
CASR P41180 VAR_078182 p.Ile839Thr LP/P - Hypocalcemia, autosomal dominant 1 (HYPOC1) [MIM:601198]
CASR P41180 VAR_078183 p.Thr972Met LP/P rs200620134 Hypocalciuric hypercalcemia, familial 1 (HHC1) [MIM:145980]
CASS4 Q9NQ75 VAR_054084 p.Arg491Lys LB/B rs16979936 -
CASS4 Q9NQ75 VAR_054085 p.Thr629Asn LB/B rs6069755 -
CASS4 Q9NQ75 VAR_054086 p.Pro660Ser LB/B rs35031530 -
CASS4 Q9NQ75 VAR_054087 p.Gln780His LB/B rs7272702 -
CAST P20810 VAR_005298 p.Glu592Gly LB/B - -
CAST P20810 VAR_022686 p.Cys408Ser LB/B rs754615 -
CAST P20810 VAR_030741 p.Glu380Lys LB/B rs1643702 -
CAST P20810 VAR_030742 p.Ala537Val LB/B rs4948 -
CASZ1 Q86V15 VAR_077466 p.Leu38Pro LP/P - -
CATSPER1 Q8NEC5 VAR_033304 p.Gly133Ser LB/B rs1203998 -
CATSPER1 Q8NEC5 VAR_033305 p.Val652Ile LB/B rs3814747 -
CATSPER1 Q8NEC5 VAR_033306 p.Thr730Pro LB/B rs34958219 -
CATSPER2 Q96P56 VAR_033307 p.Glu8Gly LB/B rs2614835 -
CATSPER2 Q96P56 VAR_033308 p.Val57Ile LB/B rs8042868 -
CATSPER3 Q86XQ3 VAR_033309 p.Asn204Lys LB/B rs3896260 -
CATSPER4 Q7RTX7 VAR_033310 p.Gln77Arg LB/B rs11247866 -
CATSPER4 Q7RTX7 VAR_033311 p.Leu124Phe LB/B rs12138368 -
CATSPER4 Q7RTX7 VAR_033312 p.Ile293Val LB/B rs17257155 -
CATSPER4 Q7RTX7 VAR_033313 p.Asp436Asn LB/B rs6657616 -
CATSPERB Q9H7T0 VAR_061634 p.Phe318Tyr LB/B rs57706558 -
CATSPERD Q86XM0 VAR_032270 p.Thr504Ala LB/B rs17854252 -

CATSPERD Q86XM0 VAR_032271 p.Thr743Ser LB/B rs2305925 -
CATSPERD Q86XM0 VAR_061714 p.Met212Thr LB/B rs57680462 -
CATSPERE Q5SY80 VAR_027661 p.Thr66Lys LB/B rs11586356 -
CATSPERE Q5SY80 VAR_035494 p.Thr653Ile US - A breast cancer sample
CATSPERE Q5SY80 VAR_061566 p.Thr56Ser LB/B rs58602830 -
CATSPERG Q6ZRH7 VAR_059631 p.Leu29Pro LB/B rs2302182 -
CATSPERG Q6ZRH7 VAR_059632 p.Ser650Arg LB/B rs3745953 -
CATSPERG Q6ZRH7 VAR_059633 p.Met1137Thr LB/B rs2286550 -
CATSPERZ Q9NTU4 VAR_046370 p.Pro68Leu LB/B rs2286614 -
CAV1 Q03135 VAR_015103 p.Pro132Leu US rs1213469537 Breast cancer
CAV2 P51636 VAR_012071 p.Gln130Glu LB/B rs8940 -
CAV3 P56539 VAR_001403 p.Pro105Leu LP/P rs116840805 Rippling muscle disease 2 (RMD2) [MIM:606072]
CAV3 P56539 VAR_010742 p.Val57Met LP/P rs116840795 HyperCKmia (HYPCK) [MIM:123320]
CAV3 P56539 VAR_010743 p.Cys72Trp LB/B rs116840776 -
CAV3 P56539 VAR_011512 p.Arg27Gln LP/P rs116840778 HyperCKmia (HYPCK) [MIM:123320]
CAV3 P56539 VAR_011512 p.Arg27Gln LP/P rs116840778 Myopathy, distal, Tateyama type (MPDT) [MIM:614321]
CAV3 P56539 VAR_011512 p.Arg27Gln LP/P rs116840778 Rippling muscle disease 2 (RMD2) [MIM:606072]
CAV3 P56539 VAR_011513 p.Ala46Thr LP/P rs116840789 Rippling muscle disease 2 (RMD2) [MIM:606072]
CAV3 P56539 VAR_011514 p.Ala46Val LP/P rs116840773 Rippling muscle disease 2 (RMD2) [MIM:606072]
CAV3 P56539 VAR_015374 p.Asp28Glu LP/P rs116840782 Rippling muscle disease 2 (RMD2) [MIM:606072]
CAV3 P56539 VAR_016207 p.Leu87Pro LP/P rs28936685 Rippling muscle disease 2 (RMD2) [MIM:606072]
CAV3 P56539 VAR_016208 p.Ala93Thr LP/P rs28936686 Rippling muscle disease 2 (RMD2) [MIM:606072]
CAV3 P56539 VAR_021016 p.Asn33Lys LP/P rs1008642 Myopathy, distal, Tateyama type (MPDT) [MIM:614321]
CAV3 P56539 VAR_021016 p.Asn33Lys LP/P rs1008642 Rippling muscle disease 2 (RMD2) [MIM:606072]
CAV3 P56539 VAR_021017 p.Val44Glu LP/P rs116840788 Rippling muscle disease 2 (RMD2) [MIM:606072]
CAV3 P56539 VAR_021018 p.Thr64Pro LP/P rs199476332 Rippling muscle disease 2 (RMD2) [MIM:606072]
CAV3 P56539 VAR_026696 p.Ser61Arg LB/B rs116840796 -
CAV3 P56539 VAR_029540 p.Pro29Leu LP/P rs116840786 HyperCKmia (HYPCK) [MIM:123320]
CAV3 P56539 VAR_029541 p.Ser53Gly LP/P rs116840794 Rippling muscle disease 2 (RMD2) [MIM:606072]
CAV3 P56539 VAR_029542 p.Gly56Ser LB/B rs72546667 -
CAV3 P56539 VAR_029543 p.Thr64Ser LP/P rs121909280 Cardiomyopathy, familial hypertrophic (CMH) [MIM:192600]
CAV3 P56539 VAR_029545 p.Arg126His LB/B rs116840777 -
CAV3 P56539 VAR_043694 p.Val14Leu LP/P rs121909281 Sudden infant death syndrome (SIDS) [MIM:272120]
CAV3 P56539 VAR_043695 p.Thr78Met LP/P rs72546668 Long QT syndrome 9 (LQT9) [MIM:611818]
CAV3 P56539 VAR_043695 p.Thr78Met LP/P rs72546668 Sudden infant death syndrome (SIDS) [MIM:272120]
CAV3 P56539 VAR_043696 p.Leu79Arg LP/P rs121909282 Long QT syndrome 9 (LQT9) [MIM:611818]
CAV3 P56539 VAR_043696 p.Leu79Arg LP/P rs121909282 Sudden infant death syndrome (SIDS) [MIM:272120]
CAV3 P56539 VAR_043697 p.Ala85Thr LP/P rs104893715 Long QT syndrome 9 (LQT9) [MIM:611818]
CAV3 P56539 VAR_043698 p.Phe97Cys LP/P rs104893714 Long QT syndrome 9 (LQT9) [MIM:611818]
CAV3 P56539 VAR_043699 p.Ser141Arg LP/P rs104893713 Long QT syndrome 9 (LQT9) [MIM:611818]
CAVIN1 Q6NZI2 VAR_034416 p.Glu193Gln LB/B rs35308568 -
CAVIN1 Q6NZI2 VAR_035982 p.Pro14Thr US - A breast cancer sample
CAVIN2 O95810 VAR_034422 p.Glu130Asp LB/B rs35012125 -

CAVIN3 Q969G5 VAR_042851 p.Arg8Pro LB/B rs2682123 -
CAVIN3 Q969G5 VAR_042852 p.Ala104Thr LB/B rs10839551 -
CAVIN3 Q969G5 VAR_042853 p.Leu158Pro LB/B rs1051992 -
CAVIN3 Q969G5 VAR_042854 p.Leu255Phe LB/B rs12294600 -
CBFA2T3 O75081 VAR_035374 p.Glu429Gly LB/B rs1053526 -
CBFA2T3 O75081 VAR_035447 p.Arg306His US rs745972870 A colorectal cancer sample
CBFA2T3 O75081 VAR_035448 p.Glu518Lys US rs774310781 A colorectal cancer sample
CBFA2T3 O75081 VAR_035449 p.Ala534Val US rs553618592 A colorectal cancer sample
CBFB Q13951 VAR_036226 p.Pro100Ala US rs1280921900 A breast cancer sample
CBL P22681 VAR_057211 p.Leu620Phe LB/B rs2227988 -
CBL P22681 VAR_057212 p.Pro782Leu LB/B rs2229073 -
CBL P22681 VAR_057213 p.Val904Ile LB/B rs17122769 -
CBL P22681 VAR_064332 p.Gln367Pro LP/P rs267606704 Noonan syndrome-like disorder with or without juvenile myelomonocytic leukemia (NSLL) [MIM:613563]
CBL P22681 VAR_064333 p.Lys382Glu LP/P rs267606705 Noonan syndrome-like disorder with or without juvenile myelomonocytic leukemia (NSLL) [MIM:613563]
CBL P22681 VAR_064334 p.Asp390Tyr LP/P rs267606707 Noonan syndrome-like disorder with or without juvenile myelomonocytic leukemia (NSLL) [MIM:613563]
CBL P22681 VAR_064335 p.Arg420Gln LP/P rs267606708 Noonan syndrome-like disorder with or without juvenile myelomonocytic leukemia (NSLL) [MIM:613563]
CBL P22681 VAR_071040 p.Lys287Arg US - -
CBL P22681 VAR_071042 p.Tyr371His US rs267606706 -
CBL P22681 VAR_071043 p.Arg499Leu US - -
CBLB Q13191 VAR_025303 p.Arg584Lys LB/B rs17853100 -
CBLB Q13191 VAR_039241 p.Asn883Asp LB/B rs35835913 -
CBLC Q9ULV8 VAR_018298 p.His405Tyr LB/B rs3208856 -
CBLIF P27352 VAR_022742 p.Gln23Arg LB/B rs35211634 -
CBLIF P27352 VAR_022743 p.Ser46Leu LP/P rs121434322 Hereditary intrinsic factor deficiency (IFD) [MIM:261000]
CBLIF P27352 VAR_022744 p.Asn255Ser LB/B rs35867471 -
CBLIF P27352 VAR_048753 p.Gly65Arg LB/B rs11825834 -
CBLL2 Q8N7E2 VAR_030340 p.Asp166Glu LB/B rs5951426 -
CBLL2 Q8N7E2 VAR_030341 p.Ser287Phe LB/B rs12860105 -
CBR1 P16152 VAR_031706 p.Pro131Ser LB/B rs41557318 -
CBR1 P16152 VAR_059053 p.Val88Ile LB/B rs1143663 -
CBR3 O75828 VAR_033868 p.Cys4Tyr LB/B rs8133052 -
CBR3 O75828 VAR_033869 p.Leu84Val LB/B rs9282628 -
CBR3 O75828 VAR_033870 p.Val93Ile LB/B rs2835285 -
CBR3 O75828 VAR_033871 p.Pro131Ser LB/B rs16993929 -
CBR3 O75828 VAR_033872 p.Met235Leu LB/B rs4987121 -
CBR3 O75828 VAR_033873 p.Val244Met LB/B rs1056892 -
CBR4 Q8N4T8 VAR_039049 p.Leu70Met LB/B rs2877380 -
CBS P35520 VAR_002171 p.Pro78Arg LP/P rs786204608 Cystathionine beta-synthase deficiency (CBSD) [MIM:236200]
CBS P35520 VAR_002172 p.Pro88Ser LP/P - Cystathionine beta-synthase deficiency (CBSD) [MIM:236200]
CBS P35520 VAR_002173 p.Lys102Asn LP/P rs786204609 Cystathionine beta-synthase deficiency (CBSD) [MIM:236200]

CBS P35520 VAR_002174 p.Ala114Val LP/P rs121964964 Cystathionine beta-synthase deficiency (CBSD) [MIM:236200]
CBS P35520 VAR_002175 p.Arg125Gln LP/P rs781444670 Cystathionine beta-synthase deficiency (CBSD) [MIM:236200]
CBS P35520 VAR_002176 p.Glu131Asp LP/P rs1555875351 Cystathionine beta-synthase deficiency (CBSD) [MIM:236200]
CBS P35520 VAR_002177 p.Glu144Lys LP/P rs121964966 Cystathionine beta-synthase deficiency (CBSD) [MIM:236200]
CBS P35520 VAR_002178 p.Pro145Leu LP/P rs121964963 Cystathionine beta-synthase deficiency (CBSD) [MIM:236200]
CBS P35520 VAR_002179 p.Cys165Tyr LP/P rs1347651454 Cystathionine beta-synthase deficiency (CBSD) [MIM:236200]
CBS P35520 VAR_002180 p.Val168Met LP/P rs121964970 Cystathionine beta-synthase deficiency (CBSD) [MIM:236200]
CBS P35520 VAR_002181 p.Arg224His LP/P rs761647392 Cystathionine beta-synthase deficiency (CBSD) [MIM:236200]
CBS P35520 VAR_002182 p.Glu239Lys LP/P - Cystathionine beta-synthase deficiency (CBSD) [MIM:236200]
CBS P35520 VAR_002183 p.Thr257Met LP/P rs758236584 Cystathionine beta-synthase deficiency (CBSD) [MIM:236200]
CBS P35520 VAR_002184 p.Ile278Thr LP/P rs5742905 Cystathionine beta-synthase deficiency (CBSD) [MIM:236200]
CBS P35520 VAR_002185 p.Pro290Leu LP/P rs760912339 Cystathionine beta-synthase deficiency (CBSD) [MIM:236200]
CBS P35520 VAR_002186 p.Gly307Ser LP/P rs121964962 Cystathionine beta-synthase deficiency (CBSD) [MIM:236200]
CBS P35520 VAR_002187 p.Ala331Val LP/P rs777919630 Cystathionine beta-synthase deficiency (CBSD) [MIM:236200]
CBS P35520 VAR_002188 p.Arg336Cys LP/P rs398123151 Cystathionine beta-synthase deficiency (CBSD) [MIM:236200]
CBS P35520 VAR_002189 p.Arg369His LP/P rs11700812 Cystathionine beta-synthase deficiency (CBSD) [MIM:236200]
CBS P35520 VAR_002190 p.Val371Met LP/P rs372010465 Cystathionine beta-synthase deficiency (CBSD) [MIM:236200]
CBS P35520 VAR_002191 p.Lys384Glu LP/P rs121964967 Cystathionine beta-synthase deficiency (CBSD) [MIM:236200]
CBS P35520 VAR_002192 p.Asp444Asn LP/P rs28934891 Cystathionine beta-synthase deficiency (CBSD) [MIM:236200]
CBS P35520 VAR_002193 p.Val454Glu LP/P - Cystathionine beta-synthase deficiency (CBSD) [MIM:236200]
CBS P35520 VAR_002194 p.Leu539Ser LP/P rs121964968 Cystathionine beta-synthase deficiency (CBSD) [MIM:236200]
CBS P35520 VAR_008049 p.Pro49Leu LP/P rs148865119 Cystathionine beta-synthase deficiency (CBSD) [MIM:236200]
CBS P35520 VAR_008050 p.Arg58Trp LP/P rs555959266 Cystathionine beta-synthase deficiency (CBSD) [MIM:236200]
CBS P35520 VAR_008051 p.Gly85Arg LP/P rs863223435 Cystathionine beta-synthase deficiency (CBSD) [MIM:236200]
CBS P35520 VAR_008052 p.Lys102Gln LB/B rs34040148 -

CBS P35520 VAR_008053 p.Gly116Arg LP/P rs760214620 Cystathionine beta-synthase deficiency (CBSD) [MIM:236200]
CBS P35520 VAR_008054 p.Arg121Cys LP/P rs775992753 Cystathionine beta-synthase deficiency (CBSD) [MIM:236200]
CBS P35520 VAR_008055 p.Arg121His LP/P rs770095972 Cystathionine beta-synthase deficiency (CBSD) [MIM:236200]
CBS P35520 VAR_008056 p.Arg121Leu LP/P rs770095972 Cystathionine beta-synthase deficiency (CBSD) [MIM:236200]
CBS P35520 VAR_008057 p.Arg125Trp LP/P rs886057100 Cystathionine beta-synthase deficiency (CBSD) [MIM:236200]
CBS P35520 VAR_008058 p.Met126Val LP/P - Cystathionine beta-synthase deficiency (CBSD) [MIM:236200]
CBS P35520 VAR_008059 p.Glu128Asp LP/P rs374593242 Cystathionine beta-synthase deficiency (CBSD) [MIM:236200]
CBS P35520 VAR_008060 p.Gly139Arg LP/P rs121964965 Cystathionine beta-synthase deficiency (CBSD) [MIM:236200]
CBS P35520 VAR_008061 p.Gly148Arg LP/P rs755952006 Cystathionine beta-synthase deficiency (CBSD) [MIM:236200]
CBS P35520 VAR_008062 p.Gly151Arg LP/P rs373782713 Cystathionine beta-synthase deficiency (CBSD) [MIM:236200]
CBS P35520 VAR_008064 p.Ile152Met LP/P - Cystathionine beta-synthase deficiency (CBSD) [MIM:236200]
CBS P35520 VAR_008065 p.Ala155Thr LP/P rs1429138569 Cystathionine beta-synthase deficiency (CBSD) [MIM:236200]
CBS P35520 VAR_008066 p.Glu176Lys LP/P rs762065361 Cystathionine beta-synthase deficiency (CBSD) [MIM:236200]
CBS P35520 VAR_008067 p.Val180Ala LP/P rs1555875010 Cystathionine beta-synthase deficiency (CBSD) [MIM:236200]
CBS P35520 VAR_008068 p.Thr191Met LP/P rs121964973 Cystathionine beta-synthase deficiency (CBSD) [MIM:236200]
CBS P35520 VAR_008069 p.Asp198Val LP/P - Cystathionine beta-synthase deficiency (CBSD) [MIM:236200]
CBS P35520 VAR_008070 p.Ala226Thr LP/P rs763835246 Cystathionine beta-synthase deficiency (CBSD) [MIM:236200]
CBS P35520 VAR_008071 p.Asp234Asn LP/P rs773734233 Cystathionine beta-synthase deficiency (CBSD) [MIM:236200]
CBS P35520 VAR_008072 p.Thr262Met LP/P rs149119723 Cystathionine beta-synthase deficiency (CBSD) [MIM:236200]
CBS P35520 VAR_008073 p.Arg266Gly LP/P - Cystathionine beta-synthase deficiency (CBSD) [MIM:236200]
CBS P35520 VAR_008074 p.Arg266Lys LP/P rs121964969 Cystathionine beta-synthase deficiency (CBSD) [MIM:236200]
CBS P35520 VAR_008076 p.Glu302Lys LP/P rs779270933 Cystathionine beta-synthase deficiency (CBSD) [MIM:236200]
CBS P35520 VAR_008077 p.Gly305Arg LP/P - Cystathionine beta-synthase deficiency (CBSD) [MIM:236200]
CBS P35520 VAR_008078 p.Val320Ala LP/P rs781567152 Cystathionine beta-synthase deficiency (CBSD) [MIM:236200]

CBS P35520 VAR_008079 p.Ala331Glu LP/P rs777919630 Cystathionine beta-synthase deficiency (CBSD) [MIM:236200]
CBS P35520 VAR_008080 p.Arg336His LP/P rs760417941 Cystathionine beta-synthase deficiency (CBSD) [MIM:236200]
CBS P35520 VAR_008081 p.Ser352Asn LP/P - Cystathionine beta-synthase deficiency (CBSD) [MIM:236200]
CBS P35520 VAR_008082 p.Thr353Met LP/P rs121964972 Cystathionine beta-synthase deficiency (CBSD) [MIM:236200]
CBS P35520 VAR_008083 p.Val354Met LP/P rs267606146 Cystathionine beta-synthase deficiency (CBSD) [MIM:236200]
CBS P35520 VAR_008084 p.Arg369Cys LP/P rs117687681 Cystathionine beta-synthase deficiency (CBSD) [MIM:236200]
CBS P35520 VAR_008085 p.Cys370Tyr LP/P rs757920190 Cystathionine beta-synthase deficiency (CBSD) [MIM:236200]
CBS P35520 VAR_008086 p.Lys384Asn LP/P - Cystathionine beta-synthase deficiency (CBSD) [MIM:236200]
CBS P35520 VAR_008087 p.Met391Ile LP/P - Cystathionine beta-synthase deficiency (CBSD) [MIM:236200]
CBS P35520 VAR_008088 p.Thr434Asn LP/P rs1555872506 Cystathionine beta-synthase deficiency (CBSD) [MIM:236200]
CBS P35520 VAR_008089 p.Ile435Thr LP/P - Cystathionine beta-synthase deficiency (CBSD) [MIM:236200]
CBS P35520 VAR_008090 p.Arg439Gln LP/P rs756467921 Cystathionine beta-synthase deficiency (CBSD) [MIM:236200]
CBS P35520 VAR_008091 p.Ser466Leu LP/P rs121964971 Cystathionine beta-synthase deficiency (CBSD) [MIM:236200]
CBS P35520 VAR_008092 p.Arg491Cys LP/P rs1339830457 Cystathionine beta-synthase deficiency (CBSD) [MIM:236200]
CBS P35520 VAR_008093 p.Val534Asp LP/P - Cystathionine beta-synthase deficiency (CBSD) [MIM:236200]
CBS P35520 VAR_021790 p.His65Arg LP/P rs1191141364 Cystathionine beta-synthase deficiency (CBSD) [MIM:236200]
CBS P35520 VAR_021791 p.Leu101Pro LP/P rs786204757 Cystathionine beta-synthase deficiency (CBSD) [MIM:236200]
CBS P35520 VAR_021792 p.Cys109Arg LP/P rs778220779 Cystathionine beta-synthase deficiency (CBSD) [MIM:236200]
CBS P35520 VAR_021793 p.Ile143Met LP/P rs370167302 Cystathionine beta-synthase deficiency (CBSD) [MIM:236200]
CBS P35520 VAR_021794 p.Asn228Lys LP/P rs1464223176 Cystathionine beta-synthase deficiency (CBSD) [MIM:236200]
CBS P35520 VAR_021795 p.Thr262Arg LP/P - Cystathionine beta-synthase deficiency (CBSD) [MIM:236200]
CBS P35520 VAR_021796 p.Cys275Tyr LP/P - Cystathionine beta-synthase deficiency (CBSD) [MIM:236200]
CBS P35520 VAR_021797 p.Leu338Pro LP/P - Cystathionine beta-synthase deficiency (CBSD) [MIM:236200]
CBS P35520 VAR_021798 p.Gly347Ser LP/P rs771298943 Cystathionine beta-synthase deficiency (CBSD) [MIM:236200]

CBS P35520 VAR_021799 p.Ser349Asn LP/P - Cystathionine beta-synthase deficiency (CBSD) [MIM:236200]
CBS P35520 VAR_021800 p.Ala355Pro LP/P rs1192581453 Cystathionine beta-synthase deficiency (CBSD) [MIM:236200]
CBS P35520 VAR_021801 p.Arg379Gln LP/P rs763036586 Cystathionine beta-synthase deficiency (CBSD) [MIM:236200]
CBS P35520 VAR_021802 p.Pro422Leu LP/P rs28934892 Cystathionine beta-synthase deficiency (CBSD) [MIM:236200]
CBS P35520 VAR_021803 p.Leu456Pro LP/P - Cystathionine beta-synthase deficiency (CBSD) [MIM:236200]
CBS P35520 VAR_046921 p.Arg18Cys LB/B rs201827340 -
CBS P35520 VAR_046922 p.Ala69Pro LB/B rs17849313 -
CBS P35520 VAR_046923 p.Arg125Pro LP/P - Cystathionine beta-synthase deficiency (CBSD) [MIM:236200]
CBS P35520 VAR_046924 p.Leu154Gln LP/P - Cystathionine beta-synthase deficiency (CBSD) [MIM:236200]
CBS P35520 VAR_046925 p.Ala155Val LP/P - Cystathionine beta-synthase deficiency (CBSD) [MIM:236200]
CBS P35520 VAR_046926 p.Val168Ala LP/P - Cystathionine beta-synthase deficiency (CBSD) [MIM:236200]
CBS P35520 VAR_046927 p.Met173Val LP/P - Cystathionine beta-synthase deficiency (CBSD) [MIM:236200]
CBS P35520 VAR_046928 p.Asn228Ser LP/P rs1555874803 Cystathionine beta-synthase deficiency (CBSD) [MIM:236200]
CBS P35520 VAR_046929 p.Ala231Pro LP/P - Cystathionine beta-synthase deficiency (CBSD) [MIM:236200]
CBS P35520 VAR_046932 p.Ala288Pro LP/P - Cystathionine beta-synthase deficiency (CBSD) [MIM:236200]
CBS P35520 VAR_046933 p.Ala288Thr LP/P rs141502207 Cystathionine beta-synthase deficiency (CBSD) [MIM:236200]
CBS P35520 VAR_046934 p.Ala361Thr LP/P rs745764562 Cystathionine beta-synthase deficiency (CBSD) [MIM:236200]
CBS P35520 VAR_046935 p.Asp376Asn LP/P rs1170128038 Cystathionine beta-synthase deficiency (CBSD) [MIM:236200]
CBS P35520 VAR_046936 p.Arg379Trp LP/P rs769080151 Cystathionine beta-synthase deficiency (CBSD) [MIM:236200]
CBS P35520 VAR_046937 p.Gln526Lys LP/P - Cystathionine beta-synthase deficiency (CBSD) [MIM:236200]
CBS P35520 VAR_046938 p.Arg548Gln LB/B rs150828989 -
CBS P35520 VAR_066099 p.Pro200Leu LP/P rs758712880 Cystathionine beta-synthase deficiency (CBSD) [MIM:236200]
CBS P35520 VAR_066100 p.Ile278Ser LP/P - Cystathionine beta-synthase deficiency (CBSD) [MIM:236200]
CBS P35520 VAR_066101 p.Asp281Asn LP/P - Cystathionine beta-synthase deficiency (CBSD) [MIM:236200]
CBS P35520 VAR_066102 p.Asp321Val LP/P - Cystathionine beta-synthase deficiency (CBSD) [MIM:236200]
CBS P35520 VAR_066103 p.Ala446Ser LP/P - Cystathionine beta-synthase deficiency (CBSD) [MIM:236200]

CBS P35520 VAR_074590 p.Thr87Asn LP/P - Cystathionine beta-synthase deficiency (CBSD) [MIM:236200]
CBS P35520 VAR_074592 p.Pro427Leu LP/P rs863223434 Cystathionine beta-synthase deficiency (CBSD) [MIM:236200]
CBS P35520 VAR_074593 p.Val449Gly LP/P - Cystathionine beta-synthase deficiency (CBSD) [MIM:236200]
CBS P35520 VAR_074594 p.Ser500Leu LP/P rs755106884 Cystathionine beta-synthase deficiency (CBSD) [MIM:236200]
CBS P35520 VAR_074595 p.Leu540Gln LP/P - Cystathionine beta-synthase deficiency (CBSD) [MIM:236200]
CBWD1 Q9BRT8 VAR_026979 p.Ala8Val LB/B rs16925054 -
CBWD5 Q5RIA9 VAR_067710 p.Thr147Ala LB/B rs1411096827 -
CBWD5 Q5RIA9 VAR_067711 p.Glu288Asp LB/B rs1168844980 -
CBX2 Q14781 VAR_063751 p.Pro98Leu LP/P rs121908255 46,XY sex reversal 5 (SRXY5) [MIM:613080]
CBX2 Q14781 VAR_063752 p.Arg443Pro LP/P rs121908256 46,XY sex reversal 5 (SRXY5) [MIM:613080]
CBX8 Q9HC52 VAR_014954 p.Gly317Val LB/B rs4889891 -
CBY2 Q8NA61 VAR_054064 p.Lys329Glu LB/B rs7317245 -
CC2D1A Q6P1N0 VAR_026670 p.Thr339Pro LB/B rs11883041 -
CC2D1A Q6P1N0 VAR_026671 p.Thr635Ser LB/B rs2290663 -
CC2D1A Q6P1N0 VAR_026672 p.Thr801Met LB/B rs2305777 -
CC2D1B Q5T0F9 VAR_062191 p.Met35Thr LB/B rs11555349 -
CC2D2A Q9P2K1 VAR_038489 p.Glu376Ala LB/B rs16892095 -
CC2D2A Q9P2K1 VAR_038490 p.Val660Ile LB/B rs16892134 -
CC2D2A Q9P2K1 VAR_055321 p.Gln1096His LP/P rs863225169 Joubert syndrome 9 (JBTS9) [MIM:612285]
CC2D2A Q9P2K1 VAR_055322 p.Pro1122Ser LP/P rs118204051 Joubert syndrome 9 (JBTS9) [MIM:612285]
CC2D2A Q9P2K1 VAR_055323 p.Arg1528Cys LP/P rs118204052 COACH syndrome 2 (COACH2) [MIM:619111]
CC2D2A Q9P2K1 VAR_055323 p.Arg1528Cys LP/P rs118204052 Joubert syndrome 9 (JBTS9) [MIM:612285]
CC2D2A Q9P2K1 VAR_055324 p.Leu1551Pro LP/P rs763425007 Joubert syndrome 9 (JBTS9) [MIM:612285]
CC2D2A Q9P2K1 VAR_062293 p.Thr1114Met LP/P rs386833752 Joubert syndrome 9 (JBTS9) [MIM:612285]
CC2D2A Q9P2K1 VAR_062293 p.Thr1114Met LP/P rs386833752 Meckel syndrome 6 (MKS6) [MIM:612284]
CC2D2A Q9P2K1 VAR_062804 p.Pro721Ser LP/P rs199768782 Joubert syndrome 9 (JBTS9) [MIM:612285]
CC2D2A Q9P2K1 VAR_062805 p.Lys800Glu LB/B rs751256652 -
CC2D2A Q9P2K1 VAR_062806 p.Asp1556Val LP/P rs201502401 Joubert syndrome 9 (JBTS9) [MIM:612285]
CC2D2A Q9P2K1 VAR_063804 p.Thr1116Met LP/P rs267606709 COACH syndrome 2 (COACH2) [MIM:619111]
CC2D2A Q9P2K1 VAR_063804 p.Thr1116Met LP/P rs267606709 Joubert syndrome 9 (JBTS9) [MIM:612285]
CC2D2A Q9P2K1 VAR_067535 p.Glu1447Ala LP/P rs387907058 Joubert syndrome 9 (JBTS9) [MIM:612285]
CC2D2A Q9P2K1 VAR_068169 p.Glu1126Lys LP/P rs1473532901 Joubert syndrome 9 (JBTS9) [MIM:612285]
CC2D2A Q9P2K1 VAR_069045 p.Asn1520Ser LP/P rs1478902342 Joubert syndrome 9 (JBTS9) [MIM:612285]
CC2D2A Q9P2K1 VAR_069046 p.Tyr1568His LP/P - Joubert syndrome 9 (JBTS9) [MIM:612285]
CC2D2A Q9P2K1 VAR_075698 p.Trp1182Arg LP/P rs386833755 Joubert syndrome 9 (JBTS9) [MIM:612285]
CC2D2A Q9P2K1 VAR_076881 p.Ser117Arg US rs186264635 Joubert syndrome 9 (JBTS9) [MIM:612285]
CC2D2A Q9P2K1 VAR_076882 p.Lys507Glu LB/B rs144439937 Joubert syndrome 9 (JBTS9) [MIM:612285]
CC2D2A Q9P2K1 VAR_076883 p.Leu559Pro LP/P rs754221308 Joubert syndrome 9 (JBTS9) [MIM:612285]
CC2D2A Q9P2K1 VAR_076884 p.Leu684Ile LB/B rs190698163 -
CC2D2A Q9P2K1 VAR_076885 p.Leu701Val LB/B rs537906621 -
CC2D2A Q9P2K1 VAR_076886 p.Val1045Ala LP/P rs863225173 Joubert syndrome 9 (JBTS9) [MIM:612285]
CC2D2A Q9P2K1 VAR_076887 p.Val1151Ala LP/P rs863225170 Joubert syndrome 9 (JBTS9) [MIM:612285]
CC2D2A Q9P2K1 VAR_076888 p.Arg1284Cys LP/P rs779823379 Joubert syndrome 9 (JBTS9) [MIM:612285]
CC2D2A Q9P2K1 VAR_076889 p.Arg1284His LP/P rs754586025 Joubert syndrome 9 (JBTS9) [MIM:612285]

CC2D2A	Q9P2K1	VAR_076890	p.Arg1330Gln	US	rs763486732	Joubert syndrome 9 (JBTS9) [MIM:612285]
CC2D2A	Q9P2K1	VAR_076891	p.Val1430Ala	LP/P	rs863225168	Joubert syndrome 9 (JBTS9) [MIM:612285]
CC2D2A	Q9P2K1	VAR_077560	p.Thr1517Ser	US	rs780673487	Meckel syndrome 6 (MKS6) [MIM:612284]
CC2D2B	Q6DHV5	VAR_050697	p.Asn1100Asp	LB/B	rs17383738	-
CC2D2B	Q6DHV5	VAR_050698	p.Tyr1273His	LB/B	rs9943393	-
CC2D2B	Q6DHV5	VAR_050699	p.Gln1437Leu	LB/B	rs1336459	-
CCAR1	Q8IX12	VAR_035497	p.Glu607Lys	US	-	A colorectal cancer sample
CCAR1	Q8IX12	VAR_058330	p.Thr588Ile	LB/B	rs1782338	-
CCAR1	Q8IX12	VAR_058331	p.Glu681Gly	LB/B	rs1060145	-
CCAR1	Q8IX12	VAR_058332	p.Met747Val	LB/B	rs11542602	-
CCBE1	Q6UXH8	VAR_048971	p.Val193Gly	LB/B	rs11659589	-
CCBE1	Q6UXH8	VAR_063746	p.Cys75Ser	LP/P	rs121908250	Hennekam lymphangiectasia-lymphedema syndrome 1 (HKLLS1) [MIM:235510]
CCBE1	Q6UXH8	VAR_063747	p.Cys102Ser	LP/P	rs121908251	Hennekam lymphangiectasia-lymphedema syndrome 1 (HKLLS1) [MIM:235510]
CCBE1	Q6UXH8	VAR_063748	p.Arg158Cys	LP/P	rs121908253	Hennekam lymphangiectasia-lymphedema syndrome 1 (HKLLS1) [MIM:235510]
CCBE1	Q6UXH8	VAR_063749	p.Cys174Arg	LP/P	rs121908254	Hennekam lymphangiectasia-lymphedema syndrome 1 (HKLLS1) [MIM:235510]
CCBE1	Q6UXH8	VAR_063750	p.Gly327Arg	LP/P	rs121908252	Hennekam lymphangiectasia-lymphedema syndrome 1 (HKLLS1) [MIM:235510]
CCDC102B	Q68D86	VAR_022893	p.Cys346Phe	LB/B	rs745894	-
CCDC102B	Q68D86	VAR_022894	p.Ala429Pro	LB/B	rs9963788	-
CCDC102B	Q68D86	VAR_047331	p.Lys153Asn	LB/B	rs572020	-
CCDC102B	Q68D86	VAR_047332	p.Lys298Arg	LB/B	rs2187094	-
CCDC102B	Q68D86	VAR_047333	p.Glu370Gly	LB/B	rs34102373	-
CCDC102B	Q68D86	VAR_047334	p.Asn425Lys	LB/B	rs17080065	-
CCDC103	Q8IW40	VAR_068449	p.His154Pro	US	rs145457535	Ciliary dyskinesia, primary, 17 (CILD17) [MIM:614679]
CCDC105	Q8IYK2	VAR_030875	p.Asp434Glu	LB/B	rs17855585	-
CCDC105	Q8IYK2	VAR_030876	p.Ser444Thr	LB/B	rs8111625	-
CCDC105	Q8IYK2	VAR_030877	p.Pro499Thr	LB/B	rs8112667	-
CCDC105	Q8IYK2	VAR_056774	p.Val245Met	LB/B	rs35352238	-
CCDC105	Q8IYK2	VAR_056775	p.Met248Thr	LB/B	rs34375549	-
CCDC107	Q8WV48	VAR_031406	p.Ser190Phe	LB/B	rs2275420	-
CCDC107	Q8WV48	VAR_031407	p.Ile220Val	LB/B	rs1339374	-
CCDC107	Q8WV48	VAR_031408	p.Ser242Cys	LB/B	rs10441685	-
CCDC110	Q8TBZ0	VAR_050729	p.Pro209Gln	LB/B	rs7699687	-
CCDC110	Q8TBZ0	VAR_050730	p.Leu299Met	LB/B	rs7698680	-
CCDC110	Q8TBZ0	VAR_050731	p.Leu382Met	LB/B	rs9999097	-
CCDC110	Q8TBZ0	VAR_050732	p.Ser409Phe	LB/B	rs11132306	-
CCDC110	Q8TBZ0	VAR_050733	p.Ile614Met	LB/B	rs35596415	-
CCDC110	Q8TBZ0	VAR_050734	p.Gln669Arg	LB/B	rs6827370	-
CCDC110	Q8TBZ0	VAR_050735	p.Ser817Leu	LB/B	rs9683564	-
CCDC110	Q8TBZ0	VAR_050736	p.Leu819Met	LB/B	rs34800518	-
CCDC110	Q8TBZ0	VAR_061577	p.Tyr500Asp	LB/B	rs59319722	-
CCDC112	Q8NEF3	VAR_039147	p.His32Leu	LB/B	rs34457718	-
CCDC112	Q8NEF3	VAR_039148	p.Ser144Asn	LB/B	rs34056787	-
CCDC112	Q8NEF3	VAR_039149	p.Lys341Asn	LB/B	rs17856922	-

CCDC112 Q8NEF3 VAR_039150 p.Glu354Gly LB/B rs17852930 -
CCDC113 Q9H0I3 VAR_030880 p.Asp4Glu LB/B rs8043587 -
CCDC113 Q9H0I3 VAR_030881 p.Ser6Thr LB/B rs8043590 -
CCDC115 Q96NT0 VAR_075752 p.Asp11Tyr LP/P rs869025583 Congenital disorder of glycosylation 2O (CDG2O) [MIM:616828]
CCDC115 Q96NT0 VAR_075753 p.Leu31Ser LP/P rs751325113 Congenital disorder of glycosylation 2O (CDG2O) [MIM:616828]
CCDC116 Q8IYX3 VAR_028803 p.Arg96Cys LB/B rs861854 -
CCDC116 Q8IYX3 VAR_028804 p.Gly121Arg LB/B rs371513 -
CCDC116 Q8IYX3 VAR_028805 p.Arg122Trp LB/B rs861853 -
CCDC116 Q8IYX3 VAR_028806 p.Arg199Trp LB/B rs12170285 -
CCDC116 Q8IYX3 VAR_028807 p.Ala436Thr LB/B rs11705259 -
CCDC116 Q8IYX3 VAR_061578 p.Pro293Leu LB/B rs41279987 -
CCDC117 Q8IWD4 VAR_028823 p.Arg147Ser LB/B rs13057011 -
CCDC117 Q8IWD4 VAR_028824 p.Ser272Asn LB/B rs9613680 -
CCDC122 Q5T0U0 VAR_061579 p.Ile269Thr LB/B rs9567280 -
CCDC124 Q96CT7 VAR_053809 p.Glu138Gln LB/B rs8104153 -
CCDC125 Q86Z20 VAR_032505 p.Val13Met LB/B rs10471774 -
CCDC127 Q96BQ5 VAR_050737 p.Arg60His LB/B rs11557427 -
CCDC13 Q8IYE1 VAR_033664 p.Ser547Thr LB/B rs12495805 -
CCDC13 Q8IYE1 VAR_055093 p.Arg25Trp LB/B rs17238798 -
CCDC13 Q8IYE1 VAR_055094 p.Glu375Val LB/B rs17853515 -
CCDC130 P13994 VAR_053854 p.Ser22Cys LB/B rs12974461 -
CCDC130 P13994 VAR_053855 p.Cys336Ser LB/B rs35761244 -
CCDC136 Q96JN2 VAR_034880 p.Asp218His LB/B rs3816887 -
CCDC136 Q96JN2 VAR_034881 p.Gln1049Glu LB/B rs4728137 -
CCDC137 Q6PK04 VAR_050739 p.His127Gln LB/B rs7226091 -
CCDC137 Q6PK04 VAR_050740 p.Arg177Trp LB/B rs11150805 -
CCDC137 Q6PK04 VAR_050741 p.Arg229Gln LB/B rs11546630 -
CCDC137 Q6PK04 VAR_061582 p.Arg282Trp LB/B rs11546631 -
CCDC138 Q96M89 VAR_032420 p.Asp99Tyr LB/B rs35794776 -
CCDC138 Q96M89 VAR_032421 p.Arg115Lys LB/B rs6740879 -
CCDC14 Q49A88 VAR_043116 p.Thr365Pro LB/B rs17310144 -
CCDC141 Q6ZP82 VAR_047904 p.Arg828Trp LB/B rs12988301 -
CCDC141 Q6ZP82 VAR_047905 p.Arg935Trp LB/B rs17362588 -
CCDC141 Q6ZP82 VAR_047906 p.Asn1170Ser LB/B rs13419085 -
CCDC142 Q17RM4 VAR_037210 p.Arg534Gln LB/B rs13385919 -
CCDC144NL Q6NUI1 VAR_045892 p.Gly82Asp LB/B rs7216787 -
CCDC146 Q8IYE0 VAR_050742 p.Asn345Ser LB/B rs1109968 -
CCDC146 Q8IYE0 VAR_061583 p.Glu263Gln LB/B rs17853516 -
CCDC146 Q8IYE0 VAR_061584 p.Ile466Thr LB/B rs58545343 -
CCDC148 Q8NFR7 VAR_039982 p.Gln75Arg LB/B rs4664950 -
CCDC148 Q8NFR7 VAR_039983 p.Met157Val LB/B rs12620556 -
CCDC148 Q8NFR7 VAR_039984 p.Lys329Arg LB/B rs7559772 -
CCDC15 Q0P6D6 VAR_050748 p.Gly529Asp LB/B rs4936966 -
CCDC15 Q0P6D6 VAR_050749 p.Cys813Arg LB/B rs7107487 -
CCDC15 Q0P6D6 VAR_069401 p.Phe166Cys LB/B - -
CCDC150 Q8NCX0 VAR_042600 p.Glu156Lys LB/B rs34133636 -
CCDC152 Q4G0S7 VAR_059596 p.Arg251His LB/B rs6879044 -

CCDC153	Q494R4	VAR_044322	p.Arg101His	LB/B	rs2301574	-
CCDC157	Q569K6	VAR_038999	p.Pro191Leu	LB/B	rs12167903	-
CCDC157	Q569K6	VAR_039000	p.Ser587Ala	LB/B	rs2015035	-
CCDC157	Q569K6	VAR_060124	p.Asp51Asn	LB/B	rs740223	-
CCDC158	Q5M9N0	VAR_039909	p.Glu232Asp	LB/B	rs17001889	-
CCDC158	Q5M9N0	VAR_039910	p.Ile297Val	LB/B	rs17001885	-
CCDC159	P0C7I6	VAR_043032	p.Cys278Ser	LB/B	rs6887	-
CCDC168	Q8NDH2	VAR_044210	p.Pro30Ser	LB/B	rs11843669	-
CCDC168	Q8NDH2	VAR_044211	p.Met94Val	LB/B	rs17592459	-
CCDC168	Q8NDH2	VAR_044212	p.Gly264Ser	LB/B	rs9518825	-
CCDC168	Q8NDH2	VAR_044213	p.Ser314Pro	LB/B	rs9300758	-
CCDC168	Q8NDH2	VAR_044214	p.Leu347Pro	LB/B	rs9300757	-
CCDC168	Q8NDH2	VAR_044215	p.Gly382Ala	LB/B	rs17507841	-
CCDC168	Q8NDH2	VAR_044216	p.Arg1015Thr	LB/B	rs7982465	-
CCDC168	Q8NDH2	VAR_044217	p.Asn1121Ser	LB/B	rs7983175	-
CCDC168	Q8NDH2	VAR_044218	p.Arg1209Gln	LB/B	rs17592438	-
CCDC168	Q8NDH2	VAR_044219	p.Phe1287Leu	LB/B	rs7335290	-
CCDC168	Q8NDH2	VAR_044220	p.Leu1382Pro	LB/B	rs6491707	-
CCDC168	Q8NDH2	VAR_044221	p.Arg1446Cys	LB/B	rs9300756	-
CCDC168	Q8NDH2	VAR_044222	p.Thr1483Met	LB/B	rs17507827	-
CCDC168	Q8NDH2	VAR_044223	p.Glu1879Ala	LB/B	rs9514051	-
CCDC168	Q8NDH2	VAR_044224	p.Lys1915Glu	LB/B	rs9554897	-
CCDC169	A6NNP5	VAR_057811	p.Lys120Arg	LB/B	rs9546897	-
CCDC17	Q96LX7	VAR_034976	p.Pro353Leu	LB/B	rs3014242	-
CCDC17	Q96LX7	VAR_034977	p.Ile557Val	LB/B	rs2297654	-
CCDC17	Q96LX7	VAR_059599	p.Val470Ile	LB/B	rs17410855	-
CCDC17	Q96LX7	VAR_063516	p.Ser580Asn	LB/B	rs3014246	-
CCDC170	Q8IYT3	VAR_028856	p.Ala269Val	LB/B	rs12205837	-
CCDC170	Q8IYT3	VAR_028857	p.Phe324Ser	LB/B	rs953767	-
CCDC170	Q8IYT3	VAR_028858	p.Ala331Thr	LB/B	rs17855718	-
CCDC170	Q8IYT3	VAR_028859	p.Val604Ile	LB/B	rs6929137	-
CCDC170	Q8IYT3	VAR_028860	p.Val683Ile	LB/B	rs3734804	-
CCDC170	Q8IYT3	VAR_050805	p.Asn479Lys	LB/B	rs35159094	-
CCDC170	Q8IYT3	VAR_050806	p.Arg553Gln	LB/B	rs34430497	-
CCDC170	Q8IYT3	VAR_061591	p.Glu345Lys	LB/B	rs55868409	-
CCDC171	Q6TFL3	VAR_022803	p.Ile17Asn	LB/B	rs443563	-
CCDC171	Q6TFL3	VAR_022804	p.Ser121Thr	LB/B	rs4741510	-
CCDC171	Q6TFL3	VAR_022805	p.Lys1069Arg	LB/B	rs1539172	-
CCDC171	Q6TFL3	VAR_033685	p.Asn495Ile	LB/B	rs10962127	-
CCDC171	Q6TFL3	VAR_050841	p.Cys821Tyr	LB/B	rs34816651	-
CCDC174	Q6PII3	VAR_061573	p.Thr436Met	LB/B	rs60239620	-
CCDC175	P0C221	VAR_050871	p.Gly164Arg	LB/B	rs17834244	-
CCDC175	P0C221	VAR_050872	p.Gly507Glu	LB/B	rs4261431	-
CCDC175	P0C221	VAR_050873	p.Ser689Asn	LB/B	rs12887189	-
CCDC175	P0C221	VAR_050874	p.Ser689Arg	LB/B	rs4394993	-
CCDC178	Q5BJE1	VAR_047010	p.Ala42Thr	LB/B	rs12606658	-
CCDC178	Q5BJE1	VAR_047011	p.Val465Glu	LB/B	rs9965081	-
CCDC178	Q5BJE1	VAR_047012	p.Leu600Arg	LB/B	rs457896	-
CCDC178	Q5BJE1	VAR_047013	p.Asp601Asn	LB/B	rs466113	-

CCDC178 Q5BJE1 VAR_061624 p.Asp420Asn LB/B rs58448816 -
CCDC180 Q9P1Z9 VAR_038151 p.Pro162His LB/B rs7864805 -
CCDC180 Q9P1Z9 VAR_038152 p.Ser183Arg LB/B rs17855671 -
CCDC180 Q9P1Z9 VAR_038153 p.Leu234His LB/B rs10981558 -
CCDC180 Q9P1Z9 VAR_038154 p.Glu778Lys LB/B rs12353306 -
CCDC180 Q9P1Z9 VAR_038155 p.Ser856Cys LB/B rs2061634 -
CCDC180 Q9P1Z9 VAR_038156 p.Phe1175Leu LB/B rs3747495 -
CCDC180 Q9P1Z9 VAR_038157 p.Asp1573Asn LB/B rs2306093 -
CCDC180 Q9P1Z9 VAR_061250 p.Pro409Arg LB/B rs61261278 -
CCDC181 Q5TID7 VAR_030907 p.Phe172Ser LB/B rs3820059 -
CCDC181 Q5TID7 VAR_056764 p.Phe238Ile LB/B rs35107735 -
CCDC181 Q5TID7 VAR_056765 p.Ser323Cys LB/B rs35287513 -
CCDC183 Q5T5S1 VAR_033046 p.Met38Thr LB/B rs945386 -
CCDC183 Q5T5S1 VAR_033047 p.Leu113Arg LB/B rs4546744 -
CCDC183 Q5T5S1 VAR_033048 p.Asp129Ala LB/B rs7859194 -
CCDC183 Q5T5S1 VAR_033049 p.Trp342Arg LB/B rs2811795 -
CCDC183 Q5T5S1 VAR_063112 p.Asn421Thr LB/B rs2254143 -
CCDC184 Q52MB2 VAR_060278 p.Glu140Asp LB/B rs10783231 -
CCDC185 Q8N715 VAR_027066 p.Val195Ala LB/B rs6689850 -
CCDC185 Q8N715 VAR_027067 p.Gly329Asp LB/B rs10907376 -
CCDC185 Q8N715 VAR_027068 p.Arg331Trp LB/B rs6682552 -
CCDC185 Q8N715 VAR_027069 p.Arg380Leu LB/B rs17852896 -
CCDC186 Q7Z3E2 VAR_023047 p.Thr85Ile LB/B rs1061159 -
CCDC186 Q7Z3E2 VAR_023048 p.Arg179Gln LB/B rs12782946 -
CCDC186 Q7Z3E2 VAR_023049 p.Gln271Lys LB/B rs7095762 -
CCDC191 Q8NCU4 VAR_036913 p.Arg374Trp LB/B rs17603649 -
CCDC191 Q8NCU4 VAR_036914 p.Glu696Asp LB/B rs6784095 -
CCDC198 Q9NVL8 VAR_024310 p.Tyr235Cys LB/B rs1152530 -
CCDC22 O60826 VAR_065912 p.Thr17Ala LP/P rs863225428 Ritscher-Schinzel syndrome 2 (RTSC2) [MIM:300963]
CCDC22 O60826 VAR_075063 p.Tyr557Cys LP/P rs863225429 Ritscher-Schinzel syndrome 2 (RTSC2) [MIM:300963]
CCDC22 O60826 VAR_076265 p.Asp546Asn LB/B rs147222955 -
CCDC27 Q2M243 VAR_026159 p.Gln54Glu LB/B rs10910021 -
CCDC27 Q2M243 VAR_026160 p.Met267Thr LB/B rs1181883 -
CCDC27 Q2M243 VAR_026161 p.Thr353Met LB/B rs10910024 -
CCDC27 Q2M243 VAR_056777 p.Pro390Leu LB/B rs11806371 -
CCDC28A Q8IWP9 VAR_050744 p.Ser42Tyr LB/B rs34538642 -
CCDC28A Q8IWP9 VAR_050745 p.Pro82Leu LB/B rs2273510 -
CCDC28B Q9BUN5 VAR_056776 p.Arg25Trp LB/B rs1407134 -
CCDC30 Q5VVM6 VAR_043472 p.His772Asn LB/B rs16829829 -
CCDC32 Q9BV29 VAR_034743 p.Lys2Ile LB/B rs10152546 -
CCDC33 Q8N5R6 VAR_036625 p.Ser683Asn LB/B rs1564782 -
CCDC33 Q8N5R6 VAR_045602 p.Met635Val LB/B rs2277603 -
CCDC33 Q8N5R6 VAR_045603 p.Arg640Leu LB/B rs2277604 -
CCDC34 Q96HJ3 VAR_043866 p.Pro53Ser LB/B rs11549824 -
CCDC34 Q96HJ3 VAR_043867 p.Glu264Ala LB/B rs17244028 -
CCDC34 Q96HJ3 VAR_043868 p.Ile319Val LB/B rs16925453 -
CCDC34 Q96HJ3 VAR_050750 p.His192Asn LB/B rs12364852 -

CCDC38	Q502W7	VAR_056778	p.Val36Phe	LB/B	rs12368787	-
CCDC38	Q502W7	VAR_056779	p.Met227Val	LB/B	rs10859974	-
CCDC39	Q9UFE4	VAR_072468	p.Thr594Ile	US	rs140505857	Ciliary dyskinesia, primary, 14 (CILD14) [MIM:613807]
CCDC40	Q4G0X9	VAR_035193	p.Ala8Pro	LB/B	rs2289530	-
CCDC42	Q96M95	VAR_026282	p.Ala144Thr	LB/B	rs2288156	-
CCDC42	Q96M95	VAR_057786	p.Thr51Pro	LB/B	rs12952995	-
CCDC42	Q96M95	VAR_057787	p.Arg75Cys	LB/B	rs9893451	-
CCDC50	Q8IVM0	VAR_050754	p.Leu121Phe	LB/B	rs35380043	-
CCDC50	Q8IVM0	VAR_050755	p.Met156Thr	LB/B	rs293813	-
CCDC51	Q96ER9	VAR_032515	p.Phe360Ser	LB/B	rs7618609	-
CCDC54	Q8NEL0	VAR_032151	p.Arg38Gln	LB/B	rs709564	-
CCDC57	Q2TAC2	VAR_032516	p.Glu237Gln	LB/B	rs34543170	-
CCDC57	Q2TAC2	VAR_032517	p.Gln321Arg	LB/B	rs7406116	-
CCDC57	Q2TAC2	VAR_032518	p.Val480Met	LB/B	rs7209474	-
CCDC57	Q2TAC2	VAR_032519	p.Arg774Gly	LB/B	rs4625783	-
CCDC57	Q2TAC2	VAR_032520	p.Asp776Asn	LB/B	rs7406163	-
CCDC57	Q2TAC2	VAR_032521	p.Ala777Thr	LB/B	rs7406162	-
CCDC57	Q2TAC2	VAR_032522	p.Gln810Lys	LB/B	rs7213172	-
CCDC57	Q2TAC2	VAR_032523	p.Met833Thr	LB/B	rs11077969	-
CCDC6	Q16204	VAR_062971	p.Pro470Thr	LB/B	rs1053266	-
CCDC60	Q8IWA6	VAR_033667	p.Ile46Val	LB/B	rs1064319	-
CCDC60	Q8IWA6	VAR_033668	p.Thr115Ala	LB/B	rs2519540	-
CCDC60	Q8IWA6	VAR_033669	p.Val393Ile	LB/B	rs16949292	-
CCDC62	Q6P9F0	VAR_026715	p.Thr394Lys	LB/B	rs17855031	-
CCDC62	Q6P9F0	VAR_035498	p.Gln31Glu	US	-	A colorectal cancer sample
CCDC62	Q6P9F0	VAR_061585	p.Thr141Met	LB/B	rs58131754	-
CCDC63	Q8NA47	VAR_050758	p.Leu212Ser	LB/B	rs12371434	-
CCDC65	Q8IXS2	VAR_056780	p.His133Arg	LB/B	rs10747556	-
CCDC65	Q8IXS2	VAR_056781	p.Tyr408Cys	LB/B	rs4760600	-
CCDC66	A2RUB6	VAR_039111	p.Gln383Arg	LB/B	rs1491170	-
CCDC66	A2RUB6	VAR_039112	p.Arg460Gln	LB/B	rs7637449	-
CCDC66	A2RUB6	VAR_039113	p.Glu592Lys	LB/B	rs4681904	-
CCDC66	A2RUB6	VAR_080466	p.Glu592Gln	LB/B	rs4681904	-
CCDC66	A2RUB6	VAR_080467	p.Cys681Tyr	LB/B	rs758090911	-
CCDC68	Q9H2F9	VAR_050762	p.Val249Ala	LB/B	rs34751112	-
CCDC69	A6NI79	VAR_042584	p.Arg197Lys	LB/B	rs248427	-
CCDC7	Q96M83	VAR_024308	p.Met1267Thr	LB/B	rs2504011	-
CCDC7	Q96M83	VAR_033692	p.Gly1117Ala	LB/B	rs4448627	-
CCDC7	Q96M83	VAR_033693	p.Val1364Ile	LB/B	rs1418538	-
CCDC7	Q96M83	VAR_050766	p.Lys449Thr	LB/B	rs12268559	-
CCDC7	Q96M83	VAR_061586	p.Lys148Gln	LB/B	rs56391924	-
CCDC70	Q6NSX1	VAR_026238	p.Ile206Val	LB/B	rs1054515	-
CCDC70	Q6NSX1	VAR_050763	p.Arg126Cys	LB/B	rs17076052	-
CCDC71	Q8IV32	VAR_026236	p.Gln317Leu	LB/B	rs4955419	-
CCDC71	Q8IV32	VAR_026237	p.Trp339Arg	LB/B	rs4955418	-
CCDC74A	Q96AQ1	VAR_030268	p.Gly302Arg	LB/B	rs13660	-
CCDC74B	Q96LY2	VAR_030269	p.Thr169Met	LB/B	rs184349150	-
CCDC74B	Q96LY2	VAR_030270	p.Arg346His	LB/B	rs3177472	-

CCDC77	Q9BR77	VAR_029838	p.Ser25Arg	LB/B	rs4980895	-
CCDC77	Q9BR77	VAR_029839	p.Met335Thr	LB/B	rs735295	-
CCDC78	A2IDD5	VAR_032867	p.Trp252Arg	LB/B	rs2071950	-
CCDC8	Q9H0W5	VAR_020272	p.Lys507Asn	LB/B	rs2279517	-
CCDC8	Q9H0W5	VAR_061587	p.Gly296Arg	LB/B	rs11880658	-
CCDC8	Q9H0W5	VAR_061588	p.His383Tyr	LB/B	rs34186470	-
CCDC81	Q6ZN84	VAR_032524	p.Tyr449Cys	LB/B	rs3741005	-
CCDC82	Q8N4S0	VAR_026164	p.Gln149Glu	LB/B	rs17851661	-
CCDC82	Q8N4S0	VAR_026165	p.Gln327Arg	LB/B	rs10831519	-
CCDC82	Q8N4S0	VAR_050764	p.Lys128Arg	LB/B	rs3748261	-
CCDC83	Q8IWF9	VAR_032525	p.Thr49Ala	LB/B	rs12362209	-
CCDC86	Q9H6F5	VAR_032069	p.Gln153His	LB/B	rs2074421	-
CCDC87	Q9NVE4	VAR_033224	p.Phe217Leu	LB/B	rs17853294	-
CCDC87	Q9NVE4	VAR_056782	p.Ala156Thr	LB/B	rs1110707	-
CCDC88B	A6NC98	VAR_038523	p.Asp193Glu	LB/B	rs647152	-
CCDC88B	A6NC98	VAR_038524	p.Trp639Arg	LB/B	rs685870	-
CCDC88B	A6NC98	VAR_038525	p.Asp886Ala	LB/B	rs1318165	-
CCDC88C	Q9P219	VAR_046613	p.Ala811Glu	LB/B	rs17127223	-
CCDC88C	Q9P219	VAR_046614	p.Ala1028Val	LB/B	rs1970911	-
CCDC88C	Q9P219	VAR_046615	p.Leu1992Pro	LB/B	rs941920	-
CCDC88C	Q9P219	VAR_057777	p.Leu637Val	LB/B	rs7160308	-
CCDC88C	Q9P219	VAR_071981	p.Arg464His	LP/P	rs587782989	Spinocerebellar ataxia 40 (SCA40) [MIM:616053]
CCDC9	Q9Y3X0	VAR_033670	p.Glu215Asp	LB/B	rs2032811	-
CCDC9	Q9Y3X0	VAR_033671	p.Ala456Val	LB/B	rs35119724	-
CCDC9	Q9Y3X0	VAR_050767	p.Leu478Pro	LB/B	rs888836	-
CCDC90B	Q9GZT6	VAR_033322	p.Phe10Leu	LB/B	rs494791	-
CCDC91	Q7Z6B0	VAR_021531	p.Met261Val	LB/B	rs1133028	-
CCDC91	Q7Z6B0	VAR_021532	p.Val314Met	LB/B	rs10771427	-
CCDC92	Q53HC0	VAR_030301	p.Ser70Cys	LB/B	rs11057401	-
CCDC92	Q53HC0	VAR_030302	p.Arg281His	LB/B	rs17886730	-
CCDC92	Q53HC0	VAR_050765	p.Ala253Thr	LB/B	rs35935939	-
CCDC93	Q567U6	VAR_035499	p.His315Arg	US	-	A colorectal cancer sample
CCDC93	Q567U6	VAR_054108	p.Arg179Cys	LB/B	rs33975708	-
CCDC93	Q567U6	VAR_054109	p.Arg213Cys	LB/B	rs34095554	-
CCDC93	Q567U6	VAR_054110	p.Pro228Leu	LB/B	rs17512204	-
CCDC93	Q567U6	VAR_054111	p.Tyr465His	LB/B	rs17047557	-
CCDC96	Q2M329	VAR_026162	p.Glu96Lys	LB/B	rs871134	-
CCDC9B	Q6ZUT6	VAR_035620	p.Gly189Glu	US	-	A colorectal cancer sample
CCER1	Q8TC90	VAR_032512	p.Ile379Met	LB/B	rs17855513	-
CCER1	Q8TC90	VAR_059617	p.Asp286Glu	LB/B	rs11105882	-
CCER2	I3L3R5	VAR_079159	p.Glu33Asp	LB/B	rs76973734	-
CCER2	I3L3R5	VAR_079160	p.Leu39Pro	LB/B	rs530595113	-
CCER2	I3L3R5	VAR_079161	p.Glu64Asp	LB/B	rs375222589	-
CCER2	I3L3R5	VAR_079163	p.Gly80Val	LB/B	rs908319857	-
CCER2	I3L3R5	VAR_079164	p.Glu160Lys	LB/B	rs371603378	-
CCER2	I3L3R5	VAR_079165	p.Arg195Cys	LB/B	rs569628536	-
CCER2	I3L3R5	VAR_079168	p.Ala224Thr	LB/B	rs1301343892	-
CCER2	I3L3R5	VAR_079170	p.Glu232Ala	LB/B	rs369436329	-

CCER2	I3L3R5	VAR_079171	p.Glu242Lys	US	rs1205789753	-
CCER2	I3L3R5	VAR_079172	p.Asp249Gly	LB/B	rs565410180	-
CCHCR1	Q8TD31	VAR_017761	p.Arg102Gln	LB/B	rs130075	-
CCHCR1	Q8TD31	VAR_017762	p.Arg103Trp	LB/B	rs130065	-
CCHCR1	Q8TD31	VAR_017763	p.Arg109Trp	LB/B	rs130076	-
CCHCR1	Q8TD31	VAR_017764	p.Ser164Arg	LB/B	rs130066	-
CCHCR1	Q8TD31	VAR_017765	p.Glu275Asp	LB/B	rs130067	-
CCHCR1	Q8TD31	VAR_017766	p.Arg417Trp	LB/B	rs130068	-
CCHCR1	Q8TD31	VAR_017767	p.Arg417Gln	LB/B	rs130069	-
CCHCR1	Q8TD31	VAR_017768	p.Lys546Arg	LB/B	rs2073720	-
CCHCR1	Q8TD31	VAR_017769	p.Gly575Cys	LB/B	rs130079	-
CCHCR1	Q8TD31	VAR_017770	p.Arg627Gln	LB/B	rs130072	-
CCHCR1	Q8TD31	VAR_017771	p.Gln639His	LB/B	rs130074	-
CCHCR1	Q8TD31	VAR_017772	p.Ser776Cys	LB/B	rs1576	-
CCHCR1	Q8TD31	VAR_017782	p.Leu179Gln	LB/B	rs11540822	-
CCHCR1	Q8TD31	VAR_017783	p.Ala367Thr	LB/B	rs2027937	-
CCHCR1	Q8TD31	VAR_017784	p.Ala733Val	LB/B	rs140560656	-
CCIN	Q13939	VAR_050039	p.Ser75Asn	LB/B	rs34789048	-
CCK	P06307	VAR_018818	p.Gly32Glu	LB/B	rs11571848	-
CCK	P06307	VAR_024452	p.Arg95Trp	LB/B	rs3774395	-
CCKBR	P32239	VAR_014684	p.Leu37Phe	LB/B	rs1805000	-
CCKBR	P32239	VAR_014685	p.Val125Ile	LB/B	rs1805002	-
CCKBR	P32239	VAR_014686	p.Arg215His	LB/B	rs1805004	-
CCKBR	P32239	VAR_014687	p.Arg319Gln	LB/B	rs1805001	-
CCKBR	P32239	VAR_049388	p.Val77Gly	LB/B	rs35816985	-
CCL11	P51671	VAR_001634	p.Leu7Pro	LB/B	-	-
CCL11	P51671	VAR_001635	p.Ala23Thr	LB/B	rs1129844	-
CCL11	P51671	VAR_001636	p.Arg51Ser	LB/B	-	-
CCL11	P51671	VAR_001637	p.Lys79Arg	LB/B	-	-
CCL11	P51671	VAR_048705	p.Lys86Thr	LB/B	rs34262946	-
CCL13	Q99616	VAR_024169	p.Ala5Ser	LB/B	rs3136677	-
CCL13	Q99616	VAR_048706	p.Asn29Ser	LB/B	rs34566308	-
CCL14	Q16627	VAR_048707	p.Lys61Glu	LB/B	rs16971802	-
CCL15	Q16663	VAR_011640	p.Ile24Thr	LB/B	rs854625	-
CCL17	Q92583	VAR_048708	p.Lys5Asn	LB/B	rs34693308	-
CCL17	Q92583	VAR_048709	p.Val67Met	LB/B	rs34379253	-
CCL20	P78556	VAR_011915	p.Val47Met	LB/B	rs1049617	-
CCL22	O00626	VAR_055117	p.Asp2Ala	LB/B	rs4359426	-
CCL23	P55773	VAR_011916	p.Val106Met	LB/B	rs1003645	-
CCL24	O00175	VAR_018404	p.Ile29Leu	LB/B	rs2302006	-
CCL24	O00175	VAR_048710	p.Ser31Phe	LB/B	rs11465293	-
CCL24	O00175	VAR_048711	p.Ala102Thr	LB/B	rs11465312	-
CCL24	O00175	VAR_048712	p.Gln110Glu	LB/B	rs11465313	-
CCL25	O15444	VAR_044519	p.Thr23Ala	LB/B	rs960173	-
CCL25	O15444	VAR_044520	p.His101Arg	LB/B	rs2032887	-
CCL25	O15444	VAR_044521	p.Thr104Met	LB/B	rs1129763	-
CCL26	Q9Y258	VAR_029192	p.Leu18Arg	LB/B	rs11465333	-
CCL27	Q9Y4X3	VAR_022103	p.Ile78Val	LB/B	rs11575594	-
CCL27	Q9Y4X3	VAR_022104	p.Leu96Phe	LB/B	rs11575584	-

CCL3 P10147 VAR_048701 p.Glu78Asp LB/B rs34171309 -
CCL4 P13236 VAR_048702 p.Met12Val LB/B rs1049752 -
CCL4 P13236 VAR_048703 p.Pro20Leu LB/B rs1130750 -
CCL4 P13236 VAR_059211 p.Ser80Thr LB/B rs1719152 -
CCL5 P13501 VAR_043043 p.Ser24Phe LB/B rs377415776 -
CCL8 P80075 VAR_001633 p.Lys69Gln LB/B rs1133763 -
CCL8 P80075 VAR_048704 p.Ala14Val LB/B rs35401229 -
CCM2 Q9BSQ5 VAR_023575 p.Val53Ile LB/B rs2107732 -
CCM2 Q9BSQ5 VAR_023576 p.Val120Ile LB/B rs11552377 -
CCM2 Q9BSQ5 VAR_023577 p.Leu198Arg LP/P rs137852843 Cerebral cavernous malformations 2 (CCM2) [MIM:603284]
CCM2 Q9BSQ5 VAR_050768 p.Ser289Asn LB/B rs2289366 -
CCM2 Q9BSQ5 VAR_067352 p.Gln215His LP/P - Cerebral cavernous malformations 2 (CCM2) [MIM:603284]
CCM2 Q9BSQ5 VAR_067353 p.Leu229Gln LP/P - Cerebral cavernous malformations 2 (CCM2) [MIM:603284]
CCN1 O00622 VAR_018934 p.Arg334Trp LB/B rs9658587 -
CCN2 P29279 VAR_027925 p.His83Asp LB/B rs7451102 -
CCN3 P48745 VAR_049568 p.Arg42Gln LB/B rs2279112 -
CCN3 P48745 VAR_049569 p.Arg233His LB/B rs11538929 -
CCN4 O95388 VAR_061265 p.Ala205Ser LB/B rs35513885 -
CCN5 O76076 VAR_049566 p.Arg59Gln LB/B rs33932543 -
CCN6 O95389 VAR_016224 p.Gln56His LB/B rs1230345 -
CCN6 O95389 VAR_016225 p.Cys78Arg LP/P rs121908902 Progressive pseudorheumatoid arthropathy of childhood (PPAC) [MIM:208230]
CCN6 O95389 VAR_049567 p.Arg60Cys LB/B rs17073260 -
CCN6 O95389 VAR_081485 p.Cys78Tyr LP/P - Progressive pseudorheumatoid arthropathy of childhood (PPAC) [MIM:208230]
CCN6 O95389 VAR_081486 p.Gly83Glu LB/B rs147337485 -
CCN6 O95389 VAR_081487 p.Cys114Arg LP/P - Progressive pseudorheumatoid arthropathy of childhood (PPAC) [MIM:208230]
CCN6 O95389 VAR_081488 p.Cys114Trp LP/P - Progressive pseudorheumatoid arthropathy of childhood (PPAC) [MIM:208230]
CCN6 O95389 VAR_081490 p.Cys145Arg US - Progressive pseudorheumatoid arthropathy of childhood (PPAC) [MIM:208230]
CCN6 O95389 VAR_081491 p.Cys223Gly LP/P rs782813346 Progressive pseudorheumatoid arthropathy of childhood (PPAC) [MIM:208230]
CCN6 O95389 VAR_081492 p.Ser228Pro US - Progressive pseudorheumatoid arthropathy of childhood (PPAC) [MIM:208230]
CCN6 O95389 VAR_081493 p.Cys268Gly US - Progressive pseudorheumatoid arthropathy of childhood (PPAC) [MIM:208230]
CCN6 O95389 VAR_081495 p.Cys337Tyr LP/P - Progressive pseudorheumatoid arthropathy of childhood (PPAC) [MIM:208230]
CCN6 O95389 VAR_081652 p.Tyr99Phe US - Progressive pseudorheumatoid arthropathy of childhood (PPAC) [MIM:208230]
CCN6 O95389 VAR_081653 p.Cys100Ser US - Progressive pseudorheumatoid arthropathy of childhood (PPAC) [MIM:208230]
CCN6 O95389 VAR_081654 p.Cys114Tyr LP/P - Progressive pseudorheumatoid arthropathy of childhood (PPAC) [MIM:208230]

CCN6 O95389 VAR_081655 p.Cys145Tyr US rs121908899 Progressive pseudorheumatoid arthropathy of childhood (PPAC) [MIM:208230]
CCN6 O95389 VAR_081657 p.Gly226Val LP/P - Progressive pseudorheumatoid arthropathy of childhood (PPAC) [MIM:208230]
CCN6 O95389 VAR_081659 p.Ser334Pro LP/P rs121908903 Progressive pseudorheumatoid arthropathy of childhood (PPAC) [MIM:208230]
CCNA2 P20248 VAR_018819 p.Ile163Val LB/B rs769242 -
CCNB2 O95067 VAR_022221 p.Met100Thr LB/B rs16941036 -
CCNB2 O95067 VAR_022222 p.Ile395Thr LB/B rs28383563 -
CCNB2 O95067 VAR_053052 p.Val135Ile LB/B rs2306785 -
CCNB3 Q8WWL7 VAR_036580 p.Lys597Thr US - A colorectal cancer sample
CCNB3 Q8WWL7 VAR_047027 p.Gly1001Arg LB/B rs6614336 -
CCND2 P30279 VAR_018820 p.Gly268Arg LB/B rs3217921 -
CCND2 P30279 VAR_072370 p.Thr280Ala LP/P rs587777618 Megalencephaly-polymicrogyria-polydactyly-hydrocephalus syndrome 3 (MPPH3) [MIM:615938]
CCND2 P30279 VAR_072371 p.Thr280Asn LP/P rs587777620 Megalencephaly-polymicrogyria-polydactyly-hydrocephalus syndrome 3 (MPPH3) [MIM:615938]
CCND2 P30279 VAR_072372 p.Pro281Leu LP/P rs587777622 Megalencephaly-polymicrogyria-polydactyly-hydrocephalus syndrome 3 (MPPH3) [MIM:615938]
CCND2 P30279 VAR_072373 p.Pro281Arg LP/P rs587777622 Megalencephaly-polymicrogyria-polydactyly-hydrocephalus syndrome 3 (MPPH3) [MIM:615938]
CCND2 P30279 VAR_072374 p.Val284Gly LP/P rs777786993 Megalencephaly-polymicrogyria-polydactyly-hydrocephalus syndrome 3 (MPPH3) [MIM:615938]
CCND3 P30281 VAR_014205 p.Ser259Ala LB/B rs1051130 -
CCND3 P30281 VAR_020412 p.Pro134Ser LB/B rs3218089 -
CCND3 P30281 VAR_033726 p.Glu253Asp LB/B rs33966734 -
CCNE2 O96020 VAR_021347 p.Asn387Ser LB/B rs28399585 -
CCNG1 P51959 VAR_021079 p.Asn178His LB/B rs2069352 -
CCNG1 P51959 VAR_021080 p.Phe179Leu LB/B rs11541970 -
CCNG2 Q16589 VAR_014333 p.Leu4Val LB/B rs4150050 -
CCNG2 Q16589 VAR_014334 p.Glu28Gly LB/B rs4150051 -
CCNH P51946 VAR_013067 p.Arg28Leu LB/B rs2234942 -
CCNH P51946 VAR_013068 p.Met54Val LB/B rs3093785 -
CCNH P51946 VAR_013069 p.Lys138Arg LB/B rs2266691 -
CCNH P51946 VAR_013070 p.Val270Ala LB/B rs2230641 -
CCNI Q14094 VAR_016312 p.Val207Ile LB/B rs4252903 -
CCNI2 Q6ZMN8 VAR_043471 p.Ala91Pro LB/B rs803056 -
CCNJL Q8IV13 VAR_053053 p.His234Tyr LB/B rs13362036 -
CCNK O75909 VAR_081570 p.Lys111Glu LP/P rs1566748800 Intellectual developmental disorder with hypertelorism and distinctive facies (IDDHDF) [MIM:618147]
CCNO P22674 VAR_029081 p.Leu161Met LB/B rs13169396 -
CCNO P22674 VAR_071197 p.His239Arg LP/P rs797045150 Ciliary dyskinesia, primary, 29 (CILD29) [MIM:615872]
CCNO P22674 VAR_077581 p.Leu213Pro LP/P rs775051461 Ciliary dyskinesia, primary, 29 (CILD29) [MIM:615872]
CCNQ Q8N1B3 VAR_034642 p.Cys183Ser LB/B rs17850173 -
CCNT1 O60563 VAR_053054 p.His362Arg LB/B rs17123261 -
CCNT1 O60563 VAR_069400 p.Arg541Cys LB/B rs201951577 -
CCNYL2 Q5T2Q4 VAR_037297 p.Val57Glu LB/B rs2490085 -

CCNYL2	Q5T2Q4	VAR_037298	p.Arg176Gln	LB/B	rs2489720	-
CCNYL2	Q5T2Q4	VAR_037299	p.Ile288Val	LB/B	rs2505861	-
CCP110	O43303	VAR_019823	p.Ile252Met	LB/B	rs226891	-
CCP110	O43303	VAR_019824	p.Met375Ile	LB/B	rs7190666	-
CCP110	O43303	VAR_056788	p.Arg69Ser	LB/B	rs16972129	-
CCP110	O43303	VAR_056789	p.Pro171Leu	LB/B	rs3751821	-
CCP110	O43303	VAR_056790	p.Phe347Ile	LB/B	rs11645625	-
CCPG1	Q9ULG6	VAR_037063	p.Ser44Pro	LB/B	rs11555304	-
CCPG1	Q9ULG6	VAR_037064	p.Glu161Val	LB/B	rs17853336	-
CCPG1	Q9ULG6	VAR_037065	p.Tyr418His	LB/B	rs34958422	-
CCPG1	Q9ULG6	VAR_037066	p.Arg436Leu	LB/B	rs17857026	-
CCPG1	Q9ULG6	VAR_037067	p.Ala477Val	LB/B	rs1063562	-
CCPG1	Q9ULG6	VAR_037068	p.Ala517Asp	LB/B	rs1063563	-
CCPG1	Q9ULG6	VAR_037069	p.Gly553Asp	LB/B	rs1063565	-
CCPG1	Q9ULG6	VAR_037070	p.Gly553Ser	LB/B	rs1063564	-
CCPG1	Q9ULG6	VAR_037071	p.Asn590Lys	LB/B	rs1063566	-
CCPG1	Q9ULG6	VAR_037072	p.Lys627Glu	LB/B	rs17853335	-
CCPG1	Q9ULG6	VAR_037073	p.Thr646Ile	LB/B	rs17857027	-
CCPG1	Q9ULG6	VAR_037074	p.His673Arg	LB/B	rs1063567	-
CCR2	P41597	VAR_014339	p.Val64Ile	LB/B	rs1799864	-
CCR2	P41597	VAR_014340	p.Gly355Glu	LB/B	rs3918387	-
CCR2	P41597	VAR_020066	p.Leu45Val	LB/B	rs4987052	-
CCR3	P51677	VAR_010668	p.Cys218Ser	LB/B	-	-
CCR3	P51677	VAR_020067	p.Gly21Asp	LB/B	rs4987125	-
CCR3	P51677	VAR_049382	p.Pro39Leu	LB/B	rs5742906	-
CCR4	P51679	VAR_010669	p.Leu130Val	LB/B	-	-
CCR4	P51679	VAR_010670	p.Cys178Ser	LB/B	rs753825374	-
CCR5	P51681	VAR_003481	p.Tyr10Asp	US	-	-
CCR5	P51681	VAR_003482	p.Arg31His	LB/B	rs56340326	-
CCR5	P51681	VAR_003483	p.Pro34Leu	US	-	-
CCR5	P51681	VAR_003484	p.Lys62Arg	US	-	-
CCR5	P51681	VAR_003485	p.Tyr68His	LB/B	rs758090461	-
CCR5	P51681	VAR_003486	p.Asp95Asn	LB/B	rs149975182	-
CCR5	P51681	VAR_003487	p.Gly97Glu	US	-	-
CCR5	P51681	VAR_003488	p.Leu122Pro	US	-	-
CCR5	P51681	VAR_003489	p.Phe158Ser	US	-	-
CCR5	P51681	VAR_003490	p.Tyr176Cys	US	-	-
CCR5	P51681	VAR_003491	p.Thr177Ala	US	-	-
CCR5	P51681	VAR_003492	p.Ser185Asn	US	-	-
CCR5	P51681	VAR_003493	p.Met210Val	US	-	-
CCR5	P51681	VAR_003494	p.Tyr214Cys	US	-	-
CCR5	P51681	VAR_003495	p.Thr239Ser	US	-	-
CCR5	P51681	VAR_003496	p.Leu246Pro	LB/B	rs143181119	-
CCR5	P51681	VAR_003497	p.Thr288Met	LB/B	rs534088482	-
CCR5	P51681	VAR_003498	p.Glu302Gly	US	-	-
CCR5	P51681	VAR_003499	p.Lys303Glu	US	-	-
CCR5	P51681	VAR_003500	p.Asn306Ser	US	-	-
CCR5	P51681	VAR_003501	p.Lys322Arg	US	-	-
CCR5	P51681	VAR_003502	p.Glu333Gly	US	-	-

CCR5 P51681 VAR_003503 p.Ala335Val LB/B rs1800944 -
CCR5 P51681 VAR_003504 p.Tyr339Phe LB/B rs1800945 -
CCR5 P51681 VAR_003505 p.Glu345Gly US - -
CCR5 P51681 VAR_011839 p.Ala29Ser LB/B rs1800939 -
CCR5 P51681 VAR_011840 p.Leu55Gln LB/B rs1799863 -
CCR5 P51681 VAR_011841 p.Arg60Ser LB/B rs1800940 -
CCR5 P51681 VAR_011842 p.Arg223Gln LB/B rs1800452 -
CCR5 P51681 VAR_011843 p.Gly301Val LB/B rs1800943 -
CCR5 P51681 VAR_012481 p.Cys178Arg LB/B rs199824195 -
CCR5 P51681 VAR_024066 p.Ile12Leu LB/B - -
CCR5 P51681 VAR_024067 p.Cys20Ser LB/B rs145061115 -
CCR5 P51681 VAR_024068 p.Ile42Phe LB/B rs1475319259 -
CCR5 P51681 VAR_024069 p.Ala73Val LB/B rs56198941 -
CCR5 P51681 VAR_024070 p.Ser215Leu LB/B rs1017863136 -
CCR5 P51681 VAR_080410 p.Gly106Arg LB/B rs183662584 -
CCR7 P32248 VAR_049383 p.Met7Val LB/B rs2228015 -
CCR8 P51685 VAR_049384 p.Ala27Gly LB/B rs2853699 -
CCR9 P51686 VAR_020068 p.Met284Val LB/B rs12721497 -
CCR9 P51686 VAR_029208 p.Ile92Val LB/B rs45530037 -
CCRL2 O00421 VAR_026488 p.Phe167Tyr LB/B rs3204849 -
CCRL2 O00421 VAR_026489 p.Val168Met LB/B rs6441977 -
CCRL2 O00421 VAR_026490 p.Ile243Val LB/B rs3204850 -
CCRL2 O00421 VAR_049385 p.Tyr4Cys LB/B rs11574443 -
CCS O14618 VAR_068078 p.Arg163Trp LB/B rs142340643 -
CCSAP Q6IQ19 VAR_059594 p.Ala123Val LB/B rs6587326 -
CCSER1 Q9C0I3 VAR_059336 p.Gly382Ser LB/B rs12647859 -
CCSER2 Q9H7U1 VAR_036952 p.Asn84Ser LB/B rs3814205 -
CCSER2 Q9H7U1 VAR_036953 p.Cys755Tyr LB/B rs11201058 -
CCSER2 Q9H7U1 VAR_036954 p.Ser819Pro LB/B rs11557865 -
CCSER2 Q9H7U1 VAR_036955 p.Pro821Ser LB/B rs12569751 -
CCT3 P49368 VAR_052265 p.Leu391Phe LB/B rs2230194 -
CCT4 P50991 VAR_052266 p.Ile112Val LB/B rs2272428 -
CCT5 P48643 VAR_030658 p.His147Arg LP/P rs118203986 Neuropathy, hereditary sensory, with spastic
paraplegia, autosomal recessive (HSNSP) [MIM:256840]
CCT5 P48643 VAR_052267 p.Glu146Val LB/B rs11557652 -
CCT6A P40227 VAR_052268 p.Tyr229Cys LB/B rs33922584 -
CCT6B Q92526 VAR_057269 p.Gly247Ala LB/B rs2230553 -
CCT6B Q92526 VAR_060297 p.Arg17Gln LB/B rs9635769 -
CCT6B Q92526 VAR_060298 p.Val48Ala LB/B rs2230552 -
CCT7 Q99832 VAR_052269 p.Thr259Ala LB/B rs2231427 -
CCT8 P50990 VAR_052270 p.His4Gln LB/B rs16983693 -
CCT8 P50990 VAR_052271 p.Val409Ile LB/B rs8129954 -
CCT8L1P A6NM43 VAR_046039 p.Cys217Ser LB/B rs6969304 -
CCT8L1P A6NM43 VAR_046040 p.Asp308Asn LB/B rs12672139 -
CCT8L1P A6NM43 VAR_046041 p.Ser395Gly LB/B rs6953943 -
CCT8L2 Q96SF2 VAR_046042 p.Trp320Arg LB/B rs2236639 -
CD101 Q93033 VAR_028371 p.Asn225Ser LB/B rs3754112 -
CD101 Q93033 VAR_028372 p.Met415Val LB/B rs2249265 -
CD101 Q93033 VAR_028373 p.Arg518Gln LB/B rs17235766 -

CD101	Q93033	VAR_028374	p.Ser525Arg	LB/B	rs17235773	-
CD101	Q93033	VAR_028375	p.Val965Ile	LB/B	rs12097758	-
CD101	Q93033	VAR_028376	p.Arg988Cys	LB/B	rs12067543	-
CD101	Q93033	VAR_054434	p.Gly157Ser	LB/B	rs34999087	-
CD101	Q93033	VAR_054435	p.Thr631Ser	LB/B	rs34510762	-
CD101	Q93033	VAR_054436	p.Arg933Gln	LB/B	rs12093834	-
CD101	Q93033	VAR_054437	p.Leu955Phe	LB/B	rs34223095	-
CD101	Q93033	VAR_054438	p.Arg992Trp	LB/B	rs34248572	-
CD109	Q6YHK3	VAR_028875	p.Gly45Val	LB/B	rs9446983	-
CD109	Q6YHK3	VAR_028876	p.Tyr703Ser	LB/B	rs10455097	-
CD109	Q6YHK3	VAR_028877	p.Asn797Ser	LB/B	rs2351528	-
CD109	Q6YHK3	VAR_028878	p.Val845Ile	LB/B	rs5023688	-
CD109	Q6YHK3	VAR_028879	p.Thr1241Met	LB/B	rs2917862	-
CD109	Q6YHK3	VAR_036236	p.Gln1007Glu	US	rs1454572681	A colorectal cancer sample
CD109	Q6YHK3	VAR_036237	p.Asn1065Lys	US	-	A colorectal cancer sample
CD109	Q6YHK3	VAR_048105	p.Gly377Asp	LB/B	rs7741152	-
CD109	Q6YHK3	VAR_048106	p.Leu641Phe	LB/B	rs7742662	-
CD109	Q6YHK3	VAR_048107	p.Val1009Met	LB/B	rs35630075	-
CD109	Q6YHK3	VAR_048108	p.His1296Arg	LB/B	rs13207595	-
CD109	Q6YHK3	VAR_074179	p.Asp791Gly	LB/B	-	-
CD14	P08571	VAR_024302	p.Asn204Asp	LB/B	rs2228049	-
CD14	P08571	VAR_050771	p.Glu341Lys	LB/B	rs11556179	-
CD151	P48509	VAR_012490	p.Lys132Arg	LB/B	-	-
CD151	P48509	VAR_012491	p.Pro137Ser	LB/B	rs1431926999	-
CD151	P48509	VAR_021153	p.Arg178His	LB/B	rs779114765	-
CD151	P48509	VAR_025098	p.Thr120Met	LB/B	rs34215390	-
CD160	O95971	VAR_027747	p.Ile91Val	LB/B	rs2231373	-
CD163	Q86VB7	VAR_026574	p.Ile342Val	LB/B	rs4883263	-
CD163L1	Q9NR16	VAR_057206	p.Asp578Asn	LB/B	rs4072797	-
CD163L1	Q9NR16	VAR_057207	p.Gly582Ala	LB/B	rs4072796	-
CD163L1	Q9NR16	VAR_057208	p.Gly1055Ser	LB/B	rs36206713	-
CD163L1	Q9NR16	VAR_057209	p.Met1108Thr	LB/B	rs35480970	-
CD163L1	Q9NR16	VAR_059810	p.Leu523Met	LB/B	rs6488268	-
CD164L2	Q6UWJ8	VAR_057508	p.Val76Met	LB/B	rs2504779	-
CD177	Q8N6Q3	VAR_025858	p.Ala3Pro	LB/B	rs45441892	-
CD177	Q8N6Q3	VAR_025859	p.Leu251Ile	LB/B	rs10425835	-
CD177	Q8N6Q3	VAR_025860	p.Ala348Thr	LB/B	rs17856829	-
CD177	Q8N6Q3	VAR_026156	p.Leu119Phe	LB/B	-	-
CD177	Q8N6Q3	VAR_026157	p.Arg323Gln	LB/B	-	-
CD177	Q8N6Q3	VAR_026158	p.Phe379Ser	LB/B	-	-
CD177	Q8N6Q3	VAR_079754	p.His31Leu	LB/B	rs45553433	-
CD177	Q8N6Q3	VAR_079755	p.Gly261Ala	LB/B	-	-
CD177	Q8N6Q3	VAR_079756	p.Gly431Arg	LB/B	rs78718189	-
CD180	Q99467	VAR_021978	p.Asn53Lys	LB/B	rs16875312	-
CD180	Q99467	VAR_021979	p.Ser99Arg	LB/B	rs2230520	-
CD180	Q99467	VAR_057298	p.Val20Leu	LB/B	rs5744463	-
CD180	Q99467	VAR_057299	p.Asp259Asn	LB/B	rs5744525	-
CD180	Q99467	VAR_057300	p.Thr430Ala	LB/B	rs2230523	-
CD180	Q99467	VAR_057301	p.Phe648Leu	LB/B	rs2230524	-

CD180	Q99467	VAR_061859	p.Val356Met	LB/B	rs56752081	-
CD19	P15391	VAR_026963	p.Leu174Val	LB/B	rs2904880	-
CD19	P15391	VAR_036987	p.Arg514His	LB/B	rs34763945	-
CD1A	P06126	VAR_010209	p.Thr30Ile	LB/B	rs2269714	-
CD1A	P06126	VAR_010210	p.Cys68Trp	LB/B	rs2269715	-
CD1A	P06126	VAR_062522	p.Lys22Asn	LB/B	rs3087217	-
CD1C	P29017	VAR_031564	p.Asn70Thr	LB/B	rs3138100	-
CD1C	P29017	VAR_031565	p.Phe300Ser	LB/B	rs3138105	-
CD1D	P15813	VAR_010211	p.Thr64Ser	LB/B	rs62621276	-
CD1E	P15812	VAR_010191	p.Gln106Arg	LB/B	rs1065457	-
CD1E	P15812	VAR_010192	p.Arg164Trp	LB/B	rs199655202	-
CD1E	P15812	VAR_010193	p.Leu194Pro	LB/B	rs200741122	-
CD1E	P15812	VAR_056035	p.Gly15Glu	LB/B	rs3180089	-
CD1E	P15812	VAR_058324	p.His102Arg	LB/B	rs2873587	-
CD1E	P15812	VAR_058325	p.Ser149Asn	LB/B	rs35116276	-
CD2	P06729	VAR_017104	p.His266Gln	LB/B	rs699738	-
CD2	P06729	VAR_033608	p.His339Asn	LB/B	rs35880225	-
CD2	P06729	VAR_035504	p.Cys217Tyr	US	-	A breast cancer sample
CD200	P41217	VAR_027605	p.Ser11Cys	LB/B	rs1131199	-
CD200	P41217	VAR_027606	p.Pro46Thr	LB/B	rs2272022	-
CD200	P41217	VAR_056110	p.Val76Gly	LB/B	rs35465733	-
CD200R1	Q8TD46	VAR_014352	p.Arg89Lys	LB/B	rs2171509	-
CD200R1	Q8TD46	VAR_014353	p.Pro121Thr	LB/B	rs4596117	-
CD200R1	Q8TD46	VAR_014354	p.Gln177His	LB/B	rs9826308	-
CD200R1	Q8TD46	VAR_031022	p.Glu312Gln	LB/B	rs9865242	-
CD200R1L	Q6Q8B3	VAR_045897	p.Arg113Leu	LB/B	rs4682119	-
CD207	Q9UJ71	VAR_054781	p.Ala55Val	LB/B	rs10489990	-
CD207	Q9UJ71	VAR_054782	p.Pro213Ser	LB/B	rs17006436	-
CD207	Q9UJ71	VAR_054783	p.Val278Ala	LB/B	rs741326	-
CD207	Q9UJ71	VAR_054784	p.Asn288Asp	LB/B	rs13383830	-
CD207	Q9UJ71	VAR_056151	p.Gln136Glu	LB/B	rs17718987	-
CD207	Q9UJ71	VAR_059448	p.Ala300Pro	LB/B	rs2080391	-
CD207	Q9UJ71	VAR_063828	p.Trp264Arg	LP/P	rs200837270	Birbeck granule deficiency (BIRGD) [MIM:613393]
CD209	Q9NNX6	VAR_036689	p.Glu214Asp	LB/B	rs11465377	-
CD209	Q9NNX6	VAR_036690	p.Leu242Val	LB/B	rs11465380	-
CD209	Q9NNX6	VAR_050104	p.Glu168Asp	LB/B	rs1003686123	-
CD209	Q9NNX6	VAR_050105	p.Ala382Ser	LB/B	rs11465393	-
CD22	P20273	VAR_003913	p.Gln152Glu	LB/B	rs554866571	-
CD22	P20273	VAR_003914	p.Ser664Gly	LB/B	rs17719289	-
CD22	P20273	VAR_003915	p.Arg669Cys	LB/B	rs749980313	-
CD22	P20273	VAR_003916	p.Gly745Asp	LB/B	rs10406069	-
CD22	P20273	VAR_014133	p.Ala34Thr	LB/B	rs201453271	-
CD22	P20273	VAR_014134	p.Glu203Lys	LB/B	rs752024645	-
CD22	P20273	VAR_014135	p.Tyr639His	LB/B	rs1058407	-
CD22	P20273	VAR_049903	p.Gly551Arg	LB/B	rs35715143	-
CD226	Q15762	VAR_018632	p.Ser307Gly	LB/B	rs763361	-
CD24	P25063	VAR_016156	p.Ala57Val	LB/B	rs878859113	-
CD24	P25063	VAR_031576	p.Ser44Thr	LB/B	-	-
CD244	Q9BZW8	VAR_056036	p.Asn89Asp	LB/B	rs34846692	-

CD244 Q9BZW8 VAR_056037 p.Ser323Phe LB/B rs12064925 -
CD248 Q9HCU0 VAR_025013 p.His457Arg LB/B rs3741367 -
CD248 Q9HCU0 VAR_036399 p.Leu6Phe US - A colorectal cancer sample
CD27 P26842 VAR_016148 p.Ala59Thr LB/B rs25680 -
CD27 P26842 VAR_052348 p.His233Arg LB/B rs2532502 -
CD27 P26842 VAR_069793 p.Cys53Tyr LP/P rs397514667 Lymphoproliferative syndrome 2 (LPFS2) [MIM:615122]
CD276 Q5ZPR3 VAR_049857 p.Pro97Leu LB/B rs7173448 -
CD276 Q5ZPR3 VAR_049858 p.Arg111Ser LB/B rs7173476 -
CD276 Q5ZPR3 VAR_049859 p.Gln137Leu LB/B rs11574477 -
CD276 Q5ZPR3 VAR_049860 p.Thr160Met LB/B rs11574479 -
CD276 Q5ZPR3 VAR_049861 p.Arg267His LB/B rs11574483 -
CD276 Q5ZPR3 VAR_049862 p.Ala279Thr LB/B rs10083681 -
CD276 Q5ZPR3 VAR_049863 p.Pro315Leu LB/B rs148625372 -
CD276 Q5ZPR3 VAR_049864 p.Arg329Ser LB/B rs7173476 -
CD276 Q5ZPR3 VAR_049865 p.Thr378Met LB/B rs145827704 -
CD2AP Q9Y5K6 VAR_033672 p.Asn581Lys LB/B rs34069459 -
CD2BP2 O95400 VAR_050772 p.Gly231Asp LB/B rs13330462 -
CD2BP2 O95400 VAR_050773 p.Thr262Ile LB/B rs34391305 -
CD300A Q9UGN4 VAR_030797 p.Arg111Gln LB/B rs2272111 -
CD300C Q08708 VAR_039133 p.Thr71Ile LB/B rs11870245 -
CD300E Q496F6 VAR_039129 p.Lys19Thr LB/B rs581157 -
CD300E Q496F6 VAR_039130 p.Thr27Ala US rs1278044712 A colorectal cancer sample
CD300E Q496F6 VAR_039131 p.Gly158Arg LB/B rs1878061 -
CD300LD Q6UXZ3 VAR_059386 p.Ser6Ala LB/B rs493430 -
CD300LD Q6UXZ3 VAR_059387 p.Val89Met LB/B rs783239 -
CD300LF Q8TDQ1 VAR_027152 p.Gln218Arg LB/B rs2034310 -
CD300LF Q8TDQ1 VAR_039128 p.Val19Ala LB/B rs35489971 -
CD300LG Q6UXG3 VAR_035261 p.Asp221Asn LB/B rs17852267 -
CD300LG Q6UXG3 VAR_035262 p.Thr228Ala LB/B rs12453522 -
CD302 Q8IX05 VAR_050101 p.Arg200Ser LB/B rs34068933 -
CD320 Q9NPF0 VAR_047315 p.Gln8Arg LB/B rs2232775 -
CD320 Q9NPF0 VAR_047316 p.Gly220Arg LB/B rs2336573 -
CD320 Q9NPF0 VAR_077921 p.Ser142Gly LB/B - -
CD33 P20138 VAR_028260 p.Arg69Gly LB/B rs2455069 -
CD33 P20138 VAR_028261 p.Arg202Trp LB/B rs4082929 -
CD33 P20138 VAR_028262 p.Ile242Leu LB/B rs988337 -
CD33 P20138 VAR_028263 p.Phe243Leu LB/B rs11882250 -
CD33 P20138 VAR_028264 p.Val294Leu LB/B rs2271652 -
CD33 P20138 VAR_049904 p.Ala14Val LB/B rs12459419 -
CD33 P20138 VAR_049905 p.Trp22Arg LB/B rs35814802 -
CD33 P20138 VAR_049906 p.Ser128Asn LB/B rs34919259 -
CD33 P20138 VAR_049907 p.Gly304Arg LB/B rs35112940 -
CD33 P20138 VAR_049908 p.Thr331Ala LB/B rs35632246 -
CD33 P20138 VAR_061319 p.Val267Ile LB/B rs58981829 -
CD34 P28906 VAR_050774 p.Ala367Ser LB/B rs28362497 -
CD36 P16671 VAR_013918 p.Val154Phe LB/B rs5957 -
CD36 P16671 VAR_017913 p.Pro90Ser LP/P rs75326924 Platelet glycoprotein IV deficiency (PG4D) [MIM:608404]

CD36 P16671 VAR_017914 p.Glu123Lys LB/B rs183461468 -
CD36 P16671 VAR_017915 p.Thr174Ala LB/B rs756525492 -
CD36 P16671 VAR_017917 p.Phe254Leu LP/P rs142186404 Platelet glycoprotein IV deficiency (PG4D) [MIM:608404]
CD36 P16671 VAR_017918 p.Ile271Thr LB/B rs370072057 -
CD36 P16671 VAR_017919 p.Ile413Leu LP/P rs121918035 Platelet glycoprotein IV deficiency (PG4D) [MIM:608404]
CD36 P16671 VAR_019049 p.Ser127Leu LB/B rs201765331 -
CD36 P16671 VAR_071161 p.Arg386Trp LB/B rs148910227 -
CD36 P16671 VAR_071162 p.Thr470Ile LB/B rs200771788 -
CD38 P28907 VAR_001323 p.Arg140Trp LB/B rs1800561 -
CD3D P04234 VAR_049646 p.Gln147Arg LB/B rs45510201 -
CD3G P09693 VAR_049854 p.Val131Phe LB/B rs3753058 -
CD4 P01730 VAR_003906 p.Arg265Trp LB/B rs28919570 -
CD4 P01730 VAR_023459 p.Lys191Glu LB/B rs28917504 -
CD4 P01730 VAR_023460 p.Phe227Ser LB/B rs11064419 -
CD40 P25942 VAR_013628 p.Cys83Arg LP/P rs28931586 Immunodeficiency with hyper-IgM 3 (HIGM3) [MIM:606843]
CD40 P25942 VAR_018751 p.Ser124Leu LB/B rs11569321 -
CD40 P25942 VAR_018752 p.Pro227Ala LB/B rs11086998 -
CD40 P25942 VAR_039301 p.Cys26Gln US - Bladder carcinoma cell line Hu549
CD40 P25942 VAR_039302 p.Ser35Gly US rs750234130 Bladder carcinoma cell line Hu549
CD40 P25942 VAR_039303 p.Ser39Thr US - Bladder carcinoma cell line Hu549
CD40 P25942 VAR_077569 p.Cys37Gly LP/P - Immunodeficiency with hyper-IgM 3 (HIGM3) [MIM:606843]
CD40LG P29965 VAR_007513 p.Met36Arg LP/P rs104894774 Immunodeficiency with hyper-IgM, type 1 (HIGM1) [MIM:308230]
CD40LG P29965 VAR_007514 p.Ala123Glu LP/P rs104894778 Immunodeficiency with hyper-IgM, type 1 (HIGM1) [MIM:308230]
CD40LG P29965 VAR_007515 p.Val126Ala LP/P - Immunodeficiency with hyper-IgM, type 1 (HIGM1) [MIM:308230]
CD40LG P29965 VAR_007517 p.Trp140Cys LP/P - Immunodeficiency with hyper-IgM, type 1 (HIGM1) [MIM:308230]
CD40LG P29965 VAR_007518 p.Trp140Gly LP/P rs104894777 Immunodeficiency with hyper-IgM, type 1 (HIGM1) [MIM:308230]
CD40LG P29965 VAR_007519 p.Trp140Arg LP/P - Immunodeficiency with hyper-IgM, type 1 (HIGM1) [MIM:308230]
CD40LG P29965 VAR_007520 p.Gly144Glu LP/P rs886039326 Immunodeficiency with hyper-IgM, type 1 (HIGM1) [MIM:308230]
CD40LG P29965 VAR_007521 p.Leu155Pro LP/P rs104894769 Immunodeficiency with hyper-IgM, type 1 (HIGM1) [MIM:308230]
CD40LG P29965 VAR_007522 p.Thr211Asn LP/P rs1569377829 Immunodeficiency with hyper-IgM, type 1 (HIGM1) [MIM:308230]
CD40LG P29965 VAR_007523 p.Gly219Arg LB/B rs148594123 -
CD40LG P29965 VAR_007524 p.Gly227Val LP/P rs104894768 Immunodeficiency with hyper-IgM, type 1 (HIGM1) [MIM:308230]
CD40LG P29965 VAR_007526 p.Leu231Ser LP/P - Immunodeficiency with hyper-IgM, type 1 (HIGM1) [MIM:308230]

CD40LG P29965 VAR_007527 p.Ala235Pro LP/P rs104894771 Immunodeficiency with hyper-IgM, type 1 (HIGM1) [MIM:308230]
CD40LG P29965 VAR_007528 p.Thr254Met LP/P rs193922136 Immunodeficiency with hyper-IgM, type 1 (HIGM1) [MIM:308230]
CD40LG P29965 VAR_017922 p.Thr147Asn LP/P rs1057521127 Immunodeficiency with hyper-IgM, type 1 (HIGM1) [MIM:308230]
CD40LG P29965 VAR_017923 p.Tyr170Cys LP/P rs756468554 Immunodeficiency with hyper-IgM, type 1 (HIGM1) [MIM:308230]
CD40LG P29965 VAR_017924 p.Leu258Ser LP/P rs1569377884 Immunodeficiency with hyper-IgM, type 1 (HIGM1) [MIM:308230]
CD40LG P29965 VAR_017925 p.Gly38Arg LP/P - Immunodeficiency with hyper-IgM, type 1 (HIGM1) [MIM:308230]
CD40LG P29965 VAR_017926 p.His125Arg LP/P - Immunodeficiency with hyper-IgM, type 1 (HIGM1) [MIM:308230]
CD40LG P29965 VAR_017927 p.Gln174Arg LP/P - Immunodeficiency with hyper-IgM, type 1 (HIGM1) [MIM:308230]
CD40LG P29965 VAR_017928 p.Gly257Ser LP/P - Immunodeficiency with hyper-IgM, type 1 (HIGM1) [MIM:308230]
CD40LG P29965 VAR_017929 p.Gly116Arg LP/P - Immunodeficiency with hyper-IgM, type 1 (HIGM1) [MIM:308230]
CD40LG P29965 VAR_017930 p.Gly116Ser LP/P - Immunodeficiency with hyper-IgM, type 1 (HIGM1) [MIM:308230]
CD40LG P29965 VAR_017931 p.Val126Asp LP/P - Immunodeficiency with hyper-IgM, type 1 (HIGM1) [MIM:308230]
CD40LG P29965 VAR_017932 p.Lys143Thr LP/P - Immunodeficiency with hyper-IgM, type 1 (HIGM1) [MIM:308230]
CD40LG P29965 VAR_017933 p.Ala173Asp LP/P - Immunodeficiency with hyper-IgM, type 1 (HIGM1) [MIM:308230]
CD40LG P29965 VAR_017934 p.Thr176Ile LP/P - Immunodeficiency with hyper-IgM, type 1 (HIGM1) [MIM:308230]
CD40LG P29965 VAR_017935 p.Leu195Pro LP/P - Immunodeficiency with hyper-IgM, type 1 (HIGM1) [MIM:308230]
CD40LG P29965 VAR_017936 p.Ala208Asp LP/P - Immunodeficiency with hyper-IgM, type 1 (HIGM1) [MIM:308230]
CD40LG P29965 VAR_017937 p.His224Tyr LP/P - Immunodeficiency with hyper-IgM, type 1 (HIGM1) [MIM:308230]
CD40LG P29965 VAR_017938 p.Gly226Ala LP/P - Immunodeficiency with hyper-IgM, type 1 (HIGM1) [MIM:308230]
CD40LG P29965 VAR_017939 p.Val237Glu LP/P - Immunodeficiency with hyper-IgM, type 1 (HIGM1) [MIM:308230]
CD40LG P29965 VAR_017940 p.Gly257Asp LP/P rs1477466218 Immunodeficiency with hyper-IgM, type 1 (HIGM1) [MIM:308230]
CD44 P16070 VAR_006490 p.Arg46Pro LB/B rs369473842 -
CD44 P16070 VAR_021147 p.Lys417Arg LB/B rs9666607 -
CD44 P16070 VAR_030325 p.Thr393Met LB/B rs11607491 -
CD44 P16070 VAR_030326 p.Ile479Thr LB/B rs1467558 -
CD44 P16070 VAR_030327 p.Asp494His LB/B rs12273397 -
CD46 P15529 VAR_022262 p.Asp266Asn LB/B rs17006830 -
CD46 P15529 VAR_022263 p.Pro324Leu LB/B rs41317833 -

CD46 P15529 VAR_022264 p.Ala353Val LB/B rs35366573 -
CD46 P15529 VAR_022265 p.Val355Gly LB/B - -
CD46 P15529 VAR_026567 p.Ser13Phe LB/B rs138843816 -
CD46 P15529 VAR_026568 p.Arg59Gln LB/B rs780693519 -
CD46 P15529 VAR_026569 p.Pro165Ser LP/P rs759136081 Hemolytic uremic syndrome atypical 2 (AHUS2) [MIM:612922]
CD46 P15529 VAR_026570 p.Ser240Pro LP/P rs121909589 Hemolytic uremic syndrome atypical 2 (AHUS2) [MIM:612922]
CD46 P15529 VAR_035828 p.Cys228Tyr US - A colorectal cancer sample
CD46 P15529 VAR_063656 p.Cys35Tyr LP/P rs121909591 Hemolytic uremic syndrome atypical 2 (AHUS2) [MIM:612922]
CD46 P15529 VAR_063657 p.Trp216Cys LP/P - Hemolytic uremic syndrome atypical 2 (AHUS2) [MIM:612922]
CD46 P15529 VAR_063658 p.Pro231Arg LP/P rs1271761432 Hemolytic uremic syndrome atypical 2 (AHUS2) [MIM:612922]
CD48 P09326 VAR_020082 p.Glu102Gln LB/B rs2295615 -
CD48 P09326 VAR_049909 p.Leu241Ser LB/B rs16832307 -
CD5 P06127 VAR_020411 p.Pro224Leu LB/B rs2241002 -
CD5 P06127 VAR_024649 p.His461Arg LB/B rs637186 -
CD5 P06127 VAR_058203 p.Ala471Val LB/B rs2229177 -
CD52 P31358 VAR_014838 p.Ile41Met LB/B rs17645 -
CD52 P31358 VAR_050775 p.Asn40Ser LB/B rs1071849 -
CD55 P08174 VAR_001997 p.Arg52Leu LB/B rs28371588 -
CD55 P08174 VAR_001998 p.Arg52Pro LB/B rs28371588 -
CD55 P08174 VAR_001999 p.Leu82Arg LB/B rs147474393 -
CD55 P08174 VAR_002000 p.Ser199Leu LB/B rs1135402914 -
CD55 P08174 VAR_002001 p.Ala227Pro LB/B rs60822373 -
CD55 P08174 VAR_015884 p.Arg240His LB/B rs199705465 -
CD55 P08174 VAR_079373 p.Cys267Ser LP/P rs1135402917 Complement hyperactivation, angiopathic thrombosis, and protein-losing enteropathy (CHAPLE) [MIM:226300]
CD58 P19256 VAR_049885 p.Ser15Gly LB/B rs17426456 -
CD59 P13987 VAR_070124 p.Cys89Tyr LP/P rs397514767 Hemolytic anemia, CD59-mediated, with or without polyneuropathy (HACD59) [MIM:612300]
CD5L O43866 VAR_033728 p.Asp117Glu LB/B rs11537583 -
CD6 P30203 VAR_057202 p.Arg225Trp LB/B rs11230563 -
CD6 P30203 VAR_057203 p.Ala271Thr LB/B rs12360861 -
CD6 P30203 VAR_057204 p.Ser351Asn LB/B rs34974368 -
CD6 P30203 VAR_057205 p.Gly606Ser LB/B rs2074233 -
CD6 P30203 VAR_059809 p.Thr217Met LB/B rs11230562 -
CD6 P30203 VAR_060790 p.Ala257Val LB/B rs2074225 -
CD68 P34810 VAR_016144 p.Gln254Lys LB/B rs9901673 -
CD68 P34810 VAR_016145 p.Ala340Thr LB/B rs17607 -
CD68 P34810 VAR_049725 p.Ile329Thr LB/B rs35452170 -
CD68 P34810 VAR_049726 p.Ala350Thr LB/B rs9901675 -
CD7 P09564 VAR_049855 p.Thr113Ala LB/B rs34579511 -
CD72 P21854 VAR_033729 p.Pro234Leu LB/B rs34791102 -
CD79B P40259 VAR_057833 p.Gly137Ser LP/P rs121912424 Agammaglobulinemia 6, autosomal recessive (AGM6) [MIM:612692]
CD82 P27701 VAR_052326 p.Ile241Val LB/B rs1139971 -

CD83 Q01151 VAR_033609 p.Arg182Gln LB/B rs2230193 -
CD86 P42081 VAR_014650 p.Ala310Thr LB/B rs1129055 -
CD86 P42081 VAR_021916 p.Ser170Asn LB/B rs9282642 -
CD86 P42081 VAR_021917 p.Asp323Asn LB/B rs9282648 -
CD86 P42081 VAR_055003 p.Val185Ile LB/B rs2681417 -
CD8A P01732 VAR_021020 p.Gly111Ser LP/P rs121918660 CD8 deficiency, familial (CD8 deficiency) [MIM:608957]
CD93 Q9NPY3 VAR_013573 p.Val318Ala LB/B - -
CD93 Q9NPY3 VAR_036400 p.Ala220Val US rs138932459 A colorectal cancer sample
CD93 Q9NPY3 VAR_050102 p.Pro541Ser LB/B rs3746731 -
CD96 P40200 VAR_021928 p.Ala142Pro LB/B rs2276872 -
CD96 P40200 VAR_037578 p.Thr280Met LP/P rs119477056 C syndrome (CSYN) [MIM:211750]
CD99 P14209 VAR_014733 p.Met166Val LB/B rs4793 -
CD99 P14209 VAR_014734 p.Asn173Ile LB/B rs4717 -
CDA P32320 VAR_021559 p.Lys27Gln LB/B rs2072671 -
CDAN1 Q8IWY9 VAR_017218 p.Asn599Ser LP/P rs120074166 Anemia, congenital dyserythropoietic, 1A (CDAN1A) [MIM:224120]
CDAN1 Q8IWY9 VAR_017219 p.Pro672Leu LP/P rs120074167 Anemia, congenital dyserythropoietic, 1A (CDAN1A) [MIM:224120]
CDAN1 Q8IWY9 VAR_017220 p.Glu698Lys LP/P - Anemia, congenital dyserythropoietic, 1A (CDAN1A) [MIM:224120]
CDAN1 Q8IWY9 VAR_017221 p.Arg714Trp LP/P rs80338696 Anemia, congenital dyserythropoietic, 1A (CDAN1A) [MIM:224120]
CDAN1 Q8IWY9 VAR_017222 p.Phe868Ile LP/P rs120074168 Anemia, congenital dyserythropoietic, 1A (CDAN1A) [MIM:224120]
CDAN1 Q8IWY9 VAR_017223 p.Val869Met LP/P rs370895637 Anemia, congenital dyserythropoietic, 1A (CDAN1A) [MIM:224120]
CDAN1 Q8IWY9 VAR_017224 p.Arg1042Trp LP/P rs80338697 Anemia, congenital dyserythropoietic, 1A (CDAN1A) [MIM:224120]
CDAN1 Q8IWY9 VAR_017225 p.Asp1043Val LP/P rs80338698 Anemia, congenital dyserythropoietic, 1A (CDAN1A) [MIM:224120]
CDAN1 Q8IWY9 VAR_017226 p.Pro1130Leu LP/P rs80338699 Anemia, congenital dyserythropoietic, 1A (CDAN1A) [MIM:224120]
CDAN1 Q8IWY9 VAR_056785 p.Gln596Arg LB/B rs12917189 -
CDAN1 Q8IWY9 VAR_056786 p.Arg891Cys LB/B rs8023524 -
CDAN1 Q8IWY9 VAR_059602 p.Gln107Leu LB/B rs4265781 -
CDC14A Q9UNH5 VAR_019957 p.Arg345Gln LB/B rs28364897 -
CDC14A Q9UNH5 VAR_019958 p.Ser589Phe LB/B rs28364923 -
CDC14A Q9UNH5 VAR_035655 p.Asp493Tyr US - A colorectal cancer sample
CDC14A Q9UNH5 VAR_081128 p.Arg312Gly LP/P rs148737918 Deafness, autosomal recessive, 32, with or without immotile sperm (DFNB32) [MIM:608653]
CDC14A Q9UNH5 VAR_081129 p.Arg312Gln LP/P rs369245990 Deafness, autosomal recessive, 32, with or without immotile sperm (DFNB32) [MIM:608653]
CDC14A Q9UNH5 VAR_081130 p.Gln320Pro LP/P rs1339709390 Deafness, autosomal recessive, 32, with or without immotile sperm (DFNB32) [MIM:608653]
CDC14B O60729 VAR_019959 p.Ile302Thr LB/B rs16911114 -
CDC14B O60729 VAR_019960 p.Ile341Thr LB/B rs16911075 -
CDC14C A4D256 VAR_038327 p.Pro131Ser LB/B rs1615556 -
CDC14C A4D256 VAR_038328 p.Ile189Leu LB/B rs421206 -

CDC20	Q12834	VAR_030368	p.Val402Met	LB/B	rs45443196	-
CDC20	Q12834	VAR_030369	p.Arg479Gln	LB/B	rs45461499	-
CDC20B	Q86Y33	VAR_043860	p.Thr8Pro	LB/B	rs173042	-
CDC20B	Q86Y33	VAR_043861	p.Glu17Lys	LB/B	rs423074	-
CDC20B	Q86Y33	VAR_043862	p.Thr97Ser	LB/B	rs34132993	-
CDC20B	Q86Y33	VAR_043863	p.Arg121Cys	LB/B	rs1021580	-
CDC20B	Q86Y33	VAR_043864	p.Ser496Ala	LB/B	rs3104230	-
CDC20B	Q86Y33	VAR_043865	p.Arg503Trp	LB/B	rs444527	-
CDC23	Q9UJX2	VAR_019232	p.Glu78Gln	LB/B	rs17228304	-
CDC23	Q9UJX2	VAR_024675	p.Pro9Leu	LB/B	rs2231471	-
CDC25A	P30304	VAR_020932	p.Ser88Phe	LB/B	rs3731499	-
CDC25A	P30304	VAR_023532	p.Arg182Gly	LB/B	rs6771386	-
CDC25A	P30304	VAR_023533	p.Arg182Trp	LB/B	rs6771386	-
CDC25B	P30305	VAR_020933	p.Glu548Lys	LB/B	rs11570019	-
CDC25C	P30307	VAR_020146	p.Gly297Arg	LB/B	rs11567997	-
CDC25C	P30307	VAR_027922	p.Ser14Asn	LB/B	rs11567959	-
CDC25C	P30307	VAR_027923	p.Arg70Cys	LB/B	rs3734166	-
CDC25C	P30307	VAR_027924	p.Ser78Asn	LB/B	rs11567962	-
CDC27	P30260	VAR_014489	p.Tyr496His	LB/B	rs202052665	-
CDC27	P30260	VAR_035861	p.Gly270Ala	US	-	A breast cancer sample
CDC34	P49427	VAR_021277	p.Asp227His	LB/B	rs16990650	-
CDC37	Q16543	VAR_022220	p.Gly360Glu	LB/B	rs280528	-
CDC37L1	Q7L3B6	VAR_038755	p.Ser291Phe	LB/B	rs7036014	-
CDC42	P60953	VAR_076337	p.Tyr64Cys	LP/P	rs864309721	Takenouchi-Kosaki syndrome (TKS) [MIM:616737]
CDC42BPA	Q5VT25	VAR_040830	p.Glu50Lys	US	-	A lung neuroendocrine carcinoma sample
CDC42BPA	Q5VT25	VAR_040831	p.Thr231Met	LB/B	rs34614709	-
CDC42BPA	Q5VT25	VAR_040832	p.Ile537Thr	LB/B	rs56364976	-
CDC42BPA	Q5VT25	VAR_040833	p.Ile1418Lys	LB/B	-	-
CDC42BPA	Q5VT25	VAR_045583	p.Thr780Met	LB/B	rs56119119	-
CDC42BPA	Q5VT25	VAR_045584	p.Tyr790Cys	LB/B	rs34943764	-
CDC42BPA	Q5VT25	VAR_045585	p.Ala1148Thr	LB/B	rs56219089	-
CDC42BPA	Q5VT25	VAR_045586	p.Arg1211His	LB/B	rs961490	-
CDC42BPA	Q5VT25	VAR_045587	p.Val1317Ile	LB/B	rs1929860	-
CDC42BPA	Q5VT25	VAR_045588	p.Ala1469Val	LB/B	rs55687355	-
CDC42BPA	Q5VT25	VAR_045589	p.Thr1618Ala	LB/B	rs2297417	-
CDC42BPA	Q5VT25	VAR_045590	p.Ala1699Val	LB/B	rs2802269	-
CDC42BPA	Q5VT25	VAR_057104	p.Ala1712Val	LB/B	rs2802269	-
CDC42BPB	Q9Y5S2	VAR_025847	p.Ile1077Val	LB/B	rs34822377	-
CDC42BPB	Q9Y5S2	VAR_040834	p.Lys500Glu	US	rs1158962067	A breast infiltrating ductal carcinoma sample
CDC42BPB	Q9Y5S2	VAR_040835	p.Arg555Gln	LB/B	rs36001612	-
CDC42BPB	Q9Y5S2	VAR_040836	p.Arg671Gln	LB/B	rs55948035	-
CDC42BPB	Q9Y5S2	VAR_040837	p.Arg876Trp	US	-	A colorectal adenocarcinoma sample
CDC42BPB	Q9Y5S2	VAR_040838	p.Glu1315Lys	US	-	A lung large cell carcinoma sample
CDC42BPB	Q9Y5S2	VAR_040839	p.Ser1633Tyr	LB/B	rs56412851	-
CDC42BPB	Q9Y5S2	VAR_070886	p.Arg1203Lys	LB/B	rs146298297	-
CDC42BPG	Q6DT37	VAR_040840	p.Pro168Leu	LB/B	rs34454471	-
CDC42BPG	Q6DT37	VAR_040841	p.Ser280Phe	US	rs770462360	A glioblastoma multiforme sample
CDC42BPG	Q6DT37	VAR_040842	p.Thr362Pro	LB/B	rs55688429	-
CDC42BPG	Q6DT37	VAR_040843	p.Ala537Asp	LB/B	rs34241745	-

CDC42BPG Q6DT37 VAR_057105 p.Gln1135Arg LB/B rs3741395 -
CDC42EP2 O14613 VAR_023001 p.Asn176Ser LB/B rs4149839 -
CDC42EP2 O14613 VAR_023002 p.Ile191Phe LB/B rs7120634 -
CDC45 O75419 VAR_019286 p.Val81Ile LB/B rs13447203 -
CDC45 O75419 VAR_019287 p.Val376Met LB/B rs13447263 -
CDC45 O75419 VAR_053026 p.Met356Arg LB/B rs17209274 -
CDC45 O75419 VAR_080963 p.Gln68Arg LP/P rs879255633 Meier-Gorlin syndrome 7 (MGORS7) [MIM:617063]
CDC45 O75419 VAR_080964 p.Asn76His LP/P rs879255632 Meier-Gorlin syndrome 7 (MGORS7) [MIM:617063]
CDC45 O75419 VAR_080965 p.Glu155Gly US rs9606030 Meier-Gorlin syndrome 7 (MGORS7) [MIM:617063]
CDC45 O75419 VAR_080966 p.Arg157Cys LP/P rs540217942 Meier-Gorlin syndrome 7 (MGORS7) [MIM:617063]
CDC45 O75419 VAR_080967 p.Asp226Gly LP/P rs754080445 Meier-Gorlin syndrome 7 (MGORS7) [MIM:617063]
CDC45 O75419 VAR_080968 p.Ser264Tyr US rs151279621 Meier-Gorlin syndrome 7 (MGORS7) [MIM:617063]
CDC45 O75419 VAR_080969 p.Ala298Val LP/P rs146559223 Meier-Gorlin syndrome 7 (MGORS7) [MIM:617063]
CDC45 O75419 VAR_080970 p.Pro321Thr US - Meier-Gorlin syndrome 7 (MGORS7) [MIM:617063]
CDC45 O75419 VAR_080972 p.Pro463Leu US rs751663397 Meier-Gorlin syndrome 7 (MGORS7) [MIM:617063]
CDC45 O75419 VAR_080973 p.Pro496Leu US rs1376596361 Meier-Gorlin syndrome 7 (MGORS7) [MIM:617063]
CDC45 O75419 VAR_080974 p.Arg554Trp US rs778665661 Meier-Gorlin syndrome 7 (MGORS7) [MIM:617063]
CDC5L Q99459 VAR_050181 p.Tyr459Cys LB/B rs11572006 -
CDC6 Q99741 VAR_019349 p.Thr238Ala LB/B rs4135010 -
CDC6 Q99741 VAR_019350 p.Asp295Asn LB/B rs4135012 -
CDC6 Q99741 VAR_019351 p.Thr299Met LB/B rs4135013 -
CDC6 Q99741 VAR_019352 p.Arg378His LB/B rs4135016 -
CDC6 Q99741 VAR_019353 p.Val441Ile LB/B rs13706 -
CDC6 Q99741 VAR_065493 p.Thr323Arg LP/P rs387906842 Meier-Gorlin syndrome 5 (MGORS5) [MIM:613805]
CDC7 O00311 VAR_019255 p.Gln23Pro LB/B rs13447459 -
CDC7 O00311 VAR_019256 p.Ile99Val LB/B rs13447492 -
CDC7 O00311 VAR_019257 p.Gly112Trp LB/B rs13447493 -
CDC7 O00311 VAR_019258 p.Phe162Leu LB/B rs13447503 -
CDC7 O00311 VAR_019259 p.Lys441Arg LB/B rs13447539 -
CDC7 O00311 VAR_040403 p.Ile208Met LB/B rs34979509 -
CDC7 O00311 VAR_040404 p.Glu209Asp LB/B rs56327502 -
CDC7 O00311 VAR_040405 p.Thr472Ile LB/B rs56381770 -
CDC7 O00311 VAR_040406 p.Ser498Ala LB/B rs35055915 -
CDC73 Q6P1J9 VAR_024082 p.Leu64Pro LP/P rs121434264 Hyperparathyroidism 1 (HRPT1) [MIM:145000]
CDC73 Q6P1J9 VAR_031825 p.Leu384Pro LB/B rs35590728 -
CDC73 Q6P1J9 VAR_064927 p.Ala2Ser US - -
CDC73 Q6P1J9 VAR_064929 p.Lys34Gln US - -
CDC73 Q6P1J9 VAR_064930 p.Ser59Phe US - -
CDC73 Q6P1J9 VAR_064931 p.Leu63Pro US rs1060500015 Hyperparathyroidism 1 (HRPT1) [MIM:145000]

CDC73	Q6P1J9	VAR_064932	p.Arg91Pro	US	-	-
CDC73	Q6P1J9	VAR_064933	p.Leu95Pro	US	-	Hyperparathyroidism 1 (HRPT1) [MIM:145000]
CDC73	Q6P1J9	VAR_064934	p.Asn272Ser	LB/B	rs752383339	-
CDC73	Q6P1J9	VAR_064935	p.Arg292Lys	US	-	-
CDC73	Q6P1J9	VAR_064936	p.Asp379Asn	LP/P	rs971586985	Hyperparathyroidism 2 with jaw tumors (HRPT2) [MIM:145001]
CDCA2	Q69YH5	VAR_032350	p.Val717Ile	LB/B	rs4872318	-
CDCA2	Q69YH5	VAR_032351	p.Arg884Ser	LB/B	rs3829009	-
CDCA5	Q96FF9	VAR_050777	p.Ser156Tyr	LB/B	rs34020666	-
CDCA7	Q9BWT1	VAR_076578	p.Arg274Cys	LP/P	rs879253738	Immunodeficiency-centromeric instability-facial anomalies syndrome 3 (ICF3) [MIM:616910]
CDCA7	Q9BWT1	VAR_076579	p.Arg274His	LP/P	rs370384522	Immunodeficiency-centromeric instability-facial anomalies syndrome 3 (ICF3) [MIM:616910]
CDCA7	Q9BWT1	VAR_076580	p.Gly294Val	US	-	Immunodeficiency-centromeric instability-facial anomalies syndrome 3 (ICF3) [MIM:616910]
CDCA7	Q9BWT1	VAR_076581	p.Arg304His	US	rs772929976	Immunodeficiency-centromeric instability-facial anomalies syndrome 3 (ICF3) [MIM:616910]
CDCA7L	Q96GN5	VAR_050776	p.Arg187Ser	LB/B	rs35281045	-
CDCA7L	Q96GN5	VAR_083475	p.Asp45Asn	LB/B	rs937337760	-
CDCA8	Q53HL2	VAR_027063	p.Lys12Asn	LB/B	rs17851453	-
CDCP1	Q9H5V8	VAR_025498	p.Gln525Arg	LB/B	rs3749191	-
CDCP1	Q9H5V8	VAR_025499	p.Asp709Gly	LB/B	rs9874077	-
CDCP1	Q9H5V8	VAR_055095	p.Ala673Val	LB/B	rs35428731	-
CDCP2	Q5VXM1	VAR_035162	p.Gly244Arg	LB/B	rs3766465	-
CDH1	P12830	VAR_001306	p.His123Tyr	US	-	A gastric cancer sample
CDH1	P12830	VAR_001307	p.Thr193Pro	US	-	A diffuse gastric cancer sample
CDH1	P12830	VAR_001309	p.Asn315Ser	US	-	Lobular breast carcinoma
CDH1	P12830	VAR_001310	p.Glu336Asp	LB/B	rs267606712	-
CDH1	P12830	VAR_001311	p.Asp370Ala	US	-	A diffuse gastric cancer sample
CDH1	P12830	VAR_001314	p.Glu463Gln	US	-	A gastric carcinoma sample
CDH1	P12830	VAR_001315	p.Thr470Ile	LB/B	rs370864592	-
CDH1	P12830	VAR_001317	p.Val473Asp	US	-	A diffuse gastric cancer sample
CDH1	P12830	VAR_001318	p.Ala592Thr	US	rs35187787	A thyroid cancer sample
CDH1	P12830	VAR_001319	p.Arg598Gln	US	rs780759537	A gastric cancer sample
CDH1	P12830	VAR_001320	p.Ala617Thr	LB/B	rs33935154	-
CDH1	P12830	VAR_001321	p.Leu711Val	LB/B	rs121964871	-
CDH1	P12830	VAR_001322	p.Ser838Gly	US	rs121964872	An ovarian carcinoma sample
CDH1	P12830	VAR_008712	p.Asp244Gly	LP/P	rs1064794231	Hereditary diffuse gastric cancer (HDGC) [MIM:137215]
CDH1	P12830	VAR_008713	p.Val487Ala	LP/P	-	Hereditary diffuse gastric cancer (HDGC) [MIM:137215]
CDH1	P12830	VAR_013970	p.Ser270Ala	LB/B	rs587776399	-
CDH1	P12830	VAR_013971	p.Thr340Ala	LB/B	rs116093741	-
CDH1	P12830	VAR_021868	p.Leu630Val	LB/B	rs2276331	-
CDH1	P12830	VAR_021869	p.Cys695Arg	LB/B	rs9282655	-
CDH1	P12830	VAR_023357	p.Leu478Pro	LB/B	rs35520415	-
CDH1	P12830	VAR_023358	p.Val832Met	LP/P	rs35572355	Hereditary diffuse gastric cancer (HDGC) [MIM:137215]
CDH1	P12830	VAR_023359	p.Glu880Lys	LB/B	rs34507583	-
CDH1	P12830	VAR_033026	p.Met282Ile	US	rs200932258	A breast cancer sample

CDH1 P12830 VAR_033027 p.Asp777Asn US rs372989292 A breast cancer sample
CDH1 P12830 VAR_048500 p.Asp72Asn LB/B rs35606263 -
CDH1 P12830 VAR_048501 p.Ile393Asn LB/B rs34466743 -
CDH1 P12830 VAR_048502 p.Val473Ile LB/B rs36087757 -
CDH1 P12830 VAR_055431 p.Ala634Val LB/B rs121964878 -
CDH1 P12830 VAR_079392 p.Asp254Tyr LP/P rs1555515445 Blepharocheilodontic syndrome 1 (BCDS1) [MIM:119580]
CDH1 P12830 VAR_079393 p.Asp257Val LP/P - Blepharocheilodontic syndrome 1 (BCDS1) [MIM:119580]
CDH10 Q9Y6N8 VAR_028751 p.Ser413Phe LB/B rs1395027 -
CDH10 Q9Y6N8 VAR_036102 p.Val371Leu US - A breast cancer sample
CDH11 P55287 VAR_031945 p.Thr255Met LB/B rs35195 -
CDH11 P55287 VAR_031946 p.Met275Ile LB/B rs1130821 -
CDH11 P55287 VAR_031947 p.Ser373Ala LB/B rs35213 -
CDH12 P55289 VAR_048505 p.Val68Met LB/B rs4371716 -
CDH12 P55289 VAR_048506 p.Ile284Val LB/B rs17328673 -
CDH12 P55289 VAR_048507 p.Ile475Thr LB/B rs12108814 -
CDH13 P55290 VAR_030632 p.Leu121Ser LB/B rs7197352 -
CDH13 P55290 VAR_065747 p.Arg65Cys LB/B rs368685803 -
CDH13 P55290 VAR_065748 p.Ala103Val LB/B rs199539898 -
CDH13 P55290 VAR_065749 p.Gly113Arg LB/B rs183971768 -
CDH13 P55290 VAR_065750 p.Arg246Trp LB/B rs377210458 -
CDH13 P55290 VAR_065751 p.Glu367Gln LB/B rs200000145 -
CDH13 P55290 VAR_065752 p.Ala376Thr LB/B rs35549391 -
CDH13 P55290 VAR_065753 p.Leu643Arg LB/B rs34106627 -
CDH15 P55291 VAR_054966 p.Val8Leu US rs567903921 Mental retardation, autosomal dominant 3 (MRD3) [MIM:612580]
CDH15 P55291 VAR_054967 p.Arg60Cys LP/P rs121434539 Mental retardation, autosomal dominant 3 (MRD3) [MIM:612580]
CDH15 P55291 VAR_054968 p.Arg92Trp LP/P rs121434540 Mental retardation, autosomal dominant 3 (MRD3) [MIM:612580]
CDH15 P55291 VAR_054969 p.Ala122Val LP/P rs121434541 Mental retardation, autosomal dominant 3 (MRD3) [MIM:612580]
CDH16 O75309 VAR_021870 p.Leu191Phe LB/B rs2271024 -
CDH16 O75309 VAR_021871 p.His257Tyr LB/B rs2271023 -
CDH16 O75309 VAR_061058 p.Arg717His LB/B rs34621310 -
CDH17 Q12864 VAR_031694 p.Ile446Thr LB/B rs1131829 -
CDH17 Q12864 VAR_031695 p.Glu734Asp LB/B rs1051623 -
CDH17 Q12864 VAR_031696 p.Glu739Ala LB/B rs1051624 -
CDH17 Q12864 VAR_055567 p.Lys115Glu LB/B rs2243518 -
CDH2 P19022 VAR_028254 p.Ala21Thr LB/B rs17495042 -
CDH2 P19022 VAR_028255 p.Ala118Thr LB/B rs17445840 -
CDH2 P19022 VAR_028256 p.Ser196Thr LB/B rs1041970 -
CDH2 P19022 VAR_028257 p.Ile212Leu LB/B rs1041972 -
CDH2 P19022 VAR_028258 p.Asn845Ser LB/B rs2289664 -
CDH2 P19022 VAR_048503 p.Thr454Ala LB/B rs17857112 -
CDH2 P19022 VAR_084438 p.Val162Asp US - Agenesis of corpus callosum, cardiac, ocular, and genital syndrome (ACOGS) [MIM:618929]

CDH2 P19022 VAR_084439 p.Gln229Pro US - Arrhythmogenic right ventricular dysplasia, familial, 14 (ARVD14) [MIM:618920]
CDH2 P19022 VAR_084440 p.Asp353Asn LP/P - Agenesis of corpus callosum, cardiac, ocular, and genital syndrome (ACOGS) [MIM:618929]
CDH2 P19022 VAR_084441 p.Asp407Asn US - Arrhythmogenic right ventricular dysplasia, familial, 14 (ARVD14) [MIM:618920]
CDH2 P19022 VAR_084442 p.Asp525Gly US - Agenesis of corpus callosum, cardiac, ocular, and genital syndrome (ACOGS) [MIM:618929]
CDH2 P19022 VAR_084443 p.Asp597Asn LP/P - Agenesis of corpus callosum, cardiac, ocular, and genital syndrome (ACOGS) [MIM:618929]
CDH2 P19022 VAR_084444 p.Asp597Tyr LP/P - Agenesis of corpus callosum, cardiac, ocular, and genital syndrome (ACOGS) [MIM:618929]
CDH2 P19022 VAR_084445 p.Asn601Thr LP/P - Agenesis of corpus callosum, cardiac, ocular, and genital syndrome (ACOGS) [MIM:618929]
CDH2 P19022 VAR_084446 p.Pro603Ser US - Agenesis of corpus callosum, cardiac, ocular, and genital syndrome (ACOGS) [MIM:618929]
CDH2 P19022 VAR_084447 p.Cys613Trp LP/P - Agenesis of corpus callosum, cardiac, ocular, and genital syndrome (ACOGS) [MIM:618929]
CDH2 P19022 VAR_084448 p.Asp627Gly LP/P - Agenesis of corpus callosum, cardiac, ocular, and genital syndrome (ACOGS) [MIM:618929]
CDH2 P19022 VAR_084449 p.Tyr676Cys LP/P - Agenesis of corpus callosum, cardiac, ocular, and genital syndrome (ACOGS) [MIM:618929]
CDH20 Q9HBT6 VAR_036103 p.Met228Ile US - A breast cancer sample
CDH20 Q9HBT6 VAR_036104 p.Pro416Thr US - A breast cancer sample
CDH20 Q9HBT6 VAR_036105 p.Gln746His US - A breast cancer sample
CDH20 Q9HBT6 VAR_039119 p.Pro328His LB/B rs1943330 -
CDH20 Q9HBT6 VAR_039120 p.Gln371Arg LB/B rs35923922 -
CDH20 Q9HBT6 VAR_039121 p.Pro391Leu LB/B rs17068463 -
CDH23 Q9H251 VAR_012166 p.Arg3Cys LB/B rs7902757 -
CDH23 Q9H251 VAR_012167 p.Gly490Ala LB/B rs1227049 -
CDH23 Q9H251 VAR_012168 p.Ser496Asn LB/B rs10999947 -
CDH23 Q9H251 VAR_012169 p.Asp990Asn LP/P rs771766431 Deafness, autosomal recessive, 12 (DFNB12) [MIM:601386]
CDH23 Q9H251 VAR_012170 p.Ala1222Thr LB/B rs41281316 -
CDH23 Q9H251 VAR_012172 p.Arg1349Cys LB/B rs41281318 -
CDH23 Q9H251 VAR_012173 p.Asn1351Asp LB/B rs1227065 -
CDH23 Q9H251 VAR_012174 p.Gln1496His LP/P rs121908347 Usher syndrome 1D (USH1D) [MIM:601067]
CDH23 Q9H251 VAR_012175 p.Ala1575Thr LB/B rs1227051 -
CDH23 Q9H251 VAR_012176 p.Thr1671Ser LB/B rs749678546 -
CDH23 Q9H251 VAR_012177 p.Val1675Ile LB/B rs17712523 -
CDH23 Q9H251 VAR_012178 p.Arg1746Gln LP/P rs111033270 Usher syndrome 1D (USH1D) [MIM:601067]
CDH23 Q9H251 VAR_012179 p.Arg1804Gln LB/B rs3802711 -
CDH23 Q9H251 VAR_012180 p.Thr1999Ser LP/P rs11592462 Deafness, autosomal recessive, 12 (DFNB12) [MIM:601386]
CDH23 Q9H251 VAR_012181 p.Glu2044Lys LB/B rs10466026 -
CDH23 Q9H251 VAR_012182 p.Asp2045Asn LP/P rs121908348 Deafness, autosomal recessive, 12 (DFNB12) [MIM:601386]
CDH23 Q9H251 VAR_012183 p.Asp2202Asn LP/P rs121908349 Deafness, autosomal recessive, 12 (DFNB12) [MIM:601386]

CDH23 Q9H251 VAR_012184 p.Arg2358Gln LB/B rs4747194 -
CDH23 Q9H251 VAR_012185 p.Pro2380Leu LB/B rs4747195 -
CDH23 Q9H251 VAR_012186 p.Glu2588Gln LB/B rs41281338 -
CDH23 Q9H251 VAR_012187 p.Ile2950Asn LP/P rs752937051 Deafness, autosomal recessive, 12 (DFNB12) [MIM:601386]
CDH23 Q9H251 VAR_012188 p.Arg2956Cys LP/P rs751367894 Deafness, autosomal recessive, 12 (DFNB12) [MIM:601386]
CDH23 Q9H251 VAR_012189 p.Pro3059Thr LP/P rs780514498 Deafness, autosomal recessive, 12 (DFNB12) [MIM:601386]
CDH23 Q9H251 VAR_012190 p.Phe3125Leu LB/B rs45583140 -
CDH23 Q9H251 VAR_024030 p.Ala366Thr LB/B rs143282422 Usher syndrome 1D (USH1D) [MIM:601067]
CDH23 Q9H251 VAR_024031 p.Thr1209Ala LP/P rs41281314 Usher syndrome 1D (USH1D) [MIM:601067]
CDH23 Q9H251 VAR_024032 p.Arg1507Gln LP/P rs373480195 Usher syndrome 1D (USH1D) [MIM:601067]
CDH23 Q9H251 VAR_024033 p.Arg3189Trp LP/P rs121908353 Usher syndrome 1D (USH1D) [MIM:601067]
CDH23 Q9H251 VAR_024033 p.Arg3189Trp LP/P rs121908353 Usher syndrome 1D/F (USH1DF) [MIM:601067]
CDH23 Q9H251 VAR_024034 p.Ser3245Phe LP/P - Usher syndrome 1D (USH1D) [MIM:601067]
CDH23 Q9H251 VAR_027317 p.Asp124Gly LP/P rs751192273 Deafness, autosomal recessive, 12 (DFNB12) [MIM:601386]
CDH23 Q9H251 VAR_027318 p.Glu247Lys LP/P - Usher syndrome 1D (USH1D) [MIM:601067]
CDH23 Q9H251 VAR_027319 p.Asn452Ser LP/P rs375646885 Deafness, autosomal recessive, 12 (DFNB12) [MIM:601386]
CDH23 Q9H251 VAR_027320 p.Leu480Gln LP/P rs767928788 Deafness, autosomal recessive, 12 (DFNB12) [MIM:601386]
CDH23 Q9H251 VAR_027321 p.Ala484Pro LP/P - Usher syndrome 1D (USH1D) [MIM:601067]
CDH23 Q9H251 VAR_027322 p.Arg582Gln LP/P rs200263980 Deafness, autosomal recessive, 12 (DFNB12) [MIM:601386]
CDH23 Q9H251 VAR_027323 p.Arg1060Trp LP/P rs201536811 Deafness, autosomal recessive, 12 (DFNB12) [MIM:601386]
CDH23 Q9H251 VAR_027324 p.Gly1186Asp LP/P - Deafness, autosomal recessive, 12 (DFNB12) [MIM:601386]
CDH23 Q9H251 VAR_027325 p.Pro1206Arg LP/P - Usher syndrome 1D (USH1D) [MIM:601067]
CDH23 Q9H251 VAR_027326 p.Asp1341Asn LP/P rs121908351 Deafness, autosomal recessive, 12 (DFNB12) [MIM:601386]
CDH23 Q9H251 VAR_027327 p.Arg1437Gln LB/B rs56181447 -
CDH23 Q9H251 VAR_027328 p.Ala1586Pro LP/P rs573737471 Deafness, autosomal recessive, 12 (DFNB12) [MIM:601386]
CDH23 Q9H251 VAR_027329 p.Glu1595Lys LP/P rs778204574 Deafness, autosomal recessive, 12 (DFNB12) [MIM:601386]
CDH23 Q9H251 VAR_027330 p.Val1620Met LB/B rs41281330 -
CDH23 Q9H251 VAR_027331 p.Asp1846Asn LP/P rs746323558 Deafness, autosomal recessive, 12 (DFNB12) [MIM:601386]
CDH23 Q9H251 VAR_027332 p.Thr1887Ile LB/B rs397517340 -
CDH23 Q9H251 VAR_027333 p.Phe1888Ser LP/P rs121908352 Deafness, autosomal recessive, 12 (DFNB12) [MIM:601386]
CDH23 Q9H251 VAR_027334 p.Gly2017Ser LP/P rs183431253 Usher syndrome 1D (USH1D) [MIM:601067]
CDH23 Q9H251 VAR_027335 p.Arg2066Gln LB/B rs201887949 -
CDH23 Q9H251 VAR_027336 p.Asp2148Asn LP/P rs111033271 Deafness, autosomal recessive, 12 (DFNB12) [MIM:601386]
CDH23 Q9H251 VAR_027337 p.Val2283Ile LB/B rs41281334 -

CDH23 Q9H251 VAR_027338 p.Asp2376Asn LB/B rs9663920 -
CDH23 Q9H251 VAR_027339 p.Arg2465Trp LP/P rs760879110 Deafness, autosomal recessive, 12 (DFNB12) [MIM:601386]
CDH23 Q9H251 VAR_027340 p.Ser2517Gly LP/P rs759093040 Usher syndrome 1D (USH1D) [MIM:601067]
CDH23 Q9H251 VAR_027341 p.Arg2608His LP/P rs202052174 Deafness, autosomal recessive, 12 (DFNB12) [MIM:601386]
CDH23 Q9H251 VAR_027342 p.Gly2744Ser LP/P rs376189742 Usher syndrome 1D (USH1D) [MIM:601067]
CDH23 Q9H251 VAR_027343 p.Arg2833Gly LP/P rs760130862 Usher syndrome 1D (USH1D) [MIM:601067]
CDH23 Q9H251 VAR_027344 p.Val2933Glu LB/B - -
CDH23 Q9H251 VAR_027345 p.Asp2954Asn LB/B rs756793995 -
CDH23 Q9H251 VAR_027346 p.Asn2962Ser LB/B - -
CDH23 Q9H251 VAR_027347 p.Arg3175His LP/P rs140884994 Usher syndrome 1D (USH1D) [MIM:601067]
CDH23 Q9H251 VAR_046404 p.Pro240Leu LP/P rs121908354 Deafness, autosomal recessive, 12 (DFNB12) [MIM:601386]
CDH23 Q9H251 VAR_046405 p.Arg301Gln LP/P rs121908355 Deafness, autosomal recessive, 12 (DFNB12) [MIM:601386]
CDH23 Q9H251 VAR_046406 p.Val746Ile LB/B rs550384315 -
CDH23 Q9H251 VAR_046407 p.His755Tyr LP/P rs181255269 Usher syndrome 1D (USH1D) [MIM:601067]
CDH23 Q9H251 VAR_046408 p.Ser944Gly LB/B rs188098974 -
CDH23 Q9H251 VAR_046409 p.Glu960Lys LB/B rs111033458 -
CDH23 Q9H251 VAR_046410 p.Val1090Ile LP/P rs368487578 Usher syndrome 1D (USH1D) [MIM:601067]
CDH23 Q9H251 VAR_046411 p.Asn1098Ser LP/P rs41281310 Usher syndrome 1D (USH1D) [MIM:601067]
CDH23 Q9H251 VAR_046412 p.Arg1236Gln LB/B rs186990940 -
CDH23 Q9H251 VAR_046413 p.Asn1282Ser LB/B rs149073355 -
CDH23 Q9H251 VAR_046414 p.Arg1417Trp LP/P rs756231829 Deafness, autosomal recessive, 12 (DFNB12) [MIM:601386]
CDH23 Q9H251 VAR_046415 p.Ile1520Met LB/B - -
CDH23 Q9H251 VAR_046416 p.Met1574Thr LB/B rs1564769834 -
CDH23 Q9H251 VAR_046417 p.Val1711Ile LB/B rs181611778 -
CDH23 Q9H251 VAR_046418 p.Gln1716Pro LP/P rs758382198 Deafness, autosomal recessive, 12 (DFNB12) [MIM:601386]
CDH23 Q9H251 VAR_046419 p.Pro1788Leu LP/P rs564555435 Usher syndrome 1D (USH1D) [MIM:601067]
CDH23 Q9H251 VAR_046420 p.Val1807Met LB/B rs143993990 -
CDH23 Q9H251 VAR_046421 p.Ser1876Asn LB/B rs983665281 -
CDH23 Q9H251 VAR_046422 p.Val1908Ile LB/B rs368828743 -
CDH23 Q9H251 VAR_046423 p.Arg1912Trp LP/P rs397517344 Usher syndrome 1D (USH1D) [MIM:601067]
CDH23 Q9H251 VAR_046424 p.Asp1930Asn LP/P - Usher syndrome 1D (USH1D) [MIM:601067]
CDH23 Q9H251 VAR_046425 p.Arg2029Trp LP/P rs750880909 Deafness, autosomal recessive, 12 (DFNB12) [MIM:601386]
CDH23 Q9H251 VAR_046426 p.Ile2125Met LB/B rs16929354 -
CDH23 Q9H251 VAR_046427 p.Arg2171Cys LB/B rs781698111 -
CDH23 Q9H251 VAR_046428 p.Gln2227Pro LB/B rs778453484 -
CDH23 Q9H251 VAR_046429 p.Asp2376Val LP/P - Usher syndrome 1D (USH1D) [MIM:601067]
CDH23 Q9H251 VAR_046430 p.Leu2473Pro LB/B rs1589429497 -
CDH23 Q9H251 VAR_046431 p.Arg2489His LB/B rs141986620 -
CDH23 Q9H251 VAR_046432 p.Thr2530Ile LP/P rs781406146 Usher syndrome 1D (USH1D) [MIM:601067]
CDH23 Q9H251 VAR_046433 p.Ile2669Val LB/B - -
CDH23 Q9H251 VAR_046434 p.Gly2771Ser LP/P rs201076440 Usher syndrome 1D (USH1D) [MIM:601067]
CDH23 Q9H251 VAR_046435 p.Phe2801Val LB/B rs3802707 -

CDH23 Q9H251 VAR_046436 p.Ala2853Gln US - -
CDH23 Q9H251 VAR_046437 p.Val2968Ala LP/P rs765847991 Usher syndrome 1D (USH1D) [MIM:601067]
CDH23 Q9H251 VAR_046438 p.Arg3175Cys LB/B rs770796134 -
CDH23 Q9H251 VAR_071407 p.Asp160Asn LB/B rs1057519500 -
CDH23 Q9H251 VAR_071408 p.Asn342Ser LP/P rs1451062499 Deafness, autosomal recessive, 12 (DFNB12) [MIM:601386]
CDH23 Q9H251 VAR_071409 p.Ala361Ser LB/B rs774524441 -
CDH23 Q9H251 VAR_071410 p.Val424Met LB/B rs2305207 -
CDH23 Q9H251 VAR_071411 p.Asp428Asn LB/B rs188376296 -
CDH23 Q9H251 VAR_071412 p.Val803Ile LB/B - -
CDH23 Q9H251 VAR_071413 p.Glu956Lys LP/P rs756147087 Deafness, autosomal recessive, 12 (DFNB12) [MIM:601386]
CDH23 Q9H251 VAR_071414 p.Arg964Gln LB/B rs376560330 -
CDH23 Q9H251 VAR_071415 p.Arg1010His LB/B rs370107953 -
CDH23 Q9H251 VAR_071416 p.Gly1118Ser LB/B rs562052236 -
CDH23 Q9H251 VAR_071417 p.Val1335Ala LB/B rs1364542092 -
CDH23 Q9H251 VAR_071418 p.Thr1368Met LP/P rs762247872 Deafness, autosomal recessive, 12 (DFNB12) [MIM:601386]
CDH23 Q9H251 VAR_071419 p.Ser1415Ile LB/B - -
CDH23 Q9H251 VAR_071420 p.Ala1443Gly LB/B - -
CDH23 Q9H251 VAR_071421 p.Arg1588Trp LB/B rs137937502 -
CDH23 Q9H251 VAR_071422 p.Asp1626Ala LP/P rs1554871816 Deafness, autosomal recessive, 12 (DFNB12) [MIM:601386]
CDH23 Q9H251 VAR_071423 p.Asp1806Glu LB/B rs74145660 -
CDH23 Q9H251 VAR_071424 p.Ala2130Val LB/B - -
CDH23 Q9H251 VAR_071425 p.Asn2287Lys LP/P - Deafness, autosomal recessive, 12 (DFNB12) [MIM:601386]
CDH23 Q9H251 VAR_071426 p.Glu2438Lys LP/P rs1264310782 Deafness, autosomal recessive, 12 (DFNB12) [MIM:601386]
CDH23 Q9H251 VAR_071427 p.Met2531Val LB/B rs569138025 -
CDH23 Q9H251 VAR_071428 p.Gly2912Ser LB/B rs1381655860 -
CDH23 Q9H251 VAR_071429 p.Ala3080Thr LB/B rs369395479 -
CDH23 Q9H251 VAR_072661 p.Glu192Gln LB/B rs199514829 -
CDH23 Q9H251 VAR_072662 p.Met3062Thr LB/B rs770888523 -
CDH23 Q9H251 VAR_079747 p.Val187Ile LB/B rs369624952 -
CDH23 Q9H251 VAR_080381 p.Arg1379Leu LP/P rs767004225 Pituitary adenoma 5, multiple types (PITA5) [MIM:617540]
CDH23 Q9H251 VAR_080382 p.Arg2115His US rs1270566026 Pituitary adenoma 5, multiple types (PITA5) [MIM:617540]
CDH23 Q9H251 VAR_080383 p.Arg3138Trp US rs1052484950 Pituitary adenoma 5, multiple types (PITA5) [MIM:617540]
CDH23 Q9H251 VAR_080384 p.Asp3296Asn US rs372388344 Pituitary adenoma 5, multiple types (PITA5) [MIM:617540]
CDH26 Q8IXH8 VAR_055568 p.Ile144Thr LB/B rs6015609 -
CDH26 Q8IXH8 VAR_055569 p.Arg301Gly LB/B rs11086690 -
CDH26 Q8IXH8 VAR_055570 p.Val448Ala LB/B rs34866303 -
CDH26 Q8IXH8 VAR_055571 p.Pro479Leu LB/B rs6071067 -
CDH26 Q8IXH8 VAR_055572 p.Val615Leu LB/B rs194998 -

CDH3 P22223 VAR_015422 p.Arg503His LP/P rs121434542 Hypotrichosis congenital with juvenile macular dystrophy (HJMD) [MIM:601553]
CDH3 P22223 VAR_031929 p.Val237Met LB/B rs17854171 -
CDH3 P22223 VAR_031930 p.Arg477His LB/B rs34494880 -
CDH3 P22223 VAR_031931 p.Gln563His LB/B rs1126933 -
CDH3 P22223 VAR_033010 p.Asn322Ile LP/P rs121434543 Ectodermal dysplasia, ectrodactyly, and macular dystrophy syndrome (EEMS) [MIM:225280]
CDH4 P55283 VAR_033699 p.Lys625Arg LB/B rs6142884 -
CDH4 P55283 VAR_048504 p.Ala141Val LB/B rs34937312 -
CDH5 P33151 VAR_028003 p.Ile503Thr LB/B rs16956504 -
CDH5 P33151 VAR_028004 p.Ile517Thr LB/B rs1049970 -
CDH7 Q9ULB5 VAR_060247 p.Asn576Ser LB/B rs2291343 -
CDH7 Q9ULB5 VAR_061057 p.Asp370Glu LB/B rs2306675 -
CDH9 Q9ULB4 VAR_029799 p.Tyr6Cys LB/B rs2288467 -
CDH9 Q9ULB4 VAR_029800 p.Ala38Val LB/B rs2288466 -
CDHR1 Q96JP9 VAR_038744 p.His53Gln LB/B rs12781048 -
CDHR1 Q96JP9 VAR_038745 p.Ala212Thr LB/B rs200880106 -
CDHR1 Q96JP9 VAR_038746 p.Ala243Val LB/B rs7086200 -
CDHR1 Q96JP9 VAR_038747 p.Pro532Ala LB/B rs143662988 -
CDHR1 Q96JP9 VAR_075501 p.Pro574Ala LP/P - Cone-rod dystrophy 15 (CORD15) [MIM:613660]
CDHR1 Q96JP9 VAR_080429 p.Thr716Ser US - -
CDHR1 Q96JP9 VAR_083318 p.Thr171Ser LB/B rs759855253 -
CDHR2 Q9BYE9 VAR_021548 p.Leu1164Met LB/B rs17078347 -
CDHR2 Q9BYE9 VAR_046695 p.Gln107His LB/B rs6886860 -
CDHR2 Q9BYE9 VAR_046696 p.Ala415Gly LB/B rs3762960 -
CDHR2 Q9BYE9 VAR_046697 p.Val424Ala LB/B rs11134982 -
CDHR2 Q9BYE9 VAR_046698 p.Leu766Pro LB/B rs752138 -
CDHR2 Q9BYE9 VAR_046699 p.Thr901Met LB/B rs35018750 -
CDHR2 Q9BYE9 VAR_046700 p.Val948Met LB/B rs3749625 -
CDHR2 Q9BYE9 VAR_046701 p.Thr1128Met LB/B rs2291442 -
CDHR2 Q9BYE9 VAR_054148 p.Pro1004Leu LB/B rs371676123 -
CDHR3 Q6ZTQ4 VAR_035228 p.Val55Met LB/B rs35008315 -
CDHR3 Q6ZTQ4 VAR_035229 p.Gln61His LB/B rs34426483 -
CDHR3 Q6ZTQ4 VAR_035230 p.Cys529Tyr LB/B rs6967330 -
CDHR4 A6H8M9 VAR_039109 p.Arg5Lys LB/B rs13072748 -
CDHR5 Q9HBB8 VAR_017920 p.Arg357Ser LB/B rs2246614 -
CDHR5 Q9HBB8 VAR_017921 p.Asp389Asn LB/B rs2306066 -
CDHR5 Q9HBB8 VAR_059192 p.Cys702Ser LB/B rs2740379 -
CDHR5 Q9HBB8 VAR_060412 p.Gln165Pro LB/B rs2740374 -
CDHR5 Q9HBB8 VAR_060413 p.Pro521Ser LB/B rs2740375 -
CDIN1 Q9Y2V0 VAR_059622 p.Leu73Val LB/B rs3784678 -
CDIN1 Q9Y2V0 VAR_070876 p.Tyr94Cys LP/P rs587777101 Anemia, congenital dyserythropoietic, 1B (CDAN1B) [MIM:615631]
CDIN1 Q9Y2V0 VAR_070877 p.Leu178Gln LP/P rs587777100 Anemia, congenital dyserythropoietic, 1B (CDAN1B) [MIM:615631]
CDIN1 Q9Y2V0 VAR_082037 p.Pro20Arg US - Anemia, congenital dyserythropoietic, 1B (CDAN1B) [MIM:615631]
CDIN1 Q9Y2V0 VAR_082038 p.Tyr94Ser LP/P rs587777101 Anemia, congenital dyserythropoietic, 1B (CDAN1B) [MIM:615631]

CDIN1 Q9Y2V0 VAR_082039 p.His230Pro LP/P - Anemia, congenital dyserythropoietic, 1B (CDAN1B) [MIM:615631]
CDIN1 Q9Y2V0 VAR_082040 p.Tyr236Cys US rs768744226 Anemia, congenital dyserythropoietic, 1B (CDAN1B) [MIM:615631]
CDIPT O14735 VAR_048734 p.Arg199Cys LB/B rs1802002 -
CDK10 Q15131 VAR_041983 p.Pro96Leu LB/B rs55819627 -
CDK10 Q15131 VAR_041984 p.Asn168Ser LB/B rs56340740 -
CDK10 Q15131 VAR_041985 p.Arg342His LB/B rs55757604 -
CDK10 Q15131 VAR_041986 p.Cys358Tyr LB/B rs56242003 -
CDK11A Q9UQ88 VAR_031716 p.Arg93Trp LB/B rs1059831 -
CDK11A Q9UQ88 VAR_031717 p.Leu402Ser LB/B rs1059828 -
CDK11A Q9UQ88 VAR_060152 p.Cys57Arg LB/B rs1059832 -
CDK11A Q9UQ88 VAR_060153 p.Val658Ala LB/B rs866149312 -
CDK11A Q9UQ88 VAR_062200 p.Ser92Pro LB/B rs7531938 -
CDK11B P21127 VAR_041958 p.Arg57Cys LB/B rs752740049 -
CDK11B P21127 VAR_041959 p.Arg201Trp LB/B rs1557687207 -
CDK11B P21127 VAR_041960 p.Ser414Leu LB/B rs1241694892 -
CDK11B P21127 VAR_041961 p.Ile463Val LB/B - -
CDK11B P21127 VAR_041962 p.Leu601Gln LB/B rs200190129 -
CDK11B P21127 VAR_041963 p.Lys641Asn LB/B rs1059815 -
CDK11B P21127 VAR_041964 p.Ala670Val LB/B rs1059811 -
CDK11B P21127 VAR_045577 p.Val452Ala LB/B - -
CDK11B P21127 VAR_045578 p.Gly506Ser LB/B - -
CDK11B P21127 VAR_057775 p.Arg93Trp LB/B rs1059831 -
CDK11B P21127 VAR_062199 p.Arg109Cys LB/B rs1059830 -
CDK12 Q9NYV4 VAR_041968 p.Pro530Ala LB/B rs56121596 -
CDK12 Q9NYV4 VAR_041969 p.Arg912His US - A colorectal adenocarcinoma sample
CDK12 Q9NYV4 VAR_041970 p.Leu1189Gln LB/B rs56362165 -
CDK12 Q9NYV4 VAR_041971 p.Pro1275Leu LB/B rs34070318 -
CDK13 Q14004 VAR_022381 p.Pro356Ala LB/B rs17537669 -
CDK13 Q14004 VAR_022382 p.Leu403Phe LB/B rs3735137 -
CDK13 Q14004 VAR_022383 p.Arg410Gln LB/B rs17496261 -
CDK13 Q14004 VAR_022384 p.Thr500Ala LB/B rs3735135 -
CDK13 Q14004 VAR_022385 p.Ser624Gly LB/B rs17496275 -
CDK13 Q14004 VAR_022386 p.Arg700Leu LB/B rs1057000 -
CDK13 Q14004 VAR_022387 p.Val1062Met LB/B rs17496712 -
CDK13 Q14004 VAR_041965 p.Thr494Ala LB/B rs34624759 -
CDK13 Q14004 VAR_041966 p.Thr670Arg LB/B rs34775357 -
CDK13 Q14004 VAR_041967 p.Val1170Met LB/B rs3204309 -
CDK13 Q14004 VAR_053926 p.Ser340Phe LB/B rs13622 -
CDK13 Q14004 VAR_066526 p.Gln103Arg US - -
CDK13 Q14004 VAR_078598 p.Gly714Arg LP/P rs1057519633 Congenital heart defects, dysmorphic facial features, and intellectual developmental disorder (CHDFIDD) [MIM:617360]
CDK13 Q14004 VAR_078599 p.Gly717Arg LP/P rs1057519632 Congenital heart defects, dysmorphic facial features, and intellectual developmental disorder (CHDFIDD) [MIM:617360]
CDK13 Q14004 VAR_078600 p.Arg751Gln LP/P rs1057519634 Congenital heart defects, dysmorphic facial features, and intellectual developmental disorder (CHDFIDD) [MIM:617360]
CDK13 Q14004 VAR_078601 p.Asn842Ser LP/P rs878853160 Congenital heart defects, dysmorphic facial features, and intellectual developmental disorder (CHDFIDD) [MIM:617360]

CDK13 Q14004 VAR_079422 p.Lys734Glu LP/P rs1064795731 Congenital heart defects, dysmorphic facial features, and intellectual developmental disorder (CHDFIDD) [MIM:617360]
CDK13 Q14004 VAR_079423 p.Asn842Asp LP/P rs1554333853 Congenital heart defects, dysmorphic facial features, and intellectual developmental disorder (CHDFIDD) [MIM:617360]
CDK14 O94921 VAR_046765 p.Met432Ile US rs773301216 An ovarian mucinous carcinoma
CDK14 O94921 VAR_046766 p.Ser463Arg LB/B rs35643773 -
CDK15 Q96Q40 VAR_042016 p.Arg64Gly LB/B rs34776344 -
CDK15 Q96Q40 VAR_042017 p.Lys93Glu US - A renal clear cell carcinoma sample
CDK15 Q96Q40 VAR_042018 p.Gln127Arg LB/B rs56135556 -
CDK15 Q96Q40 VAR_042019 p.Thr255Ile LB/B rs34851370 -
CDK15 Q96Q40 VAR_042020 p.Glu276Asp US rs1490814436 A breast infiltrating ductal carcinoma sample
CDK17 Q00537 VAR_064743 p.Thr214Ile LB/B rs764448325 -
CDK18 Q07002 VAR_047802 p.Gly48Ser LB/B rs35134237 -
CDK18 Q07002 VAR_047803 p.Gly67Arg LB/B rs4623769 -
CDK18 Q07002 VAR_047804 p.Thr166Met LB/B rs17850752 -
CDK19 Q9BWU1 VAR_084392 p.Gly28Ala LP/P - Developmental and epileptic encephalopathy 87 (DEE87) [MIM:618916]
CDK19 Q9BWU1 VAR_084393 p.Gly28Arg LP/P - Developmental and epileptic encephalopathy 87 (DEE87) [MIM:618916]
CDK19 Q9BWU1 VAR_084394 p.Tyr32Cys LP/P - Developmental and epileptic encephalopathy 87 (DEE87) [MIM:618916]
CDK19 Q9BWU1 VAR_084395 p.Tyr32His LP/P - Developmental and epileptic encephalopathy 87 (DEE87) [MIM:618916]
CDK19 Q9BWU1 VAR_084396 p.Thr196Ala LP/P - Developmental and epileptic encephalopathy 87 (DEE87) [MIM:618916]
CDK19 Q9BWU1 VAR_084397 p.Phe197Leu LP/P - Developmental and epileptic encephalopathy 87 (DEE87) [MIM:618916]
CDK19 Q9BWU1 VAR_084398 p.Trp198Cys LP/P - Developmental and epileptic encephalopathy 87 (DEE87) [MIM:618916]
CDK19 Q9BWU1 VAR_084399 p.Arg200Trp LP/P - Developmental and epileptic encephalopathy 87 (DEE87) [MIM:618916]
CDK2 P24941 VAR_016157 p.Tyr15Ser LB/B rs3087335 -
CDK2 P24941 VAR_019988 p.Thr290Ser LB/B rs2069413 -
CDK2 P24941 VAR_041972 p.Pro45Leu US - A glioblastoma multiforme sample
CDK2 P24941 VAR_053927 p.Val18Leu LB/B rs11554376 -
CDK20 Q8IZL9 VAR_024762 p.Ser86Leu LB/B rs28364953 -
CDK20 Q8IZL9 VAR_024763 p.Ala137Thr LB/B rs28364955 -
CDK20 Q8IZL9 VAR_024764 p.Lys281Arg LB/B rs28364963 -
CDK20 Q8IZL9 VAR_041957 p.Ser106Asn LB/B rs41286029 -
CDK3 Q00526 VAR_021101 p.Thr226Ile LB/B rs2069532 -
CDK3 Q00526 VAR_021102 p.Met264Thr LB/B rs17884251 -
CDK3 Q00526 VAR_041973 p.Ser106Asn US - A glioblastoma multiforme sample
CDK3 Q00526 VAR_041974 p.Ile124Thr LB/B rs34918446 -
CDK3 Q00526 VAR_041975 p.Arg214His LB/B rs34670267 -
CDK4 P11802 VAR_006200 p.Arg24Cys LP/P rs11547328 Melanoma, cutaneous malignant 3 (CMM3) [MIM:609048]
CDK4 P11802 VAR_006201 p.Arg24His LP/P rs104894340 Melanoma, cutaneous malignant 3 (CMM3) [MIM:609048]

CDK4 P11802 VAR_021152 p.Asn41Ser LP/P rs144890720 Melanoma, cutaneous malignant 3 (CMM3) [MIM:609048]
CDK4 P11802 VAR_029153 p.Arg82Gln LB/B rs3211612 -
CDK4 P11802 VAR_041976 p.Arg122His LB/B rs34386532 -
CDK5 Q00535 VAR_041977 p.Glu225Asp LB/B rs35186917 -
CDK5RAP2 Q96SN8 VAR_017443 p.Glu289Gln LB/B rs4836822 -
CDK5RAP2 Q96SN8 VAR_017444 p.Val1540Leu LB/B rs4837768 -
CDK5RAP2 Q96SN8 VAR_032426 p.Arg1045Thr LB/B rs3780679 -
CDK5RAP2 Q96SN8 VAR_056831 p.Ala183Pro LB/B rs13287734 -
CDK5RAP2 Q96SN8 VAR_056832 p.Arg1607Ser LB/B rs16909747 -
CDK5RAP2 Q96SN8 VAR_059616 p.Asn1330Ile LB/B rs7875294 -
CDK5RAP3 Q96JB5 VAR_048688 p.Leu324Val LB/B rs35054799 -
CDK6 Q00534 VAR_041978 p.Asp110Asn LB/B rs35654944 -
CDK6 Q00534 VAR_041979 p.Pro199Leu US - A metastatic melanoma sample
CDK6 Q00534 VAR_072638 p.Ala197Thr LP/P rs606231255 Microcephaly 12, primary, autosomal recessive (MCPH12) [MIM:616080]
CDK7 P50613 VAR_023118 p.Gly163Ala LB/B - -
CDK7 P50613 VAR_023119 p.Thr285Met LB/B rs34584424 -
CDK8 P49336 VAR_041980 p.Asp189Asn US - A lung neuroendocrine carcinoma sample
CDK8 P49336 VAR_041981 p.Arg424Cys US - A colorectal adenocarcinoma sample
CDK8 P49336 VAR_083786 p.Val27Leu LP/P - Intellectual developmental disorder with hypotonia and behavioral abnormalities (IDDHBA) [MIM:618748]
CDK8 P49336 VAR_083787 p.Arg29Gly LP/P rs1593218885 Intellectual developmental disorder with hypotonia and behavioral abnormalities (IDDHBA) [MIM:618748]
CDK8 P49336 VAR_083788 p.Gly30Ser LP/P rs1593218890 Intellectual developmental disorder with hypotonia and behavioral abnormalities (IDDHBA) [MIM:618748]
CDK8 P49336 VAR_083789 p.Ser62Leu LP/P rs1565977796 Intellectual developmental disorder with hypotonia and behavioral abnormalities (IDDHBA) [MIM:618748]
CDK8 P49336 VAR_083790 p.Phe97Leu US - Intellectual developmental disorder with hypotonia and behavioral abnormalities (IDDHBA) [MIM:618748]
CDK8 P49336 VAR_083791 p.Arg178Gln LP/P rs1418353379 Intellectual developmental disorder with hypotonia and behavioral abnormalities (IDDHBA) [MIM:618748]
CDK8 P49336 VAR_083792 p.Val193Gly LP/P - Intellectual developmental disorder with hypotonia and behavioral abnormalities (IDDHBA) [MIM:618748]
CDK8 P49336 VAR_083793 p.Ile223Met LP/P rs1593310364 Intellectual developmental disorder with hypotonia and behavioral abnormalities (IDDHBA) [MIM:618748]
CDK9 P50750 VAR_013456 p.Gly231Ala LB/B - -
CDK9 P50750 VAR_041982 p.Phe59Leu LB/B rs55640715 -
CDK9 P50750 VAR_082140 p.Arg225Cys US rs767418586 -
CDKAL1 Q5VV42 VAR_052705 p.Lys484Arg LB/B rs9460608 -
CDKL1 Q00532 VAR_020576 p.Leu67Pro LB/B rs11570814 -
CDKL1 Q00532 VAR_020577 p.Gln275Glu LB/B rs7161563 -
CDKL1 Q00532 VAR_020578 p.Leu330Val LB/B rs9323183 -
CDKL1 Q00532 VAR_020579 p.Lys342Asn LB/B rs11570886 -
CDKL2 Q92772 VAR_041987 p.Leu98Ile US - An ovarian papillary serous adenocarcinoma sample
CDKL2 Q92772 VAR_041988 p.Arg149Gln US rs755711267 An ovarian mucinous carcinoma sample
CDKL2 Q92772 VAR_041989 p.Met197Thr LB/B rs56343717 -
CDKL2 Q92772 VAR_041990 p.Ala411Val LB/B rs56231363 -
CDKL2 Q92772 VAR_053928 p.Tyr77Ser LB/B rs35921414 -

CDKL2 Q92772 VAR_053929 p.Ile132Thr LB/B rs17000707 -
CDKL3 Q8IVW4 VAR_041991 p.Met394Thr LB/B rs35687772 -
CDKL4 Q5MAI5 VAR_041992 p.Ser38Pro LB/B rs35947084 -
CDKL4 Q5MAI5 VAR_041993 p.Arg53His LB/B rs35454041 -
CDKL4 Q5MAI5 VAR_041994 p.Phe228Cys LB/B rs56353587 -
CDKL4 Q5MAI5 VAR_041995 p.Ser288Tyr LB/B rs34819676 -
CDKL4 Q5MAI5 VAR_041996 p.Arg307Cys LB/B rs56330730 -
CDKL4 Q5MAI5 VAR_053930 p.His118Tyr LB/B rs6731369 -
CDKL5 O76039 VAR_023560 p.Cys152Phe LP/P rs122460157 Developmental and epileptic encephalopathy 2 (DEE2) [MIM:300672]
CDKL5 O76039 VAR_023561 p.Arg175Ser LP/P rs61749700 Developmental and epileptic encephalopathy 2 (DEE2) [MIM:300672]
CDKL5 O76039 VAR_023562 p.Gln791Pro LB/B rs35478150 -
CDKL5 O76039 VAR_036578 p.Asn368His US - A colorectal cancer sample
CDKL5 O76039 VAR_037635 p.Pro180Leu LP/P rs61749704 Developmental and epileptic encephalopathy 2 (DEE2) [MIM:300672]
CDKL5 O76039 VAR_037636 p.Val793Ala US rs62643617 Developmental and epileptic encephalopathy 2 (DEE2) [MIM:300672]
CDKL5 O76039 VAR_041997 p.Ala374Thr US - A metastatic melanoma sample
CDKL5 O76039 VAR_041998 p.Pro574Gln US rs199897804 An ovarian serous carcinoma sample
CDKL5 O76039 VAR_041999 p.Thr734Ala LB/B rs55803460 -
CDKL5 O76039 VAR_058022 p.Ala40Val LP/P rs122460159 Developmental and epileptic encephalopathy 2 (DEE2) [MIM:300672]
CDKL5 O76039 VAR_058023 p.Ile72Asn LP/P rs62641235 Developmental and epileptic encephalopathy 2 (DEE2) [MIM:300672]
CDKL5 O76039 VAR_058024 p.Ile72Thr LP/P rs62641235 Developmental and epileptic encephalopathy 2 (DEE2) [MIM:300672]
CDKL5 O76039 VAR_058025 p.His127Arg LP/P rs267608468 Developmental and epileptic encephalopathy 2 (DEE2) [MIM:300672]
CDKL5 O76039 VAR_058026 p.Arg178Pro LP/P rs267606715 Developmental and epileptic encephalopathy 2 (DEE2) [MIM:300672]
CDKL5 O76039 VAR_058027 p.Leu220Pro LP/P rs267608511 Developmental and epileptic encephalopathy 2 (DEE2) [MIM:300672]
CDKL5 O76039 VAR_058028 p.Thr288Ile LP/P rs267606713 Developmental and epileptic encephalopathy 2 (DEE2) [MIM:300672]
CDKL5 O76039 VAR_058029 p.Cys291Tyr LP/P rs267606714 Developmental and epileptic encephalopathy 2 (DEE2) [MIM:300672]
CDKL5 O76039 VAR_058030 p.Asn399Thr LP/P rs267608611 Developmental and epileptic encephalopathy 2 (DEE2) [MIM:300672]
CDKL5 O76039 VAR_058031 p.Arg444Cys LB/B rs61753977 -
CDKL5 O76039 VAR_058032 p.Val718Met LP/P rs267608653 Developmental and epileptic encephalopathy 2 (DEE2) [MIM:300672]
CDKL5 O76039 VAR_071103 p.Arg178Gln LP/P rs267606715 Developmental and epileptic encephalopathy 2 (DEE2) [MIM:300672]
CDKL5 O76039 VAR_078219 p.Ser196Leu LP/P rs267608501 Developmental and epileptic encephalopathy 2 (DEE2) [MIM:300672]
CDKL5 O76039 VAR_078625 p.His145Tyr US - Developmental and epileptic encephalopathy 2 (DEE2) [MIM:300672]

CDKL5 O76039 VAR_078626 p.Leu182Pro LP/P - Developmental and epileptic encephalopathy 2 (DEE2) [MIM:300672]
CDKL5 O76039 VAR_078627 p.Gly207Glu LP/P - Developmental and epileptic encephalopathy 2 (DEE2) [MIM:300672]
CDKL5 O76039 VAR_078628 p.His581Tyr US - Developmental and epileptic encephalopathy 2 (DEE2) [MIM:300672]
CDKL5 O76039 VAR_078630 p.Arg858Cys US rs773760466 Developmental and epileptic encephalopathy 2 (DEE2) [MIM:300672]
CDKL5 O76039 VAR_078712 p.Arg178Trp LP/P rs267608493 Developmental and epileptic encephalopathy 2 (DEE2) [MIM:300672]
CDKL5 O76039 VAR_078713 p.Pro647Leu LP/P - -
CDKN1A P38936 VAR_011870 p.Ser31Arg LB/B rs1801270 -
CDKN1A P38936 VAR_048686 p.Pro4Leu LB/B rs4986866 -
CDKN1A P38936 VAR_048687 p.Phe63Leu LB/B rs4986867 -
CDKN1B P46527 VAR_011871 p.Arg15Trp LB/B rs2066828 -
CDKN1B P46527 VAR_011872 p.Val109Gly LB/B rs2066827 -
CDKN1B P46527 VAR_064429 p.Pro69Leu LB/B rs777354267 -
CDKN1C P49918 VAR_068848 p.Asp274Asn LP/P rs387907225 Intrauterine growth retardation, metaphyseal dysplasia, adrenal hypoplasia congenita, and genital anomalies (IMAGE) [MIM:614732]
CDKN1C P49918 VAR_068849 p.Phe276Ser LP/P rs387907224 Intrauterine growth retardation, metaphyseal dysplasia, adrenal hypoplasia congenita, and genital anomalies (IMAGE) [MIM:614732]
CDKN1C P49918 VAR_068850 p.Phe276Val LP/P rs387907223 Intrauterine growth retardation, metaphyseal dysplasia, adrenal hypoplasia congenita, and genital anomalies (IMAGE) [MIM:614732]
CDKN1C P49918 VAR_068851 p.Lys278Glu LP/P rs387907226 Intrauterine growth retardation, metaphyseal dysplasia, adrenal hypoplasia congenita, and genital anomalies (IMAGE) [MIM:614732]
CDKN1C P49918 VAR_068852 p.Arg279Pro LP/P rs318240750 Intrauterine growth retardation, metaphyseal dysplasia, adrenal hypoplasia congenita, and genital anomalies (IMAGE) [MIM:614732]
CDKN1C P49918 VAR_075200 p.Met12Leu US rs483352966 Beckwith-Wiedemann syndrome (BWS) [MIM:130650]
CDKN1C P49918 VAR_075201 p.Leu53Pro LP/P rs483352968 Beckwith-Wiedemann syndrome (BWS) [MIM:130650]
CDKN1C P49918 VAR_075203 p.Pro70Leu LP/P rs483352970 Beckwith-Wiedemann syndrome (BWS) [MIM:130650]
CDKN1C P49918 VAR_075204 p.Pro158Ala US rs483352981 Beckwith-Wiedemann syndrome (BWS) [MIM:130650]
CDKN2A P42771 VAR_001408 p.Asp14Glu US - A biliary tract tumor
CDKN2A P42771 VAR_001409 p.Leu16Pro US rs864622263 A biliary tract tumor
CDKN2A P42771 VAR_001409 p.Leu16Pro US rs864622263 A familial melanoma
CDKN2A P42771 VAR_001410 p.Ala20Pro US rs760065045 A lung tumor
CDKN2A P42771 VAR_001410 p.Ala20Pro US rs760065045 Melanoma
CDKN2A P42771 VAR_001411 p.Ala20Ser US rs760065045 A biliary tract tumor
CDKN2A P42771 VAR_001412 p.Gly23Asp US rs1064794292 A melanoma
CDKN2A P42771 VAR_001412 p.Gly23Asp US rs1064794292 A pancreas tumor
CDKN2A P42771 VAR_001413 p.Arg24Cys LP/P - Melanoma, cutaneous malignant 2 (CMM2) [MIM:155601]
CDKN2A P42771 VAR_001414 p.Arg24Pro LP/P rs104894097 Melanoma, cutaneous malignant 2 (CMM2) [MIM:155601]
CDKN2A P42771 VAR_001415 p.Glu26Asp US - A biliary tract tumor

CDKN2A P42771 VAR_001416 p.Leu32Pro LP/P rs878853650 Melanoma, cutaneous malignant 2 (CMM2) [MIM:155601]
CDKN2A P42771 VAR_001417 p.Glu33Asp US - A biliary tract tumor
CDKN2A P42771 VAR_001418 p.Gly35Ala LP/P rs746834149 Melanoma, cutaneous malignant 2 (CMM2) [MIM:155601]
CDKN2A P42771 VAR_001419 p.Gly35Glu LP/P rs746834149 Melanoma, cutaneous malignant 2 (CMM2) [MIM:155601]
CDKN2A P42771 VAR_001420 p.Pro48Leu LP/P - Melanoma, cutaneous malignant 2 (CMM2) [MIM:155601]
CDKN2A P42771 VAR_001421 p.Ile49Ser US rs199907548 A biliary tract tumor
CDKN2A P42771 VAR_001422 p.Ile49Thr LB/B rs199907548 -
CDKN2A P42771 VAR_001423 p.Gln50Arg LP/P rs587778189 Melanoma, cutaneous malignant 2 (CMM2) [MIM:155601]
CDKN2A P42771 VAR_001424 p.Met53Ile LP/P rs104894095 Melanoma, cutaneous malignant 2 (CMM2) [MIM:155601]
CDKN2A P42771 VAR_001425 p.Ser56Ile LB/B rs104894109 -
CDKN2A P42771 VAR_001426 p.Ala57Val US rs372266620 Pancreas carcinoma
CDKN2A P42771 VAR_001427 p.Val59Gly LP/P rs104894099 Melanoma, cutaneous malignant 2 (CMM2) [MIM:155601]
CDKN2A P42771 VAR_001428 p.Ala60Thr LB/B rs769382085 -
CDKN2A P42771 VAR_001430 p.Leu62Pro LP/P - Melanoma, cutaneous malignant 2 (CMM2) [MIM:155601]
CDKN2A P42771 VAR_001431 p.His66Tyr US - Non-small cell lung carcinoma
CDKN2A P42771 VAR_001432 p.Ala68Leu LP/P rs876658534 Melanoma, cutaneous malignant 2 (CMM2) [MIM:155601]
CDKN2A P42771 VAR_001433 p.Ala68Thr US - An esophagus tumor
CDKN2A P42771 VAR_001434 p.Ala68Val LB/B rs1060501260 -
CDKN2A P42771 VAR_001435 p.Glu69Lys US - A bladder tumor
CDKN2A P42771 VAR_001436 p.Glu69Val US - A lung tumor
CDKN2A P42771 VAR_001437 p.Asn71Lys LP/P - Melanoma, cutaneous malignant 2 (CMM2) [MIM:155601]
CDKN2A P42771 VAR_001438 p.Asn71Ser LB/B rs559848002 -
CDKN2A P42771 VAR_001439 p.Cys72Gly US - An esophagus tumor
CDKN2A P42771 VAR_001440 p.Asp74Asn US rs760640852 A bladder tumor
CDKN2A P42771 VAR_001441 p.Asp74Val US - A biliary tract tumor
CDKN2A P42771 VAR_001442 p.Arg80Leu US - Neck tumor
CDKN2A P42771 VAR_001443 p.Pro81Leu US rs11552823 Some patients with melanoma
CDKN2A P42771 VAR_001444 p.His83Tyr US rs121913385 A pancreas tumor
CDKN2A P42771 VAR_001445 p.His83Asn US - A lung tumor
CDKN2A P42771 VAR_001446 p.Asp84Glu US - A bladder tumor
CDKN2A P42771 VAR_001447 p.Asp84His US - Non-small cell lung carcinoma
CDKN2A P42771 VAR_001448 p.Asp84Asn US rs11552822 An esophagus tumor
CDKN2A P42771 VAR_001449 p.Asp84Tyr LP/P rs11552822 Melanoma, cutaneous malignant 2 (CMM2) [MIM:155601]
CDKN2A P42771 VAR_001450 p.Ala85Thr LB/B rs878853646 -
CDKN2A P42771 VAR_001451 p.Arg87Pro LP/P rs878853647 Melanoma, cutaneous malignant 2 (CMM2) [MIM:155601]
CDKN2A P42771 VAR_001452 p.Glu88Asp US - A biliary tract tumor

CDKN2A P42771 VAR_001453 p.Gly89Asp LP/P rs137854599 Melanoma, cutaneous malignant 2 (CMM2) [MIM:155601]
CDKN2A P42771 VAR_001454 p.Gly89Ser LP/P rs137854597 Melanoma, cutaneous malignant 2 (CMM2) [MIM:155601]
CDKN2A P42771 VAR_001455 p.Thr93Ala US - Non-small cell lung carcinoma
CDKN2A P42771 VAR_001456 p.Val95Ala US - Non-small cell lung carcinoma
CDKN2A P42771 VAR_001457 p.Leu97Arg LP/P - Melanoma, cutaneous malignant 2 (CMM2) [MIM:155601]
CDKN2A P42771 VAR_001458 p.His98Pro LP/P - Melanoma, cutaneous malignant 2 (CMM2) [MIM:155601]
CDKN2A P42771 VAR_001459 p.His98Gln LP/P - Melanoma, cutaneous malignant 2 (CMM2) [MIM:155601]
CDKN2A P42771 VAR_001460 p.Arg99Pro LP/P rs754806883 Melanoma, cutaneous malignant 2 (CMM2) [MIM:155601]
CDKN2A P42771 VAR_001461 p.Arg99Gln US rs754806883 Non-small cell lung carcinoma
CDKN2A P42771 VAR_001462 p.Ala100Leu LP/P - Melanoma, cutaneous malignant 2 (CMM2) [MIM:155601]
CDKN2A P42771 VAR_001463 p.Ala100Pro LB/B - -
CDKN2A P42771 VAR_001464 p.Gly101Trp LP/P rs104894094 Familial atypical multiple mole melanoma-pancreatic carcinoma syndrome (FAMMMPC) [MIM:606719]
CDKN2A P42771 VAR_001464 p.Gly101Trp LP/P rs104894094 Melanoma, cutaneous malignant 2 (CMM2) [MIM:155601]
CDKN2A P42771 VAR_001466 p.Arg107Cys LP/P rs1554654024 Melanoma, cutaneous malignant 2 (CMM2) [MIM:155601]
CDKN2A P42771 VAR_001467 p.Arg107His LB/B rs370823171 -
CDKN2A P42771 VAR_001468 p.Asp108Tyr US rs121913381 Neck tumor
CDKN2A P42771 VAR_001469 p.Asp108His US - A bladder tumor
CDKN2A P42771 VAR_001470 p.Pro114Leu US rs121913386 Non-small cell lung carcinoma
CDKN2A P42771 VAR_001471 p.Leu117Met LP/P - Melanoma, cutaneous malignant 2 (CMM2) [MIM:155601]
CDKN2A P42771 VAR_001472 p.Ala118Thr LP/P rs1554653960 Melanoma, cutaneous malignant 2 (CMM2) [MIM:155601]
CDKN2A P42771 VAR_001473 p.Glu119Gln US - A biliary tract tumor
CDKN2A P42771 VAR_001474 p.Glu120Ala US - Non-small cell lung carcinoma
CDKN2A P42771 VAR_001475 p.Glu120Lys US - Non-small cell lung carcinoma
CDKN2A P42771 VAR_001476 p.Gly122Ser US rs113798404 A biliary tract tumor
CDKN2A P42771 VAR_001477 p.His123Gln US rs6413463 Leukemia
CDKN2A P42771 VAR_001478 p.Arg124His US rs747621669 An esophagus tumor
CDKN2A P42771 VAR_001479 p.Val126Asp LP/P rs104894098 Melanoma, cutaneous malignant 2 (CMM2) [MIM:155601]
CDKN2A P42771 VAR_001480 p.Ala127Ser US rs6413464 Squamous cell carcinoma
CDKN2A P42771 VAR_001481 p.Ala132Pro US - Non-small cell lung carcinoma
CDKN2A P42771 VAR_001482 p.Ala134Val US rs757497674 Non-small cell lung carcinoma
CDKN2A P42771 VAR_001483 p.His142Tyr US - Non-small cell lung carcinoma
CDKN2A P42771 VAR_001484 p.Arg144Cys US rs116150891 Squamous cell carcinoma
CDKN2A P42771 VAR_001486 p.Ala148Thr LB/B rs3731249 -
CDKN2A P42771 VAR_001487 p.Gly150Val US - Non-small cell lung carcinoma
CDKN2A P42771 VAR_012317 p.Arg87Trp LP/P rs749714198 Melanoma, cutaneous malignant 2 (CMM2) [MIM:155601]

CDKN2A P42771 VAR_015818 p.Ala102Glu LB/B rs137854598 -
CDKN2A P42771 VAR_023604 p.Leu94Gln LP/P - Melanoma, cutaneous malignant 2 (CMM2) [MIM:155601]
CDKN2A P42771 VAR_035069 p.Gly122Arg LP/P rs113798404 Melanoma, cutaneous malignant 2 (CMM2) [MIM:155601]
CDKN2A P42771 VAR_053028 p.Ala60Val US rs36204594 Melanoma
CDKN2A P42771 VAR_053029 p.His83Gln LB/B rs34968276 -
CDKN2A P42771 VAR_053030 p.Arg99Trp LB/B rs34886500 -
CDKN2A P42771 VAR_053031 p.Ala102Thr LB/B rs35741010 -
CDKN2A P42771 VAR_053032 p.Arg124Cys LB/B rs34170727 -
CDKN2A P42771 VAR_058550 p.Arg24Gln LB/B rs104894097 -
CDKN2A P42771 VAR_058551 p.Gly35Val LP/P rs746834149 Melanoma, cutaneous malignant 2 (CMM2) [MIM:155601]
CDKN2A P42771 VAR_058553 p.Gly67Arg LP/P rs758389471 Melanoma, cutaneous malignant 2 (CMM2) [MIM:155601]
CDKN2A P42771 VAR_058554 p.Glu69Gly LB/B rs372670098 -
CDKN2A P42771 VAR_058555 p.Asp74Tyr LP/P rs760640852 Melanoma, cutaneous malignant 2 (CMM2) [MIM:155601]
CDKN2A P42771 VAR_058556 p.Thr77Pro LP/P - Melanoma, cutaneous malignant 2 (CMM2) [MIM:155601]
CDKN2A P42771 VAR_058557 p.Arg80Pro LP/P rs1057519883 Melanoma, cutaneous malignant 2 (CMM2) [MIM:155601]
CDKN2A P42771 VAR_058558 p.Pro81Thr LP/P rs1334828764 Melanoma, cutaneous malignant 2 (CMM2) [MIM:155601]
CDKN2A P42771 VAR_058559 p.Pro114Ser LB/B rs104894104 -
CDKN2A Q8N726 VAR_029287 p.Pro17Ser LB/B rs3731190 -
CDKN2A Q8N726 VAR_053033 p.Gly106Arg LB/B rs4987127 -
CDKN2A Q8N726 VAR_053034 p.Pro113Leu LB/B rs34886500 -
CDKN2A Q8N726 VAR_053035 p.Gly116Asp LB/B rs35741010 -
CDKN2B P42772 VAR_001488 p.Gly47Glu US - Lung adenocarcinoma
CDKN2B P42772 VAR_001489 p.Ala50Val US rs1423790481 Lung adenocarcinoma
CDKN2C P42773 VAR_001490 p.Ala72Pro US rs771853517 Breast cancer
CDKN2C P42773 VAR_038604 p.Thr126Met LB/B rs17851380 -
CDKN3 Q16667 VAR_013842 p.Trp31Arg LP/P - Hepatocellular carcinoma (HCC) [MIM:114550]
CDKN3 Q16667 VAR_013843 p.Phe78Leu LP/P - Hepatocellular carcinoma (HCC) [MIM:114550]
CDKN3 Q16667 VAR_013844 p.Cys79Tyr LP/P - Hepatocellular carcinoma (HCC) [MIM:114550]
CDKN3 Q16667 VAR_013845 p.Asn91Lys LP/P rs760687800 Hepatocellular carcinoma (HCC) [MIM:114550]
CDKN3 Q16667 VAR_013846 p.Asp94Val LP/P - Hepatocellular carcinoma (HCC) [MIM:114550]
CDKN3 Q16667 VAR_013847 p.Leu95Phe LP/P - Hepatocellular carcinoma (HCC) [MIM:114550]
CDKN3 Q16667 VAR_013848 p.Ile108Val LP/P rs144479038 Hepatocellular carcinoma (HCC) [MIM:114550]
CDKN3 Q16667 VAR_013849 p.Asn187Ser LP/P - Hepatocellular carcinoma (HCC) [MIM:114550]
CDKN3 Q16667 VAR_013850 p.Lys195Ile LP/P - Hepatocellular carcinoma (HCC) [MIM:114550]
CDKN3 Q16667 VAR_051769 p.Ser159Phe LB/B rs1803843 -
CDO1 Q16878 VAR_023536 p.Thr45Ile LB/B rs1042867 -
CDO1 Q16878 VAR_036170 p.Glu143Gln US - A colorectal cancer sample
CDON Q4KMG0 VAR_056038 p.Lys66Arg LB/B rs7122277 -
CDON Q4KMG0 VAR_056039 p.Glu162Lys LB/B rs3740909 -
CDON Q4KMG0 VAR_056040 p.Pro351Ala LB/B rs35665264 -
CDON Q4KMG0 VAR_056041 p.Ala686Val LB/B rs12274923 -

CDON Q4KMG0 VAR_066497 p.Thr684Ser LB/B rs145983470 Holoprosencephaly 11 (HPE11) [MIM:614226]
CDON Q4KMG0 VAR_066498 p.Pro689Ala LP/P rs387906995 Holoprosencephaly 11 (HPE11) [MIM:614226]
CDON Q4KMG0 VAR_066499 p.Val691Met LP/P rs139323558 Holoprosencephaly 11 (HPE11) [MIM:614226]
CDON Q4KMG0 VAR_066500 p.Val780Glu LP/P rs387906996 Holoprosencephaly 11 (HPE11) [MIM:614226]
CDON Q4KMG0 VAR_066501 p.Thr790Ala LP/P rs387906997 Holoprosencephaly 11 (HPE11) [MIM:614226]
CDON Q4KMG0 VAR_066502 p.Ser940Arg LP/P rs369673018 Holoprosencephaly 11 (HPE11) [MIM:614226]
CDPF1 Q6NVV7 VAR_044058 p.Val5Ala LB/B rs9627281 -
CDPF1 Q6NVV7 VAR_062237 p.Leu70Arg LB/B rs55641018 -
CDR2L Q86X02 VAR_035384 p.Asp300Glu LB/B rs36057512 -
CDRT1 O95170 VAR_038340 p.Phe643Leu LB/B rs8078150 -
CDRT15 Q96T59 VAR_038121 p.Arg24Gln LB/B rs17679866 -
CDRT4 Q8N9R6 VAR_038119 p.Val73Ile LB/B rs3744332 -
CDRT4 Q8N9R6 VAR_038120 p.His122Gln LB/B rs2954759 -
CDS1 Q92903 VAR_036129 p.Lys204Thr US - A breast cancer sample
CDS1 Q92903 VAR_048736 p.Leu99Phe LB/B rs36068434 -
CDSN Q15517 VAR_022615 p.Met18Leu LB/B rs3095318 -
CDSN Q15517 VAR_022616 p.Asn143Ser LB/B rs3130984 -
CDSN Q15517 VAR_022618 p.Ser202Phe LB/B rs707913 -
CDSN Q15517 VAR_022619 p.Ser408Ala LB/B rs1042127 -
CDSN Q15517 VAR_022620 p.Ser410Leu LB/B rs3132554 -
CDSN Q15517 VAR_022621 p.Asp527Asn LB/B rs3130981 -
CDSN Q15517 VAR_022637 p.Leu56Phe LB/B rs7742033 -
CDSN Q15517 VAR_022639 p.Ser150Asn LB/B rs1184132826 -
CDSN Q15517 VAR_022641 p.Ser401Gly LB/B rs33941312 -
CDSN Q15517 VAR_046010 p.Gly145Ser LB/B rs6457328 -
CDT1 Q9H211 VAR_024408 p.Ala537Val LB/B rs3218721 -
CDT1 Q9H211 VAR_029163 p.Ala135Val LB/B rs3218725 -
CDT1 Q9H211 VAR_029164 p.Arg172Cys LB/B rs3218727 -
CDT1 Q9H211 VAR_029165 p.Glu456Ala LB/B rs3218729 -
CDT1 Q9H211 VAR_054504 p.Cys234Arg LB/B rs507329 -
CDT1 Q9H211 VAR_054505 p.Thr262Ala LB/B rs480727 -
CDT1 Q9H211 VAR_065488 p.Ala66Thr LP/P rs387906918 Meier-Gorlin syndrome 4 (MGORS4) [MIM:613804]
CDT1 Q9H211 VAR_065489 p.Gln117His LP/P rs779871947 Meier-Gorlin syndrome 4 (MGORS4) [MIM:613804]
CDT1 Q9H211 VAR_065490 p.Arg453Trp LP/P rs200672589 Meier-Gorlin syndrome 4 (MGORS4) [MIM:613804]
CDT1 Q9H211 VAR_065491 p.Arg462Gln LP/P rs387906917 Meier-Gorlin syndrome 4 (MGORS4) [MIM:613804]
CDT1 Q9H211 VAR_065492 p.Glu468Lys LP/P rs200652608 Meier-Gorlin syndrome 4 (MGORS4) [MIM:613804]
CDX1 P47902 VAR_020149 p.Pro130Arg LB/B rs2302275 -
CDX2 Q99626 VAR_014530 p.Pro293Ser LB/B rs1805107 -
CDYL Q9Y232 VAR_032936 p.Thr2Ala LB/B rs3812179 -
CDYL Q9Y232 VAR_032937 p.Ser9Pro LB/B rs3812178 -
CDYL Q9Y232 VAR_032938 p.Val48Ala LB/B rs13196069 -
CDYL Q9Y232 VAR_032939 p.Ala60Gly LB/B rs28360500 -
CEACAM1 P13688 VAR_049844 p.Gln35Lys LB/B rs8111171 -
CEACAM1 P13688 VAR_049845 p.Ala83Val LB/B rs8110904 -

CEACAM1 P13688 VAR_049846 p.Gln123His LB/B rs8111468 -
CEACAM1 P13688 VAR_049847 p.Gln376Arg LB/B rs41355544 -
CEACAM16 Q2WEN9 VAR_067769 p.Thr140Pro LP/P rs387907149 Deafness, autosomal dominant, 4B (DFNA4B) [MIM:614614]
CEACAM16 Q2WEN9 VAR_072720 p.Gly169Arg LP/P rs876661405 Deafness, autosomal dominant, 4B (DFNA4B) [MIM:614614]
CEACAM18 A8MTB9 VAR_043976 p.Leu160His LB/B rs8106673 -
CEACAM18 A8MTB9 VAR_043977 p.Thr161Ala LB/B rs12610545 -
CEACAM20 Q6UY09 VAR_056030 p.Ala41Val LB/B rs10408247 -
CEACAM20 Q6UY09 VAR_056031 p.Arg113His LB/B rs13345196 -
CEACAM20 Q6UY09 VAR_056032 p.Ile127Val LB/B rs35443082 -
CEACAM20 Q6UY09 VAR_056033 p.Ser355Leu LB/B rs16959164 -
CEACAM20 Q6UY09 VAR_056034 p.Ser369Phe LB/B rs10414398 -
CEACAM20 Q6UY09 VAR_059385 p.Arg512Cys LB/B rs8100718 -
CEACAM20 Q6UY09 VAR_061312 p.Thr87Ile LB/B rs36053277 -
CEACAM21 Q3KPI0 VAR_034651 p.Thr121Asn LB/B rs714106 -
CEACAM21 Q3KPI0 VAR_034652 p.Met198Val LB/B rs2302188 -
CEACAM3 P40198 VAR_003905 p.Ser7Pro LB/B rs1041999 -
CEACAM4 O75871 VAR_038404 p.His29Asp LB/B rs1126454 -
CEACAM4 O75871 VAR_038405 p.Lys69Arg LB/B rs3848568 -
CEACAM5 P06731 VAR_024493 p.Lys398Glu LB/B rs7249230 -
CEACAM5 P06731 VAR_031091 p.Ala340Asp LB/B rs10407503 -
CEACAM5 P06731 VAR_031092 p.Arg664Ser LB/B rs10423171 -
CEACAM5 P06731 VAR_056028 p.Gln137Pro LB/B rs3815780 -
CEACAM5 P06731 VAR_056029 p.Gly678Arg LB/B rs9621 -
CEACAM5 P06731 VAR_061310 p.Ile80Val LB/B rs12971352 -
CEACAM5 P06731 VAR_061311 p.Val83Ala LB/B rs28683503 -
CEACAM6 P40199 VAR_034680 p.Gly239Val LB/B rs11548735 -
CEACAM7 Q14002 VAR_024494 p.Phe120Ile LB/B rs8102488 -
CEACAM7 Q14002 VAR_049848 p.Ala263Val LB/B rs7259532 -
CEACAM7 Q14002 VAR_059384 p.Tyr236His LB/B rs16975478 -
CEACAM8 P31997 VAR_011721 p.Arg114Lys LB/B rs1041997 -
CEACAM8 P31997 VAR_049849 p.Gly17Val LB/B rs28367882 -
CEACAM8 P31997 VAR_049850 p.Ala45Val LB/B rs35221575 -
CEACAM8 P31997 VAR_049851 p.Leu322Val LB/B rs1126458 -
CEACAM8 P31997 VAR_049852 p.Ile340Met LB/B rs8103051 -
CEBPA P49715 VAR_072677 p.His84Leu LP/P rs28931590 Leukemia, acute myelogenous (AML) [MIM:601626]
CEBPB P17676 VAR_016300 p.Gly195Ser LB/B rs4253440 -
CEBPD P49716 VAR_037087 p.Arg248Trp LB/B rs34948549 -
CEBPZ Q03701 VAR_026043 p.Val102Ile LB/B rs2098386 -
CEBPZ Q03701 VAR_031399 p.Pro15Ser LB/B rs3213746 -
CEBPZ Q03701 VAR_031400 p.Lys303Arg LB/B rs17020328 -
CEBPZ Q03701 VAR_055622 p.Asn639Ser LB/B rs3180252 -
CECR2 Q9BXF3 VAR_027411 p.Arg293His LB/B rs5747211 -
CECR2 Q9BXF3 VAR_027412 p.Pro674Leu LB/B rs1296794 -
CEFIP Q711Q0 VAR_031441 p.Asn208Lys LB/B rs4838383 -
CEFIP Q711Q0 VAR_031442 p.His666Gln LB/B rs10857469 -
CEFIP Q711Q0 VAR_059608 p.Phe698Ser LB/B rs7921186 -

CEFIP	Q711Q0	VAR_059609	p.Asp741Gly	LB/B	rs11101093	-
CEFIP	Q711Q0	VAR_059610	p.Asp883Asn	LB/B	rs12217617	-
CEFIP	Q711Q0	VAR_059611	p.Phe958Tyr	LB/B	rs10857470	-
CEFIP	Q711Q0	VAR_059612	p.Gly1084Ala	LB/B	rs11101094	-
CEFIP	Q711Q0	VAR_059613	p.Val1254Ile	LB/B	rs11101095	-
CEFIP	Q711Q0	VAR_059614	p.Gly1337Ser	LB/B	rs10857472	-
CEFIP	Q711Q0	VAR_061604	p.Arg320Leu	LB/B	rs56206226	-
CEFIP	Q711Q0	VAR_061605	p.Asp461Ala	LB/B	rs45554335	-
CEFIP	Q711Q0	VAR_061606	p.Val522Gly	LB/B	rs61453891	-
CELA1	Q9UNI1	VAR_033645	p.Gln10His	LB/B	rs17860287	-
CELA1	Q9UNI1	VAR_033646	p.Arg44Trp	LB/B	rs17860299	-
CELA1	Q9UNI1	VAR_033647	p.Met59Val	LB/B	rs17860300	-
CELA1	Q9UNI1	VAR_033648	p.Gln243Arg	LB/B	rs17860364	-
CELA1	Q9UNI1	VAR_036295	p.Gly76Ala	US	-	A breast cancer sample
CELA2A	P08217	VAR_051837	p.Asn257Ser	LB/B	rs2303193	-
CELA2A	P08217	VAR_083326	p.Thr70Met	LP/P	rs372947070	Abdominal obesity-metabolic syndrome 4 (AOMS4) [MIM:618620]
CELA2A	P08217	VAR_083327	p.Leu85Met	US	rs558493952	Abdominal obesity-metabolic syndrome 4 (AOMS4) [MIM:618620]
CELA2A	P08217	VAR_083328	p.Asp121Asn	LP/P	rs1352544800	Abdominal obesity-metabolic syndrome 4 (AOMS4) [MIM:618620]
CELA2B	P08218	VAR_044534	p.Gly79Arg	LB/B	rs3820071	-
CELA2B	P08218	VAR_044535	p.Asp114Asn	LB/B	rs3766160	-
CELA2B	P08218	VAR_044536	p.Gln177Arg	LB/B	rs6429745	-
CELA2B	P08218	VAR_044537	p.Gly235Ser	LB/B	rs3737703	-
CELA3A	P09093	VAR_051838	p.Ala241Gly	LB/B	rs3820285	-
CELA3A	P09093	VAR_059783	p.His24Arg	LB/B	rs7531336	-
CELA3A	P09093	VAR_059784	p.Ser25Pro	LB/B	rs7533776	-
CELA3A	P09093	VAR_059785	p.His31Asn	LB/B	rs7519660	-
CELA3B	P08861	VAR_025446	p.Arg79Trp	LB/B	rs7528405	-
CELF2	O95319	VAR_052202	p.Asp438His	LB/B	rs1050942	-
CELF4	Q9BZC1	VAR_052203	p.Gly388Ser	LB/B	rs12458669	-
CELF5	Q8N6W0	VAR_033264	p.Phe65Leu	LB/B	rs17854481	-
CELF6	Q96J87	VAR_033265	p.Arg152Pro	LB/B	rs34566074	-
CELSR1	Q9NYQ6	VAR_016094	p.Ser664Trp	LB/B	rs4823850	-
CELSR1	Q9NYQ6	VAR_016095	p.Cys1126Arg	LB/B	rs4823561	-
CELSR1	Q9NYQ6	VAR_024479	p.Ile2107Val	LB/B	rs4044210	-
CELSR1	Q9NYQ6	VAR_024480	p.Thr2268Ala	LB/B	rs6007897	-
CELSR1	Q9NYQ6	VAR_049464	p.Ile587Val	LB/B	rs34141466	-
CELSR1	Q9NYQ6	VAR_049465	p.Val1242Ile	LB/B	rs6008842	-
CELSR1	Q9NYQ6	VAR_049466	p.Tyr1894His	LB/B	rs34467708	-
CELSR1	Q9NYQ6	VAR_049467	p.Leu1994Pro	LB/B	rs6008795	-
CELSR1	Q9NYQ6	VAR_049468	p.Leu1995Pro	LB/B	rs6008794	-
CELSR1	Q9NYQ6	VAR_049469	p.Thr2045Met	LB/B	rs12169391	-
CELSR1	Q9NYQ6	VAR_049470	p.Arg2219His	LB/B	rs34267201	-
CELSR1	Q9NYQ6	VAR_049471	p.Cys2797Ser	LB/B	rs12165943	-
CELSR1	Q9NYQ6	VAR_049472	p.Glu2903Gln	LB/B	rs9615351	-
CELSR1	Q9NYQ6	VAR_049473	p.Gly2948Ser	LB/B	rs35364389	-
CELSR1	Q9NYQ6	VAR_067213	p.Ala773Val	LP/P	rs12170597	Neural tube defects (NTD) [MIM:182940]

CELSR1	Q9NYQ6	VAR_067214	p.Arg2312Pro	LB/B	rs7287089	-
CELSR1	Q9NYQ6	VAR_067215	p.Arg2438Gln	LP/P	rs199688538	Neural tube defects (NTD) [MIM:182940]
CELSR1	Q9NYQ6	VAR_067216	p.Asn2739Thr	LB/B	rs148905592	-
CELSR1	Q9NYQ6	VAR_067217	p.Ser2964Leu	LP/P	rs6008777	Neural tube defects (NTD) [MIM:182940]
CELSR1	Q9NYQ6	VAR_067218	p.Pro2983Ala	LP/P	rs61741871	Neural tube defects (NTD) [MIM:182940]
CELSR2	Q9HCU4	VAR_024481	p.Tyr1639His	LB/B	rs653635	-
CELSR2	Q9HCU4	VAR_049474	p.Arg1066Gln	LB/B	rs12083590	-
CELSR2	Q9HCU4	VAR_049475	p.Gly1992Arg	LB/B	rs12567377	-
CELSR2	Q9HCU4	VAR_049476	p.Thr2387Ala	LB/B	rs17035649	-
CELSR2	Q9HCU4	VAR_083430	p.Arg2812Trp	US	rs149683589	-
CELSR3	Q9NYQ7	VAR_020022	p.Ala157Pro	LB/B	rs3733085	-
CELSR3	Q9NYQ7	VAR_020023	p.Ser805Thr	LB/B	rs3821875	-
CELSR3	Q9NYQ7	VAR_055101	p.Gln1758Arg	LB/B	rs12107252	-
CELSR3	Q9NYQ7	VAR_083108	p.Gly2136Asp	US	rs587777163	-
CELSR3	Q9NYQ7	VAR_083109	p.Met2630Ile	LB/B	rs149614835	-
CEMIP	Q8WUJ3	VAR_018165	p.Arg187Cys	LB/B	rs368854657	-
CEMIP	Q8WUJ3	VAR_018166	p.Arg187His	LB/B	rs144446375	-
CEMIP	Q8WUJ3	VAR_018167	p.His783Arg	LB/B	rs12441101	-
CEMIP	Q8WUJ3	VAR_018168	p.His783Tyr	LB/B	rs996035812	-
CEMIP	Q8WUJ3	VAR_018169	p.Val1109Ile	LB/B	rs751807711	-
CEMIP	Q8WUJ3	VAR_018170	p.Pro1169Ala	LB/B	rs16972583	-
CEMIP2	Q9UHN6	VAR_032669	p.Arg245Lys	LB/B	rs25688	-
CEMIP2	Q9UHN6	VAR_032670	p.Arg291His	LB/B	rs25689	-
CEMIP2	Q9UHN6	VAR_032671	p.Asp423Glu	LB/B	rs25695	-
CEMIP2	Q9UHN6	VAR_032672	p.Pro765Ser	LB/B	rs25692	-
CEMIP2	Q9UHN6	VAR_032673	p.Ile1010Val	LB/B	rs17057133	-
CEMIP2	Q9UHN6	VAR_032674	p.Ser1254Asn	LB/B	rs2297089	-
CEMIP2	Q9UHN6	VAR_032675	p.Gly1280Asp	LB/B	rs17475375	-
CEMIP2	Q9UHN6	VAR_062197	p.Arg291Leu	LB/B	rs25689	-
CEMIP2	Q9UHN6	VAR_062198	p.Arg291Pro	LB/B	rs25689	-
CEMP1	Q6PRD7	VAR_050792	p.Lys55Glu	LB/B	rs13331643	-
CENATAC	Q86UT8	VAR_029848	p.Trp244Gly	LB/B	rs600648	-
CENPC	Q03188	VAR_069295	p.Leu341Phe	LB/B	rs11250	-
CENPC	Q03188	VAR_069296	p.Gly389Asp	LB/B	rs1056787	-
CENPE	Q02224	VAR_049689	p.Phe1535Leu	LB/B	rs2615542	-
CENPE	Q02224	VAR_049690	p.Ser1581Arg	LB/B	rs35100664	-
CENPE	Q02224	VAR_049691	p.Glu1925Asp	LB/B	rs2306106	-
CENPE	Q02224	VAR_049692	p.Thr2090Met	LB/B	rs2243682	-
CENPE	Q02224	VAR_059370	p.Ser1911Thr	LB/B	rs1381657	-
CENPE	Q02224	VAR_072429	p.Asp933Asn	LP/P	rs144716013	Microcephaly 13, primary, autosomal recessive (MCPH13) [MIM:616051]
CENPE	Q02224	VAR_072430	p.Lys1355Glu	LP/P	rs141488085	Microcephaly 13, primary, autosomal recessive (MCPH13) [MIM:616051]
CENPF	P49454	VAR_014839	p.Asn3106Lys	LB/B	rs7289	-
CENPF	P49454	VAR_034712	p.Arg300Cys	LB/B	rs17023281	-
CENPF	P49454	VAR_034713	p.His494Gln	LB/B	rs2070065	-
CENPF	P49454	VAR_034714	p.Met701Val	LB/B	rs3795524	-
CENPF	P49454	VAR_034715	p.Gln754Glu	LB/B	rs3795523	-
CENPF	P49454	VAR_034716	p.Arg815His	LB/B	rs3795522	-

CENPF	P49454	VAR_034717	p.Tyr1018Asp	LB/B	rs3795519	-
CENPF	P49454	VAR_034718	p.Gly1033Arg	LB/B	rs3795518	-
CENPF	P49454	VAR_034719	p.Thr1105Ile	LB/B	rs12067133	-
CENPF	P49454	VAR_034720	p.Leu1412Ser	LB/B	rs3795517	-
CENPF	P49454	VAR_034723	p.Glu1915Ala	LB/B	rs3790647	-
CENPF	P49454	VAR_055049	p.Gln250Leu	LB/B	rs1050065	-
CENPF	P49454	VAR_055050	p.Asp272Gly	LB/B	rs1050066	-
CENPF	P49454	VAR_055638	p.Asp1768Asn	LB/B	rs3748692	-
CENPH	Q9H3R5	VAR_036167	p.Glu2Lys	US	rs777698677	A colorectal cancer sample
CENPJ	Q9HC77	VAR_032427	p.Met21Val	LB/B	rs35498994	-
CENPJ	Q9HC77	VAR_032428	p.Pro55Ala	LB/B	rs17081389	-
CENPJ	Q9HC77	VAR_032429	p.Asp63His	LB/B	rs7336216	-
CENPJ	Q9HC77	VAR_032430	p.Pro85Thr	LB/B	rs9511510	-
CENPJ	Q9HC77	VAR_032431	p.Glu151Gly	LB/B	rs34177811	-
CENPJ	Q9HC77	VAR_032432	p.Ser879Ala	LB/B	rs17402892	-
CENPJ	Q9HC77	VAR_032433	p.Glu1235Val	LP/P	rs121434311	Microcephaly 6, primary, autosomal recessive (MCPH6) [MIM:608393]
CENPL	Q8N0S6	VAR_027081	p.Ile117Phe	LB/B	rs12086855	-
CENPN	Q96H22	VAR_027419	p.Glu84Asp	LB/B	rs935939	-
CENPN	Q96H22	VAR_048689	p.Gln223Arg	LB/B	rs11641523	-
CENPN	Q96H22	VAR_048690	p.Glu288Lys	LB/B	rs2549887	-
CENPO	Q9BU64	VAR_027420	p.Gln34Arg	LB/B	rs1550116	-
CENPQ	Q7L2Z9	VAR_022857	p.Gly63Arg	LB/B	rs4267943	-
CENPQ	Q7L2Z9	VAR_024303	p.Asp266Gly	LB/B	rs2501968	-
CENPT	Q96BT3	VAR_027421	p.Pro115Leu	LB/B	rs12102580	-
CENPU	Q71F23	VAR_027144	p.Gly16Ser	LB/B	rs902174	-
CENPU	Q71F23	VAR_027145	p.Ile157Thr	LB/B	rs6552804	-
CENPU	Q71F23	VAR_027146	p.Ile214Met	LB/B	rs4616798	-
CENPU	Q71F23	VAR_048692	p.Gly16Arg	LB/B	rs902174	-
CENPU	Q71F23	VAR_048693	p.Ala279Thr	LB/B	rs34007339	-
CEP104	O60308	VAR_020042	p.Ala686Val	LB/B	rs2275831	-
CEP104	O60308	VAR_034036	p.Leu414Ile	LB/B	rs2275824	-
CEP112	Q8N8E3	VAR_050752	p.Lys551Glu	LB/B	rs17704679	-
CEP120	Q8N960	VAR_046126	p.Leu602Val	LB/B	rs6595440	-
CEP120	Q8N960	VAR_046127	p.Gln879His	LB/B	rs1047437	-
CEP120	Q8N960	VAR_046128	p.Val936Ile	LB/B	rs2303721	-
CEP120	Q8N960	VAR_046129	p.Arg947His	LB/B	rs2303720	-
CEP120	Q8N960	VAR_073672	p.Ala199Pro	LP/P	rs367600930	Short-rib thoracic dysplasia 13 with or without polydactyly (SRTD13) [MIM:616300]
CEP120	Q8N960	VAR_077553	p.Val194Ala	US	rs1554104276	Joubert syndrome 31 (JBTS31) [MIM:617761]
CEP120	Q8N960	VAR_077554	p.Ala549Val	US	rs775080726	Joubert syndrome 31 (JBTS31) [MIM:617761]
CEP120	Q8N960	VAR_077555	p.Leu712Phe	US	rs114280473	Joubert syndrome 31 (JBTS31) [MIM:617761]
CEP120	Q8N960	VAR_077556	p.Leu726Pro	US	rs1554102026	Joubert syndrome 31 (JBTS31) [MIM:617761]
CEP120	Q8N960	VAR_077557	p.Ile975Ser	US	rs1554098663	-
CEP126	Q9P2H0	VAR_027363	p.Ala178Thr	LB/B	rs11225086	-
CEP126	Q9P2H0	VAR_027364	p.Gly238Cys	LB/B	rs7926728	-
CEP126	Q9P2H0	VAR_027365	p.Ser275Tyr	LB/B	rs11225089	-
CEP126	Q9P2H0	VAR_027366	p.Ile281Thr	LB/B	rs11225090	-
CEP126	Q9P2H0	VAR_027367	p.Ala302Thr	LB/B	rs11225091	-

CEP126	Q9P2H0	VAR_027368	p.Pro710Ser	LB/B	rs7109614	-
CEP126	Q9P2H0	VAR_027369	p.Met1021Thr	LB/B	rs7111429	-
CEP126	Q9P2H0	VAR_027370	p.Ser1077Asn	LB/B	rs6590942	-
CEP126	Q9P2H0	VAR_068173	p.Gly668Ser	LB/B	rs76022391	-
CEP128	Q6ZU80	VAR_037835	p.Arg16Leu	LB/B	rs7160694	-
CEP128	Q6ZU80	VAR_037836	p.His732Arg	LB/B	rs327463	-
CEP131	Q9UPN4	VAR_056740	p.Val43Ile	LB/B	rs8067409	-
CEP131	Q9UPN4	VAR_060226	p.Ile70Val	LB/B	rs752612451	-
CEP131	Q9UPN4	VAR_060227	p.Thr272Ala	LB/B	rs2466773	-
CEP131	Q9UPN4	VAR_060228	p.Thr397Ala	LB/B	rs2659015	-
CEP131	Q9UPN4	VAR_060229	p.Val473Ala	LB/B	rs2659016	-
CEP135	Q66GS9	VAR_057785	p.Ile769Leu	LB/B	rs3214045	-
CEP152	O94986	VAR_047932	p.Ser54Leu	LB/B	rs2289181	-
CEP152	O94986	VAR_050779	p.Ser793Ile	LB/B	rs2289178	-
CEP152	O94986	VAR_050780	p.Leu914Val	LB/B	rs16961560	-
CEP152	O94986	VAR_050781	p.Val1106Ala	LB/B	rs16961557	-
CEP152	O94986	VAR_063813	p.Gln265Pro	LP/P	rs267606717	Microcephaly 9, primary, autosomal recessive (MCPH9) [MIM:614852]
CEP152	O94986	VAR_065258	p.Lys667Arg	LP/P	rs200879436	Seckel syndrome 5 (SCKL5) [MIM:613823]
CEP162	Q5TB80	VAR_033301	p.Cys266Ser	LB/B	rs17790493	-
CEP162	Q5TB80	VAR_033302	p.Glu272Gln	LB/B	rs16874323	-
CEP162	Q5TB80	VAR_051293	p.Ser342Cys	LB/B	rs17790493	-
CEP162	Q5TB80	VAR_051294	p.Glu348Gln	LB/B	rs16874323	-
CEP164	Q9UPV0	VAR_037511	p.Ser94Asn	LB/B	rs490262	-
CEP164	Q9UPV0	VAR_037512	p.Thr988Ser	LB/B	rs2305830	-
CEP164	Q9UPV0	VAR_037513	p.Gln1119Arg	LB/B	rs573455	-
CEP164	Q9UPV0	VAR_068503	p.Gln11Pro	LP/P	rs387907309	Nephronophthisis 15 (NPHP15) [MIM:614845]
CEP164	Q9UPV0	VAR_068504	p.Arg93Trp	LP/P	rs387907310	Nephronophthisis 15 (NPHP15) [MIM:614845]
CEP170	Q5SW79	VAR_031437	p.Gly213Ser	LB/B	rs2631092	-
CEP192	Q8TEP8	VAR_037514	p.Thr1053Ala	LB/B	rs10048340	-
CEP192	Q8TEP8	VAR_037515	p.Val1365Met	LB/B	rs2282542	-
CEP192	Q8TEP8	VAR_050782	p.Gln1109Pro	LB/B	rs11080623	-
CEP192	Q8TEP8	VAR_050783	p.Arg1544His	LB/B	rs7228940	-
CEP192	Q8TEP8	VAR_050784	p.Ser1552Pro	LB/B	rs578208	-
CEP192	Q8TEP8	VAR_050785	p.Leu1701Phe	LB/B	rs6505780	-
CEP192	Q8TEP8	VAR_050786	p.Ser2051Asn	LB/B	rs2027698	-
CEP192	Q8TEP8	VAR_050787	p.Leu2121Pro	LB/B	rs474337	-
CEP192	Q8TEP8	VAR_050788	p.Lys2271Glu	LB/B	rs3737379	-
CEP192	Q8TEP8	VAR_050789	p.Arg2449Leu	LB/B	rs1786263	-
CEP250	Q9BV73	VAR_015649	p.Gln995His	LB/B	rs2296403	-
CEP250	Q9BV73	VAR_021858	p.Arg1441Gln	LB/B	rs3748433	-
CEP250	Q9BV73	VAR_050898	p.Gln1072Glu	LB/B	rs17092706	-
CEP250	Q9BV73	VAR_081747	p.Ala609Val	US	rs145878385	-
CEP290	O15078	VAR_028356	p.Trp7Cys	LP/P	rs62635288	Joubert syndrome 5 (JBTS5) [MIM:610188]
CEP290	O15078	VAR_028356	p.Trp7Cys	LP/P	rs62635288	Senior-Loken syndrome 6 (SLSN6) [MIM:610189]
CEP290	O15078	VAR_031058	p.Lys838Glu	LB/B	rs11104738	-
CEP290	O15078	VAR_031059	p.Leu906Trp	LB/B	rs7970228	-
CEP290	O15078	VAR_031060	p.Arg1237His	LB/B	rs7307793	-
CEP290	O15078	VAR_031061	p.Ile1836Val	LB/B	rs11104729	-

CEP290	O15078	VAR_064397	p.Glu277Gln	LB/B	rs45502896	-
CEP290	O15078	VAR_064398	p.Asp664Gly	LB/B	rs79705698	-
CEP290	O15078	VAR_064399	p.Ala1566Pro	LB/B	-	-
CEP290	O15078	VAR_064400	p.Leu1694Pro	LB/B	-	-
CEP290	O15078	VAR_064401	p.Asn2228Lys	LB/B	rs373711746	-
CEP290	O15078	VAR_066997	p.Arg2210Cys	LB/B	rs374852145	-
CEP290	O15078	VAR_067192	p.Ser2263Gly	US	rs77778467	-
CEP290	O15078	VAR_068168	p.Glu534Lys	LP/P	rs895126773	Joubert syndrome 5 (JBTS5) [MIM:610188]
CEP290	O15078	VAR_075696	p.Ile2134Thr	LB/B	rs117852025	Joubert syndrome 5 (JBTS5) [MIM:610188]
CEP295	Q9C0D2	VAR_059337	p.Gln80Lys	LB/B	rs7128850	-
CEP295	Q9C0D2	VAR_059338	p.Arg208Gln	LB/B	rs10831088	-
CEP295	Q9C0D2	VAR_059339	p.Ala499Glu	LB/B	rs4753495	-
CEP295	Q9C0D2	VAR_059340	p.Glu1026Lys	LB/B	rs3802771	-
CEP295	Q9C0D2	VAR_059341	p.Ala1270Gly	LB/B	rs2298707	-
CEP295	Q9C0D2	VAR_059342	p.Gly1441Glu	LB/B	rs3802773	-
CEP295	Q9C0D2	VAR_059343	p.Leu1459Arg	LB/B	rs3802774	-
CEP350	Q5VT06	VAR_026126	p.Glu945Gln	LB/B	rs2477120	-
CEP350	Q5VT06	VAR_048671	p.Thr1445Ala	LB/B	rs16855164	-
CEP350	Q5VT06	VAR_059202	p.Arg892Thr	LB/B	rs6692219	-
CEP350	Q5VT06	VAR_059203	p.Gly1213Val	LB/B	rs12125245	-
CEP350	Q5VT06	VAR_059204	p.Thr1446Ala	LB/B	rs16855164	-
CEP350	Q5VT06	VAR_059205	p.Ser1517Ala	LB/B	rs12124336	-
CEP350	Q5VT06	VAR_061092	p.Thr2044Pro	LB/B	rs56173179	-
CEP41	Q9BYV8	VAR_067053	p.Met36Thr	LP/P	rs368178632	-
CEP41	Q9BYV8	VAR_067054	p.Gln89Glu	US	-	-
CEP41	Q9BYV8	VAR_067055	p.Arg179His	LP/P	rs140259402	-
CEP41	Q9BYV8	VAR_067056	p.Pro206Ala	US	rs143303575	-
CEP41	Q9BYV8	VAR_067057	p.Cys240Gly	US	rs113941736	-
CEP41	Q9BYV8	VAR_067058	p.Arg360Cys	LP/P	rs371812716	-
CEP43	O95684	VAR_051000	p.Lys271Asn	LB/B	rs17856382	-
CEP43	O95684	VAR_061651	p.Ala190Gly	LB/B	rs34617108	-
CEP44	Q9C0F1	VAR_033116	p.Gly147Ser	LB/B	rs4695918	-
CEP55	Q53EZ4	VAR_022996	p.Thr99Ala	LB/B	rs7080916	-
CEP55	Q53EZ4	VAR_022997	p.His378Leu	LB/B	rs2293277	-
CEP55	Q53EZ4	VAR_026559	p.His57Gln	LB/B	rs3740370	-
CEP55	Q53EZ4	VAR_056791	p.Cys236Arg	LB/B	rs7072484	-
CEP57	Q86XR8	VAR_059839	p.Arg448Gly	LB/B	rs644799	-
CEP57L1	Q8IYX8	VAR_052396	p.Asp194Glu	LB/B	rs351733	-
CEP63	Q96MT8	VAR_020604	p.Ser651Leu	LB/B	rs1127826	-
CEP68	Q76N32	VAR_022363	p.Gly74Ser	LB/B	rs7572857	-
CEP68	Q76N32	VAR_050794	p.Arg27Gly	LB/B	rs12611491	-
CEP68	Q76N32	VAR_050795	p.Leu397Pro	LB/B	rs35501092	-
CEP68	Q76N32	VAR_050796	p.Arg462Cys	LB/B	rs35694840	-
CEP68	Q76N32	VAR_050797	p.Glu473Gln	LB/B	rs35089924	-
CEP70	Q8NHQ1	VAR_021177	p.Ser135Asn	LB/B	rs1673607	-
CEP70	Q8NHQ1	VAR_061590	p.Asn537Ser	LB/B	rs35340237	-
CEP72	Q9P209	VAR_050798	p.Pro238Leu	LB/B	rs869955	-
CEP72	Q9P209	VAR_050799	p.Pro412Thr	LB/B	rs12522955	-
CEP72	Q9P209	VAR_050800	p.Thr509Ala	LB/B	rs868649	-

CEP83 Q9Y592 VAR_058397 p.Gln78Arg LB/B rs2271979 -
CEP83 Q9Y592 VAR_071266 p.Leu87Pro LP/P - Nephronophthisis 18 (NPHP18) [MIM:615862]
CEP83 Q9Y592 VAR_071268 p.Arg511Pro LP/P rs587777487 Nephronophthisis 18 (NPHP18) [MIM:615862]
CEP85 Q6P2H3 VAR_033665 p.Ala542Thr LB/B rs7550997 -
CEP85 Q6P2H3 VAR_053938 p.Arg48His LB/B rs35831900 -
CEP85 Q6P2H3 VAR_053939 p.Ser213Asn LB/B rs3795686 -
CEP85 Q6P2H3 VAR_053940 p.Gln668His LB/B rs36013141 -
CEP85L Q5SZL2 VAR_034670 p.Ser137Gly LB/B rs3734381 -
CEP85L Q5SZL2 VAR_034671 p.Asp166Val LB/B rs9489444 -
CEP85L Q5SZL2 VAR_034672 p.Pro251Thr LB/B rs3734382 -
CEP85L Q5SZL2 VAR_036247 p.Ser345Phe US - A breast cancer sample
CEP85L Q5SZL2 VAR_053941 p.Gln532His LB/B rs9489410 -
CEP85L Q5SZL2 VAR_053942 p.Met640Val LB/B rs7743702 -
CEP85L Q5SZL2 VAR_084269 p.Ser58Cys LP/P - Lissencephaly 10 (LIS10) [MIM:618873]
CEP85L Q5SZL2 VAR_084270 p.Asp65Asn LP/P - Lissencephaly 10 (LIS10) [MIM:618873]
CEP85L Q5SZL2 VAR_084271 p.Ile68Thr US - Lissencephaly 10 (LIS10) [MIM:618873]
CEP85L Q5SZL2 VAR_084272 p.Gly69Arg US - Lissencephaly 10 (LIS10) [MIM:618873]
CEP89 Q96ST8 VAR_063122 p.Arg194Trp LB/B rs3764633 -
CEP89 Q96ST8 VAR_063123 p.Val398Ala LB/B rs4805825 -
CEP95 Q96GE4 VAR_033666 p.Met165Ile LB/B rs9910506 -
CEP95 Q96GE4 VAR_050751 p.Ala811Val LB/B rs11550922 -
CER1 O95813 VAR_021591 p.Arg19Trp LB/B rs10115703 -
CER1 O95813 VAR_021592 p.Ala65Gly LB/B rs3747532 -
CER1 O95813 VAR_021593 p.Val179Ile LB/B rs7036635 -
CERK Q8TCT0 VAR_053685 p.Ile191Val LB/B rs16995615 -
CERK Q8TCT0 VAR_053686 p.Thr211Met LB/B rs9306515 -
CERK Q8TCT0 VAR_053687 p.Leu306Phe LB/B rs13057352 -
CERKL Q49MI3 VAR_053688 p.Leu232Phe LB/B rs10185262 -
CERKL Q49MI3 VAR_053689 p.Glu514Gly LB/B rs35955809 -
CERKL Q49MI3 VAR_065182 p.Arg106Ser LP/P rs569826109 Retinitis pigmentosa 26 (RP26) [MIM:608380]
CERS1 P27544 VAR_073336 p.His183Gln LP/P rs200024180 Epilepsy, progressive myoclonic 8 (EPM8) [MIM:616230]
CERS2 Q96G23 VAR_052325 p.Glu115Ala LB/B rs267738 -
CERS3 Q8IU89 VAR_019328 p.Arg370Gly LB/B rs2439928 -
CERS3 Q8IU89 VAR_057276 p.Asp342Gly LB/B rs1023783 -
CERS3 Q8IU89 VAR_061847 p.Tyr45Cys LB/B rs60405735 -
CERS4 Q9HA82 VAR_019556 p.Gly301Ser LB/B rs2288413 -
CERS4 Q9HA82 VAR_019557 p.Ala366Thr LB/B rs36259 -
CERS4 Q9HA82 VAR_034065 p.Arg119Gln LB/B rs17159388 -
CERS4 Q9HA82 VAR_060263 p.Ala353Val LB/B rs17160348 -
CERS4 Q9HA82 VAR_060264 p.Arg379Gln LB/B rs17160349 -
CERS5 Q8N5B7 VAR_019558 p.Cys75Arg LB/B rs7302981 -
CERT1 Q9Y5P4 VAR_061815 p.Lys599Arg LB/B rs55882089 -
CERT1 Q9Y5P4 VAR_069403 p.Ser138Cys US - -
CERT1 Q9Y5P4 VAR_073721 p.Ser132Leu LP/P rs1064794019 Mental retardation, autosomal dominant 34 (MRD34) [MIM:616351]
CERT1 Q9Y5P4 VAR_078652 p.Gly243Arg LP/P - Mental retardation, autosomal dominant 34 (MRD34) [MIM:616351]
CES1 P23141 VAR_014314 p.Ser75Asn LB/B rs2307240 -

CES1 P23141 VAR_014594 p.Arg199His LB/B rs2307243 -
CES1 P23141 VAR_014595 p.Asp203Glu LB/B rs2307227 -
CES1 P23141 VAR_046954 p.Gly143Glu LB/B rs71647871 -
CES2 O00748 VAR_018396 p.Arg34Trp LB/B rs72547531 -
CES2 O00748 VAR_018397 p.Arg206His LB/B rs8192924 -
CES3 Q6UWW8 VAR_060699 p.Val129Ile LB/B rs61745806 -
CES3 Q6UWW8 VAR_060700 p.Ala151Thr LB/B rs71647891 -
CES3 Q6UWW8 VAR_060701 p.Tyr160His LB/B rs71647892 -
CES3 Q6UWW8 VAR_060702 p.Glu191Lys LB/B rs61742964 -
CES3 Q6UWW8 VAR_060703 p.Ile213Asn LB/B rs71647894 -
CES3 Q6UWW8 VAR_060704 p.Arg367Trp LB/B rs61743167 -
CES3 Q6UWW8 VAR_060705 p.Ala523Val LB/B rs71649615 -
CES3 Q6UWW8 VAR_060706 p.Ile555Val LB/B rs8059252 -
CES5A Q6NT32 VAR_036836 p.Arg71Gln LB/B rs2397965 -
CES5A Q6NT32 VAR_036837 p.Glu261Lys LB/B rs11076126 -
CES5A Q6NT32 VAR_036838 p.His344Gln LB/B rs11860946 -
CES5A Q6NT32 VAR_036839 p.Gly499Arg LB/B rs16955812 -
CES5A Q6NT32 VAR_036840 p.Asp537Glu LB/B rs11860456 -
CETN3 O15182 VAR_030846 p.Val10Leu LB/B rs4873 -
CETP P11597 VAR_004172 p.Asp459Gly LP/P rs2303790 Hyperalphalipoproteinemia 1 (HALP1)
[MIM:143470]
CETP P11597 VAR_013919 p.Gly331Ser LB/B rs5881 -
CETP P11597 VAR_013920 p.Ala390Pro LB/B rs5880 -
CETP P11597 VAR_013921 p.Val422Ile LB/B rs5882 -
CETP P11597 VAR_013922 p.Arg468Gln LB/B rs1800777 -
CETP P11597 VAR_013923 p.Val486Met LB/B rs5887 -
CETP P11597 VAR_017018 p.Ala15Gly LB/B rs34065661 -
CETP P11597 VAR_017019 p.Val385Met LB/B rs34855278 -
CETP P11597 VAR_031127 p.Val455Met LB/B rs2228667 -
CETP P11597 VAR_033098 p.Arg154Trp LB/B rs34716057 -
CETP P11597 VAR_033099 p.Leu168Pro LP/P - Hyperalphalipoproteinemia 1 (HALP1) [MIM:143470]
CETP P11597 VAR_033100 p.Arg299Cys LP/P rs142459781 Hyperalphalipoproteinemia 1 (HALP1)
[MIM:143470]
CFAP157 Q5JU67 VAR_050845 p.Tyr483Ser LB/B rs497632 -
CFAP161 Q6P656 VAR_050883 p.Pro284Ser LB/B rs2279997 -
CFAP206 Q8IYR0 VAR_033679 p.Arg154Lys LB/B rs13219364 -
CFAP206 Q8IYR0 VAR_033680 p.Gly170Ala LB/B rs16879281 -
CFAP20DC Q6ZVT6 VAR_056772 p.Ser158Arg LB/B rs13324082 -
CFAP20DC Q6ZVT6 VAR_056773 p.Asp304Glu LB/B rs35778488 -
CFAP20DC Q6ZVT6 VAR_061575 p.Val387Met LB/B rs34631714 -
CFAP20DC Q6ZVT6 VAR_061576 p.Ser404Asn LB/B rs34322986 -
CFAP221 Q4G0U5 VAR_039232 p.Val637Ile LB/B rs2272058 -
CFAP221 Q4G0U5 VAR_039233 p.Met795Ile LB/B rs11686014 -
CFAP251 Q8TBY9 VAR_034623 p.Glu61Gly LB/B rs12824001 -
CFAP251 Q8TBY9 VAR_034624 p.Thr383Met LB/B rs34768683 -
CFAP251 Q8TBY9 VAR_034625 p.Leu445Phe LB/B rs11043265 -
CFAP251 Q8TBY9 VAR_060044 p.Gly66Glu LB/B rs12824001 -
CFAP298 P57076 VAR_070200 p.Arg33Trp LB/B rs753786167 -

CFAP298 P57076 VAR_070201 p.Asp141Gly US rs140727644 Ciliary dyskinesia, primary, 26 (CILD26) [MIM:615500]
CFAP298 P57076 VAR_070202 p.Asp173Tyr LB/B rs540473945 -
CFAP299 Q6V702 VAR_034913 p.Thr138Met LB/B rs11947742 -
CFAP299 Q6V702 VAR_034914 p.Val220Ile LB/B rs1052325 -
CFAP300 Q9BRQ4 VAR_080475 p.His259Arg LP/P rs1555071691 Ciliary dyskinesia, primary, 38 (CILD38) [MIM:618063]
CFAP36 Q96G28 VAR_030795 p.Asp243Gly LB/B rs1045910 -
CFAP36 Q96G28 VAR_030796 p.Ile246Phe LB/B rs1045920 -
CFAP36 Q96G28 VAR_050725 p.Glu6Asp LB/B rs34891804 -
CFAP410 O43822 VAR_016155 p.Thr150Ile LB/B rs2277809 -
CFAP410 O43822 VAR_050927 p.Gly153Ser LB/B rs9306099 -
CFAP410 O43822 VAR_075924 p.Arg73Pro LP/P rs140451304 Spondylometaphyseal dysplasia, axial (SMDAX) [MIM:602271]
CFAP410 O43822 VAR_075925 p.Tyr107His LP/P rs763623409 Spondylometaphyseal dysplasia, axial (SMDAX) [MIM:602271]
CFAP410 O43822 VAR_075926 p.Pro116Leu LP/P rs922930539 Spondylometaphyseal dysplasia, axial (SMDAX) [MIM:602271]
CFAP410 O43822 VAR_075927 p.Leu224Pro LP/P rs1114167892 Spondylometaphyseal dysplasia, axial (SMDAX) [MIM:602271]
CFAP410 O43822 VAR_079180 p.Cys61Tyr LP/P rs1057518441 Retinal dystrophy with or without macular staphyloma (RDMS) [MIM:617547]
CFAP410 O43822 VAR_079181 p.Tyr107Cys LP/P rs1131690801 Retinal dystrophy with or without macular staphyloma (RDMS) [MIM:617547]
CFAP410 O43822 VAR_079182 p.Val111Met LP/P rs555164150 Spondylometaphyseal dysplasia, axial (SMDAX) [MIM:602271]
CFAP43 Q8NDM7 VAR_037130 p.Ile393Thr LB/B rs10883979 -
CFAP43 Q8NDM7 VAR_037131 p.Leu616Phe LB/B rs35901897 -
CFAP43 Q8NDM7 VAR_037132 p.Val634Ile LB/B rs17116635 -
CFAP43 Q8NDM7 VAR_037133 p.Ala835Ser LB/B rs12262825 -
CFAP43 Q8NDM7 VAR_080878 p.Arg85Trp US rs376788209 Spermatogenic failure 19 (SPGF19) [MIM:617592]
CFAP43 Q8NDM7 VAR_080879 p.Ser129Tyr US rs1131692266 Spermatogenic failure 19 (SPGF19) [MIM:617592]
CFAP43 Q8NDM7 VAR_080880 p.Val347Ala US rs147356105 Spermatogenic failure 19 (SPGF19) [MIM:617592]
CFAP44 Q96MT7 VAR_053435 p.Lys284Asn LB/B rs16845107 -
CFAP44 Q96MT7 VAR_062105 p.Lys53Glu LB/B rs59722850 -
CFAP45 Q9UL16 VAR_059600 p.Glu291Gly LB/B rs16842789 -
CFAP46 Q8IYW2 VAR_023208 p.Ala2331Thr LB/B rs4880433 -
CFAP46 Q8IYW2 VAR_023209 p.Ser2540Gly LB/B rs2254419 -
CFAP46 Q8IYW2 VAR_046201 p.Ser264Asn LB/B rs12781609 -
CFAP46 Q8IYW2 VAR_056823 p.Cys2125Arg LB/B rs10870341 -
CFAP46 Q8IYW2 VAR_056824 p.Glu2249Lys LB/B rs12356978 -
CFAP46 Q8IYW2 VAR_056825 p.Leu2499Phe LB/B rs3750587 -
CFAP46 Q8IYW2 VAR_056826 p.Lys2658Gln LB/B rs35981039 -
CFAP47 Q6ZTR5 VAR_056856 p.Val236Met LB/B rs2336029 -
CFAP47 Q6ZTR5 VAR_060280 p.Cys345Arg LB/B rs6632427 -
CFAP47 Q6ZTR5 VAR_060281 p.Ala561Thr LB/B rs11795910 -

CFAP47	Q6ZTR5	VAR_060282	p.His634Tyr	LB/B	rs17852470	-
CFAP52	Q8N1V2	VAR_026057	p.Glu336Lys	LB/B	rs6503235	-
CFAP53	Q96M91	VAR_050746	p.Arg231Cys	LB/B	rs12607385	-
CFAP53	Q96M91	VAR_050747	p.Glu294Lys	LB/B	rs35193847	-
CFAP53	Q96M91	VAR_077590	p.Arg158Gly	US	rs886037751	Heterotaxy, visceral, 6, autosomal (HTX6) [MIM:614779]
CFAP54	Q96N23	VAR_056834	p.Pro11Ser	LB/B	rs3809197	-
CFAP54	Q96N23	VAR_056835	p.Thr558Ala	LB/B	rs2160501	-
CFAP54	Q96N23	VAR_056836	p.Leu562Phe	LB/B	rs2160502	-
CFAP57	Q96MR6	VAR_026850	p.Ala183Ser	LB/B	rs6663799	-
CFAP57	Q96MR6	VAR_026851	p.Asn241Asp	LB/B	rs663824	-
CFAP57	Q96MR6	VAR_026852	p.Cys345Ser	LB/B	rs11210805	-
CFAP57	Q96MR6	VAR_066494	p.Asp523Tyr	US	rs387907122	-
CFAP58	Q5T655	VAR_030255	p.Ser496Thr	LB/B	rs11192036	-
CFAP58	Q5T655	VAR_030256	p.Gln804His	LB/B	rs7087328	-
CFAP61	Q8NHU2	VAR_027981	p.Pro74Arg	LB/B	rs17852602	-
CFAP61	Q8NHU2	VAR_027982	p.His254Arg	LB/B	rs6075614	-
CFAP61	Q8NHU2	VAR_027983	p.Val369Ile	LB/B	rs6081901	-
CFAP61	Q8NHU2	VAR_027984	p.Pro371Leu	LB/B	rs6075628	-
CFAP61	Q8NHU2	VAR_027985	p.Asp505Glu	LB/B	rs7344530	-
CFAP61	Q8NHU2	VAR_027986	p.Pro660Leu	LB/B	rs6081930	-
CFAP61	Q8NHU2	VAR_027987	p.Val671Ile	LB/B	rs6046740	-
CFAP61	Q8NHU2	VAR_069396	p.Thr790Pro	LB/B	-	-
CFAP65	Q6ZU64	VAR_032500	p.Met66Leu	LB/B	rs6736922	-
CFAP65	Q6ZU64	VAR_050726	p.Val672Ile	LB/B	rs13403802	-
CFAP65	Q6ZU64	VAR_050727	p.Lys806Met	LB/B	rs9653262	-
CFAP65	Q6ZU64	VAR_050728	p.Asn1858Ser	LB/B	rs11893183	-
CFAP65	Q6ZU64	VAR_083171	p.Asn1007Lys	US	-	Spermatogenic failure 40 (SPGF40) [MIM:618664]
CFAP65	Q6ZU64	VAR_083172	p.Leu1016Arg	US	-	Spermatogenic failure 40 (SPGF40) [MIM:618664]
CFAP65	Q6ZU64	VAR_083656	p.Asp639Glu	LB/B	-	-
CFAP65	Q6ZU64	VAR_083657	p.Ile1240Phe	LB/B	-	-
CFAP69	A5D8W1	VAR_039219	p.Ser22Arg	LB/B	rs17862129	-
CFAP69	A5D8W1	VAR_039220	p.Arg306Cys	LB/B	rs17866223	-
CFAP69	A5D8W1	VAR_039221	p.Pro459Leu	LB/B	rs17865959	-
CFAP69	A5D8W1	VAR_039222	p.Val490Met	LB/B	rs1029365	-
CFAP69	A5D8W1	VAR_039223	p.Thr885Met	LB/B	rs1134956	-
CFAP70	Q5T0N1	VAR_032877	p.Trp849Leu	LB/B	rs12256262	-
CFAP70	Q5T0N1	VAR_032878	p.Asn944Asp	LB/B	rs4294502	-
CFAP70	Q5T0N1	VAR_083155	p.Phe60Ile	US	-	Spermatogenic failure 41 (SPGF41) [MIM:618670]
CFAP74	Q9C0B2	VAR_039873	p.Ser50Ile	LB/B	rs13303083	-
CFAP74	Q9C0B2	VAR_039874	p.Ile363Val	LB/B	rs16824588	-
CFAP74	Q9C0B2	VAR_039875	p.Gly628Cys	LB/B	rs3820011	-
CFAP77	Q6ZQR2	VAR_039959	p.Gly137Arg	LB/B	rs7047726	-
CFAP77	Q6ZQR2	VAR_039960	p.Arg172Trp	US	rs149346027	A colorectal cancer sample
CFAP77	Q6ZQR2	VAR_039961	p.Arg184His	LB/B	rs11243798	-
CFAP77	Q6ZQR2	VAR_062164	p.Lys244Gln	LB/B	rs34650498	-
CFAP91	Q7Z4T9	VAR_030243	p.Ala207Pro	LB/B	rs6438544	-
CFAP91	Q7Z4T9	VAR_030244	p.Ser253Thr	LB/B	rs9817771	-
CFAP91	Q7Z4T9	VAR_030245	p.Ser320Cys	LB/B	rs9819218	-

CFAP97 Q9P2B7 VAR_036915 p.Thr238Ala LB/B rs1133657 -
CFAP97 Q9P2B7 VAR_036916 p.Leu443Ser LB/B rs6820332 -
CFB P00751 VAR_006492 p.Trp28Arg LB/B - -
CFB P00751 VAR_006493 p.Trp28Gln LB/B - -
CFB P00751 VAR_006494 p.Arg32Gln LB/B rs641153 -
CFB P00751 VAR_006495 p.Ala736Ser LB/B - -
CFB P00751 VAR_016274 p.Leu9His LB/B rs4151667 -
CFB P00751 VAR_016275 p.Arg32Trp LB/B rs12614 -
CFB P00751 VAR_016276 p.Gly252Ser LB/B rs4151651 -
CFB P00751 VAR_016277 p.Lys565Glu LB/B rs4151659 -
CFB P00751 VAR_016278 p.Asp651Glu LB/B rs4151660 -
CFB P00751 VAR_063221 p.Phe286Leu LP/P rs117905900 Hemolytic uremic syndrome atypical 4 (AHUS4) [MIM:612924]
CFB P00751 VAR_063222 p.Lys323Glu LP/P rs121909748 Hemolytic uremic syndrome atypical 4 (AHUS4) [MIM:612924]
CFB P00751 VAR_063659 p.Ser166Pro LP/P - Hemolytic uremic syndrome atypical 4 (AHUS4) [MIM:612924]
CFB P00751 VAR_063660 p.Arg203Gln LP/P rs745794224 Hemolytic uremic syndrome atypical 4 (AHUS4) [MIM:612924]
CFB P00751 VAR_063661 p.Ile242Leu LP/P rs144812066 Hemolytic uremic syndrome atypical 4 (AHUS4) [MIM:612924]
CFB P00751 VAR_063662 p.Lys323Gln LP/P - Hemolytic uremic syndrome atypical 4 (AHUS4) [MIM:612924]
CFB P00751 VAR_063663 p.Met458Ile LP/P rs200837114 Hemolytic uremic syndrome atypical 4 (AHUS4) [MIM:612924]
CFB P00751 VAR_063664 p.Lys533Arg LB/B rs149101394 Hemolytic uremic syndrome atypical 4 (AHUS4) [MIM:612924]
CFC1 P0CG37 VAR_024322 p.Arg78Trp LB/B rs2579433 -
CFC1 P0CG37 VAR_024323 p.Arg112Cys LP/P rs104893611 Heterotaxy, visceral, 2, autosomal (HTX2) [MIM:605376]
CFC1 P0CG37 VAR_024324 p.Arg189Cys LB/B rs1350439781 -
CFD P00746 VAR_034866 p.Val213Gly LP/P rs267606720 Complement factor D deficiency (CFDD) [MIM:613912]
CFD P00746 VAR_034867 p.Cys214Arg LP/P rs267606721 Complement factor D deficiency (CFDD) [MIM:613912]
CFD P00746 VAR_034868 p.Ile248Met LB/B rs2230216 -
CFDP1 Q9UEE9 VAR_048408 p.Ala60Thr LB/B rs16963331 -
CFH P08603 VAR_001979 p.Tyr402His LB/B rs1061170 -
CFH P08603 VAR_019405 p.Cys536Arg LP/P rs121913052 Complement factor H deficiency (CFHD) [MIM:609814]
CFH P08603 VAR_019406 p.Cys959Tyr LP/P rs121913053 Complement factor H deficiency (CFHD) [MIM:609814]
CFH P08603 VAR_019407 p.Leu1189Arg LP/P rs121913055 Hemolytic uremic syndrome atypical 1 (AHUS1) [MIM:235400]
CFH P08603 VAR_019408 p.Ser1191Leu LP/P rs460897 Hemolytic uremic syndrome atypical 1 (AHUS1) [MIM:235400]
CFH P08603 VAR_020261 p.Glu936Asp LB/B rs1065489 -
CFH P08603 VAR_023836 p.Val62Ile LB/B rs800292 -
CFH P08603 VAR_025092 p.Ile551Thr LB/B rs35453854 -

CFH	P08603	VAR_025093	p.Ser890Ile	LB/B	rs515299	-
CFH	P08603	VAR_025094	p.Val1007Ile	LB/B	-	-
CFH	P08603	VAR_025095	p.Thr1017Ile	LB/B	rs34362004	-
CFH	P08603	VAR_025096	p.Asn1050Tyr	LB/B	rs35274867	-
CFH	P08603	VAR_025097	p.Ile1059Thr	LB/B	rs35343172	-
CFH	P08603	VAR_025864	p.Arg78Gly	LP/P	-	Hemolytic uremic syndrome atypical 1 (AHUS1) [MIM:235400]
CFH	P08603	VAR_025865	p.Cys630Trp	LP/P	-	Hemolytic uremic syndrome atypical 1 (AHUS1) [MIM:235400]
CFH	P08603	VAR_025866	p.Glu850Lys	LP/P	rs762443267	Hemolytic uremic syndrome atypical 1 (AHUS1) [MIM:235400]
CFH	P08603	VAR_025867	p.Gln950His	LP/P	rs149474608	Hemolytic uremic syndrome atypical 1 (AHUS1) [MIM:235400]
CFH	P08603	VAR_025868	p.Tyr951His	LP/P	rs777049051	Hemolytic uremic syndrome atypical 1 (AHUS1) [MIM:235400]
CFH	P08603	VAR_025869	p.Thr956Met	LP/P	rs145975787	Hemolytic uremic syndrome atypical 1 (AHUS1) [MIM:235400]
CFH	P08603	VAR_025870	p.Trp978Cys	LP/P	-	Hemolytic uremic syndrome atypical 1 (AHUS1) [MIM:235400]
CFH	P08603	VAR_025871	p.Tyr1021Phe	LP/P	-	Hemolytic uremic syndrome atypical 1 (AHUS1) [MIM:235400]
CFH	P08603	VAR_025872	p.Cys1043Arg	LP/P	-	Hemolytic uremic syndrome atypical 1 (AHUS1) [MIM:235400]
CFH	P08603	VAR_025873	p.Gln1076Glu	LP/P	rs62625015	Complement factor H deficiency (CFHD) [MIM:609814]
CFH	P08603	VAR_025874	p.Asp1119Gly	LP/P	rs575109631	Complement factor H deficiency (CFHD) [MIM:609814]
CFH	P08603	VAR_025875	p.Val1134Gly	LP/P	-	Hemolytic uremic syndrome atypical 1 (AHUS1) [MIM:235400]
CFH	P08603	VAR_025876	p.Tyr1142Asp	LP/P	-	Hemolytic uremic syndrome atypical 1 (AHUS1) [MIM:235400]
CFH	P08603	VAR_025877	p.Trp1157Arg	LP/P	-	Hemolytic uremic syndrome atypical 1 (AHUS1) [MIM:235400]
CFH	P08603	VAR_025878	p.Cys1163Trp	LP/P	-	Hemolytic uremic syndrome atypical 1 (AHUS1) [MIM:235400]
CFH	P08603	VAR_025879	p.Trp1183Leu	LP/P	-	Hemolytic uremic syndrome atypical 1 (AHUS1) [MIM:235400]
CFH	P08603	VAR_025880	p.Trp1183Arg	LP/P	-	Hemolytic uremic syndrome atypical 1 (AHUS1) [MIM:235400]
CFH	P08603	VAR_025881	p.Thr1184Arg	LP/P	-	Complement factor H deficiency (CFHD) [MIM:609814]
CFH	P08603	VAR_025882	p.Gly1194Asp	LP/P	rs761877050	Hemolytic uremic syndrome atypical 1 (AHUS1) [MIM:235400]
CFH	P08603	VAR_025883	p.Val1197Ala	LP/P	rs460184	Hemolytic uremic syndrome atypical 1 (AHUS1) [MIM:235400]
CFH	P08603	VAR_025884	p.Glu1198Ala	LP/P	-	Hemolytic uremic syndrome atypical 1 (AHUS1) [MIM:235400]
CFH	P08603	VAR_025885	p.Arg1210Cys	LP/P	rs121913059	Complement factor H deficiency (CFHD) [MIM:609814]

CFH P08603 VAR_025885 p.Arg1210Cys LP/P rs121913059 Macular degeneration, age-related, 4 (ARMD4) [MIM:610698]
CFH P08603 VAR_025886 p.Arg1215Gly LP/P rs121913051 Hemolytic uremic syndrome atypical 1 (AHUS1) [MIM:235400]
CFH P08603 VAR_025887 p.Arg1215Gln LP/P - Complement factor H deficiency (CFHD) [MIM:609814]
CFH P08603 VAR_025888 p.Pro1226Ser LP/P - Hemolytic uremic syndrome atypical 1 (AHUS1) [MIM:235400]
CFH P08603 VAR_031978 p.Arg127Leu LP/P rs121913058 Complement factor H deficiency (CFHD) [MIM:609814]
CFH P08603 VAR_031980 p.Gln400Lys LP/P rs201671665 Hemolytic uremic syndrome atypical 1 (AHUS1) [MIM:235400]
CFH P08603 VAR_031981 p.Cys431Ser LP/P rs121913056 Complement factor H deficiency (CFHD) [MIM:609814]
CFH P08603 VAR_031982 p.Cys673Ser LP/P - Complement factor H deficiency (CFHD) [MIM:609814]
CFH P08603 VAR_031983 p.Cys673Tyr LP/P rs1391815797 Hemolytic uremic syndrome atypical 1 (AHUS1) [MIM:235400]
CFH P08603 VAR_031984 p.His893Arg LP/P rs1573076722 Hemolytic uremic syndrome atypical 1 (AHUS1) [MIM:235400]
CFH P08603 VAR_031985 p.Cys915Ser LP/P - Hemolytic uremic syndrome atypical 1 (AHUS1) [MIM:235400]
CFH P08603 VAR_031986 p.Phe1199Ser LP/P - Hemolytic uremic syndrome atypical 1 (AHUS1) [MIM:235400]
CFH P08603 VAR_043892 p.Thr493Arg LB/B rs1061171 -
CFH P08603 VAR_043893 p.Arg567Gly LB/B rs757756991 -
CFH P08603 VAR_043894 p.Val1007Leu LB/B rs534399 -
CFH P08603 VAR_043895 p.Arg1078Ser LB/B rs121913062 -
CFH P08603 VAR_043896 p.Gln1143Glu LB/B rs15809 -
CFH P08603 VAR_055683 p.Asn997Thr LB/B rs17575212 -
CFH P08603 VAR_055684 p.Ala1010Thr LB/B rs11539862 -
CFH P08603 VAR_063648 p.Cys325Tyr LP/P - Hemolytic uremic syndrome atypical 1 (AHUS1) [MIM:235400]
CFH P08603 VAR_063649 p.Val609Ile LP/P rs148165372 Hemolytic uremic syndrome atypical 1 (AHUS1) [MIM:235400]
CFH P08603 VAR_063650 p.Ile1169Leu LP/P - Hemolytic uremic syndrome atypical 1 (AHUS1) [MIM:235400]
CFH P08603 VAR_063651 p.Trp1183Cys LP/P - Hemolytic uremic syndrome atypical 1 (AHUS1) [MIM:235400]
CFHR1 Q03591 VAR_001980 p.His157Tyr LB/B rs425757 -
CFHR1 Q03591 VAR_001981 p.Leu159Val LB/B rs410232 -
CFHR1 Q03591 VAR_001982 p.Glu175Gln LB/B rs388862 -
CFHR1 Q03591 VAR_048816 p.Ala296Val LB/B rs16840561 -
CFHR3 Q02985 VAR_048817 p.His71Tyr LB/B rs17575274 -
CFHR4 Q92496 VAR_047151 p.Gly553Glu LB/B rs10494745 -
CFHR4 Q92496 VAR_070195 p.Glu125Asp LB/B rs10801578 -
CFHR4 Q92496 VAR_070196 p.Asn210Ser LB/B rs7417769 -
CFHR5 Q9BXR6 VAR_035827 p.Asn216Ser US rs147488267 A breast cancer sample
CFHR5 Q9BXR6 VAR_048818 p.Pro46Ser LB/B rs12097550 -
CFHR5 Q9BXR6 VAR_048819 p.Arg356His LB/B rs35662416 -
CFHR5 Q9BXR6 VAR_048820 p.Leu521Ile LB/B rs35957013 -

CFHR5	Q9BXR6	VAR_048821	p.Leu529Arg	LB/B	rs16840956	-
CFHR5	Q9BXR6	VAR_063652	p.Tyr277Asn	LB/B	rs318240756	-
CFHR5	Q9BXR6	VAR_063653	p.Val379Leu	LB/B	rs111327589	-
CFHR5	Q9BXR6	VAR_069090	p.Leu105Arg	LB/B	rs318240754	-
CFHR5	Q9BXR6	VAR_069091	p.Ser195Thr	LB/B	rs318240755	-
CFHR5	Q9BXR6	VAR_069092	p.Trp436Cys	LB/B	rs201265664	-
CFI	P05156	VAR_026757	p.His418Leu	LP/P	rs121964912	Complement factor I deficiency (CFI deficiency) [MIM:610984]
CFI	P05156	VAR_030343	p.Ile340Thr	LP/P	rs769419740	Hemolytic uremic syndrome atypical 3 (AHUS3) [MIM:612923]
CFI	P05156	VAR_030344	p.Asp524Val	LP/P	rs121964914	Hemolytic uremic syndrome atypical 3 (AHUS3) [MIM:612923]
CFI	P05156	VAR_034907	p.Gly243Asp	LP/P	rs121964916	Complement factor I deficiency (CFI deficiency) [MIM:610984]
CFI	P05156	VAR_034908	p.Thr300Ala	LB/B	rs11098044	-
CFI	P05156	VAR_063665	p.Pro64Leu	LP/P	rs773187287	Hemolytic uremic syndrome atypical 3 (AHUS3) [MIM:612923]
CFI	P05156	VAR_063666	p.Gly119Arg	LP/P	rs141853578	Hemolytic uremic syndrome atypical 3 (AHUS3) [MIM:612923]
CFI	P05156	VAR_063666	p.Gly119Arg	LP/P	rs141853578	Macular degeneration, age-related, 13 (ARMD13) [MIM:615439]
CFI	P05156	VAR_063667	p.His183Arg	LP/P	rs75612300	Hemolytic uremic syndrome atypical 3 (AHUS3) [MIM:612923]
CFI	P05156	VAR_063668	p.Gly287Arg	LP/P	rs182078921	Hemolytic uremic syndrome atypical 3 (AHUS3) [MIM:612923]
CFI	P05156	VAR_063669	p.Arg317Trp	LP/P	rs121964917	Hemolytic uremic syndrome atypical 3 (AHUS3) [MIM:612923]
CFI	P05156	VAR_063670	p.Ile416Leu	LP/P	rs61733901	Hemolytic uremic syndrome atypical 3 (AHUS3) [MIM:612923]
CFI	P05156	VAR_063671	p.Asp519Asn	LP/P	rs121964918	Hemolytic uremic syndrome atypical 3 (AHUS3) [MIM:612923]
CFI	P05156	VAR_063672	p.Lys522Thr	LP/P	-	Hemolytic uremic syndrome atypical 3 (AHUS3) [MIM:612923]
CFI	P05156	VAR_070843	p.Gly188Ala	LB/B	-	-
CFL2	Q9Y281	VAR_031989	p.Ala35Thr	LP/P	rs80358250	Nemaline myopathy 7 (NEM7) [MIM:610687]
CFL2	Q9Y281	VAR_036458	p.Ile47Met	US	-	A breast cancer sample
CFL2	Q9Y281	VAR_075983	p.Val7Met	US	rs397515451	Nemaline myopathy 7 (NEM7) [MIM:610687]
CFLAR	O15519	VAR_048619	p.Leu203Ile	LB/B	rs13424615	-
CFP	P27918	VAR_002002	p.Arg100Trp	LP/P	rs132630259	Properdin deficiency (PFD) [MIM:312060]
CFP	P27918	VAR_002003	p.Gln343Arg	LP/P	-	Properdin deficiency (PFD) [MIM:312060]
CFP	P27918	VAR_002004	p.Tyr414Asp	LP/P	rs132630261	Properdin deficiency (PFD) [MIM:312060]
CFP	P27918	VAR_013139	p.Gly298Val	LP/P	rs28935480	Properdin deficiency (PFD) [MIM:312060]
CFP	P27918	VAR_020395	p.Val53Met	LB/B	rs8177068	-
CFP	P27918	VAR_020396	p.Pro204Leu	LB/B	rs8177076	-
CFP	P27918	VAR_020397	p.Gly250Ser	LB/B	rs8177077	-
CFP	P27918	VAR_035813	p.Thr3Ile	US	-	A breast cancer sample
CFP	P27918	VAR_083038	p.Cys32Tyr	LP/P	-	Properdin deficiency (PFD) [MIM:312060]
CFP	P27918	VAR_083039	p.Glu244Lys	LP/P	-	Properdin deficiency (PFD) [MIM:312060]
CFTR	P13569	VAR_000101	p.Ser13Phe	LP/P	rs397508635	Cystic fibrosis (CF) [MIM:219700]

CFTR P13569 VAR_000102 p.Arg31Cys LB/B rs1800073 -
CFTR P13569 VAR_000103 p.Arg31Leu LP/P rs149353983 Cystic fibrosis (CF) [MIM:219700]
CFTR P13569 VAR_000104 p.Ser42Phe LP/P rs143456784 Cystic fibrosis (CF) [MIM:219700]
CFTR P13569 VAR_000105 p.Asp44Gly US rs1800074 Cystic fibrosis (CF) [MIM:219700]
CFTR P13569 VAR_000106 p.Asp44Val LB/B rs1800074 -
CFTR P13569 VAR_000107 p.Ser50Tyr LP/P rs397508220 Congenital bilateral absence of the vas deferens (CBAVD) [MIM:277180]
CFTR P13569 VAR_000108 p.Trp57Gly LP/P rs397508272 Cystic fibrosis (CF) [MIM:219700]
CFTR P13569 VAR_000109 p.Pro67Leu LP/P rs368505753 Cystic fibrosis (CF) [MIM:219700]
CFTR P13569 VAR_000110 p.Arg74Trp LP/P rs115545701 Congenital bilateral absence of the vas deferens (CBAVD) [MIM:277180]
CFTR P13569 VAR_000110 p.Arg74Trp LP/P rs115545701 Cystic fibrosis (CF) [MIM:219700]
CFTR P13569 VAR_000111 p.Arg75Gln LB/B rs1800076 -
CFTR P13569 VAR_000112 p.Gly85Glu LP/P rs75961395 Cystic fibrosis (CF) [MIM:219700]
CFTR P13569 VAR_000113 p.Phe87Leu LP/P rs397508403 Cystic fibrosis (CF) [MIM:219700]
CFTR P13569 VAR_000114 p.Gly91Arg LP/P rs121908750 Cystic fibrosis (CF) [MIM:219700]
CFTR P13569 VAR_000115 p.Glu92Lys LP/P rs121908751 Cystic fibrosis (CF) [MIM:219700]
CFTR P13569 VAR_000116 p.Gln98Arg LP/P rs397508464 Cystic fibrosis (CF) [MIM:219700]
CFTR P13569 VAR_000117 p.Ile105Ser LP/P - Cystic fibrosis (CF) [MIM:219700]
CFTR P13569 VAR_000118 p.Tyr109Cys LP/P rs121909031 Cystic fibrosis (CF) [MIM:219700]
CFTR P13569 VAR_000119 p.Asp110His US rs113993958 Congenital bilateral absence of the vas deferens (CBAVD) [MIM:277180]
CFTR P13569 VAR_000119 p.Asp110His US rs113993958 Cystic fibrosis (CF) [MIM:219700]
CFTR P13569 VAR_000120 p.Pro111Leu LP/P rs140502196 Congenital bilateral absence of the vas deferens (CBAVD) [MIM:277180]
CFTR P13569 VAR_000121 p.Arg117Cys LP/P rs77834169 Cystic fibrosis (CF) [MIM:219700]
CFTR P13569 VAR_000122 p.Arg117His LP/P rs78655421 Congenital bilateral absence of the vas deferens (CBAVD) [MIM:277180]
CFTR P13569 VAR_000122 p.Arg117His LP/P rs78655421 Cystic fibrosis (CF) [MIM:219700]
CFTR P13569 VAR_000123 p.Arg117Leu LP/P rs78655421 Cystic fibrosis (CF) [MIM:219700]
CFTR P13569 VAR_000124 p.Arg117Pro LP/P rs78655421 Cystic fibrosis (CF) [MIM:219700]
CFTR P13569 VAR_000125 p.Ala120Thr LP/P rs201958172 Cystic fibrosis (CF) [MIM:219700]
CFTR P13569 VAR_000126 p.His139Arg LP/P rs76371115 Cystic fibrosis (CF) [MIM:219700]
CFTR P13569 VAR_000127 p.Ala141Asp LP/P rs397508700 Cystic fibrosis (CF) [MIM:219700]
CFTR P13569 VAR_000128 p.Ile148Thr US rs35516286 Cystic fibrosis (CF) [MIM:219700]
CFTR P13569 VAR_000129 p.Gly149Arg LP/P rs397508718 Congenital bilateral absence of the vas deferens (CBAVD) [MIM:277180]
CFTR P13569 VAR_000130 p.Gly178Arg LP/P rs80282562 Cystic fibrosis (CF) [MIM:219700]
CFTR P13569 VAR_000132 p.Glu193Lys LP/P rs397508759 Congenital bilateral absence of the vas deferens (CBAVD) [MIM:277180]
CFTR P13569 VAR_000132 p.Glu193Lys LP/P rs397508759 Cystic fibrosis (CF) [MIM:219700]
CFTR P13569 VAR_000133 p.His199Gln LP/P rs397508765 Cystic fibrosis (CF) [MIM:219700]
CFTR P13569 VAR_000134 p.His199Tyr LP/P rs121908802 Cystic fibrosis (CF) [MIM:219700]
CFTR P13569 VAR_000135 p.Pro205Ser LP/P rs121908803 Cystic fibrosis (CF) [MIM:219700]
CFTR P13569 VAR_000136 p.Leu206Trp LP/P rs121908752 Congenital bilateral absence of the vas deferens (CBAVD) [MIM:277180]
CFTR P13569 VAR_000136 p.Leu206Trp LP/P rs121908752 Cystic fibrosis (CF) [MIM:219700]
CFTR P13569 VAR_000137 p.Cys225Arg US rs397508780 Cystic fibrosis (CF) [MIM:219700]

CFTR P13569 VAR_000138 p.Met244Lys LP/P rs397508790 Congenital bilateral absence of the vas deferens (CBAVD) [MIM:277180]
CFTR P13569 VAR_000139 p.Arg258Gly LP/P rs191456345 Congenital bilateral absence of the vas deferens (CBAVD) [MIM:277180]
CFTR P13569 VAR_000140 p.Asn287Tyr LP/P rs397508804 Cystic fibrosis (CF) [MIM:219700]
CFTR P13569 VAR_000141 p.Arg297Gln LP/P rs143486492 Cystic fibrosis (CF) [MIM:219700]
CFTR P13569 VAR_000142 p.Tyr301Cys US rs150691494 Cystic fibrosis (CF) [MIM:219700]
CFTR P13569 VAR_000143 p.Ser307Asn LP/P rs397508817 Cystic fibrosis (CF) [MIM:219700]
CFTR P13569 VAR_000144 p.Phe311Leu LP/P rs121909016 Cystic fibrosis (CF) [MIM:219700]
CFTR P13569 VAR_000146 p.Gly314Glu LP/P rs75763344 Cystic fibrosis (CF) [MIM:219700]
CFTR P13569 VAR_000147 p.Gly314Arg LP/P rs397508819 Cystic fibrosis (CF) [MIM:219700]
CFTR P13569 VAR_000148 p.Arg334Trp LP/P rs121909011 Congenital bilateral absence of the vas deferens (CBAVD) [MIM:277180]
CFTR P13569 VAR_000148 p.Arg334Trp LP/P rs121909011 Cystic fibrosis (CF) [MIM:219700]
CFTR P13569 VAR_000150 p.Ile336Lys LP/P rs397508139 Cystic fibrosis (CF) [MIM:219700]
CFTR P13569 VAR_000151 p.Thr338Ile LP/P rs77409459 Cystic fibrosis (CF) [MIM:219700]
CFTR P13569 VAR_000152 p.Leu346Pro LP/P rs397508146 Cystic fibrosis (CF) [MIM:219700]
CFTR P13569 VAR_000153 p.Arg347His LP/P rs77932196 Cystic fibrosis (CF) [MIM:219700]
CFTR P13569 VAR_000154 p.Arg347Leu LP/P rs77932196 Cystic fibrosis (CF) [MIM:219700]
CFTR P13569 VAR_000155 p.Arg347Pro LP/P rs77932196 Cystic fibrosis (CF) [MIM:219700]
CFTR P13569 VAR_000156 p.Arg352Gln LP/P rs121908753 Cystic fibrosis (CF) [MIM:219700]
CFTR P13569 VAR_000157 p.Gln359Lys LP/P rs76879328 Cystic fibrosis (CF) [MIM:219700]
CFTR P13569 VAR_000160 p.Ala455Glu LP/P rs74551128 Cystic fibrosis (CF) [MIM:219700]
CFTR P13569 VAR_000161 p.Val456Phe LP/P rs397508195 Cystic fibrosis (CF) [MIM:219700]
CFTR P13569 VAR_000162 p.Gly458Val LP/P rs121909009 Cystic fibrosis (CF) [MIM:219700]
CFTR P13569 VAR_000163 p.Leu467Phe LB/B rs1800089 -
CFTR P13569 VAR_000164 p.Val470Met LB/B rs213950 -
CFTR P13569 VAR_000165 p.Gly480Cys LP/P rs79282516 Cystic fibrosis (CF) [MIM:219700]
CFTR P13569 VAR_000166 p.Ser492Phe LP/P rs121909017 Cystic fibrosis (CF) [MIM:219700]
CFTR P13569 VAR_000167 p.Glu504Gln LP/P rs397508223 Cystic fibrosis (CF) [MIM:219700]
CFTR P13569 VAR_000168 p.Ile506Val LB/B rs1800091 -
CFTR P13569 VAR_000169 p.Ile507Val LB/B rs1801178 -
CFTR P13569 VAR_000172 p.Phe508Cys LB/B rs74571530 -
CFTR P13569 VAR_000173 p.Asp513Gly LP/P rs397508225 Congenital bilateral absence of the vas deferens (CBAVD) [MIM:277180]
CFTR P13569 VAR_000174 p.Val520Phe LP/P rs77646904 Cystic fibrosis (CF) [MIM:219700]
CFTR P13569 VAR_000175 p.Gly544Val LP/P rs397508241 Congenital bilateral absence of the vas deferens (CBAVD) [MIM:277180]
CFTR P13569 VAR_000176 p.Ser549Asn LP/P rs121908755 Cystic fibrosis (CF) [MIM:219700]
CFTR P13569 VAR_000177 p.Ser549Ile LP/P rs121908755 Cystic fibrosis (CF) [MIM:219700]
CFTR P13569 VAR_000178 p.Ser549Arg LP/P rs121909005 Cystic fibrosis (CF) [MIM:219700]
CFTR P13569 VAR_000179 p.Gly551Asp LP/P rs75527207 Cystic fibrosis (CF) [MIM:219700]
CFTR P13569 VAR_000180 p.Gly551Ser LP/P rs121909013 Cystic fibrosis (CF) [MIM:219700]
CFTR P13569 VAR_000181 p.Arg553Gln LP/P rs121909044 Cystic fibrosis (CF) [MIM:219700]
CFTR P13569 VAR_000182 p.Leu558Ser LP/P rs193922504 Cystic fibrosis (CF) [MIM:219700]
CFTR P13569 VAR_000183 p.Ala559Thr LP/P rs75549581 Cystic fibrosis (CF) [MIM:219700]
CFTR P13569 VAR_000184 p.Arg560Lys LP/P rs80055610 Cystic fibrosis (CF) [MIM:219700]
CFTR P13569 VAR_000185 p.Arg560Ser LP/P rs397508267 Cystic fibrosis (CF) [MIM:219700]
CFTR P13569 VAR_000186 p.Arg560Thr LP/P rs80055610 Cystic fibrosis (CF) [MIM:219700]

CFTR P13569 VAR_000187 p.Val562Ile US rs1800097 Congenital bilateral absence of the vas deferens (CBAVD) [MIM:277180]
CFTR P13569 VAR_000187 p.Val562Ile US rs1800097 Cystic fibrosis (CF) [MIM:219700]
CFTR P13569 VAR_000188 p.Val562Leu LP/P rs1800097 Cystic fibrosis (CF) [MIM:219700]
CFTR P13569 VAR_000189 p.Tyr563Asn LP/P rs121909006 Cystic fibrosis (CF) [MIM:219700]
CFTR P13569 VAR_000190 p.Tyr569Cys LP/P rs397508277 Cystic fibrosis (CF) [MIM:219700]
CFTR P13569 VAR_000191 p.Tyr569Asp LP/P rs397508276 Cystic fibrosis (CF) [MIM:219700]
CFTR P13569 VAR_000192 p.Tyr569His LP/P rs397508276 Cystic fibrosis (CF) [MIM:219700]
CFTR P13569 VAR_000193 p.Leu571Ser LP/P rs397508280 Cystic fibrosis (CF) [MIM:219700]
CFTR P13569 VAR_000194 p.Asp572Asn LP/P rs397508282 Cystic fibrosis (CF) [MIM:219700]
CFTR P13569 VAR_000195 p.Pro574His LP/P rs121908758 Cystic fibrosis (CF) [MIM:219700]
CFTR P13569 VAR_000196 p.Gly576Ala US rs1800098 Congenital bilateral absence of the vas deferens (CBAVD) [MIM:277180]
CFTR P13569 VAR_000197 p.Asp579Gly LP/P rs397508288 Cystic fibrosis (CF) [MIM:219700]
CFTR P13569 VAR_000198 p.Ile601Phe LP/P rs397508306 Cystic fibrosis (CF) [MIM:219700]
CFTR P13569 VAR_000199 p.Leu610Ser LP/P rs397508311 Cystic fibrosis (CF) [MIM:219700]
CFTR P13569 VAR_000200 p.Ala613Thr LP/P rs201978662 Cystic fibrosis (CF) [MIM:219700]
CFTR P13569 VAR_000201 p.Asp614Gly LP/P rs201124247 Cystic fibrosis (CF) [MIM:219700]
CFTR P13569 VAR_000202 p.Ile618Thr LP/P rs139468767 Cystic fibrosis (CF) [MIM:219700]
CFTR P13569 VAR_000203 p.Leu619Ser LP/P rs397508313 Cystic fibrosis (CF) [MIM:219700]
CFTR P13569 VAR_000204 p.His620Pro LP/P rs397508314 Cystic fibrosis (CF) [MIM:219700]
CFTR P13569 VAR_000205 p.His620Gln LP/P rs397508315 Cystic fibrosis (CF) [MIM:219700]
CFTR P13569 VAR_000206 p.Gly622Asp LP/P rs121908759 Congenital bilateral absence of the vas deferens (CBAVD) [MIM:277180]
CFTR P13569 VAR_000207 p.Gly628Arg LP/P rs397508316 Cystic fibrosis (CF) [MIM:219700]
CFTR P13569 VAR_000208 p.Leu633Pro LP/P rs397508318 Cystic fibrosis (CF) [MIM:219700]
CFTR P13569 VAR_000209 p.Asp648Val LP/P rs121909033 Cystic fibrosis (CF) [MIM:219700]
CFTR P13569 VAR_000210 p.Asp651Asn LP/P rs780526529 Cystic fibrosis (CF) [MIM:219700]
CFTR P13569 VAR_000211 p.Thr665Ser LP/P rs1177201180 Cystic fibrosis (CF) [MIM:219700]
CFTR P13569 VAR_000212 p.Arg668Cys US rs1800100 Congenital bilateral absence of the vas deferens (CBAVD) [MIM:277180]
CFTR P13569 VAR_000213 p.Phe693Leu US rs145540754 Cystic fibrosis (CF) [MIM:219700]
CFTR P13569 VAR_000214 p.Val754Met LP/P rs150157202 Cystic fibrosis (CF) [MIM:219700]
CFTR P13569 VAR_000215 p.Arg766Met LP/P rs397508363 Congenital bilateral absence of the vas deferens (CBAVD) [MIM:277180]
CFTR P13569 VAR_000216 p.Arg792Gly LP/P rs145449046 Congenital bilateral absence of the vas deferens (CBAVD) [MIM:277180]
CFTR P13569 VAR_000217 p.Ala800Gly LP/P rs397508373 Congenital bilateral absence of the vas deferens (CBAVD) [MIM:277180]
CFTR P13569 VAR_000218 p.Ile807Met LP/P rs1800103 Congenital bilateral absence of the vas deferens (CBAVD) [MIM:277180]
CFTR P13569 VAR_000219 p.Glu822Lys LP/P rs397508378 Cystic fibrosis (CF) [MIM:219700]
CFTR P13569 VAR_000220 p.Glu826Lys LB/B rs397508381 -
CFTR P13569 VAR_000221 p.Cys866Tyr LP/P rs193922506 Cystic fibrosis (CF) [MIM:219700]
CFTR P13569 VAR_000222 p.Ser912Leu LB/B rs121909034 -
CFTR P13569 VAR_000223 p.Tyr913Cys LP/P rs121909008 Cystic fibrosis (CF) [MIM:219700]
CFTR P13569 VAR_000224 p.Tyr917Cys LP/P rs397508428 Cystic fibrosis (CF) [MIM:219700]
CFTR P13569 VAR_000225 p.His949Tyr LP/P rs121909035 Cystic fibrosis (CF) [MIM:219700]

CFTR P13569 VAR_000226 p.Met952Ile US rs151048781 Congenital bilateral absence of the vas deferens (CBAVD) [MIM:277180]
CFTR P13569 VAR_000226 p.Met952Ile US rs151048781 Cystic fibrosis (CF) [MIM:219700]
CFTR P13569 VAR_000227 p.Leu997Phe US rs1800111 Congenital bilateral absence of the vas deferens (CBAVD) [MIM:277180]
CFTR P13569 VAR_000227 p.Leu997Phe US rs1800111 Cystic fibrosis (CF) [MIM:219700]
CFTR P13569 VAR_000228 p.Ile1005Arg LP/P rs397508479 Cystic fibrosis (CF) [MIM:219700]
CFTR P13569 VAR_000229 p.Ala1006Glu LP/P rs397508480 Cystic fibrosis (CF) [MIM:219700]
CFTR P13569 VAR_000230 p.Pro1013Leu LP/P rs193922516 Cystic fibrosis (CF) [MIM:219700]
CFTR P13569 VAR_000231 p.Met1028Ile LP/P rs200553511 Cystic fibrosis (CF) [MIM:219700]
CFTR P13569 VAR_000232 p.Phe1052Val LP/P rs150212784 Cystic fibrosis (CF) [MIM:219700]
CFTR P13569 VAR_000233 p.Gly1061Arg LP/P rs142394380 Cystic fibrosis (CF) [MIM:219700]
CFTR P13569 VAR_000234 p.Leu1065Pro LP/P rs121909036 Cystic fibrosis (CF) [MIM:219700]
CFTR P13569 VAR_000235 p.Leu1065Arg LP/P rs121909036 Cystic fibrosis (CF) [MIM:219700]
CFTR P13569 VAR_000236 p.Arg1066Cys LP/P rs78194216 Cystic fibrosis (CF) [MIM:219700]
CFTR P13569 VAR_000237 p.Arg1066His LP/P rs121909019 Cystic fibrosis (CF) [MIM:219700]
CFTR P13569 VAR_000238 p.Arg1066Leu LP/P rs121909019 Cystic fibrosis (CF) [MIM:219700]
CFTR P13569 VAR_000239 p.Ala1067Thr LP/P rs121909020 Cystic fibrosis (CF) [MIM:219700]
CFTR P13569 VAR_000240 p.Ala1067Val LB/B rs1800114 -
CFTR P13569 VAR_000241 p.Arg1070Gln LP/P rs78769542 Cystic fibrosis (CF) [MIM:219700]
CFTR P13569 VAR_000242 p.Arg1070Pro LP/P rs78769542 Cystic fibrosis (CF) [MIM:219700]
CFTR P13569 VAR_000243 p.Gln1071Pro LP/P rs121909037 Cystic fibrosis (CF) [MIM:219700]
CFTR P13569 VAR_000244 p.Pro1072Leu LP/P - Cystic fibrosis (CF) [MIM:219700]
CFTR P13569 VAR_000245 p.Leu1077Pro LP/P rs139304906 Cystic fibrosis (CF) [MIM:219700]
CFTR P13569 VAR_000246 p.His1085Arg LP/P rs79635528 Cystic fibrosis (CF) [MIM:219700]
CFTR P13569 VAR_000247 p.Trp1098Arg LP/P rs397508531 Cystic fibrosis (CF) [MIM:219700]
CFTR P13569 VAR_000248 p.Met1101Lys LP/P rs36210737 Cystic fibrosis (CF) [MIM:219700]
CFTR P13569 VAR_000249 p.Met1137Val LP/P rs397508553 Cystic fibrosis (CF) [MIM:219700]
CFTR P13569 VAR_000251 p.Asp1152His LP/P rs75541969 Congenital bilateral absence of the vas deferens (CBAVD) [MIM:277180]
CFTR P13569 VAR_000251 p.Asp1152His LP/P rs75541969 Cystic fibrosis (CF) [MIM:219700]
CFTR P13569 VAR_000252 p.Arg1162Leu LB/B rs1800120 -
CFTR P13569 VAR_000253 p.Thr1220Ile LB/B rs1800123 -
CFTR P13569 VAR_000254 p.Ile1234Val LP/P rs75389940 Cystic fibrosis (CF) [MIM:219700]
CFTR P13569 VAR_000255 p.Ser1235Arg LP/P rs34911792 Cystic fibrosis (CF) [MIM:219700]
CFTR P13569 VAR_000256 p.Gly1244Glu LP/P rs267606723 Cystic fibrosis (CF) [MIM:219700]
CFTR P13569 VAR_000257 p.Gly1249Glu LP/P rs121909040 Cystic fibrosis (CF) [MIM:219700]
CFTR P13569 VAR_000258 p.Ser1251Asn LP/P rs74503330 Cystic fibrosis (CF) [MIM:219700]
CFTR P13569 VAR_000259 p.Ser1255Pro LP/P rs121909041 Cystic fibrosis (CF) [MIM:219700]
CFTR P13569 VAR_000260 p.Asp1270Asn LP/P rs11971167 Congenital bilateral absence of the vas deferens (CBAVD) [MIM:277180]
CFTR P13569 VAR_000260 p.Asp1270Asn LP/P rs11971167 Cystic fibrosis (CF) [MIM:219700]
CFTR P13569 VAR_000261 p.Trp1282Arg LP/P rs397508616 Cystic fibrosis (CF) [MIM:219700]
CFTR P13569 VAR_000262 p.Arg1283Met LP/P rs77902683 Cystic fibrosis (CF) [MIM:219700]
CFTR P13569 VAR_000263 p.Phe1286Ser LP/P rs121909028 Cystic fibrosis (CF) [MIM:219700]
CFTR P13569 VAR_000264 p.Gln1291His LP/P rs121909015 Cystic fibrosis (CF) [MIM:219700]
CFTR P13569 VAR_000265 p.Gln1291Arg LP/P rs397508621 Cystic fibrosis (CF) [MIM:219700]
CFTR P13569 VAR_000266 p.Asn1303His LP/P rs121909042 Cystic fibrosis (CF) [MIM:219700]
CFTR P13569 VAR_000267 p.Asn1303Lys LP/P rs80034486 Cystic fibrosis (CF) [MIM:219700]

CFTR P13569 VAR_000268 p.Gly1349Asp LP/P rs193922525 Cystic fibrosis (CF) [MIM:219700]
CFTR P13569 VAR_000269 p.Ala1364Val LP/P rs397508670 Congenital bilateral absence of the vas deferens (CBAVD) [MIM:277180]
CFTR P13569 VAR_000270 p.Val1397Glu LP/P rs397508691 Cystic fibrosis (CF) [MIM:219700]
CFTR P13569 VAR_009895 p.Leu138Pro LB/B rs1800078 -
CFTR P13569 VAR_009896 p.Arg170His US rs1800079 Congenital bilateral absence of the vas deferens (CBAVD) [MIM:277180]
CFTR P13569 VAR_009897 p.Ser182Gly LB/B rs1800080 -
CFTR P13569 VAR_009898 p.Val322Met LB/B rs1800085 -
CFTR P13569 VAR_009899 p.Thr351Ser LB/B rs1800086 -
CFTR P13569 VAR_009900 p.Gln353His LB/B rs1800087 -
CFTR P13569 VAR_009901 p.Ile506Met LB/B rs1800092 -
CFTR P13569 VAR_009902 p.Ser654Gly LB/B rs1800099 -
CFTR P13569 VAR_009903 p.Tyr903His LB/B rs1800106 -
CFTR P13569 VAR_009904 p.Ser909Ile LB/B rs1800107 -
CFTR P13569 VAR_009905 p.Leu967Ser LB/B rs1800110 -
CFTR P13569 VAR_011564 p.Arg1070Trp LP/P rs202179988 Congenital bilateral absence of the vas deferens (CBAVD) [MIM:277180]
CFTR P13569 VAR_011565 p.Met1101Arg LP/P rs36210737 Cystic fibrosis (CF) [MIM:219700]
CFTR P13569 VAR_048150 p.Lys532Glu LB/B rs35032490 -
CFTR P13569 VAR_048151 p.Ser605Phe LB/B rs766874 -
CFTR P13569 VAR_048152 p.Arg1453Trp LB/B rs4148725 -
CFTR P13569 VAR_080303 p.Val232Asp US rs397508783 Congenital bilateral absence of the vas deferens (CBAVD) [MIM:277180]
CFTR P13569 VAR_080304 p.Asp443Tyr US rs147422190 Congenital bilateral absence of the vas deferens (CBAVD) [MIM:277180]
CFTR P13569 VAR_080306 p.Ile556Val US rs75789129 Congenital bilateral absence of the vas deferens (CBAVD) [MIM:277180]
CFTR P13569 VAR_080307 p.Ala561Glu LP/P rs121909047 Cystic fibrosis (CF) [MIM:219700]
CFTR P13569 VAR_080311 p.Val938Gly US rs193922511 Congenital bilateral absence of the vas deferens (CBAVD) [MIM:277180]
CFTR P13569 VAR_080312 p.Ala959Val US rs397508448 Congenital bilateral absence of the vas deferens (CBAVD) [MIM:277180]
CFTR P13569 VAR_080313 p.Ser977Phe US rs141033578 Congenital bilateral absence of the vas deferens (CBAVD) [MIM:277180]
CFTR P13569 VAR_080315 p.Ile1027Thr LB/B rs1800112 -
CFTR P13569 VAR_080316 p.Tyr1032Cys US rs144055758 Congenital bilateral absence of the vas deferens (CBAVD) [MIM:277180]
CFTR P13569 VAR_080318 p.Gly1069Arg US rs200321110 Congenital bilateral absence of the vas deferens (CBAVD) [MIM:277180]
CFTR P13569 VAR_080320 p.Val1153Glu US rs397508567 Congenital bilateral absence of the vas deferens (CBAVD) [MIM:277180]
CFTR P13569 VAR_080322 p.Lys1200Glu US - Cystic fibrosis (CF) [MIM:219700]
CFTR P13569 VAR_080325 p.Gln1352His US rs113857788 Congenital bilateral absence of the vas deferens (CBAVD) [MIM:277180]
CGAS Q8N884 VAR_033677 p.Pro261His LB/B rs610913 -
CGAS Q8N884 VAR_050811 p.Thr35Asn LB/B rs9352000 -
CGB1 A6NKQ9 VAR_055838 p.Pro185Ser LB/B rs35371968 -
CGB3 P0DN86 VAR_003188 p.Asp137Ala LB/B rs200199557 -

CGB3	P0DN86	VAR_014585	p.Phe4Leu	LB/B	rs767100833	-
CGB3	P0DN86	VAR_014586	p.Lys22Arg	LB/B	rs201575305	-
CGB3	P0DN86	VAR_014587	p.Thr35Ile	LB/B	rs201780746	-
CGB3	P0DN86	VAR_015231	p.Thr18Ala	LB/B	rs201240617	-
CGB3	P0DN86	VAR_015232	p.Pro24Met	US	-	-
CGB3	P0DN86	VAR_015233	p.Arg28Trp	LB/B	rs1261895475	-
CGB3	P0DN86	VAR_015234	p.Arg30His	LB/B	rs201373221	-
CGB3	P0DN86	VAR_015235	p.Ser147Cys	LB/B	-	-
CGN	Q9P2M7	VAR_057809	p.Arg479Gln	LB/B	rs12038198	-
CGNL1	Q0VF96	VAR_037606	p.Thr380Pro	LB/B	rs1280395	-
CGNL1	Q0VF96	VAR_037607	p.Ser459Phe	LB/B	rs7182648	-
CGNL1	Q0VF96	VAR_037608	p.Thr511Ala	LB/B	rs1280396	-
CGNL1	Q0VF96	VAR_037609	p.Leu1101Val	LB/B	rs1620402	-
CGNL1	Q0VF96	VAR_037610	p.Met1270Val	LB/B	rs16977594	-
CGREF1	Q99674	VAR_047648	p.Ala212Val	LB/B	rs11893478	-
CGREF1	Q99674	VAR_047649	p.Lys232Asn	LB/B	rs11889831	-
CGREF1	Q99674	VAR_047650	p.Gly243Glu	LB/B	rs1057389	-
CGREF1	Q99674	VAR_061088	p.Ile116Met	LB/B	rs2384572	-
CGREF1	Q99674	VAR_081928	p.Glu226Gly	LB/B	rs74360681	-
CGREF1	Q99674	VAR_081929	p.Lys232Glu	LB/B	rs11889832	-
CGRRF1	Q99675	VAR_052081	p.Cys117Tyr	LB/B	rs11555279	-
CH25H	O95992	VAR_048899	p.Leu133Pro	LB/B	rs17117295	-
CHAC2	Q8WUX2	VAR_038123	p.Arg82Gly	LB/B	rs17851583	-
CHAD	O15335	VAR_030631	p.Thr350Ile	LB/B	rs2231510	-
CHAD	O15335	VAR_052019	p.Arg312Gln	LB/B	rs35218093	-
CHADL	Q6NUI6	VAR_059805	p.Gln710Arg	LB/B	rs9619955	-
CHADL	Q6NUI6	VAR_061805	p.Asp721Asn	LB/B	rs9619954	-
CHAF1A	Q13111	VAR_055329	p.Asp167Val	LB/B	rs35651457	-
CHAF1A	Q13111	VAR_055330	p.Met239Val	LB/B	rs2230635	-
CHAF1A	Q13111	VAR_055331	p.Lys850Arg	LB/B	rs8100525	-
CHAF1A	Q13111	VAR_055332	p.Ala923Val	LB/B	rs9352	-
CHAF1A	Q13111	VAR_055333	p.Ala950Ser	LB/B	rs243383	-
CHAF1B	Q13112	VAR_053387	p.Lys506Gln	LB/B	rs74900401	-
CHAMP1	Q96JM3	VAR_027270	p.Leu568Val	LB/B	rs3764522	-
CHAMP1	Q96JM3	VAR_027271	p.Pro604Arg	LB/B	rs12428067	-
CHAMP1	Q96JM3	VAR_052910	p.Lys591Arg	LB/B	rs35564629	-
CHAT	P28329	VAR_011666	p.Leu210Pro	LP/P	rs121912820	Myasthenic syndrome, congenital, 6, presynaptic (CMS6) [MIM:254210]
CHAT	P28329	VAR_011667	p.Pro211Ala	LP/P	rs121912815	Myasthenic syndrome, congenital, 6, presynaptic (CMS6) [MIM:254210]
CHAT	P28329	VAR_011668	p.Ile305Thr	LP/P	rs75466054	Myasthenic syndrome, congenital, 6, presynaptic (CMS6) [MIM:254210]
CHAT	P28329	VAR_011669	p.Arg420Cys	LP/P	rs121912822	Myasthenic syndrome, congenital, 6, presynaptic (CMS6) [MIM:254210]
CHAT	P28329	VAR_011670	p.Glu441Lys	LP/P	rs121912816	Myasthenic syndrome, congenital, 6, presynaptic (CMS6) [MIM:254210]
CHAT	P28329	VAR_011671	p.Arg482Gly	LP/P	rs121912818	Myasthenic syndrome, congenital, 6, presynaptic (CMS6) [MIM:254210]

CHAT P28329 VAR_011672 p.Ser498Leu LP/P rs121912821 Myasthenic syndrome, congenital, 6, presynaptic (CMS6) [MIM:254210]
CHAT P28329 VAR_011673 p.Val506Leu LP/P rs121912817 Myasthenic syndrome, congenital, 6, presynaptic (CMS6) [MIM:254210]
CHAT P28329 VAR_011674 p.Arg560His LP/P rs121912819 Myasthenic syndrome, congenital, 6, presynaptic (CMS6) [MIM:254210]
CHAT P28329 VAR_011675 p.Ala120Thr LB/B rs3810950 -
CHAT P28329 VAR_011676 p.Ala392Gly LB/B - -
CHAT P28329 VAR_038605 p.Ile336Thr LP/P rs121912823 Myasthenic syndrome, congenital, 6, presynaptic (CMS6) [MIM:254210]
CHAT P28329 VAR_046683 p.Asp47Glu LB/B rs3810948 -
CHAT P28329 VAR_046684 p.Arg222Pro LB/B rs8178989 -
CHAT P28329 VAR_046685 p.Leu243Phe LB/B rs8178990 -
CHAT P28329 VAR_046686 p.Pro299Leu LB/B rs868749 -
CHAT P28329 VAR_046687 p.Asp400Asn LB/B rs8178991 -
CHAT P28329 VAR_046688 p.Val461Met LB/B rs4838544 -
CHCHD10 Q8WYQ3 VAR_071805 p.Pro34Ser LP/P rs551521196 Frontotemporal dementia and/or amyotrophic lateral sclerosis 2 (FTDALS2) [MIM:615911]
CHCHD10 Q8WYQ3 VAR_071806 p.Ser59Leu LP/P rs587777574 Frontotemporal dementia and/or amyotrophic lateral sclerosis 2 (FTDALS2) [MIM:615911]
CHCHD10 Q8WYQ3 VAR_073283 p.Arg15Ser US rs730880032 Myopathy, isolated mitochondrial, autosomal dominant (IMMD) [MIM:616209]
CHCHD10 Q8WYQ3 VAR_073284 p.Gly58Arg LP/P rs730880033 Myopathy, isolated mitochondrial, autosomal dominant (IMMD) [MIM:616209]
CHCHD10 Q8WYQ3 VAR_073285 p.Gly66Val LP/P rs730880031 Spinal muscular atrophy, Jokela type (SMAJ) [MIM:615048]
CHCHD2 Q9Y6H1 VAR_048699 p.His78Asn LB/B rs11546418 -
CHCHD2 Q9Y6H1 VAR_076293 p.Pro2Leu LB/B rs142444896 -
CHCHD2 Q9Y6H1 VAR_076294 p.Gly4Arg LB/B rs778328496 -
CHCHD2 Q9Y6H1 VAR_076295 p.Pro14Ser LB/B rs137965562 -
CHCHD2 Q9Y6H1 VAR_076296 p.Pro34Leu LB/B rs371198317 -
CHCHD2 Q9Y6H1 VAR_076297 p.Ala37Val LB/B rs1427631250 -
CHCHD2 Q9Y6H1 VAR_076298 p.Ala49Val LB/B rs151213700 -
CHCHD2 Q9Y6H1 VAR_076299 p.Thr61Ile LP/P rs864309650 Parkinson disease 22 (PARK22) [MIM:616710]
CHCHD2 Q9Y6H1 VAR_076300 p.Ala93Val LB/B rs748182315 -
CHCHD2 Q9Y6H1 VAR_076301 p.Arg145Gln US rs752169833 Parkinson disease 22 (PARK22) [MIM:616710]
CHCHD6 Q9BRQ6 VAR_024412 p.Ala95Ser LB/B rs2272487 -
CHD1 O14646 VAR_055652 p.Pro264Thr LB/B rs10062803 -
CHD1 O14646 VAR_080265 p.Arg141Gly LP/P rs1064795875 Pilarowski-Bjornsson syndrome (PILBOS) [MIM:617682]
CHD1 O14646 VAR_080266 p.Arg460Lys LP/P rs1554078856 Pilarowski-Bjornsson syndrome (PILBOS) [MIM:617682]
CHD1 O14646 VAR_080267 p.Arg618Gln LP/P rs1554078349 Pilarowski-Bjornsson syndrome (PILBOS) [MIM:617682]
CHD1 O14646 VAR_080268 p.Arg1708Gln LP/P rs1293161341 Pilarowski-Bjornsson syndrome (PILBOS) [MIM:617682]
CHD1L Q86WJ1 VAR_042954 p.Arg25Pro LB/B rs11588753 -
CHD1L Q86WJ1 VAR_042955 p.His350Gln LB/B rs17356233 -
CHD1L Q86WJ1 VAR_042956 p.Glu649Ala LB/B rs13374920 -

CHD1L Q86WJ1 VAR_042957 p.Ser743Cys LB/B rs2275249 -
CHD1L Q86WJ1 VAR_042958 p.Ser885Ala LB/B rs4950394 -
CHD2 O14647 VAR_061099 p.Gly1574Ala LB/B rs56227200 -
CHD2 O14647 VAR_070209 p.Trp548Arg LP/P rs864309537 Epileptic encephalopathy, childhood-onset (EEOC) [MIM:615369]
CHD2 O14647 VAR_070210 p.Leu823Pro LP/P rs864309540 Epileptic encephalopathy, childhood-onset (EEOC) [MIM:615369]
CHD3 Q12873 VAR_048728 p.Ala3Val LB/B rs931543 -
CHD3 Q12873 VAR_081507 p.His886Arg LP/P rs1567855081 Snijders Blok-Campeau syndrome (SNIBCPS) [MIM:618205]
CHD3 Q12873 VAR_081508 p.Leu915Phe LP/P rs1567855669 Snijders Blok-Campeau syndrome (SNIBCPS) [MIM:618205]
CHD3 Q12873 VAR_081509 p.Glu921Lys LP/P rs1567855704 Snijders Blok-Campeau syndrome (SNIBCPS) [MIM:618205]
CHD3 Q12873 VAR_081510 p.Gly961Glu LP/P rs1567856045 Snijders Blok-Campeau syndrome (SNIBCPS) [MIM:618205]
CHD3 Q12873 VAR_081511 p.Arg985Gln LP/P rs1567856331 Snijders Blok-Campeau syndrome (SNIBCPS) [MIM:618205]
CHD3 Q12873 VAR_081512 p.Arg985Trp LP/P rs1555611722 Snijders Blok-Campeau syndrome (SNIBCPS) [MIM:618205]
CHD3 Q12873 VAR_081514 p.Asp1120His LP/P - Snijders Blok-Campeau syndrome (SNIBCPS) [MIM:618205]
CHD3 Q12873 VAR_081515 p.Arg1121Pro LP/P rs1567860112 Snijders Blok-Campeau syndrome (SNIBCPS) [MIM:618205]
CHD3 Q12873 VAR_081516 p.Thr1136Ile LP/P rs1567860640 Snijders Blok-Campeau syndrome (SNIBCPS) [MIM:618205]
CHD3 Q12873 VAR_081517 p.Trp1158Arg LP/P rs1567860891 Snijders Blok-Campeau syndrome (SNIBCPS) [MIM:618205]
CHD3 Q12873 VAR_081518 p.Asn1159Lys LP/P rs754919272 Snijders Blok-Campeau syndrome (SNIBCPS) [MIM:618205]
CHD3 Q12873 VAR_081519 p.His1161Arg LP/P rs1567860919 Snijders Blok-Campeau syndrome (SNIBCPS) [MIM:618205]
CHD3 Q12873 VAR_081520 p.Arg1169Trp LP/P rs1567861468 Snijders Blok-Campeau syndrome (SNIBCPS) [MIM:618205]
CHD3 Q12873 VAR_081521 p.His1171Arg LP/P rs1567861489 Snijders Blok-Campeau syndrome (SNIBCPS) [MIM:618205]
CHD3 Q12873 VAR_081522 p.Arg1172Gln LP/P rs1567861501 Snijders Blok-Campeau syndrome (SNIBCPS) [MIM:618205]
CHD3 Q12873 VAR_081523 p.Arg1187Pro US rs1567861571 Snijders Blok-Campeau syndrome (SNIBCPS) [MIM:618205]
CHD3 Q12873 VAR_081524 p.Leu1236Pro LP/P rs1567861894 Snijders Blok-Campeau syndrome (SNIBCPS) [MIM:618205]
CHD3 Q12873 VAR_081525 p.Arg1342Gln LP/P rs1567863732 Snijders Blok-Campeau syndrome (SNIBCPS) [MIM:618205]
CHD3 Q12873 VAR_081526 p.Arg1881Leu LP/P rs1567877108 Snijders Blok-Campeau syndrome (SNIBCPS) [MIM:618205]
CHD4 Q14839 VAR_031674 p.Glu139Asp LB/B rs1639122 -
CHD4 Q14839 VAR_031675 p.Ser1648Leu LB/B rs35512811 -
CHD4 Q14839 VAR_031676 p.Ile1655Val LB/B rs16932768 -

CHD4 Q14839 VAR_077146 p.Ser851Tyr LP/P rs886039916 Sifrim-Hitz-Weiss syndrome (SIHIWES) [MIM:617159]
CHD4 Q14839 VAR_077147 p.Gly1003Asp LP/P - Sifrim-Hitz-Weiss syndrome (SIHIWES) [MIM:617159]
CHD4 Q14839 VAR_077148 p.Arg1068His LP/P rs886039915 Sifrim-Hitz-Weiss syndrome (SIHIWES) [MIM:617159]
CHD4 Q14839 VAR_077149 p.Arg1127Gln LP/P rs886039917 Sifrim-Hitz-Weiss syndrome (SIHIWES) [MIM:617159]
CHD4 Q14839 VAR_077150 p.Trp1148Leu LP/P rs886039919 Sifrim-Hitz-Weiss syndrome (SIHIWES) [MIM:617159]
CHD4 Q14839 VAR_077151 p.Arg1173Leu LP/P rs886039918 Sifrim-Hitz-Weiss syndrome (SIHIWES) [MIM:617159]
CHD4 Q14839 VAR_077152 p.Val1608Ile US rs201992075 Sifrim-Hitz-Weiss syndrome (SIHIWES) [MIM:617159]
CHD5 Q8TDI0 VAR_035475 p.Val45Met US rs1470692239 A breast cancer sample
CHD5 Q8TDI0 VAR_035476 p.Asp119Asn US - A breast cancer sample
CHD5 Q8TDI0 VAR_035477 p.Arg667Gly US - A breast cancer sample
CHD5 Q8TDI0 VAR_048729 p.Ser1253Ile LB/B rs6657997 -
CHD5 Q8TDI0 VAR_048730 p.Ser1539Pro LB/B rs2843493 -
CHD6 Q8TD26 VAR_023363 p.His2161Gln LB/B rs3817893 -
CHD6 Q8TD26 VAR_059213 p.Gln780His LB/B rs4474937 -
CHD7 Q9P2D1 VAR_021059 p.Ile1028Val LP/P rs121434338 CHARGE syndrome (CHARGES) [MIM:214800]
CHD7 Q9P2D1 VAR_021060 p.Leu1257Arg LP/P rs121434339 CHARGE syndrome (CHARGES) [MIM:214800]
CHD7 Q9P2D1 VAR_033245 p.Trp1031Gly LP/P - CHARGE syndrome (CHARGES) [MIM:214800]
CHD7 Q9P2D1 VAR_033246 p.Gln1214Arg LP/P - CHARGE syndrome (CHARGES) [MIM:214800]
CHD7 Q9P2D1 VAR_033247 p.Leu1294Pro LP/P rs864309609 CHARGE syndrome (CHARGES) [MIM:214800]
CHD7 Q9P2D1 VAR_033248 p.Leu1815Pro LP/P - CHARGE syndrome (CHARGES) [MIM:214800]
CHD7 Q9P2D1 VAR_033249 p.His2096Arg LP/P rs587783451 CHARGE syndrome (CHARGES) [MIM:214800]
CHD7 Q9P2D1 VAR_033250 p.Arg2319Ser LP/P rs121434341 CHARGE syndrome (CHARGES) [MIM:214800]
CHD7 Q9P2D1 VAR_033251 p.Phe2750Leu LB/B rs3750308 -
CHD7 Q9P2D1 VAR_048731 p.Met340Val LB/B rs41305525 -
CHD7 Q9P2D1 VAR_054623 p.His55Arg LP/P rs121434345 Hypogonadotropic hypogonadism 5 with or without anosmia (HH5) [MIM:612370]
CHD7 Q9P2D1 VAR_054624 p.Ser834Phe LP/P rs121434344 Hypogonadotropic hypogonadism 5 with or without anosmia (HH5) [MIM:612370]
CHD7 Q9P2D1 VAR_054625 p.Ala2789Thr LB/B rs200140270 -
CHD7 Q9P2D1 VAR_054626 p.Pro2880Leu LP/P rs113938624 Hypogonadotropic hypogonadism 5 with or without anosmia (HH5) [MIM:612370]
CHD7 Q9P2D1 VAR_054627 p.Lys2948Glu LP/P rs1467824778 Hypogonadotropic hypogonadism 5 with or without anosmia (HH5) [MIM:612370]
CHD7 Q9P2D1 VAR_068104 p.Met41Ile US rs756851968 CHARGE syndrome (CHARGES) [MIM:214800]
CHD7 Q9P2D1 VAR_068105 p.Pro86Arg US rs1355349547 CHARGE syndrome (CHARGES) [MIM:214800]
CHD7 Q9P2D1 VAR_068106 p.Ser103Thr LB/B rs41272435 -
CHD7 Q9P2D1 VAR_068107 p.Gln201Arg LB/B rs764496155 -
CHD7 Q9P2D1 VAR_068108 p.Val238Met US rs200898742 CHARGE syndrome (CHARGES) [MIM:214800]
CHD7 Q9P2D1 VAR_068109 p.Pro369Ala LB/B rs766747354 -
CHD7 Q9P2D1 VAR_068110 p.Ser466Leu LB/B rs71640285 -
CHD7 Q9P2D1 VAR_068111 p.Gly522Val LB/B rs142962579 -
CHD7 Q9P2D1 VAR_068112 p.Pro558Ala US rs746837682 CHARGE syndrome (CHARGES) [MIM:214800]
CHD7 Q9P2D1 VAR_068113 p.Gly636Val LB/B rs529321177 -

CHD7 Q9P2D1 VAR_068114 p.Ser699Thr US - CHARGE syndrome (CHARGES) [MIM:214800]
CHD7 Q9P2D1 VAR_068115 p.Asp728Asn US rs756365280 CHARGE syndrome (CHARGES) [MIM:214800]
CHD7 Q9P2D1 VAR_068116 p.Gly744Ser LB/B rs141947938 -
CHD7 Q9P2D1 VAR_068117 p.Glu871Asp LP/P - CHARGE syndrome (CHARGES) [MIM:214800]
CHD7 Q9P2D1 VAR_068118 p.Thr894Ala US rs377662366 CHARGE syndrome (CHARGES) [MIM:214800]
CHD7 Q9P2D1 VAR_068119 p.Lys907Thr US - CHARGE syndrome (CHARGES) [MIM:214800]
CHD7 Q9P2D1 VAR_068120 p.Thr917Met US rs1165711448 CHARGE syndrome (CHARGES) [MIM:214800]
CHD7 Q9P2D1 VAR_068121 p.Arg938Lys US rs763978472 CHARGE syndrome (CHARGES) [MIM:214800]
CHD7 Q9P2D1 VAR_068122 p.Arg944His US rs117506164 CHARGE syndrome (CHARGES) [MIM:214800]
CHD7 Q9P2D1 VAR_068123 p.Arg947Gln US rs768481542 CHARGE syndrome (CHARGES) [MIM:214800]
CHD7 Q9P2D1 VAR_068124 p.Leu1020Ser LP/P rs1057521077 CHARGE syndrome (CHARGES) [MIM:214800]
CHD7 Q9P2D1 VAR_068125 p.Glu1203Gln US - CHARGE syndrome (CHARGES) [MIM:214800]
CHD7 Q9P2D1 VAR_068126 p.Val1208Asp US rs886040988 CHARGE syndrome (CHARGES) [MIM:214800]
CHD7 Q9P2D1 VAR_068127 p.Leu1322Pro US - CHARGE syndrome (CHARGES) [MIM:214800]
CHD7 Q9P2D1 VAR_068128 p.Arg1345Cys US rs1563644113 CHARGE syndrome (CHARGES) [MIM:214800]
CHD7 Q9P2D1 VAR_068128 p.Arg1345Cys US rs1563644113 Hypogonadotropic hypogonadism 5 with or without anosmia (HH5) [MIM:612370]
CHD7 Q9P2D1 VAR_068129 p.Gln1395His LP/P - CHARGE syndrome (CHARGES) [MIM:214800]
CHD7 Q9P2D1 VAR_068130 p.Thr1416Arg US rs770166812 CHARGE syndrome (CHARGES) [MIM:214800]
CHD7 Q9P2D1 VAR_068131 p.Lys1457Gln US - CHARGE syndrome (CHARGES) [MIM:214800]
CHD7 Q9P2D1 VAR_068132 p.Phe1576Cys US - CHARGE syndrome (CHARGES) [MIM:214800]
CHD7 Q9P2D1 VAR_068133 p.Gly1617Ser US rs886040993 CHARGE syndrome (CHARGES) [MIM:214800]
CHD7 Q9P2D1 VAR_068134 p.Gly1684Ser LP/P rs1554602465 CHARGE syndrome (CHARGES) [MIM:214800]
CHD7 Q9P2D1 VAR_068134 p.Gly1684Ser LP/P rs1554602465 Hypogonadotropic hypogonadism 5 with or without anosmia (HH5) [MIM:612370]
CHD7 Q9P2D1 VAR_068135 p.Leu1739Arg LP/P - CHARGE syndrome (CHARGES) [MIM:214800]
CHD7 Q9P2D1 VAR_068136 p.Asp1791Glu US - CHARGE syndrome (CHARGES) [MIM:214800]
CHD7 Q9P2D1 VAR_068137 p.Gly1802Asp LP/P - CHARGE syndrome (CHARGES) [MIM:214800]
CHD7 Q9P2D1 VAR_068138 p.Asp1866Gly US - CHARGE syndrome (CHARGES) [MIM:214800]
CHD7 Q9P2D1 VAR_068139 p.Ala1950Thr US rs201423234 CHARGE syndrome (CHARGES) [MIM:214800]
CHD7 Q9P2D1 VAR_068140 p.Arg2065His US rs1197494895 CHARGE syndrome (CHARGES) [MIM:214800]
CHD7 Q9P2D1 VAR_068141 p.Arg2065Ser LP/P - CHARGE syndrome (CHARGES) [MIM:214800]
CHD7 Q9P2D1 VAR_068142 p.Ser2084Gly US rs201083157 CHARGE syndrome (CHARGES) [MIM:214800]
CHD7 Q9P2D1 VAR_068143 p.Gly2103Asp US rs794727555 CHARGE syndrome (CHARGES) [MIM:214800]
CHD7 Q9P2D1 VAR_068144 p.Gly2108Arg LP/P rs121434343 CHARGE syndrome (CHARGES) [MIM:214800]
CHD7 Q9P2D1 VAR_068144 p.Gly2108Arg LP/P rs121434343 Hypogonadotropic hypogonadism 5 with or without anosmia (HH5) [MIM:612370]
CHD7 Q9P2D1 VAR_068145 p.Ile2116Asn LP/P - CHARGE syndrome (CHARGES) [MIM:214800]
CHD7 Q9P2D1 VAR_068146 p.Ala2160Thr LB/B rs61753399 -
CHD7 Q9P2D1 VAR_068147 p.Ala2225Thr LB/B rs374408098 -
CHD7 Q9P2D1 VAR_068148 p.Arg2319Cys US rs121434341 CHARGE syndrome (CHARGES) [MIM:214800]
CHD7 Q9P2D1 VAR_068149 p.Gly2330Ala LB/B rs77704609 -
CHD7 Q9P2D1 VAR_068150 p.Arg2418Gly LP/P - CHARGE syndrome (CHARGES) [MIM:214800]
CHD7 Q9P2D1 VAR_068151 p.Arg2495Ser US rs547209998 CHARGE syndrome (CHARGES) [MIM:214800]
CHD7 Q9P2D1 VAR_068152 p.Met2527Leu LB/B rs192129249 -
CHD7 Q9P2D1 VAR_068153 p.Arg2653Gln LB/B rs747082615 -
CHD7 Q9P2D1 VAR_068154 p.Pro2683Ser US rs201319489 CHARGE syndrome (CHARGES) [MIM:214800]
CHD7 Q9P2D1 VAR_068155 p.Arg2702Cys US rs1373315351 CHARGE syndrome (CHARGES) [MIM:214800]
CHD7 Q9P2D1 VAR_068156 p.Ala2733Thr US rs370231679 CHARGE syndrome (CHARGES) [MIM:214800]

CHD7	Q9P2D1	VAR_068157	p.Leu2806Val	LB/B	rs45521933	-
CHD7	Q9P2D1	VAR_068158	p.Ser2857Ala	LB/B	-	-
CHD7	Q9P2D1	VAR_068159	p.Val2931Met	US	rs370271088	CHARGE syndrome (CHARGES) [MIM:214800]
CHD7	Q9P2D1	VAR_068160	p.Leu2984Phe	LB/B	rs184814820	-
CHD7	Q9P2D1	VAR_068374	p.Met37Leu	LB/B	rs1416709395	-
CHD7	Q9P2D1	VAR_068375	p.Tyr72Cys	US	rs767819417	CHARGE syndrome (CHARGES) [MIM:214800]
CHD7	Q9P2D1	VAR_068376	p.Thr93Ala	LB/B	rs398124317	-
CHD7	Q9P2D1	VAR_068377	p.Ala99Pro	US	rs779024959	CHARGE syndrome (CHARGES) [MIM:214800]
CHD7	Q9P2D1	VAR_068378	p.Pro167Leu	LB/B	rs61742851	-
CHD7	Q9P2D1	VAR_068379	p.Val238Leu	LB/B	rs200898742	-
CHD7	Q9P2D1	VAR_068380	p.Gln254Glu	US	rs1554581354	CHARGE syndrome (CHARGES) [MIM:214800]
CHD7	Q9P2D1	VAR_068381	p.Arg286Gly	LB/B	rs61995713	-
CHD7	Q9P2D1	VAR_068382	p.Pro439Ser	US	rs772369092	CHARGE syndrome (CHARGES) [MIM:214800]
CHD7	Q9P2D1	VAR_068383	p.His524Pro	LB/B	rs78962949	-
CHD7	Q9P2D1	VAR_068384	p.Gln596Lys	LB/B	-	-
CHD7	Q9P2D1	VAR_068385	p.Ser699Gly	US	-	CHARGE syndrome (CHARGES) [MIM:214800]
CHD7	Q9P2D1	VAR_068386	p.Lys812Asn	LB/B	rs61978638	-
CHD7	Q9P2D1	VAR_068387	p.Trp840Cys	LP/P	-	CHARGE syndrome (CHARGES) [MIM:214800]
CHD7	Q9P2D1	VAR_068388	p.Thr942Ala	US	rs370194460	CHARGE syndrome (CHARGES) [MIM:214800]
CHD7	Q9P2D1	VAR_068389	p.Gly975Arg	US	-	CHARGE syndrome (CHARGES) [MIM:214800]
CHD7	Q9P2D1	VAR_068390	p.Trp1031Arg	LP/P	-	CHARGE syndrome (CHARGES) [MIM:214800]
CHD7	Q9P2D1	VAR_068391	p.Ile1081Ser	US	-	CHARGE syndrome (CHARGES) [MIM:214800]
CHD7	Q9P2D1	VAR_068392	p.Thr1082Asn	LP/P	-	CHARGE syndrome (CHARGES) [MIM:214800]
CHD7	Q9P2D1	VAR_068393	p.Cys1101Arg	LP/P	-	CHARGE syndrome (CHARGES) [MIM:214800]
CHD7	Q9P2D1	VAR_068394	p.Cys1251Arg	US	-	CHARGE syndrome (CHARGES) [MIM:214800]
CHD7	Q9P2D1	VAR_068395	p.Leu1292Pro	LP/P	-	CHARGE syndrome (CHARGES) [MIM:214800]
CHD7	Q9P2D1	VAR_068396	p.Arg1317Cys	US	rs373301291	CHARGE syndrome (CHARGES) [MIM:214800]
CHD7	Q9P2D1	VAR_068397	p.Cys1318Arg	LP/P	-	CHARGE syndrome (CHARGES) [MIM:214800]
CHD7	Q9P2D1	VAR_068398	p.Arg1345His	LP/P	-	CHARGE syndrome (CHARGES) [MIM:214800]
CHD7	Q9P2D1	VAR_068399	p.Pro1594Ser	LB/B	-	-
CHD7	Q9P2D1	VAR_068400	p.Gly1617Asp	US	-	CHARGE syndrome (CHARGES) [MIM:214800]
CHD7	Q9P2D1	VAR_068401	p.Gly1619Val	US	-	CHARGE syndrome (CHARGES) [MIM:214800]
CHD7	Q9P2D1	VAR_068402	p.Ala1672Val	LB/B	rs61737194	-
CHD7	Q9P2D1	VAR_068403	p.Gly1797Val	LP/P	-	CHARGE syndrome (CHARGES) [MIM:214800]
CHD7	Q9P2D1	VAR_068404	p.Asp1812Gly	LP/P	-	CHARGE syndrome (CHARGES) [MIM:214800]
CHD7	Q9P2D1	VAR_068405	p.Asp1812His	LP/P	-	CHARGE syndrome (CHARGES) [MIM:214800]
CHD7	Q9P2D1	VAR_068406	p.Ala1972Gly	LB/B	rs1013310877	-
CHD7	Q9P2D1	VAR_068407	p.Arg2062Trp	LB/B	rs886063038	-
CHD7	Q9P2D1	VAR_068408	p.Leu2074Pro	US	-	CHARGE syndrome (CHARGES) [MIM:214800]
CHD7	Q9P2D1	VAR_068408	p.Leu2074Pro	US	-	Hypogonadotropic hypogonadism 5 with or without anosmia (HH5) [MIM:612370]
CHD7	Q9P2D1	VAR_068409	p.Trp2091Arg	LP/P	-	CHARGE syndrome (CHARGES) [MIM:214800]
CHD7	Q9P2D1	VAR_068410	p.Asp2097Gly	US	-	CHARGE syndrome (CHARGES) [MIM:214800]
CHD7	Q9P2D1	VAR_068411	p.Val2102Ile	US	-	CHARGE syndrome (CHARGES) [MIM:214800]
CHD7	Q9P2D1	VAR_068412	p.Thr2112Met	US	rs758409717	-
CHD7	Q9P2D1	VAR_068413	p.Asn2118Asp	LB/B	-	-
CHD7	Q9P2D1	VAR_068414	p.Ala2259Thr	US	rs200806228	CHARGE syndrome (CHARGES) [MIM:214800]
CHD7	Q9P2D1	VAR_068414	p.Ala2259Thr	US	rs200806228	Hypogonadotropic hypogonadism 5 with or without anosmia (HH5) [MIM:612370]

CHD7 Q9P2D1 VAR_068415 p.Gly2286Ala LP/P - CHARGE syndrome (CHARGES) [MIM:214800]
CHD7 Q9P2D1 VAR_068416 p.Lys2312Thr US - CHARGE syndrome (CHARGES) [MIM:214800]
CHD7 Q9P2D1 VAR_068417 p.Leu2366Arg US rs541818422 CHARGE syndrome (CHARGES) [MIM:214800]
CHD7 Q9P2D1 VAR_068418 p.Ala2415Ser LB/B rs41315633 -
CHD7 Q9P2D1 VAR_068419 p.Lys2464Glu US - CHARGE syndrome (CHARGES) [MIM:214800]
CHD7 Q9P2D1 VAR_068420 p.Gly2488Asp LB/B rs398124324 -
CHD7 Q9P2D1 VAR_068421 p.Arg2491Cys LB/B rs755492299 -
CHD7 Q9P2D1 VAR_068422 p.Ile2725Val LB/B rs113877656 -
CHD7 Q9P2D1 VAR_068423 p.Ala2780Val LB/B rs775132352 -
CHD7 Q9P2D1 VAR_069032 p.Leu511Val LB/B - -
CHD7 Q9P2D1 VAR_069033 p.Ser527Ala LB/B - -
CHD7 Q9P2D1 VAR_069034 p.Leu1745Pro US - CHARGE syndrome (CHARGES) [MIM:214800]
CHD7 Q9P2D1 VAR_069035 p.Arg2077Lys US - -
CHD7 Q9P2D1 VAR_072954 p.Gly117Asp LB/B rs372110761 -
CHD7 Q9P2D1 VAR_072956 p.Arg758His LP/P rs202208393 Hypogonadotropic hypogonadism 5 with or without anosmia (HH5) [MIM:612370]
CHD7 Q9P2D1 VAR_072957 p.Arg886Trp LP/P rs772260091 Hypogonadotropic hypogonadism 5 with or without anosmia (HH5) [MIM:612370]
CHD7 Q9P2D1 VAR_072958 p.Arg944Ser LP/P rs587783435 Hypogonadotropic hypogonadism 5 with or without anosmia (HH5) [MIM:612370]
CHD7 Q9P2D1 VAR_072959 p.Asn1030Ser LP/P rs886041167 Hypogonadotropic hypogonadism 5 with or without anosmia (HH5) [MIM:612370]
CHD7 Q9P2D1 VAR_072960 p.Lys1291Glu LP/P - Hypogonadotropic hypogonadism 5 with or without anosmia (HH5) [MIM:612370]
CHD7 Q9P2D1 VAR_072961 p.Leu1302Pro LP/P rs1563643394 CHARGE syndrome (CHARGES) [MIM:214800]
CHD7 Q9P2D1 VAR_072962 p.Leu1375Phe LP/P - Hypogonadotropic hypogonadism 5 with or without anosmia (HH5) [MIM:612370]
CHD7 Q9P2D1 VAR_072963 p.Arg1592Trp US rs773187713 CHARGE syndrome (CHARGES) [MIM:214800]
CHD7 Q9P2D1 VAR_072964 p.Val1742Asp LP/P - CHARGE syndrome (CHARGES) [MIM:214800]
CHD7 Q9P2D1 VAR_072965 p.Met1838Val LP/P rs759918327 Hypogonadotropic hypogonadism 5 with or without anosmia (HH5) [MIM:612370]
CHD7 Q9P2D1 VAR_072966 p.Arg1912Gly LP/P - Hypogonadotropic hypogonadism 5 with or without anosmia (HH5) [MIM:612370]
CHD7 Q9P2D1 VAR_072967 p.Arg2065Cys LP/P - Hypogonadotropic hypogonadism 5 with or without anosmia (HH5) [MIM:612370]
CHD7 Q9P2D1 VAR_072968 p.Arg2398Gly LP/P - Hypogonadotropic hypogonadism 5 with or without anosmia (HH5) [MIM:612370]
CHD7 Q9P2D1 VAR_072969 p.Gln2833Pro LP/P - Hypogonadotropic hypogonadism 5 with or without anosmia (HH5) [MIM:612370]
CHD7 Q9P2D1 VAR_078703 p.Gly2108Trp LP/P - CHARGE syndrome (CHARGES) [MIM:214800]
CHD8 Q9HCK8 VAR_078704 p.Val744Ile US rs996150988 Autism 18 (AUTS18) [MIM:615032]
CHD9 Q3L8U1 VAR_047355 p.Asp2312Glu LB/B rs6499548 -
CHDH Q8NE62 VAR_020421 p.Glu40Ala LB/B rs9001 -
CHDH Q8NE62 VAR_049357 p.Asn441Ser LB/B rs34974961 -
CHDH Q8NE62 VAR_055097 p.Leu78Arg LB/B rs12676 -
CHEK1 O14757 VAR_021123 p.Arg156Gln LB/B rs3731410 -
CHEK1 O14757 VAR_024571 p.Ile471Val LB/B rs506504 -
CHEK1 O14757 VAR_040407 p.Glu223Val LB/B rs35817404 -
CHEK1 O14757 VAR_040408 p.Val312Met LB/B rs34097480 -

CHEK2	O96017	VAR_008554	p.Arg145Trp	US	rs137853007	Colon cancer
CHEK2	O96017	VAR_008554	p.Arg145Trp	LP/P	rs137853007	Li-Fraumeni syndrome 2 (LFS2) [MIM:609265]
CHEK2	O96017	VAR_008555	p.Ile157Thr	LB/B	rs17879961	-
CHEK2	O96017	VAR_019101	p.Ala17Ser	US	rs137853008	An osteogenic sarcoma sample
CHEK2	O96017	VAR_019102	p.Pro85Leu	US	rs17883862	An osteogenic sarcoma sample
CHEK2	O96017	VAR_019103	p.Arg180Cys	LP/P	rs77130927	Prostate cancer (PC) [MIM:176807]
CHEK2	O96017	VAR_019104	p.Arg181Cys	LP/P	rs137853010	Prostate cancer (PC) [MIM:176807]
CHEK2	O96017	VAR_019105	p.Arg181His	LP/P	rs121908701	Prostate cancer (PC) [MIM:176807]
CHEK2	O96017	VAR_019106	p.Glu239Lys	LP/P	rs121908702	Prostate cancer (PC) [MIM:176807]
CHEK2	O96017	VAR_019107	p.Glu64Lys	LP/P	rs141568342	Prostate cancer (PC) [MIM:176807]
CHEK2	O96017	VAR_019108	p.Arg145Pro	LP/P	rs587781667	Prostate cancer (PC) [MIM:176807]
CHEK2	O96017	VAR_019109	p.Gly167Arg	LP/P	rs72552322	Prostate cancer (PC) [MIM:176807]
CHEK2	O96017	VAR_019110	p.Arg180His	LP/P	rs137853009	Prostate cancer (PC) [MIM:176807]
CHEK2	O96017	VAR_019111	p.Ile251Phe	US	rs587780189	Prostate cancer (PC) [MIM:176807]
CHEK2	O96017	VAR_019112	p.Arg318His	US	rs143611747	Prostate cancer (PC) [MIM:176807]
CHEK2	O96017	VAR_019113	p.Thr323Pro	LP/P	rs750984976	Prostate cancer (PC) [MIM:176807]
CHEK2	O96017	VAR_019114	p.Tyr327Cys	US	rs587780194	Prostate cancer (PC) [MIM:176807]
CHEK2	O96017	VAR_019115	p.Thr476Lys	LP/P	-	Prostate cancer (PC) [MIM:176807]
CHEK2	O96017	VAR_021117	p.Leu436Met	LB/B	rs17882922	-
CHEK2	O96017	VAR_021118	p.Asn446Lys	LB/B	rs17880867	-
CHEK2	O96017	VAR_021119	p.Phe447Ile	LB/B	rs17881473	-
CHEK2	O96017	VAR_021120	p.Ile448Ser	LB/B	rs17886163	-
CHEK2	O96017	VAR_021121	p.Glu501Lys	LB/B	rs17883172	-
CHEK2	O96017	VAR_021122	p.Leu512Val	LB/B	rs17882942	-
CHEK2	O96017	VAR_022461	p.Arg117Gly	LP/P	rs28909982	Breast cancer (BC) [MIM:114480]
CHEK2	O96017	VAR_022462	p.Arg137Gln	LB/B	rs368570187	-
CHEK2	O96017	VAR_022463	p.Ser428Phe	LB/B	rs137853011	-
CHEK2	O96017	VAR_024572	p.Arg406His	LB/B	rs200649225	-
CHEK2	O96017	VAR_026630	p.Thr59Lys	US	rs149991239	Multiple cancers
CHEK2	O96017	VAR_029154	p.Asp347Asn	LB/B	rs28909980	-
CHEK2	O96017	VAR_029155	p.Ser500Cys	LB/B	rs28909981	-
CHEK2	O96017	VAR_066012	p.His371Tyr	LB/B	rs531398630	-
CHEK2	O96017	VAR_073020	p.Tyr390Cys	LP/P	rs200928781	Breast cancer (BC) [MIM:114480]
CHERP	Q8IWX8	VAR_034833	p.Asn199His	LB/B	rs1043448	-
CHFR	Q96EP1	VAR_017582	p.Pro166Leu	US	rs1176037831	A patient with non small cell lung carcinomas
CHFR	Q96EP1	VAR_017583	p.Arg202Pro	US	-	A patient with non small cell lung carcinomas
CHFR	Q96EP1	VAR_017584	p.Gly270Arg	LB/B	rs115096950	-
CHFR	Q96EP1	VAR_017585	p.Ala497Val	LB/B	rs2306541	-
CHFR	Q96EP1	VAR_017586	p.Phe536Ser	US	-	A patient with non small cell lung carcinomas
CHFR	Q96EP1	VAR_017587	p.Val580Met	LB/B	rs2306536	-
CHGA	P10645	VAR_025636	p.Glu176Lys	LB/B	rs9658654	-
CHGA	P10645	VAR_025637	p.Glu264Asp	LB/B	rs9658655	-
CHGA	P10645	VAR_025638	p.Arg271Trp	LB/B	rs9658662	-
CHGA	P10645	VAR_025639	p.Ala274Gly	LB/B	rs9658663	-
CHGA	P10645	VAR_025640	p.Gly315Ser	LB/B	rs9658664	-
CHGA	P10645	VAR_025641	p.Leu332Pro	LB/B	rs9658665	-
CHGA	P10645	VAR_025642	p.Asp369Asn	LB/B	rs2228575	-
CHGA	P10645	VAR_025643	p.Gly382Ser	LB/B	rs9658667	-
CHGA	P10645	VAR_025644	p.Pro388Leu	LB/B	rs9658668	-

CHGA	P10645	VAR_025645	p.Arg399Trp	LB/B	rs729940	-
CHGA	P10645	VAR_047417	p.Arg61Gln	LB/B	rs3742712	-
CHGA	P10645	VAR_072687	p.Arg392Gln	LB/B	rs9658669	-
CHGB	P05060	VAR_020287	p.Arg178Gln	LB/B	rs910122	-
CHGB	P05060	VAR_022012	p.Arg417His	LB/B	rs742711	-
CHGB	P05060	VAR_024414	p.Lys117Asn	LB/B	rs236150	-
CHGB	P05060	VAR_024415	p.Thr243Ala	LB/B	rs236151	-
CHGB	P05060	VAR_024416	p.Ala353Gly	LB/B	rs236152	-
CHGB	P05060	VAR_028235	p.Asp145Asn	LB/B	rs6133278	-
CHGB	P05060	VAR_028236	p.Asn200His	LB/B	rs881118	-
CHGB	P05060	VAR_028237	p.Arg232Gln	LB/B	rs6139873	-
CHGB	P05060	VAR_028238	p.Pro413Leu	LB/B	rs742710	-
CHGB	P05060	VAR_043578	p.Ser93Thr	LB/B	rs6085324	-
CHI3L1	P36222	VAR_019838	p.Arg145Gly	LB/B	rs880633	-
CHI3L1	P36222	VAR_019839	p.Ile311Thr	LB/B	rs1049407	-
CHI3L2	Q15782	VAR_033731	p.Val184Ile	LB/B	rs34049547	-
CHI3L2	Q15782	VAR_049198	p.Ala182Val	LB/B	rs11556868	-
CHI3L2	Q15782	VAR_061189	p.Arg318Trp	LB/B	rs13721	-
CHIA	Q9BZP6	VAR_033730	p.Val324Gly	LB/B	rs2256721	-
CHIA	Q9BZP6	VAR_049192	p.Gly102Arg	LB/B	rs3818822	-
CHIA	Q9BZP6	VAR_049193	p.Ile339Val	LB/B	rs2275253	-
CHIA	Q9BZP6	VAR_049194	p.Phe354Ser	LB/B	rs2275254	-
CHIA	Q9BZP6	VAR_049195	p.Phe377Leu	LB/B	rs36011905	-
CHIA	Q9BZP6	VAR_049196	p.Val432Gly	LB/B	rs2256721	-
CHIA	Q9BZP6	VAR_063030	p.Asn45Asp	LB/B	rs41282492	-
CHIA	Q9BZP6	VAR_063031	p.Asp47Asn	LB/B	rs41282494	-
CHIA	Q9BZP6	VAR_063032	p.Arg61Met	LB/B	rs41282496	-
CHIA	Q9BZP6	VAR_063033	p.Lys125Arg	LB/B	rs61756687	-
CHID1	Q9BWS9	VAR_031173	p.Arg331Gln	LB/B	rs1127800	-
CHID1	Q9BWS9	VAR_031174	p.Ala338Val	LB/B	rs6682	-
CHIT1	Q13231	VAR_022138	p.Gly102Ser	LB/B	rs2297950	-
CHIT1	Q13231	VAR_024458	p.Ala442Gly	LB/B	rs1065761	-
CHIT1	Q13231	VAR_049190	p.Arg40His	LB/B	rs35920428	-
CHIT1	Q13231	VAR_049191	p.Gln171His	LB/B	rs12562058	-
CHIT1	Q13231	VAR_065914	p.Glu74Lys	LB/B	rs137852607	-
CHKA	P35790	VAR_054863	p.Ser220Gly	LB/B	rs17853641	-
CHKA	P35790	VAR_054864	p.Leu422Gln	LB/B	rs17853642	-
CHKB	Q9Y259	VAR_081796	p.Glu283Lys	LP/P	-	Muscular dystrophy, congenital, megaconial type (MDCMC) [MIM:602541]
CHKB	Q9Y259	VAR_081798	p.Thr301Ile	LB/B	rs147485527	-
CHKB	Q9Y259	VAR_081800	p.Gln328Arg	LB/B	rs141381896	-
CHKB	Q9Y259	VAR_081801	p.Arg377Leu	LP/P	rs772705206	Muscular dystrophy, congenital, megaconial type (MDCMC) [MIM:602541]
CHL1	O00533	VAR_027167	p.Leu17Phe	LB/B	rs2272522	-
CHL1	O00533	VAR_027168	p.Thr287Ala	LB/B	rs13060847	-
CHL1	O00533	VAR_027169	p.Ile1034Val	LB/B	rs6442827	-
CHL1	O00533	VAR_035505	p.Leu411Ile	US	-	A colorectal cancer sample
CHM	P24386	VAR_008273	p.Gln471Leu	LP/P	-	Choroideremia (CHM) [MIM:303100]
CHM	P24386	VAR_066847	p.His507Arg	LP/P	rs397514603	Choroideremia (CHM) [MIM:303100]

CHM P24386 VAR_066848 p.Leu550Pro LP/P - Choroideremia (CHM) [MIM:303100]
CHMP2B Q9UQN3 VAR_023383 p.Asp148Tyr LP/P rs63750653 Frontotemporal dementia and/or amyotrophic lateral sclerosis 7 (FTDALS7) [MIM:600795]
CHMP2B Q9UQN3 VAR_038373 p.Ile29Val LP/P rs63750818 Frontotemporal dementia and/or amyotrophic lateral sclerosis 7 (FTDALS7) [MIM:600795]
CHMP2B Q9UQN3 VAR_038374 p.Gln206His LP/P rs63751126 Frontotemporal dementia and/or amyotrophic lateral sclerosis 7 (FTDALS7) [MIM:600795]
CHMP2B Q9UQN3 VAR_068689 p.Thr104Asn LP/P rs281864934 Frontotemporal dementia and/or amyotrophic lateral sclerosis 7 (FTDALS7) [MIM:600795]
CHMP4A Q9BY43 VAR_023384 p.Gly153Arg LB/B rs2295322 -
CHMP4B Q9H444 VAR_037579 p.Asp129Val LP/P rs118203965 Cataract 31, multiple types (CTRCT31) [MIM:605387]
CHMP4B Q9H444 VAR_037580 p.Glu161Lys LP/P rs118203966 Cataract 31, multiple types (CTRCT31) [MIM:605387]
CHMP4C Q96CF2 VAR_052028 p.Ala232Thr LB/B rs35094336 -
CHMP6 Q96FZ7 VAR_061807 p.Gly55Ser LB/B rs61037507 -
CHN1 P15882 VAR_047940 p.Leu20Phe LP/P rs121912792 Duane retraction syndrome 2 (DURS2) [MIM:604356]
CHN1 P15882 VAR_047941 p.Ile126Met LP/P rs121912793 Duane retraction syndrome 2 (DURS2) [MIM:604356]
CHN1 P15882 VAR_047942 p.Tyr143His LP/P rs121912794 Duane retraction syndrome 2 (DURS2) [MIM:604356]
CHN1 P15882 VAR_047943 p.Ala223Val LP/P rs121912795 Duane retraction syndrome 2 (DURS2) [MIM:604356]
CHN1 P15882 VAR_047944 p.Gly228Ser LP/P rs121912796 Duane retraction syndrome 2 (DURS2) [MIM:604356]
CHN1 P15882 VAR_047945 p.Pro252Gln LP/P rs121912797 Duane retraction syndrome 2 (DURS2) [MIM:604356]
CHN1 P15882 VAR_047946 p.Glu313Lys LP/P rs121912798 Duane retraction syndrome 2 (DURS2) [MIM:604356]
CHN2 P52757 VAR_022118 p.His204Arg LB/B rs3750103 -
CHN2 P52757 VAR_049136 p.Pro438Ser LB/B rs34971642 -
CHORDC1 Q9UHD1 VAR_038676 p.Ala329Asp LB/B rs1045861 -
CHP2 O43745 VAR_048664 p.Arg127Pro LB/B rs35641939 -
CHPF Q8IZ52 VAR_047394 p.Gln371Arg LB/B rs6436155 -
CHPT1 Q8WUD6 VAR_032612 p.Phe162Ser LB/B rs3205421 -
CHPT1 Q8WUD6 VAR_032613 p.Tyr323Ser US - -
CHRAC1 Q9NRG0 VAR_013755 p.Cys55Tyr LB/B rs2231522 -
CHRAC1 Q9NRG0 VAR_013756 p.His126Arg LB/B rs2231524 -
CHRD Q9H2X0 VAR_021517 p.Met630Leu LB/B rs16858780 -
CHRD Q9H2X0 VAR_048727 p.Pro94Ser LB/B rs34095724 -
CHRDL1 Q9BU40 VAR_068175 p.Cys260Phe LP/P rs387906713 Megalocornea 1, X-linked (MGC1) [MIM:309300]
CHRDL2 Q6WN34 VAR_055651 p.Pro335Leu LB/B rs35903991 -
CHRM3 P20309 VAR_033461 p.Val65Ile LB/B rs2067481 -
CHRM3 P20309 VAR_049368 p.Leu431Pro LB/B rs16839102 -
CHRNA1 P02708 VAR_000282 p.Gly198Ser LP/P rs137852801 Myasthenic syndrome, congenital, 1A, slow-channel (CMS1A) [MIM:601462]

CHRNA1 P02708 VAR_000283 p.Val201Met LP/P rs137852799 Myasthenic syndrome, congenital, 1A, slow-channel (CMS1A) [MIM:601462]
CHRNA1 P02708 VAR_000284 p.Asn262Lys LP/P rs137852798 Myasthenic syndrome, congenital, 1A, slow-channel (CMS1A) [MIM:601462]
CHRNA1 P02708 VAR_000285 p.Thr299Ile LP/P rs137852800 Myasthenic syndrome, congenital, 1A, slow-channel (CMS1A) [MIM:601462]
CHRNA1 P02708 VAR_000286 p.Ser314Ile LP/P rs137852802 Myasthenic syndrome, congenital, 1A, slow-channel (CMS1A) [MIM:601462]
CHRNA1 P02708 VAR_021206 p.Phe278Val LP/P rs137852805 Myasthenic syndrome, congenital, 1B, fast-channel (CMS1B) [MIM:608930]
CHRNA1 P02708 VAR_021207 p.Val294Phe LP/P rs137852803 Myasthenic syndrome, congenital, 1A, slow-channel (CMS1A) [MIM:601462]
CHRNA1 P02708 VAR_021208 p.Phe301Leu LP/P rs137852806 Myasthenic syndrome, congenital, 1B, fast-channel (CMS1B) [MIM:608930]
CHRNA1 P02708 VAR_021209 p.Val330Ile LP/P rs137852804 Myasthenic syndrome, congenital, 1B, fast-channel (CMS1B) [MIM:608930]
CHRNA1 P02708 VAR_038599 p.Val177Leu LP/P rs137852807 Myasthenic syndrome, congenital, 1B, fast-channel (CMS1B) [MIM:608930]
CHRNA1 P02708 VAR_038600 p.Asp383Val LB/B rs6739001 -
CHRNA1 P02708 VAR_038601 p.Cys463Trp LP/P rs137852808 Myasthenic syndrome, congenital, 1A, slow-channel (CMS1A) [MIM:601462]
CHRNA1 P02708 VAR_043904 p.Arg254Leu LP/P rs137852809 Multiple pterygium syndrome, lethal type (LMPS) [MIM:253290]
CHRNA10 Q9GZZ6 VAR_048172 p.Glu355Ala LB/B rs2231547 -
CHRNA2 Q15822 VAR_027639 p.Thr22Ile LB/B rs2472553 -
CHRNA2 Q15822 VAR_027640 p.Thr125Ala LB/B rs891398 -
CHRNA2 Q15822 VAR_027641 p.Ile279Asn LP/P rs104894063 Epilepsy, nocturnal frontal lobe, 4 (ENFL4) [MIM:610353]
CHRNA2 Q15822 VAR_076498 p.Arg376Trp US rs1018084204 Seizures, benign familial infantile, 6 (BFIS6) [MIM:610353]
CHRNA3 P32297 VAR_059110 p.Arg37His LB/B rs8192475 -
CHRNA4 P43681 VAR_000295 p.Ser280Phe LP/P rs121909580 Epilepsy, nocturnal frontal lobe, 1 (ENFL1) [MIM:600513]
CHRNA4 P43681 VAR_017531 p.Ser280Leu LP/P - Epilepsy, nocturnal frontal lobe, 1 (ENFL1) [MIM:600513]
CHRNA4 P43681 VAR_023402 p.Glu387Gly LB/B rs45604738 -
CHRNA4 P43681 VAR_023403 p.Ser517Leu LB/B rs45622132 -
CHRNA5 P30532 VAR_046211 p.Val134Ile LB/B rs2229961 -
CHRNA5 P30532 VAR_046212 p.Asp398Asn LB/B rs16969968 -
CHRNA6 Q15825 VAR_048171 p.Asn447Ser LB/B rs16891583 -
CHRNA9 Q9UGM1 VAR_025425 p.Asn442Ser LB/B rs10009228 -
CHRNA9 Q9UGM1 VAR_031151 p.Arg96Gln LB/B rs10024518 -
CHRNA9 Q9UGM1 VAR_060996 p.Ala315Val LB/B rs55633891 -
CHRNB1 P11230 VAR_000287 p.Leu285Met LP/P rs137852811 Myasthenic syndrome, congenital, 2A, slow-channel (CMS2A) [MIM:616313]
CHRNB1 P11230 VAR_000288 p.Val289Met LP/P rs137852810 Myasthenic syndrome, congenital, 2A, slow-channel (CMS2A) [MIM:616313]
CHRNB1 P11230 VAR_048169 p.Glu32Gly LB/B rs17856697 -
CHRNB1 P11230 VAR_070842 p.Asp124Tyr LB/B rs17856698 -

CHRNB1 P11230 VAR_077363 p.Val289Ala LP/P - Myasthenic syndrome, congenital, 2A, slow-channel (CMS2A) [MIM:616313]
CHRNB2 P17787 VAR_012714 p.Val287Leu LP/P rs74315291 Epilepsy, nocturnal frontal lobe, 3 (ENFL3) [MIM:605375]
CHRNB2 P17787 VAR_012715 p.Val287Met LP/P rs74315291 Epilepsy, nocturnal frontal lobe, 3 (ENFL3) [MIM:605375]
CHRNB2 P17787 VAR_021564 p.Gln397His LB/B rs55685423 -
CHRNB3 Q05901 VAR_048173 p.Lys451Glu LB/B rs35327613 -
CHRNB4 P30926 VAR_013241 p.Arg136Trp LB/B rs141876090 -
CHRNB4 P30926 VAR_013242 p.Ser140Gly LB/B rs56218866 -
CHRNB4 P30926 VAR_013243 p.Met467Val LB/B rs61737502 -
CHRNB4 P30926 VAR_048174 p.Thr91Ile LB/B rs12914008 -
CHRND Q07001 VAR_019566 p.Ser289Phe LP/P rs121909502 Myasthenic syndrome, congenital, 3A, slow-channel (CMS3A) [MIM:616321]
CHRND Q07001 VAR_021210 p.Glu80Lys LP/P rs121909504 Myasthenic syndrome, congenital, 3B, fast-channel (CMS3B) [MIM:616322]
CHRND Q07001 VAR_021211 p.Pro271Gln LP/P rs121909503 Myasthenic syndrome, congenital, 3B, fast-channel (CMS3B) [MIM:616322]
CHRND Q07001 VAR_021212 p.Gln288Glu LB/B rs41265127 -
CHRND Q07001 VAR_036031 p.Asp398Glu US - A breast cancer sample
CHRND Q07001 VAR_043905 p.Phe95Leu LP/P rs121909506 Multiple pterygium syndrome, lethal type (LMPS) [MIM:253290]
CHRND Q07001 VAR_073691 p.Leu42Pro LP/P - Myasthenic syndrome, congenital, 3B, fast-channel (CMS3B) [MIM:616322]
CHRND Q07001 VAR_073692 p.Ile79Lys LP/P rs121909509 Myasthenic syndrome, congenital, 3B, fast-channel (CMS3B) [MIM:616322]
CHRND Q07001 VAR_073693 p.Val114Leu LB/B rs760395222 -
CHRND Q07001 VAR_073694 p.Glu402Lys LP/P rs145955590 Myasthenic syndrome, congenital, 3C, associated with acetylcholine receptor deficiency (CMS3C) [MIM:616323]
CHRNE Q04844 VAR_000289 p.Pro141Leu LP/P rs121909512 Myasthenic syndrome, congenital, 4B, fast-channel (CMS4B) [MIM:616324]
CHRNE Q04844 VAR_000290 p.Arg167Leu LP/P rs121909514 Myasthenic syndrome, congenital, 4C, associated with acetylcholine receptor deficiency (CMS4C) [MIM:608931]
CHRNE Q04844 VAR_000291 p.Pro265Leu LP/P rs759226183 Myasthenic syndrome, congenital, 4C, associated with acetylcholine receptor deficiency (CMS4C) [MIM:608931]
CHRNE Q04844 VAR_000292 p.Thr284Pro LP/P rs121909510 Myasthenic syndrome, congenital, 4A, slow-channel (CMS4A) [MIM:605809]
CHRNE Q04844 VAR_000293 p.Leu289Phe LP/P rs121909511 Myasthenic syndrome, congenital, 4A, slow-channel (CMS4A) [MIM:605809]
CHRNE Q04844 VAR_000294 p.Arg331Trp LP/P rs121909515 Myasthenic syndrome, congenital, 4C, associated with acetylcholine receptor deficiency (CMS4C) [MIM:608931]
CHRNE Q04844 VAR_019567 p.Leu98Pro LP/P rs28929768 Myasthenic syndrome, congenital, 4A, slow-channel (CMS4A) [MIM:605809]
CHRNE Q04844 VAR_019568 p.Leu241Phe LP/P rs28999110 Myasthenic syndrome, congenital, 4A, slow-channel (CMS4A) [MIM:605809]
CHRNE Q04844 VAR_021213 p.Gly13Arg LP/P rs372635387 Myasthenic syndrome, congenital, 4B, fast-channel (CMS4B) [MIM:616324]
CHRNE Q04844 VAR_021214 p.Ser163Leu LP/P rs121909516 Myasthenic syndrome, congenital, 4B, fast-channel (CMS4B) [MIM:616324]

CHRNE Q04844 VAR_021215 p.Ala431Pro LP/P rs121909517 Myasthenic syndrome, congenital, 4B, fast-channel (CMS4B) [MIM:616324]
CHRNE Q04844 VAR_048170 p.Gly18Val LB/B rs4790235 -
CHRNE Q04844 VAR_071629 p.Trp75Arg LP/P rs193919341 Myasthenic syndrome, congenital, 4B, fast-channel (CMS4B) [MIM:616324]
CHRNE Q04844 VAR_077364 p.Val285Ala LP/P - Myasthenic syndrome, congenital, 4A, slow-channel (CMS4A) [MIM:605809]
CHRNG P07510 VAR_030753 p.Val107Gly LP/P rs267606726 Multiple pterygium syndrome, Escobar variant (EVMPS) [MIM:265000]
CHRNG P07510 VAR_030753 p.Val107Gly LP/P rs267606726 Multiple pterygium syndrome, lethal type (LMPS) [MIM:253290]
CHRNG P07510 VAR_030754 p.Ala149Thr LB/B rs2289080 -
CHRNG P07510 VAR_030755 p.Arg239Cys LP/P rs121912670 Multiple pterygium syndrome, Escobar variant (EVMPS) [MIM:265000]
CHRNG P07510 VAR_030755 p.Arg239Cys LP/P rs121912670 Multiple pterygium syndrome, lethal type (LMPS) [MIM:253290]
CHST10 O43529 VAR_021470 p.Asp258Asn LB/B rs3748932 -
CHST10 O43529 VAR_033737 p.Val20Leu LB/B rs35177621 -
CHST12 Q9NRB3 VAR_021471 p.Pro52His LB/B rs3735099 -
CHST12 Q9NRB3 VAR_021472 p.Thr61Ser LB/B rs3735100 -
CHST12 Q9NRB3 VAR_021473 p.Arg109Ser LB/B rs17132395 -
CHST12 Q9NRB3 VAR_021474 p.Ala145Pro LB/B rs17132399 -
CHST12 Q9NRB3 VAR_033738 p.Pro94Leu LB/B rs12536223 -
CHST13 Q8NET6 VAR_021477 p.Ala271Val LB/B rs1056523 -
CHST13 Q8NET6 VAR_021478 p.Arg317Gln LB/B rs1056522 -
CHST13 Q8NET6 VAR_053698 p.Pro146Ser LB/B rs34311016 -
CHST14 Q8NCH0 VAR_063754 p.Arg135Gly LP/P rs267606727 Ehlers-Danlos syndrome, musculocontractural type 1 (EDSMC1) [MIM:601776]
CHST14 Q8NCH0 VAR_063755 p.Leu137Gln LP/P rs267606728 Ehlers-Danlos syndrome, musculocontractural type 1 (EDSMC1) [MIM:601776]
CHST14 Q8NCH0 VAR_063756 p.Arg213Pro LP/P rs121908257 Ehlers-Danlos syndrome, musculocontractural type 1 (EDSMC1) [MIM:601776]
CHST14 Q8NCH0 VAR_063757 p.Tyr293Cys LP/P rs121908258 Ehlers-Danlos syndrome, musculocontractural type 1 (EDSMC1) [MIM:601776]
CHST14 Q8NCH0 VAR_064555 p.Pro281Leu LP/P rs267606729 Ehlers-Danlos syndrome, musculocontractural type 1 (EDSMC1) [MIM:601776]
CHST14 Q8NCH0 VAR_064556 p.Cys289Ser LP/P rs267606731 Ehlers-Danlos syndrome, musculocontractural type 1 (EDSMC1) [MIM:601776]
CHST3 Q7LGC8 VAR_021413 p.Arg304Gln LP/P rs28937593 Spondyloepiphyseal dysplasia with congenital joint dislocations (SEDCJD) [MIM:143095]
CHST3 Q7LGC8 VAR_021414 p.Ile348Met LB/B rs3740128 -
CHST3 Q7LGC8 VAR_021415 p.Arg357Gln LB/B rs3740129 -
CHST3 Q7LGC8 VAR_047856 p.Arg222Trp LP/P rs121908617 Spondyloepiphyseal dysplasia with congenital joint dislocations (SEDCJD) [MIM:143095]
CHST3 Q7LGC8 VAR_047857 p.Leu259Pro LP/P rs121908616 Spondyloepiphyseal dysplasia with congenital joint dislocations (SEDCJD) [MIM:143095]
CHST3 Q7LGC8 VAR_047858 p.Leu307Pro LP/P rs121908618 Spondyloepiphyseal dysplasia with congenital joint dislocations (SEDCJD) [MIM:143095]

CHST3 Q7LGC8 VAR_047859 p.Glu372Lys LP/P rs267606734 Spondyloepiphyseal dysplasia with congenital joint dislocations (SEDCJD) [MIM:143095]
CHST4 Q8NCG5 VAR_052528 p.His361Gln LB/B rs3813744 -
CHST5 Q9GZS9 VAR_021416 p.Thr318Met LB/B rs3826107 -
CHST5 Q9GZS9 VAR_057993 p.Ala311Thr LB/B rs7206332 -
CHST6 Q9GZX3 VAR_021417 p.Leu15Pro LP/P - Macular dystrophy, corneal (MCD) [MIM:217800]
CHST6 Q9GZX3 VAR_021418 p.Leu22Arg LP/P rs68043642 Macular dystrophy, corneal (MCD) [MIM:217800]
CHST6 Q9GZX3 VAR_021419 p.Pro31Ser LP/P rs72547549 Macular dystrophy, corneal (MCD) [MIM:217800]
CHST6 Q9GZX3 VAR_021420 p.His42Tyr LP/P - Macular dystrophy, corneal (MCD) [MIM:217800]
CHST6 Q9GZX3 VAR_021421 p.Arg50Cys LP/P rs28937877 Macular dystrophy, corneal (MCD) [MIM:217800]
CHST6 Q9GZX3 VAR_021422 p.Ser51Leu LP/P rs370335460 Macular dystrophy, corneal (MCD) [MIM:217800]
CHST6 Q9GZX3 VAR_021423 p.Gly52Asp LP/P - Macular dystrophy, corneal (MCD) [MIM:217800]
CHST6 Q9GZX3 VAR_021424 p.Ser53Leu LP/P - Macular dystrophy, corneal (MCD) [MIM:217800]
CHST6 Q9GZX3 VAR_021425 p.Leu59Pro LP/P - Macular dystrophy, corneal (MCD) [MIM:217800]
CHST6 Q9GZX3 VAR_021426 p.Asn61Thr LP/P rs72547548 Macular dystrophy, corneal (MCD) [MIM:217800]
CHST6 Q9GZX3 VAR_021427 p.Val66Leu LP/P rs72547547 Macular dystrophy, corneal (MCD) [MIM:217800]
CHST6 Q9GZX3 VAR_021428 p.Tyr68His LP/P rs775742450 Macular dystrophy, corneal (MCD) [MIM:217800]
CHST6 Q9GZX3 VAR_021429 p.Met70Leu LP/P - Macular dystrophy, corneal (MCD) [MIM:217800]
CHST6 Q9GZX3 VAR_021430 p.Pro72Ser LP/P rs377617168 Macular dystrophy, corneal (MCD) [MIM:217800]
CHST6 Q9GZX3 VAR_021431 p.Val76Met LP/P - Macular dystrophy, corneal (MCD) [MIM:217800]
CHST6 Q9GZX3 VAR_021432 p.Arg93His LP/P - Macular dystrophy, corneal (MCD) [MIM:217800]
CHST6 Q9GZX3 VAR_021433 p.Arg97Pro LP/P rs72547546 Macular dystrophy, corneal (MCD) [MIM:217800]
CHST6 Q9GZX3 VAR_021434 p.Ser98Trp LP/P - Macular dystrophy, corneal (MCD) [MIM:217800]
CHST6 Q9GZX3 VAR_021435 p.Cys102Gly LP/P rs121917822 Macular dystrophy, corneal (MCD) [MIM:217800]
CHST6 Q9GZX3 VAR_021436 p.Cys102Tyr LP/P - Macular dystrophy, corneal (MCD) [MIM:217800]
CHST6 Q9GZX3 VAR_021437 p.Met104Val LP/P rs1158093021 Macular dystrophy, corneal (MCD) [MIM:217800]
CHST6 Q9GZX3 VAR_021438 p.Phe107Ser LP/P rs72547545 Macular dystrophy, corneal (MCD) [MIM:217800]
CHST6 Q9GZX3 VAR_021439 p.Tyr110Cys LP/P rs72547544 Macular dystrophy, corneal (MCD) [MIM:217800]
CHST6 Q9GZX3 VAR_021440 p.Phe121Leu LP/P rs1265310255 Macular dystrophy, corneal (MCD) [MIM:217800]
CHST6 Q9GZX3 VAR_021441 p.Gln122Pro LP/P rs758105699 Macular dystrophy, corneal (MCD) [MIM:217800]
CHST6 Q9GZX3 VAR_021442 p.Arg127Cys LP/P - Macular dystrophy, corneal (MCD) [MIM:217800]
CHST6 Q9GZX3 VAR_021443 p.Ala128Val LP/P rs72547543 Macular dystrophy, corneal (MCD) [MIM:217800]
CHST6 Q9GZX3 VAR_021444 p.Ser131Pro LP/P - Macular dystrophy, corneal (MCD) [MIM:217800]
CHST6 Q9GZX3 VAR_021445 p.Leu152Pro LP/P rs142954809 Macular dystrophy, corneal (MCD) [MIM:217800]
CHST6 Q9GZX3 VAR_021446 p.Arg162Gly LP/P rs117435647 Macular dystrophy, corneal (MCD) [MIM:217800]
CHST6 Q9GZX3 VAR_021447 p.Arg166Pro LP/P rs72547542 Macular dystrophy, corneal (MCD) [MIM:217800]
CHST6 Q9GZX3 VAR_021448 p.Lys174Arg LP/P rs28937877 Macular dystrophy, corneal (MCD) [MIM:217800]
CHST6 Q9GZX3 VAR_021449 p.Arg177His LP/P - Macular dystrophy, corneal (MCD) [MIM:217800]
CHST6 Q9GZX3 VAR_021450 p.Val198Glu LP/P - Macular dystrophy, corneal (MCD) [MIM:217800]
CHST6 Q9GZX3 VAR_021451 p.Leu200Arg LP/P rs28937879 Macular dystrophy, corneal (MCD) [MIM:217800]
CHST6 Q9GZX3 VAR_021452 p.Arg202Ser LP/P - Macular dystrophy, corneal (MCD) [MIM:217800]
CHST6 Q9GZX3 VAR_021453 p.Asp203Glu LP/P rs28937878 Macular dystrophy, corneal (MCD) [MIM:217800]
CHST6 Q9GZX3 VAR_021454 p.Pro204Gln LP/P rs759870075 Macular dystrophy, corneal (MCD) [MIM:217800]
CHST6 Q9GZX3 VAR_021455 p.Arg205Leu LP/P - Macular dystrophy, corneal (MCD) [MIM:217800]

CHST6 Q9GZX3 VAR_021456 p.Arg205Gln LP/P rs377706989 Macular dystrophy, corneal (MCD) [MIM:217800]
CHST6 Q9GZX3 VAR_021457 p.Ala206Thr LP/P rs374493344 Macular dystrophy, corneal (MCD) [MIM:217800]
CHST6 Q9GZX3 VAR_021458 p.Ala206Val LP/P - Macular dystrophy, corneal (MCD) [MIM:217800]
CHST6 Q9GZX3 VAR_021459 p.Ser210Phe LP/P rs745571211 Macular dystrophy, corneal (MCD) [MIM:217800]
CHST6 Q9GZX3 VAR_021460 p.Arg211Gln LP/P rs771397083 Macular dystrophy, corneal (MCD) [MIM:217800]
CHST6 Q9GZX3 VAR_021461 p.Arg211Trp LP/P rs202175444 Macular dystrophy, corneal (MCD) [MIM:217800]
CHST6 Q9GZX3 VAR_021462 p.Ala217Thr LP/P rs752785520 Macular dystrophy, corneal (MCD) [MIM:217800]
CHST6 Q9GZX3 VAR_021463 p.Asp221Glu LP/P - Macular dystrophy, corneal (MCD) [MIM:217800]
CHST6 Q9GZX3 VAR_021464 p.Asp221Tyr LP/P - Macular dystrophy, corneal (MCD) [MIM:217800]
CHST6 Q9GZX3 VAR_021465 p.His249Pro LP/P rs72547540 Macular dystrophy, corneal (MCD) [MIM:217800]
CHST6 Q9GZX3 VAR_021466 p.Tyr268Cys LP/P rs72547539 Macular dystrophy, corneal (MCD) [MIM:217800]
CHST6 Q9GZX3 VAR_021467 p.Glu274Lys LP/P rs72547538 Macular dystrophy, corneal (MCD) [MIM:217800]
CHST6 Q9GZX3 VAR_021468 p.Leu276Pro LP/P rs121917824 Macular dystrophy, corneal (MCD) [MIM:217800]
CHST6 Q9GZX3 VAR_021469 p.Tyr358Asp LP/P - Macular dystrophy, corneal (MCD) [MIM:217800]
CHST6 Q9GZX3 VAR_033735 p.Asn369Asp LB/B rs35036798 -
CHST6 Q9GZX3 VAR_075522 p.Ser118Phe US - Macular dystrophy, corneal (MCD) [MIM:217800]
CHST6 Q9GZX3 VAR_075523 p.Arg177Gly LP/P - Macular dystrophy, corneal (MCD) [MIM:217800]
CHST6 Q9GZX3 VAR_075524 p.Pro186Arg LP/P rs376162109 Macular dystrophy, corneal (MCD) [MIM:217800]
CHST6 Q9GZX3 VAR_075525 p.Arg205Trp LP/P rs750219546 Macular dystrophy, corneal (MCD) [MIM:217800]
CHST6 Q9GZX3 VAR_075526 p.His308Tyr US - Macular dystrophy, corneal (MCD) [MIM:217800]
CHST6 Q9GZX3 VAR_075527 p.Tyr358His LP/P rs1384294258 Macular dystrophy, corneal (MCD) [MIM:217800]
CHST8 Q9H2A9 VAR_036538 p.Arg247His US rs1261984908 A colorectal cancer sample
CHST8 Q9H2A9 VAR_067723 p.Arg77Trp LP/P rs149660944 Peeling skin syndrome 3 (PSS3) [MIM:616265]
CHST9 Q7L1S5 VAR_055150 p.Ser122Asn LB/B rs17694469 -
CHST9 Q7L1S5 VAR_079095 p.Asn378Ser LB/B rs758130927 -
CHSY1 Q86X52 VAR_021173 p.Pro359Ser LB/B rs3743193 -
CHSY1 Q86X52 VAR_028009 p.Gln652His LB/B rs4426333 -
CHSY1 Q86X52 VAR_065822 p.Pro539Arg LP/P rs387906985 Temtamy preaxial brachydactyly syndrome (TPBS) [MIM:605282]
CHSY3 Q70JA7 VAR_021174 p.Asp764Gly LB/B rs2015018 -
CHSY3 Q70JA7 VAR_027540 p.Gly615Glu LB/B rs10068403 -
CHTF18 Q8WVB6 VAR_043990 p.Ser63Phe LB/B rs2277902 -
CHTF18 Q8WVB6 VAR_043991 p.Gln82Pro LB/B rs2277901 -
CHTF18 Q8WVB6 VAR_043992 p.Lys244Arg LB/B rs3765263 -
CHTF18 Q8WVB6 VAR_043993 p.Ala466Ser LB/B rs34595992 -
CHTF18 Q8WVB6 VAR_043994 p.Pro928Leu LB/B rs2294451 -
CHUK O15111 VAR_021359 p.Val268Ile LB/B rs2230804 -
CHUK O15111 VAR_040565 p.Ser126Cys LB/B rs34427437 -
CHUK O15111 VAR_040566 p.Val155Ala LB/B rs2230803 -
CIAO3 Q9H6Q4 VAR_053911 p.Val38Met LB/B rs8045850 -
CIAO3 Q9H6Q4 VAR_053912 p.His444Arg LB/B rs7188554 -

CIAPIN1 Q6FI81 VAR_033747 p.Ala34Glu LB/B rs11557672 -
CIAPIN1 Q6FI81 VAR_033748 p.Gln52Glu LB/B rs11557674 -
CIB1 Q99828 VAR_019565 p.Ser44Thr LB/B rs3210935 -
CIB1 Q99828 VAR_048636 p.Ile106Thr LB/B rs11551250 -
CIB2 O75838 VAR_069086 p.Glu64Asp LP/P rs145415848 Usher syndrome 1J (USH1J) [MIM:614869]
CIB2 O75838 VAR_069087 p.Phe91Ser LP/P rs397515411 Deafness, autosomal recessive, 48 (DFNB48) [MIM:609439]
CIB2 O75838 VAR_069088 p.Cys99Trp LP/P rs370965183 Deafness, autosomal recessive, 48 (DFNB48) [MIM:609439]
CIB2 O75838 VAR_069089 p.Ile123Thr LP/P rs397515412 Deafness, autosomal recessive, 48 (DFNB48) [MIM:609439]
CIB2 O75838 VAR_074552 p.Arg66Trp US rs780168150 Deafness, autosomal recessive, 48 (DFNB48) [MIM:609439]
CIB2 O75838 VAR_077559 p.Arg186Trp LP/P rs370359511 Deafness, autosomal recessive, 48 (DFNB48) [MIM:609439]
CIB2 O75838 VAR_080825 p.Val75Met US rs758743502 -
CIB3 Q96Q77 VAR_060268 p.Gly139Glu LB/B rs6512087 -
CIB4 A0PJX0 VAR_048637 p.His181Arg LB/B rs935172 -
CIBAR2 Q6ZTR7 VAR_043035 p.Glu22Lys LB/B rs9934891 -
CIC Q96RK0 VAR_028302 p.Ser982Gly LB/B rs17339472 -
CIC Q96RK0 VAR_035936 p.Glu104Lys US - A breast cancer sample
CIC Q96RK0 VAR_035937 p.Ala652Thr US rs770323488 A breast cancer sample
CIC Q96RK0 VAR_065090 p.Arg492Trp US rs373584239 Mental retardation, autosomal dominant 45 (MRD45) [MIM:617600]
CIDEA O60543 VAR_048738 p.Val115Phe LB/B rs11545881 -
CIITA P33076 VAR_005128 p.Gly500Ala LB/B rs4774 -
CIITA P33076 VAR_015551 p.Leu469Pro LP/P - Bare lymphocyte syndrome 2 (BLS2) [MIM:209920]
CIITA P33076 VAR_015552 p.Ala658Gly LB/B rs2229319 -
CIITA P33076 VAR_015553 p.Phe962Ser LP/P - Bare lymphocyte syndrome 2 (BLS2) [MIM:209920]
CIITA P33076 VAR_029270 p.Leu45Val LB/B rs2229317 -
CIITA P33076 VAR_047907 p.Arg174Gly LB/B rs8046121 -
CIITA P33076 VAR_047908 p.Gln900Arg LB/B rs7197779 -
CIITA P33076 VAR_057711 p.Val782Ala LB/B rs13336804 -
CIITA P33076 VAR_060104 p.Ser781Leu LB/B rs13330686 -
CILK1 Q9UPZ9 VAR_042001 p.Phe115Tyr US - A renal clear cell carcinoma sample
CILK1 Q9UPZ9 VAR_042002 p.Val320Ile LB/B rs33936662 -
CILK1 Q9UPZ9 VAR_042003 p.Thr471Lys LB/B rs56164633 -
CILK1 Q9UPZ9 VAR_042004 p.Arg476Gln LB/B rs55895113 -
CILK1 Q9UPZ9 VAR_042005 p.Ala615Thr US rs55932059 Juvenile myoclonic epilepsy 10 (EJM10) [MIM:617924]
CILK1 Q9UPZ9 VAR_053931 p.Pro98Leu LB/B rs1493105 -
CILK1 Q9UPZ9 VAR_057994 p.Arg272Gln LP/P rs118203918 Endocrine-cerebroosteodysplasia (ECO) [MIM:612651]
CILK1 Q9UPZ9 VAR_080554 p.Ile102Leu US rs748539319 Juvenile myoclonic epilepsy 10 (EJM10) [MIM:617924]
CILK1 Q9UPZ9 VAR_080555 p.Lys220Glu LP/P - Juvenile myoclonic epilepsy 10 (EJM10) [MIM:617924]
CILK1 Q9UPZ9 VAR_080556 p.Lys305Thr LP/P rs765078446 Juvenile myoclonic epilepsy 10 (EJM10) [MIM:617924]
CILP O75339 VAR_022768 p.Trp59Leu LB/B rs2585033 -

CILP O75339 VAR_022769 p.Ser327Phe LB/B - -
CILP O75339 VAR_022770 p.Ile395Thr LB/B rs2073711 -
CILP O75339 VAR_022771 p.Lys575Glu LB/B rs2679118 -
CILP O75339 VAR_022772 p.Ala895Val LB/B rs771628304 -
CILP O75339 VAR_022773 p.Gln979Arg LB/B rs2679117 -
CILP O75339 VAR_022774 p.Asp1101Asn LB/B rs769023414 -
CILP O75339 VAR_022775 p.Gly1166Ser LB/B rs938952 -
CILP O75339 VAR_022776 p.Val1168Ala LB/B rs747702148 -
CILP O75339 VAR_069430 p.Arg495His LB/B rs149286218 -
CILP O75339 VAR_069431 p.Ser1032Thr LB/B rs768702821 -
CINP Q9BW66 VAR_039979 p.Arg164His LB/B rs7011 -
CINP Q9BW66 VAR_039980 p.Asp177Asn US - A colorectal cancer sample
CIP2A Q8TCG1 VAR_046939 p.Lys126Arg LB/B rs7648496 -
CIP2A Q8TCG1 VAR_046940 p.Arg229Gln LB/B rs2278911 -
CIP2A Q8TCG1 VAR_046941 p.Thr409Ile LB/B rs9856308 -
CIP2A Q8TCG1 VAR_046942 p.Thr447Ala LB/B rs34944683 -
CIP2A Q8TCG1 VAR_046943 p.Pro494Leu LB/B rs13083928 -
CIP2A Q8TCG1 VAR_046944 p.Ser572Ala LB/B rs34172460 -
CIP2A Q8TCG1 VAR_046945 p.Arg680Thr LB/B rs6777766 -
CIP2A Q8TCG1 VAR_046946 p.Ile759Val LB/B rs13071874 -
CIPC Q9C0C6 VAR_028881 p.Leu31Phe LB/B rs11552006 -
CIPC Q9C0C6 VAR_049526 p.Thr203Ala LB/B rs759593 -
CIT O14578 VAR_040417 p.Gly7Glu LB/B rs36054900 -
CIT O14578 VAR_040418 p.Arg9Gln LB/B rs56193743 -
CIT O14578 VAR_040419 p.Leu183Phe LB/B - -
CIT O14578 VAR_077442 p.Gly106Val LP/P rs886037892 Microcephaly 17, primary, autosomal recessive (MCPH17) [MIM:617090]
CIT O14578 VAR_077443 p.Lys126Gln LP/P rs886037893 Microcephaly 17, primary, autosomal recessive (MCPH17) [MIM:617090]
CIT O14578 VAR_077444 p.Asp230Val LP/P rs886037894 Microcephaly 17, primary, autosomal recessive (MCPH17) [MIM:617090]
CITED1 Q99966 VAR_053038 p.His96Gln LB/B rs3012627 -
CIZ1 Q9ULV3 VAR_056820 p.Ala219Thr LB/B rs45588035 -
CIZ1 Q9ULV3 VAR_056821 p.Ser578Phe LB/B rs12334 -
CIZ1 Q9ULV3 VAR_056822 p.Val638Met LB/B rs11549266 -
CIZ1 Q9ULV3 VAR_063105 p.Glu370Gly LB/B rs45554035 -
CIZ1 Q9ULV3 VAR_063106 p.Arg847Gln LB/B rs11549260 -
CIZ1 Q9ULV3 VAR_067971 p.Pro47Ser LP/P - -
CIZ1 Q9ULV3 VAR_067972 p.Pro50Leu LB/B rs747696276 -
CIZ1 Q9ULV3 VAR_067973 p.Ser264Gly LP/P rs397514566 -
CIZ1 Q9ULV3 VAR_067974 p.Gln394Glu LB/B rs200010931 -
CIZ1 Q9ULV3 VAR_067975 p.Ser577Phe LB/B rs780188256 -
CIZ1 Q9ULV3 VAR_067976 p.Arg672Met LP/P - -
CKAP2 Q8WWK9 VAR_054018 p.Met236Lys LB/B rs35975899 -
CKAP2 Q8WWK9 VAR_069359 p.Ile323Val LB/B rs7335867 -
CKAP2L Q8IYA6 VAR_039735 p.Leu19Phe LB/B rs36093393 -
CKAP2L Q8IYA6 VAR_039736 p.Lys26Arg LB/B rs35593767 -
CKAP2L Q8IYA6 VAR_039737 p.Asn62Ser LB/B rs17042344 -
CKAP2L Q8IYA6 VAR_039738 p.Thr104Ile LB/B rs13007595 -

CKAP2L Q8IYA6 VAR_039739 p.Arg263Ser LB/B rs17042341 -
CKAP2L Q8IYA6 VAR_039740 p.Ile375Val LB/B rs6731822 -
CKAP2L Q8IYA6 VAR_039741 p.Pro379Ala LB/B rs2676126 -
CKAP2L Q8IYA6 VAR_039742 p.Ser519Gly LB/B rs36046436 -
CKAP2L Q8IYA6 VAR_039743 p.Leu614Ser LB/B rs3811040 -
CKAP2L Q8IYA6 VAR_039744 p.Glu706Asp LB/B rs3811039 -
CKAP4 Q07065 VAR_027853 p.Ala348Thr LB/B rs3088113 -
CKAP5 Q14008 VAR_045627 p.Tyr785Cys LB/B rs11038988 -
CKB P12277 VAR_025838 p.Lys177Arg LB/B rs36002620 -
CKB P12277 VAR_025839 p.Ser309Leu LB/B rs35156510 -
CKB P12277 VAR_049674 p.Leu360Phe LB/B rs12505 -
CKM P06732 VAR_018680 p.Glu83Gly LB/B rs11559024 -
CKM P06732 VAR_018681 p.Leu127Val LB/B rs17875653 -
CKM P06732 VAR_018682 p.Gly243Ala LB/B rs17875625 -
CKM P06732 VAR_049675 p.Thr166Met LB/B rs17357122 -
CLASP1 Q7Z460 VAR_053818 p.Ile233Thr LB/B rs17761055 -
CLASRP Q8N2M8 VAR_016809 p.Lys174Glu LB/B rs4803794 -
CLASRP Q8N2M8 VAR_035490 p.Leu213Ser US - A breast cancer sample
CLC Q05315 VAR_014765 p.Ala28Val LB/B rs17608 -
CLCA1 A8K7I4 VAR_043146 p.Asn357Ser LB/B rs2734705 -
CLCA1 A8K7I4 VAR_043147 p.Met524Thr LB/B rs2791494 -
CLCA1 A8K7I4 VAR_054654 p.Leu65Phe LB/B rs2145412 -
CLCA1 A8K7I4 VAR_054655 p.Arg152Lys LB/B rs2753386 -
CLCA1 A8K7I4 VAR_054656 p.Glu406Val LB/B rs1142185 -
CLCA1 A8K7I4 VAR_054657 p.Lys426Arg LB/B rs4647852 -
CLCA1 A8K7I4 VAR_054658 p.Tyr661His LB/B rs5744409 -
CLCA1 A8K7I4 VAR_054659 p.Lys760Asn LB/B rs2791483 -
CLCA2 Q9UQC9 VAR_043148 p.Gln306Glu LB/B rs17409304 -
CLCA2 Q9UQC9 VAR_043149 p.Gly754Glu US - A breast cancer sample
CLCA2 Q9UQC9 VAR_054057 p.Val80Ile LB/B rs11580625 -
CLCA2 Q9UQC9 VAR_054058 p.Gly534Asp LB/B rs1413426 -
CLCA3P Q9Y6N3 VAR_035296 p.Tyr84Thr LB/B rs2292830 -
CLCA3P Q9Y6N3 VAR_035297 p.Ile104Thr LB/B rs2292829 -
CLCA4 Q14CN2 VAR_045816 p.Pro43Ser LB/B rs2231580 -
CLCA4 Q14CN2 VAR_045817 p.Asp443Val LB/B rs2839932 -
CLCA4 Q14CN2 VAR_045818 p.Met449Leu LB/B rs1011048 -
CLCA4 Q14CN2 VAR_045819 p.Val810Leu LB/B rs2231604 -
CLCC1 Q96S66 VAR_034673 p.Ser368Arg LB/B rs168107 -
CLCC1 Q96S66 VAR_083125 p.Asp25Glu LP/P rs750180668 Retinitis pigmentosa 32 (RP32) [MIM:609913]
CLCF1 Q9UBD9 VAR_028354 p.Arg197Leu LP/P rs104894203 Crisponi/Cold-induced sweating syndrome 2 (CISS2) [MIM:610313]
CLCN1 P35523 VAR_001582 p.Arg105Cys LP/P rs201509501 Myotonia congenita, autosomal recessive (MCAR) [MIM:255700]
CLCN1 P35523 VAR_001583 p.Gly118Trp LB/B rs10282312 -
CLCN1 P35523 VAR_001584 p.Asp136Gly LP/P - Myotonia congenita, autosomal recessive (MCAR) [MIM:255700]
CLCN1 P35523 VAR_001585 p.Tyr150Cys LP/P - Myotonia congenita, autosomal recessive (MCAR) [MIM:255700]

CLCN1 P35523 VAR_001586 p.Phe161Val LP/P - Myotonia congenita, autosomal dominant (MCAD) [MIM:160800]
CLCN1 P35523 VAR_001586 p.Phe161Val LP/P - Myotonia congenita, autosomal recessive (MCAR) [MIM:255700]
CLCN1 P35523 VAR_001587 p.Val165Gly LP/P rs1586485438 Myotonia congenita, autosomal recessive (MCAR) [MIM:255700]
CLCN1 P35523 VAR_001588 p.Phe167Leu LP/P rs149729531 Myotonia congenita, autosomal recessive (MCAR) [MIM:255700]
CLCN1 P35523 VAR_001589 p.Gly200Arg LP/P rs1563074523 Myotonia congenita, autosomal dominant (MCAD) [MIM:160800]
CLCN1 P35523 VAR_001589 p.Gly200Arg LP/P rs1563074523 Myotonia congenita, autosomal recessive (MCAR) [MIM:255700]
CLCN1 P35523 VAR_001590 p.Gly230Glu LP/P rs80356700 Myotonia congenita, autosomal dominant (MCAD) [MIM:160800]
CLCN1 P35523 VAR_001590 p.Gly230Glu LP/P rs80356700 Myotonia congenita, autosomal recessive (MCAR) [MIM:255700]
CLCN1 P35523 VAR_001591 p.Val236Leu LP/P rs776173406 Myotonia congenita, autosomal recessive (MCAR) [MIM:255700]
CLCN1 P35523 VAR_001592 p.Tyr261Cys LP/P rs200621976 Myotonia congenita, autosomal recessive (MCAR) [MIM:255700]
CLCN1 P35523 VAR_001593 p.Gly285Glu LP/P rs150885084 Myotonia congenita, autosomal recessive (MCAR) [MIM:255700]
CLCN1 P35523 VAR_001594 p.Val286Ala LP/P rs80356689 Myotonia congenita, autosomal dominant (MCAD) [MIM:160800]
CLCN1 P35523 VAR_001595 p.Ile290Met LP/P rs80356690 Myotonia congenita, autosomal dominant (MCAD) [MIM:160800]
CLCN1 P35523 VAR_001596 p.Glu291Lys LP/P rs121912805 Myotonia congenita, autosomal recessive (MCAR) [MIM:255700]
CLCN1 P35523 VAR_001597 p.Arg300Gln LB/B rs118066140 -
CLCN1 P35523 VAR_001598 p.Phe307Ser LP/P rs80356701 Myotonia congenita, autosomal dominant (MCAD) [MIM:160800]
CLCN1 P35523 VAR_001599 p.Ala313Thr LP/P rs80356692 Myotonia congenita, autosomal dominant (MCAD) [MIM:160800]
CLCN1 P35523 VAR_001599 p.Ala313Thr LP/P rs80356692 Myotonia congenita, autosomal recessive (MCAR) [MIM:255700]
CLCN1 P35523 VAR_001600 p.Arg317Gln LP/P rs80356702 Myotonia congenita, autosomal dominant (MCAD) [MIM:160800]
CLCN1 P35523 VAR_001601 p.Val327Ile LP/P rs774396430 Myotonia congenita, autosomal recessive (MCAR) [MIM:255700]
CLCN1 P35523 VAR_001602 p.Ile329Thr LP/P - Myotonia congenita, autosomal recessive (MCAR) [MIM:255700]
CLCN1 P35523 VAR_001603 p.Arg338Gln LP/P rs80356703 Myotonia congenita, autosomal dominant (MCAD) [MIM:160800]
CLCN1 P35523 VAR_001603 p.Arg338Gln LP/P rs80356703 Myotonia congenita, autosomal recessive (MCAR) [MIM:255700]
CLCN1 P35523 VAR_001604 p.Phe413Cys LP/P rs121912799 Myotonia congenita, autosomal recessive (MCAR) [MIM:255700]
CLCN1 P35523 VAR_001605 p.Ala415Val LP/P - Myotonia congenita, autosomal recessive (MCAR) [MIM:255700]

CLCN1 P35523 VAR_001606 p.Ala437Thr LB/B rs41276054 -
CLCN1 P35523 VAR_001607 p.Pro480Leu LP/P rs80356694 Myotonia congenita, autosomal dominant (MCAD) [MIM:160800]
CLCN1 P35523 VAR_001608 p.Gly482Arg LP/P rs746125212 Myotonia congenita, autosomal recessive (MCAR) [MIM:255700]
CLCN1 P35523 VAR_001609 p.Met485Val LP/P rs146457619 Myotonia congenita, autosomal recessive (MCAR) [MIM:255700]
CLCN1 P35523 VAR_001610 p.Arg496Ser LP/P rs121912801 Myotonia congenita, autosomal recessive (MCAR) [MIM:255700]
CLCN1 P35523 VAR_001611 p.Gln552Arg LP/P rs80356696 Myotonia congenita, autosomal dominant (MCAD) [MIM:160800]
CLCN1 P35523 VAR_001611 p.Gln552Arg LP/P rs80356696 Myotonia congenita, autosomal recessive (MCAR) [MIM:255700]
CLCN1 P35523 VAR_001612 p.Ile556Asn LP/P rs80356697 Myotonia congenita, autosomal dominant (MCAD) [MIM:160800]
CLCN1 P35523 VAR_001612 p.Ile556Asn LP/P rs80356697 Myotonia congenita, autosomal recessive (MCAR) [MIM:255700]
CLCN1 P35523 VAR_001613 p.Val563Ile LP/P - Myotonia congenita, autosomal recessive (MCAR) [MIM:255700]
CLCN1 P35523 VAR_001614 p.Phe708Leu LP/P - Myotonia congenita, autosomal recessive (MCAR) [MIM:255700]
CLCN1 P35523 VAR_036300 p.Glu548Lys US rs546411827 A breast cancer sample
CLCN1 P35523 VAR_047779 p.Pro727Leu LB/B rs13438232 -
CLCN1 P35523 VAR_075588 p.Gln43Arg LP/P rs868831424 Myotonia congenita, autosomal recessive (MCAR) [MIM:255700]
CLCN1 P35523 VAR_075589 p.Ser70Leu US rs769312894 Myotonia congenita, autosomal recessive (MCAR) [MIM:255700]
CLCN1 P35523 VAR_075590 p.Thr82Ala US rs772100356 Myotonia congenita, autosomal recessive (MCAR) [MIM:255700]
CLCN1 P35523 VAR_075591 p.Met128Val LP/P rs80356699 Myotonia congenita, autosomal dominant (MCAD) [MIM:160800]
CLCN1 P35523 VAR_075592 p.Tyr137Asp LP/P rs748639603 Myotonia congenita, autosomal recessive (MCAR) [MIM:255700]
CLCN1 P35523 VAR_075593 p.Gln154Arg LB/B rs111482384 -
CLCN1 P35523 VAR_075594 p.Gln160His LP/P rs771532474 Myotonia congenita, autosomal recessive (MCAR) [MIM:255700]
CLCN1 P35523 VAR_075595 p.Trp164Arg LP/P - Myotonia congenita, autosomal recessive (MCAR) [MIM:255700]
CLCN1 P35523 VAR_075596 p.Gly190Ser LP/P rs797045032 Myotonia congenita, autosomal recessive (MCAR) [MIM:255700]
CLCN1 P35523 VAR_075597 p.Glu193Lys LP/P rs80356686 Myotonia congenita, autosomal dominant (MCAD) [MIM:160800]
CLCN1 P35523 VAR_075598 p.Ile197Arg LP/P - Myotonia congenita, autosomal recessive (MCAR) [MIM:255700]
CLCN1 P35523 VAR_075599 p.Leu198Pro LP/P rs1347382107 Myotonia congenita, autosomal dominant (MCAD) [MIM:160800]
CLCN1 P35523 VAR_075600 p.Gly270Val LP/P - Myotonia congenita, autosomal recessive (MCAR) [MIM:255700]

CLCN1 P35523 VAR_075601 p.Cys277Arg LP/P rs757109632 Myotonia congenita, autosomal recessive (MCAR) [MIM:255700]
CLCN1 P35523 VAR_075602 p.Cys277Tyr LP/P - Myotonia congenita, autosomal recessive (MCAR) [MIM:255700]
CLCN1 P35523 VAR_075603 p.Gln412Pro LP/P rs1279658001 Myotonia congenita, autosomal recessive (MCAR) [MIM:255700]
CLCN1 P35523 VAR_075604 p.Arg453Trp US rs376026619 Myotonia congenita, autosomal recessive (MCAR) [MIM:255700]
CLCN1 P35523 VAR_075605 p.Phe484Leu LP/P rs1312002847 Myotonia congenita, autosomal dominant (MCAD) [MIM:160800]
CLCN1 P35523 VAR_075606 p.Gly499Arg LP/P rs121912807 Myotonia congenita, autosomal recessive (MCAR) [MIM:255700]
CLCN1 P35523 VAR_075607 p.Ile527Thr US - Myotonia congenita, autosomal recessive (MCAR) [MIM:255700]
CLCN1 P35523 VAR_075608 p.Thr533Ile US - Myotonia congenita, autosomal recessive (MCAR) [MIM:255700]
CLCN1 P35523 VAR_075609 p.Val536Leu US rs777685454 Myotonia congenita, autosomal recessive (MCAR) [MIM:255700]
CLCN1 P35523 VAR_075610 p.Lys614Asn LB/B rs140205115 -
CLCN1 P35523 VAR_075611 p.Leu628Pro US - Myotonia congenita, autosomal recessive (MCAR) [MIM:255700]
CLCN1 P35523 VAR_075612 p.Val640Gly LP/P - Myotonia congenita, autosomal recessive (MCAR) [MIM:255700]
CLCN1 P35523 VAR_075613 p.Gly845Ser US rs755433272 Myotonia congenita, autosomal recessive (MCAR) [MIM:255700]
CLCN1 P35523 VAR_075614 p.Gly855Glu US rs1554439879 Myotonia congenita, autosomal recessive (MCAR) [MIM:255700]
CLCN1 P35523 VAR_075615 p.Pro932Leu US rs80356706 Myotonia congenita, autosomal recessive (MCAR) [MIM:255700]
CLCN1 P35523 VAR_075616 p.Val947Glu US - Myotonia congenita, autosomal recessive (MCAR) [MIM:255700]
CLCN1 P35523 VAR_077244 p.Pro480His LP/P - Myotonia congenita, autosomal dominant (MCAD) [MIM:160800]
CLCN1 P35523 VAR_079520 p.Glu950Lys US rs201506176 Myotonia congenita, autosomal dominant (MCAD) [MIM:160800]
CLCN2 P51788 VAR_015989 p.Gly715Glu US rs137852681 Juvenile absence epilepsy 2 (JAE2) [MIM:607628]
CLCN2 P51788 VAR_054550 p.Thr668Ser LB/B rs9820367 -
CLCN2 P51788 VAR_054551 p.Glu718Asp LB/B rs2228292 -
CLCN2 P51788 VAR_057886 p.Pro48Arg LB/B rs115661422 -
CLCN2 P51788 VAR_057887 p.Arg68His LB/B rs61729156 -
CLCN2 P51788 VAR_057888 p.Gly199Ala LB/B rs863225248 -
CLCN2 P51788 VAR_057889 p.Arg235Gln LP/P rs71318369 Juvenile myoclonic epilepsy 8 (EJM8) [MIM:607628]
CLCN2 P51788 VAR_057890 p.Arg577Gln LP/P rs137852682 Epilepsy, idiopathic generalized 11 (EIG11) [MIM:607628]
CLCN2 P51788 VAR_057891 p.Arg644Cys LB/B rs148545588 -
CLCN2 P51788 VAR_057892 p.Arg646Gln LB/B rs115961753 -
CLCN2 P51788 VAR_057893 p.Arg725Trp LB/B rs114702742 -
CLCN2 P51788 VAR_057894 p.Arg747His LB/B rs144164281 -

CLCN2 P51788 VAR_058426 p.Ser719Leu US rs138573287 -
CLCN2 P51788 VAR_070977 p.Ala500Val LP/P rs587777111 Leukoencephalopathy with ataxia (LKPAT) [MIM:615651]
CLCN2 P51788 VAR_081154 p.Met22Lys LP/P rs758379595 Hyperaldosteronism, familial, 2 (HALD2) [MIM:605635]
CLCN2 P51788 VAR_081155 p.Gly24Asp LP/P rs1085307938 Hyperaldosteronism, familial, 2 (HALD2) [MIM:605635]
CLCN2 P51788 VAR_081156 p.Tyr26Asn LP/P rs1553857113 Hyperaldosteronism, familial, 2 (HALD2) [MIM:605635]
CLCN2 P51788 VAR_081157 p.Arg172Gln LP/P rs1293789661 Hyperaldosteronism, familial, 2 (HALD2) [MIM:605635]
CLCN2 P51788 VAR_081159 p.Ser865Arg LP/P rs1553853557 Hyperaldosteronism, familial, 2 (HALD2) [MIM:605635]
CLCN4 P51793 VAR_077819 p.Gly78Ser LP/P rs1569226551 Raynaud-Claes syndrome (MRXSRC) [MIM:300114]
CLCN4 P51793 VAR_077820 p.Leu221Val LP/P rs1569230006 Raynaud-Claes syndrome (MRXSRC) [MIM:300114]
CLCN4 P51793 VAR_077821 p.Val536Met LP/P rs1569231897 Raynaud-Claes syndrome (MRXSRC) [MIM:300114]
CLCN4 P51793 VAR_077822 p.Gly544Arg LP/P rs587777161 Raynaud-Claes syndrome (MRXSRC) [MIM:300114]
CLCN4 P51793 VAR_077823 p.Gly731Arg LP/P rs1569233549 Raynaud-Claes syndrome (MRXSRC) [MIM:300114]
CLCN4 P51793 VAR_083577 p.Asp15Asn US rs879255591 Raynaud-Claes syndrome (MRXSRC) [MIM:300114]
CLCN4 P51793 VAR_083578 p.Val212Gly LP/P rs879255580 Raynaud-Claes syndrome (MRXSRC) [MIM:300114]
CLCN4 P51793 VAR_083579 p.Leu221Pro LP/P rs879255581 Raynaud-Claes syndrome (MRXSRC) [MIM:300114]
CLCN4 P51793 VAR_083580 p.Val275Met LP/P rs879255585 Raynaud-Claes syndrome (MRXSRC) [MIM:300114]
CLCN4 P51793 VAR_083581 p.Ser534Leu LP/P rs879255582 Raynaud-Claes syndrome (MRXSRC) [MIM:300114]
CLCN4 P51793 VAR_083582 p.Ala555Val LP/P rs879255583 Raynaud-Claes syndrome (MRXSRC) [MIM:300114]
CLCN4 P51793 VAR_083583 p.Arg718Trp LP/P rs879255584 Raynaud-Claes syndrome (MRXSRC) [MIM:300114]
CLCN5 P51795 VAR_001616 p.Gly127Val LP/P rs151340629 Nephrolithiasis 2 (NPHL2) [MIM:300009]
CLCN5 P51795 VAR_001617 p.Leu270Arg LP/P rs151340622 Nephrolithiasis 2 (NPHL2) [MIM:300009]
CLCN5 P51795 VAR_001618 p.Ser314Leu LP/P rs151340626 Hypophosphatemic rickets, X-linked recessive (XLRHR) [MIM:300554]
CLCN5 P51795 VAR_001619 p.Arg350Pro LP/P rs151340628 Low molecular weight proteinuria with hypercalciuria and nephrocalcinosis (LMWPHN) [MIM:308990]
CLCN5 P51795 VAR_001620 p.Gly576Glu LP/P rs151340625 Nephrolithiasis 1 (NPHL1) [MIM:310468]
CLCN5 P51795 VAR_001621 p.Gly582Arg LP/P - Nephrolithiasis 2 (NPHL2) [MIM:300009]
CLCN5 P51795 VAR_001622 p.Ser590Pro LP/P rs151340623 Nephrolithiasis 2 (NPHL2) [MIM:300009]
CLCN5 P51795 VAR_001623 p.Glu597Asp LP/P - Nephrolithiasis 2 (NPHL2) [MIM:300009]
CLCN5 P51795 VAR_048694 p.Met212Ile LB/B rs34800648 -
CLCN5 P51795 VAR_065591 p.Gly249Asp LP/P - Nephrolithiasis 2 (NPHL2) [MIM:300009]
CLCN5 P51795 VAR_065592 p.Ser273Leu LP/P - Nephrolithiasis 2 (NPHL2) [MIM:300009]

CLCN5 P51795 VAR_065593 p.Gly282Ala LP/P - Nephrolithiasis 2 (NPHL2) [MIM:300009]
CLCN5 P51795 VAR_065594 p.Cys289Arg LP/P - Nephrolithiasis 2 (NPHL2) [MIM:300009]
CLCN5 P51795 VAR_065595 p.Cys291Arg LP/P - Nephrolithiasis 2 (NPHL2) [MIM:300009]
CLCN5 P51795 VAR_065596 p.Leu295Pro LP/P rs273585645 Nephrolithiasis 2 (NPHL2) [MIM:300009]
CLCN5 P51795 VAR_065597 p.Gly330Val LP/P rs151340630 Nephrolithiasis 2 (NPHL2) [MIM:300009]
CLCN5 P51795 VAR_065598 p.Glu337Ala LP/P - Nephrolithiasis 2 (NPHL2) [MIM:300009]
CLCN5 P51795 VAR_065600 p.Ser340Gly LP/P - Nephrolithiasis 2 (NPHL2) [MIM:300009]
CLCN5 P51795 VAR_065601 p.Ser340Arg LP/P - Nephrolithiasis 2 (NPHL2) [MIM:300009]
CLCN5 P51795 VAR_065602 p.Tyr342Cys LP/P rs273585644 Nephrolithiasis 2 (NPHL2) [MIM:300009]
CLCN5 P51795 VAR_065603 p.Phe343Leu LP/P - Nephrolithiasis 2 (NPHL2) [MIM:300009]
CLCN5 P51795 VAR_065604 p.Leu348Phe LP/P rs273585648 Nephrolithiasis 2 (NPHL2) [MIM:300009]
CLCN5 P51795 VAR_065605 p.Asn410Lys LP/P rs273585646 Nephrolithiasis 2 (NPHL2) [MIM:300009]
CLCN5 P51795 VAR_065606 p.Gly532Asp LP/P - Nephrolithiasis 2 (NPHL2) [MIM:300009]
CLCN5 P51795 VAR_065607 p.Leu539Pro LP/P - Nephrolithiasis 2 (NPHL2) [MIM:300009]
CLCN5 P51795 VAR_065608 p.Gly583Glu LP/P - Nephrolithiasis 2 (NPHL2) [MIM:300009]
CLCN5 P51795 VAR_065609 p.Gly583Arg LP/P rs273585647 Nephrolithiasis 2 (NPHL2) [MIM:300009]
CLCN5 P51795 VAR_065610 p.Arg586Trp LP/P rs797044812 Nephrolithiasis 2 (NPHL2) [MIM:300009]
CLCN5 P51795 VAR_065611 p.Ile594Lys LP/P - Low molecular weight proteinuria with hypercalciuria and nephrocalcinosis (LMWPHN) [MIM:308990]
CLCN5 P51795 VAR_065612 p.Ser615Asn LP/P - Nephrolithiasis 2 (NPHL2) [MIM:300009]
CLCN5 P51795 VAR_065613 p.Lys616Glu LP/P - Nephrolithiasis 2 (NPHL2) [MIM:300009]
CLCN5 P51795 VAR_065614 p.Trp617Gly LP/P rs273585650 Nephrolithiasis 2 (NPHL2) [MIM:300009]
CLCN5 P51795 VAR_065615 p.Thr727Ser LP/P rs144207967 Nephrolithiasis 2 (NPHL2) [MIM:300009]
CLCN5 P51795 VAR_075519 p.Gly403Arg US - Nephrolithiasis 2 (NPHL2) [MIM:300009]
CLCN6 P51797 VAR_023051 p.Glu198Gly LB/B rs198400 -
CLCN7 P51798 VAR_017838 p.Arg762Gln LP/P rs121434433 Osteopetrosis, autosomal dominant 2 (OPTA2) [MIM:166600]
CLCN7 P51798 VAR_017838 p.Arg762Gln LP/P rs121434433 Osteopetrosis, autosomal recessive 4 (OPTB4) [MIM:611490]
CLCN7 P51798 VAR_017839 p.Leu766Pro LP/P rs121434434 Osteopetrosis, autosomal recessive 4 (OPTB4) [MIM:611490]
CLCN7 P51798 VAR_017840 p.Arg767Trp LP/P rs121434435 Osteopetrosis, autosomal dominant 2 (OPTA2) [MIM:166600]
CLCN7 P51798 VAR_017840 p.Arg767Trp LP/P rs121434435 Osteopetrosis, autosomal recessive 4 (OPTB4) [MIM:611490]
CLCN7 P51798 VAR_020997 p.Gly215Arg LP/P rs397515539 Osteopetrosis, autosomal dominant 2 (OPTA2) [MIM:166600]
CLCN7 P51798 VAR_020998 p.Gly240Arg LP/P rs1360480518 Osteopetrosis, autosomal recessive 4 (OPTB4) [MIM:611490]
CLCN7 P51798 VAR_020999 p.Pro249Arg LP/P - Osteopetrosis, autosomal recessive 4 (OPTB4) [MIM:611490]
CLCN7 P51798 VAR_021000 p.Arg286Gln LP/P rs760956030 Osteopetrosis, autosomal dominant 2 (OPTA2) [MIM:166600]
CLCN7 P51798 VAR_021001 p.Met332Val LP/P - Osteopetrosis, autosomal recessive 4 (OPTB4) [MIM:611490]
CLCN7 P51798 VAR_021002 p.Val418Met LB/B rs12926089 -
CLCN7 P51798 VAR_021003 p.Leu490Phe LP/P - Osteopetrosis, autosomal dominant 2 (OPTA2) [MIM:166600]

CLCN7 P51798 VAR_021004 p.Arg526Trp LP/P rs1233085260 Osteopetrosis, autosomal recessive 4 (OPTB4) [MIM:611490]
CLCN7 P51798 VAR_021005 p.Leu614Pro LP/P rs1064794323 Osteopetrosis, autosomal recessive 4 (OPTB4) [MIM:611490]
CLCN7 P51798 VAR_021006 p.Gly677Val LP/P - Osteopetrosis, autosomal dominant 2 (OPTA2) [MIM:166600]
CLCN7 P51798 VAR_021007 p.Ser744Phe LP/P rs1320932332 Osteopetrosis, autosomal recessive 4 (OPTB4) [MIM:611490]
CLCN7 P51798 VAR_021008 p.Arg767Gln LP/P rs772579858 Osteopetrosis, autosomal recessive 4 (OPTB4) [MIM:611490]
CLCN7 P51798 VAR_037427 p.Ile261Phe LP/P rs121434436 Osteopetrosis, autosomal recessive 4 (OPTB4) [MIM:611490]
CLCN7 P51798 VAR_064637 p.Leu132Pro LP/P - Osteopetrosis, autosomal recessive 4 (OPTB4) [MIM:611490]
CLCN7 P51798 VAR_064638 p.Asn214Ser LP/P rs367567630 Osteopetrosis, autosomal recessive 4 (OPTB4) [MIM:611490]
CLCN7 P51798 VAR_064640 p.Phe318Leu LP/P - Osteopetrosis, autosomal dominant 2 (OPTA2) [MIM:166600]
CLCN7 P51798 VAR_064641 p.Arg403Gln LP/P rs765444328 Osteopetrosis, autosomal recessive 4 (OPTB4) [MIM:611490]
CLCN7 P51798 VAR_064642 p.Gly521Arg LP/P rs368190250 Osteopetrosis, autosomal recessive 4 (OPTB4) [MIM:611490]
CLCN7 P51798 VAR_064643 p.Arg526Gln LP/P rs139329533 Osteopetrosis, autosomal recessive 4 (OPTB4) [MIM:611490]
CLCN7 P51798 VAR_064644 p.Leu549Pro LP/P - Osteopetrosis, autosomal recessive 4 (OPTB4) [MIM:611490]
CLCN7 P51798 VAR_064645 p.Leu651Pro LP/P - Osteopetrosis, autosomal recessive 4 (OPTB4) [MIM:611490]
CLCN7 P51798 VAR_064646 p.Phe758Leu LP/P rs760740877 Osteopetrosis, autosomal dominant 2 (OPTA2) [MIM:166600]
CLCN7 P51798 VAR_064647 p.Arg762Trp LP/P rs1490598538 Osteopetrosis, autosomal recessive 4 (OPTB4) [MIM:611490]
CLCN7 P51798 VAR_064648 p.Arg767Pro LP/P - Osteopetrosis, autosomal recessive 4 (OPTB4) [MIM:611490]
CLCN7 P51798 VAR_075576 p.Leu213Phe US - Osteopetrosis, autosomal dominant 2 (OPTA2) [MIM:166600]
CLCN7 P51798 VAR_075577 p.Leu224Arg US - Osteopetrosis, autosomal recessive 4 (OPTB4) [MIM:611490]
CLCN7 P51798 VAR_075578 p.Arg286Trp US rs1291061962 Osteopetrosis, autosomal dominant 2 (OPTA2) [MIM:166600]
CLCN7 P51798 VAR_075579 p.Ser290Tyr US - Osteopetrosis, autosomal dominant 2 (OPTA2) [MIM:166600]
CLCN7 P51798 VAR_075580 p.Ala299Val US rs977932714 Osteopetrosis, autosomal recessive 4 (OPTB4) [MIM:611490]
CLCN7 P51798 VAR_075581 p.Arg326Gly US - Osteopetrosis, autosomal dominant 2 (OPTA2) [MIM:166600]
CLCN7 P51798 VAR_075582 p.Gly347Arg US - Osteopetrosis, autosomal dominant 2 (OPTA2) [MIM:166600]

CLCN7 P51798 VAR_075583 p.Ser473Asn US - Osteopetrosis, autosomal dominant 2 (OPTA2) [MIM:166600]
CLCN7 P51798 VAR_075584 p.Leu564Pro US - Osteopetrosis, autosomal dominant 2 (OPTA2) [MIM:166600]
CLCN7 P51798 VAR_083175 p.Tyr715Cys LP/P rs1057517718 Hypopigmentation, organomegaly, and delayed myelination and development (HOD) [MIM:618541]
CLCNKA P51800 VAR_014465 p.Ala447Thr LB/B rs1805152 -
CLCNKA P51800 VAR_019787 p.Arg83Gly LB/B rs10927887 -
CLCNKA P51800 VAR_019788 p.Tyr315Phe LB/B rs12126269 -
CLCNKA P51800 VAR_030784 p.Met67Ile LB/B rs17855678 -
CLCNKA P51800 VAR_033768 p.Arg45His LB/B rs35932996 -
CLCNKA P51800 VAR_048695 p.Arg8His LB/B rs9442189 -
CLCNKA P51800 VAR_059209 p.Arg534Trp LB/B rs12140223 -
CLCNKA P51800 VAR_061095 p.Pro683Leu LB/B rs12746751 -
CLCNKA P51800 VAR_063074 p.Trp80Cys LP/P rs121909137 Bartter syndrome 4B, neonatal, with sensorineural deafness (BARTS4B) [MIM:613090]
CLCNKA P51800 VAR_068971 p.His357Gln LB/B rs79751787 -
CLCNKB P51801 VAR_001624 p.Pro124Leu LP/P rs121909131 Bartter syndrome 3 (BARTS3) [MIM:607364]
CLCNKB P51801 VAR_001625 p.Ala204Thr LP/P rs121909132 Bartter syndrome 3 (BARTS3) [MIM:607364]
CLCNKB P51801 VAR_001626 p.Ala349Asp LP/P rs121909134 Bartter syndrome 3 (BARTS3) [MIM:607364]
CLCNKB P51801 VAR_001627 p.Tyr432His LP/P rs121909135 Bartter syndrome 3 (BARTS3) [MIM:607364]
CLCNKB P51801 VAR_001628 p.Arg438Cys LP/P rs121909133 Bartter syndrome 3 (BARTS3) [MIM:607364]
CLCNKB P51801 VAR_014466 p.Ser88Arg LB/B rs5256 -
CLCNKB P51801 VAR_014467 p.Asn143His LB/B rs5259 -
CLCNKB P51801 VAR_014468 p.Val334Leu LB/B rs5251 -
CLCNKB P51801 VAR_014469 p.Met562Thr LB/B rs5253 -
CLCNKB P51801 VAR_024409 p.Lys578Glu LB/B rs2275166 -
CLCNKB P51801 VAR_033770 p.Phe4Leu LB/B rs34851419 -
CLCNKB P51801 VAR_033771 p.Val104Ile LB/B rs35530360 -
CLCNKB P51801 VAR_033772 p.Ala214Gly LB/B rs1889789 -
CLCNKB P51801 VAR_033773 p.Ile419Val LB/B rs6650119 -
CLCNKB P51801 VAR_046797 p.Arg27Leu LB/B rs2015352 -
CLCNKB P51801 VAR_046799 p.Arg395Trp LB/B rs34255952 -
CLCNKB P51801 VAR_046800 p.Thr481Ser LB/B rs12140311 -
CLCNKB P51801 VAR_046801 p.Ser660Leu LB/B rs5255 -
CLCNKB P51801 VAR_069104 p.Ala287Val LB/B rs7367494 -
CLDN10 P78369 VAR_080053 p.Asn48Lys LP/P rs759408749 HELIX syndrome (HELIX) [MIM:617671]
CLDN10 P78369 VAR_080054 p.Ser131Leu LP/P rs1555299783 HELIX syndrome (HELIX) [MIM:617671]
CLDN14 O95500 VAR_010738 p.Val85Asp LP/P rs74315437 Deafness, autosomal recessive, 29 (DFNB29) [MIM:614035]
CLDN14 O95500 VAR_017227 p.Thr4Met LB/B rs113831133 -
CLDN14 O95500 VAR_069979 p.Arg81His LP/P rs368027306 Deafness, autosomal recessive, 29 (DFNB29) [MIM:614035]
CLDN14 O95500 VAR_069980 p.Ile86Val LB/B - -
CLDN14 O95500 VAR_069981 p.Ser87Ile LP/P - Deafness, autosomal recessive, 29 (DFNB29) [MIM:614035]
CLDN14 O95500 VAR_069982 p.Ala94Val LP/P - Deafness, autosomal recessive, 29 (DFNB29) [MIM:614035]

CLDN14 O95500 VAR_069983 p.Gly232Arg LP/P rs786204841 Deafness, autosomal recessive, 29 (DFNB29) [MIM:614035]
CLDN16 Q9Y5I7 VAR_008172 p.Gly239Arg LP/P rs104893721 Hypomagnesemia 3 (HOMG3) [MIM:248250]
CLDN16 Q9Y5I7 VAR_008173 p.Met71Arg LP/P rs104893724 Hypomagnesemia 3 (HOMG3) [MIM:248250]
CLDN16 Q9Y5I7 VAR_008174 p.Leu167Pro LP/P rs104893725 Hypomagnesemia 3 (HOMG3) [MIM:248250]
CLDN16 Q9Y5I7 VAR_008175 p.Gly191Arg LP/P rs104893722 Hypomagnesemia 3 (HOMG3) [MIM:248250]
CLDN16 Q9Y5I7 VAR_008176 p.Gly198Asp LP/P rs104893723 Hypomagnesemia 3 (HOMG3) [MIM:248250]
CLDN16 Q9Y5I7 VAR_008177 p.Phe232Cys LP/P rs104893726 Hypomagnesemia 3 (HOMG3) [MIM:248250]
CLDN16 Q9Y5I7 VAR_008178 p.Gly233Asp LP/P rs104893727 Hypomagnesemia 3 (HOMG3) [MIM:248250]
CLDN16 Q9Y5I7 VAR_008179 p.Ser235Phe LP/P rs104893728 Hypomagnesemia 3 (HOMG3) [MIM:248250]
CLDN16 Q9Y5I7 VAR_017228 p.His141Asp LP/P - Hypomagnesemia 3 (HOMG3) [MIM:248250]
CLDN16 Q9Y5I7 VAR_017229 p.Leu145Pro LP/P rs104893731 Hypomagnesemia 3 (HOMG3) [MIM:248250]
CLDN16 Q9Y5I7 VAR_017230 p.Arg149Leu LP/P rs968906940 Hypomagnesemia 3 (HOMG3) [MIM:248250]
CLDN16 Q9Y5I7 VAR_017231 p.Leu151Phe LP/P rs104893729 Hypomagnesemia 3 (HOMG3) [MIM:248250]
CLDN16 Q9Y5I7 VAR_017232 p.Leu151Pro LP/P - Hypomagnesemia 3 (HOMG3) [MIM:248250]
CLDN16 Q9Y5I7 VAR_017233 p.Leu151Trp LP/P rs104893730 Hypomagnesemia 3 (HOMG3) [MIM:248250]
CLDN16 Q9Y5I7 VAR_017234 p.Gly198Ala LP/P - Hypomagnesemia 3 (HOMG3) [MIM:248250]
CLDN16 Q9Y5I7 VAR_017235 p.Ala209Thr LP/P rs1270704258 Hypomagnesemia 3 (HOMG3) [MIM:248250]
CLDN16 Q9Y5I7 VAR_017236 p.Arg216Thr LP/P - Hypomagnesemia 3 (HOMG3) [MIM:248250]
CLDN16 Q9Y5I7 VAR_017237 p.Ser235Pro LP/P - Hypomagnesemia 3 (HOMG3) [MIM:248250]
CLDN17 P56750 VAR_033774 p.Ala82Thr LB/B rs35531957 -
CLDN18 P56856 VAR_033775 p.Met149Leu LB/B rs17204075 -
CLDN19 Q8N6F1 VAR_031238 p.Leu13Phe LB/B rs12065961 -
CLDN19 Q8N6F1 VAR_031239 p.Gly20Asp LP/P rs118203979 Hypomagnesemia 5, renal, with or without ocular involvement (HOMG5) [MIM:248190]
CLDN19 Q8N6F1 VAR_031240 p.Gln57Glu LP/P rs118203980 Hypomagnesemia 5, renal, with or without ocular involvement (HOMG5) [MIM:248190]
CLDN19 Q8N6F1 VAR_031241 p.Leu90Pro LP/P rs118203981 Hypomagnesemia 5, renal, with or without ocular involvement (HOMG5) [MIM:248190]
CLDN23 Q96B33 VAR_059219 p.Val210Met LB/B rs12548737 -
CLDN24 A6NM45 VAR_055660 p.Leu18Phe LB/B rs7688467 -
CLDN25 C9JDP6 VAR_063401 p.His219Tyr LB/B rs35111413 -
CLDN6 P56747 VAR_017151 p.Ile143Val LB/B rs2257295 -
CLDN7 O95471 VAR_014538 p.Val197Ala LB/B rs4562 -
CLDN7 O95471 VAR_030736 p.Ala133Thr LB/B rs17849410 -
CLDN8 P56748 VAR_020387 p.Ser151Pro LB/B rs686364 -
CLDN8 P56748 VAR_024417 p.Thr129Ala LB/B rs685967 -
CLDN8 P56748 VAR_048739 p.Thr25Ala LB/B rs1557294 -
CLEC10A Q8IUN9 VAR_021262 p.Cys35Arg LB/B rs90951 -
CLEC10A Q8IUN9 VAR_050113 p.Arg73Lys LB/B rs16956478 -
CLEC10A Q8IUN9 VAR_050114 p.Thr100Met LB/B rs35318160 -
CLEC10A Q8IUN9 VAR_050115 p.Ala203Gly LB/B rs35101468 -
CLEC11A Q9Y240 VAR_050116 p.Pro104Arg LB/B rs2303688 -
CLEC12A Q5QGZ9 VAR_037669 p.Lys244Gln LB/B rs479499 -
CLEC12B Q2HXU8 VAR_037670 p.Thr6Asn LB/B rs1359082 -
CLEC12B Q2HXU8 VAR_037671 p.Val116Leu LB/B rs637790 -
CLEC16A Q2KHT3 VAR_030288 p.Gly906Glu LB/B rs2241100 -
CLEC18A A5D8T8 VAR_059449 p.Val118Ala LB/B rs2549097 -
CLEC18A A5D8T8 VAR_074610 p.Thr151Met LB/B rs75776403 -

CLEC18A	A5D8T8	VAR_074611	p.Ser339Arg	LB/B	-	-
CLEC1A	Q8NC01	VAR_050106	p.Gly26Ala	LB/B	rs2306894	-
CLEC1B	Q9P126	VAR_031047	p.Ile20Val	LB/B	rs612593	-
CLEC1B	Q9P126	VAR_031048	p.Ser24Pro	LB/B	rs2273986	-
CLEC1B	Q9P126	VAR_031049	p.Ser28Phe	LB/B	rs2273987	-
CLEC1B	Q9P126	VAR_031050	p.Gly64Asp	LB/B	rs583903	-
CLEC2A	Q6UVW9	VAR_029629	p.Gly136Asp	LB/B	rs526680	-
CLEC2D	Q9UHP7	VAR_038172	p.Asn19Lys	LB/B	rs16914640	-
CLEC2D	Q9UHP7	VAR_038173	p.Leu23Val	LB/B	rs3764022	-
CLEC3A	O75596	VAR_021259	p.Gln197Lys	LB/B	rs2072663	-
CLEC3B	P05452	VAR_004189	p.Ala55Ser	LB/B	-	-
CLEC3B	P05452	VAR_004190	p.Val58Met	LB/B	-	-
CLEC3B	P05452	VAR_012318	p.Gly106Ser	LB/B	rs13963	-
CLEC4A	Q9UMR7	VAR_021260	p.His36Leu	LB/B	rs2024301	-
CLEC4D	Q8WXI8	VAR_021261	p.Ser32Gly	LB/B	rs4304840	-
CLEC4F	Q8N1N0	VAR_054429	p.Arg101Gln	LB/B	rs2075221	-
CLEC4F	Q8N1N0	VAR_054430	p.Arg351His	LB/B	rs722896	-
CLEC4F	Q8N1N0	VAR_054431	p.Lys564Arg	LB/B	rs2287101	-
CLEC4M	Q9H2X3	VAR_021957	p.Asp291Asn	LB/B	rs2277998	-
CLEC4M	Q9H2X3	VAR_050107	p.Arg164Gln	LB/B	rs11465376	-
CLEC4M	Q9H2X3	VAR_050108	p.Tyr205Cys	LB/B	rs479448	-
CLEC4M	Q9H2X3	VAR_050109	p.Tyr251Cys	LB/B	rs479448	-
CLEC5A	Q9NY25	VAR_050110	p.Arg141His	LB/B	rs35942193	-
CLEC7A	Q9BXN2	VAR_050111	p.Ile223Ser	LB/B	rs16910527	-
CLEC9A	Q6UXN8	VAR_050112	p.Ala107Gly	LB/B	rs11831360	-
CLGN	O14967	VAR_024400	p.Ala160Ser	LB/B	rs2567241	-
CLGN	O14967	VAR_033776	p.Val290Ile	LB/B	rs2175563	-
CLGN	O14967	VAR_048590	p.Arg352Trp	LB/B	rs12513290	-
CLHC1	Q8NHS4	VAR_039944	p.Ala178Val	LB/B	rs9677948	-
CLHC1	Q8NHS4	VAR_039945	p.Val426Ile	LB/B	rs6716066	-
CLHC1	Q8NHS4	VAR_039946	p.Ala503Val	LB/B	rs14026	-
CLHC1	Q8NHS4	VAR_039947	p.Asp552Tyr	LB/B	rs3186099	-
CLIC2	O15247	VAR_068898	p.His101Gln	LP/P	rs398122917	Mental retardation, X-linked, syndromic, 32 (MRXS32) [MIM:300886]
CLIC3	O95833	VAR_020424	p.Pro38His	LB/B	rs2292923	-
CLIC5	Q9NZA1	VAR_047541	p.Pro257His	LB/B	rs35822882	-
CLIC5	Q9NZA1	VAR_059208	p.Thr114Ala	LB/B	rs723580	-
CLIC6	Q96NY7	VAR_014139	p.Asp632Gly	LB/B	rs3171439	-
CLIP1	P30622	VAR_020398	p.Asp1080Glu	LB/B	rs1129167	-
CLIP1	P30622	VAR_036446	p.Met1213Ile	US	rs1183319975	A breast cancer sample
CLIP1	P30622	VAR_048672	p.Arg780Trp	LB/B	rs3741447	-
CLIP1	P30622	VAR_048673	p.Ser941Pro	LB/B	rs17883517	-
CLIP1	P30622	VAR_048674	p.Ala1224Ser	LB/B	rs17881033	-
CLIP1	P30622	VAR_059206	p.Ser162Pro	LB/B	rs7963597	-
CLIP2	Q9UDT6	VAR_023618	p.Arg977Pro	LB/B	rs2522943	-
CLIP2	Q9UDT6	VAR_055636	p.Asp961Glu	LB/B	rs17145468	-
CLIP3	Q96DZ5	VAR_027962	p.Asp175Val	LB/B	rs17851002	-
CLIP4	Q8N3C7	VAR_048675	p.Arg486Leu	LB/B	rs3100246	-
CLIP4	Q8N3C7	VAR_048676	p.Thr613Pro	LB/B	rs34327508	-

CLK1 P49759 VAR_040409 p.Ser61Phe LB/B rs55989135 -
CLK1 P49759 VAR_040410 p.Arg118Gly LB/B rs56135616 -
CLK1 P49759 VAR_040411 p.Pro307Ser LB/B rs35412475 -
CLK1 P49759 VAR_040412 p.Met440Thr LB/B rs35393352 -
CLK1 P49759 VAR_046551 p.Asn99Asp LB/B rs6735666 -
CLK1 P49759 VAR_051620 p.Glu459Gly LB/B rs12709 -
CLK3 P49761 VAR_040413 p.Arg486Cys LB/B rs975796055 -
CLK3 P49761 VAR_045579 p.Gln607Arg LB/B rs910378995 -
CLK3 P49761 VAR_045580 p.Arg628Trp LB/B rs920443187 -
CLK4 Q9HAZ1 VAR_040414 p.Leu352Phe LB/B rs35272416 -
CLK4 Q9HAZ1 VAR_040415 p.Ile363Val LB/B rs55746655 -
CLLU1 Q5K131 VAR_034663 p.Ser9Phe LB/B rs12580153 -
CLMN Q96JQ2 VAR_050866 p.Pro963Leu LB/B rs10149705 -
CLMP Q9H6B4 VAR_049824 p.Arg69His LB/B rs2276348 -
CLMP Q9H6B4 VAR_069713 p.Val124Asp LP/P rs587776967 Congenital short bowel syndrome (CSBS) [MIM:615237]
CLN3 Q13286 VAR_005131 p.Leu101Pro LP/P rs386833714 Ceroid lipofuscinosis, neuronal, 3 (CLN3) [MIM:204200]
CLN3 Q13286 VAR_005132 p.Leu170Pro LP/P rs386833727 Ceroid lipofuscinosis, neuronal, 3 (CLN3) [MIM:204200]
CLN3 Q13286 VAR_005133 p.Glu295Lys LP/P rs121434286 Ceroid lipofuscinosis, neuronal, 3 (CLN3) [MIM:204200]
CLN3 Q13286 VAR_005134 p.Val330Phe LP/P rs386833744 Ceroid lipofuscinosis, neuronal, 3 (CLN3) [MIM:204200]
CLN3 Q13286 VAR_005135 p.Arg334Cys LP/P rs386833694 Ceroid lipofuscinosis, neuronal, 3 (CLN3) [MIM:204200]
CLN3 Q13286 VAR_005136 p.Arg334His LP/P rs386833695 Ceroid lipofuscinosis, neuronal, 3 (CLN3) [MIM:204200]
CLN3 Q13286 VAR_066892 p.Cys134Arg LP/P rs386833719 Ceroid lipofuscinosis, neuronal, 3 (CLN3) [MIM:204200]
CLN3 Q13286 VAR_066893 p.Gly187Ala LP/P rs386833730 Ceroid lipofuscinosis, neuronal, 3 (CLN3) [MIM:204200]
CLN3 Q13286 VAR_066894 p.Gly189Arg LP/P rs386833731 Ceroid lipofuscinosis, neuronal, 3 (CLN3) [MIM:204200]
CLN5 O75503 VAR_005137 p.Asp230Asn LP/P rs28940280 Ceroid lipofuscinosis, neuronal, 5 (CLN5) [MIM:256731]
CLN5 O75503 VAR_005138 p.Lys319Arg LB/B rs1800209 -
CLN5 O75503 VAR_042700 p.Arg63His LP/P rs104894386 Ceroid lipofuscinosis, neuronal, 5 (CLN5) [MIM:256731]
CLN5 O75503 VAR_042701 p.Tyr209Asp LP/P rs386833981 Ceroid lipofuscinosis, neuronal, 5 (CLN5) [MIM:256731]
CLN5 O75503 VAR_042702 p.Arg63Pro LP/P rs104894386 Ceroid lipofuscinosis, neuronal, 5 (CLN5) [MIM:256731]
CLN5 O75503 VAR_059031 p.Glu219Ala LB/B rs11842935 -
CLN5 O75503 VAR_059032 p.Trp330Cys LP/P rs386833968 Ceroid lipofuscinosis, neuronal, 5 (CLN5) [MIM:256731]
CLN5 O75503 VAR_066895 p.Trp26Arg LB/B rs199727787 -
CLN5 O75503 VAR_066896 p.Cys77Tyr LP/P rs267606738 Ceroid lipofuscinosis, neuronal, 5 (CLN5) [MIM:256731]

CLN5 O75503 VAR_066897 p.Asn143Ser LP/P rs386833975 Ceroid lipofuscinosis, neuronal, 5 (CLN5) [MIM:256731]
CLN5 O75503 VAR_066898 p.Leu149Pro LP/P rs386833976 Ceroid lipofuscinosis, neuronal, 5 (CLN5) [MIM:256731]
CLN5 O75503 VAR_066899 p.Pro156Ser LP/P rs386833977 Ceroid lipofuscinosis, neuronal, 5 (CLN5) [MIM:256731]
CLN5 O75503 VAR_066900 p.Trp158Arg LP/P rs147065248 Ceroid lipofuscinosis, neuronal, 5 (CLN5) [MIM:256731]
CLN5 O75503 VAR_066901 p.Trp158Ser LP/P rs386833978 Ceroid lipofuscinosis, neuronal, 5 (CLN5) [MIM:256731]
CLN5 O75503 VAR_066902 p.Asn193Lys LB/B rs138611001 -
CLN5 O75503 VAR_066903 p.Tyr325Cys LP/P rs148862100 Ceroid lipofuscinosis, neuronal, 5 (CLN5) [MIM:256731]
CLN6 Q9NWW5 VAR_015683 p.Gly123Asp LP/P rs104894484 Ceroid lipofuscinosis, neuronal, 6 (CLN6) [MIM:601780]
CLN6 Q9NWW5 VAR_015686 p.Trp300Arg LP/P rs750937323 Ceroid lipofuscinosis, neuronal, 6 (CLN6) [MIM:601780]
CLN6 Q9NWW5 VAR_021549 p.Arg62His LP/P rs751486476 Ceroid lipofuscinosis, neuronal, 6 (CLN6) [MIM:601780]
CLN6 Q9NWW5 VAR_021550 p.Glu72Gln LB/B rs104894483 -
CLN6 Q9NWW5 VAR_021551 p.Tyr221Ser LP/P rs764571295 Ceroid lipofuscinosis, neuronal, 6 (CLN6) [MIM:601780]
CLN6 Q9NWW5 VAR_021552 p.Met241Thr LP/P rs1555438255 Ceroid lipofuscinosis, neuronal, 6 (CLN6) [MIM:601780]
CLN6 Q9NWW5 VAR_021554 p.Pro299Leu LP/P rs758921701 Ceroid lipofuscinosis, neuronal, 6 (CLN6) [MIM:601780]
CLN6 Q9NWW5 VAR_058436 p.Pro159Leu LP/P rs919850756 Ceroid lipofuscinosis, neuronal, 6 (CLN6) [MIM:601780]
CLN6 Q9NWW5 VAR_058437 p.Tyr221Cys LP/P rs764571295 Ceroid lipofuscinosis, neuronal, 6 (CLN6) [MIM:601780]
CLN6 Q9NWW5 VAR_065834 p.Arg6Thr LP/P rs154774636 Ceroid lipofuscinosis, neuronal, 4A (CLN4A) [MIM:204300]
CLN6 Q9NWW5 VAR_065835 p.Ala34Thr LB/B rs146198681 -
CLN6 Q9NWW5 VAR_065836 p.Leu47Phe LP/P rs154774635 Ceroid lipofuscinosis, neuronal, 4A (CLN4A) [MIM:204300]
CLN6 Q9NWW5 VAR_065837 p.Leu67Pro LP/P rs154774633 Ceroid lipofuscinosis, neuronal, 4A (CLN4A) [MIM:204300]
CLN6 Q9NWW5 VAR_065838 p.Asn77Lys LP/P rs154774641 Ceroid lipofuscinosis, neuronal, 4A (CLN4A) [MIM:204300]
CLN6 Q9NWW5 VAR_065839 p.Arg103Gln LP/P rs154774634 Ceroid lipofuscinosis, neuronal, 4A (CLN4A) [MIM:204300]
CLN6 Q9NWW5 VAR_065840 p.Arg149His LP/P rs154774638 Ceroid lipofuscinosis, neuronal, 4A (CLN4A) [MIM:204300]
CLN6 Q9NWW5 VAR_065841 p.Phe238Thr LP/P - Ceroid lipofuscinosis, neuronal, 4A (CLN4A) [MIM:204300]
CLN6 Q9NWW5 VAR_065842 p.Ser308Thr LB/B rs143578698 -
CLN6 Q9NWW5 VAR_066904 p.Ala12Thr LB/B rs112239768 -
CLN6 Q9NWW5 VAR_066905 p.Gly17Ser US rs763944821 Ceroid lipofuscinosis, neuronal, 6 (CLN6) [MIM:601780]

CLN6 Q9NWW5 VAR_066906 p.Asn90Lys LP/P - Ceroid lipofuscinosis, neuronal, 6 (CLN6) [MIM:601780]
CLN6 Q9NWW5 VAR_066907 p.Ser104Phe LP/P - Ceroid lipofuscinosis, neuronal, 6 (CLN6)
[MIM:601780]
CLN6 Q9NWW5 VAR_066908 p.Arg149Cys LP/P rs747229909 Ceroid lipofuscinosis, neuronal, 6 (CLN6)
[MIM:601780]
CLN6 Q9NWW5 VAR_066909 p.Leu169Pro LP/P rs1344658850 Ceroid lipofuscinosis, neuronal, 6 (CLN6)
[MIM:601780]
CLN6 Q9NWW5 VAR_066910 p.Phe186Ser LP/P - Ceroid lipofuscinosis, neuronal, 6 (CLN6)
[MIM:601780]
CLN6 Q9NWW5 VAR_066911 p.Phe234Leu LP/P rs959199004 Ceroid lipofuscinosis, neuronal, 6 (CLN6)
[MIM:601780]
CLN6 Q9NWW5 VAR_066912 p.Arg252His LP/P rs374681194 Ceroid lipofuscinosis, neuronal, 6 (CLN6)
[MIM:601780]
CLN6 Q9NWW5 VAR_066913 p.Gly259Ser LP/P rs150363441 Ceroid lipofuscinosis, neuronal, 6 (CLN6)
[MIM:601780]
CLN6 Q9NWW5 VAR_066914 p.Pro297Thr LP/P rs1194940137 Ceroid lipofuscinosis, neuronal, 6 (CLN6)
[MIM:601780]
CLN8 Q9UBY8 VAR_013174 p.Arg24Gly LP/P rs104894064 Ceroid lipofuscinosis, neuronal, 8, Northern
epilepsy variant (CLN8NE) [MIM:610003]
CLN8 Q9UBY8 VAR_013175 p.Ala155Val LB/B rs386834128 -
CLN8 Q9UBY8 VAR_026554 p.Leu16Met LP/P rs386834129 Ceroid lipofuscinosis, neuronal, 8 (CLN8)
[MIM:600143]
CLN8 Q9UBY8 VAR_026555 p.Thr170Met LP/P rs188259026 Ceroid lipofuscinosis, neuronal, 8 (CLN8)
[MIM:600143]
CLN8 Q9UBY8 VAR_026556 p.Arg204Cys LP/P rs104894060 Ceroid lipofuscinosis, neuronal, 8 (CLN8)
[MIM:600143]
CLN8 Q9UBY8 VAR_026557 p.Trp263Cys LP/P rs28940569 Ceroid lipofuscinosis, neuronal, 8 (CLN8)
[MIM:600143]
CLN8 Q9UBY8 VAR_031704 p.His92Tyr LB/B rs34030778 -
CLN8 Q9UBY8 VAR_058438 p.Tyr158Cys LP/P rs386834130 Ceroid lipofuscinosis, neuronal, 8 (CLN8)
[MIM:600143]
CLN8 Q9UBY8 VAR_058439 p.Gly237Arg LP/P rs746645358 Ceroid lipofuscinosis, neuronal, 8 (CLN8)
[MIM:600143]
CLN8 Q9UBY8 VAR_060573 p.Ala30Pro LP/P rs137852883 Ceroid lipofuscinosis, neuronal, 8 (CLN8)
[MIM:600143]
CLN8 Q9UBY8 VAR_060575 p.Gln194Arg LP/P rs386834133 Ceroid lipofuscinosis, neuronal, 8 (CLN8)
[MIM:600143]
CLN8 Q9UBY8 VAR_066920 p.Arg70His LP/P rs386834124 Ceroid lipofuscinosis, neuronal, 8 (CLN8)
[MIM:600143]
CLN8 Q9UBY8 VAR_066921 p.Gln76Arg LP/P rs386834125 Ceroid lipofuscinosis, neuronal, 8 (CLN8)
[MIM:600143]
CLN8 Q9UBY8 VAR_066922 p.Ile107Ser LP/P rs386834126 Ceroid lipofuscinosis, neuronal, 8 (CLN8)
[MIM:600143]
CLN8 Q9UBY8 VAR_066923 p.Asn125Ser LB/B rs142269885 -
CLN8 Q9UBY8 VAR_066924 p.His139Tyr LP/P rs386834127 Ceroid lipofuscinosis, neuronal, 8 (CLN8)
[MIM:600143]
CLN8 Q9UBY8 VAR_066926 p.Gly221Ser LP/P rs386834136 Ceroid lipofuscinosis, neuronal, 8 (CLN8)
[MIM:600143]
CLN8 Q9UBY8 VAR_066927 p.Pro229Ala LB/B rs150047904 -

CLN8 Q9UBY8 VAR_066928 p.Glu269Val LP/P rs139003032 Ceroid lipofuscinosis, neuronal, 8 (CLN8) [MIM:600143]
CLN8 Q9UBY8 VAR_075367 p.Arg204Leu LP/P rs386834134 Ceroid lipofuscinosis, neuronal, 8 (CLN8) [MIM:600143]
CLN8 Q9UBY8 VAR_075368 p.Leu207Arg US rs781166361 Ceroid lipofuscinosis, neuronal, 8 (CLN8) [MIM:600143]
CLNK Q7Z7G1 VAR_037984 p.Ser65Gly LB/B rs16869924 -
CLNS1A P54105 VAR_015736 p.Gln20His LB/B - -
CLNS1A P54105 VAR_015737 p.Met218Thr LB/B - -
CLOCK O15516 VAR_029076 p.Leu395Ile LB/B rs6855837 -
CLOCK O15516 VAR_029077 p.His542Arg LB/B rs3762836 -
CLOCK O15516 VAR_040061 p.Ser208Cys LB/B rs34897046 -
CLOCK O15516 VAR_040062 p.Glu380Lys LB/B rs1056478 -
CLP1 Q92989 VAR_070952 p.Arg140His LP/P rs587777616 Pontocerebellar hypoplasia 10 (PCH10) [MIM:615803]
CLPB Q9H078 VAR_048740 p.Arg295Thr LB/B rs7938203 -
CLPB Q9H078 VAR_073397 p.Thr268Met LP/P rs200032855 3-methylglutaconic aciduria 7 (MGCA7) [MIM:616271]
CLPB Q9H078 VAR_073398 p.Tyr272Cys LP/P rs777313457 3-methylglutaconic aciduria 7 (MGCA7) [MIM:616271]
CLPB Q9H078 VAR_073399 p.Arg408Gly LP/P rs144078282 3-methylglutaconic aciduria 7 (MGCA7) [MIM:616271]
CLPB Q9H078 VAR_073400 p.Met411Ile LP/P rs786205137 3-methylglutaconic aciduria 7 (MGCA7) [MIM:616271]
CLPB Q9H078 VAR_073402 p.Cys486Arg LP/P rs886041118 3-methylglutaconic aciduria 7 (MGCA7) [MIM:616271]
CLPB Q9H078 VAR_073403 p.Glu501Lys LP/P rs748915609 3-methylglutaconic aciduria 7 (MGCA7) [MIM:616271]
CLPB Q9H078 VAR_073404 p.Tyr567Cys LP/P rs150857620 3-methylglutaconic aciduria 7 (MGCA7) [MIM:616271]
CLPB Q9H078 VAR_073405 p.Ala591Val LP/P rs748010262 3-methylglutaconic aciduria 7 (MGCA7) [MIM:616271]
CLPB Q9H078 VAR_073406 p.Tyr617Cys LP/P rs786205138 3-methylglutaconic aciduria 7 (MGCA7) [MIM:616271]
CLPB Q9H078 VAR_073407 p.Gly646Val LP/P rs759500860 3-methylglutaconic aciduria 7 (MGCA7) [MIM:616271]
CLPB Q9H078 VAR_073408 p.Ile682Asn LP/P rs886041120 3-methylglutaconic aciduria 7 (MGCA7) [MIM:616271]
CLPP Q16740 VAR_070092 p.Thr145Pro LP/P rs398123033 Perrault syndrome 3 (PRLTS3) [MIM:614129]
CLPP Q16740 VAR_070093 p.Cys147Ser LP/P rs398123034 Perrault syndrome 3 (PRLTS3) [MIM:614129]
CLPP Q16740 VAR_074160 p.Tyr229Asp LP/P - Perrault syndrome 3 (PRLTS3) [MIM:614129]
CLPS P04118 VAR_047105 p.Arg109Cys LB/B rs41270082 -
CLPS P04118 VAR_053040 p.Leu8Pro LB/B rs2766597 -
CLPSL1 A2RUU4 VAR_043560 p.Phe15Ser LB/B rs34109614 -
CLPTM1 O96005 VAR_071064 p.Tyr478Cys LB/B rs140564801 -
CLPTM1L Q96KA5 VAR_042754 p.Lys313Asn US - A breast cancer sample
CLPTM1L Q96KA5 VAR_042755 p.Thr537Met LB/B rs33955038 -
CLPX O76031 VAR_048826 p.Ile488Thr LB/B rs35754835 -
CLPX O76031 VAR_081001 p.Gly298Asp LP/P - Protoporphyria, erythropoietic, 2 (EPP2) [MIM:618015]

CLRN1 P58418 VAR_012241 p.Met120Lys LP/P rs121908141 Usher syndrome 3A (USH3A) [MIM:276902]
CLRN1 P58418 VAR_030345 p.Asn48Lys LP/P rs111033258 Usher syndrome 3A (USH3A) [MIM:276902]
CLRN1 P58418 VAR_030346 p.Leu150Pro LP/P rs121908142 Usher syndrome 3A (USH3A) [MIM:276902]
CLRN1 P58418 VAR_053825 p.Lys7Ile LB/B rs3796241 -
CLRN1 P58418 VAR_054555 p.Cys40Gly LP/P rs121908143 Usher syndrome 3A (USH3A) [MIM:276902]
CLRN1 P58418 VAR_054556 p.Ser105Pro LP/P - Usher syndrome 3A (USH3A) [MIM:276902]
CLRN1 P58418 VAR_066673 p.Pro31Leu LP/P rs374390376 Retinitis pigmentosa 61 (RP61) [MIM:614180]
CLRN1 P58418 VAR_066674 p.Leu154Trp LP/P rs775098953 Retinitis pigmentosa 61 (RP61) [MIM:614180]
CLRN1 P58418 VAR_071434 p.Ile168Asn LP/P - Usher syndrome 3A (USH3A) [MIM:276902]
CLRN2 A0PK11 VAR_053826 p.Leu113Val LB/B rs13147559 -
CLRN2 A0PK11 VAR_053827 p.Ala153Thr LB/B rs2597791 -
CLRN3 Q8NCR9 VAR_053828 p.Phe75Ile LB/B rs35070529 -
CLRN3 Q8NCR9 VAR_069399 p.Ile22Asn LB/B - -
CLSPN Q9HAW4 VAR_023439 p.Asn525Ser LB/B rs7537203 -
CLSPN Q9HAW4 VAR_035674 p.His439Arg US - A breast cancer sample
CLSPN Q9HAW4 VAR_050867 p.Pro892Thr LB/B rs34390044 -
CLSPN Q9HAW4 VAR_050868 p.Ser1280Leu LB/B rs35490896 -
CLSTN1 O94985 VAR_039552 p.Val474Ala LB/B rs17853245 -
CLSTN1 O94985 VAR_039553 p.Ser524Cys LB/B rs17853244 -
CLSTN1 O94985 VAR_039554 p.Pro583Arg LB/B rs17853243 -
CLSTN1 O94985 VAR_039555 p.Pro857His LB/B rs17855572 -
CLSTN1 O94985 VAR_039556 p.Phe870Ser LB/B rs17855573 -
CLSTN1 O94985 VAR_048582 p.Ala332Thr LB/B rs7550295 -
CLSTN2 Q9H4D0 VAR_036112 p.Ser193Ile US - A colorectal cancer sample
CLSTN2 Q9H4D0 VAR_036113 p.Arg765Gln US rs766718816 A colorectal cancer sample
CLSTN2 Q9H4D0 VAR_039557 p.Val366Ile LB/B rs7632885 -
CLSTN2 Q9H4D0 VAR_055615 p.Ile331Thr LB/B rs17348572 -
CLSTN3 Q9BQT9 VAR_036114 p.His874Tyr US - A colorectal cancer sample
CLSTN3 Q9BQT9 VAR_048583 p.Ser209Gly LB/B rs7302230 -
CLTC Q00610 VAR_080722 p.Pro890Leu LP/P rs1555606635 Mental retardation, autosomal dominant 56 (MRD56) [MIM:617854]
CLTC Q00610 VAR_080723 p.Leu1047Pro US rs1555607159 Mental retardation, autosomal dominant 56 (MRD56) [MIM:617854]
CLTC Q00610 VAR_080724 p.Trp1108Arg US - Mental retardation, autosomal dominant 56 (MRD56) [MIM:617854]
CLTCL1 P53675 VAR_055653 p.Pro61Leu LB/B rs3747059 -
CLTCL1 P53675 VAR_055654 p.Lys205Arg LB/B rs5746697 -
CLTCL1 P53675 VAR_055655 p.Tyr279Cys LB/B rs807459 -
CLTCL1 P53675 VAR_055656 p.Glu691Lys LB/B rs1060374 -
CLTCL1 P53675 VAR_055657 p.Lys941Arg LB/B rs35398725 -
CLTCL1 P53675 VAR_055658 p.Arg945His LB/B rs36077768 -
CLTCL1 P53675 VAR_055659 p.Arg1046Cys LB/B rs712952 -
CLTCL1 P53675 VAR_059214 p.Asn1195Ser LB/B rs807547 -
CLTCL1 P53675 VAR_059215 p.Met1316Val LB/B rs1061325 -
CLTCL1 P53675 VAR_059216 p.Ile1394Thr LB/B rs1633399 -
CLTCL1 P53675 VAR_059217 p.Val1592Met LB/B rs2073738 -
CLTCL1 P53675 VAR_059218 p.Arg1620His LB/B rs5748024 -
CLU P10909 VAR_019366 p.Asn317His LB/B rs9331936 -
CLU P10909 VAR_019367 p.Asp328Asn LB/B rs9331938 -

CLU	P10909	VAR_019368	p.Ser396Leu	LB/B	rs13494	-
CLUAP1	Q96AJ1	VAR_050869	p.Ala68Ser	LB/B	rs34115694	-
CLUAP1	Q96AJ1	VAR_050870	p.Arg401Trp	LB/B	rs9790	-
CLUH	O75153	VAR_034008	p.Ala633Val	LB/B	rs11078312	-
CLYBL	Q8N0X4	VAR_032099	p.Asp28Tyr	LB/B	rs17577293	-
CLYBL	Q8N0X4	VAR_032100	p.Val128Ile	LB/B	rs35680839	-
CLYBL	Q8N0X4	VAR_032101	p.Ile241Val	LB/B	rs3783185	-
CMA1	P23946	VAR_011770	p.Gly46Arg	LB/B	rs5246	-
CMA1	P23946	VAR_011771	p.His66Arg	LB/B	rs5247	-
CMA1	P23946	VAR_029190	p.Arg98His	LB/B	rs13306252	-
CMBL	Q96DG6	VAR_036751	p.Tyr155Cys	LB/B	rs35489000	-
CMC2	Q9NRP2	VAR_033816	p.Thr11Ser	LB/B	rs2303217	-
CMPK2	Q5EBM0	VAR_055997	p.Lys433Arg	LB/B	rs6712141	-
CMSS1	Q9BQ75	VAR_033658	p.Val166Ile	LB/B	rs11537816	-
CMSS1	Q9BQ75	VAR_047645	p.Glu138Gly	LB/B	rs11537817	-
CMTM1	Q8IZ96	VAR_053039	p.Ser10Thr	LB/B	rs16956746	-
CMTM2	Q8TAZ6	VAR_022154	p.Ile122Thr	LB/B	rs2290182	-
CMTM6	Q9NX76	VAR_061998	p.Thr91Ala	LB/B	rs35574803	-
CMTR2	Q8IYT2	VAR_039998	p.Leu60Phe	LB/B	rs3096380	-
CMTR2	Q8IYT2	VAR_039999	p.Phe163Tyr	LB/B	rs17853360	-
CMTR2	Q8IYT2	VAR_040000	p.Asn416Ser	LB/B	rs3803704	-
CMTR2	Q8IYT2	VAR_040001	p.Thr608Lys	LB/B	rs3096381	-
CMTR2	Q8IYT2	VAR_040002	p.Phe753Leu	LB/B	rs16970857	-
CMYA5	Q8N3K9	VAR_042471	p.Tyr64Cys	LB/B	rs16877109	-
CMYA5	Q8N3K9	VAR_042472	p.Gln175His	LB/B	rs6895605	-
CMYA5	Q8N3K9	VAR_042473	p.Asp190Gly	LB/B	rs10942901	-
CMYA5	Q8N3K9	VAR_042474	p.Gly349Asp	LB/B	rs1366271	-
CMYA5	Q8N3K9	VAR_042475	p.Gly591Asp	LB/B	rs16877124	-
CMYA5	Q8N3K9	VAR_042476	p.Val1006Ala	LB/B	rs6893869	-
CMYA5	Q8N3K9	VAR_042477	p.Ala1295Val	LB/B	rs4704585	-
CMYA5	Q8N3K9	VAR_042478	p.Ile1309Val	LB/B	rs16877133	-
CMYA5	Q8N3K9	VAR_042479	p.Ala1333Val	LB/B	rs16877135	-
CMYA5	Q8N3K9	VAR_042480	p.Ile1380Val	LB/B	rs13158477	-
CMYA5	Q8N3K9	VAR_042481	p.Ala1567Glu	LB/B	rs1428223	-
CMYA5	Q8N3K9	VAR_042482	p.Ser1599Ala	LB/B	rs1428224	-
CMYA5	Q8N3K9	VAR_042483	p.Leu1669Ser	LB/B	rs1019762	-
CMYA5	Q8N3K9	VAR_042484	p.Ile1713Asn	LB/B	rs16877141	-
CMYA5	Q8N3K9	VAR_042485	p.Ile1721Val	LB/B	rs1428225	-
CMYA5	Q8N3K9	VAR_042486	p.Ala1875Val	LB/B	rs16877147	-
CMYA5	Q8N3K9	VAR_042487	p.Asp1917Gly	LB/B	rs16877150	-
CMYA5	Q8N3K9	VAR_042488	p.Ser1920Gly	LB/B	rs16877151	-
CMYA5	Q8N3K9	VAR_042489	p.Val2262Leu	LB/B	rs6859595	-
CMYA5	Q8N3K9	VAR_042490	p.Lys2383Glu	LB/B	rs7721884	-
CMYA5	Q8N3K9	VAR_042491	p.Thr2693Ile	LB/B	rs28362541	-
CMYA5	Q8N3K9	VAR_042492	p.Lys2906Asn	LB/B	rs2278239	-
CMYA5	Q8N3K9	VAR_042493	p.Gly2935Arg	LB/B	rs2278240	-
CMYA5	Q8N3K9	VAR_042494	p.His3358Gln	LB/B	rs3828611	-
CMYA5	Q8N3K9	VAR_042495	p.Lys3583Glu	LB/B	rs12514461	-
CMYA5	Q8N3K9	VAR_042496	p.Arg3927Gln	LB/B	rs1129770	-

CMYA5	Q8N3K9	VAR_042497	p.Pro4063Leu	LB/B	rs10043986	-
CMYA5	Q8N3K9	VAR_061611	p.Ser651Arg	LB/B	rs57544556	-
CNBD1	Q8NA66	VAR_032859	p.Asp64Asn	LB/B	rs10504829	-
CNBD1	Q8NA66	VAR_032860	p.Gln69Lys	LB/B	rs16894901	-
CNBD1	Q8NA66	VAR_061108	p.Met257Val	LB/B	rs60556175	-
CNBD2	Q96M20	VAR_056853	p.Arg37His	LB/B	rs17347958	-
CNBD2	Q96M20	VAR_056854	p.Thr208Ala	LB/B	rs6142471	-
CNBD2	Q96M20	VAR_056855	p.Pro375Ser	LB/B	rs6060750	-
CNDP1	Q96KN2	VAR_027147	p.Gly6Arg	LB/B	rs11151964	-
CNDP1	Q96KN2	VAR_027149	p.Ile113Val	LB/B	rs4263028	-
CNDP2	Q96KP4	VAR_057154	p.Tyr126His	LB/B	rs2278161	-
CNGA1	P29973	VAR_009295	p.Arg32Gln	LB/B	rs76537883	-
CNGA1	P29973	VAR_009296	p.Asp118Asn	LB/B	rs28642966	-
CNGA1	P29973	VAR_009297	p.Ser320Phe	LP/P	rs62625014	Retinitis pigmentosa 49 (RP49) [MIM:613756]
CNGA1	P29973	VAR_047385	p.Asn122Asp	LB/B	rs28642966	-
CNGA2	Q16280	VAR_036603	p.Arg97His	US	rs561786347	A breast cancer sample
CNGA2	Q16280	VAR_036604	p.Arg399Gln	US	rs150539917	A breast cancer sample
CNGA2	Q16280	VAR_048748	p.Asp118His	LB/B	rs6627455	-
CNGA2	Q16280	VAR_048749	p.Glu663Lys	LB/B	rs714147	-
CNGA2	Q16280	VAR_061107	p.Trp139Leu	LB/B	rs35350051	-
CNGA3	Q16281	VAR_010902	p.Thr153Met	LB/B	rs34314205	-
CNGA3	Q16281	VAR_010903	p.Pro163Leu	LP/P	rs104893612	Achromatopsia 2 (ACHM2) [MIM:216900]
CNGA3	Q16281	VAR_010904	p.Arg283Gln	LP/P	rs104893614	Achromatopsia 2 (ACHM2) [MIM:216900]
CNGA3	Q16281	VAR_010905	p.Arg283Trp	LP/P	rs104893613	Achromatopsia 2 (ACHM2) [MIM:216900]
CNGA3	Q16281	VAR_010906	p.Thr291Arg	LP/P	rs104893616	Achromatopsia 2 (ACHM2) [MIM:216900]
CNGA3	Q16281	VAR_010907	p.Val529Met	LP/P	rs104893619	Achromatopsia 2 (ACHM2) [MIM:216900]
CNGA3	Q16281	VAR_010908	p.Phe547Leu	LP/P	rs104893617	Achromatopsia 2 (ACHM2) [MIM:216900]
CNGA3	Q16281	VAR_010909	p.Gly557Arg	LP/P	rs104893615	Achromatopsia 2 (ACHM2) [MIM:216900]
CNGA3	Q16281	VAR_010910	p.Arg410Trp	LP/P	rs137852608	Achromatopsia 2 (ACHM2) [MIM:216900]
CNGA3	Q16281	VAR_021963	p.Glu198Lys	LB/B	rs2271041	-
CNGA3	Q16281	VAR_047565	p.Pro48Leu	LB/B	rs62156348	-
CNGA3	Q16281	VAR_047566	p.Asp162Val	LP/P	rs747447519	Achromatopsia 2 (ACHM2) [MIM:216900]
CNGA3	Q16281	VAR_047567	p.Tyr181Cys	LP/P	-	Achromatopsia 2 (ACHM2) [MIM:216900]
CNGA3	Q16281	VAR_047568	p.Asn182Tyr	LP/P	-	Achromatopsia 2 (ACHM2) [MIM:216900]
CNGA3	Q16281	VAR_047569	p.Leu186Phe	LP/P	-	Achromatopsia 2 (ACHM2) [MIM:216900]
CNGA3	Q16281	VAR_047570	p.Cys191Tyr	LP/P	rs761554853	Achromatopsia 2 (ACHM2) [MIM:216900]
CNGA3	Q16281	VAR_047571	p.Glu194Lys	LP/P	-	Achromatopsia 2 (ACHM2) [MIM:216900]
CNGA3	Q16281	VAR_047572	p.Arg223Trp	LP/P	rs138958917	Achromatopsia 2 (ACHM2) [MIM:216900]
CNGA3	Q16281	VAR_047573	p.Thr224Arg	LP/P	-	Achromatopsia 2 (ACHM2) [MIM:216900]
CNGA3	Q16281	VAR_047574	p.Glu228Lys	US	rs147415641	Achromatopsia 2 (ACHM2) [MIM:216900]
CNGA3	Q16281	VAR_047575	p.Phe249Ser	LP/P	-	Achromatopsia 2 (ACHM2) [MIM:216900]
CNGA3	Q16281	VAR_047576	p.Asp260Asn	LP/P	rs374258471	Achromatopsia 2 (ACHM2) [MIM:216900]
CNGA3	Q16281	VAR_047577	p.Tyr263Asp	LP/P	rs943314733	Achromatopsia 2 (ACHM2) [MIM:216900]
CNGA3	Q16281	VAR_047578	p.Gly267Asp	LP/P	rs781673067	Achromatopsia 2 (ACHM2) [MIM:216900]
CNGA3	Q16281	VAR_047579	p.Arg277Cys	LP/P	rs104893620	Achromatopsia 2 (ACHM2) [MIM:216900]
CNGA3	Q16281	VAR_047580	p.Arg277His	LP/P	rs778114016	Achromatopsia 2 (ACHM2) [MIM:216900]
CNGA3	Q16281	VAR_047582	p.Ser341Pro	LP/P	rs1227761587	Achromatopsia 2 (ACHM2) [MIM:216900]
CNGA3	Q16281	VAR_047583	p.Thr369Ser	LP/P	rs766637612	Achromatopsia 2 (ACHM2) [MIM:216900]
CNGA3	Q16281	VAR_047584	p.Pro372Ser	LP/P	rs1464167194	Achromatopsia 2 (ACHM2) [MIM:216900]

CNGA3	Q16281	VAR_047585	p.Phe380Ser	LP/P	-	Achromatopsia 2 (ACHM2) [MIM:216900]
CNGA3	Q16281	VAR_047586	p.Ser401Pro	LP/P	rs916035276	Achromatopsia 2 (ACHM2) [MIM:216900]
CNGA3	Q16281	VAR_047587	p.Met406Thr	LP/P	rs1553450734	Achromatopsia 2 (ACHM2) [MIM:216900]
CNGA3	Q16281	VAR_047588	p.Arg427Cys	LP/P	rs141386891	Achromatopsia 2 (ACHM2) [MIM:216900]
CNGA3	Q16281	VAR_047589	p.Arg436Trp	LP/P	rs104893621	Achromatopsia 2 (ACHM2) [MIM:216900]
CNGA3	Q16281	VAR_047590	p.Arg439Trp	LP/P	rs749842881	Achromatopsia 2 (ACHM2) [MIM:216900]
CNGA3	Q16281	VAR_047591	p.Ala469Thr	LP/P	rs117522010	Achromatopsia 2 (ACHM2) [MIM:216900]
CNGA3	Q16281	VAR_047592	p.Asn471Ser	LP/P	rs373954146	Achromatopsia 2 (ACHM2) [MIM:216900]
CNGA3	Q16281	VAR_047593	p.Asp485Val	LP/P	-	Achromatopsia 2 (ACHM2) [MIM:216900]
CNGA3	Q16281	VAR_047594	p.Cys510Ser	LP/P	rs908111816	Achromatopsia 2 (ACHM2) [MIM:216900]
CNGA3	Q16281	VAR_047595	p.Gly513Glu	LP/P	-	Achromatopsia 2 (ACHM2) [MIM:216900]
CNGA3	Q16281	VAR_047596	p.Gly516Glu	LP/P	-	Achromatopsia 2 (ACHM2) [MIM:216900]
CNGA3	Q16281	VAR_047597	p.Ile522Thr	LP/P	-	Achromatopsia 2 (ACHM2) [MIM:216900]
CNGA3	Q16281	VAR_047598	p.Gly525Asp	LP/P	-	Achromatopsia 2 (ACHM2) [MIM:216900]
CNGA3	Q16281	VAR_047599	p.Gly548Arg	LP/P	rs781227859	Achromatopsia 2 (ACHM2) [MIM:216900]
CNGA3	Q16281	VAR_047600	p.Arg563His	LP/P	rs552069173	Achromatopsia 2 (ACHM2) [MIM:216900]
CNGA3	Q16281	VAR_047601	p.Thr565Met	LP/P	rs201747279	Achromatopsia 2 (ACHM2) [MIM:216900]
CNGA3	Q16281	VAR_047602	p.Arg569His	LP/P	rs201782746	Achromatopsia 2 (ACHM2) [MIM:216900]
CNGA3	Q16281	VAR_047603	p.Tyr573Cys	LP/P	-	Achromatopsia 2 (ACHM2) [MIM:216900]
CNGA3	Q16281	VAR_047604	p.Glu590Lys	LP/P	rs763041373	Achromatopsia 2 (ACHM2) [MIM:216900]
CNGA3	Q16281	VAR_047605	p.Glu593Lys	LP/P	rs774676415	Achromatopsia 2 (ACHM2) [MIM:216900]
CNGA3	Q16281	VAR_066860	p.Leu527Met	US	-	-
CNGA3	Q16281	VAR_069398	p.Trp335Cys	LB/B	-	-
CNGA3	Q16281	VAR_071435	p.Asn120Asp	LB/B	rs199859850	-
CNGA3	Q16281	VAR_071436	p.Trp171Cys	LP/P	rs762773298	Achromatopsia 2 (ACHM2) [MIM:216900]
CNGA3	Q16281	VAR_071438	p.Arg223Gln	LP/P	rs762668060	Achromatopsia 2 (ACHM2) [MIM:216900]
CNGA3	Q16281	VAR_071439	p.Thr224Ile	LP/P	-	-
CNGA3	Q16281	VAR_071440	p.Thr247Met	LB/B	rs148616345	-
CNGA3	Q16281	VAR_071441	p.Pro258Arg	LP/P	-	-
CNGA3	Q16281	VAR_071442	p.Arg274Lys	LP/P	-	Achromatopsia 2 (ACHM2) [MIM:216900]
CNGA3	Q16281	VAR_071443	p.Leu278Pro	LP/P	rs763421555	Achromatopsia 2 (ACHM2) [MIM:216900]
CNGA3	Q16281	VAR_071444	p.Phe322Ser	LP/P	-	Achromatopsia 2 (ACHM2) [MIM:216900]
CNGA3	Q16281	VAR_071445	p.Phe330Ser	LP/P	-	-
CNGA3	Q16281	VAR_071446	p.Ser334Phe	LP/P	-	-
CNGA3	Q16281	VAR_071447	p.Arg436Gln	LP/P	rs767083685	Achromatopsia 2 (ACHM2) [MIM:216900]
CNGA3	Q16281	VAR_071448	p.Asp533His	LP/P	rs775332304	-
CNGA3	Q16281	VAR_071450	p.Ser570Asn	LP/P	-	-
CNGA3	Q16281	VAR_071451	p.Arg646His	LB/B	rs141577844	-
CNGA3	Q16281	VAR_075493	p.Ala323Asp	LP/P	-	Achromatopsia 2 (ACHM2) [MIM:216900]
CNGA4	Q8IV77	VAR_038480	p.Glu553Val	LB/B	rs325706	-
CNGB1	Q14028	VAR_058691	p.Arg100His	LB/B	rs13336595	-
CNGB1	Q14028	VAR_059225	p.Leu479Ile	LB/B	rs2303783	-
CNGB1	Q14028	VAR_059226	p.Val535Ala	LB/B	rs12927214	-
CNGB1	Q14028	VAR_059227	p.Asn731Lys	LB/B	rs376270	-
CNGB1	Q14028	VAR_059228	p.Leu745Ile	LB/B	rs10459809	-
CNGB1	Q14028	VAR_059229	p.Lys911Arg	LB/B	rs2303785	-
CNGB1	Q14028	VAR_059230	p.Ala961Ser	LB/B	rs16942445	-
CNGB1	Q14028	VAR_060491	p.Gly993Val	LP/P	rs121918532	Retinitis pigmentosa 45 (RP45) [MIM:613767]
CNGB3	Q9NQW8	VAR_018109	p.Cys234Trp	LB/B	rs6471482	-

CNGB3 Q9NQW8 VAR_018110 p.Thr298Pro LB/B rs4961206 -
CNGB3 Q9NQW8 VAR_018111 p.Ser435Phe LP/P rs121918344 Achromatopsia 3 (ACHM3) [MIM:262300]
CNGB3 Q9NQW8 VAR_018112 p.Glu755Gly LB/B rs3735972 -
CNGB3 Q9NQW8 VAR_024418 p.Ile307Val LB/B rs13265557 -
CNGB3 Q9NQW8 VAR_025524 p.Arg203Gln LB/B rs16916632 -
CNGB3 Q9NQW8 VAR_025525 p.Pro750Ser LB/B rs3735971 -
CNGB3 Q9NQW8 VAR_047606 p.Arg25His LB/B rs141098074 -
CNGB3 Q9NQW8 VAR_047607 p.Asn27Ser LB/B rs35807406 -
CNGB3 Q9NQW8 VAR_047608 p.Gly107Arg US rs146688972 Achromatopsia 3 (ACHM3) [MIM:262300]
CNGB3 Q9NQW8 VAR_047609 p.Lys148Glu LP/P rs369138501 Achromatopsia 3 (ACHM3) [MIM:262300]
CNGB3 Q9NQW8 VAR_047610 p.Ser156Phe LP/P rs139207764 Achromatopsia 3 (ACHM3) [MIM:262300]
CNGB3 Q9NQW8 VAR_047611 p.Glu199Lys US rs114305748 Achromatopsia 3 (ACHM3) [MIM:262300]
CNGB3 Q9NQW8 VAR_047612 p.Pro309Leu LP/P rs1554612145 Achromatopsia 3 (ACHM3) [MIM:262300]
CNGB3 Q9NQW8 VAR_047613 p.Arg403Gln US rs147876778 -
CNGB3 Q9NQW8 VAR_047614 p.Met466Thr US rs35010099 Achromatopsia 3 (ACHM3) [MIM:262300]
CNGB3 Q9NQW8 VAR_047615 p.Tyr469Asp LP/P rs35365413 Stargardt disease 1 (STGD1) [MIM:248200]
CNGB3 Q9NQW8 VAR_047616 p.Asp494Asn US - Achromatopsia 3 (ACHM3) [MIM:262300]
CNGB3 Q9NQW8 VAR_047617 p.Asp513Tyr US rs765884344 Achromatopsia 3 (ACHM3) [MIM:262300]
CNGB3 Q9NQW8 VAR_047618 p.Phe525Asn LP/P - Achromatopsia 3 (ACHM3) [MIM:262300]
CNGB3 Q9NQW8 VAR_047619 p.Gly558Cys LP/P rs749413012 Achromatopsia 3 (ACHM3) [MIM:262300]
CNGB3 Q9NQW8 VAR_047620 p.Leu595Phe LP/P rs1554604849 Achromatopsia 3 (ACHM3) [MIM:262300]
CNGB3 Q9NQW8 VAR_047621 p.Thr672Pro US - Achromatopsia 3 (ACHM3) [MIM:262300]
CNIH4 Q9P003 VAR_048830 p.Ala3Gly LB/B rs12123896 -
CNKSR1 Q969H4 VAR_057790 p.Arg662Trp LB/B rs17163640 -
CNKSR2 Q8WXI2 VAR_035681 p.Arg46His US rs771705122 A colorectal cancer sample
CNMD O75829 VAR_024413 p.Val175Ile LB/B rs7330220 -
CNMD O75829 VAR_048719 p.Phe116Leu LB/B rs3742298 -
CNNM1 Q9NRU3 VAR_057737 p.Arg819Gln LB/B rs2298316 -
CNNM2 Q9H8M5 VAR_065259 p.Arg38Gln LB/B rs76057237 -
CNNM2 Q9H8M5 VAR_065260 p.Thr568Ile LP/P rs387906975 Hypomagnesemia 6 (HOMG6) [MIM:613882]
CNNM2 Q9H8M5 VAR_073848 p.Glu122Lys LP/P rs786205909 Hypomagnesemia, seizures, and mental retardation 1 (HOMGSMR1) [MIM:616418]
CNNM2 Q9H8M5 VAR_073849 p.Ser269Trp LP/P rs794726858 Hypomagnesemia, seizures, and mental retardation 1 (HOMGSMR1) [MIM:616418]
CNNM2 Q9H8M5 VAR_073850 p.Leu330Phe LP/P - Hypomagnesemia, seizures, and mental retardation 1 (HOMGSMR1) [MIM:616418]
CNNM2 Q9H8M5 VAR_073851 p.Glu357Lys LP/P rs786205910 Hypomagnesemia, seizures, and mental retardation 1 (HOMGSMR1) [MIM:616418]
CNNM4 Q6P4Q7 VAR_033365 p.Gly126Arg LB/B rs17855817 -
CNNM4 Q6P4Q7 VAR_035946 p.Val134Leu US - A breast cancer sample
CNNM4 Q6P4Q7 VAR_058319 p.Ser196Pro LP/P - Jalili syndrome (JALIS) [MIM:217080]
CNNM4 Q6P4Q7 VAR_058320 p.Ser200Tyr LP/P rs79424354 Jalili syndrome (JALIS) [MIM:217080]
CNNM4 Q6P4Q7 VAR_058321 p.Arg236Gln LP/P rs75267011 Jalili syndrome (JALIS) [MIM:217080]
CNNM4 Q6P4Q7 VAR_058322 p.Leu324Pro LP/P rs74552543 Jalili syndrome (JALIS) [MIM:217080]
CNOT1 A5YKK6 VAR_038254 p.Asp603Ala LB/B rs17854028 -
CNOT1 A5YKK6 VAR_083066 p.Arg535Cys LP/P rs1567417422 Holoprosencephaly 12 with or without pancreatic agenesis (HPE12) [MIM:618500]
CNOT1 A5YKK6 VAR_084314 p.Gln642Glu US - Vissers-Bodmer syndrome (VIBOS) [MIM:619033]
CNOT1 A5YKK6 VAR_084315 p.Glu897Lys LP/P - Vissers-Bodmer syndrome (VIBOS) [MIM:619033]

CNOT1 A5YKK6 VAR_084316 p.Arg900Cys LP/P - Vissers-Bodmer syndrome (VIBOS) [MIM:619033]
CNOT1 A5YKK6 VAR_084317 p.Thr1038Ile US - Vissers-Bodmer syndrome (VIBOS) [MIM:619033]
CNOT1 A5YKK6 VAR_084318 p.Val1089Leu US - Vissers-Bodmer syndrome (VIBOS) [MIM:619033]
CNOT1 A5YKK6 VAR_084319 p.Leu1148Pro LP/P - Vissers-Bodmer syndrome (VIBOS) [MIM:619033]
CNOT1 A5YKK6 VAR_084320 p.Asp1188Gly LP/P - Vissers-Bodmer syndrome (VIBOS) [MIM:619033]
CNOT1 A5YKK6 VAR_084321 p.Lys1241Arg LP/P - Vissers-Bodmer syndrome (VIBOS) [MIM:619033]
CNOT1 A5YKK6 VAR_084322 p.Thr1419Ala LP/P rs1038725612 Vissers-Bodmer syndrome (VIBOS) [MIM:619033]
CNOT1 A5YKK6 VAR_084323 p.Phe1428Ser LP/P - Vissers-Bodmer syndrome (VIBOS) [MIM:619033]
CNOT1 A5YKK6 VAR_084324 p.Arg1478Cys LP/P rs1567396193 Vissers-Bodmer syndrome (VIBOS) [MIM:619033]
CNOT1 A5YKK6 VAR_084325 p.Gln1494His LP/P - Vissers-Bodmer syndrome (VIBOS) [MIM:619033]
CNOT1 A5YKK6 VAR_084326 p.Tyr1572Asp LP/P - Vissers-Bodmer syndrome (VIBOS) [MIM:619033]
CNOT1 A5YKK6 VAR_084327 p.Asn2216Ser LP/P rs1184460054 Vissers-Bodmer syndrome (VIBOS) [MIM:619033]
CNOT10 Q9H9A5 VAR_037957 p.Pro736Ser LB/B rs17849684 -
CNOT10 Q9H9A5 VAR_053982 p.Thr348Ser LB/B rs11558687 -
CNOT2 Q9NZN8 VAR_048750 p.Ala460Thr LB/B rs11178192 -
CNOT3 O75175 VAR_083405 p.Glu20Gln LP/P - Intellectual developmental disorder with speech delay, autism and dysmorphic facies (IDDSADF) [MIM:618672]
CNOT3 O75175 VAR_083406 p.Leu48Val LP/P - Intellectual developmental disorder with speech delay, autism and dysmorphic facies (IDDSADF) [MIM:618672]
CNOT3 O75175 VAR_083407 p.Lys119Glu US - Intellectual developmental disorder with speech delay, autism and dysmorphic facies (IDDSADF) [MIM:618672]
CNOT3 O75175 VAR_083408 p.Glu147Lys US - Intellectual developmental disorder with speech delay, autism and dysmorphic facies (IDDSADF) [MIM:618672]
CNOT3 O75175 VAR_083409 p.Arg188Cys LP/P - Intellectual developmental disorder with speech delay, autism and dysmorphic facies (IDDSADF) [MIM:618672]
CNOT3 O75175 VAR_083410 p.Arg188His LP/P - Intellectual developmental disorder with speech delay, autism and dysmorphic facies (IDDSADF) [MIM:618672]
CNOT3 O75175 VAR_083413 p.Arg697Gln US - Intellectual developmental disorder with speech delay, autism and dysmorphic facies (IDDSADF) [MIM:618672]
CNOT4 O95628 VAR_027833 p.Ala7Gly LB/B rs17480616 -
CNOT8 Q9UFF9 VAR_048751 p.Leu32Pro LB/B rs1139980 -
CNOT9 Q92600 VAR_042429 p.Ile143Thr LB/B - -
CNP P09543 VAR_033746 p.Gln207Arg LB/B rs34353668 -
CNPPD1 Q9BV87 VAR_022825 p.Ile262Thr LB/B rs1043160 -
CNPPD1 Q9BV87 VAR_024298 p.Leu292Pro LB/B rs1127102 -
CNPPD1 Q9BV87 VAR_024299 p.Pro366Leu LB/B rs17655123 -
CNPPD1 Q9BV87 VAR_024300 p.Leu383Pro LB/B rs4674361 -
CNPPD1 Q9BV87 VAR_056769 p.Arg344Thr LB/B rs1043161 -
CNPY3 Q9BT09 VAR_037731 p.Met145Ile LB/B rs1063252 -
CNPY3 Q9BT09 VAR_037732 p.Ser231Ile LB/B rs9471969 -
CNPY3 Q9BT09 VAR_080491 p.Gly125Arg US rs1554292759 Developmental and epileptic encephalopathy 60 (DEE60) [MIM:617929]
CNPY4 Q8N129 VAR_062216 p.Gly168Arg LB/B rs60551236 -
CNR2 P34972 VAR_029209 p.His316Tyr LB/B rs2229579 -
CNR2 P34972 VAR_054310 p.Gln63Arg LB/B rs2501432 -
CNST Q6PJW8 VAR_032530 p.Ser28Asn LB/B rs35286882 -

CNST	Q6PJW8	VAR_032531	p.Leu87Ser	LB/B	rs6702823	-
CNST	Q6PJW8	VAR_032532	p.Arg399Cys	LB/B	rs12075111	-
CNST	Q6PJW8	VAR_056762	p.Gln183Arg	LB/B	rs12091148	-
CNTD1	Q8N815	VAR_037708	p.Thr145Pro	LB/B	rs12947820	-
CNTF	P26441	VAR_013924	p.His182Arg	LB/B	rs6266	-
CNTF	P26441	VAR_033777	p.Asn49Ser	LB/B	rs17152779	-
CNTLN	Q9NXG0	VAR_025608	p.Arg562Cys	LB/B	rs3808782	-
CNTLN	Q9NXG0	VAR_025609	p.Thr695Ile	LB/B	rs7035276	-
CNTLN	Q9NXG0	VAR_025610	p.Thr1376Ala	LB/B	rs2499057	-
CNTLN	Q9NXG0	VAR_056840	p.Thr284Ala	LB/B	rs3808795	-
CNTLN	Q9NXG0	VAR_056841	p.Glu291Asp	LB/B	rs3808794	-
CNTN1	Q12860	VAR_011722	p.Val798Leu	LB/B	rs1056020	-
CNTN1	Q12860	VAR_035506	p.Pro794His	US	-	A colorectal cancer sample
CNTN1	Q12860	VAR_049866	p.Glu824Gly	LB/B	rs11553341	-
CNTN2	Q02246	VAR_021918	p.Ala145Thr	LB/B	rs2275697	-
CNTN2	Q02246	VAR_021919	p.Arg657Trp	LB/B	rs2305276	-
CNTN2	Q02246	VAR_029129	p.Pro366Leu	LB/B	rs2229866	-
CNTN2	Q02246	VAR_049867	p.Val1024Ile	LB/B	rs17416074	-
CNTN3	Q9P232	VAR_019906	p.Ser630Asn	LB/B	rs626578	-
CNTN3	Q9P232	VAR_056042	p.Asn708Ser	LB/B	rs626578	-
CNTN4	Q8IWV2	VAR_035507	p.Thr176Pro	US	-	A colorectal cancer sample
CNTN4	Q8IWV2	VAR_035508	p.Lys420Asn	US	-	A colorectal cancer sample
CNTN5	O94779	VAR_019907	p.Ser23Ala	LB/B	rs10790978	-
CNTN5	O94779	VAR_019908	p.Leu70Arg	LB/B	rs7125822	-
CNTN5	O94779	VAR_019909	p.Asn81Ser	LB/B	rs10893933	-
CNTN5	O94779	VAR_019910	p.Ile530Val	LB/B	rs11223168	-
CNTN5	O94779	VAR_019911	p.Tyr1065Phe	LB/B	rs1944169	-
CNTN5	O94779	VAR_019912	p.Ser1079Thr	LB/B	rs1216183	-
CNTN5	O94779	VAR_033610	p.Met1094Val	LB/B	rs35208161	-
CNTN6	Q9UQ52	VAR_019913	p.Ala440Ser	LB/B	rs265771	-
CNTN6	Q9UQ52	VAR_033611	p.Phe150Ser	LB/B	rs6808056	-
CNTN6	Q9UQ52	VAR_035509	p.Thr108Ala	US	-	A breast cancer sample
CNTN6	Q9UQ52	VAR_035510	p.Ser585Cys	US	-	A breast cancer sample
CNTN6	Q9UQ52	VAR_065744	p.Arg303Gln	LB/B	rs41293401	-
CNTN6	Q9UQ52	VAR_065745	p.Phe314Val	LB/B	rs774763830	-
CNTN6	Q9UQ52	VAR_065746	p.Glu954Val	LB/B	rs1447631177	-
CNTNAP1	P78357	VAR_050267	p.Val522Leu	LB/B	rs35437096	-
CNTNAP1	P78357	VAR_078818	p.Cys323Arg	LP/P	rs768554986	Lethal congenital contracture syndrome 7 (LCCS7) [MIM:616286]
CNTNAP1	P78357	VAR_078819	p.Arg388Pro	LP/P	rs779027563	Neuropathy, congenital hypomyelinating, 3 (CHN3) [MIM:618186]
CNTNAP1	P78357	VAR_078823	p.Arg764Cys	LP/P	rs761805324	Neuropathy, congenital hypomyelinating, 3 (CHN3) [MIM:618186]
CNTNAP1	P78357	VAR_081766	p.Pro50Gln	LP/P	-	Neuropathy, congenital hypomyelinating, 3 (CHN3) [MIM:618186]
CNTNAP1	P78357	VAR_081767	p.Leu212Pro	LP/P	rs1567969825	Neuropathy, congenital hypomyelinating, 3 (CHN3) [MIM:618186]
CNTNAP1	P78357	VAR_081770	p.Arg714Pro	LP/P	-	Neuropathy, congenital hypomyelinating, 3 (CHN3) [MIM:618186]

CNTNAP2 Q9UHC6 VAR_046227 p.Arg114Gln LB/B rs189731792 -
CNTNAP2 Q9UHC6 VAR_046228 p.Thr218Met LB/B rs771028883 -
CNTNAP2 Q9UHC6 VAR_046229 p.Leu226Met LB/B rs372345438 -
CNTNAP2 Q9UHC6 VAR_046230 p.Arg283Cys LB/B rs794727802 -
CNTNAP2 Q9UHC6 VAR_046231 p.Ser382Asn LB/B rs371839994 -
CNTNAP2 Q9UHC6 VAR_046232 p.Asn407Ser LB/B rs143877693 -
CNTNAP2 Q9UHC6 VAR_046233 p.Asn418Asp LB/B rs772179690 -
CNTNAP2 Q9UHC6 VAR_046234 p.Glu680Lys LB/B rs368905425 -
CNTNAP2 Q9UHC6 VAR_046235 p.Pro699Gln LB/B rs764412489 -
CNTNAP2 Q9UHC6 VAR_046236 p.Tyr716Cys LB/B rs760930032 -
CNTNAP2 Q9UHC6 VAR_046237 p.Gly731Ser LB/B rs369867547 -
CNTNAP2 Q9UHC6 VAR_046238 p.Gly779Asp LB/B rs200413148 -
CNTNAP2 Q9UHC6 VAR_046239 p.Ile869Thr LB/B rs121908445 -
CNTNAP2 Q9UHC6 VAR_046240 p.Arg906His LB/B rs759801195 -
CNTNAP2 Q9UHC6 VAR_046241 p.Asp1038Asn LB/B rs144003410 -
CNTNAP2 Q9UHC6 VAR_046242 p.Val1102Ala LB/B rs111599875 -
CNTNAP2 Q9UHC6 VAR_046243 p.Ser1114Gly LB/B rs983036503 -
CNTNAP2 Q9UHC6 VAR_046244 p.Arg1119His LB/B rs774709566 -
CNTNAP2 Q9UHC6 VAR_046245 p.Asp1129His LB/B rs781236853 -
CNTNAP2 Q9UHC6 VAR_046246 p.Ala1227Thr LB/B rs761684414 -
CNTNAP2 Q9UHC6 VAR_046247 p.Ile1253Thr LB/B rs767821521 -
CNTNAP2 Q9UHC6 VAR_046248 p.Thr1278Ile LB/B rs760047247 -
CNTNAP3 Q9BZ76 VAR_046710 p.Ala628Ser LB/B rs1758272 -
CNTNAP3 Q9BZ76 VAR_046711 p.Arg845His LB/B rs7852039 -
CNTNAP4 Q9C0A0 VAR_050268 p.Gln513His LB/B rs6564343 -
CNTNAP4 Q9C0A0 VAR_050269 p.Asp1155Glu LB/B rs7202925 -
CNTNAP4 Q9C0A0 VAR_050270 p.Asn1300Ser LB/B rs34198820 -
CNTNAP4 Q9C0A0 VAR_061371 p.Leu276Val LB/B rs34251012 -
CNTNAP4 Q9C0A0 VAR_061372 p.Gln786Arg LB/B rs12933808 -
CNTNAP5 Q8WYK1 VAR_038518 p.Ser452Leu LB/B rs17727261 -
CNTNAP5 Q8WYK1 VAR_038519 p.Thr1195Met LB/B rs34165507 -
CNTRL Q7Z7A1 VAR_039559 p.Val56Ile LB/B rs10818503 -
CNTRL Q7Z7A1 VAR_039560 p.Pro216Leu LB/B rs10818504 -
CNTRL Q7Z7A1 VAR_039561 p.Ala889Thr LB/B rs17292952 -
CNTRL Q7Z7A1 VAR_061622 p.Met1146Val LB/B rs35342437 -
CNTROB Q8N137 VAR_024787 p.Pro578Gln LB/B rs11650083 -
CNTROB Q8N137 VAR_050877 p.Arg439Gln LB/B rs35421969 -
COA3 Q9Y2R0 VAR_084180 p.Tyr72Cys US - Mitochondrial complex IV deficiency, nuclear type 14 (MC4DN14) [MIM:619058]
COA5 Q86WW8 VAR_065499 p.Ala53Pro LP/P rs387907099 Mitochondrial complex IV deficiency, nuclear type 9 (MC4DN9) [MIM:616500]
COA6 Q5JTJ3 VAR_075046 p.Trp59Cys LP/P rs1558123786 Mitochondrial complex IV deficiency, nuclear type 13 (MC4DN13) [MIM:616501]
COA6 Q5JTJ3 VAR_075047 p.Trp66Arg LP/P rs875989827 Mitochondrial complex IV deficiency, nuclear type 13 (MC4DN13) [MIM:616501]
COA7 Q96BR5 VAR_031401 p.Lys219Arg LB/B rs443751 -
COA7 Q96BR5 VAR_082218 p.Asp6Gly LP/P rs780572767 Spinocerebellar ataxia, autosomal recessive, with axonal neuropathy 3 (SCAN3) [MIM:618387]

COA7 Q96BR5 VAR_082219 p.Arg39Trp US rs768084335 Spinocerebellar ataxia, autosomal recessive, with axonal neuropathy 3 (SCAN3) [MIM:618387]
COA7 Q96BR5 VAR_082220 p.Tyr137Cys LP/P rs961876891 Spinocerebellar ataxia, autosomal recessive, with axonal neuropathy 3 (SCAN3) [MIM:618387]
COA7 Q96BR5 VAR_082221 p.Ser149Ile US rs1558102448 Spinocerebellar ataxia, autosomal recessive, with axonal neuropathy 3 (SCAN3) [MIM:618387]
COA8 Q96IL0 VAR_023000 p.Pro27Ala LB/B rs2274268 -
COA8 Q96IL0 VAR_033745 p.Asn88Ser LB/B rs35960830 -
COA8 Q96IL0 VAR_082030 p.Phe118Ser US rs587777786 Mitochondrial complex IV deficiency, nuclear type 17 (MC4DN17) [MIM:619061]
COASY Q13057 VAR_030299 p.Ser55Tyr LB/B rs615942 -
COASY Q13057 VAR_070975 p.Arg499Cys LP/P rs140709867 Neurodegeneration with brain iron accumulation 6 (NBIA6) [MIM:615643]
COASY Q13057 VAR_082223 p.Ala214Val US - Neurodegeneration with brain iron accumulation 6 (NBIA6) [MIM:615643]
COBL O75128 VAR_029043 p.Asp577Ala LB/B rs10230120 -
COBL O75128 VAR_029044 p.Val607Ile LB/B rs2240090 -
COBL O75128 VAR_029045 p.Asp927Asn LB/B rs17134128 -
COBL O75128 VAR_029046 p.Ala1015Pro LB/B rs17134127 -
COBL O75128 VAR_050894 p.Pro526Leu LB/B rs17656599 -
COBL O75128 VAR_050895 p.His919Gln LB/B rs2240089 -
COCH O43405 VAR_008532 p.Pro51Ser LP/P rs28938175 Deafness, autosomal dominant, 9 (DFNA9) [MIM:601369]
COCH O43405 VAR_008533 p.Val66Gly LP/P rs121908927 Deafness, autosomal dominant, 9 (DFNA9) [MIM:601369]
COCH O43405 VAR_008534 p.Gly88Glu LP/P rs121908928 Deafness, autosomal dominant, 9 (DFNA9) [MIM:601369]
COCH O43405 VAR_008535 p.Ile109Asn LP/P rs121908930 Deafness, autosomal dominant, 9 (DFNA9) [MIM:601369]
COCH O43405 VAR_008536 p.Trp117Arg LP/P rs121908929 Deafness, autosomal dominant, 9 (DFNA9) [MIM:601369]
COCH O43405 VAR_011925 p.Thr352Ser LB/B rs1045644 -
COCH O43405 VAR_011926 p.Pro532Ser LB/B rs1801963 -
COCH O43405 VAR_017175 p.Ala119Thr LP/P rs121908931 Deafness, autosomal dominant, 9 (DFNA9) [MIM:601369]
COCH O43405 VAR_022259 p.Gly135Arg LB/B rs28400035 -
COCH O43405 VAR_022260 p.Asp281Asn LB/B rs28362775 -
COCH O43405 VAR_022261 p.Ile402Val LB/B rs28362778 -
COCH O43405 VAR_050896 p.Glu518Gly LB/B rs17097468 -
COCH O43405 VAR_070034 p.Cys162Tyr LP/P - Deafness, autosomal dominant, 9 (DFNA9) [MIM:601369]
COCH O43405 VAR_072249 p.Gly87Val LP/P - Deafness, autosomal dominant, 9 (DFNA9) [MIM:601369]
COCH O43405 VAR_072250 p.Gly87Trp LP/P - Deafness, autosomal dominant, 9 (DFNA9) [MIM:601369]
COCH O43405 VAR_072251 p.Ile109Thr LP/P rs121908930 Deafness, autosomal dominant, 9 (DFNA9) [MIM:601369]
COCH O43405 VAR_072252 p.Met512Thr LP/P rs121908934 Deafness, autosomal dominant, 9 (DFNA9) [MIM:601369]

COCH O43405 VAR_072253 p.Phe527Cys LP/P - Deafness, autosomal dominant, 9 (DFNA9) [MIM:601369]
COCH O43405 VAR_072254 p.Cys542Tyr LP/P rs121908932 Deafness, autosomal dominant, 9 (DFNA9) [MIM:601369]
COCH O43405 VAR_079876 p.Gly38Asp LP/P - Deafness, autosomal dominant, 9 (DFNA9) [MIM:601369]
COG1 Q8WTW3 VAR_020415 p.Asn392Ser LB/B rs1026128 -
COG1 Q8WTW3 VAR_048756 p.Tyr744Cys LB/B rs7208207 -
COG1 Q8WTW3 VAR_059231 p.Met357Leu LB/B rs4375725 -
COG2 Q14746 VAR_029274 p.Asn304Lys LB/B rs6681346 -
COG2 Q14746 VAR_048757 p.Arg288His LB/B rs34796217 -
COG2 Q14746 VAR_048758 p.Val589Ile LB/B rs34109129 -
COG2 Q14746 VAR_078769 p.Trp634Gly LP/P rs1085307117 Congenital disorder of glycosylation 2Q (CDG2Q) [MIM:617395]
COG3 Q96JB2 VAR_036454 p.Arg620Cys US rs747042102 A breast cancer sample
COG3 Q96JB2 VAR_055663 p.Asn747Ser LB/B rs2274285 -
COG4 Q9H9E3 VAR_058009 p.Thr158Ile LB/B rs3931036 -
COG4 Q9H9E3 VAR_063767 p.Arg729Trp LP/P - Congenital disorder of glycosylation 2J (CDG2J) [MIM:613489]
COG4 Q9H9E3 VAR_081564 p.Gly512Arg LP/P rs1555575860 Saul-Wilson syndrome (SWILS) [MIM:618150]
COG5 Q9UP83 VAR_039142 p.Phe330Leu LB/B rs2269970 -
COG5 Q9UP83 VAR_039143 p.Ser558Pro LB/B - -
COG5 Q9UP83 VAR_039182 p.Ile365Val LB/B rs34087251 -
COG5 Q9UP83 VAR_055664 p.His452Arg LB/B rs35393416 -
COG6 Q9Y2V7 VAR_048759 p.Ala10Thr LB/B rs3812882 -
COG6 Q9Y2V7 VAR_048760 p.Cys32Ser LB/B rs3812883 -
COG6 Q9Y2V7 VAR_048761 p.His300Tyr LB/B rs34555836 -
COG6 Q9Y2V7 VAR_061110 p.Met447Thr LB/B rs41286961 -
COG6 Q9Y2V7 VAR_068240 p.Gly549Val LP/P rs387906959 Congenital disorder of glycosylation 2L (CDG2L) [MIM:614576]
COG7 P83436 VAR_048762 p.Thr605Met LB/B rs16940094 -
COG8 Q96MW5 VAR_047655 p.Leu517Arg LB/B rs3027 -
COL10A1 Q03692 VAR_001838 p.Gly18Glu LP/P rs111033551 Schmid type metaphyseal chondrodysplasia (SMCD) [MIM:156500]
COL10A1 Q03692 VAR_001839 p.Gly18Arg LP/P rs111033550 Schmid type metaphyseal chondrodysplasia (SMCD) [MIM:156500]
COL10A1 Q03692 VAR_001840 p.Gly545Arg LB/B rs2228547 -
COL10A1 Q03692 VAR_001841 p.Cys591Arg LP/P rs111033546 Schmid type metaphyseal chondrodysplasia (SMCD) [MIM:156500]
COL10A1 Q03692 VAR_001842 p.Gly595Glu LP/P rs111033553 Schmid type metaphyseal chondrodysplasia (SMCD) [MIM:156500]
COL10A1 Q03692 VAR_001843 p.Tyr597His LP/P - Schmid type metaphyseal chondrodysplasia (SMCD) [MIM:156500]
COL10A1 Q03692 VAR_001844 p.Tyr598Asp LP/P rs111033544 Schmid type metaphyseal chondrodysplasia (SMCD) [MIM:156500]
COL10A1 Q03692 VAR_001845 p.Ser600Pro LP/P rs111033555 Schmid type metaphyseal chondrodysplasia (SMCD) [MIM:156500]
COL10A1 Q03692 VAR_001846 p.Leu614Pro LP/P rs111033545 Schmid type metaphyseal chondrodysplasia (SMCD) [MIM:156500]

COL10A1 Q03692 VAR_001847 p.Asn617Lys LP/P - Schmid type metaphyseal chondrodysplasia (SMCD) [MIM:156500]
COL10A1 Q03692 VAR_001848 p.Gly618Val LP/P - Schmid type metaphyseal chondrodysplasia (SMCD) [MIM:156500]
COL10A1 Q03692 VAR_001849 p.Leu644Arg LP/P - Schmid type metaphyseal chondrodysplasia (SMCD) [MIM:156500]
COL10A1 Q03692 VAR_001850 p.Asp648Gly LP/P - Schmid type metaphyseal chondrodysplasia (SMCD) [MIM:156500]
COL10A1 Q03692 VAR_008039 p.Tyr597Cys LP/P rs111033554 Schmid type metaphyseal chondrodysplasia (SMCD) [MIM:156500]
COL10A1 Q03692 VAR_023186 p.Met27Thr LB/B rs1064583 -
COL10A1 Q03692 VAR_023187 p.Arg198His LB/B rs148785195 -
COL10A1 Q03692 VAR_023188 p.Tyr582Asp LP/P - Schmid type metaphyseal chondrodysplasia (SMCD) [MIM:156500]
COL10A1 Q03692 VAR_023189 p.Gly595Arg LP/P - Schmid type metaphyseal chondrodysplasia (SMCD) [MIM:156500]
COL10A1 Q03692 VAR_023190 p.Val603Met LB/B rs143769451 -
COL10A1 Q03692 VAR_023191 p.Trp651Arg LP/P rs111033549 Schmid type metaphyseal chondrodysplasia (SMCD) [MIM:156500]
COL10A1 Q03692 VAR_023192 p.Gln653Pro LP/P rs1271742789 Schmid type metaphyseal chondrodysplasia (SMCD) [MIM:156500]
COL10A1 Q03692 VAR_023193 p.Ser671Pro LP/P rs111033552 Schmid type metaphyseal chondrodysplasia (SMCD) [MIM:156500]
COL10A1 Q03692 VAR_048767 p.Gly98Arg LB/B rs2243370 -
COL11A1 P12107 VAR_013583 p.Gly625Val LP/P rs121912943 Stickler syndrome 2 (STL2) [MIM:604841]
COL11A1 P12107 VAR_013584 p.Gly676Arg LP/P rs749663226 Stickler syndrome 2 (STL2) [MIM:604841]
COL11A1 P12107 VAR_013587 p.Gly1516Val LP/P rs1553193910 Stickler syndrome 2 (STL2) [MIM:604841]
COL11A1 P12107 VAR_035743 p.Ala1326Val US - A breast cancer sample
COL11A1 P12107 VAR_035744 p.Gln1328Lys US rs750014974 A breast cancer sample
COL11A1 P12107 VAR_035745 p.Gln1328Leu US - A breast cancer sample
COL11A1 P12107 VAR_047723 p.Trp8Gly LB/B rs12025888 -
COL11A1 P12107 VAR_047724 p.Asp46Glu LB/B rs11164663 -
COL11A1 P12107 VAR_047725 p.Gly559Ser LB/B rs12143815 -
COL11A1 P12107 VAR_047726 p.Pro1323Leu LB/B rs3753841 -
COL11A1 P12107 VAR_047727 p.Ser1535Pro LB/B rs1676486 -
COL11A1 P12107 VAR_047728 p.Leu1805Phe LB/B rs1975916 -
COL11A1 P12107 VAR_063675 p.Gly565Val LP/P - Stickler syndrome 2 (STL2) [MIM:604841]
COL11A1 P12107 VAR_063676 p.Gly1027Arg LP/P - Stickler syndrome 2 (STL2) [MIM:604841]
COL11A1 P12107 VAR_063678 p.Gly1513Asp LP/P rs1553193913 Stickler syndrome 2 (STL2) [MIM:604841]
COL11A1 P12107 VAR_065904 p.Gly796Arg LP/P - Fibrochondrogenesis 1 (FBCG1) [MIM:228520]
COL11A1 P12107 VAR_065905 p.Gly1042Arg LP/P - Fibrochondrogenesis 1 (FBCG1) [MIM:228520]
COL11A2 P13942 VAR_001907 p.Gly661Arg LP/P rs121912945 Otospondylomegaepiphyseal dysplasia, autosomal recessive (OSMEDB) [MIM:215150]
COL11A2 P13942 VAR_010655 p.Gly808Glu LP/P rs121912948 Deafness, autosomal dominant, 13 (DFNA13) [MIM:601868]
COL11A2 P13942 VAR_010656 p.Arg1034Cys LP/P rs121912947 Deafness, autosomal dominant, 13 (DFNA13) [MIM:601868]
COL11A2 P13942 VAR_013591 p.Asp593Gly LB/B - -
COL11A2 P13942 VAR_013592 p.Glu824Lys LB/B rs1799909 -

COL11A2 P13942 VAR_013593 p.Pro879Leu LB/B rs747883362 -
COL11A2 P13942 VAR_013595 p.Gly1441Glu LP/P rs121912946 Otospondylomegaepiphyseal dysplasia, autosomal dominant (OSMEDA) [MIM:184840]
COL11A2 P13942 VAR_013596 p.Pro1316Thr LB/B rs2229784 -
COL11A2 P13942 VAR_013597 p.Arg1600Gln LB/B rs1799912 -
COL11A2 P13942 VAR_025276 p.Pro621Thr LP/P rs121912952 Deafness, autosomal recessive, 53 (DFNB53) [MIM:609706]
COL11A2 P13942 VAR_033797 p.Glu1628Asp LB/B rs2229790 -
COL11A2 P13942 VAR_048804 p.Pro236Ser LB/B rs35116188 -
COL11A2 P13942 VAR_048805 p.Glu276Lys LB/B rs9277934 -
COL11A2 P13942 VAR_048806 p.Leu894Pro LB/B rs2855430 -
COL11A2 P13942 VAR_048807 p.Pro1722Leu LB/B rs2229792 -
COL11A2 P13942 VAR_072731 p.Ala37Ser LP/P rs606231410 Deafness, autosomal recessive, 53 (DFNB53) [MIM:609706]
COL11A2 P13942 VAR_072732 p.Pro888Thr LP/P rs864309523 Deafness, autosomal recessive, 53 (DFNB53) [MIM:609706]
COL11A2 P13942 VAR_079875 p.Pro1422Leu LB/B rs555936333 -
COL12A1 Q99715 VAR_048768 p.Ala461Pro LB/B rs34730529 -
COL12A1 Q99715 VAR_048769 p.Ile1738Thr LB/B rs240736 -
COL12A1 Q99715 VAR_048770 p.Glu2160Val LB/B rs35523808 -
COL12A1 Q99715 VAR_048771 p.Ile2596Val LB/B rs35710072 -
COL12A1 Q99715 VAR_061111 p.Arg2021Gln LB/B rs34438461 -
COL12A1 Q99715 VAR_061112 p.Gln3048His LB/B rs57396313 -
COL12A1 Q99715 VAR_074546 p.Arg1965Cys US rs200487396 Bethlem myopathy 2 (BTHLM2) [MIM:616471]
COL12A1 Q99715 VAR_074547 p.Ile2334Thr LP/P rs796052093 Bethlem myopathy 2 (BTHLM2) [MIM:616471]
COL12A1 Q99715 VAR_074548 p.Gly2786Asp LP/P rs796052094 Bethlem myopathy 2 (BTHLM2) [MIM:616471]
COL12A1 Q99715 VAR_074549 p.Gly3058Ser LB/B rs970547 -
COL13A1 Q5TAT6 VAR_055670 p.His531Gln LB/B rs1061954 -
COL14A1 Q05707 VAR_048772 p.Asn563His LB/B rs4870723 -
COL14A1 Q05707 VAR_048773 p.Pro855Leu LB/B rs2305606 -
COL14A1 Q05707 VAR_048774 p.Val922Ile LB/B rs11774228 -
COL14A1 Q05707 VAR_048775 p.Val1342Leu LB/B rs17833992 -
COL14A1 Q05707 VAR_061113 p.Thr636Ala LB/B rs56815167 -
COL15A1 P39059 VAR_033787 p.Arg163His LB/B rs2075662 -
COL15A1 P39059 VAR_033788 p.Met204Val LB/B rs2075663 -
COL15A1 P39059 VAR_033789 p.Thr391Met LB/B rs10988532 -
COL15A1 P39059 VAR_033790 p.Ala442Thr LB/B rs16918128 -
COL15A1 P39059 VAR_033791 p.Gly446Arg LB/B rs35934703 -
COL15A1 P39059 VAR_033792 p.Glu506Asp LB/B rs35250850 -
COL15A1 P39059 VAR_033793 p.Pro531Arg LB/B rs35529307 -
COL15A1 P39059 VAR_033794 p.Lys989Arg LB/B rs35642150 -
COL15A1 P39059 VAR_033795 p.Lys1001Arg LB/B rs35544077 -
COL15A1 P39059 VAR_033796 p.Val1332Ile LB/B rs10519 -
COL15A1 P39059 VAR_048776 p.Gly504Val LB/B rs2297603 -
COL15A1 P39059 VAR_061114 p.Pro705Leu LB/B rs41308900 -
COL16A1 Q07092 VAR_031440 p.Thr62Lys LB/B rs2228552 -
COL16A1 Q07092 VAR_048778 p.Arg418Gln LB/B rs6699645 -
COL16A1 Q07092 VAR_048779 p.Gly745Ser LB/B rs34770879 -
COL17A1 Q9UMD9 VAR_017593 p.Thr210Met LB/B rs805708 -

COL17A1 Q9UMD9 VAR_017594 p.Met231Ile LB/B rs1054113 -
COL17A1 Q9UMD9 VAR_017595 p.Met238Thr LB/B - -
COL17A1 Q9UMD9 VAR_017596 p.Ser265Cys LP/P - Generalized atrophic benign epidermolysis bullosa (GABEB) [MIM:226650]
COL17A1 Q9UMD9 VAR_017597 p.Gly428Ser LB/B rs805698 -
COL17A1 Q9UMD9 VAR_017598 p.Gly627Val LP/P - Generalized atrophic benign epidermolysis bullosa (GABEB) [MIM:226650]
COL17A1 Q9UMD9 VAR_017599 p.Gly633Asp LP/P rs121912773 Generalized atrophic benign epidermolysis bullosa (GABEB) [MIM:226650]
COL17A1 Q9UMD9 VAR_017600 p.Met703Val LB/B rs805722 -
COL17A1 Q9UMD9 VAR_017601 p.Arg1303Gln LP/P rs121912771 Generalized atrophic benign epidermolysis bullosa (GABEB) [MIM:226650]
COL17A1 Q9UMD9 VAR_017602 p.Asp1370Gly LB/B rs17116350 -
COL17A1 Q9UMD9 VAR_048781 p.Thr4Ala LB/B rs17116471 -
COL17A1 Q9UMD9 VAR_074627 p.Thr939Ile LP/P rs797045142 Epithelial recurrent erosion dystrophy (ERED) [MIM:122400]
COL18A1 P39060 VAR_012709 p.Asp1675Asn LB/B rs12483377 -
COL18A1 P39060 VAR_018053 p.Gln49Leu LB/B rs61735029 -
COL18A1 P39060 VAR_018054 p.Gly111Arg LB/B rs114139997 -
COL18A1 P39060 VAR_018055 p.Val1076Ile LB/B rs62000962 -
COL18A1 P39060 VAR_018056 p.Pro1121Arg LB/B rs79980197 -
COL18A1 P39060 VAR_059232 p.Ala288Thr LB/B rs11702494 -
COL18A1 P39060 VAR_059233 p.Gln1195His LB/B rs2230693 -
COL18A1 P39060 VAR_061115 p.Thr379Met LB/B rs8133886 -
COL18A1 P39060 VAR_084282 p.Glu184Lys US - Glaucoma, primary closed-angle (GLCC) [MIM:618880]
COL19A1 Q14993 VAR_024419 p.Ala352Gly LB/B rs2273426 -
COL19A1 Q14993 VAR_035746 p.Gly361Asp US rs1313704586 A breast cancer sample
COL19A1 Q14993 VAR_035747 p.Lys1019Asn US rs771562232 A breast cancer sample
COL19A1 Q14993 VAR_048782 p.Gly406Glu LB/B rs13204209 -
COL19A1 Q14993 VAR_048783 p.Glu496Gly LB/B rs13204209 -
COL1A1 P02452 VAR_001642 p.Gly197Cys LB/B rs8179178 -
COL1A1 P02452 VAR_001643 p.Pro205Ala LB/B rs72667032 -
COL1A1 P02452 VAR_001644 p.Gly221Cys LP/P rs72667037 Osteogenesis imperfecta 1 (OI1) [MIM:166200]
COL1A1 P02452 VAR_001645 p.Gly224Cys LP/P rs72667038 Osteogenesis imperfecta 1 (OI1) [MIM:166200]
COL1A1 P02452 VAR_001646 p.Gly263Arg LP/P rs72645323 Osteogenesis imperfecta 1 (OI1) [MIM:166200]
COL1A1 P02452 VAR_001647 p.Gly263Val LP/P rs72645324 Osteogenesis imperfecta 1 (OI1) [MIM:166200]
COL1A1 P02452 VAR_001648 p.Gly272Cys LP/P rs72645331 Osteogenesis imperfecta 1 (OI1) [MIM:166200]
COL1A1 P02452 VAR_001649 p.Gly275Asp LP/P rs72645333 Osteogenesis imperfecta 2 (OI2) [MIM:166210]
COL1A1 P02452 VAR_001650 p.Gly332Arg LP/P rs72645357 Osteogenesis imperfecta 3 (OI3) [MIM:259420]
COL1A1 P02452 VAR_001651 p.Gly350Arg LP/P rs72645363 Osteogenesis imperfecta 3 (OI3) [MIM:259420]
COL1A1 P02452 VAR_001652 p.Gly353Cys LP/P rs66721653 Osteogenesis imperfecta 4 (OI4) [MIM:166220]
COL1A1 P02452 VAR_001653 p.Gly356Cys LP/P rs72645365 Osteogenesis imperfecta 4 (OI4) [MIM:166220]
COL1A1 P02452 VAR_001654 p.Gly383Cys LP/P rs67182491 Osteogenesis imperfecta 4 (OI4) [MIM:166220]
COL1A1 P02452 VAR_001655 p.Gly389Cys LB/B rs66548636 -
COL1A1 P02452 VAR_001656 p.Gly389Arg LP/P rs66548636 Osteogenesis imperfecta 2 (OI2) [MIM:166210]
COL1A1 P02452 VAR_001657 p.Gly398Ala LP/P rs66501246 Osteogenesis imperfecta 4 (OI4) [MIM:166220]
COL1A1 P02452 VAR_001658 p.Gly398Asp LP/P rs66501246 Osteogenesis imperfecta 2 (OI2) [MIM:166210]
COL1A1 P02452 VAR_001659 p.Gly401Cys LP/P rs72648322 Osteogenesis imperfecta 4 (OI4) [MIM:166220]
COL1A1 P02452 VAR_001660 p.Gly404Cys LB/B rs66893386 -

COL1A1	P02452	VAR_001661	p.Gly422Cys	LP/P	rs72648328	Osteogenesis imperfecta 2 (OI2) [MIM:166210]
COL1A1	P02452	VAR_001662	p.Gly425Ser	LP/P	rs72648330	Osteogenesis imperfecta 2 (OI2) [MIM:166210]
COL1A1	P02452	VAR_001663	p.Gly434Val	LP/P	rs72648333	Osteogenesis imperfecta 2 (OI2) [MIM:166210]
COL1A1	P02452	VAR_001664	p.Gly476Arg	LP/P	rs57377812	Osteogenesis imperfecta 2 (OI2) [MIM:166210]
COL1A1	P02452	VAR_001665	p.Gly527Cys	LP/P	rs72648353	Osteogenesis imperfecta 4 (OI4) [MIM:166220]
COL1A1	P02452	VAR_001666	p.Gly530Ser	LP/P	rs67682641	Osteogenesis imperfecta 2 (OI2) [MIM:166210]
COL1A1	P02452	VAR_001666	p.Gly530Ser	LP/P	rs67682641	Osteogenesis imperfecta 3 (OI3) [MIM:259420]
COL1A1	P02452	VAR_001666	p.Gly530Ser	LP/P	rs67682641	Osteogenesis imperfecta 4 (OI4) [MIM:166220]
COL1A1	P02452	VAR_001667	p.Gly533Asp	LP/P	rs72648356	Osteogenesis imperfecta 2 (OI2) [MIM:166210]
COL1A1	P02452	VAR_001668	p.Gly560Ser	LP/P	rs67507747	Osteogenesis imperfecta 4 (OI4) [MIM:166220]
COL1A1	P02452	VAR_001669	p.Gly560Cys	LP/P	rs67507747	Osteogenesis imperfecta 4 (OI4) [MIM:166220]
COL1A1	P02452	VAR_001670	p.Gly560Arg	LP/P	rs67507747	Osteogenesis imperfecta 2 (OI2) [MIM:166210]
COL1A1	P02452	VAR_001671	p.Arg564His	LB/B	rs1800211	-
COL1A1	P02452	VAR_001672	p.Gly569Arg	LP/P	rs72648363	Osteogenesis imperfecta 2 (OI2) [MIM:166210]
COL1A1	P02452	VAR_001673	p.Gly593Cys	LP/P	rs66527965	Osteogenesis imperfecta 3 (OI3) [MIM:259420]
COL1A1	P02452	VAR_001673	p.Gly593Cys	LP/P	rs66527965	Osteogenesis imperfecta 4 (OI4) [MIM:166220]
COL1A1	P02452	VAR_001674	p.Gly593Ser	LP/P	rs66527965	Osteogenesis imperfecta 2 (OI2) [MIM:166210]
COL1A1	P02452	VAR_001674	p.Gly593Ser	LP/P	rs66527965	Osteogenesis imperfecta 3 (OI3) [MIM:259420]
COL1A1	P02452	VAR_001676	p.Gly656Ser	LP/P	rs72651629	Osteogenesis imperfecta 2 (OI2) [MIM:166210]
COL1A1	P02452	VAR_001677	p.Gly701Cys	LP/P	rs68114505	Osteogenesis imperfecta 4 (OI4) [MIM:166220]
COL1A1	P02452	VAR_001678	p.Gly704Cys	LP/P	rs67368147	Osteogenesis imperfecta 3 (OI3) [MIM:259420]
COL1A1	P02452	VAR_001679	p.Gly719Asp	LP/P	rs72651646	Osteogenesis imperfecta 2 (OI2) [MIM:166210]
COL1A1	P02452	VAR_001680	p.Gly719Ser	LP/P	rs72651645	Osteogenesis imperfecta 3 (OI3) [MIM:259420]
COL1A1	P02452	VAR_001681	p.Gly728Arg	LP/P	rs72651648	Osteogenesis imperfecta 2 (OI2) [MIM:166210]
COL1A1	P02452	VAR_001682	p.Gly737Asp	LP/P	rs72651651	Osteogenesis imperfecta 2 (OI2) [MIM:166210]
COL1A1	P02452	VAR_001683	p.Gly743Ser	LP/P	rs72651652	Osteogenesis imperfecta 2 (OI2) [MIM:166210]
COL1A1	P02452	VAR_001684	p.Gly743Val	LP/P	rs72651653	Osteogenesis imperfecta 2 (OI2) [MIM:166210]
COL1A1	P02452	VAR_001685	p.Gly764Val	LP/P	rs72651657	Osteogenesis imperfecta 2 (OI2) [MIM:166210]
COL1A1	P02452	VAR_001686	p.Gly767Ser	LP/P	rs72651658	Osteogenesis imperfecta 3 (OI3) [MIM:259420]
COL1A1	P02452	VAR_001687	p.Gly776Ser	LP/P	rs72651660	Osteogenesis imperfecta 2 (OI2) [MIM:166210]
COL1A1	P02452	VAR_001688	p.Gly809Ser	LP/P	rs72651663	Osteogenesis imperfecta 2 (OI2) [MIM:166210]
COL1A1	P02452	VAR_001689	p.Gly815Val	LP/P	rs66929517	Osteogenesis imperfecta 2 (OI2) [MIM:166210]
COL1A1	P02452	VAR_001690	p.Gly821Ser	LP/P	rs67693970	Osteogenesis imperfecta 3 (OI3) [MIM:259420]
COL1A1	P02452	VAR_001691	p.Pro823Ala	LB/B	rs1800214	-
COL1A1	P02452	VAR_001692	p.Gly839Ser	LP/P	rs72653131	Osteogenesis imperfecta 2 (OI2) [MIM:166210]
COL1A1	P02452	VAR_001693	p.Gly842Arg	LP/P	rs72653134	Osteogenesis imperfecta 2 (OI2) [MIM:166210]
COL1A1	P02452	VAR_001694	p.Gly845Arg	LP/P	rs72653136	Osteogenesis imperfecta 2 (OI2) [MIM:166210]
COL1A1	P02452	VAR_001695	p.Gly851Asp	LP/P	rs72653137	Osteogenesis imperfecta 2 (OI2) [MIM:166210]
COL1A1	P02452	VAR_001696	p.Gly869Cys	LP/P	rs72653143	Osteogenesis imperfecta 2 (OI2) [MIM:166210]
COL1A1	P02452	VAR_001697	p.Gly884Ser	LP/P	rs1567755602	Osteogenesis imperfecta 2 (OI2) [MIM:166210]
COL1A1	P02452	VAR_001697	p.Gly884Ser	LP/P	rs1567755602	Osteogenesis imperfecta 3 (OI3) [MIM:259420]
COL1A1	P02452	VAR_001698	p.Gly896Cys	LP/P	rs72653152	Osteogenesis imperfecta 2 (OI2) [MIM:166210]
COL1A1	P02452	VAR_001699	p.Gly926Cys	LP/P	rs72653154	Osteogenesis imperfecta 2 (OI2) [MIM:166210]
COL1A1	P02452	VAR_001700	p.Gly980Val	LP/P	rs72653166	Osteogenesis imperfecta 2 (OI2) [MIM:166210]
COL1A1	P02452	VAR_001701	p.Gly1010Ser	LP/P	rs72653169	Osteogenesis imperfecta 4 (OI4) [MIM:166220]
COL1A1	P02452	VAR_001702	p.Gly1022Ser	LP/P	rs66523073	Osteogenesis imperfecta 3 (OI3) [MIM:259420]
COL1A1	P02452	VAR_001703	p.Gly1022Val	LP/P	rs67771061	Osteogenesis imperfecta 2 (OI2) [MIM:166210]
COL1A1	P02452	VAR_001704	p.Gly1025Arg	LP/P	rs72653172	Osteogenesis imperfecta 2 (OI2) [MIM:166210]
COL1A1	P02452	VAR_001705	p.Gly1040Ser	LP/P	rs72653178	Osteogenesis imperfecta 2 (OI2) [MIM:166210]

COL1A1	P02452	VAR_001705	p.Gly1040Ser	LP/P	rs72653178	Osteogenesis imperfecta 3 (OI3) [MIM:259420]
COL1A1	P02452	VAR_001706	p.Gly1043Ser	LP/P	-	Osteogenesis imperfecta 2 (OI2) [MIM:166210]
COL1A1	P02452	VAR_001708	p.Gly1049Ser	LP/P	rs67641695	Osteogenesis imperfecta 3 (OI3) [MIM:259420]
COL1A1	P02452	VAR_001709	p.Gly1058Ser	LP/P	rs72654795	Osteogenesis imperfecta 3 (OI3) [MIM:259420]
COL1A1	P02452	VAR_001709	p.Gly1058Ser	LP/P	rs72654795	Osteogenesis imperfecta 4 (OI4) [MIM:166220]
COL1A1	P02452	VAR_001710	p.Gly1061Asp	LP/P	rs72654797	Osteogenesis imperfecta 2 (OI2) [MIM:166210]
COL1A1	P02452	VAR_001711	p.Gly1061Ser	LP/P	rs72654796	Osteogenesis imperfecta 4 (OI4) [MIM:166220]
COL1A1	P02452	VAR_001712	p.Thr1075Ala	LB/B	rs1800215	-
COL1A1	P02452	VAR_001713	p.Gly1076Ser	LP/P	rs67394386	Osteogenesis imperfecta 3 (OI3) [MIM:259420]
COL1A1	P02452	VAR_001714	p.Gly1079Ser	LP/P	rs72654802	Osteogenesis imperfecta 1 (OI1) [MIM:166200]
COL1A1	P02452	VAR_001714	p.Gly1079Ser	LP/P	rs72654802	Osteogenesis imperfecta 2 (OI2) [MIM:166210]
COL1A1	P02452	VAR_001715	p.Gly1082Cys	LP/P	rs72656303	Osteogenesis imperfecta 2 (OI2) [MIM:166210]
COL1A1	P02452	VAR_001716	p.Gly1088Ala	LP/P	rs72656305	Osteogenesis imperfecta 2 (OI2) [MIM:166210]
COL1A1	P02452	VAR_001717	p.Gly1091Ser	LP/P	rs72656306	Osteogenesis imperfecta 2 (OI2) [MIM:166210]
COL1A1	P02452	VAR_001718	p.Gly1100Asp	LP/P	rs72656308	Osteogenesis imperfecta 2 (OI2) [MIM:166210]
COL1A1	P02452	VAR_001719	p.Gly1106Ala	LP/P	rs72656311	Osteogenesis imperfecta 2 (OI2) [MIM:166210]
COL1A1	P02452	VAR_001720	p.Gly1124Cys	LP/P	rs72656312	Osteogenesis imperfecta 2 (OI2) [MIM:166210]
COL1A1	P02452	VAR_001721	p.Gly1142Ser	LP/P	rs72656317	Osteogenesis imperfecta 2 (OI2) [MIM:166210]
COL1A1	P02452	VAR_001722	p.Gly1151Ser	LP/P	rs72656320	Osteogenesis imperfecta 3 (OI3) [MIM:259420]
COL1A1	P02452	VAR_001723	p.Gly1151Val	LP/P	rs72656321	Osteogenesis imperfecta 2 (OI2) [MIM:166210]
COL1A1	P02452	VAR_001724	p.Gly1154Arg	LP/P	rs72656322	Osteogenesis imperfecta 2 (OI2) [MIM:166210]
COL1A1	P02452	VAR_001725	p.Gly1166Cys	LP/P	rs72656324	Osteogenesis imperfecta 2 (OI2) [MIM:166210]
COL1A1	P02452	VAR_001726	p.Gly1172Asp	LP/P	rs72656325	Osteogenesis imperfecta 2 (OI2) [MIM:166210]
COL1A1	P02452	VAR_001727	p.Gly1181Ser	LP/P	rs72656330	Osteogenesis imperfecta 2 (OI2) [MIM:166210]
COL1A1	P02452	VAR_001728	p.Gly1184Val	LP/P	rs72656331	Osteogenesis imperfecta 2 (OI2) [MIM:166210]
COL1A1	P02452	VAR_001729	p.Gly1187Ser	LP/P	rs72656332	Osteogenesis imperfecta 2 (OI2) [MIM:166210]
COL1A1	P02452	VAR_001729	p.Gly1187Ser	LP/P	rs72656332	Osteogenesis imperfecta 3 (OI3) [MIM:259420]
COL1A1	P02452	VAR_001730	p.Gly1187Val	LP/P	rs66948146	Osteogenesis imperfecta 2 (OI2) [MIM:166210]
COL1A1	P02452	VAR_001731	p.Gly1195Cys	LP/P	rs72656334	Osteogenesis imperfecta 1 (OI1) [MIM:166200]
COL1A1	P02452	VAR_001732	p.Asp1277His	LP/P	rs72656342	Osteogenesis imperfecta 2 (OI2) [MIM:166210]
COL1A1	P02452	VAR_001733	p.Trp1312Cys	LP/P	rs72656343	Osteogenesis imperfecta 2 (OI2) [MIM:166210]
COL1A1	P02452	VAR_001735	p.Leu1388Arg	LP/P	rs72656348	Osteogenesis imperfecta 2 (OI2) [MIM:166210]
COL1A1	P02452	VAR_001736	p.Thr1434Ser	LB/B	rs1800220	-
COL1A1	P02452	VAR_001737	p.Leu1464Pro	LP/P	rs72656353	Osteogenesis imperfecta 3 (OI3) [MIM:259420]
COL1A1	P02452	VAR_008118	p.Gly866Ser	LP/P	rs67445413	Osteogenesis imperfecta 2 (OI2) [MIM:166210]
COL1A1	P02452	VAR_008118	p.Gly866Ser	LP/P	rs67445413	Osteogenesis imperfecta 3 (OI3) [MIM:259420]
COL1A1	P02452	VAR_013579	p.Arg312Cys	LP/P	rs72645347	Ehlers-Danlos syndrome, classic type, 1 (EDSCL1) [MIM:130000]
COL1A1	P02452	VAR_030013	p.Gly1019Ala	LB/B	rs1135348	-
COL1A1	P02452	VAR_030014	p.Ser1251Thr	LB/B	rs3205325	-
COL1A1	P02452	VAR_030015	p.Gln1391Lys	LB/B	rs2586486	-
COL1A1	P02452	VAR_030016	p.Pro1438Arg	LB/B	rs17857117	-
COL1A1	P02452	VAR_030017	p.Pro1460His	LB/B	rs17853657	-
COL1A1	P02452	VAR_033097	p.Arg1014Cys	LP/P	rs72653170	Caffey disease (CAFYD) [MIM:114000]
COL1A1	P02452	VAR_033778	p.Arg1141Gln	LB/B	rs41316713	-
COL1A1	P02452	VAR_033779	p.Val1177Ile	LB/B	rs41316719	-
COL1A1	P02452	VAR_033780	p.Lys1430Asn	LB/B	rs1059454	-
COL1A1	P02452	VAR_033781	p.Thr1431Pro	LB/B	rs1059454	-
COL1A1	P02452	VAR_063290	p.Gly22Arg	LP/P	rs72667007	Osteogenesis imperfecta 2 (OI2) [MIM:166210]

COL1A1	P02452	VAR_063291	p.Pro146Thr	US	rs756846639	Osteogenesis imperfecta 2 (OI2) [MIM:166210]
COL1A1	P02452	VAR_063292	p.Gly194Arg	LP/P	rs72667024	Osteogenesis imperfecta 1 (OI1) [MIM:166200]
COL1A1	P02452	VAR_063293	p.Gly197Arg	LP/P	-	Osteogenesis imperfecta 4 (OI4) [MIM:166220]
COL1A1	P02452	VAR_063294	p.Gly200Val	LP/P	rs72667029	Osteogenesis imperfecta 1 (OI1) [MIM:166200]
COL1A1	P02452	VAR_063295	p.Gly203Val	LP/P	rs72667031	Osteogenesis imperfecta 3 (OI3) [MIM:259420]
COL1A1	P02452	VAR_063296	p.Gly242Asp	LB/B	rs72645315	-
COL1A1	P02452	VAR_063297	p.Gly257Arg	LP/P	rs72645321	Osteogenesis imperfecta 4 (OI4) [MIM:166220]
COL1A1	P02452	VAR_063298	p.Gly266Glu	LP/P	rs72645325	Osteogenesis imperfecta 1 (OI1) [MIM:166200]
COL1A1	P02452	VAR_063299	p.Gly287Ser	LP/P	rs72645340	Osteogenesis imperfecta 1 (OI1) [MIM:166200]
COL1A1	P02452	VAR_063300	p.Glu288Lys	US	rs72645341	Osteogenesis imperfecta 1 (OI1) [MIM:166200]
COL1A1	P02452	VAR_063301	p.Glu288Val	US	-	Osteogenesis imperfecta 2 (OI2) [MIM:166210]
COL1A1	P02452	VAR_063302	p.Gly320Val	LP/P	rs72645353	Osteogenesis imperfecta 1 (OI1) [MIM:166200]
COL1A1	P02452	VAR_063303	p.Gly338Cys	LP/P	rs66664580	Osteogenesis imperfecta 4 (OI4) [MIM:166220]
COL1A1	P02452	VAR_063304	p.Val349Phe	LP/P	rs72645362	Osteogenesis imperfecta 1 (OI1) [MIM:166200]
COL1A1	P02452	VAR_063305	p.Gly353Asp	LP/P	-	Osteogenesis imperfecta 2 (OI2) [MIM:166210]
COL1A1	P02452	VAR_063306	p.Gly353Ser	LP/P	rs66721653	Osteogenesis imperfecta 4 (OI4) [MIM:166220]
COL1A1	P02452	VAR_063307	p.Gly368Val	LP/P	-	Osteogenesis imperfecta 2 (OI2) [MIM:166210]
COL1A1	P02452	VAR_063308	p.Ala390Thr	US	rs116794104	Osteogenesis imperfecta 2 (OI2) [MIM:166210]
COL1A1	P02452	VAR_063309	p.Gly455Asp	LP/P	-	Osteogenesis imperfecta 2 (OI2) [MIM:166210]
COL1A1	P02452	VAR_063310	p.Gly470Val	LP/P	-	Osteogenesis imperfecta 2 (OI2) [MIM:166210]
COL1A1	P02452	VAR_063311	p.Gly509Val	LP/P	-	Osteogenesis imperfecta 2 (OI2) [MIM:166210]
COL1A1	P02452	VAR_063312	p.Gly548Ala	LP/P	-	Osteogenesis imperfecta 2 (OI2) [MIM:166210]
COL1A1	P02452	VAR_063313	p.Pro555Arg	LP/P	rs72648359	Osteogenesis imperfecta 1 (OI1) [MIM:166200]
COL1A1	P02452	VAR_063314	p.Arg574Cys	US	rs72648365	-
COL1A1	P02452	VAR_063315	p.Gly581Arg	LP/P	rs72648366	Osteogenesis imperfecta 2 (OI2) [MIM:166210]
COL1A1	P02452	VAR_063316	p.Gly602Arg	LP/P	rs72651615	Osteogenesis imperfecta 2 (OI2) [MIM:166210]
COL1A1	P02452	VAR_063317	p.Gly605Asp	LP/P	-	Osteogenesis imperfecta 2 (OI2) [MIM:166210]
COL1A1	P02452	VAR_063318	p.Gly614Arg	LP/P	-	Osteogenesis imperfecta 2 (OI2) [MIM:166210]
COL1A1	P02452	VAR_063319	p.Gly647Ser	LP/P	rs72651627	Osteogenesis imperfecta 1 (OI1) [MIM:166200]
COL1A1	P02452	VAR_063320	p.Gly683Ser	LP/P	rs72651636	Osteogenesis imperfecta 4 (OI4) [MIM:166220]
COL1A1	P02452	VAR_063321	p.Gly722Ser	LP/P	rs72651647	Osteogenesis imperfecta 1 (OI1) [MIM:166200]
COL1A1	P02452	VAR_063322	p.Gly734Val	LP/P	rs72651649	Osteogenesis imperfecta 2 (OI2) [MIM:166210]
COL1A1	P02452	VAR_063323	p.Gly740Arg	LP/P	-	Osteogenesis imperfecta 2 (OI2) [MIM:166210]
COL1A1	P02452	VAR_063324	p.Gly824Arg	LP/P	-	Osteogenesis imperfecta 2 (OI2) [MIM:166210]
COL1A1	P02452	VAR_063325	p.Gly833Asp	LP/P	rs67067133	Osteogenesis imperfecta 2 (OI2) [MIM:166210]
COL1A1	P02452	VAR_063326	p.Asn855His	US	-	Osteogenesis imperfecta 2 (OI2) [MIM:166210]
COL1A1	P02452	VAR_063327	p.Gly875Ser	LP/P	rs72653145	Osteogenesis imperfecta 2 (OI2) [MIM:166210]
COL1A1	P02452	VAR_063328	p.Gly896Asp	LP/P	-	Osteogenesis imperfecta 2 (OI2) [MIM:166210]
COL1A1	P02452	VAR_063329	p.Gly906Ser	US	rs145446512	-
COL1A1	P02452	VAR_063330	p.Gly947Cys	LP/P	rs72653159	Osteogenesis imperfecta 2 (OI2) [MIM:166210]
COL1A1	P02452	VAR_063331	p.Gly977Asp	LP/P	-	Osteogenesis imperfecta 2 (OI2) [MIM:166210]
COL1A1	P02452	VAR_063332	p.Gly1001Cys	LP/P	rs72653167	Osteogenesis imperfecta 2 (OI2) [MIM:166210]
COL1A1	P02452	VAR_063334	p.Gly1055Asp	LP/P	-	Osteogenesis imperfecta 2 (OI2) [MIM:166210]
COL1A1	P02452	VAR_063335	p.Arg1066Cys	LP/P	rs72654799	-
COL1A1	P02452	VAR_063336	p.Arg1093Cys	US	rs72656307	-
COL1A1	P02452	VAR_063337	p.Gly1094Ser	LP/P	-	Osteogenesis imperfecta 2 (OI2) [MIM:166210]
COL1A1	P02452	VAR_063338	p.Gly1157Asp	LP/P	rs72656323	Osteogenesis imperfecta 1 (OI1) [MIM:166200]
COL1A1	P02452	VAR_063339	p.Asp1219Glu	LP/P	rs72656339	Osteogenesis imperfecta 1 (OI1) [MIM:166200]
COL1A1	P02452	VAR_063340	p.Arg1356His	LB/B	rs149820303	-

COL1A1	P02452	VAR_063341	p.Asp1413Asn	LP/P	rs72656349	Osteogenesis imperfecta 2 (OI2) [MIM:166210]
COL1A1	P02452	VAR_063342	p.Gly848Arg	LP/P	-	Osteogenesis imperfecta 2 (OI2) [MIM:166210]
COL1A1	P02452	VAR_066385	p.Asp1219Asn	LB/B	rs72656338	-
COL1A1	P02452	VAR_074158	p.Gly773Cys	LP/P	-	Osteogenesis imperfecta 2 (OI2) [MIM:166210]
COL1A1	P02452	VAR_074159	p.Gly1088Glu	US	-	Osteogenesis imperfecta 1 (OI1) [MIM:166200]
COL1A2	P08123	VAR_001852	p.Gly211Asp	LP/P	rs72656378	Osteogenesis imperfecta 1 (OI1) [MIM:166200]
COL1A2	P08123	VAR_001853	p.Ile249Asn	LB/B	rs1800228	-
COL1A2	P08123	VAR_001854	p.Ala276Thr	LB/B	rs1800231	-
COL1A2	P08123	VAR_001855	p.Gly328Ser	LP/P	rs66612022	Osteogenesis imperfecta 1 (OI1) [MIM:166200]
COL1A2	P08123	VAR_001856	p.Gly334Cys	LP/P	-	Osteogenesis imperfecta 2 (OI2) [MIM:166210]
COL1A2	P08123	VAR_001857	p.Gly337Cys	LP/P	rs67865220	Osteogenesis imperfecta 3 (OI3) [MIM:259420]
COL1A2	P08123	VAR_001858	p.Gly337Ser	LP/P	rs67865220	Osteogenesis imperfecta 3 (OI3) [MIM:259420]
COL1A2	P08123	VAR_001860	p.Gly349Cys	LP/P	rs66773001	Osteogenesis imperfecta 3 (OI3) [MIM:259420]
COL1A2	P08123	VAR_001861	p.Gly409Val	LP/P	rs72658109	Osteogenesis imperfecta 2 (OI2) [MIM:166210]
COL1A2	P08123	VAR_001862	p.Gly433Glu	LP/P	rs72658114	Osteogenesis imperfecta 2 (OI2) [MIM:166210]
COL1A2	P08123	VAR_001863	p.Gly460Ser	LP/P	rs72658118	Osteogenesis imperfecta 3 (OI3) [MIM:259420]
COL1A2	P08123	VAR_001864	p.Gly511Asp	LP/P	rs66999265	Osteogenesis imperfecta 2 (OI2) [MIM:166210]
COL1A2	P08123	VAR_001865	p.Gly517Arg	LP/P	rs72658126	Osteogenesis imperfecta 3 (OI3) [MIM:259420]
COL1A2	P08123	VAR_001866	p.Gly547Arg	LP/P	rs72658136	Osteogenesis imperfecta 2 (OI2) [MIM:166210]
COL1A2	P08123	VAR_001867	p.Pro549Ala	LB/B	rs42524	-
COL1A2	P08123	VAR_001868	p.Gly562Cys	LP/P	rs72658138	Osteogenesis imperfecta 2 (OI2) [MIM:166210]
COL1A2	P08123	VAR_001869	p.Gly586Arg	LP/P	rs72658139	Osteogenesis imperfecta 2 (OI2) [MIM:166210]
COL1A2	P08123	VAR_001870	p.Gly592Ser	LP/P	rs72658141	Osteogenesis imperfecta 2 (OI2) [MIM:166210]
COL1A2	P08123	VAR_001871	p.Gly634Val	LP/P	rs72658147	Osteogenesis imperfecta 4 (OI4) [MIM:166220]
COL1A2	P08123	VAR_001872	p.Gly637Asp	LP/P	rs72658148	Osteogenesis imperfecta 2 (OI2) [MIM:166210]
COL1A2	P08123	VAR_001873	p.Gly640Ser	LP/P	-	Osteogenesis imperfecta 2 (OI2) [MIM:166210]
COL1A2	P08123	VAR_001874	p.Gly670Asp	LP/P	rs72658155	Osteogenesis imperfecta 2 (OI2) [MIM:166210]
COL1A2	P08123	VAR_001875	p.Gly676Val	LP/P	rs66883877	Osteogenesis imperfecta 3 (OI3) [MIM:259420]
COL1A2	P08123	VAR_001875	p.Gly676Val	LP/P	rs66883877	Osteogenesis imperfecta 4 (OI4) [MIM:166220]
COL1A2	P08123	VAR_001876	p.Arg708Gln	US	rs72658163	-
COL1A2	P08123	VAR_001877	p.Gly715Asp	LP/P	rs72658167	Osteogenesis imperfecta 2 (OI2) [MIM:166210]
COL1A2	P08123	VAR_001878	p.Gly730Cys	LP/P	rs72658171	Osteogenesis imperfecta 2 (OI2) [MIM:166210]
COL1A2	P08123	VAR_001879	p.Gly736Cys	LP/P	rs72658173	Osteogenesis imperfecta 1 (OI1) [MIM:166200]
COL1A2	P08123	VAR_001880	p.Ala743Gly	LB/B	rs408535	-
COL1A2	P08123	VAR_001881	p.Gly751Ser	LP/P	rs72658176	Osteogenesis imperfecta 4 (OI4) [MIM:166220]
COL1A2	P08123	VAR_001882	p.Gly754Arg	LP/P	-	Osteogenesis imperfecta 2 (OI2) [MIM:166210]
COL1A2	P08123	VAR_001883	p.Gly766Val	LP/P	rs72658183	Osteogenesis imperfecta 4 (OI4) [MIM:166220]
COL1A2	P08123	VAR_001884	p.Gly778Ser	LP/P	rs72658186	Osteogenesis imperfecta 3 (OI3) [MIM:259420]
COL1A2	P08123	VAR_001885	p.Gly784Arg	LP/P	rs66592844	Osteogenesis imperfecta 2 (OI2) [MIM:166210]
COL1A2	P08123	VAR_001886	p.Gly787Cys	LP/P	rs72658187	Osteogenesis imperfecta 2 (OI2) [MIM:166210]
COL1A2	P08123	VAR_001887	p.Gly790Asp	LP/P	rs72658188	Osteogenesis imperfecta 2 (OI2) [MIM:166210]
COL1A2	P08123	VAR_001888	p.Gly796Ser	LP/P	rs66716547	Osteogenesis imperfecta 2 (OI2) [MIM:166210]
COL1A2	P08123	VAR_001889	p.Arg822His	LB/B	rs1800240	-
COL1A2	P08123	VAR_001890	p.Gly835Ser	LP/P	rs72658193	Osteogenesis imperfecta 1 (OI1) [MIM:166200]
COL1A2	P08123	VAR_001891	p.Gly877Cys	LP/P	rs72658201	Osteogenesis imperfecta 2 (OI2) [MIM:166210]
COL1A2	P08123	VAR_001892	p.Gly892Asp	LP/P	rs72659304	Osteogenesis imperfecta 3 (OI3) [MIM:259420]
COL1A2	P08123	VAR_001892	p.Gly892Asp	LP/P	rs72659304	Osteogenesis imperfecta 4 (OI4) [MIM:166220]
COL1A2	P08123	VAR_001893	p.Gly895Asp	LP/P	rs72659305	Osteogenesis imperfecta 2 (OI2) [MIM:166210]
COL1A2	P08123	VAR_001894	p.Gly949Ser	LP/P	rs72659312	Osteogenesis imperfecta 3 (OI3) [MIM:259420]

COL1A2	P08123	VAR_001895	p.Gly955Ser	LP/P	rs66507857	Osteogenesis imperfecta 2 (OI2) [MIM:166210]
COL1A2	P08123	VAR_001896	p.Gly997Asp	LP/P	rs72659317	Osteogenesis imperfecta 2 (OI2) [MIM:166210]
COL1A2	P08123	VAR_001897	p.Gly1012Ser	LP/P	rs72659319	Osteogenesis imperfecta 3 (OI3) [MIM:259420]
COL1A2	P08123	VAR_001897	p.Gly1012Ser	LP/P	rs72659319	Osteogenesis imperfecta 4 (OI4) [MIM:166220]
COL1A2	P08123	VAR_001898	p.Leu1022Phe	LB/B	rs392609	-
COL1A2	P08123	VAR_001899	p.Gly1066Asp	LP/P	rs72659331	Osteogenesis imperfecta 2 (OI2) [MIM:166210]
COL1A2	P08123	VAR_001900	p.Gly1078Cys	LP/P	-	Osteogenesis imperfecta 2 (OI2) [MIM:166210]
COL1A2	P08123	VAR_001901	p.Gly1096Ala	LP/P	rs72659337	Osteogenesis imperfecta 3 (OI3) [MIM:259420]
COL1A2	P08123	VAR_001902	p.Gly1102Arg	LP/P	rs67768540	Osteogenesis imperfecta 4 (OI4) [MIM:166220]
COL1A2	P08123	VAR_001903	p.Pro1101Leu	LB/B	-	-
COL1A2	P08123	VAR_001904	p.Thr1148Pro	LP/P	rs1800250	Osteogenesis imperfecta 3 (OI3) [MIM:259420]
COL1A2	P08123	VAR_001905	p.Asp1189Glu	LB/B	rs422361	-
COL1A2	P08123	VAR_001906	p.Ser1198Pro	LB/B	rs384487	-
COL1A2	P08123	VAR_008119	p.Gly331Asp	LP/P	rs67729041	Osteogenesis imperfecta 3 (OI3) [MIM:259420]
COL1A2	P08123	VAR_008120	p.Gly973Val	LP/P	rs67609234	Osteogenesis imperfecta 3 (OI3) [MIM:259420]
COL1A2	P08123	VAR_030116	p.Thr59Pro	LB/B	rs1800221	-
COL1A2	P08123	VAR_030118	p.Val270Ile	LB/B	rs368468	-
COL1A2	P08123	VAR_030119	p.Ala483Val	LB/B	rs414408	-
COL1A2	P08123	VAR_030121	p.Pro678His	LB/B	rs409108	-
COL1A2	P08123	VAR_030122	p.Gln1354His	LB/B	rs418570	-
COL1A2	P08123	VAR_033040	p.Asn528Ser	LB/B	rs41317144	-
COL1A2	P08123	VAR_033041	p.Ala564Thr	LB/B	rs41317153	-
COL1A2	P08123	VAR_055677	p.Leu344Val	LB/B	rs16868573	-
COL1A2	P08123	VAR_063343	p.Gly193Ser	LP/P	rs72656370	Osteogenesis imperfecta 4 (OI4) [MIM:166220]
COL1A2	P08123	VAR_063344	p.Gly202Arg	LP/P	rs72656376	Osteogenesis imperfecta 4 (OI4) [MIM:166220]
COL1A2	P08123	VAR_063345	p.Arg234Cys	LP/P	rs1206388800	Osteogenesis imperfecta 2 (OI2) [MIM:166210]
COL1A2	P08123	VAR_063346	p.Gly247Arg	LP/P	-	Osteogenesis imperfecta 1 (OI1) [MIM:166200]
COL1A2	P08123	VAR_063347	p.Gly253Asp	LP/P	rs72656385	Osteogenesis imperfecta 2 (OI2) [MIM:166210]
COL1A2	P08123	VAR_063348	p.Gly256Val	LP/P	rs67525025	Osteogenesis imperfecta 4 (OI4) [MIM:166220]
COL1A2	P08123	VAR_063349	p.Gly283Arg	LP/P	-	Osteogenesis imperfecta 2 (OI2) [MIM:166210]
COL1A2	P08123	VAR_063350	p.Gly319Arg	LP/P	rs72656393	Osteogenesis imperfecta 1 (OI1) [MIM:166200]
COL1A2	P08123	VAR_063351	p.Gly325Glu	LP/P	rs72656395	Osteogenesis imperfecta 4 (OI4) [MIM:166220]
COL1A2	P08123	VAR_063352	p.Gly358Ser	LP/P	rs66619856	Osteogenesis imperfecta 3 (OI3) [MIM:259420]
COL1A2	P08123	VAR_063353	p.Gly397Glu	LP/P	-	Osteogenesis imperfecta 2 (OI2) [MIM:166210]
COL1A2	P08123	VAR_063354	p.Gly454Cys	LP/P	rs72658117	Osteogenesis imperfecta 2 (OI2) [MIM:166210]
COL1A2	P08123	VAR_063355	p.Gly457Leu	LP/P	-	Osteogenesis imperfecta 2 (OI2) [MIM:166210]
COL1A2	P08123	VAR_063357	p.Gly526Glu	LP/P	rs72658130	Osteogenesis imperfecta 2 (OI2) [MIM:166210]
COL1A2	P08123	VAR_063358	p.Gly562Val	LP/P	-	Osteogenesis imperfecta 2 (OI2) [MIM:166210]
COL1A2	P08123	VAR_063359	p.Gly601Ser	LB/B	rs72658143	-
COL1A2	P08123	VAR_063360	p.Gly625Asp	LP/P	rs72658145	Osteogenesis imperfecta 2 (OI2) [MIM:166210]
COL1A2	P08123	VAR_063361	p.Gly676Asp	LP/P	rs66883877	Osteogenesis imperfecta 3 (OI3) [MIM:259420]
COL1A2	P08123	VAR_063363	p.Gly733Cys	LP/P	rs72658172	Osteogenesis imperfecta 1 (OI1) [MIM:166200]
COL1A2	P08123	VAR_063364	p.Gly739Arg	LP/P	rs72658174	Osteogenesis imperfecta 2 (OI2) [MIM:166210]
COL1A2	P08123	VAR_063365	p.Gly748Val	LP/P	-	Osteogenesis imperfecta 2 (OI2) [MIM:166210]
COL1A2	P08123	VAR_063366	p.Gly754Cys	LP/P	rs72658177	Osteogenesis imperfecta 4 (OI4) [MIM:166220]
COL1A2	P08123	VAR_063370	p.Gly820Ser	LP/P	rs72658191	Osteogenesis imperfecta 3 (OI3) [MIM:259420]
COL1A2	P08123	VAR_063371	p.Gly835Cys	LP/P	-	Osteogenesis imperfecta 3 (OI3) [MIM:259420]
COL1A2	P08123	VAR_063372	p.Gly856Arg	LP/P	-	Osteogenesis imperfecta 3 (OI3) [MIM:259420]
COL1A2	P08123	VAR_063373	p.Gly856Val	LP/P	-	Osteogenesis imperfecta 2 (OI2) [MIM:166210]

COL1A2	P08123	VAR_063374	p.Gly955Asp	LP/P	-	Osteogenesis imperfecta 2 (OI2) [MIM:166210]
COL1A2	P08123	VAR_063375	p.Gly982Asp	LP/P	rs67422093	Osteogenesis imperfecta 2 (OI2) [MIM:166210]
COL1A2	P08123	VAR_063377	p.Gly991Val	LP/P	rs72659316	Osteogenesis imperfecta 3 (OI3) [MIM:259420]
COL1A2	P08123	VAR_063378	p.Gly1003Asp	LP/P	rs1114167414	Osteogenesis imperfecta 2 (OI2) [MIM:166210]
COL1A2	P08123	VAR_063379	p.Gly1027Glu	LP/P	rs72659323	Osteogenesis imperfecta 2 (OI2) [MIM:166210]
COL1A2	P08123	VAR_063381	p.Gly1087Asp	LP/P	rs72659335	Osteogenesis imperfecta 3 (OI3) [MIM:259420]
COL1A2	P08123	VAR_063383	p.Cys1195Tyr	LP/P	rs72659342	Osteogenesis imperfecta 1 (OI1) [MIM:166200]
COL1A2	P08123	VAR_066386	p.Ala1119Thr	US	-	-
COL1A2	P08123	VAR_069633	p.Arg1067His	LB/B	rs530026906	-
COL20A1	Q9P218	VAR_055671	p.Pro134Leu	LB/B	rs753686	-
COL21A1	Q96P44	VAR_038555	p.Leu277Pro	LB/B	rs2764043	-
COL21A1	Q96P44	VAR_038556	p.Thr343Met	LB/B	rs35471617	-
COL21A1	Q96P44	VAR_038557	p.Ile495Thr	LB/B	rs35583895	-
COL21A1	Q96P44	VAR_038558	p.Gly560Ser	LB/B	rs9382581	-
COL21A1	Q96P44	VAR_038559	p.Ala747Asp	LB/B	rs9464337	-
COL21A1	Q96P44	VAR_038560	p.Leu821Pro	LB/B	rs12209452	-
COL21A1	Q96P44	VAR_038561	p.Pro827Ala	LB/B	rs1555131	-
COL22A1	Q8NFW1	VAR_038562	p.Ser320Gly	LB/B	rs2292927	-
COL22A1	Q8NFW1	VAR_038563	p.Pro703Thr	LB/B	rs10111520	-
COL22A1	Q8NFW1	VAR_038564	p.Ala938Asp	LB/B	rs4909444	-
COL23A1	Q86Y22	VAR_026964	p.Thr287Ala	LB/B	rs890802	-
COL24A1	Q17RW2	VAR_038565	p.Gly1423Arg	LB/B	rs7520146	-
COL24A1	Q17RW2	VAR_055672	p.Pro151Leu	LB/B	rs1027819	-
COL24A1	Q17RW2	VAR_055673	p.Ile293Thr	LB/B	rs17128866	-
COL24A1	Q17RW2	VAR_055674	p.Met481Leu	LB/B	rs10493784	-
COL24A1	Q17RW2	VAR_055675	p.Pro546Ser	LB/B	rs11161732	-
COL24A1	Q17RW2	VAR_055676	p.Pro731Ser	LB/B	rs641712	-
COL24A1	Q17RW2	VAR_061116	p.Arg641His	LB/B	rs60891279	-
COL24A1	Q17RW2	VAR_062865	p.Ala61Val	LB/B	rs11161747	-
COL25A1	Q9BXS0	VAR_073325	p.Gly382Arg	LP/P	rs780209390	Fibrosis of extraocular muscles, congenital, 5 (CFEOM5) [MIM:616219]
COL26A1	Q96A83	VAR_057530	p.Pro427Leu	LB/B	rs17471501	-
COL27A1	Q8IZC6	VAR_048784	p.Val89Ile	LB/B	rs2567707	-
COL27A1	Q8IZC6	VAR_048785	p.Gln120Arg	LB/B	rs2567706	-
COL27A1	Q8IZC6	VAR_048786	p.Ala265Thr	LB/B	rs34578955	-
COL27A1	Q8IZC6	VAR_048787	p.Arg349Cys	LB/B	rs34973417	-
COL27A1	Q8IZC6	VAR_048788	p.Ala422Thr	LB/B	rs2241671	-
COL27A1	Q8IZC6	VAR_048789	p.Ile537Thr	LB/B	rs2808770	-
COL27A1	Q8IZC6	VAR_048790	p.Ile611Phe	LB/B	rs2567705	-
COL27A1	Q8IZC6	VAR_048791	p.Pro720Arg	LB/B	rs35446342	-
COL27A1	Q8IZC6	VAR_048792	p.Pro1116Gln	LB/B	rs7048607	-
COL27A1	Q8IZC6	VAR_048793	p.Arg1348Gln	LB/B	rs1631319	-
COL27A1	Q8IZC6	VAR_048794	p.Arg1354Gln	LB/B	rs10982134	-
COL27A1	Q8IZC6	VAR_048795	p.Met1808Val	LB/B	rs3736252	-
COL27A1	Q8IZC6	VAR_072564	p.Gly697Arg	LP/P	rs140950220	Steel syndrome (STLS) [MIM:615155]
COL28A1	Q2UY09	VAR_038566	p.Ala189Gly	LB/B	rs7804532	-
COL28A1	Q2UY09	VAR_038567	p.Ile239Val	LB/B	rs10486180	-
COL28A1	Q2UY09	VAR_038568	p.Thr327Ser	LB/B	rs10486176	-
COL28A1	Q2UY09	VAR_038569	p.Glu433Asp	LB/B	rs6952195	-

COL28A1 Q2UY09 VAR_038570 p.Ala472Pro LB/B rs17167927 -
COL28A1 Q2UY09 VAR_038571 p.Arg741Gln LB/B rs17167102 -
COL28A1 Q2UY09 VAR_061117 p.Ile437Met LB/B rs55745506 -
COL2A1 P02458 VAR_001738 p.Gly267Asp LP/P rs121912872 Stickler syndrome 1 non-syndromic ocular (STL1O) [MIM:609508]
COL2A1 P02458 VAR_001739 p.Arg275Cys LP/P rs121912876 Czech dysplasia (CZECHD) [MIM:609162]
COL2A1 P02458 VAR_001741 p.Gly303Asp LP/P rs121912877 Kniest dysplasia (KD) [MIM:156550]
COL2A1 P02458 VAR_001742 p.Gly354Arg LB/B rs121912871 -
COL2A1 P02458 VAR_001743 p.Gly375Arg LP/P - Spondyloepiphyseal dysplasia congenital type (SEDC) [MIM:183900]
COL2A1 P02458 VAR_001744 p.Gly447Ser LP/P - Spondyloepiphyseal dysplasia congenital type (SEDC) [MIM:183900]
COL2A1 P02458 VAR_001745 p.Gly492Val LP/P rs121912881 Spondyloepimetaphyseal dysplasia, Strudwick type (SEMDSTWK) [MIM:184250]
COL2A1 P02458 VAR_001746 p.Gly504Cys LP/P rs121912880 Spondyloepimetaphyseal dysplasia, Strudwick type (SEMDSTWK) [MIM:184250]
COL2A1 P02458 VAR_001747 p.Gly510Asp LP/P - Achondrogenesis 2 (ACG2) [MIM:200610]
COL2A1 P02458 VAR_001748 p.Arg719Cys LP/P rs121912865 Osteoarthritis with mild chondrodysplasia (OSCDP) [MIM:604864]
COL2A1 P02458 VAR_001749 p.Gly774Ser LP/P rs121912867 Spondyloepiphyseal dysplasia congenital type (SEDC) [MIM:183900]
COL2A1 P02458 VAR_001751 p.Gly804Ala US - -
COL2A1 P02458 VAR_001752 p.Gly891Arg LP/P rs121912879 Achondrogenesis 2 (ACG2) [MIM:200610]
COL2A1 P02458 VAR_001752 p.Gly891Arg LP/P rs121912879 Spondyloepiphyseal dysplasia congenital type (SEDC) [MIM:183900]
COL2A1 P02458 VAR_001753 p.Gly909Cys LP/P rs121912875 Spondyloepimetaphyseal dysplasia, Strudwick type (SEMDSTWK) [MIM:184250]
COL2A1 P02458 VAR_001754 p.Gly969Ser LP/P rs121912878 Achondrogenesis 2 (ACG2) [MIM:200610]
COL2A1 P02458 VAR_001755 p.Arg989Cys LP/P rs121912874 Spondyloepiphyseal dysplasia congenital type (SEDC) [MIM:183900]
COL2A1 P02458 VAR_001756 p.Gly1005Ser LB/B rs753342774 -
COL2A1 P02458 VAR_001757 p.Gly1017Val LP/P - Achondrogenesis 2 (ACG2) [MIM:200610]
COL2A1 P02458 VAR_001758 p.Gly1053Glu LB/B rs121912868 -
COL2A1 P02458 VAR_001759 p.Gly1110Cys LP/P - Achondrogenesis 2 (ACG2) [MIM:200610]
COL2A1 P02458 VAR_001760 p.Gly1113Cys US - -
COL2A1 P02458 VAR_001761 p.Gly1143Ser LP/P - Achondrogenesis 2 (ACG2) [MIM:200610]
COL2A1 P02458 VAR_001763 p.Gly1176Ser LP/P - Spondyloepiphyseal dysplasia congenital type (SEDC) [MIM:183900]
COL2A1 P02458 VAR_001764 p.Gly1188Arg LP/P - Achondrogenesis 2 (ACG2) [MIM:200610]
COL2A1 P02458 VAR_001765 p.Gly1197Ser LP/P rs121912870 Spondyloepiphyseal dysplasia congenital type (SEDC) [MIM:183900]
COL2A1 P02458 VAR_017105 p.Thr1439Met LP/P rs121912886 Spondyloepiphyseal dysplasia congenital type (SEDC) [MIM:183900]
COL2A1 P02458 VAR_017638 p.Thr9Ser LB/B rs3803183 -
COL2A1 P02458 VAR_017639 p.Gly453Asp LP/P rs794727339 Achondrogenesis 2 (ACG2) [MIM:200610]
COL2A1 P02458 VAR_017640 p.Gly453Val LP/P - Achondrogenesis 2 (ACG2) [MIM:200610]
COL2A1 P02458 VAR_017641 p.Gly771Asp LP/P - Achondrogenesis 2 (ACG2) [MIM:200610]
COL2A1 P02458 VAR_017642 p.Gly780Arg LP/P - Achondrogenesis 2 (ACG2) [MIM:200610]
COL2A1 P02458 VAR_017643 p.Gly795Arg LP/P - Achondrogenesis 2 (ACG2) [MIM:200610]

COL2A1 P02458 VAR_017644 p.Gly894Glu LP/P - Achondrogenesis 2 (ACG2) [MIM:200610]
COL2A1 P02458 VAR_017645 p.Arg904Cys LP/P rs121912882 Multiple epiphyseal dysplasia with myopia and conductive deafness (EDMMD) [MIM:132450]
COL2A1 P02458 VAR_017645 p.Arg904Cys LP/P rs121912882 Stickler syndrome 1 (STL1) [MIM:108300]
COL2A1 P02458 VAR_017646 p.Gly948Asp LP/P - Achondrogenesis 2 (ACG2) [MIM:200610]
COL2A1 P02458 VAR_017647 p.Gly981Ser LP/P - Achondrogenesis 2 (ACG2) [MIM:200610]
COL2A1 P02458 VAR_017649 p.Gly1065Val LP/P - Achondrogenesis 2 (ACG2) [MIM:200610]
COL2A1 P02458 VAR_017650 p.Gly1119Arg LP/P - Achondrogenesis 2 (ACG2) [MIM:200610]
COL2A1 P02458 VAR_017651 p.Gly1173Arg LP/P rs121912883 Spondyloepiphyseal dysplasia congenital type (SEDC) [MIM:183900]
COL2A1 P02458 VAR_017652 p.Val1331Ile LB/B rs12721427 -
COL2A1 P02458 VAR_019836 p.Pro158Leu LB/B rs1050861 -
COL2A1 P02458 VAR_023925 p.Gly318Arg LP/P rs121912894 Rhegmatogenous retinal detachment autosomal dominant (DRRD) [MIM:609508]
COL2A1 P02458 VAR_023926 p.Gly516Asp LP/P rs121912888 Achondrogenesis 2 (ACG2) [MIM:200610]
COL2A1 P02458 VAR_023927 p.Arg565Cys LP/P rs121912884 Stickler syndrome 1 (STL1) [MIM:108300]
COL2A1 P02458 VAR_023928 p.Leu667Phe LP/P rs121912885 Rhegmatogenous retinal detachment autosomal dominant (DRRD) [MIM:609508]
COL2A1 P02458 VAR_023929 p.Gly717Ser LP/P rs387906558 Avascular necrosis of femoral head, primary, 1 (ANFH1) [MIM:608805]
COL2A1 P02458 VAR_023930 p.Gly855Ser LP/P rs1193507525 Spondyloepiphyseal dysplasia congenital type (SEDC) [MIM:183900]
COL2A1 P02458 VAR_023931 p.Gly897Val LP/P - Spondyloepimetaphyseal dysplasia, Strudwick type (SEMDSTWK) [MIM:184250]
COL2A1 P02458 VAR_023932 p.Arg992Gly LP/P rs121912895 Spondyloepimetaphyseal dysplasia, Strudwick type (SEMDSTWK) [MIM:184250]
COL2A1 P02458 VAR_023933 p.Gly1170Ser LP/P rs121912891 Avascular necrosis of femoral head, primary, 1 (ANFH1) [MIM:608805]
COL2A1 P02458 VAR_023933 p.Gly1170Ser LP/P rs121912891 Legg-Calve-Perthes disease (LCPD) [MIM:150600]
COL2A1 P02458 VAR_023934 p.Gly1305Asp LB/B rs121912887 -
COL2A1 P02458 VAR_023935 p.Tyr1391Cys LP/P rs121912889 Platyspondylic lethal skeletal dysplasia Torrance type (PLSD-T) [MIM:151210]
COL2A1 P02458 VAR_024819 p.Gly513Ser LP/P rs1555167156 Achondrogenesis 2 (ACG2) [MIM:200610]
COL2A1 P02458 VAR_024820 p.Gly717Val LP/P - Achondrogenesis 2 (ACG2) [MIM:200610]
COL2A1 P02458 VAR_024821 p.Gly771Ala LP/P - Achondrogenesis 2 (ACG2) [MIM:200610]
COL2A1 P02458 VAR_024822 p.Thr1390Asn LP/P - Platyspondylic lethal skeletal dysplasia Torrance type (PLSD-T) [MIM:151210]
COL2A1 P02458 VAR_024823 p.Thr1448Pro LP/P - Platyspondylic lethal skeletal dysplasia Torrance type (PLSD-T) [MIM:151210]
COL2A1 P02458 VAR_024824 p.Asp1469His LP/P - Platyspondylic lethal skeletal dysplasia Torrance type (PLSD-T) [MIM:151210]
COL2A1 P02458 VAR_024826 p.Cys1485Gly LP/P - Platyspondylic lethal skeletal dysplasia Torrance type (PLSD-T) [MIM:151210]
COL2A1 P02458 VAR_033782 p.Glu142Asp LB/B rs34392760 -
COL2A1 P02458 VAR_033783 p.Thr638Ile LB/B rs41263847 -
COL2A1 P02458 VAR_033784 p.Ala1051Thr LB/B rs41272041 -
COL2A1 P02458 VAR_033785 p.Gly1405Ser LB/B rs2070739 -

COL2A1 P02458 VAR_063891 p.Cys57Tyr LP/P rs121912898 Stickler syndrome 1 non-syndromic ocular (STL1O) [MIM:609508]
COL2A1 P02458 VAR_063892 p.Gly240Asp LP/P rs1592232040 Stickler syndrome 1 (STL1) [MIM:108300]
COL2A1 P02458 VAR_063893 p.Gly270Arg LP/P - Stickler syndrome 1 (STL1) [MIM:108300]
COL2A1 P02458 VAR_063894 p.Gly282Asp LP/P - Stickler syndrome 1 (STL1) [MIM:108300]
COL2A1 P02458 VAR_063895 p.Gly453Ala LP/P rs794727339 Stickler syndrome 1 (STL1) [MIM:108300]
COL2A1 P02458 VAR_063896 p.Gly501Arg LP/P - Stickler syndrome 1 (STL1) [MIM:108300]
COL2A1 P02458 VAR_063897 p.Asp547Val LP/P - Achondrogenesis 2 (ACG2) [MIM:200610]
COL2A1 P02458 VAR_063898 p.Gly1158Ala LP/P - Stickler syndrome 1 (STL1) [MIM:108300]
COL2A1 P02458 VAR_066836 p.Gly1176Val US - -
COL2A1 P02458 VAR_066837 p.Gly1179Arg US - -
COL2A1 P02458 VAR_075729 p.Gly207Arg LP/P rs869312907 Spondyloepiphyseal dysplasia, Stanescu type (SEDSTN) [MIM:616583]
COL2A1 P02458 VAR_075730 p.Thr1383Met LP/P rs138498898 Avascular necrosis of femoral head, primary, 1 (ANFH1) [MIM:608805]
COL2A1 P02458 VAR_079748 p.Arg1459Cys LB/B rs148838496 -
COL3A1 P02461 VAR_001767 p.Leu169Phe LB/B rs111391222 -
COL3A1 P02461 VAR_001768 p.Gly183Cys LP/P rs121912926 Ehlers-Danlos syndrome, vascular type (EDSVASC) [MIM:130050]
COL3A1 P02461 VAR_001769 p.Gly201Arg LP/P rs587779436 Ehlers-Danlos syndrome, vascular type (EDSVASC) [MIM:130050]
COL3A1 P02461 VAR_001770 p.Gly228Glu LP/P rs587779555 Ehlers-Danlos syndrome, vascular type (EDSVASC) [MIM:130050]
COL3A1 P02461 VAR_001771 p.Gly303Arg LP/P rs121912919 Ehlers-Danlos syndrome, vascular type (EDSVASC) [MIM:130050]
COL3A1 P02461 VAR_001772 p.Gly540Arg LP/P rs587779584 Ehlers-Danlos syndrome, vascular type (EDSVASC) [MIM:130050]
COL3A1 P02461 VAR_001773 p.Gly567Glu LP/P - Ehlers-Danlos syndrome, vascular type (EDSVASC) [MIM:130050]
COL3A1 P02461 VAR_001774 p.Gly582Ser LP/P rs121912923 Ehlers-Danlos syndrome, vascular type (EDSVASC) [MIM:130050]
COL3A1 P02461 VAR_001775 p.Pro602Thr LB/B rs35795890 -
COL3A1 P02461 VAR_001776 p.Pro635Leu LB/B rs902780774 -
COL3A1 P02461 VAR_001777 p.Gly666Asp LP/P rs121912921 Ehlers-Danlos syndrome, vascular type (EDSVASC) [MIM:130050]
COL3A1 P02461 VAR_001778 p.Ala698Thr LB/B rs1800255 -
COL3A1 P02461 VAR_001779 p.Gly726Arg LP/P rs587779638 Ehlers-Danlos syndrome, vascular type (EDSVASC) [MIM:130050]
COL3A1 P02461 VAR_001780 p.Gly756Glu LP/P - Ehlers-Danlos syndrome, vascular type (EDSVASC) [MIM:130050]
COL3A1 P02461 VAR_001781 p.Gly762Cys LP/P - Ehlers-Danlos syndrome, vascular type (EDSVASC) [MIM:130050]
COL3A1 P02461 VAR_001782 p.Gly786Arg LP/P rs113485686 Ehlers-Danlos syndrome, vascular type (EDSVASC) [MIM:130050]
COL3A1 P02461 VAR_001783 p.Gly804Ser LP/P rs121912920 Ehlers-Danlos syndrome, vascular type (EDSVASC) [MIM:130050]
COL3A1 P02461 VAR_001784 p.Gly828Arg LP/P - Ehlers-Danlos syndrome, vascular type (EDSVASC) [MIM:130050]

COL3A1 P02461 VAR_001785 p.Gly909Asp LP/P - Ehlers-Danlos syndrome, vascular type (EDSVASC) [MIM:130050]
COL3A1 P02461 VAR_001786 p.Gly936Arg LP/P rs587779566 Ehlers-Danlos syndrome, vascular type (EDSVASC) [MIM:130050]
COL3A1 P02461 VAR_001787 p.Gly936Ser LP/P - Ehlers-Danlos syndrome, vascular type (EDSVASC) [MIM:130050]
COL3A1 P02461 VAR_001788 p.Gly939Asp LP/P rs112978464 Ehlers-Danlos syndrome, vascular type (EDSVASC) [MIM:130050]
COL3A1 P02461 VAR_001789 p.Gly957Ser LP/P rs121912913 Ehlers-Danlos syndrome, vascular type (EDSVASC) [MIM:130050]
COL3A1 P02461 VAR_001790 p.Gly960Val LP/P rs121912922 Ehlers-Danlos syndrome, vascular type (EDSVASC) [MIM:130050]
COL3A1 P02461 VAR_001791 p.Gly996Glu LP/P rs587779576 Ehlers-Danlos syndrome, vascular type (EDSVASC) [MIM:130050]
COL3A1 P02461 VAR_001792 p.Gly1014Glu LP/P rs121912916 Ehlers-Danlos syndrome, vascular type (EDSVASC) [MIM:130050]
COL3A1 P02461 VAR_001793 p.Gly1050Asp LP/P rs121912914 Ehlers-Danlos syndrome, vascular type (EDSVASC) [MIM:130050]
COL3A1 P02461 VAR_001794 p.Gly1071Val LP/P rs587779709 Ehlers-Danlos syndrome, vascular type (EDSVASC) [MIM:130050]
COL3A1 P02461 VAR_001795 p.Gly1077Val LP/P rs121912915 Ehlers-Danlos syndrome, vascular type (EDSVASC) [MIM:130050]
COL3A1 P02461 VAR_001796 p.Gly1101Glu LP/P rs121912924 Ehlers-Danlos syndrome, vascular type (EDSVASC) [MIM:130050]
COL3A1 P02461 VAR_001797 p.Gly1104Ala LP/P - Ehlers-Danlos syndrome, vascular type (EDSVASC) [MIM:130050]
COL3A1 P02461 VAR_001798 p.Gly1164Ser US - -
COL3A1 P02461 VAR_001799 p.Gly1167Val LP/P rs587779578 Ehlers-Danlos syndrome, vascular type (EDSVASC) [MIM:130050]
COL3A1 P02461 VAR_001800 p.Gly1170Asp LP/P rs587779465 Ehlers-Danlos syndrome, vascular type (EDSVASC) [MIM:130050]
COL3A1 P02461 VAR_001801 p.Gly1173Glu LP/P rs121912918 Ehlers-Danlos syndrome, vascular type (EDSVASC) [MIM:130050]
COL3A1 P02461 VAR_001802 p.Gly1176Val LP/P - Ehlers-Danlos syndrome, vascular type (EDSVASC) [MIM:130050]
COL3A1 P02461 VAR_001803 p.Gly1182Glu LP/P rs111505097 Ehlers-Danlos syndrome, vascular type (EDSVASC) [MIM:130050]
COL3A1 P02461 VAR_001804 p.Gly1185Asp LP/P rs121912917 Ehlers-Danlos syndrome, vascular type (EDSVASC) [MIM:130050]
COL3A1 P02461 VAR_001805 p.Gly1185Val LP/P rs121912917 Ehlers-Danlos syndrome, vascular type (EDSVASC) [MIM:130050]
COL3A1 P02461 VAR_001806 p.Gly1188Glu LP/P rs112456072 Ehlers-Danlos syndrome, vascular type (EDSVASC) [MIM:130050]
COL3A1 P02461 VAR_001807 p.Gly1188Arg LP/P rs587779504 Ehlers-Danlos syndrome, vascular type (EDSVASC) [MIM:130050]
COL3A1 P02461 VAR_011095 p.Gly183Asp LP/P rs587779420 Ehlers-Danlos syndrome, vascular type (EDSVASC) [MIM:130050]
COL3A1 P02461 VAR_011096 p.Gly183Ser LP/P rs121912926 Ehlers-Danlos syndrome, vascular type (EDSVASC) [MIM:130050]

COL3A1 P02461 VAR_011097 p.Gly192Val LP/P rs587779710 Ehlers-Danlos syndrome, vascular type (EDSVASC) [MIM:130050]
COL3A1 P02461 VAR_011098 p.Gly204Asp LP/P rs587779626 Ehlers-Danlos syndrome, vascular type (EDSVASC) [MIM:130050]
COL3A1 P02461 VAR_011099 p.Gly204Ser LP/P rs587779711 Ehlers-Danlos syndrome, vascular type (EDSVASC) [MIM:130050]
COL3A1 P02461 VAR_011100 p.Gly210Asp LP/P - Ehlers-Danlos syndrome, vascular type (EDSVASC) [MIM:130050]
COL3A1 P02461 VAR_011101 p.Gly219Cys LP/P rs587779624 Ehlers-Danlos syndrome, vascular type (EDSVASC) [MIM:130050]
COL3A1 P02461 VAR_011102 p.Gly225Val LP/P rs587779533 Ehlers-Danlos syndrome, vascular type (EDSVASC) [MIM:130050]
COL3A1 P02461 VAR_011103 p.Gly240Arg LP/P rs587779468 Ehlers-Danlos syndrome, vascular type (EDSVASC) [MIM:130050]
COL3A1 P02461 VAR_011104 p.Gly243Val LP/P rs587779629 Ehlers-Danlos syndrome, vascular type (EDSVASC) [MIM:130050]
COL3A1 P02461 VAR_011105 p.Gly249Asp LP/P rs121912927 Ehlers-Danlos syndrome, vascular type (EDSVASC) [MIM:130050]
COL3A1 P02461 VAR_011106 p.Gly249Val LP/P rs121912927 Ehlers-Danlos syndrome, vascular type (EDSVASC) [MIM:130050]
COL3A1 P02461 VAR_011107 p.Gly252Asp LP/P rs587779464 Ehlers-Danlos syndrome, vascular type (EDSVASC) [MIM:130050]
COL3A1 P02461 VAR_011108 p.Gly252Arg LP/P rs587779705 Ehlers-Danlos syndrome, vascular type (EDSVASC) [MIM:130050]
COL3A1 P02461 VAR_011109 p.Gly252Val LP/P rs587779464 Ehlers-Danlos syndrome, vascular type (EDSVASC) [MIM:130050]
COL3A1 P02461 VAR_011110 p.Gly255Val LP/P rs587779605 Ehlers-Danlos syndrome, vascular type (EDSVASC) [MIM:130050]
COL3A1 P02461 VAR_011111 p.Gly264Arg LP/P - Ehlers-Danlos syndrome, vascular type (EDSVASC) [MIM:130050]
COL3A1 P02461 VAR_011112 p.Gly267Val LP/P rs587779427 Ehlers-Danlos syndrome, vascular type (EDSVASC) [MIM:130050]
COL3A1 P02461 VAR_011113 p.Gly321Val LP/P rs587779588 Ehlers-Danlos syndrome, vascular type (EDSVASC) [MIM:130050]
COL3A1 P02461 VAR_011114 p.Gly327Asp LP/P - Ehlers-Danlos syndrome, vascular type (EDSVASC) [MIM:130050]
COL3A1 P02461 VAR_011115 p.Gly345Arg LP/P rs587779419 Ehlers-Danlos syndrome, vascular type (EDSVASC) [MIM:130050]
COL3A1 P02461 VAR_011116 p.Gly417Arg LP/P rs587779637 Ehlers-Danlos syndrome, vascular type (EDSVASC) [MIM:130050]
COL3A1 P02461 VAR_011117 p.Gly444Arg LP/P rs587779489 Ehlers-Danlos syndrome, vascular type (EDSVASC) [MIM:130050]
COL3A1 P02461 VAR_011118 p.Gly489Glu LP/P rs587779476 Ehlers-Danlos syndrome, vascular type (EDSVASC) [MIM:130050]
COL3A1 P02461 VAR_011119 p.Gly501Arg LP/P rs587779523 Ehlers-Danlos syndrome, vascular type (EDSVASC) [MIM:130050]
COL3A1 P02461 VAR_011120 p.Gly519Val LP/P - Ehlers-Danlos syndrome, vascular type (EDSVASC) [MIM:130050]

COL3A1 P02461 VAR_011121 p.Gly549Glu LP/P rs587779679 Ehlers-Danlos syndrome, vascular type (EDSVASC) [MIM:130050]
COL3A1 P02461 VAR_011122 p.Gly552Glu LP/P rs121912928 Ehlers-Danlos syndrome, vascular type (EDSVASC) [MIM:130050]
COL3A1 P02461 VAR_011123 p.Gly588Asp LP/P rs587779691 Ehlers-Danlos syndrome, vascular type (EDSVASC) [MIM:130050]
COL3A1 P02461 VAR_011124 p.Gly636Arg LP/P rs587779522 Ehlers-Danlos syndrome, vascular type (EDSVASC) [MIM:130050]
COL3A1 P02461 VAR_011125 p.Gly657Glu LP/P rs587779699 Ehlers-Danlos syndrome, vascular type (EDSVASC) [MIM:130050]
COL3A1 P02461 VAR_011126 p.Gly660Asp LP/P rs587779493 Ehlers-Danlos syndrome, vascular type (EDSVASC) [MIM:130050]
COL3A1 P02461 VAR_011127 p.Pro668Thr LB/B rs1801183 -
COL3A1 P02461 VAR_011128 p.Gly699Arg LP/P rs587779668 Ehlers-Danlos syndrome, vascular type (EDSVASC) [MIM:130050]
COL3A1 P02461 VAR_011129 p.Gly738Ser LP/P rs121912925 Ehlers-Danlos syndrome, vascular type (EDSVASC) [MIM:130050]
COL3A1 P02461 VAR_011130 p.Gly738Val LP/P rs587779615 Ehlers-Danlos syndrome, vascular type (EDSVASC) [MIM:130050]
COL3A1 P02461 VAR_011131 p.Gly744Val LP/P rs587779697 Ehlers-Danlos syndrome, vascular type (EDSVASC) [MIM:130050]
COL3A1 P02461 VAR_011132 p.Gly828Trp LP/P rs587779486 Ehlers-Danlos syndrome, vascular type (EDSVASC) [MIM:130050]
COL3A1 P02461 VAR_011133 p.Gly852Cys LP/P rs587779690 Ehlers-Danlos syndrome, vascular type (EDSVASC) [MIM:130050]
COL3A1 P02461 VAR_011134 p.Gly879Val LP/P rs587779645 Ehlers-Danlos syndrome, vascular type (EDSVASC) [MIM:130050]
COL3A1 P02461 VAR_011135 p.Gly882Asp LP/P rs587779622 Ehlers-Danlos syndrome, vascular type (EDSVASC) [MIM:130050]
COL3A1 P02461 VAR_011136 p.Gly900Asp LP/P rs587779599 Ehlers-Danlos syndrome, vascular type (EDSVASC) [MIM:130050]
COL3A1 P02461 VAR_011137 p.Gly903Glu LP/P rs587779505 Ehlers-Danlos syndrome, vascular type (EDSVASC) [MIM:130050]
COL3A1 P02461 VAR_011138 p.Gly909Val LP/P rs587779483 Ehlers-Danlos syndrome, vascular type (EDSVASC) [MIM:130050]
COL3A1 P02461 VAR_011139 p.Gly918Glu LP/P rs587779662 Ehlers-Danlos syndrome, vascular type (EDSVASC) [MIM:130050]
COL3A1 P02461 VAR_011140 p.Gly924Cys LP/P rs587779471 Ehlers-Danlos syndrome, vascular type (EDSVASC) [MIM:130050]
COL3A1 P02461 VAR_011141 p.Gly942Glu LP/P rs587779517 Ehlers-Danlos syndrome, vascular type (EDSVASC) [MIM:130050]
COL3A1 P02461 VAR_011142 p.Gly966Val LP/P rs587779571 Ehlers-Danlos syndrome, vascular type (EDSVASC) [MIM:130050]
COL3A1 P02461 VAR_011143 p.Gly972Ala LP/P rs587779559 Ehlers-Danlos syndrome, vascular type (EDSVASC) [MIM:130050]
COL3A1 P02461 VAR_011144 p.Gly984Thr LP/P rs1559061242 Ehlers-Danlos syndrome, vascular type (EDSVASC) [MIM:130050]
COL3A1 P02461 VAR_011145 p.Gly999Arg LP/P rs587779548 Ehlers-Danlos syndrome, vascular type (EDSVASC) [MIM:130050]

COL3A1 P02461 VAR_011146 p.Gly1011Glu LP/P rs587779552 Ehlers-Danlos syndrome, vascular type (EDSVASC) [MIM:130050]
COL3A1 P02461 VAR_011147 p.Gly1032Val LP/P rs587779428 Ehlers-Danlos syndrome, vascular type (EDSVASC) [MIM:130050]
COL3A1 P02461 VAR_011148 p.Gly1035Cys LP/P rs587779704 Ehlers-Danlos syndrome, vascular type (EDSVASC) [MIM:130050]
COL3A1 P02461 VAR_011149 p.Gly1044Asp LP/P - Ehlers-Danlos syndrome, vascular type (EDSVASC) [MIM:130050]
COL3A1 P02461 VAR_011150 p.Gly1050Val LP/P rs121912914 Ehlers-Danlos syndrome, vascular type (EDSVASC) [MIM:130050]
COL3A1 P02461 VAR_011151 p.Gly1089Asp LP/P rs587779672 Ehlers-Danlos syndrome, vascular type (EDSVASC) [MIM:130050]
COL3A1 P02461 VAR_011152 p.Gly1098Asp LP/P - Ehlers-Danlos syndrome, vascular type (EDSVASC) [MIM:130050]
COL3A1 P02461 VAR_011153 p.Gly1098Val LP/P rs587779614 Ehlers-Danlos syndrome, vascular type (EDSVASC) [MIM:130050]
COL3A1 P02461 VAR_011154 p.Gly1161Val LP/P rs587779473 Ehlers-Danlos syndrome, vascular type (EDSVASC) [MIM:130050]
COL3A1 P02461 VAR_011155 p.Gly1164Glu LP/P rs587779431 Ehlers-Danlos syndrome, vascular type (EDSVASC) [MIM:130050]
COL3A1 P02461 VAR_011156 p.Gly1164Arg LP/P rs587779553 Ehlers-Danlos syndrome, vascular type (EDSVASC) [MIM:130050]
COL3A1 P02461 VAR_011157 p.Gly1170Val LP/P rs587779465 Ehlers-Danlos syndrome, vascular type (EDSVASC) [MIM:130050]
COL3A1 P02461 VAR_011158 p.Gly1173Arg LP/P rs587779521 Ehlers-Danlos syndrome, vascular type (EDSVASC) [MIM:130050]
COL3A1 P02461 VAR_011159 p.Gly1179Arg LP/P rs587779574 Ehlers-Danlos syndrome, vascular type (EDSVASC) [MIM:130050]
COL3A1 P02461 VAR_020012 p.Ile1205Val LB/B rs2271683 -
COL3A1 P02461 VAR_030115 p.His1353Gln LB/B rs1516446 -
COL3A1 P02461 VAR_035738 p.Gly420Ser US rs587779692 A colorectal cancer sample
COL3A1 P02461 VAR_035739 p.Arg1434Cys US rs747324731 A colorectal cancer sample
COL3A1 P02461 VAR_037007 p.Gly297Arg LP/P rs1553507557 Ehlers-Danlos syndrome, vascular type (EDSVASC) [MIM:130050]
COL3A1 P02461 VAR_055665 p.Gly534Glu LB/B rs41263744 -
COL3A1 P02461 VAR_055666 p.Ala679Thr LB/B rs41263773 -
COL3A1 P02461 VAR_055667 p.Pro686Ala LB/B rs41263775 -
COL3A1 P02461 VAR_082043 p.Pro49Ala LP/P rs1234344050 Polymicrogyria with or without vascular-type Ehlers-Danlos syndrome (PMGEDSV) [MIM:618343]
COL3A1 P02461 VAR_082046 p.Gly1284Glu LP/P rs587779528 Polymicrogyria with or without vascular-type Ehlers-Danlos syndrome (PMGEDSV) [MIM:618343]
COL4A1 P02462 VAR_020013 p.Gln1334His LB/B rs3742207 -
COL4A1 P02462 VAR_030027 p.Val7Leu LB/B rs9515185 -
COL4A1 P02462 VAR_030028 p.Gly562Glu LP/P rs121912857 Brain small vessel disease 1 with or without ocular anomalies (BSVD1) [MIM:175780]
COL4A1 P02462 VAR_030029 p.Gly749Ser LP/P rs113994109 Brain small vessel disease 1 with or without ocular anomalies (BSVD1) [MIM:175780]
COL4A1 P02462 VAR_030030 p.Gly1130Asp LP/P rs113994111 Brain small vessel disease 1 with or without ocular anomalies (BSVD1) [MIM:175780]

COL4A1 P02462 VAR_030031 p.Gly1236Arg LP/P rs113994112 Brain small vessel disease 1 with or without ocular anomalies (BSVD1) [MIM:175780]
COL4A1 P02462 VAR_030032 p.Gly1423Arg LP/P rs113994113 Brain small vessel disease 1 with or without ocular anomalies (BSVD1) [MIM:175780]
COL4A1 P02462 VAR_030511 p.Pro555Thr LB/B rs536174 -
COL4A1 P02462 VAR_044158 p.Pro304Leu LB/B rs34843786 -
COL4A1 P02462 VAR_044159 p.Gly498Val LP/P rs113994104 Hereditary angiopathy with nephropathy aneurysms and muscle cramps (HANAC) [MIM:611773]
COL4A1 P02462 VAR_044160 p.Gly519Arg LP/P rs113994105 Hereditary angiopathy with nephropathy aneurysms and muscle cramps (HANAC) [MIM:611773]
COL4A1 P02462 VAR_044161 p.Gly528Glu LP/P rs113994106 Hereditary angiopathy with nephropathy aneurysms and muscle cramps (HANAC) [MIM:611773]
COL4A1 P02462 VAR_064493 p.Gly498Arg LP/P rs267606744 Hereditary angiopathy with nephropathy aneurysms and muscle cramps (HANAC) [MIM:611773]
COL4A1 P02462 VAR_064494 p.Gly510Arg LP/P rs267606743 Hereditary angiopathy with nephropathy aneurysms and muscle cramps (HANAC) [MIM:611773]
COL4A1 P02462 VAR_064494 p.Gly510Arg LP/P rs267606743 Tortuosity of retinal arteries (RATOR) [MIM:180000]
COL4A1 P02462 VAR_064495 p.Gly525Leu LP/P rs281865426 Hereditary angiopathy with nephropathy aneurysms and muscle cramps (HANAC) [MIM:611773]
COL4A1 P02462 VAR_064496 p.Gly720Asp LP/P rs113994108 Brain small vessel disease 1 with or without ocular anomalies (BSVD1) [MIM:175780]
COL4A1 P02462 VAR_064497 p.Gly755Arg LP/P rs672601346 Brain small vessel disease 1 with or without ocular anomalies (BSVD1) [MIM:175780]
COL4A1 P02462 VAR_064498 p.Gly805Arg LP/P rs113994110 Brain small vessel disease 1 with or without ocular anomalies (BSVD1) [MIM:175780]
COL4A1 P02462 VAR_064499 p.Gly1580Arg LP/P rs113994114 Brain small vessel disease 1 with or without ocular anomalies (BSVD1) [MIM:175780]
COL4A1 P02462 VAR_073809 p.Ala144Val LB/B - -
COL4A1 P02462 VAR_073810 p.Pro352Leu LP/P rs200786329 Intracerebral hemorrhage (ICH) [MIM:614519]
COL4A1 P02462 VAR_073811 p.Arg538Gly LP/P rs397514624 Intracerebral hemorrhage (ICH) [MIM:614519]
COL4A1 P02462 VAR_073812 p.Gly655Arg LP/P - Schizencephaly (SCHZC) [MIM:269160]
COL4A1 P02462 VAR_073813 p.Gly708Arg LP/P rs672601349 Brain small vessel disease 1 with or without ocular anomalies (BSVD1) [MIM:175780]
COL4A1 P02462 VAR_073814 p.Gly773Arg LP/P rs672601347 Brain small vessel disease 1 with or without ocular anomalies (BSVD1) [MIM:175780]
COL4A1 P02462 VAR_073815 p.Gly870Arg LP/P - Schizencephaly (SCHZC) [MIM:269160]
COL4A1 P02462 VAR_073816 p.Gly882Asp LP/P - Brain small vessel disease 1 with or without ocular anomalies (BSVD1) [MIM:175780]
COL4A1 P02462 VAR_073817 p.Gly897Ser LP/P - Schizencephaly (SCHZC) [MIM:269160]
COL4A1 P02462 VAR_073818 p.Gly948Ser LP/P rs1555303073 Schizencephaly (SCHZC) [MIM:269160]
COL4A1 P02462 VAR_073819 p.Gly1041Glu LP/P - Schizencephaly (SCHZC) [MIM:269160]
COL4A1 P02462 VAR_073820 p.Gly1082Glu LP/P - Schizencephaly (SCHZC) [MIM:269160]
COL4A1 P02462 VAR_073821 p.Gly1266Arg LP/P - Brain small vessel disease 1 with or without ocular anomalies (BSVD1) [MIM:175780]
COL4A1 P02462 VAR_073822 p.Gly1326Arg LP/P rs587777379 Schizencephaly (SCHZC) [MIM:269160]
COL4A1 P02462 VAR_073823 p.Gly1332Asp LP/P - Schizencephaly (SCHZC) [MIM:269160]
COL4A1 P02462 VAR_073824 p.Met1531Val LB/B rs1343193102 -
COL4A1 P02462 VAR_073825 p.Glu1615Lys LP/P - Schizencephaly (SCHZC) [MIM:269160]

COL4A1 P02462 VAR_073826 p.Asn1627Lys LP/P rs672601348 Brain small vessel disease 1 with or without ocular anomalies (BSVD1) [MIM:175780]
COL4A2 P08572 VAR_048796 p.Arg517Lys LB/B rs7990383 -
COL4A2 P08572 VAR_048797 p.Gly683Ala LB/B rs3803230 -
COL4A2 P08572 VAR_067551 p.Val192Phe LB/B rs62621885 -
COL4A2 P08572 VAR_067552 p.Lys701Arg LB/B rs78829338 -
COL4A2 P08572 VAR_067553 p.Arg1109Gln LB/B rs184812559 -
COL4A2 P08572 VAR_067554 p.Glu1123Gly LB/B rs117412802 -
COL4A2 P08572 VAR_067555 p.Gln1150Lys LB/B rs62621875 -
COL4A2 P08572 VAR_067556 p.Gly1389Arg LP/P - -
COL4A2 P08572 VAR_067557 p.Val1399Ile LB/B rs45520539 -
COL4A2 P08572 VAR_067558 p.Ala1690Thr LB/B rs201105747 -
COL4A2 P08572 VAR_067836 p.Pro718Ser LB/B rs9583500 -
COL4A2 P08572 VAR_067837 p.Gly1037Glu LP/P rs387906603 Brain small vessel disease 2 (BSVD2) [MIM:614483]
COL4A2 P08572 VAR_067838 p.Gly1152Asp LP/P rs387906602 Brain small vessel disease 2 (BSVD2) [MIM:614483]
COL4A3 Q01955 VAR_001908 p.Leu1474Pro LB/B rs200302125 -
COL4A3 Q01955 VAR_001909 p.Gln1495Arg LB/B rs77964815 -
COL4A3 Q01955 VAR_011202 p.Gly43Arg LB/B rs13424243 -
COL4A3 Q01955 VAR_011203 p.Glu162Gly LB/B rs6436669 -
COL4A3 Q01955 VAR_011204 p.Gly297Glu LP/P - Alport syndrome 2, autosomal recessive (ATS2) [MIM:203780]
COL4A3 Q01955 VAR_011205 p.Asp326Tyr LB/B rs55703767 -
COL4A3 Q01955 VAR_011206 p.Gly407Arg LP/P - Alport syndrome 2, autosomal recessive (ATS2) [MIM:203780]
COL4A3 Q01955 VAR_011207 p.Arg408His LB/B rs34505188 -
COL4A3 Q01955 VAR_011208 p.His451Arg LB/B rs11677877 -
COL4A3 Q01955 VAR_011209 p.Pro574Leu LB/B rs28381984 -
COL4A3 Q01955 VAR_011210 p.Gly640Arg LP/P rs200672668 Alport syndrome 2, autosomal recessive (ATS2) [MIM:203780]
COL4A3 Q01955 VAR_011211 p.Gly1167Arg LP/P rs267606745 Alport syndrome 3, autosomal dominant (ATS3) [MIM:104200]
COL4A3 Q01955 VAR_011212 p.Gly1207Glu LP/P - Alport syndrome 2, autosomal recessive (ATS2) [MIM:203780]
COL4A3 Q01955 VAR_011213 p.Arg1215Gln US rs200443942 Alport syndrome 2, autosomal recessive (ATS2) [MIM:203780]
COL4A3 Q01955 VAR_011214 p.Asp1269Glu LB/B rs57611801 -
COL4A3 Q01955 VAR_011215 p.Gly1277Ser LP/P rs190598500 Alport syndrome 2, autosomal recessive (ATS2) [MIM:203780]
COL4A3 Q01955 VAR_011216 p.Ile1330Thr US rs767033956 Alport syndrome 2, autosomal recessive (ATS2) [MIM:203780]
COL4A3 Q01955 VAR_011217 p.Gly1334Glu LP/P rs375290088 Alport syndrome 2, autosomal recessive (ATS2) [MIM:203780]
COL4A3 Q01955 VAR_011218 p.Asp1347Glu US rs73996414 Alport syndrome 2, autosomal recessive (ATS2) [MIM:203780]
COL4A3 Q01955 VAR_011219 p.Arg1661Cys US rs201697532 Alport syndrome 2, autosomal recessive (ATS2) [MIM:203780]
COL4A3 Q01955 VAR_030944 p.Leu141Pro LB/B rs10178458 -

COL4A3 Q01955 VAR_030945 p.Gly532Asp LP/P rs371405814 Alport syndrome 2, autosomal recessive (ATS2)
[MIM:203780]
COL4A3 Q01955 VAR_030946 p.Gly739Arg LP/P rs375040636 Alport syndrome 2, autosomal recessive (ATS2)
[MIM:203780]
COL4A3 Q01955 VAR_030947 p.Gly853Arg LP/P rs763726708 Alport syndrome 2, autosomal recessive (ATS2)
[MIM:203780]
COL4A3 Q01955 VAR_030948 p.Gly985Val LP/P rs121912827 Hematuria, benign familial (BFH) [MIM:141200]
COL4A3 Q01955 VAR_030949 p.Gly1015Glu LP/P rs121912826 Hematuria, benign familial (BFH) [MIM:141200]
COL4A3 Q01955 VAR_030950 p.Gly1216Arg LP/P - Alport syndrome 2, autosomal recessive (ATS2)
[MIM:203780]
COL4A3 Q01955 VAR_061118 p.Lys834Arg LB/B rs56226424 -
COL4A3 Q01955 VAR_080826 p.Gly631Val US - Alport syndrome 2, autosomal recessive (ATS2)
[MIM:203780]
COL4A4 P53420 VAR_001912 p.Gly897Glu LP/P rs121912860 Hematuria, benign familial (BFH) [MIM:141200]
COL4A4 P53420 VAR_001913 p.Gly1201Ser LP/P rs121912858 Alport syndrome 2, autosomal recessive (ATS2)
[MIM:203780]
COL4A4 P53420 VAR_008149 p.Gly545Ala LB/B rs1800516 -
COL4A4 P53420 VAR_008150 p.Glu570Gln LB/B - -
COL4A4 P53420 VAR_008151 p.Ala931Thr LB/B rs75875272 -
COL4A4 P53420 VAR_008152 p.Pro1004Leu LB/B rs1800517 -
COL4A4 P53420 VAR_008153 p.Gly1030Val LP/P rs772699709 Alport syndrome 2, autosomal recessive (ATS2)
[MIM:203780]
COL4A4 P53420 VAR_008154 p.Pro1402Ser LB/B - -
COL4A4 P53420 VAR_008155 p.Pro1572Leu LP/P rs121912863 Alport syndrome 2, autosomal recessive (ATS2)
[MIM:203780]
COL4A4 P53420 VAR_022069 p.Pro482Ser LB/B rs2229814 -
COL4A4 P53420 VAR_031622 p.Ile6Thr LB/B rs16823264 -
COL4A4 P53420 VAR_031623 p.Gly116Glu LP/P rs1553696235 Hematuria, benign familial (BFH) [MIM:141200]
COL4A4 P53420 VAR_031624 p.Gly960Arg LP/P rs769783985 Hematuria, benign familial (BFH) [MIM:141200]
COL4A4 P53420 VAR_031625 p.Gly999Glu LP/P rs13027659 Hematuria, benign familial (BFH) [MIM:141200]
COL4A4 P53420 VAR_031626 p.Pro1132Leu LP/P - Hematuria, benign familial (BFH) [MIM:141200]
COL4A4 P53420 VAR_031627 p.Val1327Met LB/B rs2229813 -
COL4A4 P53420 VAR_031628 p.Ser1403Pro LB/B rs3752895 -
COL4A4 P53420 VAR_055680 p.Glu594Gly LB/B rs35998949 -
COL4A4 P53420 VAR_055681 p.Val670Ile LB/B rs34236495 -
COL4A4 P53420 VAR_055682 p.Pro759Leu LB/B rs36121515 -
COL4A5 P29400 VAR_001914 p.Gly54Asp LP/P rs104886043 Alport syndrome 1, X-linked (ATS1)
[MIM:301050]
COL4A5 P29400 VAR_001915 p.Gly129Glu LP/P rs281874723 Alport syndrome 1, X-linked (ATS1)
[MIM:301050]
COL4A5 P29400 VAR_001916 p.Gly129Val LP/P rs281874723 Alport syndrome 1, X-linked (ATS1)
[MIM:301050]
COL4A5 P29400 VAR_001917 p.Gly174Arg LP/P rs104886055 Alport syndrome 1, X-linked (ATS1)
[MIM:301050]
COL4A5 P29400 VAR_001918 p.Gly177Arg LP/P rs104886056 Alport syndrome 1, X-linked (ATS1)
[MIM:301050]
COL4A5 P29400 VAR_001919 p.Gly216Arg LP/P rs104886067 Alport syndrome 1, X-linked (ATS1)
[MIM:301050]

COL4A5 P29400 VAR_001920 p.Gly219Ser LP/P rs104886075 Alport syndrome 1, X-linked (ATS1) [MIM:301050]
COL4A5 P29400 VAR_001921 p.Gly289Val LP/P rs104886450 Alport syndrome 1, X-linked (ATS1) [MIM:301050]
COL4A5 P29400 VAR_001922 p.Gly292Val LP/P rs104886078 Alport syndrome 1, X-linked (ATS1) [MIM:301050]
COL4A5 P29400 VAR_001923 p.Gly325Glu LP/P rs104886091 Alport syndrome 1, X-linked (ATS1) [MIM:301050]
COL4A5 P29400 VAR_001924 p.Gly325Arg LP/P rs104886088 Alport syndrome 1, X-linked (ATS1) [MIM:301050]
COL4A5 P29400 VAR_001925 p.Gly365Glu LP/P rs104886096 Alport syndrome 1, X-linked (ATS1) [MIM:301050]
COL4A5 P29400 VAR_001927 p.Gly371Glu LP/P rs104886097 Alport syndrome 1, X-linked (ATS1) [MIM:301050]
COL4A5 P29400 VAR_001928 p.Gly374Ala LP/P rs104886108 Alport syndrome 1, X-linked (ATS1) [MIM:301050]
COL4A5 P29400 VAR_001929 p.Gly383Asp LP/P rs104886105 Alport syndrome 1, X-linked (ATS1) [MIM:301050]
COL4A5 P29400 VAR_001930 p.Gly400Glu LP/P rs104886107 Alport syndrome 1, X-linked (ATS1) [MIM:301050]
COL4A5 P29400 VAR_001931 p.Gly406Val LP/P rs104886100 Alport syndrome 1, X-linked (ATS1) [MIM:301050]
COL4A5 P29400 VAR_001932 p.Gly409Asp LP/P rs104886101 Alport syndrome 1, X-linked (ATS1) [MIM:301050]
COL4A5 P29400 VAR_001933 p.Ala430Asp LB/B rs142883891 -
COL4A5 P29400 VAR_001934 p.Ile444Ser LB/B rs2272946 -
COL4A5 P29400 VAR_001936 p.Gly466Glu LP/P rs104886114 Alport syndrome 1, X-linked (ATS1) [MIM:301050]
COL4A5 P29400 VAR_001937 p.Gly494Asp LP/P rs104886118 Alport syndrome 1, X-linked (ATS1) [MIM:301050]
COL4A5 P29400 VAR_001939 p.Gly521Cys LP/P rs104886121 Alport syndrome 1, X-linked (ATS1) [MIM:301050]
COL4A5 P29400 VAR_001940 p.Gly521Ser LP/P rs104886121 Alport syndrome 1, X-linked (ATS1) [MIM:301050]
COL4A5 P29400 VAR_001941 p.Gly567Ala LP/P rs104886137 Alport syndrome 1, X-linked (ATS1) [MIM:301050]
COL4A5 P29400 VAR_001942 p.Gly609Val LP/P rs104886140 Alport syndrome 1, X-linked (ATS1) [MIM:301050]
COL4A5 P29400 VAR_001943 p.Gly638Val LP/P rs104886134 Alport syndrome 1, X-linked (ATS1) [MIM:301050]
COL4A5 P29400 VAR_001944 p.Gly638Ala LP/P rs104886134 Alport syndrome 1, X-linked (ATS1) [MIM:301050]
COL4A5 P29400 VAR_001945 p.Gly653Arg LP/P rs104886150 Alport syndrome 1, X-linked (ATS1) [MIM:301050]
COL4A5 P29400 VAR_001946 p.Lys664Asn LB/B rs34077552 -
COL4A5 P29400 VAR_001947 p.Gly684Val LP/P rs104886160 Alport syndrome 1, X-linked (ATS1) [MIM:301050]
COL4A5 P29400 VAR_001948 p.Gly740Glu LP/P rs104886165 Alport syndrome 1, X-linked (ATS1) [MIM:301050]

COL4A5 P29400 VAR_001949 p.Gly772Asp LP/P rs104886173 Alport syndrome 1, X-linked (ATS1) [MIM:301050]
COL4A5 P29400 VAR_001950 p.Gly796Arg LP/P rs104886177 Alport syndrome 1, X-linked (ATS1) [MIM:301050]
COL4A5 P29400 VAR_001951 p.Gly852Arg LP/P rs104886186 Alport syndrome 1, X-linked (ATS1) [MIM:301050]
COL4A5 P29400 VAR_001952 p.Gly866Glu LP/P rs104886188 Alport syndrome 1, X-linked (ATS1) [MIM:301050]
COL4A5 P29400 VAR_001953 p.Gly869Arg LP/P rs104886189 Alport syndrome 1, X-linked (ATS1) [MIM:301050]
COL4A5 P29400 VAR_001954 p.Gly872Arg LP/P rs104886190 Alport syndrome 1, X-linked (ATS1) [MIM:301050]
COL4A5 P29400 VAR_001956 p.Gly1104Val LP/P rs104886224 Alport syndrome 1, X-linked (ATS1) [MIM:301050]
COL4A5 P29400 VAR_001957 p.Gly1143Asp LP/P rs104886229 Alport syndrome 1, X-linked (ATS1) [MIM:301050]
COL4A5 P29400 VAR_001958 p.Gly1143Ser LP/P rs104886228 Alport syndrome 1, X-linked (ATS1) [MIM:301050]
COL4A5 P29400 VAR_001959 p.Gly1182Arg LP/P rs104886242 Alport syndrome 1, X-linked (ATS1) [MIM:301050]
COL4A5 P29400 VAR_001960 p.Gly1241Cys LP/P rs104886255 Alport syndrome 1, X-linked (ATS1) [MIM:301050]
COL4A5 P29400 VAR_001961 p.Gly1270Ser LP/P rs104886257 Alport syndrome 1, X-linked (ATS1) [MIM:301050]
COL4A5 P29400 VAR_001962 p.Gly1379Val LP/P rs104886269 Alport syndrome 1, X-linked (ATS1) [MIM:301050]
COL4A5 P29400 VAR_001963 p.Arg1410Cys LP/P rs104886270 Alport syndrome 1, X-linked (ATS1) [MIM:301050]
COL4A5 P29400 VAR_001964 p.Gly1421Trp LP/P rs104886272 Alport syndrome 1, X-linked (ATS1) [MIM:301050]
COL4A5 P29400 VAR_001965 p.Arg1422Cys LP/P rs144282156 Alport syndrome 1, X-linked (ATS1) [MIM:301050]
COL4A5 P29400 VAR_001966 p.Gly1451Ser LP/P rs104886280 Alport syndrome 1, X-linked (ATS1) [MIM:301050]
COL4A5 P29400 VAR_001967 p.Ala1498Asp LP/P rs104886284 Alport syndrome 1, X-linked (ATS1) [MIM:301050]
COL4A5 P29400 VAR_001968 p.Pro1517Thr LP/P rs201220208 Alport syndrome 1, X-linked (ATS1) [MIM:301050]
COL4A5 P29400 VAR_001969 p.Trp1538Ser LP/P rs104886293 Alport syndrome 1, X-linked (ATS1) [MIM:301050]
COL4A5 P29400 VAR_001970 p.Arg1563Gln LP/P rs281874743 Alport syndrome 1, X-linked (ATS1) [MIM:301050]
COL4A5 P29400 VAR_001971 p.Cys1564Ser LP/P rs104886287 Alport syndrome 1, X-linked (ATS1) [MIM:301050]
COL4A5 P29400 VAR_001972 p.Gly1596Asp LP/P rs104886297 Alport syndrome 1, X-linked (ATS1) [MIM:301050]
COL4A5 P29400 VAR_001973 p.Leu1649Arg LP/P rs104886303 Alport syndrome 1, X-linked (ATS1) [MIM:301050]

COL4A5 P29400 VAR_001974 p.Arg1677Gln LP/P rs104886308 Alport syndrome 1, X-linked (ATS1) [MIM:301050]
COL4A5 P29400 VAR_007991 p.Gly114Ser LP/P - Alport syndrome 1, X-linked (ATS1) [MIM:301050]
COL4A5 P29400 VAR_007992 p.Gly331Val LP/P rs104886092 Alport syndrome 1, X-linked (ATS1) [MIM:301050]
COL4A5 P29400 VAR_007993 p.Gly472Arg LP/P rs104886116 Alport syndrome 1, X-linked (ATS1) [MIM:301050]
COL4A5 P29400 VAR_007994 p.Gly545Arg LP/P rs104886126 Alport syndrome 1, X-linked (ATS1) [MIM:301050]
COL4A5 P29400 VAR_007995 p.Gly545Val LP/P rs104886127 Alport syndrome 1, X-linked (ATS1) [MIM:301050]
COL4A5 P29400 VAR_007996 p.Gly561Arg LP/P rs104886136 Alport syndrome 1, X-linked (ATS1) [MIM:301050]
COL4A5 P29400 VAR_007997 p.Gly579Arg LP/P rs104886139 Alport syndrome 1, X-linked (ATS1) [MIM:301050]
COL4A5 P29400 VAR_007998 p.Gly635Asp LP/P rs281874683 Alport syndrome 1, X-linked (ATS1) [MIM:301050]
COL4A5 P29400 VAR_007999 p.Gly638Ser LP/P rs104886147 Alport syndrome 1, X-linked (ATS1) [MIM:301050]
COL4A5 P29400 VAR_008000 p.Gly669Ala LP/P rs104886151 Alport syndrome 1, X-linked (ATS1) [MIM:301050]
COL4A5 P29400 VAR_008001 p.Gly687Glu LP/P rs104886168 Alport syndrome 1, X-linked (ATS1) [MIM:301050]
COL4A5 P29400 VAR_008002 p.Gly743Asp LP/P rs104886166 Alport syndrome 1, X-linked (ATS1) [MIM:301050]
COL4A5 P29400 VAR_008003 p.Gly808Glu LP/P rs104886180 Alport syndrome 1, X-linked (ATS1) [MIM:301050]
COL4A5 P29400 VAR_008005 p.Gly852Glu LP/P rs104886187 Alport syndrome 1, X-linked (ATS1) [MIM:301050]
COL4A5 P29400 VAR_008006 p.Gly878Arg LP/P - Alport syndrome 1, X-linked (ATS1) [MIM:301050]
COL4A5 P29400 VAR_008008 p.Gly1107Arg LP/P rs104886225 Alport syndrome 1, X-linked (ATS1) [MIM:301050]
COL4A5 P29400 VAR_008009 p.Gly1161Arg LP/P rs104886235 Alport syndrome 1, X-linked (ATS1) [MIM:301050]
COL4A5 P29400 VAR_008010 p.Gly1211Arg LP/P rs104886246 Alport syndrome 1, X-linked (ATS1) [MIM:301050]
COL4A5 P29400 VAR_008011 p.Gly1220Asp LP/P rs104886251 Alport syndrome 1, X-linked (ATS1) [MIM:301050]
COL4A5 P29400 VAR_008012 p.Gly1333Ser LP/P rs104886266 Alport syndrome 1, X-linked (ATS1) [MIM:301050]
COL4A5 P29400 VAR_008013 p.Gly1427Val LP/P rs104886274 Alport syndrome 1, X-linked (ATS1) [MIM:301050]
COL4A5 P29400 VAR_008014 p.Gly1442Asp LP/P rs104886277 Alport syndrome 1, X-linked (ATS1) [MIM:301050]
COL4A5 P29400 VAR_008015 p.Gly1486Ala LP/P rs104886282 Alport syndrome 1, X-linked (ATS1) [MIM:301050]
COL4A5 P29400 VAR_008016 p.Pro1559Ala LB/B rs104886295 -
COL4A5 P29400 VAR_011220 p.Gly177Cys LP/P rs104886056 Alport syndrome 1, X-linked (ATS1) [MIM:301050]

COL4A5 P29400 VAR_011221 p.Gly192Arg LP/P rs104886060 Alport syndrome 1, X-linked (ATS1) [MIM:301050]
COL4A5 P29400 VAR_011222 p.Gly204Val LP/P rs104886063 Alport syndrome 1, X-linked (ATS1) [MIM:301050]
COL4A5 P29400 VAR_011223 p.Gly230Arg LP/P rs104886076 Alport syndrome 1, X-linked (ATS1) [MIM:301050]
COL4A5 P29400 VAR_011224 p.Gly239Glu LP/P rs104886068 Alport syndrome 1, X-linked (ATS1) [MIM:301050]
COL4A5 P29400 VAR_011225 p.Gly264Arg LP/P rs104886069 Alport syndrome 1, X-linked (ATS1) [MIM:301050]
COL4A5 P29400 VAR_011226 p.Gly292Arg LP/P rs104886073 Alport syndrome 1, X-linked (ATS1) [MIM:301050]
COL4A5 P29400 VAR_011227 p.Gly295Asp LP/P rs104886079 Alport syndrome 1, X-linked (ATS1) [MIM:301050]
COL4A5 P29400 VAR_011228 p.Gly298Ser LP/P rs104886080 Alport syndrome 1, X-linked (ATS1) [MIM:301050]
COL4A5 P29400 VAR_011229 p.Gly319Arg LP/P rs104886085 Alport syndrome 1, X-linked (ATS1) [MIM:301050]
COL4A5 P29400 VAR_011230 p.Gly412Val LP/P rs104886102 Alport syndrome 1, X-linked (ATS1) [MIM:301050]
COL4A5 P29400 VAR_011231 p.Gly415Arg LP/P rs104886103 Alport syndrome 1, X-linked (ATS1) [MIM:301050]
COL4A5 P29400 VAR_011232 p.Gly420Glu LP/P rs281874663 Alport syndrome 1, X-linked (ATS1) [MIM:301050]
COL4A5 P29400 VAR_011233 p.Gly420Val LP/P rs281874663 Alport syndrome 1, X-linked (ATS1) [MIM:301050]
COL4A5 P29400 VAR_011234 p.Gly423Glu LP/P rs104886110 Alport syndrome 1, X-linked (ATS1) [MIM:301050]
COL4A5 P29400 VAR_011235 p.Gly491Glu LP/P rs104886117 Alport syndrome 1, X-linked (ATS1) [MIM:301050]
COL4A5 P29400 VAR_011236 p.Gly497Cys LP/P rs104886120 Alport syndrome 1, X-linked (ATS1) [MIM:301050]
COL4A5 P29400 VAR_011237 p.Gly524Asp LP/P rs104886119 Alport syndrome 1, X-linked (ATS1) [MIM:301050]
COL4A5 P29400 VAR_011238 p.Gly558Arg LP/P rs104886129 Alport syndrome 1, X-linked (ATS1) [MIM:301050]
COL4A5 P29400 VAR_011239 p.Gly573Asp LP/P rs104886138 Alport syndrome 1, X-linked (ATS1) [MIM:301050]
COL4A5 P29400 VAR_011240 p.Gly579Glu LP/P rs104886130 Alport syndrome 1, X-linked (ATS1) [MIM:301050]
COL4A5 P29400 VAR_011241 p.Gly603Val LP/P rs104886133 Alport syndrome 1, X-linked (ATS1) [MIM:301050]
COL4A5 P29400 VAR_011242 p.Gly609Arg LP/P rs104886135 Alport syndrome 1, X-linked (ATS1) [MIM:301050]
COL4A5 P29400 VAR_011243 p.Pro619Ser LB/B rs1569494314 -
COL4A5 P29400 VAR_011244 p.Gly621Cys LP/P rs104886141 Alport syndrome 1, X-linked (ATS1) [MIM:301050]
COL4A5 P29400 VAR_011245 p.Gly624Asp LP/P rs104886142 Alport syndrome 1, X-linked (ATS1) [MIM:301050]

COL4A5 P29400 VAR_011246 p.Gly629Asp LP/P rs104886144 Alport syndrome 1, X-linked (ATS1) [MIM:301050]
COL4A5 P29400 VAR_011247 p.Gly632Asp LP/P rs104886145 Alport syndrome 1, X-linked (ATS1) [MIM:301050]
COL4A5 P29400 VAR_011248 p.Glu633Lys LP/P rs104886146 Alport syndrome 1, X-linked (ATS1) [MIM:301050]
COL4A5 P29400 VAR_011249 p.Gly681Asp LP/P rs104886158 Alport syndrome 1, X-linked (ATS1) [MIM:301050]
COL4A5 P29400 VAR_011250 p.Gly722Glu LP/P rs104886163 Alport syndrome 1, X-linked (ATS1) [MIM:301050]
COL4A5 P29400 VAR_011251 p.Pro739Ala LB/B rs104886164 -
COL4A5 P29400 VAR_011252 p.Pro739Ser LP/P rs104886164 Alport syndrome 1, X-linked (ATS1) [MIM:301050]
COL4A5 P29400 VAR_011253 p.Gly802Arg LP/P rs104886179 Alport syndrome 1, X-linked (ATS1) [MIM:301050]
COL4A5 P29400 VAR_011255 p.Gly811Val LP/P rs104886183 Alport syndrome 1, X-linked (ATS1) [MIM:301050]
COL4A5 P29400 VAR_011256 p.Gly822Arg LP/P rs104886184 Alport syndrome 1, X-linked (ATS1) [MIM:301050]
COL4A5 P29400 VAR_011258 p.Met898Val LP/P rs104886192 Alport syndrome 1, X-linked (ATS1) [MIM:301050]
COL4A5 P29400 VAR_011259 p.Gly902Val LP/P rs104886361 Alport syndrome 1, X-linked (ATS1) [MIM:301050]
COL4A5 P29400 VAR_011260 p.Gly911Glu LP/P rs104886363 Alport syndrome 1, X-linked (ATS1) [MIM:301050]
COL4A5 P29400 VAR_011261 p.Gly941Cys LP/P rs104886196 Alport syndrome 1, X-linked (ATS1) [MIM:301050]
COL4A5 P29400 VAR_011262 p.Gly947Asp LP/P rs104886370 Alport syndrome 1, X-linked (ATS1) [MIM:301050]
COL4A5 P29400 VAR_011263 p.Gly953Val LP/P rs78972735 Alport syndrome 1, X-linked (ATS1) [MIM:301050]
COL4A5 P29400 VAR_011264 p.Gly1006Ala LP/P - Alport syndrome 1, X-linked (ATS1) [MIM:301050]
COL4A5 P29400 VAR_011265 p.Gly1006Val LP/P rs104886202 Alport syndrome 1, X-linked (ATS1) [MIM:301050]
COL4A5 P29400 VAR_011266 p.Gly1015Glu LP/P - Alport syndrome 1, X-linked (ATS1) [MIM:301050]
COL4A5 P29400 VAR_011267 p.Gly1015Val LP/P rs104886211 Alport syndrome 1, X-linked (ATS1) [MIM:301050]
COL4A5 P29400 VAR_011268 p.Gly1030Ser LP/P rs104886210 Alport syndrome 1, X-linked (ATS1) [MIM:301050]
COL4A5 P29400 VAR_011269 p.Gly1036Val LP/P rs104886212 Alport syndrome 1, X-linked (ATS1) [MIM:301050]
COL4A5 P29400 VAR_011270 p.Gly1039Ser LP/P rs104886214 Alport syndrome 1, X-linked (ATS1) [MIM:301050]
COL4A5 P29400 VAR_011271 p.Gly1045Glu LP/P rs104886215 Alport syndrome 1, X-linked (ATS1) [MIM:301050]
COL4A5 P29400 VAR_011272 p.Gly1066Arg LP/P rs104886219 Alport syndrome 1, X-linked (ATS1) [MIM:301050]
COL4A5 P29400 VAR_011273 p.Gly1066Ser LP/P rs104886219 Alport syndrome 1, X-linked (ATS1) [MIM:301050]

COL4A5 P29400 VAR_011274 p.Gly1086Asp LP/P rs104886232 Alport syndrome 1, X-linked (ATS1) [MIM:301050]
COL4A5 P29400 VAR_011275 p.Gly1158Arg LP/P rs104886389 Alport syndrome 1, X-linked (ATS1) [MIM:301050]
COL4A5 P29400 VAR_011276 p.Gly1167Ser LP/P rs104886236 Alport syndrome 1, X-linked (ATS1) [MIM:301050]
COL4A5 P29400 VAR_011277 p.Gly1170Ser LP/P rs104886237 Alport syndrome 1, X-linked (ATS1) [MIM:301050]
COL4A5 P29400 VAR_011278 p.Gly1196Arg LP/P rs104886244 Alport syndrome 1, X-linked (ATS1) [MIM:301050]
COL4A5 P29400 VAR_011279 p.Gly1205Cys LP/P rs104886245 Alport syndrome 1, X-linked (ATS1) [MIM:301050]
COL4A5 P29400 VAR_011280 p.Gly1211Glu LP/P rs104886247 Alport syndrome 1, X-linked (ATS1) [MIM:301050]
COL4A5 P29400 VAR_011281 p.Gly1229Asp LP/P rs104886253 Alport syndrome 1, X-linked (ATS1) [MIM:301050]
COL4A5 P29400 VAR_011282 p.Gly1244Asp LP/P rs104886261 Alport syndrome 1, X-linked (ATS1) [MIM:301050]
COL4A5 P29400 VAR_011283 p.Gly1252Ser LP/P rs104886262 Alport syndrome 1, X-linked (ATS1) [MIM:301050]
COL4A5 P29400 VAR_011284 p.Gly1261Glu LP/P rs104886264 Alport syndrome 1, X-linked (ATS1) [MIM:301050]
COL4A5 P29400 VAR_011285 p.Gly1357Ser LP/P rs104886267 Alport syndrome 1, X-linked (ATS1) [MIM:301050]
COL4A5 P29400 VAR_011286 p.Leu1428Met LB/B rs1569508163 -
COL4A5 P29400 VAR_011287 p.Ser1488Phe LP/P rs104886283 Alport syndrome 1, X-linked (ATS1) [MIM:301050]
COL4A5 P29400 VAR_011288 p.Arg1511His US rs104886285 Alport syndrome 1, X-linked (ATS1) [MIM:301050]
COL4A5 P29400 VAR_011289 p.Cys1567Arg LP/P rs104886288 Alport syndrome 1, X-linked (ATS1) [MIM:301050]
COL4A5 P29400 VAR_011290 p.Arg1677Pro LP/P rs104886308 Alport syndrome 1, X-linked (ATS1) [MIM:301050]
COL4A5 P29400 VAR_011291 p.Cys1678Trp LP/P rs104886311 Alport syndrome 1, X-linked (ATS1) [MIM:301050]
COL4A5 P29400 VAR_071932 p.Gly123Glu LP/P rs1569488429 Alport syndrome 1, X-linked (ATS1) [MIM:301050]
COL4A6 Q14031 VAR_015216 p.Ser455Ala LB/B rs1042065 -
COL4A6 Q14031 VAR_015217 p.Asn1110Lys LB/B rs1042067 -
COL4A6 Q14031 VAR_032972 p.Pro1126Ser LB/B rs35179844 -
COL4A6 Q14031 VAR_032973 p.Ile1162Val LB/B rs34466065 -
COL4A6 Q14031 VAR_032974 p.Leu1362Pro LB/B rs35363062 -
COL4A6 Q14031 VAR_035748 p.Gly1130Glu US - A colorectal cancer sample
COL4A6 Q14031 VAR_059242 p.Ser455Pro LB/B rs1042065 -
COL4A6 Q14031 VAR_070936 p.Gly591Ser LP/P rs779748859 Deafness, X-linked, 6 (DFNX6) [MIM:300914]
COL5A1 P20908 VAR_001808 p.Cys1639Ser LP/P rs80338764 Ehlers-Danlos syndrome, classic type, 1 (EDSCL1) [MIM:130000]
COL5A1 P20908 VAR_015412 p.Gly530Ser LP/P rs61735045 Ehlers-Danlos syndrome, classic type, 1 (EDSCL1) [MIM:130000]

COL5A1 P20908 VAR_015413 p.Gly1489Asp LP/P - Ehlers-Danlos syndrome, classic type, 1 (EDSCL1) [MIM:130000]
COL5A1 P20908 VAR_057902 p.Leu25Pro LP/P - Ehlers-Danlos syndrome, classic type, 1 (EDSCL1) [MIM:130000]
COL5A1 P20908 VAR_057903 p.Leu25Arg LP/P - Ehlers-Danlos syndrome, classic type, 1 (EDSCL1) [MIM:130000]
COL5A1 P20908 VAR_057904 p.Ala114Asp LB/B rs147589613 -
COL5A1 P20908 VAR_057905 p.Asp192Asn LB/B rs138579182 -
COL5A1 P20908 VAR_057906 p.Asp229Asn US - -
COL5A1 P20908 VAR_057907 p.Pro393Ser US - -
COL5A1 P20908 VAR_057908 p.Asn951Ser LB/B rs61736966 -
COL5A1 P20908 VAR_057909 p.Gly1486Cys LP/P - Ehlers-Danlos syndrome, classic type, 1 (EDSCL1) [MIM:130000]
COL5A1 P20908 VAR_064702 p.Pro908Leu LB/B rs772211736 -
COL5A1 P20908 VAR_075702 p.Glu863Val LB/B rs139788610 -
COL5A1 P20908 VAR_075703 p.Val1140Met LB/B rs149616140 -
COL5A2 P05997 VAR_013588 p.Gly963Arg LP/P rs1186550791 Ehlers-Danlos syndrome, classic type, 2 (EDSCL2) [MIM:130010]
COL5A2 P05997 VAR_048799 p.Pro460Ser LB/B rs35830636 -
COL5A2 P05997 VAR_048800 p.Arg956Pro LB/B rs6434313 -
COL5A2 P05997 VAR_057910 p.Val512Ala LB/B rs35852101 -
COL5A2 P05997 VAR_057911 p.Pro833Leu LB/B rs116298748 -
COL5A2 P05997 VAR_057912 p.Thr1230Ser LB/B rs767234623 -
COL5A2 P05997 VAR_057913 p.Asp1432Val LB/B rs141777954 -
COL5A2 P05997 VAR_078424 p.Gly228Arg LP/P - Ehlers-Danlos syndrome, classic type, 2 (EDSCL2) [MIM:130010]
COL5A3 P25940 VAR_020015 p.Arg134His LB/B rs2303098 -
COL5A3 P25940 VAR_020016 p.Arg1207Pro LB/B rs2287813 -
COL5A3 P25940 VAR_020017 p.Val1428Met LB/B rs3815746 -
COL5A3 P25940 VAR_020018 p.Ile1594Met LB/B rs3745581 -
COL5A3 P25940 VAR_020019 p.Val1691Ile LB/B rs2277969 -
COL5A3 P25940 VAR_055678 p.Arg1042Pro LB/B rs2161468 -
COL5A3 P25940 VAR_055679 p.Ala1488Pro LB/B rs3745584 -
COL5A3 P25940 VAR_060789 p.Arg322Gly LB/B rs2287803 -
COL6A1 P12109 VAR_013580 p.Lys121Arg LP/P rs121912936 Bethlem myopathy 1 (BTHLM1) [MIM:158810]
COL6A1 P12109 VAR_013581 p.Gly305Val LP/P - Bethlem myopathy 1 (BTHLM1) [MIM:158810]
COL6A1 P12109 VAR_013582 p.Gly341Asp LP/P rs121912935 Bethlem myopathy 1 (BTHLM1) [MIM:158810]
COL6A1 P12109 VAR_048763 p.Arg439Gln LB/B rs35059000 -
COL6A1 P12109 VAR_048764 p.Arg850His LB/B rs1053312 -
COL6A1 P12109 VAR_058213 p.Ser116Asn LB/B rs11553519 -
COL6A1 P12109 VAR_058214 p.Gly272Asp LP/P rs1064793840 Bethlem myopathy 1 (BTHLM1) [MIM:158810]
COL6A1 P12109 VAR_058215 p.Pro274Leu LP/P rs201093313 Bethlem myopathy 1 (BTHLM1) [MIM:158810]
COL6A1 P12109 VAR_058216 p.Gly275Arg LP/P rs1556425467 Bethlem myopathy 1 (BTHLM1) [MIM:158810]
COL6A1 P12109 VAR_058217 p.Gly281Arg LP/P rs267606746 Ullrich congenital muscular dystrophy 1 (UCMD1) [MIM:254090]
COL6A1 P12109 VAR_058218 p.Gly284Arg LP/P rs121912938 Ullrich congenital muscular dystrophy 1 (UCMD1) [MIM:254090]
COL6A1 P12109 VAR_058219 p.Gly290Arg LP/P rs121912939 Bethlem myopathy 1 (BTHLM1) [MIM:158810]

COL6A1 P12109 VAR_058219 p.Gly290Arg LP/P rs121912939 Ullrich congenital muscular dystrophy 1 (UCMD1) [MIM:254090]
COL6A1 P12109 VAR_058220 p.Gly332Ser LB/B rs11701912 -
COL6A1 P12109 VAR_058221 p.Gly341Val LP/P rs121912935 Bethlem myopathy 1 (BTHLM1) [MIM:158810]
COL6A1 P12109 VAR_058222 p.Lys571Thr LP/P rs751040647 Bethlem myopathy 1 (BTHLM1) [MIM:158810]
COL6A1 P12109 VAR_058223 p.Thr881Met LB/B rs150432347 -
COL6A1 P12109 VAR_058224 p.Ser890Leu LB/B rs13051496 -
COL6A1 P12109 VAR_081097 p.Asp43Ala LP/P rs786205555 -
COL6A2 P12110 VAR_013589 p.Gly271Ser LP/P rs121912940 Bethlem myopathy 1 (BTHLM1) [MIM:158810]
COL6A2 P12110 VAR_013590 p.Asp621Asn LP/P rs267606750 Bethlem myopathy 1 (BTHLM1) [MIM:158810]
COL6A2 P12110 VAR_030315 p.Ser399Asn LB/B rs2839110 -
COL6A2 P12110 VAR_030316 p.Arg680His LB/B rs1042917 -
COL6A2 P12110 VAR_048801 p.Asp227Asn LB/B rs35881321 -
COL6A2 P12110 VAR_048802 p.Gly935Arg LB/B rs35548026 -
COL6A2 P12110 VAR_048803 p.Ile1015Leu LB/B rs11910483 -
COL6A2 P12110 VAR_058225 p.Glu106Lys LB/B rs141703710 -
COL6A2 P12110 VAR_058226 p.Gly283Arg LP/P rs267606748 Ullrich congenital muscular dystrophy 1 (UCMD1) [MIM:254090]
COL6A2 P12110 VAR_058227 p.Arg489Gln LB/B rs61735828 -
COL6A2 P12110 VAR_058228 p.Arg498His LP/P rs267606749 Ullrich congenital muscular dystrophy 1 (UCMD1) [MIM:254090]
COL6A2 P12110 VAR_058229 p.Pro518Ser LB/B rs141166141 -
COL6A2 P12110 VAR_058230 p.Gly531Arg LP/P - Ullrich congenital muscular dystrophy 1 (UCMD1) [MIM:254090]
COL6A2 P12110 VAR_058231 p.Gly700Ser LP/P rs794727418 Bethlem myopathy 1 (BTHLM1) [MIM:158810]
COL6A2 P12110 VAR_058232 p.Arg724Cys LB/B rs150098077 -
COL6A2 P12110 VAR_058233 p.Cys777Arg LP/P rs267606747 Bethlem myopathy 1 (BTHLM1) [MIM:158810]
COL6A2 P12110 VAR_058234 p.Arg784His LB/B rs75120695 -
COL6A2 P12110 VAR_058235 p.Val804Gly LB/B rs779847082 -
COL6A2 P12110 VAR_058236 p.Leu837Pro LP/P rs1255514828 Ullrich congenital muscular dystrophy 1 (UCMD1) [MIM:254090]
COL6A2 P12110 VAR_058237 p.Arg853Gln LB/B rs144830948 -
COL6A2 P12110 VAR_058238 p.Arg876Ser LP/P rs387906608 Ullrich congenital muscular dystrophy 1 (UCMD1) [MIM:254090]
COL6A2 P12110 VAR_058239 p.Ser895Arg LB/B rs141233891 -
COL6A2 P12110 VAR_058241 p.Pro932Leu LP/P rs117725825 Bethlem myopathy 1 (BTHLM1) [MIM:158810]
COL6A2 P12110 VAR_076959 p.Arg377Cys LB/B rs144801620 -
COL6A2 P12110 VAR_076960 p.Asp446Asn LB/B rs535007570 -
COL6A2 P12110 VAR_076961 p.Val728Met LB/B rs200585528 -
COL6A2 P12110 VAR_076962 p.Arg843Gln LB/B rs201736323 -
COL6A2 P12110 VAR_076963 p.Phe1010Cys LB/B rs1051148162 -
COL6A3 P12111 VAR_001910 p.Gly1679Glu LP/P rs121434553 Bethlem myopathy 1 (BTHLM1) [MIM:158810]
COL6A3 P12111 VAR_001911 p.Asp2831His LB/B rs36104025 -
COL6A3 P12111 VAR_047279 p.Thr538Met LB/B rs34741387 -
COL6A3 P12111 VAR_047280 p.Arg659His LB/B rs36092870 -
COL6A3 P12111 VAR_047281 p.Val886Glu LB/B rs9630964 -
COL6A3 P12111 VAR_047282 p.Lys1088Gln LB/B rs11896521 -
COL6A3 P12111 VAR_047283 p.Pro2218Leu LB/B rs36117715 -
COL6A3 P12111 VAR_047284 p.Asn2805Thr LB/B rs35848091 -

COL6A3	P12111	VAR_047285	p.Met2927Thr	LB/B	rs6728818	-
COL6A3	P12111	VAR_047286	p.Met2988Val	LB/B	rs11690358	-
COL6A3	P12111	VAR_047287	p.Ala3012Pro	LB/B	rs2270669	-
COL6A3	P12111	VAR_047288	p.Thr3069Ile	LB/B	rs1131296	-
COL6A3	P12111	VAR_058242	p.Leu411Val	LB/B	rs113716915	-
COL6A3	P12111	VAR_058243	p.Asp491His	LB/B	rs112010940	-
COL6A3	P12111	VAR_058244	p.Thr492Ser	LB/B	rs113897824	-
COL6A3	P12111	VAR_058245	p.Arg677His	LB/B	rs35227432	-
COL6A3	P12111	VAR_058246	p.Ala807Thr	LB/B	rs113155945	-
COL6A3	P12111	VAR_058247	p.Ala830Ser	LB/B	rs77181645	-
COL6A3	P12111	VAR_058248	p.Lys1014Glu	LP/P	rs114284669	Bethlem myopathy 1 (BTHLM1) [MIM:158810]
COL6A3	P12111	VAR_058249	p.Arg1064Gln	LB/B	rs112638391	-
COL6A3	P12111	VAR_058250	p.Glu1386Lys	LP/P	rs146092501	Bethlem myopathy 1 (BTHLM1) [MIM:158810]
COL6A3	P12111	VAR_058251	p.Arg1395Gln	LB/B	rs80272723	-
COL6A3	P12111	VAR_058252	p.Asn1467Asp	LP/P	rs138049094	Bethlem myopathy 1 (BTHLM1) [MIM:158810]
COL6A3	P12111	VAR_058253	p.Arg1576Gln	LB/B	rs61729839	-
COL6A3	P12111	VAR_058254	p.Arg1632Gln	LB/B	rs111231885	-
COL6A3	P12111	VAR_058255	p.Asp1674Asn	LP/P	rs778940391	Ullrich congenital muscular dystrophy 1 (UCMD1) [MIM:254090]
COL6A3	P12111	VAR_058256	p.Pro1687Ser	LB/B	rs35273032	-
COL6A3	P12111	VAR_058257	p.Leu1726Arg	LP/P	rs121434555	Bethlem myopathy 1 (BTHLM1) [MIM:158810]
COL6A3	P12111	VAR_058258	p.Val1985Met	LP/P	rs200478135	Bethlem myopathy 1 (BTHLM1) [MIM:158810]
COL6A3	P12111	VAR_058259	p.Gly2047Asp	LP/P	-	Bethlem myopathy 1 (BTHLM1) [MIM:158810]
COL6A3	P12111	VAR_058260	p.Gly2056Arg	LP/P	-	Bethlem myopathy 1 (BTHLM1) [MIM:158810]
COL6A3	P12111	VAR_058261	p.Gly2080Asp	LP/P	rs794727188	Bethlem myopathy 1 (BTHLM1) [MIM:158810]
COL6A3	P12111	VAR_058262	p.Asp2431Val	LB/B	-	-
COL6A3	P12111	VAR_058263	p.Glu2453Lys	LB/B	rs886044364	-
COL6A3	P12111	VAR_058264	p.Ala2941Val	LB/B	rs11903206	-
COL6A3	P12111	VAR_073836	p.Arg2501His	LP/P	rs541928674	Dystonia 27 (DYT27) [MIM:616411]
COL6A3	P12111	VAR_073837	p.Ala2554Thr	LP/P	rs786205870	Dystonia 27 (DYT27) [MIM:616411]
COL6A3	P12111	VAR_073838	p.Arg3043His	LP/P	rs552651651	Dystonia 27 (DYT27) [MIM:616411]
COL6A3	P12111	VAR_073839	p.Pro3082Arg	LP/P	rs182976977	Dystonia 27 (DYT27) [MIM:616411]
COL6A5	A8TX70	VAR_043607	p.Gln2188Arg	LB/B	rs9883988	-
COL6A5	A8TX70	VAR_043608	p.Gly2205Asp	LB/B	rs819085	-
COL6A5	A8TX70	VAR_059234	p.Glu455Lys	LB/B	rs1453241	-
COL6A5	A8TX70	VAR_059235	p.Asn641His	LB/B	rs9882852	-
COL6A5	A8TX70	VAR_059236	p.His805Arg	LB/B	rs16827168	-
COL6A5	A8TX70	VAR_059237	p.Asp982Gly	LB/B	rs11917356	-
COL6A5	A8TX70	VAR_059238	p.Ile1114Met	LB/B	rs1353613	-
COL6A5	A8TX70	VAR_059239	p.Thr1280Pro	LB/B	rs12488457	-
COL6A5	A8TX70	VAR_059240	p.Cys1477Ser	LB/B	rs1497312	-
COL6A5	A8TX70	VAR_059241	p.Ser1589Pro	LB/B	rs16827497	-
COL6A5	A8TX70	VAR_061119	p.Asp2175Asn	LB/B	rs60021408	-
COL6A6	A6NMZ7	VAR_043609	p.Glu345Lys	LB/B	rs4613427	-
COL6A6	A6NMZ7	VAR_043610	p.Ala370Thr	LB/B	rs9830253	-
COL6A6	A6NMZ7	VAR_043611	p.Glu461Ala	LB/B	rs11921769	-
COL6A6	A6NMZ7	VAR_043612	p.Arg1739Gln	LB/B	rs16830494	-
COL6A6	A6NMZ7	VAR_043613	p.His1799Arg	LB/B	rs7614116	-
COL6A6	A6NMZ7	VAR_061120	p.Pro556Ser	LB/B	rs59021909	-

COL7A1 Q02388 VAR_001809 p.Lys142Arg US rs121912856 Epidermolysis bullosa dystrophica, autosomal recessive (RDEB) [MIM:226600]
COL7A1 Q02388 VAR_001810 p.Pro595Leu LB/B rs2228561 -
COL7A1 Q02388 VAR_001811 p.Pro1277Leu LB/B rs35761247 -
COL7A1 Q02388 VAR_001812 p.Gly1557Arg LP/P - Epidermolysis bullosa dystrophica, autosomal dominant (DDEB) [MIM:131750]
COL7A1 Q02388 VAR_001813 p.Gly1782Arg LP/P rs374718902 Epidermolysis bullosa dystrophica, autosomal recessive (RDEB) [MIM:226600]
COL7A1 Q02388 VAR_001814 p.Gly1982Trp LP/P - Epidermolysis bullosa dystrophica, autosomal recessive (RDEB) [MIM:226600]
COL7A1 Q02388 VAR_001815 p.Gly2003Arg LP/P rs121912832 Epidermolysis bullosa dystrophica, autosomal dominant (DDEB) [MIM:131750]
COL7A1 Q02388 VAR_001816 p.Arg2008Gly LP/P rs1055680335 Epidermolysis bullosa dystrophica, autosomal recessive (RDEB) [MIM:226600]
COL7A1 Q02388 VAR_001817 p.Gly2025Ala LP/P rs766931219 Epidermolysis bullosa dystrophica, autosomal recessive (RDEB) [MIM:226600]
COL7A1 Q02388 VAR_001818 p.Gly2034Arg LP/P rs121912844 Epidermolysis bullosa dystrophica, autosomal dominant (DDEB) [MIM:131750]
COL7A1 Q02388 VAR_001818 p.Gly2034Arg LP/P rs121912844 Epidermolysis bullosa dystrophica, with subcorneal cleavage (EBDSC) [MIM:131750]
COL7A1 Q02388 VAR_001819 p.Gly2040Ser LP/P rs121912829 Epidermolysis bullosa dystrophica, autosomal dominant (DDEB) [MIM:131750]
COL7A1 Q02388 VAR_001820 p.Gly2043Arg LP/P rs121912836 Epidermolysis bullosa dystrophica, autosomal dominant (DDEB) [MIM:131750]
COL7A1 Q02388 VAR_001821 p.Gly2049Glu LP/P - Epidermolysis bullosa dystrophica, autosomal recessive (RDEB) [MIM:226600]
COL7A1 Q02388 VAR_001822 p.Gly2055Glu LP/P rs1553854678 Epidermolysis bullosa dystrophica, autosomal dominant (DDEB) [MIM:131750]
COL7A1 Q02388 VAR_001823 p.Arg2063Trp LP/P rs121912849 Epidermolysis bullosa dystrophica, autosomal recessive (RDEB) [MIM:226600]
COL7A1 Q02388 VAR_001825 p.Gly2073Asp LP/P - Epidermolysis bullosa dystrophica, autosomal recessive (RDEB) [MIM:226600]
COL7A1 Q02388 VAR_001826 p.Gly2076Asp LP/P rs121912850 Epidermolysis bullosa dystrophica, autosomal dominant (DDEB) [MIM:131750]
COL7A1 Q02388 VAR_001827 p.Gly2079Glu LP/P - Epidermolysis bullosa dystrophica, autosomal dominant (DDEB) [MIM:131750]
COL7A1 Q02388 VAR_001828 p.Gly2242Arg LP/P rs121912837 Epidermolysis bullosa pruriginosa (EBP) [MIM:604129]
COL7A1 Q02388 VAR_001829 p.Gly2351Arg LB/B rs1800013 -
COL7A1 Q02388 VAR_001830 p.Gly2569Arg LP/P - Epidermolysis bullosa dystrophica, autosomal recessive (RDEB) [MIM:226600]
COL7A1 Q02388 VAR_001831 p.Gly2575Arg LP/P rs760891216 Epidermolysis bullosa dystrophica, autosomal recessive (RDEB) [MIM:226600]
COL7A1 Q02388 VAR_001832 p.Gly2623Cys LP/P rs121912831 Epidermolysis bullosa dystrophica, pretibial type (PR-DEB) [MIM:131850]
COL7A1 Q02388 VAR_001833 p.Gly2653Arg LP/P rs121912851 Epidermolysis bullosa dystrophica, autosomal recessive (RDEB) [MIM:226600]
COL7A1 Q02388 VAR_001834 p.Gly2671Val LP/P - Epidermolysis bullosa dystrophica, autosomal recessive (RDEB) [MIM:226600]

COL7A1 Q02388 VAR_001835 p.Gly2674Arg LP/P - Epidermolysis bullosa dystrophica, autosomal recessive (RDEB) [MIM:226600]
COL7A1 Q02388 VAR_001836 p.Gly2749Arg LP/P rs121912853 Epidermolysis bullosa dystrophica, autosomal recessive (RDEB) [MIM:226600]
COL7A1 Q02388 VAR_001837 p.Met2798Lys LP/P rs121912828 Epidermolysis bullosa dystrophica, autosomal recessive (RDEB) [MIM:226600]
COL7A1 Q02388 VAR_011160 p.Gly1347Arg LP/P rs121912833 Epidermolysis bullosa dystrophica, autosomal recessive (RDEB) [MIM:226600]
COL7A1 Q02388 VAR_011161 p.Gly1519Asp LP/P rs121912835 Transient bullous dermolysis of the newborn (TBDN) [MIM:131705]
COL7A1 Q02388 VAR_011162 p.Gly1522Glu LP/P rs387906605 Epidermolysis bullosa dystrophica, autosomal dominant (DDEB) [MIM:131750]
COL7A1 Q02388 VAR_011163 p.Gly1604Arg LP/P rs1560234201 Epidermolysis bullosa dystrophica, autosomal recessive (RDEB) [MIM:226600]
COL7A1 Q02388 VAR_011164 p.Gly1652Arg LP/P rs1439299333 Epidermolysis bullosa dystrophica, autosomal recessive (RDEB) [MIM:226600]
COL7A1 Q02388 VAR_011165 p.Gly1703Glu LP/P rs770304825 Epidermolysis bullosa dystrophica, autosomal recessive (RDEB) [MIM:226600]
COL7A1 Q02388 VAR_011166 p.Arg1772Trp LP/P rs1032335328 Epidermolysis bullosa dystrophica, autosomal recessive (RDEB) [MIM:226600]
COL7A1 Q02388 VAR_011167 p.Gly1776Arg LP/P - Epidermolysis bullosa dystrophica, autosomal dominant (DDEB) [MIM:131750]
COL7A1 Q02388 VAR_011168 p.Gly1791Glu LP/P rs1575450640 Epidermolysis bullosa pruriginosa (EBP) [MIM:604129]
COL7A1 Q02388 VAR_011169 p.Gly1812Arg LP/P - Epidermolysis bullosa dystrophica, autosomal recessive (RDEB) [MIM:226600]
COL7A1 Q02388 VAR_011170 p.Gly2006Ala LP/P - Epidermolysis bullosa dystrophica, autosomal dominant (DDEB) [MIM:131750]
COL7A1 Q02388 VAR_011171 p.Gly2006Asp LP/P rs121912842 Epidermolysis bullosa dystrophica, autosomal dominant (DDEB) [MIM:131750]
COL7A1 Q02388 VAR_011172 p.Arg2008Cys LP/P rs1055680335 Epidermolysis bullosa dystrophica, autosomal recessive (RDEB) [MIM:226600]
COL7A1 Q02388 VAR_011173 p.Gly2009Arg LP/P - Epidermolysis bullosa dystrophica, autosomal recessive (RDEB) [MIM:226600]
COL7A1 Q02388 VAR_011174 p.Gly2015Glu LP/P rs121912843 Epidermolysis bullosa dystrophica, autosomal dominant (DDEB) [MIM:131750]
COL7A1 Q02388 VAR_011175 p.Gly2028Ala LP/P - Epidermolysis bullosa dystrophica, autosomal dominant (DDEB) [MIM:131750]
COL7A1 Q02388 VAR_011176 p.Gly2028Arg LP/P rs762162799 Epidermolysis bullosa dystrophica, autosomal dominant (DDEB) [MIM:131750]
COL7A1 Q02388 VAR_011176 p.Gly2028Arg LP/P rs762162799 Epidermolysis bullosa pruriginosa (EBP) [MIM:604129]
COL7A1 Q02388 VAR_011177 p.Gly2031Ser LP/P rs121912838 Epidermolysis bullosa dystrophica, autosomal recessive (RDEB) [MIM:226600]
COL7A1 Q02388 VAR_011178 p.Gly2034Trp LP/P - Epidermolysis bullosa dystrophica, autosomal dominant (DDEB) [MIM:131750]
COL7A1 Q02388 VAR_011179 p.Gly2037Glu LP/P rs121912846 Epidermolysis bullosa dystrophica, autosomal dominant (DDEB) [MIM:131750]

COL7A1 Q02388 VAR_011180 p.Gly2040Asp LP/P - Epidermolysis bullosa dystrophica, autosomal dominant (DDEB) [MIM:131750]
COL7A1 Q02388 VAR_011181 p.Gly2040Val LP/P - Epidermolysis bullosa dystrophica, autosomal dominant (DDEB) [MIM:131750]
COL7A1 Q02388 VAR_011182 p.Gly2043Trp LP/P rs121912836 Epidermolysis bullosa dystrophica, autosomal dominant (DDEB) [MIM:131750]
COL7A1 Q02388 VAR_011183 p.Gly2046Val LP/P - Epidermolysis bullosa dystrophica, autosomal dominant (DDEB) [MIM:131750]
COL7A1 Q02388 VAR_011184 p.Gly2064Arg LP/P rs866061439 Epidermolysis bullosa dystrophica, autosomal dominant (DDEB) [MIM:131750]
COL7A1 Q02388 VAR_011185 p.Gly2079Arg LP/P - Epidermolysis bullosa dystrophica, autosomal dominant (DDEB) [MIM:131750]
COL7A1 Q02388 VAR_011186 p.Gly2132Asp LP/P rs755669902 Epidermolysis bullosa dystrophica, autosomal recessive (RDEB) [MIM:226600]
COL7A1 Q02388 VAR_011187 p.Gly2192Ser LP/P - Epidermolysis bullosa dystrophica, autosomal recessive (RDEB) [MIM:226600]
COL7A1 Q02388 VAR_011188 p.Gly2207Arg LP/P - Epidermolysis bullosa dystrophica, autosomal dominant (DDEB) [MIM:131750]
COL7A1 Q02388 VAR_011189 p.Gly2251Glu LP/P rs121912834 Transient bullous dermolysis of the newborn (TBDN) [MIM:131705]
COL7A1 Q02388 VAR_011190 p.Gly2263Val LP/P - Epidermolysis bullosa dystrophica, autosomal recessive (RDEB) [MIM:226600]
COL7A1 Q02388 VAR_011191 p.Gly2287Arg LP/P rs121912839 Epidermolysis bullosa dystrophica, autosomal recessive (RDEB) [MIM:226600]
COL7A1 Q02388 VAR_011192 p.Gly2316Arg LP/P - Epidermolysis bullosa dystrophica, autosomal recessive (RDEB) [MIM:226600]
COL7A1 Q02388 VAR_011193 p.Gly2348Arg US - -
COL7A1 Q02388 VAR_011194 p.Gly2366Ser LP/P rs1560204600 Epidermolysis bullosa dystrophica, autosomal recessive (RDEB) [MIM:226600]
COL7A1 Q02388 VAR_011195 p.Gly2369Ser LP/P - Epidermolysis bullosa pruriginosa (EBP) [MIM:604129]
COL7A1 Q02388 VAR_011196 p.Gly2674Asp LP/P - Epidermolysis bullosa dystrophica, autosomal recessive (RDEB) [MIM:226600]
COL7A1 Q02388 VAR_011197 p.Gly2713Asp LP/P rs369591910 Epidermolysis bullosa dystrophica, autosomal dominant (DDEB) [MIM:131750]
COL7A1 Q02388 VAR_011198 p.Gly2713Arg LP/P - Epidermolysis bullosa pruriginosa (EBP) [MIM:604129]
COL7A1 Q02388 VAR_011199 p.Gly2740Ala LP/P - Epidermolysis bullosa dystrophica, autosomal recessive (RDEB) [MIM:226600]
COL7A1 Q02388 VAR_011200 p.Gly2775Ser LP/P rs1333259313 Epidermolysis bullosa dystrophica, autosomal recessive (RDEB) [MIM:226600]
COL7A1 Q02388 VAR_011201 p.Arg2791Trp LP/P rs142566193 Epidermolysis bullosa dystrophica, autosomal dominant (DDEB) [MIM:131750]
COL7A1 Q02388 VAR_015519 p.Gly1595Arg LP/P rs121912840 Nail disorder, non-syndromic congenital, 8 (NDNC8) [MIM:607523]
COL7A1 Q02388 VAR_015520 p.Gly1815Arg LP/P rs121912841 Nail disorder, non-syndromic congenital, 8 (NDNC8) [MIM:607523]
COL7A1 Q02388 VAR_033786 p.Pro2429Leu LB/B rs2229822 -
COL7A1 Q02388 VAR_035740 p.Thr119Pro US - A breast cancer sample
COL7A1 Q02388 VAR_035741 p.Pro1364Thr US - A breast cancer sample
COL7A1 Q02388 VAR_035742 p.Arg1366Trp US rs147089666 A breast cancer sample

COL7A1 Q02388 VAR_048766 p.Arg1120Lys LB/B rs2228563 -
COL7A1 Q02388 VAR_064994 p.Gly1845Arg LP/P - Epidermolysis bullosa dystrophica, autosomal recessive (RDEB) [MIM:226600]
COL7A1 Q02388 VAR_064995 p.Lys1981Arg LP/P - Epidermolysis bullosa dystrophica, autosomal recessive (RDEB) [MIM:226600]
COL7A1 Q02388 VAR_064996 p.Arg2069Cys LP/P rs121912855 Epidermolysis bullosa dystrophica, autosomal recessive (RDEB) [MIM:226600]
COL7A1 Q02388 VAR_064997 p.Gly2070Arg LP/P - Epidermolysis bullosa dystrophica, autosomal dominant (DDEB) [MIM:131750]
COL7A1 Q02388 VAR_064998 p.Gly2221Ala LP/P - Epidermolysis bullosa dystrophica, autosomal recessive (RDEB) [MIM:226600]
COL7A1 Q02388 VAR_064999 p.Gly2296Glu LP/P - Epidermolysis bullosa dystrophica, autosomal recessive (RDEB) [MIM:226600]
COL7A1 Q02388 VAR_065000 p.Gly2557Arg LP/P - Epidermolysis bullosa dystrophica, autosomal recessive (RDEB) [MIM:226600]
COL7A1 Q02388 VAR_065001 p.Arg2622Trp LP/P rs139318843 Epidermolysis bullosa dystrophica, autosomal recessive (RDEB) [MIM:226600]
COL8A2 P25067 VAR_017893 p.Gly3Arg LB/B rs115156902 -
COL8A2 P25067 VAR_017894 p.Arg155Gln LB/B rs75864656 -
COL8A2 P25067 VAR_017895 p.Arg304Gln LP/P rs369487110 Corneal dystrophy, Fuchs endothelial, 1 (FECD1) [MIM:136800]
COL8A2 P25067 VAR_017896 p.Gly357Arg US rs199786966 Corneal dystrophy, Fuchs endothelial, 1 (FECD1) [MIM:136800]
COL8A2 P25067 VAR_017897 p.Arg434His LP/P rs201235688 Corneal dystrophy, Fuchs endothelial, 1 (FECD1) [MIM:136800]
COL8A2 P25067 VAR_017898 p.Gln455Lys LP/P rs80358191 Corneal dystrophy, Fuchs endothelial, 1 (FECD1) [MIM:136800]
COL8A2 P25067 VAR_017898 p.Gln455Lys LP/P rs80358191 Corneal dystrophy, posterior polymorphous, 2 (PPCD2) [MIM:609140]
COL8A2 P25067 VAR_017899 p.Pro575Leu US rs145553904 Corneal dystrophy, Fuchs endothelial, 1 (FECD1) [MIM:136800]
COL8A2 P25067 VAR_017900 p.Thr645Ile LB/B rs200767854 -
COL8A2 P25067 VAR_021387 p.Thr502Met LB/B rs117860804 -
COL9A1 P20849 VAR_023326 p.Arg870Lys LB/B rs1056921 -
COL9A1 P20849 VAR_023327 p.Val882Leu LB/B rs1056923 -
COL9A1 P20849 VAR_026463 p.Ser339Pro LB/B rs592121 -
COL9A1 P20849 VAR_026464 p.Gln621Arg LB/B rs1135056 -
COL9A1 P20849 VAR_055668 p.Glu684Lys LB/B rs35470562 -
COL9A1 P20849 VAR_055669 p.Met767Val LB/B rs6910140 -
COL9A2 Q14055 VAR_012658 p.Gln326Trp LP/P rs137853213 Intervertebral disc disease (IDD) [MIM:603932]
COL9A2 Q14055 VAR_012659 p.Gln326Arg LB/B rs2228564 -
COL9A2 Q14055 VAR_020014 p.Val581Ile LB/B rs3737821 -
COL9A2 Q14055 VAR_026465 p.Thr246Met LB/B rs2228565 -
COL9A2 Q14055 VAR_026466 p.Leu335Val LB/B rs2228567 -
COL9A3 Q14050 VAR_026467 p.Arg103Gln LB/B rs142639450 -
COL9A3 Q14050 VAR_026468 p.Arg103Trp LB/B rs61734651 -
COL9A3 Q14050 VAR_026469 p.Pro296Leu LB/B rs45628843 -
COL9A3 Q14050 VAR_026470 p.Arg402Gln LB/B rs373519549 -
COL9A3 Q14050 VAR_026471 p.Ala435Glu LB/B rs751557 -

COL9A3 Q14050 VAR_048808 p.Pro94Ser LB/B rs35908728 -
COL9A3 Q14050 VAR_072736 p.Gly35Asp LP/P rs1390736361 Multiple epiphyseal dysplasia 3 (EDM3) [MIM:600969]
COLEC10 Q9Y6Z7 VAR_078812 p.Cys176Trp LP/P rs773764995 3MC syndrome 3 (3MC3) [MIM:248340]
COLEC11 Q9BWP8 VAR_038143 p.His219Arg LB/B rs7567833 -
COLEC11 Q9BWP8 VAR_065901 p.Ser169Pro LP/P rs387907075 3MC syndrome 2 (3MC2) [MIM:265050]
COLEC11 Q9BWP8 VAR_065902 p.Gly204Ser LP/P rs387907076 3MC syndrome 2 (3MC2) [MIM:265050]
COLEC11 Q9BWP8 VAR_078813 p.Ala166Thr US - 3MC syndrome 2 (3MC2) [MIM:265050]
COLEC12 Q5KU26 VAR_038853 p.Lys91Glu LB/B rs17855029 -
COLEC12 Q5KU26 VAR_038854 p.Ile487Val LB/B rs8098850 -
COLEC12 Q5KU26 VAR_038855 p.Ser522Pro LB/B rs2305025 -
COLEC12 Q5KU26 VAR_038856 p.Gly606Ser LB/B rs2305027 -
COLGALT1 Q8NBJ5 VAR_081752 p.Leu151Arg LP/P rs1478523191 Brain small vessel disease 3 (BSVD3) [MIM:618360]
COLGALT1 Q8NBJ5 VAR_081753 p.Ala154Pro LP/P rs181844791 Brain small vessel disease 3 (BSVD3) [MIM:618360]
COLGALT1 Q8NBJ5 VAR_081754 p.Gly377Arg LP/P rs1568481244 Brain small vessel disease 3 (BSVD3) [MIM:618360]
COLGALT2 Q8IYK4 VAR_036978 p.Val475Ile US - A breast cancer sample
COLQ Q9Y215 VAR_010133 p.Pro59Gln LP/P - Myasthenic syndrome, congenital, 5 (CMS5) [MIM:603034]
COLQ Q9Y215 VAR_010134 p.Asp342Glu LP/P rs758554049 Myasthenic syndrome, congenital, 5 (CMS5) [MIM:603034]
COLQ Q9Y215 VAR_010135 p.Arg410Gln LP/P rs1025361623 Myasthenic syndrome, congenital, 5 (CMS5) [MIM:603034]
COLQ Q9Y215 VAR_010136 p.Tyr430Ser LP/P rs121908923 Myasthenic syndrome, congenital, 5 (CMS5) [MIM:603034]
COLQ Q9Y215 VAR_010137 p.Cys444Tyr LP/P - Myasthenic syndrome, congenital, 5 (CMS5) [MIM:603034]
COLQ Q9Y215 VAR_048809 p.Ser312Gly LB/B rs6782980 -
COLQ Q9Y215 VAR_071710 p.Ile337Thr LP/P rs1057521153 Myasthenic syndrome, congenital, 5 (CMS5) [MIM:603034]
COMMD10 Q9Y6G5 VAR_061122 p.Ile128Ser LB/B rs1129495 -
COMMD2 Q86X83 VAR_028010 p.Ile113Leu LB/B rs9843784 -
COMMD2 Q86X83 VAR_028011 p.Gln177His LB/B rs1546732 -
COMMD3 Q9UBI1 VAR_061121 p.Arg18Gly LB/B rs11552445 -
COMMD5 Q9GZQ3 VAR_020130 p.Ala6Thr LB/B rs1209879 -
COMMD5 Q9GZQ3 VAR_048812 p.Gln69His LB/B rs421427 -
COMMD6 Q7Z4G1 VAR_048813 p.His52Asn LB/B rs1063485 -
COMMD8 Q9NX08 VAR_048814 p.Ala17Pro LB/B rs35444219 -
COMP P49747 VAR_007614 p.Asp290Asn LP/P - Pseudoachondroplasia (PSACH) [MIM:177170]
COMP P49747 VAR_007615 p.Gly299Arg LP/P - Pseudoachondroplasia (PSACH) [MIM:177170]
COMP P49747 VAR_007616 p.Cys328Arg LP/P rs137852653 Pseudoachondroplasia (PSACH) [MIM:177170]
COMP P49747 VAR_007617 p.Asp342Tyr LP/P rs137852652 Multiple epiphyseal dysplasia 1 (EDM1) [MIM:132400]
COMP P49747 VAR_007618 p.Asp349Val LP/P - Pseudoachondroplasia (PSACH) [MIM:177170]
COMP P49747 VAR_007619 p.Asp361Val LP/P - Multiple epiphyseal dysplasia 1 (EDM1) [MIM:132400]
COMP P49747 VAR_007620 p.Asp361Tyr LP/P - Multiple epiphyseal dysplasia 1 (EDM1) [MIM:132400]
COMP P49747 VAR_007622 p.Cys371Ser LP/P - Multiple epiphyseal dysplasia 1 (EDM1) [MIM:132400]

COMP	P49747	VAR_007625	p.Cys387Gly	LP/P	-	Pseudoachondroplasia (PSACH) [MIM:177170]
COMP	P49747	VAR_007627	p.Asp408Tyr	LP/P	-	Multiple epiphyseal dysplasia 1 (EDM1) [MIM:132400]
COMP	P49747	VAR_007628	p.Gly440Glu	LP/P	-	Pseudoachondroplasia (PSACH) [MIM:177170]
COMP	P49747	VAR_007629	p.Gly440Arg	LP/P	rs1601053997	Pseudoachondroplasia (PSACH) [MIM:177170]
COMP	P49747	VAR_007630	p.Asn453Ser	LP/P	rs28936668	Multiple epiphyseal dysplasia 1 (EDM1) [MIM:132400]
COMP	P49747	VAR_007632	p.Cys468Tyr	LP/P	rs137852651	Pseudoachondroplasia (PSACH) [MIM:177170]
COMP	P49747	VAR_007634	p.Asp472Tyr	LP/P	rs137852650	Pseudoachondroplasia (PSACH) [MIM:177170]
COMP	P49747	VAR_007635	p.Asp473Gly	LP/P	rs28936669	Pseudoachondroplasia (PSACH) [MIM:177170]
COMP	P49747	VAR_007637	p.Asp482Gly	LP/P	-	Pseudoachondroplasia (PSACH) [MIM:177170]
COMP	P49747	VAR_007639	p.Asp518Asn	LP/P	rs1359984033	Pseudoachondroplasia (PSACH) [MIM:177170]
COMP	P49747	VAR_007640	p.Asn523Lys	LP/P	rs137852654	Multiple epiphyseal dysplasia 1 (EDM1) [MIM:132400]
COMP	P49747	VAR_007641	p.Thr585Met	LP/P	rs312262900	Pseudoachondroplasia (PSACH) [MIM:177170]
COMP	P49747	VAR_007642	p.Thr585Arg	LP/P	rs312262900	Multiple epiphyseal dysplasia 1 (EDM1) [MIM:132400]
COMP	P49747	VAR_007642	p.Thr585Arg	LP/P	rs312262900	Pseudoachondroplasia (PSACH) [MIM:177170]
COMP	P49747	VAR_016254	p.Glu50Asp	LB/B	-	-
COMP	P49747	VAR_016255	p.Leu51Trp	LB/B	-	-
COMP	P49747	VAR_016257	p.Ala109Gly	LB/B	-	-
COMP	P49747	VAR_016258	p.Arg224Gly	LB/B	-	-
COMP	P49747	VAR_016261	p.Arg285Pro	LB/B	-	-
COMP	P49747	VAR_017102	p.Cys348Arg	LP/P	rs137852656	Pseudoachondroplasia (PSACH) [MIM:177170]
COMP	P49747	VAR_017103	p.Gly719Asp	LP/P	rs137852655	Pseudoachondroplasia (PSACH) [MIM:177170]
COMP	P49747	VAR_026239	p.Pro276Arg	LP/P	rs1311845746	Multiple epiphyseal dysplasia 1 (EDM1) [MIM:132400]
COMP	P49747	VAR_026240	p.Asp420Ala	LP/P	-	Multiple epiphyseal dysplasia 1 (EDM1) [MIM:132400]
COMP	P49747	VAR_046796	p.Arg381Cys	LB/B	rs3179763	-
COMP	P49747	VAR_066789	p.Gly167Glu	LP/P	rs763887855	Multiple epiphyseal dysplasia 1 (EDM1) [MIM:132400]
COMP	P49747	VAR_066790	p.Pro234Ser	LP/P	rs557483957	Pseudoachondroplasia (PSACH) [MIM:177170]
COMP	P49747	VAR_066791	p.Asp290Gly	LP/P	rs1568556118	Pseudoachondroplasia (PSACH) [MIM:177170]
COMP	P49747	VAR_066792	p.Ser298Leu	LP/P	-	Multiple epiphyseal dysplasia 1 (EDM1) [MIM:132400]
COMP	P49747	VAR_066793	p.Ala311Asp	LP/P	-	Multiple epiphyseal dysplasia 1 (EDM1) [MIM:132400]
COMP	P49747	VAR_066794	p.Asp317Gly	LP/P	-	Multiple epiphyseal dysplasia 1 (EDM1) [MIM:132400]
COMP	P49747	VAR_066795	p.Asp326Gly	LP/P	-	Multiple epiphyseal dysplasia 1 (EDM1) [MIM:132400]
COMP	P49747	VAR_066796	p.Asp326Tyr	LP/P	-	Pseudoachondroplasia (PSACH) [MIM:177170]
COMP	P49747	VAR_066798	p.Cys348Phe	LP/P	-	Multiple epiphyseal dysplasia 1 (EDM1) [MIM:132400]
COMP	P49747	VAR_066800	p.Cys371Tyr	LP/P	rs1057521130	Multiple epiphyseal dysplasia 1 (EDM1) [MIM:132400]
COMP	P49747	VAR_066801	p.Asp374Asn	LP/P	-	Multiple epiphyseal dysplasia 1 (EDM1) [MIM:132400]
COMP	P49747	VAR_066802	p.Asp376Asn	LP/P	-	Multiple epiphyseal dysplasia 1 (EDM1) [MIM:132400]
COMP	P49747	VAR_066803	p.Asp378Val	LP/P	-	Pseudoachondroplasia (PSACH) [MIM:177170]
COMP	P49747	VAR_066804	p.Asp385Asn	LP/P	-	Multiple epiphyseal dysplasia 1 (EDM1) [MIM:132400]
COMP	P49747	VAR_066805	p.Asp385Tyr	LP/P	rs1601054715	Multiple epiphyseal dysplasia 1 (EDM1) [MIM:132400]
COMP	P49747	VAR_066807	p.Cys387Arg	LP/P	-	Pseudoachondroplasia (PSACH) [MIM:177170]
COMP	P49747	VAR_066808	p.Asp397His	LP/P	-	Multiple epiphyseal dysplasia 1 (EDM1) [MIM:132400]
COMP	P49747	VAR_066810	p.Gly404Arg	LP/P	-	Multiple epiphyseal dysplasia 1 (EDM1) [MIM:132400]

COMP P49747 VAR_066811 p.Cys410Tyr LP/P - Multiple epiphyseal dysplasia 1 (EDM1) [MIM:132400]
COMP P49747 VAR_066812 p.Asn415Lys LP/P - Multiple epiphyseal dysplasia 1 (EDM1) [MIM:132400]
COMP P49747 VAR_066813 p.Gly427Glu LP/P - Multiple epiphyseal dysplasia 1 (EDM1) [MIM:132400]
COMP P49747 VAR_066815 p.Asp446Asn LP/P - Pseudoachondroplasia (PSACH) [MIM:177170]
COMP P49747 VAR_066816 p.Cys448Ser LP/P - Pseudoachondroplasia (PSACH) [MIM:177170]
COMP P49747 VAR_066819 p.Asp473His LP/P - Pseudoachondroplasia (PSACH) [MIM:177170]
COMP P49747 VAR_066820 p.Asp475Asn LP/P - Pseudoachondroplasia (PSACH) [MIM:177170]
COMP P49747 VAR_066821 p.Gly501Asp LP/P rs1555791425 Multiple epiphyseal dysplasia 1 (EDM1) [MIM:132400]
COMP P49747 VAR_066822 p.Asp507Gly LP/P - Pseudoachondroplasia (PSACH) [MIM:177170]
COMP P49747 VAR_066823 p.Asp511Gly LP/P - Pseudoachondroplasia (PSACH) [MIM:177170]
COMP P49747 VAR_066824 p.Asp515Gly LP/P - Pseudoachondroplasia (PSACH) [MIM:177170]
COMP P49747 VAR_066825 p.Thr529Ile LP/P rs312262903 Pseudoachondroplasia (PSACH) [MIM:177170]
COMP P49747 VAR_066826 p.Arg718Pro LP/P rs149551600 Multiple epiphyseal dysplasia 1 (EDM1) [MIM:132400]
COMP P49747 VAR_066827 p.Arg718Trp LP/P rs28936368 Multiple epiphyseal dysplasia 1 (EDM1) [MIM:132400]
COMP P49747 VAR_066828 p.Gly719Ser LP/P rs312262904 Pseudoachondroplasia (PSACH) [MIM:177170]
COMP P49747 VAR_066829 p.Gln756Arg LB/B rs61752496 -
COMT P21964 VAR_005139 p.Val158Met LB/B rs4680 -
COMT P21964 VAR_013925 p.Cys34Ser LB/B rs6270 -
COMT P21964 VAR_013926 p.Ala72Ser LB/B rs6267 -
COMT P21964 VAR_020274 p.Ala102Thr LB/B rs5031015 -
COMT P21964 VAR_020275 p.Ala146Val LB/B rs4986871 -
COPA P53621 VAR_033803 p.Val1040Gly LB/B rs34997807 -
COPA P53621 VAR_066525 p.Ile164Val US - -
COPA P53621 VAR_073844 p.Lys230Asn LP/P rs864309710 Autoimmune interstitial lung, joint, and kidney disease (AILJK) [MIM:616414]
COPA P53621 VAR_073845 p.Arg233His LP/P rs794727993 Autoimmune interstitial lung, joint, and kidney disease (AILJK) [MIM:616414]
COPA P53621 VAR_073846 p.Glu241Lys LP/P rs794727995 Autoimmune interstitial lung, joint, and kidney disease (AILJK) [MIM:616414]
COPA P53621 VAR_073847 p.Asp243Gly LP/P rs794727994 Autoimmune interstitial lung, joint, and kidney disease (AILJK) [MIM:616414]
COPB2 P35606 VAR_080601 p.Arg254Cys US rs1229568621 Microcephaly 19, primary, autosomal recessive (MCPH19) [MIM:617800]
COPE O14579 VAR_054032 p.Ser13Cys LB/B rs2231987 -
COPE O14579 VAR_054033 p.Thr117Ile LB/B rs10330 -
COPG1 Q9Y678 VAR_054039 p.Met681Thr LB/B rs15648 -
COPG2 Q9UBF2 VAR_060181 p.Ile547Leu LB/B rs10128 -
COPG2 Q9UBF2 VAR_060182 p.Pro626Leu LB/B rs17333054 -
COPRS Q9NQ92 VAR_043538 p.Ser43Gly LB/B rs8068049 -
COQ10A Q96MF6 VAR_025703 p.Pro79His LB/B rs11543258 -
COQ10A Q96MF6 VAR_048828 p.Pro231Ser LB/B rs3184994 -
COQ10B Q9H8M1 VAR_033823 p.Leu48Phe LB/B rs34946819 -
COQ2 Q96H96 VAR_025701 p.Tyr247Cys LP/P - Coenzyme Q10 deficiency, primary, 1 (COQ10D1) [MIM:607426]
COQ2 Q96H96 VAR_068161 p.Ser96Asn LP/P - Coenzyme Q10 deficiency, primary, 1 (COQ10D1) [MIM:607426]

COQ2	Q96H96	VAR_068162	p.Arg147His	LP/P	-	Coenzyme Q10 deficiency, primary, 1 (COQ10D1) [MIM:607426]
COQ2	Q96H96	VAR_068163	p.Asn178Ser	LP/P	-	Coenzyme Q10 deficiency, primary, 1 (COQ10D1) [MIM:607426]
COQ2	Q96H96	VAR_070237	p.Leu16Val	LB/B	rs6818847	-
COQ2	Q96H96	VAR_070238	p.Pro22Leu	LB/B	-	-
COQ2	Q96H96	VAR_070239	p.Phe29Leu	LP/P	-	Multiple system atrophy 1 (MSA1) [MIM:146500]
COQ2	Q96H96	VAR_070240	p.Pro49His	LP/P	-	Multiple system atrophy 1 (MSA1) [MIM:146500]
COQ2	Q96H96	VAR_070241	p.Ser57Thr	LP/P	-	Multiple system atrophy 1 (MSA1) [MIM:146500]
COQ2	Q96H96	VAR_070242	p.Arg69His	LB/B	-	-
COQ2	Q96H96	VAR_070243	p.Met78Val	LP/P	-	Multiple system atrophy 1 (MSA1) [MIM:146500]
COQ2	Q96H96	VAR_070244	p.Ile97Thr	LP/P	-	Multiple system atrophy 1 (MSA1) [MIM:146500]
COQ2	Q96H96	VAR_070245	p.Pro107Ser	LP/P	-	Multiple system atrophy 1 (MSA1) [MIM:146500]
COQ2	Q96H96	VAR_070246	p.Ser113Phe	LP/P	-	Multiple system atrophy 1 (MSA1) [MIM:146500]
COQ2	Q96H96	VAR_070247	p.Thr267Ala	LP/P	-	Multiple system atrophy 1 (MSA1) [MIM:146500]
COQ2	Q96H96	VAR_070248	p.Ser297Cys	LP/P	-	Multiple system atrophy 1 (MSA1) [MIM:146500]
COQ2	Q96H96	VAR_070249	p.Asn336His	LB/B	-	-
COQ2	Q96H96	VAR_070250	p.Arg337Gln	LP/P	-	Multiple system atrophy 1 (MSA1) [MIM:146500]
COQ2	Q96H96	VAR_070251	p.Val343Ala	LP/P	rs397514727	Multiple system atrophy 1 (MSA1) [MIM:146500]
COQ2	Q96H96	VAR_076913	p.Met132Arg	LP/P	-	Coenzyme Q10 deficiency, primary, 1 (COQ10D1) [MIM:607426]
COQ2	Q96H96	VAR_076914	p.Ala252Val	LP/P	-	Coenzyme Q10 deficiency, primary, 1 (COQ10D1) [MIM:607426]
COQ2	Q96H96	VAR_078121	p.Gly340Ala	US	-	Coenzyme Q10 deficiency, primary, 1 (COQ10D1) [MIM:607426]
COQ3	Q9NZJ6	VAR_020789	p.Ser272Gly	LB/B	rs6925344	-
COQ3	Q9NZJ6	VAR_020790	p.Tyr329His	LB/B	rs4144164	-
COQ3	Q9NZJ6	VAR_061925	p.Lys134Glu	LB/B	rs11548336	-
COQ4	Q9Y3A0	VAR_048829	p.Arg20Gln	LB/B	rs9697215	-
COQ4	Q9Y3A0	VAR_054861	p.Gly50Ala	LB/B	rs3003601	-
COQ4	Q9Y3A0	VAR_073356	p.Leu52Ser	LP/P	rs786204770	Coenzyme Q10 deficiency, primary, 7 (COQ10D7) [MIM:616276]
COQ4	Q9Y3A0	VAR_073357	p.Pro64Ser	LP/P	rs766317663	Coenzyme Q10 deficiency, primary, 7 (COQ10D7) [MIM:616276]
COQ4	Q9Y3A0	VAR_073358	p.Arg145Gly	LP/P	rs774395996	Coenzyme Q10 deficiency, primary, 7 (COQ10D7) [MIM:616276]
COQ4	Q9Y3A0	VAR_073360	p.Arg240Cys	LP/P	rs143441644	Coenzyme Q10 deficiency, primary, 7 (COQ10D7) [MIM:616276]
COQ5	Q5HYK3	VAR_025702	p.Ala152Thr	LB/B	rs3742049	-
COQ6	Q9Y2Z9	VAR_014953	p.Val406Met	LB/B	rs8500	-
COQ6	Q9Y2Z9	VAR_033813	p.Asp339Val	LB/B	rs2074930	-
COQ6	Q9Y2Z9	VAR_033814	p.Thr395Met	LB/B	rs34746680	-
COQ6	Q9Y2Z9	VAR_052691	p.Asp300Tyr	LB/B	rs1044640	-
COQ6	Q9Y2Z9	VAR_068216	p.Gly255Arg	LP/P	rs1057519350	Coenzyme Q10 deficiency, primary, 6 (COQ10D6) [MIM:614650]
COQ6	Q9Y2Z9	VAR_068217	p.Glu287Lys	LB/B	rs17851169	-
COQ6	Q9Y2Z9	VAR_068218	p.Ala353Asp	LP/P	rs397514479	Coenzyme Q10 deficiency, primary, 6 (COQ10D6) [MIM:614650]
COQ6	Q9Y2Z9	VAR_075225	p.Asp208His	LP/P	rs606231262	-

COQ6 Q9Y2Z9 VAR_078122 p.Pro261Leu US rs371260604 Coenzyme Q10 deficiency, primary, 6 (COQ10D6) [MIM:614650]
COQ7 Q99807 VAR_055148 p.Thr103Met LB/B rs11074359 -
COQ7 Q99807 VAR_076370 p.Val141Glu LP/P rs864321686 Coenzyme Q10 deficiency, primary, 8 (COQ10D8) [MIM:616733]
COQ8A Q8NI60 VAR_020319 p.His85Gln LB/B rs2297411 -
COQ8A Q8NI60 VAR_044402 p.Arg213Trp LP/P rs119468005 Coenzyme Q10 deficiency, primary, 4 (COQ10D4) [MIM:612016]
COQ8A Q8NI60 VAR_044403 p.Gly272Asp LP/P rs119468006 Coenzyme Q10 deficiency, primary, 4 (COQ10D4) [MIM:612016]
COQ8A Q8NI60 VAR_044404 p.Gly272Val LP/P rs119468006 Coenzyme Q10 deficiency, primary, 4 (COQ10D4) [MIM:612016]
COQ8A Q8NI60 VAR_044405 p.Tyr514Cys LP/P rs119468008 Coenzyme Q10 deficiency, primary, 4 (COQ10D4) [MIM:612016]
COQ8A Q8NI60 VAR_044406 p.Gly549Ser LP/P rs119468009 Coenzyme Q10 deficiency, primary, 4 (COQ10D4) [MIM:612016]
COQ8A Q8NI60 VAR_044407 p.Glu551Lys LP/P rs119468004 Coenzyme Q10 deficiency, primary, 4 (COQ10D4) [MIM:612016]
COQ8A Q8NI60 VAR_045576 p.Ile341Thr LB/B rs55798516 -
COQ8A Q8NI60 VAR_072622 p.Arg271Cys LP/P rs145034527 Coenzyme Q10 deficiency, primary, 4 (COQ10D4) [MIM:612016]
COQ8A Q8NI60 VAR_072623 p.Arg299Trp LP/P rs201908721 Coenzyme Q10 deficiency, primary, 4 (COQ10D4) [MIM:612016]
COQ8A Q8NI60 VAR_072624 p.Ala304Thr LP/P rs778798354 Coenzyme Q10 deficiency, primary, 4 (COQ10D4) [MIM:612016]
COQ8A Q8NI60 VAR_072625 p.Ala304Val LP/P rs748118737 Coenzyme Q10 deficiency, primary, 4 (COQ10D4) [MIM:612016]
COQ8A Q8NI60 VAR_072626 p.Tyr429Cys LP/P rs144147839 Coenzyme Q10 deficiency, primary, 4 (COQ10D4) [MIM:612016]
COQ8A Q8NI60 VAR_072627 p.Pro602Arg LP/P rs61995958 Coenzyme Q10 deficiency, primary, 4 (COQ10D4) [MIM:612016]
COQ8A Q8NI60 VAR_076860 p.Phe578Val US - Coenzyme Q10 deficiency, primary, 4 (COQ10D4) [MIM:612016]
COQ8B Q96D53 VAR_029995 p.His174Arg LB/B rs3865452 -
COQ8B Q96D53 VAR_041420 p.Arg78Cys LB/B rs11538384 -
COQ8B Q96D53 VAR_041421 p.Thr318Met LB/B rs55899516 -
COQ8B Q96D53 VAR_041422 p.Thr352Arg LB/B rs36012476 -
COQ8B Q96D53 VAR_041423 p.Thr462Met LB/B rs56083906 -
COQ8B Q96D53 VAR_070552 p.Arg178Trp LP/P rs398122978 Nephrotic syndrome 9 (NPHS9) [MIM:615573]
COQ8B Q96D53 VAR_070553 p.Asp286Gly LP/P rs398122979 Nephrotic syndrome 9 (NPHS9) [MIM:615573]
COQ8B Q96D53 VAR_070554 p.Arg320Trp LP/P rs369573693 Nephrotic syndrome 9 (NPHS9) [MIM:615573]
COQ8B Q96D53 VAR_070555 p.Arg343Trp LP/P rs398122981 Nephrotic syndrome 9 (NPHS9) [MIM:615573]
COQ8B Q96D53 VAR_070556 p.Arg477Gln LP/P rs1057519347 Nephrotic syndrome 9 (NPHS9) [MIM:615573]
COQ8B Q96D53 VAR_076861 p.Leu98Arg LP/P - Nephrotic syndrome 9 (NPHS9) [MIM:615573]
COQ8B Q96D53 VAR_076862 p.Pro310Leu LP/P - Nephrotic syndrome 9 (NPHS9) [MIM:615573]
COQ8B Q96D53 VAR_076863 p.Ala498Glu LP/P - Nephrotic syndrome 9 (NPHS9) [MIM:615573]
CORIN Q9Y5Q5 VAR_038000 p.Cys13Tyr LB/B rs2289433 -
CORIN Q9Y5Q5 VAR_038001 p.His525Arg LB/B rs11934749 -
CORIN Q9Y5Q5 VAR_067795 p.Lys317Glu LP/P rs387906894 Pre-eclampsia/eclampsia 5 (PEE5) [MIM:614595]

CORIN Q9Y5Q5 VAR_067796 p.Asp444Gly LB/B rs13105608 -
CORIN Q9Y5Q5 VAR_067797 p.Ser472Gly LP/P rs387906895 Pre-eclampsia/eclampsia 5 (PEE5) [MIM:614595]
CORO1A P31146 VAR_011956 p.Arg415Lys LB/B rs1804109 -
CORO1A P31146 VAR_070447 p.Val134Met LP/P rs397514755 Immunodeficiency 8 (IMD8) [MIM:615401]
CORO1B Q9BR76 VAR_035877 p.Val411Met US rs756117196 A colorectal cancer sample
CORO1B Q9BR76 VAR_053389 p.Arg476Leu LB/B rs2286624 -
CORO2A Q92828 VAR_053390 p.Arg296His LB/B rs2231666 -
CORO2A Q92828 VAR_053391 p.Arg495Leu LB/B rs35787916 -
CORO2B Q9UQ03 VAR_035878 p.Pro318Leu US rs1314209013 A colorectal cancer sample
CORO2B Q9UQ03 VAR_058323 p.Leu238Val LB/B rs17852400 -
CORO7 P57737 VAR_057585 p.Ala174Val LB/B rs17137007 -
CORO7 P57737 VAR_057586 p.Arg193Gln LB/B rs3747579 -
CORO7 P57737 VAR_057587 p.Leu257Ser LB/B rs35357594 -
CORO7 P57737 VAR_057588 p.Ala403Thr LB/B rs9928967 -
COX10 Q12887 VAR_026562 p.Thr196Lys LP/P rs104894555 Mitochondrial complex IV deficiency, nuclear type 3 (MC4DN3) [MIM:619046]
COX10 Q12887 VAR_026563 p.Asn204Lys LP/P rs104894560 Mitochondrial complex IV deficiency, nuclear type 3 (MC4DN3) [MIM:619046]
COX10 Q12887 VAR_026564 p.Pro225Leu LP/P rs104894556 Mitochondrial complex IV deficiency, nuclear type 3 (MC4DN3) [MIM:619046]
COX10 Q12887 VAR_026565 p.Asp336Gly LP/P rs104894557 Mitochondrial complex IV deficiency, nuclear type 3 (MC4DN3) [MIM:619046]
COX10 Q12887 VAR_026566 p.Asp336Val LP/P rs104894557 Mitochondrial complex IV deficiency, nuclear type 3 (MC4DN3) [MIM:619046]
COX10 Q12887 VAR_057371 p.Thr28Ile LB/B rs16948978 -
COX10 Q12887 VAR_057372 p.Thr62Ser LB/B rs2230351 -
COX10 Q12887 VAR_057373 p.Tyr97Cys LB/B rs16948986 -
COX10 Q12887 VAR_060233 p.Arg159Gln LB/B rs2072279 -
COX10 Q12887 VAR_060234 p.Gly340Asp LB/B rs1050214 -
COX10 Q12887 VAR_064768 p.Leu258His LB/B rs587780911 -
COX10 Q12887 VAR_076181 p.Gly288Arg LP/P rs753048807 Mitochondrial complex IV deficiency, nuclear type 3 (MC4DN3) [MIM:619046]
COX10 Q12887 VAR_076182 p.Pro420Leu LP/P rs773079584 Mitochondrial complex IV deficiency, nuclear type 3 (MC4DN3) [MIM:619046]
COX11 Q9Y6N1 VAR_048831 p.Pro74Leu LB/B rs34080917 -
COX14 Q96I36 VAR_067038 p.Met19Ile LP/P rs587776904 Mitochondrial complex IV deficiency, nuclear type 10 (MC4DN10) [MIM:619053]
COX15 Q7KZN9 VAR_019596 p.Arg217Trp LP/P rs28939711 Mitochondrial complex IV deficiency, nuclear type 6 (MC4DN6) [MIM:615119]
COX15 Q7KZN9 VAR_033117 p.Ser344Pro LP/P rs397514662 Mitochondrial complex IV deficiency, nuclear type 6 (MC4DN6) [MIM:615119]
COX20 Q5RI15 VAR_080123 p.Thr52Pro LP/P rs587777004 Mitochondrial complex IV deficiency, nuclear type 11 (MC4DN11) [MIM:619054]
COX20 Q5RI15 VAR_080124 p.Asn118Ser LB/B rs61749963 -
COX4I1 P13073 VAR_002170 p.Tyr38Phe LB/B - -
COX4I1 P13073 VAR_061127 p.Ala3Thr LB/B rs11557187 -
COX4I1 P13073 VAR_084182 p.Pro152Thr LP/P - Mitochondrial complex IV deficiency, nuclear type 16 (MC4DN16) [MIM:619060]
COX4I2 Q96KJ9 VAR_033815 p.Arg161His LB/B rs11907253 -

COX4I2 Q96KJ9 VAR_058101 p.Glu138Lys LP/P rs119455950 Exocrine pancreatic insufficiency dyserythropoietic anemia and calvarial hyperostosis (EPIDACH) [MIM:612714]
COX5A P20674 VAR_078264 p.Arg107Cys LP/P - Mitochondrial complex IV deficiency, nuclear type 20 (MC4DN20) [MIM:619064]
COX6A2 Q02221 VAR_084183 p.Ser39Arg LP/P - Mitochondrial complex IV deficiency, nuclear type 18 (MC4DN18) [MIM:619062]
COX6A2 Q02221 VAR_084184 p.Cys43Arg LP/P rs1275864234 Mitochondrial complex IV deficiency, nuclear type 18 (MC4DN18) [MIM:619062]
COX6B1 P14854 VAR_046775 p.Arg20His LP/P rs121909602 Mitochondrial complex IV deficiency, nuclear type 7 (MC4DN7) [MIM:619051]
COX6B1 P14854 VAR_084178 p.Arg20Cys LP/P - Mitochondrial complex IV deficiency, nuclear type 7 (MC4DN7) [MIM:619051]
COX7A2 P14406 VAR_012319 p.Glu40Asp LB/B - -
COX7B2 Q8TF08 VAR_026231 p.His27Gln LB/B rs201505906 -
CP P00450 VAR_025655 p.Ile63Thr LB/B rs759185877 -
CP P00450 VAR_025656 p.Pro477Leu LB/B rs35331711 -
CP P00450 VAR_025657 p.Asp544Glu LB/B rs701753 -
CP P00450 VAR_025658 p.Thr551Ile LB/B rs61733458 -
CP P00450 VAR_025659 p.Arg793His LB/B rs115552500 -
CP P00450 VAR_025660 p.Thr841Arg LB/B rs56033670 -
CP P00450 VAR_032815 p.Arg367Cys LB/B rs34624984 -
CPA1 P15085 VAR_048593 p.Ala208Thr LB/B rs34474469 -
CPA1 P15085 VAR_054311 p.His276Arg LB/B rs17849959 -
CPA2 P48052 VAR_031204 p.Glu82Gly LB/B rs17850135 -
CPA3 P15088 VAR_033725 p.Thr171Met LB/B rs12489516 -
CPA3 P15088 VAR_048602 p.Ala81Ser LB/B rs2270523 -
CPA4 Q9UI42 VAR_020393 p.Pro157Thr LB/B rs3735051 -
CPA4 Q9UI42 VAR_020394 p.Gly303Cys LB/B rs2171492 -
CPA4 Q9UI42 VAR_048594 p.Leu27Phe LB/B rs34587586 -
CPA4 Q9UI42 VAR_048595 p.Arg183Leu LB/B rs3735053 -
CPA5 Q8WXQ8 VAR_017191 p.Pro79Ser LB/B rs17388190 -
CPA5 Q8WXQ8 VAR_017192 p.Leu336Ser LB/B rs11761888 -
CPA5 Q8WXQ8 VAR_017193 p.Glu338Asp LB/B rs17854248 -
CPA5 Q8WXQ8 VAR_048596 p.Ser378Gly LB/B rs11765961 -
CPA6 Q8N4T0 VAR_024241 p.Phe45Leu LB/B rs10957393 -
CPA6 Q8N4T0 VAR_025003 p.Ser173Cys LB/B rs17853192 -
CPA6 Q8N4T0 VAR_048597 p.Asn249Ser LB/B rs17343819 -
CPA6 Q8N4T0 VAR_066946 p.Gly267Arg LP/P rs61738009 Epilepsy, familial temporal lobe, 5 (ETL5) [MIM:614417]
CPA6 Q8N4T0 VAR_066947 p.Ala270Val LP/P rs114402678 Febrile seizures, familial, 11 (FEB11) [MIM:614418]
CPAMD8 Q8IZJ3 VAR_038655 p.Arg251Trp LB/B rs10426545 -
CPAMD8 Q8IZJ3 VAR_038656 p.Met265Thr LB/B rs4808551 -
CPAMD8 Q8IZJ3 VAR_038657 p.Arg294Gln LB/B rs3745340 -
CPAMD8 Q8IZJ3 VAR_038658 p.Asp539Glu LB/B rs3745335 -
CPAMD8 Q8IZJ3 VAR_038659 p.His546Arg LB/B rs1824152 -
CPAMD8 Q8IZJ3 VAR_038660 p.Pro736His LB/B rs9305083 -
CPAMD8 Q8IZJ3 VAR_038661 p.Val1156Ile LB/B rs2250918 -
CPAMD8 Q8IZJ3 VAR_038662 p.Thr1268Ile LB/B rs706761 -

CPAMD8 Q8IZJ3 VAR_038663 p.Gln1843Arg LB/B rs1054533 -
CPAMD8 Q8IZJ3 VAR_077933 p.Ser1404Pro LP/P rs1057519340 Anterior segment dysgenesis 8 (ASGD8)
[MIM:617319]
CPB1 P15086 VAR_048598 p.Asp208Asn LB/B rs1059502 -
CPB2 Q96IY4 VAR_022258 p.Ile347Thr LB/B rs1926447 -
CPB2 Q96IY4 VAR_032565 p.Ala169Thr LB/B rs3742264 -
CPD O75976 VAR_027771 p.Lys36Glu LB/B rs17857300 -
CPD O75976 VAR_027772 p.Glu454Gly LB/B rs17857301 -
CPD O75976 VAR_027773 p.His505Asn LB/B rs17854355 -
CPD O75976 VAR_027774 p.Thr899Ile LB/B rs1860543 -
CPE P16870 VAR_036011 p.Arg297Gln US rs541147146 A colorectal cancer sample
CPE P16870 VAR_048599 p.Trp235Arg LB/B rs34516004 -
CPEB1 Q9BZB8 VAR_076388 p.Met233Ile LB/B - -
CPEB3 Q8NE35 VAR_029776 p.Arg324Trp LB/B rs17853616 -
CPED1 A4D0V7 VAR_038664 p.Ile326Thr LB/B rs17143165 -
CPED1 A4D0V7 VAR_038665 p.Ala551Gly LB/B rs41281692 -
CPED1 A4D0V7 VAR_038666 p.Glu708Gly LB/B rs35793694 -
CPED1 A4D0V7 VAR_038667 p.Lys949Thr LB/B rs798911 -
CPLANE1 Q9H799 VAR_042948 p.Ile1437Thr LB/B rs6859950 -
CPLANE1 Q9H799 VAR_042949 p.Phe2033Cys LB/B rs10076911 -
CPLANE1 Q9H799 VAR_042950 p.Ile2143Val LB/B rs6884652 -
CPLANE1 Q9H799 VAR_042951 p.Pro2592Leu LB/B rs16903518 -
CPLANE1 Q9H799 VAR_042952 p.Gly3062Arg LB/B rs7702892 -
CPLANE1 Q9H799 VAR_068165 p.Arg1336Trp LP/P rs367543061 Joubert syndrome 17 (JBTS17) [MIM:614615]
CPLANE1 Q9H799 VAR_068166 p.Pro1794Leu LB/B rs75589774 -
CPLANE1 Q9H799 VAR_068167 p.Phe2033Ser LB/B rs10076911 -
CPLANE1 Q9H799 VAR_072544 p.Val2837Leu LP/P - Orofaciodigital syndrome 6 (OFD6) [MIM:277170]
CPLANE1 Q9H799 VAR_072553 p.Ser1127Leu LP/P rs375009168 Orofaciodigital syndrome 6 (OFD6)
[MIM:277170]
CPLANE1 Q9H799 VAR_072554 p.Arg1184Cys LP/P rs1434631255 Orofaciodigital syndrome 6 (OFD6)
[MIM:277170]
CPLANE1 Q9H799 VAR_072555 p.Arg1193Cys LP/P rs149170427 Orofaciodigital syndrome 6 (OFD6)
[MIM:277170]
CPLANE1 Q9H799 VAR_072556 p.Asp1287His LP/P rs606231261 Orofaciodigital syndrome 6 (OFD6)
[MIM:277170]
CPLANE1 Q9H799 VAR_076776 p.Trp322Gly LB/B - -
CPLANE1 Q9H799 VAR_076777 p.Ser875Phe LB/B rs794727154 -
CPLANE1 Q9H799 VAR_076778 p.Leu1196Arg LB/B - -
CPLANE1 Q9H799 VAR_076779 p.Gln1345Arg LP/P rs869312898 Orofaciodigital syndrome 6 (OFD6)
[MIM:277170]
CPLANE1 Q9H799 VAR_076780 p.Ser1772Gly LB/B rs79377186 -
CPLANE1 Q9H799 VAR_076781 p.Pro2750Ser LB/B rs377107065 -
CPLANE1 Q9H799 VAR_077558 p.Ala1200Val US rs141153181 Orofaciodigital syndrome 6 (OFD6)
[MIM:277170]
CPLANE2 Q9BU20 VAR_031772 p.Glu86Gly LB/B rs17849687 -
CPLANE2 Q9BU20 VAR_079173 p.Ile161Leu LB/B - -
CPLX1 O14810 VAR_080797 p.Leu128Met US rs371709824 Developmental and epileptic encephalopathy 63
(DEE63) [MIM:617976]
CPM P14384 VAR_048600 p.Arg24His LB/B rs7978197 -

CPM P14384 VAR_048601 p.Val133Ile LB/B rs7309831 -
CPN1 P15169 VAR_042415 p.Gly178Asp LP/P rs61751507 Carboxypeptidase N deficiency (CPND) [MIM:212070]
CPN2 P22792 VAR_019721 p.Val536Met LB/B rs11711157 -
CPN2 P22792 VAR_019722 p.Ala305Thr LB/B rs3732477 -
CPN2 P22792 VAR_065186 p.Gln509Arg LB/B rs4974538 -
CPNE1 Q99829 VAR_024423 p.Gln211Arg LB/B rs6579255 -
CPNE1 Q99829 VAR_048846 p.Pro347Arg LB/B rs12481228 -
CPNE1 Q99829 VAR_048847 p.Pro535Leu LB/B rs11543239 -
CPNE3 O75131 VAR_024424 p.Thr412Met LB/B rs2304789 -
CPNE3 O75131 VAR_048848 p.Glu252Asp LB/B rs41333046 -
CPNE5 Q9HCH3 VAR_020358 p.Asn33Ser LB/B rs3734334 -
CPNE5 Q9HCH3 VAR_021954 p.Arg582His LB/B rs3830138 -
CPNE7 Q9UBL6 VAR_021955 p.Phe77Leu LB/B rs455527 -
CPNE7 Q9UBL6 VAR_021956 p.Pro633Leu LB/B rs3751682 -
CPNE7 Q9UBL6 VAR_033822 p.Arg397Gln LB/B rs28568523 -
CPNE7 Q9UBL6 VAR_048849 p.Lys507Glu LB/B rs35731090 -
CPO Q8IVL8 VAR_027850 p.Met85Ile LB/B rs13420911 -
CPO Q8IVL8 VAR_027851 p.Ser134Arg LB/B rs11903403 -
CPO Q8IVL8 VAR_036012 p.Lys273Asn US rs896000260 A colorectal cancer sample
CPOX P36551 VAR_002152 p.Gly189Ser LP/P rs759347283 Hereditary coproporphyria (HCP) [MIM:121300]
CPOX P36551 VAR_002153 p.Gly197Trp LP/P - Hereditary coproporphyria (HCP) [MIM:121300]
CPOX P36551 VAR_002154 p.Glu201Lys LP/P rs1374394802 Hereditary coproporphyria (HCP) [MIM:121300]
CPOX P36551 VAR_002155 p.Pro249Ser LP/P - Hereditary coproporphyria (HCP) [MIM:121300]
CPOX P36551 VAR_002156 p.Asn272His LB/B rs1131857 -
CPOX P36551 VAR_002157 p.Gly280Arg LP/P - Hereditary coproporphyria (HCP) [MIM:121300]
CPOX P36551 VAR_002158 p.Val294Ile LB/B rs2228056 -
CPOX P36551 VAR_002159 p.His295Asp LP/P rs121917870 Hereditary coproporphyria (HCP) [MIM:121300]
CPOX P36551 VAR_002160 p.Arg331Trp LP/P rs121917866 Hereditary coproporphyria (HCP) [MIM:121300]
CPOX P36551 VAR_002162 p.Lys404Glu LP/P rs121917868 Harderoporphyria (HARPO) [MIM:618892]
CPOX P36551 VAR_002163 p.Trp427Arg LP/P - Hereditary coproporphyria (HCP) [MIM:121300]
CPOX P36551 VAR_019067 p.Ser208Phe LP/P rs121917872 Hereditary coproporphyria (HCP) [MIM:121300]
CPOX P36551 VAR_019068 p.Arg328Cys LP/P rs121917873 Hereditary coproporphyria (HCP) [MIM:121300]
CPOX P36551 VAR_019069 p.Arg447Cys LP/P rs28931603 Hereditary coproporphyria (HCP) [MIM:121300]
CPOX P36551 VAR_023444 p.Val135Ala LP/P rs201826432 Hereditary coproporphyria (HCP) [MIM:121300]
CPOX P36551 VAR_023445 p.Leu214Arg LP/P - Hereditary coproporphyria (HCP) [MIM:121300]
CPOX P36551 VAR_023446 p.Pro249Arg LP/P - Hereditary coproporphyria (HCP) [MIM:121300]
CPOX P36551 VAR_048827 p.Arg352Cys LB/B rs11921054 -
CPOX P36551 VAR_058005 p.Gly279Arg LP/P rs121917874 Hereditary coproporphyria (HCP) [MIM:121300]
CPOX P36551 VAR_084512 p.His327Arg LP/P - Harderoporphyria (HARPO) [MIM:618892]
CPPED1 Q9BRF8 VAR_039204 p.Ala19Asp LB/B rs3748976 -
CPPED1 Q9BRF8 VAR_039205 p.Val86Ile LB/B rs3748980 -
CPPED1 Q9BRF8 VAR_039206 p.Lys241Arg LB/B rs1713480 -
CPPED1 Q9BRF8 VAR_039207 p.His290Pro LB/B rs11645068 -
CPQ Q9Y646 VAR_037466 p.Ser138Asn LB/B rs34088584 -
CPS1 P31327 VAR_006834 p.Thr344Ala LB/B rs1047883 -
CPS1 P31327 VAR_006835 p.Thr544Met LP/P rs121912592 Carbamoyl phosphate synthetase 1 deficiency (CPS1D) [MIM:237300]

CPS1 P31327 VAR_014077 p.His337Arg LP/P rs28940283 Carbamoyl phosphate synthetase 1 deficiency (CPS1D) [MIM:237300]
CPS1 P31327 VAR_017562 p.Val457Gly LP/P rs371350538 Carbamoyl phosphate synthetase 1 deficiency (CPS1D) [MIM:237300]
CPS1 P31327 VAR_017563 p.Gln810Arg LP/P rs1553513864 Carbamoyl phosphate synthetase 1 deficiency (CPS1D) [MIM:237300]
CPS1 P31327 VAR_017564 p.Leu843Ser LP/P - Carbamoyl phosphate synthetase 1 deficiency (CPS1D) [MIM:237300]
CPS1 P31327 VAR_017565 p.Lys875Glu LB/B rs147062907 -
CPS1 P31327 VAR_017566 p.Phe1266Ser LB/B rs1047886 -
CPS1 P31327 VAR_017567 p.Met1283Leu LB/B rs1047887 -
CPS1 P31327 VAR_017568 p.Gly1376Ser LB/B rs140578009 -
CPS1 P31327 VAR_017569 p.Thr1406Asn LB/B rs1047891 -
CPS1 P31327 VAR_030675 p.Arg850His LP/P rs767694281 Carbamoyl phosphate synthetase 1 deficiency (CPS1D) [MIM:237300]
CPS1 P31327 VAR_030676 p.Ser918Pro LP/P - Carbamoyl phosphate synthetase 1 deficiency (CPS1D) [MIM:237300]
CPS1 P31327 VAR_061752 p.Thr344Ser LB/B rs1047883 -
CPS1 P31327 VAR_063560 p.Gly79Glu LP/P rs1265394565 Carbamoyl phosphate synthetase 1 deficiency (CPS1D) [MIM:237300]
CPS1 P31327 VAR_063561 p.Tyr212Asn LP/P - Carbamoyl phosphate synthetase 1 deficiency (CPS1D) [MIM:237300]
CPS1 P31327 VAR_063562 p.Lys280Asn LP/P rs753751183 Carbamoyl phosphate synthetase 1 deficiency (CPS1D) [MIM:237300]
CPS1 P31327 VAR_063563 p.Ala438Pro LP/P rs772497399 Carbamoyl phosphate synthetase 1 deficiency (CPS1D) [MIM:237300]
CPS1 P31327 VAR_063564 p.Arg587His LP/P rs1553512642 Carbamoyl phosphate synthetase 1 deficiency (CPS1D) [MIM:237300]
CPS1 P31327 VAR_063565 p.Gly593Arg LP/P rs1048119191 Carbamoyl phosphate synthetase 1 deficiency (CPS1D) [MIM:237300]
CPS1 P31327 VAR_063566 p.Glu651Lys LP/P - Carbamoyl phosphate synthetase 1 deficiency (CPS1D) [MIM:237300]
CPS1 P31327 VAR_063567 p.Asn674Ile LP/P - Carbamoyl phosphate synthetase 1 deficiency (CPS1D) [MIM:237300]
CPS1 P31327 VAR_063568 p.Arg780His LP/P rs758724746 Carbamoyl phosphate synthetase 1 deficiency (CPS1D) [MIM:237300]
CPS1 P31327 VAR_063569 p.Arg850Cys LP/P rs1015051007 Carbamoyl phosphate synthetase 1 deficiency (CPS1D) [MIM:237300]
CPS1 P31327 VAR_063570 p.Gly982Asp LP/P rs121912595 Carbamoyl phosphate synthetase 1 deficiency (CPS1D) [MIM:237300]
CPS1 P31327 VAR_063571 p.Gln1103Arg LP/P - Carbamoyl phosphate synthetase 1 deficiency (CPS1D) [MIM:237300]
CPS1 P31327 VAR_063572 p.Val1141Gly LP/P - Carbamoyl phosphate synthetase 1 deficiency (CPS1D) [MIM:237300]
CPS1 P31327 VAR_063573 p.His1195Pro LP/P - Carbamoyl phosphate synthetase 1 deficiency (CPS1D) [MIM:237300]
CPS1 P31327 VAR_063574 p.Ile1215Val US rs141373204 Carbamoyl phosphate synthetase 1 deficiency (CPS1D) [MIM:237300]

CPS1 P31327 VAR_063575 p.Asn1241Lys LP/P - Carbamoyl phosphate synthetase 1 deficiency (CPS1D) [MIM:237300]
CPS1 P31327 VAR_064062 p.Ser123Phe LP/P - Carbamoyl phosphate synthetase 1 deficiency (CPS1D) [MIM:237300]
CPS1 P31327 VAR_064063 p.Thr471Asn LP/P - Carbamoyl phosphate synthetase 1 deficiency (CPS1D) [MIM:237300]
CPS1 P31327 VAR_064064 p.Gln678Pro LP/P - Carbamoyl phosphate synthetase 1 deficiency (CPS1D) [MIM:237300]
CPS1 P31327 VAR_064065 p.Pro774Leu LP/P - Carbamoyl phosphate synthetase 1 deficiency (CPS1D) [MIM:237300]
CPS1 P31327 VAR_064066 p.Pro1411Leu LP/P rs1202306773 Carbamoyl phosphate synthetase 1 deficiency (CPS1D) [MIM:237300]
CPS1 P31327 VAR_064067 p.Arg1453Gln LP/P - Carbamoyl phosphate synthetase 1 deficiency (CPS1D) [MIM:237300]
CPS1 P31327 VAR_064068 p.Arg1453Trp LP/P rs933813349 Carbamoyl phosphate synthetase 1 deficiency (CPS1D) [MIM:237300]
CPS1 P31327 VAR_064069 p.Tyr1491His LP/P rs1553519513 Carbamoyl phosphate synthetase 1 deficiency (CPS1D) [MIM:237300]
CPS1 P31327 VAR_066104 p.Gly301Glu LP/P rs973321068 Carbamoyl phosphate synthetase 1 deficiency (CPS1D) [MIM:237300]
CPS1 P31327 VAR_066105 p.Tyr389Cys LP/P - Carbamoyl phosphate synthetase 1 deficiency (CPS1D) [MIM:237300]
CPS1 P31327 VAR_066106 p.Leu390Arg LP/P - Carbamoyl phosphate synthetase 1 deficiency (CPS1D) [MIM:237300]
CPS1 P31327 VAR_066107 p.Arg718Lys LP/P - Carbamoyl phosphate synthetase 1 deficiency (CPS1D) [MIM:237300]
CPS1 P31327 VAR_066108 p.Arg721Gln LP/P rs752339705 Carbamoyl phosphate synthetase 1 deficiency (CPS1D) [MIM:237300]
CPS1 P31327 VAR_066109 p.Ala724Pro LP/P - Carbamoyl phosphate synthetase 1 deficiency (CPS1D) [MIM:237300]
CPS1 P31327 VAR_066110 p.Ala726Thr LP/P - Carbamoyl phosphate synthetase 1 deficiency (CPS1D) [MIM:237300]
CPS1 P31327 VAR_066111 p.Asp767Val LP/P - Carbamoyl phosphate synthetase 1 deficiency (CPS1D) [MIM:237300]
CPS1 P31327 VAR_066112 p.Met792Ile LP/P rs1553513429 Carbamoyl phosphate synthetase 1 deficiency (CPS1D) [MIM:237300]
CPS1 P31327 VAR_066113 p.Val978Glu LP/P - Carbamoyl phosphate synthetase 1 deficiency (CPS1D) [MIM:237300]
CPS1 P31327 VAR_066114 p.Gly982Val LP/P rs121912595 Carbamoyl phosphate synthetase 1 deficiency (CPS1D) [MIM:237300]
CPS1 P31327 VAR_066115 p.Tyr984His LP/P - Carbamoyl phosphate synthetase 1 deficiency (CPS1D) [MIM:237300]
CPS1 P31327 VAR_066116 p.Ile986Thr LP/P rs1553516442 Carbamoyl phosphate synthetase 1 deficiency (CPS1D) [MIM:237300]
CPS1 P31327 VAR_066117 p.Gly987Cys LP/P rs1553516443 Carbamoyl phosphate synthetase 1 deficiency (CPS1D) [MIM:237300]
CPS1 P31327 VAR_066118 p.Phe992Ser LP/P rs990390709 Carbamoyl phosphate synthetase 1 deficiency (CPS1D) [MIM:237300]

CPS1 P31327 VAR_066119 p.Asn1016Ser LP/P rs749238466 Carbamoyl phosphate synthetase 1 deficiency (CPS1D) [MIM:237300]
CPS1 P31327 VAR_066120 p.Pro1017Leu LP/P - Carbamoyl phosphate synthetase 1 deficiency (CPS1D) [MIM:237300]
CPS1 P31327 VAR_066121 p.Thr1022Ile LP/P rs1437651658 Carbamoyl phosphate synthetase 1 deficiency (CPS1D) [MIM:237300]
CPS1 P31327 VAR_066122 p.Glu1034Gly LP/P - Carbamoyl phosphate synthetase 1 deficiency (CPS1D) [MIM:237300]
CPS1 P31327 VAR_066123 p.His1045Arg LP/P rs1241423400 Carbamoyl phosphate synthetase 1 deficiency (CPS1D) [MIM:237300]
CPS1 P31327 VAR_066124 p.Gln1059Arg LP/P - Carbamoyl phosphate synthetase 1 deficiency (CPS1D) [MIM:237300]
CPS1 P31327 VAR_066125 p.Ala1065Glu LP/P rs770471782 Carbamoyl phosphate synthetase 1 deficiency (CPS1D) [MIM:237300]
CPS1 P31327 VAR_066126 p.Arg1089Cys LP/P rs1392559810 Carbamoyl phosphate synthetase 1 deficiency (CPS1D) [MIM:237300]
CPS1 P31327 VAR_066127 p.Ala1155Glu LP/P - Carbamoyl phosphate synthetase 1 deficiency (CPS1D) [MIM:237300]
CPS1 P31327 VAR_066128 p.Ala1155Val LP/P rs766125631 Carbamoyl phosphate synthetase 1 deficiency (CPS1D) [MIM:237300]
CPS1 P31327 VAR_066129 p.Ser1203Leu LP/P rs149518280 Carbamoyl phosphate synthetase 1 deficiency (CPS1D) [MIM:237300]
CPS1 P31327 VAR_066130 p.Arg1228Gln LP/P rs778117194 Carbamoyl phosphate synthetase 1 deficiency (CPS1D) [MIM:237300]
CPS1 P31327 VAR_066131 p.Glu1255Asp LP/P - Carbamoyl phosphate synthetase 1 deficiency (CPS1D) [MIM:237300]
CPS1 P31327 VAR_066132 p.Arg1262Pro LP/P - Carbamoyl phosphate synthetase 1 deficiency (CPS1D) [MIM:237300]
CPS1 P31327 VAR_066133 p.Arg1262Gln LP/P rs750670270 Carbamoyl phosphate synthetase 1 deficiency (CPS1D) [MIM:237300]
CPS1 P31327 VAR_066134 p.Asp1274His LP/P - Carbamoyl phosphate synthetase 1 deficiency (CPS1D) [MIM:237300]
CPS1 P31327 VAR_066135 p.Cys1327Arg LP/P - Carbamoyl phosphate synthetase 1 deficiency (CPS1D) [MIM:237300]
CPS1 P31327 VAR_066136 p.Gly1333Glu LP/P rs372645328 Carbamoyl phosphate synthetase 1 deficiency (CPS1D) [MIM:237300]
CPS1 P31327 VAR_066137 p.Arg1371Leu LP/P - Carbamoyl phosphate synthetase 1 deficiency (CPS1D) [MIM:237300]
CPS1 P31327 VAR_066138 p.Thr1391Met LP/P rs1392934477 Carbamoyl phosphate synthetase 1 deficiency (CPS1D) [MIM:237300]
CPS1 P31327 VAR_066139 p.Leu1398Val LP/P - Carbamoyl phosphate synthetase 1 deficiency (CPS1D) [MIM:237300]
CPS1 P31327 VAR_066140 p.Pro1439Leu LP/P - Carbamoyl phosphate synthetase 1 deficiency (CPS1D) [MIM:237300]
CPS1 P31327 VAR_066141 p.Pro1462Arg LP/P - Carbamoyl phosphate synthetase 1 deficiency (CPS1D) [MIM:237300]
CPS1 P31327 VAR_066142 p.Ala589Thr LP/P rs777233486 Carbamoyl phosphate synthetase 1 deficiency (CPS1D) [MIM:237300]

CPS1 P31327 VAR_066143 p.Ala640Ser LP/P rs142693704 Carbamoyl phosphate synthetase 1 deficiency (CPS1D) [MIM:237300]
CPS1 P31327 VAR_066144 p.Asn716Lys LP/P rs369061090 Carbamoyl phosphate synthetase 1 deficiency (CPS1D) [MIM:237300]
CPS1 P31327 VAR_066145 p.Arg803Cys LP/P rs201716417 Carbamoyl phosphate synthetase 1 deficiency (CPS1D) [MIM:237300]
CPS1 P31327 VAR_066146 p.Arg803Gly LP/P rs201716417 Carbamoyl phosphate synthetase 1 deficiency (CPS1D) [MIM:237300]
CPS1 P31327 VAR_066147 p.Arg803Ser LP/P rs201716417 Carbamoyl phosphate synthetase 1 deficiency (CPS1D) [MIM:237300]
CPS1 P31327 VAR_066148 p.Phe805Leu LP/P rs1553513861 Carbamoyl phosphate synthetase 1 deficiency (CPS1D) [MIM:237300]
CPS1 P31327 VAR_066149 p.Phe805Ser LP/P - Carbamoyl phosphate synthetase 1 deficiency (CPS1D) [MIM:237300]
CPS1 P31327 VAR_066150 p.Arg814Trp LP/P rs772782772 Carbamoyl phosphate synthetase 1 deficiency (CPS1D) [MIM:237300]
CPS1 P31327 VAR_066151 p.Cys816Arg LP/P rs1553513870 Carbamoyl phosphate synthetase 1 deficiency (CPS1D) [MIM:237300]
CPS1 P31327 VAR_066152 p.Gly911Glu LP/P rs1388955593 Carbamoyl phosphate synthetase 1 deficiency (CPS1D) [MIM:237300]
CPS1 P31327 VAR_066153 p.Gly911Val LP/P - Carbamoyl phosphate synthetase 1 deficiency (CPS1D) [MIM:237300]
CPS1 P31327 VAR_066154 p.Ser913Leu LP/P rs754706559 Carbamoyl phosphate synthetase 1 deficiency (CPS1D) [MIM:237300]
CPS1 P31327 VAR_066155 p.Asp914Gly LP/P - Carbamoyl phosphate synthetase 1 deficiency (CPS1D) [MIM:237300]
CPS1 P31327 VAR_066156 p.Asp914His LP/P rs765484849 Carbamoyl phosphate synthetase 1 deficiency (CPS1D) [MIM:237300]
CPS1 P31327 VAR_066157 p.Arg932Thr LP/P - Carbamoyl phosphate synthetase 1 deficiency (CPS1D) [MIM:237300]
CPS1 P31327 VAR_066158 p.Ala949Thr LP/P rs537170841 Carbamoyl phosphate synthetase 1 deficiency (CPS1D) [MIM:237300]
CPS1 P31327 VAR_066159 p.Leu958Pro LP/P - Carbamoyl phosphate synthetase 1 deficiency (CPS1D) [MIM:237300]
CPS1 P31327 VAR_066160 p.Tyr959Cys LP/P rs1191587211 Carbamoyl phosphate synthetase 1 deficiency (CPS1D) [MIM:237300]
CPS1 P31327 VAR_066161 p.Tyr962Cys LP/P rs955666400 Carbamoyl phosphate synthetase 1 deficiency (CPS1D) [MIM:237300]
CPS1 P31327 VAR_066162 p.Gly982Ser LP/P rs757059355 Carbamoyl phosphate synthetase 1 deficiency (CPS1D) [MIM:237300]
CPS1 P31327 VAR_066163 p.Ser998Phe LP/P rs1404696893 Carbamoyl phosphate synthetase 1 deficiency (CPS1D) [MIM:237300]
CPS1 P31327 VAR_066164 p.Ile1054Arg LP/P - Carbamoyl phosphate synthetase 1 deficiency (CPS1D) [MIM:237300]
CPS1 P31327 VAR_066165 p.Arg1089Leu LP/P rs1280211937 Carbamoyl phosphate synthetase 1 deficiency (CPS1D) [MIM:237300]
CPS1 P31327 VAR_066166 p.Ser1203Pro LP/P rs1319489001 Carbamoyl phosphate synthetase 1 deficiency (CPS1D) [MIM:237300]

CPS1 P31327 VAR_066167 p.Asp1205Asn LP/P - Carbamoyl phosphate synthetase 1 deficiency (CPS1D) [MIM:237300]
CPS1 P31327 VAR_066168 p.Ser1331Pro LP/P - Carbamoyl phosphate synthetase 1 deficiency (CPS1D) [MIM:237300]
CPS1 P31327 VAR_066169 p.Ala1378Thr LP/P rs1245373037 Carbamoyl phosphate synthetase 1 deficiency (CPS1D) [MIM:237300]
CPS1 P31327 VAR_066170 p.Thr1443Ala LP/P - Carbamoyl phosphate synthetase 1 deficiency (CPS1D) [MIM:237300]
CPS1 P31327 VAR_066171 p.Ala43Val LP/P - Carbamoyl phosphate synthetase 1 deficiency (CPS1D) [MIM:237300]
CPS1 P31327 VAR_066172 p.Gly58Asp LP/P - Carbamoyl phosphate synthetase 1 deficiency (CPS1D) [MIM:237300]
CPS1 P31327 VAR_066173 p.Ser65Phe LP/P rs375979196 Carbamoyl phosphate synthetase 1 deficiency (CPS1D) [MIM:237300]
CPS1 P31327 VAR_066174 p.Val71Gly LP/P - Carbamoyl phosphate synthetase 1 deficiency (CPS1D) [MIM:237300]
CPS1 P31327 VAR_066175 p.Pro87Ser LP/P rs1553509297 Carbamoyl phosphate synthetase 1 deficiency (CPS1D) [MIM:237300]
CPS1 P31327 VAR_066176 p.Tyr89Asp LP/P - Carbamoyl phosphate synthetase 1 deficiency (CPS1D) [MIM:237300]
CPS1 P31327 VAR_066177 p.Asp165Gly LP/P - Carbamoyl phosphate synthetase 1 deficiency (CPS1D) [MIM:237300]
CPS1 P31327 VAR_066178 p.Asp224Val LP/P - Carbamoyl phosphate synthetase 1 deficiency (CPS1D) [MIM:237300]
CPS1 P31327 VAR_066179 p.Arg233Cys LP/P rs767905306 Carbamoyl phosphate synthetase 1 deficiency (CPS1D) [MIM:237300]
CPS1 P31327 VAR_066180 p.His243Pro LP/P rs752902711 Carbamoyl phosphate synthetase 1 deficiency (CPS1D) [MIM:237300]
CPS1 P31327 VAR_066181 p.Gly258Glu LP/P - Carbamoyl phosphate synthetase 1 deficiency (CPS1D) [MIM:237300]
CPS1 P31327 VAR_066182 p.Gly263Glu LP/P rs1471393474 Carbamoyl phosphate synthetase 1 deficiency (CPS1D) [MIM:237300]
CPS1 P31327 VAR_066183 p.Ala304Val LP/P rs775920437 Carbamoyl phosphate synthetase 1 deficiency (CPS1D) [MIM:237300]
CPS1 P31327 VAR_066184 p.Gly317Glu LP/P rs1273594946 Carbamoyl phosphate synthetase 1 deficiency (CPS1D) [MIM:237300]
CPS1 P31327 VAR_066185 p.Asp358His LP/P rs149930500 Carbamoyl phosphate synthetase 1 deficiency (CPS1D) [MIM:237300]
CPS1 P31327 VAR_066186 p.Pro382Leu LP/P rs201407486 Carbamoyl phosphate synthetase 1 deficiency (CPS1D) [MIM:237300]
CPS1 P31327 VAR_066187 p.Gly401Arg US rs760895692 Carbamoyl phosphate synthetase 1 deficiency (CPS1D) [MIM:237300]
CPS1 P31327 VAR_066188 p.Gly431Arg LP/P rs778766382 Carbamoyl phosphate synthetase 1 deficiency (CPS1D) [MIM:237300]
CPS1 P31327 VAR_066189 p.Gly432Val LP/P - Carbamoyl phosphate synthetase 1 deficiency (CPS1D) [MIM:237300]
CPS1 P31327 VAR_066190 p.Ala438Thr LP/P rs772497399 Carbamoyl phosphate synthetase 1 deficiency (CPS1D) [MIM:237300]

CPS1 P31327 VAR_066191 p.Lys450Glu LP/P - Carbamoyl phosphate synthetase 1 deficiency (CPS1D) [MIM:237300]
CPS1 P31327 VAR_066192 p.Ala498Pro LP/P - Carbamoyl phosphate synthetase 1 deficiency (CPS1D) [MIM:237300]
CPS1 P31327 VAR_066193 p.Val531Glu LP/P - Carbamoyl phosphate synthetase 1 deficiency (CPS1D) [MIM:237300]
CPS1 P31327 VAR_066194 p.Val531Gly LP/P - Carbamoyl phosphate synthetase 1 deficiency (CPS1D) [MIM:237300]
CPS1 P31327 VAR_066195 p.Arg587Cys LP/P rs1242028775 Carbamoyl phosphate synthetase 1 deficiency (CPS1D) [MIM:237300]
CPS1 P31327 VAR_066196 p.Arg587Leu LP/P - Carbamoyl phosphate synthetase 1 deficiency (CPS1D) [MIM:237300]
CPS1 P31327 VAR_066197 p.Ser597Leu LP/P - Carbamoyl phosphate synthetase 1 deficiency (CPS1D) [MIM:237300]
CPS1 P31327 VAR_066198 p.Val622Met LP/P rs1553512962 Carbamoyl phosphate synthetase 1 deficiency (CPS1D) [MIM:237300]
CPS1 P31327 VAR_066199 p.Gly628Asp LP/P rs1275599086 Carbamoyl phosphate synthetase 1 deficiency (CPS1D) [MIM:237300]
CPS1 P31327 VAR_066200 p.Ile632Arg LP/P rs1553512974 Carbamoyl phosphate synthetase 1 deficiency (CPS1D) [MIM:237300]
CPS1 P31327 VAR_066201 p.Arg638Pro LP/P rs757205958 Carbamoyl phosphate synthetase 1 deficiency (CPS1D) [MIM:237300]
CPS1 P31327 VAR_066202 p.Cys648Tyr LP/P - Carbamoyl phosphate synthetase 1 deficiency (CPS1D) [MIM:237300]
CPS1 P31327 VAR_066203 p.Asp654Val LP/P - Carbamoyl phosphate synthetase 1 deficiency (CPS1D) [MIM:237300]
CPS1 P31327 VAR_066204 p.Asn674Lys LP/P rs1248368809 Carbamoyl phosphate synthetase 1 deficiency (CPS1D) [MIM:237300]
CPS1 P31327 VAR_066205 p.Asn698Ser LP/P - Carbamoyl phosphate synthetase 1 deficiency (CPS1D) [MIM:237300]
CPS1 P31327 VAR_070211 p.Gly530Val US rs1250316045 -
CPS1 P31327 VAR_075404 p.Ser123Tyr US - Carbamoyl phosphate synthetase 1 deficiency (CPS1D) [MIM:237300]
CPS1 P31327 VAR_075405 p.Arg174Trp US rs1553509661 Carbamoyl phosphate synthetase 1 deficiency (CPS1D) [MIM:237300]
CPS1 P31327 VAR_075406 p.Asn355Asp LP/P rs1472190012 Carbamoyl phosphate synthetase 1 deficiency (CPS1D) [MIM:237300]
CPS1 P31327 VAR_075407 p.Thr871Pro LP/P - Carbamoyl phosphate synthetase 1 deficiency (CPS1D) [MIM:237300]
CPS1 P31327 VAR_075408 p.Ile937Asn LP/P rs760714614 Carbamoyl phosphate synthetase 1 deficiency (CPS1D) [MIM:237300]
CPS1 P31327 VAR_075409 p.Gly964Asp LP/P rs534815243 Carbamoyl phosphate synthetase 1 deficiency (CPS1D) [MIM:237300]
CPS1 P31327 VAR_075410 p.Glu1194Asp LP/P - Carbamoyl phosphate synthetase 1 deficiency (CPS1D) [MIM:237300]
CPS1 P31327 VAR_075411 p.Ile1254Phe US - Carbamoyl phosphate synthetase 1 deficiency (CPS1D) [MIM:237300]
CPS1 P31327 VAR_075413 p.Leu1381Ser LP/P - Carbamoyl phosphate synthetase 1 deficiency (CPS1D) [MIM:237300]

CPS1 P31327 VAR_075806 p.Leu341Ser LP/P - Carbamoyl phosphate synthetase 1 deficiency (CPS1D) [MIM:237300]
CPS1 P31327 VAR_075807 p.Gly661Arg LP/P - Carbamoyl phosphate synthetase 1 deficiency (CPS1D) [MIM:237300]
CPS1 P31327 VAR_075808 p.Thr1167Arg LP/P - Carbamoyl phosphate synthetase 1 deficiency (CPS1D) [MIM:237300]
CPSF1 Q10570 VAR_083937 p.Asp1275Tyr US - Myopia 27 (MYP27) [MIM:618827]
CPSF3 Q9UKF6 VAR_035873 p.Asp578Asn US - A breast cancer sample
CPSF3 Q9UKF6 VAR_037646 p.Glu142Gly LB/B rs17850770 -
CPT1A P50416 VAR_020546 p.Arg123Cys LP/P rs80356775 Carnitine palmitoyltransferase 1A deficiency (CPT1AD) [MIM:255120]
CPT1A P50416 VAR_020547 p.Ala275Thr LB/B rs2229738 -
CPT1A P50416 VAR_020548 p.Cys304Trp LP/P rs80356789 Carnitine palmitoyltransferase 1A deficiency (CPT1AD) [MIM:255120]
CPT1A P50416 VAR_020549 p.Thr314Ile LP/P rs80356776 Carnitine palmitoyltransferase 1A deficiency (CPT1AD) [MIM:255120]
CPT1A P50416 VAR_020550 p.Arg357Trp LP/P rs80356777 Carnitine palmitoyltransferase 1A deficiency (CPT1AD) [MIM:255120]
CPT1A P50416 VAR_020551 p.Glu360Gly LP/P rs80356787 Carnitine palmitoyltransferase 1A deficiency (CPT1AD) [MIM:255120]
CPT1A P50416 VAR_020553 p.Ala414Val LP/P rs80356790 Carnitine palmitoyltransferase 1A deficiency (CPT1AD) [MIM:255120]
CPT1A P50416 VAR_020554 p.Asp454Gly LP/P rs80356778 Carnitine palmitoyltransferase 1A deficiency (CPT1AD) [MIM:255120]
CPT1A P50416 VAR_020555 p.Pro479Leu LP/P rs80356779 Carnitine palmitoyltransferase 1A deficiency (CPT1AD) [MIM:255120]
CPT1A P50416 VAR_020556 p.Leu484Pro LP/P rs80356793 Carnitine palmitoyltransferase 1A deficiency (CPT1AD) [MIM:255120]
CPT1A P50416 VAR_020557 p.Tyr498Cys LP/P rs80356791 Carnitine palmitoyltransferase 1A deficiency (CPT1AD) [MIM:255120]
CPT1A P50416 VAR_020558 p.Gly709Glu LP/P rs28936374 Carnitine palmitoyltransferase 1A deficiency (CPT1AD) [MIM:255120]
CPT1A P50416 VAR_020559 p.Gly710Glu LP/P rs80356780 Carnitine palmitoyltransferase 1A deficiency (CPT1AD) [MIM:255120]
CPT1A P50416 VAR_046767 p.Arg316Gly LP/P rs80356796 Carnitine palmitoyltransferase 1A deficiency (CPT1AD) [MIM:255120]
CPT1A P50416 VAR_046768 p.Phe343Val LP/P rs80356783 Carnitine palmitoyltransferase 1A deficiency (CPT1AD) [MIM:255120]
CPT1A P50416 VAR_046769 p.Gly465Trp LP/P rs80356784 Carnitine palmitoyltransferase 1A deficiency (CPT1AD) [MIM:255120]
CPT1B Q92523 VAR_011739 p.Glu531Lys LB/B rs470117 -
CPT1B Q92523 VAR_011740 p.Ser664Tyr LB/B rs1804702 -
CPT1B Q92523 VAR_020029 p.Ile66Val LB/B rs3213445 -
CPT1B Q92523 VAR_021854 p.Gly320Asp LB/B rs2269383 -
CPT1B Q92523 VAR_024188 p.Ser427Cys LB/B rs8142477 -
CPT1C Q8TCG5 VAR_073433 p.Arg37Cys LP/P rs786204767 Spastic paraplegia 73, autosomal dominant (SPG73) [MIM:616282]
CPT2 P23786 VAR_001391 p.Pro50His LP/P rs28936375 Carnitine palmitoyltransferase 2 deficiency, myopathic, stress-induced (CPT2D) [MIM:255110]

CPT2 P23786 VAR_001392 p.Ser113Leu LP/P rs74315294 Carnitine palmitoyltransferase 2 deficiency, myopathic, stress-induced (CPT2D) [MIM:255110]
CPT2 P23786 VAR_001393 p.Glu174Lys LP/P rs28936674 Carnitine palmitoyltransferase 2 deficiency, myopathic, stress-induced (CPT2D) [MIM:255110]
CPT2 P23786 VAR_001394 p.Phe352Cys LB/B rs2229291 -
CPT2 P23786 VAR_001395 p.Val368Ile LB/B rs1799821 -
CPT2 P23786 VAR_001396 p.Phe383Tyr LP/P rs74315295 Carnitine palmitoyltransferase 2 deficiency, myopathic, stress-induced (CPT2D) [MIM:255110]
CPT2 P23786 VAR_001397 p.Asp553Asn LP/P rs28936376 Carnitine palmitoyltransferase 2 deficiency, myopathic, stress-induced (CPT2D) [MIM:255110]
CPT2 P23786 VAR_001398 p.Tyr628Ser LP/P rs28936673 Carnitine palmitoyltransferase 2 deficiency, infantile (CPT2DI) [MIM:600649]
CPT2 P23786 VAR_001399 p.Arg631Cys LP/P rs74315293 Carnitine palmitoyltransferase 2 deficiency, infantile (CPT2DI) [MIM:600649]
CPT2 P23786 VAR_001399 p.Arg631Cys LP/P rs74315293 Carnitine palmitoyltransferase 2 deficiency, myopathic, stress-induced (CPT2D) [MIM:255110]
CPT2 P23786 VAR_001400 p.Met647Val LB/B rs1799822 -
CPT2 P23786 VAR_007966 p.Met214Thr LP/P rs515726174 Carnitine palmitoyltransferase 2 deficiency, myopathic, stress-induced (CPT2D) [MIM:255110]
CPT2 P23786 VAR_007967 p.Pro227Leu LP/P rs74315298 Carnitine palmitoyltransferase 2 deficiency, myopathic, stress-induced (CPT2D) [MIM:255110]
CPT2 P23786 VAR_007968 p.Phe448Leu LP/P rs74315297 Carnitine palmitoyltransferase 2 deficiency, myopathic, stress-induced (CPT2D) [MIM:255110]
CPT2 P23786 VAR_007969 p.Tyr479Phe LP/P rs749895856 Carnitine palmitoyltransferase 2 deficiency, myopathic, stress-induced (CPT2D) [MIM:255110]
CPT2 P23786 VAR_007970 p.Arg503Cys LP/P rs74315296 Carnitine palmitoyltransferase 2 deficiency, myopathic, stress-induced (CPT2D) [MIM:255110]
CPT2 P23786 VAR_007971 p.Gly549Asp LP/P rs186044004 Carnitine palmitoyltransferase 2 deficiency, myopathic, stress-induced (CPT2D) [MIM:255110]
CPT2 P23786 VAR_011741 p.Ser588Cys LB/B rs1871748 -
CPT2 P23786 VAR_020540 p.Arg151Gln LP/P rs515726177 Carnitine palmitoyltransferase 2 deficiency, myopathic, stress-induced (CPT2D) [MIM:255110]
CPT2 P23786 VAR_020541 p.Tyr210Asp LP/P - Carnitine palmitoyltransferase 2 deficiency, myopathic, stress-induced (CPT2D) [MIM:255110]
CPT2 P23786 VAR_020542 p.Arg296Gln LP/P rs764849762 Carnitine palmitoyltransferase 2 deficiency, myopathic, stress-induced (CPT2D) [MIM:255110]
CPT2 P23786 VAR_020543 p.Gln550Arg LP/P - Carnitine palmitoyltransferase 2 deficiency, myopathic, stress-induced (CPT2D) [MIM:255110]
CPT2 P23786 VAR_020544 p.Gly600Arg LP/P - Carnitine palmitoyltransferase 2 deficiency, myopathic, stress-induced (CPT2D) [MIM:255110]
CPT2 P23786 VAR_020545 p.Pro604Ser LP/P - Carnitine palmitoyltransferase 2 deficiency, myopathic, stress-induced (CPT2D) [MIM:255110]
CPT2 P23786 VAR_037976 p.Asp213Gly LP/P rs74315300 Carnitine palmitoyltransferase 2 deficiency, myopathic, stress-induced (CPT2D) [MIM:255110]
CPT2 P23786 VAR_066567 p.Pro504Leu LB/B rs368311455 -
CPT2 P23786 VAR_066568 p.Val605Leu LB/B rs751557097 -
CPVL Q9H3G5 VAR_022612 p.Ala435Val LB/B rs7313 -
CPVL Q9H3G5 VAR_048681 p.Ser11Leu LB/B rs36074676 -
CPVL Q9H3G5 VAR_048682 p.Arg25His LB/B rs34219043 -

CPVL Q9H3G5 VAR_048683 p.Arg398His LB/B rs1052200 -
CPXCR1 Q8N123 VAR_035218 p.Tyr3Ser LB/B rs5940915 -
CPXCR1 Q8N123 VAR_035219 p.Arg131His LB/B rs5984611 -
CPXM2 Q8N436 VAR_048603 p.Gln750Arg LB/B rs7088479 -
CPZ Q66K79 VAR_027883 p.Leu5Pro LB/B rs2302583 -
CPZ Q66K79 VAR_027884 p.Thr501Met LB/B rs9991535 -
CPZ Q66K79 VAR_047244 p.Pro6Leu LB/B rs34964084 -
CPZ Q66K79 VAR_047245 p.Gln130Leu LB/B rs35993494 -
CPZ Q66K79 VAR_047246 p.Ile486Thr LB/B rs7378066 -
CR1 P17927 VAR_013819 p.His1208Arg LB/B rs2274567 -
CR1 P17927 VAR_013820 p.Thr1408Ile LB/B - -
CR1 P17927 VAR_013821 p.Lys1590Glu LB/B rs17047660 -
CR1 P17927 VAR_013822 p.Arg1601Gly LB/B rs17047661 -
CR1 P17927 VAR_013823 p.Ser1610Thr LB/B rs4844609 -
CR1 P17927 VAR_013824 p.Ile1615Val LB/B rs6691117 -
CR1 P17927 VAR_013825 p.Pro1827Arg LB/B rs3811381 -
CR1 P17927 VAR_013826 p.His1850Asp LB/B - -
CR1 P17927 VAR_020263 p.Thr1408Met LB/B rs3737002 -
CR1 P17927 VAR_055685 p.Asn1540Ser LB/B rs17259045 -
CR1 P17927 VAR_055686 p.Thr1969Ala LB/B rs2296160 -
CR1L Q2VPA4 VAR_038677 p.Arg116Gly LB/B rs2296158 -
CR1L Q2VPA4 VAR_038678 p.Ile139Val LB/B rs3085 -
CR1L Q2VPA4 VAR_038679 p.Asn402Asp LB/B rs12729569 -
CR1L Q2VPA4 VAR_038680 p.Ile455Val LB/B rs6683902 -
CR1L Q2VPA4 VAR_038681 p.Leu491Pro LB/B rs2796257 -
CR2 P20023 VAR_016164 p.Ser639Asn LB/B rs17615 -
CR2 P20023 VAR_016165 p.Ile993Val LB/B rs17618 -
CR2 P20023 VAR_016166 p.Ala1003Glu LB/B rs17617 -
CRACD Q6ZU35 VAR_044205 p.Leu269Ile LB/B rs6823339 -
CRACD Q6ZU35 VAR_044206 p.Arg655Pro LB/B rs7672073 -
CRACD Q6ZU35 VAR_044207 p.Arg710Gln LB/B rs3796546 -
CRACD Q6ZU35 VAR_044208 p.Ser776Leu LB/B rs3796547 -
CRACD Q6ZU35 VAR_079174 p.Glu249Gln LB/B rs1379374919 -
CRACDL Q6NV74 VAR_047335 p.Ser315Cys LB/B rs3731660 -
CRACR2A Q9BSW2 VAR_031483 p.Arg7Gly LB/B rs9788233 -
CRACR2A Q9BSW2 VAR_031484 p.Ala98Thr LB/B rs17836273 -
CRACR2A Q9BSW2 VAR_031485 p.Ala128Val LB/B rs242017 -
CRACR2A Q9BSW2 VAR_031486 p.His136Pro LB/B rs34088152 -
CRACR2A Q9BSW2 VAR_031487 p.Glu154Lys LB/B rs242018 -
CRACR2A Q9BSW2 VAR_031488 p.His212Gln LB/B rs36030417 -
CRACR2B Q8N4Y2 VAR_031479 p.Arg77Gln LB/B rs7126805 -
CRACR2B Q8N4Y2 VAR_031480 p.Lys153Glu LB/B rs28558789 -
CRACR2B Q8N4Y2 VAR_031481 p.Gly173Ser LB/B rs35567200 -
CRACR2B Q8N4Y2 VAR_031482 p.Ser248Ile LB/B rs4075289 -
CRADD P78560 VAR_067536 p.Gly128Arg LP/P rs387906861 Mental retardation, autosomal recessive 34, with variant lissencephaly (MRT34) [MIM:614499]
CRAT P43155 VAR_047780 p.Leu372Met LB/B rs3118635 -
CRAT P43155 VAR_047781 p.Ala624Pro LB/B rs17459086 -

CRAT	P43155	VAR_080636	p.Arg321His	US	rs138665095	Neurodegeneration with brain iron accumulation 8 (NBIA8) [MIM:617917]
CRB1	P82279	VAR_011641	p.Ala161Val	LP/P	rs62635651	Retinitis pigmentosa 12 (RP12) [MIM:600105]
CRB1	P82279	VAR_011642	p.Cys250Trp	LP/P	rs62635652	Retinitis pigmentosa 12 (RP12) [MIM:600105]
CRB1	P82279	VAR_011643	p.Thr745Met	LP/P	rs28939720	Leber congenital amaurosis 8 (LCA8) [MIM:613835]
CRB1	P82279	VAR_011643	p.Thr745Met	LP/P	rs28939720	Retinitis pigmentosa 12 (RP12) [MIM:600105]
CRB1	P82279	VAR_011644	p.Arg764Cys	LP/P	rs62635654	Leber congenital amaurosis 8 (LCA8) [MIM:613835]
CRB1	P82279	VAR_011644	p.Arg764Cys	LP/P	rs62635654	Retinitis pigmentosa 12 (RP12) [MIM:600105]
CRB1	P82279	VAR_011645	p.Cys948Tyr	LP/P	rs62645748	Leber congenital amaurosis 8 (LCA8) [MIM:613835]
CRB1	P82279	VAR_011645	p.Cys948Tyr	LP/P	rs62645748	Retinitis pigmentosa 12 (RP12) [MIM:600105]
CRB1	P82279	VAR_011646	p.Met1041Thr	LP/P	rs62635656	Retinitis pigmentosa 12 (RP12) [MIM:600105]
CRB1	P82279	VAR_011647	p.Leu1071Pro	LP/P	rs62635657	Retinitis pigmentosa 12 (RP12) [MIM:600105]
CRB1	P82279	VAR_011648	p.Ile1100Arg	LP/P	rs62635659	Leber congenital amaurosis 8 (LCA8) [MIM:613835]
CRB1	P82279	VAR_011649	p.Cys1181Arg	LP/P	rs62636291	Retinitis pigmentosa 12 (RP12) [MIM:600105]
CRB1	P82279	VAR_022941	p.Phe144Val	LP/P	rs62636262	Leber congenital amaurosis 8 (LCA8) [MIM:613835]
CRB1	P82279	VAR_022942	p.Val162Met	LP/P	rs137853138	Pigmented paravenous chorioretinal atrophy (PPCRA) [MIM:172870]
CRB1	P82279	VAR_022943	p.Cys195Phe	LP/P	rs764256655	Retinitis pigmentosa 12 (RP12) [MIM:600105]
CRB1	P82279	VAR_022944	p.Ile205Thr	US	rs62645749	Leber congenital amaurosis 8 (LCA8) [MIM:613835]
CRB1	P82279	VAR_022945	p.Thr289Met	LB/B	rs62636263	-
CRB1	P82279	VAR_022946	p.Cys383Tyr	LP/P	rs62645754	Leber congenital amaurosis 8 (LCA8) [MIM:613835]
CRB1	P82279	VAR_022947	p.Tyr433Cys	LP/P	rs62636288	Retinitis pigmentosa 12 (RP12) [MIM:600105]
CRB1	P82279	VAR_022948	p.Cys480Gly	LP/P	-	Leber congenital amaurosis 8 (LCA8) [MIM:613835]
CRB1	P82279	VAR_022949	p.Cys480Arg	LP/P	rs62636264	Leber congenital amaurosis 8 (LCA8) [MIM:613835]
CRB1	P82279	VAR_022950	p.Val578Glu	LP/P	rs1266363944	Retinitis pigmentosa 12 (RP12) [MIM:600105]
CRB1	P82279	VAR_022951	p.Asp584Tyr	LP/P	-	Leber congenital amaurosis 8 (LCA8) [MIM:613835]
CRB1	P82279	VAR_022952	p.Cys587Tyr	LP/P	rs1471328495	Retinitis pigmentosa 12 (RP12) [MIM:600105]
CRB1	P82279	VAR_022953	p.Gln679Glu	LB/B	rs62636286	-
CRB1	P82279	VAR_022954	p.Cys681Tyr	LP/P	rs62636266	Leber congenital amaurosis 8 (LCA8) [MIM:613835]
CRB1	P82279	VAR_022955	p.Glu710Gln	LP/P	rs62645755	Leber congenital amaurosis 8 (LCA8) [MIM:613835]
CRB1	P82279	VAR_022956	p.Met741Thr	LP/P	rs62636267	Leber congenital amaurosis 8 (LCA8) [MIM:613835]
CRB1	P82279	VAR_022958	p.Arg769His	LB/B	rs62636287	-
CRB1	P82279	VAR_022959	p.Arg769Gln	LB/B	-	-
CRB1	P82279	VAR_022960	p.Pro836Thr	LP/P	rs116471343	Retinitis pigmentosa 12 (RP12) [MIM:600105]
CRB1	P82279	VAR_022961	p.Asp837His	LP/P	rs62636289	Retinitis pigmentosa 12 (RP12) [MIM:600105]
CRB1	P82279	VAR_022962	p.Gly846Arg	LP/P	rs539189291	Retinitis pigmentosa 12 (RP12) [MIM:600105]
CRB1	P82279	VAR_022963	p.Gly850Ser	LP/P	rs776591659	Leber congenital amaurosis 8 (LCA8) [MIM:613835]
CRB1	P82279	VAR_022963	p.Gly850Ser	LP/P	rs776591659	Retinitis pigmentosa 12 (RP12) [MIM:600105]
CRB1	P82279	VAR_022964	p.Ile852Thr	LP/P	rs62636271	Leber congenital amaurosis 8 (LCA8) [MIM:613835]
CRB1	P82279	VAR_022965	p.Cys891Gly	LP/P	rs62635658	Retinitis pigmentosa 12 (RP12) [MIM:600105]
CRB1	P82279	VAR_022966	p.Asn894Ser	US	rs62636290	-
CRB1	P82279	VAR_022967	p.Arg905Gln	LB/B	rs114052315	-
CRB1	P82279	VAR_022968	p.Gly959Ser	US	rs557111131	Retinitis pigmentosa 12 (RP12) [MIM:600105]
CRB1	P82279	VAR_022970	p.Asn986Ile	LP/P	-	Retinitis pigmentosa 12 (RP12) [MIM:600105]
CRB1	P82279	VAR_022971	p.Ile989Thr	LP/P	-	Leber congenital amaurosis 8 (LCA8) [MIM:613835]

CRB1	P82279	VAR_022972	p.Ser1025Ile	LP/P	rs62636274	Leber congenital amaurosis 8 (LCA8) [MIM:613835]
CRB1	P82279	VAR_022973	p.Ile1100Thr	LP/P	rs62635659	Retinitis pigmentosa 12 (RP12) [MIM:600105]
CRB1	P82279	VAR_022974	p.Gly1103Arg	LP/P	rs62636275	Leber congenital amaurosis 8 (LCA8) [MIM:613835]
CRB1	P82279	VAR_022974	p.Gly1103Arg	LP/P	rs62636275	Retinitis pigmentosa 12 (RP12) [MIM:600105]
CRB1	P82279	VAR_022975	p.Leu1107Pro	LP/P	rs62636276	Leber congenital amaurosis 8 (LCA8) [MIM:613835]
CRB1	P82279	VAR_022976	p.Leu1107Arg	LP/P	rs62636276	Leber congenital amaurosis 8 (LCA8) [MIM:613835]
CRB1	P82279	VAR_022976	p.Leu1107Arg	LP/P	rs62636276	Retinitis pigmentosa 12 (RP12) [MIM:600105]
CRB1	P82279	VAR_022977	p.Gly1205Arg	LP/P	rs574742644	Leber congenital amaurosis 8 (LCA8) [MIM:613835]
CRB1	P82279	VAR_022978	p.Cys1218Phe	LP/P	-	Leber congenital amaurosis 8 (LCA8) [MIM:613835]
CRB1	P82279	VAR_022979	p.Asn1317His	LP/P	rs62636281	Leber congenital amaurosis 8 (LCA8) [MIM:613835]
CRB1	P82279	VAR_022980	p.Cys1321Ser	LP/P	rs62635649	Leber congenital amaurosis 8 (LCA8) [MIM:613835]
CRB1	P82279	VAR_022981	p.Arg1331His	LB/B	rs62636285	-
CRB1	P82279	VAR_022982	p.Ala1354Thr	LP/P	rs200469148	Retinitis pigmentosa 12 (RP12) [MIM:600105]
CRB1	P82279	VAR_022983	p.Arg1383His	LP/P	rs200573274	Retinitis pigmentosa 12 (RP12) [MIM:600105]
CRB1	P82279	VAR_064180	p.Cys27Phe	LP/P	rs1460946384	Retinitis pigmentosa 12 (RP12) [MIM:600105]
CRB1	P82279	VAR_064181	p.Cys1165Trp	LP/P	-	Retinitis pigmentosa 12 (RP12) [MIM:600105]
CRB1	P82279	VAR_067125	p.Cys45Trp	LP/P	rs145141811	Retinitis pigmentosa 12 (RP12) [MIM:600105]
CRB1	P82279	VAR_067126	p.Cys157Ser	LP/P	-	Retinitis pigmentosa 12 (RP12) [MIM:600105]
CRB1	P82279	VAR_067128	p.Glu222Lys	LB/B	rs114846212	-
CRB1	P82279	VAR_067129	p.Cys310Tyr	LP/P	rs779835125	-
CRB1	P82279	VAR_067130	p.Asn312Lys	LP/P	-	Retinitis pigmentosa 12 (RP12) [MIM:600105]
CRB1	P82279	VAR_067131	p.Gly333Asp	LP/P	rs587783015	Leber congenital amaurosis 8 (LCA8) [MIM:613835]
CRB1	P82279	VAR_067132	p.Cys438Tyr	LP/P	rs1571522690	Leber congenital amaurosis 8 (LCA8) [MIM:613835]
CRB1	P82279	VAR_067133	p.Gly454Arg	LP/P	rs954595597	Leber congenital amaurosis 8 (LCA8) [MIM:613835]
CRB1	P82279	VAR_067134	p.Phe488Ser	LB/B	rs777377174	-
CRB1	P82279	VAR_067135	p.Asp491Val	US	-	-
CRB1	P82279	VAR_067136	p.Leu535Pro	LP/P	rs113082791	Leber congenital amaurosis 8 (LCA8) [MIM:613835]
CRB1	P82279	VAR_067137	p.Asp564Tyr	LP/P	-	Leber congenital amaurosis 8 (LCA8) [MIM:613835]
CRB1	P82279	VAR_067138	p.Ser635Pro	US	-	Leber congenital amaurosis 8 (LCA8) [MIM:613835]
CRB1	P82279	VAR_067139	p.Trp675Cys	LP/P	-	Retinitis pigmentosa 12 (RP12) [MIM:600105]
CRB1	P82279	VAR_067140	p.Glu710Val	LP/P	rs145282040	Retinitis pigmentosa 12 (RP12) [MIM:600105]
CRB1	P82279	VAR_067141	p.Ser740Phe	LP/P	-	Retinitis pigmentosa 12 (RP12) [MIM:600105]
CRB1	P82279	VAR_067142	p.Leu753Pro	LP/P	rs896160584	Leber congenital amaurosis 8 (LCA8) [MIM:613835]
CRB1	P82279	VAR_067144	p.Thr821Met	LB/B	rs142857810	-
CRB1	P82279	VAR_067145	p.Val901Ile	US	-	Retinitis pigmentosa 12 (RP12) [MIM:600105]
CRB1	P82279	VAR_067146	p.Ala937Thr	US	rs114630940	Leber congenital amaurosis 8 (LCA8) [MIM:613835]
CRB1	P82279	VAR_067147	p.Cys939Tyr	LP/P	rs1411345985	Leber congenital amaurosis 8 (LCA8) [MIM:613835]

CRB1 P82279 VAR_067148 p.Ile1003Thr LP/P rs1409740542 Leber congenital amaurosis 8 (LCA8) [MIM:613835]
CRB1 P82279 VAR_067149 p.Leu1012Ser LP/P - Retinitis pigmentosa 12 (RP12) [MIM:600105]
CRB1 P82279 VAR_067150 p.Ser1025Asn LP/P - Retinitis pigmentosa 12 (RP12) [MIM:600105]
CRB1 P82279 VAR_067151 p.Thr1099Lys LP/P - Retinitis pigmentosa 12 (RP12) [MIM:600105]
CRB1 P82279 VAR_067152 p.Tyr1161Cys LP/P rs1414707912 Leber congenital amaurosis 8 (LCA8) [MIM:613835]
CRB1 P82279 VAR_067153 p.Cys1174Gly LP/P rs917768074 Leber congenital amaurosis 8 (LCA8) [MIM:613835]
CRB1 P82279 VAR_067153 p.Cys1174Gly LP/P rs917768074 Retinitis pigmentosa 12 (RP12) [MIM:600105]
CRB1 P82279 VAR_067154 p.Tyr1198Cys LP/P - -
CRB1 P82279 VAR_067155 p.Cys1223Ser LP/P - -
CRB1 P82279 VAR_067156 p.Pro1305Leu LP/P rs1391910861 Retinitis pigmentosa 12 (RP12) [MIM:600105]
CRB1 P82279 VAR_067157 p.Cys1332Phe LP/P rs377543137 Leber congenital amaurosis 8 (LCA8) [MIM:613835]
CRB1 P82279 VAR_067158 p.Ala1365Asp LP/P - -
CRB1 P82279 VAR_067159 p.Pro1381Leu LP/P - Leber congenital amaurosis 8 (LCA8) [MIM:613835]
CRB1 P82279 VAR_068363 p.Lys534Asn LP/P - Retinitis pigmentosa 12 (RP12) [MIM:600105]
CRB1 P82279 VAR_079623 p.Leu479Pro LP/P rs963201816 -
CRB1 P82279 VAR_079624 p.Arg764His US rs375040930 -
CRB1 P82279 VAR_079625 p.Ala921Pro LP/P - -
CRB1 P82279 VAR_079626 p.Cys948Arg LP/P rs62645747 Leber congenital amaurosis 8 (LCA8) [MIM:613835]
CRB1 P82279 VAR_079627 p.Asp1031Asn LP/P - -
CRB2 Q5IJ48 VAR_022984 p.Pro46Leu LB/B rs73571404 -
CRB2 Q5IJ48 VAR_022985 p.Val97Leu LB/B - -
CRB2 Q5IJ48 VAR_022986 p.Pro116Leu LB/B rs542211566 -
CRB2 Q5IJ48 VAR_022987 p.Met145Thr LB/B rs1105223 -
CRB2 Q5IJ48 VAR_022988 p.Gly159Ala LB/B rs1105222 -
CRB2 Q5IJ48 VAR_022989 p.Glu187Asp LB/B - -
CRB2 Q5IJ48 VAR_022990 p.Ala351Thr LB/B rs199679542 -
CRB2 Q5IJ48 VAR_022991 p.Arg534Gln LB/B rs370059953 -
CRB2 Q5IJ48 VAR_022992 p.Arg610Trp LB/B rs145286619 -
CRB2 Q5IJ48 VAR_022993 p.His746Gln LB/B rs757353722 -
CRB2 Q5IJ48 VAR_022994 p.Thr1110Met LB/B rs73571431 -
CRB2 Q5IJ48 VAR_048974 p.Thr90Asn LB/B rs2808415 -
CRB2 Q5IJ48 VAR_061153 p.Val709Ala LB/B rs2488602 -
CRB2 Q5IJ48 VAR_073266 p.Cys620Ser LP/P rs879255250 Focal segmental glomerulosclerosis 9 (FSGS9) [MIM:616220]
CRB2 Q5IJ48 VAR_073267 p.Arg628Cys LP/P rs202128397 Focal segmental glomerulosclerosis 9 (FSGS9) [MIM:616220]
CRB2 Q5IJ48 VAR_073268 p.Cys629Ser LP/P rs879255252 Focal segmental glomerulosclerosis 9 (FSGS9) [MIM:616220]
CRB2 Q5IJ48 VAR_073269 p.Arg633Trp LP/P rs730880377 Ventriculomegaly with cystic kidney disease (VMCKD) [MIM:219730]
CRB2 Q5IJ48 VAR_073270 p.Glu643Ala US rs730880300 Ventriculomegaly with cystic kidney disease (VMCKD) [MIM:219730]
CRB2 Q5IJ48 VAR_073271 p.Asn800Lys US rs765676223 -
CRB2 Q5IJ48 VAR_073272 p.Arg1249Gln LP/P rs147412276 Focal segmental glomerulosclerosis 9 (FSGS9) [MIM:616220]

CRB2 Q5IJ48 VAR_084123 p.Trp498Cys US rs144803819 Focal segmental glomerulosclerosis 9 (FSGS9) [MIM:616220]
CRB2 Q5IJ48 VAR_084124 p.Ala654Pro US rs372093386 -
CRB2 Q5IJ48 VAR_084126 p.Gly839Trp US - Focal segmental glomerulosclerosis 9 (FSGS9) [MIM:616220]
CRB2 Q5IJ48 VAR_084127 p.Thr902Met US rs1463148582 Focal segmental glomerulosclerosis 9 (FSGS9) [MIM:616220]
CRB2 Q5IJ48 VAR_084128 p.Pro1064Ser US rs868484209 Focal segmental glomerulosclerosis 9 (FSGS9) [MIM:616220]
CRB2 Q5IJ48 VAR_084129 p.Asp1076Ala US - Focal segmental glomerulosclerosis 9 (FSGS9) [MIM:616220]
CRB2 Q5IJ48 VAR_084131 p.Arg1115Cys US rs1219047251 Focal segmental glomerulosclerosis 9 (FSGS9) [MIM:616220]
CRB2 Q5IJ48 VAR_084132 p.Cys1129Arg US - Focal segmental glomerulosclerosis 9 (FSGS9) [MIM:616220]
CRB2 Q5IJ48 VAR_084133 p.Asn1184Thr US - Focal segmental glomerulosclerosis 9 (FSGS9) [MIM:616220]
CRB2 Q5IJ48 VAR_084134 p.Arg1249Gly US - Retinitis pigmentosa (RP) [MIM:268000]
CRBN Q96SW2 VAR_079409 p.Cys391Arg LP/P rs797045036 Mental retardation, autosomal recessive 2A (MRT2A) [MIM:607417]
CRCT1 Q9UGL9 VAR_050906 p.Cys96Tyr LB/B rs16834168 -
CREB1 P16220 VAR_068077 p.Asp102Gly LB/B rs387906617 -
CREB3L1 Q96BA8 VAR_032392 p.Ala411Thr LB/B rs35652107 -
CREB3L2 Q70SY1 VAR_062386 p.Val130Ile LB/B rs273957 -
CREB3L4 Q8TEY5 VAR_048444 p.Pro95Ser LB/B rs11264743 -
CREBBP Q92793 VAR_015578 p.Arg1378Pro LP/P rs121434626 Rubinstein-Taybi syndrome 1 (RSTS1) [MIM:180849]
CREBBP Q92793 VAR_027953 p.Val1414Ile LB/B rs130015 -
CREBBP Q92793 VAR_035080 p.Glu1278Lys LP/P rs267606752 Rubinstein-Taybi syndrome 1 (RSTS1) [MIM:180849]
CREBBP Q92793 VAR_035081 p.Thr1447Ile LP/P - Rubinstein-Taybi syndrome 1 (RSTS1) [MIM:180849]
CREBBP Q92793 VAR_035082 p.Tyr1450His LP/P rs1555473499 Rubinstein-Taybi syndrome 1 (RSTS1) [MIM:180849]
CREBBP Q92793 VAR_035083 p.His1470Arg LP/P rs797044860 Rubinstein-Taybi syndrome 1 (RSTS1) [MIM:180849]
CREBBP Q92793 VAR_035084 p.Arg1664His LP/P rs1596791996 Rubinstein-Taybi syndrome 1 (RSTS1) [MIM:180849]
CREBBP Q92793 VAR_037305 p.Tyr1175Cys LP/P rs28937315 Rubinstein-Taybi syndrome 1 (RSTS1) [MIM:180849]
CREBBP Q92793 VAR_072912 p.Gln503His LB/B rs748447855 -
CREBBP Q92793 VAR_072913 p.Pro532Thr LB/B rs902901184 -
CREBBP Q92793 VAR_072914 p.Ile546Asn LB/B - -
CREBBP Q92793 VAR_072915 p.Tyr650Phe LP/P - Rubinstein-Taybi syndrome 1 (RSTS1) [MIM:180849]
CREBBP Q92793 VAR_072916 p.Ala789Thr LP/P rs746728741 Rubinstein-Taybi syndrome 1 (RSTS1) [MIM:180849]
CREBBP Q92793 VAR_072917 p.Thr910Ala LP/P rs143247685 Rubinstein-Taybi syndrome 1 (RSTS1) [MIM:180849]
CREBBP Q92793 VAR_072918 p.Glu1278Ala LP/P - Rubinstein-Taybi syndrome 1 (RSTS1) [MIM:180849]
CREBBP Q92793 VAR_072919 p.Asp1406Tyr LP/P - Rubinstein-Taybi syndrome 1 (RSTS1) [MIM:180849]

CREBBP Q92793 VAR_072920 p.Gln1415Pro LP/P - Rubinstein-Taybi syndrome 1 (RSTS1) [MIM:180849]
CREBBP Q92793 VAR_072921 p.Pro1475Thr LP/P - Rubinstein-Taybi syndrome 1 (RSTS1) [MIM:180849]
CREBBP Q92793 VAR_072922 p.Tyr1503Phe LP/P - Rubinstein-Taybi syndrome 1 (RSTS1) [MIM:180849]
CREBBP Q92793 VAR_072923 p.Leu1507Pro LP/P rs1057520191 Rubinstein-Taybi syndrome 1 (RSTS1) [MIM:180849]
CREBBP Q92793 VAR_072924 p.Asp1543Asn LP/P - Rubinstein-Taybi syndrome 1 (RSTS1) [MIM:180849]
CREBBP Q92793 VAR_078557 p.Cys1710Arg LP/P rs1567265203 Menke-Hennekam syndrome 1 (MKHK1) [MIM:618332]
CREBBP Q92793 VAR_078558 p.Leu1747Arg LP/P - Menke-Hennekam syndrome 1 (MKHK1) [MIM:618332]
CREBBP Q92793 VAR_078559 p.Arg1786Pro LP/P - Menke-Hennekam syndrome 1 (MKHK1) [MIM:618332]
CREBBP Q92793 VAR_078560 p.Cys1819Phe LP/P - Menke-Hennekam syndrome 1 (MKHK1) [MIM:618332]
CREBBP Q92793 VAR_078561 p.Cys1826Trp LP/P - Menke-Hennekam syndrome 1 (MKHK1) [MIM:618332]
CREBBP Q92793 VAR_078562 p.Cys1838Tyr LP/P - Menke-Hennekam syndrome 1 (MKHK1) [MIM:618332]
CREBBP Q92793 VAR_078563 p.Arg1867Gln LP/P rs1131691326 Menke-Hennekam syndrome 1 (MKHK1) [MIM:618332]
CREBBP Q92793 VAR_078564 p.Arg1867Trp LP/P - Menke-Hennekam syndrome 1 (MKHK1) [MIM:618332]
CREBBP Q92793 VAR_078565 p.Arg1868Trp LP/P rs886039491 Menke-Hennekam syndrome 1 (MKHK1) [MIM:618332]
CREBBP Q92793 VAR_078566 p.Met1872Val LP/P rs797045037 Menke-Hennekam syndrome 1 (MKHK1) [MIM:618332]
CREBBP Q92793 VAR_081979 p.His1719Asp LP/P - Menke-Hennekam syndrome 1 (MKHK1) [MIM:618332]
CREBBP Q92793 VAR_081980 p.Glu1724Lys LP/P rs1567265131 Menke-Hennekam syndrome 1 (MKHK1) [MIM:618332]
CREBBP Q92793 VAR_081981 p.Ala1782Val LP/P - Menke-Hennekam syndrome 1 (MKHK1) [MIM:618332]
CREBBP Q92793 VAR_081982 p.His1829Asp LP/P - Menke-Hennekam syndrome 1 (MKHK1) [MIM:618332]
CREBBP Q92793 VAR_081984 p.Arg1868Gln LP/P rs1567263168 Menke-Hennekam syndrome 1 (MKHK1) [MIM:618332]
CREBBP Q92793 VAR_081985 p.Ala1870Pro LP/P - Menke-Hennekam syndrome 1 (MKHK1) [MIM:618332]
CREBRF Q8IUR6 VAR_034795 p.Thr483Ala LB/B rs17854147 -
CREG2 Q8IUH2 VAR_021252 p.Pro96Gln LB/B rs11554173 -
CRELD1 Q96HD1 VAR_023764 p.Arg107His LP/P rs28941780 Atrioventricular septal defect 2 (AVSD2) [MIM:606217]
CRELD1 Q96HD1 VAR_023765 p.Pro162Ala LP/P rs121912626 Atrioventricular septal defect 2 (AVSD2) [MIM:606217]
CRELD1 Q96HD1 VAR_023766 p.Thr311Ile LP/P rs28942092 Atrioventricular septal defect 2 (AVSD2) [MIM:606217]
CRELD1 Q96HD1 VAR_023767 p.Arg329Cys LP/P rs28942091 Atrioventricular septal defect 2 (AVSD2) [MIM:606217]
CRELD1 Q96HD1 VAR_046653 p.Met13Val LB/B rs279552 -

CRELD1 Q96HD1 VAR_046654 p.Pro128Arg LB/B rs2302787 -
CRELD2 Q6UXH1 VAR_028892 p.Asp182Glu LB/B rs8139422 -
CRELD2 Q6UXH1 VAR_028893 p.Ser295Ala LB/B rs11545762 -
CRELD2 Q6UXH1 VAR_028894 p.Glu325Gly LB/B rs11545763 -
CREM Q03060 VAR_055561 p.Gln254Arg LB/B rs1804604 -
CRH P06850 VAR_075504 p.Pro30Arg US rs748404250 -
CRHR2 Q13324 VAR_049455 p.Glu220Asp LB/B rs34625936 -
CRIM1 Q9NZV1 VAR_050907 p.Glu502Lys LB/B rs12997487 -
CRIM1 Q9NZV1 VAR_061625 p.Val781Ile LB/B rs59929305 -
CRIPAK Q8N1N5 VAR_038794 p.Cys89Tyr LB/B rs13097 -
CRIPAK Q8N1N5 VAR_038795 p.His95Pro LB/B rs9328733 -
CRIPAK Q8N1N5 VAR_050908 p.Val120Met LB/B rs141796647 -
CRIPAK Q8N1N5 VAR_061628 p.His95Arg LB/B rs9328733 -
CRIPT Q9P021 VAR_078425 p.Cys3Tyr LP/P rs757078301 Short stature with microcephaly and distinctive facies (SSMCF) [MIM:615789]
CRISP2 P16562 VAR_048832 p.Asn131Ser LB/B rs34457011 -
CRISP3 P54108 VAR_011718 p.Ser106Pro LB/B rs495335 -
CRISP3 P54108 VAR_011719 p.Ala134Ser LB/B rs1864312 -
CRISPLD1 Q9H336 VAR_027255 p.Ala286Ser LB/B rs1945 -
CRISPLD2 Q9H0B8 VAR_027256 p.Ser105Gly LB/B rs12051468 -
CRISPLD2 Q9H0B8 VAR_027257 p.Thr322Ser LB/B rs721005 -
CRISPLD2 Q9H0B8 VAR_074672 p.Asp137Gly LB/B rs1297496215 -
CRLF1 O75462 VAR_017865 p.Arg81His LP/P rs104894670 Crisponi/Cold-induced sweating syndrome 1 (CISS1) [MIM:272430]
CRLF1 O75462 VAR_017866 p.Leu374Arg LP/P rs104894668 Crisponi/Cold-induced sweating syndrome 1 (CISS1) [MIM:272430]
CRLF1 O75462 VAR_028355 p.Arg176Lys LB/B rs11672248 -
CRLF1 O75462 VAR_033113 p.Trp76Gly LP/P rs137853143 Crisponi/Cold-induced sweating syndrome 1 (CISS1) [MIM:272430]
CRLF1 O75462 VAR_070817 p.Leu74Pro LP/P rs1295488778 Crisponi/Cold-induced sweating syndrome 1 (CISS1) [MIM:272430]
CRLF1 O75462 VAR_070818 p.Tyr75Asp LP/P - Crisponi/Cold-induced sweating syndrome 1 (CISS1) [MIM:272430]
CRLF1 O75462 VAR_070819 p.Asn113Ile LP/P - Crisponi/Cold-induced sweating syndrome 1 (CISS1) [MIM:272430]
CRLF1 O75462 VAR_070820 p.Leu114Pro LP/P rs774359694 Crisponi/Cold-induced sweating syndrome 1 (CISS1) [MIM:272430]
CRLF1 O75462 VAR_070821 p.Pro138Leu LP/P rs137853930 Crisponi/Cold-induced sweating syndrome 1 (CISS1) [MIM:272430]
CRLF1 O75462 VAR_070822 p.Ser145Pro LP/P - Crisponi/Cold-induced sweating syndrome 1 (CISS1) [MIM:272430]
CRLF1 O75462 VAR_070823 p.Arg216Cys LP/P rs556029569 Crisponi/Cold-induced sweating syndrome 1 (CISS1) [MIM:272430]
CRLF1 O75462 VAR_070824 p.Phe268Ser LP/P rs761982168 Crisponi/Cold-induced sweating syndrome 1 (CISS1) [MIM:272430]
CRLF1 O75462 VAR_070825 p.Trp284Cys LP/P rs137853927 Crisponi/Cold-induced sweating syndrome 1 (CISS1) [MIM:272430]
CRLF1 O75462 VAR_070826 p.Arg312Pro LP/P rs137853933 Crisponi/Cold-induced sweating syndrome 1 (CISS1) [MIM:272430]

CRLF1 O75462 VAR_070827 p.Arg340Cys LP/P rs771459625 Crisponi/Cold-induced sweating syndrome 1 (CISS1) [MIM:272430]
CRLF3 Q8IUI8 VAR_032539 p.Leu389Pro LB/B rs11867457 -
CRLF3 Q8IUI8 VAR_049180 p.Val202Met LB/B rs3764418 -
CRMP1 Q14194 VAR_037745 p.Val461Ile LB/B rs34611001 -
CRNKL1 Q9BZJ0 VAR_024995 p.Gly35Arg LB/B rs7508949 -
CRNKL1 Q9BZJ0 VAR_049318 p.Phe51Leu LB/B rs2273058 -
CRNKL1 Q9BZJ0 VAR_049319 p.Gln111His LB/B rs2255258 -
CRNKL1 Q9BZJ0 VAR_049320 p.Thr158Ala LB/B rs2255255 -
CRNKL1 Q9BZJ0 VAR_049321 p.Val843Ile LB/B rs35201190 -
CRNN Q9UBG3 VAR_048469 p.Ala27Val LB/B rs35639220 -
CRNN Q9UBG3 VAR_048470 p.Gln374His LB/B rs6695830 -
CRNN Q9UBG3 VAR_048471 p.Gly480Ser LP/P rs3829868 Esophageal cancer (ESCR) [MIM:133239]
CROCC Q5TZA2 VAR_059628 p.Ala439Val LB/B rs4463721 -
CROCC Q5TZA2 VAR_059629 p.Asp586His LB/B rs9435714 -
CROCC Q5TZA2 VAR_059630 p.Arg1097Pro LB/B rs6669627 -
CROCC Q5TZA2 VAR_061626 p.Arg7Gly LB/B rs6586566 -
CROCC Q5TZA2 VAR_061627 p.Arg372Gln LB/B rs57442576 -
CROCC Q5TZA2 VAR_083473 p.Arg1216Trp LB/B rs749003874 -
CROCC Q5TZA2 VAR_083474 p.Arg1866Cys LB/B rs374780265 -
CROT Q9UKG9 VAR_048612 p.Arg94His LB/B rs3827653 -
CROT Q9UKG9 VAR_048613 p.Val474Leu LB/B rs7785206 -
CRPPA A4D126 VAR_068101 p.Ala122Pro LP/P rs387907162 Muscular dystrophy-dystroglycanopathy congenital with brain and eye anomalies A7 (MDDGA7) [MIM:614643]
CRPPA A4D126 VAR_068102 p.Arg126His LP/P rs752817129 Muscular dystrophy-dystroglycanopathy congenital with brain and eye anomalies A7 (MDDGA7) [MIM:614643]
CRPPA A4D126 VAR_068103 p.Ala216Asp LP/P rs387907160 Muscular dystrophy-dystroglycanopathy congenital with brain and eye anomalies A7 (MDDGA7) [MIM:614643]
CRPPA A4D126 VAR_069741 p.Asp156Asn LP/P rs397514547 Muscular dystrophy-dystroglycanopathy congenital with brain and eye anomalies A7 (MDDGA7) [MIM:614643]
CRPPA A4D126 VAR_069742 p.Met213Arg LP/P rs397515408 Muscular dystrophy-dystroglycanopathy congenital with brain and eye anomalies A7 (MDDGA7) [MIM:614643]
CRPPA A4D126 VAR_069743 p.Tyr226His LP/P rs1282788711 Muscular dystrophy-dystroglycanopathy congenital with brain and eye anomalies A7 (MDDGA7) [MIM:614643]
CRPPA A4D126 VAR_069744 p.Thr238Ile LP/P rs397515409 Muscular dystrophy-dystroglycanopathy congenital with brain and eye anomalies A7 (MDDGA7) [MIM:614643]
CRPPA A4D126 VAR_071955 p.Gly54Ala LP/P rs587777797 Muscular dystrophy-dystroglycanopathy limb-girdle C7 (MDDGC7) [MIM:616052]
CRPPA A4D126 VAR_078948 p.Pro149Leu LP/P rs369219851 Muscular dystrophy-dystroglycanopathy limb-girdle C7 (MDDGC7) [MIM:616052]
CRPPA A4D126 VAR_078949 p.Arg205His LP/P rs566179705 Muscular dystrophy-dystroglycanopathy congenital with brain and eye anomalies A7 (MDDGA7) [MIM:614643]
CRPPA A4D126 VAR_078951 p.Tyr226Cys LP/P rs1289931198 Muscular dystrophy-dystroglycanopathy limb-girdle C7 (MDDGC7) [MIM:616052]
CRPPA A4D126 VAR_078970 p.Ala53Thr LP/P - Muscular dystrophy-dystroglycanopathy limb-girdle C7 (MDDGC7) [MIM:616052]
CRTAC1 Q9NQ79 VAR_048972 p.Ala253Thr LB/B rs35853031 -
CRTAC1 Q9NQ79 VAR_048973 p.Val569Met LB/B rs2297935 -
CRTAC1 Q9NQ79 VAR_061152 p.Glu658Lys LB/B rs56007204 -

CRTAM	O95727	VAR_032999	p.Lys321Arg	LB/B	rs2272094	-
CRTAM	O95727	VAR_033000	p.Ala368Gly	LB/B	rs1916036	-
CRTAM	O95727	VAR_049868	p.Glu16Ala	LB/B	rs35411582	-
CRTAM	O95727	VAR_049869	p.Ala78Asp	LB/B	rs34397316	-
CRTAM	O95727	VAR_049870	p.Asp173Gly	LB/B	rs35136295	-
CRTAP	O75718	VAR_032846	p.Glu137Asp	LB/B	rs17850371	-
CRTAP	O75718	VAR_053050	p.Leu261Val	LB/B	rs1135127	-
CRTAP	O75718	VAR_054442	p.Leu67Pro	LP/P	rs72659358	Osteogenesis imperfecta 7 (OI7) [MIM:610682]
CRTAP	O75718	VAR_063599	p.Ala13Glu	LP/P	rs137853938	Osteogenesis imperfecta 7 (OI7) [MIM:610682]
CRTAP	O75718	VAR_063600	p.Lys157Glu	LP/P	rs137853942	Osteogenesis imperfecta 7 (OI7) [MIM:610682]
CRTC1	Q6UUV9	VAR_053934	p.Thr286Ala	LB/B	rs3746266	-
CRTC1	Q6UUV9	VAR_053935	p.Val311Ile	LB/B	rs36070283	-
CRTC1	Q6UUV9	VAR_053936	p.Thr328Ala	LB/B	rs3746266	-
CRTC2	Q53ET0	VAR_038756	p.Met147Val	LB/B	rs11264680	-
CRTC2	Q53ET0	VAR_038757	p.Arg379Cys	LB/B	rs150423770	-
CRTC3	Q6UUV7	VAR_038758	p.Ser72Asn	LB/B	rs8033595	-
CRTC3	Q6UUV7	VAR_038759	p.Leu346Ser	LB/B	-	-
CRX	O43186	VAR_003750	p.Arg41Trp	LP/P	rs104894672	Cone-rod dystrophy 2 (CORD2) [MIM:120970]
CRX	O43186	VAR_003751	p.Glu80Ala	LP/P	rs104894671	Cone-rod dystrophy 2 (CORD2) [MIM:120970]
CRX	O43186	VAR_007946	p.Arg41Gln	LP/P	rs61748436	Retinitis pigmentosa (RP) [MIM:268000]
CRX	O43186	VAR_007948	p.Ala158Thr	LB/B	rs61748445	-
CRX	O43186	VAR_007949	p.Val242Met	LP/P	rs61748459	Cone-rod dystrophy 2 (CORD2) [MIM:120970]
CRX	O43186	VAR_008282	p.Gly122Asp	LB/B	rs61748441	-
CRX	O43186	VAR_008714	p.Arg90Trp	LP/P	rs104894673	Leber congenital amaurosis 7 (LCA7) [MIM:613829]
CRX	O43186	VAR_036438	p.Ser141Phe	US	rs1165723137	A breast cancer sample
CRX	O43186	VAR_063919	p.Lys88Asn	LP/P	-	Leber congenital amaurosis 7 (LCA7) [MIM:613829]
CRX	O43186	VAR_067189	p.Glu42Lys	US	rs863224863	Leber congenital amaurosis 7 (LCA7) [MIM:613829]
CRX	O43186	VAR_067190	p.Thr154Ala	LB/B	rs763651232	-
CRX	O43186	VAR_076956	p.His10Asp	LB/B	rs139340178	-
CRX	O43186	VAR_076957	p.Val66Ile	LB/B	rs61748438	-
CRX	O43186	VAR_076958	p.Arg115Gln	US	rs750727986	Retinitis pigmentosa (RP) [MIM:268000]
CRYAA	P02489	VAR_003819	p.Arg116Cys	LP/P	rs74315439	Cataract 9, multiple types (CTRCT9) [MIM:604219]
CRYAA	P02489	VAR_036564	p.Asp105His	US	-	A breast cancer sample
CRYAA	P02489	VAR_038375	p.Arg49Cys	LP/P	rs74315441	Cataract 9, multiple types (CTRCT9) [MIM:604219]
CRYAA	P02489	VAR_046892	p.Arg21Leu	LP/P	-	Cataract 9, multiple types (CTRCT9) [MIM:604219]
CRYAA	P02489	VAR_046893	p.Arg116His	LP/P	rs121912973	Cataract 9, multiple types (CTRCT9) [MIM:604219]
CRYAA	P02489	VAR_070032	p.Arg12Cys	LP/P	rs397515624	Cataract 9, multiple types (CTRCT9) [MIM:604219]
CRYAB	P02511	VAR_007899	p.Arg120Gly	LP/P	rs104894201	Myopathy, myofibrillar, 2 (MFM2) [MIM:608810]
CRYAB	P02511	VAR_014607	p.Ser41Tyr	LB/B	rs2234703	-
CRYAB	P02511	VAR_014608	p.Pro51Leu	LB/B	rs2234704	-
CRYAB	P02511	VAR_069528	p.Asp109His	LP/P	rs387907339	Myopathy, myofibrillar, 2 (MFM2) [MIM:608810]
CRYAB	P02511	VAR_070035	p.Gly154Ser	LP/P	rs150516929	Cardiomyopathy, dilated 1II (CMD1II) [MIM:615184]
CRYAB	P02511	VAR_070036	p.Arg157His	LP/P	rs141638421	Cardiomyopathy, dilated 1II (CMD1II) [MIM:615184]
CRYAB	P02511	VAR_079841	p.Asp109Gly	LP/P	rs1114167341	-
CRYBA2	P53672	VAR_070029	p.Val50Met	LP/P	-	Cataract 42 (CTRCT42) [MIM:115900]
CRYBA2	P53672	VAR_070208	p.Pro7Ser	LB/B	rs141631259	-

CRYBA4 P53673 VAR_014903 p.Thr84Met LB/B rs4277 -
CRYBA4 P53673 VAR_029528 p.Leu69Pro LP/P rs74315487 Cataract 23, multiple types (CTRCT23)
[MIM:610425]
CRYBA4 P53673 VAR_029529 p.Phe94Ser LP/P rs74315486 Cataract 23, multiple types (CTRCT23)
[MIM:610425]
CRYBA4 P53673 VAR_033824 p.Val36Met LB/B rs35520672 -
CRYBA4 P53673 VAR_078868 p.Gly64Trp LP/P rs1114167427 Cataract 23, multiple types (CTRCT23)
[MIM:610425]
CRYBB1 P53674 VAR_065296 p.Ser129Arg LP/P rs1114167433 -
CRYBB1 P53674 VAR_070030 p.Val96Phe LP/P - Cataract 17, multiple types (CTRCT17) [MIM:611544]
CRYBB2 P43320 VAR_038431 p.Ala65Ser LB/B rs16986560 -
CRYBB3 P26998 VAR_025277 p.Arg105Gln LB/B rs17670506 -
CRYBB3 P26998 VAR_025278 p.His113Asp LB/B rs9608378 -
CRYBB3 P26998 VAR_025279 p.Val159Ile LB/B rs4455261 -
CRYBB3 P26998 VAR_025280 p.Gly165Arg LP/P rs74315490 Cataract 22, multiple types (CTRCT22)
[MIM:609741]
CRYBB3 P26998 VAR_070031 p.Val194Glu LP/P rs587777601 Cataract 22, multiple types (CTRCT22)
[MIM:609741]
CRYBG1 Q9Y4K1 VAR_055687 p.Gly267Asp LB/B rs11968933 -
CRYBG1 Q9Y4K1 VAR_055688 p.Gln293Pro LB/B rs1159148 -
CRYBG1 Q9Y4K1 VAR_055689 p.Cys491Arg LB/B rs3747787 -
CRYBG1 Q9Y4K1 VAR_055690 p.Leu1096Val LB/B rs1350902 -
CRYBG1 Q9Y4K1 VAR_055691 p.Glu1196Ala LB/B rs783396 -
CRYBG1 Q9Y4K1 VAR_055692 p.Cys1395Tyr LB/B rs2297970 -
CRYBG1 Q9Y4K1 VAR_055693 p.Thr1445Ser LB/B rs1676015 -
CRYBG2 Q8N1P7 VAR_048835 p.Ser1294Asn LB/B rs10751735 -
CRYBG3 Q68DQ2 VAR_039906 p.Arg2381His LB/B rs11918990 -
CRYBG3 Q68DQ2 VAR_039907 p.Tyr2709Cys LB/B rs17302349 -
CRYBG3 Q68DQ2 VAR_039908 p.Asn2874His LB/B rs4857302 -
CRYGA P11844 VAR_021139 p.Pro148Leu LB/B rs763049410 -
CRYGB P07316 VAR_021140 p.Ser73Ile LB/B - -
CRYGB P07316 VAR_021141 p.Ile111Leu LB/B rs796287 -
CRYGB P07316 VAR_029517 p.Arg90Thr LB/B rs2241980 -
CRYGC P07315 VAR_021142 p.Thr5Pro LP/P rs104893618 Cataract 2, multiple types (CTRCT2) [MIM:604307]
CRYGC P07315 VAR_021143 p.Arg48His LB/B rs61751949 -
CRYGC P07315 VAR_021144 p.Arg168Trp US rs28931604 Cataract 2, multiple types (CTRCT2) [MIM:604307]
CRYGC P07315 VAR_038432 p.Phe6Leu LB/B rs2242072 -
CRYGC P07315 VAR_067212 p.Gly129Cys LP/P rs137853924 Cataract 2, multiple types (CTRCT2)
[MIM:604307]
CRYGD P07320 VAR_010733 p.Arg15Cys LP/P rs121909595 Cataract 4, multiple types (CTRCT4)
[MIM:115700]
CRYGD P07320 VAR_010734 p.Arg37Ser LP/P rs121909597 Cataract 4, multiple types (CTRCT4) [MIM:115700]
CRYGD P07320 VAR_010735 p.Arg59His LP/P rs121909596 Cataract 4, multiple types (CTRCT4) [MIM:115700]
CRYGD P07320 VAR_021145 p.Pro24Thr LP/P rs28931605 Cataract 4, multiple types (CTRCT4) [MIM:115700]
CRYGD P07320 VAR_021146 p.Met102Val LB/B - -
CRYGD P07320 VAR_034955 p.Pro24Ser LP/P rs28931605 Cataract 4, multiple types (CTRCT4) [MIM:115700]
CRYGD P07320 VAR_034956 p.Glu107Ala LP/P - Cataract 4, multiple types (CTRCT4) [MIM:115700]
CRYGD P07320 VAR_064829 p.Trp43Arg LP/P - Cataract 4, multiple types (CTRCT4) [MIM:115700]

CRYGS P22914 VAR_069797 p.Gly18Val LP/P rs104893736 Cataract 20, multiple types (CTRCT20) [MIM:116100]
CRYM Q14894 VAR_073781 p.Lys314Thr LP/P rs104894512 Deafness, autosomal dominant, 40 (DFNA40) [MIM:616357]
CRYZ Q08257 VAR_022913 p.Pro66Ser LB/B rs11551729 -
CRYZ Q08257 VAR_022914 p.Ile176Val LB/B rs3819946 -
CRYZ Q08257 VAR_048200 p.Glu183Lys LB/B rs17095822 -
CRYZL1 O95825 VAR_027835 p.Ala39Thr LB/B rs13050238 -
CSAG1 Q6PB30 VAR_032868 p.Tyr28Phe LB/B rs1894359 -
CSAG1 Q6PB30 VAR_047336 p.Arg62Lys LB/B rs2515848 -
CSE1L P55060 VAR_029327 p.Ile754Val LB/B rs2229042 -
CSE1L P55060 VAR_036558 p.Cys842Phe US - A colorectal cancer sample
CSE1L P55060 VAR_048836 p.Val968Leu LB/B rs3505 -
CSF1 P09603 VAR_020454 p.Leu408Pro LB/B rs1058885 -
CSF1 P09603 VAR_020455 p.Ser496Phe LB/B rs12721516 -
CSF1 P09603 VAR_022146 p.Ala531Val LB/B rs2229167 -
CSF1 P09603 VAR_029320 p.Gly438Arg LB/B rs2229165 -
CSF1 P09603 VAR_048810 p.Ser341Asn LB/B rs12565736 -
CSF1 P09603 VAR_048811 p.Phe489Ser LB/B rs333971 -
CSF1R P07333 VAR_011953 p.Tyr969Cys LB/B rs1801271 -
CSF1R P07333 VAR_042038 p.Val32Gly LB/B rs56048668 -
CSF1R P07333 VAR_042039 p.His362Arg LB/B rs10079250 -
CSF1R P07333 VAR_042040 p.Gly413Ser LB/B rs34951517 -
CSF1R P07333 VAR_042041 p.Leu536Val LB/B rs55942044 -
CSF1R P07333 VAR_042042 p.Pro693His US - A lung squamous cell carcinoma sample
CSF1R P07333 VAR_042043 p.Glu920Asp LB/B rs34030164 -
CSF1R P07333 VAR_042044 p.Arg921Gln LB/B rs56059682 -
CSF1R P07333 VAR_049718 p.Val279Met LB/B rs3829986 -
CSF1R P07333 VAR_061290 p.Ala245Ser LB/B rs41338945 -
CSF1R P07333 VAR_067397 p.Gly589Glu LP/P rs281860268 Leukoencephalopathy, diffuse hereditary, with spheroids (HDLS) [MIM:221820]
CSF1R P07333 VAR_067398 p.Glu633Lys LP/P rs281860269 Leukoencephalopathy, diffuse hereditary, with spheroids (HDLS) [MIM:221820]
CSF1R P07333 VAR_067399 p.Arg710His LB/B rs201569135 -
CSF1R P07333 VAR_067400 p.Gly747Arg LB/B rs41355444 -
CSF1R P07333 VAR_067401 p.Met766Thr LP/P rs281860270 Leukoencephalopathy, diffuse hereditary, with spheroids (HDLS) [MIM:221820]
CSF1R P07333 VAR_067402 p.Ala770Pro LP/P rs281860271 Leukoencephalopathy, diffuse hereditary, with spheroids (HDLS) [MIM:221820]
CSF1R P07333 VAR_067404 p.Ile775Asn LP/P rs281860273 Leukoencephalopathy, diffuse hereditary, with spheroids (HDLS) [MIM:221820]
CSF1R P07333 VAR_067405 p.Ile794Thr LP/P rs281860274 Leukoencephalopathy, diffuse hereditary, with spheroids (HDLS) [MIM:221820]
CSF1R P07333 VAR_067406 p.Asp837Tyr LP/P rs387906662 Leukoencephalopathy, diffuse hereditary, with spheroids (HDLS) [MIM:221820]
CSF1R P07333 VAR_067407 p.Phe849Ser LP/P rs281860277 Leukoencephalopathy, diffuse hereditary, with spheroids (HDLS) [MIM:221820]
CSF1R P07333 VAR_067409 p.Leu868Pro LP/P rs281860278 Leukoencephalopathy, diffuse hereditary, with spheroids (HDLS) [MIM:221820]

CSF1R P07333 VAR_067410 p.Met875Thr LP/P rs281860279 Leukoencephalopathy, diffuse hereditary, with spheroids (HDLS) [MIM:221820]
CSF1R P07333 VAR_067411 p.Pro878Thr LP/P rs281860280 Leukoencephalopathy, diffuse hereditary, with spheroids (HDLS) [MIM:221820]
CSF1R P07333 VAR_072081 p.Cys653Arg LP/P rs690016559 Leukoencephalopathy, diffuse hereditary, with spheroids (HDLS) [MIM:221820]
CSF1R P07333 VAR_072082 p.Ile843Phe LP/P rs690016558 Leukoencephalopathy, diffuse hereditary, with spheroids (HDLS) [MIM:221820]
CSF1R P07333 VAR_072083 p.Ile906Thr LP/P rs690016560 Leukoencephalopathy, diffuse hereditary, with spheroids (HDLS) [MIM:221820]
CSF1R P07333 VAR_083140 p.Pro132Leu LP/P rs1351319114 Brain abnormalities, neurodegeneration, and dysosteosclerosis (BANDDOS) [MIM:618476]
CSF1R P07333 VAR_083143 p.His643Gln LP/P rs184499252 Brain abnormalities, neurodegeneration, and dysosteosclerosis (BANDDOS) [MIM:618476]
CSF1R P07333 VAR_083144 p.Gly765Asp LP/P rs690016566 Leukoencephalopathy, diffuse hereditary, with spheroids (HDLS) [MIM:221820]
CSF1R P07333 VAR_083145 p.Ala781Glu LP/P rs587777247 Leukoencephalopathy, diffuse hereditary, with spheroids (HDLS) [MIM:221820]
CSF1R P07333 VAR_083146 p.Arg782His LP/P rs281860281 Leukoencephalopathy, diffuse hereditary, with spheroids (HDLS) [MIM:221820]
CSF1R P07333 VAR_083147 p.Pro824Ser LP/P - Leukoencephalopathy, diffuse hereditary, with spheroids (HDLS) [MIM:221820]
CSF2 P04141 VAR_001975 p.Ile117Thr LB/B rs25882 -
CSF2 P04141 VAR_013089 p.Thr115Ile LB/B rs2069640 -
CSF2RA P15509 VAR_058507 p.Gly196Arg LP/P rs137852353 Pulmonary surfactant metabolism dysfunction 4 (SMDP4) [MIM:300770]
CSF2RB P32927 VAR_014801 p.Pro603Thr LB/B rs1801122 -
CSF2RB P32927 VAR_014802 p.Gly647Val LB/B rs1801115 -
CSF2RB P32927 VAR_014803 p.Val652Met LB/B rs1801114 -
CSF2RB P32927 VAR_042521 p.Glu249Gln LB/B rs16845 -
CSF2RB P32927 VAR_042522 p.Pro696Ser LB/B rs16997517 -
CSF3 P09919 VAR_013073 p.Leu157Met LB/B rs2227329 -
CSF3 P09919 VAR_013074 p.Ala174Thr LB/B rs2227330 -
CSF3R Q99062 VAR_014325 p.Met231Thr LB/B rs3917973 -
CSF3R Q99062 VAR_014326 p.Asp320Asn LB/B rs3918018 -
CSF3R Q99062 VAR_014327 p.Gln346Arg LB/B rs3917974 -
CSF3R Q99062 VAR_014328 p.Glu405Lys LB/B rs3918019 -
CSF3R Q99062 VAR_014329 p.Arg440Gln LB/B rs3918020 -
CSF3R Q99062 VAR_014330 p.Asp510His LB/B rs3917991 -
CSF3R Q99062 VAR_014331 p.Tyr562His LB/B rs3917996 -
CSF3R Q99062 VAR_014332 p.Arg583Cys LB/B rs3917997 -
CSF3R Q99062 VAR_062517 p.Pro229His LP/P rs764202764 -
CSF3R Q99062 VAR_063065 p.Thr640Asn LP/P rs121918426 Hereditary neutrophilia (NEUTROPHILIA) [MIM:162830]
CSF3R Q99062 VAR_077011 p.Arg308Cys LP/P rs606231473 Neutropenia, severe congenital 7, autosomal recessive (SCN7) [MIM:617014]
CSGALNACT1Q8TDX6 VAR_055647 p.Val137Ile LB/B rs17128518 -
CSGALNACT1Q8TDX6 VAR_055648 p.Phe473Tyr LB/B rs17128366 -
CSGALNACT1Q8TDX6 VAR_060391 p.Ser193Asn LB/B rs7017776 -

CSGALNACT1Q8TDX6 VAR_078123 p.His234Arg US rs200092345 -
CSGALNACT1Q8TDX6 VAR_078124 p.Pro384Arg LP/P rs746391651 Skeletal dysplasia, mild, with joint laxity and advanced bone age (SDJLABA) [MIM:618870]
CSGALNACT1Q8TDX6 VAR_078125 p.Met509Arg US rs533235539 -
CSGALNACT1Q8TDX6 VAR_084187 p.Asn264Ser LP/P - Skeletal dysplasia, mild, with joint laxity and advanced bone age (SDJLABA) [MIM:618870]
CSGALNACT1Q8TDX6 VAR_084188 p.Asp432Tyr LP/P - Skeletal dysplasia, mild, with joint laxity and advanced bone age (SDJLABA) [MIM:618870]
CSGALNACT2Q8N6G5 VAR_048715 p.Glu215Lys LB/B rs11238456 -
CSGALNACT2Q8N6G5 VAR_048716 p.Pro479Ser LB/B rs2435381 -
CSHL1 Q14406 VAR_059807 p.Asp141Glu LB/B rs2727307 -
CSK P41240 VAR_025203 p.Gly287Asp LB/B rs34866753 -
CSK P41240 VAR_025204 p.Arg398Gln LB/B rs34616395 -
CSK P41240 VAR_025205 p.His442Arg LB/B rs35556162 -
CSK P41240 VAR_041678 p.Pro45Leu LB/B - -
CSKMT A8MUP2 VAR_046283 p.Gly125Ser LB/B rs11231181 -
CSMD1 Q96PZ7 VAR_056846 p.Gly3311Trp LB/B rs34079122 -
CSMD1 Q96PZ7 VAR_059634 p.Met2040Ile LB/B rs6995799 -
CSMD1 Q96PZ7 VAR_059635 p.Gln3369His LB/B rs34337712 -
CSMD1 Q96PZ7 VAR_059636 p.Ser3478Asn LB/B rs11984691 -
CSMD1 Q96PZ7 VAR_076444 p.Pro2260Ala LB/B rs1254099921 -
CSMD1 Q96PZ7 VAR_076445 p.Tyr3539Cys LB/B rs573209145 -
CSMD2 Q7Z408 VAR_024330 p.His538Arg LB/B rs474474 -
CSMD2 Q7Z408 VAR_028081 p.Leu167Pro LB/B rs16836099 -
CSMD2 Q7Z408 VAR_028082 p.Arg2096Lys LB/B rs1874045 -
CSMD2 Q7Z408 VAR_028083 p.Ala2117Val LB/B rs11588581 -
CSMD2 Q7Z408 VAR_028084 p.Met2554Val LB/B rs2641962 -
CSMD2 Q7Z408 VAR_028085 p.Leu2661Arg LB/B rs3820206 -
CSMD2 Q7Z408 VAR_028086 p.Ser3406Phe LB/B rs1617468 -
CSMD2 Q7Z408 VAR_050917 p.Leu2661Val LB/B rs34850622 -
CSMD3 Q7Z407 VAR_017404 p.Ile219Met LB/B rs2219898 -
CSMD3 Q7Z407 VAR_017405 p.Val3000Leu LB/B rs2193430 -
CSMD3 Q7Z407 VAR_017406 p.Asn3621His LB/B rs1592624 -
CSMD3 Q7Z407 VAR_035688 p.Trp322Gly US - A colorectal cancer sample
CSMD3 Q7Z407 VAR_035689 p.Arg3079His US rs780986269 A colorectal cancer sample
CSMD3 Q7Z407 VAR_035690 p.Arg3359Gln US rs139968762 A colorectal cancer sample
CSMD3 Q7Z407 VAR_064703 p.Ser2020Cys US - -
CSN1S1 P47710 VAR_048614 p.Ala117Val LB/B rs10030475 -
CSN3 P07498 VAR_026338 p.Tyr75Cys LB/B rs17850702 -
CSN3 P07498 VAR_026339 p.Arg110Leu LB/B rs1048152 -
CSN3 P07498 VAR_026340 p.Ala145Thr LB/B rs3775739 -
CSNK1A1 P48729 VAR_042073 p.Asp297His US - A breast pleomorphic lobular carcinoma sample
CSNK1A1L Q8N752 VAR_034047 p.Arg170Ser LB/B rs17773251 -
CSNK1A1L Q8N752 VAR_036450 p.Arg21Gln US rs373362769 A colorectal cancer sample
CSNK1A1L Q8N752 VAR_042074 p.Ser5Gly LB/B rs56224973 -
CSNK1A1L Q8N752 VAR_042075 p.Arg21Trp LB/B rs56158728 -
CSNK1A1L Q8N752 VAR_042076 p.Asp42Glu LB/B rs9576175 -
CSNK1A1L Q8N752 VAR_042077 p.Glu177Lys LB/B rs17054882 -
CSNK1A1L Q8N752 VAR_042078 p.Pro220Leu LB/B rs56252856 -

CSNK1A1L Q8N752 VAR_042079 p.Lys230Asn LB/B rs56252523 -
CSNK1A1L Q8N752 VAR_042080 p.Ala257Thr LB/B rs55895045 -
CSNK1D P48730 VAR_029075 p.Thr44Ala LP/P rs104894561 Advanced sleep phase syndrome, familial, 2 (FASPS2) [MIM:615224]
CSNK1D P48730 VAR_036451 p.Ser97Cys US - Breast cancer samples
CSNK1D P48730 VAR_042081 p.Pro401Ala LB/B rs56124628 -
CSNK1D P48730 VAR_069801 p.His46Arg LP/P rs397514693 Advanced sleep phase syndrome, familial, 2 (FASPS2) [MIM:615224]
CSNK1E P49674 VAR_042082 p.Arg256Leu US - A lung adenocarcinoma sample
CSNK1E P49674 VAR_042083 p.His413Arg LB/B rs35665927 -
CSNK1G1 Q9HCP0 VAR_042084 p.Arg206Lys LB/B rs55799101 -
CSNK1G1 Q9HCP0 VAR_042085 p.Val329Ile LB/B rs55699712 -
CSNK1G1 Q9HCP0 VAR_078686 p.Arg230Trp LP/P rs587777544 -
CSNK1G2 P78368 VAR_042086 p.Phe189Leu LB/B rs55702630 -
CSNK1G2 P78368 VAR_042087 p.Glu194Gly LB/B rs55780547 -
CSNK1G2 P78368 VAR_042088 p.Ile196Thr LB/B rs55923222 -
CSNK1G2 P78368 VAR_042089 p.Tyr206Cys LB/B rs56264133 -
CSNK1G2 P78368 VAR_042090 p.Tyr206His LB/B rs56108438 -
CSNK1G2 P78368 VAR_042091 p.Arg207Ser LB/B rs56340103 -
CSNK1G2 P78368 VAR_042092 p.Glu208Gln LB/B rs55818316 -
CSNK1G2 P78368 VAR_042093 p.Arg217Cys LB/B rs55754218 -
CSNK1G2 P78368 VAR_042094 p.Thr223Met LB/B rs56038081 -
CSNK2A1 P68400 VAR_077045 p.Arg47Gln LP/P rs869312845 Okur-Chung neurodevelopmental syndrome (OCNDS) [MIM:617062]
CSNK2A1 P68400 VAR_077046 p.Tyr50Ser LP/P rs869312849 Okur-Chung neurodevelopmental syndrome (OCNDS) [MIM:617062]
CSNK2A1 P68400 VAR_077047 p.Asp175Gly LP/P rs869312848 Okur-Chung neurodevelopmental syndrome (OCNDS) [MIM:617062]
CSNK2A1 P68400 VAR_077048 p.Lys198Arg LP/P rs869312840 Okur-Chung neurodevelopmental syndrome (OCNDS) [MIM:617062]
CSNK2A2 P19784 VAR_040416 p.Glu188Ala LB/B rs55911801 -
CSNK2A3 Q8NEV1 VAR_069225 p.Ile133Thr US rs2071460 A lung carcinoma sample
CSNK2B P67870 VAR_083651 p.Arg86Cys US - Poirier-Bienvenu neurodevelopmental syndrome (POBINDS) [MIM:618732]
CSNK2B P67870 VAR_083652 p.Arg111Pro LP/P - Poirier-Bienvenu neurodevelopmental syndrome (POBINDS) [MIM:618732]
CSNK2B P67870 VAR_083653 p.Cys137Phe LP/P - Poirier-Bienvenu neurodevelopmental syndrome (POBINDS) [MIM:618732]
CSNK2B P67870 VAR_083654 p.Cys137Gly LP/P - Poirier-Bienvenu neurodevelopmental syndrome (POBINDS) [MIM:618732]
CSNK2B P67870 VAR_083655 p.Leu187Arg LP/P - Poirier-Bienvenu neurodevelopmental syndrome (POBINDS) [MIM:618732]
CSPG4 Q6UVK1 VAR_061733 p.Arg1703His LB/B rs8023621 -
CSPG5 O95196 VAR_055089 p.Gly188Val LB/B rs3732530 -
CSPG5 O95196 VAR_055090 p.Thr417Pro LB/B rs34016925 -
CSPP1 Q1MSJ5 VAR_033045 p.Arg907His LB/B rs16933182 -
CSPP1 Q1MSJ5 VAR_047014 p.Trp1135Arg LB/B rs1808140 -
CSRNP1 Q96S65 VAR_055100 p.Val453Ile LB/B rs1274958 -
CSRNP2 Q9H175 VAR_053016 p.Thr436Met LB/B rs11542510 -

CSRNP3 Q8WYN3 VAR_035993 p.Pro474Leu US rs766623860 A colorectal cancer sample
CSRP1 P21291 VAR_050144 p.Lys108Ile LB/B rs3738283 -
CSRP3 P50461 VAR_015401 p.Trp4Arg US rs45550635 Cardiomyopathy, dilated 1M (CMD1M) [MIM:607482]
CSRP3 P50461 VAR_045932 p.Leu44Pro LP/P rs104894205 Cardiomyopathy, familial hypertrophic 12 (CMH12) [MIM:612124]
CSRP3 P50461 VAR_045934 p.Cys58Gly LP/P rs104894204 Cardiomyopathy, familial hypertrophic 12 (CMH12) [MIM:612124]
CSRP3 P50461 VAR_076805 p.Gly72Arg US rs45552933 Cardiomyopathy, dilated 1M (CMD1M) [MIM:607482]
CST1 P01037 VAR_028932 p.Tyr4His LB/B rs6076122 -
CST1 P01037 VAR_028933 p.Pro31Leu LB/B rs2070856 -
CST1 P01037 VAR_028934 p.Asn129Asp LB/B rs3188319 -
CST1 P01037 VAR_028935 p.Arg131Met LB/B rs3188320 -
CST1 P01037 VAR_028936 p.Lys135Asn LB/B rs3188322 -
CST3 P01034 VAR_002207 p.Leu94Gln LP/P rs28939068 Amyloidosis 6 (AMYL6) [MIM:105150]
CST3 P01034 VAR_011893 p.Ala25Thr LP/P rs1064039 Macular degeneration, age-related, 11 (ARMD11) [MIM:611953]
CST4 P01036 VAR_036549 p.Thr77Asn US - A breast cancer sample
CST4 P01036 VAR_048852 p.Asp36Asn LB/B rs3210291 -
CST5 P28325 VAR_002208 p.Cys46Arg LB/B rs1799841 -
CST8 O60676 VAR_014527 p.Ala142Pro LB/B rs1054633 -
CST8 O60676 VAR_061130 p.Ala52Val LB/B rs35190670 -
CST9 Q5W186 VAR_038045 p.Leu48Phe LB/B rs2983640 -
CST9L Q9H4G1 VAR_022079 p.His109Pro LB/B rs2295564 -
CSTA P01040 VAR_048851 p.Thr96Met LB/B rs34173813 -
CSTB P04080 VAR_002206 p.Gly4Arg LP/P rs74315443 Epilepsy, progressive myoclonic 1 (EPM1) [MIM:254800]
CSTL1 Q9H114 VAR_024425 p.Trp88Arg LB/B rs3746736 -
CSTL1 Q9H114 VAR_024426 p.Thr96Met LB/B rs3746737 -
CSTL1 Q9H114 VAR_033841 p.Thr59Ala LB/B rs7361799 -
CSTL1 Q9H114 VAR_033842 p.Tyr62Phe LB/B rs16985357 -
CSTL1 Q9H114 VAR_033843 p.Arg66Lys LB/B rs17757442 -
CT45A3 Q8NHU0 VAR_075898 p.Glu55Ala LB/B rs200277856 -
CTAG2 O75638 VAR_007855 p.Arg6Gln LB/B rs17855367 -
CTAG2 O75638 VAR_007856 p.Glu89Gln LB/B rs17328091 -
CTAG2 O75638 VAR_057512 p.Pro99Ala LB/B rs5987003 -
CTAGE1 Q96RT6 VAR_046956 p.Ile682Val LB/B rs9946136 -
CTBP1 Q13363 VAR_080622 p.Arg342Trp LP/P rs869320802 Hypotonia, ataxia, developmental delay, and tooth enamel defect syndrome (HADDTS) [MIM:617915]
CTBS Q01459 VAR_020160 p.Asp310Tyr LB/B rs3768249 -
CTBS Q01459 VAR_049197 p.Val274Ile LB/B rs15911 -
CTC1 Q2NKJ3 VAR_032282 p.Ile820Val LB/B rs3027238 -
CTC1 Q2NKJ3 VAR_032283 p.Ile1005Val LB/B rs3826543 -
CTC1 Q2NKJ3 VAR_067369 p.Ala227Val LP/P rs199473673 Cerebroretinal microangiopathy with calcifications and cysts 1 (CRMCC1) [MIM:612199]
CTC1 Q2NKJ3 VAR_067370 p.Val259Met LP/P rs387907080 Cerebroretinal microangiopathy with calcifications and cysts 1 (CRMCC1) [MIM:612199]

CTC1 Q2NKJ3 VAR_067371 p.Gly503Arg LP/P rs1320809462 Cerebroretinal microangiopathy with calcifications and cysts 1 (CRMCC1) [MIM:612199]
CTC1 Q2NKJ3 VAR_067372 p.Val665Gly LP/P rs199473676 Cerebroretinal microangiopathy with calcifications and cysts 1 (CRMCC1) [MIM:612199]
CTC1 Q2NKJ3 VAR_067373 p.Arg840Trp LP/P rs373905859 Cerebroretinal microangiopathy with calcifications and cysts 1 (CRMCC1) [MIM:612199]
CTC1 Q2NKJ3 VAR_067374 p.Val871Met LP/P rs369255297 Cerebroretinal microangiopathy with calcifications and cysts 1 (CRMCC1) [MIM:612199]
CTC1 Q2NKJ3 VAR_067375 p.Arg975Gly LP/P rs199473678 Cerebroretinal microangiopathy with calcifications and cysts 1 (CRMCC1) [MIM:612199]
CTC1 Q2NKJ3 VAR_067377 p.Arg987Trp LP/P rs202138550 Cerebroretinal microangiopathy with calcifications and cysts 1 (CRMCC1) [MIM:612199]
CTC1 Q2NKJ3 VAR_067378 p.Leu1142His LP/P rs199473681 Cerebroretinal microangiopathy with calcifications and cysts 1 (CRMCC1) [MIM:612199]
CTCF P49711 VAR_013141 p.Arg339Trp US rs1243010179 A Wilms' tumor
CTCF P49711 VAR_013142 p.Lys344Glu US rs1215280530 A breast tumor
CTCF P49711 VAR_013143 p.His345Arg US - A prostate tumor
CTCF P49711 VAR_013144 p.Arg448Gln US - A Wilms' tumor
CTCF P49711 VAR_070776 p.Arg567Trp LP/P rs879255516 Mental retardation, autosomal dominant 21 (MRD21) [MIM:615502]
CTCF P49711 VAR_079375 p.Arg278Cys US rs1266478000 -
CTCF P49711 VAR_079376 p.Arg342His US - -
CTCF P49711 VAR_079377 p.Lys365Thr US - -
CTCFL Q8NI51 VAR_023213 p.Glu50Gln LB/B rs6070128 -
CTCFL Q8NI51 VAR_023214 p.Thr177Ala LB/B rs6025606 -
CTCFL Q8NI51 VAR_032766 p.Gln525Glu LB/B rs6070122 -
CTCFL Q8NI51 VAR_057374 p.Arg448His LB/B rs6092491 -
CTDNEP1 O95476 VAR_034699 p.Thr12Ala LB/B rs3744399 -
CTDP1 Q9Y5B0 VAR_018264 p.Thr340Met LB/B rs2279103 -
CTDP1 Q9Y5B0 VAR_032763 p.Leu755Ser LB/B rs34967023 -
CTDP1 Q9Y5B0 VAR_060440 p.Ser282Phe LB/B rs4799078 -
CTDP1 Q9Y5B0 VAR_060441 p.Pro519His LB/B rs557503 -
CTDSP1 Q9GZU7 VAR_049054 p.Ala56Thr LB/B rs2227249 -
CTDSPL O15194 VAR_019683 p.Ser121Pro LB/B - -
CTDSPL O15194 VAR_019684 p.Asn127Ser LB/B rs1341429725 -
CTDSPL O15194 VAR_019685 p.Val132Gly LB/B - -
CTDSPL2 Q05D32 VAR_042886 p.Ala244Val LB/B rs871923 -
CTF1 Q16619 VAR_014938 p.Ala92Thr LB/B rs2234933 -
CTH P32929 VAR_015450 p.Thr67Ile LP/P rs28941785 Cystathioninuria (CSTNU) [MIM:219500]
CTH P32929 VAR_015451 p.Gln240Glu LP/P rs28941786 Cystathioninuria (CSTNU) [MIM:219500]
CTH P32929 VAR_015452 p.Ser403Ile LB/B rs1021737 -
CTHRC1 Q96CG8 VAR_066589 p.Gln44Pro LB/B rs387907029 -
CTIF O43310 VAR_020041 p.Pro82Leu LB/B rs2277712 -
CTIF O43310 VAR_035749 p.Val389Leu US rs751006365 A breast cancer sample
CTIF O43310 VAR_035750 p.Met438Ile US - A breast cancer sample
CTLA4 P16410 VAR_013577 p.Thr17Ala LB/B rs231775 -
CTLA4 P16410 VAR_072681 p.Arg70Trp LP/P rs606231422 Autoimmune lymphoproliferative syndrome 5 (ALPS5) [MIM:616100]
CTNNA1 P35221 VAR_022303 p.Ala179Val LB/B rs28363394 -

CTNNA1 P35221 VAR_022304 p.Pro219Ser LB/B rs28363406 -
CTNNA1 P35221 VAR_076586 p.Arg54Cys LP/P rs781520852 Macular dystrophy, patterned, 2 (MDPT2) [MIM:608970]
CTNNA1 P35221 VAR_076587 p.Glu307Lys LP/P rs869320697 Macular dystrophy, patterned, 2 (MDPT2) [MIM:608970]
CTNNA1 P35221 VAR_076588 p.Leu318Ser LP/P rs869320696 Macular dystrophy, patterned, 2 (MDPT2) [MIM:608970]
CTNNA1 P35221 VAR_076589 p.Ile431Met LP/P rs755215402 Macular dystrophy, patterned, 2 (MDPT2) [MIM:608970]
CTNNA3 Q9UI47 VAR_053369 p.Ser596Asn LB/B rs4548513 -
CTNNA3 Q9UI47 VAR_062093 p.Arg535Cys LB/B rs41274090 -
CTNNA3 Q9UI47 VAR_070998 p.Val94Asp LP/P rs587777134 Arrhythmogenic right ventricular dysplasia, familial, 13 (ARVD13) [MIM:615616]
CTNNAL1 Q9UBT7 VAR_020924 p.Asn91Thr LB/B rs28361109 -
CTNNAL1 Q9UBT7 VAR_020925 p.Glu203Lys LB/B rs28361118 -
CTNNAL1 Q9UBT7 VAR_020926 p.Glu527Gln LB/B rs7021366 -
CTNNAL1 Q9UBT7 VAR_020927 p.Ile593Asn LB/B rs28361167 -
CTNNAL1 Q9UBT7 VAR_020928 p.Met716Arg LB/B rs28361182 -
CTNNAL1 Q9UBT7 VAR_033845 p.Thr424Ser LB/B rs16913734 -
CTNNAL1 Q9UBT7 VAR_053370 p.Asp555Glu LB/B rs34922868 -
CTNNB1 P35222 VAR_017612 p.Ser23Arg US rs1413975856 Hepatocellular carcinoma
CTNNB1 P35222 VAR_017614 p.Asp32Ala US rs121913396 Hepatocellular carcinoma
CTNNB1 P35222 VAR_017615 p.Asp32Gly US rs121913396 Hepatocellular carcinoma
CTNNB1 P35222 VAR_017615 p.Asp32Gly LP/P rs121913396 Pilomatrixoma (PTR) [MIM:132600]
CTNNB1 P35222 VAR_017616 p.Asp32Tyr US rs28931588 Hepatocellular carcinoma
CTNNB1 P35222 VAR_017616 p.Asp32Tyr LP/P rs28931588 Pilomatrixoma (PTR) [MIM:132600]
CTNNB1 P35222 VAR_017617 p.Ser33Phe US rs121913400 Hepatocellular carcinoma
CTNNB1 P35222 VAR_017617 p.Ser33Phe LP/P rs121913400 Medulloblastoma (MDB) [MIM:155255]
CTNNB1 P35222 VAR_017617 p.Ser33Phe LP/P rs121913400 Pilomatrixoma (PTR) [MIM:132600]
CTNNB1 P35222 VAR_017618 p.Ser33Leu US - Hepatocellular carcinoma
CTNNB1 P35222 VAR_017619 p.Ser33Tyr LP/P rs121913400 Colorectal cancer (CRC) [MIM:114500]
CTNNB1 P35222 VAR_017619 p.Ser33Tyr LP/P rs121913400 Pilomatrixoma (PTR) [MIM:132600]
CTNNB1 P35222 VAR_017620 p.Gly34Glu LP/P rs28931589 Pilomatrixoma (PTR) [MIM:132600]
CTNNB1 P35222 VAR_017621 p.Gly34Arg US rs121913399 Hepatocellular carcinoma
CTNNB1 P35222 VAR_017622 p.Gly34Val LB/B rs28931589 -
CTNNB1 P35222 VAR_017623 p.Ile35Ser US - Hepatocellular carcinoma
CTNNB1 P35222 VAR_017624 p.Ser37Ala US rs121913228 Hepatocellular carcinoma
CTNNB1 P35222 VAR_017624 p.Ser37Ala LP/P rs121913228 Medulloblastoma (MDB) [MIM:155255]
CTNNB1 P35222 VAR_017625 p.Ser37Cys LP/P rs121913403 Ovarian cancer (OC) [MIM:167000]
CTNNB1 P35222 VAR_017625 p.Ser37Cys LP/P rs121913403 Pilomatrixoma (PTR) [MIM:132600]
CTNNB1 P35222 VAR_017626 p.Ser37Phe LP/P rs121913403 Pilomatrixoma (PTR) [MIM:132600]
CTNNB1 P35222 VAR_017627 p.Ser37Tyr US rs121913403 Hepatocellular carcinoma
CTNNB1 P35222 VAR_017629 p.Thr41Ala US rs121913412 Hepatocellular carcinoma
CTNNB1 P35222 VAR_017630 p.Thr41Ile US rs121913413 Hepatocellular carcinoma
CTNNB1 P35222 VAR_017630 p.Thr41Ile LP/P rs121913413 Ovarian cancer (OC) [MIM:167000]
CTNNB1 P35222 VAR_017630 p.Thr41Ile LP/P rs121913413 Pilomatrixoma (PTR) [MIM:132600]
CTNNB1 P35222 VAR_017631 p.Ser45Phe US rs121913409 Hepatocellular carcinoma
CTNNB1 P35222 VAR_017632 p.Ser45Pro US rs121913407 Hepatocellular carcinoma
CTNNB1 P35222 VAR_018954 p.Met688Val LB/B rs4135384 -

CTNNB1 P35222 VAR_072282 p.Leu388Pro LP/P - Neurodevelopmental disorder with spastic diplegia and visual defects (NEDSDV) [MIM:615075]
CTNNB1 P35222 VAR_079200 p.Arg710Cys US rs748653573 Vitreoretinopathy, exudative 7 (EVR7) [MIM:617572]
CTNNBL1 Q8WYA6 VAR_059638 p.Asn507Asp LB/B rs4811236 -
CTNND1 O60716 VAR_020929 p.Tyr217Cys LB/B rs11570194 -
CTNND1 O60716 VAR_020930 p.Arg464Cys LB/B rs11570199 -
CTNND1 O60716 VAR_020931 p.Arg915Lys LB/B rs11570222 -
CTNND1 O60716 VAR_038255 p.Ser171Phe LB/B rs11229133 -
CTNND2 Q9UQB3 VAR_036162 p.Pro1159Ser US - A colorectal cancer sample
CTNND2 Q9UQB3 VAR_062270 p.Gly275Cys US rs61749844 -
CTNND2 Q9UQB3 VAR_062271 p.Ala482Thr LB/B rs61750706 -
CTNND2 Q9UQB3 VAR_062272 p.Gly810Arg LB/B rs61754599 -
CTNND2 Q9UQB3 VAR_073387 p.Gly34Ser LP/P rs151129181 -
CTNND2 Q9UQB3 VAR_073388 p.Pro189Leu LP/P rs149573037 -
CTNND2 Q9UQB3 VAR_073389 p.Pro224Leu LP/P rs769623284 -
CTNND2 Q9UQB3 VAR_073390 p.Arg330His LB/B rs199506424 -
CTNND2 Q9UQB3 VAR_073391 p.Arg454His LP/P rs750266236 -
CTNND2 Q9UQB3 VAR_073392 p.Asp465Asn LB/B rs200377770 -
CTNND2 Q9UQB3 VAR_073393 p.Gln507Pro LP/P rs142843736 -
CTNND2 Q9UQB3 VAR_073394 p.Arg713Cys LP/P rs768575356 -
CTNND2 Q9UQB3 VAR_073395 p.Thr862Met US rs773517967 -
CTNS O60931 VAR_010285 p.Val42Ile LB/B rs35086888 -
CTNS O60931 VAR_010286 p.Gly169Asp LP/P rs121908126 Cystinosis, nephropathic type (CTNS) [MIM:219800]
CTNS O60931 VAR_010287 p.Lys280Arg LP/P - Cystinosis, late-onset juvenile or adolescent nephropathic type (CTNSJAN) [MIM:219900]
CTNS O60931 VAR_010288 p.Asn323Lys LP/P rs121908128 Cystinosis, late-onset juvenile or adolescent nephropathic type (CTNSJAN) [MIM:219900]
CTNS O60931 VAR_010677 p.Ile133Phe LP/P rs886040970 Cystinosis, nephropathic type (CTNS) [MIM:219800]
CTNS O60931 VAR_010678 p.Ser139Phe LP/P rs267606754 Cystinosis, nephropathic type (CTNS) [MIM:219800]
CTNS O60931 VAR_010680 p.Leu158Pro LP/P rs113994206 Cystinosis, nephropathic type (CTNS) [MIM:219800]
CTNS O60931 VAR_010681 p.Trp182Arg LP/P rs764168489 Cystinosis, nephropathic type (CTNS) [MIM:219800]
CTNS O60931 VAR_010682 p.Gly197Arg LP/P rs113994207 Cystinosis, adult, non-nephropathic type (CTNSANN) [MIM:219750]
CTNS O60931 VAR_010683 p.Asp205Asn LP/P rs113994208 Cystinosis, nephropathic type (CTNS) [MIM:219800]
CTNS O60931 VAR_010690 p.Asp305Gly LP/P rs1263951539 Cystinosis, nephropathic type (CTNS) [MIM:219800]
CTNS O60931 VAR_010691 p.Asp305Tyr LP/P - Cystinosis, nephropathic type (CTNS) [MIM:219800]
CTNS O60931 VAR_010692 p.Gly308Arg LP/P rs746307931 Cystinosis, nephropathic type (CTNS) [MIM:219800]
CTNS O60931 VAR_010694 p.Leu338Pro LP/P - Cystinosis, nephropathic type (CTNS) [MIM:219800]
CTNS O60931 VAR_010695 p.Gly339Arg LP/P rs121908127 Cystinosis, nephropathic type (CTNS) [MIM:219800]

CTNS O60931 VAR_010698 p.Asp346Asn LP/P rs757535731 Cystinosis, nephropathic type (CTNS) [MIM:219800]
CTNS O60931 VAR_012314 p.Lys292Arg US rs1800527 -
CTNS O60931 VAR_012315 p.Ser298Asn LP/P rs1212133760 Cystinosis, nephropathic type (CTNS) [MIM:219800]
CTNS O60931 VAR_037318 p.Gly110Val LP/P rs121908129 Cystinosis, nephropathic type (CTNS) [MIM:219800]
CTNS O60931 VAR_037319 p.Asn177Thr LP/P - Cystinosis, late-onset juvenile or adolescent nephropathic type (CTNSJAN) [MIM:219900]
CTNS O60931 VAR_037320 p.Pro200Leu LP/P - Cystinosis, late-onset juvenile or adolescent nephropathic type (CTNSJAN) [MIM:219900]
CTNS O60931 VAR_037321 p.Gln222Arg LP/P rs1327959008 Cystinosis, nephropathic type (CTNS) [MIM:219800]
CTNS O60931 VAR_037322 p.Asn288Lys LP/P - Cystinosis, nephropathic type (CTNS) [MIM:219800]
CTNS O60931 VAR_060371 p.Thr260Ile LB/B rs161400 -
CTNS O60931 VAR_067490 p.Arg151Gly LP/P rs1555563010 Cystinosis, nephropathic type (CTNS) [MIM:219800]
CTNS O60931 VAR_067491 p.Gly157Asp LP/P - Cystinosis, nephropathic type (CTNS) [MIM:219800]
CTNS O60931 VAR_067492 p.Tyr173Cys LP/P rs1555563446 Cystinosis, nephropathic type (CTNS) [MIM:219800]
CTNS O60931 VAR_067493 p.Asn177Ser LP/P - Cystinosis, nephropathic type (CTNS) [MIM:219800]
CTNS O60931 VAR_067494 p.Met287Ile LP/P rs922106812 Cystinosis, nephropathic type (CTNS) [MIM:219800]
CTNS O60931 VAR_067495 p.Gly308Val LP/P rs908965524 Cystinosis, nephropathic type (CTNS) [MIM:219800]
CTNS O60931 VAR_067496 p.Gly309Asp LP/P - Cystinosis, nephropathic type (CTNS) [MIM:219800]
CTNS O60931 VAR_067497 p.Gly337Arg LP/P - Cystinosis, nephropathic type (CTNS) [MIM:219800]
CTNS O60931 VAR_067498 p.Leu338Arg LP/P - Cystinosis, nephropathic type (CTNS) [MIM:219800]
CTNS O60931 VAR_084186 p.Ser141Phe LP/P - Cystinosis, nephropathic type (CTNS) [MIM:219800]
CTPS1 P17812 VAR_027055 p.Ser571Ile LB/B rs17856308 -
CTRB1 P17538 VAR_014566 p.Thr250Ala LB/B rs4737 -
CTRB1 P17538 VAR_057158 p.Asp222His LB/B rs8061550 -
CTRB2 Q6GPI1 VAR_062766 p.Ala250Thr LB/B rs4737 -
CTRC Q99895 VAR_010928 p.Arg80Trp LB/B rs779643710 -
CTRC Q99895 VAR_043516 p.Asp35His LB/B rs184977421 -
CTRC Q99895 VAR_043517 p.Asp35Asn LB/B rs184977421 -
CTRC Q99895 VAR_043518 p.Arg37Gln LB/B rs145868278 -
CTRC Q99895 VAR_043519 p.Gln48Arg LB/B rs536812916 -
CTRC Q99895 VAR_043520 p.Ala73Thr LP/P rs515726209 Pancreatitis, hereditary (PCTT) [MIM:167800]
CTRC Q99895 VAR_043521 p.Lys172Glu LB/B rs34949635 -
CTRC Q99895 VAR_043522 p.Gly217Arg LP/P rs202058123 Pancreatitis, hereditary (PCTT) [MIM:167800]
CTRC Q99895 VAR_043523 p.Gly217Ser LP/P rs202058123 Pancreatitis, hereditary (PCTT) [MIM:167800]
CTRC Q99895 VAR_043524 p.Gly218Ser LB/B - -
CTRC Q99895 VAR_043525 p.Leu220Arg US - -
CTRC Q99895 VAR_043526 p.Glu225Ala LB/B rs201486613 -
CTRC Q99895 VAR_043527 p.Val235Ile LP/P rs140993290 Pancreatitis, hereditary (PCTT) [MIM:167800]
CTRC Q99895 VAR_043528 p.Pro249Leu LP/P rs142560329 Pancreatitis, hereditary (PCTT) [MIM:167800]
CTRC Q99895 VAR_043529 p.Arg254Trp LP/P rs121909293 Pancreatitis, hereditary (PCTT) [MIM:167800]
CTRC Q99895 VAR_043530 p.Asp260Asn LB/B rs540753875 -

CTRC	Q99895	VAR_070520	p.Gly18Arg	US	rs200576965	-
CTRC	Q99895	VAR_070521	p.Arg29Gln	US	rs772024986	-
CTRC	Q99895	VAR_070522	p.Gly32Val	LP/P	-	Pancreatitis, hereditary (PCTT) [MIM:167800]
CTRC	Q99895	VAR_070523	p.Asp35Tyr	US	-	-
CTRC	Q99895	VAR_070524	p.Gly61Arg	US	rs769482036	-
CTRC	Q99895	VAR_070525	p.Lys151Asn	US	-	-
CTRC	Q99895	VAR_070526	p.Cys155Tyr	LP/P	-	Pancreatitis, hereditary (PCTT) [MIM:167800]
CTRC	Q99895	VAR_070527	p.Arg162His	US	rs775404479	-
CTRC	Q99895	VAR_070528	p.Gln178Arg	LP/P	rs200678111	Pancreatitis, hereditary (PCTT) [MIM:167800]
CTRC	Q99895	VAR_070529	p.Met200Val	US	rs146235499	-
CTRC	Q99895	VAR_070530	p.Ile209Met	LB/B	-	-
CTRC	Q99895	VAR_070531	p.Glu225Lys	US	-	-
CTRC	Q99895	VAR_070532	p.Gly227Ser	LB/B	rs567745213	-
CTRC	Q99895	VAR_070533	p.Ser239Ala	US	-	-
CTRC	Q99895	VAR_070534	p.Ser239Cys	US	-	-
CTRC	Q99895	VAR_070535	p.Arg246Cys	LB/B	rs200412314	-
CTRC	Q99895	VAR_070537	p.Lys247Glu	US	-	-
CTRC	Q99895	VAR_070538	p.Val250Glu	LP/P	-	Pancreatitis, hereditary (PCTT) [MIM:167800]
CTRC	Q99895	VAR_070539	p.Arg254Gln	US	rs755811899	-
CTRC	Q99895	VAR_070540	p.Ala257Thr	US	rs200406696	-
CTRC	Q99895	VAR_070541	p.Asn263Ser	US	rs769975164	-
CTRL	P40313	VAR_021939	p.His173Arg	LB/B	rs1134760	-
CTRL	P40313	VAR_051834	p.Thr150Ile	LB/B	rs11552953	-
CTSA	P10619	VAR_001385	p.Gln49Arg	LP/P	rs137854541	Galactosialidosis (GSL) [MIM:256540]
CTSA	P10619	VAR_001386	p.Trp65Arg	LP/P	rs28934603	Galactosialidosis (GSL) [MIM:256540]
CTSA	P10619	VAR_001387	p.Ser90Leu	LP/P	rs137854542	Galactosialidosis (GSL) [MIM:256540]
CTSA	P10619	VAR_001388	p.Tyr249Asn	LP/P	rs137854544	Galactosialidosis (GSL) [MIM:256540]
CTSA	P10619	VAR_001389	p.Tyr395Cys	LP/P	rs137854543	Galactosialidosis (GSL) [MIM:256540]
CTSA	P10619	VAR_001390	p.Phe440Val	LP/P	rs137854540	Galactosialidosis (GSL) [MIM:256540]
CTSA	P10619	VAR_063018	p.Ser51Tyr	LP/P	rs538562022	Galactosialidosis (GSL) [MIM:256540]
CTSA	P10619	VAR_063019	p.Val132Met	LP/P	rs137854545	Galactosialidosis (GSL) [MIM:256540]
CTSA	P10619	VAR_063020	p.Leu236Pro	LP/P	rs137854546	Galactosialidosis (GSL) [MIM:256540]
CTSA	P10619	VAR_063021	p.Met406Thr	LP/P	rs137854548	Galactosialidosis (GSL) [MIM:256540]
CTSA	P10619	VAR_063022	p.Gly439Ser	LP/P	rs137854547	Galactosialidosis (GSL) [MIM:256540]
CTSA	P10619	VAR_063023	p.Lys453Glu	LP/P	rs137854549	Galactosialidosis (GSL) [MIM:256540]
CTSB	P07858	VAR_006724	p.Leu26Val	LB/B	rs12338	-
CTSB	P07858	VAR_014696	p.Ser235Asn	LB/B	rs17573	-
CTSB	P07858	VAR_051511	p.Ser53Gly	LB/B	rs1803250	-
CTSB	P07858	VAR_051512	p.Pro91Leu	LB/B	rs11548596	-
CTSC	P53634	VAR_009541	p.Val249Phe	LP/P	-	Papillon-Lefevre syndrome (PLS) [MIM:245000]
CTSC	P53634	VAR_009542	p.Gln252Leu	LP/P	rs104894207	Papillon-Lefevre syndrome (PLS) [MIM:245000]
CTSC	P53634	VAR_009543	p.Arg272Pro	LP/P	rs587777534	Papillon-Lefevre syndrome (PLS) [MIM:245000]
CTSC	P53634	VAR_009544	p.Gly301Ser	LP/P	rs104894214	Papillon-Lefevre syndrome (PLS) [MIM:245000]
CTSC	P53634	VAR_009545	p.Arg339Cys	LP/P	rs1044703733	Papillon-Lefevre syndrome (PLS) [MIM:245000]
CTSC	P53634	VAR_009546	p.Tyr347Cys	LP/P	rs104894211	Papillon-Lefevre syndrome (PLS) [MIM:245000]
CTSC	P53634	VAR_009546	p.Tyr347Cys	LP/P	rs104894211	Periodontititis, aggressive, 1 (AP1) [MIM:170650]
CTSC	P53634	VAR_016933	p.Trp39Ser	LP/P	rs104894210	Papillon-Lefevre syndrome (PLS) [MIM:245000]
CTSC	P53634	VAR_016934	p.His127Pro	LP/P	rs104894216	Papillon-Lefevre syndrome (PLS) [MIM:245000]
CTSC	P53634	VAR_016935	p.Gln286Arg	LP/P	rs104894208	Haim-Munk syndrome (HMS) [MIM:245010]

CTSC	P53634	VAR_016935	p.Gln286Arg	LP/P	rs104894208	Papillon-Lefevre syndrome (PLS) [MIM:245000]
CTSC	P53634	VAR_016936	p.Trp429Cys	LP/P	rs104894215	Papillon-Lefevre syndrome (PLS) [MIM:245000]
CTSC	P53634	VAR_016943	p.Ile153Thr	LB/B	rs217086	-
CTSC	P53634	VAR_016944	p.Tyr340Cys	LP/P	-	Papillon-Lefevre syndrome (PLS) [MIM:245000]
CTSC	P53634	VAR_016945	p.Glu401Lys	LB/B	rs200627023	-
CTSC	P53634	VAR_016946	p.Ile453Val	LB/B	rs3888798	-
CTSC	P53634	VAR_019036	p.Val129Glu	LP/P	rs760130711	Papillon-Lefevre syndrome (PLS) [MIM:245000]
CTSC	P53634	VAR_019037	p.Gly139Arg	LP/P	rs749103588	Papillon-Lefevre syndrome (PLS) [MIM:245000]
CTSC	P53634	VAR_019038	p.Asp236Tyr	LP/P	rs764724707	Papillon-Lefevre syndrome (PLS) [MIM:245000]
CTSC	P53634	VAR_019039	p.Arg272His	LP/P	rs587777534	Papillon-Lefevre syndrome (PLS) [MIM:245000]
CTSC	P53634	VAR_019040	p.Cys291Tyr	LP/P	rs748729285	Papillon-Lefevre syndrome (PLS) [MIM:245000]
CTSC	P53634	VAR_019041	p.Gly300Asp	LP/P	-	Papillon-Lefevre syndrome (PLS) [MIM:245000]
CTSC	P53634	VAR_019042	p.Gly300Ser	LP/P	-	Papillon-Lefevre syndrome (PLS) [MIM:245000]
CTSC	P53634	VAR_019043	p.Gly301Val	LP/P	-	Papillon-Lefevre syndrome (PLS) [MIM:245000]
CTSC	P53634	VAR_019044	p.Tyr304Asn	LP/P	-	Papillon-Lefevre syndrome (PLS) [MIM:245000]
CTSC	P53634	VAR_019045	p.Gln312Arg	LP/P	rs1484758757	Papillon-Lefevre syndrome (PLS) [MIM:245000]
CTSC	P53634	VAR_019046	p.Glu319Gly	LP/P	rs1294233227	Papillon-Lefevre syndrome (PLS) [MIM:245000]
CTSC	P53634	VAR_019047	p.Tyr412Cys	LP/P	rs28937571	Periodontititis, aggressive, 1 (AP1) [MIM:170650]
CTSC	P53634	VAR_019048	p.Glu447Gly	LP/P	-	Papillon-Lefevre syndrome (PLS) [MIM:245000]
CTSC	P53634	VAR_027249	p.His405Asn	LP/P	-	Papillon-Lefevre syndrome (PLS) [MIM:245000]
CTSC	P53634	VAR_027250	p.His405Arg	LP/P	rs151269219	Papillon-Lefevre syndrome (PLS) [MIM:245000]
CTSC	P53634	VAR_039686	p.Tyr294His	LP/P	-	Papillon-Lefevre syndrome (PLS) [MIM:245000]
CTSD	P07339	VAR_011621	p.Ala58Val	LB/B	rs17571	-
CTSD	P07339	VAR_029362	p.Phe229Ile	LP/P	rs121912789	Ceroid lipofuscinosis, neuronal, 10 (CLN10) [MIM:610127]
CTSD	P07339	VAR_029363	p.Trp383Cys	LP/P	rs121912790	Ceroid lipofuscinosis, neuronal, 10 (CLN10) [MIM:610127]
CTSD	P07339	VAR_058490	p.Gly282Arg	LB/B	rs147278302	-
CTSE	P14091	VAR_014572	p.Thr324Ile	LB/B	rs6503	-
CTSE	P14091	VAR_061731	p.Ile82Val	LB/B	rs57621203	-
CTSF	Q9UBX1	VAR_051513	p.Gln153Arg	LB/B	rs11550508	-
CTSF	Q9UBX1	VAR_070159	p.Tyr231Cys	LP/P	rs143889283	Ceroid lipofuscinosis, neuronal, 13 (CLN13) [MIM:615362]
CTSF	Q9UBX1	VAR_070160	p.Gln321Arg	LP/P	rs397514731	Ceroid lipofuscinosis, neuronal, 13 (CLN13) [MIM:615362]
CTSF	Q9UBX1	VAR_070161	p.Gly458Ala	LP/P	rs397514732	Ceroid lipofuscinosis, neuronal, 13 (CLN13) [MIM:615362]
CTSF	Q9UBX1	VAR_070162	p.Ser480Leu	LP/P	rs397514733	Ceroid lipofuscinosis, neuronal, 13 (CLN13) [MIM:615362]
CTSG	P08311	VAR_006491	p.Asn125Ser	LB/B	rs45567233	-
CTSH	P09668	VAR_036478	p.Gly126Arg	US	-	A colorectal cancer sample
CTSH	P09668	VAR_057038	p.Gly11Arg	LB/B	rs2289702	-
CTSH	P09668	VAR_057039	p.Ala23Thr	LB/B	rs35001431	-
CTSH	P09668	VAR_060368	p.Cys26Ser	LB/B	rs1036938	-
CTSH	P09668	VAR_067717	p.His179Tyr	LB/B	rs1130856	-
CTSK	P43235	VAR_006725	p.Gly146Arg	LP/P	rs74315302	Pycnodysostosis (PKND) [MIM:265800]
CTSK	P43235	VAR_006726	p.Leu309Pro	LP/P	rs29001685	Pycnodysostosis (PKND) [MIM:265800]
CTSK	P43235	VAR_015738	p.Gly79Glu	LP/P	rs74315305	Pycnodysostosis (PKND) [MIM:265800]
CTSK	P43235	VAR_015739	p.Ala277Val	LP/P	rs74315304	Pycnodysostosis (PKND) [MIM:265800]

CTSK	P43235	VAR_074023	p.Arg122Pro	LP/P	-	Pycnodysostosis (PKND) [MIM:265800]
CTSK	P43235	VAR_074024	p.Tyr283Cys	LP/P	-	Pycnodysostosis (PKND) [MIM:265800]
CTSL3P	Q5NE16	VAR_057040	p.Ser123Gly	LB/B	rs11141967	-
CTSS	P25774	VAR_025385	p.Arg113Trp	LB/B	rs2230061	-
CTSS	P25774	VAR_025386	p.Ser161Thr	LB/B	rs1059604	-
CTSW	P56202	VAR_057041	p.Gln218Arg	LB/B	rs606830	-
CTSW	P56202	VAR_058847	p.Ser139Gly	LB/B	rs604630	-
CTSZ	Q9UBR2	VAR_010254	p.Pro36Ser	LB/B	rs778998634	-
CTSZ	Q9UBR2	VAR_010255	p.Ala129Arg	US	-	-
CTSZ	Q9UBR2	VAR_033719	p.Ala286Thr	LB/B	rs34069356	-
CTTNBP2	Q8WZ74	VAR_025535	p.Leu1213Val	LB/B	rs62617115	-
CTTNBP2	Q8WZ74	VAR_048294	p.Gln1148Lys	LB/B	rs10274022	-
CTTNBP2NL	Q9P2B4	VAR_050925	p.Val296Met	LB/B	rs1175640	-
CTTNBP2NL	Q9P2B4	VAR_050926	p.Ser409Gly	LB/B	rs12137578	-
CTU1	Q7Z7A3	VAR_031402	p.Ala107Val	LB/B	rs17855403	-
CTU2	Q2VPK5	VAR_032595	p.Met253Val	LB/B	rs11549837	-
CTU2	Q2VPK5	VAR_032596	p.Val332Ile	LB/B	rs4782321	-
CTU2	Q2VPK5	VAR_032597	p.Gln416Arg	LB/B	rs8059048	-
CTU2	Q2VPK5	VAR_062244	p.His186Tyr	LB/B	rs2290895	-
CTXN3	Q4LDR2	VAR_031763	p.Glu17Val	LB/B	rs248709	-
CTXN3	Q4LDR2	VAR_053885	p.Met23Ile	LB/B	rs2280170	-
CUBN	O60494	VAR_025284	p.Phe124Ile	LB/B	rs1801220	-
CUBN	O60494	VAR_025285	p.Phe253Ser	LB/B	rs1801222	-
CUBN	O60494	VAR_025286	p.Pro389Thr	LB/B	rs1801224	-
CUBN	O60494	VAR_025287	p.Tyr1032His	LB/B	rs1801227	-
CUBN	O60494	VAR_025288	p.Pro1297Leu	LP/P	rs121434430	Imerslund-Grasbeck syndrome 1 (IGS1) [MIM:261100]
CUBN	O60494	VAR_025289	p.Asn1545Tyr	LB/B	-	-
CUBN	O60494	VAR_025290	p.Pro1559Ser	LB/B	rs1801231	-
CUBN	O60494	VAR_025291	p.Val1769Ile	LB/B	rs74116778	-
CUBN	O60494	VAR_025292	p.Leu2153Phe	LB/B	rs62619939	-
CUBN	O60494	VAR_025293	p.Cys2162Tyr	LB/B	rs1276712	-
CUBN	O60494	VAR_025294	p.Pro2575Arg	LB/B	rs3740168	-
CUBN	O60494	VAR_025295	p.Gly2691Arg	LB/B	rs1801237	-
CUBN	O60494	VAR_025296	p.Ser2717Trp	LB/B	rs2796835	-
CUBN	O60494	VAR_025297	p.Leu2879Ile	LB/B	rs1801238	-
CUBN	O60494	VAR_025298	p.Ile2984Val	LB/B	rs1801239	-
CUBN	O60494	VAR_025299	p.Glu3002Gly	LB/B	rs1801240	-
CUBN	O60494	VAR_025300	p.Thr3422Ile	LB/B	rs1801230	-
CUBN	O60494	VAR_025301	p.Asn3552Lys	LB/B	rs1801232	-
CUBN	O60494	VAR_035829	p.His786Gln	US	rs1228797857	A breast cancer sample
CUBN	O60494	VAR_035830	p.Ala2252Val	US	rs529856485	A colorectal cancer sample
CUBN	O60494	VAR_035831	p.Ala2914Val	US	rs45551835	A breast cancer sample
CUBN	O60494	VAR_035832	p.Ile3189Val	US	rs111265129	A breast cancer sample
CUBN	O60494	VAR_047443	p.Gly66Arg	LB/B	rs12259370	-
CUBN	O60494	VAR_047444	p.Ile504Met	LB/B	rs2228053	-
CUBN	O60494	VAR_047445	p.His730Tyr	LB/B	rs7905349	-
CUBN	O60494	VAR_047446	p.Leu969Val	LB/B	rs11254354	-
CUBN	O60494	VAR_047447	p.Arg1775Trp	LB/B	rs1276708	-

CUBN O60494 VAR_047448 p.Gly1840Ser LB/B rs2271462 -
CUBN O60494 VAR_047449 p.Ser1935Gly LB/B rs41289305 -
CUBN O60494 VAR_047450 p.Pro1971Thr LB/B rs2356590 -
CUBN O60494 VAR_047451 p.Phe2263Cys LB/B rs2271460 -
CUBN O60494 VAR_047452 p.Arg2444Gln LB/B rs11254274 -
CUBN O60494 VAR_047453 p.Glu2968Gln LB/B rs45569534 -
CUBN O60494 VAR_055714 p.Thr3432Ser LB/B rs7898873 -
CUBN O60494 VAR_061154 p.Ala335Thr LB/B rs57335729 -
CUBN O60494 VAR_064704 p.Ser3258Gly US - -
CUBN O60494 VAR_084400 p.Thr55Met US - Proteinuria, chronic benign (PROCHOB) [MIM:618884]
CUBN O60494 VAR_084401 p.Pro337Leu LP/P - Imerslund-Grasbeck syndrome 1 (IGS1) [MIM:261100]
CUBN O60494 VAR_084402 p.Gly1112Glu US - Imerslund-Grasbeck syndrome 1 (IGS1) [MIM:261100]
CUBN O60494 VAR_084404 p.Asn1303His US - Proteinuria, chronic benign (PROCHOB) [MIM:618884]
CUBN O60494 VAR_084406 p.Ala1690Val US - -
CUBN O60494 VAR_084408 p.Asp1854Tyr US - Proteinuria, chronic benign (PROCHOB) [MIM:618884]
CUBN O60494 VAR_084409 p.Gly1928Val US - Proteinuria, chronic benign (PROCHOB) [MIM:618884]
CUBN O60494 VAR_084410 p.Ser1947Tyr US - Proteinuria, chronic benign (PROCHOB) [MIM:618884]
CUBN O60494 VAR_084412 p.Asn2157Asp US - -
CUBN O60494 VAR_084413 p.Leu2261Arg US - Proteinuria, chronic benign (PROCHOB) [MIM:618884]
CUBN O60494 VAR_084414 p.Cys2599Trp US - Proteinuria, chronic benign (PROCHOB) [MIM:618884]
CUBN O60494 VAR_084415 p.Pro2822Leu US - Proteinuria, chronic benign (PROCHOB) [MIM:618884]
CUBN O60494 VAR_084419 p.Tyr3018Ser US - Proteinuria, chronic benign (PROCHOB) [MIM:618884]
CUBN O60494 VAR_084420 p.Gly3027Arg US - Proteinuria, chronic benign (PROCHOB) [MIM:618884]
CUBN O60494 VAR_084421 p.Trp3308Arg US - Proteinuria, chronic benign (PROCHOB) [MIM:618884]
CUBN O60494 VAR_084423 p.Ser3366Cys US - Proteinuria, chronic benign (PROCHOB) [MIM:618884]
CUBN O60494 VAR_084424 p.Asp3492Tyr US - Proteinuria, chronic benign (PROCHOB) [MIM:618884]
CUBN O60494 VAR_084425 p.Gly3520Asp US - Proteinuria, chronic benign (PROCHOB) [MIM:618884]
CUBN O60494 VAR_084426 p.Asp3609His US - Proteinuria, chronic benign (PROCHOB) [MIM:618884]
CUEDC1 Q9NWM3 VAR_021951 p.Pro205Ser LB/B rs2304942 -
CUEDC1 Q9NWM3 VAR_033765 p.Arg316Gln LB/B rs34800498 -
CUEDC1 Q9NWM3 VAR_050933 p.Arg169His LB/B rs17762338 -
CUL2 Q13617 VAR_011374 p.Asn109Ser LB/B - -
CUL3 Q13618 VAR_017194 p.Asp13His LB/B rs2969802 -
CUL3 Q13618 VAR_017195 p.Val567Ile LB/B rs3738952 -
CUL3 Q13618 VAR_048839 p.Arg184Ser LB/B rs17480168 -
CUL3 Q13618 VAR_067532 p.Asp413Gly LP/P rs199469656 Pseudohypoaldosteronism 2E (PHA2E) [MIM:614496]
CUL3 Q13618 VAR_067533 p.Lys459Arg LP/P rs199469658 Pseudohypoaldosteronism 2E (PHA2E) [MIM:614496]
CUL3 Q13618 VAR_078688 p.His719Arg US rs763087632 -
CUL4A Q13619 VAR_020341 p.Lys644Arg LB/B rs2302757 -
CUL4B Q13620 VAR_032272 p.Leu103Pro LB/B rs61759504 -
CUL4B Q13620 VAR_032273 p.Thr213Ile US rs763692058 Mental retardation, X-linked, syndromic, 15 (MRXS15) [MIM:300354]
CUL4B Q13620 VAR_032274 p.Arg572Cys LP/P rs121434615 Mental retardation, X-linked, syndromic, 15 (MRXS15) [MIM:300354]
CUL4B Q13620 VAR_032275 p.Val745Ala LP/P - Mental retardation, X-linked, syndromic, 15 (MRXS15) [MIM:300354]
CUL7 Q14999 VAR_026121 p.Gln813Arg LB/B rs9381231 -

CUL7	Q14999	VAR_026122	p.Leu1014Arg	US	rs61752334	3M syndrome 1 (3M1) [MIM:273750]
CUL7	Q14999	VAR_026123	p.Gln1246Gly	LP/P	-	3M syndrome 1 (3M1) [MIM:273750]
CUL7	Q14999	VAR_026124	p.His1464Pro	LP/P	rs121918229	3M syndrome 1 (3M1) [MIM:273750]
CUL7	Q14999	VAR_048841	p.Ser616Gly	LB/B	rs7774330	-
CUL7	Q14999	VAR_048842	p.Arg852Gln	LB/B	rs34574340	-
CUL7	Q14999	VAR_048843	p.Gln1246His	LB/B	rs36071170	-
CUL7	Q14999	VAR_071120	p.Leu1588Pro	LP/P	rs759300846	3M syndrome 1 (3M1) [MIM:273750]
CUL9	Q8IWT3	VAR_048844	p.His2058Pro	LB/B	rs2273709	-
CUL9	Q8IWT3	VAR_048845	p.Thr2180Ile	LB/B	rs11962520	-
CUSTOS	Q96C57	VAR_030491	p.Gly44Arg	LB/B	rs11537857	-
CUTC	Q9NTM9	VAR_036363	p.Pro77Leu	US	-	A breast cancer sample
CUX1	Q13948	VAR_024923	p.Ala464Thr	LB/B	rs803064	-
CUX1	Q13948	VAR_024924	p.Ile545Val	LB/B	rs2230103	-
CUX1	Q13948	VAR_036285	p.Ser490Gly	US	-	A breast cancer sample
CUX1	Q13948	VAR_036286	p.Arg609Cys	US	rs1287689842	A colorectal cancer sample
CUX2	O14529	VAR_065096	p.Val1472Leu	LB/B	rs6490073	-
CUX2	O14529	VAR_081600	p.Glu590Lys	LP/P	rs1565909334	Developmental and epileptic encephalopathy 67 (DEE67) [MIM:618141]
CUZD1	Q86UP6	VAR_061992	p.Gly156Ser	LB/B	rs35120257	-
CWC22	Q9HCG8	VAR_057513	p.Ala656Val	LB/B	rs17778270	-
CWC22	Q9HCG8	VAR_057514	p.Asp741Val	LB/B	rs11903115	-
CWC22	Q9HCG8	VAR_057515	p.Arg794Gln	LB/B	rs1046356	-
CWC27	Q6UX04	VAR_037686	p.Pro256Ala	LB/B	rs7735338	-
CWF19L1	Q69YN2	VAR_038264	p.Cys160Tyr	LB/B	rs2270962	-
CWF19L1	Q69YN2	VAR_038265	p.Pro259Leu	LB/B	rs7073610	-
CWF19L1	Q69YN2	VAR_038266	p.Arg523His	LB/B	rs35490714	-
CWF19L1	Q69YN2	VAR_038267	p.Arg526Gln	LB/B	rs7922946	-
CWF19L2	Q2TBE0	VAR_038268	p.Pro210Thr	LB/B	rs608634	-
CWF19L2	Q2TBE0	VAR_038269	p.His443Tyr	LB/B	rs659040	-
CWF19L2	Q2TBE0	VAR_038270	p.His445Gln	LB/B	rs35968518	-
CWF19L2	Q2TBE0	VAR_038271	p.Gly537Arg	LB/B	rs17106909	-
CWF19L2	Q2TBE0	VAR_038272	p.Tyr894Cys	LB/B	rs3758911	-
CWH43	Q9H720	VAR_039234	p.Pro2Thr	LB/B	rs3747690	-
CWH43	Q9H720	VAR_039235	p.His689Asn	LB/B	rs1051447	-
CX3CL1	P78423	VAR_048714	p.Ala240Val	LB/B	rs35860084	-
CX3CR1	P49238	VAR_010041	p.Thr57Ala	LB/B	rs199811198	-
CX3CR1	P49238	VAR_010042	p.Val122Ile	LB/B	rs143001773	-
CX3CR1	P49238	VAR_010043	p.Val249Ile	LB/B	rs3732379	-
CX3CR1	P49238	VAR_010044	p.Thr280Met	LB/B	rs3732378	-
CX3CR1	P49238	VAR_022062	p.Val147Ile	LB/B	rs3732380	-
CX3CR1	P49238	VAR_049386	p.Glu13Asp	LB/B	rs41535248	-
CXADR	P78310	VAR_049871	p.Ser323Arg	LB/B	rs34727960	-
CXCL11	O14625	VAR_048700	p.Asn55Ser	LB/B	rs4859596	-
CXCL16	Q9H2A7	VAR_015424	p.Ile123Thr	LB/B	-	-
CXCL16	Q9H2A7	VAR_015425	p.Ala181Val	LB/B	rs2277680	-
CXCL3	P19876	VAR_059210	p.His3Arg	LB/B	rs352043	-
CXCR1	P25024	VAR_003479	p.Ser276Thr	LB/B	rs2234671	-
CXCR1	P25024	VAR_016236	p.Arg71Cys	LB/B	rs1805038	-
CXCR1	P25024	VAR_016237	p.Ala306Thr	LB/B	rs201583693	-

CXCR1 P25024 VAR_016238 p.Arg335Cys LB/B rs16858808 -
CXCR1 P25024 VAR_021061 p.Met31Arg LB/B rs16858811 -
CXCR1 P25024 VAR_021062 p.Ser342Leu LB/B rs16858806 -
CXCR1 P25024 VAR_026525 p.Met268Leu LB/B rs9282752 -
CXCR2 P25025 VAR_014679 p.Arg80Cys LB/B rs138773569 -
CXCR3 P49682 VAR_016240 p.Arg292Gln LB/B rs139226823 -
CXCR3 P49682 VAR_016241 p.Ala363Thr LB/B rs766348940 -
CXCR5 P32302 VAR_011838 p.Gly344Ser LB/B rs665648 -
CXCR5 P32302 VAR_035757 p.Glu34Lys US - A breast cancer sample
CXCR6 O00574 VAR_003506 p.Asp25Ala US - -
CXCR6 O00574 VAR_024253 p.Glu3Lys LB/B rs2234355 -
CXorf1 O96002 VAR_033766 p.Ser36Ala LB/B rs3752359 -
CXorf38 Q8TB03 VAR_050936 p.Thr176Ala LB/B rs17145855 -
CXorf58 Q96LI9 VAR_029857 p.Arg24Cys LB/B rs2707164 -
CXorf58 Q96LI9 VAR_029858 p.Arg187His LB/B rs16982852 -
CXorf65 A6NEN9 VAR_045896 p.Arg156His LB/B rs12009522 -
CXorf66 Q5JRM2 VAR_046157 p.Pro233Leu LB/B rs5955139 -
CYB561 P49447 VAR_081731 p.Gly88Arg LP/P rs772361572 Orthostatic hypotension 2 (ORTHYP2) [MIM:618182]
CYB5A P00167 VAR_080834 p.His44Leu LP/P rs1555688659 Methemoglobinemia and ambiguous genitalia (METAG) [MIM:250790]
CYB5D1 Q6P9G0 VAR_037486 p.Phe20Leu LB/B rs12453250 -
CYB5D2 Q8WUJ1 VAR_037487 p.Arg7Gly US rs200936057 A colorectal cancer sample
CYB5D2 Q8WUJ1 VAR_037488 p.Arg7Pro US - A colorectal cancer sample
CYB5R1 Q9UHQ9 VAR_032320 p.Asn44Ser LB/B rs2232842 -
CYB5R2 Q6BCY4 VAR_032321 p.Glu15Ala LB/B rs11041525 -
CYB5R2 Q6BCY4 VAR_032322 p.Asn209Asp LB/B rs12801394 -
CYB5R3 P00387 VAR_004619 p.Arg58Gln LP/P rs121965007 Methemoglobinemia CYB5R3-related (METHB-CYB5R3) [MIM:250800]
CYB5R3 P00387 VAR_004620 p.Val106Met LP/P rs121965009 Methemoglobinemia CYB5R3-related (METHB-CYB5R3) [MIM:250800]
CYB5R3 P00387 VAR_004621 p.Ser128Pro LP/P rs121965006 Methemoglobinemia CYB5R3-related (METHB-CYB5R3) [MIM:250800]
CYB5R3 P00387 VAR_004622 p.Leu149Pro LP/P rs121965008 Methemoglobinemia CYB5R3-related (METHB-CYB5R3) [MIM:250800]
CYB5R3 P00387 VAR_010750 p.Leu73Pro LP/P rs121965013 Methemoglobinemia CYB5R3-related (METHB-CYB5R3) [MIM:250800]
CYB5R3 P00387 VAR_010751 p.Thr117Ser LB/B rs1800457 -
CYB5R3 P00387 VAR_010752 p.Ala179Val LP/P rs201232518 Methemoglobinemia CYB5R3-related (METHB-CYB5R3) [MIM:250800]
CYB5R3 P00387 VAR_010753 p.Cys204Arg LP/P rs121965011 Methemoglobinemia CYB5R3-related (METHB-CYB5R3) [MIM:250800]
CYB5R3 P00387 VAR_010754 p.Cys204Tyr LP/P rs121965015 Methemoglobinemia CYB5R3-related (METHB-CYB5R3) [MIM:250800]
CYB5R3 P00387 VAR_018419 p.Ser66Pro LB/B rs1130706 -
CYB5R3 P00387 VAR_037316 p.Gly292Asp LP/P rs121965016 Methemoglobinemia CYB5R3-related (METHB-CYB5R3) [MIM:250800]
CYB5R4 Q7L1T6 VAR_032323 p.Gln187Arg LB/B rs143478181 -
CYB5R4 Q7L1T6 VAR_032324 p.His223Arg LB/B rs141290525 -

CYB5R4 Q7L1T6 VAR_032325 p.Ser282Pro LB/B rs10080628 -
CYB5R4 Q7L1T6 VAR_036240 p.Asp371Tyr US - A breast cancer sample
CYB5R4 Q7L1T6 VAR_036241 p.Leu390Met US - A breast cancer sample
CYB5R4 Q7L1T6 VAR_047967 p.Arg140His LB/B rs61762820 -
CYB5R4 Q7L1T6 VAR_047968 p.Pro267Ala LB/B rs61382555 -
CYB5R4 Q7L1T6 VAR_047969 p.Pro316Ser LB/B rs10080628 -
CYB5RL Q6IPT4 VAR_043577 p.Arg47Lys LB/B rs946448 -
CYBA P13498 VAR_005122 p.Tyr72His LB/B rs4673 -
CYBA P13498 VAR_005123 p.Arg90Gln LP/P rs104894513 Granulomatous disease, chronic, cytochrome-b-negative, autosomal recessive (ARCGD) [MIM:233690]
CYBA P13498 VAR_005124 p.His94Arg LP/P rs104894510 Granulomatous disease, chronic, cytochrome-b-negative, autosomal recessive (ARCGD) [MIM:233690]
CYBA P13498 VAR_005125 p.Ser118Arg LP/P rs104894514 Granulomatous disease, chronic, cytochrome-b-negative, autosomal recessive (ARCGD) [MIM:233690]
CYBA P13498 VAR_005126 p.Pro156Gln LP/P rs104894515 Granulomatous disease, chronic, cytochrome-b-negative, autosomal recessive (ARCGD) [MIM:233690]
CYBA P13498 VAR_012755 p.Gly24Arg LP/P rs28941476 Granulomatous disease, chronic, cytochrome-b-negative, autosomal recessive (ARCGD) [MIM:233690]
CYBA P13498 VAR_054801 p.Val174Ala LB/B rs1049254 -
CYBA P13498 VAR_060576 p.Gly25Val LP/P rs179363891 Granulomatous disease, chronic, cytochrome-b-negative, autosomal recessive (ARCGD) [MIM:233690]
CYBA P13498 VAR_060577 p.Leu52Pro LP/P rs179363890 Granulomatous disease, chronic, cytochrome-b-negative, autosomal recessive (ARCGD) [MIM:233690]
CYBA P13498 VAR_060578 p.Glu53Val LP/P rs179363893 Granulomatous disease, chronic, cytochrome-b-negative, autosomal recessive (ARCGD) [MIM:233690]
CYBA P13498 VAR_060579 p.Arg90Trp LP/P rs179363892 Granulomatous disease, chronic, cytochrome-b-negative, autosomal recessive (ARCGD) [MIM:233690]
CYBA P13498 VAR_060580 p.Ala124Val LP/P rs179363894 Granulomatous disease, chronic, cytochrome-b-negative, autosomal recessive (ARCGD) [MIM:233690]
CYBA P13498 VAR_060581 p.Ala125Thr LP/P rs119103269 Granulomatous disease, chronic, cytochrome-b-negative, autosomal recessive (ARCGD) [MIM:233690]
CYBA P13498 VAR_060582 p.Glu171Gly LB/B rs72667005 -
CYBA P13498 VAR_060583 p.Glu193Asp LB/B rs72667006 -
CYBA P13498 VAR_071860 p.Gly25Asp LP/P rs179363891 Granulomatous disease, chronic, cytochrome-b-negative, autosomal recessive (ARCGD) [MIM:233690]
CYBB P04839 VAR_002432 p.His101Arg LP/P rs137854591 Granulomatous disease, chronic, X-linked (CGD) [MIM:306400]
CYBB P04839 VAR_002433 p.Ala156Thr LP/P rs137854590 Granulomatous disease, chronic, X-linked (CGD) [MIM:306400]
CYBB P04839 VAR_002434 p.His209Tyr LP/P rs137854587 Granulomatous disease, chronic, X-linked (CGD) [MIM:306400]
CYBB P04839 VAR_002435 p.Glu225Val LP/P rs151344494 Granulomatous disease, chronic, X-linked (CGD) [MIM:306400]
CYBB P04839 VAR_002436 p.Cys244Ser LP/P rs137854589 Granulomatous disease, chronic, X-linked (CGD) [MIM:306400]
CYBB P04839 VAR_002437 p.Cys244Tyr LP/P rs137854589 Granulomatous disease, chronic, X-linked (CGD) [MIM:306400]
CYBB P04839 VAR_002438 p.Pro339His LP/P rs151344470 Granulomatous disease, chronic, X-linked (CGD) [MIM:306400]

CYBB P04839 VAR_002439 p.Gly389Ala LP/P rs137854586 Granulomatous disease, chronic, X-linked (CGD) [MIM:306400]
CYBB P04839 VAR_002440 p.Pro415His LP/P rs137854585 Granulomatous disease, chronic, X-linked (CGD) [MIM:306400]
CYBB P04839 VAR_002441 p.Asp500Gly LP/P rs137854593 Granulomatous disease, chronic, X-linked (CGD) [MIM:306400]
CYBB P04839 VAR_007873 p.Gly20Arg LP/P rs151344455 Granulomatous disease, chronic, X-linked (CGD) [MIM:306400]
CYBB P04839 VAR_007874 p.Arg54Ser LP/P rs151344456 Granulomatous disease, chronic, X-linked (CGD) [MIM:306400]
CYBB P04839 VAR_007875 p.Cys59Arg LP/P rs151344457 Granulomatous disease, chronic, X-linked (CGD) [MIM:306400]
CYBB P04839 VAR_007876 p.His101Tyr LP/P rs137854594 Granulomatous disease, chronic, X-linked (CGD) [MIM:306400]
CYBB P04839 VAR_007877 p.His119Arg LP/P rs151344458 Granulomatous disease, chronic, X-linked (CGD) [MIM:306400]
CYBB P04839 VAR_007878 p.His209Gln LP/P rs151344459 Granulomatous disease, chronic, X-linked (CGD) [MIM:306400]
CYBB P04839 VAR_007880 p.His222Asn LP/P rs151344460 Granulomatous disease, chronic, X-linked (CGD) [MIM:306400]
CYBB P04839 VAR_007881 p.His222Arg LP/P rs151344462 Granulomatous disease, chronic, X-linked (CGD) [MIM:306400]
CYBB P04839 VAR_007882 p.His222Tyr LP/P rs151344460 Granulomatous disease, chronic, X-linked (CGD) [MIM:306400]
CYBB P04839 VAR_007883 p.Gly223Leu LP/P rs151344463 Granulomatous disease, chronic, X-linked (CGD) [MIM:306400]
CYBB P04839 VAR_007884 p.Cys244Arg LP/P rs151344465 Granulomatous disease, chronic, X-linked (CGD) [MIM:306400]
CYBB P04839 VAR_007885 p.Glu309Lys LP/P rs151344466 Granulomatous disease, chronic, X-linked (CGD) [MIM:306400]
CYBB P04839 VAR_007886 p.Gly322Glu LP/P rs151344467 Granulomatous disease, chronic, X-linked (CGD) [MIM:306400]
CYBB P04839 VAR_007887 p.Ile325Phe LP/P rs151344468 Granulomatous disease, chronic, X-linked (CGD) [MIM:306400]
CYBB P04839 VAR_007888 p.Ser333Pro LP/P rs151344469 Granulomatous disease, chronic, X-linked (CGD) [MIM:306400]
CYBB P04839 VAR_007889 p.Arg356Pro LP/P rs151344471 Granulomatous disease, chronic, X-linked (CGD) [MIM:306400]
CYBB P04839 VAR_007890 p.Met405Arg LP/P rs151344472 Granulomatous disease, chronic, X-linked (CGD) [MIM:306400]
CYBB P04839 VAR_007891 p.Gly408Glu LP/P rs151344474 Granulomatous disease, chronic, X-linked (CGD) [MIM:306400]
CYBB P04839 VAR_007892 p.Gly408Arg LP/P rs151344473 Granulomatous disease, chronic, X-linked (CGD) [MIM:306400]
CYBB P04839 VAR_007893 p.Pro415Leu LP/P rs137854585 Granulomatous disease, chronic, X-linked (CGD) [MIM:306400]
CYBB P04839 VAR_007894 p.Ser422Pro LP/P rs151344475 Granulomatous disease, chronic, X-linked (CGD) [MIM:306400]

CYBB P04839 VAR_007895 p.Trp453Arg LP/P rs151344476 Granulomatous disease, chronic, X-linked (CGD) [MIM:306400]
CYBB P04839 VAR_007896 p.Trp516Cys LP/P rs151344477 Granulomatous disease, chronic, X-linked (CGD) [MIM:306400]
CYBB P04839 VAR_007897 p.Val534Asp LP/P rs151344478 Granulomatous disease, chronic, X-linked (CGD) [MIM:306400]
CYBB P04839 VAR_007898 p.Cys537Arg LP/P rs151344454 Granulomatous disease, chronic, X-linked (CGD) [MIM:306400]
CYBB P04839 VAR_008845 p.Ala57Glu LP/P rs151344481 Granulomatous disease, chronic, X-linked (CGD) [MIM:306400]
CYBB P04839 VAR_016880 p.His303Asn LP/P rs137854595 Granulomatous disease, chronic, X-linked (CGD) [MIM:306400]
CYBB P04839 VAR_016881 p.Pro304Arg LP/P rs137854596 Granulomatous disease, chronic, X-linked (CGD) [MIM:306400]
CYBB P04839 VAR_025613 p.Tyr41Asp LP/P rs151344453 Granulomatous disease, chronic, X-linked (CGD) [MIM:306400]
CYBB P04839 VAR_025614 p.Arg54Met LP/P rs151344479 Granulomatous disease, chronic, X-linked (CGD) [MIM:306400]
CYBB P04839 VAR_025615 p.Ala55Asp LP/P rs151344480 Granulomatous disease, chronic, X-linked (CGD) [MIM:306400]
CYBB P04839 VAR_025616 p.His209Arg LP/P rs151344482 Granulomatous disease, chronic, X-linked (CGD) [MIM:306400]
CYBB P04839 VAR_025617 p.Ala224Gly LP/P rs151344483 Granulomatous disease, chronic, X-linked (CGD) [MIM:306400]
CYBB P04839 VAR_025618 p.His338Tyr LP/P rs151344484 Granulomatous disease, chronic, X-linked (CGD) [MIM:306400]
CYBB P04839 VAR_025619 p.Ser344Phe LP/P rs151344485 Granulomatous disease, chronic, X-linked (CGD) [MIM:306400]
CYBB P04839 VAR_025620 p.Gly364Arg LB/B rs141756032 -
CYBB P04839 VAR_025621 p.Gly389Glu LP/P rs137854586 Granulomatous disease, chronic, X-linked (CGD) [MIM:306400]
CYBB P04839 VAR_025622 p.Leu420Pro LP/P rs151344486 Granulomatous disease, chronic, X-linked (CGD) [MIM:306400]
CYBB P04839 VAR_025623 p.Trp516Arg LP/P rs151344487 Granulomatous disease, chronic, X-linked (CGD) [MIM:306400]
CYBB P04839 VAR_025624 p.Asp517Glu LB/B rs151344452 -
CYBB P04839 VAR_047264 p.Trp18Cys LP/P - Granulomatous disease, chronic, X-linked (CGD) [MIM:306400]
CYBB P04839 VAR_047266 p.Cys59Trp LP/P rs151344488 Granulomatous disease, chronic, X-linked (CGD) [MIM:306400]
CYBB P04839 VAR_047267 p.Gly179Arg LP/P rs151344491 Granulomatous disease, chronic, X-linked (CGD) [MIM:306400]
CYBB P04839 VAR_047268 p.Ser193Phe LP/P rs151344493 Granulomatous disease, chronic, X-linked (CGD) [MIM:306400]
CYBB P04839 VAR_047269 p.Phe205Ile LP/P rs151344496 Granulomatous disease, chronic, X-linked (CGD) [MIM:306400]
CYBB P04839 VAR_047271 p.Thr307Pro LP/P rs151344489 Granulomatous disease, chronic, X-linked (CGD) [MIM:306400]

CYBB P04839 VAR_047273 p.Leu342Gln LP/P rs151344495 Granulomatous disease, chronic, X-linked (CGD) [MIM:306400]
CYBB P04839 VAR_047274 p.Gly472Ser LB/B rs13306300 -
CYBB P04839 VAR_047275 p.Leu505Arg LP/P rs151344490 Granulomatous disease, chronic, X-linked (CGD) [MIM:306400]
CYBB P04839 VAR_047276 p.Leu546Pro LP/P rs151344492 Granulomatous disease, chronic, X-linked (CGD) [MIM:306400]
CYBB P04839 VAR_065365 p.Thr178Pro LP/P rs151344497 Immunodeficiency 34 (IMD34) [MIM:300645]
CYBB P04839 VAR_065366 p.Gln231Pro LP/P rs151344498 Immunodeficiency 34 (IMD34) [MIM:300645]
CYBB P04839 VAR_068012 p.Ala488Asp LP/P - Granulomatous disease, chronic, X-linked (CGD) [MIM:306400]
CYBB P04839 VAR_068013 p.Asp500Glu LP/P - Granulomatous disease, chronic, X-linked (CGD) [MIM:306400]
CYBB P04839 VAR_071861 p.Lys299Asn LP/P - Granulomatous disease, chronic, X-linked (CGD) [MIM:306400]
CYBB P04839 VAR_071862 p.His338Asp LP/P - Granulomatous disease, chronic, X-linked (CGD) [MIM:306400]
CYBB P04839 VAR_071863 p.Gly412Glu LP/P - Granulomatous disease, chronic, X-linked (CGD) [MIM:306400]
CYBB P04839 VAR_078386 p.Ala409Gly LP/P - Granulomatous disease, chronic, X-linked (CGD) [MIM:306400]
CYBRD1 Q53TN4 VAR_038065 p.Met156Thr LB/B rs16859487 -
CYBRD1 Q53TN4 VAR_038066 p.Arg226His LB/B rs62181680 -
CYBRD1 Q53TN4 VAR_038067 p.Ser266Asn LB/B rs10455 -
CYC1 P08574 VAR_013631 p.Leu89Val LB/B - -
CYC1 P08574 VAR_025163 p.Met76Val LB/B rs7820984 -
CYC1 P08574 VAR_070847 p.Trp96Cys LP/P rs587777041 Mitochondrial complex III deficiency, nuclear 6 (MC3DN6) [MIM:615453]
CYC1 P08574 VAR_070848 p.Leu215Phe LP/P rs587777042 Mitochondrial complex III deficiency, nuclear 6 (MC3DN6) [MIM:615453]
CYCS P99999 VAR_002204 p.Met66Leu LB/B - -
CYCS P99999 VAR_044450 p.Gly42Ser LP/P rs121918552 Thrombocytopenia 4 (THC4) [MIM:612004]
CYCS P99999 VAR_048850 p.Lys56Arg LB/B rs11548795 -
CYFIP1 Q7L576 VAR_053849 p.Ala532Pro LB/B rs34683919 -
CYFIP1 Q7L576 VAR_053850 p.Gly820Asp LB/B rs17137190 -
CYFIP1 Q7L576 VAR_053851 p.Gly820Ser LB/B rs7170637 -
CYFIP2 Q96F07 VAR_030953 p.Lys320Glu LB/B rs3207362 -
CYFIP2 Q96F07 VAR_080817 p.Arg87Cys LP/P rs1131692231 Developmental and epileptic encephalopathy 65 (DEE65) [MIM:618008]
CYFIP2 Q96F07 VAR_080818 p.Arg87Leu LP/P rs1554108163 Developmental and epileptic encephalopathy 65 (DEE65) [MIM:618008]
CYFIP2 Q96F07 VAR_080819 p.Arg87Pro LP/P rs1554108163 Developmental and epileptic encephalopathy 65 (DEE65) [MIM:618008]
CYLC1 P35663 VAR_050937 p.Asp399His LB/B rs12008888 -
CYLC2 Q14093 VAR_020100 p.Lys190Glu LB/B rs2298050 -
CYLC2 Q14093 VAR_020101 p.Gly208Asp LB/B rs2298051 -
CYLC2 Q14093 VAR_020102 p.Ala319Glu LB/B rs3763636 -
CYLC2 Q14093 VAR_050938 p.Asp146Tyr LB/B rs13293961 -
CYLC2 Q14093 VAR_050939 p.Gly184Asp LB/B rs10990424 -

CYLD Q9NQC7 VAR_045967 p.Glu747Gly LP/P rs121908389 Brooke-Spiegler syndrome (BRSS) [MIM:605041]
CYLD Q9NQC7 VAR_045967 p.Glu747Gly LP/P rs121908389 Multiple familial trichoepithelioma 1 (MFT1) [MIM:601606]
CYP11A1 P05108 VAR_013944 p.Glu314Lys LB/B rs6161 -
CYP11A1 P05108 VAR_016949 p.Ala189Val LP/P rs121912811 Adrenal insufficiency, congenital, with 46,XY sex reversal (AICSR) [MIM:613743]
CYP11A1 P05108 VAR_016951 p.Arg353Trp LP/P rs72547508 Adrenal insufficiency, congenital, with 46,XY sex reversal (AICSR) [MIM:613743]
CYP11A1 P05108 VAR_065241 p.Leu141Trp LP/P rs121912813 Adrenal insufficiency, congenital, with 46,XY sex reversal (AICSR) [MIM:613743]
CYP11A1 P05108 VAR_065242 p.Leu222Pro LP/P rs387906601 Adrenal insufficiency, congenital, with 46,XY sex reversal (AICSR) [MIM:613743]
CYP11A1 P05108 VAR_065243 p.Ala359Val LP/P rs121912812 Adrenal insufficiency, congenital, with 46,XY sex reversal (AICSR) [MIM:613743]
CYP11A1 P05108 VAR_065244 p.Val415Glu LP/P rs121912814 Adrenal insufficiency, congenital, with 46,XY sex reversal (AICSR) [MIM:613743]
CYP11B1 P15538 VAR_001260 p.Pro42Ser LP/P rs104894069 Adrenal hyperplasia 4 (AH4) [MIM:202010]
CYP11B1 P15538 VAR_001261 p.Asn133His LP/P rs104894067 Adrenal hyperplasia 4 (AH4) [MIM:202010]
CYP11B1 P15538 VAR_001262 p.Thr318Met LP/P rs104894061 Adrenal hyperplasia 4 (AH4) [MIM:202010]
CYP11B1 P15538 VAR_001263 p.Thr319Met LP/P rs104894068 Adrenal hyperplasia 4 (AH4) [MIM:202010]
CYP11B1 P15538 VAR_001264 p.Arg374Gln LP/P rs104894062 Adrenal hyperplasia 4 (AH4) [MIM:202010]
CYP11B1 P15538 VAR_001265 p.Arg448His LP/P rs28934586 Adrenal hyperplasia 4 (AH4) [MIM:202010]
CYP11B1 P15538 VAR_008687 p.Phe494Cys LB/B - -
CYP11B1 P15538 VAR_014145 p.Cys10Tyr LB/B rs6405 -
CYP11B1 P15538 VAR_014146 p.Arg43Gln LP/P rs4534 Adrenal hyperplasia 4 (AH4) [MIM:202010]
CYP11B1 P15538 VAR_014147 p.Met160Ile LB/B rs5287 -
CYP11B1 P15538 VAR_014148 p.Leu293Val LB/B rs5292 -
CYP11B1 P15538 VAR_014149 p.Ala348Thr LB/B rs6407 -
CYP11B1 P15538 VAR_014150 p.Ala386Val LB/B rs4541 -
CYP11B1 P15538 VAR_014638 p.Asp63His LB/B rs5282 -
CYP11B1 P15538 VAR_014639 p.Lys173Arg LB/B rs142163070 -
CYP11B1 P15538 VAR_014640 p.Phe257Leu LB/B rs5288 -
CYP11B1 P15538 VAR_014641 p.Ser281Asn LB/B rs5291 -
CYP11B1 P15538 VAR_014642 p.Tyr439His LB/B rs5294 -
CYP11B1 P15538 VAR_048462 p.Thr248Ile LB/B rs34620645 -
CYP11B1 P15538 VAR_048463 p.Arg404His LB/B rs4998896 -
CYP11B1 P15538 VAR_065196 p.Gly379Val LP/P - Adrenal hyperplasia 4 (AH4) [MIM:202010]
CYP11B1 P15538 VAR_065197 p.Arg454Cys LP/P rs1563867899 Adrenal hyperplasia 4 (AH4) [MIM:202010]
CYP11B1 P15538 VAR_065666 p.Pro94Leu LP/P rs104894070 Adrenal hyperplasia 4 (AH4) [MIM:202010]
CYP11B1 P15538 VAR_065667 p.Thr318Arg LP/P rs104894061 Adrenal hyperplasia 4 (AH4) [MIM:202010]
CYP11B1 P15538 VAR_074493 p.Pro42Leu LP/P rs193922538 Adrenal hyperplasia 4 (AH4) [MIM:202010]
CYP11B1 P15538 VAR_074494 p.Phe79Ile LP/P rs1489638195 Adrenal hyperplasia 4 (AH4) [MIM:202010]
CYP11B1 P15538 VAR_074495 p.Leu83Ser LP/P - Adrenal hyperplasia 4 (AH4) [MIM:202010]
CYP11B1 P15538 VAR_074496 p.Met88Ile LP/P rs193922539 Adrenal hyperplasia 4 (AH4) [MIM:202010]
CYP11B1 P15538 VAR_074497 p.Trp116Cys LP/P rs772003869 Adrenal hyperplasia 4 (AH4) [MIM:202010]
CYP11B1 P15538 VAR_074498 p.Trp116Gly LP/P rs772733691 Adrenal hyperplasia 4 (AH4) [MIM:202010]
CYP11B1 P15538 VAR_074499 p.His125Arg LP/P rs757389720 Adrenal hyperplasia 4 (AH4) [MIM:202010]
CYP11B1 P15538 VAR_074500 p.Val129Met LP/P rs377423817 Adrenal hyperplasia 4 (AH4) [MIM:202010]
CYP11B1 P15538 VAR_074501 p.Pro135Ser LP/P - Adrenal hyperplasia 4 (AH4) [MIM:202010]

CYP11B1	P15538	VAR_074502	p.Phe139Leu	LP/P	-	Adrenal hyperplasia 4 (AH4) [MIM:202010]
CYP11B1	P15538	VAR_074503	p.Arg143Trp	LP/P	rs140336749	Adrenal hyperplasia 4 (AH4) [MIM:202010]
CYP11B1	P15538	VAR_074504	p.Ser150Leu	LP/P	rs142484434	Adrenal hyperplasia 4 (AH4) [MIM:202010]
CYP11B1	P15538	VAR_074505	p.Leu158Pro	LP/P	rs1554653191	Adrenal hyperplasia 4 (AH4) [MIM:202010]
CYP11B1	P15538	VAR_074506	p.Pro159Leu	LP/P	rs370266763	Adrenal hyperplasia 4 (AH4) [MIM:202010]
CYP11B1	P15538	VAR_074508	p.Ala165Asp	LP/P	rs1554653185	Adrenal hyperplasia 4 (AH4) [MIM:202010]
CYP11B1	P15538	VAR_074509	p.Thr196Ala	LP/P	-	Adrenal hyperplasia 4 (AH4) [MIM:202010]
CYP11B1	P15538	VAR_074511	p.Gly267Asp	LP/P	-	Adrenal hyperplasia 4 (AH4) [MIM:202010]
CYP11B1	P15538	VAR_074512	p.Leu299Pro	LP/P	rs387907573	Adrenal hyperplasia 4 (AH4) [MIM:202010]
CYP11B1	P15538	VAR_074513	p.Ala306Val	LP/P	rs387907572	Adrenal hyperplasia 4 (AH4) [MIM:202010]
CYP11B1	P15538	VAR_074514	p.Glu310Lys	LP/P	rs387907574	Adrenal hyperplasia 4 (AH4) [MIM:202010]
CYP11B1	P15538	VAR_074515	p.Gly314Arg	LP/P	rs1336285846	Adrenal hyperplasia 4 (AH4) [MIM:202010]
CYP11B1	P15538	VAR_074516	p.Thr318Pro	LP/P	rs1296969984	Adrenal hyperplasia 4 (AH4) [MIM:202010]
CYP11B1	P15538	VAR_074517	p.Phe321Val	LP/P	rs1453371113	Adrenal hyperplasia 4 (AH4) [MIM:202010]
CYP11B1	P15538	VAR_074518	p.Ala331Val	LP/P	rs1326688256	Adrenal hyperplasia 4 (AH4) [MIM:202010]
CYP11B1	P15538	VAR_074519	p.Arg332Gln	LP/P	rs149881706	Adrenal hyperplasia 4 (AH4) [MIM:202010]
CYP11B1	P15538	VAR_074520	p.Arg341Ser	LP/P	rs372115638	Adrenal hyperplasia 4 (AH4) [MIM:202010]
CYP11B1	P15538	VAR_074521	p.Arg366Cys	LP/P	rs773245244	Adrenal hyperplasia 4 (AH4) [MIM:202010]
CYP11B1	P15538	VAR_074522	p.Ala368Asp	LP/P	rs104894071	Adrenal hyperplasia 4 (AH4) [MIM:202010]
CYP11B1	P15538	VAR_074523	p.Glu371Gly	LP/P	rs368944209	Adrenal hyperplasia 4 (AH4) [MIM:202010]
CYP11B1	P15538	VAR_074524	p.Arg384Gly	LP/P	-	Adrenal hyperplasia 4 (AH4) [MIM:202010]
CYP11B1	P15538	VAR_074525	p.Arg384Gln	LP/P	rs764598023	Adrenal hyperplasia 4 (AH4) [MIM:202010]
CYP11B1	P15538	VAR_074526	p.Thr401Ala	LP/P	rs201300785	Adrenal hyperplasia 4 (AH4) [MIM:202010]
CYP11B1	P15538	VAR_074527	p.Arg427His	LP/P	rs754432887	Adrenal hyperplasia 4 (AH4) [MIM:202010]
CYP11B1	P15538	VAR_074529	p.Val441Gly	LP/P	rs772169059	Adrenal hyperplasia 4 (AH4) [MIM:202010]
CYP11B1	P15538	VAR_074530	p.Gly444Asp	LP/P	rs779103938	Adrenal hyperplasia 4 (AH4) [MIM:202010]
CYP11B1	P15538	VAR_074531	p.Arg448Cys	LP/P	rs1221010438	Adrenal hyperplasia 4 (AH4) [MIM:202010]
CYP11B1	P15538	VAR_074532	p.Arg453Gln	LP/P	rs1447069098	Adrenal hyperplasia 4 (AH4) [MIM:202010]
CYP11B1	P15538	VAR_074534	p.Leu489Ser	LP/P	rs750428278	Adrenal hyperplasia 4 (AH4) [MIM:202010]
CYP11B1	P15538	VAR_075553	p.Arg141Gln	US	rs267601810	Adrenal hyperplasia 4 (AH4) [MIM:202010]
CYP11B1	P15538	VAR_075554	p.Arg332Gly	LP/P	-	Adrenal hyperplasia 4 (AH4) [MIM:202010]
CYP11B2	P19099	VAR_001266	p.Lys173Arg	LB/B	rs4539	-
CYP11B2	P19099	VAR_001267	p.Arg181Trp	LP/P	rs28931609	Corticosterone methyloxidase 2 deficiency (CMO-2 deficiency) [MIM:610600]
CYP11B2	P19099	VAR_001268	p.Glu198Asp	LP/P	rs104894072	Corticosterone methyloxidase 2 deficiency (CMO-2 deficiency) [MIM:610600]
CYP11B2	P19099	VAR_001269	p.Val386Ala	LP/P	rs61757294	Corticosterone methyloxidase 2 deficiency (CMO-2 deficiency) [MIM:610600]
CYP11B2	P19099	VAR_014151	p.Ala29Thr	LB/B	rs6438	-
CYP11B2	P19099	VAR_014152	p.Arg30Gln	LB/B	rs6441	-
CYP11B2	P19099	VAR_014153	p.Ile248Thr	LB/B	rs4547	-
CYP11B2	P19099	VAR_014154	p.Asn281Ser	LB/B	rs4537	-
CYP11B2	P19099	VAR_014155	p.Ile339Thr	LB/B	rs4544	-
CYP11B2	P19099	VAR_014156	p.Gly435Ser	LB/B	rs4545	-
CYP11B2	P19099	VAR_014643	p.Asn222Thr	LB/B	rs5308	-
CYP11B2	P19099	VAR_014644	p.Glu383Val	LB/B	rs5312	-
CYP11B2	P19099	VAR_014645	p.Val403Glu	LB/B	rs5315	-
CYP11B2	P19099	VAR_014646	p.Phe487Val	LB/B	rs5317	-

CYP11B2 P19099 VAR_018471 p.Thr185Ile LP/P rs121912978 Corticosterone methyloxidase 2 deficiency (CMO-2 deficiency) [MIM:610600]
CYP11B2 P19099 VAR_018472 p.Leu461Pro LP/P rs72554627 Corticosterone methyloxidase 1 deficiency (CMO-1 deficiency) [MIM:203400]
CYP11B2 P19099 VAR_018473 p.Thr498Ala LP/P rs72554626 Corticosterone methyloxidase 2 deficiency (CMO-2 deficiency) [MIM:610600]
CYP17A1 P05093 VAR_001271 p.Tyr64Ser LP/P rs1183147390 Adrenal hyperplasia 5 (AH5) [MIM:202110]
CYP17A1 P05093 VAR_001272 p.Ser106Pro LP/P rs104894135 Adrenal hyperplasia 5 (AH5) [MIM:202110]
CYP17A1 P05093 VAR_001274 p.Pro342Thr LP/P rs104894137 Adrenal hyperplasia 5 (AH5) [MIM:202110]
CYP17A1 P05093 VAR_001275 p.Arg347His LP/P rs61754278 Adrenal hyperplasia 5 (AH5) [MIM:202110]
CYP17A1 P05093 VAR_001276 p.Arg358Gln LP/P rs104894139 Adrenal hyperplasia 5 (AH5) [MIM:202110]
CYP17A1 P05093 VAR_001277 p.His373Leu LP/P rs760695410 Adrenal hyperplasia 5 (AH5) [MIM:202110]
CYP17A1 P05093 VAR_001278 p.Arg440His LP/P rs777638364 Adrenal hyperplasia 5 (AH5) [MIM:202110]
CYP17A1 P05093 VAR_001280 p.Arg496Cys LP/P rs1250463562 Adrenal hyperplasia 5 (AH5) [MIM:202110]
CYP17A1 P05093 VAR_011755 p.Cys22Trp LB/B rs762563 -
CYP17A1 P05093 VAR_013147 p.Phe93Cys LP/P rs104894146 Adrenal hyperplasia 5 (AH5) [MIM:202110]
CYP17A1 P05093 VAR_022745 p.Pro35Leu LP/P - Adrenal hyperplasia 5 (AH5) [MIM:202110]
CYP17A1 P05093 VAR_022746 p.Arg96Trp LP/P rs104894138 Adrenal hyperplasia 5 (AH5) [MIM:202110]
CYP17A1 P05093 VAR_022747 p.Phe114Val LP/P rs104894147 Adrenal hyperplasia 5 (AH5) [MIM:202110]
CYP17A1 P05093 VAR_022748 p.Asp116Val LP/P rs104894148 Adrenal hyperplasia 5 (AH5) [MIM:202110]
CYP17A1 P05093 VAR_022749 p.Asn177Asp LP/P - Adrenal hyperplasia 5 (AH5) [MIM:202110]
CYP17A1 P05093 VAR_022750 p.Tyr329Asp LP/P rs104894144 Adrenal hyperplasia 5 (AH5) [MIM:202110]
CYP17A1 P05093 VAR_022752 p.Arg347Cys LP/P rs104894149 Adrenal hyperplasia 5 (AH5) [MIM:202110]
CYP17A1 P05093 VAR_022753 p.Arg362Cys LP/P rs104894142 Adrenal hyperplasia 5 (AH5) [MIM:202110]
CYP17A1 P05093 VAR_022754 p.Trp406Arg LP/P rs104894143 Adrenal hyperplasia 5 (AH5) [MIM:202110]
CYP17A1 P05093 VAR_022755 p.Phe417Cys LP/P rs104894140 Adrenal hyperplasia 5 (AH5) [MIM:202110]
CYP17A1 P05093 VAR_022756 p.Pro428Leu LP/P rs104894145 Adrenal hyperplasia 5 (AH5) [MIM:202110]
CYP17A1 P05093 VAR_022757 p.Arg496His LP/P rs763398879 Adrenal hyperplasia 5 (AH5) [MIM:202110]
CYP17A1 P05093 VAR_073043 p.Arg96Gln LP/P rs104894153 Adrenal hyperplasia 5 (AH5) [MIM:202110]
CYP17A1 P05093 VAR_073044 p.Trp121Arg LP/P - Adrenal hyperplasia 5 (AH5) [MIM:202110]
CYP17A1 P05093 VAR_073045 p.Ala174Glu LP/P - Adrenal hyperplasia 5 (AH5) [MIM:202110]
CYP17A1 P05093 VAR_073046 p.His373Asn LP/P rs1423560123 Adrenal hyperplasia 5 (AH5) [MIM:202110]
CYP17A1 P05093 VAR_073047 p.Trp406Leu LP/P - Adrenal hyperplasia 5 (AH5) [MIM:202110]
CYP19A1 P11511 VAR_016962 p.Arg365Gln LP/P rs80051519 Aromatase deficiency (AROD) [MIM:613546]
CYP19A1 P11511 VAR_016963 p.Arg375Cys LP/P rs121434536 Aromatase deficiency (AROD) [MIM:613546]
CYP19A1 P11511 VAR_016964 p.Arg435Cys LP/P rs121434534 Aromatase deficiency (AROD) [MIM:613546]
CYP19A1 P11511 VAR_016965 p.Cys437Tyr LP/P rs78310315 Aromatase deficiency (AROD) [MIM:613546]
CYP19A1 P11511 VAR_018406 p.Arg264Cys LB/B rs700519 -
CYP19A1 P11511 VAR_023428 p.Trp39Arg LB/B rs2236722 -
CYP19A1 P11511 VAR_023429 p.Thr201Met LB/B rs28757184 -
CYP19A1 P11511 VAR_054152 p.Arg375Leu LB/B rs762631156 -
CYP19A1 P11511 VAR_072784 p.Arg192His LP/P rs765057534 Aromatase deficiency (AROD) [MIM:613546]
CYP19A1 P11511 VAR_077526 p.Arg264His LB/B rs2304462 -
CYP19A1 P11511 VAR_079486 p.Ser314Pro US - -
CYP1A1 P04798 VAR_001243 p.Ile462Val LB/B rs1048943 -
CYP1A1 P04798 VAR_008342 p.Thr461Asn LB/B rs1799814 -
CYP1A1 P04798 VAR_009280 p.Arg279Trp LB/B rs34260157 -
CYP1A1 P04798 VAR_016937 p.Met331Ile LB/B rs56313657 -
CYP1A1 P04798 VAR_016938 p.Ile448Asn LB/B rs72547509 -

CYP1A1	P04798	VAR_016939	p.Arg464Cys	LB/B	rs41279188	-
CYP1A1	P04798	VAR_016940	p.Arg464Ser	LB/B	rs41279188	-
CYP1A1	P04798	VAR_016941	p.Arg477Trp	LB/B	rs56240201	-
CYP1A1	P04798	VAR_016942	p.Pro492Arg	LB/B	rs28399430	-
CYP1A1	P04798	VAR_020122	p.Ile286Thr	LB/B	rs4987133	-
CYP1A1	P04798	VAR_023194	p.Gly45Asp	LB/B	rs4646422	-
CYP1A1	P04798	VAR_023195	p.Ile78Thr	LB/B	rs17861094	-
CYP1A1	P04798	VAR_024706	p.Arg93Trp	LB/B	rs2229150	-
CYP1A1	P04798	VAR_024707	p.Thr173Arg	LB/B	rs28399427	-
CYP1A1	P04798	VAR_024708	p.Val482Met	LB/B	rs28399429	-
CYP1A1	P04798	VAR_033817	p.Met66Val	LB/B	rs35035798	-
CYP1A1	P04798	VAR_033818	p.Phe470Val	LB/B	rs36121583	-
CYP1A2	P05177	VAR_008349	p.Phe21Leu	LB/B	rs56160784	-
CYP1A2	P05177	VAR_020793	p.Asp348Asn	LB/B	rs56276455	-
CYP1A2	P05177	VAR_020794	p.Ile386Phe	LB/B	rs72547516	-
CYP1A2	P05177	VAR_020795	p.Cys406Tyr	LB/B	rs55889066	-
CYP1A2	P05177	VAR_020796	p.Arg431Trp	LB/B	rs28399424	-
CYP1A2	P05177	VAR_020848	p.Thr83Met	LB/B	rs138652540	-
CYP1A2	P05177	VAR_020849	p.Glu168Gln	LB/B	rs72547512	-
CYP1A2	P05177	VAR_020850	p.Phe186Leu	LB/B	rs72547513	-
CYP1A2	P05177	VAR_020851	p.Ser212Cys	LB/B	rs758748797	-
CYP1A2	P05177	VAR_020852	p.Gly299Ser	LB/B	rs35796837	-
CYP1A2	P05177	VAR_020853	p.Thr438Ile	LB/B	rs45486893	-
CYP1A2	P05177	VAR_023196	p.Ser18Cys	LB/B	rs17861152	-
CYP1A2	P05177	VAR_024709	p.Ser298Arg	LB/B	rs17861157	-
CYP1A2	P05177	VAR_024710	p.Ile314Val	LB/B	rs28399418	-
CYP1A2	P05177	VAR_025182	p.Pro42Arg	LB/B	rs72547511	-
CYP1A2	P05177	VAR_025183	p.Gly73Arg	LB/B	rs45565238	-
CYP1A2	P05177	VAR_025184	p.Asp104Asn	LB/B	rs34067076	-
CYP1A2	P05177	VAR_025185	p.Leu111Phe	LB/B	rs45442197	-
CYP1A2	P05177	VAR_025186	p.Phe205Val	LB/B	rs45540640	-
CYP1A2	P05177	VAR_025187	p.Arg281Trp	LB/B	rs45468096	-
CYP1A2	P05177	VAR_025188	p.Arg377Gln	LB/B	rs72547515	-
CYP1A2	P05177	VAR_025189	p.Arg456His	LB/B	rs72547517	-
CYP1A2	P05177	VAR_055563	p.Arg457Trp	LB/B	rs34151816	-
CYP1B1	Q16678	VAR_001244	p.Gly61Glu	LP/P	rs28936700	Glaucoma 3, primary congenital, A (GLC3A) [MIM:231300]
CYP1B1	Q16678	VAR_001245	p.Gly365Trp	LP/P	rs55771538	Glaucoma 3, primary congenital, A (GLC3A) [MIM:231300]
CYP1B1	Q16678	VAR_001246	p.Asp374Asn	LP/P	rs104893622	Glaucoma 3, primary congenital, A (GLC3A) [MIM:231300]
CYP1B1	Q16678	VAR_001247	p.Arg469Trp	LP/P	rs28936701	Glaucoma 3, primary congenital, A (GLC3A) [MIM:231300]
CYP1B1	Q16678	VAR_001248	p.Leu432Val	LB/B	rs1056836	-
CYP1B1	Q16678	VAR_008350	p.Trp57Cys	LP/P	rs72549387	Glaucoma 3, primary congenital, A (GLC3A) [MIM:231300]
CYP1B1	Q16678	VAR_008351	p.Pro379Leu	LB/B	rs56305281	-
CYP1B1	Q16678	VAR_008352	p.Glu387Lys	LP/P	rs55989760	Glaucoma 3, primary congenital, A (GLC3A) [MIM:231300]

CYP1B1 Q16678 VAR_008353 p.Arg390His LP/P rs56010818 Glaucoma 3, primary congenital, A (GLC3A) [MIM:231300]
CYP1B1 Q16678 VAR_008354 p.Pro437Leu LP/P rs56175199 Glaucoma 3, primary congenital, A (GLC3A) [MIM:231300]
CYP1B1 Q16678 VAR_008355 p.Asn453Ser LB/B rs1800440 -
CYP1B1 Q16678 VAR_011752 p.Arg48Gly LB/B rs10012 -
CYP1B1 Q16678 VAR_011753 p.Ala119Ser LB/B rs1056827 -
CYP1B1 Q16678 VAR_016034 p.Arg368His LP/P rs79204362 Glaucoma 1, open angle, A (GLC1A) [MIM:137750]
CYP1B1 Q16678 VAR_016034 p.Arg368His LP/P rs79204362 Glaucoma 3, primary congenital, A (GLC3A) [MIM:231300]
CYP1B1 Q16678 VAR_018774 p.Ala443Gly US rs4986888 Glaucoma 3, primary congenital, A (GLC3A) [MIM:231300]
CYP1B1 Q16678 VAR_018869 p.Ser206Asn LB/B rs9341248 -
CYP1B1 Q16678 VAR_018870 p.Arg266Leu LB/B rs9341250 -
CYP1B1 Q16678 VAR_028735 p.Gln68Arg LB/B rs9282670 -
CYP1B1 Q16678 VAR_028736 p.Tyr81Asn LP/P rs9282671 Glaucoma 3, primary congenital, A (GLC3A) [MIM:231300]
CYP1B1 Q16678 VAR_028737 p.Asp441His LB/B rs4986887 -
CYP1B1 Q16678 VAR_028738 p.Asp449Glu LB/B rs1056837 -
CYP1B1 Q16678 VAR_054227 p.Ser28Trp LP/P rs780002791 Glaucoma 3, primary congenital, A (GLC3A) [MIM:231300]
CYP1B1 Q16678 VAR_054228 p.Pro52Leu LB/B rs201824781 -
CYP1B1 Q16678 VAR_054229 p.Leu77Pro LP/P - Glaucoma 3, primary congenital, A (GLC3A) [MIM:231300]
CYP1B1 Q16678 VAR_054230 p.Ala115Pro LP/P rs764338357 Glaucoma 3, primary congenital, A (GLC3A) [MIM:231300]
CYP1B1 Q16678 VAR_054231 p.Met132Arg LP/P - Glaucoma 3, primary congenital, A (GLC3A) [MIM:231300]
CYP1B1 Q16678 VAR_054232 p.Gln144His LB/B - -
CYP1B1 Q16678 VAR_054233 p.Gln144Pro LP/P - Glaucoma 3, primary congenital, A (GLC3A) [MIM:231300]
CYP1B1 Q16678 VAR_054234 p.Gln144Arg LP/P rs753847648 Glaucoma 3, primary congenital, A (GLC3A) [MIM:231300]
CYP1B1 Q16678 VAR_054235 p.Arg145Trp LP/P - Glaucoma 3, primary congenital, A (GLC3A) [MIM:231300]
CYP1B1 Q16678 VAR_054236 p.Gly184Ser LB/B - -
CYP1B1 Q16678 VAR_054237 p.Ala189Pro LB/B rs1326854156 -
CYP1B1 Q16678 VAR_054238 p.Asp192Val LP/P - Glaucoma 3, primary congenital, A (GLC3A) [MIM:231300]
CYP1B1 Q16678 VAR_054239 p.Pro193Leu LP/P rs529769268 Glaucoma 3, primary congenital, A (GLC3A) [MIM:231300]
CYP1B1 Q16678 VAR_054240 p.Val198Ile LP/P rs59472972 Glaucoma 3, primary congenital, A (GLC3A) [MIM:231300]
CYP1B1 Q16678 VAR_054241 p.Asn203Ser LP/P rs1426636145 Glaucoma 3, primary congenital, A (GLC3A) [MIM:231300]
CYP1B1 Q16678 VAR_054242 p.Ser215Ile LP/P rs72549384 Glaucoma 3, primary congenital, A (GLC3A) [MIM:231300]

CYP1B1 Q16678 VAR_054243 p.Glu229Lys LP/P rs57865060 Glaucoma 3, primary congenital, A (GLC3A) [MIM:231300]
CYP1B1 Q16678 VAR_054244 p.Gly232Arg LP/P rs104893628 Glaucoma 3, primary congenital, A (GLC3A) [MIM:231300]
CYP1B1 Q16678 VAR_054245 p.Ser239Arg LP/P - Glaucoma 3, primary congenital, A (GLC3A) [MIM:231300]
CYP1B1 Q16678 VAR_054247 p.Val320Leu US rs72549382 Glaucoma 3, primary congenital, A (GLC3A) [MIM:231300]
CYP1B1 Q16678 VAR_054248 p.Ala330Phe US - Glaucoma 3, primary congenital, A (GLC3A) [MIM:231300]
CYP1B1 Q16678 VAR_054249 p.Ala330Ser LB/B rs752456881 -
CYP1B1 Q16678 VAR_054251 p.Leu345Phe LP/P rs66583685 Glaucoma 3, primary congenital, A (GLC3A) [MIM:231300]
CYP1B1 Q16678 VAR_054253 p.Val364Met LP/P rs72549379 Glaucoma 3, primary congenital, A (GLC3A) [MIM:231300]
CYP1B1 Q16678 VAR_054254 p.Ala388Thr LP/P - Glaucoma 3, primary congenital, A (GLC3A) [MIM:231300]
CYP1B1 Q16678 VAR_054255 p.Arg390Cys LP/P rs148542782 Glaucoma 3, primary congenital, A (GLC3A) [MIM:231300]
CYP1B1 Q16678 VAR_054256 p.Arg390Ser LP/P rs148542782 Glaucoma 3, primary congenital, A (GLC3A) [MIM:231300]
CYP1B1 Q16678 VAR_054257 p.Ile399Ser LP/P rs72549378 Glaucoma 3, primary congenital, A (GLC3A) [MIM:231300]
CYP1B1 Q16678 VAR_054258 p.Val409Phe LP/P rs957253424 Glaucoma 3, primary congenital, A (GLC3A) [MIM:231300]
CYP1B1 Q16678 VAR_054259 p.Val422Gly LB/B - -
CYP1B1 Q16678 VAR_054260 p.Asn423Tyr LP/P rs104893629 Glaucoma 3, primary congenital, A (GLC3A) [MIM:231300]
CYP1B1 Q16678 VAR_054261 p.Arg444Gln LP/P rs72549376 Glaucoma 3, primary congenital, A (GLC3A) [MIM:231300]
CYP1B1 Q16678 VAR_054262 p.Phe445Cys LP/P - Glaucoma 3, primary congenital, A (GLC3A) [MIM:231300]
CYP1B1 Q16678 VAR_054263 p.Gly466Asp LP/P rs868208502 Glaucoma 3, primary congenital, A (GLC3A) [MIM:231300]
CYP1B1 Q16678 VAR_054264 p.Glu499Gly LP/P rs72549372 Glaucoma 3, primary congenital, A (GLC3A) [MIM:231300]
CYP1B1 Q16678 VAR_054265 p.Ser515Leu US - Glaucoma 3, primary congenital, A (GLC3A) [MIM:231300]
CYP1B1 Q16678 VAR_054266 p.Val518Ala LB/B - -
CYP1B1 Q16678 VAR_054267 p.Arg523Thr LP/P - Glaucoma 3, primary congenital, A (GLC3A) [MIM:231300]
CYP1B1 Q16678 VAR_054268 p.Asp530Gly LP/P - Glaucoma 3, primary congenital, A (GLC3A) [MIM:231300]
CYP20A1 Q6UW02 VAR_059153 p.Ser97Leu LB/B rs2043449 -
CYP20A1 Q6UW02 VAR_059154 p.Leu346Phe LB/B rs1048013 -
CYP21A2 P08686 VAR_001281 p.Pro30Leu LP/P rs9378251 Adrenal hyperplasia 3 (AH3) [MIM:201910]
CYP21A2 P08686 VAR_001282 p.Lys98Arg LB/B rs1268071078 -
CYP21A2 P08686 VAR_001283 p.Lys102Arg LB/B rs6474 -
CYP21A2 P08686 VAR_001284 p.Pro105Leu LP/P rs550051210 Adrenal hyperplasia 3 (AH3) [MIM:201910]

CYP21A2	P08686	VAR_001285	p.Cys169Tyr	LP/P	-	Adrenal hyperplasia 3 (AH3) [MIM:201910]
CYP21A2	P08686	VAR_001286	p.Ile172Asn	LP/P	rs6475	Adrenal hyperplasia 3 (AH3) [MIM:201910]
CYP21A2	P08686	VAR_001287	p.Asp183Glu	LB/B	rs397515531	-
CYP21A2	P08686	VAR_001288	p.Ile236Asn	LP/P	rs111647200	Adrenal hyperplasia 3 (AH3) [MIM:201910]
CYP21A2	P08686	VAR_001289	p.Val237Glu	LP/P	rs12530380	Adrenal hyperplasia 3 (AH3) [MIM:201910]
CYP21A2	P08686	VAR_001290	p.Met239Lys	LP/P	rs6476	Adrenal hyperplasia 3 (AH3) [MIM:201910]
CYP21A2	P08686	VAR_001291	p.Ser268Thr	LB/B	rs6472	-
CYP21A2	P08686	VAR_001292	p.Val281Leu	LP/P	rs6471	Adrenal hyperplasia 3 (AH3) [MIM:201910]
CYP21A2	P08686	VAR_001293	p.Gly291Ser	LP/P	rs201552310	Adrenal hyperplasia 3 (AH3) [MIM:201910]
CYP21A2	P08686	VAR_001294	p.Arg339His	LP/P	rs72552754	Adrenal hyperplasia 3 (AH3) [MIM:201910]
CYP21A2	P08686	VAR_001295	p.Arg341Trp	LP/P	rs72552755	Adrenal hyperplasia 3 (AH3) [MIM:201910]
CYP21A2	P08686	VAR_001296	p.Arg356Pro	LP/P	-	Adrenal hyperplasia 3 (AH3) [MIM:201910]
CYP21A2	P08686	VAR_001297	p.Arg356Gln	LP/P	rs574370139	Adrenal hyperplasia 3 (AH3) [MIM:201910]
CYP21A2	P08686	VAR_001298	p.Arg356Trp	LP/P	rs7769409	Adrenal hyperplasia 3 (AH3) [MIM:201910]
CYP21A2	P08686	VAR_001299	p.Glu380Asp	LP/P	rs72552756	Adrenal hyperplasia 3 (AH3) [MIM:201910]
CYP21A2	P08686	VAR_001300	p.Pro453Ser	LP/P	rs6445	Adrenal hyperplasia 3 (AH3) [MIM:201910]
CYP21A2	P08686	VAR_001301	p.Arg483Pro	LP/P	rs200005406	Adrenal hyperplasia 3 (AH3) [MIM:201910]
CYP21A2	P08686	VAR_001302	p.Asn493Ser	LB/B	rs6473	-
CYP21A2	P08686	VAR_007923	p.Gly64Glu	LP/P	-	Adrenal hyperplasia 3 (AH3) [MIM:201910]
CYP21A2	P08686	VAR_007924	p.Ala362Val	LP/P	-	Adrenal hyperplasia 3 (AH3) [MIM:201910]
CYP21A2	P08686	VAR_018364	p.His62Leu	LP/P	rs9378252	Adrenal hyperplasia 3 (AH3) [MIM:201910]
CYP21A2	P08686	VAR_018365	p.Gly291Arg	LP/P	rs201552310	Adrenal hyperplasia 3 (AH3) [MIM:201910]
CYP21A2	P08686	VAR_018366	p.Ser301Tyr	LP/P	-	Adrenal hyperplasia 3 (AH3) [MIM:201910]
CYP21A2	P08686	VAR_018367	p.Arg341Pro	LP/P	rs747079101	Adrenal hyperplasia 3 (AH3) [MIM:201910]
CYP21A2	P08686	VAR_018368	p.Arg483Gln	LP/P	rs200005406	Adrenal hyperplasia 3 (AH3) [MIM:201910]
CYP21A2	P08686	VAR_026059	p.Ala15Thr	US	rs63749090	Adrenal hyperplasia 3 (AH3) [MIM:201910]
CYP21A2	P08686	VAR_026060	p.Pro30Gln	LP/P	-	Adrenal hyperplasia 3 (AH3) [MIM:201910]
CYP21A2	P08686	VAR_026061	p.Gly90Val	LP/P	-	Adrenal hyperplasia 3 (AH3) [MIM:201910]
CYP21A2	P08686	VAR_026062	p.Arg124His	LP/P	rs72552750	Adrenal hyperplasia 3 (AH3) [MIM:201910]
CYP21A2	P08686	VAR_026063	p.Gly178Ala	LP/P	rs72552751	Adrenal hyperplasia 3 (AH3) [MIM:201910]
CYP21A2	P08686	VAR_026064	p.Val211Leu	LP/P	-	Adrenal hyperplasia 3 (AH3) [MIM:201910]
CYP21A2	P08686	VAR_026065	p.Leu261Pro	LP/P	rs750337015	Adrenal hyperplasia 3 (AH3) [MIM:201910]
CYP21A2	P08686	VAR_026066	p.Val281Gly	LP/P	-	Adrenal hyperplasia 3 (AH3) [MIM:201910]
CYP21A2	P08686	VAR_026067	p.Met283Leu	LP/P	-	Adrenal hyperplasia 3 (AH3) [MIM:201910]
CYP21A2	P08686	VAR_026068	p.Gly291Cys	LP/P	-	Adrenal hyperplasia 3 (AH3) [MIM:201910]
CYP21A2	P08686	VAR_026069	p.Leu300Phe	LP/P	rs765001985	Adrenal hyperplasia 3 (AH3) [MIM:201910]
CYP21A2	P08686	VAR_026070	p.Val304Met	LB/B	rs151344505	-
CYP21A2	P08686	VAR_026071	p.Leu317Met	LP/P	-	Adrenal hyperplasia 3 (AH3) [MIM:201910]
CYP21A2	P08686	VAR_026072	p.Arg354Cys	LP/P	rs772900496	Adrenal hyperplasia 3 (AH3) [MIM:201910]
CYP21A2	P08686	VAR_026073	p.Arg354His	LP/P	rs760216630	Adrenal hyperplasia 3 (AH3) [MIM:201910]
CYP21A2	P08686	VAR_026074	p.Leu363Trp	LP/P	-	Adrenal hyperplasia 3 (AH3) [MIM:201910]
CYP21A2	P08686	VAR_026075	p.His365Tyr	LP/P	rs1330554738	Adrenal hyperplasia 3 (AH3) [MIM:201910]
CYP21A2	P08686	VAR_026076	p.Gly375Ser	LB/B	rs151344506	-
CYP21A2	P08686	VAR_026077	p.Arg408Cys	LP/P	rs72552757	Adrenal hyperplasia 3 (AH3) [MIM:201910]
CYP21A2	P08686	VAR_026078	p.Gly424Ser	LP/P	rs72552758	Adrenal hyperplasia 3 (AH3) [MIM:201910]
CYP21A2	P08686	VAR_026079	p.Arg426His	LP/P	rs151344504	Adrenal hyperplasia 3 (AH3) [MIM:201910]
CYP21A2	P08686	VAR_026080	p.Arg435Cys	LP/P	rs767333157	Adrenal hyperplasia 3 (AH3) [MIM:201910]
CYP21A2	P08686	VAR_026081	p.Arg479Leu	LP/P	rs184649564	Adrenal hyperplasia 3 (AH3) [MIM:201910]
CYP21A2	P08686	VAR_026082	p.Pro482Ser	LP/P	rs776989258	Adrenal hyperplasia 3 (AH3) [MIM:201910]

CYP21A2	P08686	VAR_026083	p.Arg483Trp	LP/P	rs759736443	Adrenal hyperplasia 3 (AH3) [MIM:201910]
CYP21A2	P08686	VAR_065668	p.Gly56Arg	LP/P	rs1413433421	Adrenal hyperplasia 3 (AH3) [MIM:201910]
CYP21A2	P08686	VAR_065669	p.Ile77Thr	LP/P	rs1333278223	Adrenal hyperplasia 3 (AH3) [MIM:201910]
CYP21A2	P08686	VAR_065670	p.Leu107Arg	LP/P	rs957886272	Adrenal hyperplasia 3 (AH3) [MIM:201910]
CYP21A2	P08686	VAR_065671	p.Lys121Gln	LP/P	rs547552654	Adrenal hyperplasia 3 (AH3) [MIM:201910]
CYP21A2	P08686	VAR_065672	p.Leu142Pro	LP/P	rs755020999	Adrenal hyperplasia 3 (AH3) [MIM:201910]
CYP21A2	P08686	VAR_065673	p.Leu167Pro	LP/P	-	Adrenal hyperplasia 3 (AH3) [MIM:201910]
CYP21A2	P08686	VAR_065674	p.Ile230Thr	LP/P	-	Adrenal hyperplasia 3 (AH3) [MIM:201910]
CYP21A2	P08686	VAR_065675	p.Arg233Lys	LP/P	-	Adrenal hyperplasia 3 (AH3) [MIM:201910]
CYP21A2	P08686	VAR_065676	p.Gly292Asp	LP/P	-	Adrenal hyperplasia 3 (AH3) [MIM:201910]
CYP21A2	P08686	VAR_065677	p.Glu320Lys	LP/P	-	Adrenal hyperplasia 3 (AH3) [MIM:201910]
CYP21A2	P08686	VAR_065678	p.Arg369Trp	LP/P	rs781074931	Adrenal hyperplasia 3 (AH3) [MIM:201910]
CYP21A2	P08686	VAR_075372	p.Cys169Arg	LP/P	-	Adrenal hyperplasia 3 (AH3) [MIM:201910]
CYP21A2	P08686	VAR_075373	p.Gly178Arg	LP/P	rs772317717	Adrenal hyperplasia 3 (AH3) [MIM:201910]
CYP21A2	P08686	VAR_075374	p.Tyr191His	LP/P	-	Adrenal hyperplasia 3 (AH3) [MIM:201910]
CYP21A2	P08686	VAR_075375	p.Leu198Phe	LP/P	rs143240527	Adrenal hyperplasia 3 (AH3) [MIM:201910]
CYP21A2	P08686	VAR_075376	p.His282Asn	LP/P	-	Adrenal hyperplasia 3 (AH3) [MIM:201910]
CYP21A2	P08686	VAR_075377	p.Trp302Arg	LP/P	-	Adrenal hyperplasia 3 (AH3) [MIM:201910]
CYP21A2	P08686	VAR_075378	p.Arg426Cys	LP/P	rs1370167869	Adrenal hyperplasia 3 (AH3) [MIM:201910]
CYP21A2	P08686	VAR_077582	p.Leu12Met	US	-	Adrenal hyperplasia 3 (AH3) [MIM:201910]
CYP21A2	P08686	VAR_077583	p.Arg16Cys	LB/B	rs757608533	-
CYP21A2	P08686	VAR_077584	p.Ser113Phe	LP/P	rs1296268275	Adrenal hyperplasia 3 (AH3) [MIM:201910]
CYP21A2	P08686	VAR_077585	p.Ser202Gly	LB/B	rs372964292	-
CYP21A2	P08686	VAR_077586	p.Pro267Leu	LB/B	rs61732108	-
CYP21A2	P08686	VAR_077588	p.Thr450Met	LB/B	rs1319651744	-
CYP21A2	P08686	VAR_077589	p.Thr450Pro	LP/P	-	Adrenal hyperplasia 3 (AH3) [MIM:201910]
CYP24A1	Q07973	VAR_048464	p.Arg157Gln	LB/B	rs35051736	-
CYP24A1	Q07973	VAR_048465	p.Met374Thr	LB/B	rs6022990	-
CYP24A1	Q07973	VAR_048466	p.Leu409Ser	LP/P	rs6068812	Hypercalcemia, infantile, 1 (HCINF1) [MIM:143880]
CYP24A1	Q07973	VAR_066409	p.Arg159Gln	LP/P	rs387907322	Hypercalcemia, infantile, 1 (HCINF1) [MIM:143880]
CYP24A1	Q07973	VAR_066410	p.Glu322Lys	LP/P	rs387907324	Hypercalcemia, infantile, 1 (HCINF1) [MIM:143880]
CYP24A1	Q07973	VAR_066411	p.Arg396Trp	LP/P	rs114368325	Hypercalcemia, infantile, 1 (HCINF1) [MIM:143880]
CYP26B1	Q9NR63	VAR_024383	p.Leu264Ser	LB/B	rs2241057	-
CYP26B1	Q9NR63	VAR_038722	p.Val181Met	LB/B	rs142999899	-
CYP26B1	Q9NR63	VAR_038723	p.Ala185Val	LB/B	rs765423228	-
CYP26B1	Q9NR63	VAR_038724	p.Arg191His	LB/B	rs76025186	-
CYP26B1	Q9NR63	VAR_038725	p.Asp227Asn	LB/B	rs143738797	-
CYP26B1	Q9NR63	VAR_038726	p.Glu380Lys	LB/B	rs2286965	-
CYP26B1	Q9NR63	VAR_038727	p.Ala420Gly	LB/B	rs7568553	-
CYP26B1	Q9NR63	VAR_038728	p.Arg473Cys	LB/B	rs61751056	-
CYP26B1	Q9NR63	VAR_038729	p.Val479Ile	LB/B	rs148075682	-
CYP26B1	Q9NR63	VAR_067923	p.Ser146Pro	LP/P	rs281875232	Radiohumeral fusions with other skeletal and craniofacial anomalies (RHFCA) [MIM:614416]
CYP26B1	Q9NR63	VAR_067924	p.Arg363Leu	LP/P	rs281875231	Radiohumeral fusions with other skeletal and craniofacial anomalies (RHFCA) [MIM:614416]

CYP26B1 Q9NR63 VAR_075982 p.His64Arg LB/B - -
CYP26C1 Q6V0L0 VAR_022886 p.Arg245Gln LB/B rs11187265 -
CYP27A1 Q02318 VAR_001303 p.Arg395Cys LP/P rs121908096 Cerebrotendinous xanthomatosis (CTX) [MIM:213700]
CYP27A1 Q02318 VAR_001304 p.Arg479Cys LP/P rs72551322 Cerebrotendinous xanthomatosis (CTX) [MIM:213700]
CYP27A1 Q02318 VAR_012285 p.Arg395Ser LP/P rs121908096 Cerebrotendinous xanthomatosis (CTX) [MIM:213700]
CYP27A1 Q02318 VAR_012286 p.Arg405Gln LP/P rs121908099 Cerebrotendinous xanthomatosis (CTX) [MIM:213700]
CYP27A1 Q02318 VAR_012287 p.Arg474Gln LP/P rs121908097 Cerebrotendinous xanthomatosis (CTX) [MIM:213700]
CYP27A1 Q02318 VAR_012288 p.Arg474Trp LP/P rs121908098 Cerebrotendinous xanthomatosis (CTX) [MIM:213700]
CYP27A1 Q02318 VAR_016966 p.Gly145Glu LP/P rs72551313 Cerebrotendinous xanthomatosis (CTX) [MIM:213700]
CYP27A1 Q02318 VAR_048467 p.Thr175Met LB/B rs2229381 -
CYP27A1 Q02318 VAR_061046 p.Ala169Val LB/B rs59443548 -
CYP27B1 O15528 VAR_016952 p.Arg107His LP/P rs28934604 Rickets vitamin D-dependent 1A (VDDR1A) [MIM:264700]
CYP27B1 O15528 VAR_016953 p.Gly125Glu LP/P rs28934605 Rickets vitamin D-dependent 1A (VDDR1A) [MIM:264700]
CYP27B1 O15528 VAR_016954 p.Glu189Gly LP/P rs118204012 Rickets vitamin D-dependent 1A (VDDR1A) [MIM:264700]
CYP27B1 O15528 VAR_016955 p.Thr321Arg LP/P rs118204007 Rickets vitamin D-dependent 1A (VDDR1A) [MIM:264700]
CYP27B1 O15528 VAR_016956 p.Arg335Pro LP/P rs28934606 Rickets vitamin D-dependent 1A (VDDR1A) [MIM:264700]
CYP27B1 O15528 VAR_016957 p.Leu343Phe LP/P rs118204011 Rickets vitamin D-dependent 1A (VDDR1A) [MIM:264700]
CYP27B1 O15528 VAR_016958 p.Pro382Ser LP/P rs28934607 Rickets vitamin D-dependent 1A (VDDR1A) [MIM:264700]
CYP27B1 O15528 VAR_016959 p.Arg389His LP/P rs118204009 Rickets vitamin D-dependent 1A (VDDR1A) [MIM:264700]
CYP27B1 O15528 VAR_016960 p.Arg389Gly LP/P rs118204010 Rickets vitamin D-dependent 1A (VDDR1A) [MIM:264700]
CYP27B1 O15528 VAR_016961 p.Thr409Ile LP/P rs118204008 Rickets vitamin D-dependent 1A (VDDR1A) [MIM:264700]
CYP27B1 O15528 VAR_016967 p.Glu189Lys LP/P - Rickets vitamin D-dependent 1A (VDDR1A) [MIM:264700]
CYP27B1 O15528 VAR_016968 p.Arg389Cys LP/P rs118204010 Rickets vitamin D-dependent 1A (VDDR1A) [MIM:264700]
CYP27B1 O15528 VAR_016969 p.Gln65His LP/P rs868704228 Rickets vitamin D-dependent 1A (VDDR1A) [MIM:264700]
CYP27B1 O15528 VAR_016970 p.Ser323Tyr LP/P - Rickets vitamin D-dependent 1A (VDDR1A) [MIM:264700]
CYP27B1 O15528 VAR_016971 p.Arg429Pro LP/P rs568165874 Rickets vitamin D-dependent 1A (VDDR1A) [MIM:264700]

CYP27B1 O15528 VAR_016972 p.Arg453Cys LP/P rs767480544 Rickets vitamin D-dependent 1A (VDDR1A) [MIM:264700]
CYP27B1 O15528 VAR_016973 p.Val478Gly LP/P - Rickets vitamin D-dependent 1A (VDDR1A) [MIM:264700]
CYP27B1 O15528 VAR_016974 p.Pro497Arg LP/P rs1161799032 Rickets vitamin D-dependent 1A (VDDR1A) [MIM:264700]
CYP27B1 O15528 VAR_018841 p.Val166Leu LB/B rs8176344 -
CYP27C1 Q4G0S4 VAR_033120 p.Thr529Met LB/B rs35075135 -
CYP2A13 Q16696 VAR_013835 p.Arg257Cys LB/B rs8192789 -
CYP2A13 Q16696 VAR_018334 p.Arg25Gln LB/B rs8192784 -
CYP2A13 Q16696 VAR_018335 p.Arg101Gln LB/B rs148044792 -
CYP2A13 Q16696 VAR_018337 p.Asp158Glu LB/B rs112337232 -
CYP2A13 Q16696 VAR_018338 p.Phe453Tyr LB/B rs72547590 -
CYP2A13 Q16696 VAR_018339 p.Arg494Cys LB/B rs138870349 -
CYP2A13 Q16696 VAR_018356 p.Val323Leu LB/B - -
CYP2A6 P11509 VAR_001249 p.Leu160His LB/B rs1801272 -
CYP2A6 P11509 VAR_008356 p.Gly479Val LB/B rs5031017 -
CYP2A6 P11509 VAR_011577 p.Arg128Gln LB/B rs4986891 -
CYP2A6 P11509 VAR_011578 p.Ile471Thr LB/B rs5031016 -
CYP2A6 P11509 VAR_011579 p.Arg485Leu LB/B rs28399468 -
CYP2A6 P11509 VAR_018330 p.Gly5Arg LB/B rs28399434 -
CYP2A6 P11509 VAR_018331 p.Ser29Asn LB/B rs28399435 -
CYP2A6 P11509 VAR_018332 p.Lys194Glu LB/B rs199916117 -
CYP2A6 P11509 VAR_018333 p.Arg203Ser LB/B rs56256500 -
CYP2A6 P11509 VAR_018375 p.Glu419Asp LB/B rs8192730 -
CYP2A6 P11509 VAR_024711 p.Phe118Leu LB/B rs28399440 -
CYP2A6 P11509 VAR_024712 p.Ser224Pro LB/B rs28399447 -
CYP2A6 P11509 VAR_024713 p.Val365Met LB/B rs28399454 -
CYP2A6 P11509 VAR_024714 p.Asn418Asp LB/B rs28399463 -
CYP2A6 P11509 VAR_024715 p.Lys476Arg LB/B rs6413474 -
CYP2A6 P11509 VAR_048448 p.Thr294Ser LB/B rs4997557 -
CYP2A6 P11509 VAR_055033 p.Tyr392Phe LB/B rs1809810 -
CYP2A6 P11509 VAR_055034 p.Arg203Cys LB/B rs56256500 -
CYP2A6 P11509 VAR_055035 p.Val110Leu LB/B rs72549435 -
CYP2A6 P11509 VAR_055036 p.Arg128Leu LB/B rs4986891 -
CYP2A6 P11509 VAR_055037 p.Ser131Ala LB/B rs59552350 -
CYP2A6 P11509 VAR_055038 p.Asn438Tyr LB/B rs143731390 -
CYP2A6 P11509 VAR_059149 p.Val292Met LB/B rs2644906 -
CYP2A7 P20853 VAR_047815 p.Phe61Ile LB/B rs10425176 -
CYP2A7 P20853 VAR_047816 p.Cys64Arg LB/B rs10425169 -
CYP2A7 P20853 VAR_047817 p.His274Arg LB/B rs4079366 -
CYP2A7 P20853 VAR_047818 p.Ala301Gly LB/B rs2545754 -
CYP2A7 P20853 VAR_047819 p.Arg311Cys LB/B rs3869579 -
CYP2A7 P20853 VAR_047820 p.Met368Thr LB/B rs2261144 -
CYP2A7 P20853 VAR_061043 p.Asp169Glu LB/B rs4142867 -
CYP2A7 P20853 VAR_061044 p.Val479Gly LB/B rs12460590 -
CYP2B6 P20813 VAR_016924 p.Pro167Ala LB/B rs3826711 -
CYP2B6 P20813 VAR_016925 p.Gln172His LB/B rs3745274 -
CYP2B6 P20813 VAR_016926 p.Lys262Arg LB/B rs2279343 -

CYP2B6	P20813	VAR_016927	p.Arg22Cys	LB/B	rs8192709	-
CYP2B6	P20813	VAR_016928	p.Ser259Arg	LB/B	rs45482602	-
CYP2B6	P20813	VAR_016929	p.Arg487Cys	LB/B	rs3211371	-
CYP2B6	P20813	VAR_016948	p.Lys139Glu	LB/B	rs12721655	-
CYP2B6	P20813	VAR_023563	p.Gln21Leu	LB/B	rs34883432	-
CYP2B6	P20813	VAR_023564	p.Met46Val	LB/B	rs35303484	-
CYP2B6	P20813	VAR_023565	p.Gly99Glu	LB/B	rs36060847	-
CYP2B6	P20813	VAR_023566	p.Arg140Gln	LB/B	rs35773040	-
CYP2B6	P20813	VAR_023567	p.Ile391Asn	LB/B	rs35979566	-
CYP2B6	P20813	VAR_024716	p.Ile328Thr	LB/B	rs28399499	-
CYP2B6	P20813	VAR_025206	p.Thr26Ser	LB/B	rs33973337	-
CYP2B6	P20813	VAR_025207	p.Asp28Gly	LB/B	rs33980385	-
CYP2B6	P20813	VAR_025208	p.Arg29Ser	LB/B	rs33926104	-
CYP2B6	P20813	VAR_025209	p.Asn289Lys	LB/B	rs34277950	-
CYP2B6	P20813	VAR_025210	p.Thr306Ser	LB/B	rs34698757	-
CYP2B6	P20813	VAR_033819	p.Arg29Pro	LB/B	rs34284776	-
CYP2C18	P33260	VAR_001254	p.Thr385Met	LB/B	rs1126545	-
CYP2C19	P33261	VAR_001255	p.Val331Ile	LB/B	rs3758581	-
CYP2C19	P33261	VAR_008357	p.Trp120Arg	LB/B	rs41291556	-
CYP2C19	P33261	VAR_008358	p.Arg132Gln	LB/B	rs72552267	-
CYP2C19	P33261	VAR_008359	p.Arg433Trp	LB/B	rs56337013	-
CYP2C19	P33261	VAR_020123	p.Pro227Leu	LB/B	rs6413438	-
CYP2C19	P33261	VAR_021268	p.Leu17Pro	LB/B	rs55752064	-
CYP2C19	P33261	VAR_021269	p.Ile19Leu	LB/B	rs17882687	-
CYP2C19	P33261	VAR_021270	p.Glu92Asp	LB/B	rs17878459	-
CYP2C19	P33261	VAR_021271	p.Glu122Ala	LB/B	rs17885179	-
CYP2C19	P33261	VAR_021272	p.Arg144His	LB/B	rs17884712	-
CYP2C19	P33261	VAR_021273	p.Arg150His	LB/B	rs58973490	-
CYP2C19	P33261	VAR_021274	p.Arg410Cys	LB/B	rs17879685	-
CYP2C19	P33261	VAR_021275	p.Arg442Cys	LB/B	rs192154563	-
CYP2C19	P33261	VAR_024083	p.Ser51Gly	LB/B	rs1564657013	-
CYP2C19	P33261	VAR_024084	p.Ala161Pro	LB/B	rs181297724	-
CYP2C19	P33261	VAR_024085	p.Arg329His	LB/B	rs138142612	-
CYP2C19	P33261	VAR_024718	p.Met74Thr	LB/B	rs28399505	-
CYP2C19	P33261	VAR_024719	p.Phe168Leu	LB/B	rs28399510	-
CYP2C8	P10632	VAR_001250	p.Glu154Asp	LB/B	-	-
CYP2C8	P10632	VAR_001251	p.Asn193Lys	LB/B	-	-
CYP2C8	P10632	VAR_001252	p.Lys249Arg	LB/B	-	-
CYP2C8	P10632	VAR_001253	p.His411Leu	LB/B	-	-
CYP2C8	P10632	VAR_011754	p.Ile264Met	LB/B	rs1058930	-
CYP2C8	P10632	VAR_012238	p.Arg139Lys	LB/B	rs11572080	-
CYP2C8	P10632	VAR_012239	p.Ile269Phe	LB/B	rs11572103	-
CYP2C8	P10632	VAR_012240	p.Lys399Arg	LB/B	rs10509681	-
CYP2C8	P10632	VAR_016947	p.Leu390Ser	LB/B	rs72558194	-
CYP2C8	P10632	VAR_018958	p.Ile244Val	LB/B	rs11572102	-
CYP2C8	P10632	VAR_075541	p.Gly171Ser	LB/B	rs142886225	-
CYP2C8	P10632	VAR_075542	p.Arg186Gly	LB/B	rs72558195	-
CYP2C8	P10632	VAR_075543	p.Ile223Met	LB/B	-	-
CYP2C8	P10632	VAR_075544	p.Ala238Pro	LB/B	rs188934928	-

CYP2C8	P10632	VAR_075545	p.Lys247Arg	LB/B	rs769460274	-
CYP2C8	P10632	VAR_075546	p.Lys383Asn	LB/B	-	-
CYP2C9	P11712	VAR_008343	p.Arg144Cys	LB/B	rs1799853	-
CYP2C9	P11712	VAR_008344	p.Tyr358Cys	LB/B	rs1057909	-
CYP2C9	P11712	VAR_008345	p.Ile359Leu	LB/B	rs1057910	-
CYP2C9	P11712	VAR_008346	p.Gly417Asp	LB/B	-	-
CYP2C9	P11712	VAR_013515	p.Ile359Thr	LB/B	rs56165452	-
CYP2C9	P11712	VAR_013516	p.Asp360Glu	LB/B	rs28371686	-
CYP2C9	P11712	VAR_018862	p.Leu19Ile	LB/B	rs67807361	-
CYP2C9	P11712	VAR_018863	p.Arg150His	LB/B	rs7900194	-
CYP2C9	P11712	VAR_018864	p.His251Arg	LB/B	rs2256871	-
CYP2C9	P11712	VAR_018865	p.Glu272Gly	LB/B	rs9332130	-
CYP2C9	P11712	VAR_018866	p.Arg335Trp	LB/B	rs28371685	-
CYP2C9	P11712	VAR_018867	p.Pro489Ser	LB/B	rs9332239	-
CYP2C9	P11712	VAR_024717	p.Leu413Pro	LB/B	rs28371687	-
CYP2C9	P11712	VAR_075286	p.Arg125His	LB/B	rs72558189	-
CYP2C9	P11712	VAR_075287	p.Arg125Leu	LB/B	rs72558189	-
CYP2C9	P11712	VAR_075288	p.Asn204His	LB/B	-	-
CYP2C9	P11712	VAR_075289	p.Ile434Phe	LB/B	-	-
CYP2D6	P10635	VAR_001256	p.Gly42Arg	LB/B	rs5030862	-
CYP2D6	P10635	VAR_008336	p.Pro34Ser	LB/B	rs1065852	-
CYP2D6	P10635	VAR_008337	p.Thr107Ile	LB/B	rs28371706	-
CYP2D6	P10635	VAR_008338	p.Gly169Arg	LB/B	rs5030865	-
CYP2D6	P10635	VAR_008339	p.Gly212Glu	LB/B	rs5030866	-
CYP2D6	P10635	VAR_008340	p.Arg296Cys	LB/B	rs16947	-
CYP2D6	P10635	VAR_008341	p.Ser486Thr	LB/B	rs1135840	-
CYP2D6	P10635	VAR_008348	p.His324Pro	LB/B	rs5030867	-
CYP2D6	P10635	VAR_008366	p.Val11Met	LB/B	rs769258	-
CYP2D6	P10635	VAR_008367	p.Arg26His	LB/B	rs28371696	-
CYP2D6	P10635	VAR_008368	p.Arg28Cys	LB/B	rs138100349	-
CYP2D6	P10635	VAR_008369	p.Ala85Val	LB/B	rs267608310	-
CYP2D6	P10635	VAR_008370	p.Ala237Ser	LB/B	rs28371717	-
CYP2D6	P10635	VAR_008371	p.Ile297Leu	LB/B	rs949717872	-
CYP2D6	P10635	VAR_008372	p.Arg343Gly	LB/B	rs267608295	-
CYP2D6	P10635	VAR_008373	p.Ile369Thr	LB/B	-	-
CYP2D6	P10635	VAR_008374	p.Glu410Lys	LB/B	rs769157652	-
CYP2D6	P10635	VAR_014633	p.Ser311Leu	LB/B	rs1800754	-
CYP2D6	P10635	VAR_024720	p.Leu91Met	LB/B	rs28371703	-
CYP2D6	P10635	VAR_024721	p.His94Arg	LB/B	rs28371704	-
CYP2D6	P10635	VAR_024722	p.Phe120Ile	LB/B	rs1135822	-
CYP2D6	P10635	VAR_024723	p.Glu155Lys	LB/B	rs28371710	-
CYP2D6	P10635	VAR_024724	p.Glu418Lys	LB/B	rs28371733	-
CYP2D6	P10635	VAR_024725	p.Pro469Ala	LB/B	rs1135833	-
CYP2D6	P10635	VAR_024726	p.His478Tyr	LB/B	rs28371735	-
CYP2D6	P10635	VAR_045679	p.Leu231Pro	LB/B	rs17002853	-
CYP2D6	P10635	VAR_045680	p.Ala300Gly	LB/B	rs1058170	-
CYP2D6	P10635	VAR_045681	p.Arg365His	LB/B	rs1058172	-
CYP2D6	P10635	VAR_059150	p.Arg329Leu	LB/B	rs3915951	-
CYP2D6	P10635	VAR_059151	p.Gly373Ser	LB/B	rs61737946	-

CYP2D6 P10635 VAR_072764 p.Ala5Val LB/B rs773790593 -
CYP2D6 P10635 VAR_072765 p.Arg25Gln LB/B rs138417770 -
CYP2D6 P10635 VAR_072766 p.Val104Ala LB/B rs76187628 -
CYP2D6 P10635 VAR_072767 p.Leu142Ser LB/B rs375135093 -
CYP2D6 P10635 VAR_072768 p.Lys147Arg LB/B rs569229126 -
CYP2D6 P10635 VAR_072769 p.Cys161Ser LB/B - -
CYP2D6 P10635 VAR_072770 p.Phe164Leu LB/B - -
CYP2D6 P10635 VAR_072771 p.Glu215Lys LB/B rs567606867 -
CYP2D6 P10635 VAR_072772 p.Phe219Ser LB/B rs371793722 -
CYP2D6 P10635 VAR_072773 p.Thr249Pro LB/B - -
CYP2D6 P10635 VAR_072774 p.Val327Met LB/B - -
CYP2D6 P10635 VAR_072775 p.Asp336Asn LB/B rs745746329 -
CYP2D6 P10635 VAR_072776 p.Asp337Gly LB/B rs748712690 -
CYP2D6 P10635 VAR_072777 p.Val342Met LB/B rs750996195 -
CYP2D6 P10635 VAR_072778 p.Arg344Gln LB/B rs76088846 -
CYP2D6 P10635 VAR_072779 p.Arg440Cys LB/B rs777560972 -
CYP2D6 P10635 VAR_072780 p.Phe457Leu LB/B - -
CYP2D6 P10635 VAR_072781 p.His463Asp LB/B - -
CYP2D6 P10635 VAR_072782 p.Arg497Cys LB/B rs370580423 -
CYP2D7 A0A087X1C5 VAR_072632 p.Ser70Asn LB/B rs11090077 -
CYP2D7 A0A087X1C5 VAR_072633 p.Ser311Leu LB/B rs1800754 -
CYP2D7 A0A087X1C5 VAR_072636 p.His383Arg LB/B rs56127449 -
CYP2D7 A0A087X1C5 VAR_072637 p.Lys428Glu LB/B rs2070907 -
CYP2E1 P05181 VAR_008360 p.Arg76His LB/B rs72559710 -
CYP2E1 P05181 VAR_008361 p.Val179Ile LB/B rs6413419 -
CYP2E1 P05181 VAR_008362 p.Val389Ile LB/B rs55897648 -
CYP2E1 P05181 VAR_024727 p.His457Leu LB/B rs28969387 -
CYP2E1 P05181 VAR_055382 p.Asn219Asp LB/B rs41299426 -
CYP2E1 P05181 VAR_055383 p.Ser366Cys LB/B rs41299434 -
CYP2F1 P24903 VAR_058863 p.Ser38Pro LB/B rs58285195 -
CYP2F1 P24903 VAR_058864 p.Arg98Pro LB/B rs57670668 -
CYP2F1 P24903 VAR_058865 p.Asp218Asn LB/B rs305974 -
CYP2F1 P24903 VAR_058866 p.Gln266His LB/B rs75405062 -
CYP2F1 P24903 VAR_058867 p.Leu391Pro LB/B rs144315434 -
CYP2F1 P24903 VAR_058868 p.Pro490Leu LB/B rs7246981 -
CYP2J2 P51589 VAR_014317 p.Thr143Ala LB/B rs55753213 -
CYP2J2 P51589 VAR_014318 p.Arg158Cys LB/B rs56307989 -
CYP2J2 P51589 VAR_014319 p.Ile192Asn LB/B rs66515830 -
CYP2J2 P51589 VAR_014320 p.Asp342Asn LB/B rs56053398 -
CYP2J2 P51589 VAR_014321 p.Asn404Tyr LB/B rs72547598 -
CYP2J2 P51589 VAR_022084 p.Asn124Ser LB/B rs2228113 -
CYP2J2 P51589 VAR_029159 p.Arg49Ser LB/B rs11572190 -
CYP2J2 P51589 VAR_029160 p.Val113Met LB/B rs11572242 -
CYP2R1 Q6VVX0 VAR_021534 p.Leu99Pro LP/P rs61495246 Rickets vitamin D-dependent 1B (VDDR1B) [MIM:600081]
CYP2R1 Q6VVX0 VAR_075532 p.Lys242Asn US - -
CYP2S1 Q96SQ9 VAR_033820 p.Pro466Leu LB/B rs34971233 -
CYP2U1 Q7Z449 VAR_069575 p.Cys262Arg LP/P rs397514515 Spastic paraplegia 56, autosomal recessive (SPG56) [MIM:615030]

CYP2U1	Q7Z449	VAR_069576	p.Asp316Val	LP/P	rs397514513	Spastic paraplegia 56, autosomal recessive (SPG56) [MIM:615030]
CYP2U1	Q7Z449	VAR_069577	p.Glu380Gly	LP/P	rs397514514	Spastic paraplegia 56, autosomal recessive (SPG56) [MIM:615030]
CYP2U1	Q7Z449	VAR_069578	p.Arg488Trp	LP/P	rs141431913	Spastic paraplegia 56, autosomal recessive (SPG56) [MIM:615030]
CYP2W1	Q8TAV3	VAR_027413	p.Ala181Thr	LB/B	rs3735684	-
CYP2W1	Q8TAV3	VAR_071912	p.Glu58Ala	LB/B	rs1316523256	-
CYP2W1	Q8TAV3	VAR_071913	p.Val432Ile	LB/B	rs78873069	-
CYP2W1	Q8TAV3	VAR_071914	p.Gln482His	LB/B	rs773499447	-
CYP2W1	Q8TAV3	VAR_071915	p.Pro488Leu	LB/B	rs3808348	-
CYP39A1	Q9NYL5	VAR_031609	p.Arg23Pro	LB/B	rs12192544	-
CYP39A1	Q9NYL5	VAR_031610	p.Arg103His	LB/B	rs2277119	-
CYP39A1	Q9NYL5	VAR_031611	p.Tyr288His	LB/B	rs17856332	-
CYP39A1	Q9NYL5	VAR_031612	p.Asn324Lys	LB/B	rs7761731	-
CYP39A1	Q9NYL5	VAR_083092	p.Lys329Gln	LB/B	rs41273654	-
CYP3A4	P08684	VAR_008363	p.Ser222Pro	LB/B	rs55785340	-
CYP3A4	P08684	VAR_008364	p.Met445Thr	LB/B	rs4986910	-
CYP3A4	P08684	VAR_011597	p.Leu15Pro	LB/B	rs12721634	-
CYP3A4	P08684	VAR_011598	p.Gly56Asp	LB/B	rs56324128	-
CYP3A4	P08684	VAR_011599	p.Ile118Val	LB/B	rs55951658	-
CYP3A4	P08684	VAR_011600	p.Arg130Gln	LB/B	rs72552799	-
CYP3A4	P08684	VAR_011601	p.Arg162Gln	LB/B	rs4986907	-
CYP3A4	P08684	VAR_011602	p.Val170Ile	LB/B	rs72552798	-
CYP3A4	P08684	VAR_011603	p.Asp174His	LB/B	rs4986908	-
CYP3A4	P08684	VAR_011604	p.Thr185Ser	LB/B	rs12721627	-
CYP3A4	P08684	VAR_011605	p.Pro218Arg	LB/B	rs55901263	-
CYP3A4	P08684	VAR_011606	p.Thr363Met	LB/B	rs67784355	-
CYP3A4	P08684	VAR_011607	p.Leu373Phe	LB/B	rs12721629	-
CYP3A4	P08684	VAR_011608	p.Pro416Leu	LB/B	rs4986909	-
CYP3A4	P08684	VAR_014322	p.Phe189Ser	LB/B	rs4987161	-
CYP3A4	P08684	VAR_014323	p.Leu293Pro	LB/B	rs28371759	-
CYP3A4	P08684	VAR_014324	p.Pro467Ser	LB/B	rs4986913	-
CYP3A4	P08684	VAR_037547	p.Lys96Glu	LB/B	rs3091339	-
CYP3A4	P08684	VAR_037548	p.Ser252Ala	LB/B	rs3208363	-
CYP3A4	P08684	VAR_037549	p.Thr349Asn	LB/B	rs10250778	-
CYP3A4	P08684	VAR_037550	p.Ile431Thr	LB/B	rs1041988	-
CYP3A4	P08684	VAR_084337	p.Ile301Thr	LP/P	-	Vitamin D-dependent rickets 3 (VDDR3) [MIM:619073]
CYP3A43	Q9HB55	VAR_018052	p.Pro340Ala	LB/B	rs680055	-
CYP3A43	Q9HB55	VAR_048449	p.Thr27Ala	LB/B	rs45558032	-
CYP3A43	Q9HB55	VAR_048450	p.Met145Ile	LB/B	rs45450092	-
CYP3A43	Q9HB55	VAR_048451	p.Met275Ile	LB/B	rs45621431	-
CYP3A5	P20815	VAR_008365	p.Thr398Asn	LB/B	rs28365083	-
CYP3A5	P20815	VAR_024728	p.His30Tyr	LB/B	rs28383468	-
CYP3A5	P20815	VAR_024729	p.Asp277Glu	LB/B	rs28383477	-
CYP3A5	P20815	VAR_024730	p.Ala337Thr	LB/B	rs28383479	-
CYP3A5	P20815	VAR_024731	p.Arg28Cys	LB/B	rs55817950	-
CYP3A5	P20815	VAR_024732	p.Gln200Arg	LB/B	rs56411402	-
CYP3A5	P20815	VAR_024733	p.Phe446Ser	LB/B	rs41279854	-

CYP3A5	P20815	VAR_029161	p.Ile371Val	LB/B	rs28365092	-
CYP3A5	P20815	VAR_029162	p.Ile488Thr	LB/B	rs28365085	-
CYP3A7	P24462	VAR_020124	p.Thr409Arg	LB/B	rs2257401	-
CYP3A7	P24462	VAR_055564	p.Val71Ala	LB/B	rs45580339	-
CYP4A11	Q02928	VAR_019160	p.Phe434Ser	LB/B	rs1126742	-
CYP4A11	Q02928	VAR_044377	p.Ser353Gly	LB/B	rs3899049	-
CYP4A11	Q02928	VAR_048452	p.Asn226Ser	LB/B	rs12759923	-
CYP4A22	Q5TCH4	VAR_044349	p.Arg11Cys	LB/B	rs76011927	-
CYP4A22	Q5TCH4	VAR_044350	p.Lys121Arg	LB/B	rs2758717	-
CYP4A22	Q5TCH4	VAR_044351	p.Arg126Trp	LB/B	rs12564525	-
CYP4A22	Q5TCH4	VAR_044352	p.Gly130Ser	LB/B	rs2056900	-
CYP4A22	Q5TCH4	VAR_044353	p.Asn152Tyr	LB/B	rs2056899	-
CYP4A22	Q5TCH4	VAR_044354	p.Val185Phe	LB/B	rs4926581	-
CYP4A22	Q5TCH4	VAR_044355	p.Ser226Asn	LB/B	rs35202523	-
CYP4A22	Q5TCH4	VAR_044356	p.Cys230Ser	LB/B	rs35156123	-
CYP4A22	Q5TCH4	VAR_044357	p.Cys231Arg	LB/B	rs10789501	-
CYP4A22	Q5TCH4	VAR_044358	p.Lys276Thr	LB/B	rs6661132	-
CYP4A22	Q5TCH4	VAR_044359	p.Leu428Pro	LB/B	rs2405599	-
CYP4A22	Q5TCH4	VAR_044360	p.Met491Ile	LB/B	rs2758714	-
CYP4A22	Q5TCH4	VAR_044361	p.Leu509Phe	LB/B	rs4926600	-
CYP4A22	Q5TCH4	VAR_061045	p.Tyr104Phe	LB/B	rs61507155	-
CYP4B1	P13584	VAR_018357	p.Arg173Trp	LB/B	rs4646487	-
CYP4B1	P13584	VAR_018358	p.Ser322Gly	LB/B	rs45467195	-
CYP4B1	P13584	VAR_018359	p.Met331Ile	LB/B	rs2297810	-
CYP4B1	P13584	VAR_018360	p.Arg340Cys	LB/B	rs4646491	-
CYP4B1	P13584	VAR_018361	p.Val345Ile	LB/B	rs1557501779	-
CYP4B1	P13584	VAR_018362	p.Arg375Cys	LB/B	rs2297809	-
CYP4B1	P13584	VAR_048453	p.Arg264Trp	LB/B	rs45446505	-
CYP4B1	P13584	VAR_048454	p.Tyr329Ser	LB/B	rs12094024	-
CYP4B1	P13584	VAR_048455	p.Phe354Cys	LB/B	rs17102592	-
CYP4B1	P13584	VAR_048456	p.Arg482Gln	LB/B	rs45622937	-
CYP4B1	P13584	VAR_055377	p.Ala111Val	LB/B	rs45559437	-
CYP4B1	P13584	VAR_055378	p.Arg274Gln	LB/B	rs45578838	-
CYP4F11	Q9HBI6	VAR_060265	p.Arg146Cys	LB/B	rs57519667	-
CYP4F11	Q9HBI6	VAR_060266	p.Cys276Arg	LB/B	rs8104361	-
CYP4F11	Q9HBI6	VAR_071198	p.Asp446Asn	LB/B	rs1060463	-
CYP4F12	Q9HCS2	VAR_013244	p.Pro13Leu	LB/B	rs16995376	-
CYP4F12	Q9HCS2	VAR_013245	p.Asn76Asp	LB/B	rs609636	-
CYP4F12	Q9HCS2	VAR_013246	p.Val90Ile	LB/B	rs609290	-
CYP4F12	Q9HCS2	VAR_013247	p.Arg188Cys	LB/B	rs2285888	-
CYP4F12	Q9HCS2	VAR_048459	p.Thr16Met	LB/B	rs16995378	-
CYP4F12	Q9HCS2	VAR_048460	p.Ser522Gly	LB/B	rs593818	-
CYP4F2	P78329	VAR_013116	p.Ser7Tyr	LB/B	rs3093104	-
CYP4F2	P78329	VAR_013117	p.Trp12Gly	LB/B	rs3093105	-
CYP4F2	P78329	VAR_013118	p.Gly185Val	LB/B	rs3093153	-
CYP4F2	P78329	VAR_013119	p.Val433Met	LB/B	rs2108622	-
CYP4F2	P78329	VAR_013120	p.Leu519Met	LB/B	rs3093200	-
CYP4F2	P78329	VAR_020125	p.Ala269Asp	LB/B	rs1805040	-
CYP4F22	Q6NT55	VAR_033118	p.Ser178Cys	LB/B	rs16980531	-

CYP4F22 Q6NT55 VAR_033119 p.Lys505Gln LB/B rs7256787 -
CYP4F22 Q6NT55 VAR_037441 p.Phe59Leu LP/P rs118091316 Ichthyosis, congenital, autosomal recessive 5 (ARCI5) [MIM:604777]
CYP4F22 Q6NT55 VAR_037442 p.Arg243His LP/P rs118203937 Ichthyosis, congenital, autosomal recessive 5 (ARCI5) [MIM:604777]
CYP4F22 Q6NT55 VAR_037443 p.Arg372Trp LP/P rs201129618 Ichthyosis, congenital, autosomal recessive 5 (ARCI5) [MIM:604777]
CYP4F22 Q6NT55 VAR_037444 p.His435Tyr LP/P rs118203935 Ichthyosis, congenital, autosomal recessive 5 (ARCI5) [MIM:604777]
CYP4F22 Q6NT55 VAR_037445 p.His436Asp LP/P rs118203936 Ichthyosis, congenital, autosomal recessive 5 (ARCI5) [MIM:604777]
CYP4F3 Q08477 VAR_001258 p.Ala269Asp LB/B rs1805040 -
CYP4F3 Q08477 VAR_020664 p.Val270Ile LB/B rs28371536 -
CYP4F3 Q08477 VAR_020665 p.Ile271Thr LB/B rs28371479 -
CYP4F3 Q08477 VAR_048457 p.His96Gln LB/B rs34923393 -
CYP4F3 Q08477 VAR_048458 p.Tyr106Cys LB/B rs35888783 -
CYP4F8 P98187 VAR_038347 p.Tyr125Phe LB/B rs2072600 -
CYP4F8 P98187 VAR_038348 p.Pro447Gln LB/B rs2056822 -
CYP4V2 Q6ZWL3 VAR_023084 p.Trp44Arg LP/P rs119103282 Bietti crystalline corneoretinal dystrophy (BCD) [MIM:210370]
CYP4V2 Q6ZWL3 VAR_023085 p.Gly61Ser LP/P rs119103285 Bietti crystalline corneoretinal dystrophy (BCD) [MIM:210370]
CYP4V2 Q6ZWL3 VAR_023086 p.Glu79Asp LP/P rs199476185 Bietti crystalline corneoretinal dystrophy (BCD) [MIM:210370]
CYP4V2 Q6ZWL3 VAR_023087 p.Ile111Thr LP/P rs119103283 Bietti crystalline corneoretinal dystrophy (BCD) [MIM:210370]
CYP4V2 Q6ZWL3 VAR_023088 p.Met123Val LP/P rs149684063 Bietti crystalline corneoretinal dystrophy (BCD) [MIM:210370]
CYP4V2 Q6ZWL3 VAR_023089 p.His331Pro LP/P rs199476197 Bietti crystalline corneoretinal dystrophy (BCD) [MIM:210370]
CYP4V2 Q6ZWL3 VAR_023090 p.Ser341Pro LP/P rs199476199 Bietti crystalline corneoretinal dystrophy (BCD) [MIM:210370]
CYP4V2 Q6ZWL3 VAR_023091 p.Arg508His LP/P rs119103284 Bietti crystalline corneoretinal dystrophy (BCD) [MIM:210370]
CYP4V2 Q6ZWL3 VAR_033821 p.Gln259Lys LB/B rs13146272 -
CYP4V2 Q6ZWL3 VAR_038606 p.Leu22Val LB/B rs1055138 -
CYP4V2 Q6ZWL3 VAR_038607 p.Ser213Asn LB/B rs34331648 -
CYP4V2 Q6ZWL3 VAR_055379 p.Glu275Lys LB/B rs34745240 -
CYP4V2 Q6ZWL3 VAR_055380 p.Val372Ile LB/B rs61755911 -
CYP4V2 Q6ZWL3 VAR_055381 p.Arg443Gln LB/B rs72646291 -
CYP4Z1 Q86W10 VAR_048461 p.Pro393Leu LB/B rs28463559 -
CYP51A1 Q16850 VAR_023470 p.Val13Ala LB/B rs2229188 -
CYP7A1 P22680 VAR_001259 p.Phe100Ser LB/B - -
CYP7A1 P22680 VAR_018376 p.Asn233Ser LB/B rs8192874 -
CYP7A1 P22680 VAR_018377 p.Asp347Asn LB/B rs8192875 -
CYP7A1 P22680 VAR_059152 p.His86Asn LB/B rs62621283 -
CYP7B1 O75881 VAR_044382 p.Gly57Arg LP/P rs121908614 Spastic paraplegia 5A, autosomal recessive (SPG5A) [MIM:270800]

CYP7B1 O75881 VAR_044383 p.Phe216Ser LP/P rs121908612 Spastic paraplegia 5A, autosomal recessive (SPG5A) [MIM:270800]
CYP7B1 O75881 VAR_044384 p.Ser363Phe LP/P rs121908610 Spastic paraplegia 5A, autosomal recessive (SPG5A) [MIM:270800]
CYP7B1 O75881 VAR_044385 p.Arg417His LP/P rs121908611 Spastic paraplegia 5A, autosomal recessive (SPG5A) [MIM:270800]
CYP7B1 O75881 VAR_075505 p.Leu19Pro LB/B rs72554624 -
CYP7B1 O75881 VAR_075506 p.Gly87Val LP/P rs587777221 Spastic paraplegia 5A, autosomal recessive (SPG5A) [MIM:270800]
CYP7B1 O75881 VAR_075507 p.His106Tyr LB/B - -
CYP7B1 O75881 VAR_075508 p.Gly147Asp US rs754730601 Spastic paraplegia 5A, autosomal recessive (SPG5A) [MIM:270800]
CYP7B1 O75881 VAR_075509 p.Gly198Arg US - Spastic paraplegia 5A, autosomal recessive (SPG5A) [MIM:270800]
CYP7B1 O75881 VAR_075510 p.His285Leu LP/P rs750781606 Spastic paraplegia 5A, autosomal recessive (SPG5A) [MIM:270800]
CYP7B1 O75881 VAR_075511 p.Leu287Ser LB/B - -
CYP7B1 O75881 VAR_075512 p.Thr297Ala LP/P rs587777222 Spastic paraplegia 5A, autosomal recessive (SPG5A) [MIM:270800]
CYP7B1 O75881 VAR_075514 p.Arg324His LB/B rs59035258 -
CYP7B1 O75881 VAR_075515 p.Arg417Cys LP/P rs367916692 Spastic paraplegia 5A, autosomal recessive (SPG5A) [MIM:270800]
CYP7B1 O75881 VAR_075516 p.Gly443Ala LP/P rs1190562443 Spastic paraplegia 5A, autosomal recessive (SPG5A) [MIM:270800]
CYP7B1 O75881 VAR_075517 p.Phe470Ile LP/P rs267606758 Spastic paraplegia 5A, autosomal recessive (SPG5A) [MIM:270800]
CYP7B1 O75881 VAR_075518 p.Arg486Cys LP/P rs116171274 Spastic paraplegia 5A, autosomal recessive (SPG5A) [MIM:270800]
CYP8B1 Q9UNU6 VAR_010381 p.Arg234His LB/B rs199955644 -
CYP8B1 Q9UNU6 VAR_055102 p.Ser88Pro LB/B rs9865715 -
CYP8B1 Q9UNU6 VAR_055103 p.Lys238Arg LB/B rs35764459 -
CYP8B1 Q9UNU6 VAR_055104 p.Leu357Phe LB/B rs35637877 -
CYREN Q9BWK5 VAR_039320 p.Pro82Leu US rs776124276 A colorectal cancer sample
CYRIB Q9NUQ9 VAR_019704 p.Asn169Lys LB/B - -
CYSTM1 Q9H1C7 VAR_034629 p.Cys90Ser LB/B rs17852164 -
CYTH4 Q9UIA0 VAR_051920 p.Met74Val LB/B rs16998061 -
CYTIP O60759 VAR_023534 p.Asp37Asn LB/B rs1042038 -
CYTIP O60759 VAR_051287 p.Gln83Glu LB/B rs2229345 -
CYTIP O60759 VAR_064706 p.Pro329Ser US - -
CYTL1 Q9NRR1 VAR_050940 p.Ser51Leu LB/B rs35755546 -
CYTL1 Q9NRR1 VAR_050941 p.Arg136Cys LB/B rs11722554 -
CYYR1 Q96J86 VAR_044042 p.Arg95His LB/B rs35253087 -
CYYR1 Q96J86 VAR_044043 p.Pro111Ser LB/B rs1215339708 -
D2HGDH Q8N465 VAR_025889 p.Arg15Gly LB/B rs4675887 -
D2HGDH Q8N465 VAR_025890 p.Ile147Ser LP/P rs121434361 D-2-hydroxyglutaric aciduria 1 (D2HGA1) [MIM:600721]
D2HGDH Q8N465 VAR_025891 p.Asp375Tyr LP/P rs267606759 D-2-hydroxyglutaric aciduria 1 (D2HGA1) [MIM:600721]
D2HGDH Q8N465 VAR_025892 p.Gly436Val LB/B - -

D2HGDH Q8N465 VAR_025893 p.Asn439Asp LP/P rs121434362 D-2-hydroxyglutaric aciduria 1 (D2HGA1) [MIM:600721]
D2HGDH Q8N465 VAR_025894 p.Val444Ala LP/P rs121434360 D-2-hydroxyglutaric aciduria 1 (D2HGA1) [MIM:600721]
D2HGDH Q8N465 VAR_050433 p.Val338Ile LB/B rs1106639 -
D2HGDH Q8N465 VAR_050434 p.Ala361Val LB/B rs1105273 -
DAAM2 Q86T65 VAR_055805 p.Arg105His LB/B rs6919807 -
DAAM2 Q86T65 VAR_055806 p.Arg617His LB/B rs34699846 -
DAB1 O75553 VAR_056857 p.Val71Ile LB/B rs1855377 -
DAB2 P98082 VAR_031705 p.Thr586Ile LB/B rs700241 -
DAB2 P98082 VAR_050942 p.Ser634Asn LB/B rs3733801 -
DAB2IP Q5VWQ8 VAR_056858 p.Ser59Phe LB/B rs7027492 -
DACT1 Q9NYF0 VAR_036461 p.Gly124Cys US - A colorectal cancer sample
DACT1 Q9NYF0 VAR_036462 p.Ser682Leu US rs1198900887 A colorectal cancer sample
DACT1 Q9NYF0 VAR_053057 p.Asp446Asn LB/B rs34015825 -
DACT1 Q9NYF0 VAR_053058 p.Ala464Val LB/B rs17832998 -
DACT1 Q9NYF0 VAR_053059 p.Ser628Ala LB/B rs17094821 -
DACT1 Q9NYF0 VAR_053060 p.Gly697Ser LB/B rs698025 -
DACT1 Q9NYF0 VAR_068427 p.Arg45Trp US rs778976254 Neural tube defects (NTD) [MIM:182940]
DACT1 Q9NYF0 VAR_068428 p.Asp142Gly US - -
DACT1 Q9NYF0 VAR_068429 p.Asn356Lys LP/P - Neural tube defects (NTD) [MIM:182940]
DACT1 Q9NYF0 VAR_068430 p.Val702Gly US rs1028180302 -
DACT1 Q9NYF0 VAR_068431 p.Asp800Gly LB/B rs773720154 -
DACT1 Q9NYF0 VAR_068432 p.Thr808Lys US - -
DACT2 Q5SW24 VAR_059969 p.Glu351Gly LB/B rs6925614 -
DACT2 Q5SW24 VAR_059970 p.Thr541Pro LB/B rs10945501 -
DAD1 P61803 VAR_018825 p.Ala83Thr LB/B rs5742796 -
DAG1 Q14118 VAR_024335 p.Ser14Trp LB/B rs2131107 -
DAG1 Q14118 VAR_065266 p.Thr192Met LP/P rs193922955 Muscular dystrophy-dystroglycanopathy limb-girdle C9 (MDDGC9) [MIM:613818]
DAG1 Q14118 VAR_075809 p.Val74Ile LP/P rs189360006 Muscular dystrophy-dystroglycanopathy limb-girdle C9 (MDDGC9) [MIM:613818]
DAG1 Q14118 VAR_075810 p.Asp111Asn LP/P rs117209107 Muscular dystrophy-dystroglycanopathy limb-girdle C9 (MDDGC9) [MIM:613818]
DAG1 Q14118 VAR_075811 p.Cys669Phe LP/P rs797045023 Muscular dystrophy-dystroglycanopathy congenital with brain and eye anomalies A9 (MDDGA9) [MIM:616538]
DAGLA Q9Y4D2 VAR_027274 p.Pro889Leu LB/B rs3741252 -
DAGLA Q9Y4D2 VAR_049822 p.Gly735Val LB/B rs35056845 -
DAGLA Q9Y4D2 VAR_049823 p.Asp945Glu LB/B rs34956386 -
DAGLB Q8NCG7 VAR_027275 p.Gln664Arg LB/B rs2303361 -
DALRD3 Q5D0E6 VAR_038349 p.Gln299Arg LB/B rs3087866 -
DAOA P59103 VAR_014313 p.Arg30Lys LB/B rs2391191 -
DAOA P59103 VAR_050943 p.Lys62Glu LB/B rs9558562 -
DAP3 P51398 VAR_061811 p.Val240Phe LB/B rs57692591 -
DAPK1 P53355 VAR_033235 p.Val416Ile LB/B rs12343465 -
DAPK1 P53355 VAR_040420 p.Ala461Ser LB/B - -
DAPK1 P53355 VAR_040421 p.Ser519Ala LB/B - -
DAPK1 P53355 VAR_040422 p.Cys540Tyr LB/B rs56327474 -
DAPK1 P53355 VAR_040423 p.Met941Thr LB/B - -

DAPK1 P53355 VAR_040424 p.Arg977Trp LB/B - -
DAPK1 P53355 VAR_040425 p.Lys978Asn LB/B - -
DAPK1 P53355 VAR_040426 p.Tyr993Cys LB/B - -
DAPK1 P53355 VAR_040427 p.Asp994Glu LB/B - -
DAPK1 P53355 VAR_040428 p.Glu1005Gln LB/B - -
DAPK1 P53355 VAR_040429 p.Asp1007Tyr LB/B - -
DAPK1 P53355 VAR_040430 p.Leu1008Pro LB/B - -
DAPK1 P53355 VAR_040431 p.Arg1010Cys LB/B rs371784492 -
DAPK1 P53355 VAR_040432 p.Thr1018Ala LB/B - -
DAPK1 P53355 VAR_040433 p.Met1272Ile LB/B rs56169226 -
DAPK1 P53355 VAR_040434 p.Ser1346Asn LB/B rs1056719 -
DAPK1 P53355 VAR_040435 p.Gly1405Val LB/B rs36220450 -
DAPK1 P53355 VAR_060693 p.Pro591Leu LB/B rs36214022 -
DAPK1 P53355 VAR_060694 p.Ile622Met LB/B rs36215047 -
DAPK2 Q9UIK4 VAR_040436 p.Arg60Trp LB/B rs56047843 -
DAPK2 Q9UIK4 VAR_040437 p.Arg271Trp LB/B rs34270163 -
DAPK3 O43293 VAR_040438 p.Thr112Met US - A colorectal adenocarcinoma sample
DAPK3 O43293 VAR_040439 p.Asp161Asn US - An ovarian mucinous carcinoma sample
DAPK3 O43293 VAR_040440 p.Pro216Ser US - A lung neuroendocrine carcinoma sample
DAPL1 A0PJW8 VAR_038402 p.Leu60Pro LB/B rs9869 -
DAPL1 A0PJW8 VAR_038403 p.Ala66Thr LB/B rs12535 -
DARS1 P14868 VAR_027611 p.Leu426Phe LB/B rs1803165 -
DARS1 P14868 VAR_070038 p.Met256Leu LP/P rs886037635 Hypomyelination with brainstem and spinal cord involvement and leg spasticity (HBSL) [MIM:615281]
DARS1 P14868 VAR_070039 p.Ala274Val LP/P rs369152939 Hypomyelination with brainstem and spinal cord involvement and leg spasticity (HBSL) [MIM:615281]
DARS1 P14868 VAR_070040 p.Asp367Tyr LP/P rs370064817 Hypomyelination with brainstem and spinal cord involvement and leg spasticity (HBSL) [MIM:615281]
DARS1 P14868 VAR_070041 p.Arg460His LP/P rs587776985 Hypomyelination with brainstem and spinal cord involvement and leg spasticity (HBSL) [MIM:615281]
DARS1 P14868 VAR_070042 p.Pro464Leu LP/P rs148806569 Hypomyelination with brainstem and spinal cord involvement and leg spasticity (HBSL) [MIM:615281]
DARS1 P14868 VAR_070043 p.Arg487Cys LP/P rs587776984 Hypomyelination with brainstem and spinal cord involvement and leg spasticity (HBSL) [MIM:615281]
DARS1 P14868 VAR_070044 p.Arg494Cys LP/P rs147077598 Hypomyelination with brainstem and spinal cord involvement and leg spasticity (HBSL) [MIM:615281]
DARS1 P14868 VAR_070045 p.Arg494Gly LP/P rs147077598 Hypomyelination with brainstem and spinal cord involvement and leg spasticity (HBSL) [MIM:615281]
DARS2 Q6PI48 VAR_027612 p.Leu10Val LB/B rs4427454 -
DARS2 Q6PI48 VAR_034525 p.Lys196Arg LB/B rs35515638 -
DARS2 Q6PI48 VAR_037015 p.Ser45Gly LP/P rs121918209 Leukoencephalopathy with brainstem and spinal cord involvement and lactate elevation (LBSL) [MIM:611105]
DARS2 Q6PI48 VAR_037016 p.Cys152Phe LP/P rs121918208 Leukoencephalopathy with brainstem and spinal cord involvement and lactate elevation (LBSL) [MIM:611105]
DARS2 Q6PI48 VAR_037017 p.Arg179His LP/P rs121918210 Leukoencephalopathy with brainstem and spinal cord involvement and lactate elevation (LBSL) [MIM:611105]
DARS2 Q6PI48 VAR_037018 p.Gln184Lys LP/P rs1469160736 Leukoencephalopathy with brainstem and spinal cord involvement and lactate elevation (LBSL) [MIM:611105]

DARS2 Q6PI48 VAR_037019 p.Gln248Lys LP/P rs772489337 Leukoencephalopathy with brainstem and spinal cord involvement and lactate elevation (LBSL) [MIM:611105]
DARS2 Q6PI48 VAR_037020 p.Arg263Gln LP/P rs121918207 Leukoencephalopathy with brainstem and spinal cord involvement and lactate elevation (LBSL) [MIM:611105]
DARS2 Q6PI48 VAR_037021 p.Asp560Val LP/P rs770525873 Leukoencephalopathy with brainstem and spinal cord involvement and lactate elevation (LBSL) [MIM:611105]
DARS2 Q6PI48 VAR_037022 p.Leu613Phe LP/P rs121918212 Leukoencephalopathy with brainstem and spinal cord involvement and lactate elevation (LBSL) [MIM:611105]
DARS2 Q6PI48 VAR_037023 p.Leu626Gln LP/P rs121918213 Leukoencephalopathy with brainstem and spinal cord involvement and lactate elevation (LBSL) [MIM:611105]
DARS2 Q6PI48 VAR_037024 p.Leu626Val LP/P rs121918205 Leukoencephalopathy with brainstem and spinal cord involvement and lactate elevation (LBSL) [MIM:611105]
DARS2 Q6PI48 VAR_037025 p.Tyr629Cys LP/P rs761675657 Leukoencephalopathy with brainstem and spinal cord involvement and lactate elevation (LBSL) [MIM:611105]
DAW1 Q8N136 VAR_026853 p.Thr27Ile LB/B rs11894733 -
DAW1 Q8N136 VAR_026854 p.Thr121Ser LB/B rs1715828 -
DAW1 Q8N136 VAR_033811 p.Gly259Ser LB/B rs35027781 -
DAW1 Q8N136 VAR_033812 p.Leu317Phe LB/B rs35395984 -
DAW1 Q8N136 VAR_035891 p.Thr129Met US rs113776284 A breast cancer sample
DAZAP1 Q96EP5 VAR_035480 p.Ser381Thr US - A breast cancer sample
DAZAP2 Q15038 VAR_061639 p.Ser102Ala LB/B rs57917280 -
DAZL Q92904 VAR_017780 p.Thr12Ala LB/B rs11710967 -
DAZL Q92904 VAR_017781 p.Thr54Ala LB/B rs121918346 -
DAZL Q92904 VAR_069231 p.Arg115Gly US - -
DBF4 Q9UBU7 VAR_052970 p.Tyr112Asn LB/B rs1476703 -
DBF4 Q9UBU7 VAR_052971 p.His575Arg LB/B rs2041049 -
DBH P09172 VAR_002196 p.Ala318Ser LB/B rs4531 -
DBH P09172 VAR_013947 p.Ala211Thr LB/B rs5320 -
DBH P09172 VAR_013948 p.Arg549Cys LB/B rs6271 -
DBH P09172 VAR_014706 p.Glu181Gln LB/B rs5319 -
DBH P09172 VAR_014707 p.Lys239Asn LB/B rs5321 -
DBH P09172 VAR_014708 p.Glu250Gln LB/B rs5323 -
DBH P09172 VAR_014709 p.Asp290Asn LB/B rs5324 -
DBH P09172 VAR_014710 p.Leu317Pro LB/B rs5325 -
DBH P09172 VAR_022758 p.Val101Met LP/P rs267606760 Orthostatic hypotension 1 (ORTHYP1) [MIM:223360]
DBH P09172 VAR_022759 p.Asp114Glu LP/P rs77576840 Orthostatic hypotension 1 (ORTHYP1) [MIM:223360]
DBH P09172 VAR_022760 p.Asp345Asn LP/P rs267606761 Orthostatic hypotension 1 (ORTHYP1) [MIM:223360]
DBH P09172 VAR_048838 p.Gly12Ser LB/B rs5318 -
DBI P07108 VAR_048160 p.Asp39Asn LB/B rs8192504 -
DBI P07108 VAR_048161 p.Met71Val LB/B rs8192506 -
DBI P07108 VAR_048162 p.Gly86Arg LB/B rs8192507 -
DBN1 Q16643 VAR_035910 p.Glu278Lys US - A breast cancer sample
DBN1 Q16643 VAR_035911 p.Glu640Gln US - A breast cancer sample
DBN1 Q16643 VAR_047365 p.Ile446Val LB/B rs2544809 -
DBN1 Q16643 VAR_047366 p.Ser553Pro LB/B rs28538572 -

DBT	P11182	VAR_004978	p.Phe276Cys	LP/P	rs121964999	Maple syrup urine disease 2 (MSUD2) [MIM:248600]
DBT	P11182	VAR_015099	p.Ile98Met	LP/P	rs121965001	Maple syrup urine disease 2 (MSUD2) [MIM:248600]
DBT	P11182	VAR_015100	p.Gly384Ser	LP/P	rs12021720	Maple syrup urine disease 2 (MSUD2) [MIM:248600]
DBX2	Q6ZNG2	VAR_034969	p.Met171Val	LB/B	rs2731038	-
DCAF1	Q9Y4B6	VAR_051486	p.Asn267Asp	LB/B	rs3749318	-
DCAF1	Q9Y4B6	VAR_051487	p.Leu378Phe	LB/B	rs17712228	-
DCAF1	Q9Y4B6	VAR_051488	p.Leu1031Pro	LB/B	rs9835229	-
DCAF11	Q8TEB1	VAR_020121	p.Arg207His	LB/B	rs3825584	-
DCAF12	Q5T6F0	VAR_035322	p.Arg131Gln	LB/B	rs11557154	-
DCAF12L1	Q5VU92	VAR_035323	p.Asp19Gly	LB/B	rs11095722	-
DCAF12L2	Q5VW00	VAR_062104	p.His147Gln	LB/B	rs10126452	-
DCAF13	Q9NV06	VAR_037035	p.Ile42Val	LB/B	rs3134253	-
DCAF13	Q9NV06	VAR_037036	p.Asn70Ser	LB/B	rs13272825	-
DCAF16	Q9NXF7	VAR_034917	p.Asn45Ser	LB/B	rs34085539	-
DCAF16	Q9NXF7	VAR_034918	p.Thr129Ile	LB/B	rs7690457	-
DCAF17	Q5H9S7	VAR_050711	p.His185Gln	LB/B	rs3731984	-
DCAF4	Q8WV16	VAR_027908	p.Trp22Cys	LB/B	rs2302588	-
DCAF4	Q8WV16	VAR_027909	p.Lys193Thr	LB/B	rs17856582	-
DCAF4	Q8WV16	VAR_027910	p.Asn266Ser	LB/B	rs7155812	-
DCAF4	Q8WV16	VAR_027911	p.Leu334Phe	LB/B	rs17856583	-
DCAF4	Q8WV16	VAR_027912	p.Ser345Cys	LB/B	rs3815460	-
DCAF4	Q8WV16	VAR_027913	p.Arg439Ile	LB/B	rs17856584	-
DCAF6	Q58WW2	VAR_035020	p.Val547Ala	LB/B	rs11558511	-
DCAF8	Q5TAQ9	VAR_071265	p.Arg317Cys	LP/P	rs587777425	Giant axonal neuropathy 2, autosomal dominant (GAN2) [MIM:610100]
DCAF8L1	A6NGE4	VAR_038060	p.Arg549Trp	LB/B	rs12388557	-
DCAF8L2	P0C7V8	VAR_045620	p.Thr345Ala	LB/B	rs5926895	-
DCAKD	Q8WVC6	VAR_038406	p.Asn84Ile	LB/B	rs17850104	-
DCANP1	Q8TF63	VAR_025758	p.Thr75Pro	LB/B	rs1031844	-
DCANP1	Q8TF63	VAR_025759	p.Asn97Asp	LB/B	rs12520809	-
DCBLD2	Q96PD2	VAR_050944	p.Ile144Met	LB/B	rs9838238	-
DCBLD2	Q96PD2	VAR_050945	p.Asp723Asn	LB/B	rs16840208	-
DCC	P43146	VAR_003909	p.Met168Thr	US	rs121912967	A esophageal carcinoma
DCC	P43146	VAR_003910	p.Arg201Gly	LB/B	rs2229080	-
DCC	P43146	VAR_003911	p.Pro1375His	US	rs387906555	A colorectal carcinoma
DCC	P43146	VAR_024495	p.Met1017Val	LB/B	rs984274	-
DCC	P43146	VAR_035511	p.Phe1039Ser	US	-	A colorectal cancer sample
DCC	P43146	VAR_056043	p.Ile759Met	LB/B	rs2278339	-
DCC	P43146	VAR_060257	p.Phe23Leu	LB/B	rs9951523	-
DCC	P43146	VAR_060258	p.Leu679Arg	LB/B	rs2271042	-
DCC	P43146	VAR_060259	p.His1191Leu	LB/B	rs2270950	-
DCC	P43146	VAR_079146	p.Arg597Pro	US	rs1057519056	Mirror movements 1 (MRMV1) [MIM:157600]
DCC	P43146	VAR_079147	p.Met743Leu	US	rs199651452	Mirror movements 1 (MRMV1) [MIM:157600]
DCC	P43146	VAR_079148	p.Val754Met	US	rs775565634	Mirror movements 1 (MRMV1) [MIM:157600]
DCC	P43146	VAR_079149	p.Val793Gly	LP/P	rs1057519054	Mirror movements 1 (MRMV1) [MIM:157600]
DCC	P43146	VAR_079150	p.Gly805Glu	LP/P	rs1057519055	Mirror movements 1 (MRMV1) [MIM:157600]

DCC P43146 VAR_079151 p.Ala893Thr US rs1057519057 Mirror movements 1 (MRMV1) [MIM:157600]
DCC P43146 VAR_079152 p.Met1217Val US rs1057519058 Mirror movements 1 (MRMV1) [MIM:157600]
DCC P43146 VAR_079153 p.Ala1250Thr US rs748112308 Mirror movements 1 (MRMV1) [MIM:157600]
DCC P43146 VAR_079287 p.Gln691Lys US rs1555652216 Gaze palsy, familial horizontal, with progressive scoliosis, 2, with impaired intellectual development (HGPPS2) [MIM:617542]
DCDC1 M0R2J8 VAR_033767 p.Val83Met LB/B rs2761591 -
DCDC1 M0R2J8 VAR_037284 p.Glu7Gly LB/B rs11031357 -
DCDC2 Q9UHG0 VAR_022890 p.Ser221Gly LB/B rs2274305 -
DCDC2 Q9UHG0 VAR_050946 p.Pro152Ala LB/B rs33914824 -
DCDC2 Q9UHG0 VAR_050947 p.Lys456Asn LB/B rs9460973 -
DCDC2 Q9UHG0 VAR_074667 p.Gln424Pro LP/P rs794729665 Deafness, autosomal recessive, 66 (DFNB66) [MIM:610212]
DCDC2 Q9UHG0 VAR_077245 p.Lys17Asn LP/P rs1042640142 Sclerosing cholangitis, neonatal (NSC) [MIM:617394]
DCHS1 Q96JQ0 VAR_036110 p.Arg1583Trp US rs148882462 A colorectal cancer sample
DCHS1 Q96JQ0 VAR_048577 p.Thr1949Met LB/B rs4758443 -
DCHS1 Q96JQ0 VAR_048578 p.Val2331Ile LB/B rs7924553 -
DCHS1 Q96JQ0 VAR_048579 p.Arg2359Cys LB/B rs2659875 -
DCHS1 Q96JQ0 VAR_061074 p.Leu2172Gln LB/B rs56920123 -
DCHS1 Q96JQ0 VAR_070928 p.Asn2370Ile LP/P rs483352919 Van Maldergem syndrome 1 (VMLDS1) [MIM:601390]
DCHS1 Q96JQ0 VAR_075048 p.Pro197Leu LB/B rs145099391 -
DCHS1 Q96JQ0 VAR_075049 p.Arg2330Cys LP/P rs768737101 Mitral valve prolapse 2 (MVP2) [MIM:607829]
DCHS1 Q96JQ0 VAR_075050 p.Arg2513His LP/P rs201457110 Mitral valve prolapse 2 (MVP2) [MIM:607829]
DCLK1 O15075 VAR_045673 p.Gly29Cys US - A gastric adenocarcinoma sample
DCLK1 O15075 VAR_045674 p.Thr46Met US - A gastric adenocarcinoma sample
DCLK1 O15075 VAR_045675 p.Arg93Gln US rs1366698690 A gastric adenocarcinoma sample
DCLK1 O15075 VAR_045676 p.Ser291Phe US - A gastric adenocarcinoma sample
DCLK1 O15075 VAR_045677 p.Arg292His LB/B rs56185003 -
DCLK2 Q8N568 VAR_040441 p.Gly119Cys LB/B rs56327537 -
DCLK2 Q8N568 VAR_040442 p.Arg372His LB/B rs34386880 -
DCLK2 Q8N568 VAR_040443 p.Ile583Val LB/B rs35745104 -
DCLK2 Q8N568 VAR_073158 p.Val212Met US rs759398144 -
DCLK3 Q9C098 VAR_040444 p.Arg24Gln LB/B rs56070233 -
DCLK3 Q9C098 VAR_040445 p.Pro108Leu US - A breast infiltrating ductal carcinoma sample
DCLK3 Q9C098 VAR_040446 p.Glu422Lys US rs373605259 A colorectal adenocarcinoma sample
DCLK3 Q9C098 VAR_040447 p.Ser472Asn US - A lung large cell carcinoma sample
DCLK3 Q9C098 VAR_040448 p.Arg554Cys US rs374711533 A lung squamous cell carcinoma sample
DCLK3 Q9C098 VAR_040449 p.Gly570Arg US - A renal clear cell carcinoma sample
DCLK3 Q9C098 VAR_040450 p.Val596Ala US - A colorectal adenocarcinoma sample
DCLK3 Q9C098 VAR_040451 p.Glu633Asp LB/B rs35704209 -
DCLRE1A Q6PJP8 VAR_023286 p.Lys58Glu LB/B rs17235066 -
DCLRE1A Q6PJP8 VAR_023287 p.Glu59Asp LB/B rs17228665 -
DCLRE1A Q6PJP8 VAR_023288 p.Gly71Asp LB/B rs17228672 -
DCLRE1A Q6PJP8 VAR_023289 p.Pro287Leu LB/B rs17235094 -
DCLRE1A Q6PJP8 VAR_023290 p.Asp317His LB/B rs3750898 -
DCLRE1A Q6PJP8 VAR_023291 p.Ile859Phe LB/B rs11196530 -
DCLRE1A Q6PJP8 VAR_030574 p.Gly582Trp LB/B rs17855759 -
DCLRE1B Q9H816 VAR_023292 p.Arg46Leu LB/B rs28381069 -

DCLRE1B Q9H816 VAR_023293 p.His61Tyr LB/B rs11552449 -
DCLRE1B Q9H816 VAR_023294 p.Asp462Asn LB/B rs28381079 -
DCLRE1B Q9H816 VAR_048891 p.Asn510Tyr LB/B rs35397235 -
DCLRE1C Q96SD1 VAR_023077 p.His35Asp LP/P rs121908159 Omenn syndrome (OS) [MIM:603554]
DCLRE1C Q96SD1 VAR_023078 p.Gly118Val LP/P - Severe combined immunodeficiency autosomal recessive T-cell-negative/B-cell-negative/NK-cell-positive with sensitivity to ionizing radiation (RSSCID) [MIM:602450]
DCLRE1C Q96SD1 VAR_023079 p.Gly135Glu LP/P - Severe combined immunodeficiency autosomal recessive T-cell-negative/B-cell-negative/NK-cell-positive with sensitivity to ionizing radiation (RSSCID) [MIM:602450]
DCLRE1C Q96SD1 VAR_048892 p.Pro171Arg LB/B rs35441642 -
DCLRE1C Q96SD1 VAR_048893 p.His243Arg LB/B rs12768894 -
DCLRE1C Q96SD1 VAR_048894 p.Ser320Cys LB/B rs41298896 -
DCLRE1C Q96SD1 VAR_060689 p.Ala140Val LB/B rs41297016 -
DCLRE1C Q96SD1 VAR_060690 p.Gly153Arg LB/B rs41297018 -
DCLRE1C Q96SD1 VAR_060691 p.Leu329Met LB/B rs41299658 -
DCN P07585 VAR_011975 p.Glu273Gln LB/B rs1803344 -
DCN P07585 VAR_014351 p.Thr268Met LB/B rs3138268 -
DCP1B Q8IZD4 VAR_047395 p.Asn195Asp LB/B rs12423058 -
DCP1B Q8IZD4 VAR_047396 p.Asn216Ser LB/B rs34730825 -
DCP1B Q8IZD4 VAR_047397 p.Ser301Thr LB/B rs2470449 -
DCP1B Q8IZD4 VAR_047398 p.Arg344His LB/B rs715146 -
DCP2 Q8IU60 VAR_059528 p.Leu16Phe LB/B rs33555 -
DCPS Q96C86 VAR_027958 p.Gly73Glu LB/B rs11557735 -
DCPS Q96C86 VAR_073956 p.Thr316Met LP/P rs137941190 Al-Raqad syndrome (ARS) [MIM:616459]
DCST1 Q5T197 VAR_030868 p.Met512Leu LB/B rs11264300 -
DCST1 Q5T197 VAR_056859 p.Thr158Ile LB/B rs9427170 -
DCSTAMP Q9H295 VAR_051438 p.Asp349Gly LB/B rs3802204 -
DCTN1 Q14203 VAR_001373 p.Ala163Pro LB/B - -
DCTN1 Q14203 VAR_015850 p.Gly59Ser LP/P rs121909342 Neuronopathy, distal hereditary motor, 7B (HMN7B) [MIM:607641]
DCTN1 Q14203 VAR_048677 p.Leu287Met LB/B rs13420401 -
DCTN1 Q14203 VAR_048678 p.Arg495Gln LB/B rs17721059 -
DCTN1 Q14203 VAR_063867 p.Gly71Ala LP/P rs67586389 Perry syndrome (PERRYS) [MIM:168605]
DCTN1 Q14203 VAR_063868 p.Gly71Glu LP/P rs67586389 Perry syndrome (PERRYS) [MIM:168605]
DCTN1 Q14203 VAR_063869 p.Gly71Arg LP/P rs72466485 Perry syndrome (PERRYS) [MIM:168605]
DCTN1 Q14203 VAR_063870 p.Thr72Pro LP/P rs72466486 Perry syndrome (PERRYS) [MIM:168605]
DCTN1 Q14203 VAR_063871 p.Gln74Pro LP/P rs72466487 Perry syndrome (PERRYS) [MIM:168605]
DCTN1 Q14203 VAR_063872 p.Met571Thr LP/P rs121909343 Amyotrophic lateral sclerosis (ALS) [MIM:105400]
DCTN1 Q14203 VAR_063873 p.Arg785Trp LP/P rs121909344 Amyotrophic lateral sclerosis (ALS) [MIM:105400]
DCTN1 Q14203 VAR_063874 p.Arg1101Lys LP/P rs121909345 Amyotrophic lateral sclerosis (ALS) [MIM:105400]
DCTN1 Q14203 VAR_063875 p.Thr1249Ile US rs72466496 Amyotrophic lateral sclerosis (ALS) [MIM:105400]
DCTN1 Q14203 VAR_071452 p.Phe52Leu LP/P rs886039227 Perry syndrome (PERRYS) [MIM:168605]
DCTN1 Q14203 VAR_071453 p.Tyr78Cys LP/P rs886039229 Perry syndrome (PERRYS) [MIM:168605]
DCTN1 Q14203 VAR_073287 p.Tyr670Phe US rs765819985 -
DCTN1 Q14203 VAR_076920 p.Ile196Val LB/B rs55862001 -
DCTN4 Q9UJW0 VAR_024336 p.Phe342Leu LB/B rs11954652 -
DCTN4 Q9UJW0 VAR_024337 p.Ser438Asn LB/B rs3733923 -

DCTN4 Q9UJW0 VAR_033847 p.Tyr263Cys LB/B rs35772018 -
DCTN4 Q9UJW0 VAR_054037 p.Pro95Thr LB/B rs11550931 -
DCUN1D3 Q8IWE4 VAR_072689 p.Gly2Ser US - A cancer
DCUN1D3 Q8IWE4 VAR_072690 p.Ser239Phe US - A cancer
DCX O43602 VAR_007819 p.Leu43Ser LP/P rs587783521 Lissencephaly, X-linked 1 (LISX1) [MIM:300067]
DCX O43602 VAR_007820 p.Ser47Arg LP/P rs104894783 Lissencephaly, X-linked 1 (LISX1) [MIM:300067]
DCX O43602 VAR_007820 p.Ser47Arg LP/P rs104894783 Subcortical band heterotopia X-linked (SBHX) [MIM:300067]
DCX O43602 VAR_007821 p.Arg59Leu LP/P rs122457137 Lissencephaly, X-linked 1 (LISX1) [MIM:300067]
DCX O43602 VAR_007821 p.Arg59Leu LP/P rs122457137 Subcortical band heterotopia X-linked (SBHX) [MIM:300067]
DCX O43602 VAR_007822 p.Arg59His LP/P rs122457137 Subcortical band heterotopia X-linked (SBHX) [MIM:300067]
DCX O43602 VAR_007823 p.Asp62Asn LP/P rs104894779 Lissencephaly, X-linked 1 (LISX1) [MIM:300067]
DCX O43602 VAR_007823 p.Asp62Asn LP/P rs104894779 Subcortical band heterotopia X-linked (SBHX) [MIM:300067]
DCX O43602 VAR_007824 p.Arg78Leu LP/P rs104894784 Subcortical band heterotopia X-linked (SBHX) [MIM:300067]
DCX O43602 VAR_007825 p.Asp86His LP/P - Subcortical band heterotopia X-linked (SBHX) [MIM:300067]
DCX O43602 VAR_007826 p.Gly100Ala LP/P - Lissencephaly, X-linked 1 (LISX1) [MIM:300067]
DCX O43602 VAR_007826 p.Gly100Ala LP/P - Subcortical band heterotopia X-linked (SBHX) [MIM:300067]
DCX O43602 VAR_007827 p.Arg102Ser LP/P - Lissencephaly, X-linked 1 (LISX1) [MIM:300067]
DCX O43602 VAR_007828 p.Tyr125His LP/P rs104894781 Lissencephaly, X-linked 1 (LISX1) [MIM:300067]
DCX O43602 VAR_007828 p.Tyr125His LP/P rs104894781 Subcortical band heterotopia X-linked (SBHX) [MIM:300067]
DCX O43602 VAR_007829 p.Tyr125Asp LP/P - Subcortical band heterotopia X-linked (SBHX) [MIM:300067]
DCX O43602 VAR_007830 p.Arg178Leu LP/P rs587783559 Subcortical band heterotopia X-linked (SBHX) [MIM:300067]
DCX O43602 VAR_007831 p.Arg186Cys LP/P rs587783562 Subcortical band heterotopia X-linked (SBHX) [MIM:300067]
DCX O43602 VAR_007832 p.Pro191Arg LP/P rs587783566 Subcortical band heterotopia X-linked (SBHX) [MIM:300067]
DCX O43602 VAR_007833 p.Arg192Trp LP/P rs104894780 Lissencephaly, X-linked 1 (LISX1) [MIM:300067]
DCX O43602 VAR_007833 p.Arg192Trp LP/P rs104894780 Subcortical band heterotopia X-linked (SBHX) [MIM:300067]
DCX O43602 VAR_007834 p.Asn200Lys LP/P - Subcortical band heterotopia X-linked (SBHX) [MIM:300067]
DCX O43602 VAR_007835 p.Thr203Arg LP/P rs104894782 Lissencephaly, X-linked 1 (LISX1) [MIM:300067]
DCX O43602 VAR_007835 p.Thr203Arg LP/P rs104894782 Subcortical band heterotopia X-linked (SBHX) [MIM:300067]
DCX O43602 VAR_007836 p.Ile214Thr LP/P rs587783574 Subcortical band heterotopia X-linked (SBHX) [MIM:300067]
DCX O43602 VAR_007837 p.Thr222Ile LP/P - Subcortical band heterotopia X-linked (SBHX) [MIM:300067]
DCX O43602 VAR_007838 p.Gly223Glu LP/P - Subcortical band heterotopia X-linked (SBHX) [MIM:300067]

DCX O43602 VAR_007839 p.Val236Ile LP/P rs1324159050 Subcortical band heterotopia X-linked (SBHX) [MIM:300067]
DCX O43602 VAR_007840 p.Ile250Asn LP/P - Subcortical band heterotopia X-linked (SBHX) [MIM:300067]
DCX O43602 VAR_007841 p.Ile250Thr LP/P - Subcortical band heterotopia X-linked (SBHX) [MIM:300067]
DCX O43602 VAR_007842 p.Gly253Asp LP/P - Subcortical band heterotopia X-linked (SBHX) [MIM:300067]
DCX O43602 VAR_010202 p.Arg78His LB/B rs104894784 -
DCX O43602 VAR_010536 p.Arg89Gly LP/P rs104894785 Subcortical band heterotopia X-linked (SBHX) [MIM:300067]
DCX O43602 VAR_026022 p.Thr42Ile LP/P - Lissencephaly, X-linked 1 (LISX1) [MIM:300067]
DCX O43602 VAR_026023 p.Lys50Asn LP/P rs587783523 Subcortical band heterotopia X-linked (SBHX) [MIM:300067]
DCX O43602 VAR_026024 p.Asn60Asp LP/P - Lissencephaly, X-linked 1 (LISX1) [MIM:300067]
DCX O43602 VAR_026025 p.Gly67Glu LP/P - Subcortical band heterotopia X-linked (SBHX) [MIM:300067]
DCX O43602 VAR_026026 p.Ala71Ser LP/P rs104894786 Lissencephaly, X-linked 1 (LISX1) [MIM:300067]
DCX O43602 VAR_026027 p.Leu97Arg LP/P rs587783537 Subcortical band heterotopia X-linked (SBHX) [MIM:300067]
DCX O43602 VAR_026028 p.Ile104Thr LP/P - Subcortical band heterotopia X-linked (SBHX) [MIM:300067]
DCX O43602 VAR_026029 p.Arg178Cys LP/P rs587783558 Subcortical band heterotopia X-linked (SBHX) [MIM:300067]
DCX O43602 VAR_026030 p.Pro191Leu LP/P - Subcortical band heterotopia X-linked (SBHX) [MIM:300067]
DCX O43602 VAR_026031 p.Arg196His LP/P rs56030372 Lissencephaly, X-linked 1 (LISX1) [MIM:300067]
DCX O43602 VAR_026032 p.Arg196Ser LB/B rs587783568 -
DCX O43602 VAR_026033 p.Asn200Ile LP/P - Subcortical band heterotopia X-linked (SBHX) [MIM:300067]
DCX O43602 VAR_026034 p.Thr203Ala LP/P rs587783570 Subcortical band heterotopia X-linked (SBHX) [MIM:300067]
DCX O43602 VAR_026035 p.Gly223Val LP/P - Subcortical band heterotopia X-linked (SBHX) [MIM:300067]
DCX O43602 VAR_026036 p.Phe243Leu LP/P - Lissencephaly, X-linked 1 (LISX1) [MIM:300067]
DCX O43602 VAR_026037 p.Ala251Ser LP/P rs587783585 Subcortical band heterotopia X-linked (SBHX) [MIM:300067]
DCX O43602 VAR_026038 p.Ala251Val LP/P - Subcortical band heterotopia X-linked (SBHX) [MIM:300067]
DCX O43602 VAR_077482 p.Asp262Gly LP/P rs398124557 Lissencephaly, X-linked 1 (LISX1) [MIM:300067]
DCX O43602 VAR_077482 p.Asp262Gly LP/P rs398124557 Subcortical band heterotopia X-linked (SBHX) [MIM:300067]
DDB1 Q16531 VAR_023074 p.Leu427Phe LB/B rs28720299 -
DDB2 Q92466 VAR_010141 p.Lys244Glu LP/P rs121434639 Xeroderma pigmentosum complementation group E (XP-E) [MIM:278740]
DDB2 Q92466 VAR_010142 p.Arg273His LP/P rs121434640 Xeroderma pigmentosum complementation group E (XP-E) [MIM:278740]
DDB2 Q92466 VAR_016337 p.Met215Thr LB/B rs4647750 -
DDB2 Q92466 VAR_016338 p.Ala293Thr LB/B rs4647751 -

DDC P20711 VAR_014788 p.Met17Val LB/B rs6264 -
DDC P20711 VAR_014789 p.Pro210Leu LB/B rs6262 -
DDC P20711 VAR_014790 p.Met217Val LB/B rs6263 -
DDC P20711 VAR_019214 p.Glu61Asp LB/B rs11575292 -
DDC P20711 VAR_019215 p.Met239Ile LB/B rs11575377 -
DDC P20711 VAR_019216 p.Met239Leu LB/B rs11575376 -
DDC P20711 VAR_019217 p.Arg462Gln LB/B rs11575542 -
DDC P20711 VAR_019309 p.Gly102Ser LP/P rs137853207 Aromatic L-amino-acid decarboxylase deficiency (AADCD) [MIM:608643]
DDC P20711 VAR_046137 p.Pro47His LP/P rs780542462 Aromatic L-amino-acid decarboxylase deficiency (AADCD) [MIM:608643]
DDC P20711 VAR_046138 p.Ala91Val LP/P rs137853211 Aromatic L-amino-acid decarboxylase deficiency (AADCD) [MIM:608643]
DDC P20711 VAR_046139 p.Ser147Arg LP/P rs137853210 Aromatic L-amino-acid decarboxylase deficiency (AADCD) [MIM:608643]
DDC P20711 VAR_046140 p.Ser250Phe LP/P rs137853208 Aromatic L-amino-acid decarboxylase deficiency (AADCD) [MIM:608643]
DDC P20711 VAR_046141 p.Ala275Thr LP/P rs137853212 Aromatic L-amino-acid decarboxylase deficiency (AADCD) [MIM:608643]
DDC P20711 VAR_046142 p.Phe309Leu LP/P rs137853209 Aromatic L-amino-acid decarboxylase deficiency (AADCD) [MIM:608643]
DDC P20711 VAR_046143 p.Arg347Gln LP/P rs201951824 Aromatic L-amino-acid decarboxylase deficiency (AADCD) [MIM:608643]
DDC P20711 VAR_046144 p.Leu408Ile LP/P - Aromatic L-amino-acid decarboxylase deficiency (AADCD) [MIM:608643]
DDHD2 O94830 VAR_036930 p.Thr186Met LB/B rs2306899 -
DDHD2 O94830 VAR_069574 p.Asp660His LP/P rs375168720 Spastic paraplegia 54, autosomal recessive (SPG54) [MIM:615033]
DDI1 Q8WTU0 VAR_032259 p.Gly136Ser LB/B rs7102675 -
DDI1 Q8WTU0 VAR_032260 p.Ser142Gly LB/B rs17856633 -
DDI1 Q8WTU0 VAR_032261 p.Gly239Glu LB/B rs17851870 -
DDIAS Q8IXT1 VAR_037326 p.Ile217Thr LB/B rs17853911 -
DDIAS Q8IXT1 VAR_037327 p.Arg460Ser LB/B rs7947780 -
DDIAS Q8IXT1 VAR_037328 p.Asp757Asn LB/B rs35711622 -
DDIAS Q8IXT1 VAR_037329 p.Pro795Arg LB/B rs11826199 -
DDIAS Q8IXT1 VAR_037330 p.Val842Ile LB/B rs7130899 -
DDIT3 P35638 VAR_036000 p.Ala115Val US - A colorectal cancer sample
DDIT4L Q96D03 VAR_053971 p.Ser161Phe LB/B rs11553154 -
DDN O94850 VAR_059651 p.Glu661Gly LB/B rs10783299 -
DDO Q99489 VAR_014939 p.Gln189Glu LB/B rs17622 -
DDO Q99489 VAR_014940 p.His230Tyr LB/B rs17621 -
DDO Q99489 VAR_014941 p.Leu255Arg LB/B rs17623 -
DDO Q99489 VAR_036244 p.Phe136Leu US - A breast cancer sample
DDOST P39656 VAR_047911 p.Arg8Gly LB/B rs537816 -
DDOST P39656 VAR_067544 p.Gly217Asp LP/P rs387906831 Congenital disorder of glycosylation 1R (CDG1R) [MIM:614507]
DDR1 Q08345 VAR_041492 p.Ser17Gly LB/B rs55901302 -
DDR1 Q08345 VAR_041493 p.Val100Ala LB/B rs34544756 -
DDR1 Q08345 VAR_041494 p.Arg169Gln LB/B rs55980643 -

DDR1 Q08345 VAR_041495 p.Ala170Asp LB/B rs56231803 -
DDR1 Q08345 VAR_041496 p.Arg306Trp LB/B rs56024191 -
DDR1 Q08345 VAR_041497 p.Ser496Ala US - A lung squamous cell carcinoma sample
DDR1 Q08345 VAR_049716 p.Leu833Val LB/B rs2524235 -
DDR2 Q16832 VAR_041498 p.Arg105Ser US - A lung large cell carcinoma sample
DDR2 Q16832 VAR_041499 p.Met441Ile LB/B rs34722354 -
DDR2 Q16832 VAR_041500 p.Arg478Cys LB/B rs34869543 -
DDR2 Q16832 VAR_041501 p.Val543Phe LB/B rs55973200 -
DDR2 Q16832 VAR_063050 p.Thr713Ile LP/P rs121964865 Spondyloepimetaphyseal dysplasia, short limb-hand type (SEMD-SL) [MIM:271665]
DDR2 Q16832 VAR_063051 p.Ile726Arg LP/P rs121964864 Spondyloepimetaphyseal dysplasia, short limb-hand type (SEMD-SL) [MIM:271665]
DDR2 Q16832 VAR_063052 p.Arg752Cys LP/P rs121964863 Spondyloepimetaphyseal dysplasia, short limb-hand type (SEMD-SL) [MIM:271665]
DDR2 Q16832 VAR_065719 p.Glu113Lys LP/P rs397514747 Spondyloepimetaphyseal dysplasia, short limb-hand type (SEMD-SL) [MIM:271665]
DDR2 Q16832 VAR_075417 p.Arg124Trp LP/P - Spondyloepimetaphyseal dysplasia, short limb-hand type (SEMD-SL) [MIM:271665]
DDR2 Q16832 VAR_081931 p.Leu610Pro LP/P - Warburg-Cinotti syndrome (WRCN) [MIM:618175]
DDR2 Q16832 VAR_081932 p.Tyr740Cys LP/P - Warburg-Cinotti syndrome (WRCN) [MIM:618175]
DDRGK1 Q96HY6 VAR_016923 p.Ala303Thr LB/B rs11591 -
DDX10 Q13206 VAR_035840 p.Leu566Val US - A breast cancer sample
DDX11 Q96FC9 VAR_024808 p.Ile39Ser LB/B - -
DDX11 Q96FC9 VAR_024809 p.Gln567Glu LB/B rs2075322 -
DDX11 Q96FC9 VAR_024810 p.Thr575Met LB/B rs17857386 -
DDX11 Q96FC9 VAR_052175 p.Arg856His LB/B rs1046457 -
DDX11 Q96FC9 VAR_052176 p.Cys864Arg LB/B rs3893679 -
DDX11 Q96FC9 VAR_052177 p.Cys951Arg LB/B rs1046458 -
DDX11 Q96FC9 VAR_052178 p.Trp966Cys LB/B rs14330 -
DDX11 Q96FC9 VAR_069099 p.Arg263Gln LP/P rs201968272 Warsaw breakage syndrome (WBRS) [MIM:613398]
DDX18 Q9NVP1 VAR_013293 p.Thr94Ser LB/B rs1052637 -
DDX18 Q9NVP1 VAR_033857 p.Lys647Arg LB/B rs10179772 -
DDX18 Q9NVP1 VAR_035841 p.Gly41Arg US - A breast cancer sample
DDX19B Q9UMR2 VAR_052160 p.Val149Leu LB/B rs34607244 -
DDX20 Q9UHI6 VAR_057231 p.Ile636Thr LB/B rs197412 -
DDX20 Q9UHI6 VAR_057232 p.Arg693Ser LB/B rs197414 -
DDX20 Q9UHI6 VAR_057233 p.Ile762Thr LB/B rs85276 -
DDX21 Q9NR30 VAR_052161 p.Thr27Ile LB/B rs17556220 -
DDX24 Q9GZR7 VAR_052162 p.Glu316Lys LB/B rs35413935 -
DDX27 Q96GQ7 VAR_022849 p.Gly766Ser LB/B rs1130146 -
DDX28 Q9NUL7 VAR_052163 p.Thr4Ala LB/B rs237831 -
DDX31 Q9H8H2 VAR_023065 p.Ile799Val LB/B rs306547 -
DDX31 Q9H8H2 VAR_023066 p.Arg843Gln LB/B rs306548 -
DDX31 Q9H8H2 VAR_052164 p.Glu153Lys LB/B rs17402080 -
DDX31 Q9H8H2 VAR_052165 p.Arg687Gln LB/B rs34246652 -
DDX39A O00148 VAR_052166 p.Val142Ile LB/B rs36127505 -
DDX3X O00571 VAR_035839 p.Arg294Thr US - A breast cancer sample

DDX3X O00571 VAR_075731 p.Ile214Thr LP/P - Intellectual developmental disorder, X-linked, syndromic, Snijders Blok type (MRXSSB) [MIM:300958]
DDX3X O00571 VAR_075732 p.Ala233Val LP/P rs796052223 Intellectual developmental disorder, X-linked, syndromic, Snijders Blok type (MRXSSB) [MIM:300958]
DDX3X O00571 VAR_075734 p.Leu235Pro LP/P rs796052224 Intellectual developmental disorder, X-linked, syndromic, Snijders Blok type (MRXSSB) [MIM:300958]
DDX3X O00571 VAR_075735 p.Val300Phe LP/P - Intellectual developmental disorder, X-linked, syndromic, Snijders Blok type (MRXSSB) [MIM:300958]
DDX3X O00571 VAR_075736 p.Arg326His LP/P rs797045025 Intellectual developmental disorder, X-linked, syndromic, Snijders Blok type (MRXSSB) [MIM:300958]
DDX3X O00571 VAR_075737 p.Arg351Gln LP/P rs1057518707 Intellectual developmental disorder, X-linked, syndromic, Snijders Blok type (MRXSSB) [MIM:300958]
DDX3X O00571 VAR_075738 p.Arg362Cys LP/P rs797045026 Intellectual developmental disorder, X-linked, syndromic, Snijders Blok type (MRXSSB) [MIM:300958]
DDX3X O00571 VAR_075739 p.Arg376Cys LP/P rs796052231 Intellectual developmental disorder, X-linked, syndromic, Snijders Blok type (MRXSSB) [MIM:300958]
DDX3X O00571 VAR_075740 p.Leu392Pro LP/P rs796052232 Intellectual developmental disorder, X-linked, syndromic, Snijders Blok type (MRXSSB) [MIM:300958]
DDX3X O00571 VAR_075741 p.Gln417Pro LP/P rs796052233 Intellectual developmental disorder, X-linked, syndromic, Snijders Blok type (MRXSSB) [MIM:300958]
DDX3X O00571 VAR_075742 p.Arg475Gly LP/P rs1064794574 Intellectual developmental disorder, X-linked, syndromic, Snijders Blok type (MRXSSB) [MIM:300958]
DDX3X O00571 VAR_075743 p.Arg480Ser LP/P - Intellectual developmental disorder, X-linked, syndromic, Snijders Blok type (MRXSSB) [MIM:300958]
DDX3X O00571 VAR_075744 p.Arg488His LP/P rs796052235 Intellectual developmental disorder, X-linked, syndromic, Snijders Blok type (MRXSSB) [MIM:300958]
DDX3X O00571 VAR_075745 p.Ile507Thr LP/P rs797045024 Intellectual developmental disorder, X-linked, syndromic, Snijders Blok type (MRXSSB) [MIM:300958]
DDX3X O00571 VAR_075746 p.Asn509Ile LP/P - Intellectual developmental disorder, X-linked, syndromic, Snijders Blok type (MRXSSB) [MIM:300958]
DDX3X O00571 VAR_075747 p.Ile514Thr LP/P rs796052226 Intellectual developmental disorder, X-linked, syndromic, Snijders Blok type (MRXSSB) [MIM:300958]
DDX3X O00571 VAR_075748 p.Arg534His LP/P - Intellectual developmental disorder, X-linked, syndromic, Snijders Blok type (MRXSSB) [MIM:300958]
DDX3X O00571 VAR_075750 p.Pro568Leu LP/P rs1057519430 Intellectual developmental disorder, X-linked, syndromic, Snijders Blok type (MRXSSB) [MIM:300958]
DDX3X O00571 VAR_083115 p.Arg528His US - -
DDX4 Q9NQI0 VAR_019574 p.Gly148Asp LB/B rs2306259 -
DDX4 Q9NQI0 VAR_052159 p.Ile287Val LB/B rs2305123 -
DDX41 Q9UJV9 VAR_076360 p.Arg164Trp US rs142143752 Myeloproliferative/lymphoproliferative neoplasms, familial (MPLPF) [MIM:616871]
DDX41 Q9UJV9 VAR_076361 p.Ile396Thr US rs747072227 Myeloproliferative/lymphoproliferative neoplasms, familial (MPLPF) [MIM:616871]
DDX41 Q9UJV9 VAR_076362 p.Arg525His LP/P rs869312828 Myeloproliferative/lymphoproliferative neoplasms, familial (MPLPF) [MIM:616871]
DDX43 Q9NXZ2 VAR_057234 p.Lys625Glu LB/B rs311686 -
DDX43 Q9NXZ2 VAR_057235 p.Gln629Arg LB/B rs311685 -
DDX46 Q7L014 VAR_028079 p.Glu207Gln LB/B rs10447293 -
DDX47 Q9H0S4 VAR_083613 p.Asp8Tyr US rs577622688 -

DDX47 Q9H0S4 VAR_083614 p.Gln107Glu US - -
DDX49 Q9Y6V7 VAR_033858 p.Arg222His LB/B rs35802425 -
DDX49 Q9Y6V7 VAR_033859 p.Ser296Ala LB/B rs35614860 -
DDX49 Q9Y6V7 VAR_052167 p.Arg413Trp LB/B rs16995781 -
DDX5 P17844 VAR_029241 p.Ser480Ala LB/B rs1140409 -
DDX51 Q8N8A6 VAR_055299 p.Arg41Gln LB/B rs17857214 -
DDX51 Q8N8A6 VAR_055300 p.Glu134Val LB/B rs17855642 -
DDX51 Q8N8A6 VAR_055301 p.Gln175Lys LB/B rs17855639 -
DDX51 Q8N8A6 VAR_055302 p.Pro249Leu LB/B rs17857213 -
DDX51 Q8N8A6 VAR_055303 p.Gln295Arg LB/B rs1133690 -
DDX51 Q8N8A6 VAR_055304 p.Gln406Lys LB/B rs17853968 -
DDX51 Q8N8A6 VAR_055305 p.Gln652Lys LB/B rs17853969 -
DDX51 Q8N8A6 VAR_061825 p.Ala322Val LB/B rs60927391 -
DDX52 Q9Y2R4 VAR_060235 p.Met403Val LB/B rs7216445 -
DDX53 Q86TM3 VAR_052168 p.Val62Ala LB/B rs4412516 -
DDX53 Q86TM3 VAR_052169 p.Met381Ile LB/B rs5925720 -
DDX53 Q86TM3 VAR_052170 p.Arg391Met LB/B rs5926203 -
DDX53 Q86TM3 VAR_064707 p.Asn141Thr US - -
DDX54 Q8TDD1 VAR_033860 p.Pro821Leu LB/B rs1048889 -
DDX54 Q8TDD1 VAR_052171 p.Arg570His LB/B rs35519289 -
DDX54 Q8TDD1 VAR_052172 p.Arg693Gln LB/B rs11564 -
DDX54 Q8TDD1 VAR_052173 p.Val712Ala LB/B rs10354 -
DDX54 Q8TDD1 VAR_083615 p.Trp20Arg US rs774035439 -
DDX54 Q8TDD1 VAR_083616 p.Asn216Ser US rs764707570 -
DDX54 Q8TDD1 VAR_083617 p.Val286Met US rs148961522 -
DDX54 Q8TDD1 VAR_083618 p.Leu298Phe US - -
DDX54 Q8TDD1 VAR_083619 p.Arg611Gln US rs777276705 -
DDX55 Q8NHQ9 VAR_027789 p.Val101Leu LB/B rs17881657 -
DDX55 Q8NHQ9 VAR_027790 p.Glu154Gly LB/B rs17886035 -
DDX55 Q8NHQ9 VAR_027791 p.Asn264Ser LB/B rs11057306 -
DDX55 Q8NHQ9 VAR_027792 p.Asn556Ser LB/B rs10773019 -
DDX58 O95786 VAR_023747 p.Arg7Cys LB/B rs10813831 -
DDX58 O95786 VAR_023748 p.Asp580Glu LB/B rs17217280 -
DDX58 O95786 VAR_073667 p.Cys268Phe LP/P rs786204848 Singleton-Merten syndrome 2 (SGMRT2) [MIM:616298]
DDX58 O95786 VAR_073668 p.Glu373Ala LP/P rs786204847 Singleton-Merten syndrome 2 (SGMRT2) [MIM:616298]
DDX59 Q5T1V6 VAR_031424 p.Ile107Val LB/B rs3795634 -
DDX59 Q5T1V6 VAR_033001 p.Ser472Arg LB/B rs17854157 -
DDX59 Q5T1V6 VAR_035842 p.Pro77Thr US - A breast cancer sample
DDX59 Q5T1V6 VAR_070198 p.Val367Gly LP/P rs587777067 Orofaciodigital syndrome 5 (OFD5) [MIM:174300]
DDX59 Q5T1V6 VAR_070199 p.Gly534Arg LP/P rs886037652 Orofaciodigital syndrome 5 (OFD5) [MIM:174300]
DDX6 P26196 VAR_083368 p.His372Arg LP/P - Intellectual developmental disorder with impaired language and dysmorphic facies (IDDILF) [MIM:618653]
DDX6 P26196 VAR_083369 p.Arg373Gln LP/P - Intellectual developmental disorder with impaired language and dysmorphic facies (IDDILF) [MIM:618653]

DDX6 P26196 VAR_083370 p.Cys390Arg LP/P - Intellectual developmental disorder with impaired language and dysmorphic facies (IDDILF) [MIM:618653]
DDX6 P26196 VAR_083371 p.Thr391Ile LP/P - Intellectual developmental disorder with impaired language and dysmorphic facies (IDDILF) [MIM:618653]
DDX6 P26196 VAR_083372 p.Thr391Pro LP/P - Intellectual developmental disorder with impaired language and dysmorphic facies (IDDILF) [MIM:618653]
DDX60 Q8IY21 VAR_055895 p.Val672Met LB/B rs550625 -
DDX60 Q8IY21 VAR_055896 p.Ile998Val LB/B rs576619 -
DDX60L Q5H9U9 VAR_055897 p.Cys336Tyr LB/B rs12507582 -
DDX60L Q5H9U9 VAR_055898 p.Asn355Lys LB/B rs10029536 -
DDX60L Q5H9U9 VAR_055899 p.Val409Leu LB/B rs13151700 -
DDX60L Q5H9U9 VAR_055900 p.Ala583Thr LB/B rs2319850 -
DDX60L Q5H9U9 VAR_055901 p.Ser1080Cys LB/B rs17540213 -
DEAF1 O75398 VAR_013725 p.Glu186Val US rs751727919 A primary colorectal cancer
DEAF1 O75398 VAR_013726 p.Lys191Ile US - A primary colorectal cancer
DEAF1 O75398 VAR_013727 p.Lys191Asn US - A primary colorectal cancer
DEAF1 O75398 VAR_013729 p.Glu202Asp US - A primary colorectal cancer
DEAF1 O75398 VAR_013730 p.Arg218Lys US rs1127312 A primary colorectal cancer
DEAF1 O75398 VAR_013732 p.Glu356His US - A primary colorectal cancer
DEAF1 O75398 VAR_013733 p.Ser364Asn US - A primary colorectal cancer
DEAF1 O75398 VAR_013734 p.Gln367His US - A primary colorectal cancer
DEAF1 O75398 VAR_013735 p.Val370Leu US - A primary colorectal cancer
DEAF1 O75398 VAR_013736 p.Tyr397Phe US - A primary colorectal cancer
DEAF1 O75398 VAR_013737 p.Val442Ala US - A primary colorectal cancer
DEAF1 O75398 VAR_013738 p.Glu449Lys US - A primary colorectal cancer
DEAF1 O75398 VAR_013740 p.Gln468His US - A primary colorectal cancer
DEAF1 O75398 VAR_013741 p.His479Leu US - A primary colorectal cancer
DEAF1 O75398 VAR_013742 p.Glu498Lys US - A primary colorectal cancer
DEAF1 O75398 VAR_013743 p.Thr526Asn US - A primary colorectal cancer
DEAF1 O75398 VAR_013744 p.Arg530Leu US - A primary colorectal cancer
DEAF1 O75398 VAR_013746 p.Gln542His US - A primary colorectal cancer
DEAF1 O75398 VAR_013747 p.Ala545Gly US rs34114147 A primary colorectal cancer
DEAF1 O75398 VAR_013748 p.Ala545Val US - A primary colorectal cancer
DEAF1 O75398 VAR_065089 p.Ile228Ser LP/P rs587777406 Vulto-van Silfout-de Vries syndrome (VSVS) [MIM:615828]
DEAF1 O75398 VAR_071371 p.Arg224Trp LP/P rs587777408 Vulto-van Silfout-de Vries syndrome (VSVS) [MIM:615828]
DEAF1 O75398 VAR_071372 p.Arg226Trp LB/B rs587777623 -
DEAF1 O75398 VAR_071373 p.Arg254Ser LP/P rs587777409 Vulto-van Silfout-de Vries syndrome (VSVS) [MIM:615828]
DEAF1 O75398 VAR_071374 p.Gln264Pro LP/P rs587777407 Vulto-van Silfout-de Vries syndrome (VSVS) [MIM:615828]
DECR1 Q16698 VAR_012034 p.Lys333Asn LB/B rs15094 -
DEF6 Q9H4E7 VAR_033193 p.Asn287Thr LB/B rs2395617 -
DEF6 Q9H4E7 VAR_033194 p.Arg578His LB/B rs9296146 -
DEF8 Q6ZN54 VAR_061485 p.Gln90Glu LB/B rs7194844 -
DEFA4 P12838 VAR_036315 p.Arg74Gln US rs762433303 A colorectal cancer sample
DEFA4 P12838 VAR_048861 p.Ala8Pro LB/B rs28661751 -
DEFA4 P12838 VAR_061132 p.Ala8Val LB/B rs28488529 -

DEFA5 Q01523 VAR_059245 p.Arg71His LB/B rs7839771 -
DEFB1 P60022 VAR_014925 p.Ala48Val LB/B rs1800967 -
DEFB1 P60022 VAR_014926 p.Cys67Ser LB/B rs1800968 -
DEFB1 P60022 VAR_018405 p.Val38Ile LB/B rs2738047 -
DEFB104A Q8WTQ1 VAR_024767 p.Ile10Val LB/B rs2680507 -
DEFB108B Q8NET1 VAR_048862 p.Arg36Trp LB/B rs12793731 -
DEFB108B Q8NET1 VAR_059246 p.Ile27Val LB/B rs12285436 -
DEFB108B Q8NET1 VAR_059247 p.His45Leu LB/B rs12285495 -
DEFB108B Q8NET1 VAR_059248 p.Gln53Arg LB/B rs7120658 -
DEFB116 Q30KQ4 VAR_048863 p.Gln19Leu LB/B rs6119768 -
DEFB118 Q96PH6 VAR_061133 p.Ile56Val LB/B rs34173055 -
DEFB125 Q8N687 VAR_061134 p.His62Tyr LB/B rs41276376 -
DEFB127 Q9H1M4 VAR_048864 p.Gly31Arg LB/B rs12624954 -
DEFB127 Q9H1M4 VAR_048865 p.Arg71Ser LB/B rs16995685 -
DEFB128 Q7Z7B8 VAR_048866 p.Lys27Asn LB/B rs4813043 -
DEFB129 Q9H1M3 VAR_024327 p.Thr149Ser LB/B rs1053783 -
DEGS1 O15121 VAR_082594 p.Met37Thr US - Leukodystrophy, hypomyelinating, 18 (HLD18) [MIM:618404]
DEGS1 O15121 VAR_082596 p.Asn113Asp LP/P - Leukodystrophy, hypomyelinating, 18 (HLD18) [MIM:618404]
DEGS1 O15121 VAR_082597 p.His132Arg US - Leukodystrophy, hypomyelinating, 18 (HLD18) [MIM:618404]
DEGS1 O15121 VAR_082598 p.Arg133Trp LP/P - Leukodystrophy, hypomyelinating, 18 (HLD18) [MIM:618404]
DEGS1 O15121 VAR_082600 p.Asn189Asp US - Leukodystrophy, hypomyelinating, 18 (HLD18) [MIM:618404]
DEGS1 O15121 VAR_082601 p.Asn255Ser LP/P - Leukodystrophy, hypomyelinating, 18 (HLD18) [MIM:618404]
DEGS1 O15121 VAR_082602 p.Ala280Val LP/P - Leukodystrophy, hypomyelinating, 18 (HLD18) [MIM:618404]
DEGS2 Q6QHC5 VAR_055698 p.Ala57Thr LB/B rs4905937 -
DEGS2 Q6QHC5 VAR_060347 p.Ser8Asn LB/B rs7157599 -
DEK P35659 VAR_050949 p.Val140Ala LB/B rs17336208 -
DELE1 Q14154 VAR_018272 p.Phe128Leu LB/B rs10036567 -
DELE1 Q14154 VAR_018273 p.Ala247Thr LB/B rs351260 -
DELE1 Q14154 VAR_028197 p.Ala95Thr LB/B rs17850821 -
DELE1 Q14154 VAR_028198 p.Arg468Cys LB/B rs10056676 -
DELE1 Q14154 VAR_055940 p.Gly60Asp LB/B rs34438707 -
DELEC1 Q9P2X7 VAR_050948 p.Ala60Val LB/B rs2269700 -
DENND1B Q6P3S1 VAR_035055 p.Val216Met LB/B rs7546381 -
DENND1C Q8IV53 VAR_035056 p.Ala23Thr LB/B rs10416003 -
DENND1C Q8IV53 VAR_035057 p.Arg489Cys LB/B rs35001260 -
DENND1C Q8IV53 VAR_035058 p.Ala542Val LB/B rs35810378 -
DENND2A Q9ULE3 VAR_026856 p.Pro156His LB/B rs269243 -
DENND2A Q9ULE3 VAR_026857 p.Glu729Lys LB/B rs2293177 -
DENND2A Q9ULE3 VAR_026858 p.Ile777Thr LB/B rs6464833 -
DENND2B P78524 VAR_027101 p.Lys316Asn LB/B rs3794153 -
DENND2B P78524 VAR_027102 p.Asp399Glu LB/B rs3812762 -
DENND2B P78524 VAR_027103 p.Ile620Thr LB/B rs17853683 -

DENND2B P78524 VAR_027104 p.Ser774Gly LB/B rs17853682 -
DENND2B P78524 VAR_030642 p.Ser657Phe LB/B rs11042047 -
DENND2C Q68D51 VAR_026859 p.Asp2Glu LB/B rs7541738 -
DENND2C Q68D51 VAR_026860 p.Asp170Gly LB/B rs12136548 -
DENND2D Q9H6A0 VAR_050950 p.Ser282Asn LB/B rs35742969 -
DENND3 A2RUS2 VAR_035053 p.Ser143Asn LB/B rs307761 -
DENND3 A2RUS2 VAR_035054 p.Gln364Arg LB/B rs11997191 -
DENND4A Q7Z401 VAR_025362 p.Leu284Pro LB/B rs17854146 -
DENND4C Q5VZ89 VAR_022891 p.Thr1266Ala LB/B rs17818730 -
DENND4C Q5VZ89 VAR_022892 p.Asn1343His LB/B rs6475322 -
DENND4C Q5VZ89 VAR_061640 p.Val1227Gly LB/B rs34267952 -
DENND5A Q6IQ26 VAR_028409 p.Glu224Gln LB/B rs952374 -
DENND5A Q6IQ26 VAR_078555 p.Asp541Gly US rs1057519309 Developmental and epileptic encephalopathy 49 (DEE49) [MIM:617281]
DENND5B Q6ZUT9 VAR_040076 p.Arg52Lys LB/B rs4930979 -
DENND5B Q6ZUT9 VAR_040077 p.His487Asn LB/B rs1056320 -
DEPDC1 Q5TB30 VAR_059798 p.Ile404Arg LB/B rs3790479 -
DEPDC1B Q8WUY9 VAR_031819 p.Arg332Met LB/B rs17856590 -
DEPDC1B Q8WUY9 VAR_031820 p.Val395Leu LB/B rs17851707 -
DEPDC4 Q8N2C3 VAR_031815 p.His122Arg LB/B rs7307415 -
DEPDC5 O75140 VAR_024338 p.Ser491Thr LB/B rs8138516 -
DEPDC5 O75140 VAR_053953 p.Ala641Val LB/B rs16989528 -
DEPDC5 O75140 VAR_053954 p.Ser712Phe LB/B rs16989535 -
DEPDC5 O75140 VAR_069263 p.Ala452Val LP/P rs202226316 Epilepsy, familial focal, with variable foci 1 (FFEVF1) [MIM:604364]
DEPDC5 O75140 VAR_069264 p.Arg485Gln LP/P rs886039278 Epilepsy, familial focal, with variable foci 1 (FFEVF1) [MIM:604364]
DEPDC5 O75140 VAR_069265 p.Ser1073Arg LP/P rs754608531 Epilepsy, familial focal, with variable foci 1 (FFEVF1) [MIM:604364]
DEPDC5 O75140 VAR_069266 p.Ser1104Leu US rs79027628 Epilepsy, familial focal, with variable foci 1 (FFEVF1) [MIM:604364]
DEPDC5 O75140 VAR_072363 p.Val90Ile LP/P rs768456731 Epilepsy, familial focal, with variable foci 1 (FFEVF1) [MIM:604364]
DEPDC5 O75140 VAR_072364 p.Val272Leu LP/P rs187334123 Epilepsy, familial focal, with variable foci 1 (FFEVF1) [MIM:604364]
DEPDC5 O75140 VAR_072365 p.Thr864Met LP/P rs564667614 Epilepsy, familial focal, with variable foci 1 (FFEVF1) [MIM:604364]
DEPDC5 O75140 VAR_072366 p.Ser1162Gly LP/P rs886039280 Epilepsy, familial focal, with variable foci 1 (FFEVF1) [MIM:604364]
DEPDC5 O75140 VAR_077128 p.His214Asp US rs886039276 Epilepsy, familial focal, with variable foci 1 (FFEVF1) [MIM:604364]
DEPDC5 O75140 VAR_077129 p.Gln542Pro US rs886039279 Epilepsy, familial focal, with variable foci 1 (FFEVF1) [MIM:604364]
DEPDC5 O75140 VAR_077130 p.Lys1065Arg US rs757609394 Epilepsy, familial focal, with variable foci 1 (FFEVF1) [MIM:604364]
DEPDC5 O75140 VAR_077131 p.Thr1081Pro US rs142540948 Epilepsy, familial focal, with variable foci 1 (FFEVF1) [MIM:604364]
DEPDC5 O75140 VAR_077132 p.Ser1154Phe US rs578244490 Epilepsy, familial focal, with variable foci 1 (FFEVF1) [MIM:604364]

DEPDC5 O75140 VAR_077133 p.Arg1268Gln US rs886039281 Epilepsy, familial focal, with variable foci 1 (FFEVF1) [MIM:604364]
DEPDC7 Q96QD5 VAR_053972 p.Ala192Thr LB/B rs34161108 -
DEPDC7 Q96QD5 VAR_062212 p.Thr464Ile LB/B rs17852859 -
DEPP1 Q9NTK1 VAR_050951 p.Ile44Met LB/B rs11555140 -
DEPTOR Q8TB45 VAR_031816 p.Asn148Ser LB/B rs34057546 -
DEPTOR Q8TB45 VAR_031817 p.Asn204Ser LB/B rs2271900 -
DEPTOR Q8TB45 VAR_031818 p.Ser389Asn LB/B rs4871827 -
DERL1 Q9BUN8 VAR_019516 p.Ile171Val LB/B rs2272722 -
DERL3 Q96Q80 VAR_019517 p.Ala211Val LB/B rs1128127 -
DERL3 Q96Q80 VAR_048897 p.Phe149Leu LB/B rs3177243 -
DES P17661 VAR_007900 p.Ala337Pro LP/P rs59962885 Myopathy, myofibrillar, 1 (MFM1) [MIM:601419]
DES P17661 VAR_007901 p.Ala360Pro LP/P rs121913000 Myopathy, myofibrillar, 1 (MFM1) [MIM:601419]
DES P17661 VAR_007902 p.Asn393Ile LP/P rs121913001 Myopathy, myofibrillar, 1 (MFM1) [MIM:601419]
DES P17661 VAR_009189 p.Leu345Pro LP/P rs57639980 Myopathy, myofibrillar, 1 (MFM1) [MIM:601419]
DES P17661 VAR_018771 p.Leu385Pro LP/P rs57955682 Myopathy, myofibrillar, 1 (MFM1) [MIM:601419]
DES P17661 VAR_018772 p.Gln389Pro LP/P rs121913004 Myopathy, myofibrillar, 1 (MFM1) [MIM:601419]
DES P17661 VAR_018773 p.Ile451Met LP/P rs121913002 Cardiomyopathy, dilated 1I (CMD1I) [MIM:604765]
DES P17661 VAR_018773 p.Ile451Met LP/P rs121913002 Myopathy, myofibrillar, 1 (MFM1) [MIM:601419]
DES P17661 VAR_042448 p.Ser2Ile LP/P rs58999456 Myopathy, myofibrillar, 1 (MFM1) [MIM:601419]
DES P17661 VAR_042449 p.Ser46Phe LP/P rs60794845 Myopathy, myofibrillar, 1 (MFM1) [MIM:601419]
DES P17661 VAR_042450 p.Ser46Tyr LP/P rs60794845 Myopathy, myofibrillar, 1 (MFM1) [MIM:601419]
DES P17661 VAR_042451 p.Ala213Val LB/B rs41272699 -
DES P17661 VAR_042452 p.Glu245Asp LP/P rs267607486 Myopathy, myofibrillar, 1 (MFM1) [MIM:601419]
DES P17661 VAR_042453 p.Asn342Asp LP/P rs267607482 Myopathy, myofibrillar, 1 (MFM1) [MIM:601419]
DES P17661 VAR_042454 p.Arg350Pro LP/P rs57965306 Myopathy, myofibrillar, 1 (MFM1) [MIM:601419]
DES P17661 VAR_042454 p.Arg350Pro LP/P rs57965306 Neurogenic scapuloperoneal syndrome Kaeser type (Kaeser syndrome) [MIM:181400]
DES P17661 VAR_042455 p.Arg355Pro LP/P rs61368398 Myopathy, myofibrillar, 1 (MFM1) [MIM:601419]
DES P17661 VAR_042456 p.Ala357Pro LP/P rs58898021 Myopathy, myofibrillar, 1 (MFM1) [MIM:601419]
DES P17661 VAR_042457 p.Leu370Pro LP/P rs59308628 Myopathy, myofibrillar, 1 (MFM1) [MIM:601419]
DES P17661 VAR_042458 p.Arg406Trp LP/P rs121913003 Myopathy, myofibrillar, 1 (MFM1) [MIM:601419]
DES P17661 VAR_042459 p.Thr442Ile LP/P rs121913005 Myopathy, myofibrillar, 1 (MFM1) [MIM:601419]
DES P17661 VAR_042460 p.Lys449Met LP/P - Myopathy, myofibrillar, 1 (MFM1) [MIM:601419]
DES P17661 VAR_042461 p.Lys449Thr LP/P rs267607485 Myopathy, myofibrillar, 1 (MFM1) [MIM:601419]
DES P17661 VAR_042462 p.Arg454Trp LP/P rs267607490 Myopathy, myofibrillar, 1 (MFM1) [MIM:601419]
DES P17661 VAR_042463 p.Ser460Ile LP/P rs267607491 Myopathy, myofibrillar, 1 (MFM1) [MIM:601419]
DES P17661 VAR_067207 p.Ser7Phe LP/P rs903985237 Myopathy, myofibrillar, 1 (MFM1) [MIM:601419]
DES P17661 VAR_067208 p.Ser13Phe LP/P rs62636495 Myopathy, myofibrillar, 1 (MFM1) [MIM:601419]
DES P17661 VAR_067209 p.Leu338Arg LP/P rs57496341 Myopathy, myofibrillar, 1 (MFM1) [MIM:601419]
DES P17661 VAR_067210 p.Asp399Tyr LP/P rs61130669 Myopathy, myofibrillar, 1 (MFM1) [MIM:601419]
DES P17661 VAR_067211 p.Glu401Lys LP/P rs57694264 Myopathy, myofibrillar, 1 (MFM1) [MIM:601419]
DES P17661 VAR_069074 p.Pro419Ser LP/P rs62635763 Myopathy, myofibrillar, 1 (MFM1) [MIM:601419]
DES P17661 VAR_069191 p.Asn116Ser LP/P rs267607499 Myopathy, myofibrillar, 1 (MFM1) [MIM:601419]
DES P17661 VAR_069192 p.Lys241Glu LB/B rs201945924 -
DES P17661 VAR_075228 p.Ala120Asp LP/P - Cardiomyopathy, dilated 1I (CMD1I) [MIM:604765]
DES P17661 VAR_075229 p.Leu136Pro LP/P rs397516695 Cardiomyopathy, dilated 1I (CMD1I) [MIM:604765]
DES P17661 VAR_075230 p.His326Arg US - Cardiomyopathy, dilated 1I (CMD1I) [MIM:604765]
DES P17661 VAR_079048 p.Arg16Cys LP/P rs60798368 Myopathy, myofibrillar, 1 (MFM1) [MIM:601419]

DES P17661 VAR_079049 p.Thr453Ile LP/P rs267607488 Myopathy, myofibrillar, 1 (MFM1) [MIM:601419]
DEUP1 Q05D60 VAR_050759 p.Ser175Phe LB/B rs34205920 -
DEUP1 Q05D60 VAR_050760 p.Glu440Gln LB/B rs12288277 -
DEUP1 Q05D60 VAR_050761 p.Gln504Lys LB/B rs2259633 -
DEUP1 Q05D60 VAR_059601 p.Glu440Gly LB/B rs12282288 -
DFFB O76075 VAR_009305 p.Arg196Lys LB/B rs12738235 -
DFFB O76075 VAR_048737 p.Lys277Arg LB/B rs12564400 -
DGAT2 Q96PD7 VAR_033864 p.Arg317Gly LB/B rs34421064 -
DGAT2 Q96PD7 VAR_033865 p.Met361Ile LB/B rs34113941 -
DGAT2 Q96PD7 VAR_077236 p.Tyr223His US rs869025595 -
DGCR2 P98153 VAR_020046 p.Val473Ala LB/B rs2072123 -
DGCR6 Q14129 VAR_033866 p.Ala117Val LB/B rs16983281 -
DGCR6L Q9BY27 VAR_055870 p.Val115Leu LB/B rs1056818 -
DGCR6L Q9BY27 VAR_055871 p.Asn195Lys LB/B rs1056804 -
DGCR8 Q8WYQ5 VAR_050952 p.Ile174Val LB/B rs35987994 -
DGCR8 Q8WYQ5 VAR_050953 p.Asn725Asp LB/B rs11546015 -
DGKA P23743 VAR_031563 p.His538Tyr LB/B rs17852990 -
DGKB Q9Y6T7 VAR_048858 p.Gly789Ser LB/B rs34616903 -
DGKE P52429 VAR_036120 p.Leu99Arg US - A breast cancer sample
DGKE P52429 VAR_069804 p.Arg63Pro LP/P rs312262694 Hemolytic uremic syndrome atypical 7 (AHUS7) [MIM:615008]
DGKE P52429 VAR_069805 p.Arg273Pro LP/P rs312262695 Hemolytic uremic syndrome atypical 7 (AHUS7) [MIM:615008]
DGKG P49619 VAR_020259 p.Thr142Ser LB/B rs1004588 -
DGKG P49619 VAR_020260 p.Arg370Trp LB/B rs3213770 -
DGKG P49619 VAR_024430 p.Arg316Lys LB/B rs2193587 -
DGKG P49619 VAR_036119 p.Glu706Lys US - A breast cancer sample
DGKH Q86XP1 VAR_033867 p.Val1201Ala LB/B rs17646069 -
DGKI O75912 VAR_010190 p.Leu153Phe LB/B rs61757580 -
DGKK Q5KSL6 VAR_048859 p.Asp1118Asn LB/B rs4074320 -
DGKQ P52824 VAR_058478 p.Pro27Leu LB/B rs17855876 -
DGKZ Q13574 VAR_069059 p.Gln523Lys LB/B rs17854149 -
DGLUCY Q7Z3D6 VAR_018738 p.Arg10Cys LB/B rs10142502 -
DGLUCY Q7Z3D6 VAR_018739 p.Asp502Asn LB/B rs2295524 -
DGLUCY Q7Z3D6 VAR_052599 p.Ser237Asn LB/B rs34302825 -
DGLUCY Q7Z3D6 VAR_052600 p.Ala372Thr LB/B rs12895348 -
DGLUCY Q7Z3D6 VAR_052601 p.Asp507Asn LB/B rs34523602 -
DGLUCY Q7Z3D6 VAR_052602 p.Gly583Asp LB/B rs34748911 -
DGUOK Q16854 VAR_019417 p.Arg142Lys LP/P rs104893631 Mitochondrial DNA depletion syndrome 3 (MTDPS3) [MIM:251880]
DGUOK Q16854 VAR_019418 p.Glu227Lys LP/P rs104893632 Mitochondrial DNA depletion syndrome 3 (MTDPS3) [MIM:251880]
DGUOK Q16854 VAR_023789 p.Leu250Ser LP/P rs749464475 Mitochondrial DNA depletion syndrome 3 (MTDPS3) [MIM:251880]
DGUOK Q16854 VAR_076979 p.Glu44Lys LP/P rs762550967 Progressive external ophthalmoplegia with mitochondrial DNA deletions, autosomal recessive 4 (PEOB4) [MIM:617070]
DGUOK Q16854 VAR_076980 p.Asn46Ser LP/P rs763615602 Portal hypertension, non-cirrhotic (NCPH) [MIM:617068]

DGUOK Q16854 VAR_076981 p.Asn154Lys LP/P rs144181978 Progressive external ophthalmoplegia with mitochondrial DNA deletions, autosomal recessive 4 (PEOB4) [MIM:617070]
DGUOK Q16854 VAR_076982 p.Gln170Arg US rs74874677 -
DGUOK Q16854 VAR_076983 p.Leu266Arg LB/B rs886037846 -
DHCR24 Q15392 VAR_012732 p.Glu191Lys LP/P rs119475041 Desmosterolosis (DESMOS) [MIM:602398]
DHCR24 Q15392 VAR_012733 p.Asn294Thr LP/P rs281797257 Desmosterolosis (DESMOS) [MIM:602398]
DHCR24 Q15392 VAR_012734 p.Lys306Asn LP/P rs281797256 Desmosterolosis (DESMOS) [MIM:602398]
DHCR24 Q15392 VAR_012735 p.Tyr471Ser LP/P rs28939092 Desmosterolosis (DESMOS) [MIM:602398]
DHCR24 Q15392 VAR_081889 p.Arg94His LP/P rs387906939 Desmosterolosis (DESMOS) [MIM:602398]
DHCR24 Q15392 VAR_081890 p.Glu480Lys LP/P rs387906940 Desmosterolosis (DESMOS) [MIM:602398]
DHCR7 Q9UBM7 VAR_012717 p.Pro51Ser LP/P rs104886035 Smith-Lemli-Opitz syndrome (SLOS) [MIM:270400]
DHCR7 Q9UBM7 VAR_012718 p.Thr93Met LP/P rs80338853 Smith-Lemli-Opitz syndrome (SLOS) [MIM:270400]
DHCR7 Q9UBM7 VAR_012719 p.Leu99Pro LP/P rs104886041 Smith-Lemli-Opitz syndrome (SLOS) [MIM:270400]
DHCR7 Q9UBM7 VAR_012720 p.His119Leu LP/P rs28938174 Smith-Lemli-Opitz syndrome (SLOS) [MIM:270400]
DHCR7 Q9UBM7 VAR_012721 p.Leu157Pro LP/P rs753960624 Smith-Lemli-Opitz syndrome (SLOS) [MIM:270400]
DHCR7 Q9UBM7 VAR_012722 p.Gly244Arg LP/P rs121909764 Smith-Lemli-Opitz syndrome (SLOS) [MIM:270400]
DHCR7 Q9UBM7 VAR_012723 p.Ala247Val LP/P rs886041354 Smith-Lemli-Opitz syndrome (SLOS) [MIM:270400]
DHCR7 Q9UBM7 VAR_012724 p.Trp248Cys LP/P rs104894212 Smith-Lemli-Opitz syndrome (SLOS) [MIM:270400]
DHCR7 Q9UBM7 VAR_012725 p.Thr289Ile LP/P rs121909765 Smith-Lemli-Opitz syndrome (SLOS) [MIM:270400]
DHCR7 Q9UBM7 VAR_012726 p.Val326Leu LP/P rs80338859 Smith-Lemli-Opitz syndrome (SLOS) [MIM:270400]
DHCR7 Q9UBM7 VAR_012727 p.Arg352Trp LP/P rs80338860 Smith-Lemli-Opitz syndrome (SLOS) [MIM:270400]
DHCR7 Q9UBM7 VAR_012728 p.Cys380Ser LP/P - Smith-Lemli-Opitz syndrome (SLOS) [MIM:270400]
DHCR7 Q9UBM7 VAR_012729 p.Arg404Cys LP/P rs61757582 Smith-Lemli-Opitz syndrome (SLOS) [MIM:270400]
DHCR7 Q9UBM7 VAR_012730 p.Gly410Ser LP/P rs80338862 Smith-Lemli-Opitz syndrome (SLOS) [MIM:270400]
DHCR7 Q9UBM7 VAR_016975 p.Glu448Lys LP/P rs80338864 Smith-Lemli-Opitz syndrome (SLOS) [MIM:270400]
DHCR7 Q9UBM7 VAR_023148 p.Leu68Pro LP/P rs104886038 Smith-Lemli-Opitz syndrome (SLOS) [MIM:270400]
DHCR7 Q9UBM7 VAR_023149 p.Gln107His LP/P rs104886040 Smith-Lemli-Opitz syndrome (SLOS) [MIM:270400]
DHCR7 Q9UBM7 VAR_023150 p.Leu109Pro LP/P rs121912195 Smith-Lemli-Opitz syndrome (SLOS) [MIM:270400]
DHCR7 Q9UBM7 VAR_023151 p.Ser113Cys LP/P - Smith-Lemli-Opitz syndrome (SLOS) [MIM:270400]
DHCR7 Q9UBM7 VAR_023152 p.Gly138Val LP/P - Smith-Lemli-Opitz syndrome (SLOS) [MIM:270400]
DHCR7 Q9UBM7 VAR_023153 p.Ile145Leu LP/P rs1555146475 Smith-Lemli-Opitz syndrome (SLOS) [MIM:270400]

DHCR7	Q9UBM7	VAR_023154	p.Gly147Asp	LP/P	rs777425801	Smith-Lemli-Opitz syndrome (SLOS) [MIM:270400]
DHCR7	Q9UBM7	VAR_023155	p.Thr154Met	LP/P	rs143312232	Smith-Lemli-Opitz syndrome (SLOS) [MIM:270400]
DHCR7	Q9UBM7	VAR_023156	p.Ser169Leu	LP/P	rs80338855	Smith-Lemli-Opitz syndrome (SLOS) [MIM:270400]
DHCR7	Q9UBM7	VAR_023157	p.Trp182Cys	LP/P	-	Smith-Lemli-Opitz syndrome (SLOS) [MIM:270400]
DHCR7	Q9UBM7	VAR_023158	p.Trp182Leu	LP/P	rs536394774	Smith-Lemli-Opitz syndrome (SLOS) [MIM:270400]
DHCR7	Q9UBM7	VAR_023159	p.Cys183Tyr	LP/P	-	Smith-Lemli-Opitz syndrome (SLOS) [MIM:270400]
DHCR7	Q9UBM7	VAR_023160	p.Lys198Glu	LP/P	-	Smith-Lemli-Opitz syndrome (SLOS) [MIM:270400]
DHCR7	Q9UBM7	VAR_023161	p.Phe235Ser	LP/P	rs1555146061	Smith-Lemli-Opitz syndrome (SLOS) [MIM:270400]
DHCR7	Q9UBM7	VAR_023162	p.Arg242Cys	LP/P	rs80338856	Smith-Lemli-Opitz syndrome (SLOS) [MIM:270400]
DHCR7	Q9UBM7	VAR_023163	p.Arg242His	LP/P	rs80338857	Smith-Lemli-Opitz syndrome (SLOS) [MIM:270400]
DHCR7	Q9UBM7	VAR_023164	p.Phe255Leu	LP/P	-	Smith-Lemli-Opitz syndrome (SLOS) [MIM:270400]
DHCR7	Q9UBM7	VAR_023165	p.Val281Met	LP/P	rs398123607	Smith-Lemli-Opitz syndrome (SLOS) [MIM:270400]
DHCR7	Q9UBM7	VAR_023166	p.Ile297Thr	LP/P	-	Smith-Lemli-Opitz syndrome (SLOS) [MIM:270400]
DHCR7	Q9UBM7	VAR_023167	p.Cys311Gly	LP/P	-	Smith-Lemli-Opitz syndrome (SLOS) [MIM:270400]
DHCR7	Q9UBM7	VAR_023168	p.Cys311Tyr	LP/P	-	Smith-Lemli-Opitz syndrome (SLOS) [MIM:270400]
DHCR7	Q9UBM7	VAR_023169	p.Tyr324His	LP/P	rs1173707321	Smith-Lemli-Opitz syndrome (SLOS) [MIM:270400]
DHCR7	Q9UBM7	VAR_023170	p.Gly344Arg	LP/P	-	Smith-Lemli-Opitz syndrome (SLOS) [MIM:270400]
DHCR7	Q9UBM7	VAR_023171	p.Arg352Gln	LP/P	rs121909768	Smith-Lemli-Opitz syndrome (SLOS) [MIM:270400]
DHCR7	Q9UBM7	VAR_023172	p.Val353Ala	LP/P	-	Smith-Lemli-Opitz syndrome (SLOS) [MIM:270400]
DHCR7	Q9UBM7	VAR_023173	p.Arg362Cys	LP/P	rs371302153	Smith-Lemli-Opitz syndrome (SLOS) [MIM:270400]
DHCR7	Q9UBM7	VAR_023174	p.Cys380Arg	LP/P	rs373306653	Smith-Lemli-Opitz syndrome (SLOS) [MIM:270400]
DHCR7	Q9UBM7	VAR_023175	p.Cys380Tyr	LP/P	rs779709646	Smith-Lemli-Opitz syndrome (SLOS) [MIM:270400]
DHCR7	Q9UBM7	VAR_023176	p.Ser397Leu	LP/P	rs773134475	Smith-Lemli-Opitz syndrome (SLOS) [MIM:270400]
DHCR7	Q9UBM7	VAR_023177	p.Arg404Ser	LP/P	rs61757582	Smith-Lemli-Opitz syndrome (SLOS) [MIM:270400]
DHCR7	Q9UBM7	VAR_023178	p.His405Tyr	LP/P	-	Smith-Lemli-Opitz syndrome (SLOS) [MIM:270400]
DHCR7	Q9UBM7	VAR_023179	p.Tyr408His	LP/P	rs1046560765	Smith-Lemli-Opitz syndrome (SLOS) [MIM:270400]
DHCR7	Q9UBM7	VAR_023180	p.Gly410Arg	LP/P	rs80338862	Smith-Lemli-Opitz syndrome (SLOS) [MIM:270400]
DHCR7	Q9UBM7	VAR_023181	p.His426Pro	LP/P	rs1354718634	Smith-Lemli-Opitz syndrome (SLOS) [MIM:270400]
DHCR7	Q9UBM7	VAR_023182	p.Arg443Cys	LP/P	rs535561852	Smith-Lemli-Opitz syndrome (SLOS) [MIM:270400]

DHCR7 Q9UBM7 VAR_023183 p.Arg446Gln LP/P rs751604696 Smith-Lemli-Opitz syndrome (SLOS) [MIM:270400]
DHCR7 Q9UBM7 VAR_023184 p.Glu448Gln LP/P rs80338864 Smith-Lemli-Opitz syndrome (SLOS) [MIM:270400]
DHCR7 Q9UBM7 VAR_023185 p.Arg450Leu LP/P rs542266962 Smith-Lemli-Opitz syndrome (SLOS) [MIM:270400]
DHCR7 Q9UBM7 VAR_052154 p.Gly425Ser LB/B rs760242 -
DHCR7 Q9UBM7 VAR_067456 p.Ser5Leu LB/B rs1127869 -
DHCR7 Q9UBM7 VAR_074180 p.Cys118Arg LB/B - -
DHDDS Q86SQ9 VAR_028088 p.Val253Met LB/B rs3816539 -
DHDDS Q86SQ9 VAR_065356 p.Lys42Glu LP/P rs147394623 Retinitis pigmentosa 59 (RP59) [MIM:613861]
DHDDS Q86SQ9 VAR_080708 p.Arg37His US rs1553121073 Developmental delay and seizures with or without movement abnormalities (DEDSM) [MIM:617836]
DHDDS Q86SQ9 VAR_080709 p.Arg211Gln US rs1553122926 Developmental delay and seizures with or without movement abnormalities (DEDSM) [MIM:617836]
DHDH Q9UQ10 VAR_038174 p.Ala2Pro LB/B rs10401800 -
DHDH Q9UQ10 VAR_038175 p.Ser66Asn LB/B rs2270941 -
DHDH Q9UQ10 VAR_038176 p.Val200Met LB/B rs35453148 -
DHDH Q9UQ10 VAR_038177 p.Val247Ala LB/B rs11666105 -
DHDH Q9UQ10 VAR_038178 p.Gly282Arg LB/B rs3765148 -
DHFR P00374 VAR_065818 p.Leu80Phe LP/P rs387906619 Megaloblastic anemia due to dihydrofolate reductase deficiency (DHFRD) [MIM:613839]
DHFR P00374 VAR_065819 p.Asp153Val LP/P rs121913223 Megaloblastic anemia due to dihydrofolate reductase deficiency (DHFRD) [MIM:613839]
DHFR2 Q86XF0 VAR_061135 p.Val166Ile LB/B rs17855824 -
DHH O43323 VAR_054873 p.Leu162Pro LP/P rs111033589 46,XY sex reversal 7 (SRXY7) [MIM:233420]
DHODH Q02127 VAR_022094 p.Lys7Gln LB/B rs3213422 -
DHODH Q02127 VAR_062412 p.Gly19Glu LP/P rs267606765 Postaxial acrofacial dysostosis (POADS) [MIM:263750]
DHODH Q02127 VAR_062413 p.Arg135Cys LP/P rs201230446 Postaxial acrofacial dysostosis (POADS) [MIM:263750]
DHODH Q02127 VAR_062414 p.Gly152Arg LP/P rs267606766 Postaxial acrofacial dysostosis (POADS) [MIM:263750]
DHODH Q02127 VAR_062415 p.Arg199Cys LP/P rs267606769 Postaxial acrofacial dysostosis (POADS) [MIM:263750]
DHODH Q02127 VAR_062416 p.Gly202Ala LP/P rs267606767 Postaxial acrofacial dysostosis (POADS) [MIM:263750]
DHODH Q02127 VAR_062417 p.Gly202Asp LP/P rs267606767 Postaxial acrofacial dysostosis (POADS) [MIM:263750]
DHODH Q02127 VAR_062418 p.Arg244Trp LP/P rs267606768 Postaxial acrofacial dysostosis (POADS) [MIM:263750]
DHODH Q02127 VAR_062419 p.Thr284Ile LP/P - Postaxial acrofacial dysostosis (POADS) [MIM:263750]
DHODH Q02127 VAR_062420 p.Arg346Trp LP/P rs201947120 Postaxial acrofacial dysostosis (POADS) [MIM:263750]
DHODH Q02127 VAR_062421 p.Asp392Gly LP/P rs779076692 Postaxial acrofacial dysostosis (POADS) [MIM:263750]
DHPS P49366 VAR_043005 p.Glu174Asp LB/B rs10425108 -
DHPS P49366 VAR_082649 p.Asn173Ser LP/P rs758100382 Neurodevelopmental disorder with seizures and speech and walking impairment (NEDSSWI) [MIM:618480]

DHRS1 Q96LJ7 VAR_052318 p.Thr241Ile LB/B rs10134537 -
DHRS13 Q6UX07 VAR_037348 p.Arg191Gln LB/B rs2277666 -
DHRS13 Q6UX07 VAR_037349 p.Leu336Gln LB/B rs4795472 -
DHRS2 Q13268 VAR_035846 p.Ala272Val US rs148991912 A colorectal cancer sample
DHRS3 O75911 VAR_067443 p.Val2Ala LB/B rs1128251 -
DHRS4 Q9BTZ2 VAR_057272 p.Ala31Thr LB/B rs1043442 -
DHRS4 Q9BTZ2 VAR_061846 p.Pro202Ser LB/B rs1043650 -
DHRS4L2 Q6PKH6 VAR_037395 p.Leu17Met LB/B rs2273947 -
DHRS7 Q9Y394 VAR_052319 p.Arg218Gln LB/B rs34583017 -
DHRS7C A6NNS2 VAR_043150 p.Ser227Leu LB/B rs2280490 -
DHRS9 Q9BPW9 VAR_052320 p.Asp286His LB/B rs11695788 -
DHRSX Q8N5I4 VAR_016100 p.Glu297Lys LB/B rs12010 -
DHRSX Q8N5I4 VAR_055354 p.Val247Leu LB/B rs1127915 -
DHRSX Q8N5I4 VAR_055355 p.His292Arg LB/B rs3210910 -
DHTKD1 Q96HY7 VAR_036715 p.Phe20Leu LB/B rs1279138 -
DHTKD1 Q96HY7 VAR_036716 p.Tyr272Asp LB/B rs3740015 -
DHTKD1 Q96HY7 VAR_036717 p.Arg308Leu LB/B rs17849603 -
DHTKD1 Q96HY7 VAR_036718 p.Asn350Asp LB/B rs34716552 -
DHTKD1 Q96HY7 VAR_036719 p.Ile607Met LB/B rs2062988 -
DHTKD1 Q96HY7 VAR_069585 p.Gly729Arg LP/P rs117225135 2-aminoadipic 2-oxoadipic aciduria (AMOXAD) [MIM:204750]
DHX16 O60231 VAR_057236 p.Lys352Glu LB/B rs17189239 -
DHX16 O60231 VAR_057237 p.Leu502Phe LB/B rs17189232 -
DHX16 O60231 VAR_057238 p.Asp566Gly LB/B rs9262138 -
DHX16 O60231 VAR_083621 p.Gly427Glu LP/P - Neuromuscular oculoauditory syndrome (NMOAS) [MIM:618733]
DHX16 O60231 VAR_083622 p.Phe582Ile US - Neuromuscular oculoauditory syndrome (NMOAS) [MIM:618733]
DHX16 O60231 VAR_083623 p.Thr674Met US - Neuromuscular oculoauditory syndrome (NMOAS) [MIM:618733]
DHX16 O60231 VAR_083624 p.Gln697His US - Neuromuscular oculoauditory syndrome (NMOAS) [MIM:618733]
DHX29 Q7Z478 VAR_026985 p.Pro630His LB/B rs17854904 -
DHX29 Q7Z478 VAR_052180 p.Asp309Ala LB/B rs35874395 -
DHX30 Q7L2E3 VAR_080611 p.Arg493His LP/P rs1057519436 Neurodevelopmental disorder with severe motor impairment and absent language (NEDMIAL) [MIM:617804]
DHX30 Q7L2E3 VAR_080612 p.His562Arg LP/P rs1060499733 Neurodevelopmental disorder with severe motor impairment and absent language (NEDMIAL) [MIM:617804]
DHX30 Q7L2E3 VAR_080613 p.Gly781Asp LP/P rs1553706775 Neurodevelopmental disorder with severe motor impairment and absent language (NEDMIAL) [MIM:617804]
DHX30 Q7L2E3 VAR_080614 p.Arg782Trp LP/P rs753242774 Neurodevelopmental disorder with severe motor impairment and absent language (NEDMIAL) [MIM:617804]
DHX30 Q7L2E3 VAR_080615 p.Arg785Cys LP/P rs1085307451 Neurodevelopmental disorder with severe motor impairment and absent language (NEDMIAL) [MIM:617804]
DHX30 Q7L2E3 VAR_080616 p.Arg785His LP/P rs1553706799 Neurodevelopmental disorder with severe motor impairment and absent language (NEDMIAL) [MIM:617804]
DHX32 Q7L7V1 VAR_035843 p.Pro209Arg US - A breast cancer sample
DHX32 Q7L7V1 VAR_052181 p.Glu271Asp LB/B rs11244674 -
DHX32 Q7L7V1 VAR_052182 p.Asp301Ala LB/B rs35772239 -

DHX32 Q7L7V1 VAR_052183 p.Val430Leu LB/B rs17153669 -
DHX33 Q9H6R0 VAR_057239 p.Arg118Cys LB/B rs8069315 -
DHX33 Q9H6R0 VAR_057240 p.His483Asp LB/B rs11653658 -
DHX34 Q14147 VAR_057241 p.Arg17Trp LB/B rs12984558 -
DHX34 Q14147 VAR_057242 p.Gly117Asp LB/B rs8113564 -
DHX34 Q14147 VAR_083626 p.Asn441Ser US rs549149043 -
DHX34 Q14147 VAR_083627 p.Arg776Pro US rs764483792 -
DHX35 Q9H5Z1 VAR_020211 p.Pro703Leu LB/B rs3752302 -
DHX35 Q9H5Z1 VAR_052184 p.Ile189Thr LB/B rs36053162 -
DHX36 Q9H2U1 VAR_027140 p.Glu151Lys LB/B rs1058299 -
DHX36 Q9H2U1 VAR_027141 p.Ser416Cys LB/B rs9438 -
DHX36 Q9H2U1 VAR_027142 p.Ile583Asn LB/B rs17853513 -
DHX37 Q8IY37 VAR_052185 p.Arg458Gln LB/B rs11057939 -
DHX37 Q8IY37 VAR_052186 p.Lys508Asn LB/B rs35165507 -
DHX37 Q8IY37 VAR_052187 p.Val717Ile LB/B rs35016004 -
DHX37 Q8IY37 VAR_052188 p.Ser869Gly LB/B rs4516060 -
DHX37 Q8IY37 VAR_052189 p.Arg1081Gln LB/B rs4447263 -
DHX37 Q8IY37 VAR_061826 p.Met96Ile LB/B rs11558556 -
DHX37 Q8IY37 VAR_083628 p.Arg93Gln US rs575837056 Neurodevelopmental disorder with brain anomalies and with or without vertebral or cardiac anomalies (NEDBAVC) [MIM:618731]
DHX37 Q8IY37 VAR_083629 p.Glu167Ser US - Neurodevelopmental disorder with brain anomalies and with or without vertebral or cardiac anomalies (NEDBAVC) [MIM:618731]
DHX37 Q8IY37 VAR_083630 p.Thr304Met LP/P - 46,XY sex reversal 11 (SRXY11) [MIM:273250]
DHX37 Q8IY37 VAR_083631 p.Arg308Gln LP/P rs1384892917 46,XY sex reversal 11 (SRXY11) [MIM:273250]
DHX37 Q8IY37 VAR_083632 p.Arg334Leu US - 46,XY sex reversal 11 (SRXY11) [MIM:273250]
DHX37 Q8IY37 VAR_083633 p.Arg334Trp US - 46,XY sex reversal 11 (SRXY11) [MIM:273250]
DHX37 Q8IY37 VAR_083634 p.Asp382Gly US rs1424699115 Neurodevelopmental disorder with brain anomalies and with or without vertebral or cardiac anomalies (NEDBAVC) [MIM:618731]
DHX37 Q8IY37 VAR_083635 p.Asn419Lys US rs1060499737 Neurodevelopmental disorder with brain anomalies and with or without vertebral or cardiac anomalies (NEDBAVC) [MIM:618731]
DHX37 Q8IY37 VAR_083636 p.Leu467Val US rs149331610 Neurodevelopmental disorder with brain anomalies and with or without vertebral or cardiac anomalies (NEDBAVC) [MIM:618731]
DHX37 Q8IY37 VAR_083637 p.Arg487His US rs779613772 Neurodevelopmental disorder with brain anomalies and with or without vertebral or cardiac anomalies (NEDBAVC) [MIM:618731]
DHX37 Q8IY37 VAR_083638 p.Ser595Phe LP/P - 46,XY sex reversal 11 (SRXY11) [MIM:273250]
DHX37 Q8IY37 VAR_083639 p.Ser626Leu US - 46,XY sex reversal 11 (SRXY11) [MIM:273250]
DHX37 Q8IY37 VAR_083640 p.Arg674Gln LP/P - 46,XY sex reversal 11 (SRXY11) [MIM:273250]
DHX37 Q8IY37 VAR_083641 p.Arg674Trp LP/P - 46,XY sex reversal 11 (SRXY11) [MIM:273250]
DHX37 Q8IY37 VAR_083642 p.Val731Met US rs754186165 Neurodevelopmental disorder with brain anomalies and with or without vertebral or cardiac anomalies (NEDBAVC) [MIM:618731]
DHX37 Q8IY37 VAR_083643 p.Gly1030Glu US rs754141645 46,XY sex reversal 11 (SRXY11) [MIM:273250]
DHX37 Q8IY37 VAR_083644 p.Thr1094Met US rs1277857720 Neurodevelopmental disorder with brain anomalies and with or without vertebral or cardiac anomalies (NEDBAVC) [MIM:618731]
DHX38 Q92620 VAR_015518 p.Thr1217Ala LB/B - -
DHX38 Q92620 VAR_081338 p.Arg324Gln LP/P rs766053952 Retinitis pigmentosa 84 (RP84) [MIM:618220]
DHX38 Q92620 VAR_081339 p.Gly332Asp US rs587777554 Retinitis pigmentosa 84 (RP84) [MIM:618220]
DHX57 Q6P158 VAR_033861 p.Ser433Gly LB/B rs35371077 -
DHX57 Q6P158 VAR_052190 p.Ser410Phe LB/B rs11893062 -
DHX57 Q6P158 VAR_052191 p.Asn587Ser LB/B rs7598922 -

DHX58 Q96C10 VAR_019394 p.Gln425Arg LB/B rs2074158 -
DHX58 Q96C10 VAR_019395 p.Arg523Gln LB/B rs2074160 -
DHX58 Q96C10 VAR_049336 p.Thr76Ala LB/B rs34891485 -
DHX58 Q96C10 VAR_049337 p.Arg95Gln LB/B rs35118457 -
DHX58 Q96C10 VAR_083645 p.Trp8Cys US rs200502586 -
DHX8 Q14562 VAR_052174 p.Ala1069Gly LB/B rs34285079 -
DHX8 Q14562 VAR_083620 p.Arg168Trp US - -
DHX9 Q08211 VAR_052179 p.Ile894Val LB/B rs1049264 -
DIABLO Q9NR28 VAR_066487 p.Ser126Leu LP/P rs387906893 Deafness, autosomal dominant, 64 (DFNA64) [MIM:614152]
DIAPH1 O60610 VAR_079874 p.Pro678Ser LP/P rs186370335 Deafness, autosomal dominant 1, with or without thrombocytopenia (DFNA1) [MIM:124900]
DIAPH2 O60879 VAR_049095 p.Phe425Leu LB/B rs20361 -
DIAPH2 O60879 VAR_049096 p.Leu426Val LB/B rs20361 -
DIAPH3 Q9NSV4 VAR_049097 p.Asn363Ser LB/B rs36084898 -
DIAPH3 Q9NSV4 VAR_049098 p.Phe773Leu LB/B rs35579086 -
DIAPH3 Q9NSV4 VAR_049099 p.Glu1041Gly LB/B rs7491389 -
DICER1 Q9UPY3 VAR_063150 p.Leu1583Arg LP/P rs137852976 Pleuropulmonary blastoma (PPB) [MIM:601200]
DICER1 Q9UPY3 VAR_065301 p.Ser839Phe LP/P rs387906934 Goiter multinodular 1, with or without Sertoli-Leydig cell tumors (MNG1) [MIM:138800]
DICER1 Q9UPY3 VAR_067091 p.Glu1705Lys US - Non-epithelial ovarian tumor
DICER1 Q9UPY3 VAR_067092 p.Asp1709Glu US - Non-epithelial ovarian tumor
DICER1 Q9UPY3 VAR_067093 p.Asp1709Gly US rs1555366979 Non-epithelial ovarian tumor
DICER1 Q9UPY3 VAR_067094 p.Asp1709Asn US - Non-epithelial ovarian tumor
DICER1 Q9UPY3 VAR_067095 p.Asp1810His US - Non-epithelial ovarian tumor
DICER1 Q9UPY3 VAR_067096 p.Asp1810Asn US rs775912475 Non-epithelial ovarian tumor
DICER1 Q9UPY3 VAR_067097 p.Asp1810Tyr US - Non-epithelial ovarian tumor
DICER1 Q9UPY3 VAR_067098 p.Glu1813Gly US - Non-epithelial ovarian tumor
DICER1 Q9UPY3 VAR_067099 p.Glu1813Lys US - Non-epithelial ovarian tumor
DICER1 Q9UPY3 VAR_067100 p.Glu1813Gln US - Non-epithelial ovarian tumor
DICER1 Q9UPY3 VAR_081917 p.Pro435Leu US - -
DICER1 Q9UPY3 VAR_081918 p.Asp1709Tyr LP/P - Global developmental delay, lung cysts, overgrowth, and Wilms tumor (GLOW) [MIM:618272]
DICER1 Q9UPY3 VAR_081919 p.Asp1713Val LP/P - Global developmental delay, lung cysts, overgrowth, and Wilms tumor (GLOW) [MIM:618272]
DICER1 Q9UPY3 VAR_081920 p.Arg1898Gly US - -
DIDO1 Q9BTC0 VAR_028310 p.Pro13Leu LB/B rs6090161 -
DIDO1 Q9BTC0 VAR_028311 p.Pro276Leu LB/B rs6090160 -
DIDO1 Q9BTC0 VAR_057093 p.Met544Thr LB/B rs1883848 -
DIDO1 Q9BTC0 VAR_057094 p.Ala556Thr LB/B rs1883847 -
DIDO1 Q9BTC0 VAR_057095 p.Ala793Gly LB/B rs750077 -
DIDO1 Q9BTC0 VAR_057096 p.Pro1220Gln LB/B rs6011441 -
DIDO1 Q9BTC0 VAR_061740 p.Ser1708Cys LB/B rs41282984 -
DIO2 Q92813 VAR_047549 p.Thr92Ala LB/B rs225014 -
DIO2 Q92813 VAR_049640 p.Ala81Asp LB/B rs2839859 -
DIP2A Q14689 VAR_047372 p.Pro191Ala LB/B rs7283507 -
DIP2A Q14689 VAR_047373 p.Ser372Asn LB/B rs16979312 -
DIP2B Q9P265 VAR_038861 p.Ile792Val LB/B rs11169525 -
DIP2C Q9Y2E4 VAR_035905 p.Ala586Glu US - A breast cancer sample

DIP2C	Q9Y2E4	VAR_035906	p.Gly622Ser	US	rs771008735	A colorectal cancer sample
DIP2C	Q9Y2E4	VAR_035907	p.Val1264Met	US	rs929297365	A breast cancer sample
DIPK1B	Q5VUD6	VAR_032295	p.Ser158Gly	LB/B	rs945384	-
DIPK2B	Q9H7Y0	VAR_047103	p.Arg128Lys	LB/B	rs1132201	-
DIPK2B	Q9H7Y0	VAR_047104	p.Arg146Gln	LB/B	rs9969	-
DIRC1	Q969H9	VAR_022549	p.Ser51Ala	LB/B	rs72902678	-
DIRC1	Q969H9	VAR_061641	p.Leu92Ser	LB/B	rs58846152	-
DIS3	Q9Y2L1	VAR_023099	p.Asn269Ser	LB/B	rs4883918	-
DIS3	Q9Y2L1	VAR_023100	p.Thr326Arg	LB/B	rs7332388	-
DIS3L	Q8TF46	VAR_038056	p.Ile518Val	LB/B	rs34668776	-
DIS3L	Q8TF46	VAR_038057	p.Asp614Gly	LB/B	rs3803412	-
DIS3L	Q8TF46	VAR_038058	p.Asn747Ser	LB/B	rs17258507	-
DIS3L2	Q8IYB7	VAR_038059	p.Pro12Ser	LB/B	rs723044	-
DIS3L2	Q8IYB7	VAR_067577	p.Arg483Gly	LP/P	rs186865544	-
DIS3L2	Q8IYB7	VAR_067578	p.Cys489Tyr	LP/P	rs387907116	Perlman syndrome (PRLMNS) [MIM:267000]
DIS3L2	Q8IYB7	VAR_067579	p.Arg576His	LP/P	rs200386096	-
DIS3L2	Q8IYB7	VAR_079527	p.Met443Lys	US	-	Perlman syndrome (PRLMNS) [MIM:267000]
DISC1	Q9NRI5	VAR_022437	p.Arg264Gln	LB/B	rs3738401	-
DISC1	Q9NRI5	VAR_022438	p.Ser704Cys	LB/B	rs821616	-
DISC1	Q9NRI5	VAR_026704	p.Leu607Phe	LB/B	rs6675281	-
DISC1	Q9NRI5	VAR_030422	p.Gly5Val	LB/B	rs3738400	-
DISC1	Q9NRI5	VAR_050954	p.Leu330Phe	LB/B	rs34622148	-
DISC1	Q9NRI5	VAR_061642	p.Ala116Val	LB/B	rs56020408	-
DISC1	Q9NRI5	VAR_061643	p.Thr328Asn	LB/B	rs55795950	-
DISC1	Q9NRI5	VAR_061644	p.Gly531Arg	LB/B	rs56229136	-
DISP1	Q96F81	VAR_037077	p.Glu103Lys	LB/B	rs2609383	-
DISP2	A7MBM2	VAR_037078	p.Pro47Ala	LB/B	rs1898883	-
DISP2	A7MBM2	VAR_037079	p.Cys56Ser	LB/B	rs1898882	-
DISP2	A7MBM2	VAR_037080	p.Gly388Glu	LB/B	rs35043215	-
DISP2	A7MBM2	VAR_037081	p.Gly1145Ser	LB/B	rs2412512	-
DISP2	A7MBM2	VAR_037082	p.Arg1247Trp	LB/B	rs3743142	-
DISP3	Q9P2K9	VAR_036796	p.Leu51Val	LB/B	rs3738159	-
DISP3	Q9P2K9	VAR_036797	p.Gly182Ser	LB/B	rs2817580	-
DISP3	Q9P2K9	VAR_036798	p.Ala650Thr	LB/B	rs2072994	-
DISP3	Q9P2K9	VAR_036799	p.Gly661Ala	LB/B	rs2072993	-
DISP3	Q9P2K9	VAR_036800	p.Arg948His	LB/B	rs12096312	-
DISP3	Q9P2K9	VAR_061496	p.Gly39Arg	LB/B	rs41274528	-
DIXDC1	Q155Q3	VAR_032294	p.Lys300Arg	LB/B	rs34575249	-
DKC1	O60832	VAR_006811	p.Phe36Val	LP/P	rs121912293	Dyskeratosis congenita, X-linked (DKCX) [MIM:305000]
DKC1	O60832	VAR_006813	p.Pro40Arg	LP/P	rs121912292	Dyskeratosis congenita, X-linked (DKCX) [MIM:305000]
DKC1	O60832	VAR_006814	p.Leu72Tyr	LP/P	rs121912294	Dyskeratosis congenita, X-linked (DKCX) [MIM:305000]
DKC1	O60832	VAR_006815	p.Gly402Glu	LP/P	rs121912295	Dyskeratosis congenita, X-linked (DKCX) [MIM:305000]
DKC1	O60832	VAR_009264	p.Ala353Val	LP/P	rs121912288	Dyskeratosis congenita, X-linked (DKCX) [MIM:305000]

DKC1 O60832 VAR_009264 p.Ala353Val LP/P rs121912288 Hoyeraal-Hreidarsson syndrome (HHS) [MIM:305000]
DKC1 O60832 VAR_010076 p.Ala2Val LP/P rs121912303 Dyskeratosis congenita, X-linked (DKCX) [MIM:305000]
DKC1 O60832 VAR_010077 p.Lys39Glu LP/P rs121912296 Dyskeratosis congenita, X-linked (DKCX) [MIM:305000]
DKC1 O60832 VAR_010078 p.Glu41Lys LP/P rs121912302 Dyskeratosis congenita, X-linked (DKCX) [MIM:305000]
DKC1 O60832 VAR_010079 p.Arg65Thr LP/P rs121912301 Dyskeratosis congenita, X-linked (DKCX) [MIM:305000]
DKC1 O60832 VAR_010080 p.Thr66Ala LP/P rs121912297 Dyskeratosis congenita, X-linked (DKCX) [MIM:305000]
DKC1 O60832 VAR_010081 p.Leu321Val LP/P rs2728726 Dyskeratosis congenita, X-linked (DKCX) [MIM:305000]
DKC1 O60832 VAR_010082 p.Met350Ile LP/P rs121912298 Dyskeratosis congenita, X-linked (DKCX) [MIM:305000]
DKC1 O60832 VAR_010083 p.Met350Thr LP/P rs121912300 Dyskeratosis congenita, X-linked (DKCX) [MIM:305000]
DKC1 O60832 VAR_010084 p.Gly402Arg LP/P rs121912299 Dyskeratosis congenita, X-linked (DKCX) [MIM:305000]
DKC1 O60832 VAR_015674 p.Ile38Thr LP/P rs28936072 Hoyeraal-Hreidarsson syndrome (HHS) [MIM:305000]
DKC1 O60832 VAR_015675 p.Thr49Met LP/P rs121912304 Hoyeraal-Hreidarsson syndrome (HHS) [MIM:305000]
DKC1 O60832 VAR_015676 p.Ser121Gly LP/P rs121912305 Hoyeraal-Hreidarsson syndrome (HHS) [MIM:305000]
DKC1 O60832 VAR_022553 p.Gly223Asp LB/B rs2728533 -
DKC1 O60832 VAR_063821 p.Leu56Ser LP/P rs121912287 Dyskeratosis congenita, X-linked (DKCX) [MIM:305000]
DKC1 O60832 VAR_063822 p.Leu72Phe LP/P rs121912306 Dyskeratosis congenita, X-linked (DKCX) [MIM:305000]
DKC1 O60832 VAR_063823 p.Leu317Phe LP/P rs121912290 Dyskeratosis congenita, X-linked (DKCX) [MIM:305000]
DKC1 O60832 VAR_063824 p.Arg322Gln LP/P rs121912291 Dyskeratosis congenita, X-linked (DKCX) [MIM:305000]
DKC1 O60832 VAR_063825 p.Pro409Leu LP/P rs121912289 Dyskeratosis congenita, X-linked (DKCX) [MIM:305000]
DKC1 O60832 VAR_080707 p.Leu54Val LP/P - Dyskeratosis congenita, X-linked (DKCX) [MIM:305000]
DKK2 Q9UBU2 VAR_021966 p.Arg146Gln LB/B rs17037102 -
DKK3 Q9UBP4 VAR_030787 p.Arg335Gly LB/B rs3206824 -
DKK3 Q9UBP4 VAR_057516 p.Glu49Asp LB/B rs11544816 -
DKKL1 Q9UK85 VAR_021967 p.Met109Arg LB/B rs2303759 -
DKKL1 Q9UK85 VAR_021968 p.Glu214Lys LB/B rs2288481 -
DKKL1 Q9UK85 VAR_024432 p.Ala29Thr LB/B rs919364 -
DKKL1 Q9UK85 VAR_053061 p.Leu24Arg LB/B rs2303757 -
DKKL1 Q9UK85 VAR_053062 p.Leu104Ile LB/B rs35693137 -
DKKL1 Q9UK85 VAR_053063 p.Gly187Ser LB/B rs1054770 -
DLAT P10515 VAR_047410 p.Ala43Val LB/B rs2303436 -
DLAT P10515 VAR_047411 p.Ser98Phe LB/B rs537057 -

DLAT P10515 VAR_047412 p.Leu99Phe LB/B rs537060 -
DLAT P10515 VAR_047413 p.Gln209Arg LB/B rs11553595 -
DLAT P10515 VAR_047414 p.Asp313Val LB/B rs11553592 -
DLAT P10515 VAR_047415 p.Val318Ala LB/B rs627441 -
DLAT P10515 VAR_047416 p.Asp451Asn LB/B rs10891314 -
DLC1 Q96QB1 VAR_014229 p.Asn712Ser LB/B rs1044092 -
DLC1 Q96QB1 VAR_014230 p.Val791Met LB/B rs532841 -
DLC1 Q96QB1 VAR_014231 p.Thr959Ala LB/B rs121908500 -
DLC1 Q96QB1 VAR_014232 p.His998Gln LB/B rs149295187 -
DLC1 Q96QB1 VAR_014233 p.Val1025Ala LB/B - -
DLC1 Q96QB1 VAR_014234 p.Glu1199Val LB/B rs1044093 -
DLC1 Q96QB1 VAR_014235 p.Ser1209Cys LB/B rs1044094 -
DLC1 Q96QB1 VAR_059293 p.Arg27Cys LB/B rs34575560 -
DLC1 Q96QB1 VAR_059294 p.Leu81Val LB/B rs3816748 -
DLC1 Q96QB1 VAR_059295 p.Gln254His LB/B rs11203495 -
DLC1 Q96QB1 VAR_059296 p.Asn255Asp LB/B rs11203494 -
DLC1 Q96QB1 VAR_059297 p.Thr260Ile LB/B rs3816747 -
DLC1 Q96QB1 VAR_059298 p.Gln320His LB/B rs34591797 -
DLD P09622 VAR_006907 p.Lys72Glu LP/P rs121964987 Dihydrolipoamide dehydrogenase deficiency (DLDD) [MIM:246900]
DLD P09622 VAR_006908 p.Pro488Leu LP/P rs121964988 Dihydrolipoamide dehydrogenase deficiency (DLDD) [MIM:246900]
DLD P09622 VAR_014555 p.Leu331Val LB/B rs17624 -
DLD P09622 VAR_015820 p.Gly229Cys LP/P rs121964990 Dihydrolipoamide dehydrogenase deficiency (DLDD) [MIM:246900]
DLD P09622 VAR_015821 p.Arg495Gly LP/P rs121964989 Dihydrolipoamide dehydrogenase deficiency (DLDD) [MIM:246900]
DLD P09622 VAR_031922 p.Lys104Thr LB/B rs1130477 -
DLD P09622 VAR_076985 p.Ile47Thr LP/P rs397514651 Dihydrolipoamide dehydrogenase deficiency (DLDD) [MIM:246900]
DLD P09622 VAR_076987 p.Met361Val LP/P rs121964993 Dihydrolipoamide dehydrogenase deficiency (DLDD) [MIM:246900]
DLD P09622 VAR_076988 p.Glu375Lys LP/P rs121964992 Dihydrolipoamide dehydrogenase deficiency (DLDD) [MIM:246900]
DLD P09622 VAR_076989 p.Ile393Thr LP/P rs121964991 Dihydrolipoamide dehydrogenase deficiency (DLDD) [MIM:246900]
DLD P09622 VAR_076990 p.Asp479Val LP/P rs397514649 Dihydrolipoamide dehydrogenase deficiency (DLDD) [MIM:246900]
DLD P09622 VAR_076991 p.Arg482Gly LP/P rs397514650 Dihydrolipoamide dehydrogenase deficiency (DLDD) [MIM:246900]
DLEC1 Q9Y238 VAR_035908 p.Pro351Arg US - A breast cancer sample
DLEC1 Q9Y238 VAR_056860 p.Leu79Arg LB/B rs7625806 -
DLEC1 Q9Y238 VAR_056861 p.Ser192Phe LB/B rs34012183 -
DLEC1 Q9Y238 VAR_056862 p.Lys1022Asn LB/B rs36012922 -
DLEC1 Q9Y238 VAR_056863 p.Asn1150Asp LB/B rs9840172 -
DLEC1 Q9Y238 VAR_056864 p.Leu1227Pro LB/B rs9810085 -
DLEU7 Q6UYE1 VAR_031280 p.Ala83Val LB/B rs898861 -
DLG1 Q12959 VAR_054334 p.Lys140Arg LB/B rs1802668 -
DLG1 Q12959 VAR_054335 p.Arg278Gln LB/B rs1134986 -

DLG1	Q12959	VAR_054336	p.Pro899Leu	LB/B	rs34492126	-
DLG3	Q92796	VAR_036591	p.Gly40Arg	US	rs1449722258	A colorectal cancer sample
DLG5	Q8TDM6	VAR_027897	p.Gln140Arg	LB/B	rs1248696	-
DLG5	Q8TDM6	VAR_027898	p.Pro1481Gln	LB/B	rs2289310	-
DLG5	Q8TDM6	VAR_027899	p.Ala1600Val	LB/B	rs4979794	-
DLGAP1	O14490	VAR_053648	p.Arg816Gln	LB/B	rs35822832	-
DLGAP2	Q9P1A6	VAR_023073	p.Pro419Gln	LB/B	rs2301963	-
DLGAP2	Q9P1A6	VAR_036750	p.Thr620Met	LB/B	rs7463888	-
DLGAP3	O95886	VAR_079371	p.Thr763Pro	LB/B	rs758215471	-
DLGAP4	Q9Y2H0	VAR_057716	p.Ala486Thr	LB/B	rs6019652	-
DLGAP4	Q9Y2H0	VAR_057717	p.Arg861Gln	LB/B	rs2275807	-
DLGAP5	Q15398	VAR_023774	p.Gly69Glu	LB/B	rs2274271	-
DLGAP5	Q15398	VAR_057718	p.Gln324His	LB/B	rs8010791	-
DLGAP5	Q15398	VAR_057719	p.Thr469Ile	LB/B	rs17128275	-
DLGAP5	Q15398	VAR_062147	p.Glu753Gln	LB/B	rs35954941	-
DLK1	P80370	VAR_055715	p.Gln73Leu	LB/B	rs34686110	-
DLK1	P80370	VAR_055716	p.Val104Met	LB/B	rs2273607	-
DLK1	P80370	VAR_055717	p.Ser260Asn	LB/B	rs1058009	-
DLK1	P80370	VAR_060335	p.Arg101Gly	LB/B	rs6575799	-
DLK1	P80370	VAR_060336	p.Asp108Gly	LB/B	rs1058006	-
DLK2	Q6UY11	VAR_048977	p.Gly301Arg	LB/B	rs35192247	-
DLL1	O00548	VAR_048976	p.Val444Met	LB/B	rs16901311	-
DLL1	O00548	VAR_083468	p.Cys179Phe	LP/P	-	Neurodevelopmental disorder with non-specific brain abnormalities and with or without seizures (NEDBAS) [MIM:618709]
DLL3	Q9NYJ7	VAR_009952	p.Gly385Asp	LP/P	rs104894674	Spondylocostal dysostosis 1, autosomal recessive (SCDO1) [MIM:277300]
DLL3	Q9NYJ7	VAR_016776	p.Leu218Pro	LB/B	rs1110627	-
DLL3	Q9NYJ7	VAR_046782	p.Ala115Thr	LB/B	rs71647811	-
DLL3	Q9NYJ7	VAR_046783	p.Leu142Gln	LB/B	rs55741253	-
DLL3	Q9NYJ7	VAR_046784	p.Phe172Cys	LB/B	rs8107127	-
DLL4	Q9NR61	VAR_075858	p.Ala121Pro	LP/P	rs796065350	Adams-Oliver syndrome 6 (AOS6) [MIM:616589]
DLL4	Q9NR61	VAR_075859	p.Arg186Cys	LP/P	rs796065348	Adams-Oliver syndrome 6 (AOS6) [MIM:616589]
DLL4	Q9NR61	VAR_075860	p.Phe195Leu	LP/P	rs796065351	Adams-Oliver syndrome 6 (AOS6) [MIM:616589]
DLL4	Q9NR61	VAR_075861	p.Pro267Thr	LP/P	rs796065349	Adams-Oliver syndrome 6 (AOS6) [MIM:616589]
DLL4	Q9NR61	VAR_075862	p.Cys390Arg	LP/P	rs796065347	Adams-Oliver syndrome 6 (AOS6) [MIM:616589]
DLL4	Q9NR61	VAR_075863	p.Cys390Tyr	LP/P	rs796065346	Adams-Oliver syndrome 6 (AOS6) [MIM:616589]
DLL4	Q9NR61	VAR_075864	p.Cys455Trp	LP/P	rs796065345	Adams-Oliver syndrome 6 (AOS6) [MIM:616589]
DLST	P36957	VAR_004976	p.Pro213Ala	LB/B	-	-
DLST	P36957	VAR_004977	p.Pro384Thr	LB/B	-	-
DLST	P36957	VAR_083034	p.Arg231Gln	US	rs771616810	Paragangliomas 7 (PGL7) [MIM:618475]
DLST	P36957	VAR_083035	p.Asp304Asn	LB/B	rs373295097	-
DLST	P36957	VAR_083036	p.Gly374Glu	LP/P	rs1270341616	Paragangliomas 7 (PGL7) [MIM:618475]
DLST	P36957	VAR_083037	p.Tyr422Cys	US	rs778239022	Paragangliomas 7 (PGL7) [MIM:618475]
DLX1	P56177	VAR_028443	p.Ser136Cys	LB/B	rs17853565	-
DLX5	P56178	VAR_033874	p.Ser234Arg	LB/B	rs35273378	-
DLX5	P56178	VAR_067413	p.Gln178Pro	LP/P	rs387906737	Split-hand/foot malformation 1 with sensorineural hearing loss, autosomal recessive (SHFM1D) [MIM:220600]
DMAC1	Q96GE9	VAR_056819	p.Pro28Thr	LB/B	rs1127430	-
DMAC2	Q9NW81	VAR_057798	p.Arg9Cys	LB/B	rs2231938	-

DMAC2	Q9NW81	VAR_057799	p.His23Arg	LB/B	rs2231939	-
DMAC2	Q9NW81	VAR_057800	p.Asn34Ser	LB/B	rs2231940	-
DMAC2	Q9NW81	VAR_057801	p.Glu230Lys	LB/B	rs2231943	-
DMAC2	Q9NW81	VAR_060165	p.Cys159Ser	LB/B	rs1043413	-
DMAC2L	Q99766	VAR_060296	p.Pro18Leu	LB/B	rs2275592	-
DMBT1	Q9UGM3	VAR_024788	p.Pro42Thr	LB/B	rs11523871	-
DMBT1	Q9UGM3	VAR_024789	p.Ser52Trp	LB/B	rs75209396	-
DMBT1	Q9UGM3	VAR_024790	p.Ser54Leu	LB/B	rs3013236	-
DMBT1	Q9UGM3	VAR_024791	p.Val60Ala	LB/B	-	-
DMBT1	Q9UGM3	VAR_024792	p.Pro65Leu	LB/B	rs185045706	-
DMBT1	Q9UGM3	VAR_024793	p.Gln337Leu	LB/B	-	-
DMBT1	Q9UGM3	VAR_024794	p.Pro357Ser	LB/B	rs141757453	-
DMBT1	Q9UGM3	VAR_024795	p.Arg364Gly	LB/B	-	-
DMBT1	Q9UGM3	VAR_024796	p.Gln420His	US	rs104894156	A glioma sample
DMBT1	Q9UGM3	VAR_024797	p.Gly607Val	US	-	A glioma sample
DMBT1	Q9UGM3	VAR_024798	p.Thr649Met	LB/B	rs189478437	-
DMBT1	Q9UGM3	VAR_024799	p.Arg656Trp	LB/B	rs989419742	-
DMBT1	Q9UGM3	VAR_024800	p.Thr780Met	LB/B	rs199704744	-
DMBT1	Q9UGM3	VAR_024801	p.Pro856Ser	LB/B	rs144450471	-
DMBT1	Q9UGM3	VAR_024802	p.His1084Tyr	LB/B	rs2277244	-
DMBT1	Q9UGM3	VAR_024803	p.Met1169Thr	LB/B	rs149099696	-
DMBT1	Q9UGM3	VAR_024804	p.Arg1176Trp	LB/B	rs761527369	-
DMBT1	Q9UGM3	VAR_024805	p.Val1545Met	LB/B	rs189221852	-
DMBT1	Q9UGM3	VAR_024806	p.His1732Ser	US	-	-
DMBT1	Q9UGM3	VAR_024807	p.Thr1961Pro	LB/B	-	-
DMBT1	Q9UGM3	VAR_044417	p.Asn322Asp	LB/B	rs1969620	-
DMBT1	Q9UGM3	VAR_044418	p.Arg1860Leu	LB/B	rs7099177	-
DMBT1	Q9UGM3	VAR_052994	p.Arg670Cys	LB/B	rs2277237	-
DMBT1	Q9UGM3	VAR_052995	p.Thr719Met	LB/B	rs2277238	-
DMBT1	Q9UGM3	VAR_057981	p.Gly162Glu	US	rs200664624	A glioma cell line
DMBT1	Q9UGM3	VAR_057982	p.Asn546Ser	US	rs200713568	A glioma cell line
DMBT1	Q9UGM3	VAR_057983	p.Ser1095Pro	LB/B	rs200551848	-
DMBT1	Q9UGM3	VAR_057984	p.Ser1102Thr	LB/B	rs566926424	-
DMBT1	Q9UGM3	VAR_057985	p.Arg1434Trp	LB/B	-	-
DMBT1	Q9UGM3	VAR_057986	p.Val2255Met	LB/B	rs183135544	-
DMBT1	Q9UGM3	VAR_080764	p.Thr969Arg	US	rs1195952221	-
DMBX1	Q8NFW5	VAR_049578	p.Ala205Pro	LB/B	rs34614765	-
DMBX1	Q8NFW5	VAR_082142	p.Arg123Trp	US	rs730882203	-
DMC1	Q14565	VAR_018960	p.Met200Val	LB/B	rs2227914	-
DMC1	Q14565	VAR_061757	p.Gly150Asp	LB/B	rs58396845	-
DMD	P11532	VAR_005147	p.Leu54Arg	LP/P	-	Duchenne muscular dystrophy (DMD) [MIM:310200]
DMD	P11532	VAR_005148	p.Gln133Pro	LB/B	rs1800256	-
DMD	P11532	VAR_005149	p.Ala168Asp	LP/P	-	Becker muscular dystrophy (BMD) [MIM:300376]
DMD	P11532	VAR_005150	p.Tyr231Asn	LP/P	-	Becker muscular dystrophy (BMD) [MIM:300376]
DMD	P11532	VAR_005151	p.Gln365His	LB/B	rs1800266	-
DMD	P11532	VAR_005153	p.Leu623Ile	LB/B	rs1800259	-
DMD	P11532	VAR_005154	p.Lys773Glu	LP/P	-	Duchenne muscular dystrophy (DMD) [MIM:310200]
DMD	P11532	VAR_005155	p.Ala784Gly	LB/B	rs1800260	-
DMD	P11532	VAR_005156	p.Asp882Gly	LB/B	rs228406	-

DMD	P11532	VAR_005157	p.Val1197Phe	LB/B	rs1800262	-
DMD	P11532	VAR_005158	p.Thr1245Ile	LB/B	rs1800269	-
DMD	P11532	VAR_005159	p.Ala1278Pro	LB/B	rs1800270	-
DMD	P11532	VAR_005160	p.Lys1377Asn	LB/B	rs1800263	-
DMD	P11532	VAR_005161	p.Gln1469Leu	LB/B	rs1057872	-
DMD	P11532	VAR_005162	p.Arg1745His	LB/B	rs1801187	-
DMD	P11532	VAR_005163	p.Arg1844Ser	LB/B	rs1801186	-
DMD	P11532	VAR_005164	p.Arg2155Trp	LB/B	rs1800273	-
DMD	P11532	VAR_005165	p.Arg2191Trp	LB/B	-	-
DMD	P11532	VAR_005167	p.Lys2366Gln	LB/B	rs1800275	-
DMD	P11532	VAR_005168	p.Glu2910Val	LB/B	rs41305353	-
DMD	P11532	VAR_005169	p.Asn2912Asp	LB/B	rs1800278	-
DMD	P11532	VAR_005170	p.His2921Arg	LP/P	rs1800279	Becker muscular dystrophy (BMD) [MIM:300376]
DMD	P11532	VAR_005171	p.Gln2937Arg	LB/B	rs1800280	-
DMD	P11532	VAR_005172	p.Ala3421Val	LP/P	-	Becker muscular dystrophy (BMD) [MIM:300376]
DMD	P11532	VAR_023537	p.Lys18Asn	LP/P	-	Cardiomyopathy, dilated, X-linked 3B (CMD3B) [MIM:302045]
DMD	P11532	VAR_023538	p.Asp165Val	US	-	-
DMD	P11532	VAR_023539	p.Ala171Pro	LP/P	-	Becker muscular dystrophy (BMD) [MIM:300376]
DMD	P11532	VAR_023540	p.Thr279Ala	LP/P	-	Cardiomyopathy, dilated, X-linked 3B (CMD3B) [MIM:302045]
DMD	P11532	VAR_023541	p.Asp645Gly	LP/P	-	Duchenne muscular dystrophy (DMD) [MIM:310200]
DMD	P11532	VAR_023542	p.Asn1672Lys	LB/B	rs16990264	-
DMD	P11532	VAR_023543	p.Asn2299Thr	LB/B	-	-
DMD	P11532	VAR_023544	p.Phe3228Leu	LP/P	-	Cardiomyopathy, dilated, X-linked 3B (CMD3B) [MIM:302045]
DMD	P11532	VAR_023545	p.Cys3313Phe	LP/P	-	Duchenne muscular dystrophy (DMD) [MIM:310200]
DMD	P11532	VAR_023546	p.Asp3335His	LP/P	-	Duchenne muscular dystrophy (DMD) [MIM:310200]
DMD	P11532	VAR_023547	p.Cys3340Tyr	LP/P	-	Duchenne muscular dystrophy (DMD) [MIM:310200]
DMD	P11532	VAR_036353	p.Leu334Phe	US	-	A colorectal cancer sample
DMD	P11532	VAR_036354	p.Glu1219Gln	US	-	A breast cancer sample
DMD	P11532	VAR_036355	p.Arg1470His	US	-	A breast cancer sample
DMD	P11532	VAR_036356	p.Ala2164Val	US	-	A colorectal cancer sample
DMD	P11532	VAR_057642	p.Thr409Ser	LB/B	rs34155804	-
DMD	P11532	VAR_057643	p.Ala573Val	LB/B	rs5972599	-
DMD	P11532	VAR_057644	p.Thr715Ser	LB/B	rs16998350	-
DMD	P11532	VAR_057645	p.Thr1136Ser	LB/B	rs3827462	-
DMD	P11532	VAR_057646	p.Phe1388Val	LB/B	rs28715870	-
DMD	P11532	VAR_057647	p.Arg2108Cys	LB/B	rs16990169	-
DMD	P11532	VAR_062110	p.Ser666Leu	LB/B	rs34563188	-
DMD	P11532	VAR_065764	p.Trp118Arg	US	-	-
DMGDH	Q9UI17	VAR_011505	p.His109Arg	LP/P	rs121908331	DMGDH deficiency (DMGDHD) [MIM:605850]
DMGDH	Q9UI17	VAR_014950	p.Ser279Pro	LB/B	rs532964	-
DMGDH	Q9UI17	VAR_014951	p.Ala530Gly	LB/B	rs1805073	-
DMGDH	Q9UI17	VAR_014952	p.Ser646Pro	LB/B	rs1805074	-
DMKN	Q6E0U4	VAR_042720	p.Ala13Asp	US	-	A colorectal cancer sample
DMKN	Q6E0U4	VAR_042721	p.Ala415Ser	LB/B	rs2293696	-
DMKN	Q6E0U4	VAR_047337	p.Asp427Ala	LB/B	rs909072	-
DMKN	Q6E0U4	VAR_056865	p.Glu69Asp	LB/B	rs12460932	-

DMKN Q6E0U4 VAR_056866 p.Asn139Ser LB/B rs7247001 -
DMKN Q6E0U4 VAR_059652 p.Val91Ala LB/B rs4806163 -
DMKN Q6E0U4 VAR_059653 p.Gly280Ser LB/B rs11667007 -
DMP1 Q13316 VAR_030750 p.Ser69Cys LB/B rs10019009 -
DMP1 Q13316 VAR_030751 p.Asp117Asn US rs140719182 One individual with tumoral calcinosis
DMP1 Q13316 VAR_030752 p.Arg272His LB/B rs145237146 -
DMP1 Q13316 VAR_033848 p.Lys463Arg LB/B rs34661425 -
DMPK Q09013 VAR_040452 p.Leu428Val US - A lung small cell carcinoma sample
DMPK Q09013 VAR_058334 p.Leu423Val LB/B rs527221 -
DMRT1 Q9Y5R6 VAR_009954 p.Ser45Thr LB/B rs3739583 -
DMRT1 Q9Y5R6 VAR_009955 p.Tyr221Ser LB/B - -
DMRT1 Q9Y5R6 VAR_009956 p.Arg281Ser LB/B - -
DMRT1 Q9Y5R6 VAR_009957 p.Pro295Leu LB/B rs902482318 -
DMRT2 Q9Y5R5 VAR_067719 p.Ala381Pro LB/B rs3824419 -
DMRT2 Q9Y5R5 VAR_067720 p.Glu458Gln LB/B rs17641078 -
DMRT3 Q9NQL9 VAR_030591 p.Ala164Thr LB/B rs10978001 -
DMRT3 Q9NQL9 VAR_030592 p.Asn261Thr LB/B rs7854621 -
DMRT3 Q9NQL9 VAR_030593 p.Gly356Val LB/B rs16927037 -
DMRTA1 Q5VZB9 VAR_036331 p.Arg342Cys US rs372873186 A colorectal cancer sample
DMTF1 Q9Y222 VAR_039577 p.Val479Ile LB/B rs1558049 -
DMXL1 Q9Y485 VAR_057589 p.Ile221Met LB/B rs7700801 -
DMXL1 Q9Y485 VAR_057590 p.Ser851Asn LB/B rs4895362 -
DMXL1 Q9Y485 VAR_057591 p.Val2067Met LB/B rs9790916 -
DMXL1 Q9Y485 VAR_057592 p.Thr2107Ser LB/B rs9791092 -
DMXL2 Q8TDJ6 VAR_057593 p.Thr497Met LB/B rs17524906 -
DMXL2 Q8TDJ6 VAR_057594 p.Asn903Asp LB/B rs16953073 -
DMXL2 Q8TDJ6 VAR_057595 p.Ser1288Pro LB/B rs12102203 -
DMXL2 Q8TDJ6 VAR_057596 p.Asp1481Gly LB/B rs35349640 -
DMXL2 Q8TDJ6 VAR_062094 p.Glu144Gln LB/B rs35097381 -
DMXL2 Q8TDJ6 VAR_069028 p.Asn1698Ser LB/B rs149028181 -
DMXL2 Q8TDJ6 VAR_079484 p.Arg2417His LP/P - Deafness, autosomal dominant, 71 (DFNA71) [MIM:617605]
DMXL2 Q8TDJ6 VAR_083447 p.Ala1712Val LP/P rs372749193 Developmental and epileptic encephalopathy 81 (DEE81) [MIM:618663]
DNA2 P51530 VAR_069905 p.Arg198His LP/P rs1272393477 Progressive external ophthalmoplegia with mitochondrial DNA deletions, autosomal dominant, 6 (PEOA6) [MIM:615156]
DNA2 P51530 VAR_069906 p.Lys227Glu LP/P rs760412883 Progressive external ophthalmoplegia with mitochondrial DNA deletions, autosomal dominant, 6 (PEOA6) [MIM:615156]
DNA2 P51530 VAR_069907 p.Val637Ile LP/P rs746522359 Progressive external ophthalmoplegia with mitochondrial DNA deletions, autosomal dominant, 6 (PEOA6) [MIM:615156]
DNAAF1 Q8NEP3 VAR_047662 p.Asp387Glu LB/B rs36062234 -
DNAAF1 Q8NEP3 VAR_047663 p.Lys393Arg LB/B rs17856705 -
DNAAF1 Q8NEP3 VAR_047664 p.Glu432Asp LB/B rs9972733 -
DNAAF1 Q8NEP3 VAR_047665 p.Pro502Leu LB/B rs11644164 -
DNAAF1 Q8NEP3 VAR_047666 p.Phe545Cys LB/B rs17856706 -
DNAAF1 Q8NEP3 VAR_047667 p.Leu633Ser LB/B rs2288020 -
DNAAF1 Q8NEP3 VAR_047668 p.Leu659Pro LB/B rs2288022 -
DNAAF1 Q8NEP3 VAR_047669 p.Leu659Val LB/B rs2288021 -
DNAAF1 Q8NEP3 VAR_047670 p.Ser675Thr LB/B rs2288023 -

DNAAF1 Q8NEP3 VAR_047671 p.Gly703Arg LB/B rs4150188 -
DNAAF1 Q8NEP3 VAR_047672 p.Pro712Ala LB/B rs4150187 -
DNAAF1 Q8NEP3 VAR_063098 p.Leu175Arg LP/P rs267607227 Ciliary dyskinesia, primary, 13 (CILD13) [MIM:613193]
DNAAF10 Q96MX6 VAR_053438 p.Met241Val LB/B rs13009282 -
DNAAF11 Q86X45 VAR_023603 p.Thr232Ile LB/B rs2293979 -
DNAAF11 Q86X45 VAR_031223 p.Ile466Thr LB/B rs9297853 -
DNAAF11 Q86X45 VAR_069038 p.Ala74Pro LP/P rs397514596 Ciliary dyskinesia, primary, 19 (CILD19) [MIM:614935]
DNAAF11 Q86X45 VAR_069039 p.Asp146His LP/P rs200321595 Ciliary dyskinesia, primary, 19 (CILD19) [MIM:614935]
DNAAF11 Q86X45 VAR_084433 p.Asn61Lys US - Ciliary dyskinesia, primary, 19 (CILD19) [MIM:614935]
DNAAF11 Q86X45 VAR_084434 p.Cys87Arg LP/P - Ciliary dyskinesia, primary, 19 (CILD19) [MIM:614935]
DNAAF2 Q9NVR5 VAR_024309 p.Asp768Gly LB/B rs9989177 -
DNAAF2 Q9NVR5 VAR_057788 p.Glu62Asp LB/B rs2985684 -
DNAAF3 Q8N9W5 VAR_055306 p.Glu292Gly LB/B rs2365725 -
DNAAF3 Q8N9W5 VAR_055307 p.Gly331Trp LB/B rs7508641 -
DNAAF3 Q8N9W5 VAR_055308 p.Asn365Asp LB/B rs890872 -
DNAAF3 Q8N9W5 VAR_067300 p.Leu61Pro LP/P rs387907151 Ciliary dyskinesia, primary, 2 (CILD2) [MIM:606763]
DNAAF3 Q8N9W5 VAR_067301 p.Ala278Thr LB/B rs200775946 -
DNAAF4 Q8WXU2 VAR_017383 p.Pro2Ser LB/B rs143493699 -
DNAAF4 Q8WXU2 VAR_017384 p.Val91Ile LB/B rs17819126 -
DNAAF4 Q8WXU2 VAR_017385 p.Glu191Gly LB/B rs600753 -
DNAAF4 Q8WXU2 VAR_017386 p.Ser420Cys LB/B rs77641439 -
DNAAF4 Q8WXU2 VAR_026214 p.Asn38Lys LB/B rs16976354 -
DNAAF4 Q8WXU2 VAR_026215 p.Ala332Val LB/B rs17855756 -
DNAAF5 Q86Y56 VAR_056911 p.Val632Ala LB/B rs4720951 -
DNAAF5 Q86Y56 VAR_060463 p.Arg560Cys LB/B rs73258248 -
DNAAF5 Q86Y56 VAR_060464 p.Arg743Lys LB/B rs3922641 -
DNAAF5 Q86Y56 VAR_068969 p.Leu795Pro LP/P rs397514561 Ciliary dyskinesia, primary, 18 (CILD18) [MIM:614874]
DNAAF6 Q9NQM4 VAR_083178 p.Asp133Tyr LP/P - Ciliary dyskinesia, primary, 36, X-linked (CILD36) [MIM:300991]
DNAAF8 Q8IYS4 VAR_033157 p.Glu88Asp LB/B rs17137215 -
DNAAF8 Q8IYS4 VAR_033158 p.Pro143Ser LB/B rs17137230 -
DNAAF8 Q8IYS4 VAR_033159 p.Ser190Leu LB/B rs35599524 -
DNAAF8 Q8IYS4 VAR_033160 p.Glu232Lys LB/B rs35002791 -
DNAAF8 Q8IYS4 VAR_033161 p.Ala241Val LB/B rs7202010 -
DNAAF8 Q8IYS4 VAR_033162 p.Arg302Cys LB/B rs2075469 -
DNAAF8 Q8IYS4 VAR_033163 p.Gln354Arg LB/B rs737700 -
DNAAF8 Q8IYS4 VAR_061618 p.Pro465Leu LB/B rs17853375 -
DNAAF9 Q5TEA3 VAR_050923 p.Thr481Pro LB/B rs16988463 -
DNAAF9 Q5TEA3 VAR_050924 p.Arg577Gly LB/B rs2422864 -
DNAAF9 Q5TEA3 VAR_059637 p.Thr86Met LB/B rs8124486 -
DNAAF9 Q5TEA3 VAR_065913 p.Phe265Leu LB/B rs149767043 -
DNAH1 Q9P2D7 VAR_038912 p.Glu205Asp LB/B rs10460963 -
DNAH1 Q9P2D7 VAR_038913 p.Val441Leu LB/B rs13060192 -
DNAH1 Q9P2D7 VAR_038914 p.Val1502Met LB/B rs17052095 -

DNAH1	Q9P2D7	VAR_038915	p.Arg1663Cys	LB/B	rs17052097	-
DNAH1	Q9P2D7	VAR_062176	p.Asn2384Ser	LB/B	rs56002041	-
DNAH1	Q9P2D7	VAR_064924	p.His3832Arg	LB/B	rs365048	-
DNAH1	Q9P2D7	VAR_079510	p.Lys1154Gln	LP/P	rs544674332	Ciliary dyskinesia, primary, 37 (CILD37) [MIM:617577]
DNAH1	Q9P2D7	VAR_079511	p.Val1287Gly	LP/P	rs1131692251	Spermatogenic failure 18 (SPGF18) [MIM:617576]
DNAH1	Q9P2D7	VAR_079512	p.Asp1293Asn	LP/P	rs140883175	Spermatogenic failure 18 (SPGF18) [MIM:617576]
DNAH1	Q9P2D7	VAR_080890	p.Thr1372Met	US	rs374644342	Spermatogenic failure 18 (SPGF18) [MIM:617576]
DNAH1	Q9P2D7	VAR_080894	p.Leu2071Arg	US	rs757396103	Spermatogenic failure 18 (SPGF18) [MIM:617576]
DNAH1	Q9P2D7	VAR_080895	p.Val2150Met	US	rs778635747	Spermatogenic failure 18 (SPGF18) [MIM:617576]
DNAH1	Q9P2D7	VAR_080898	p.Arg3229Cys	US	-	Spermatogenic failure 18 (SPGF18) [MIM:617576]
DNAH1	Q9P2D7	VAR_080900	p.Arg4096Leu	US	rs748247721	Spermatogenic failure 18 (SPGF18) [MIM:617576]
DNAH1	Q9P2D7	VAR_080901	p.Arg4133Cys	US	rs776800142	Spermatogenic failure 18 (SPGF18) [MIM:617576]
DNAH1	Q9P2D7	VAR_080902	p.Ala4174Thr	US	rs765046623	Spermatogenic failure 18 (SPGF18) [MIM:617576]
DNAH10	Q8IVF4	VAR_038916	p.Ile480Val	LB/B	rs10846559	-
DNAH10	Q8IVF4	VAR_060135	p.Ser167Pro	LB/B	rs11057353	-
DNAH10	Q8IVF4	VAR_060136	p.Thr1724Met	LB/B	rs34934281	-
DNAH10	Q8IVF4	VAR_060137	p.Asp1767Glu	LB/B	rs7969937	-
DNAH10	Q8IVF4	VAR_060138	p.Arg1865Lys	LB/B	rs35685787	-
DNAH10	Q8IVF4	VAR_060139	p.Arg2403Trp	LB/B	rs7977449	-
DNAH10	Q8IVF4	VAR_060140	p.Asn2483Ser	LB/B	rs11835416	-
DNAH10	Q8IVF4	VAR_062177	p.Thr1986Met	LB/B	rs33935373	-
DNAH11	Q96DT5	VAR_013851	p.Glu34Leu	LB/B	rs398123604	-
DNAH11	Q96DT5	VAR_013852	p.Gln639Arg	LB/B	rs12670130	-
DNAH11	Q96DT5	VAR_013853	p.Ser654Cys	LB/B	rs62441683	-
DNAH11	Q96DT5	VAR_013854	p.Val1023Ala	LB/B	rs10269582	-
DNAH11	Q96DT5	VAR_013855	p.Thr1038Ala	LB/B	rs10224537	-
DNAH11	Q96DT5	VAR_013856	p.Asp1635Gly	LB/B	rs17144835	-
DNAH11	Q96DT5	VAR_013857	p.Asn2634Ser	LB/B	rs9639393	-
DNAH11	Q96DT5	VAR_013858	p.Ile2675Val	LB/B	rs72657364	-
DNAH11	Q96DT5	VAR_013859	p.Arg2997Gln	US	rs35865357	Ciliary dyskinesia, primary, 7 (CILD7) [MIM:611884]
DNAH11	Q96DT5	VAR_013860	p.Ala3467Thr	LB/B	rs2214326	-
DNAH11	Q96DT5	VAR_013861	p.Val3708Leu	LB/B	rs4722064	-
DNAH11	Q96DT5	VAR_013862	p.Ser3758Pro	LB/B	rs17145720	-
DNAH11	Q96DT5	VAR_013863	p.Thr4170Ile	LB/B	rs12537531	-
DNAH11	Q96DT5	VAR_042944	p.Glu34Val	LB/B	rs2285944	-
DNAH11	Q96DT5	VAR_042945	p.Met1316Val	LB/B	rs17144788	-
DNAH11	Q96DT5	VAR_042946	p.Met4165Val	LB/B	rs6461613	-
DNAH11	Q96DT5	VAR_060141	p.Tyr2586His	LB/B	rs2003417	-

DNAH11	Q96DT5	VAR_072472	p.Glu117Val	LP/P	rs72655968	Ciliary dyskinesia, primary, 7 (CILD7) [MIM:611884]
DNAH11	Q96DT5	VAR_072473	p.Leu2383Pro	LP/P	rs72657353	Ciliary dyskinesia, primary, 7 (CILD7) [MIM:611884]
DNAH11	Q96DT5	VAR_072474	p.Leu4327Ser	LP/P	rs72658826	Ciliary dyskinesia, primary, 7 (CILD7) [MIM:611884]
DNAH12	Q6ZR08	VAR_034829	p.Val32Ala	LB/B	rs9311651	-
DNAH12	Q6ZR08	VAR_034830	p.Ser139Asn	LB/B	rs6778837	-
DNAH12	Q6ZR08	VAR_034831	p.Asp224Glu	LB/B	rs6445902	-
DNAH12	Q6ZR08	VAR_034832	p.Thr231Ala	LB/B	rs7629743	-
DNAH12	Q6ZR08	VAR_037390	p.Tyr2740Phe	LB/B	rs17057989	-
DNAH12	Q6ZR08	VAR_037391	p.Gly2893Ser	LB/B	rs4060726	-
DNAH12	Q6ZR08	VAR_060142	p.Thr467Pro	LB/B	rs6806444	-
DNAH12	Q6ZR08	VAR_060143	p.Asp1549Asn	LB/B	rs6773904	-
DNAH12	Q6ZR08	VAR_060144	p.Tyr1704His	LB/B	rs4462937	-
DNAH12	Q6ZR08	VAR_060145	p.Arg1748Cys	LB/B	rs17050836	-
DNAH12	Q6ZR08	VAR_060146	p.Lys1754Asn	LB/B	rs17793014	-
DNAH12	Q6ZR08	VAR_060147	p.Thr1763Ile	LB/B	rs4681982	-
DNAH14	Q0VDD8	VAR_032116	p.Thr220Ser	LB/B	rs41267347	-
DNAH14	Q0VDD8	VAR_032117	p.Pro274Leu	LB/B	rs41267349	-
DNAH14	Q0VDD8	VAR_057764	p.Lys2671Glu	LB/B	rs6667999	-
DNAH17	Q9UFH2	VAR_039581	p.Asp492Asn	LB/B	rs34868091	-
DNAH17	Q9UFH2	VAR_062178	p.Ile792Val	LB/B	rs16971526	-
DNAH17	Q9UFH2	VAR_062179	p.Ile963Thr	LB/B	rs11651537	-
DNAH17	Q9UFH2	VAR_062180	p.Met1972Val	LB/B	rs1462210081	-
DNAH17	Q9UFH2	VAR_083233	p.Cys1829Tyr	LP/P	-	Spermatogenic failure 39 (SPGF39) [MIM:618643]
DNAH17	Q9UFH2	VAR_083235	p.Pro3499Leu	LP/P	rs1598474055	Spermatogenic failure 39 (SPGF39) [MIM:618643]
DNAH17	Q9UFH2	VAR_083236	p.Leu3595Pro	LP/P	rs1418782829	Spermatogenic failure 39 (SPGF39) [MIM:618643]
DNAH2	Q9P225	VAR_039407	p.Ala100Val	LB/B	rs35664870	-
DNAH2	Q9P225	VAR_039408	p.Ser312Thr	LB/B	rs3744254	-
DNAH2	Q9P225	VAR_039409	p.Glu1326Gly	LB/B	rs11868946	-
DNAH2	Q9P225	VAR_039410	p.Arg2548His	LB/B	rs11656500	-
DNAH2	Q9P225	VAR_039411	p.Thr3600Ile	LB/B	rs7213894	-
DNAH2	Q9P225	VAR_060134	p.Arg2904His	LB/B	rs2309808	-
DNAH3	Q8TD57	VAR_039412	p.Ile484Leu	US	-	A colorectal cancer sample
DNAH3	Q8TD57	VAR_039413	p.Arg545Trp	LB/B	rs16970910	-
DNAH3	Q8TD57	VAR_039414	p.Ile1565Met	LB/B	rs330150	-
DNAH3	Q8TD57	VAR_039415	p.Val1583Ile	LB/B	rs16970832	-
DNAH3	Q8TD57	VAR_039416	p.Ser1608Phe	US	-	A colorectal cancer sample
DNAH3	Q8TD57	VAR_039417	p.Thr1752Met	LB/B	rs13332291	-
DNAH3	Q8TD57	VAR_039418	p.Ile2399Asn	LB/B	rs34179606	-
DNAH3	Q8TD57	VAR_039419	p.Ile2804Val	LB/B	rs12929546	-
DNAH3	Q8TD57	VAR_039420	p.Lys2949Thr	LB/B	rs33928718	-
DNAH3	Q8TD57	VAR_039421	p.Glu3457Lys	LB/B	rs3743695	-
DNAH3	Q8TD57	VAR_039422	p.Leu3639Ile	LB/B	rs34771199	-
DNAH3	Q8TD57	VAR_039423	p.Arg3645Cys	LB/B	rs12924551	-
DNAH3	Q8TD57	VAR_039424	p.Arg3744Trp	LB/B	rs2301620	-

DNAH5	Q8TE73	VAR_019603	p.His12Gln	LB/B	rs339445	-
DNAH5	Q8TE73	VAR_019604	p.Gly24Glu	LB/B	rs1530496	-
DNAH5	Q8TE73	VAR_019605	p.Thr558Ala	LB/B	rs1530498	-
DNAH5	Q8TE73	VAR_019606	p.Gln2463Arg	LB/B	rs10078391	-
DNAH5	Q8TE73	VAR_019607	p.Leu2862Phe	LB/B	rs10513155	-
DNAH5	Q8TE73	VAR_019608	p.Gly3519Arg	LP/P	rs79967166	Ciliary dyskinesia, primary, 3 (CILD3) [MIM:608644]
DNAH5	Q8TE73	VAR_019609	p.Ala4134Val	LB/B	rs30168	-
DNAH5	Q8TE73	VAR_019610	p.Thr4220Ala	LB/B	rs2277046	-
DNAH5	Q8TE73	VAR_019611	p.Ile4450Val	LB/B	rs3734110	-
DNAH5	Q8TE73	VAR_027903	p.Ile766Leu	LB/B	rs4701997	-
DNAH5	Q8TE73	VAR_027904	p.Ser1006Gly	LB/B	rs16902886	-
DNAH5	Q8TE73	VAR_027905	p.Met1081Val	LB/B	rs16902880	-
DNAH5	Q8TE73	VAR_027906	p.Thr3791Ile	LB/B	rs17263496	-
DNAH5	Q8TE73	VAR_030705	p.Arg1716Leu	LP/P	rs74799487	Ciliary dyskinesia, primary, 3 (CILD3) [MIM:608644]
DNAH5	Q8TE73	VAR_030706	p.Ser2264Asn	LP/P	rs78484669	Ciliary dyskinesia, primary, 3 (CILD3) [MIM:608644]
DNAH5	Q8TE73	VAR_030707	p.Glu2347Lys	LP/P	-	Ciliary dyskinesia, primary, 3 (CILD3) [MIM:608644]
DNAH5	Q8TE73	VAR_030708	p.Arg2501Pro	LP/P	rs78853309	Ciliary dyskinesia, primary, 3 (CILD3) [MIM:608644]
DNAH5	Q8TE73	VAR_030709	p.Phe2843Ser	LP/P	rs77377082	Ciliary dyskinesia, primary, 3 (CILD3) [MIM:608644]
DNAH5	Q8TE73	VAR_030710	p.Trp3409Ser	LP/P	rs755407407	Ciliary dyskinesia, primary, 3 (CILD3) [MIM:608644]
DNAH5	Q8TE73	VAR_030711	p.Ser3843Leu	LP/P	rs78346432	Ciliary dyskinesia, primary, 3 (CILD3) [MIM:608644]
DNAH5	Q8TE73	VAR_030712	p.Gly4205Val	LP/P	rs79185772	Ciliary dyskinesia, primary, 3 (CILD3) [MIM:608644]
DNAH5	Q8TE73	VAR_053840	p.Leu591Arg	LB/B	rs35090077	-
DNAH5	Q8TE73	VAR_053841	p.Leu591Val	LB/B	rs35090077	-
DNAH5	Q8TE73	VAR_053842	p.Gln620Lys	LB/B	rs34076967	-
DNAH5	Q8TE73	VAR_053843	p.Lys765Asn	LB/B	rs4701997	-
DNAH5	Q8TE73	VAR_053844	p.Arg2425His	LB/B	rs35900306	-
DNAH5	Q8TE73	VAR_072469	p.Arg1454Gln	LP/P	rs542708170	Ciliary dyskinesia, primary, 3 (CILD3) [MIM:608644]
DNAH5	Q8TE73	VAR_072470	p.Ala2881Gly	LP/P	rs727502973	Ciliary dyskinesia, primary, 3 (CILD3) [MIM:608644]
DNAH5	Q8TE73	VAR_072471	p.Asp3605Asn	LP/P	-	Ciliary dyskinesia, primary, 3 (CILD3) [MIM:608644]
DNAH6	Q9C0G6	VAR_080035	p.His3471Gln	US	rs61731722	-
DNAH7	Q8WXX0	VAR_038580	p.His169Pro	LB/B	rs1072599	-
DNAH7	Q8WXX0	VAR_038581	p.Ala280Thr	LB/B	rs2375643	-
DNAH7	Q8WXX0	VAR_038582	p.Ile315Val	LB/B	rs17838596	-
DNAH7	Q8WXX0	VAR_038583	p.Ser438Asn	LB/B	rs16843720	-
DNAH7	Q8WXX0	VAR_038584	p.Arg545Cys	LB/B	rs10931715	-
DNAH7	Q8WXX0	VAR_038585	p.Asp565His	LB/B	rs2635718	-
DNAH7	Q8WXX0	VAR_038586	p.Lys675Glu	LB/B	rs10198893	-
DNAH7	Q8WXX0	VAR_038587	p.Lys825Glu	LB/B	rs6719500	-
DNAH7	Q8WXX0	VAR_038588	p.Pro1422Thr	LB/B	rs168192	-

DNAH7	Q8WXX0	VAR_038589	p.Glu1525Lys	LB/B	rs13415574	-
DNAH7	Q8WXX0	VAR_038590	p.Arg1886Gln	LB/B	rs13034775	-
DNAH7	Q8WXX0	VAR_038591	p.Pro1940Leu	LB/B	rs2375544	-
DNAH7	Q8WXX0	VAR_038592	p.Pro1971Leu	LB/B	rs2889109	-
DNAH7	Q8WXX0	VAR_038593	p.Met2020Thr	LB/B	rs10184131	-
DNAH7	Q8WXX0	VAR_038594	p.Asn2459Lys	LB/B	rs16841199	-
DNAH7	Q8WXX0	VAR_038595	p.Thr2569Ile	LB/B	rs2293066	-
DNAH7	Q8WXX0	VAR_038596	p.Ile2809Val	LB/B	rs16841018	-
DNAH7	Q8WXX0	VAR_038597	p.Leu3319Pro	LB/B	rs13411834	-
DNAH7	Q8WXX0	VAR_038598	p.Arg3386His	LB/B	rs6708527	-
DNAH8	Q96JB1	VAR_030171	p.Asn71Ser	LB/B	rs6935293	-
DNAH8	Q96JB1	VAR_030172	p.Gly473Arg	LB/B	rs1738254	-
DNAH8	Q96JB1	VAR_030173	p.Ile573Val	LB/B	rs3823430	-
DNAH8	Q96JB1	VAR_030174	p.Ala727Thr	LB/B	rs1678674	-
DNAH8	Q96JB1	VAR_030175	p.Gly807Glu	LB/B	rs874808	-
DNAH8	Q96JB1	VAR_030176	p.Glu1202Lys	LB/B	rs9357283	-
DNAH8	Q96JB1	VAR_030177	p.Thr2444Asn	LB/B	rs862432	-
DNAH8	Q96JB1	VAR_030178	p.Thr4106Met	LB/B	rs1537232	-
DNAH8	Q96JB1	VAR_030179	p.Ile4271Val	LB/B	rs10484847	-
DNAH8	Q96JB1	VAR_036213	p.Thr2226Met	US	-	A colorectal cancer sample
DNAH9	Q9NYC9	VAR_036214	p.Arg771Leu	US	-	A breast cancer sample
DNAH9	Q9NYC9	VAR_036215	p.Asp2653His	US	-	A breast cancer sample
DNAH9	Q9NYC9	VAR_036216	p.Thr3664Asn	US	rs138874996	A breast cancer sample
DNAH9	Q9NYC9	VAR_046312	p.Arg151His	LB/B	rs17599639	-
DNAH9	Q9NYC9	VAR_046313	p.Gln445Arg	LB/B	rs9892256	-
DNAH9	Q9NYC9	VAR_046314	p.Arg842Trp	LB/B	rs16945138	-
DNAH9	Q9NYC9	VAR_046315	p.Arg1158Trp	LB/B	rs8070501	-
DNAH9	Q9NYC9	VAR_046316	p.Thr1221Ala	LB/B	rs9916482	-
DNAH9	Q9NYC9	VAR_046317	p.Met2087Val	LB/B	rs9892290	-
DNAH9	Q9NYC9	VAR_046318	p.Asn2195Ser	LB/B	rs3744581	-
DNAH9	Q9NYC9	VAR_046319	p.Gln2438His	LB/B	rs2277658	-
DNAH9	Q9NYC9	VAR_046320	p.Lys2961Arg	LB/B	rs11870983	-
DNAH9	Q9NYC9	VAR_046321	p.Lys2968Asn	LB/B	rs11871037	-
DNAH9	Q9NYC9	VAR_046322	p.Arg3726Gln	LB/B	rs16945431	-
DNAH9	Q9NYC9	VAR_046323	p.Arg3726Trp	LB/B	rs3760436	-
DNAH9	Q9NYC9	VAR_046324	p.Asp4036Asn	LB/B	rs17612861	-
DNAH9	Q9NYC9	VAR_046325	p.Met4374Ile	LB/B	rs1990236	-
DNAH9	Q9NYC9	VAR_046326	p.Arg4443Cys	LB/B	rs9913494	-
DNAH9	Q9NYC9	VAR_046327	p.Trp4462Arg	LB/B	rs8074656	-
DNAH9	Q9NYC9	VAR_081803	p.Gly1674Arg	US	rs140721719	Ciliary dyskinesia, primary, 40 (CILD40) [MIM:618300]
DNAH9	Q9NYC9	VAR_081804	p.Lys1881Glu	LP/P	rs1567747142	Ciliary dyskinesia, primary, 40 (CILD40) [MIM:618300]
DNAH9	Q9NYC9	VAR_081806	p.Arg2965His	LP/P	rs375908701	Ciliary dyskinesia, primary, 40 (CILD40) [MIM:618300]
DNAH9	Q9NYC9	VAR_081807	p.Arg3398Leu	LP/P	rs763238622	Ciliary dyskinesia, primary, 40 (CILD40) [MIM:618300]
DNAH9	Q9NYC9	VAR_081809	p.Asp4123Asn	LP/P	rs1267599270	Ciliary dyskinesia, primary, 40 (CILD40) [MIM:618300]

DNAI1 Q9UI46 VAR_016774 p.Ala8Ser LB/B rs11547035 -
DNAI1 Q9UI46 VAR_016775 p.Gly515Ser LP/P rs79833450 Kartagener syndrome (KTGS) [MIM:244400]
DNAI1 Q9UI46 VAR_033876 p.Ala60Val LB/B rs16931549 -
DNAI1 Q9UI46 VAR_033877 p.Gln326His LB/B rs16931555 -
DNAI1 Q9UI46 VAR_033878 p.Val335Ile LB/B rs11793196 -
DNAI1 Q9UI46 VAR_033879 p.Val487Gly LB/B rs11999454 -
DNAI2 Q9GZS0 VAR_033880 p.Ala558Thr LB/B rs1979370 -
DNAI2 Q9GZS0 VAR_061140 p.Val495Ile LB/B rs28725418 -
DNAI3 Q8IWG1 VAR_057630 p.Thr674Ala LB/B rs17121745 -
DNAI3 Q8IWG1 VAR_057631 p.Arg798His LB/B rs709783 -
DNAI4 Q5VTH9 VAR_057635 p.Gly16Ala LB/B rs1886686 -
DNAI4 Q5VTH9 VAR_057636 p.Cys33Trp LB/B rs3008858 -
DNAI4 Q5VTH9 VAR_057637 p.Arg832Gln LB/B rs482082 -
DNAI7 Q6TDU7 VAR_043008 p.Arg33Ser LB/B rs10842496 -
DNAI7 Q6TDU7 VAR_062232 p.Ala633Glu LB/B rs859146 -
DNAJA3 Q96EY1 VAR_027965 p.Asn75Tyr LB/B rs1139653 -
DNAJA4 Q8WW22 VAR_069064 p.His226Arg LB/B rs17852881 -
DNAJB11 Q9UBS4 VAR_016092 p.Ile264Val LB/B rs8147 -
DNAJB11 Q9UBS4 VAR_081002 p.Pro54Arg LP/P rs1553849919 Polycystic kidney disease 6 with or without polycystic liver disease (PKD6) [MIM:618061]
DNAJB11 Q9UBS4 VAR_081003 p.Leu77Pro LP/P rs1553850185 Polycystic kidney disease 6 with or without polycystic liver disease (PKD6) [MIM:618061]
DNAJB12 Q9NXW2 VAR_017864 p.Glu304Lys LB/B rs3750784 -
DNAJB13 P59910 VAR_077053 p.Met278Arg LP/P rs754776389 Ciliary dyskinesia, primary, 34 (CILD34) [MIM:617091]
DNAJB2 P25686 VAR_048910 p.Gly270Arg LB/B rs34127289 -
DNAJB2 P25686 VAR_073286 p.Tyr5Cys LP/P rs730882140 Distal spinal muscular atrophy, autosomal recessive, 5 (DSMA5) [MIM:614881]
DNAJB3 Q8WWF6 VAR_061142 p.Asp85Glu LB/B rs34622615 -
DNAJB3 Q8WWF6 VAR_061143 p.Asp98Glu LB/B rs34150486 -
DNAJB6 O75190 VAR_067833 p.Phe89Ile LP/P rs387907150 Muscular dystrophy, limb-girdle, autosomal dominant 1 (LGMDD1) [MIM:603511]
DNAJB6 O75190 VAR_067834 p.Phe93Leu LP/P rs387907046 Muscular dystrophy, limb-girdle, autosomal dominant 1 (LGMDD1) [MIM:603511]
DNAJB6 O75190 VAR_067835 p.Pro96Arg LP/P rs387907047 Muscular dystrophy, limb-girdle, autosomal dominant 1 (LGMDD1) [MIM:603511]
DNAJB6 O75190 VAR_079428 p.Pro96Leu US - -
DNAJB7 Q7Z6W7 VAR_017779 p.Glu41Ala LB/B rs2269619 -
DNAJB8 Q8NHS0 VAR_033881 p.Met153Leu LB/B rs35948511 -
DNAJB9 Q9UBS3 VAR_048911 p.Arg136His LB/B rs17155937 -
DNAJC10 Q8IXB1 VAR_031247 p.Asp76Asn LB/B rs6729801 -
DNAJC10 Q8IXB1 VAR_031248 p.Tyr414Cys LB/B rs11681366 -
DNAJC10 Q8IXB1 VAR_031249 p.His646Gln LB/B rs288334 -
DNAJC10 Q8IXB1 VAR_048912 p.Leu347Ile LB/B rs13414223 -
DNAJC11 Q9NVH1 VAR_027078 p.Val267Met LB/B rs12137794 -
DNAJC11 Q9NVH1 VAR_027079 p.Thr290Ala LB/B rs200454 -
DNAJC11 Q9NVH1 VAR_055703 p.Thr290Ser LB/B rs200454 -
DNAJC12 Q9UKB3 VAR_048913 p.Met124Ile LB/B rs35690028 -
DNAJC12 Q9UKB3 VAR_048914 p.Cys129Trp LB/B rs36099123 -

DNAJC12 Q9UKB3 VAR_078797 p.Arg72Pro LP/P rs1035794099 Hyperphenylalaninemia, mild, non-BH4-deficient (HPANBH4) [MIM:617384]
DNAJC13 O75165 VAR_047458 p.Ala1463Ser LB/B rs3762672 -
DNAJC13 O75165 VAR_047459 p.Phe1487Cys LB/B rs4405917 -
DNAJC13 O75165 VAR_047460 p.Val1995Ile LB/B rs10935014 -
DNAJC13 O75165 VAR_061144 p.Pro1515Ser LB/B rs55825559 -
DNAJC13 O75165 VAR_073784 p.Glu264Gln LB/B - -
DNAJC13 O75165 VAR_073785 p.Asn855Ser US rs387907571 Parkinson disease (PARK) [MIM:168600]
DNAJC13 O75165 VAR_073786 p.Thr1082Ile LB/B rs202127368 -
DNAJC13 O75165 VAR_073787 p.Arg2115Leu LB/B rs770715465 -
DNAJC13 O75165 VAR_076716 p.Leu556Ser LB/B rs749000301 -
DNAJC13 O75165 VAR_076717 p.Asp674Ala LB/B rs199541720 -
DNAJC13 O75165 VAR_076718 p.Val722Leu US rs146930051 Parkinson disease (PARK) [MIM:168600]
DNAJC13 O75165 VAR_076719 p.Arg903Lys LB/B rs141952333 -
DNAJC13 O75165 VAR_076720 p.Leu997Phe LB/B rs752189478 -
DNAJC13 O75165 VAR_076721 p.Asn1135Ser LB/B rs751747947 -
DNAJC13 O75165 VAR_076722 p.Arg1266Gln US rs766013346 Parkinson disease (PARK) [MIM:168600]
DNAJC13 O75165 VAR_076723 p.Glu1291Gly LB/B rs61748101 -
DNAJC13 O75165 VAR_076724 p.Arg1516His LB/B rs139620588 -
DNAJC13 O75165 VAR_076725 p.Glu1740Gln LB/B rs142160751 -
DNAJC13 O75165 VAR_076726 p.Thr1895Met US rs145242123 Parkinson disease (PARK) [MIM:168600]
DNAJC13 O75165 VAR_076727 p.Ala2057Ser LB/B rs138693725 -
DNAJC13 O75165 VAR_076728 p.Leu2170Trp LB/B rs140537885 -
DNAJC15 Q9Y5T4 VAR_027077 p.Arg35Gly LB/B rs12015 -
DNAJC21 Q5F1R6 VAR_036163 p.Asp214Asn US - A breast cancer sample
DNAJC21 Q5F1R6 VAR_061145 p.Glu433Lys LB/B rs34908091 -
DNAJC21 Q5F1R6 VAR_076802 p.Pro32Ala LP/P rs879253818 Bone marrow failure syndrome 3 (BMFS3) [MIM:617052]
DNAJC24 Q6P3W2 VAR_036397 p.Asn23Asp US rs111299050 A breast cancer sample
DNAJC30 Q96LL9 VAR_024433 p.Gly34Arg LB/B rs1128349 -
DNAJC30 Q96LL9 VAR_048915 p.Phe167Leu LB/B rs13244259 -
DNAJC5 Q9H3Z4 VAR_066555 p.Leu115Arg LP/P rs387907043 Ceroid lipofuscinosis, neuronal, 4B (CLN4B) [MIM:162350]
DNAJC5G Q8N7S2 VAR_033882 p.Val4Gly LB/B rs17005979 -
DNAJC5G Q8N7S2 VAR_033883 p.His51Arg LB/B rs13414011 -
DNAJC6 O75061 VAR_026908 p.Ser671Asn LB/B rs4915691 -
DNAJC6 O75061 VAR_077924 p.Met76Leu LB/B rs61757223 -
DNAJC6 O75061 VAR_077925 p.Leu152Pro LB/B - -
DNAJC6 O75061 VAR_077926 p.Ile264Val LB/B - -
DNAJC6 O75061 VAR_077927 p.Cys441Ser LB/B rs145329294 -
DNAJC6 O75061 VAR_077928 p.Arg562Cys LB/B rs770127313 -
DNAJC6 O75061 VAR_077929 p.Arg870Gly LP/P rs879255630 Parkinson disease 19B, early-onset (PARK19B) [MIM:615528]
DNAL1 Q4LDG9 VAR_065739 p.Asn150Ser LP/P rs387907021 Ciliary dyskinesia, primary, 16 (CILD16) [MIM:614017]
DNALI1 O14645 VAR_014473 p.Ala65Val LB/B rs11749 -
DNALI1 O14645 VAR_035701 p.Ile120Met US rs1266159589 A colorectal cancer sample
DNASE1 P24855 VAR_002264 p.Gln31Glu LB/B rs77254040 -
DNASE1 P24855 VAR_002265 p.Pro154Ala LB/B rs1799891 -

DNASE1 P24855 VAR_002266 p.Arg244Gln LB/B rs1053874 -
DNASE1 P24855 VAR_009300 p.Val114Met LB/B rs530214101 -
DNASE1 P24855 VAR_009301 p.Arg207Cys LB/B rs148373909 -
DNASE1 P24855 VAR_024434 p.Arg2Ser LB/B rs8176927 -
DNASE1 P24855 VAR_024435 p.Gly127Arg LB/B rs8176919 -
DNASE1 P24855 VAR_029172 p.Arg107Gly LB/B rs8176928 -
DNASE1 P24855 VAR_029173 p.Cys231Tyr LB/B rs8176940 -
DNASE1 P24855 VAR_029174 p.Ala246Pro LB/B rs8176939 -
DNASE1 P24855 VAR_029175 p.Gly262Asp LB/B rs8176924 -
DNASE1L1 P49184 VAR_048869 p.Val122Ile LB/B rs34952165 -
DNASE1L3 Q13609 VAR_036079 p.Leu19Val US rs763778721 A breast cancer sample
DNASE1L3 Q13609 VAR_036080 p.Gly82Arg US rs74350392 A breast cancer sample
DNASE1L3 Q13609 VAR_036081 p.Tyr117Ser US - A colorectal cancer sample
DNASE1L3 Q13609 VAR_059249 p.Asn96Lys LB/B rs12491947 -
DNASE1L3 Q13609 VAR_061137 p.Ile243Met LB/B rs76440799 -
DNASE1L3 Q13609 VAR_076797 p.Arg206Cys LB/B rs35677470 -
DNASE2 O00115 VAR_012044 p.Arg314Leu LB/B rs1061192 -
DNASE2 O00115 VAR_048870 p.Arg39Ile LB/B rs36075196 -
DNASE2 O00115 VAR_048871 p.His204Arg LB/B rs16978744 -
DNASE2B Q8WZ79 VAR_048872 p.Lys47Arg LB/B rs3754274 -
DNASE2B Q8WZ79 VAR_059250 p.Gln3His LB/B rs3738573 -
DNASE2B Q8WZ79 VAR_059251 p.Arg51Lys LB/B rs3754274 -
DNER Q8NFT8 VAR_028380 p.Pro433Leu LB/B rs17853365 -
DNHD1 Q96M86 VAR_033353 p.Arg1358Cys LB/B rs12574381 -
DNHD1 Q96M86 VAR_033354 p.Lys1896Asn LB/B rs16915277 -
DNHD1 Q96M86 VAR_033355 p.Phe2041Leu LB/B rs11825154 -
DNHD1 Q96M86 VAR_037388 p.Arg3830His LB/B rs10769699 -
DNHD1 Q96M86 VAR_037389 p.Ile4666Thr LB/B rs11604362 -
DNHD1 Q96M86 VAR_039308 p.Val240Glu LB/B rs2555158 -
DNHD1 Q96M86 VAR_039309 p.Asp317Asn LB/B rs2555152 -
DNHD1 Q96M86 VAR_039310 p.His418Tyr LB/B rs4758423 -
DNHD1 Q96M86 VAR_039311 p.Gln560Glu LB/B rs11603869 -
DNHD1 Q96M86 VAR_056829 p.Gln279Pro LB/B rs11605196 -
DNHD1 Q96M86 VAR_056830 p.Phe403Leu LB/B rs11040904 -
DNLZ Q5SXM8 VAR_053993 p.Pro169His LB/B rs3812553 -
DNLZ Q5SXM8 VAR_053994 p.Ser178Thr LB/B rs3812552 -
DNM1 Q05193 VAR_048904 p.Asp744Asn LB/B rs1042007 -
DNM1 Q05193 VAR_073710 p.Ala177Pro LP/P rs587777860 Developmental and epileptic encephalopathy 31 (DEE31) [MIM:616346]
DNM1 Q05193 VAR_073711 p.Lys206Asn LP/P rs587777861 Developmental and epileptic encephalopathy 31 (DEE31) [MIM:616346]
DNM1 Q05193 VAR_073712 p.Arg237Trp LP/P rs760270633 Developmental and epileptic encephalopathy 31 (DEE31) [MIM:616346]
DNM1 Q05193 VAR_073713 p.Gly359Ala LP/P rs587777862 Developmental and epileptic encephalopathy 31 (DEE31) [MIM:616346]
DNM1L O00429 VAR_022446 p.Ser71Thr LB/B rs1064610 -
DNM1L O00429 VAR_030489 p.Glu426Asp LB/B rs2389105 -
DNM1L O00429 VAR_063704 p.Ala395Asp LP/P rs121908531 Encephalopathy due to defective mitochondrial and peroxisomal fission 1 (EMPF1) [MIM:614388]

DNM1L O00429 VAR_076316 p.Gly362Asp US rs879255685 Encephalopathy due to defective mitochondrial and peroxisomal fission 1 (EMPF1) [MIM:614388]
DNM1L O00429 VAR_076317 p.Gly362Ser LP/P rs886037861 Encephalopathy due to defective mitochondrial and peroxisomal fission 1 (EMPF1) [MIM:614388]
DNM1L O00429 VAR_076318 p.Arg403Cys LP/P rs863223953 Encephalopathy due to defective mitochondrial and peroxisomal fission 1 (EMPF1) [MIM:614388]
DNM1L O00429 VAR_080869 p.Glu2Ala LP/P rs1555229948 Optic atrophy 5 (OPA5) [MIM:610708]
DNM1L O00429 VAR_080870 p.Ser36Gly LP/P rs879255688 Encephalopathy due to defective mitochondrial and peroxisomal fission 1 (EMPF1) [MIM:614388]
DNM1L O00429 VAR_080871 p.Ala192Glu LP/P rs1555119216 Optic atrophy 5 (OPA5) [MIM:610708]
DNM1L O00429 VAR_080872 p.Leu406Ser LP/P - Encephalopathy due to defective mitochondrial and peroxisomal fission 1 (EMPF1) [MIM:614388]
DNM1P46 Q6ZS02 VAR_032224 p.Leu147Val LB/B rs4965539 -
DNM2 P50570 VAR_031961 p.Pro263Leu LB/B rs3745674 -
DNM2 P50570 VAR_031962 p.Glu368Lys LP/P rs121909092 Myopathy, centronuclear, 1 (CNM1) [MIM:160150]
DNM2 P50570 VAR_031963 p.Arg369Gln LP/P rs121909089 Myopathy, centronuclear, 1 (CNM1) [MIM:160150]
DNM2 P50570 VAR_031964 p.Arg369Trp LP/P rs121909090 Myopathy, centronuclear, 1 (CNM1) [MIM:160150]
DNM2 P50570 VAR_031965 p.Arg465Trp LP/P rs121909091 Myopathy, centronuclear, 1 (CNM1) [MIM:160150]
DNM2 P50570 VAR_031967 p.Lys562Glu LP/P rs121909088 Charcot-Marie-Tooth disease, dominant, intermediate type, B (CMTDIB) [MIM:606482]
DNM2 P50570 VAR_039041 p.Ala618Thr LP/P rs773598203 Myopathy, centronuclear, 1 (CNM1) [MIM:160150]
DNM2 P50570 VAR_039042 p.Ser619Leu LP/P rs121909095 Myopathy, centronuclear, 1 (CNM1) [MIM:160150]
DNM2 P50570 VAR_039043 p.Ser619Trp LP/P rs121909095 Myopathy, centronuclear, 1 (CNM1) [MIM:160150]
DNM2 P50570 VAR_062574 p.Gly537Cys LP/P rs121909093 Charcot-Marie-Tooth disease 2M (CMT2M) [MIM:606482]
DNM2 P50570 VAR_062575 p.Leu570His LP/P rs121909094 Charcot-Marie-Tooth disease 2M (CMT2M) [MIM:606482]
DNM2 P50570 VAR_062576 p.Glu650Lys LP/P - Myopathy, centronuclear, 1 (CNM1) [MIM:160150]
DNM2 P50570 VAR_068365 p.Glu368Gln LP/P - Myopathy, centronuclear, 1 (CNM1) [MIM:160150]
DNM2 P50570 VAR_068366 p.Arg522Cys LP/P - Myopathy, centronuclear, 1 (CNM1) [MIM:160150]
DNM2 P50570 VAR_068367 p.Arg522His LP/P rs587783595 Myopathy, centronuclear, 1 (CNM1) [MIM:160150]
DNM2 P50570 VAR_068368 p.Arg523Gly LP/P rs587783596 Myopathy, centronuclear, 1 (CNM1) [MIM:160150]
DNM2 P50570 VAR_068369 p.Glu560Lys LP/P rs879254086 Myopathy, centronuclear, 1 (CNM1) [MIM:160150]
DNM2 P50570 VAR_068370 p.Ala618Asp LP/P rs1555715869 Myopathy, centronuclear, 1 (CNM1) [MIM:160150]
DNM2 P50570 VAR_068371 p.Leu621Pro LP/P rs587783597 Myopathy, centronuclear, 1 (CNM1) [MIM:160150]
DNM2 P50570 VAR_068372 p.Pro627His LP/P - Myopathy, centronuclear, 1 (CNM1) [MIM:160150]

DNM2 P50570 VAR_068373 p.Pro627Arg LP/P rs587783598 Myopathy, centronuclear, 1 (CNM1) [MIM:160150]
DNM2 P50570 VAR_068425 p.Gly358Arg LP/P rs267606772 Charcot-Marie-Tooth disease 2M (CMT2M) [MIM:606482]
DNM2 P50570 VAR_070163 p.Phe379Val LP/P rs397514735 Lethal congenital contracture syndrome 5 (LCCS5) [MIM:615368]
DNMBP Q6XZF7 VAR_024339 p.Cys1413Trp LB/B rs11190305 -
DNMBP Q6XZF7 VAR_050955 p.Glu81Asp LB/B rs12267912 -
DNMBP Q6XZF7 VAR_050956 p.Asn373Lys LB/B rs35924554 -
DNMBP Q6XZF7 VAR_050957 p.Asn914Lys LB/B rs7919507 -
DNMT1 P26358 VAR_024605 p.His97Arg LB/B rs16999593 -
DNMT1 P26358 VAR_051960 p.Ile311Val LB/B rs2228612 -
DNMT1 P26358 VAR_065966 p.Tyr495Cys LP/P rs199473690 Neuropathy, hereditary sensory, 1E (HSN1E) [MIM:614116]
DNMT1 P26358 VAR_070055 p.Ala554Val LP/P rs397509392 Cerebellar ataxia, deafness, and narcolepsy, autosomal dominant (ADCADN) [MIM:604121]
DNMT1 P26358 VAR_070056 p.Gly589Ala LP/P rs397509393 Cerebellar ataxia, deafness, and narcolepsy, autosomal dominant (ADCADN) [MIM:604121]
DNMT1 P26358 VAR_070057 p.Val590Phe LP/P rs397509391 Cerebellar ataxia, deafness, and narcolepsy, autosomal dominant (ADCADN) [MIM:604121]
DNMT3A Q9Y6K1 VAR_067234 p.Gly699Asp US rs761064473 A patient with chronic myelomonocytic leukemia
DNMT3A Q9Y6K1 VAR_067236 p.Arg882Cys LP/P rs377577594 Leukemia, acute myelogenous (AML) [MIM:601626]
DNMT3A Q9Y6K1 VAR_067236 p.Arg882Cys LP/P rs377577594 Tatton-Brown-Rahman syndrome (TBRS) [MIM:615879]
DNMT3A Q9Y6K1 VAR_067237 p.Arg882His LP/P rs147001633 Leukemia, acute myelogenous (AML) [MIM:601626]
DNMT3A Q9Y6K1 VAR_067237 p.Arg882His LP/P rs147001633 Tatton-Brown-Rahman syndrome (TBRS) [MIM:615879]
DNMT3A Q9Y6K1 VAR_067238 p.Arg882Pro US rs147001633 A patient with chronic myelomonocytic leukemia
DNMT3A Q9Y6K1 VAR_071463 p.Ile310Asn LP/P rs587777508 Tatton-Brown-Rahman syndrome (TBRS) [MIM:615879]
DNMT3A Q9Y6K1 VAR_071464 p.Gly532Ser LP/P rs951361433 Tatton-Brown-Rahman syndrome (TBRS) [MIM:615879]
DNMT3A Q9Y6K1 VAR_071465 p.Met548Lys LP/P rs587777509 Tatton-Brown-Rahman syndrome (TBRS) [MIM:615879]
DNMT3A Q9Y6K1 VAR_071466 p.Cys549Arg LP/P - Tatton-Brown-Rahman syndrome (TBRS) [MIM:615879]
DNMT3A Q9Y6K1 VAR_071467 p.Leu648Pro LP/P rs587777507 Tatton-Brown-Rahman syndrome (TBRS) [MIM:615879]
DNMT3A Q9Y6K1 VAR_071468 p.Pro700Leu LP/P rs772368909 Tatton-Brown-Rahman syndrome (TBRS) [MIM:615879]
DNMT3A Q9Y6K1 VAR_071469 p.Arg749Cys LP/P rs754613602 Tatton-Brown-Rahman syndrome (TBRS) [MIM:615879]
DNMT3A Q9Y6K1 VAR_071470 p.Asn838Asp LP/P rs961377711 Tatton-Brown-Rahman syndrome (TBRS) [MIM:615879]

DNMT3A Q9Y6K1 VAR_071471 p.Phe902Ser LP/P rs587777510 Tatton-Brown-Rahman syndrome (TBRS) [MIM:615879]
DNMT3A Q9Y6K1 VAR_071472 p.Pro904Leu LP/P rs149095705 Tatton-Brown-Rahman syndrome (TBRS) [MIM:615879]
DNMT3A Q9Y6K1 VAR_077522 p.Tyr365Cys US rs144062658 Tatton-Brown-Rahman syndrome (TBRS) [MIM:615879]
DNMT3A Q9Y6K1 VAR_077523 p.Asp529Asn US rs962805778 Tatton-Brown-Rahman syndrome (TBRS) [MIM:615879]
DNMT3A Q9Y6K1 VAR_077524 p.Arg771Gln US rs757823678 Tatton-Brown-Rahman syndrome (TBRS) [MIM:615879]
DNMT3A Q9Y6K1 VAR_077525 p.Val778Gly US rs979932565 Tatton-Brown-Rahman syndrome (TBRS) [MIM:615879]
DNMT3A Q9Y6K1 VAR_083539 p.Trp330Arg LP/P - Heyn-Sproul-Jackson syndrome (HESJAS) [MIM:618724]
DNMT3A Q9Y6K1 VAR_083540 p.Asp333Asn LP/P - Heyn-Sproul-Jackson syndrome (HESJAS) [MIM:618724]
DNMT3B Q9UBC3 VAR_011499 p.Ala603Thr LP/P rs121908943 Immunodeficiency-centromeric instability-facial anomalies syndrome 1 (ICF1) [MIM:242860]
DNMT3B Q9UBC3 VAR_011500 p.Gly663Ser LP/P rs121908942 Immunodeficiency-centromeric instability-facial anomalies syndrome 1 (ICF1) [MIM:242860]
DNMT3B Q9UBC3 VAR_011501 p.Val726Gly LP/P rs121908941 Immunodeficiency-centromeric instability-facial anomalies syndrome 1 (ICF1) [MIM:242860]
DNMT3B Q9UBC3 VAR_011503 p.Asp817Gly LP/P rs121908939 Immunodeficiency-centromeric instability-facial anomalies syndrome 1 (ICF1) [MIM:242860]
DNMT3B Q9UBC3 VAR_011504 p.Val818Met LP/P rs121908940 Immunodeficiency-centromeric instability-facial anomalies syndrome 1 (ICF1) [MIM:242860]
DNMT3B Q9UBC3 VAR_011506 p.Ala585Val LP/P - Immunodeficiency-centromeric instability-facial anomalies syndrome 1 (ICF1) [MIM:242860]
DNMT3B Q9UBC3 VAR_011507 p.Val606Ala LP/P rs867732105 Immunodeficiency-centromeric instability-facial anomalies syndrome 1 (ICF1) [MIM:242860]
DNMT3B Q9UBC3 VAR_011508 p.Val699Gly LP/P - Immunodeficiency-centromeric instability-facial anomalies syndrome 1 (ICF1) [MIM:242860]
DNMT3B Q9UBC3 VAR_011509 p.Ala766Pro LP/P rs1191203668 Immunodeficiency-centromeric instability-facial anomalies syndrome 1 (ICF1) [MIM:242860]
DNMT3B Q9UBC3 VAR_011510 p.His814Arg LP/P rs1219696128 Immunodeficiency-centromeric instability-facial anomalies syndrome 1 (ICF1) [MIM:242860]
DNMT3B Q9UBC3 VAR_022579 p.Ser270Pro LP/P rs121908947 Immunodeficiency-centromeric instability-facial anomalies syndrome 1 (ICF1) [MIM:242860]
DNMT3B Q9UBC3 VAR_022580 p.Leu664Pro LP/P - Immunodeficiency-centromeric instability-facial anomalies syndrome 1 (ICF1) [MIM:242860]
DNMT3B Q9UBC3 VAR_022581 p.Arg840Gln LP/P rs121908946 Immunodeficiency-centromeric instability-facial anomalies syndrome 1 (ICF1) [MIM:242860]
DNMT3B Q9UBC3 VAR_033885 p.Arg54Pro LB/B rs17123590 -
DNMT3B Q9UBC3 VAR_077527 p.Cys527Arg LP/P - Facioscapulohumeral muscular dystrophy 2 (FSHD2) [MIM:158901]
DNMT3B Q9UBC3 VAR_077528 p.Ala585Thr LP/P rs750849178 Immunodeficiency-centromeric instability-facial anomalies syndrome 1 (ICF1) [MIM:242860]
DNMT3B Q9UBC3 VAR_077529 p.Pro691Leu LP/P rs889145646 Facioscapulohumeral muscular dystrophy 2 (FSHD2) [MIM:158901]

DNMT3B Q9UBC3 VAR_077530 p.Val836Met US rs866792483 Immunodeficiency-centromeric instability-facial anomalies syndrome 1 (ICF1) [MIM:242860]
DNMT3L Q9UJW3 VAR_051962 p.Arg278Gly LB/B rs7354779 -
DNTT P04053 VAR_058200 p.Arg112Gly LB/B rs6584066 -
DNTTIP1 Q9H147 VAR_014956 p.Ala183Thr LB/B rs408911 -
DNTTIP2 Q5QJE6 VAR_038748 p.Glu309Asp LB/B rs3747965 -
DNTTIP2 Q5QJE6 VAR_038749 p.Thr341Ala LB/B rs3179879 -
DNTTIP2 Q5QJE6 VAR_038750 p.Ala430Val LB/B rs35650636 -
DNTTIP2 Q5QJE6 VAR_038751 p.Tyr676Phe LB/B rs12748154 -
DNTTIP2 Q5QJE6 VAR_061710 p.Gly477Glu LB/B rs41292661 -
DOC2A Q14183 VAR_019656 p.Gly48Ser LB/B rs1140239 -
DOC2B Q14184 VAR_065743 p.Arg209Leu LB/B rs369343321 -
DOCK1 Q14185 VAR_059971 p.Ala1793Thr LB/B rs869801 -
DOCK11 Q5JSL3 VAR_034854 p.Ile813Phe LB/B rs16995229 -
DOCK2 Q92608 VAR_015822 p.Ser1746Thr LB/B rs2270898 -
DOCK2 Q92608 VAR_022137 p.Thr1779Ser LB/B rs2270898 -
DOCK2 Q92608 VAR_053064 p.Asp1558Ala LB/B rs13179480 -
DOCK2 Q92608 VAR_073859 p.Arg751Ser LP/P rs1581090174 Immunodeficiency 40 (IMD40) [MIM:616433]
DOCK2 Q92608 VAR_073860 p.Arg1104Trp LP/P rs780318765 Immunodeficiency 40 (IMD40) [MIM:616433]
DOCK3 Q8IZD9 VAR_081832 p.Arg392Gln US rs199600118 Neurodevelopmental disorder with impaired intellectual development, hypotonia, and ataxia (NEDIDHA) [MIM:618292]
DOCK3 Q8IZD9 VAR_081833 p.Lys1296Arg US rs201184598 Neurodevelopmental disorder with impaired intellectual development, hypotonia, and ataxia (NEDIDHA) [MIM:618292]
DOCK3 Q8IZD9 VAR_081834 p.Met1674Leu US rs142515812 Neurodevelopmental disorder with impaired intellectual development, hypotonia, and ataxia (NEDIDHA) [MIM:618292]
DOCK4 Q8N1I0 VAR_015823 p.Thr87Ile LB/B rs1396518456 -
DOCK4 Q8N1I0 VAR_015824 p.Glu606Gln LB/B - -
DOCK4 Q8N1I0 VAR_015825 p.Lys1059Thr US - -
DOCK4 Q8N1I0 VAR_015826 p.Pro1718Leu US - -
DOCK4 Q8N1I0 VAR_015827 p.Pro1733Ala LB/B rs150569245 -
DOCK4 Q8N1I0 VAR_015828 p.Ser1755Pro US - -
DOCK4 Q8N1I0 VAR_015829 p.Val1884Met LB/B rs369715294 -
DOCK4 Q8N1I0 VAR_015830 p.Val1914Ile LB/B rs12705795 -
DOCK4 Q8N1I0 VAR_015831 p.Pro1917Leu LB/B rs199706346 -
DOCK4 Q8N1I0 VAR_015832 p.Ser1926Leu LB/B rs34597439 -
DOCK4 Q8N1I0 VAR_057517 p.Asn535Asp LB/B rs12705801 -
DOCK4 Q8N1I0 VAR_057518 p.Arg853His LB/B rs2074130 -
DOCK4 Q8N1I0 VAR_057519 p.Arg1570Lys LB/B rs3757650 -
DOCK4 Q8N1I0 VAR_057520 p.Phe1580Leu LB/B rs3757651 -
DOCK4 Q8N1I0 VAR_057521 p.Gln1822Lys LB/B rs10281942 -
DOCK5 Q9H7D0 VAR_033886 p.Gln250Arg LB/B rs17053341 -
DOCK5 Q9H7D0 VAR_033887 p.Glu1836Lys LB/B rs35688737 -
DOCK5 Q9H7D0 VAR_053065 p.Gln1023Arg LB/B rs2271111 -
DOCK5 Q9H7D0 VAR_053066 p.Lys1285Arg LB/B rs2659585 -
DOCK6 Q96HP0 VAR_029830 p.Pro250Leu LB/B rs12978266 -
DOCK6 Q96HP0 VAR_029831 p.Pro555Leu LB/B rs12609039 -
DOCK6 Q96HP0 VAR_029832 p.Gly665Arg LB/B rs17001264 -
DOCK6 Q96HP0 VAR_029833 p.Val1420Leu LB/B rs8108071 -
DOCK6 Q96HP0 VAR_057522 p.Arg826Cys LB/B rs35881692 -

DOCK6 Q96HP0 VAR_057523 p.Ala1442Thr LB/B rs34243815 -
DOCK7 Q96N67 VAR_057524 p.Ile824Thr LB/B rs35400360 -
DOCK8 Q8NF50 VAR_033888 p.Pro97Thr LB/B rs529208 -
DOCK8 Q8NF50 VAR_033889 p.Glu237Lys LB/B rs11789099 -
DOCK8 Q8NF50 VAR_033890 p.Asn413Ser LB/B rs10970979 -
DOCK8 Q8NF50 VAR_033891 p.Ala597Val LB/B rs17673268 -
DOCK8 Q8NF50 VAR_033892 p.Arg1008Trp LB/B rs16937932 -
DOCK8 Q8NF50 VAR_033893 p.Ala1970Pro LB/B rs34908836 -
DOCK8 Q8NF50 VAR_059972 p.Glu169Lys LB/B rs11789099 -
DOCK8 Q8NF50 VAR_063753 p.Lys473Arg LP/P rs112321280 Hyper-IgE recurrent infection syndrome 2, autosomal recessive (HIES2) [MIM:243700]
DOCK8 Q8NF50 VAR_071964 p.Ile652Val LB/B rs1381340726 -
DOCK8-AS1 Q5T8R8 VAR_050824 p.Leu43Arg LB/B rs636922 -
DOCK8-AS1 Q5T8R8 VAR_050825 p.Arg178Gln LB/B rs2236547 -
DOCK8-AS1 Q5T8R8 VAR_050826 p.Arg231Gly LB/B rs540473 -
DOCK9 Q9BZ29 VAR_053067 p.Lys1416Glu LB/B rs16955934 -
DOCK9 Q9BZ29 VAR_062000 p.Ala455Thr LB/B rs56010605 -
DOK2 O60496 VAR_030951 p.Ala152Pro LB/B rs1140295 -
DOK2 O60496 VAR_030952 p.Ser394Ala LB/B rs2242241 -
DOK2 O60496 VAR_053068 p.Pro274Leu LB/B rs34215892 -
DOK3 Q7L591 VAR_057525 p.Arg12Pro LB/B rs3749728 -
DOK3 Q7L591 VAR_062002 p.Gly22Arg LB/B rs41275297 -
DOK7 Q18PE1 VAR_027544 p.Gly180Ala LP/P rs118203994 Myasthenic syndrome, congenital, 10 (CMS10) [MIM:254300]
DOK7 Q18PE1 VAR_027545 p.Asp197Asn LB/B rs16844422 -
DOK7 Q18PE1 VAR_027546 p.Arg261His LB/B rs16844460 -
DOK7 Q18PE1 VAR_027547 p.Gln296Arg LB/B rs6811423 -
DOK7 Q18PE1 VAR_027548 p.Pro415Ser LB/B rs16844464 -
DOK7 Q18PE1 VAR_027549 p.Gly427Asp LB/B rs2020433 -
DOK7 Q18PE1 VAR_027550 p.Arg451Trp LB/B rs16844470 -
DOK7 Q18PE1 VAR_027551 p.Gly461Asp LB/B rs9684786 -
DOK7 Q18PE1 VAR_031246 p.Arg158Gln LP/P rs754633490 Myasthenic syndrome, congenital, 10 (CMS10) [MIM:254300]
DOK7 Q18PE1 VAR_050508 p.Gly379Arg LB/B rs6831659 -
DOK7 Q18PE1 VAR_068750 p.Glu3Lys LP/P rs763233743 Myasthenic syndrome, congenital, 10 (CMS10) [MIM:254300]
DOK7 Q18PE1 VAR_068751 p.Pro31Thr LP/P - Myasthenic syndrome, congenital, 10 (CMS10) [MIM:254300]
DOK7 Q18PE1 VAR_068752 p.Ala33Val LP/P - Myasthenic syndrome, congenital, 10 (CMS10) [MIM:254300]
DOK7 Q18PE1 VAR_068753 p.Ser45Leu LB/B rs62272670 -
DOK7 Q18PE1 VAR_068754 p.Thr77Met LP/P rs940346413 Myasthenic syndrome, congenital, 10 (CMS10) [MIM:254300]
DOK7 Q18PE1 VAR_068755 p.Ala99Val LB/B rs138010842 -
DOK7 Q18PE1 VAR_068756 p.Gly109Cys LP/P - Myasthenic syndrome, congenital, 10 (CMS10) [MIM:254300]
DOK7 Q18PE1 VAR_068757 p.Val116Met LP/P rs1429428597 Myasthenic syndrome, congenital, 10 (CMS10) [MIM:254300]

DOK7 Q18PE1 VAR_068758 p.His132Gln LP/P - Myasthenic syndrome, congenital, 10 (CMS10) [MIM:254300]
DOK7 Q18PE1 VAR_068759 p.Val139Leu LP/P rs571769859 Myasthenic syndrome, congenital, 10 (CMS10) [MIM:254300]
DOK7 Q18PE1 VAR_068760 p.Pro146Leu LP/P rs770987150 Myasthenic syndrome, congenital, 10 (CMS10) [MIM:254300]
DOK7 Q18PE1 VAR_068761 p.Leu157Arg LP/P - Myasthenic syndrome, congenital, 10 (CMS10) [MIM:254300]
DOK7 Q18PE1 VAR_068762 p.Gly161Arg LP/P rs758131044 Myasthenic syndrome, congenital, 10 (CMS10) [MIM:254300]
DOK7 Q18PE1 VAR_068763 p.Gly166Arg LP/P rs781227659 Myasthenic syndrome, congenital, 10 (CMS10) [MIM:254300]
DOK7 Q18PE1 VAR_068764 p.Gly171Asp LP/P rs1286619522 Myasthenic syndrome, congenital, 10 (CMS10) [MIM:254300]
DOK7 Q18PE1 VAR_068765 p.Gly171Arg LP/P - Myasthenic syndrome, congenital, 10 (CMS10) [MIM:254300]
DOK7 Q18PE1 VAR_068766 p.Gly172Arg LP/P rs768892432 Myasthenic syndrome, congenital, 10 (CMS10) [MIM:254300]
DOK7 Q18PE1 VAR_068767 p.Gly180Val LP/P - Myasthenic syndrome, congenital, 10 (CMS10) [MIM:254300]
DOK7 Q18PE1 VAR_068768 p.His272Gln LB/B rs115614731 -
DOK7 Q18PE1 VAR_068769 p.Arg323Cys LB/B rs150728781 -
DOK7 Q18PE1 VAR_068770 p.Glu382Lys LB/B rs560463670 -
DOK7 Q18PE1 VAR_068771 p.Arg402Gln LB/B rs370039804 -
DOK7 Q18PE1 VAR_068772 p.Ala440Thr LB/B rs753026831 -
DOK7 Q18PE1 VAR_068773 p.Pro469His LP/P rs147185207 Myasthenic syndrome, congenital, 10 (CMS10) [MIM:254300]
DOK7 Q18PE1 VAR_068774 p.Pro503Thr LB/B rs184556570 -
DOLK Q9UPQ8 VAR_032851 p.Cys99Ser LP/P rs137853109 Congenital disorder of glycosylation 1M (CDG1M) [MIM:610768]
DOLK Q9UPQ8 VAR_032852 p.Tyr441Ser LP/P rs137853110 Congenital disorder of glycosylation 1M (CDG1M) [MIM:610768]
DOLK Q9UPQ8 VAR_049709 p.Asp224Val LB/B rs17485436 -
DONSON Q9NYP3 VAR_079330 p.Ser28Arg LB/B rs768071555 -
DONSON Q9NYP3 VAR_079331 p.Cys278Arg LP/P - Microcephaly, short stature, and limb abnormalities (MISSLA) [MIM:617604]
DONSON Q9NYP3 VAR_079332 p.Tyr282Cys LP/P - Microcephaly, short stature, and limb abnormalities (MISSLA) [MIM:617604]
DONSON Q9NYP3 VAR_079333 p.Phe292Leu LP/P rs779803447 Microcephaly, short stature, and limb abnormalities (MISSLA) [MIM:617604]
DONSON Q9NYP3 VAR_079338 p.Pro433Ser LP/P - Microcephaly, short stature, and limb abnormalities (MISSLA) [MIM:617604]
DONSON Q9NYP3 VAR_079339 p.Met446Thr LP/P rs1135401959 Microcephaly, short stature, and limb abnormalities (MISSLA) [MIM:617604]
DONSON Q9NYP3 VAR_079340 p.Lys489Thr US rs146664036 Microcephaly, short stature, and limb abnormalities (MISSLA) [MIM:617604]
DONSON Q9NYP3 VAR_079341 p.Glu504Lys LP/P rs374688527 Microcephaly, short stature, and limb abnormalities (MISSLA) [MIM:617604]
DOP1A Q5JWR5 VAR_034690 p.Arg596Gln LB/B rs4706980 -

DOP1A Q5JWR5 VAR_034691 p.Gln1781Leu LB/B rs9444039 -
DOP1A Q5JWR5 VAR_036607 p.Asp1155His US - A breast cancer sample
DOP1B Q9Y3R5 VAR_027939 p.Cys1118Gly LB/B rs4817788 -
DOP1B Q9Y3R5 VAR_027940 p.Pro1149His LB/B rs3746866 -
DOP1B Q9Y3R5 VAR_027941 p.Arg1217Ser LB/B rs3746867 -
DOP1B Q9Y3R5 VAR_027942 p.Gly2139Glu LB/B rs3827183 -
DOP1B Q9Y3R5 VAR_034688 p.Ser1021Trp LB/B rs7278340 -
DOT1L Q8TEK3 VAR_014287 p.Leu726Met LB/B rs880525 -
DOT1L Q8TEK3 VAR_014288 p.Gly1386Ser LB/B rs3815308 -
DOT1L Q8TEK3 VAR_014289 p.Val1418Leu LB/B rs2302061 -
DPAGT1 Q9H3H5 VAR_011391 p.Ile393Val LB/B rs643788 -
DPAGT1 Q9H3H5 VAR_017243 p.Tyr170Cys LP/P rs28934876 Congenital disorder of glycosylation 1J (CDG1J) [MIM:608093]
DPAGT1 Q9H3H5 VAR_036422 p.Met9Ile US - A breast cancer sample
DPAGT1 Q9H3H5 VAR_068810 p.Met108Ile LP/P rs376039938 Myasthenic syndrome, congenital, 13 (CMS13) [MIM:614750]
DPAGT1 Q9H3H5 VAR_068811 p.Val117Ile LP/P rs387907243 Myasthenic syndrome, congenital, 13 (CMS13) [MIM:614750]
DPAGT1 Q9H3H5 VAR_068812 p.Leu120Met LP/P rs387907244 Myasthenic syndrome, congenital, 13 (CMS13) [MIM:614750]
DPAGT1 Q9H3H5 VAR_068813 p.Gly160Ser LP/P rs762676399 Myasthenic syndrome, congenital, 13 (CMS13) [MIM:614750]
DPAGT1 Q9H3H5 VAR_068814 p.Gly192Ser LP/P rs768464558 Myasthenic syndrome, congenital, 13 (CMS13) [MIM:614750]
DPAGT1 Q9H3H5 VAR_068815 p.Val264Gly LP/P rs387907245 Myasthenic syndrome, congenital, 13 (CMS13) [MIM:614750]
DPCD Q9BVM2 VAR_039574 p.Ser56Arg LB/B rs1331419018 -
DPCD Q9BVM2 VAR_039575 p.Leu156Ser LB/B rs7006 -
DPEP1 P16444 VAR_036496 p.Arg246His US rs1043397364 A colorectal cancer sample
DPEP1 P16444 VAR_061375 p.Glu351Lys LB/B rs1126464 -
DPEP1 P16444 VAR_061376 p.Glu351Gln LB/B rs1126464 -
DPEP2 Q9H4A9 VAR_033894 p.His468Asp LB/B rs1133090 -
DPEP2 Q9H4A9 VAR_060230 p.Arg201Pro LB/B rs255051 -
DPF1 Q92782 VAR_079177 p.Trp20Ser LB/B - -
DPF2 Q92785 VAR_081047 p.Cys276Phe LP/P rs1555031372 Coffin-Siris syndrome 7 (CSS7) [MIM:618027]
DPF2 Q92785 VAR_081048 p.Cys330Trp LP/P rs1555031500 Coffin-Siris syndrome 7 (CSS7) [MIM:618027]
DPF2 Q92785 VAR_081049 p.Asp346Gly LP/P rs1555032044 Coffin-Siris syndrome 7 (CSS7) [MIM:618027]
DPF2 Q92785 VAR_081050 p.Arg350His LP/P rs1555032051 Coffin-Siris syndrome 7 (CSS7) [MIM:618027]
DPF2 Q92785 VAR_081051 p.Trp369Arg LP/P - Coffin-Siris syndrome 7 (CSS7) [MIM:618027]
DPF3 Q92784 VAR_047771 p.Arg177His LB/B rs17855717 -
DPH1 Q9BZG8 VAR_036702 p.Ala7Val US rs778705666 Ovarian cancer
DPH1 Q9BZG8 VAR_036703 p.Ala34Asp US - Ovarian cancer
DPH1 Q9BZG8 VAR_036704 p.Leu335Val US rs35394823 Ovarian cancer
DPH1 Q9BZG8 VAR_036705 p.Ser389Arg US - Ovarian cancer
DPH1 Q9BZG8 VAR_055706 p.Lys226Arg LB/B rs1131600 -
DPH1 Q9BZG8 VAR_059255 p.Ile51Met LB/B rs8070453 -
DPH1 Q9BZG8 VAR_076412 p.Met6Lys LP/P rs757167361 Developmental delay with short stature, dysmorphic facial features, and sparse hair (DEDSSH) [MIM:616901]

DPH1 Q9BZG8 VAR_076413 p.Leu234Pro LP/P rs730882250 Developmental delay with short stature, dysmorphic facial features, and sparse hair (DEDSSH) [MIM:616901]
DPH6 Q7L8W6 VAR_031403 p.Gly41Glu LB/B rs34907758 -
DPH6 Q7L8W6 VAR_031404 p.Pro236Arg LB/B rs10519996 -
DPH7 Q9BTV6 VAR_053437 p.Gly158Arg LB/B rs821314 -
DPM1 O60762 VAR_012341 p.Arg92Gly LP/P rs121908583 Congenital disorder of glycosylation 1E (CDG1E) [MIM:608799]
DPM1 O60762 VAR_019841 p.Ser248Pro LP/P rs587777114 Congenital disorder of glycosylation 1E (CDG1E) [MIM:608799]
DPM1 O60762 VAR_070592 p.Gly152Val LP/P rs587777116 Congenital disorder of glycosylation 1E (CDG1E) [MIM:608799]
DPM2 O94777 VAR_033895 p.Thr76Ser LB/B rs7997 -
DPM2 O94777 VAR_069745 p.Tyr23Cys LP/P rs397514503 Congenital disorder of glycosylation 1U (CDG1U) [MIM:615042]
DPM3 Q9P2X0 VAR_062518 p.Leu85Ser LP/P rs121908155 Muscular dystrophy-dystroglycanopathy limb-girdle C15 (MDDGC15) [MIM:612937]
DPP10 Q8N608 VAR_057061 p.Ala340Pro LB/B rs2053724 -
DPP10 Q8N608 VAR_057062 p.Ser517Asn LB/B rs13421193 -
DPP10 Q8N608 VAR_059759 p.Val401Ile LB/B rs1446495 -
DPP3 Q9NY33 VAR_021850 p.Arg678His LB/B rs2305535 -
DPP3 Q9NY33 VAR_033494 p.Arg76His LB/B rs11826683 -
DPP3 Q9NY33 VAR_033495 p.Gln145His LB/B rs11550299 -
DPP3 Q9NY33 VAR_051597 p.Glu690Lys LB/B rs12421620 -
DPP4 P27487 VAR_084545 p.Val266Ile LB/B - -
DPP6 P42658 VAR_051579 p.Leu854Pro LB/B rs3734960 -
DPP6 P42658 VAR_073680 p.Met385Leu LP/P rs786205143 Mental retardation, autosomal dominant 33 (MRD33) [MIM:616311]
DPP7 Q9UHL4 VAR_047087 p.Ala89Gly LB/B rs10747049 -
DPPA2 Q7Z7J5 VAR_028087 p.Arg131Gln LB/B rs9809030 -
DPPA3 Q6W0C5 VAR_030533 p.Glu51Gln LB/B rs2024320 -
DPPA4 Q7L190 VAR_060372 p.Ile55Val LB/B rs3762648 -
DPPA4 Q7L190 VAR_060373 p.Ser226Phe LB/B rs3905734 -
DPT Q07507 VAR_048888 p.Val201Ile LB/B rs6698023 -
DPY19L1 Q2PZI1 VAR_037332 p.Gly502Val LB/B rs1637696 -
DPY19L2 Q6NUT2 VAR_037333 p.Met37Val LB/B rs10878075 -
DPY19L2 Q6NUT2 VAR_037334 p.Ala41Val LB/B rs10878074 -
DPY19L2 Q6NUT2 VAR_037335 p.Ser51Ala LB/B rs10878073 -
DPY19L2 Q6NUT2 VAR_062214 p.Val757Ile LB/B rs12314553 -
DPY19L2P2 Q6ZN68 VAR_043009 p.Met66Val LB/B rs7796589 -
DPY19L2P2 Q6ZN68 VAR_043010 p.Asn333Ile LB/B rs17136078 -
DPY19L3 Q6ZPD9 VAR_037336 p.Met350Val LB/B rs8105178 -
DPYD Q12882 VAR_005173 p.Cys29Arg LP/P rs1801265 Dihydropyrimidine dehydrogenase deficiency (DPYDD) [MIM:274270]
DPYD Q12882 VAR_005174 p.Arg235Trp LP/P rs1801266 Dihydropyrimidine dehydrogenase deficiency (DPYDD) [MIM:274270]
DPYD Q12882 VAR_005175 p.Ser534Asn LB/B rs1801158 -
DPYD Q12882 VAR_005176 p.Ile543Val LB/B rs1801159 -
DPYD Q12882 VAR_005177 p.Arg886His LP/P rs1801267 Dihydropyrimidine dehydrogenase deficiency (DPYDD) [MIM:274270]

DPYD Q12882 VAR_005178 p.Val995Phe LB/B rs1801268 -
DPYD Q12882 VAR_014760 p.Val732Ile LB/B rs1801160 -
DPYD Q12882 VAR_054034 p.Met166Val LB/B rs2297595 -
DPYS Q14117 VAR_002267 p.Thr68Arg LP/P - Dihydropyrimidinase deficiency (DPYSD) [MIM:222748]
DPYS Q14117 VAR_002268 p.Gln334Arg LP/P rs121964923 Dihydropyrimidinase deficiency (DPYSD) [MIM:222748]
DPYS Q14117 VAR_002269 p.Trp360Arg LP/P rs121964924 Dihydropyrimidinase deficiency (DPYSD) [MIM:222748]
DPYS Q14117 VAR_002270 p.Gly435Arg LP/P rs267606773 Dihydropyrimidinase deficiency (DPYSD) [MIM:222748]
DPYS Q14117 VAR_002271 p.Arg490Thr LP/P - Dihydropyrimidinase deficiency (DPYSD) [MIM:222748]
DPYSL2 Q16555 VAR_022016 p.Ala118Thr LB/B rs2228979 -
DPYSL2 Q16555 VAR_036316 p.Arg481Cys US rs1337153084 A colorectal cancer sample
DPYSL3 Q14195 VAR_020485 p.Ala442Ser LB/B rs2304044 -
DR1 Q01658 VAR_034506 p.Glu171Asp LB/B rs3088371 -
DRAM2 Q6UX65 VAR_075074 p.Tyr27His LP/P rs786205662 Cone-rod dystrophy 21 (CORD21) [MIM:616502]
DRAM2 Q6UX65 VAR_075075 p.Ser44Asn LP/P rs786205665 Cone-rod dystrophy 21 (CORD21) [MIM:616502]
DRAM2 Q6UX65 VAR_075077 p.His121Leu LP/P rs786205664 Cone-rod dystrophy 21 (CORD21) [MIM:616502]
DRAXIN Q8NBI3 VAR_030114 p.Leu37Phe LB/B rs11121804 -
DRC1 Q96MC2 VAR_030625 p.Lys357Glu LB/B rs3795958 -
DRC1 Q96MC2 VAR_030626 p.Trp399Arg LB/B rs939820 -
DRC1 Q96MC2 VAR_030627 p.Val633Phe LB/B rs12623642 -
DRC1 Q96MC2 VAR_030628 p.Ser702Ile LB/B rs3172008 -
DRC1 Q96MC2 VAR_057758 p.Val734Met LB/B rs35313480 -
DRC3 Q9H069 VAR_025625 p.Arg191Trp LB/B rs4584886 -
DRC3 Q9H069 VAR_051120 p.Arg159Gln LB/B rs8072048 -
DRC3 Q9H069 VAR_051121 p.Ala364Val LB/B rs11656629 -
DRC7 Q8IY82 VAR_030898 p.Cys186Ser LB/B rs7196016 -
DRC7 Q8IY82 VAR_030899 p.Pro433Leu LB/B rs3809611 -
DRC7 Q8IY82 VAR_030900 p.Asn581Lys LB/B rs2923144 -
DRC7 Q8IY82 VAR_030901 p.Cys766Arg LB/B rs2923147 -
DRC7 Q8IY82 VAR_050738 p.Leu120Met LB/B rs11649000 -
DRC7 Q8IY82 VAR_055286 p.Pro521His LB/B rs17853687 -
DRC7 Q8IY82 VAR_061580 p.Asp51Asn LB/B rs55645458 -
DRC7 Q8IY82 VAR_061581 p.Val241Glu LB/B rs58373934 -
DRD1 P21728 VAR_014670 p.Thr37Pro LB/B rs5327 -
DRD1 P21728 VAR_014671 p.Thr37Arg LB/B rs879844008 -
DRD1 P21728 VAR_014672 p.Arg50Ser LB/B rs5330 -
DRD1 P21728 VAR_014673 p.Ser199Ala LB/B rs5331 -
DRD1 P21728 VAR_064577 p.Lys81Arg LB/B - -
DRD1 P21728 VAR_064578 p.Ser259Tyr LB/B rs74414188 -
DRD2 P14416 VAR_003462 p.Ser311Cys LB/B rs1801028 -
DRD2 P14416 VAR_014674 p.Pro310Ser LB/B rs1800496 -
DRD2 P14416 VAR_017143 p.Val154Ile US rs104894220 -
DRD2 P14416 VAR_064579 p.Lys327Glu LB/B rs71653614 -
DRD3 P35462 VAR_003463 p.Ser9Gly LB/B rs6280 -
DRD4 P21917 VAR_003464 p.Val194Gly LB/B rs1800443 -
DRD5 P21918 VAR_003458 p.Ala269Val LB/B rs538877978 -

DRD5 P21918 VAR_003459 p.Pro330Gln LB/B rs1800762 -
DRD5 P21918 VAR_003460 p.Asn351Asp LB/B rs1577263526 -
DRD5 P21918 VAR_003461 p.Ser453Cys LB/B rs776114395 -
DRD5 P21918 VAR_011837 p.Leu88Arg LB/B rs6282 -
DRD5 P21918 VAR_024254 p.Val238Ile LB/B rs2227852 -
DRD5 P21918 VAR_029210 p.Cys62Ser LB/B rs2227840 -
DRD5 P21918 VAR_029211 p.Gly110Glu LB/B rs2227849 -
DRD5 P21918 VAR_029212 p.Phe207Val LB/B rs2227845 -
DRD5 P21918 VAR_029213 p.Ser233Asn LB/B rs2227843 -
DRD5 P21918 VAR_029215 p.Ala286Val LB/B rs2227850 -
DRD5 P21918 VAR_061217 p.Thr297Pro LB/B rs2227851 -
DRG2 P55039 VAR_067452 p.Thr194Met LB/B rs17855350 -
DRG2 P55039 VAR_067453 p.Ser224Thr LB/B rs61256737 -
DRICH1 Q6PGQ1 VAR_039014 p.Ile4Thr LB/B rs3827318 -
DROSHA Q9NRR4 VAR_051866 p.Pro67Thr LB/B rs35342496 -
DROSHA Q9NRR4 VAR_061778 p.Ser321Leu LB/B rs55656741 -
DRP2 Q13474 VAR_033898 p.Val68Leu LB/B rs7066252 -
DSC1 Q08554 VAR_055579 p.Val460Ile LB/B rs17800159 -
DSC1 Q08554 VAR_055580 p.Cys848Phe LB/B rs985861 -
DSC1 Q08554 VAR_061059 p.Ser93Phe LB/B rs35338395 -
DSC2 Q02487 VAR_024388 p.Ile776Val LB/B rs1893963 -
DSC2 Q02487 VAR_029480 p.Asn11Ser LB/B rs868333 -
DSC2 Q02487 VAR_062391 p.Thr358Ile LB/B rs139399951 -
DSC2 Q02487 VAR_062392 p.Arg798Gln LB/B rs61731921 -
DSC2 Q02487 VAR_065687 p.Arg203Cys LP/P rs142331975 Arrhythmogenic right ventricular dysplasia, familial, 11 (ARVD11) [MIM:610476]
DSC2 Q02487 VAR_065688 p.Ile231Thr LP/P rs1390387214 Arrhythmogenic right ventricular dysplasia, familial, 11 (ARVD11) [MIM:610476]
DSC2 Q02487 VAR_065689 p.Thr275Met LP/P rs397517404 Arrhythmogenic right ventricular dysplasia, familial, 11 (ARVD11) [MIM:610476]
DSC2 Q02487 VAR_065690 p.Thr340Ala LP/P rs368299411 Arrhythmogenic right ventricular dysplasia, familial, 11 (ARVD11) [MIM:610476]
DSC2 Q02487 VAR_065691 p.Ala596Val LB/B rs148185335 -
DSC2 Q02487 VAR_065692 p.Gln638His LB/B rs147742157 -
DSC2 Q02487 VAR_078340 p.Val364Met LP/P - Arrhythmogenic right ventricular dysplasia, familial, 11 (ARVD11) [MIM:610476]
DSC3 Q14574 VAR_048515 p.Ala28Asp LB/B rs2852003 -
DSC3 Q14574 VAR_048516 p.Ser78Thr LB/B rs276937 -
DSC3 Q14574 VAR_048517 p.Arg102Lys LB/B rs276938 -
DSC3 Q14574 VAR_048518 p.Lys180Gln LB/B rs35296997 -
DSC3 Q14574 VAR_048519 p.Arg199Trp LB/B rs276921 -
DSC3 Q14574 VAR_048520 p.Asn239Ser LB/B rs35630063 -
DSCAM O60469 VAR_020080 p.Asp232Glu LB/B rs2297270 -
DSCAML1 Q8TD84 VAR_035512 p.Val659Ile US rs533175875 A colorectal cancer sample
DSCAML1 Q8TD84 VAR_035513 p.Val1702Ile US rs147907435 A colorectal cancer sample
DSCC1 Q9BVC3 VAR_038682 p.His376Arg LB/B rs1055130 -
DSCR9 P59020 VAR_024340 p.Arg76Leu LB/B rs13864 -
DSCR9 P59020 VAR_033849 p.Gly23Val LB/B rs1888464 -
DSE Q9UL01 VAR_034481 p.Thr25Ile LB/B rs10485183 -

DSE Q9UL01 VAR_053833 p.Pro34Leu LB/B rs35548455 -
DSE Q9UL01 VAR_053834 p.Ile282Val LB/B rs34994230 -
DSE Q9UL01 VAR_070911 p.Ser268Leu LP/P rs398122361 Ehlers-Danlos syndrome, musculocontractural type 2 (EDSMC2) [MIM:615539]
DSEL Q8IZU8 VAR_030833 p.Pro673Ser LB/B rs2279269 -
DSEL Q8IZU8 VAR_030834 p.Tyr730Cys LB/B rs12953840 -
DSEL Q8IZU8 VAR_036528 p.Lys1090Glu US - A colorectal cancer sample
DSEL Q8IZU8 VAR_057759 p.Thr832Ser LB/B rs35479856 -
DSG1 Q02413 VAR_020364 p.Tyr841Phe LB/B rs3752095 -
DSG1 Q02413 VAR_024385 p.Asn493Thr LB/B rs8091003 -
DSG1 Q02413 VAR_024386 p.Thr498Asn LB/B rs8091117 -
DSG1 Q02413 VAR_055573 p.Thr395Ser LB/B rs16961655 -
DSG1 Q02413 VAR_055574 p.Tyr528Ser LB/B rs16961689 -
DSG1 Q02413 VAR_055575 p.Asp538Asn LB/B rs34302455 -
DSG1 Q02413 VAR_055576 p.Met665Ile LB/B rs35360042 -
DSG1 Q02413 VAR_055577 p.Leu821Gln LB/B rs16961692 -
DSG1 Q02413 VAR_060248 p.Met11Val LB/B rs1426310 -
DSG1 Q02413 VAR_060249 p.Asp828Asn LB/B rs3752094 -
DSG2 Q14126 VAR_029365 p.Arg46Gln LP/P rs121913008 Arrhythmogenic right ventricular dysplasia, familial, 10 (ARVD10) [MIM:610193]
DSG2 Q14126 VAR_029366 p.Arg49His LP/P rs121913006 Arrhythmogenic right ventricular dysplasia, familial, 10 (ARVD10) [MIM:610193]
DSG2 Q14126 VAR_029367 p.Cys507Tyr LP/P rs121913009 Arrhythmogenic right ventricular dysplasia, familial, 10 (ARVD10) [MIM:610193]
DSG2 Q14126 VAR_029368 p.Gly812Cys LP/P rs121913010 Arrhythmogenic right ventricular dysplasia, familial, 10 (ARVD10) [MIM:610193]
DSG2 Q14126 VAR_048508 p.Tyr89Cys LB/B rs2230232 -
DSG2 Q14126 VAR_048509 p.Ile293Val LB/B rs2230234 -
DSG2 Q14126 VAR_048510 p.Val515Ile LB/B rs2230235 -
DSG2 Q14126 VAR_048511 p.Arg773Lys LB/B rs2278792 -
DSG2 Q14126 VAR_048512 p.Met863Leu LB/B rs16962093 -
DSG2 Q14126 VAR_048513 p.Thr903Ile LB/B rs34065672 -
DSG2 Q14126 VAR_062387 p.Val56Met LB/B rs121913013 -
DSG2 Q14126 VAR_062388 p.Val158Gly LB/B rs191143292 -
DSG2 Q14126 VAR_062389 p.Glu713Lys LB/B rs79241126 -
DSG2 Q14126 VAR_062390 p.Val920Gly LB/B rs142841727 -
DSG2 Q14126 VAR_065686 p.Thr335Ala LP/P rs191564916 Arrhythmogenic right ventricular dysplasia, familial, 10 (ARVD10) [MIM:610193]
DSG3 P32926 VAR_055578 p.Val509Met LB/B rs16961975 -
DSG3 P32926 VAR_059178 p.Thr912Ala LB/B rs1380866 -
DSG4 Q86SJ6 VAR_024387 p.Ile644Leu LB/B rs4799570 -
DSG4 Q86SJ6 VAR_033700 p.Ile535Thr LB/B rs7229252 -
DSG4 Q86SJ6 VAR_048514 p.Ala154Thr LB/B rs13381457 -
DSP P15924 VAR_015402 p.Ser299Arg US rs121912992 Arrhythmogenic right ventricular dysplasia, familial, 8 (ARVD8) [MIM:607450]
DSP P15924 VAR_015569 p.Asn287Lys LP/P rs121912993 Skin fragility-woolly hair syndrome (SFWHS) [MIM:607655]
DSP P15924 VAR_015570 p.Arg2366Cys LP/P rs28931610 Skin fragility-woolly hair syndrome (SFWHS) [MIM:607655]

DSP P15924 VAR_018158 p.Gly2375Arg LB/B rs376923069 -
DSP P15924 VAR_020468 p.Tyr1512Cys LB/B rs2076299 -
DSP P15924 VAR_023814 p.Arg1255Lys LP/P rs777407386 Arrhythmogenic right ventricular dysplasia, familial, 8 (ARVD8) [MIM:607450]
DSP P15924 VAR_023815 p.Arg1738Gln LB/B rs6929069 -
DSP P15924 VAR_023816 p.Arg1775Ile US rs34738426 Arrhythmogenic right ventricular dysplasia, familial, 8 (ARVD8) [MIM:607450]
DSP P15924 VAR_033862 p.Ile305Phe LB/B rs17604693 -
DSP P15924 VAR_065693 p.Ile445Val LP/P rs934142779 Arrhythmogenic right ventricular dysplasia, familial, 8 (ARVD8) [MIM:607450]
DSP P15924 VAR_065694 p.Ala1505Val LB/B rs375919492 -
DSP P15924 VAR_065695 p.Asn1526Lys LB/B rs28763966 -
DSP P15924 VAR_065696 p.Arg1537Cys LB/B rs28763967 -
DSP P15924 VAR_065697 p.Glu1833Val LB/B rs78652302 -
DSP P15924 VAR_072432 p.Thr564Ile LP/P rs606231295 Cardiomyopathy, dilated, with woolly hair, keratoderma, and tooth agenesis (DCWHKTA) [MIM:615821]
DSP P15924 VAR_072433 p.Ser597Leu LP/P rs606231294 Cardiomyopathy, dilated, with woolly hair, keratoderma, and tooth agenesis (DCWHKTA) [MIM:615821]
DSPP Q9NZW4 VAR_012280 p.Pro17Thr LP/P rs121912986 Deafness, autosomal dominant, 39, with dentinogenesis imperfecta 1 (DFNA39/DGI1) [MIM:605594]
DSPP Q9NZW4 VAR_012281 p.Val18Phe LP/P rs121912987 Deafness, autosomal dominant, 39, with dentinogenesis imperfecta 1 (DFNA39/DGI1) [MIM:605594]
DSPP Q9NZW4 VAR_012281 p.Val18Phe LP/P rs121912987 Dentinogenesis imperfecta, Shields type 3 (DGI3) [MIM:125500]
DSPP Q9NZW4 VAR_030661 p.Arg68Trp LP/P rs36094464 Dentinogenesis imperfecta, Shields type 2 (DGI2) [MIM:125490]
DSPP Q9NZW4 VAR_036861 p.Tyr6Asp LP/P rs121912988 Dentin dysplasia 2 (DTDP2) [MIM:125420]
DSPP Q9NZW4 VAR_036862 p.Ala15Val LP/P rs121912989 Dentinogenesis imperfecta, Shields type 2 (DGI2) [MIM:125490]
DSPP Q9NZW4 VAR_047551 p.Asp243Asn LB/B rs3750025 -
DSPP Q9NZW4 VAR_054443 p.Pro17Ser LB/B rs121912986 -
DSPP Q9NZW4 VAR_070252 p.Pro17Leu LP/P - Dentinogenesis imperfecta, Shields type 3 (DGI3) [MIM:125500]
DSPP Q9NZW4 VAR_070253 p.Val18Asp LP/P - Dentinogenesis imperfecta, Shields type 2 (DGI2) [MIM:125490]
DST Q03001 VAR_063045 p.Asn1319Lys LB/B rs35014998 -
DST Q03001 VAR_063046 p.Gln2332Arg LB/B rs16888053 -
DST Q03001 VAR_063047 p.Gln3720Arg LB/B rs4712138 -
DST Q03001 VAR_063048 p.Thr5138Ala LB/B rs4715631 -
DSTN P60981 VAR_036459 p.Gly139Glu US - A colorectal cancer sample
DSTYK Q6XUX3 VAR_057101 p.Leu432Val LB/B rs35845538 -
DSTYK Q6XUX3 VAR_071324 p.Arg29Gln US rs200780796 Congenital anomalies of the kidney and urinary tract 1 (CAKUT1) [MIM:610805]
DSTYK Q6XUX3 VAR_071325 p.Asp200Gly LP/P - Congenital anomalies of the kidney and urinary tract 1 (CAKUT1) [MIM:610805]
DSTYK Q6XUX3 VAR_071326 p.Ser843Leu LP/P rs778586547 Congenital anomalies of the kidney and urinary tract 1 (CAKUT1) [MIM:610805]
DTD2 Q96FN9 VAR_028802 p.Arg6Trp LB/B rs17097904 -
DTHD1 Q6ZMT9 VAR_046339 p.Gly26Arg LB/B rs16992035 -

DTHD1	Q6ZMT9	VAR_046340	p.Val179Asp	LB/B	rs1995319	-
DTHD1	Q6ZMT9	VAR_046341	p.Arg552Cys	LB/B	rs12507599	-
DTHD1	Q6ZMT9	VAR_046342	p.Arg662His	LB/B	rs9654132	-
DTL	Q9NZJ0	VAR_030353	p.Ala436Val	LB/B	rs3135474	-
DTL	Q9NZJ0	VAR_030354	p.Lys694Thr	LB/B	rs6540718	-
DTL	Q9NZJ0	VAR_062095	p.Ser425Asn	LB/B	rs35137676	-
DTNA	Q9Y4J8	VAR_026744	p.Pro121Leu	US	rs104894654	Left ventricular non-compaction 1 (LVNC1) [MIM:604169]
DTNA	Q9Y4J8	VAR_055320	p.Ala180Glu	LB/B	rs1048081	-
DTNB	O60941	VAR_069146	p.Pro97His	LB/B	rs17854576	-
DTNB	O60941	VAR_069147	p.Ile116Thr	LB/B	rs17854577	-
DTNBP1	Q96EV8	VAR_029644	p.Pro272Ser	LB/B	rs17470454	-
DTNBP1	Q96EV8	VAR_053069	p.Gly214Asp	LB/B	rs16876589	-
DTWD1	Q8N5C7	VAR_036757	p.Leu9Pro	LB/B	rs11539522	-
DTWD1	Q8N5C7	VAR_036758	p.Glu13Lys	LB/B	rs11539519	-
DTWD1	Q8N5C7	VAR_036759	p.Ser25Pro	LB/B	rs11539521	-
DTX2	Q86UW9	VAR_016920	p.Ala94Thr	LB/B	rs2462312	-
DTX2	Q86UW9	VAR_016921	p.Gly384Glu	LB/B	rs1638152	-
DTX2	Q86UW9	VAR_016922	p.Thr421Ala	LB/B	rs6979487	-
DTX3L	Q8TDB6	VAR_036098	p.Lys209Asn	US	-	A breast cancer sample
DTX3L	Q8TDB6	VAR_048895	p.Arg425Lys	LB/B	rs2332285	-
DTX3L	Q8TDB6	VAR_048896	p.Lys668Met	LB/B	rs9868175	-
DUOX1	Q9NRD9	VAR_025321	p.Cys1026Arg	LB/B	rs16939752	-
DUOX1	Q9NRD9	VAR_025322	p.Leu1178Phe	LB/B	rs2458236	-
DUOX1	Q9NRD9	VAR_049104	p.Ile962Thr	LB/B	rs16939743	-
DUOX2	Q9NRD8	VAR_025323	p.Gln36His	LP/P	-	Thyroid dyshormonogenesis 6 (TDH6) [MIM:607200]
DUOX2	Q9NRD8	VAR_025324	p.Pro138Leu	LB/B	rs2001616	-
DUOX2	Q9NRD8	VAR_025325	p.Arg376Trp	LP/P	rs119472029	Thyroid dyshormonogenesis 6 (TDH6) [MIM:607200]
DUOX2	Q9NRD8	VAR_047075	p.Ser1067Leu	LB/B	rs269868	-
DUOX2	Q9NRD8	VAR_061177	p.His678Arg	LB/B	rs57659670	-
DUOX2	Q9NRD8	VAR_064619	p.Gly1518Ser	LP/P	rs368512412	Thyroid dyshormonogenesis 6 (TDH6) [MIM:607200]
DUOX2	Q9NRD8	VAR_075549	p.Arg1211Cys	US	rs374410986	-
DUOX2	Q9NRD8	VAR_075550	p.Arg1492Cys	US	rs747720952	-
DUOXA1	Q1HG43	VAR_029630	p.Ser313Gly	LB/B	rs16977686	-
DUOXA1	Q1HG43	VAR_057753	p.Pro19Leu	LB/B	rs34734975	-
DUOXA2	Q1HG44	VAR_047367	p.Arg100Gly	LB/B	rs2576090	-
DUOXA2	Q1HG44	VAR_074025	p.Ile26Met	US	-	Thyroid dyshormonogenesis 5 (TDH5) [MIM:274900]
DUS3L	Q96G46	VAR_027092	p.Arg185Gly	LB/B	rs2436487	-
DUS3L	Q96G46	VAR_027093	p.Asp609Asn	LB/B	rs12977803	-
DUS4L	O95620	VAR_027094	p.Arg230Gln	LB/B	rs6957510	-
DUS4L	O95620	VAR_048938	p.Thr178Ala	LB/B	rs6956789	-
DUSP1	P28562	VAR_025201	p.Ala56Thr	LB/B	rs34013988	-
DUSP1	P28562	VAR_025202	p.Tyr187His	LB/B	rs34471628	-
DUSP12	Q9UNI6	VAR_033899	p.Ala51Glu	LB/B	rs35106830	-
DUSP13	Q6B8I1	VAR_058495	p.Lys73Arg	LB/B	rs7912300	-
DUSP13	Q9UII6	VAR_025431	p.Cys156Tyr	LB/B	rs3088142	-
DUSP13	Q9UII6	VAR_057130	p.Arg62Gln	LB/B	rs16932004	-

DUSP13 Q9UII6 VAR_057131 p.Arg190Gly LB/B rs16931996 -
DUSP16 Q9BY84 VAR_051753 p.Thr23Met LB/B rs36049447 -
DUSP16 Q9BY84 VAR_051754 p.Val366Met LB/B rs3809199 -
DUSP19 Q8WTR2 VAR_051755 p.Ser216Arg LB/B rs16823987 -
DUSP21 Q9H596 VAR_019423 p.Met186Thr LB/B rs1045031 -
DUSP21 Q9H596 VAR_035644 p.Arg167Cys US rs1372839121 A colorectal cancer sample
DUSP22 Q9NRW4 VAR_026912 p.Arg119His LB/B rs7768224 -
DUSP23 Q9BVJ7 VAR_023199 p.Gly131Ser LB/B rs1129923 -
DUSP23 Q9BVJ7 VAR_051756 p.Glu124Val LB/B rs11544443 -
DUSP29 Q68J44 VAR_033376 p.Ser137Arg LB/B rs16931938 -
DUSP29 Q68J44 VAR_051757 p.Asp66Asn LB/B rs11594934 -
DUSP5 Q16690 VAR_020298 p.Glu154Asp LB/B rs2282238 -
DUSP5 Q16690 VAR_047368 p.Pro322Leu LB/B rs35101549 -
DUSP5 Q16690 VAR_059777 p.Ala220Thr LB/B rs1889566 -
DUSP5 Q16690 VAR_059778 p.Ala220Val LB/B rs1889565 -
DUSP6 Q16828 VAR_015113 p.Val114Leu LB/B rs2279574 -
DUSP6 Q16828 VAR_051750 p.Ser144Ala LB/B rs770087 -
DUSP6 Q16828 VAR_051751 p.Asn313Ile LB/B rs12828557 -
DUSP6 Q16828 VAR_069943 p.Phe77Ile LP/P rs587776978 Hypogonadotropic hypogonadism 19 with or without anosmia (HH19) [MIM:615269]
DUSP6 Q16828 VAR_069944 p.Ser182Phe LP/P rs139318648 Hypogonadotropic hypogonadism 19 with or without anosmia (HH19) [MIM:615269]
DUSP6 Q16828 VAR_069945 p.Asn189Ser LP/P rs143946794 Hypogonadotropic hypogonadism 19 with or without anosmia (HH19) [MIM:615269]
DUSP6 Q16828 VAR_069946 p.Thr346Met LP/P rs146089505 Hypogonadotropic hypogonadism 19 with or without anosmia (HH19) [MIM:615269]
DUSP7 Q16829 VAR_051752 p.Ser235Asn LB/B rs34821455 -
DUT P33316 VAR_022314 p.Pro100Ser LB/B rs28381104 -
DUX5 Q96PT3 VAR_059347 p.Ala60Val LB/B rs10865697 -
DUX5 Q96PT3 VAR_059348 p.Pro118Leu LB/B rs9755233 -
DUX5 Q96PT3 VAR_059349 p.Gln119Pro LB/B rs12374009 -
DUX5 Q96PT3 VAR_059350 p.Ser180Ala LB/B rs12632317 -
DUX5 Q96PT3 VAR_059351 p.Ser180Thr LB/B rs12632317 -
DVL2 O14641 VAR_064708 p.Ile282Thr US - -
DVL3 Q92997 VAR_025519 p.Trp433Leu LB/B rs17853048 -
DVL3 Q92997 VAR_036116 p.Arg216Thr US - A breast cancer sample
DXO O77932 VAR_027492 p.Ser28Thr LB/B rs1056694 -
DXO O77932 VAR_027493 p.Asp63Glu LB/B rs2746396 -
DXO O77932 VAR_027494 p.His261Gln LB/B rs17207867 -
DXO O77932 VAR_027495 p.Ala332Val LB/B rs12205138 -
DYDC2 Q96IM9 VAR_048889 p.Gly143Ser LB/B rs1047951 -
DYM Q7RTS9 VAR_022740 p.Glu87Lys LP/P rs120074164 Smith-McCort dysplasia 1 (SMC1) [MIM:607326]
DYM Q7RTS9 VAR_054499 p.Asn469Tyr LP/P rs120074163 Dyggve-Melchior-Clausen syndrome (DMC) [MIM:223800]
DYM Q7RTS9 VAR_065293 p.Cys542Arg LP/P rs120074165 Smith-McCort dysplasia 1 (SMC1) [MIM:607326]
DYNAP Q8N1N2 VAR_033751 p.Val38Ala LB/B rs35428499 -
DYNAP Q8N1N2 VAR_033752 p.Thr189Pro LB/B rs9947055 -
DYNC1H1 Q14204 VAR_020889 p.Asp3902Asn LB/B rs17512818 -
DYNC1H1 Q14204 VAR_020890 p.His4029Gln LB/B rs10129889 -

DYNC1H1 Q14204 VAR_065085 p.His3822Pro LP/P rs387906739 Mental retardation, autosomal dominant 13 (MRD13) [MIM:614563]
DYNC1H1 Q14204 VAR_066651 p.His306Arg LP/P rs387906738 Charcot-Marie-Tooth disease 2O (CMT2O) [MIM:614228]
DYNC1H1 Q14204 VAR_066651 p.His306Arg LP/P rs387906738 Spinal muscular atrophy, lower extremity-predominant 1, autosomal dominant (SMALED1) [MIM:158600]
DYNC1H1 Q14204 VAR_067820 p.Ile584Leu LP/P rs387906741 Spinal muscular atrophy, lower extremity-predominant 1, autosomal dominant (SMALED1) [MIM:158600]
DYNC1H1 Q14204 VAR_067821 p.Lys671Glu LP/P rs387906742 Spinal muscular atrophy, lower extremity-predominant 1, autosomal dominant (SMALED1) [MIM:158600]
DYNC1H1 Q14204 VAR_067822 p.Tyr970Cys LP/P rs387906743 Spinal muscular atrophy, lower extremity-predominant 1, autosomal dominant (SMALED1) [MIM:158600]
DYNC1H1 Q14204 VAR_067823 p.Glu1518Lys LP/P rs387906740 Mental retardation, autosomal dominant 13 (MRD13) [MIM:614563]
DYNC1H1 Q14204 VAR_069437 p.Glu142Ala LB/B - -
DYNC1H1 Q14204 VAR_069438 p.Val1250Leu LB/B rs369914512 -
DYNC1H1 Q14204 VAR_069439 p.Val2247Met LB/B rs1064796963 -
DYNC1H1 Q14204 VAR_069440 p.Arg4143Cys LB/B rs1316357429 -
DYNC1H1 Q14204 VAR_069441 p.Ala4285Ser LB/B rs749486351 -
DYNC1H1 Q14204 VAR_069442 p.Ala4421Thr LB/B rs376492799 -
DYNC1H1 Q14204 VAR_069443 p.Ile4507Ser LB/B - -
DYNC1H1 Q14204 VAR_069444 p.Ser4603Gly LB/B - -
DYNC1H1 Q14204 VAR_070580 p.Lys129Ile LP/P rs1555407885 Mental retardation, autosomal dominant 13 (MRD13) [MIM:614563]
DYNC1H1 Q14204 VAR_070582 p.Arg1567Gln LP/P rs797044901 Mental retardation, autosomal dominant 13 (MRD13) [MIM:614563]
DYNC1H1 Q14204 VAR_070583 p.Arg1962Cys LP/P rs879253881 Mental retardation, autosomal dominant 13 (MRD13) [MIM:614563]
DYNC1H1 Q14204 VAR_070584 p.Lys3241Thr LP/P rs1555411145 Mental retardation, autosomal dominant 13 (MRD13) [MIM:614563]
DYNC1H1 Q14204 VAR_070585 p.Lys3336Asn LP/P rs397509410 Mental retardation, autosomal dominant 13 (MRD13) [MIM:614563]
DYNC1H1 Q14204 VAR_070586 p.Arg3344Gln LP/P rs397509412 Mental retardation, autosomal dominant 13 (MRD13) [MIM:614563]
DYNC1H1 Q14204 VAR_070587 p.Arg3384Gln LP/P rs397509411 Mental retardation, autosomal dominant 13 (MRD13) [MIM:614563]
DYNC1H1 Q14204 VAR_072092 p.Gln1194Arg LP/P rs1555408964 Charcot-Marie-Tooth disease 2O (CMT2O) [MIM:614228]
DYNC1H1 Q14204 VAR_072093 p.Glu3048Lys LP/P rs1555410941 Charcot-Marie-Tooth disease 2O (CMT2O) [MIM:614228]
DYNC1H1 Q14204 VAR_073155 p.Glu94Lys US - -
DYNC1H1 Q14204 VAR_073156 p.Arg264Leu LP/P rs713993043 Spinal muscular atrophy, lower extremity-predominant 1, autosomal dominant (SMALED1) [MIM:158600]
DYNC1H1 Q14204 VAR_073157 p.Arg598Cys LP/P rs587780564 Charcot-Marie-Tooth disease 2O (CMT2O) [MIM:614228]
DYNC1H1 Q14204 VAR_073157 p.Arg598Cys LP/P rs587780564 Spinal muscular atrophy, lower extremity-predominant 1, autosomal dominant (SMALED1) [MIM:158600]
DYNC1H1 Q14204 VAR_078241 p.Pro776Leu LP/P rs1057518083 Spinal muscular atrophy, lower extremity-predominant 1, autosomal dominant (SMALED1) [MIM:158600]

DYNC1H1 Q14204 VAR_078242 p.Gly1132Glu LP/P - Spinal muscular atrophy, lower extremity-predominant 1, autosomal dominant (SMALED1) [MIM:158600]
DYNC1I1 O14576 VAR_048905 p.Asn582Thr LB/B rs35077523 -
DYNC1I1 O14576 VAR_064709 p.His373Leu US - -
DYNC1I2 Q13409 VAR_082947 p.Tyr247Cys LP/P - Neurodevelopmental disorder with microcephaly and structural brain anomalies (NEDMIBA) [MIM:618492]
DYNC1I2 Q13409 VAR_082949 p.Pro516Ala LB/B rs767705533 -
DYNC1LI1 Q9Y6G9 VAR_023325 p.Gln277Arg LB/B rs2303857 -
DYNC1LI1 Q9Y6G9 VAR_061141 p.Met147Thr LB/B rs34181332 -
DYNC2H1 Q8NCM8 VAR_038862 p.Thr302Pro LB/B rs12803695 -
DYNC2H1 Q8NCM8 VAR_038863 p.Gln304Leu LB/B rs12146610 -
DYNC2H1 Q8NCM8 VAR_038864 p.His341Tyr LB/B rs17301182 -
DYNC2H1 Q8NCM8 VAR_038865 p.Arg456Gln LB/B rs17099969 -
DYNC2H1 Q8NCM8 VAR_038866 p.Arg789Lys LB/B rs7358374 -
DYNC2H1 Q8NCM8 VAR_038867 p.Arg1221Lys LB/B rs12794914 -
DYNC2H1 Q8NCM8 VAR_038868 p.Thr1288Ala LB/B rs17301750 -
DYNC2H1 Q8NCM8 VAR_038869 p.Lys1413Arg LB/B rs688906 -
DYNC2H1 Q8NCM8 VAR_038870 p.Arg2871Gln LB/B rs589623 -
DYNC2H1 Q8NCM8 VAR_038871 p.Ala3680Val LB/B rs10895391 -
DYNC2H1 Q8NCM8 VAR_038872 p.Ser3976Asn LB/B rs4754914 -
DYNC2H1 Q8NCM8 VAR_038873 p.Gln4139Pro LB/B rs1793493 -
DYNC2H1 Q8NCM8 VAR_063242 p.Phe209Ile LP/P rs771511132 Short-rib thoracic dysplasia 3 with or without polydactyly (SRTD3) [MIM:613091]
DYNC2H1 Q8NCM8 VAR_063243 p.Arg587Cys LP/P rs137853030 Short-rib thoracic dysplasia 3 with or without polydactyly (SRTD3) [MIM:613091]
DYNC2H1 Q8NCM8 VAR_063244 p.Ile1240Thr LP/P rs137853028 Short-rib thoracic dysplasia 3 with or without polydactyly (SRTD3) [MIM:613091]
DYNC2H1 Q8NCM8 VAR_063245 p.Gln1537Arg LP/P rs137853033 Short-rib thoracic dysplasia 3 with or without polydactyly (SRTD3) [MIM:613091]
DYNC2H1 Q8NCM8 VAR_063246 p.Thr1987Ala LP/P rs137853035 Short-rib thoracic dysplasia 3 with or without polydactyly (SRTD3) [MIM:613091]
DYNC2H1 Q8NCM8 VAR_063247 p.Met1991Leu LP/P rs137853025 Short-rib thoracic dysplasia 3 with or without polydactyly (SRTD3) [MIM:613091]
DYNC2H1 Q8NCM8 VAR_063248 p.Arg2205His LP/P rs137853031 Short-rib thoracic dysplasia 3 with or without polydactyly (SRTD3) [MIM:613091]
DYNC2H1 Q8NCM8 VAR_063249 p.Gly2461Val LP/P rs137853034 Short-rib thoracic dysplasia 3 with or without polydactyly (SRTD3) [MIM:613091]
DYNC2H1 Q8NCM8 VAR_063250 p.Asp3015Gly LP/P rs137853027 Short-rib thoracic dysplasia 3 with or without polydactyly (SRTD3) [MIM:613091]
DYNC2H1 Q8NCM8 VAR_063251 p.Leu3762Val LP/P - Short-rib thoracic dysplasia 3 with or without polydactyly (SRTD3) [MIM:613091]
DYNC2H1 Q8NCM8 VAR_069591 p.Arg330Cys LP/P rs397514637 Short-rib thoracic dysplasia 3 with or without polydactyly (SRTD3) [MIM:613091]
DYNC2H1 Q8NCM8 VAR_069592 p.Arg338Gly LP/P rs1322077043 Short-rib thoracic dysplasia 3 with or without polydactyly (SRTD3) [MIM:613091]
DYNC2H1 Q8NCM8 VAR_069593 p.Arg430Cys LP/P rs374073337 Short-rib thoracic dysplasia 3 with or without polydactyly (SRTD3) [MIM:613091]
DYNC2H1 Q8NCM8 VAR_069594 p.Lys495Arg LP/P - Short-rib thoracic dysplasia 3 with or without polydactyly (SRTD3) [MIM:613091]

DYNC2H1 Q8NCM8 VAR_069595 p.Leu871Pro LP/P - Short-rib thoracic dysplasia 3 with or without polydactyly (SRTD3) [MIM:613091]
DYNC2H1 Q8NCM8 VAR_069596 p.Leu1228Ile US rs189806840 Short-rib thoracic dysplasia 3 with or without polydactyly (SRTD3) [MIM:613091]
DYNC2H1 Q8NCM8 VAR_069597 p.Met1379Val LP/P - Short-rib thoracic dysplasia 3 with or without polydactyly (SRTD3) [MIM:613091]
DYNC2H1 Q8NCM8 VAR_069598 p.Arg1423Cys LP/P rs745870321 Short-rib thoracic dysplasia 3 with or without polydactyly (SRTD3) [MIM:613091]
DYNC2H1 Q8NCM8 VAR_069599 p.Gly1442Asp LP/P rs763571787 Short-rib thoracic dysplasia 3 with or without polydactyly (SRTD3) [MIM:613091]
DYNC2H1 Q8NCM8 VAR_069600 p.Met1991Lys LP/P rs1202784860 Short-rib thoracic dysplasia 3 with or without polydactyly (SRTD3) [MIM:613091]
DYNC2H1 Q8NCM8 VAR_069601 p.Met2227Val LP/P rs750249486 Short-rib thoracic dysplasia 3 with or without polydactyly (SRTD3) [MIM:613091]
DYNC2H1 Q8NCM8 VAR_069602 p.Ala2304Thr LP/P rs747348765 Short-rib thoracic dysplasia 3 with or without polydactyly (SRTD3) [MIM:613091]
DYNC2H1 Q8NCM8 VAR_069603 p.Asn2362Ser LP/P - Short-rib thoracic dysplasia 3 with or without polydactyly (SRTD3) [MIM:613091]
DYNC2H1 Q8NCM8 VAR_069604 p.Arg2481Gln LP/P rs781326398 Short-rib thoracic dysplasia 3 with or without polydactyly (SRTD3) [MIM:613091]
DYNC2H1 Q8NCM8 VAR_069605 p.Pro2496Ser LP/P rs397514636 Short-rib thoracic dysplasia 3 with or without polydactyly (SRTD3) [MIM:613091]
DYNC2H1 Q8NCM8 VAR_069606 p.Arg2532Trp LP/P rs1350329646 Short-rib thoracic dysplasia 3 with or without polydactyly (SRTD3) [MIM:613091]
DYNC2H1 Q8NCM8 VAR_069607 p.Val2555Met LP/P rs746195428 Short-rib thoracic dysplasia 3 with or without polydactyly (SRTD3) [MIM:613091]
DYNC2H1 Q8NCM8 VAR_069608 p.Tyr2573Cys LP/P rs1278825521 Short-rib thoracic dysplasia 3 with or without polydactyly (SRTD3) [MIM:613091]
DYNC2H1 Q8NCM8 VAR_069609 p.Ile2640Thr LP/P rs1265669915 Short-rib thoracic dysplasia 3 with or without polydactyly (SRTD3) [MIM:613091]
DYNC2H1 Q8NCM8 VAR_069610 p.Arg2662Gln LP/P rs397514635 Short-rib thoracic dysplasia 3 with or without polydactyly (SRTD3) [MIM:613091]
DYNC2H1 Q8NCM8 VAR_069611 p.Ile2819Met LP/P rs1060501431 Short-rib thoracic dysplasia 3 with or without polydactyly (SRTD3) [MIM:613091]
DYNC2H1 Q8NCM8 VAR_069612 p.Pro3381Leu LP/P rs368631447 Short-rib thoracic dysplasia 3 with or without polydactyly (SRTD3) [MIM:613091]
DYNC2H1 Q8NCM8 VAR_069613 p.Arg3806Cys LP/P rs754753584 Short-rib thoracic dysplasia 3 with or without polydactyly (SRTD3) [MIM:613091]
DYNC2H1 Q8NCM8 VAR_069614 p.Trp3847Gly LP/P rs752554582 Short-rib thoracic dysplasia 3 with or without polydactyly (SRTD3) [MIM:613091]
DYNC2H1 Q8NCM8 VAR_069615 p.Gly3909Asp LP/P rs201479015 -
DYNC2H1 Q8NCM8 VAR_069616 p.Leu4232Arg LP/P - Short-rib thoracic dysplasia 3 with or without polydactyly (SRTD3) [MIM:613091]
DYNC2I1 Q8WVS4 VAR_026841 p.Glu91Gly LB/B rs17837851 -
DYNC2I1 Q8WVS4 VAR_026842 p.Gln273Arg LB/B rs2788478 -
DYNC2I1 Q8WVS4 VAR_070197 p.Thr749Met LP/P rs587777065 Short-rib thoracic dysplasia 8 with or without polydactyly (SRTD8) [MIM:615503]
DYNC2I2 Q96EX3 VAR_070962 p.Ala22Val LB/B rs201715229 -

DYNC2I2 Q96EX3 VAR_070963 p.Cys148Phe LP/P - Short-rib thoracic dysplasia 11 with or without polydactyly (SRTD11) [MIM:615633]
DYNC2I2 Q96EX3 VAR_070964 p.Arg206Cys LB/B rs148543026 -
DYNC2I2 Q96EX3 VAR_070965 p.Ala341Val LP/P rs587777091 Short-rib thoracic dysplasia 11 with or without polydactyly (SRTD11) [MIM:615633]
DYNC2I2 Q96EX3 VAR_070966 p.Thr354Met LP/P rs587777092 Short-rib thoracic dysplasia 11 with or without polydactyly (SRTD11) [MIM:615633]
DYNC2I2 Q96EX3 VAR_070967 p.Pro390Leu LP/P - Short-rib thoracic dysplasia 11 with or without polydactyly (SRTD11) [MIM:615633]
DYNC2I2 Q96EX3 VAR_070968 p.Gly393Ser LP/P rs587777096 Short-rib thoracic dysplasia 11 with or without polydactyly (SRTD11) [MIM:615633]
DYNC2I2 Q96EX3 VAR_070969 p.Ser410Ile LP/P - Short-rib thoracic dysplasia 11 with or without polydactyly (SRTD11) [MIM:615633]
DYNC2I2 Q96EX3 VAR_070970 p.Lys436Arg LP/P rs587777098 Short-rib thoracic dysplasia 11 with or without polydactyly (SRTD11) [MIM:615633]
DYNC2I2 Q96EX3 VAR_070971 p.Arg447Gln LP/P rs587777094 Short-rib thoracic dysplasia 11 with or without polydactyly (SRTD11) [MIM:615633]
DYNC2I2 Q96EX3 VAR_070972 p.Arg447Trp LP/P rs587777093 Short-rib thoracic dysplasia 11 with or without polydactyly (SRTD11) [MIM:615633]
DYNC2I2 Q96EX3 VAR_083840 p.Arg182Trp LP/P - Short-rib thoracic dysplasia 11 with or without polydactyly (SRTD11) [MIM:615633]
DYNC2LI1 Q8TCX1 VAR_038874 p.Phe33Ser LB/B rs2288709 -
DYNC2LI1 Q8TCX1 VAR_038875 p.Pro58Ser LB/B rs17854966 -
DYNC2LI1 Q8TCX1 VAR_038876 p.Ile230Leu LB/B rs11556157 -
DYNC2LI1 Q8TCX1 VAR_077814 p.Leu117Val LP/P rs201948500 Short-rib thoracic dysplasia 15 with polydactyly (SRTD15) [MIM:617088]
DYNC2LI1 Q8TCX1 VAR_077815 p.Thr220Ile LP/P rs886037860 Short-rib thoracic dysplasia 15 with polydactyly (SRTD15) [MIM:617088]
DYNLRB1 Q9NP97 VAR_049124 p.Ser13Arg LB/B rs1063616 -
DYNLRB1 Q9NP97 VAR_049125 p.Ile71Phe LB/B rs10036 -
DYNLRB2 Q8TF09 VAR_049126 p.His14Arg LB/B rs13332289 -
DYNLT2 Q8IZS6 VAR_042499 p.Ile67Val LB/B rs116980543 -
DYNLT2 Q8IZS6 VAR_042500 p.Arg88Ile LB/B rs2027063 -
DYNLT2 Q8IZS6 VAR_042501 p.Val121Ala LB/B rs13194101 -
DYNLT5 Q8N7M0 VAR_031852 p.Glu49Asp LB/B rs1060575 -
DYNLT5 Q8N7M0 VAR_055700 p.Leu143Ile LB/B rs2133173 -
DYRK1A Q13627 VAR_009395 p.Tyr415Phe LB/B - -
DYRK1A Q13627 VAR_009396 p.Gln681His LB/B - -
DYRK1A Q13627 VAR_040453 p.Ala679Pro LB/B rs55720916 -
DYRK1B Q9Y463 VAR_040454 p.Leu28Pro LB/B rs34587974 -
DYRK1B Q9Y463 VAR_040455 p.Arg102His LB/B rs55687541 -
DYRK1B Q9Y463 VAR_040456 p.Ser234Gly LB/B rs35858874 -
DYRK1B Q9Y463 VAR_040457 p.Gln275Arg US - A metastatic melanoma sample
DYRK1B Q9Y463 VAR_071773 p.His90Pro LP/P rs587777380 Abdominal obesity-metabolic syndrome 3 (AOMS3) [MIM:615812]
DYRK1B Q9Y463 VAR_071774 p.Arg102Cys LP/P rs367643250 Abdominal obesity-metabolic syndrome 3 (AOMS3) [MIM:615812]
DYRK2 Q92630 VAR_040458 p.Ser98Gly LB/B rs35139851 -
DYRK2 Q92630 VAR_040459 p.Pro198Leu US rs1384093596 A glioblastoma multiforme sample

DYRK2 Q92630 VAR_040460 p.His245Asn LB/B rs34166200 -
DYRK2 Q92630 VAR_040461 p.Asn295Ser LB/B rs56293072 -
DYRK2 Q92630 VAR_040462 p.Arg451Gln LB/B rs35688869 -
DYRK2 Q92630 VAR_040463 p.Phe455Tyr LB/B rs55774594 -
DYRK3 O43781 VAR_040464 p.Met239Leu LB/B - -
DYRK4 Q9NR20 VAR_010721 p.Val95Ile LB/B rs746486416 -
DYRK4 Q9NR20 VAR_010722 p.Asn189Ser LB/B rs3741927 -
DYRK4 Q9NR20 VAR_014948 p.Asp454Val LB/B rs1801016 -
DYRK4 Q9NR20 VAR_033900 p.Ala61Thr LB/B rs12306130 -
DYRK4 Q9NR20 VAR_040465 p.Ala70Ser LB/B - -
DYSF O75923 VAR_012308 p.Pro791Arg LP/P rs121908956 Miyoshi muscular dystrophy 1 (MMD1) [MIM:254130]
DYSF O75923 VAR_012308 p.Pro791Arg LP/P rs121908956 Muscular dystrophy, limb-girdle, autosomal recessive 2 (LGMDR2) [MIM:253601]
DYSF O75923 VAR_012309 p.Ile1298Val LP/P rs121908954 Miyoshi muscular dystrophy 1 (MMD1) [MIM:254130]
DYSF O75923 VAR_012309 p.Ile1298Val LP/P rs121908954 Muscular dystrophy, limb-girdle, autosomal recessive 2 (LGMDR2) [MIM:253601]
DYSF O75923 VAR_012310 p.His1857Arg LP/P rs199601326 Miyoshi muscular dystrophy 1 (MMD1) [MIM:254130]
DYSF O75923 VAR_012311 p.Arg2042Cys LP/P rs121908955 Miyoshi muscular dystrophy 1 (MMD1) [MIM:254130]
DYSF O75923 VAR_012311 p.Arg2042Cys LP/P rs121908955 Muscular dystrophy, limb-girdle, autosomal recessive 2 (LGMDR2) [MIM:253601]
DYSF O75923 VAR_020308 p.Arg1242His LB/B rs2303603 -
DYSF O75923 VAR_024853 p.Ala170Glu LB/B rs34999029 -
DYSF O75923 VAR_024854 p.Leu189Val LB/B rs13407355 -
DYSF O75923 VAR_024855 p.Arg253Trp LB/B rs149827237 -
DYSF O75923 VAR_024856 p.Leu266Pro US - -
DYSF O75923 VAR_024857 p.Gly299Glu LP/P rs1258728780 Miyoshi muscular dystrophy 1 (MMD1) [MIM:254130]
DYSF O75923 VAR_024858 p.Cys456Trp LP/P - Miyoshi muscular dystrophy 1 (MMD1) [MIM:254130]
DYSF O75923 VAR_024859 p.Arg555Trp LP/P rs377735262 Miyoshi muscular dystrophy 1 (MMD1) [MIM:254130]
DYSF O75923 VAR_024859 p.Arg555Trp LP/P rs377735262 Muscular dystrophy, limb-girdle, autosomal recessive 2 (LGMDR2) [MIM:253601]
DYSF O75923 VAR_024860 p.Arg959Trp LP/P rs202218890 Miyoshi muscular dystrophy 1 (MMD1) [MIM:254130]
DYSF O75923 VAR_024860 p.Arg959Trp LP/P rs202218890 Muscular dystrophy, limb-girdle, autosomal recessive 2 (LGMDR2) [MIM:253601]
DYSF O75923 VAR_024861 p.Arg1022Gln LB/B rs34211915 -
DYSF O75923 VAR_024862 p.Arg1038Gln LP/P rs150877497 Muscular dystrophy, limb-girdle, autosomal recessive 2 (LGMDR2) [MIM:253601]
DYSF O75923 VAR_024863 p.Arg1046His LP/P rs121908958 Miyoshi muscular dystrophy 1 (MMD1) [MIM:254130]
DYSF O75923 VAR_024865 p.Ile1208Met US rs148858485 Muscular dystrophy, limb-girdle, autosomal recessive 2 (LGMDR2) [MIM:253601]
DYSF O75923 VAR_024866 p.Leu1276Val US - -
DYSF O75923 VAR_024867 p.Arg1331Leu LB/B rs61742872 -

DYSF O75923 VAR_024868 p.Glu1335Lys LP/P rs758993965 Miyoshi muscular dystrophy 1 (MMD1) [MIM:254130]
DYSF O75923 VAR_024868 p.Glu1335Lys LP/P rs758993965 Muscular dystrophy, limb-girdle, autosomal recessive 2 (LGMDR2) [MIM:253601]
DYSF O75923 VAR_024869 p.Asn1351Ser LB/B rs139529811 -
DYSF O75923 VAR_024870 p.Arg1693Gln LP/P rs779987458 Miyoshi muscular dystrophy 1 (MMD1) [MIM:254130]
DYSF O75923 VAR_024871 p.Glu1748Val US - -
DYSF O75923 VAR_024872 p.Arg2000Gln LP/P rs115407852 Miyoshi muscular dystrophy 1 (MMD1) [MIM:254130]
DYSF O75923 VAR_035893 p.Ile1325Met US - A breast cancer sample
DYSF O75923 VAR_035894 p.Leu1349Val US - A breast cancer sample
DYSF O75923 VAR_049055 p.Ile834Val LB/B rs34671418 -
DYSF O75923 VAR_049056 p.Ala1072Pro LB/B rs34660230 -
DYSF O75923 VAR_057834 p.Trp52Arg LP/P rs1553508863 Muscular dystrophy, limb-girdle, autosomal recessive 2 (LGMDR2) [MIM:253601]
DYSF O75923 VAR_057835 p.Val67Asp LP/P rs121908957 Miyoshi muscular dystrophy 1 (MMD1) [MIM:254130]
DYSF O75923 VAR_057835 p.Val67Asp LP/P rs121908957 Muscular dystrophy, limb-girdle, autosomal recessive 2 (LGMDR2) [MIM:253601]
DYSF O75923 VAR_057836 p.Ala84Val LB/B rs772008300 -
DYSF O75923 VAR_057837 p.Gly155Arg LP/P rs200970855 Muscular dystrophy, limb-girdle, autosomal recessive 2 (LGMDR2) [MIM:253601]
DYSF O75923 VAR_057838 p.Gly234Glu LP/P rs141497053 Muscular dystrophy, limb-girdle, autosomal recessive 2 (LGMDR2) [MIM:253601]
DYSF O75923 VAR_057839 p.Ile284Thr LP/P rs1553522164 Muscular dystrophy, limb-girdle, autosomal recessive 2 (LGMDR2) [MIM:253601]
DYSF O75923 VAR_057840 p.Gly299Arg LP/P rs121908963 Muscular dystrophy, limb-girdle, autosomal recessive 2 (LGMDR2) [MIM:253601]
DYSF O75923 VAR_057841 p.Gly299Trp LP/P rs121908963 Miyoshi muscular dystrophy 1 (MMD1) [MIM:254130]
DYSF O75923 VAR_057842 p.Gly335Ala LB/B - -
DYSF O75923 VAR_057843 p.Ser340Arg LB/B rs766891289 -
DYSF O75923 VAR_057844 p.Val374Leu LB/B rs150724610 -
DYSF O75923 VAR_057846 p.Glu389Gln LP/P - Miyoshi muscular dystrophy 1 (MMD1) [MIM:254130]
DYSF O75923 VAR_057847 p.Asp390Asn LB/B rs886042389 -
DYSF O75923 VAR_057848 p.Gly426Arg LP/P rs886042093 Miyoshi muscular dystrophy 1 (MMD1) [MIM:254130]
DYSF O75923 VAR_057849 p.Gly426Val LP/P - Miyoshi muscular dystrophy 1 (MMD1) [MIM:254130]
DYSF O75923 VAR_057850 p.Gly519Arg LP/P rs121908962 Miyoshi muscular dystrophy 1 (MMD1) [MIM:254130]
DYSF O75923 VAR_057851 p.Gly618Arg LP/P rs201049092 Miyoshi muscular dystrophy 1 (MMD1) [MIM:254130]
DYSF O75923 VAR_057851 p.Gly618Arg LP/P rs201049092 Muscular dystrophy, limb-girdle, autosomal recessive 2 (LGMDR2) [MIM:253601]
DYSF O75923 VAR_057852 p.Gly621Arg LP/P rs886043900 Muscular dystrophy, limb-girdle, autosomal recessive 2 (LGMDR2) [MIM:253601]
DYSF O75923 VAR_057853 p.Asp625Tyr LP/P rs121908960 Muscular dystrophy, limb-girdle, autosomal recessive 2 (LGMDR2) [MIM:253601]

DYSF O75923 VAR_057854 p.Pro731Arg LP/P - Muscular dystrophy, limb-girdle, autosomal recessive 2 (LGMDR2) [MIM:253601]
DYSF O75923 VAR_057855 p.Arg819Gln LB/B rs748636047 -
DYSF O75923 VAR_057856 p.Trp930Cys US rs727503910 Muscular dystrophy, limb-girdle, autosomal recessive 2 (LGMDR2) [MIM:253601]
DYSF O75923 VAR_057857 p.Trp999Cys LP/P rs28937581 Miyoshi muscular dystrophy 1 (MMD1) [MIM:254130]
DYSF O75923 VAR_057858 p.Pro1029Leu LP/P - Miyoshi muscular dystrophy 1 (MMD1) [MIM:254130]
DYSF O75923 VAR_057859 p.Arg1041Cys LP/P rs144598063 Miyoshi muscular dystrophy 1 (MMD1) [MIM:254130]
DYSF O75923 VAR_057860 p.Leu1228Pro LP/P - Muscular dystrophy, limb-girdle, autosomal recessive 2 (LGMDR2) [MIM:253601]
DYSF O75923 VAR_057861 p.Ile1325Val LB/B rs145401010 -
DYSF O75923 VAR_057862 p.Leu1341Pro LP/P rs757917335 Muscular dystrophy, limb-girdle, autosomal recessive 2 (LGMDR2) [MIM:253601]
DYSF O75923 VAR_057863 p.Cys1361Arg LP/P rs776472879 Miyoshi muscular dystrophy 1 (MMD1) [MIM:254130]
DYSF O75923 VAR_057864 p.Tyr1505Cys LP/P rs757820496 Muscular dystrophy, limb-girdle, autosomal recessive 2 (LGMDR2) [MIM:253601]
DYSF O75923 VAR_057865 p.Lys1526Thr LP/P rs76086153 Muscular dystrophy, limb-girdle, autosomal recessive 2 (LGMDR2) [MIM:253601]
DYSF O75923 VAR_057866 p.Gly1543Asp LP/P - Muscular dystrophy, limb-girdle, autosomal recessive 2 (LGMDR2) [MIM:253601]
DYSF O75923 VAR_057867 p.Arg1581His LB/B rs185596534 -
DYSF O75923 VAR_057868 p.Thr1662Arg LP/P - Miyoshi muscular dystrophy 1 (MMD1) [MIM:254130]
DYSF O75923 VAR_057869 p.Cys1678Ser LB/B rs753279446 -
DYSF O75923 VAR_057870 p.Gly1679Glu LP/P - Miyoshi muscular dystrophy 1 (MMD1) [MIM:254130]
DYSF O75923 VAR_057871 p.Arg1693Trp LP/P rs863225021 Miyoshi muscular dystrophy 1 (MMD1) [MIM:254130]
DYSF O75923 VAR_057872 p.Glu1734Gly LP/P rs121908961 Muscular dystrophy, limb-girdle, autosomal recessive 2 (LGMDR2) [MIM:253601]
DYSF O75923 VAR_057873 p.Arg1768Trp US rs746243052 Muscular dystrophy, limb-girdle, autosomal recessive 2 (LGMDR2) [MIM:253601]
DYSF O75923 VAR_057874 p.Asp1837Asn LP/P rs398123794 Miyoshi muscular dystrophy 1 (MMD1) [MIM:254130]
DYSF O75923 VAR_057875 p.Gly1842Asp LP/P rs1131692158 Miyoshi muscular dystrophy 1 (MMD1) [MIM:254130]
DYSF O75923 VAR_057876 p.Leu1922Pro LP/P - Miyoshi muscular dystrophy 1 (MMD1) [MIM:254130]
DYSF O75923 VAR_057878 p.Cys1942Gly LP/P - Miyoshi muscular dystrophy 1 (MMD1) [MIM:254130]
DYSF O75923 VAR_057879 p.Gly1967Ser LB/B - -
DYSF O75923 VAR_057880 p.Pro1970Ser LP/P rs1057521141 Muscular dystrophy, limb-girdle, autosomal recessive 2 (LGMDR2) [MIM:253601]
DYSF O75923 VAR_057881 p.Pro2068Leu LP/P rs149732545 Miyoshi muscular dystrophy 1 (MMD1) [MIM:254130]
DYSF O75923 VAR_061170 p.Arg1096His LB/B rs59915619 -
DYTN A2CJ06 VAR_050960 p.Tyr241Cys LB/B rs16838593 -
DYTN A2CJ06 VAR_050961 p.Gln474Lys LB/B rs2115591 -
DZIP1 Q86YF9 VAR_019456 p.Pro736Ser LB/B rs11070136 -
DZIP1 Q86YF9 VAR_052710 p.Thr172Met LB/B rs9561921 -

DZIP1 Q86YF9 VAR_052711 p.Met664Leu LB/B rs34303958 -
DZIP1L Q8IYY4 VAR_042756 p.Arg321Trp LB/B rs2724693 -
DZIP1L Q8IYY4 VAR_042757 p.Thr545Ala LB/B rs446644 -
DZIP1L Q8IYY4 VAR_042758 p.Ala551Val LB/B rs11917468 -
DZIP1L Q8IYY4 VAR_042759 p.Arg593His LB/B rs374045 -
DZIP1L Q8IYY4 VAR_042760 p.Lys645Glu LB/B rs442800 -
DZIP1L Q8IYY4 VAR_078962 p.Ala90Val LP/P rs555349004 Polycystic kidney disease 5 (PKD5) [MIM:617610]
DZIP1L Q8IYY4 VAR_078963 p.Gln91His LP/P rs1135402754 Polycystic kidney disease 5 (PKD5) [MIM:617610]
E2F1 Q01094 VAR_013607 p.Arg252His LB/B rs3213172 -
E2F1 Q01094 VAR_013608 p.Val276Met LB/B rs3213173 -
E2F1 Q01094 VAR_013609 p.Thr311Asn LB/B rs3213174 -
E2F1 Q01094 VAR_013610 p.Gly393Ser LB/B rs3213176 -
E2F1 Q01094 VAR_048907 p.Gly200Ser LB/B rs35385772 -
E2F2 Q14209 VAR_018990 p.Gly205Arg LB/B rs2229297 -
E2F2 Q14209 VAR_018991 p.Gln226His LB/B rs2075995 -
E2F3 O00716 VAR_014341 p.Gly344Arg LB/B rs4134973 -
E2F3 O00716 VAR_014342 p.Asp389Asn LB/B rs4134982 -
E2F4 Q16254 VAR_014936 p.Thr293Pro LB/B rs1801013 -
E2F5 Q15329 VAR_014348 p.Gly18Ala LB/B rs4150841 -
E2F7 Q96AV8 VAR_034732 p.Phe72Leu LB/B rs310791 -
E2F7 Q96AV8 VAR_034733 p.Met626Val LB/B rs3829295 -
E2F7 Q96AV8 VAR_034734 p.His854Gln LB/B rs310831 -
E2F8 A0AVK6 VAR_034735 p.Ile674Val LB/B rs793274 -
E4F1 Q66K89 VAR_060270 p.Arg167His LB/B rs26839 -
E4F1 Q66K89 VAR_060271 p.Val355Ile LB/B rs59784157 -
EAPP Q56P03 VAR_031915 p.Gln168Glu LB/B rs17352411 -
EARS2 Q5JPH6 VAR_028840 p.Ser457Gly LB/B rs6497671 -
EARS2 Q5JPH6 VAR_069235 p.Arg55His LP/P rs770862902 Combined oxidative phosphorylation deficiency 12 (COXPD12) [MIM:614924]
EARS2 Q5JPH6 VAR_069236 p.Lys65Glu LP/P rs397514595 Combined oxidative phosphorylation deficiency 12 (COXPD12) [MIM:614924]
EARS2 Q5JPH6 VAR_069237 p.Glu96Lys LP/P rs397514593 Combined oxidative phosphorylation deficiency 12 (COXPD12) [MIM:614924]
EARS2 Q5JPH6 VAR_069238 p.Arg107His LP/P rs1021330566 Combined oxidative phosphorylation deficiency 12 (COXPD12) [MIM:614924]
EARS2 Q5JPH6 VAR_069239 p.Arg108Trp LP/P rs376103091 Combined oxidative phosphorylation deficiency 12 (COXPD12) [MIM:614924]
EARS2 Q5JPH6 VAR_069240 p.Gly110Ser LP/P rs201842633 Combined oxidative phosphorylation deficiency 12 (COXPD12) [MIM:614924]
EARS2 Q5JPH6 VAR_069241 p.Cys167Tyr LP/P rs397514594 Combined oxidative phosphorylation deficiency 12 (COXPD12) [MIM:614924]
EARS2 Q5JPH6 VAR_069242 p.Arg168Gly LP/P rs397514591 Combined oxidative phosphorylation deficiency 12 (COXPD12) [MIM:614924]
EARS2 Q5JPH6 VAR_069243 p.Gly204Ser LP/P rs397514592 Combined oxidative phosphorylation deficiency 12 (COXPD12) [MIM:614924]
EARS2 Q5JPH6 VAR_069244 p.Gly224Ser LP/P rs141129877 Combined oxidative phosphorylation deficiency 12 (COXPD12) [MIM:614924]
EARS2 Q5JPH6 VAR_069245 p.Gly317Cys LP/P rs746838793 Combined oxidative phosphorylation deficiency 12 (COXPD12) [MIM:614924]

EARS2 Q5JPH6 VAR_069247 p.Arg516Gln LP/P rs201727231 Combined oxidative phosphorylation deficiency 12 (COXPD12) [MIM:614924]
EARS2 Q5JPH6 VAR_076183 p.Arg107Cys LP/P rs1355685453 Combined oxidative phosphorylation deficiency 12 (COXPD12) [MIM:614924]
EARS2 Q5JPH6 VAR_076184 p.Arg489Gln LP/P rs757965573 Combined oxidative phosphorylation deficiency 12 (COXPD12) [MIM:614924]
EBF2 Q9HAK2 VAR_048754 p.Gly559Ser LB/B rs17054477 -
EBF3 Q9H4W6 VAR_078033 p.Asn66Asp LP/P rs1057519518 Hypotonia, ataxia, and delayed development syndrome (HADDS) [MIM:617330]
EBF3 Q9H4W6 VAR_078034 p.Tyr141Cys LP/P rs1057519519 Hypotonia, ataxia, and delayed development syndrome (HADDS) [MIM:617330]
EBF3 Q9H4W6 VAR_078036 p.Arg163Leu LP/P rs1057519389 Hypotonia, ataxia, and delayed development syndrome (HADDS) [MIM:617330]
EBF3 Q9H4W6 VAR_078037 p.Arg163Pro LP/P rs1057519389 Hypotonia, ataxia, and delayed development syndrome (HADDS) [MIM:617330]
EBF3 Q9H4W6 VAR_078038 p.Arg163Gln LP/P rs1057519389 Hypotonia, ataxia, and delayed development syndrome (HADDS) [MIM:617330]
EBF3 Q9H4W6 VAR_078039 p.Gly171Asp LP/P rs1057519437 Hypotonia, ataxia, and delayed development syndrome (HADDS) [MIM:617330]
EBF3 Q9H4W6 VAR_078040 p.Pro177Leu LP/P rs869312668 Hypotonia, ataxia, and delayed development syndrome (HADDS) [MIM:617330]
EBF3 Q9H4W6 VAR_078041 p.Lys193Asn LP/P rs1057519520 Hypotonia, ataxia, and delayed development syndrome (HADDS) [MIM:617330]
EBF3 Q9H4W6 VAR_078042 p.Arg209Trp LP/P rs779003155 Hypotonia, ataxia, and delayed development syndrome (HADDS) [MIM:617330]
EBI3 Q14213 VAR_024342 p.Val201Ile LB/B rs4740 -
EBI3 Q14213 VAR_049171 p.Ala174Val LB/B rs1803524 -
EBNA1BP2 Q99848 VAR_024437 p.Arg223His LB/B rs7163 -
EBP Q15125 VAR_012105 p.Glu80Lys LP/P rs104894800 Chondrodysplasia punctata 2, X-linked dominant (CDPX2) [MIM:302960]
EBP Q15125 VAR_012106 p.Arg110Gln LP/P - Chondrodysplasia punctata 2, X-linked dominant (CDPX2) [MIM:302960]
EBP Q15125 VAR_012107 p.Arg147Gly LP/P - Chondrodysplasia punctata 2, X-linked dominant (CDPX2) [MIM:302960]
EBP Q15125 VAR_012108 p.Arg147His LP/P rs28935174 Chondrodysplasia punctata 2, X-linked dominant (CDPX2) [MIM:302960]
EBP Q15125 VAR_074633 p.Leu18Pro LP/P rs104894795 MEND syndrome (MEND) [MIM:300960]
EBP Q15125 VAR_074634 p.Trp47Cys LP/P rs587783599 MEND syndrome (MEND) [MIM:300960]
EBP Q15125 VAR_074635 p.Trp47Arg LP/P rs878854359 MEND syndrome (MEND) [MIM:300960]
EBP Q15125 VAR_074636 p.Ile75Asn LP/P rs797045153 MEND syndrome (MEND) [MIM:300960]
EBP Q15125 VAR_074637 p.Glu103Lys LP/P - Chondrodysplasia punctata 2, X-linked dominant (CDPX2) [MIM:302960]
ECD O95905 VAR_012191 p.Arg281Gly LB/B rs151023501 -
ECD O95905 VAR_051970 p.Arg45Gln LB/B rs3812619 -
ECD O95905 VAR_051971 p.Glu452Gln LB/B rs3736518 -
ECD O95905 VAR_051972 p.Asn501Ser LB/B rs36152134 -
ECD O95905 VAR_051973 p.Asp634Gly LB/B rs2271904 -
ECE1 P42892 VAR_011972 p.Thr341Ile LB/B rs1076669 -

ECE1 P42892 VAR_026747 p.Arg754Cys LP/P rs3026906 Hirschsprung disease, cardiac defects, and autonomic dysfunction (HCAD) [MIM:613870]
ECE2 P0DPD6 VAR_037085 p.Arg499Gln LB/B rs35875049 -
ECEL1 O95672 VAR_012813 p.His10Gln LB/B rs2741281 -
ECEL1 O95672 VAR_012814 p.His328Tyr LB/B rs1529874 -
ECEL1 O95672 VAR_069747 p.Arg418Ser LP/P rs587776919 Arthrogryposis, distal, 5D (DA5D) [MIM:615065]
ECEL1 O95672 VAR_069993 p.Arg404Cys LP/P rs532757890 Arthrogryposis, distal, 5D (DA5D) [MIM:615065]
ECEL1 O95672 VAR_069994 p.Gly607Ser LP/P - Arthrogryposis, distal, 5D (DA5D) [MIM:615065]
ECEL1 O95672 VAR_069995 p.Cys760Arg LP/P rs587777129 Arthrogryposis, distal, 5D (DA5D) [MIM:615065]
ECH1 Q13011 VAR_014927 p.Glu41Ala LB/B rs9419 -
ECH1 Q13011 VAR_033913 p.Gly217Arg LB/B rs2229259 -
ECHDC2 Q86YB7 VAR_036951 p.Asn119Asp LB/B rs17854314 -
ECHDC3 Q96DC8 VAR_043126 p.Ala69Thr LB/B rs4750090 -
ECHDC3 Q96DC8 VAR_043127 p.Ala151Thr LB/B rs7910140 -
ECHDC3 Q96DC8 VAR_043128 p.Asp162Asn LB/B rs35986488 -
ECHS1 P30084 VAR_022273 p.Val11Ala LB/B rs10466126 -
ECHS1 P30084 VAR_022274 p.Thr75Ile LB/B rs1049951 -
ECHS1 P30084 VAR_073373 p.Ala2Val LP/P rs587776498 Mitochondrial short-chain enoyl-CoA hydratase 1 deficiency (ECHS1D) [MIM:616277]
ECHS1 P30084 VAR_073374 p.Ala158Asp LP/P rs786204001 Mitochondrial short-chain enoyl-CoA hydratase 1 deficiency (ECHS1D) [MIM:616277]
ECHS1 P30084 VAR_076185 p.Phe33Ser LP/P - Mitochondrial short-chain enoyl-CoA hydratase 1 deficiency (ECHS1D) [MIM:616277]
ECHS1 P30084 VAR_076186 p.Arg54His LP/P rs375266808 Mitochondrial short-chain enoyl-CoA hydratase 1 deficiency (ECHS1D) [MIM:616277]
ECHS1 P30084 VAR_076187 p.Asn59Ser LP/P rs201865375 Mitochondrial short-chain enoyl-CoA hydratase 1 deficiency (ECHS1D) [MIM:616277]
ECHS1 P30084 VAR_076188 p.Ile66Thr LP/P rs371063211 Mitochondrial short-chain enoyl-CoA hydratase 1 deficiency (ECHS1D) [MIM:616277]
ECHS1 P30084 VAR_076189 p.Glu77Gln LP/P rs1426014295 Mitochondrial short-chain enoyl-CoA hydratase 1 deficiency (ECHS1D) [MIM:616277]
ECHS1 P30084 VAR_076190 p.Gly90Arg US rs1085307550 Mitochondrial short-chain enoyl-CoA hydratase 1 deficiency (ECHS1D) [MIM:616277]
ECHS1 P30084 VAR_076191 p.Ala132Thr LP/P rs770931871 Mitochondrial short-chain enoyl-CoA hydratase 1 deficiency (ECHS1D) [MIM:616277]
ECHS1 P30084 VAR_076192 p.Asp150Gly LP/P - Mitochondrial short-chain enoyl-CoA hydratase 1 deficiency (ECHS1D) [MIM:616277]
ECHS1 P30084 VAR_076193 p.Gln159Arg LP/P rs375032130 Mitochondrial short-chain enoyl-CoA hydratase 1 deficiency (ECHS1D) [MIM:616277]
ECHS1 P30084 VAR_076194 p.Gly195Ser LP/P rs761989177 Mitochondrial short-chain enoyl-CoA hydratase 1 deficiency (ECHS1D) [MIM:616277]
ECHS1 P30084 VAR_076195 p.Cys225Arg LP/P rs769429279 Mitochondrial short-chain enoyl-CoA hydratase 1 deficiency (ECHS1D) [MIM:616277]
ECHS1 P30084 VAR_076196 p.Lys273Glu LP/P rs565090080 Mitochondrial short-chain enoyl-CoA hydratase 1 deficiency (ECHS1D) [MIM:616277]
ECHS1 P30084 VAR_076479 p.Ala138Val LP/P rs864309656 Mitochondrial short-chain enoyl-CoA hydratase 1 deficiency (ECHS1D) [MIM:616277]
ECHS1 P30084 VAR_076480 p.Glu281Gly US - Mitochondrial short-chain enoyl-CoA hydratase 1 deficiency (ECHS1D) [MIM:616277]

ECI2 O75521 VAR_058493 p.Met47Ile LB/B rs3177253 -
ECI2 O75521 VAR_058494 p.Ala344Val LB/B rs7166 -
ECM1 Q16610 VAR_014761 p.Gly415Ser LB/B rs13294 -
ECM1 Q16610 VAR_014762 p.Gly528Arg LB/B rs1050901 -
ECM1 Q16610 VAR_014763 p.Ser535Phe LB/B rs1050904 -
ECM1 Q16610 VAR_018690 p.Thr130Met LB/B rs3737240 -
ECM1 Q16610 VAR_018691 p.Phe167Ile LP/P rs121909116 Lipoid proteinosis (LiP) [MIM:247100]
ECM2 O94769 VAR_024646 p.Gln56Pro LB/B rs10120210 -
ECM2 O94769 VAR_052010 p.Thr109Ser LB/B rs35496743 -
ECM2 O94769 VAR_052011 p.Arg204Gln LB/B rs34758505 -
ECPAS Q5VYK3 VAR_055702 p.Thr472Ser LB/B rs16916091 -
ECRG4 Q9H1Z8 VAR_050962 p.Ala52Thr LB/B rs10187689 -
ECSIT Q9BQ95 VAR_032907 p.Arg278Cys LB/B rs34803265 -
ECSIT Q9BQ95 VAR_032908 p.Gly406Arg LB/B rs2302971 -
ECT2 Q9H8V3 VAR_035975 p.Thr833Pro US - A breast cancer sample
ECT2 Q9H8V3 VAR_047064 p.Ser15Thr LB/B rs34703432 -
ECT2L Q008S8 VAR_043482 p.Glu527Lys LB/B rs1529151 -
ECT2L Q008S8 VAR_043483 p.Ala594Thr LB/B rs11968285 -
ECT2L Q008S8 VAR_051984 p.Gln483His LB/B rs12198781 -
EDA Q92838 VAR_005179 p.Tyr61His LP/P rs132630308 Ectodermal dysplasia 1, hypohidrotic, X-linked (XHED) [MIM:305100]
EDA Q92838 VAR_005180 p.Glu63Lys LP/P rs132630311 Ectodermal dysplasia 1, hypohidrotic, X-linked (XHED) [MIM:305100]
EDA Q92838 VAR_005181 p.Arg69Leu LP/P rs132630309 Ectodermal dysplasia 1, hypohidrotic, X-linked (XHED) [MIM:305100]
EDA Q92838 VAR_005182 p.Arg155Cys LP/P rs132630312 Ectodermal dysplasia 1, hypohidrotic, X-linked (XHED) [MIM:305100]
EDA Q92838 VAR_005183 p.Arg156Cys LP/P rs132630313 Ectodermal dysplasia 1, hypohidrotic, X-linked (XHED) [MIM:305100]
EDA Q92838 VAR_005184 p.Arg156His LP/P rs132630314 Ectodermal dysplasia 1, hypohidrotic, X-linked (XHED) [MIM:305100]
EDA Q92838 VAR_005185 p.Pro209Leu LP/P rs132630315 Ectodermal dysplasia 1, hypohidrotic, X-linked (XHED) [MIM:305100]
EDA Q92838 VAR_005186 p.Gly224Ala LP/P rs132630316 Ectodermal dysplasia 1, hypohidrotic, X-linked (XHED) [MIM:305100]
EDA Q92838 VAR_005187 p.His252Leu LP/P rs879255552 Ectodermal dysplasia 1, hypohidrotic, X-linked (XHED) [MIM:305100]
EDA Q92838 VAR_005188 p.Gly299Ser LP/P rs397516679 Ectodermal dysplasia 1, hypohidrotic, X-linked (XHED) [MIM:305100]
EDA Q92838 VAR_005189 p.Ala349Thr LP/P rs132630317 Ectodermal dysplasia 1, hypohidrotic, X-linked (XHED) [MIM:305100]
EDA Q92838 VAR_005190 p.Ala356Asp LP/P - Ectodermal dysplasia 1, hypohidrotic, X-linked (XHED) [MIM:305100]
EDA Q92838 VAR_005191 p.Arg357Pro LP/P rs61747506 Ectodermal dysplasia 1, hypohidrotic, X-linked (XHED) [MIM:305100]
EDA Q92838 VAR_010611 p.His54Tyr LP/P - Ectodermal dysplasia 1, hypohidrotic, X-linked (XHED) [MIM:305100]
EDA Q92838 VAR_010612 p.Leu55Arg LP/P - Ectodermal dysplasia 1, hypohidrotic, X-linked (XHED) [MIM:305100]

EDA Q92838 VAR_010613 p.Gly291Arg LP/P rs397516677 Ectodermal dysplasia 1, hypohidrotic, X-linked (XHED) [MIM:305100]
EDA Q92838 VAR_010614 p.Gly291Trp LP/P - Ectodermal dysplasia 1, hypohidrotic, X-linked (XHED) [MIM:305100]
EDA Q92838 VAR_010615 p.Asp298His LP/P - Ectodermal dysplasia 1, hypohidrotic, X-linked (XHED) [MIM:305100]
EDA Q92838 VAR_011077 p.Gly255Cys LP/P - Ectodermal dysplasia 1, hypohidrotic, X-linked (XHED) [MIM:305100]
EDA Q92838 VAR_011078 p.Gly255Asp LP/P rs1064793105 Ectodermal dysplasia 1, hypohidrotic, X-linked (XHED) [MIM:305100]
EDA Q92838 VAR_011079 p.Trp274Gly LP/P - Ectodermal dysplasia 1, hypohidrotic, X-linked (XHED) [MIM:305100]
EDA Q92838 VAR_011080 p.Cys332Tyr LP/P rs1602624745 Ectodermal dysplasia 1, hypohidrotic, X-linked (XHED) [MIM:305100]
EDA Q92838 VAR_013484 p.Cys60Arg LP/P - Ectodermal dysplasia 1, hypohidrotic, X-linked (XHED) [MIM:305100]
EDA Q92838 VAR_013485 p.His252Tyr LP/P - Ectodermal dysplasia 1, hypohidrotic, X-linked (XHED) [MIM:305100]
EDA Q92838 VAR_013486 p.Gly269Val LP/P - Ectodermal dysplasia 1, hypohidrotic, X-linked (XHED) [MIM:305100]
EDA Q92838 VAR_013487 p.Phe302Ser LP/P - Ectodermal dysplasia 1, hypohidrotic, X-linked (XHED) [MIM:305100]
EDA Q92838 VAR_013488 p.Thr378Met LP/P rs1569407346 Ectodermal dysplasia 1, hypohidrotic, X-linked (XHED) [MIM:305100]
EDA Q92838 VAR_029534 p.Arg65Gly LP/P rs132630319 Tooth agenesis, selective, X-linked, 1 (STHAGX1) [MIM:313500]
EDA Q92838 VAR_036590 p.Pro118Leu US - A colorectal cancer sample
EDA Q92838 VAR_054454 p.Arg153Cys LP/P rs397516662 Ectodermal dysplasia 1, hypohidrotic, X-linked (XHED) [MIM:305100]
EDA Q92838 VAR_054455 p.Arg156Ser LP/P - Ectodermal dysplasia 1, hypohidrotic, X-linked (XHED) [MIM:305100]
EDA Q92838 VAR_054456 p.Lys158Asn LP/P rs727504649 Ectodermal dysplasia 1, hypohidrotic, X-linked (XHED) [MIM:305100]
EDA Q92838 VAR_054460 p.Gly189Glu LP/P - Ectodermal dysplasia 1, hypohidrotic, X-linked (XHED) [MIM:305100]
EDA Q92838 VAR_054462 p.Gly198Ala LP/P - Ectodermal dysplasia 1, hypohidrotic, X-linked (XHED) [MIM:305100]
EDA Q92838 VAR_054463 p.Gly207Arg LP/P - Ectodermal dysplasia 1, hypohidrotic, X-linked (XHED) [MIM:305100]
EDA Q92838 VAR_054464 p.Gly218Asp LP/P - Ectodermal dysplasia 1, hypohidrotic, X-linked (XHED) [MIM:305100]
EDA Q92838 VAR_054466 p.Asp298Tyr LP/P - Ectodermal dysplasia 1, hypohidrotic, X-linked (XHED) [MIM:305100]
EDA Q92838 VAR_054467 p.Gln306His LP/P - Ectodermal dysplasia 1, hypohidrotic, X-linked (XHED) [MIM:305100]
EDA Q92838 VAR_054468 p.Val307Gly LP/P - Ectodermal dysplasia 1, hypohidrotic, X-linked (XHED) [MIM:305100]
EDA Q92838 VAR_054469 p.Tyr320Cys LP/P - Ectodermal dysplasia 1, hypohidrotic, X-linked (XHED) [MIM:305100]

EDA Q92838 VAR_054470 p.Tyr343Cys LP/P - Ectodermal dysplasia 1, hypohidrotic, X-linked (XHED) [MIM:305100]
EDA Q92838 VAR_054471 p.Gln358Glu LP/P rs132630320 Ectodermal dysplasia 1, hypohidrotic, X-linked (XHED) [MIM:305100]
EDA Q92838 VAR_054472 p.Ile360Asn LP/P - Ectodermal dysplasia 1, hypohidrotic, X-linked (XHED) [MIM:305100]
EDA Q92838 VAR_054473 p.Asn372Asp LP/P - Ectodermal dysplasia 1, hypohidrotic, X-linked (XHED) [MIM:305100]
EDA Q92838 VAR_054474 p.Met373Ile LP/P - Ectodermal dysplasia 1, hypohidrotic, X-linked (XHED) [MIM:305100]
EDA Q92838 VAR_054475 p.Ser374Arg LP/P - Ectodermal dysplasia 1, hypohidrotic, X-linked (XHED) [MIM:305100]
EDA Q92838 VAR_054476 p.Thr378Pro LP/P - Ectodermal dysplasia 1, hypohidrotic, X-linked (XHED) [MIM:305100]
EDA Q92838 VAR_064858 p.Arg156Gly LP/P - Ectodermal dysplasia 1, hypohidrotic, X-linked (XHED) [MIM:305100]
EDA Q92838 VAR_064860 p.Gly207Val LP/P - Ectodermal dysplasia 1, hypohidrotic, X-linked (XHED) [MIM:305100]
EDA Q92838 VAR_064861 p.Thr211Arg LP/P - Ectodermal dysplasia 1, hypohidrotic, X-linked (XHED) [MIM:305100]
EDA Q92838 VAR_064862 p.Leu266Arg LP/P - Ectodermal dysplasia 1, hypohidrotic, X-linked (XHED) [MIM:305100]
EDA Q92838 VAR_064863 p.Trp274Arg LP/P - Ectodermal dysplasia 1, hypohidrotic, X-linked (XHED) [MIM:305100]
EDA Q92838 VAR_064864 p.Leu293Pro LP/P - Ectodermal dysplasia 1, hypohidrotic, X-linked (XHED) [MIM:305100]
EDA Q92838 VAR_064865 p.Leu296Val LP/P - Ectodermal dysplasia 1, hypohidrotic, X-linked (XHED) [MIM:305100]
EDA Q92838 VAR_064866 p.Gly299Asp LP/P - Ectodermal dysplasia 1, hypohidrotic, X-linked (XHED) [MIM:305100]
EDA Q92838 VAR_064867 p.Val323Gly LP/P - Ectodermal dysplasia 1, hypohidrotic, X-linked (XHED) [MIM:305100]
EDA Q92838 VAR_064868 p.Thr338Met LP/P rs132630321 Ectodermal dysplasia 1, hypohidrotic, X-linked (XHED) [MIM:305100]
EDA Q92838 VAR_064868 p.Thr338Met LP/P rs132630321 Tooth agenesis, selective, X-linked, 1 (STHAGX1) [MIM:313500]
EDA Q92838 VAR_064869 p.Cys346Tyr LP/P - Ectodermal dysplasia 1, hypohidrotic, X-linked (XHED) [MIM:305100]
EDA Q92838 VAR_064870 p.Ala356Val LP/P rs876657639 Ectodermal dysplasia 1, hypohidrotic, X-linked (XHED) [MIM:305100]
EDA Q92838 VAR_064871 p.Gly381Arg LP/P - Ectodermal dysplasia 1, hypohidrotic, X-linked (XHED) [MIM:305100]
EDA Q92838 VAR_067250 p.Leu354Pro LP/P - Ectodermal dysplasia 1, hypohidrotic, X-linked (XHED) [MIM:305100]
EDA Q92838 VAR_067319 p.Ser319Arg LP/P - Ectodermal dysplasia 1, hypohidrotic, X-linked (XHED) [MIM:305100]
EDA Q92838 VAR_071454 p.Ala259Glu LP/P rs879255611 Tooth agenesis, selective, X-linked, 1 (STHAGX1) [MIM:313500]

EDA Q92838 VAR_071455 p.Ile260Ser LP/P - Tooth agenesis, selective, X-linked, 1 (STHAGX1) [MIM:313500]
EDA Q92838 VAR_071456 p.Arg289Cys LP/P rs879255551 Tooth agenesis, selective, X-linked, 1 (STHAGX1) [MIM:313500]
EDA Q92838 VAR_071457 p.Arg289Leu LP/P - Tooth agenesis, selective, X-linked, 1 (STHAGX1) [MIM:313500]
EDA Q92838 VAR_071458 p.Arg334His LP/P rs142948132 Tooth agenesis, selective, X-linked, 1 (STHAGX1) [MIM:313500]
EDA Q92838 VAR_071459 p.Phe379Val LP/P - Tooth agenesis, selective, X-linked, 1 (STHAGX1) [MIM:313500]
EDA Q92838 VAR_075310 p.Leu51Gln US - Ectodermal dysplasia 1, hypohidrotic, X-linked (XHED) [MIM:305100]
EDA Q92838 VAR_075311 p.Ser125Cys US - Ectodermal dysplasia 1, hypohidrotic, X-linked (XHED) [MIM:305100]
EDA Q92838 VAR_075312 p.Gln132Pro US - Ectodermal dysplasia 1, hypohidrotic, X-linked (XHED) [MIM:305100]
EDA Q92838 VAR_075313 p.Arg153His US rs140642493 Ectodermal dysplasia 1, hypohidrotic, X-linked (XHED) [MIM:305100]
EDA Q92838 VAR_075316 p.Tyr304His US - Ectodermal dysplasia 1, hypohidrotic, X-linked (XHED) [MIM:305100]
EDA Q92838 VAR_075317 p.Asp316Glu US - Ectodermal dysplasia 1, hypohidrotic, X-linked (XHED) [MIM:305100]
EDA Q92838 VAR_075318 p.Cys332Phe US - Ectodermal dysplasia 1, hypohidrotic, X-linked (XHED) [MIM:305100]
EDA Q92838 VAR_075319 p.Gly350Asp US - Ectodermal dysplasia 1, hypohidrotic, X-linked (XHED) [MIM:305100]
EDA Q92838 VAR_075320 p.Gln358His US - Ectodermal dysplasia 1, hypohidrotic, X-linked (XHED) [MIM:305100]
EDA Q92838 VAR_075321 p.Gly381Val US - Ectodermal dysplasia 1, hypohidrotic, X-linked (XHED) [MIM:305100]
EDA Q92838 VAR_077561 p.Gly221Asp US - Ectodermal dysplasia 1, hypohidrotic, X-linked (XHED) [MIM:305100]
EDA2R Q9HAV5 VAR_044511 p.Arg57Lys LB/B rs1385699 -
EDA2R Q9HAV5 VAR_044512 p.Thr129Ala LB/B rs1385698 -
EDAR Q9UNE0 VAR_013448 p.Cys87Arg LP/P rs121908451 Ectodermal dysplasia 10B, hypohidrotic/hair/tooth type, autosomal recessive (ECTD10B) [MIM:224900]
EDAR Q9UNE0 VAR_013449 p.Arg89His LP/P rs121908450 Ectodermal dysplasia 10B, hypohidrotic/hair/tooth type, autosomal recessive (ECTD10B) [MIM:224900]
EDAR Q9UNE0 VAR_013450 p.Arg420Gln LP/P rs121908453 Ectodermal dysplasia 10A, hypohidrotic/hair/nail type, autosomal dominant (ECTD10A) [MIM:129490]
EDAR Q9UNE0 VAR_013450 p.Arg420Gln LP/P rs121908453 Ectodermal dysplasia 10B, hypohidrotic/hair/tooth type, autosomal recessive (ECTD10B) [MIM:224900]
EDAR Q9UNE0 VAR_020011 p.Val370Ala LB/B rs3827760 -
EDAR Q9UNE0 VAR_054444 p.Cys47Tyr LP/P rs778903951 Ectodermal dysplasia 10B, hypohidrotic/hair/tooth type, autosomal recessive (ECTD10B) [MIM:224900]
EDAR Q9UNE0 VAR_054445 p.Asp110Ala LP/P rs121908455 Ectodermal dysplasia 10B, hypohidrotic/hair/tooth type, autosomal recessive (ECTD10B) [MIM:224900]
EDAR Q9UNE0 VAR_054446 p.Cys148Arg LP/P - Ectodermal dysplasia 10B, hypohidrotic/hair/tooth type, autosomal recessive (ECTD10B) [MIM:224900]

EDAR Q9UNE0 VAR_054447 p.Arg375His LP/P rs121908454 Ectodermal dysplasia 10B, hypohidrotic/hair/tooth type, autosomal recessive (ECTD10B) [MIM:224900]
EDAR Q9UNE0 VAR_054448 p.Leu377Phe LP/P - Ectodermal dysplasia 10B, hypohidrotic/hair/tooth type, autosomal recessive (ECTD10B) [MIM:224900]
EDAR Q9UNE0 VAR_054449 p.Gly382Ser LP/P rs747806672 Ectodermal dysplasia 10B, hypohidrotic/hair/tooth type, autosomal recessive (ECTD10B) [MIM:224900]
EDAR Q9UNE0 VAR_054450 p.Thr403Met LP/P - Ectodermal dysplasia 10B, hypohidrotic/hair/tooth type, autosomal recessive (ECTD10B) [MIM:224900]
EDAR Q9UNE0 VAR_054451 p.Thr413Pro LP/P - Ectodermal dysplasia 10B, hypohidrotic/hair/tooth type, autosomal recessive (ECTD10B) [MIM:224900]
EDAR Q9UNE0 VAR_054452 p.Ile418Thr LP/P - Ectodermal dysplasia 10B, hypohidrotic/hair/tooth type, autosomal recessive (ECTD10B) [MIM:224900]
EDAR Q9UNE0 VAR_054453 p.Trp434Cys LP/P rs528478080 Ectodermal dysplasia 10B, hypohidrotic/hair/tooth type, autosomal recessive (ECTD10B) [MIM:224900]
EDAR Q9UNE0 VAR_064830 p.Arg98Gln LP/P rs144473052 Ectodermal dysplasia 10B, hypohidrotic/hair/tooth type, autosomal recessive (ECTD10B) [MIM:224900]
EDAR Q9UNE0 VAR_064831 p.Arg358Gln LP/P rs886039564 Ectodermal dysplasia 10B, hypohidrotic/hair/tooth type, autosomal recessive (ECTD10B) [MIM:224900]
EDAR Q9UNE0 VAR_064833 p.Ile408Phe LP/P - Ectodermal dysplasia 10B, hypohidrotic/hair/tooth type, autosomal recessive (ECTD10B) [MIM:224900]
EDAR Q9UNE0 VAR_064834 p.Trp434Arg LP/P rs773885029 Ectodermal dysplasia 10B, hypohidrotic/hair/tooth type, autosomal recessive (ECTD10B) [MIM:224900]
EDAR Q9UNE0 VAR_077562 p.Val370Leu LB/B rs1267372612 -
EDARADD Q8WWZ3 VAR_013482 p.Glu152Lys LP/P rs74315309 Ectodermal dysplasia 11B, hypohidrotic/hair/tooth type, autosomal recessive (ECTD11B) [MIM:614941]
EDARADD Q8WWZ3 VAR_050963 p.Met9Ile LB/B rs966365 -
EDARADD Q8WWZ3 VAR_054509 p.Ser103Phe LB/B rs114632254 -
EDARADD Q8WWZ3 VAR_054510 p.Leu122Arg LP/P rs121908116 Ectodermal dysplasia 11A, hypohidrotic/hair/nail type, autosomal dominant (ECTD11A) [MIM:614940]
EDARADD Q8WWZ3 VAR_064835 p.Asp114Tyr LP/P - Ectodermal dysplasia 11A, hypohidrotic/hair/nail type, autosomal dominant (ECTD11A) [MIM:614940]
EDC3 Q96F86 VAR_073963 p.Phe54Ser LP/P rs1057517676 Mental retardation, autosomal recessive 50 (MRT50) [MIM:616460]
EDDM3A Q14507 VAR_050968 p.Gly62Cys LB/B rs34552133 -
EDDM3B P56851 VAR_038946 p.Leu5Val LB/B rs3827906 -
EDEM2 Q9BV94 VAR_012165 p.Ala456Thr LB/B rs3746429 -
EDEM2 Q9BV94 VAR_055842 p.Arg510Gln LB/B rs6060248 -
EDEM2 Q9BV94 VAR_055843 p.Leu556Phe LB/B rs1052056 -
EDEM3 Q9BZQ6 VAR_059306 p.Ile820Ser LB/B rs9425343 -
EDN1 P05305 VAR_014188 p.Lys198Asn LB/B rs5370 -
EDN1 P05305 VAR_048933 p.Val186Ile LB/B rs6413478 -
EDN1 P05305 VAR_071152 p.Val64Asp LP/P rs587777233 Question mark ears, isolated (QME) [MIM:612798]
EDN1 P05305 VAR_071153 p.Pro77His LP/P rs587777232 Auriculocondylar syndrome 3 (ARCND3) [MIM:615706]
EDN1 P05305 VAR_071154 p.Lys91Glu LP/P rs587777231 Auriculocondylar syndrome 3 (ARCND3) [MIM:615706]
EDN2 P20800 VAR_018817 p.Pro168Leu LB/B rs11572371 -
EDN2 P20800 VAR_033914 p.Phe131Leu LB/B rs5798 -
EDN3 P14138 VAR_002353 p.Cys159Phe LP/P rs74315384 Waardenburg syndrome 4B (WS4B) [MIM:613265]

EDN3 P14138 VAR_009078 p.Ala17Thr LB/B rs11570255 -
EDN3 P14138 VAR_009079 p.Ala224Thr LP/P rs11570351 Hirschsprung disease 4 (HSCR4) [MIM:613712]
EDN3 P14138 VAR_015238 p.Tyr127Cys LP/P rs752400458 Waardenburg syndrome 4B (WS4B) [MIM:613265]
EDNRA P25101 VAR_035758 p.Ile136Leu US - A breast cancer sample
EDNRA P25101 VAR_073788 p.Tyr129Phe LP/P rs786205230 Mandibulofacial dysostosis with alopecia (MFDA) [MIM:616367]
EDNRA P25101 VAR_073789 p.Glu303Lys LP/P rs876657388 Mandibulofacial dysostosis with alopecia (MFDA) [MIM:616367]
EDNRB P24530 VAR_003469 p.Gly57Ser LB/B rs1801710 -
EDNRB P24530 VAR_003470 p.Ala183Gly LP/P rs104894388 Waardenburg syndrome 4A (WS4A) [MIM:277580]
EDNRB P24530 VAR_003471 p.Trp276Cys LP/P rs104894387 Hirschsprung disease 2 (HSCR2) [MIM:600155]
EDNRB P24530 VAR_003472 p.Ser305Asn LB/B rs5352 -
EDNRB P24530 VAR_003473 p.Arg319Trp LP/P rs200363611 Hirschsprung disease 2 (HSCR2) [MIM:600155]
EDNRB P24530 VAR_003474 p.Met374Ile LP/P - Hirschsprung disease 2 (HSCR2) [MIM:600155]
EDNRB P24530 VAR_003475 p.Pro383Leu LP/P - Hirschsprung disease 2 (HSCR2) [MIM:600155]
EDNRB P24530 VAR_014675 p.Leu7Gln LB/B rs5345 -
EDNRB P24530 VAR_014676 p.Leu17Phe LB/B rs5346 -
EDNRB P24530 VAR_014677 p.Phe112Val LB/B rs5347 -
EDNRB P24530 VAR_014678 p.Thr244Met LB/B rs5350 -
EDNRB P24530 VAR_015294 p.Phe292Leu LP/P - Waardenburg syndrome 4A (WS4A) [MIM:277580]
EDNRB P24530 VAR_019285 p.Pro5Thr LB/B rs12720160 -
EDNRB P24530 VAR_024255 p.Arg76Met LB/B rs2228271 -
EDNRB P24530 VAR_078312 p.Leu17Pro LP/P - -
EDNRB P24530 VAR_078313 p.Asn137Tyr LP/P - -
EDNRB P24530 VAR_078314 p.Pro156Arg LP/P - -
EDRF1 Q3B7T1 VAR_035862 p.Asn45Ser US rs143528650 A colorectal cancer sample
EDRF1 Q3B7T1 VAR_035863 p.Leu95Phe US - A colorectal cancer sample
EEA1 Q15075 VAR_052980 p.Lys810Gln LB/B rs10745623 -
EED O75530 VAR_078316 p.Arg236Thr LP/P rs1131692176 Cohen-Gibson syndrome (COGIS) [MIM:617561]
EED O75530 VAR_078317 p.Arg302Ser LP/P rs1131692173 Cohen-Gibson syndrome (COGIS) [MIM:617561]
EED O75530 VAR_079255 p.Asn194Ser LP/P - Cohen-Gibson syndrome (COGIS) [MIM:617561]
EED O75530 VAR_079256 p.Arg236Gly LP/P - Cohen-Gibson syndrome (COGIS) [MIM:617561]
EED O75530 VAR_079257 p.His258Tyr LP/P rs1131692174 Cohen-Gibson syndrome (COGIS) [MIM:617561]
EED O75530 VAR_079258 p.Arg302Gly LP/P rs1131692175 Cohen-Gibson syndrome (COGIS) [MIM:617561]
EEF1A2 Q05639 VAR_069395 p.Gly70Ser LP/P rs587777162 Developmental and epileptic encephalopathy 33 (DEE33) [MIM:616409]
EEF1A2 Q05639 VAR_073807 p.Glu122Lys LP/P rs786205866 Mental retardation, autosomal dominant 38 (MRD38) [MIM:616393]
EEF1A2 Q05639 VAR_073808 p.Asp252His LP/P rs786205865 Mental retardation, autosomal dominant 38 (MRD38) [MIM:616393]
EEF1A2 Q05639 VAR_079033 p.Ala92Thr US - -
EEF1AKMT1 Q8WVE0 VAR_037216 p.Thr193Asn LB/B rs11549810 -
EEF1AKMT2 Q5JPI9 VAR_050295 p.Arg67Gln LB/B rs4347339 -
EEF1AKMT3 Q96AZ1 VAR_032869 p.Ser28Leu LB/B rs34913183 -
EEF1AKMT4 P0DPD7 VAR_047752 p.His101Tyr LB/B rs7633387 -
EEF1AKNMT Q8N6R0 VAR_034040 p.Met105Val LB/B rs2232816 -
EEF1AKNMT Q8N6R0 VAR_034041 p.Met359Ile LB/B rs2232819 -
EEF1AKNMT Q8N6R0 VAR_064730 p.Tyr16Cys LB/B rs774565178 -

EEF1AKNMT	Q8N6R0	VAR_080810	p.Arg544Gln	LB/B	rs145666727	-
EEF2	P13639	VAR_070792	p.Pro596His	LP/P	rs587777052	Spinocerebellar ataxia 26 (SCA26) [MIM:609306]
EEF2K	O00418	VAR_033915	p.His23Arg	LB/B	rs9935059	-
EEF2K	O00418	VAR_033916	p.Pro75Ala	LB/B	rs17841292	-
EEF2K	O00418	VAR_041534	p.Thr291Met	US	rs147978363	A colorectal adenocarcinoma sample
EEF2K	O00418	VAR_041535	p.Arg433Trp	LB/B	rs56137739	-
EEF2K	O00418	VAR_041536	p.Asp609His	LB/B	-	-
EEF2K	O00418	VAR_058405	p.Gln361Arg	LB/B	rs4783453	-
EEF2KMT	Q96G04	VAR_033854	p.Ser123Cys	LB/B	rs9673733	-
EEF2KMT	Q96G04	VAR_060160	p.Arg270Trp	LB/B	rs3204207	-
EEF2KMT	Q96G04	VAR_067704	p.Val230Ile	LB/B	rs148557961	-
EEF2KMT	Q96G04	VAR_067705	p.His272Asp	LB/B	rs12928528	-
EEF2KMT	Q96G04	VAR_067706	p.Thr329Ile	LB/B	rs760584474	-
EEFSEC	P57772	VAR_055712	p.Ala435Val	LB/B	rs34326479	-
EEPD1	Q7L9B9	VAR_056867	p.Ala358Ser	LB/B	rs3815682	-
EEPD1	Q7L9B9	VAR_056868	p.Pro416Ser	LB/B	rs196594	-
EEPD1	Q7L9B9	VAR_060374	p.Ser343Asn	LB/B	rs196586	-
EFCAB11	Q9BUY7	VAR_032129	p.Ala15Val	LB/B	rs35435801	-
EFCAB11	Q9BUY7	VAR_032130	p.Asp31Gly	LB/B	rs34486581	-
EFCAB11	Q9BUY7	VAR_032131	p.Thr45Ala	LB/B	rs34911716	-
EFCAB12	Q6NXP0	VAR_033697	p.Glu66Gly	LB/B	rs3774787	-
EFCAB12	Q6NXP0	VAR_048667	p.Arg215Gly	LB/B	rs6790768	-
EFCAB12	Q6NXP0	VAR_048668	p.Pro541Ser	LB/B	rs12637267	-
EFCAB12	Q6NXP0	VAR_061089	p.Pro36Leu	LB/B	rs58932597	-
EFCAB12	Q6NXP0	VAR_061090	p.His280Arg	LB/B	rs9836111	-
EFCAB13	Q8IY85	VAR_031228	p.Val312Ile	LB/B	rs4968318	-
EFCAB13	Q8IY85	VAR_031229	p.Met617Val	LB/B	rs17855599	-
EFCAB13	Q8IY85	VAR_035465	p.Gln286His	US	-	A breast cancer sample
EFCAB13	Q8IY85	VAR_061091	p.Ile279Val	LB/B	rs55853213	-
EFCAB14	O75071	VAR_048669	p.Leu337Pro	LB/B	rs6665021	-
EFCAB14	O75071	VAR_074176	p.Pro239Ser	LB/B	-	-
EFCAB3	Q8N7B9	VAR_028377	p.Gly341Arg	LB/B	rs12602985	-
EFCAB3	Q8N7B9	VAR_028378	p.Met364Ile	LB/B	rs1056642	-
EFCAB3	Q8N7B9	VAR_028379	p.Ser370Ala	LB/B	rs1056643	-
EFCAB5	A4FU69	VAR_038343	p.Leu237Val	LB/B	rs9897794	-
EFCAB5	A4FU69	VAR_038344	p.Ile278Lys	LB/B	rs4795524	-
EFCAB5	A4FU69	VAR_038345	p.Arg561Ser	LB/B	rs9900546	-
EFCAB5	A4FU69	VAR_048644	p.Ala1145Asp	LB/B	rs9894896	-
EFCAB5	A4FU69	VAR_048645	p.Val1252Ala	LB/B	rs4499292	-
EFCAB5	A4FU69	VAR_048646	p.Arg1274Thr	LB/B	rs35724168	-
EFCAB6	Q5THR3	VAR_048648	p.Val166Ala	LB/B	rs16990981	-
EFCAB6	Q5THR3	VAR_048649	p.Arg199Gly	LB/B	rs3747203	-
EFCAB6	Q5THR3	VAR_048650	p.Thr351Ala	LB/B	rs5764214	-
EFCAB6	Q5THR3	VAR_048651	p.Ser384Ala	LB/B	rs6006438	-
EFCAB6	Q5THR3	VAR_048652	p.His400Tyr	LB/B	rs137794	-
EFCAB6	Q5THR3	VAR_048653	p.Thr680Ala	LB/B	rs137731	-
EFCAB6	Q5THR3	VAR_048654	p.Asp780Asn	LB/B	rs12159591	-
EFCAB6	Q5THR3	VAR_048655	p.Arg800Trp	LB/B	rs6006514	-
EFCAB6	Q5THR3	VAR_048656	p.Ala1059Val	LB/B	rs9614382	-

EFCAB7 A8K855 VAR_038493 p.Phe27Ile LB/B rs17125106 -
EFCAB7 A8K855 VAR_038494 p.Ser186Gly LB/B rs9436246 -
EFCAB7 A8K855 VAR_038495 p.Thr248Ile LB/B rs6693255 -
EFCAB7 A8K855 VAR_038496 p.Met262Thr LB/B rs6657480 -
EFCAB7 A8K855 VAR_038497 p.Arg375Lys LB/B rs2273367 -
EFCC1 Q9HA90 VAR_050753 p.Arg528Gln LB/B rs3732430 -
EFEMP1 Q12805 VAR_009512 p.Ile220Phe LB/B rs748965004 -
EFEMP1 Q12805 VAR_009513 p.Arg345Trp LP/P rs121434491 Doyne honeycomb retinal dystrophy (DHRD) [MIM:126600]
EFEMP2 O95967 VAR_027019 p.Glu57Lys LP/P rs119489101 Cutis laxa, autosomal recessive, 1B (ARCL1B) [MIM:614437]
EFEMP2 O95967 VAR_027020 p.Ile259Val LB/B rs601314 -
EFEMP2 O95967 VAR_067069 p.Cys267Tyr LP/P rs193302866 Cutis laxa, autosomal recessive, 1B (ARCL1B) [MIM:614437]
EFEMP2 O95967 VAR_067070 p.Arg279Cys LP/P rs119489102 Cutis laxa, autosomal recessive, 1B (ARCL1B) [MIM:614437]
EFEMP2 O95967 VAR_084029 p.Glu126Lys LP/P - Cutis laxa, autosomal recessive, 1B (ARCL1B) [MIM:614437]
EFEMP2 O95967 VAR_084030 p.Glu126Val LP/P - Cutis laxa, autosomal recessive, 1B (ARCL1B) [MIM:614437]
EFEMP2 O95967 VAR_084031 p.Asp203Ala LP/P - Cutis laxa, autosomal recessive, 1B (ARCL1B) [MIM:614437]
EFEMP2 O95967 VAR_084032 p.Arg227Cys LP/P - Cutis laxa, autosomal recessive, 1B (ARCL1B) [MIM:614437]
EFEMP2 O95967 VAR_084033 p.Ala397Thr LP/P - Cutis laxa, autosomal recessive, 1B (ARCL1B) [MIM:614437]
EFHB Q8N7U6 VAR_027750 p.Val331Ile LB/B rs2931403 -
EFHB Q8N7U6 VAR_027751 p.Thr382Ile LB/B rs2929366 -
EFHB Q8N7U6 VAR_027752 p.Gln663Pro LB/B rs9868950 -
EFHB Q8N7U6 VAR_055296 p.Gly99Val LB/B rs17795400 -
EFHB Q8N7U6 VAR_055297 p.Pro269Ser LB/B rs13078867 -
EFHB Q8N7U6 VAR_055298 p.Arg826Trp LB/B rs11917204 -
EFHC1 Q5JVL4 VAR_023619 p.Pro77Thr LP/P rs149055334 Juvenile myoclonic epilepsy 1 (EJM1) [MIM:254770]
EFHC1 Q5JVL4 VAR_023620 p.Arg159Trp LB/B rs3804506 -
EFHC1 Q5JVL4 VAR_023621 p.Arg182His LB/B rs3804505 -
EFHC1 Q5JVL4 VAR_023622 p.Asp210Asn LP/P rs137852777 Juvenile myoclonic epilepsy 1 (EJM1) [MIM:254770]
EFHC1 Q5JVL4 VAR_023623 p.Arg221His LP/P rs79761183 Juvenile myoclonic epilepsy 1 (EJM1) [MIM:254770]
EFHC1 Q5JVL4 VAR_023624 p.Phe229Leu US rs137852776 Juvenile myoclonic epilepsy 1 (EJM1) [MIM:254770]
EFHC1 Q5JVL4 VAR_023625 p.Asp253Tyr LP/P rs137852778 Juvenile myoclonic epilepsy 1 (EJM1) [MIM:254770]
EFHC1 Q5JVL4 VAR_023626 p.Ile619Leu LB/B rs17851770 -
EFHC1 Q5JVL4 VAR_026531 p.Arg285Ile LB/B rs17851771 -
EFHC1 Q5JVL4 VAR_043154 p.Ile174Val LB/B rs137852779 -
EFHC1 Q5JVL4 VAR_043155 p.Cys259Tyr LB/B rs137852780 -
EFHC1 Q5JVL4 VAR_043156 p.Arg294His LB/B rs1570624 -

EFHC1	Q5JVL4	VAR_043157	p.Arg353Trp	LP/P	rs527295360	Juvenile myoclonic epilepsy 1 (EJM1) [MIM:254770]
EFHC1	Q5JVL4	VAR_043158	p.Ala394Ser	US	-	-
EFHC1	Q5JVL4	VAR_043159	p.Met448Thr	LB/B	rs1266787	-
EFHC1	Q5JVL4	VAR_048666	p.Glu357Lys	LB/B	rs505760	-
EFHC1	Q5JVL4	VAR_072108	p.Arg118Cys	LP/P	rs764096785	Juvenile myoclonic epilepsy 1 (EJM1) [MIM:254770]
EFHC1	Q5JVL4	VAR_072109	p.Arg153Gln	US	rs745600475	Juvenile myoclonic epilepsy 1 (EJM1) [MIM:254770]
EFHC1	Q5JVL4	VAR_072110	p.Arg182Cys	US	rs200191497	Juvenile myoclonic epilepsy 1 (EJM1) [MIM:254770]
EFHC1	Q5JVL4	VAR_079772	p.His89Arg	LP/P	rs543160745	Juvenile myoclonic epilepsy 1 (EJM1) [MIM:254770]
EFHC1	Q5JVL4	VAR_079773	p.Arg221Cys	LB/B	rs139197513	-
EFHC1	Q5JVL4	VAR_079774	p.Glu322Lys	LP/P	-	Juvenile myoclonic epilepsy 1 (EJM1) [MIM:254770]
EFHC1	Q5JVL4	VAR_079775	p.Tyr355Cys	LP/P	rs767833659	Juvenile myoclonic epilepsy 1 (EJM1) [MIM:254770]
EFHC1	Q5JVL4	VAR_079776	p.Arg372Trp	LP/P	rs371151471	Juvenile myoclonic epilepsy 1 (EJM1) [MIM:254770]
EFHC1	Q5JVL4	VAR_079777	p.Lys378Glu	LP/P	-	Juvenile myoclonic epilepsy 1 (EJM1) [MIM:254770]
EFHC1	Q5JVL4	VAR_079778	p.Arg436Cys	LP/P	rs377286138	Juvenile myoclonic epilepsy 1 (EJM1) [MIM:254770]
EFHC1	Q5JVL4	VAR_079779	p.Tyr485His	LP/P	rs779322943	Juvenile myoclonic epilepsy 1 (EJM1) [MIM:254770]
EFHC1	Q5JVL4	VAR_079780	p.Asn519Ser	LP/P	rs527539103	Juvenile myoclonic epilepsy 1 (EJM1) [MIM:254770]
EFHC1	Q5JVL4	VAR_079781	p.Val556Leu	LP/P	rs772265107	Juvenile myoclonic epilepsy 1 (EJM1) [MIM:254770]
EFHC1	Q5JVL4	VAR_079782	p.Ile619Ser	LP/P	rs142458862	Juvenile myoclonic epilepsy 1 (EJM1) [MIM:254770]
EFHC1	Q5JVL4	VAR_079783	p.Tyr631Cys	LP/P	rs574948354	Juvenile myoclonic epilepsy 1 (EJM1) [MIM:254770]
EFHC2	Q5JST6	VAR_027698	p.Asn31Ser	LB/B	rs17146914	-
EFHC2	Q5JST6	VAR_027699	p.Glu208Lys	LB/B	rs7062352	-
EFHC2	Q5JST6	VAR_027700	p.Ser430Tyr	LB/B	rs2208592	-
EFHC2	Q5JST6	VAR_027701	p.Glu507Gln	LB/B	rs3747354	-
EFHC2	Q5JST6	VAR_061086	p.Cys57Tyr	LB/B	rs61636783	-
EFHD1	Q9BUP0	VAR_047966	p.Lys186Arg	LB/B	rs11550699	-
EFL1	Q7Z2Z2	VAR_037746	p.Glu478Asp	LB/B	rs2292189	-
EFL1	Q7Z2Z2	VAR_037747	p.Ile617Val	LB/B	rs1128431	-
EFL1	Q7Z2Z2	VAR_037748	p.Lys711Arg	LB/B	rs2292071	-
EFL1	Q7Z2Z2	VAR_080513	p.Met882Lys	US	rs1316615934	Shwachman-Diamond syndrome 2 (SDS2) [MIM:617941]
EFL1	Q7Z2Z2	VAR_080514	p.Arg1095Gln	US	rs376095522	Shwachman-Diamond syndrome 2 (SDS2) [MIM:617941]
EFNA1	P20827	VAR_014791	p.Asp159Val	LB/B	rs4745	-
EFNA3	P52797	VAR_048937	p.Val190Met	LB/B	rs17723260	-
EFNA5	P52803	VAR_012035	p.Asn55Lys	LB/B	rs469062	-
EFNB1	P98172	VAR_023127	p.Pro27Arg	LP/P	-	Craniofrontonasal syndrome (CFNS) [MIM:304110]

EFNB1	P98172	VAR_023128	p.Pro54Leu	LP/P	rs104894801	Craniofrontonasal syndrome (CFNS) [MIM:304110]
EFNB1	P98172	VAR_023129	p.Ile62Thr	LP/P	-	Craniofrontonasal syndrome (CFNS) [MIM:304110]
EFNB1	P98172	VAR_023130	p.Leu98Ser	LP/P	-	Craniofrontonasal syndrome (CFNS) [MIM:304110]
EFNB1	P98172	VAR_023131	p.Thr111Ile	LP/P	rs104894796	Craniofrontonasal syndrome (CFNS) [MIM:304110]
EFNB1	P98172	VAR_023132	p.Gln115Pro	LP/P	-	Craniofrontonasal syndrome (CFNS) [MIM:304110]
EFNB1	P98172	VAR_023133	p.Pro119His	LP/P	-	Craniofrontonasal syndrome (CFNS) [MIM:304110]
EFNB1	P98172	VAR_023134	p.Pro119Ser	LP/P	-	Craniofrontonasal syndrome (CFNS) [MIM:304110]
EFNB1	P98172	VAR_023135	p.Pro119Thr	LP/P	-	Craniofrontonasal syndrome (CFNS) [MIM:304110]
EFNB1	P98172	VAR_023136	p.Thr137Ala	LP/P	-	Craniofrontonasal syndrome (CFNS) [MIM:304110]
EFNB1	P98172	VAR_023137	p.Ser138Phe	LP/P	-	Craniofrontonasal syndrome (CFNS) [MIM:304110]
EFNB1	P98172	VAR_023138	p.Gly151Ser	LP/P	rs28936069	Craniofrontonasal syndrome (CFNS) [MIM:304110]
EFNB1	P98172	VAR_023139	p.Gly151Val	LP/P	rs28936070	Craniofrontonasal syndrome (CFNS) [MIM:304110]
EFNB1	P98172	VAR_023140	p.Cys153Ser	LP/P	-	Craniofrontonasal syndrome (CFNS) [MIM:304110]
EFNB1	P98172	VAR_023141	p.Cys153Tyr	LP/P	-	Craniofrontonasal syndrome (CFNS) [MIM:304110]
EFNB1	P98172	VAR_023142	p.Arg154His	LB/B	rs146636295	-
EFNB1	P98172	VAR_023143	p.Thr155Pro	LP/P	-	Craniofrontonasal syndrome (CFNS) [MIM:304110]
EFNB1	P98172	VAR_023144	p.Met158Ile	LP/P	rs28935170	Craniofrontonasal syndrome (CFNS) [MIM:304110]
EFNB1	P98172	VAR_023145	p.Met158Val	LP/P	rs28936071	Craniofrontonasal syndrome (CFNS) [MIM:304110]
EFNB1	P98172	VAR_023146	p.Ser182Arg	LP/P	-	Craniofrontonasal syndrome (CFNS) [MIM:304110]
EFNB1	P98172	VAR_023147	p.Val189Ala	LB/B	rs16989105	-
EFNB1	P98172	VAR_059256	p.Thr172Met	LB/B	rs7889678	-
EFNB3	Q15768	VAR_002356	p.Arg166Gln	LB/B	rs1165640333	-
EFR3A	Q14156	VAR_047247	p.Gly358Arg	LB/B	rs2270877	-
EFR3A	Q14156	VAR_047248	p.Asn365Asp	LB/B	rs1051221	-
EFR3A	Q14156	VAR_075101	p.Pro14Arg	LB/B	-	-
EFR3A	Q14156	VAR_075102	p.Lys50Glu	LB/B	-	-
EFR3A	Q14156	VAR_075103	p.Gly55Cys	LB/B	rs749463078	-
EFR3A	Q14156	VAR_075104	p.Arg70Cys	LB/B	rs1212454955	-
EFR3A	Q14156	VAR_075105	p.Phe100Leu	LB/B	-	-
EFR3A	Q14156	VAR_075106	p.Leu118Pro	LB/B	-	-
EFR3A	Q14156	VAR_075107	p.Phe123Leu	LB/B	rs1323253445	-
EFR3A	Q14156	VAR_075108	p.Met194Val	LB/B	rs780864616	-
EFR3A	Q14156	VAR_075109	p.Gly243Ala	LB/B	-	-
EFR3A	Q14156	VAR_075110	p.Asp268Gly	LB/B	-	-
EFR3A	Q14156	VAR_075111	p.Glu320Asp	LB/B	-	-
EFR3A	Q14156	VAR_075112	p.Ala321Ser	LB/B	rs774959333	-
EFR3A	Q14156	VAR_075113	p.Val337Leu	LB/B	-	-
EFR3A	Q14156	VAR_075114	p.Phe338Ser	LB/B	-	-
EFR3A	Q14156	VAR_075115	p.Asn354Asp	LB/B	rs754610866	-
EFR3A	Q14156	VAR_075116	p.Thr451Met	LB/B	rs770980074	-
EFR3A	Q14156	VAR_075117	p.Asp504Gly	LB/B	-	-
EFR3A	Q14156	VAR_075118	p.Leu508Pro	LB/B	-	-
EFR3A	Q14156	VAR_075119	p.Ile510Val	LB/B	-	-
EFR3A	Q14156	VAR_075120	p.Gln528Arg	LB/B	-	-
EFR3A	Q14156	VAR_075121	p.Arg532Trp	LB/B	rs779475356	-
EFR3A	Q14156	VAR_075122	p.Ile534Thr	LB/B	rs374094815	-
EFR3A	Q14156	VAR_075123	p.Asp570Val	LB/B	-	-

EFR3A Q14156 VAR_075124 p.Met646Val LB/B rs759848268 -
EFR3A Q14156 VAR_075125 p.Thr785Ala LB/B rs1467962026 -
EFS O43281 VAR_035912 p.Met361Ile US rs1463886157 A colorectal cancer sample
EFS O43281 VAR_054088 p.Thr7Ala LB/B rs2231798 -
EFS O43281 VAR_054089 p.Val100Met LB/B rs2231801 -
EFTUD2 Q15029 VAR_014931 p.Gly773Val LB/B rs1056505 -
EFTUD2 Q15029 VAR_067580 p.Arg262Trp LP/P rs387906877 Mandibulofacial dysostosis with microcephaly (MFDM) [MIM:610536]
EFTUD2 Q15029 VAR_067581 p.Cys476Arg LP/P - Mandibulofacial dysostosis with microcephaly (MFDM) [MIM:610536]
EFTUD2 Q15029 VAR_067582 p.Leu637Arg LP/P rs387906879 Mandibulofacial dysostosis with microcephaly (MFDM) [MIM:610536]
EGF P01133 VAR_002275 p.Met708Ile LB/B rs2237051 -
EGF P01133 VAR_020161 p.Ser16Arg LB/B rs11568849 -
EGF P01133 VAR_020162 p.Arg431Lys LB/B rs11568943 -
EGF P01133 VAR_020163 p.Gly723Arg LB/B rs6413481 -
EGF P01133 VAR_020164 p.Asp784Val LB/B rs11569017 -
EGF P01133 VAR_020165 p.Met842Thr LB/B rs11569046 -
EGF P01133 VAR_020166 p.Leu1043Phe LB/B rs11569098 -
EGF P01133 VAR_020968 p.Asp257His LB/B rs11568911 -
EGF P01133 VAR_020969 p.Ser638Arg LB/B rs11568992 -
EGF P01133 VAR_020970 p.Glu920Val LB/B rs4698803 -
EGF P01133 VAR_020971 p.Asp981Glu LB/B rs11569086 -
EGF P01133 VAR_020972 p.Ala1084Gly LB/B rs11569111 -
EGF P01133 VAR_033825 p.His151Tyr LB/B rs9991664 -
EGF P01133 VAR_033826 p.Leu292His LB/B rs35191533 -
EGF P01133 VAR_039474 p.Pro1070Leu LP/P rs121434567 Hypomagnesemia 4 (HOMG4) [MIM:611718]
EGFEM1P Q0D2K5 VAR_044016 p.Gly59Asp LB/B rs603638 -
EGFEM1P Q0D2K5 VAR_044017 p.Pro157Leu LB/B rs678690 -
EGFL6 Q8IUX8 VAR_033366 p.Glu66Lys LB/B rs16979010 -
EGFL6 Q8IUX8 VAR_033367 p.Arg164Cys LB/B rs34613284 -
EGFL6 Q8IUX8 VAR_033368 p.Leu508Phe LB/B rs34550481 -
EGFL6 Q8IUX8 VAR_033369 p.Asp535Asn LB/B rs16979033 -
EGFL7 Q9UHF1 VAR_019791 p.Val153Ile LB/B rs2297538 -
EGFL7 Q9UHF1 VAR_048981 p.Pro183Ser LB/B rs35863900 -
EGFL7 Q9UHF1 VAR_048982 p.Ala186Gly LB/B rs34142075 -
EGFL7 Q9UHF1 VAR_070951 p.Gly114Arg LB/B rs61736886 -
EGFL8 Q99944 VAR_019792 p.Arg86Lys LB/B rs3096697 -
EGFL8 Q99944 VAR_019793 p.Ala204Glu LB/B rs2071289 -
EGFL8 Q99944 VAR_048983 p.Gly277Cys LB/B rs35587174 -
EGFLAM Q63HQ2 VAR_035302 p.Arg111His LB/B rs2561111 -
EGFLAM Q63HQ2 VAR_035303 p.Trp229Arg LB/B rs1465567 -
EGFLAM Q63HQ2 VAR_035304 p.Thr473Met LB/B rs16903965 -
EGFLAM Q63HQ2 VAR_035305 p.His576Asn LB/B rs6897179 -
EGFLAM Q63HQ2 VAR_055718 p.Arg26Pro LB/B rs12522205 -
EGFLAM Q63HQ2 VAR_055719 p.Val745Met LB/B rs2561818 -
EGFR P00533 VAR_019293 p.Arg98Gln LB/B rs17289589 -
EGFR P00533 VAR_019294 p.Pro266Arg LB/B rs17336639 -
EGFR P00533 VAR_019295 p.Arg521Lys LB/B rs2227983 -

EGFR P00533 VAR_019296 p.Val674Ile LB/B rs17337079 -
EGFR P00533 VAR_019297 p.Gly719Ser LB/B rs28929495 -
EGFR P00533 VAR_019298 p.Leu858Arg LB/B rs121434568 -
EGFR P00533 VAR_019299 p.Arg962Gly LB/B rs17337451 -
EGFR P00533 VAR_019300 p.His988Pro LB/B rs17290699 -
EGFR P00533 VAR_026084 p.Glu709Ala LB/B rs397517085 -
EGFR P00533 VAR_026085 p.Glu709Lys LB/B rs727504256 -
EGFR P00533 VAR_026086 p.Gly719Ala LB/B rs121913428 -
EGFR P00533 VAR_026087 p.Gly719Cys LB/B rs28929495 -
EGFR P00533 VAR_026088 p.Gly719Asp LB/B rs121913428 -
EGFR P00533 VAR_026089 p.Gly724Ser LB/B rs1051753269 -
EGFR P00533 VAR_026090 p.Glu734Lys LB/B rs121913420 -
EGFR P00533 VAR_026093 p.Leu747Phe US - -
EGFR P00533 VAR_026095 p.Arg748Pro US - -
EGFR P00533 VAR_026097 p.Gln787Arg US - -
EGFR P00533 VAR_026098 p.Thr790Met LB/B rs121434569 -
EGFR P00533 VAR_026099 p.Leu833Val LB/B rs397517126 -
EGFR P00533 VAR_026100 p.Val834Leu LB/B rs397517127 -
EGFR P00533 VAR_026101 p.Leu858Met LB/B rs121913443 -
EGFR P00533 VAR_026102 p.Leu861Gln LB/B rs121913444 -
EGFR P00533 VAR_026103 p.Gly873Glu US - -
EGFR P00533 VAR_042095 p.Leu1034Arg LB/B rs34352568 -
EGFR P00533 VAR_042096 p.Ala1210Val LB/B rs35918369 -
EGFR P00533 VAR_069498 p.Glu709Gly LB/B rs397517085 -
EGFR P00533 VAR_069502 p.Ser768Ile LB/B rs121913465 -
EGFR P00533 VAR_069503 p.Val769Met LB/B rs147149347 -
EGFR P00533 VAR_069504 p.His835Leu LB/B rs397517128 -
EGFR P00533 VAR_069505 p.Leu838Val LB/B rs864621996 -
EGFR P00533 VAR_072435 p.Gly428Asp LP/P rs606231253 Inflammatory skin and bowel disease, neonatal, 2 (NISBD2) [MIM:616069]
EGLN1 Q9GZT9 VAR_027371 p.Pro317Arg LP/P rs80358193 Erythrocytosis, familial, 3 (ECYT3) [MIM:609820]
EGLN1 Q9GZT9 VAR_045902 p.Arg371His LP/P rs119476044 Erythrocytosis, familial, 3 (ECYT3) [MIM:609820]
EGLN1 Q9GZT9 VAR_071858 p.Asp4Glu LB/B rs186996510 -
EGLN1 Q9GZT9 VAR_071859 p.Cys127Ser LB/B rs12097901 -
EGLN3 Q9H6Z9 VAR_050449 p.Val136Leu LB/B rs17102002 -
EGLN3 Q9H6Z9 VAR_050450 p.Ser234Thr LB/B rs17101995 -
EGR1 P18146 VAR_029330 p.Asn144Lys LB/B rs28365166 -
EGR1 P18146 VAR_029331 p.Ser145Arg LB/B rs28365164 -
EGR1 P18146 VAR_029332 p.Glu219Asp LB/B rs28365165 -
EGR1 P18146 VAR_052712 p.Thr28Ile LB/B rs13181973 -
EGR2 P11161 VAR_007735 p.Ile268Asn LP/P rs104894158 Neuropathy, congenital hypomyelinating, 1, autosomal recessive (CHN1) [MIM:605253]
EGR2 P11161 VAR_007736 p.Asp355Val LP/P - Charcot-Marie-Tooth disease 1D (CMT1D) [MIM:607678]
EGR2 P11161 VAR_007738 p.Arg409Trp LP/P rs104894159 Charcot-Marie-Tooth disease 1D (CMT1D) [MIM:607678]
EGR2 P11161 VAR_009874 p.Arg359Trp LP/P rs104894161 Charcot-Marie-Tooth disease 1D (CMT1D) [MIM:607678]
EGR2 P11161 VAR_009874 p.Arg359Trp LP/P rs104894161 Dejerine-Sottas syndrome (DSS) [MIM:145900]

EGR2 P11161 VAR_009875 p.Arg381His LP/P rs281865137 Charcot-Marie-Tooth disease 1D (CMT1D) [MIM:607678]
EGR2 P11161 VAR_029958 p.Arg381Cys LP/P - Charcot-Marie-Tooth disease 1D (CMT1D) [MIM:607678]
EGR2 P11161 VAR_029959 p.Asp383Tyr LP/P rs104894160 Charcot-Marie-Tooth disease 1D (CMT1D) [MIM:607678]
EGR2 P11161 VAR_083343 p.Thr387Asn US rs281865139 Charcot-Marie-Tooth disease 1D (CMT1D) [MIM:607678]
EGR2 P11161 VAR_083344 p.Asp411Gly LP/P - Dejerine-Sottas syndrome (DSS) [MIM:145900]
EGR2 P11161 VAR_083345 p.Glu412Gly LP/P rs749558026 Charcot-Marie-Tooth disease 1D (CMT1D) [MIM:607678]
EGR2 P11161 VAR_083346 p.Glu412Lys LP/P rs121434563 Dejerine-Sottas syndrome (DSS) [MIM:145900]
EHBP1 Q8NDI1 VAR_031992 p.Lys755Gln LB/B rs17432615 -
EHBP1 Q8NDI1 VAR_035913 p.Arg395Thr US - A breast cancer sample
EHBP1L1 Q8N3D4 VAR_031993 p.Arg307Gln LB/B rs3741380 -
EHBP1L1 Q8N3D4 VAR_031994 p.Val538Gly LB/B rs6591182 -
EHBP1L1 Q8N3D4 VAR_031995 p.Asp569Val LB/B rs1194099 -
EHBP1L1 Q8N3D4 VAR_031996 p.Thr599Ile LB/B rs7931052 -
EHBP1L1 Q8N3D4 VAR_031997 p.Thr648Ile LB/B rs7931269 -
EHBP1L1 Q8N3D4 VAR_061645 p.Val133Leu LB/B rs1194100 -
EHD2 Q9NZN4 VAR_033917 p.Gly57Ser LB/B rs34140460 -
EHD4 Q9H223 VAR_053070 p.Val154Ile LB/B rs11549015 -
EHF Q9NZC4 VAR_048941 p.Ala96Val LB/B rs9804460 -
EHHADH Q08426 VAR_047132 p.Thr75Ile LB/B rs1062553 -
EHHADH Q08426 VAR_047133 p.Ala274Thr LB/B rs2302819 -
EHHADH Q08426 VAR_047134 p.Thr606Pro LB/B rs1042438 -
EHHADH Q08426 VAR_047135 p.Gln685Lys LB/B rs11919970 -
EHHADH Q08426 VAR_047136 p.Leu715Ser LB/B rs11927618 -
EHHADH Q08426 VAR_054329 p.Val40Gly LB/B rs1062551 -
EHHADH Q08426 VAR_054330 p.Ile41Arg LB/B rs1062552 -
EHHADH Q08426 VAR_054331 p.Ala325Gly LB/B rs1062555 -
EHHADH Q08426 VAR_054332 p.Lys598Thr LB/B rs1042437 -
EHHADH Q08426 VAR_070949 p.Glu3Lys LP/P rs398124646 Fanconi renotubular syndrome 3 (FRTS3) [MIM:615605]
EHMT1 Q9H9B1 VAR_027642 p.Ala388Thr LB/B rs11137198 -
EHMT1 Q9H9B1 VAR_036345 p.Ala43Val US rs79514677 A breast cancer sample
EHMT1 Q9H9B1 VAR_036346 p.Tyr1173Phe US - A breast cancer sample
EHMT1 Q9H9B1 VAR_069183 p.Cys1075Tyr LP/P - Kleefstra syndrome 1 (KLEFS1) [MIM:610253]
EHMT2 Q96KQ7 VAR_027973 p.Thr55Asn LB/B rs7887 -
EHMT2 Q96KQ7 VAR_027974 p.Tyr1165Phe LB/B rs13919 -
EI24 O14681 VAR_065459 p.Asp30Gly US - Some patients with early onset breast cancer
EI24 O14681 VAR_065460 p.Pro195Trp US - Some patients with early onset breast cancer
EI24 O14681 VAR_065461 p.Ile196Asp US - Some patients with early onset breast cancer
EI24 O14681 VAR_065462 p.His197Tyr US - Some patients with early onset breast cancer
EI24 O14681 VAR_065463 p.Val199His US - Some patients with early onset breast cancer
EI24 O14681 VAR_065464 p.Thr319Ala US rs375652371 Some patients with early onset breast cancer
EID2 Q8N6I1 VAR_038351 p.Ala6Thr LB/B rs7252027 -
EID2 Q8N6I1 VAR_050964 p.Glu60Ala LB/B rs3746086 -
EIF1 P41567 VAR_052505 p.Leu59Pro LB/B rs3390 -
EIF1 P41567 VAR_052506 p.Arg90Gly LB/B rs3387 -

EIF1AD Q8N9N8 VAR_037851 p.Asp23Asn LB/B rs17849919 -
EIF1AD Q8N9N8 VAR_037852 p.Ser159Asn LB/B rs2276017 -
EIF2A Q9BY44 VAR_032066 p.Thr97Ser LB/B rs1132979 -
EIF2A Q9BY44 VAR_032067 p.Glu582Lys LB/B rs17850813 -
EIF2AK1 Q9BQI3 VAR_015732 p.Lys558Arg LB/B rs2640 -
EIF2AK1 Q9BQI3 VAR_040466 p.Arg117Thr LB/B rs34889754 -
EIF2AK1 Q9BQI3 VAR_040467 p.Lys132Thr LB/B rs34851195 -
EIF2AK1 Q9BQI3 VAR_040468 p.Arg134Lys LB/B rs55744865 -
EIF2AK1 Q9BQI3 VAR_040469 p.Pro139Ser LB/B rs55963745 -
EIF2AK1 Q9BQI3 VAR_040470 p.Arg145His LB/B rs55971369 -
EIF2AK1 Q9BQI3 VAR_040471 p.Gly202Ser US rs1216460058 A lung adenocarcinoma sample
EIF2AK1 Q9BQI3 VAR_040472 p.Phe292Leu LB/B rs55982710 -
EIF2AK1 Q9BQI3 VAR_040473 p.Leu319His LB/B rs34909691 -
EIF2AK1 Q9BQI3 VAR_084259 p.Ile448Val US - Leukoencephalopathy, motor delay, spasticity, and dysarthria syndrome (LEMSPAD) [MIM:618878]
EIF2AK2 P19525 VAR_040474 p.Val428Glu LB/B rs56219559 -
EIF2AK2 P19525 VAR_040475 p.Leu439Val US - A lung adenocarcinoma sample
EIF2AK2 P19525 VAR_040476 p.Ile506Val LB/B rs34821155 -
EIF2AK2 P19525 VAR_084260 p.Met11Leu US - Leukoencephalopathy, developmental delay, and episodic neurologic regression syndrome (LEUDEN) [MIM:618877]
EIF2AK2 P19525 VAR_084261 p.Asn32Ser US - Leukoencephalopathy, developmental delay, and episodic neurologic regression syndrome (LEUDEN) [MIM:618877]
EIF2AK2 P19525 VAR_084262 p.Ser97Phe US - Leukoencephalopathy, developmental delay, and episodic neurologic regression syndrome (LEUDEN) [MIM:618877]
EIF2AK2 P19525 VAR_084263 p.Ala109Ser US - Leukoencephalopathy, developmental delay, and episodic neurologic regression syndrome (LEUDEN) [MIM:618877]
EIF2AK2 P19525 VAR_084264 p.Ala109Val US - Leukoencephalopathy, developmental delay, and episodic neurologic regression syndrome (LEUDEN) [MIM:618877]
EIF2AK2 P19525 VAR_084265 p.Leu114Gln US - -
EIF2AK2 P19525 VAR_084266 p.Tyr133Phe US - Leukoencephalopathy, developmental delay, and episodic neurologic regression syndrome (LEUDEN) [MIM:618877]
EIF2AK2 P19525 VAR_084267 p.Gly325Ser US - Leukoencephalopathy, developmental delay, and episodic neurologic regression syndrome (LEUDEN) [MIM:618877]
EIF2AK2 P19525 VAR_084268 p.Ser461Cys US - Leukoencephalopathy, developmental delay, and episodic neurologic regression syndrome (LEUDEN) [MIM:618877]
EIF2AK3 Q9NZJ5 VAR_011408 p.Arg588Gln LP/P rs121908569 Wolcott-Rallison syndrome (WRS) [MIM:226980]
EIF2AK3 Q9NZJ5 VAR_011409 p.Ser136Cys LB/B rs867529 -
EIF2AK3 Q9NZJ5 VAR_011410 p.Gln166Arg LB/B rs13045 -
EIF2AK3 Q9NZJ5 VAR_011411 p.Ala704Ser LB/B rs1805165 -
EIF2AK3 Q9NZJ5 VAR_040477 p.Asp566Val LB/B rs55791823 -
EIF2AK3 Q9NZJ5 VAR_040478 p.Pro716Leu LB/B rs55861585 -
EIF2AK4 Q9P2K8 VAR_040479 p.His137Arg LB/B rs35509999 -
EIF2AK4 Q9P2K8 VAR_040480 p.Arg166Trp LB/B rs34439704 -
EIF2AK4 Q9P2K8 VAR_040481 p.Ile441Leu LB/B rs2291627 -
EIF2AK4 Q9P2K8 VAR_040482 p.Asp872Val LB/B rs34665481 -
EIF2AK4 Q9P2K8 VAR_040483 p.His939Tyr US - A lung neuroendocrine carcinoma sample
EIF2AK4 Q9P2K8 VAR_040484 p.Thr1060Arg LB/B rs55781333 -
EIF2AK4 Q9P2K8 VAR_040485 p.Gly1306Cys LB/B rs35602605 -
EIF2AK4 Q9P2K8 VAR_040486 p.Lys1336Arg LB/B rs35480871 -

EIF2AK4 Q9P2K8 VAR_040487 p.Gln1406His LB/B rs55721315 -
EIF2AK4 Q9P2K8 VAR_070990 p.Arg585Gln LP/P rs587777106 Pulmonary venoocclusive disease 2, autosomal recessive (PVOD2) [MIM:234810]
EIF2AK4 Q9P2K8 VAR_070991 p.Leu643Arg LP/P rs757852728 Pulmonary venoocclusive disease 2, autosomal recessive (PVOD2) [MIM:234810]
EIF2B1 Q14232 VAR_015404 p.Asn208Tyr LP/P rs113994007 Leukodystrophy with vanishing white matter (VWM) [MIM:603896]
EIF2B1 Q14232 VAR_068450 p.Val183Phe LP/P rs863225048 Leukodystrophy with vanishing white matter (VWM) [MIM:603896]
EIF2B2 P49770 VAR_012289 p.Glu213Gly LP/P rs104894425 Leukodystrophy with vanishing white matter (VWM) [MIM:603896]
EIF2B2 P49770 VAR_012290 p.Val316Asp LP/P rs104894426 Leukodystrophy with vanishing white matter (VWM) [MIM:603896]
EIF2B2 P49770 VAR_012321 p.Lys273Arg LP/P rs113994016 Leukodystrophy with vanishing white matter (VWM) [MIM:603896]
EIF2B2 P49770 VAR_012322 p.Gly329Val LP/P rs113994020 Leukodystrophy with vanishing white matter (VWM) [MIM:603896]
EIF2B2 P49770 VAR_016842 p.Ser171Phe LP/P rs104894428 Leukodystrophy with vanishing white matter (VWM) [MIM:603896]
EIF2B2 P49770 VAR_068451 p.Val85Glu LP/P rs397514648 Leukodystrophy with vanishing white matter (VWM) [MIM:603896]
EIF2B2 P49770 VAR_068452 p.Pro196Ser LP/P rs113994011 Leukodystrophy with vanishing white matter (VWM) [MIM:603896]
EIF2B2 P49770 VAR_068453 p.Gly200Val LP/P rs113994012 Leukodystrophy with vanishing white matter (VWM) [MIM:603896]
EIF2B2 P49770 VAR_068454 p.Cys268Tyr LP/P - Leukodystrophy with vanishing white matter (VWM) [MIM:603896]
EIF2B2 P49770 VAR_079034 p.Ala127Val US rs150617429 -
EIF2B3 Q9NR50 VAR_015409 p.Ala87Val LP/P rs113994022 Leukodystrophy with vanishing white matter (VWM) [MIM:603896]
EIF2B3 Q9NR50 VAR_015410 p.Arg225Gln LP/P rs113994024 Leukodystrophy with vanishing white matter (VWM) [MIM:603896]
EIF2B3 Q9NR50 VAR_048920 p.Asp288Glu LB/B rs3738247 -
EIF2B3 Q9NR50 VAR_068470 p.Leu27Gln LP/P rs397514647 Leukodystrophy with vanishing white matter (VWM) [MIM:603896]
EIF2B3 Q9NR50 VAR_068471 p.Gly47Glu LP/P - Leukodystrophy with vanishing white matter (VWM) [MIM:603896]
EIF2B3 Q9NR50 VAR_068472 p.Ile346Thr LP/P rs119474039 Leukodystrophy with vanishing white matter (VWM) [MIM:603896]
EIF2B4 Q9UI10 VAR_015405 p.Ala228Val LP/P rs113994027 Leukodystrophy with vanishing white matter (VWM) [MIM:603896]
EIF2B4 Q9UI10 VAR_015406 p.Arg306Gly LB/B rs78599355 -
EIF2B4 Q9UI10 VAR_015407 p.Arg357Gln LP/P rs113994033 Leukodystrophy with vanishing white matter (VWM) [MIM:603896]
EIF2B4 Q9UI10 VAR_015408 p.Arg374Cys LP/P rs113994035 Leukodystrophy with vanishing white matter (VWM) [MIM:603896]
EIF2B4 Q9UI10 VAR_016843 p.Cys465Arg LP/P rs113994038 Leukodystrophy with vanishing white matter (VWM) [MIM:603896]

EIF2B4 Q9UI10 VAR_016844 p.Tyr489His LP/P rs113994040 Leukodystrophy with vanishing white matter (VWM) [MIM:603896]
EIF2B4 Q9UI10 VAR_048918 p.Ala93Val LB/B rs34155621 -
EIF2B4 Q9UI10 VAR_068455 p.Arg209Gln LP/P rs113994028 Leukodystrophy with vanishing white matter (VWM) [MIM:603896]
EIF2B4 Q9UI10 VAR_068456 p.Leu269Arg LP/P rs113994031 Leukodystrophy with vanishing white matter (VWM) [MIM:603896]
EIF2B5 Q13144 VAR_012291 p.Thr91Ala LP/P rs28939717 Leukodystrophy with vanishing white matter (VWM) [MIM:603896]
EIF2B5 Q13144 VAR_012292 p.Arg113His LP/P rs113994049 Leukodystrophy with vanishing white matter (VWM) [MIM:603896]
EIF2B5 Q13144 VAR_012293 p.Gly386Val LP/P rs113994074 Leukodystrophy with vanishing white matter (VWM) [MIM:603896]
EIF2B5 Q13144 VAR_012294 p.Trp628Arg LP/P rs28937596 Leukodystrophy with vanishing white matter (VWM) [MIM:603896]
EIF2B5 Q13144 VAR_012323 p.Val73Gly LP/P rs113994045 Leukodystrophy with vanishing white matter (VWM) [MIM:603896]
EIF2B5 Q13144 VAR_012324 p.Leu106Phe LP/P rs113994048 Leukodystrophy with vanishing white matter (VWM) [MIM:603896]
EIF2B5 Q13144 VAR_012325 p.Arg299His LP/P rs113994060 Leukodystrophy with vanishing white matter (VWM) [MIM:603896]
EIF2B5 Q13144 VAR_012326 p.Arg315Gly LP/P rs113994063 Leukodystrophy with vanishing white matter (VWM) [MIM:603896]
EIF2B5 Q13144 VAR_012327 p.Arg315His LP/P rs113994064 Leukodystrophy with vanishing white matter (VWM) [MIM:603896]
EIF2B5 Q13144 VAR_012328 p.Arg339Pro LP/P rs113994069 Leukodystrophy with vanishing white matter (VWM) [MIM:603896]
EIF2B5 Q13144 VAR_012329 p.Arg339Gln LP/P rs113994069 Leukodystrophy with vanishing white matter (VWM) [MIM:603896]
EIF2B5 Q13144 VAR_012330 p.Arg339Trp LP/P rs113994068 Leukodystrophy with vanishing white matter (VWM) [MIM:603896]
EIF2B5 Q13144 VAR_012331 p.Val430Ala LP/P rs113994079 Leukodystrophy with vanishing white matter (VWM) [MIM:603896]
EIF2B5 Q13144 VAR_012332 p.Ile587Val LB/B rs843358 -
EIF2B5 Q13144 VAR_012333 p.Glu650Lys LP/P rs113994085 Leukodystrophy with vanishing white matter (VWM) [MIM:603896]
EIF2B5 Q13144 VAR_016845 p.Arg195Cys LP/P rs113994055 Leukodystrophy with vanishing white matter (VWM) [MIM:603896]
EIF2B5 Q13144 VAR_016846 p.Arg195His LP/P rs113994054 Leukodystrophy with vanishing white matter (VWM) [MIM:603896]
EIF2B5 Q13144 VAR_048919 p.Asn200Thr LB/B rs2971409 -
EIF2B5 Q13144 VAR_068457 p.Asp62Val LP/P rs1560105986 Leukodystrophy with vanishing white matter (VWM) [MIM:603896]
EIF2B5 Q13144 VAR_068458 p.Leu68Ser LP/P rs113994044 Leukodystrophy with vanishing white matter (VWM) [MIM:603896]
EIF2B5 Q13144 VAR_068459 p.Ala74Thr LP/P rs113994046 Leukodystrophy with vanishing white matter (VWM) [MIM:603896]
EIF2B5 Q13144 VAR_068460 p.Arg113Cys LP/P rs113994050 Leukodystrophy with vanishing white matter (VWM) [MIM:603896]

EIF2B5 Q13144 VAR_068461 p.Arg269Gly LP/P rs113994058 Leukodystrophy with vanishing white matter (VWM) [MIM:603896]
EIF2B5 Q13144 VAR_068462 p.Arg269Gln LP/P rs113994057 Leukodystrophy with vanishing white matter (VWM) [MIM:603896]
EIF2B5 Q13144 VAR_068463 p.Asp270His LP/P rs397514646 Leukodystrophy with vanishing white matter (VWM) [MIM:603896]
EIF2B5 Q13144 VAR_068464 p.Cys310Phe LP/P rs113994062 Leukodystrophy with vanishing white matter (VWM) [MIM:603896]
EIF2B5 Q13144 VAR_068465 p.Arg315Cys LP/P rs113994063 Leukodystrophy with vanishing white matter (VWM) [MIM:603896]
EIF2B5 Q13144 VAR_068466 p.Cys335Arg LP/P rs113994067 Leukodystrophy with vanishing white matter (VWM) [MIM:603896]
EIF2B5 Q13144 VAR_068467 p.Cys335Ser LP/P - Leukodystrophy with vanishing white matter (VWM) [MIM:603896]
EIF2B5 Q13144 VAR_068468 p.Asn376Asp LP/P - Leukodystrophy with vanishing white matter (VWM) [MIM:603896]
EIF2B5 Q13144 VAR_068469 p.Ser447Leu LP/P rs113994080 Leukodystrophy with vanishing white matter (VWM) [MIM:603896]
EIF2D P41214 VAR_052507 p.Thr210Ile LB/B rs35252702 -
EIF2S2 P20042 VAR_048909 p.Glu177Asp LB/B rs17856024 -
EIF2S3 P41091 VAR_002352 p.Lys125Arg LB/B rs16997659 -
EIF2S3 P41091 VAR_077139 p.Ile222Thr LP/P rs886040855 MEHMO syndrome (MEHMO) [MIM:300148]
EIF2S3 P41091 VAR_077140 p.Ile259Met US rs886040856 MEHMO syndrome (MEHMO) [MIM:300148]
EIF2S3 P41091 VAR_078100 p.Ser108Arg US rs1057515578 MEHMO syndrome (MEHMO) [MIM:300148]
EIF3A Q14152 VAR_024438 p.Glu386Lys LB/B rs967185 -
EIF3A Q14152 VAR_048921 p.Lys694Asn LB/B rs431898 -
EIF3A Q14152 VAR_048922 p.Asp993Glu LB/B rs532138 -
EIF3B P55884 VAR_047972 p.Ser64Pro LB/B rs9690787 -
EIF3B P55884 VAR_047973 p.Asp793Glu LB/B rs1063257 -
EIF3D O15371 VAR_074184 p.Arg310Cys LB/B rs745920273 -
EIF3E P60228 VAR_046480 p.Ala185Val LB/B rs17856554 -
EIF3F O00303 VAR_014452 p.Trp172Leu LB/B rs1044058 -
EIF3F O00303 VAR_029267 p.Pro39Leu LB/B rs1043738 -
EIF3F O00303 VAR_081783 p.Phe232Val LP/P rs141976414 Intellectual developmental disorder, autosomal recessive 67 (MRT67) [MIM:618295]
EIF3J O75822 VAR_034007 p.Ala141Thr LB/B rs2303578 -
EIF3M Q7L2H7 VAR_036752 p.Gly37Arg LB/B rs11557143 -
EIF3M Q7L2H7 VAR_036753 p.Glu80Gly US - A breast cancer sample
EIF3M Q7L2H7 VAR_036754 p.Gln346Arg LB/B rs1802363 -
EIF4A2 Q14240 VAR_035838 p.Val181Leu US - A breast cancer sample
EIF4A2 Q14240 VAR_052158 p.Gln93His LB/B rs11538616 -
EIF4A3 P38919 VAR_071090 p.Asp270Gly LP/P rs587777204 Richieri-Costa-Pereira syndrome (RCPS) [MIM:268305]
EIF4B P23588 VAR_064710 p.Pro203Arg US - -
EIF4E1B A6NMX2 VAR_044209 p.Asp227Tyr LB/B rs13163938 -
EIF4G1 Q04637 VAR_036117 p.Pro696Leu US rs754755344 A colorectal cancer sample
EIF4G1 Q04637 VAR_055704 p.Tyr311Cys LB/B rs16858632 -
EIF4G1 Q04637 VAR_055705 p.Leu1233Pro LB/B rs2230570 -
EIF4G1 Q04637 VAR_061147 p.Thr161Ala LB/B rs13319149 -

EIF4G1 Q04637 VAR_061148 p.Pro1229Ala LB/B rs35629949 -
EIF4G1 Q04637 VAR_063040 p.Met432Val LB/B rs2178403 -
EIF4G1 Q04637 VAR_066571 p.Pro71Ser LB/B rs113810947 -
EIF4G1 Q04637 VAR_066573 p.Ala502Val LP/P rs111290936 Parkinson disease 18 (PARK18) [MIM:614251]
EIF4G1 Q04637 VAR_066574 p.Gly686Cys US rs112019125 -
EIF4G1 Q04637 VAR_066575 p.Ile806Val LB/B rs62287499 -
EIF4G1 Q04637 VAR_066576 p.Thr829Ser LB/B rs111500185 -
EIF4G1 Q04637 VAR_066577 p.Ser1164Arg US rs113169049 -
EIF4G1 Q04637 VAR_066578 p.Arg1197Trp US rs113388242 -
EIF4G1 Q04637 VAR_066579 p.Arg1205His LP/P rs112176450 Parkinson disease 18 (PARK18) [MIM:614251]
EIF4G1 Q04637 VAR_066580 p.Asn1257Ser LB/B rs73053766 -
EIF4G1 Q04637 VAR_079031 p.Arg201His US rs34838305 -
EIF4G2 P78344 VAR_048923 p.Leu236Met LB/B rs34885591 -
EIF4G3 O43432 VAR_034009 p.Pro496Ala LB/B rs35176330 -
EIF4G3 O43432 VAR_048924 p.Gln378Arg LB/B rs35731992 -
EIF4G3 O43432 VAR_048925 p.Asp1185Glu LB/B rs2230572 -
EIF5 P55010 VAR_036467 p.Lys418Met US - A breast cancer sample
EIF5A2 Q9GZV4 VAR_027943 p.Glu42Asp LB/B rs776545602 -
EIF5B O60841 VAR_055954 p.Ser337Gly LB/B rs10642 -
EIF5B O60841 VAR_055955 p.Arg360Gly LB/B rs3205296 -
EIF5B O60841 VAR_060587 p.Lys522Thr LB/B rs7558074 -
ELAC1 Q9H777 VAR_017424 p.Met355Val LB/B rs34524743 -
ELAC2 Q9BQ52 VAR_017425 p.Arg211Gln LP/P rs148419785 Prostate cancer, hereditary, 2 (HPC2) [MIM:614731]
ELAC2 Q9BQ52 VAR_017426 p.Ser217Leu LP/P rs4792311 Prostate cancer, hereditary, 2 (HPC2) [MIM:614731]
ELAC2 Q9BQ52 VAR_017427 p.Gly487Arg LP/P rs752234492 Prostate cancer, hereditary, 2 (HPC2) [MIM:614731]
ELAC2 Q9BQ52 VAR_017428 p.Ala541Thr LP/P rs5030739 Prostate cancer, hereditary, 2 (HPC2) [MIM:614731]
ELAC2 Q9BQ52 VAR_017429 p.Glu622Val LP/P rs119484087 Prostate cancer, hereditary, 2 (HPC2) [MIM:614731]
ELAC2 Q9BQ52 VAR_017430 p.Ser627Leu LB/B rs78105154 -
ELAC2 Q9BQ52 VAR_017431 p.Arg781His LP/P rs119484086 Prostate cancer, hereditary, 2 (HPC2) [MIM:614731]
ELAC2 Q9BQ52 VAR_017432 p.Gly806Arg LP/P rs770669443 Prostate cancer, hereditary, 2 (HPC2) [MIM:614731]
ELAC2 Q9BQ52 VAR_038210 p.Ser52Phe LB/B rs9895963 -
ELAC2 Q9BQ52 VAR_038211 p.Asp436Asn LB/B rs3760317 -
ELAC2 Q9BQ52 VAR_070844 p.Phe154Leu LP/P rs397515465 Combined oxidative phosphorylation deficiency 17 (COXPD17) [MIM:615440]
ELAC2 Q9BQ52 VAR_070845 p.Leu423Phe LP/P rs397515466 Combined oxidative phosphorylation deficiency 17 (COXPD17) [MIM:615440]
ELAC2 Q9BQ52 VAR_070846 p.Thr520Ile LP/P rs397515463 Combined oxidative phosphorylation deficiency 17 (COXPD17) [MIM:615440]
ELANE P08246 VAR_019237 p.Val219Ile LB/B rs17216656 -
ELANE P08246 VAR_019238 p.Pro257Leu LB/B rs17216663 -
ELANE P08246 VAR_019239 p.Pro262Leu LB/B rs17216670 -

ELANE P08246 VAR_038609 p.Cys55Tyr LP/P - Neutropenia, severe congenital 1, autosomal dominant (SCN1) [MIM:202700]
ELANE P08246 VAR_038610 p.Ala57Thr LP/P - Neutropenia, severe congenital 1, autosomal dominant (SCN1) [MIM:202700]
ELANE P08246 VAR_038611 p.Ile60Thr LP/P - Neutropenia, severe congenital 1, autosomal dominant (SCN1) [MIM:202700]
ELANE P08246 VAR_038612 p.Cys71Arg LP/P rs28931611 Neutropenia, severe congenital 1, autosomal dominant (SCN1) [MIM:202700]
ELANE P08246 VAR_038613 p.Cys71Ser LP/P - Neutropenia, severe congenital 1, autosomal dominant (SCN1) [MIM:202700]
ELANE P08246 VAR_038614 p.Gly85Glu LP/P - Neutropenia, severe congenital 1, autosomal dominant (SCN1) [MIM:202700]
ELANE P08246 VAR_038615 p.Val98Leu LP/P rs267606781 Neutropenia, severe congenital 1, autosomal dominant (SCN1) [MIM:202700]
ELANE P08246 VAR_038616 p.Val101Leu LP/P rs137854449 Neutropenia, severe congenital 1, autosomal dominant (SCN1) [MIM:202700]
ELANE P08246 VAR_038617 p.Val101Met LP/P rs137854449 Neutropenia, severe congenital 1, autosomal dominant (SCN1) [MIM:202700]
ELANE P08246 VAR_038619 p.Ser126Leu LP/P rs137854450 Cyclic haematopoiesis (CH) [MIM:162800]
ELANE P08246 VAR_038619 p.Ser126Leu LP/P rs137854450 Neutropenia, severe congenital 1, autosomal dominant (SCN1) [MIM:202700]
ELANE P08246 VAR_038620 p.Pro139Leu LP/P rs137854448 Cyclic haematopoiesis (CH) [MIM:162800]
ELANE P08246 VAR_038620 p.Pro139Leu LP/P rs137854448 Neutropenia, severe congenital 1, autosomal dominant (SCN1) [MIM:202700]
ELANE P08246 VAR_038621 p.Cys151Ser LP/P - Neutropenia, severe congenital 1, autosomal dominant (SCN1) [MIM:202700]
ELANE P08246 VAR_038623 p.Pro205Arg LP/P rs1555710077 Neutropenia, severe congenital 1, autosomal dominant (SCN1) [MIM:202700]
ELANE P08246 VAR_038624 p.Gly210Val LP/P - Neutropenia, severe congenital 1, autosomal dominant (SCN1) [MIM:202700]
ELANE P08246 VAR_038625 p.Gly214Arg LP/P rs137854451 Neutropenia, severe congenital 1, autosomal dominant (SCN1) [MIM:202700]
ELANE P08246 VAR_064512 p.Ala25Val LP/P rs1396230082 Neutropenia, severe congenital 1, autosomal dominant (SCN1) [MIM:202700]
ELANE P08246 VAR_064513 p.Ala166Thr LP/P rs201788817 Neutropenia, severe congenital 1, autosomal dominant (SCN1) [MIM:202700]
ELANE P08246 VAR_070696 p.Pro42Leu LP/P - Neutropenia, severe congenital 1, autosomal dominant (SCN1) [MIM:202700]
ELANE P08246 VAR_070697 p.Phe43Leu LP/P - Cyclic haematopoiesis (CH) [MIM:162800]
ELANE P08246 VAR_070697 p.Phe43Leu LP/P - Neutropenia, severe congenital 1, autosomal dominant (SCN1) [MIM:202700]
ELANE P08246 VAR_070698 p.Met44Arg LP/P - Neutropenia, severe congenital 1, autosomal dominant (SCN1) [MIM:202700]
ELANE P08246 VAR_070699 p.Val45Glu LP/P - Neutropenia, severe congenital 1, autosomal dominant (SCN1) [MIM:202700]
ELANE P08246 VAR_070700 p.Val45Leu LP/P - Cyclic haematopoiesis (CH) [MIM:162800]
ELANE P08246 VAR_070701 p.Ser46Cys LP/P - Neutropenia, severe congenital 1, autosomal dominant (SCN1) [MIM:202700]
ELANE P08246 VAR_070702 p.Ser46Phe LP/P rs878855320 Cyclic haematopoiesis (CH) [MIM:162800]

ELANE P08246 VAR_070702 p.Ser46Phe LP/P rs878855320 Neutropenia, severe congenital 1, autosomal dominant (SCN1) [MIM:202700]
ELANE P08246 VAR_070703 p.Leu47Pro LP/P rs878855319 Neutropenia, severe congenital 1, autosomal dominant (SCN1) [MIM:202700]
ELANE P08246 VAR_070704 p.Leu47Arg LP/P - Neutropenia, severe congenital 1, autosomal dominant (SCN1) [MIM:202700]
ELANE P08246 VAR_070705 p.Leu49Pro LP/P - Neutropenia, severe congenital 1, autosomal dominant (SCN1) [MIM:202700]
ELANE P08246 VAR_070706 p.His53Leu LP/P - Neutropenia, severe congenital 1, autosomal dominant (SCN1) [MIM:202700]
ELANE P08246 VAR_070707 p.His53Gln LP/P - Cyclic haematopoiesis (CH) [MIM:162800]
ELANE P08246 VAR_070708 p.His53Tyr LP/P rs1131691882 Neutropenia, severe congenital 1, autosomal dominant (SCN1) [MIM:202700]
ELANE P08246 VAR_070709 p.Cys55Ser LP/P - Neutropenia, severe congenital 1, autosomal dominant (SCN1) [MIM:202700]
ELANE P08246 VAR_070710 p.Gly56Arg LP/P - Neutropenia, severe congenital 1, autosomal dominant (SCN1) [MIM:202700]
ELANE P08246 VAR_070711 p.Ala57Ser LP/P - Neutropenia, severe congenital 1, autosomal dominant (SCN1) [MIM:202700]
ELANE P08246 VAR_070712 p.Ala57Val LP/P rs1057520110 Neutropenia, severe congenital 1, autosomal dominant (SCN1) [MIM:202700]
ELANE P08246 VAR_070713 p.Leu59Pro LP/P - Neutropenia, severe congenital 1, autosomal dominant (SCN1) [MIM:202700]
ELANE P08246 VAR_070715 p.Ala61Val LP/P rs137854447 Cyclic haematopoiesis (CH) [MIM:162800]
ELANE P08246 VAR_070715 p.Ala61Val LP/P rs137854447 Neutropenia, severe congenital 1, autosomal dominant (SCN1) [MIM:202700]
ELANE P08246 VAR_070718 p.Ser67Trp LP/P - Neutropenia, severe congenital 1, autosomal dominant (SCN1) [MIM:202700]
ELANE P08246 VAR_070719 p.Cys71Phe LP/P rs878855315 Neutropenia, severe congenital 1, autosomal dominant (SCN1) [MIM:202700]
ELANE P08246 VAR_070720 p.Cys71Tyr LP/P - Neutropenia, severe congenital 1, autosomal dominant (SCN1) [MIM:202700]
ELANE P08246 VAR_070721 p.Val72Gly LP/P - Neutropenia, severe congenital 1, autosomal dominant (SCN1) [MIM:202700]
ELANE P08246 VAR_070722 p.Val80Gly LP/P - Neutropenia, severe congenital 1, autosomal dominant (SCN1) [MIM:202700]
ELANE P08246 VAR_070723 p.Arg81Pro LP/P - Cyclic haematopoiesis (CH) [MIM:162800]
ELANE P08246 VAR_070723 p.Arg81Pro LP/P - Neutropenia, severe congenital 1, autosomal dominant (SCN1) [MIM:202700]
ELANE P08246 VAR_070724 p.Val82Met LP/P - Cyclic haematopoiesis (CH) [MIM:162800]
ELANE P08246 VAR_070724 p.Val82Met LP/P - Neutropenia, severe congenital 1, autosomal dominant (SCN1) [MIM:202700]
ELANE P08246 VAR_070725 p.Leu84Pro LP/P rs1064793108 Neutropenia, severe congenital 1, autosomal dominant (SCN1) [MIM:202700]
ELANE P08246 VAR_070726 p.Gly85Arg LP/P - Neutropenia, severe congenital 1, autosomal dominant (SCN1) [MIM:202700]
ELANE P08246 VAR_070727 p.Gln97Leu LP/P - Cyclic haematopoiesis (CH) [MIM:162800]
ELANE P08246 VAR_070728 p.Val98Met LP/P rs267606781 Neutropenia, severe congenital 1, autosomal dominant (SCN1) [MIM:202700]

ELANE P08246 VAR_070729 p.Arg103Leu LP/P rs745455816 Neutropenia, severe congenital 1, autosomal dominant (SCN1) [MIM:202700]
ELANE P08246 VAR_070730 p.Arg103Pro LP/P - Neutropenia, severe congenital 1, autosomal dominant (SCN1) [MIM:202700]
ELANE P08246 VAR_070731 p.Ile104Asn LP/P - Cyclic haematopoiesis (CH) [MIM:162800]
ELANE P08246 VAR_070732 p.Ile118Val LB/B rs1382122842 -
ELANE P08246 VAR_070733 p.Ile120Phe LP/P rs1131691520 Cyclic haematopoiesis (CH) [MIM:162800]
ELANE P08246 VAR_070733 p.Ile120Phe LP/P rs1131691520 Neutropenia, severe congenital 1, autosomal dominant (SCN1) [MIM:202700]
ELANE P08246 VAR_070734 p.Ile120Asn LP/P - Neutropenia, severe congenital 1, autosomal dominant (SCN1) [MIM:202700]
ELANE P08246 VAR_070735 p.Ile120Ser LP/P - Neutropenia, severe congenital 1, autosomal dominant (SCN1) [MIM:202700]
ELANE P08246 VAR_070736 p.Leu121Pro LP/P - Neutropenia, severe congenital 1, autosomal dominant (SCN1) [MIM:202700]
ELANE P08246 VAR_070737 p.Leu123His LP/P - Neutropenia, severe congenital 1, autosomal dominant (SCN1) [MIM:202700]
ELANE P08246 VAR_070738 p.Asn124Ile LP/P - Neutropenia, severe congenital 1, autosomal dominant (SCN1) [MIM:202700]
ELANE P08246 VAR_070739 p.Gly125Arg LB/B rs377698556 -
ELANE P08246 VAR_070740 p.Ala127Asp LP/P - Neutropenia, severe congenital 1, autosomal dominant (SCN1) [MIM:202700]
ELANE P08246 VAR_070741 p.Ala127Pro LP/P - Neutropenia, severe congenital 1, autosomal dominant (SCN1) [MIM:202700]
ELANE P08246 VAR_070742 p.Ala131Thr US rs201729066 Neutropenia, severe congenital 1, autosomal dominant (SCN1) [MIM:202700]
ELANE P08246 VAR_070743 p.Val135Met LB/B rs774457980 -
ELANE P08246 VAR_070744 p.Ala136Asp LP/P - Neutropenia, severe congenital 1, autosomal dominant (SCN1) [MIM:202700]
ELANE P08246 VAR_070745 p.Pro139Arg LP/P - Neutropenia, severe congenital 1, autosomal dominant (SCN1) [MIM:202700]
ELANE P08246 VAR_070746 p.Arg143His US rs200993994 Cyclic haematopoiesis (CH) [MIM:162800]
ELANE P08246 VAR_070747 p.Cys151Phe LP/P rs57246956 Neutropenia, severe congenital 1, autosomal dominant (SCN1) [MIM:202700]
ELANE P08246 VAR_070748 p.Cys151Trp LP/P - Neutropenia, severe congenital 1, autosomal dominant (SCN1) [MIM:202700]
ELANE P08246 VAR_070749 p.Cys151Tyr US rs57246956 Neutropenia, severe congenital 1, autosomal dominant (SCN1) [MIM:202700]
ELANE P08246 VAR_070750 p.Leu152Pro LP/P - Neutropenia, severe congenital 1, autosomal dominant (SCN1) [MIM:202700]
ELANE P08246 VAR_070751 p.Ala153Asp LP/P - Neutropenia, severe congenital 1, autosomal dominant (SCN1) [MIM:202700]
ELANE P08246 VAR_070752 p.Ala153Pro LP/P - Neutropenia, severe congenital 1, autosomal dominant (SCN1) [MIM:202700]
ELANE P08246 VAR_070753 p.Trp156Cys LP/P - Neutropenia, severe congenital 1, autosomal dominant (SCN1) [MIM:202700]
ELANE P08246 VAR_070754 p.Trp156Arg LP/P - Neutropenia, severe congenital 1, autosomal dominant (SCN1) [MIM:202700]
ELANE P08246 VAR_070755 p.Gly203Cys LP/P - Cyclic haematopoiesis (CH) [MIM:162800]

ELANE P08246 VAR_070755 p.Gly203Cys LP/P - Neutropenia, severe congenital 1, autosomal dominant (SCN1) [MIM:202700]
ELANE P08246 VAR_070756 p.Gly203Arg LP/P rs201139487 Neutropenia, severe congenital 1, autosomal dominant (SCN1) [MIM:202700]
ELANE P08246 VAR_070757 p.Leu206Phe LP/P rs137854446 Cyclic haematopoiesis (CH) [MIM:162800]
ELANE P08246 VAR_070758 p.Leu206Ser LP/P - Neutropenia, severe congenital 1, autosomal dominant (SCN1) [MIM:202700]
ELANE P08246 VAR_070759 p.Cys208Gly LP/P - Neutropenia, severe congenital 1, autosomal dominant (SCN1) [MIM:202700]
ELANE P08246 VAR_070760 p.Asn209Ile LP/P - Cyclic haematopoiesis (CH) [MIM:162800]
ELANE P08246 VAR_070761 p.Gly210Trp LP/P - Cyclic haematopoiesis (CH) [MIM:162800]
ELANE P08246 VAR_070762 p.Gly214Glu LP/P - Neutropenia, severe congenital 1, autosomal dominant (SCN1) [MIM:202700]
ELANE P08246 VAR_070763 p.Arg220Gln LP/P rs137854445 Cyclic haematopoiesis (CH) [MIM:162800]
ELANE P08246 VAR_070763 p.Arg220Gln LP/P rs137854445 Neutropenia, severe congenital 1, autosomal dominant (SCN1) [MIM:202700]
ELANE P08246 VAR_070764 p.Ala233Pro LP/P - Neutropenia, severe congenital 1, autosomal dominant (SCN1) [MIM:202700]
ELANE P08246 VAR_070765 p.Val235Glu LP/P - Neutropenia, severe congenital 1, autosomal dominant (SCN1) [MIM:202700]
ELANE P08246 VAR_070766 p.Val235Gly LP/P - Neutropenia, severe congenital 1, autosomal dominant (SCN1) [MIM:202700]
ELAPOR1 Q6UXG2 VAR_032138 p.Ile86Val LB/B rs678238 -
ELAPOR1 Q6UXG2 VAR_032139 p.Thr623Pro LB/B rs659543 -
ELAPOR1 Q6UXG2 VAR_032140 p.Leu1009Pro LB/B rs1052878 -
ELAPOR1 Q6UXG2 VAR_035751 p.Ser829Arg US - A breast cancer sample
ELAPOR2 A8MWY0 VAR_043161 p.Asn539Tyr LB/B rs1029366 -
ELAPOR2 A8MWY0 VAR_043162 p.Leu729Val LB/B rs34412146 -
ELAPOR2 A8MWY0 VAR_043163 p.Ser767Arg LB/B rs34577440 -
ELAVL4 P26378 VAR_052204 p.Pro275Ser LB/B rs2494876 -
ELAVL4 P26378 VAR_058091 p.Asp171Gly LB/B rs17853533 -
ELAVL4 P26378 VAR_058092 p.Ala361Thr LB/B rs17853531 -
ELF1 P32519 VAR_048942 p.Asn58Ser LB/B rs7799 -
ELF1 P32519 VAR_048943 p.Thr343Ser LB/B rs1056820 -
ELF1 P32519 VAR_048944 p.Thr403Ile LB/B rs7323148 -
ELF3 P78545 VAR_048945 p.Gln317Lys LB/B rs1135542 -
ELK1 P19419 VAR_017108 p.Gly144Ser LB/B rs1997639 -
ELK1 P19419 VAR_017109 p.Ser183Asn LB/B rs1059579 -
ELK3 P41970 VAR_048946 p.Pro169Leu LB/B rs35332676 -
ELL P55199 VAR_053072 p.Ser297Asn LB/B rs2303694 -
ELL P55199 VAR_053073 p.Arg387Trp LB/B rs35245196 -
ELL2 O00472 VAR_058406 p.Ala298Thr LB/B rs3815768 -
ELL3 Q9HB65 VAR_018992 p.Gln11Glu LB/B rs2277531 -
ELL3 Q9HB65 VAR_053074 p.Trp140Arg LB/B rs35454865 -
ELMO1 Q92556 VAR_065824 p.Ile362Ser LB/B - -
ELMO2 Q96JJ3 VAR_048928 p.Glu695Asp LB/B rs34630674 -
ELMO3 Q96BJ8 VAR_055402 p.Lys13Gln LB/B rs12923138 -
ELMO3 Q96BJ8 VAR_055403 p.Asn95Asp LB/B rs8058861 -
ELMO3 Q96BJ8 VAR_055404 p.Arg316Cys LB/B rs33948247 -

ELMOD3 Q96FG2 VAR_030362 p.Arg66Cys LB/B rs7564372 -
ELMOD3 Q96FG2 VAR_030363 p.Thr70Ile LB/B rs955592 -
ELMOD3 Q96FG2 VAR_070125 p.Leu265Ser LP/P rs587777040 Deafness, autosomal recessive, 88 (DFNB88) [MIM:615429]
ELN P15502 VAR_020882 p.Gly422Ser LB/B rs2071307 -
ELN P15502 VAR_056869 p.Gly610Arg LB/B rs17855988 -
ELN P15502 VAR_072395 p.Pro211Ser US rs1064793880 -
ELOA Q14241 VAR_020104 p.Thr145Met LB/B rs2235541 -
ELOA Q14241 VAR_033850 p.Val324Ile LB/B rs520713 -
ELOA Q14241 VAR_033851 p.Ala516Val LB/B rs550252 -
ELOA2 Q8IYF1 VAR_035914 p.Arg498Gln US rs138369139 A colorectal cancer sample
ELOA2 Q8IYF1 VAR_050965 p.Arg179Pro LB/B rs2571028 -
ELOA2 Q8IYF1 VAR_050966 p.Cys254Phe LB/B rs2010834 -
ELOA2 Q8IYF1 VAR_050967 p.Ala403Ser LB/B rs892586 -
ELOA2 Q8IYF1 VAR_061646 p.Ala446Thr LB/B rs3744863 -
ELOA3DP A6NLF2 VAR_044378 p.Pro375Leu LB/B rs2261291 -
ELOA3P Q8NG57 VAR_059654 p.Leu375Pro LB/B rs879354865 -
ELOVL1 Q9BW60 VAR_083193 p.Ser165Phe LP/P - Ichthyotic keratoderma, spasticity, hypomyelination, and dysmorphic facies (IKSHD) [MIM:618527]
ELOVL2 Q9NXB9 VAR_039039 p.Thr216Ala LB/B rs17855038 -
ELOVL2 Q9NXB9 VAR_039040 p.Val225Met LB/B rs6919726 -
ELOVL4 Q9GZR5 VAR_012492 p.Met299Val LB/B rs3812153 -
ELOVL4 Q9GZR5 VAR_017043 p.Ile267Thr LB/B rs148594713 -
ELOVL4 Q9GZR5 VAR_072565 p.Leu168Phe LP/P rs587777598 Spinocerebellar ataxia 34 (SCA34) [MIM:133190]
ELOVL5 Q9NYP7 VAR_072361 p.Leu72Val LP/P rs587777671 Spinocerebellar ataxia 38 (SCA38) [MIM:615957]
ELOVL5 Q9NYP7 VAR_072362 p.Gly230Val LP/P rs587777670 Spinocerebellar ataxia 38 (SCA38) [MIM:615957]
ELP1 O95163 VAR_011327 p.Arg696Pro LP/P rs137853022 Neuropathy, hereditary sensory and autonomic, 3 (HSAN3) [MIM:223900]
ELP1 O95163 VAR_047476 p.Arg70Cys LB/B rs3737311 -
ELP1 O95163 VAR_047477 p.Met182Lys LB/B rs10521092 -
ELP1 O95163 VAR_047478 p.Glu312Lys LB/B rs1140064 -
ELP1 O95163 VAR_047479 p.Arg525Gln LB/B rs838827 -
ELP1 O95163 VAR_047480 p.Gly765Glu LB/B rs2230792 -
ELP1 O95163 VAR_047481 p.Ile816Leu LB/B rs2230793 -
ELP1 O95163 VAR_047482 p.Ile830Met LB/B rs2230794 -
ELP1 O95163 VAR_047483 p.Thr848Asn LB/B rs10979599 -
ELP1 O95163 VAR_047484 p.Lys952Ile LB/B rs2230798 -
ELP1 O95163 VAR_047485 p.Gly1013Ser LB/B rs2230795 -
ELP1 O95163 VAR_047486 p.Cys1072Ser LB/B rs3204145 -
ELP1 O95163 VAR_047487 p.Pro1158Leu LB/B rs1538660 -
ELP2 Q6IA86 VAR_024072 p.Thr128Pro LB/B rs1785934 -
ELP2 Q6IA86 VAR_024073 p.Val305Met LB/B rs1785928 -
ELP2 Q6IA86 VAR_024074 p.His359Arg LB/B rs16967474 -
ELP2 Q6IA86 VAR_024075 p.Glu795Gly LB/B rs12607773 -
ELP2 Q6IA86 VAR_024076 p.His806Pro LB/B rs1044128 -
ELP2 Q6IA86 VAR_033804 p.Ile541Thr LB/B rs28463092 -
ELP2 Q6IA86 VAR_033805 p.Thr543Pro LB/B rs17563617 -
ELP2 Q6IA86 VAR_033806 p.Thr815Pro LB/B rs1044134 -

ELP2 Q6IA86 VAR_077989 p.His206Arg US rs773432002 Mental retardation, autosomal recessive 58 (MRT58) [MIM:617270]
ELP2 Q6IA86 VAR_077990 p.Arg462Trp US rs767713084 Mental retardation, autosomal recessive 58 (MRT58) [MIM:617270]
ELP4 Q96EB1 VAR_053881 p.Ile300Leu LB/B rs34804357 -
ELP5 Q8TE02 VAR_031205 p.Asp303Tyr LB/B rs17849664 -
ELP5 Q8TE02 VAR_053882 p.Glu14Lys LB/B rs2521988 -
ELSPBP1 Q96BH3 VAR_036760 p.Cys100Trp LB/B rs3745751 -
ELSPBP1 Q96BH3 VAR_036761 p.Asp170Asn LB/B rs35362679 -
ELSPBP1 Q96BH3 VAR_036762 p.Glu199Lys LB/B rs2303690 -
ELSPBP1 Q96BH3 VAR_036763 p.Asp215Asn LB/B rs6509358 -
EMC1 Q8N766 VAR_027359 p.Leu295Ser LB/B rs3850531 -
EMC1 Q8N766 VAR_027360 p.Ser345Thr LB/B rs709683 -
EMC1 Q8N766 VAR_027361 p.Ser347Asn LB/B rs709682 -
EMC1 Q8N766 VAR_076915 p.Thr82Met LP/P rs869320625 Cerebellar atrophy, visual impairment, and psychomotor retardation (CAVIPMR) [MIM:616875]
EMC1 Q8N766 VAR_076916 p.Ala144Thr US rs869320623 -
EMC1 Q8N766 VAR_076917 p.Gly471Arg US rs879253819 Cerebellar atrophy, visual impairment, and psychomotor retardation (CAVIPMR) [MIM:616875]
EMC1 Q8N766 VAR_076918 p.Gly868Arg LP/P rs869320626 Cerebellar atrophy, visual impairment, and psychomotor retardation (CAVIPMR) [MIM:616875]
EMC4 Q5J8M3 VAR_053775 p.Pro98Thr LB/B rs11544437 -
EMC9 Q9Y3B6 VAR_052532 p.Ala97Val LB/B rs11574512 -
EMD P50402 VAR_005198 p.Ser54Phe LP/P - Emery-Dreifuss muscular dystrophy 1, X-linked (EDMD1) [MIM:310300]
EMD P50402 VAR_005199 p.Pro183His LP/P rs104894805 Emery-Dreifuss muscular dystrophy 1, X-linked (EDMD1) [MIM:310300]
EMD P50402 VAR_005200 p.Pro183Thr LP/P rs104894806 Emery-Dreifuss muscular dystrophy 1, X-linked (EDMD1) [MIM:310300]
EMD P50402 VAR_016016 p.Gln133His LP/P - Emery-Dreifuss muscular dystrophy 1, X-linked (EDMD1) [MIM:310300]
EMD P50402 VAR_038433 p.Asp149His LB/B rs2070818 -
EME1 Q96AY2 VAR_025337 p.Ile49Val LB/B rs9896405 -
EME1 Q96AY2 VAR_025338 p.Glu69Asp LB/B rs3760413 -
EME1 Q96AY2 VAR_025339 p.Ile350Thr LB/B rs12450550 -
EME1 Q96AY2 VAR_055708 p.Lys5Asn LB/B rs35248609 -
EME1 Q96AY2 VAR_055709 p.Phe63Leu LB/B rs17714854 -
EME1 Q96AY2 VAR_055710 p.Val347Ile LB/B rs7222520 -
EMG1 Q92979 VAR_050237 p.Ala34Gly LB/B rs11064480 -
EMG1 Q92979 VAR_062480 p.Asp86Gly LP/P rs74435397 Bowen-Conradi syndrome (BWCNS) [MIM:211180]
EMID1 Q96A84 VAR_019803 p.Ala107Gly LB/B rs743920 -
EMILIN1 Q9Y6C2 VAR_046095 p.Arg149Gln LB/B rs2736976 -
EMILIN1 Q9Y6C2 VAR_046096 p.Gln536Arg LB/B rs36069611 -
EMILIN1 Q9Y6C2 VAR_046097 p.Glu903Lys LB/B rs36045790 -
EMILIN1 Q9Y6C2 VAR_077591 p.Ala22Thr US rs753862645 -
EMILIN2 Q9BXX0 VAR_057528 p.Ala215Thr LB/B rs16943977 -
EMILIN2 Q9BXX0 VAR_057529 p.Met259Val LB/B rs35267664 -
EMILIN2 Q9BXX0 VAR_062003 p.Pro903Ser LB/B rs56288451 -
EMILIN3 Q9NT22 VAR_053075 p.Ser532Asn LB/B rs2235592 -

EML1 O00423 VAR_031720 p.Ala377Val LB/B rs34198557 -
EML1 O00423 VAR_031721 p.His552Asn LB/B rs17853154 -
EML1 O00423 VAR_031722 p.Ser556Pro LB/B rs2250718 -
EML1 O00423 VAR_071075 p.Trp225Arg LP/P rs886037937 Band heterotopia (BH) [MIM:600348]
EML1 O00423 VAR_071076 p.Thr243Ala LP/P rs886037936 Band heterotopia (BH) [MIM:600348]
EML2 O95834 VAR_022026 p.Arg357His LB/B rs3816045 -
EML2 O95834 VAR_024697 p.Glu235Asp LB/B rs1545040 -
EML2 O95834 VAR_031723 p.Met33Val LB/B rs12151009 -
EML2 O95834 VAR_031724 p.Leu187Phe LB/B rs7252175 -
EML2 O95834 VAR_035879 p.Val484Leu US rs1270442107 A colorectal cancer sample
EML3 Q32P44 VAR_031725 p.Gln620Lys LB/B rs34098002 -
EML4 Q9HC35 VAR_031726 p.Lys283Glu LB/B rs6736913 -
EML4 Q9HC35 VAR_031727 p.Ile382Val LB/B rs10202624 -
EML4 Q9HC35 VAR_031728 p.Lys398Arg LB/B rs28651764 -
EML4 Q9HC35 VAR_031729 p.Ser978Leu LB/B rs28364731 -
EML5 Q05BV3 VAR_031730 p.Ile269Val LB/B rs17188228 -
EMP1 P54849 VAR_050608 p.Ser57Asn LB/B rs34412222 -
EMP2 P54851 VAR_071478 p.Phe7Leu LP/P rs730882194 Nephrotic syndrome 10 (NPHS10) [MIM:615861]
EMP2 P54851 VAR_071479 p.Ala10Thr LP/P rs587777482 Nephrotic syndrome 10 (NPHS10) [MIM:615861]
EMP3 P54852 VAR_050609 p.Ile125Val LB/B rs4893 -
EN2 P19622 VAR_021985 p.Leu121Phe LB/B rs3735653 -
ENAM Q9NRM1 VAR_020105 p.Pro724Leu LB/B rs3796703 -
ENAM Q9NRM1 VAR_024311 p.Phe576Leu LB/B rs2609428 -
ENAM Q9NRM1 VAR_024312 p.Ile648Thr LB/B rs7671281 -
ENAM Q9NRM1 VAR_024313 p.Arg763Gln LB/B rs3796704 -
ENAM Q9NRM1 VAR_047076 p.Asp767Gly LB/B rs3796705 -
ENAM Q9NRM1 VAR_073665 p.Ser216Leu LP/P rs867263935 Amelogenesis imperfecta 1B (AI1B) [MIM:104500]
ENAM Q9NRM1 VAR_073665 p.Ser216Leu LP/P rs867263935 Amelogenesis imperfecta 1C (AI1C) [MIM:204650]
ENC1 O14682 VAR_050040 p.Ile256Ser LB/B rs16872126 -
ENDOD1 O94919 VAR_022044 p.Val350Met LB/B rs3740862 -
ENDOD1 O94919 VAR_022045 p.Gly446Val LB/B rs3740861 -
ENDOG Q14249 VAR_031691 p.Ser12Leu LB/B rs2293969 -
ENDOV Q8N8Q3 VAR_046285 p.Val29Ile LB/B rs35549084 -
ENDOV Q8N8Q3 VAR_046286 p.Arg112Gln LB/B rs34933300 -
ENDOV Q8N8Q3 VAR_046287 p.Lys114Arg LB/B rs41298706 -
ENDOV Q8N8Q3 VAR_046288 p.His141Tyr LB/B rs41299812 -
ENDOV Q8N8Q3 VAR_046289 p.Asp201Asn LB/B rs35929621 -
ENG P17813 VAR_005192 p.Thr5Met LB/B rs35400405 -
ENG P17813 VAR_005193 p.Gly52Val LP/P - Telangiectasia, hereditary hemorrhagic, 1 (HHT1) [MIM:187300]
ENG P17813 VAR_005194 p.Cys53Arg LP/P - Telangiectasia, hereditary hemorrhagic, 1 (HHT1) [MIM:187300]
ENG P17813 VAR_005195 p.Trp149Cys LP/P rs878853657 Telangiectasia, hereditary hemorrhagic, 1 (HHT1) [MIM:187300]
ENG P17813 VAR_005197 p.Leu306Pro LP/P - Telangiectasia, hereditary hemorrhagic, 1 (HHT1) [MIM:187300]

ENG P17813 VAR_009120 p.Ala160Asp LP/P - Telangiectasia, hereditary hemorrhagic, 1 (HHT1) [MIM:187300]
ENG P17813 VAR_009121 p.Leu221Pro LP/P rs1554810378 Telangiectasia, hereditary hemorrhagic, 1 (HHT1) [MIM:187300]
ENG P17813 VAR_014764 p.Asp366His LB/B rs1800956 -
ENG P17813 VAR_026774 p.Leu8Pro LP/P rs1564466414 Telangiectasia, hereditary hemorrhagic, 1 (HHT1) [MIM:187300]
ENG P17813 VAR_026775 p.Val49Phe LP/P rs1252348200 Telangiectasia, hereditary hemorrhagic, 1 (HHT1) [MIM:187300]
ENG P17813 VAR_026776 p.Leu107Arg LP/P - Telangiectasia, hereditary hemorrhagic, 1 (HHT1) [MIM:187300]
ENG P17813 VAR_026780 p.Ile263Thr LP/P - Telangiectasia, hereditary hemorrhagic, 1 (HHT1) [MIM:187300]
ENG P17813 VAR_026781 p.Cys412Ser LP/P - Telangiectasia, hereditary hemorrhagic, 1 (HHT1) [MIM:187300]
ENG P17813 VAR_026782 p.Val504Met LP/P rs116330805 Telangiectasia, hereditary hemorrhagic, 1 (HHT1) [MIM:187300]
ENG P17813 VAR_026783 p.Ser615Leu LB/B rs148002300 -
ENG P17813 VAR_037140 p.Gly413Val LP/P rs121918401 Telangiectasia, hereditary hemorrhagic, 1 (HHT1) [MIM:187300]
ENG P17813 VAR_070279 p.Ala11Asp LP/P - Telangiectasia, hereditary hemorrhagic, 1 (HHT1) [MIM:187300]
ENG P17813 VAR_070280 p.Val105Asp LP/P rs1588585880 Telangiectasia, hereditary hemorrhagic, 1 (HHT1) [MIM:187300]
ENG P17813 VAR_070281 p.Ala150Pro US - -
ENG P17813 VAR_070282 p.Ala175Glu LP/P - Telangiectasia, hereditary hemorrhagic, 1 (HHT1) [MIM:187300]
ENG P17813 VAR_070284 p.Arg205Pro LB/B - -
ENG P17813 VAR_070285 p.Ile220Thr LP/P - Telangiectasia, hereditary hemorrhagic, 1 (HHT1) [MIM:187300]
ENG P17813 VAR_070286 p.Leu221Gln LP/P - Telangiectasia, hereditary hemorrhagic, 1 (HHT1) [MIM:187300]
ENG P17813 VAR_070287 p.Val236Met US rs754136153 -
ENG P17813 VAR_070288 p.Val238Glu LP/P rs1060501415 Telangiectasia, hereditary hemorrhagic, 1 (HHT1) [MIM:187300]
ENG P17813 VAR_070289 p.Ile263Ser LP/P - Telangiectasia, hereditary hemorrhagic, 1 (HHT1) [MIM:187300]
ENG P17813 VAR_070290 p.Met269Arg LP/P - Telangiectasia, hereditary hemorrhagic, 1 (HHT1) [MIM:187300]
ENG P17813 VAR_070291 p.Ala308Asp LP/P - Telangiectasia, hereditary hemorrhagic, 1 (HHT1) [MIM:187300]
ENG P17813 VAR_070292 p.Val315Met US rs763508329 -
ENG P17813 VAR_070293 p.Cys363Ser LP/P rs1588580782 Telangiectasia, hereditary hemorrhagic, 1 (HHT1) [MIM:187300]
ENG P17813 VAR_070294 p.Lys374Glu US - -
ENG P17813 VAR_070295 p.Cys394Tyr LP/P - Telangiectasia, hereditary hemorrhagic, 1 (HHT1) [MIM:187300]
ENG P17813 VAR_070296 p.Met414Arg US - -

ENG P17813 VAR_070297 p.Arg437Trp LP/P rs1434169817 Telangiectasia, hereditary hemorrhagic, 1 (HHT1) [MIM:187300]
ENG P17813 VAR_070298 p.Leu490Ser LP/P rs763475207 Telangiectasia, hereditary hemorrhagic, 1 (HHT1) [MIM:187300]
ENG P17813 VAR_070299 p.Arg529His LP/P rs863223538 Telangiectasia, hereditary hemorrhagic, 1 (HHT1) [MIM:187300]
ENG P17813 VAR_070300 p.Arg529Pro LP/P - Telangiectasia, hereditary hemorrhagic, 1 (HHT1) [MIM:187300]
ENG P17813 VAR_070301 p.Gly545Asp LP/P - Telangiectasia, hereditary hemorrhagic, 1 (HHT1) [MIM:187300]
ENG P17813 VAR_070302 p.Gly545Ser LB/B rs142896669 -
ENG P17813 VAR_070303 p.Leu547Pro LP/P - Telangiectasia, hereditary hemorrhagic, 1 (HHT1) [MIM:187300]
ENG P17813 VAR_070304 p.Cys549Tyr US rs1060501421 -
ENG P17813 VAR_070305 p.Asp561Ala LB/B rs375965489 -
ENG P17813 VAR_070306 p.Gly603Arg LP/P - Telangiectasia, hereditary hemorrhagic, 1 (HHT1) [MIM:187300]
ENG P17813 VAR_070307 p.Ala604Asp LP/P - Telangiectasia, hereditary hemorrhagic, 1 (HHT1) [MIM:187300]
ENGASE Q8NFI3 VAR_060188 p.Ser596Asn LB/B rs4789879 -
ENGASE Q8NFI3 VAR_060189 p.Glu731Lys LB/B rs11871357 -
ENO1 P06733 VAR_025172 p.Asn177Lys LB/B rs11544513 -
ENO1 P06733 VAR_048936 p.Pro325Gln LB/B rs11544514 -
ENO2 P09104 VAR_002354 p.Pro264Ala LB/B - -
ENO2 P09104 VAR_002355 p.Thr395Ala LB/B - -
ENO3 P13929 VAR_020618 p.Asn71Ser LB/B rs238238 -
ENO3 P13929 VAR_020619 p.Val85Ala LB/B rs238239 -
ENO3 P13929 VAR_020620 p.Gly156Asp LP/P rs121918403 Glycogen storage disease 13 (GSD13) [MIM:612932]
ENO3 P13929 VAR_020621 p.Gly374Glu LP/P rs121918404 Glycogen storage disease 13 (GSD13) [MIM:612932]
ENOSF1 Q7L5Y1 VAR_042933 p.Asp31Glu LB/B rs34724061 -
ENOSF1 Q7L5Y1 VAR_042934 p.Met145Thr LB/B rs2612086 -
ENOSF1 Q7L5Y1 VAR_042935 p.Tyr428Ser LB/B rs2847620 -
ENOX1 Q8TC92 VAR_052205 p.Glu16Asp LB/B rs7338624 -
ENOX2 Q16206 VAR_069427 p.Val202Ile LB/B rs754363472 -
ENPEP Q07075 VAR_030359 p.Gln213Arg LB/B rs10004516 -
ENPEP Q07075 VAR_030360 p.Val218Ala LB/B rs1126483 -
ENPEP Q07075 VAR_036047 p.Arg887Thr US - A breast cancer sample
ENPEP Q07075 VAR_057056 p.Arg437His LB/B rs34949711 -
ENPEP Q07075 VAR_057057 p.Ser861Arg LB/B rs35812243 -
ENPP1 P22413 VAR_008873 p.Lys173Gln LB/B rs1044498 -
ENPP1 P22413 VAR_014141 p.Leu91Pro LP/P - Ossification of the posterior longitudinal ligament of the spine (OPLL) [MIM:602475]
ENPP1 P22413 VAR_014142 p.Tyr268His LB/B rs17847050 -
ENPP1 P22413 VAR_014143 p.Ser287Phe LP/P rs190947144 Ossification of the posterior longitudinal ligament of the spine (OPLL) [MIM:602475]
ENPP1 P22413 VAR_014144 p.Thr779Pro LB/B rs1805138 -

ENPP1 P22413 VAR_018514 p.Leu579Phe US rs121918024 Arterial calcification of infancy, generalized, 1 (GACI1) [MIM:208000]
ENPP1 P22413 VAR_018515 p.Arg774Cys LB/B rs28933977 -
ENPP1 P22413 VAR_037432 p.Asn179Ser LB/B rs2273411 -
ENPP1 P22413 VAR_037433 p.Gly342Val LP/P rs121918025 Arterial calcification of infancy, generalized, 1 (GACI1) [MIM:208000]
ENPP1 P22413 VAR_037434 p.Tyr371Phe US rs121918026 Arterial calcification of infancy, generalized, 1 (GACI1) [MIM:208000]
ENPP1 P22413 VAR_037435 p.Arg886Thr LB/B rs8192683 -
ENPP1 P22413 VAR_063719 p.Gly266Val LP/P rs121908248 Hypophosphatemic rickets, autosomal recessive, 2 (ARHR2) [MIM:613312]
ENPP1 P22413 VAR_063720 p.Tyr901Ser LP/P rs121908249 Hypophosphatemic rickets, autosomal recessive, 2 (ARHR2) [MIM:613312]
ENPP1 P22413 VAR_067910 p.Pro250Leu US rs754659608 Arterial calcification of infancy, generalized, 1 (GACI1) [MIM:208000]
ENPP1 P22413 VAR_067912 p.Pro305Thr LP/P rs374270497 Arterial calcification of infancy, generalized, 1 (GACI1) [MIM:208000]
ENPP1 P22413 VAR_067913 p.Asp538His LP/P rs387906673 Arterial calcification of infancy, generalized, 1 (GACI1) [MIM:208000]
ENPP1 P22413 VAR_067914 p.Gly586Arg LP/P rs777367269 Arterial calcification of infancy, generalized, 1 (GACI1) [MIM:208000]
ENPP1 P22413 VAR_070782 p.Cys149Ser LP/P rs397518477 Cole disease (COLED) [MIM:615522]
ENPP1 P22413 VAR_070783 p.Cys164Ser LP/P rs397518476 Cole disease (COLED) [MIM:615522]
ENPP1 P22413 VAR_070784 p.Cys177Tyr LP/P rs397518475 Cole disease (COLED) [MIM:615522]
ENPP1 P22413 VAR_077255 p.Gly92Asp LP/P - Hypophosphatemic rickets, autosomal recessive, 2 (ARHR2) [MIM:613312]
ENPP1 P22413 VAR_077256 p.Cys126Arg LP/P - Arterial calcification of infancy, generalized, 1 (GACI1) [MIM:208000]
ENPP1 P22413 VAR_077257 p.Cys133Arg LP/P - Cole disease (COLED) [MIM:615522]
ENPP1 P22413 VAR_077258 p.Cys177Ser LP/P - Cole disease (COLED) [MIM:615522]
ENPP1 P22413 VAR_077259 p.Cys195Arg LP/P rs763457176 Arterial calcification of infancy, generalized, 1 (GACI1) [MIM:208000]
ENPP1 P22413 VAR_077260 p.Cys195Ser LP/P - Arterial calcification of infancy, generalized, 1 (GACI1) [MIM:208000]
ENPP1 P22413 VAR_077261 p.Ser216Tyr US rs760786509 Arterial calcification of infancy, generalized, 1 (GACI1) [MIM:208000]
ENPP1 P22413 VAR_077262 p.Asp218Val LP/P rs1231182870 Arterial calcification of infancy, generalized, 1 (GACI1) [MIM:208000]
ENPP1 P22413 VAR_077263 p.Gly219Arg US - Hypophosphatemic rickets, autosomal recessive, 2 (ARHR2) [MIM:613312]
ENPP1 P22413 VAR_077264 p.Gly242Glu US - Arterial calcification of infancy, generalized, 1 (GACI1) [MIM:208000]
ENPP1 P22413 VAR_077265 p.Asp276Asn US rs143771474 Arterial calcification of infancy, generalized, 1 (GACI1) [MIM:208000]
ENPP1 P22413 VAR_077266 p.Tyr301Cys LP/P - Arterial calcification of infancy, generalized, 1 (GACI1) [MIM:208000]
ENPP1 P22413 VAR_077267 p.Arg349Lys US rs764735802 Arterial calcification of infancy, generalized, 1 (GACI1) [MIM:208000]

ENPP1 P22413 VAR_077268 p.Arg456Gln LP/P rs765071179 Arterial calcification of infancy, generalized, 1 (GACI1) [MIM:208000]
ENPP1 P22413 VAR_077269 p.Tyr471Cys LP/P rs148462924 Arterial calcification of infancy, generalized, 1 (GACI1) [MIM:208000]
ENPP1 P22413 VAR_077270 p.Arg481Trp US rs373044722 Arterial calcification of infancy, generalized, 1 (GACI1) [MIM:208000]
ENPP1 P22413 VAR_077271 p.His500Pro LP/P - Arterial calcification of infancy, generalized, 1 (GACI1) [MIM:208000]
ENPP1 P22413 VAR_077272 p.Ser504Arg LP/P - Arterial calcification of infancy, generalized, 1 (GACI1) [MIM:208000]
ENPP1 P22413 VAR_077273 p.Tyr513Cys LP/P rs1243920034 Arterial calcification of infancy, generalized, 1 (GACI1) [MIM:208000]
ENPP1 P22413 VAR_077274 p.Tyr570Cys LP/P rs140248167 Arterial calcification of infancy, generalized, 1 (GACI1) [MIM:208000]
ENPP1 P22413 VAR_077275 p.Leu611Val LB/B rs79079368 -
ENPP1 P22413 VAR_077276 p.Tyr659Cys LP/P rs143393727 Arterial calcification of infancy, generalized, 1 (GACI1) [MIM:208000]
ENPP1 P22413 VAR_077277 p.Glu668Lys LB/B rs115371819 -
ENPP1 P22413 VAR_077278 p.Cys726Arg LP/P - Arterial calcification of infancy, generalized, 1 (GACI1) [MIM:208000]
ENPP1 P22413 VAR_077279 p.His777Arg LP/P rs147346173 Arterial calcification of infancy, generalized, 1 (GACI1) [MIM:208000]
ENPP1 P22413 VAR_077280 p.Asn792Ser LP/P rs370184526 Arterial calcification of infancy, generalized, 1 (GACI1) [MIM:208000]
ENPP1 P22413 VAR_077280 p.Asn792Ser LP/P rs370184526 Hypophosphatemic rickets, autosomal recessive, 2 (ARHR2) [MIM:613312]
ENPP1 P22413 VAR_077281 p.Asp804His LP/P - Arterial calcification of infancy, generalized, 1 (GACI1) [MIM:208000]
ENPP1 P22413 VAR_077282 p.Arg821His LB/B rs367759638 -
ENPP1 P22413 VAR_077283 p.Arg888Trp LP/P rs184483616 Arterial calcification of infancy, generalized, 1 (GACI1) [MIM:208000]
ENPP1 P22413 VAR_081644 p.Cys120Arg LP/P - Cole disease (COLED) [MIM:615522]
ENPP2 Q13822 VAR_057472 p.Asn577Ser LB/B rs2289886 -
ENPP2 Q13822 VAR_057473 p.Ser726Leu LB/B rs16892767 -
ENPP2 Q13822 VAR_060469 p.Ser493Pro LB/B rs10283100 -
ENPP3 O14638 VAR_018516 p.Val620Met LB/B rs9321309 -
ENPP3 O14638 VAR_031253 p.Ser786Asn LB/B rs17601580 -
ENPP3 O14638 VAR_046538 p.Asn744His LB/B rs36094194 -
ENPP4 Q9Y6X5 VAR_039884 p.His144Gln LB/B rs7451713 -
ENPP4 Q9Y6X5 VAR_039885 p.Ile255Val LB/B rs9381429 -
ENPP4 Q9Y6X5 VAR_039886 p.Ser439Ala LB/B rs16874289 -
ENPP5 Q9UJA9 VAR_020248 p.Leu6Ile LB/B rs3806995 -
ENPP5 Q9UJA9 VAR_024693 p.Ile171Val LB/B rs6926570 -
ENPP5 Q9UJA9 VAR_033918 p.Arg39Pro LB/B rs34109856 -
ENPP5 Q9UJA9 VAR_052940 p.Ile69Val LB/B rs34432940 -
ENPP5 Q9UJA9 VAR_052941 p.Tyr283Cys LB/B rs16874326 -
ENPP6 Q6UWR7 VAR_026644 p.Ser419Gly LB/B rs4479748 -
ENPP6 Q6UWR7 VAR_052942 p.Asp357Asn LB/B rs4488969 -
ENPP7 Q6UWV6 VAR_021506 p.Leu4Pro LB/B rs8074547 -

ENTHD1 Q8IYW4 VAR_037449 p.Ile109Thr LB/B rs17319801 -
ENTPD1 P49961 VAR_022099 p.Val293Ile LB/B rs3793744 -
ENTPD1 P49961 VAR_071082 p.Gly210Arg LP/P rs1566229309 Spastic paraplegia 64, autosomal recessive (SPG64) [MIM:615683]
ENTPD2 Q9Y5L3 VAR_050307 p.Ala103Val LB/B rs34618694 -
ENTPD3 O75355 VAR_027541 p.Glu440Asp LB/B rs4470483 -
ENTPD3 O75355 VAR_027542 p.Ala496Val LB/B rs1047855 -
ENTPD3 O75355 VAR_027543 p.Leu505Phe LB/B rs3733167 -
ENTPD3 O75355 VAR_061384 p.Arg264Gln LB/B rs34266806 -
ENTPD3 O75355 VAR_070813 p.Ile24Val LB/B rs17852714 -
ENTPD4 Q9Y227 VAR_020444 p.Lys354Glu LB/B rs2272641 -
ENTPD4 Q9Y227 VAR_064711 p.Ile341Val LB/B rs549468877 -
ENTPD5 O75356 VAR_050308 p.Lys314Arg LB/B rs17094434 -
ENTPD6 O75354 VAR_017863 p.Leu138Val LB/B rs1044567 -
ENTPD6 O75354 VAR_027812 p.Ser14Asn LB/B rs2076559 -
ENTPD6 O75354 VAR_027813 p.Lys202Glu LB/B rs6050446 -
ENTPD6 O75354 VAR_027814 p.Ser323Asn LB/B rs6138541 -
ENTPD6 O75354 VAR_050309 p.Arg157Gln LB/B rs34007133 -
ENTPD7 Q9NQZ7 VAR_030287 p.Val276Ala LB/B rs11190245 -
ENTPD8 Q5MY95 VAR_035339 p.Leu62Pro LB/B rs6606582 -
ENTPD8 Q5MY95 VAR_061385 p.Glu428Lys LB/B rs61491031 -
ENTR1 Q96C92 VAR_039687 p.Leu157Val LB/B rs7047681 -
ENTR1 Q96C92 VAR_039688 p.Glu176Gly LB/B rs17851182 -
ENTR1 Q96C92 VAR_039689 p.Arg304Gln LB/B rs3812577 -
ENTR1 Q96C92 VAR_039690 p.Val379Met LB/B rs1131992 -
ENTR1 Q96C92 VAR_039691 p.Val428Ile LB/B rs17855450 -
EOGT Q5NDL2 VAR_070090 p.Trp207Ser LP/P rs587776993 Adams-Oliver syndrome 4 (AOS4) [MIM:615297]
EOGT Q5NDL2 VAR_070091 p.Arg377Gln LP/P rs587776995 Adams-Oliver syndrome 4 (AOS4) [MIM:615297]
EOMES O95936 VAR_036069 p.Glu667Gln US - A breast cancer sample
EOMES O95936 VAR_059827 p.Ala120Gly LB/B rs1874198 -
EP300 Q09472 VAR_014428 p.Leu827Pro US - A breast cancer sample
EP300 Q09472 VAR_014429 p.Glu1013Gly US rs1234168115 A breast cancer sample
EP300 Q09472 VAR_014430 p.Ser1650Tyr US - A pancreatic cancer sample
EP300 Q09472 VAR_014431 p.Pro2221Gln US rs28937578 A colorectal cancer sample
EP300 Q09472 VAR_020425 p.Ile997Val LB/B rs20551 -
EP300 Q09472 VAR_038376 p.Thr2174Ser LB/B rs5758252 -
EP300 Q09472 VAR_038377 p.Gln2223Pro LB/B rs1046088 -
EP300 Q09472 VAR_055554 p.Met289Val LB/B rs2230111 -
EP300 Q09472 VAR_074021 p.Asn1511Ile LB/B - -
EP300 Q09472 VAR_080731 p.Gln2007Arg US rs763892493 -
EP300 Q09472 VAR_081986 p.Gln1824Pro LP/P rs1569120903 Menke-Hennekam syndrome 2 (MKHK2) [MIM:618333]
EP400 Q96L91 VAR_046957 p.Thr1308Ile LB/B rs13377636 -
EPAS1 Q99814 VAR_042443 p.Gly537Trp LP/P rs137853036 Erythrocytosis, familial, 4 (ECYT4) [MIM:611783]
EPAS1 Q99814 VAR_061261 p.Thr766Pro LB/B rs59901247 -
EPAS1 Q99814 VAR_061262 p.Pro785Thr LB/B rs61518065 -
EPAS1 Q99814 VAR_067358 p.Pro534Leu LP/P - Erythrocytosis, familial, 4 (ECYT4) [MIM:611783]
EPAS1 Q99814 VAR_067359 p.Met535Thr LP/P - Erythrocytosis, familial, 4 (ECYT4) [MIM:611783]
EPAS1 Q99814 VAR_067360 p.Met535Val LP/P rs137853037 Erythrocytosis, familial, 4 (ECYT4) [MIM:611783]

EPAS1 Q99814 VAR_067361 p.Gly537Arg LP/P rs137853036 Erythrocytosis, familial, 4 (ECYT4) [MIM:611783]
EPAS1 Q99814 VAR_067362 p.Phe540Leu LP/P - Erythrocytosis, familial, 4 (ECYT4) [MIM:611783]
EPB41 P11171 VAR_009122 p.Val214Ile LB/B rs111642750 -
EPB41L1 Q9H4G0 VAR_066600 p.Pro854Ser LP/P rs1569376434 Mental retardation, autosomal dominant 11 (MRD11) [MIM:614257]
EPB41L2 O43491 VAR_020145 p.Gln17His LB/B rs2297852 -
EPB41L3 Q9Y2J2 VAR_048353 p.Ala555Thr LB/B rs9966357 -
EPB41L3 Q9Y2J2 VAR_048354 p.Tyr575Cys LB/B rs8082898 -
EPB41L3 Q9Y2J2 VAR_048355 p.Glu859Gln LB/B rs8096452 -
EPB41L4A Q9HCS5 VAR_055537 p.Val132Ile LB/B rs34008454 -
EPB41L4B Q9H329 VAR_048356 p.Asn816Thr LB/B rs3750450 -
EPB41L5 Q9HCM4 VAR_042699 p.Ala462Thr LB/B rs1034489 -
EPB41L5 Q9HCM4 VAR_048357 p.His334Tyr LB/B rs28930677 -
EPB42 P16452 VAR_007482 p.Ala112Thr LP/P rs104894487 Spherocytosis 5 (SPH5) [MIM:612690]
EPB42 P16452 VAR_012268 p.Arg280Gln LP/P rs121917734 Spherocytosis 5 (SPH5) [MIM:612690]
EPB42 P16452 VAR_058099 p.Asp145Tyr LP/P rs143682977 Spherocytosis 5 (SPH5) [MIM:612690]
EPB42 P16452 VAR_058100 p.Arg287Cys LP/P rs515726212 Spherocytosis 5 (SPH5) [MIM:612690]
EPC1 Q9H2F5 VAR_074181 p.Val123Leu LB/B - -
EPCAM P16422 VAR_018329 p.Met115Thr LB/B rs1126497 -
EPCAM P16422 VAR_063829 p.Cys66Tyr LP/P rs267606785 Diarrhea 5, with tufting enteropathy, congenital (DIAR5) [MIM:613217]
EPG5 Q9HCE0 VAR_035278 p.Glu844Asp LB/B rs3744999 -
EPG5 Q9HCE0 VAR_035279 p.Val1058Ala LB/B rs3744998 -
EPG5 Q9HCE0 VAR_035280 p.Ile1131Val LB/B rs3744997 -
EPG5 Q9HCE0 VAR_035281 p.Ala1511Val LB/B rs1893523 -
EPG5 Q9HCE0 VAR_035282 p.Ser1864Asn LB/B rs34064739 -
EPG5 Q9HCE0 VAR_035283 p.Arg1985Gln LB/B rs34674177 -
EPG5 Q9HCE0 VAR_036525 p.Ala1511Thr US - A breast cancer sample
EPG5 Q9HCE0 VAR_036526 p.Cys1865Tyr US rs1272061911 A breast cancer sample
EPG5 Q9HCE0 VAR_036527 p.Arg2056Trp US rs116076204 A breast cancer sample
EPG5 Q9HCE0 VAR_062210 p.Lys182Glu LB/B rs59422275 -
EPG5 Q9HCE0 VAR_069224 p.Gln336Arg LP/P rs201757275 Vici syndrome (VICIS) [MIM:242840]
EPG5 Q9HCE0 VAR_081372 p.Leu457Pro US rs746862679 Vici syndrome (VICIS) [MIM:242840]
EPG5 Q9HCE0 VAR_081373 p.Gln784Pro US rs754795342 Vici syndrome (VICIS) [MIM:242840]
EPG5 Q9HCE0 VAR_081376 p.Gly1336Glu US rs1085308061 Vici syndrome (VICIS) [MIM:242840]
EPG5 Q9HCE0 VAR_081380 p.Pro1827Ala LP/P rs1568118775 Vici syndrome (VICIS) [MIM:242840]
EPG5 Q9HCE0 VAR_081385 p.Cys2038Arg US rs375057925 Vici syndrome (VICIS) [MIM:242840]
EPG5 Q9HCE0 VAR_081387 p.Leu2092Pro US rs1568104317 Vici syndrome (VICIS) [MIM:242840]
EPG5 Q9HCE0 VAR_081388 p.Glu2414Lys US rs1568094451 Vici syndrome (VICIS) [MIM:242840]
EPHA1 P21709 VAR_028265 p.Val160Ala LB/B rs4725617 -
EPHA1 P21709 VAR_028266 p.Arg492Gln LB/B rs11768549 -
EPHA1 P21709 VAR_028267 p.Met900Val LB/B rs6967117 -
EPHA1 P21709 VAR_042115 p.Arg351Cys LB/B rs56006153 -
EPHA1 P21709 VAR_042116 p.Arg575Gln LB/B rs35719334 -
EPHA1 P21709 VAR_042117 p.Ala585Thr LB/B rs34178823 -
EPHA1 P21709 VAR_042118 p.Pro697Leu LB/B rs34372369 -
EPHA1 P21709 VAR_042119 p.Glu703Lys US - A breast pleomorphic lobular carcinoma sample
EPHA1 P21709 VAR_042120 p.Ser807Arg LB/B rs56244405 -
EPHA10 Q5JZY3 VAR_042159 p.Arg150His US rs771803475 A gastric adenocarcinoma sample

EPHA10	Q5JZY3	VAR_042160	p.Thr220Lys	LB/B	rs56276182	-
EPHA10	Q5JZY3	VAR_042161	p.Phe281Ile	LB/B	rs4653328	-
EPHA10	Q5JZY3	VAR_042162	p.Leu630Pro	LB/B	-	-
EPHA10	Q5JZY3	VAR_042163	p.His775Arg	US	-	A breast infiltrating ductal carcinoma sample
EPHA10	Q5JZY3	VAR_042164	p.Ala956Thr	LB/B	-	-
EPHA10	Q5JZY3	VAR_055992	p.Val103Leu	LB/B	rs34557762	-
EPHA10	Q5JZY3	VAR_055993	p.Leu629Pro	LB/B	rs17511304	-
EPHA10	Q5JZY3	VAR_055994	p.Val645Ile	LB/B	rs12405650	-
EPHA10	Q5JZY3	VAR_055995	p.Gly749Glu	LB/B	rs6671088	-
EPHA10	Q5JZY3	VAR_055996	p.Arg807Gln	LB/B	rs6670599	-
EPHA2	P29317	VAR_042121	p.Gly391Arg	LB/B	rs34192549	-
EPHA2	P29317	VAR_042122	p.Thr511Met	LB/B	rs55747232	-
EPHA2	P29317	VAR_042123	p.Arg568His	LB/B	rs56198600	-
EPHA2	P29317	VAR_042124	p.Gly777Ser	US	rs922655349	A gastric adenocarcinoma sample
EPHA2	P29317	VAR_042125	p.Arg876His	LB/B	rs35903225	-
EPHA2	P29317	VAR_055989	p.Lys99Asn	LB/B	rs1058372	-
EPHA2	P29317	VAR_055990	p.Met631Thr	LB/B	rs34021505	-
EPHA2	P29317	VAR_058907	p.Thr940Ile	LP/P	rs137853200	Cataract 6, multiple types (CTRCT6) [MIM:116600]
EPHA2	P29317	VAR_058908	p.Gly948Trp	LP/P	rs137853199	Cataract 6, multiple types (CTRCT6) [MIM:116600]
EPHA2	P29317	VAR_062532	p.Arg721Gln	LP/P	rs116506614	Cataract 6, multiple types (CTRCT6) [MIM:116600]
EPHA3	P29320	VAR_027919	p.Arg914His	LB/B	rs17801309	-
EPHA3	P29320	VAR_036086	p.Thr37Lys	US	-	A colorectal cancer sample
EPHA3	P29320	VAR_036087	p.Asn85Ser	US	-	A colorectal cancer sample
EPHA3	P29320	VAR_036088	p.Ile621Leu	US	rs1414714315	A colorectal cancer sample
EPHA3	P29320	VAR_036089	p.Asp806Asn	US	-	A colorectal cancer sample
EPHA3	P29320	VAR_042126	p.Ser229Tyr	US	-	A lung large cell carcinoma sample
EPHA3	P29320	VAR_042127	p.Ser449Phe	US	-	A lung neuroendocrine carcinoma sample
EPHA3	P29320	VAR_042128	p.Gly518Leu	US	-	A lung squamous cell carcinoma sample
EPHA3	P29320	VAR_042129	p.Ile564Val	LB/B	rs55712516	-
EPHA3	P29320	VAR_042130	p.Cys568Ser	LB/B	rs56077781	-
EPHA3	P29320	VAR_042131	p.Leu590Pro	LB/B	rs56081642	-
EPHA3	P29320	VAR_042132	p.Gly766Glu	US	-	A lung adenocarcinoma sample
EPHA3	P29320	VAR_042133	p.Ala777Gly	LB/B	rs34437982	-
EPHA3	P29320	VAR_042134	p.Trp924Arg	LB/B	rs35124509	-
EPHA3	P29320	VAR_065831	p.Thr660Lys	US	-	A lung carcinoma sample
EPHA3	P29320	VAR_065832	p.Thr933Met	US	rs372594677	A lung carcinoma sample
EPHA3	P29320	VAR_068853	p.Lys207Asn	US	rs200567888	A pancreatic ductal adenocarcinoma sample
EPHA4	P54764	VAR_042135	p.Arg269Gln	LB/B	rs35084379	-
EPHA4	P54764	VAR_042136	p.Gly370Glu	US	rs756952113	A bladder carcinoma NOS sample
EPHA4	P54764	VAR_042137	p.Ser399Phe	US	rs868224085	A metastatic melanoma sample
EPHA4	P54764	VAR_049721	p.Arg953Lys	LB/B	rs35341687	-
EPHA5	P54756	VAR_042138	p.Asn81Thr	LB/B	rs33932471	-
EPHA5	P54756	VAR_042139	p.Ser235Ala	LB/B	rs55710198	-
EPHA5	P54756	VAR_042140	p.Glu330Gln	LB/B	rs56205382	-
EPHA5	P54756	VAR_042141	p.Arg417Gln	US	rs199614818	A lung adenocarcinoma sample
EPHA5	P54756	VAR_042142	p.Glu503Lys	US	-	A lung large cell carcinoma sample
EPHA5	P54756	VAR_042143	p.Gly582Glu	US	rs1057520012	A lung adenocarcinoma sample

EPHA5	P54756	VAR_042144	p.Ala672Thr	LB/B	rs36050417	-
EPHA5	P54756	VAR_042145	p.Ser673Thr	LB/B	rs56359290	-
EPHA5	P54756	VAR_042146	p.Thr856Ile	US	-	A lung squamous cell carcinoma sample
EPHA5	P54756	VAR_042147	p.His959Arg	LB/B	rs56312931	-
EPHA5	P54756	VAR_042148	p.Asn1032Ser	US	-	A lung large cell carcinoma sample
EPHA5	P54756	VAR_045912	p.Tyr506Cys	LB/B	rs56074660	-
EPHA6	Q9UF33	VAR_042149	p.Phe704Ser	LB/B	-	-
EPHA6	Q9UF33	VAR_055991	p.Ala711Val	LB/B	rs4857276	-
EPHA7	Q15375	VAR_022105	p.Ile138Val	LB/B	rs2278107	-
EPHA7	Q15375	VAR_022106	p.Pro278Ser	LB/B	rs2278106	-
EPHA7	Q15375	VAR_036090	p.Arg371Trp	US	rs371089003	A colorectal cancer sample
EPHA7	Q15375	VAR_042150	p.Glu170Lys	US	-	A colorectal adenocarcinoma sample
EPHA7	Q15375	VAR_042151	p.Gly232Arg	US	-	A metastatic melanoma sample
EPHA7	Q15375	VAR_042152	p.Pro903Ser	US	-	A metastatic melanoma sample
EPHA8	P29322	VAR_022107	p.Val444Met	LB/B	rs2295021	-
EPHA8	P29322	VAR_024514	p.Glu612Gln	LB/B	rs999765	-
EPHA8	P29322	VAR_042153	p.Gly45Ser	LB/B	rs45498698	-
EPHA8	P29322	VAR_042154	p.Val60Leu	LB/B	rs56402644	-
EPHA8	P29322	VAR_042155	p.Asn123Lys	US	-	A breast infiltrating ductal carcinoma sample
EPHA8	P29322	VAR_042156	p.Arg179Cys	US	rs1557556639	A gastric adenocarcinoma sample
EPHA8	P29322	VAR_042157	p.Arg198Leu	US	-	A lung adenocarcinoma sample
EPHA8	P29322	VAR_042158	p.Pro860Leu	US	-	A metastatic melanoma sample
EPHA8	P29322	VAR_061292	p.Pro321Leu	LB/B	rs56656925	-
EPHB1	P54762	VAR_011801	p.Thr87Ser	LB/B	rs1042794	-
EPHB1	P54762	VAR_011802	p.Gly152Arg	LB/B	rs1042793	-
EPHB1	P54762	VAR_011803	p.Arg367Gly	LB/B	rs1042789	-
EPHB1	P54762	VAR_011804	p.Arg485Ser	LB/B	rs1042788	-
EPHB1	P54762	VAR_011805	p.Met847Thr	LB/B	rs1042785	-
EPHB1	P54762	VAR_042165	p.Met18Val	LB/B	rs55650774	-
EPHB1	P54762	VAR_042166	p.Thr387Met	LB/B	rs56396912	-
EPHB1	P54762	VAR_042167	p.Ser707Thr	US	-	An ovarian undifferentiated carcinoma sample
EPHB1	P54762	VAR_042168	p.Ile719Val	US	-	A gastric adenocarcinoma sample
EPHB1	P54762	VAR_042169	p.Arg743Gln	US	rs1338928289	A gastric adenocarcinoma sample
EPHB1	P54762	VAR_042170	p.Ala912Thr	LB/B	rs56345346	-
EPHB1	P54762	VAR_042171	p.Thr981Met	LB/B	rs56186270	-
EPHB1	P54762	VAR_058479	p.Arg973Trp	LB/B	rs1042784	-
EPHB2	P29323	VAR_032853	p.Arg199His	LP/P	rs201754821	Prostate cancer (PC) [MIM:176807]
EPHB2	P29323	VAR_032854	p.Ala279Ser	LP/P	rs35882952	Prostate cancer (PC) [MIM:176807]
EPHB2	P29323	VAR_032855	p.Val650Ala	LP/P	rs142173175	Prostate cancer (PC) [MIM:176807]
EPHB2	P29323	VAR_032856	p.His679Asn	LP/P	-	Prostate cancer (PC) [MIM:176807]
EPHB2	P29323	VAR_032857	p.Met883Val	LP/P	rs372653137	Prostate cancer (PC) [MIM:176807]
EPHB2	P29323	VAR_032858	p.Ile909Met	LP/P	-	Prostate cancer (PC) [MIM:176807]
EPHB2	P29323	VAR_042172	p.Cys289Gly	LB/B	-	-
EPHB2	P29323	VAR_042173	p.Ile361Val	LB/B	rs56180036	-
EPHB2	P29323	VAR_042174	p.Asp678Asn	LB/B	rs28936395	-
EPHB2	P29323	VAR_042175	p.Arg844Trp	LB/B	rs55826626	-
EPHB2	P29323	VAR_082702	p.Arg745Cys	LP/P	rs761749948	Bleeding disorder, platelet-type 22 (BDPLT22) [MIM:618462]
EPHB3	P54753	VAR_042176	p.Arg168Leu	US	-	A lung small cell carcinoma sample

EPHB3 P54753 VAR_042177 p.Arg440Cys LB/B rs56029711 -
EPHB3 P54753 VAR_042178 p.Ile579Val LB/B rs56103851 -
EPHB3 P54753 VAR_042179 p.Ile601Leu LB/B rs56129875 -
EPHB3 P54753 VAR_042180 p.Arg724Trp US rs371378866 A lung neuroendocrine carcinoma sample
EPHB4 P54760 VAR_042181 p.Pro67Leu LB/B rs34653459 -
EPHB4 P54760 VAR_042182 p.Val113Ile LB/B rs55866373 -
EPHB4 P54760 VAR_042183 p.Pro346Leu US rs267601191 A metastatic melanoma sample
EPHB4 P54760 VAR_042184 p.Ala371Val LB/B rs55720981 -
EPHB4 P54760 VAR_042185 p.Asp576Glu LB/B rs36050247 -
EPHB4 P54760 VAR_042186 p.Arg678His LB/B rs55692440 -
EPHB4 P54760 VAR_042187 p.Ala882Thr LB/B rs34918225 -
EPHB4 P54760 VAR_042188 p.Arg889Trp US rs762016655 A gastric adenocarcinoma sample
EPHB4 P54760 VAR_042189 p.Glu890Asp LB/B rs35638378 -
EPHB4 P54760 VAR_071163 p.Lys162Arg LB/B rs17854760 -
EPHB4 P54760 VAR_078063 p.Arg739Gln LP/P rs1057519263 Lymphatic malformation 7 (LMPHM7) [MIM:617300]
EPHB4 P54760 VAR_078064 p.Ile782Ser LP/P rs1057519264 Lymphatic malformation 7 (LMPHM7) [MIM:617300]
EPHB4 P54760 VAR_081689 p.Glu59Lys US rs1584667224 Capillary malformation-arteriovenous malformation 2 (CMAVM2) [MIM:618196]
EPHB4 P54760 VAR_081690 p.Arg74Pro US rs61735971 Capillary malformation-arteriovenous malformation 2 (CMAVM2) [MIM:618196]
EPHB4 P54760 VAR_081691 p.Cys107Arg US rs1562974383 Capillary malformation-arteriovenous malformation 2 (CMAVM2) [MIM:618196]
EPHB4 P54760 VAR_081695 p.Leu187Pro US rs1584666053 Capillary malformation-arteriovenous malformation 2 (CMAVM2) [MIM:618196]
EPHB4 P54760 VAR_081697 p.Cys268Arg US rs201816920 Capillary malformation-arteriovenous malformation 2 (CMAVM2) [MIM:618196]
EPHB4 P54760 VAR_081701 p.Val469Gly US rs1584662591 Capillary malformation-arteriovenous malformation 2 (CMAVM2) [MIM:618196]
EPHB4 P54760 VAR_081702 p.Ala509Gly LB/B rs146937374 -
EPHB4 P54760 VAR_081703 p.Gly516Arg US rs776305185 Capillary malformation-arteriovenous malformation 2 (CMAVM2) [MIM:618196]
EPHB4 P54760 VAR_081706 p.Lys650Asn LP/P rs1584658113 Capillary malformation-arteriovenous malformation 2 (CMAVM2) [MIM:618196]
EPHB4 P54760 VAR_081707 p.Arg656Trp US rs745584371 Capillary malformation-arteriovenous malformation 2 (CMAVM2) [MIM:618196]
EPHB4 P54760 VAR_081708 p.Glu664Lys LP/P rs1562969219 Capillary malformation-arteriovenous malformation 2 (CMAVM2) [MIM:618196]
EPHB4 P54760 VAR_081709 p.Ala725Thr US rs1159930961 Capillary malformation-arteriovenous malformation 2 (CMAVM2) [MIM:618196]
EPHB4 P54760 VAR_081711 p.Asn745Asp LP/P rs1584654433 Capillary malformation-arteriovenous malformation 2 (CMAVM2) [MIM:618196]
EPHB4 P54760 VAR_081712 p.Pro789Arg US rs753075600 Capillary malformation-arteriovenous malformation 2 (CMAVM2) [MIM:618196]
EPHB4 P54760 VAR_081713 p.Pro789Ser US rs1417508111 Capillary malformation-arteriovenous malformation 2 (CMAVM2) [MIM:618196]
EPHB4 P54760 VAR_081714 p.Asp802Gly LP/P rs776410552 Capillary malformation-arteriovenous malformation 2 (CMAVM2) [MIM:618196]

EPHB4 P54760 VAR_081716 p.Gly807Arg US rs1330628156 Capillary malformation-arteriovenous malformation 2 (CMAVM2) [MIM:618196]
EPHB4 P54760 VAR_081717 p.Pro820Leu US rs1584653650 Capillary malformation-arteriovenous malformation 2 (CMAVM2) [MIM:618196]
EPHB4 P54760 VAR_081718 p.Pro820Thr US rs1584653653 Capillary malformation-arteriovenous malformation 2 (CMAVM2) [MIM:618196]
EPHB4 P54760 VAR_081719 p.Arg838Trp LP/P rs764827256 Capillary malformation-arteriovenous malformation 2 (CMAVM2) [MIM:618196]
EPHB4 P54760 VAR_081720 p.Cys845Arg LP/P rs1584653054 Capillary malformation-arteriovenous malformation 2 (CMAVM2) [MIM:618196]
EPHB4 P54760 VAR_081721 p.Cys856Tyr LP/P rs1584653005 Capillary malformation-arteriovenous malformation 2 (CMAVM2) [MIM:618196]
EPHB4 P54760 VAR_081722 p.Arg864Trp LP/P rs769965440 Capillary malformation-arteriovenous malformation 2 (CMAVM2) [MIM:618196]
EPHB4 P54760 VAR_081723 p.Phe867Leu LP/P rs1584652949 Capillary malformation-arteriovenous malformation 2 (CMAVM2) [MIM:618196]
EPHB4 P54760 VAR_081724 p.Val870Glu US rs1584652920 Capillary malformation-arteriovenous malformation 2 (CMAVM2) [MIM:618196]
EPHB4 P54760 VAR_081725 p.Leu874Pro US rs1584652900 Capillary malformation-arteriovenous malformation 2 (CMAVM2) [MIM:618196]
EPHB6 O15197 VAR_019139 p.Gly122Ser LB/B rs8177173 -
EPHB6 O15197 VAR_019140 p.Pro282Arg LB/B rs8177143 -
EPHB6 O15197 VAR_019141 p.Ser324Ala LB/B rs8177146 -
EPHB6 O15197 VAR_019142 p.Arg499Gln LB/B rs8177175 -
EPHB6 O15197 VAR_036091 p.Asp360Asn US - A colorectal cancer sample
EPHB6 O15197 VAR_036092 p.Ala603Pro US - A colorectal cancer sample
EPHB6 O15197 VAR_036093 p.Arg719Gln US - A colorectal cancer sample
EPHB6 O15197 VAR_036094 p.Asp930Gly US - A colorectal cancer sample
EPHB6 O15197 VAR_042190 p.Ser170Thr LB/B - -
EPHB6 O15197 VAR_042191 p.Ala221Val LB/B - -
EPHB6 O15197 VAR_042192 p.Pro282His LB/B - -
EPHB6 O15197 VAR_042193 p.Arg309Gln LB/B rs55728646 -
EPHB6 O15197 VAR_042194 p.Ser332Leu LB/B rs35189999 -
EPHB6 O15197 VAR_042195 p.Ala662Val LB/B rs35984674 -
EPHB6 O15197 VAR_042196 p.Pro743Ser US - An ovarian mucinous carcinoma sample
EPHB6 O15197 VAR_042197 p.Arg813His LB/B - -
EPHB6 O15197 VAR_042198 p.Glu875Lys US - A glioblastoma multiforme sample
EPHB6 O15197 VAR_042199 p.Ile993Val LB/B - -
EPHX1 P07099 VAR_005295 p.Tyr113His LB/B rs1051740 -
EPHX1 P07099 VAR_005296 p.His139Arg LB/B rs2234922 -
EPHX1 P07099 VAR_005297 p.Thr396Ile US - -
EPHX1 P07099 VAR_013298 p.Arg49Cys LB/B rs2234697 -
EPHX1 P07099 VAR_013299 p.Leu260Pro LB/B - -
EPHX1 P07099 VAR_013300 p.Arg454Gln LB/B rs2234701 -
EPHX1 P07099 VAR_018347 p.Glu44Gln LB/B rs745306359 -
EPHX1 P07099 VAR_023303 p.Arg43Thr LB/B rs3738046 -
EPHX1 P07099 VAR_023304 p.Val285Leu LB/B rs45449793 -
EPHX1 P07099 VAR_023305 p.Thr408Met LB/B rs45495897 -
EPHX1 P07099 VAR_023306 p.Leu452Gln LB/B rs45563137 -

EPHX1	P07099	VAR_051828	p.Thr275Ala	LB/B	rs35073925	-
EPHX2	P34913	VAR_014852	p.Arg287Gln	LB/B	rs751141	-
EPHX2	P34913	VAR_033991	p.Arg103Cys	LB/B	rs17057255	-
EPHX2	P34913	VAR_051059	p.Lys55Arg	LB/B	rs41507953	-
EPHX2	P34913	VAR_055392	p.Gly21Ala	LB/B	rs72473930	-
EPHX2	P34913	VAR_055393	p.Arg52Gln	LB/B	rs72475803	-
EPHX2	P34913	VAR_055394	p.Cys154Tyr	LB/B	rs57699806	-
EPHX2	P34913	VAR_055395	p.Pro225Leu	LB/B	rs72475821	-
EPHX2	P34913	VAR_055396	p.Met369Val	LB/B	rs72475894	-
EPHX2	P34913	VAR_055397	p.Glu470Gly	LB/B	rs68053459	-
EPHX4	Q8IUS5	VAR_031235	p.Tyr321Phe	LB/B	rs17854127	-
EPM2A	O95278	VAR_019465	p.Ser25Pro	LP/P	-	Epilepsy, progressive myoclonic 2 (EPM2) [MIM:254780]
EPM2A	O95278	VAR_019466	p.Glu28Lys	LP/P	-	Epilepsy, progressive myoclonic 2 (EPM2) [MIM:254780]
EPM2A	O95278	VAR_019467	p.Trp32Gly	LP/P	rs104893955	Epilepsy, progressive myoclonic 2 (EPM2) [MIM:254780]
EPM2A	O95278	VAR_019468	p.Ala46Pro	LB/B	rs374338349	-
EPM2A	O95278	VAR_019469	p.Phe84Leu	LP/P	rs1362231306	Epilepsy, progressive myoclonic 2 (EPM2) [MIM:254780]
EPM2A	O95278	VAR_019470	p.Phe88Leu	LP/P	rs1034706422	Epilepsy, progressive myoclonic 2 (EPM2) [MIM:254780]
EPM2A	O95278	VAR_019471	p.Arg91Pro	LP/P	-	Epilepsy, progressive myoclonic 2 (EPM2) [MIM:254780]
EPM2A	O95278	VAR_019472	p.Arg108Cys	LP/P	rs137852915	Epilepsy, progressive myoclonic 2 (EPM2) [MIM:254780]
EPM2A	O95278	VAR_019473	p.Glu114Asp	LB/B	-	-
EPM2A	O95278	VAR_019474	p.Arg171His	LP/P	rs137852916	Epilepsy, progressive myoclonic 2 (EPM2) [MIM:254780]
EPM2A	O95278	VAR_019475	p.Thr187Ala	LP/P	-	Epilepsy, progressive myoclonic 2 (EPM2) [MIM:254780]
EPM2A	O95278	VAR_019476	p.Thr194Ile	LP/P	rs375544596	Epilepsy, progressive myoclonic 2 (EPM2) [MIM:254780]
EPM2A	O95278	VAR_019477	p.Gly240Ser	LP/P	-	Epilepsy, progressive myoclonic 2 (EPM2) [MIM:254780]
EPM2A	O95278	VAR_019478	p.Gly279Ser	LP/P	rs137852917	Epilepsy, progressive myoclonic 2 (EPM2) [MIM:254780]
EPM2A	O95278	VAR_019479	p.Gln293Leu	LP/P	rs796052427	Epilepsy, progressive myoclonic 2 (EPM2) [MIM:254780]
EPM2A	O95278	VAR_019480	p.Tyr294Asn	LP/P	-	Epilepsy, progressive myoclonic 2 (EPM2) [MIM:254780]
EPM2A	O95278	VAR_019481	p.Pro301Leu	LP/P	rs796052428	Epilepsy, progressive myoclonic 2 (EPM2) [MIM:254780]
EPM2A	O95278	VAR_046383	p.Lys140Asn	LP/P	-	Epilepsy, progressive myoclonic 2 (EPM2) [MIM:254780]
EPM2A	O95278	VAR_046384	p.Asn148Tyr	LP/P	-	Epilepsy, progressive myoclonic 2 (EPM2) [MIM:254780]
EPM2A	O95278	VAR_046385	p.Glu210Lys	LP/P	-	Epilepsy, progressive myoclonic 2 (EPM2) [MIM:254780]
EPM2A	O95278	VAR_046386	p.Leu310Trp	LP/P	-	Epilepsy, progressive myoclonic 2 (EPM2) [MIM:254780]

EPM2AIP1 Q7L775 VAR_050969 p.Val333Phe LB/B rs4647202 -
EPM2AIP1 Q7L775 VAR_050970 p.Ala360Ser LB/B rs4647201 -
EPN2 O95208 VAR_047923 p.Pro531Thr LB/B rs1062727 -
EPN2 O95208 VAR_053080 p.Val401Ala LB/B rs6587220 -
EPN2 O95208 VAR_053081 p.Pro532Thr LB/B rs1062727 -
EPN3 Q9H201 VAR_059973 p.Pro544Thr LB/B rs4794159 -
EPO P01588 VAR_009871 p.Pro149Gln US - A hepatocellular carcinoma
EPO P01588 VAR_078447 p.Arg177Gln LP/P rs1358275550 Diamond-Blackfan anemia-like (DBAL) [MIM:617911]
EPO P01588 VAR_080573 p.Asp70Asn US rs62483572 Erythrocytosis, familial, 5 (ECYT5) [MIM:617907]
EPO P01588 VAR_080574 p.Gly84Arg US rs137953994 Erythrocytosis, familial, 5 (ECYT5) [MIM:617907]
EPO P01588 VAR_080575 p.Glu99Gly US - -
EPO P01588 VAR_080576 p.Pro114Leu US rs11976235 Erythrocytosis, familial, 5 (ECYT5) [MIM:617907]
EPO P01588 VAR_080577 p.Ser147Cys US rs149431976 Erythrocytosis, familial, 5 (ECYT5) [MIM:617907]
EPOR P19235 VAR_027372 p.Asn487Ser US rs62638745 Erythroleukemia
EPOR P19235 VAR_027372 p.Asn487Ser LP/P rs62638745 Erythrocytosis, familial, 1 (ECYT1) [MIM:133100]
EPOR P19235 VAR_027373 p.Pro488Ser LB/B rs142094773 -
EPOR P19235 VAR_033919 p.Pro380Ala LB/B rs35423344 -
EPPIN O95925 VAR_024696 p.His92Arg LB/B rs2231838 -
EPPIN O95925 VAR_052950 p.Lys128Thr LB/B rs2231839 -
EPPK1 P58107 VAR_081533 p.Ser1094Asn US - -
EPRS1 P07814 VAR_037288 p.Ala296Pro LB/B rs35999099 -
EPRS1 P07814 VAR_037289 p.Asp308Glu LB/B rs2230301 -
EPRS1 P07814 VAR_037290 p.Gln334His LB/B rs1063236 -
EPRS1 P07814 VAR_037291 p.Pro893His LB/B rs5030751 -
EPRS1 P07814 VAR_037292 p.Ile1043Val LB/B rs5030752 -
EPRS1 P07814 VAR_037293 p.Ser1107Phe LB/B rs12144752 -
EPRS1 P07814 VAR_037294 p.Thr1399Asn LB/B rs34559775 -
EPRS1 P07814 VAR_057358 p.Glu913Gly LB/B rs2230302 -
EPRS1 P07814 VAR_080801 p.Pro1115Arg LP/P rs1288116010 Leukodystrophy, hypomyelinating, 15 (HLD15) [MIM:617951]
EPRS1 P07814 VAR_080802 p.Met1126Thr US rs1474000585 Leukodystrophy, hypomyelinating, 15 (HLD15) [MIM:617951]
EPRS1 P07814 VAR_080803 p.Pro1160Ser US rs898824971 Leukodystrophy, hypomyelinating, 15 (HLD15) [MIM:617951]
EPS15 P42566 VAR_016142 p.Ile822Met LB/B rs17567 -
EPS8 Q12929 VAR_050971 p.Asp761Glu LB/B rs7137185 -
EPS8 Q12929 VAR_050972 p.Ala806Ser LB/B rs1802658 -
EPS8L1 Q8TE68 VAR_056870 p.Gln457Glu LB/B rs1628576 -
EPS8L1 Q8TE68 VAR_060375 p.Ala4Thr LB/B rs12609976 -
EPS8L1 Q8TE68 VAR_060376 p.Arg288Gly LB/B rs1620074 -
EPS8L1 Q8TE68 VAR_060377 p.Lys669Arg LB/B rs1054940 -
EPS8L1 Q8TE68 VAR_061647 p.Leu703Pro LB/B rs60073068 -
EPS8L3 Q8TE67 VAR_026580 p.Gly163Ser LB/B rs6693815 -
EPS8L3 Q8TE67 VAR_026581 p.His293Tyr LB/B rs3818562 -
EPS8L3 Q8TE67 VAR_026582 p.Pro356Ser LB/B rs11102001 -
EPS8L3 Q8TE67 VAR_050976 p.Met35Ile LB/B rs17598321 -
EPS8L3 Q8TE67 VAR_050977 p.Arg581Gln LB/B rs35072794 -
EPS8L3 Q8TE67 VAR_083829 p.Ala8Thr US - Hypotrichosis 5 (HYPT5) [MIM:612841]

EPX	P11678	VAR_015376	p.Arg286His	LP/P	rs121434566	Eosinophil peroxidase deficiency (EPXD) [MIM:261500]
EPX	P11678	VAR_020031	p.Asn572Tyr	LB/B	rs2302311	-
EPX	P11678	VAR_025138	p.Ile40Met	LB/B	rs11079339	-
EPX	P11678	VAR_025139	p.Gln122His	LB/B	rs11652709	-
EPX	P11678	VAR_025140	p.Ala249Glu	LB/B	rs35896669	-
EPX	P11678	VAR_025141	p.Lys276Arg	LB/B	rs35074452	-
EPX	P11678	VAR_025142	p.Pro292Leu	LB/B	rs33971258	-
EPX	P11678	VAR_025143	p.Arg326Pro	LB/B	rs35832094	-
EPX	P11678	VAR_025144	p.Pro358Leu	LB/B	rs35135976	-
EPX	P11678	VAR_025145	p.Arg364His	LB/B	rs35232062	-
EPX	P11678	VAR_025146	p.Lys441Thr	LB/B	rs35750729	-
EPX	P11678	VAR_025147	p.His496Gln	LB/B	rs33955150	-
EPX	P11678	VAR_050485	p.Val35Ile	LB/B	rs34553736	-
EPX	P11678	VAR_050486	p.Val458Met	LB/B	rs34817773	-
EPX	P11678	VAR_060197	p.Arg326His	LB/B	rs35832094	-
EPX	P11678	VAR_060198	p.Arg326Leu	LB/B	-	-
EPYC	Q99645	VAR_031595	p.Ser150Cys	LB/B	rs17784152	-
EQTN	Q9NQ60	VAR_032136	p.Asn101Asp	LB/B	rs12337286	-
EQTN	Q9NQ60	VAR_032137	p.Thr274Lys	LB/B	rs41305329	-
EQTN	Q9NQ60	VAR_056727	p.Ile110Thr	LB/B	rs12341576	-
ERAL1	O75616	VAR_079209	p.Asn236Ile	LP/P	rs1131692170	Perrault syndrome 6 (PRLTS6) [MIM:617565]
ERAP1	Q9NZ08	VAR_012779	p.Arg127Pro	LB/B	rs26653	-
ERAP1	Q9NZ08	VAR_012780	p.Ile276Met	LB/B	rs26618	-
ERAP1	Q9NZ08	VAR_012781	p.Gly346Asp	LB/B	rs27895	-
ERAP1	Q9NZ08	VAR_012782	p.Met349Val	LB/B	rs2287987	-
ERAP1	Q9NZ08	VAR_012783	p.Lys528Arg	LB/B	rs30187	-
ERAP1	Q9NZ08	VAR_012784	p.Gln730Glu	LB/B	rs27044	-
ERAP1	Q9NZ08	VAR_021555	p.Asp575Asn	LB/B	rs10050860	-
ERAP1	Q9NZ08	VAR_021556	p.Arg725Gln	LB/B	rs17482078	-
ERAP1	Q9NZ08	VAR_046681	p.Glu56Lys	LB/B	rs3734016	-
ERAP1	Q9NZ08	VAR_046682	p.Asp575Gly	LB/B	rs6863093	-
ERAP2	Q6P179	VAR_038285	p.Pro214Leu	LB/B	rs3733905	-
ERAP2	Q6P179	VAR_038286	p.Lys392Asn	LB/B	rs2549782	-
ERAP2	Q6P179	VAR_038287	p.Leu669Gln	LB/B	rs17408150	-
ERAP2	Q6P179	VAR_051569	p.Leu411Arg	LB/B	rs34261036	-
ERBB2	P04626	VAR_004077	p.Ile654Val	LB/B	rs1801201	-
ERBB2	P04626	VAR_004078	p.Ile655Val	LB/B	rs1136201	-
ERBB2	P04626	VAR_016317	p.Trp452Cys	LB/B	rs4252633	-
ERBB2	P04626	VAR_016318	p.Pro1170Ala	LB/B	rs1058808	-
ERBB2	P04626	VAR_042097	p.Leu768Ser	LB/B	rs56366519	-
ERBB2	P04626	VAR_042098	p.Gly776Ser	US	rs28933369	Gastric cancer (GASC) [MIM:613659]
ERBB2	P04626	VAR_042099	p.Asn857Ser	US	rs28933370	Ovarian cancer (OC) [MIM:167000]
ERBB2	P04626	VAR_042100	p.Ala1216Asp	LB/B	rs55943169	-
ERBB2	P04626	VAR_055432	p.Leu755Pro	US	rs121913469	Lung cancer (LNCR) [MIM:211980]
ERBB2	P04626	VAR_055435	p.Glu914Lys	US	rs28933368	Glioma (GLM) [MIM:137800]
ERBB3	P21860	VAR_042101	p.Ser20Tyr	LB/B	rs34379766	-
ERBB3	P21860	VAR_042102	p.Pro30Leu	LB/B	rs56017157	-
ERBB3	P21860	VAR_042103	p.Val104Met	US	rs1057519893	An ovarian mucinous carcinoma sample

ERBB3 P21860 VAR_042104 p.Thr204Ile LB/B rs56107455 -
ERBB3 P21860 VAR_042105 p.Arg683Trp LB/B rs56387488 -
ERBB3 P21860 VAR_042106 p.Ser717Leu LB/B rs35961836 -
ERBB3 P21860 VAR_042107 p.Ile744Thr LB/B rs55787439 -
ERBB3 P21860 VAR_042108 p.Lys998Arg LB/B rs56259600 -
ERBB3 P21860 VAR_042109 p.Ser1119Cys LB/B rs773123 -
ERBB3 P21860 VAR_042110 p.Arg1127His LB/B rs2271188 -
ERBB3 P21860 VAR_042111 p.Leu1177Ile LB/B rs55699040 -
ERBB3 P21860 VAR_042112 p.Thr1254Lys LB/B rs55709407 -
ERBB3 P21860 VAR_049710 p.Asn385Ser LB/B rs12320176 -
ERBB3 P21860 VAR_049711 p.Gly1271Ser LB/B rs11171743 -
ERBB3 P21860 VAR_081641 p.Ala1337Thr LP/P rs755855285 Erythroleukemia, familial (FERLK) [MIM:133180]
ERBB4 Q15303 VAR_042113 p.Thr140Ile US - A colorectal adenocarcinoma sample
ERBB4 Q15303 VAR_042114 p.Ser303Tyr US - A lung squamous cell carcinoma sample
ERBB4 Q15303 VAR_070810 p.Arg927Gln LP/P rs397514262 Amyotrophic lateral sclerosis 19 (ALS19) [MIM:615515]
ERBB4 Q15303 VAR_070811 p.Arg1275Trp LP/P rs397514263 Amyotrophic lateral sclerosis 19 (ALS19) [MIM:615515]
ERBIN Q96RT1 VAR_019346 p.Ser274Leu LB/B rs3213837 -
ERBIN Q96RT1 VAR_019347 p.Ser1112Leu LB/B rs3805466 -
ERBIN Q96RT1 VAR_019348 p.Lys1207Glu LB/B - -
ERBIN Q96RT1 VAR_028304 p.Lys746Glu LB/B rs16894812 -
ERBIN Q96RT1 VAR_046673 p.Lys914Arg LB/B rs34521887 -
ERBIN Q96RT1 VAR_046674 p.Gly1089Val LB/B rs35601230 -
ERBIN Q96RT1 VAR_068905 p.Ala313Val LB/B rs191137999 -
ERC1 Q8IUD2 VAR_051304 p.Ser50Gly LB/B rs35037408 -
ERC1 Q8IUD2 VAR_051305 p.Thr1032Ala LB/B rs12319376 -
ERC2 O15083 VAR_050973 p.Asn542Ser LB/B rs12488237 -
ERCC1 P07992 VAR_019167 p.Ala266Thr LB/B rs3212977 -
ERCC1 P07992 VAR_032776 p.Phe231Leu LP/P rs121913028 Cerebro-oculo-facio-skeletal syndrome 4 (COFS4) [MIM:610758]
ERCC2 P18074 VAR_003622 p.Arg112His LP/P rs121913020 Trichothiodystrophy 1, photosensitive (TTD1) [MIM:601675]
ERCC2 P18074 VAR_003622 p.Arg112His LP/P rs121913020 Xeroderma pigmentosum complementation group D (XP-D) [MIM:278730]
ERCC2 P18074 VAR_003623 p.Leu461Val LP/P rs121913016 Trichothiodystrophy 1, photosensitive (TTD1) [MIM:601675]
ERCC2 P18074 VAR_003623 p.Leu461Val LP/P rs121913016 Xeroderma pigmentosum complementation group D (XP-D) [MIM:278730]
ERCC2 P18074 VAR_003625 p.Ser541Arg LP/P rs121913019 Xeroderma pigmentosum complementation group D (XP-D) [MIM:278730]
ERCC2 P18074 VAR_003626 p.Arg616Pro LP/P rs376556895 Trichothiodystrophy 1, photosensitive (TTD1) [MIM:601675]
ERCC2 P18074 VAR_003626 p.Arg616Pro LP/P rs376556895 Xeroderma pigmentosum complementation group D (XP-D) [MIM:278730]
ERCC2 P18074 VAR_003627 p.Gly602Asp LP/P rs771824813 Xeroderma pigmentosum complementation group D (XP-D) [MIM:278730]
ERCC2 P18074 VAR_003628 p.Gly675Arg US - -

ERCC2 P18074 VAR_003630 p.Arg722Trp LP/P rs121913026 Trichothiodystrophy 1, photosensitive (TTD1) [MIM:601675]
ERCC2 P18074 VAR_003631 p.Ala725Pro LP/P rs121913018 Trichothiodystrophy 1, photosensitive (TTD1) [MIM:601675]
ERCC2 P18074 VAR_008187 p.Gly47Arg LP/P rs1360631927 Xeroderma pigmentosum complementation group D (XP-D) [MIM:278730]
ERCC2 P18074 VAR_008188 p.Asp234Asn LP/P rs1340806384 Xeroderma pigmentosum complementation group D (XP-D) [MIM:278730]
ERCC2 P18074 VAR_008189 p.Cys259Tyr LP/P rs370454709 Trichothiodystrophy 1, photosensitive (TTD1) [MIM:601675]
ERCC2 P18074 VAR_008191 p.Tyr542Cys LP/P - Xeroderma pigmentosum complementation group D (XP-D) [MIM:278730]
ERCC2 P18074 VAR_008192 p.Arg601Leu LP/P rs140522180 Xeroderma pigmentosum complementation group D (XP-D) [MIM:278730]
ERCC2 P18074 VAR_008193 p.Arg616Trp LP/P rs121913024 Cerebro-oculo-facio-skeletal syndrome 2 (COFS2) [MIM:610756]
ERCC2 P18074 VAR_008193 p.Arg616Trp LP/P rs121913024 Xeroderma pigmentosum complementation group D (XP-D) [MIM:278730]
ERCC2 P18074 VAR_008194 p.Arg658Cys LP/P rs121913021 Trichothiodystrophy 1, photosensitive (TTD1) [MIM:601675]
ERCC2 P18074 VAR_008195 p.Arg658His LP/P rs762141272 Trichothiodystrophy 1, photosensitive (TTD1) [MIM:601675]
ERCC2 P18074 VAR_008196 p.Asp673Gly LP/P - Trichothiodystrophy 1, photosensitive (TTD1) [MIM:601675]
ERCC2 P18074 VAR_008197 p.Arg683Gln LP/P rs758439420 Xeroderma pigmentosum complementation group D (XP-D) [MIM:278730]
ERCC2 P18074 VAR_008198 p.Arg683Trp LP/P rs41556519 Xeroderma pigmentosum complementation group D (XP-D) [MIM:278730]
ERCC2 P18074 VAR_008199 p.Gly713Arg LP/P rs121913022 Trichothiodystrophy 1, photosensitive (TTD1) [MIM:601675]
ERCC2 P18074 VAR_011412 p.Ile199Met LB/B rs1799791 -
ERCC2 P18074 VAR_011413 p.His201Tyr LB/B rs1799792 -
ERCC2 P18074 VAR_011414 p.Asp312Asn LB/B rs1799793 -
ERCC2 P18074 VAR_011415 p.Arg616Cys LB/B - -
ERCC2 P18074 VAR_011416 p.Lys751Gln LB/B rs13181 -
ERCC2 P18074 VAR_017282 p.Thr76Ala LP/P - Xeroderma pigmentosum complementation group D (XP-D) [MIM:278730]
ERCC2 P18074 VAR_017283 p.Leu485Pro LP/P rs121913025 Xeroderma pigmentosum complementation group D (XP-D) [MIM:278730]
ERCC2 P18074 VAR_017284 p.Arg487Gly LP/P - Trichothiodystrophy 1, photosensitive (TTD1) [MIM:601675]
ERCC2 P18074 VAR_017285 p.Arg511Gln LP/P rs772572683 Xeroderma pigmentosum complementation group D (XP-D) [MIM:278730]
ERCC2 P18074 VAR_017287 p.Arg592Pro LP/P - Trichothiodystrophy 1, photosensitive (TTD1) [MIM:601675]
ERCC2 P18074 VAR_017288 p.Ala594Pro LP/P - Trichothiodystrophy 1, photosensitive (TTD1) [MIM:601675]
ERCC2 P18074 VAR_017289 p.Arg601Trp LP/P rs753641926 Xeroderma pigmentosum complementation group D (XP-D) [MIM:278730]

ERCC2 P18074 VAR_017290 p.Arg658Gly LP/P - Trichothiodystrophy 1, photosensitive (TTD1) [MIM:601675]
ERCC2 P18074 VAR_017291 p.Cys663Arg LP/P rs770367713 Trichothiodystrophy 1, photosensitive (TTD1) [MIM:601675]
ERCC2 P18074 VAR_017292 p.Arg666Trp LP/P rs752510317 Xeroderma pigmentosum complementation group D (XP-D) [MIM:278730]
ERCC2 P18074 VAR_017293 p.Asp681Asn LP/P rs121913023 Cerebro-oculo-facio-skeletal syndrome 2 (COFS2) [MIM:610756]
ERCC2 P18074 VAR_017293 p.Asp681Asn LP/P rs121913023 Xeroderma pigmentosum complementation group D (XP-D) [MIM:278730]
ERCC3 P19447 VAR_003632 p.Phe99Ser LP/P rs121913045 Xeroderma pigmentosum complementation group B (XP-B) [MIM:610651]
ERCC3 P19447 VAR_008186 p.Thr119Pro LP/P rs121913046 Trichothiodystrophy 2, photosensitive (TTD2) [MIM:616390]
ERCC3 P19447 VAR_014344 p.Ser735Pro LB/B rs4150522 -
ERCC3 P19447 VAR_014766 p.Lys117Arg LB/B rs1805161 -
ERCC3 P19447 VAR_014767 p.Gly402Cys LB/B rs1805162 -
ERCC3 P19447 VAR_017294 p.Ser704Leu LB/B rs4150521 -
ERCC3 P19447 VAR_035942 p.Lys418Gln US - A breast cancer sample
ERCC4 Q92889 VAR_005849 p.Gly703Asp LB/B - -
ERCC4 Q92889 VAR_005850 p.Arg799Trp LP/P rs121913049 Xeroderma pigmentosum complementation group F (XP-F) [MIM:278760]
ERCC4 Q92889 VAR_008200 p.Ile225Met LP/P rs764731249 Xeroderma pigmentosum complementation group F (XP-F) [MIM:278760]
ERCC4 Q92889 VAR_008201 p.Arg454Trp LP/P - Xeroderma pigmentosum complementation group F (XP-F) [MIM:278760]
ERCC4 Q92889 VAR_008202 p.Arg490Gln LP/P rs912480692 Xeroderma pigmentosum complementation group F (XP-F) [MIM:278760]
ERCC4 Q92889 VAR_008203 p.Glu502Lys LP/P - Xeroderma pigmentosum complementation group F (XP-F) [MIM:278760]
ERCC4 Q92889 VAR_008204 p.Gly513Arg LP/P rs769679311 Xeroderma pigmentosum complementation group F (XP-F) [MIM:278760]
ERCC4 Q92889 VAR_008205 p.Ile529Thr LP/P - Xeroderma pigmentosum complementation group F (XP-F) [MIM:278760]
ERCC4 Q92889 VAR_008206 p.Thr567Ala LP/P - Xeroderma pigmentosum complementation group F (XP-F) [MIM:278760]
ERCC4 Q92889 VAR_013395 p.Pro379Ser LB/B rs1799802 -
ERCC4 Q92889 VAR_013396 p.Arg415Gln LB/B rs1800067 -
ERCC4 Q92889 VAR_013397 p.Arg576Thr LB/B rs1800068 -
ERCC4 Q92889 VAR_013398 p.Leu608Pro LP/P - Xeroderma pigmentosum complementation group F (XP-F) [MIM:278760]
ERCC4 Q92889 VAR_013399 p.Ile717Thr LB/B - -
ERCC4 Q92889 VAR_013408 p.Glu875Gly LB/B rs1800124 -
ERCC4 Q92889 VAR_014769 p.Ala168Val LB/B rs2020961 -
ERCC4 Q92889 VAR_014770 p.Ser662Pro LB/B rs2020955 -
ERCC4 Q92889 VAR_014771 p.Ile706Thr LB/B rs1800069 -
ERCC4 Q92889 VAR_014772 p.Gly912Glu LB/B rs2020956 -
ERCC4 Q92889 VAR_019201 p.Ile873Val LB/B rs2020957 -
ERCC4 Q92889 VAR_034802 p.Arg153Pro LP/P rs121913050 XFE progeroid syndrome (XFEPS) [MIM:610965]

ERCC4 Q92889 VAR_057477 p.Val33Leu LB/B rs34205098 -
ERCC4 Q92889 VAR_057478 p.Ser768Phe LB/B rs12928650 -
ERCC4 Q92889 VAR_057479 p.Ala860Asp LB/B rs4986933 -
ERCC4 Q92889 VAR_070086 p.Leu230Pro LP/P rs397509402 Fanconi anemia complementation group Q (FANCQ) [MIM:615272]
ERCC4 Q92889 VAR_070087 p.Cys236Arg LP/P rs397509403 Xeroderma pigmentosum type F/Cockayne syndrome (XPF/CS) [MIM:278760]
ERCC4 Q92889 VAR_070088 p.Arg589Trp LP/P rs147105770 Xeroderma pigmentosum type F/Cockayne syndrome (XPF/CS) [MIM:278760]
ERCC4 Q92889 VAR_070089 p.Arg689Ser LP/P rs149364215 Fanconi anemia complementation group Q (FANCQ) [MIM:615272]
ERCC4 Q92889 VAR_072084 p.Arg150Cys LB/B rs145402255 -
ERCC4 Q92889 VAR_072085 p.Arg267His LB/B rs143479220 -
ERCC4 Q92889 VAR_072086 p.Thr621Ala LB/B - -
ERCC4 Q92889 VAR_072087 p.Ser786Phe LP/P rs1451008479 Fanconi anemia complementation group Q (FANCQ) [MIM:615272]
ERCC5 P28715 VAR_007732 p.Met254Val LB/B rs1047769 -
ERCC5 P28715 VAR_007733 p.Ala792Val LP/P rs121434571 Xeroderma pigmentosum complementation group G (XP-G) [MIM:278780]
ERCC5 P28715 VAR_007734 p.Asp1104His LB/B rs17655 -
ERCC5 P28715 VAR_014829 p.Ser311Cys LB/B rs2307491 -
ERCC5 P28715 VAR_015280 p.Pro72His LP/P rs121434574 Xeroderma pigmentosum complementation group G (XP-G) [MIM:278780]
ERCC5 P28715 VAR_017096 p.Ala874Thr LP/P rs121434576 Xeroderma pigmentosum complementation group G (XP-G) [MIM:278780]
ERCC5 P28715 VAR_017097 p.Leu858Pro LP/P rs121434575 Xeroderma pigmentosum complementation group G (XP-G) [MIM:278780]
ERCC5 P28715 VAR_020431 p.Val145Ile LB/B rs4987063 -
ERCC5 P28715 VAR_020432 p.Gln256Arg LB/B rs4150313 -
ERCC5 P28715 VAR_020433 p.Cys529Ser LB/B rs2227869 -
ERCC5 P28715 VAR_020434 p.Gln680Arg LB/B rs4987168 -
ERCC5 P28715 VAR_020435 p.Asn879Ser LB/B rs4150342 -
ERCC5 P28715 VAR_020436 p.Ala1119Val LB/B rs2227871 -
ERCC5 P28715 VAR_023120 p.His181Arg LB/B rs4150295 -
ERCC5 P28715 VAR_023121 p.Glu399Lys LB/B rs4150315 -
ERCC5 P28715 VAR_023122 p.Val590Ile LB/B rs4150318 -
ERCC5 P28715 VAR_023123 p.Val597Leu LB/B rs4150319 -
ERCC5 P28715 VAR_023124 p.Arg1009His LB/B rs4150387 -
ERCC5 P28715 VAR_023125 p.Gly1080Gln LB/B rs587778291 -
ERCC5 P28715 VAR_046373 p.Phe670Leu LB/B rs1803542 -
ERCC5 P28715 VAR_046374 p.Gly1053Arg LB/B rs9514066 -
ERCC5 P28715 VAR_046375 p.Gly1080Arg LB/B rs9514067 -
ERCC5 P28715 VAR_075773 p.Ala28Asp LP/P rs267607281 Xeroderma pigmentosum complementation group G (XP-G) [MIM:278780]
ERCC5 P28715 VAR_075774 p.Trp968Cys LP/P rs267607280 Xeroderma pigmentosum complementation group G (XP-G) [MIM:278780]
ERCC6 P0DP91 VAR_079009 p.Gly746Asp LP/P - Premature ovarian failure 11 (POF11) [MIM:616946]
ERCC6 P0DP91 VAR_079010 p.Arg850Lys LB/B rs4253072 -
ERCC6 P0DP91 VAR_079011 p.Val1056Ile LP/P - Premature ovarian failure 11 (POF11) [MIM:616946]

ERCC6 Q03468 VAR_001216 p.Lys255Thr LB/B - -
ERCC6 Q03468 VAR_001217 p.Gly399Asp LB/B rs2228528 -
ERCC6 Q03468 VAR_001218 p.Arg670Trp LP/P rs202080674 Cockayne syndrome B (CSB) [MIM:133540]
ERCC6 Q03468 VAR_001219 p.Trp851Arg LP/P rs368728467 Cockayne syndrome B (CSB) [MIM:133540]
ERCC6 Q03468 VAR_001220 p.Val957Gly LP/P - Cockayne syndrome B (CSB) [MIM:133540]
ERCC6 Q03468 VAR_001221 p.Pro1042Leu LP/P - Cockayne syndrome B (CSB) [MIM:133540]
ERCC6 Q03468 VAR_001222 p.Pro1095Arg LB/B rs4253208 -
ERCC6 Q03468 VAR_001223 p.Met1097Val LB/B rs2228526 -
ERCC6 Q03468 VAR_001224 p.Arg1213Gly LB/B rs2228527 -
ERCC6 Q03468 VAR_001225 p.Gln1413Arg LB/B rs2228529 -
ERCC6 Q03468 VAR_016301 p.Asp425Ala LB/B rs4253046 -
ERCC6 Q03468 VAR_016302 p.Gly446Asp LB/B rs4253047 -
ERCC6 Q03468 VAR_016303 p.Thr942Met LB/B rs2228525 -
ERCC6 Q03468 VAR_016304 p.Tyr1002Cys LB/B rs4253206 -
ERCC6 Q03468 VAR_016305 p.Arg1230Pro LB/B rs4253211 -
ERCC6 Q03468 VAR_016306 p.Val1308Leu LB/B rs2229761 -
ERCC6 Q03468 VAR_016307 p.Gly1322Val LB/B rs4253219 -
ERCC6 Q03468 VAR_016308 p.Gly1372Arg LB/B rs4253227 -
ERCC6 Q03468 VAR_016309 p.Gly1382Arg LB/B rs4253228 -
ERCC6 Q03468 VAR_016310 p.Gly1410Arg LB/B rs4253229 -
ERCC6 Q03468 VAR_016311 p.Thr1441Ile LB/B rs4253230 -
ERCC6 Q03468 VAR_036021 p.Pro591Ala US rs1184760254 A colorectal cancer sample
ERCC6 Q03468 VAR_036022 p.Arg652Leu US rs1365187961 A colorectal cancer sample
ERCC6 Q03468 VAR_036023 p.Arg1038Thr US - A breast cancer sample
ERCC6 Q03468 VAR_036024 p.Glu1119Gln US - A breast cancer sample
ERCC6 Q03468 VAR_036025 p.Glu1119Val US - A breast cancer sample
ERCC6 Q03468 VAR_037436 p.Thr1220Ile LB/B rs34704611 -
ERCC6 Q03468 VAR_037437 p.Asp1355Glu LB/B rs34917815 -
ERCC6 Q03468 VAR_054153 p.Arg134Trp LB/B rs148095899 -
ERCC6 Q03468 VAR_063511 p.Asn680Asp LP/P rs1554788393 Cockayne syndrome B (CSB) [MIM:133540]
ERCC6 Q03468 VAR_063512 p.Trp686Cys LP/P rs751292948 Cockayne syndrome B (CSB) [MIM:133540]
ERCC6 Q03468 VAR_063513 p.Ser687Leu LP/P rs1026438103 Cockayne syndrome B (CSB) [MIM:133540]
ERCC6 Q03468 VAR_063514 p.Leu871Pro LP/P - Cerebro-oculo-facio-skeletal syndrome 1 (COFS1) [MIM:214150]
ERCC6 Q03468 VAR_063515 p.Leu987Pro LP/P rs121917905 Cerebro-oculo-facio-skeletal syndrome 1 (COFS1) [MIM:214150]
ERCC6L2 Q5T890 VAR_039987 p.Val592Ala LB/B rs2274654 -
ERCC8 Q13216 VAR_016319 p.Tyr200Cys LB/B rs4647105 -
ERCC8 Q13216 VAR_025380 p.Ala160Val LP/P rs121434325 Cockayne syndrome A (CSA) [MIM:216400]
ERCC8 Q13216 VAR_025381 p.Ala205Pro LP/P rs121434326 Cockayne syndrome A (CSA) [MIM:216400]
ERCC8 Q13216 VAR_053392 p.Ser150Cys LB/B rs167037 -
ERCC8 Q13216 VAR_063507 p.Ala160Thr LP/P rs281875222 Cockayne syndrome A (CSA) [MIM:216400]
ERCC8 Q13216 VAR_063508 p.Trp194Cys LP/P rs281875223 Cockayne syndrome A (CSA) [MIM:216400]
ERCC8 Q13216 VAR_063509 p.Leu202Ser LP/P rs281875224 Cockayne syndrome A (CSA) [MIM:216400]
ERCC8 Q13216 VAR_063510 p.Asp266Gly LP/P rs281875225 Cockayne syndrome A (CSA) [MIM:216400]
ERCC8 Q13216 VAR_068177 p.Trp361Cys LP/P rs281875221 UV-sensitive syndrome 2 (UVSS2) [MIM:614621]
EREG O14944 VAR_033827 p.Arg147Gln LB/B rs35275884 -
EREG O14944 VAR_035833 p.Gly42Ala US - A breast cancer sample
ERF P50548 VAR_048947 p.Arg205His LB/B rs1053655 -

ERF P50548 VAR_070098 p.Arg65Gln LP/P rs587777009 Craniosynostosis 4 (CRS4) [MIM:600775]
ERF P50548 VAR_070099 p.Arg86Cys LP/P rs587777008 Craniosynostosis 4 (CRS4) [MIM:600775]
ERF P50548 VAR_078043 p.Tyr89Cys LP/P rs886041001 Chitayat syndrome (CHYTS) [MIM:617180]
ERGIC1 Q969X5 VAR_080480 p.Val98Glu US rs1554112524 Arthrogryposis multiplex congenita, neurogenic type (AMCN) [MIM:208100]
ERGIC3 Q9Y282 VAR_036553 p.Thr297Lys US - A colorectal cancer sample
ERGIC3 Q9Y282 VAR_048939 p.Ile113Leu LB/B rs35505616 -
ERI1 Q8IV48 VAR_018107 p.Leu16Pro LB/B rs2288672 -
ERI2 A8K979 VAR_043848 p.Ile206Thr US rs997506328 A colorectal cancer sample
ERICH1 Q86X53 VAR_035915 p.Leu365Phe US rs774405503 A colorectal cancer sample
ERICH1 Q86X53 VAR_050974 p.Arg403Ser LB/B rs1703879 -
ERICH3 Q5RHP9 VAR_035131 p.Pro264Ala LB/B rs11210490 -
ERICH3 Q5RHP9 VAR_035132 p.Arg555His LB/B rs696698 -
ERICH3 Q5RHP9 VAR_035133 p.Ile636Val LB/B rs17095653 -
ERICH3 Q5RHP9 VAR_035134 p.His691Arg LB/B rs2305549 -
ERICH3 Q5RHP9 VAR_035135 p.Leu1056Val LB/B rs11580409 -
ERICH3 Q5RHP9 VAR_035136 p.Val1528Met LB/B rs9326116 -
ERICH5 Q6P6B1 VAR_033176 p.Arg59His LB/B rs7833473 -
ERICH5 Q6P6B1 VAR_033177 p.Gln244Pro LB/B rs35368005 -
ERICH5 Q6P6B1 VAR_033178 p.His287Arg LB/B rs11994440 -
ERICH6 Q7L0X2 VAR_032893 p.Gly9Val LB/B rs16862795 -
ERICH6 Q7L0X2 VAR_032894 p.Gly433Arg LB/B rs11919896 -
ERICH6 Q7L0X2 VAR_062240 p.Arg604His LB/B rs34137455 -
ERICH6B Q5W0A0 VAR_039969 p.Glu63Asp LB/B rs12020217 -
ERICH6B Q5W0A0 VAR_039970 p.Glu99Gly LB/B rs12020731 -
ERICH6B Q5W0A0 VAR_039971 p.Ser174Thr LB/B rs17066954 -
ERICH6B Q5W0A0 VAR_039972 p.Glu178Lys LB/B rs3014939 -
ERICH6B Q5W0A0 VAR_039973 p.Leu303Pro LB/B rs11618506 -
ERICH6B Q5W0A0 VAR_039974 p.Thr427Ile LB/B rs749071 -
ERICH6B Q5W0A0 VAR_039975 p.Pro439Arg LB/B rs12429125 -
ERICH6B Q5W0A0 VAR_039976 p.His453Arg LB/B rs17066902 -
ERICH6B Q5W0A0 VAR_039977 p.Arg565Cys LB/B rs7327901 -
ERICH6B Q5W0A0 VAR_039978 p.Val653Phe LB/B rs1536207 -
ERLEC1 Q96DZ1 VAR_051493 p.Val318Leu LB/B rs2287345 -
ERLIN1 O75477 VAR_077847 p.Gly50Val LP/P rs876661322 Spastic paraplegia 62, autosomal recessive (SPG62) [MIM:615681]
ERLIN2 O94905 VAR_059140 p.Val71Ala LB/B rs2032066 -
ERMAP Q96PL5 VAR_025478 p.Ala4Val LB/B rs35757049 -
ERMAP Q96PL5 VAR_025479 p.His26Tyr LB/B rs33953680 -
ERMAP Q96PL5 VAR_025480 p.Gly35Ser LB/B rs146429994 -
ERMAP Q96PL5 VAR_025481 p.Glu47Lys LB/B rs56047316 -
ERMAP Q96PL5 VAR_025482 p.Gly57Arg LB/B rs56025238 -
ERMAP Q96PL5 VAR_025483 p.Pro60Ala LB/B rs56136737 -
ERMAP Q96PL5 VAR_025484 p.Arg81Gln LB/B rs368064875 -
ERMAP Q96PL5 VAR_025487 p.Cys259Arg LB/B rs35147822 -
ERMAP Q96PL5 VAR_025488 p.Gly263Glu LB/B rs34441268 -
ERMARD Q5T6L9 VAR_033300 p.Ser540Gly LB/B rs4716346 -
ERMARD Q5T6L9 VAR_070433 p.Ile377Asn LP/P rs398122410 Periventricular nodular heterotopia 6 (PVNH6) [MIM:615544]

ERMP1	Q7Z2K6	VAR_028945	p.Ser44Asn	LB/B	rs13284203	-
ERN1	O75460	VAR_040488	p.Asn244Ser	US	rs1397145500	A renal clear cell carcinoma sample
ERN1	O75460	VAR_040489	p.Val418Met	LB/B	rs55869215	-
ERN1	O75460	VAR_040490	p.Leu474Arg	US	rs186305118	A lung adenocarcinoma sample
ERN1	O75460	VAR_040491	p.Arg635Trp	US	rs146710304	A gastric adenocarcinoma sample
ERN1	O75460	VAR_040492	p.Asn700Ser	LB/B	rs918253870	-
ERN1	O75460	VAR_040493	p.Ser769Phe	US	-	A glioblastoma multiforme sample
ERN1	O75460	VAR_040494	p.Pro830Leu	US	rs1279653488	An ovarian serous carcinoma sample
ERN2	Q76MJ5	VAR_040495	p.Val69Ile	LB/B	rs9932495	-
ERN2	Q76MJ5	VAR_040496	p.Arg118Cys	LB/B	rs56117885	-
ERN2	Q76MJ5	VAR_040497	p.Arg184Cys	LB/B	rs34683474	-
ERN2	Q76MJ5	VAR_040498	p.Arg271Gln	LB/B	rs55772851	-
ERN2	Q76MJ5	VAR_040499	p.Ala318Thr	LB/B	rs56191901	-
ERN2	Q76MJ5	VAR_040500	p.Leu410Phe	LB/B	rs55687638	-
ERN2	Q76MJ5	VAR_040501	p.Ser487Thr	LB/B	rs26764	-
ERN2	Q76MJ5	VAR_040502	p.Leu504Phe	LB/B	rs56001432	-
ERN2	Q76MJ5	VAR_040503	p.Arg537Gln	LB/B	rs56176960	-
ERN2	Q76MJ5	VAR_040504	p.His858Tyr	LB/B	rs56137182	-
ERO1B	Q86YB8	VAR_019492	p.His465Gln	LB/B	rs1055851	-
ERO1B	Q86YB8	VAR_028012	p.Asp129Val	LB/B	rs2477599	-
ERP27	Q96DN0	VAR_052582	p.Phe52Leu	LB/B	rs35030722	-
ERRFI1	Q9UJM3	VAR_050975	p.Ile158Leu	LB/B	rs34974993	-
ERRFI1	Q9UJM3	VAR_063039	p.Asp109Asn	LB/B	rs34781518	-
ERV3-1	Q14264	VAR_017801	p.Thr90Ile	LB/B	rs6460219	-
ERV3-1	Q14264	VAR_017802	p.Cys192Tyr	LB/B	rs34639489	-
ERV3-1	Q14264	VAR_017803	p.Tyr236Cys	LB/B	rs71539632	-
ERV3-1	Q14264	VAR_017804	p.Asn481Ser	LB/B	rs4618579	-
ERV3-1	Q14264	VAR_017805	p.Leu522Pro	LB/B	rs1406592674	-
ERV3-1	Q14264	VAR_017806	p.Asn569Ser	LB/B	rs4717229	-
ERVK-18	O42043	VAR_018642	p.Cys97Tyr	LB/B	-	-
ERVK-18	O42043	VAR_018644	p.Val272Ile	LB/B	-	-
ERVK-18	O42043	VAR_018645	p.Val348Ile	LB/B	-	-
ERVK-18	O42043	VAR_018646	p.Val534Ile	LB/B	-	-
ERVW-1	Q9UQF0	VAR_018638	p.Val129Ala	LB/B	rs142852059	-
ERVW-1	Q9UQF0	VAR_018639	p.Arg138Gln	LB/B	rs55903518	-
ERVW-1	Q9UQF0	VAR_018640	p.Ser307Asn	LB/B	rs10266695	-
ERVW-1	Q9UQF0	VAR_018641	p.Ser477Phe	LB/B	rs141340741	-
ESAM	Q96AP7	VAR_049872	p.Arg273Cys	LB/B	rs12792040	-
ESCO1	Q5FWF5	VAR_022648	p.Thr221Met	LB/B	rs13381941	-
ESCO1	Q5FWF5	VAR_048167	p.Asn191Ser	LB/B	rs35087820	-
ESCO2	Q56NI9	VAR_022649	p.Trp539Gly	LP/P	rs80359868	Roberts syndrome (RBS) [MIM:268300]
ESCO2	Q56NI9	VAR_033840	p.Ala80Val	LB/B	rs4732748	-
ESCO2	Q56NI9	VAR_060994	p.Gln359Pro	LB/B	rs57479434	-
ESD	P10768	VAR_005202	p.Gly190Glu	LB/B	rs9778	-
ESD	P10768	VAR_022275	p.Gly257Asp	LB/B	rs15303	-
ESF1	Q9H501	VAR_024331	p.Ile550Thr	LB/B	rs3180370	-
ESF1	Q9H501	VAR_053082	p.Pro386Leu	LB/B	rs6079171	-
ESF1	Q9H501	VAR_053083	p.Ile824Leu	LB/B	rs34414644	-
ESPL1	Q14674	VAR_057703	p.Ser614Arg	LB/B	rs1318648	-

ESPL1 Q14674 VAR_057704 p.Arg699Gln LB/B rs34424268 -
ESPL1 Q14674 VAR_057705 p.Ile1136Val LB/B rs34130634 -
ESPL1 Q14674 VAR_057706 p.Thr1157Ala LB/B rs35428211 -
ESPL1 Q14674 VAR_057707 p.Gln1237His LB/B rs34396464 -
ESPL1 Q14674 VAR_057708 p.Lys1435Met LB/B rs1110719 -
ESPN B1AK53 VAR_043451 p.Arg322His LB/B rs3817911 -
ESPN B1AK53 VAR_043452 p.Tyr323Cys LB/B rs3817910 -
ESPN B1AK53 VAR_043453 p.Ser719Arg LP/P rs121908134 Deafness, autosomal recessive, 36, with or without vestibular involvement (DFNB36) [MIM:609006]
ESPN B1AK53 VAR_043454 p.Asp744Asn LP/P rs121908135 Deafness, autosomal recessive, 36, with or without vestibular involvement (DFNB36) [MIM:609006]
ESPN B1AK53 VAR_043455 p.Arg774Gln US rs121908136 Deafness, autosomal recessive, 36, with or without vestibular involvement (DFNB36) [MIM:609006]
ESPNL Q6ZVH7 VAR_037535 p.Arg167Gln LB/B rs34046909 -
ESPNL Q6ZVH7 VAR_037536 p.Ile568Val LB/B rs13033248 -
ESPNL Q6ZVH7 VAR_037537 p.Ala574Ser LB/B rs13006204 -
ESPNL Q6ZVH7 VAR_037538 p.Val761Ala LB/B rs4663845 -
ESPNL Q6ZVH7 VAR_037539 p.Gln829Arg LB/B rs10172220 -
ESR1 P03372 VAR_004671 p.Gly160Cys LB/B rs149308960 -
ESR1 P03372 VAR_004673 p.Gly400Val US - -
ESR1 P03372 VAR_018905 p.Gly77Ser LB/B rs9340773 -
ESR1 P03372 VAR_033028 p.His6Tyr US rs139960913 A breast cancer sample
ESR1 P03372 VAR_033029 p.Met264Ile US - A breast cancer sample
ESR1 P03372 VAR_070072 p.Gln375His LP/P rs397509428 Estrogen resistance (ESTRR) [MIM:615363]
ESR1 P03372 VAR_078516 p.Arg394His LP/P rs1131692059 Estrogen resistance (ESTRR) [MIM:615363]
ESR2 Q92731 VAR_081786 p.Gly84Val US rs1460814363 -
ESR2 Q92731 VAR_081788 p.Lys314Arg LP/P rs1567753148 Ovarian dysgenesis 8 (ODG8) [MIM:618187]
ESR2 Q92731 VAR_081789 p.Leu426Arg US rs1217623435 -
ESRP1 Q6NXG1 VAR_030073 p.Tyr196Cys LB/B rs2303454 -
ESRP1 Q6NXG1 VAR_080811 p.Leu259Val LP/P rs1554577402 Deafness, autosomal recessive, 109 (DFNB109) [MIM:618013]
ESRP2 Q9H6T0 VAR_030074 p.Ser111Leu LB/B rs12597504 -
ESRP2 Q9H6T0 VAR_030075 p.Ala528Val LB/B rs3743738 -
ESRP2 Q9H6T0 VAR_057245 p.Pro627Ser LB/B rs36054935 -
ESRRB O95718 VAR_043503 p.Ala110Val LP/P rs121909110 Deafness, autosomal recessive, 35 (DFNB35) [MIM:608565]
ESRRB O95718 VAR_043504 p.Leu320Pro LP/P - Deafness, autosomal recessive, 35 (DFNB35) [MIM:608565]
ESRRB O95718 VAR_043505 p.Val342Leu LP/P rs121909111 Deafness, autosomal recessive, 35 (DFNB35) [MIM:608565]
ESRRB O95718 VAR_043506 p.Leu347Pro LP/P - Deafness, autosomal recessive, 35 (DFNB35) [MIM:608565]
ESRRB O95718 VAR_043507 p.Pro386Ser LB/B rs61742642 -
ESRRB O95718 VAR_043508 p.Thr389Met US rs201714970 Deafness, autosomal recessive, 35 (DFNB35) [MIM:608565]
ESRRG P62508 VAR_019229 p.Thr50Met LB/B rs11572693 -
ESS2 Q96DF8 VAR_015117 p.Ala31Val LB/B rs113904207 -
ESS2 Q96DF8 VAR_015118 p.Val336Met LB/B rs17743887 -
ESS2 Q96DF8 VAR_015119 p.Ala423Val LB/B rs712965 -

ESX1	Q8N693	VAR_059352	p.Thr314Pro	LB/B	rs9697856	-	
ESYT1	Q9BSJ8	VAR_038190	p.Arg764Cys	LB/B	rs35075600	-	
ESYT2	A0FGR8	VAR_030725	p.Cys210Ser	LB/B	rs13233513	-	
ESYT2	A0FGR8	VAR_030726	p.Ser638Gly	LB/B	rs2305473	-	
ESYT3	A0FGR9	VAR_038117	p.Pro246Gln	LB/B	rs17857138	-	
ESYT3	A0FGR9	VAR_038118	p.Gly416Arg	LB/B	rs6772467	-	
ESYT3	A0FGR9	VAR_053835	p.Gly590Arg	LB/B	rs10935282	-	
ESYT3	A0FGR9	VAR_062173	p.Thr662Ser	LB/B	rs35537868	-	
ETAA1	Q9NY74	VAR_031053	p.Met221Thr	LB/B	rs13036061	-	
ETAA1	Q9NY74	VAR_031054	p.Ser389Asn	LB/B	rs3770657	-	
ETAA1	Q9NY74	VAR_031055	p.Pro715Leu	LB/B	rs3770656	-	
ETAA1	Q9NY74	VAR_031056	p.Pro771Ser	LB/B	rs3770655	-	
ETAA1	Q9NY74	VAR_035916	p.Glu50Asp	US	-	A colorectal cancer sample	
ETFA	P13804	VAR_002366	p.Gly116Arg	LP/P	rs119458971	Glutaric aciduria 2A (GA2A) [MIM:231680]	
ETFA	P13804	VAR_002367	p.Val157Gly	LP/P	rs119458969	Glutaric aciduria 2A (GA2A) [MIM:231680]	
ETFA	P13804	VAR_002368	p.Thr266Met	LP/P	rs119458970	Glutaric aciduria 2A (GA2A) [MIM:231680]	
ETFA	P13804	VAR_008547	p.Thr171Ile	LB/B	rs1801591	-	
ETFB	P38117	VAR_002369	p.Arg164Gln	LP/P	rs104894677	Glutaric aciduria 2B (GA2B) [MIM:231680]	
ETFB	P38117	VAR_008548	p.Thr154Met	LB/B	rs1130426	-	
ETFB	P38117	VAR_025804	p.Asp128Asn	LP/P	rs104894678	Glutaric aciduria 2B (GA2B) [MIM:231680]	
ETFDH	Q16134	VAR_036134	p.Val565Leu	US	rs769893690	A colorectal cancer sample	
ETFDH	Q16134	VAR_055711	p.His94Arg	LB/B	rs1140065	-	
ETFDH	Q16134	VAR_062966	p.Thr31Ile	LB/B	rs11559290	-	
ETFDH	Q16134	VAR_075438	p.Phe16Cys	LB/B	-	-	
ETFDH	Q16134	VAR_075439	p.Tyr49Cys	US	-	Glutaric aciduria 2C (GA2C) [MIM:231680]	
ETFDH	Q16134	VAR_075440	p.Ser82Phe	LP/P	rs887871605	Glutaric aciduria 2C (GA2C) [MIM:231680]	
ETFDH	Q16134	VAR_075441	p.Ser82Pro	LP/P	-	Glutaric aciduria 2C (GA2C) [MIM:231680]	
ETFDH	Q16134	VAR_075442	p.Ala84Thr	LP/P	rs121964954	Glutaric aciduria 2C (GA2C) [MIM:231680]	
ETFDH	Q16134	VAR_075443	p.His112Tyr	LP/P	-	Glutaric aciduria 2C (GA2C) [MIM:231680]	
ETFDH	Q16134	VAR_075444	p.Leu127His	LP/P	rs121964956	Glutaric aciduria 2C (GA2C) [MIM:231680]	
ETFDH	Q16134	VAR_075445	p.Leu138Arg	US	rs779896449	Glutaric aciduria 2C (GA2C) [MIM:231680]	
ETFDH	Q16134	VAR_075446	p.Arg175His	LP/P	rs121964955	Glutaric aciduria 2C (GA2C) [MIM:231680]	
ETFDH	Q16134	VAR_075447	p.Arg175Leu	LP/P	rs121964955	Glutaric aciduria 2C (GA2C) [MIM:231680]	
ETFDH	Q16134	VAR_075448	p.Asp218Asn	US	rs748289922	Glutaric aciduria 2C (GA2C) [MIM:231680]	
ETFDH	Q16134	VAR_075449	p.Gln222Pro	US	rs1482632936	Glutaric aciduria 2C (GA2C) [MIM:231680]	
ETFDH	Q16134	VAR_075450	p.Leu262Phe	US	-	Glutaric aciduria 2C (GA2C) [MIM:231680]	
ETFDH	Q16134	VAR_075451	p.Leu334Pro	US	rs377686388	Glutaric aciduria 2C (GA2C) [MIM:231680]	
ETFDH	Q16134	VAR_075452	p.His346Arg	US	rs1358691961	Glutaric aciduria 2C (GA2C) [MIM:231680]	
ETFDH	Q16134	VAR_075453	p.Leu377Pro	US	rs387907170	Glutaric aciduria 2C (GA2C) [MIM:231680]	
ETFDH	Q16134	VAR_075454	p.Arg452Lys	US	-	Glutaric aciduria 2C (GA2C) [MIM:231680]	
ETFDH	Q16134	VAR_075455	p.Pro456Leu	LP/P	rs398124152	Glutaric aciduria 2C (GA2C) [MIM:231680]	
ETFDH	Q16134	VAR_075456	p.Pro456Thr	LP/P	-	Glutaric aciduria 2C (GA2C) [MIM:231680]	
ETFDH	Q16134	VAR_075457	p.Pro483Leu	US	rs377656387	Glutaric aciduria 2C (GA2C) [MIM:231680]	
ETFDH	Q16134	VAR_075458	p.Pro562Leu	LP/P	rs993314323	Glutaric aciduria 2C (GA2C) [MIM:231680]	
ETFDH	Q16134	VAR_075459	p.Lys590Glu	LP/P	-	Glutaric aciduria 2C (GA2C) [MIM:231680]	
ETFDH	Q16134	VAR_075460	p.Gly611Glu	LP/P	rs761669036	Glutaric aciduria 2C (GA2C) [MIM:231680]	
ETHE1	O95571	VAR_023395	p.Tyr38Cys	LP/P	rs1555765564	Ethylmalonic encephalopathy (EE) [MIM:602473]	
ETHE1	O95571	VAR_023396	p.Thr136Ala	LP/P	rs1284200516	Ethylmalonic encephalopathy (EE) [MIM:602473]	
ETHE1	O95571	VAR_023397	p.Arg163Trp	LP/P	rs28940289	Ethylmalonic encephalopathy (EE) [MIM:602473]	

ETHE1	O95571	VAR_023398	p.Leu185Arg	LP/P	rs387906987	Ethylmalonic encephalopathy (EE) [MIM:602473]
ETHE1	O95571	VAR_069507	p.Leu55Pro	LP/P	rs182983506	Ethylmalonic encephalopathy (EE) [MIM:602473]
ETHE1	O95571	VAR_069508	p.Thr152Ile	LP/P	rs1317633085	Ethylmalonic encephalopathy (EE) [MIM:602473]
ETHE1	O95571	VAR_069509	p.Arg163Gln	LP/P	rs745656120	Ethylmalonic encephalopathy (EE) [MIM:602473]
ETHE1	O95571	VAR_069510	p.Thr164Lys	LP/P	rs1268640442	Ethylmalonic encephalopathy (EE) [MIM:602473]
ETHE1	O95571	VAR_069511	p.Asp196Asn	LP/P	rs763799125	Ethylmalonic encephalopathy (EE) [MIM:602473]
ETNK2	Q9NVF9	VAR_022145	p.Arg227Gln	LB/B	rs3737657	-
ETNPPL	Q8TBG4	VAR_032342	p.Ser185Pro	LB/B	rs1377210	-
ETV1	P50549	VAR_048948	p.Ser100Gly	LB/B	rs9639168	-
ETV2	O00321	VAR_071062	p.Asp90Asn	LB/B	rs2285419	-
ETV3L	Q6ZN32	VAR_039928	p.Ser19Ala	LB/B	rs16838078	-
ETV3L	Q6ZN32	VAR_039929	p.Gly263Ala	LB/B	rs12136960	-
ETV3L	Q6ZN32	VAR_039930	p.Met318Val	LB/B	rs12126791	-
ETV3L	Q6ZN32	VAR_048949	p.Arg151Trp	LB/B	rs12083811	-
ETV4	P43268	VAR_048950	p.Arg437Cys	LB/B	rs34260468	-
ETV4	P43268	VAR_069110	p.Phe195Ile	LB/B	rs150119757	-
ETV5	P41161	VAR_048951	p.Lys348Arg	LB/B	rs2228269	-
ETV6	P41212	VAR_073322	p.Pro214Leu	LP/P	rs724159947	Thrombocytopenia 5 (THC5) [MIM:616216]
ETV6	P41212	VAR_073323	p.Arg369Gln	LP/P	rs724159946	Thrombocytopenia 5 (THC5) [MIM:616216]
ETV6	P41212	VAR_073324	p.Arg399Cys	LP/P	rs724159945	Thrombocytopenia 5 (THC5) [MIM:616216]
ETV7	Q9Y603	VAR_020314	p.Pro212Leu	LB/B	rs2234079	-
ETV7	Q9Y603	VAR_048952	p.His138Tyr	LB/B	rs9470262	-
ETV7	Q9Y603	VAR_048953	p.Gly199Ser	LB/B	rs34306145	-
ETV7	Q9Y603	VAR_048954	p.Ala250Val	LB/B	rs2234080	-
ETV7	Q9Y603	VAR_059257	p.Ala86Thr	LB/B	rs9394345	-
EURL	Q9NYK6	VAR_011631	p.Gln117Arg	LB/B	rs1047976	-
EURL	Q9NYK6	VAR_011632	p.Asp136Glu	LB/B	rs1047978	-
EURL	Q9NYK6	VAR_054506	p.Asn115Lys	LB/B	rs2824495	-
EURL	Q9NYK6	VAR_054507	p.Gln195His	LB/B	rs8128004	-
EURL	Q9NYK6	VAR_054508	p.Tyr217His	LB/B	rs2824494	-
EVA1A	Q9H8M9	VAR_054077	p.Arg150His	LB/B	rs11126472	-
EVC	P57679	VAR_009942	p.Gln74Pro	LB/B	rs2291157	-
EVC	P57679	VAR_009943	p.Tyr258His	LB/B	rs6414624	-
EVC	P57679	VAR_009944	p.Ser307Pro	LP/P	rs121908426	Acrofacial dysostosis, Weyers type (WAD) [MIM:193530]
EVC	P57679	VAR_009945	p.Gly403Ser	LB/B	rs183114391	-
EVC	P57679	VAR_009946	p.Arg443Gln	LP/P	rs35953626	Ellis-van Creveld syndrome (EVC) [MIM:225500]
EVC	P57679	VAR_009947	p.Thr449Lys	LB/B	rs2302075	-
EVC	P57679	VAR_009948	p.Arg576Gln	LB/B	rs1383180	-
EVC	P57679	VAR_009949	p.Arg760Gln	LB/B	rs2279252	-
EVC	P57679	VAR_009950	p.Asp953Gly	LB/B	-	-
EVC	P57679	VAR_033852	p.Ala114Val	LB/B	rs16837598	-
EVC	P57679	VAR_033853	p.Thr372Met	LB/B	rs28483498	-
EVC	P57679	VAR_066447	p.Ser206Asn	LP/P	rs1017946059	Ellis-van Creveld syndrome (EVC) [MIM:225500]
EVC	P57679	VAR_066448	p.Leu623Pro	LP/P	rs1373632260	Ellis-van Creveld syndrome (EVC) [MIM:225500]
EVC2	Q86UK5	VAR_017209	p.Ile283Arg	LP/P	rs137852926	Ellis-van Creveld syndrome (EVC) [MIM:225500]
EVC2	Q86UK5	VAR_017210	p.Thr699Ala	LB/B	rs730469	-
EVC2	Q86UK5	VAR_017211	p.Arg950Trp	LP/P	rs137852928	Ellis-van Creveld syndrome (EVC) [MIM:225500]
EVC2	Q86UK5	VAR_035933	p.Ala630Ser	US	-	A colorectal cancer sample

EVC2	Q86UK5	VAR_035934	p.Leu994Val	US	-	A colorectal cancer sample
EVC2	Q86UK5	VAR_051089	p.Ser230Gly	LB/B	rs4689278	-
EVI2B	P34910	VAR_056871	p.Gly53Arg	LB/B	rs9903564	-
EVI5	O60447	VAR_028890	p.Ile336Val	LB/B	rs2391199	-
EVI5	O60447	VAR_028891	p.Gln612His	LB/B	rs11808092	-
EVI5	O60447	VAR_047753	p.Asp82Val	LB/B	rs1064580	-
EVL	Q9UI08	VAR_036464	p.Pro188Leu	US	rs367737727	A colorectal cancer sample
EVL	Q9UI08	VAR_036465	p.Pro247Leu	US	-	A colorectal cancer sample
EVPL	Q92817	VAR_024579	p.Asn49Ser	LB/B	rs397833081	-
EVPL	Q92817	VAR_033863	p.Gln433Arg	LB/B	rs2071192	-
EVPL	Q92817	VAR_057698	p.Tyr168Cys	LB/B	rs10445216	-
EVPL	Q92817	VAR_057699	p.Pro1814Ser	LB/B	rs7342883	-
EVPLL	A8MZ36	VAR_063399	p.Ser4Asn	LB/B	rs570145	-
EXD1	Q8NHP7	VAR_043676	p.Thr489Ala	LB/B	rs522063	-
EXD2	Q9NVH0	VAR_050980	p.Asp231Asn	LB/B	rs35010854	-
EXD2	Q9NVH0	VAR_050981	p.Gln518His	LB/B	rs8007859	-
EXD3	Q8N9H8	VAR_038945	p.Arg220Gln	LB/B	rs7389423	-
EXD3	Q8N9H8	VAR_062225	p.Arg20Gln	LB/B	rs13291830	-
EXO1	Q9UQ84	VAR_024966	p.Val27Ala	LB/B	rs1472620416	-
EXO1	Q9UQ84	VAR_024967	p.Val76Ile	LB/B	rs4149864	-
EXO1	Q9UQ84	VAR_024968	p.Arg93Gly	LB/B	rs4149865	-
EXO1	Q9UQ84	VAR_024969	p.Glu109Lys	LB/B	rs143546023	-
EXO1	Q9UQ84	VAR_024970	p.Ala137Ser	LB/B	rs147663824	-
EXO1	Q9UQ84	VAR_024971	p.Asn279Ser	LB/B	rs4149909	-
EXO1	Q9UQ84	VAR_024972	p.Asn299Ser	LB/B	rs4149910	-
EXO1	Q9UQ84	VAR_024973	p.His354Arg	LB/B	rs735943	-
EXO1	Q9UQ84	VAR_024974	p.Leu410Arg	LB/B	rs571928768	-
EXO1	Q9UQ84	VAR_024975	p.Asp428Asn	LB/B	rs4149962	-
EXO1	Q9UQ84	VAR_024976	p.Phe438Cys	LB/B	-	-
EXO1	Q9UQ84	VAR_024977	p.Thr439Met	LB/B	rs4149963	-
EXO1	Q9UQ84	VAR_024978	p.Ser456Tyr	LB/B	rs4149964	-
EXO1	Q9UQ84	VAR_024979	p.Val458Met	LB/B	rs4149965	-
EXO1	Q9UQ84	VAR_024980	p.Val460Leu	LB/B	rs4149966	-
EXO1	Q9UQ84	VAR_024981	p.Arg503Thr	LB/B	rs4149967	-
EXO1	Q9UQ84	VAR_024982	p.Glu589Lys	LB/B	rs1047840	-
EXO1	Q9UQ84	VAR_024983	p.Ser610Gly	LB/B	rs12122770	-
EXO1	Q9UQ84	VAR_024984	p.Arg634Gln	LB/B	rs4149978	-
EXO1	Q9UQ84	VAR_024985	p.Pro640Ala	LB/B	rs61736331	-
EXO1	Q9UQ84	VAR_024986	p.Pro640Ser	LB/B	rs61736331	-
EXO1	Q9UQ84	VAR_024987	p.Glu670Gly	LB/B	rs1776148	-
EXO1	Q9UQ84	VAR_024988	p.Arg723Cys	LB/B	rs1635498	-
EXO1	Q9UQ84	VAR_024989	p.His726Pro	LB/B	-	-
EXO1	Q9UQ84	VAR_024990	p.Pro757Leu	LB/B	rs9350	-
EXO1	Q9UQ84	VAR_024991	p.Gly759Glu	LB/B	rs4150001	-
EXO1	Q9UQ84	VAR_024992	p.Pro770Leu	LB/B	rs200622305	-
EXO1	Q9UQ84	VAR_024993	p.Ala827Val	LB/B	rs145975455	-
EXO1	Q9UQ84	VAR_077352	p.Ala153Val	LB/B	-	-
EXO5	Q9H790	VAR_035407	p.Asp115Asn	LB/B	rs1134586	-
EXO5	Q9H790	VAR_035408	p.Gly172Val	LB/B	rs11208299	-

EXOC2 Q96KP1 VAR_048956 p.Asn195Thr LB/B rs35600069 -
EXOC3-AS1 Q8N2X6 VAR_046197 p.Pro50Ser LB/B rs10035653 -
EXOC3-AS1 Q8N2X6 VAR_046198 p.Arg118Gly LB/B rs10035612 -
EXOC3L1 Q86VI1 VAR_036959 p.Gln561Glu LB/B rs9939768 -
EXOC3L1 Q86VI1 VAR_036960 p.Ser634Gly LB/B rs16957212 -
EXOC3L1 Q86VI1 VAR_037002 p.Tyr75Asn US rs117403380 A breast cancer sample
EXOC3L1 Q86VI1 VAR_037003 p.Ala514Asp US rs142958191 A breast cancer sample
EXOC3L2 Q2M3D2 VAR_036961 p.Asn173Asp LB/B rs10411314 -
EXOC3L4 Q17RC7 VAR_030273 p.Arg77Trp LB/B rs2297067 -
EXOC3L4 Q17RC7 VAR_030274 p.Asp93Glu LB/B rs2297066 -
EXOC3L4 Q17RC7 VAR_030275 p.Leu185His LB/B rs10131298 -
EXOC3L4 Q17RC7 VAR_030276 p.Gln675Arg LB/B rs729184 -
EXOC3L4 Q17RC7 VAR_062863 p.Gln685Glu LB/B rs744153 -
EXOC4 Q96A65 VAR_036292 p.Ser220Phe US - A colorectal cancer sample
EXOC4 Q96A65 VAR_036293 p.Ala599Thr US rs768209201 A colorectal cancer sample
EXOC5 O00471 VAR_048957 p.Glu10Asp LB/B rs35132458 -
EXOC6 Q8TAG9 VAR_044522 p.Thr396Ile LB/B rs1326331 -
EXOC6 Q8TAG9 VAR_044523 p.Leu523Val LB/B rs11187225 -
EXOC6 Q8TAG9 VAR_044524 p.Thr578Ile LB/B rs35647717 -
EXOC8 Q8IYI6 VAR_082196 p.Glu265Gly US rs483352868 -
EXOG Q9Y2C4 VAR_044320 p.Gly277Val LB/B rs1141223 -
EXOSC2 Q13868 VAR_080552 p.Gly30Val LP/P rs537467155 Short stature, hearing loss, retinitis pigmentosa, and distinctive facies (SHRF) [MIM:617763]
EXOSC2 Q13868 VAR_080553 p.Gly198Asp LP/P rs756204866 Short stature, hearing loss, retinitis pigmentosa, and distinctive facies (SHRF) [MIM:617763]
EXOSC3 Q9NQT5 VAR_054098 p.Tyr225His LB/B rs3208406 -
EXOSC3 Q9NQT5 VAR_068505 p.Gly31Ala LP/P rs387907196 Pontocerebellar hypoplasia 1B (PCH1B) [MIM:614678]
EXOSC3 Q9NQT5 VAR_068506 p.Asp132Ala LP/P rs141138948 Pontocerebellar hypoplasia 1B (PCH1B) [MIM:614678]
EXOSC3 Q9NQT5 VAR_068507 p.Ala139Pro LP/P rs387907195 Pontocerebellar hypoplasia 1B (PCH1B) [MIM:614678]
EXOSC3 Q9NQT5 VAR_068508 p.Trp238Arg LP/P rs672601332 Pontocerebellar hypoplasia 1B (PCH1B) [MIM:614678]
EXOSC3 Q9NQT5 VAR_074169 p.Val80Phe LB/B rs374550999 -
EXOSC5 Q9NQT4 VAR_030788 p.Thr5Met LB/B rs10853751 -
EXOSC5 Q9NQT4 VAR_051868 p.Cys33Trp LB/B rs34500671 -
EXOSC7 Q15024 VAR_014923 p.Val274Leu LB/B rs6794 -
EXOSC7 Q15024 VAR_032765 p.Arg169Gln LB/B rs34512144 -
EXOSC8 Q96B26 VAR_072558 p.Ala2Val LP/P rs606231285 Pontocerebellar hypoplasia 1C (PCH1C) [MIM:616081]
EXOSC8 Q96B26 VAR_072559 p.Ser272Thr LP/P rs36027220 Pontocerebellar hypoplasia 1C (PCH1C) [MIM:616081]
EXOSC9 Q06265 VAR_014924 p.Ser425Thr LB/B rs1051881 -
EXOSC9 Q06265 VAR_051867 p.Ile366Val LB/B rs1803183 -
EXOSC9 Q06265 VAR_081052 p.Leu14Pro LP/P rs139632595 Pontocerebellar hypoplasia 1D (PCH1D) [MIM:618065]
EXPH5 Q8NEV8 VAR_030538 p.Arg19Gly LB/B rs2640738 -
EXPH5 Q8NEV8 VAR_030539 p.Arg118Leu LB/B rs3741046 -

EXPH5 Q8NEV8 VAR_030540 p.Glu137Val LB/B rs2640785 -
EXPH5 Q8NEV8 VAR_030541 p.Arg328Gln LB/B rs11212684 -
EXPH5 Q8NEV8 VAR_030542 p.Met512Leu LB/B rs17108127 -
EXPH5 Q8NEV8 VAR_030543 p.Val525Phe LB/B rs12146448 -
EXPH5 Q8NEV8 VAR_030544 p.Ser676Asn LB/B rs2846412 -
EXPH5 Q8NEV8 VAR_030545 p.Asp777Asn LB/B rs3741048 -
EXPH5 Q8NEV8 VAR_030546 p.Leu853Pro LB/B rs10749920 -
EXPH5 Q8NEV8 VAR_030547 p.Asn892Tyr LB/B rs10890850 -
EXPH5 Q8NEV8 VAR_030548 p.Val899Ala LB/B rs17108112 -
EXPH5 Q8NEV8 VAR_030549 p.Asp1240Asn LB/B rs11828459 -
EXPH5 Q8NEV8 VAR_030550 p.Cys1311Arg LB/B rs877474 -
EXPH5 Q8NEV8 VAR_030551 p.Gly1663Arg LB/B rs2640779 -
EXPH5 Q8NEV8 VAR_030552 p.Asn1967Asp LB/B rs1943382 -
EXPH5 Q8NEV8 VAR_057117 p.Met1147Ile LB/B rs34012545 -
EXPH5 Q8NEV8 VAR_057118 p.Ser1236Ala LB/B rs35520914 -
EXPH5 Q8NEV8 VAR_057119 p.Thr1343Ala LB/B rs34978242 -
EXPH5 Q8NEV8 VAR_057120 p.Glu1656Lys LB/B rs35083468 -
EXPH5 Q8NEV8 VAR_057121 p.Ile1735Phe LB/B rs35717245 -
EXT1 Q16394 VAR_002370 p.Arg280Gly LP/P rs1554601483 Hereditary multiple exostoses 1 (EXT1) [MIM:133700]
EXT1 Q16394 VAR_002371 p.Arg280Ser LP/P rs1563659325 Hereditary multiple exostoses 1 (EXT1) [MIM:133700]
EXT1 Q16394 VAR_002372 p.Gly339Asp LP/P rs119103288 Hereditary multiple exostoses 1 (EXT1) [MIM:133700]
EXT1 Q16394 VAR_002373 p.Arg340Cys LP/P rs119103290 Hereditary multiple exostoses 1 (EXT1) [MIM:133700]
EXT1 Q16394 VAR_002374 p.Arg340His LP/P rs119103287 Hereditary multiple exostoses 1 (EXT1) [MIM:133700]
EXT1 Q16394 VAR_002375 p.Arg340Leu LP/P rs119103287 Hereditary multiple exostoses 1 (EXT1) [MIM:133700]
EXT1 Q16394 VAR_002376 p.Arg340Ser LP/P - Hereditary multiple exostoses 1 (EXT1) [MIM:133700]
EXT1 Q16394 VAR_012815 p.Gln27Lys LP/P - Hereditary multiple exostoses 1 (EXT1) [MIM:133700]
EXT1 Q16394 VAR_012816 p.Asp164His LP/P - Hereditary multiple exostoses 1 (EXT1) [MIM:133700]
EXT1 Q16394 VAR_012820 p.Asn316Ser LP/P - Chondrosarcoma (CHDSA) [MIM:215300]
EXT1 Q16394 VAR_012821 p.Ala486Val LP/P rs188859975 Hereditary multiple exostoses 1 (EXT1) [MIM:133700]
EXT1 Q16394 VAR_012822 p.Pro496Leu LP/P - Hereditary multiple exostoses 1 (EXT1) [MIM:133700]
EXT2 Q93063 VAR_002378 p.Asp227Asn LP/P rs121918280 Hereditary multiple exostoses 2 (EXT2) [MIM:133701]
EXT2 Q93063 VAR_012823 p.Cys85Arg LP/P - Hereditary multiple exostoses 2 (EXT2) [MIM:133701]
EXT2 Q93063 VAR_012824 p.Leu152Arg LP/P - Hereditary multiple exostoses 2 (EXT2) [MIM:133701]
EXT2 Q93063 VAR_012825 p.Arg179Ser LP/P - Hereditary multiple exostoses 2 (EXT2) [MIM:133701]
EXT2 Q93063 VAR_012826 p.Ala202Val LP/P rs771803942 Hereditary multiple exostoses 2 (EXT2) [MIM:133701]
EXT2 Q93063 VAR_012827 p.Arg223Pro LP/P rs764379119 Hereditary multiple exostoses 2 (EXT2) [MIM:133701]
EXT2 Q93063 VAR_012828 p.Ile380Thr LP/P - Hereditary multiple exostoses 2 (EXT2) [MIM:133701]
EXT2 Q93063 VAR_012829 p.Glu576Lys LB/B rs373582542 -
EXT2 Q93063 VAR_033921 p.Met42Val LB/B rs4755779 -

EXT2 Q93063 VAR_076469 p.Met87Arg LP/P rs140075817 Seizures, scoliosis, and macrocephaly/microcephaly syndrome (SSMS) [MIM:616682]
EXT2 Q93063 VAR_076470 p.Arg95Cys LP/P rs376292686 Seizures, scoliosis, and macrocephaly/microcephaly syndrome (SSMS) [MIM:616682]
EXTL1 Q92935 VAR_012830 p.His379Asn LB/B rs2736831 -
EXTL1 Q92935 VAR_049228 p.Arg163His LB/B rs34277678 -
EXTL3 O43909 VAR_049229 p.Leu706Pro LB/B rs2269452 -
EXTL3 O43909 VAR_061194 p.Ala550Val LB/B rs35781576 -
EXTL3 O43909 VAR_079089 p.Arg339Trp LP/P rs747676107 Immunoskeletal dysplasia with neurodevelopmental abnormalities (ISDNA) [MIM:617425]
EXTL3 O43909 VAR_079090 p.Val442Leu LB/B rs116659770 -
EXTL3 O43909 VAR_079091 p.Pro461Leu LP/P rs554294508 Immunoskeletal dysplasia with neurodevelopmental abnormalities (ISDNA) [MIM:617425]
EXTL3 O43909 VAR_079092 p.Arg513Cys LP/P rs1332006145 Immunoskeletal dysplasia with neurodevelopmental abnormalities (ISDNA) [MIM:617425]
EXTL3 O43909 VAR_079093 p.Asn657Ser LP/P rs770842408 Immunoskeletal dysplasia with neurodevelopmental abnormalities (ISDNA) [MIM:617425]
EXTL3 O43909 VAR_079094 p.Tyr670Asp LP/P - Immunoskeletal dysplasia with neurodevelopmental abnormalities (ISDNA) [MIM:617425]
EXTL3 O43909 VAR_080762 p.Ser646Cys US - -
EYA1 Q99502 VAR_005203 p.Ser487Pro LP/P rs121909200 Branchiootorenal syndrome 1 (BOR1) [MIM:113650]
EYA1 Q99502 VAR_005204 p.Leu505Arg LP/P rs121909201 Branchiootorenal syndrome 1 (BOR1) [MIM:113650]
EYA1 Q99502 VAR_016864 p.Glu363Lys LP/P rs121909198 Anterior segment anomalies with or without cataract (ASA) [MIM:602588]
EYA1 Q99502 VAR_016865 p.Gly426Ser LP/P rs121909199 Branchiootorenal syndrome 1 (BOR1) [MIM:113650]
EYA1 Q99502 VAR_016866 p.Asp429Gly LP/P - Branchiootorenal syndrome 1 (BOR1) [MIM:113650]
EYA1 Q99502 VAR_016867 p.Arg440Gln LP/P rs121909196 Branchiootorenal syndrome 1 (BOR1) [MIM:113650]
EYA1 Q99502 VAR_016868 p.Arg547Gly LP/P rs121909197 Anterior segment anomalies with or without cataract (ASA) [MIM:602588]
EYA1 Q99502 VAR_016869 p.Leu583Pro LP/P rs397517920 Branchiootorenal syndrome 1 (BOR1) [MIM:113650]
EYA1 Q99502 VAR_024439 p.Pro20Ala LB/B rs1445404 -
EYA1 Q99502 VAR_044452 p.Ser242Gly LP/P rs191838840 Branchiootic syndrome 1 (BOS1) [MIM:602588]
EYA1 Q99502 VAR_064942 p.Pro95Ser LP/P - Branchiootorenal syndrome 1 (BOR1) [MIM:113650]
EYA1 Q99502 VAR_064943 p.Gly140Ser LP/P - Branchiootorenal syndrome 1 (BOR1) [MIM:113650]
EYA1 Q99502 VAR_064944 p.Glu363Val LP/P - Branchiootorenal syndrome 1 (BOR1) [MIM:113650]
EYA1 Q99502 VAR_064945 p.Leu514Pro LP/P rs112340154 Branchiootorenal syndrome 1 (BOR1) [MIM:113650]
EYA1 Q99502 VAR_064946 p.Tyr527Cys LP/P - Branchiootorenal syndrome 1 (BOR1) [MIM:113650]
EYA1 Q99502 VAR_064947 p.Met569Thr LP/P - Branchiootorenal syndrome 1 (BOR1) [MIM:113650]
EYA1 Q99502 VAR_070033 p.Glu41Lys LB/B rs561111097 -
EYA2 O00167 VAR_048964 p.Pro83Ser LB/B rs2275596 -
EYA2 O00167 VAR_048965 p.Thr238Ala LB/B rs866936 -
EYA4 O95677 VAR_022932 p.Gly277Ser LB/B rs9493627 -
EYA4 O95677 VAR_036248 p.Leu152Arg US - A colorectal cancer sample

EYA4	O95677	VAR_036249	p.Asp301Asn	US	rs779172192	A colorectal cancer sample
EYA4	O95677	VAR_074570	p.Gly171Arg	LP/P	rs1471362858	Deafness, autosomal dominant, 10 (DFNA10) [MIM:601316]
EYA4	O95677	VAR_074571	p.Thr548Arg	LP/P	-	Deafness, autosomal dominant, 10 (DFNA10) [MIM:601316]
EYS	Q5T1H1	VAR_035301	p.Thr120Met	LB/B	rs12193967	-
EYS	Q5T1H1	VAR_043561	p.Leu852Pro	LB/B	rs9294631	-
EYS	Q5T1H1	VAR_063437	p.Pro94Gln	LB/B	rs111947397	-
EYS	Q5T1H1	VAR_063438	p.Val112Ile	LB/B	rs112609906	-
EYS	Q5T1H1	VAR_063439	p.Thr135Leu	US	-	-
EYS	Q5T1H1	VAR_063440	p.Val136Phe	LB/B	rs543011021	-
EYS	Q5T1H1	VAR_063441	p.Ser326Asn	LB/B	rs112822256	-
EYS	Q5T1H1	VAR_063442	p.Lys532Asn	LB/B	rs61753611	-
EYS	Q5T1H1	VAR_063443	p.Arg551Leu	LB/B	-	-
EYS	Q5T1H1	VAR_063444	p.Gln571Arg	LB/B	rs61753610	-
EYS	Q5T1H1	VAR_063445	p.Gly618Ser	LP/P	rs142450703	Retinitis pigmentosa 25 (RP25) [MIM:602772]
EYS	Q5T1H1	VAR_063446	p.Gly631Ser	LB/B	rs9342464	-
EYS	Q5T1H1	VAR_063447	p.Glu641Val	LB/B	rs17411795	-
EYS	Q5T1H1	VAR_063448	p.Asn745Ser	US	rs201652272	Retinitis pigmentosa 25 (RP25) [MIM:602772]
EYS	Q5T1H1	VAR_063449	p.Val834Ile	LB/B	rs112464110	-
EYS	Q5T1H1	VAR_063450	p.Lys938Arg	LB/B	rs367857088	-
EYS	Q5T1H1	VAR_063451	p.Thr1110Ser	LP/P	rs143327210	Retinitis pigmentosa 25 (RP25) [MIM:602772]
EYS	Q5T1H1	VAR_063452	p.Asn1163Lys	LB/B	rs150951106	-
EYS	Q5T1H1	VAR_063453	p.Cys1176Arg	LP/P	-	Retinitis pigmentosa 25 (RP25) [MIM:602772]
EYS	Q5T1H1	VAR_063454	p.Ile1232Phe	LP/P	rs190009374	Retinitis pigmentosa 25 (RP25) [MIM:602772]
EYS	Q5T1H1	VAR_063455	p.Ile1263Val	LB/B	rs17404123	-
EYS	Q5T1H1	VAR_063456	p.Gln1325Glu	LB/B	rs12663622	-
EYS	Q5T1H1	VAR_063457	p.Ile1361Val	LB/B	rs17403955	-
EYS	Q5T1H1	VAR_063458	p.Lys1365Glu	LB/B	rs16895519	-
EYS	Q5T1H1	VAR_063459	p.Leu1419Ser	LB/B	rs624851	-
EYS	Q5T1H1	VAR_063460	p.Ile1451Thr	LB/B	rs62415828	-
EYS	Q5T1H1	VAR_063461	p.Arg1515Trp	LB/B	rs62415827	-
EYS	Q5T1H1	VAR_063462	p.Ser1517Gly	LB/B	rs62415826	-
EYS	Q5T1H1	VAR_063463	p.Asp1662Val	LB/B	rs147641443	-
EYS	Q5T1H1	VAR_063464	p.Thr1664Ile	LB/B	rs561830314	-
EYS	Q5T1H1	VAR_063465	p.Asp1682Tyr	LP/P	rs75831552	Retinitis pigmentosa 25 (RP25) [MIM:602772]
EYS	Q5T1H1	VAR_063466	p.Pro1739Leu	LB/B	-	-
EYS	Q5T1H1	VAR_063467	p.Glu1747Gly	LP/P	rs535663619	Retinitis pigmentosa 25 (RP25) [MIM:602772]
EYS	Q5T1H1	VAR_063468	p.Leu1748Phe	LB/B	rs57312007	-
EYS	Q5T1H1	VAR_063469	p.Trp1837Ser	LB/B	rs199689193	-
EYS	Q5T1H1	VAR_063470	p.Leu1869Met	LP/P	-	Retinitis pigmentosa 25 (RP25) [MIM:602772]
EYS	Q5T1H1	VAR_063471	p.Leu1873Val	LB/B	rs16895517	-
EYS	Q5T1H1	VAR_063472	p.Asn1902Ile	LB/B	rs9353806	-
EYS	Q5T1H1	VAR_063473	p.Ser1915Gly	LB/B	rs188093810	-
EYS	Q5T1H1	VAR_063474	p.Thr1987Pro	LB/B	rs1278246029	-
EYS	Q5T1H1	VAR_063475	p.Thr1993Ala	LB/B	rs115066356	-
EYS	Q5T1H1	VAR_063476	p.Ile1999Val	LB/B	rs893294562	-
EYS	Q5T1H1	VAR_063477	p.Val2040Asp	LB/B	rs201580493	-
EYS	Q5T1H1	VAR_063478	p.Cys2139Tyr	LP/P	rs749909863	Retinitis pigmentosa 25 (RP25) [MIM:602772]

EYS	Q5T1H1	VAR_063479	p.Asn2151Ser	LB/B	rs141603172	-
EYS	Q5T1H1	VAR_063480	p.Leu2189Pro	LP/P	-	Retinitis pigmentosa 25 (RP25) [MIM:602772]
EYS	Q5T1H1	VAR_063481	p.Ser2211Leu	US	rs145623359	Retinitis pigmentosa 25 (RP25) [MIM:602772]
EYS	Q5T1H1	VAR_063482	p.Arg2326Gln	LB/B	rs4710457	-
EYS	Q5T1H1	VAR_063483	p.Ser2556Cys	LB/B	rs66462731	-
EYS	Q5T1H1	VAR_063484	p.His2599Arg	LB/B	rs74636274	-
EYS	Q5T1H1	VAR_063485	p.Ala2757Pro	LB/B	rs1250317776	-
EYS	Q5T1H1	VAR_063486	p.Ala2829Thr	LP/P	rs111991705	Retinitis pigmentosa 25 (RP25) [MIM:602772]
EYS	Q5T1H1	VAR_063487	p.Thr2831Ile	LB/B	rs144513453	-
EYS	Q5T1H1	VAR_063488	p.Cys2911Tyr	LP/P	-	Retinitis pigmentosa 25 (RP25) [MIM:602772]
EYS	Q5T1H1	VAR_063489	p.Gly2928Glu	LP/P	-	Retinitis pigmentosa 25 (RP25) [MIM:602772]
EYS	Q5T1H1	VAR_064417	p.Trp1484Arg	LP/P	rs1260400598	Retinitis pigmentosa 25 (RP25) [MIM:602772]
EYS	Q5T1H1	VAR_064418	p.Gly2017Val	LP/P	rs868349465	Retinitis pigmentosa 25 (RP25) [MIM:602772]
EYS	Q5T1H1	VAR_064419	p.Glu2503Lys	LP/P	rs768964978	Retinitis pigmentosa 25 (RP25) [MIM:602772]
EYS	Q5T1H1	VAR_064420	p.Gln2945Glu	LP/P	-	Retinitis pigmentosa 25 (RP25) [MIM:602772]
EZH2	Q15910	VAR_055795	p.Asp185His	LB/B	rs2302427	-
EZH2	Q15910	VAR_067228	p.Tyr641Cys	US	-	A patient with diffuse large B-cell lymphoma
EZH2	Q15910	VAR_067229	p.Tyr641Phe	LB/B	rs267601394	-
EZH2	Q15910	VAR_067230	p.Tyr641His	LB/B	rs267601395	-
EZH2	Q15910	VAR_067231	p.Tyr641Asn	LB/B	rs267601395	-
EZH2	Q15910	VAR_067232	p.Tyr641Ser	LB/B	rs267601394	-
EZH2	Q15910	VAR_067233	p.Arg685His	US	rs1554481435	A patient with chronic myelomonocytic leukemia
EZH2	Q15910	VAR_067595	p.Pro132Ser	LP/P	rs193921148	Weaver syndrome (WVS) [MIM:277590]
EZH2	Q15910	VAR_067597	p.His689Tyr	LP/P	rs193921147	Weaver syndrome (WVS) [MIM:277590]
EZH2	Q15910	VAR_078320	p.Tyr133Cys	LP/P	-	Weaver syndrome (WVS) [MIM:277590]
EZH2	Q15910	VAR_078321	p.Met134Thr	LP/P	-	Weaver syndrome (WVS) [MIM:277590]
EZH2	Q15910	VAR_078322	p.Lys156Glu	LP/P	-	Weaver syndrome (WVS) [MIM:277590]
EZH2	Q15910	VAR_078323	p.His279Arg	LP/P	-	Weaver syndrome (WVS) [MIM:277590]
EZH2	Q15910	VAR_078324	p.Cys571Trp	US	-	-
EZH2	Q15910	VAR_078325	p.Val621Met	US	rs587783625	Weaver syndrome (WVS) [MIM:277590]
EZH2	Q15910	VAR_078326	p.Tyr658Asn	LP/P	-	Weaver syndrome (WVS) [MIM:277590]
EZH2	Q15910	VAR_078327	p.Ala677Gly	LB/B	rs1057519833	-
EZH2	Q15910	VAR_078328	p.Ala677Thr	LP/P	rs397515547	Weaver syndrome (WVS) [MIM:277590]
EZH2	Q15910	VAR_078329	p.Arg679Cys	LP/P	rs587783626	Weaver syndrome (WVS) [MIM:277590]
EZH2	Q15910	VAR_078330	p.Arg685Cys	US	-	-
EZH2	Q15910	VAR_078331	p.Ser690Leu	LP/P	-	Weaver syndrome (WVS) [MIM:277590]
EZH2	Q15910	VAR_078332	p.Tyr726Asp	US	-	-
EZH2	Q15910	VAR_078334	p.Tyr736Cys	LP/P	-	Weaver syndrome (WVS) [MIM:277590]
EZH2	Q15910	VAR_078335	p.Glu740Lys	US	rs397515548	Weaver syndrome (WVS) [MIM:277590]
EZHIP	Q86X51	VAR_038938	p.Arg470Lys	LB/B	rs1875755	-
EZR	P15311	VAR_015112	p.Leu532Val	LB/B	-	-
EZR	P15311	VAR_030572	p.Arg180Cys	LB/B	rs3103004	-
EZR	P15311	VAR_030573	p.Ala494Pro	LB/B	rs2230143	-
F10	P00742	VAR_014162	p.Leu7Ile	LB/B	rs5963	-
F10	P00742	VAR_014163	p.Gln30His	LB/B	rs5961	-
F10	P00742	VAR_020176	p.Ala152Thr	LB/B	rs3211772	-
F10	P00742	VAR_020177	p.Gly192Arg	LB/B	rs3211783	-
F10	P00742	VAR_065428	p.Glu47Gly	LP/P	rs121964943	Factor X deficiency (FA10D) [MIM:227600]
F10	P00742	VAR_065429	p.Gly51Val	LP/P	rs751782758	Factor X deficiency (FA10D) [MIM:227600]

F10	P00742	VAR_065430	p.Glu54Gly	LP/P	rs121964944	Factor X deficiency (FA10D) [MIM:227600]
F10	P00742	VAR_065431	p.Glu54Lys	LP/P	rs121964939	Factor X deficiency (FA10D) [MIM:227600]
F10	P00742	VAR_065432	p.Glu72Gln	LP/P	rs121964945	Factor X deficiency (FA10D) [MIM:227600]
F10	P00742	VAR_065433	p.Glu91Lys	LP/P	rs1477329751	Factor X deficiency (FA10D) [MIM:227600]
F10	P00742	VAR_065434	p.Glu142Lys	US	rs61753266	Factor X deficiency (FA10D) [MIM:227600]
F10	P00742	VAR_065435	p.Cys149Tyr	LP/P	-	Factor X deficiency (FA10D) [MIM:227600]
F10	P00742	VAR_065436	p.Cys151Tyr	LP/P	-	Factor X deficiency (FA10D) [MIM:227600]
F10	P00742	VAR_065437	p.Gly289Arg	LP/P	rs121964946	Factor X deficiency (FA10D) [MIM:227600]
F10	P00742	VAR_065438	p.Glu304Lys	LP/P	rs747292771	Factor X deficiency (FA10D) [MIM:227600]
F10	P00742	VAR_065439	p.Asp322Asn	LP/P	rs121964942	Factor X deficiency (FA10D) [MIM:227600]
F10	P00742	VAR_065440	p.Arg327Trp	LP/P	rs770119164	Factor X deficiency (FA10D) [MIM:227600]
F10	P00742	VAR_065441	p.Val338Met	LP/P	rs121964947	Factor X deficiency (FA10D) [MIM:227600]
F10	P00742	VAR_065442	p.Glu350Lys	LP/P	rs372309538	Factor X deficiency (FA10D) [MIM:227600]
F10	P00742	VAR_065443	p.Thr358Met	LP/P	rs768222784	Factor X deficiency (FA10D) [MIM:227600]
F10	P00742	VAR_065444	p.Gly363Ser	LP/P	-	Factor X deficiency (FA10D) [MIM:227600]
F10	P00742	VAR_065445	p.Arg366Cys	LP/P	rs104894392	Factor X deficiency (FA10D) [MIM:227600]
F10	P00742	VAR_065446	p.Ser374Pro	LP/P	rs121964941	Factor X deficiency (FA10D) [MIM:227600]
F10	P00742	VAR_065447	p.Pro383Ser	LP/P	rs121964940	Factor X deficiency (FA10D) [MIM:227600]
F10	P00742	VAR_065448	p.Cys390Phe	LP/P	rs199778916	Factor X deficiency (FA10D) [MIM:227600]
F10	P00742	VAR_065449	p.Cys404Arg	LP/P	-	Factor X deficiency (FA10D) [MIM:227600]
F10	P00742	VAR_065450	p.Gly406Ser	LP/P	rs376163818	Factor X deficiency (FA10D) [MIM:227600]
F10	P00742	VAR_065451	p.Gly420Arg	LP/P	rs750759634	Factor X deficiency (FA10D) [MIM:227600]
F10	P00742	VAR_065452	p.Lys448Asn	LP/P	-	Factor X deficiency (FA10D) [MIM:227600]
F10	P00742	VAR_072751	p.Val382Ala	LP/P	-	Factor X deficiency (FA10D) [MIM:227600]
F10	P00742	VAR_072752	p.Gly421Asp	LP/P	rs758726161	Factor X deficiency (FA10D) [MIM:227600]
F10	P00742	VAR_075213	p.Gly262Asp	US	rs1393705267	Factor X deficiency (FA10D) [MIM:227600]
F11	P03951	VAR_006622	p.Phe301Leu	LP/P	rs121965064	Factor XI deficiency (FA11D) [MIM:612416]
F11	P03951	VAR_011774	p.Pro66Leu	LB/B	rs5968	-
F11	P03951	VAR_011775	p.Gln244Arg	LB/B	rs5969	-
F11	P03951	VAR_011776	p.Ile308Phe	LB/B	rs5972	-
F11	P03951	VAR_011777	p.Cys339Phe	LB/B	rs5967	-
F11	P03951	VAR_011778	p.Trp399Arg	LB/B	rs1800439	-
F11	P03951	VAR_012085	p.Asp34His	LP/P	rs281875267	Factor XI deficiency (FA11D) [MIM:612416]
F11	P03951	VAR_012086	p.Trp246Cys	LP/P	rs281875279	Factor XI deficiency (FA11D) [MIM:612416]
F11	P03951	VAR_012087	p.Ser266Asn	LP/P	rs145168351	Factor XI deficiency (FA11D) [MIM:612416]
F11	P03951	VAR_012088	p.Leu320Pro	LP/P	rs281875268	Factor XI deficiency (FA11D) [MIM:612416]
F11	P03951	VAR_012089	p.Thr322Ile	LP/P	rs281875269	Factor XI deficiency (FA11D) [MIM:612416]
F11	P03951	VAR_012090	p.Arg326Cys	LP/P	rs28934608	Factor XI deficiency (FA11D) [MIM:612416]
F11	P03951	VAR_012091	p.Glu341Lys	LP/P	rs281875270	Factor XI deficiency (FA11D) [MIM:612416]
F11	P03951	VAR_012092	p.Thr404Asn	LP/P	rs121965067	Factor XI deficiency (FA11D) [MIM:612416]
F11	P03951	VAR_012093	p.Ala430Val	LP/P	rs121965068	Factor XI deficiency (FA11D) [MIM:612416]
F11	P03951	VAR_012094	p.Phe460Val	LP/P	rs121965065	Factor XI deficiency (FA11D) [MIM:612416]
F11	P03951	VAR_012095	p.Thr493Ile	LP/P	rs1554083754	Factor XI deficiency (FA11D) [MIM:612416]
F11	P03951	VAR_012096	p.Ser594Arg	LP/P	rs28934609	Factor XI deficiency (FA11D) [MIM:612416]
F11	P03951	VAR_054894	p.Cys46Phe	LP/P	rs281875271	Factor XI deficiency (FA11D) [MIM:612416]
F11	P03951	VAR_054895	p.Cys56Arg	LP/P	rs121965069	Factor XI deficiency (FA11D) [MIM:612416]
F11	P03951	VAR_054896	p.Lys101Arg	LP/P	rs281875272	Factor XI deficiency (FA11D) [MIM:612416]
F11	P03951	VAR_054897	p.Tyr151Cys	LP/P	rs281875273	Factor XI deficiency (FA11D) [MIM:612416]
F11	P03951	VAR_054898	p.Cys255Tyr	LP/P	rs281875277	Factor XI deficiency (FA11D) [MIM:612416]

F11	P03951	VAR_054899	p.Gly263Glu	LP/P	rs281875274	Factor XI deficiency (FA11D) [MIM:612416]
F11	P03951	VAR_054900	p.Lys270Ile	LP/P	rs121965070	Factor XI deficiency (FA11D) [MIM:612416]
F11	P03951	VAR_054901	p.Gly418Val	LP/P	rs121965071	Factor XI deficiency (FA11D) [MIM:612416]
F11	P03951	VAR_054902	p.Tyr511His	LP/P	rs281875278	Factor XI deficiency (FA11D) [MIM:612416]
F11	P03951	VAR_054903	p.Pro538Leu	LP/P	rs139695003	Factor XI deficiency (FA11D) [MIM:612416]
F11	P03951	VAR_054904	p.Glu565Lys	LP/P	rs281875275	Factor XI deficiency (FA11D) [MIM:612416]
F11	P03951	VAR_054905	p.Trp587Ser	LP/P	rs121965072	Factor XI deficiency (FA11D) [MIM:612416]
F11	P03951	VAR_054906	p.Ile618Ser	LP/P	rs281875276	Factor XI deficiency (FA11D) [MIM:612416]
F11	P03951	VAR_067929	p.Gly32Arg	LP/P	rs281875259	Factor XI deficiency (FA11D) [MIM:612416]
F11	P03951	VAR_067930	p.Ala43Thr	LP/P	rs281875264	Factor XI deficiency (FA11D) [MIM:612416]
F11	P03951	VAR_067931	p.Thr51Ile	LP/P	rs281875252	Factor XI deficiency (FA11D) [MIM:612416]
F11	P03951	VAR_067932	p.Thr51Pro	LP/P	rs281875243	Factor XI deficiency (FA11D) [MIM:612416]
F11	P03951	VAR_067933	p.His53Gln	LP/P	rs281875261	Factor XI deficiency (FA11D) [MIM:612416]
F11	P03951	VAR_067934	p.Ala63Val	LP/P	rs281875244	Factor XI deficiency (FA11D) [MIM:612416]
F11	P03951	VAR_067935	p.Cys140Tyr	LP/P	rs281875256	Factor XI deficiency (FA11D) [MIM:612416]
F11	P03951	VAR_067936	p.Asp222Tyr	LP/P	rs281875245	Factor XI deficiency (FA11D) [MIM:612416]
F11	P03951	VAR_067937	p.Arg228Gln	LP/P	rs281875246	Factor XI deficiency (FA11D) [MIM:612416]
F11	P03951	VAR_067938	p.Phe241Leu	LP/P	rs281875265	Factor XI deficiency (FA11D) [MIM:612416]
F11	P03951	VAR_067939	p.Arg252Thr	LP/P	rs281875260	Factor XI deficiency (FA11D) [MIM:612416]
F11	P03951	VAR_067940	p.Ser276Cys	LP/P	rs281875247	Factor XI deficiency (FA11D) [MIM:612416]
F11	P03951	VAR_067941	p.Gly277Asp	LP/P	rs281875248	Factor XI deficiency (FA11D) [MIM:612416]
F11	P03951	VAR_067942	p.Glu315Lys	LP/P	rs281875257	Factor XI deficiency (FA11D) [MIM:612416]
F11	P03951	VAR_067943	p.Thr331Ile	LP/P	rs281875253	Factor XI deficiency (FA11D) [MIM:612416]
F11	P03951	VAR_067944	p.Leu360Pro	LP/P	rs281875254	Factor XI deficiency (FA11D) [MIM:612416]
F11	P03951	VAR_067945	p.Trp401Arg	LP/P	rs281875262	Factor XI deficiency (FA11D) [MIM:612416]
F11	P03951	VAR_067946	p.Val403Met	LP/P	rs281875266	Factor XI deficiency (FA11D) [MIM:612416]
F11	P03951	VAR_067947	p.Ile454Lys	LP/P	rs281875241	Factor XI deficiency (FA11D) [MIM:612416]
F11	P03951	VAR_067948	p.Ile481Ser	LP/P	rs281875242	Factor XI deficiency (FA11D) [MIM:612416]
F11	P03951	VAR_067949	p.Ser503Pro	LP/P	rs140068026	Factor XI deficiency (FA11D) [MIM:612416]
F11	P03951	VAR_067950	p.Asp506Gly	LP/P	rs281875258	Factor XI deficiency (FA11D) [MIM:612416]
F11	P03951	VAR_067951	p.Cys514Phe	LP/P	rs281875249	Factor XI deficiency (FA11D) [MIM:612416]
F11	P03951	VAR_067952	p.Asp526Glu	LP/P	rs281875263	Factor XI deficiency (FA11D) [MIM:612416]
F11	P03951	VAR_067953	p.Ser575Leu	LP/P	rs281875250	Factor XI deficiency (FA11D) [MIM:612416]
F11	P03951	VAR_067954	p.Glu597Lys	LP/P	rs281875251	Factor XI deficiency (FA11D) [MIM:612416]
F11	P03951	VAR_067955	p.Tyr608His	LP/P	rs281875255	Factor XI deficiency (FA11D) [MIM:612416]
F11	P03951	VAR_076515	p.Phe30Ser	LP/P	-	Factor XI deficiency (FA11D) [MIM:612416]
F11	P03951	VAR_076516	p.Ala109Thr	LP/P	rs768474112	Factor XI deficiency (FA11D) [MIM:612416]
F11	P03951	VAR_076517	p.Asp216Asn	LP/P	-	Factor XI deficiency (FA11D) [MIM:612416]
F11	P03951	VAR_076518	p.Glu543Lys	LP/P	rs142952627	Factor XI deficiency (FA11D) [MIM:612416]
F11	P03951	VAR_076519	p.His552Arg	LP/P	rs369935706	Factor XI deficiency (FA11D) [MIM:612416]
F12	P00748	VAR_006623	p.Arg372Pro	LP/P	rs118204454	Factor XII deficiency (FA12D) [MIM:234000]
F12	P00748	VAR_006624	p.Cys590Ser	LP/P	rs1157280571	Factor XII deficiency (FA12D) [MIM:234000]
F12	P00748	VAR_014336	p.Ala207Pro	LB/B	rs17876030	-
F12	P00748	VAR_014337	p.Gly545Asp	LB/B	rs17876034	-
F12	P00748	VAR_014338	p.Tyr605His	LB/B	rs17876035	-
F12	P00748	VAR_014426	p.Tyr53Cys	LP/P	rs118204455	Factor XII deficiency (FA12D) [MIM:234000]
F12	P00748	VAR_029191	p.Pro342Gln	LB/B	rs2230939	-
F12	P00748	VAR_031500	p.Arg142Pro	LP/P	-	Factor XII deficiency (FA12D) [MIM:234000]
F12	P00748	VAR_031501	p.Thr328Lys	LP/P	rs118204456	Hereditary angioedema 3 (HAE3) [MIM:610618]

F12	P00748	VAR_031502	p.Thr328Arg	LP/P	rs118204456	Hereditary angioedema 3 (HAE3) [MIM:610618]
F12	P00748	VAR_031503	p.Ala411Thr	LP/P	rs865853663	Factor XII deficiency (FA12D) [MIM:234000]
F12	P00748	VAR_031504	p.Leu414Met	LP/P	-	Factor XII deficiency (FA12D) [MIM:234000]
F12	P00748	VAR_031505	p.Arg417Gln	LP/P	rs932430490	Factor XII deficiency (FA12D) [MIM:234000]
F12	P00748	VAR_031506	p.Gln440Lys	LP/P	-	Factor XII deficiency (FA12D) [MIM:234000]
F12	P00748	VAR_031507	p.Asp461Asn	LP/P	-	Factor XII deficiency (FA12D) [MIM:234000]
F12	P00748	VAR_031508	p.Trp505Cys	LP/P	-	Factor XII deficiency (FA12D) [MIM:234000]
F12	P00748	VAR_031509	p.Gly589Arg	LP/P	rs766505234	Factor XII deficiency (FA12D) [MIM:234000]
F13A1	P00488	VAR_007471	p.Pro565Leu	LB/B	rs5982	-
F13A1	P00488	VAR_007472	p.Val651Ile	LB/B	rs5987	-
F13A1	P00488	VAR_007473	p.Gln652Glu	LB/B	rs5988	-
F13A1	P00488	VAR_007474	p.Arg682His	LP/P	rs121913064	Factor XIII subunit A deficiency (FA13AD) [MIM:613225]
F13A1	P00488	VAR_013927	p.Val35Leu	LB/B	rs5985	-
F13A1	P00488	VAR_013928	p.Thr551Ile	LB/B	rs5984	-
F13A1	P00488	VAR_013929	p.Leu589Gln	LB/B	rs5983	-
F13A1	P00488	VAR_020910	p.Val40Ile	LB/B	rs3024472	-
F13A1	P00488	VAR_020911	p.Tyr205Phe	LB/B	rs3024477	-
F13A1	P00488	VAR_060545	p.Thr650Ile	LB/B	rs17852475	-
F13A1	P00488	VAR_074280	p.Pro167Leu	LP/P	rs746272012	Factor XIII subunit A deficiency (FA13AD) [MIM:613225]
F13A1	P00488	VAR_074281	p.Arg172Gln	LP/P	rs376147795	Factor XIII subunit A deficiency (FA13AD) [MIM:613225]
F13A1	P00488	VAR_074282	p.Gly274Val	LP/P	-	Factor XIII subunit A deficiency (FA13AD) [MIM:613225]
F13A1	P00488	VAR_074283	p.His343Tyr	LP/P	-	Factor XIII subunit A deficiency (FA13AD) [MIM:613225]
F13A1	P00488	VAR_074284	p.Ala347Asp	US	-	Factor XIII subunit A deficiency (FA13AD) [MIM:613225]
F13A1	P00488	VAR_074285	p.Trp376Arg	US	-	Factor XIII subunit A deficiency (FA13AD) [MIM:613225]
F13A1	P00488	VAR_074286	p.Ser414Leu	US	rs1396702202	Factor XIII subunit A deficiency (FA13AD) [MIM:613225]
F13A1	P00488	VAR_074287	p.Gln416Arg	LP/P	-	Factor XIII subunit A deficiency (FA13AD) [MIM:613225]
F13A1	P00488	VAR_074288	p.Leu530Pro	LP/P	-	Factor XIII subunit A deficiency (FA13AD) [MIM:613225]
F13A1	P00488	VAR_074289	p.Gln602Lys	LP/P	rs757172838	Factor XIII subunit A deficiency (FA13AD) [MIM:613225]
F13A1	P00488	VAR_074290	p.Arg704Gln	LP/P	rs377484555	Factor XIII subunit A deficiency (FA13AD) [MIM:613225]
F13A1	P00488	VAR_074291	p.Arg716Gly	LP/P	rs778206273	Factor XIII subunit A deficiency (FA13AD) [MIM:613225]
F13A1	P00488	VAR_077619	p.Arg38Gln	LP/P	rs759324596	Factor XIII subunit A deficiency (FA13AD) [MIM:613225]
F13A1	P00488	VAR_077620	p.Tyr168Cys	LP/P	rs779361778	Factor XIII subunit A deficiency (FA13AD) [MIM:613225]
F13A1	P00488	VAR_077621	p.Pro290Arg	LP/P	-	Factor XIII subunit A deficiency (FA13AD) [MIM:613225]
F13A1	P00488	VAR_077622	p.Arg541Gln	LP/P	rs367679357	Factor XIII subunit A deficiency (FA13AD) [MIM:613225]
F13A1	P00488	VAR_077623	p.Gly593Ser	LP/P	rs138754417	Factor XIII subunit A deficiency (FA13AD) [MIM:613225]
F13A1	P00488	VAR_077624	p.Arg612His	LP/P	rs369187276	Factor XIII subunit A deficiency (FA13AD) [MIM:613225]

F13A1	P00488	VAR_077625	p.Asp669Gly	LP/P	rs375129902	Factor XIII subunit A deficiency (FA13AD) [MIM:613225]
F13B	P05160	VAR_007475	p.Cys450Phe	LP/P	rs121913075	Factor XIII subunit B deficiency (FA13BD) [MIM:613235]
F13B	P05160	VAR_013930	p.Met49Val	LB/B	rs6002	-
F13B	P05160	VAR_013931	p.Arg115His	LB/B	rs6003	-
F13B	P05160	VAR_013932	p.His350Arg	LB/B	rs5999	-
F13B	P05160	VAR_013933	p.Glu388Val	LB/B	rs5991	-
F13B	P05160	VAR_013934	p.Tyr543Ser	LB/B	rs6001	-
F13B	P05160	VAR_013935	p.Asp569Glu	LB/B	rs6000	-
F13B	P05160	VAR_020612	p.Ile342Thr	LB/B	rs17514281	-
F13B	P05160	VAR_020613	p.Leu529Pro	LB/B	rs17549671	-
F13B	P05160	VAR_074563	p.Cys25Arg	LP/P	rs1232302447	Factor XIII subunit B deficiency (FA13BD) [MIM:613235]
F13B	P05160	VAR_074564	p.Ile101Asn	LP/P	rs753009140	Factor XIII subunit B deficiency (FA13BD) [MIM:613235]
F13B	P05160	VAR_074565	p.Leu136Phe	US	rs757094432	Factor XIII subunit B deficiency (FA13BD) [MIM:613235]
F13B	P05160	VAR_074566	p.Val237Ile	US	rs145637157	Factor XIII subunit B deficiency (FA13BD) [MIM:613235]
F13B	P05160	VAR_074567	p.Cys336Phe	LP/P	rs778826479	Factor XIII subunit B deficiency (FA13BD) [MIM:613235]
F13B	P05160	VAR_074568	p.Val421Glu	LP/P	-	Factor XIII subunit B deficiency (FA13BD) [MIM:613235]
F13B	P05160	VAR_074569	p.Pro448Ser	LP/P	-	Factor XIII subunit B deficiency (FA13BD) [MIM:613235]
F2	P00734	VAR_006711	p.Glu200Lys	LP/P	rs62623459	Factor II deficiency (FA2D) [MIM:613679]
F2	P00734	VAR_006712	p.Arg314Cys	LP/P	rs121918477	Factor II deficiency (FA2D) [MIM:613679]
F2	P00734	VAR_006713	p.Arg314His	LP/P	rs754231232	Factor II deficiency (FA2D) [MIM:613679]
F2	P00734	VAR_006714	p.Met380Thr	LP/P	rs121918481	Factor II deficiency (FA2D) [MIM:613679]
F2	P00734	VAR_006715	p.Arg425Cys	LP/P	rs121918479	Factor II deficiency (FA2D) [MIM:613679]
F2	P00734	VAR_006716	p.Arg431His	LP/P	rs121918482	Factor II deficiency (FA2D) [MIM:613679]
F2	P00734	VAR_006717	p.Arg461Trp	LP/P	rs121918478	Factor II deficiency (FA2D) [MIM:613679]
F2	P00734	VAR_006718	p.Glu509Ala	LP/P	-	Factor II deficiency (FA2D) [MIM:613679]
F2	P00734	VAR_006719	p.Gly601Val	LP/P	rs121918480	Factor II deficiency (FA2D) [MIM:613679]
F2	P00734	VAR_011781	p.Thr165Met	LB/B	rs5896	-
F2	P00734	VAR_011782	p.Pro386Thr	LB/B	rs5897	-
F2	P00734	VAR_055232	p.Glu72Gly	LP/P	-	Factor II deficiency (FA2D) [MIM:613679]
F2	P00734	VAR_068913	p.Glu532Gln	LB/B	-	-
F2R	P25116	VAR_014167	p.Ser166Gly	LB/B	rs5893	-
F2R	P25116	VAR_049432	p.Tyr187Asn	LB/B	rs2230849	-
F2R	P25116	VAR_049433	p.Val257Leu	LB/B	rs2227832	-
F2R	P25116	VAR_049434	p.Ser412Tyr	LB/B	rs2227799	-
F2R	P25116	VAR_060680	p.Ala268Pro	LB/B	rs1055103	-
F2R	P25116	VAR_060681	p.Ala335Val	LB/B	rs17849599	-
F2RL1	P55085	VAR_012846	p.Ser21Phe	LB/B	rs2243072	-
F2RL1	P55085	VAR_012847	p.Arg270Gln	LB/B	rs2243062	-
F2RL1	P55085	VAR_012848	p.Thr291Ala	LB/B	rs2243083	-
F2RL1	P55085	VAR_049435	p.Asn30Ser	LB/B	rs616235	-
F2RL2	O00254	VAR_012849	p.Leu15Ser	LB/B	rs2069649	-
F2RL2	O00254	VAR_012850	p.Met177Val	LB/B	rs2069700	-

F2RL2 O00254 VAR_012851 p.Asn250Asp LB/B rs2069683 -
F2RL3 Q96RI0 VAR_012852 p.Phe296Val LB/B rs2227346 -
F2RL3 Q96RI0 VAR_012853 p.Pro310Leu LB/B rs2227376 -
F2RL3 Q96RI0 VAR_028300 p.Ala120Thr LB/B rs773902 -
F2RL3 Q96RI0 VAR_028301 p.Arg215Gln LB/B rs2230799 -
F3 P13726 VAR_012008 p.Arg163Trp LB/B rs5901 -
F3 P13726 VAR_014298 p.Thr36Ala LB/B rs3917604 -
F3 P13726 VAR_014299 p.Ile145Val LB/B rs3917627 -
F3 P13726 VAR_052280 p.Gly281Glu LB/B rs3789683 -
F5 P12259 VAR_001213 p.Arg534Gln LP/P rs6025 Thrombophilia due to activated protein C resistance (THPH2) [MIM:188055]
F5 P12259 VAR_001214 p.Lys858Arg LB/B rs4524 -
F5 P12259 VAR_001215 p.His865Arg LB/B rs4525 -
F5 P12259 VAR_013620 p.Arg334Gly LB/B rs118203905 -
F5 P12259 VAR_013621 p.Arg334Thr LP/P rs118203906 Thrombophilia due to activated protein C resistance (THPH2) [MIM:188055]
F5 P12259 VAR_013622 p.Arg513Lys LB/B rs6020 -
F5 P12259 VAR_013886 p.Asp107His LB/B rs6019 -
F5 P12259 VAR_013887 p.Met413Thr LB/B rs6033 -
F5 P12259 VAR_013888 p.Pro809Ser LB/B rs6031 -
F5 P12259 VAR_013889 p.Asn817Thr LB/B rs6018 -
F5 P12259 VAR_013890 p.Lys925Glu LB/B rs6032 -
F5 P12259 VAR_013891 p.His1146Gln LB/B rs6005 -
F5 P12259 VAR_013892 p.Leu1285Ile LB/B rs1046712 -
F5 P12259 VAR_013893 p.His1327Arg LB/B rs1800595 -
F5 P12259 VAR_013894 p.Glu1530Ala LB/B rs6007 -
F5 P12259 VAR_013895 p.Thr1685Ser LB/B rs6011 -
F5 P12259 VAR_013896 p.Leu1749Val LB/B rs6034 -
F5 P12259 VAR_013897 p.Met1764Val LB/B rs6030 -
F5 P12259 VAR_013898 p.Met1820Ile LB/B rs6026 -
F5 P12259 VAR_013899 p.Asp2222Gly LB/B rs6027 -
F5 P12259 VAR_017329 p.Arg2102His LP/P - Thrombophilia due to activated protein C resistance (THPH2) [MIM:188055]
F5 P12259 VAR_021297 p.Gly15Ser LB/B rs9332485 -
F5 P12259 VAR_021298 p.Thr915Ser LB/B rs9332695 -
F5 P12259 VAR_021299 p.Asn969Ser LB/B rs9332604 -
F5 P12259 VAR_021300 p.Arg980Leu LB/B rs9332605 -
F5 P12259 VAR_021301 p.Pro1404Ser LB/B rs9332608 -
F5 P12259 VAR_021302 p.Met2148Thr LB/B rs9332701 -
F5 P12259 VAR_032698 p.Ile387Thr LP/P rs118203911 Thrombophilia due to activated protein C resistance (THPH2) [MIM:188055]
F5 P12259 VAR_032699 p.Cys613Arg LP/P rs1453479152 Thrombophilia due to activated protein C resistance (THPH2) [MIM:188055]
F5 P12259 VAR_032700 p.Tyr1730Cys LP/P rs118203907 Factor V deficiency (FA5D) [MIM:227400]
F5 P12259 VAR_032701 p.Arg2102Cys LP/P rs118203910 Factor V deficiency (FA5D) [MIM:227400]
F5 P12259 VAR_034603 p.Lys2185Arg LB/B rs6679078 -
F5 P12259 VAR_035817 p.Ser775Ala US - A colorectal cancer sample
F5 P12259 VAR_047740 p.Ser781Arg LB/B rs13306350 -
F5 P12259 VAR_047741 p.Leu1397Phe LB/B rs13306334 -

F7	P08709	VAR_006497	p.Arg139Lys	LP/P	-	Factor VII deficiency (FA7D) [MIM:227500]
F7	P08709	VAR_006498	p.Arg139Gln	LP/P	rs150525536	Factor VII deficiency (FA7D) [MIM:227500]
F7	P08709	VAR_006499	p.Arg139Trp	LP/P	rs776796178	Factor VII deficiency (FA7D) [MIM:227500]
F7	P08709	VAR_006500	p.Gly157Ser	LP/P	rs763458490	Factor VII deficiency (FA7D) [MIM:227500]
F7	P08709	VAR_006501	p.Gly157Cys	LP/P	-	Factor VII deficiency (FA7D) [MIM:227500]
F7	P08709	VAR_006502	p.Gln160Arg	LP/P	rs200016360	Factor VII deficiency (FA7D) [MIM:227500]
F7	P08709	VAR_006503	p.Pro194Thr	LP/P	rs1234759020	Factor VII deficiency (FA7D) [MIM:227500]
F7	P08709	VAR_006504	p.Lys197Glu	LP/P	rs1250204261	Factor VII deficiency (FA7D) [MIM:227500]
F7	P08709	VAR_006505	p.Arg212Gln	LP/P	rs868044209	Factor VII deficiency (FA7D) [MIM:227500]
F7	P08709	VAR_006506	p.Cys238Tyr	LP/P	rs121964928	Factor VII deficiency (FA7D) [MIM:227500]
F7	P08709	VAR_006507	p.Arg283Trp	LP/P	rs779589651	Factor VII deficiency (FA7D) [MIM:227500]
F7	P08709	VAR_006508	p.Ala304Val	LP/P	rs121964931	Factor VII deficiency (FA7D) [MIM:227500]
F7	P08709	VAR_006509	p.Arg307His	LP/P	rs121964929	Factor VII deficiency (FA7D) [MIM:227500]
F7	P08709	VAR_006510	p.Glu325Lys	LP/P	rs749760143	Factor VII deficiency (FA7D) [MIM:227500]
F7	P08709	VAR_006511	p.Ala354Val	LP/P	rs36209567	Factor VII deficiency (FA7D) [MIM:227500]
F7	P08709	VAR_006512	p.Met358Ile	LP/P	rs149283257	Factor VII deficiency (FA7D) [MIM:227500]
F7	P08709	VAR_006513	p.Met358Val	LP/P	rs928183869	Factor VII deficiency (FA7D) [MIM:227500]
F7	P08709	VAR_006514	p.Arg364Gln	LP/P	rs121964926	Factor VII deficiency (FA7D) [MIM:227500]
F7	P08709	VAR_006515	p.Cys370Phe	LP/P	rs121964927	Factor VII deficiency (FA7D) [MIM:227500]
F7	P08709	VAR_006516	p.Gly402Arg	LP/P	-	Factor VII deficiency (FA7D) [MIM:227500]
F7	P08709	VAR_006517	p.Gly402Glu	LP/P	-	Factor VII deficiency (FA7D) [MIM:227500]
F7	P08709	VAR_006518	p.Arg413Gln	LB/B	rs6046	-
F7	P08709	VAR_006519	p.Thr419Met	LP/P	rs121964930	Factor VII deficiency (FA7D) [MIM:227500]
F7	P08709	VAR_013122	p.Ala352Thr	LB/B	rs3093267	-
F7	P08709	VAR_013123	p.Glu445Lys	LB/B	rs3093248	-
F7	P08709	VAR_013936	p.Val295Asp	LB/B	rs6045	-
F7	P08709	VAR_014391	p.Leu13Pro	LP/P	rs387906507	Factor VII deficiency (FA7D) [MIM:227500]
F7	P08709	VAR_014392	p.Cys389Gly	LP/P	rs121964934	Factor VII deficiency (FA7D) [MIM:227500]
F7	P08709	VAR_014405	p.Leu73Gln	LP/P	rs45572939	Factor VII deficiency (FA7D) [MIM:227500]
F7	P08709	VAR_014406	p.Glu79Gln	LP/P	-	Factor VII deficiency (FA7D) [MIM:227500]
F7	P08709	VAR_014407	p.Cys121Phe	LP/P	-	Factor VII deficiency (FA7D) [MIM:227500]
F7	P08709	VAR_014408	p.Leu125Pro	LP/P	-	Factor VII deficiency (FA7D) [MIM:227500]
F7	P08709	VAR_014409	p.Tyr128Cys	LP/P	-	Factor VII deficiency (FA7D) [MIM:227500]
F7	P08709	VAR_014410	p.Cys151Ser	LP/P	-	Factor VII deficiency (FA7D) [MIM:227500]
F7	P08709	VAR_014411	p.Gly157Val	LP/P	rs771335282	Factor VII deficiency (FA7D) [MIM:227500]
F7	P08709	VAR_014412	p.Cys195Arg	LP/P	rs372577568	Factor VII deficiency (FA7D) [MIM:227500]
F7	P08709	VAR_014413	p.Thr241Asn	LP/P	rs1160146175	Factor VII deficiency (FA7D) [MIM:227500]
F7	P08709	VAR_014414	p.Asp302His	LP/P	-	Factor VII deficiency (FA7D) [MIM:227500]
F7	P08709	VAR_014415	p.Asp302Asn	LP/P	rs770328850	Factor VII deficiency (FA7D) [MIM:227500]
F7	P08709	VAR_014416	p.Ala304Thr	LP/P	rs773627551	Factor VII deficiency (FA7D) [MIM:227500]
F7	P08709	VAR_014417	p.Arg307Cys	LP/P	rs147680958	Factor VII deficiency (FA7D) [MIM:227500]
F7	P08709	VAR_014418	p.Thr332Met	LP/P	rs200212201	Factor VII deficiency (FA7D) [MIM:227500]
F7	P08709	VAR_014419	p.Gly391Ser	LP/P	rs190485816	Factor VII deficiency (FA7D) [MIM:227500]
F7	P08709	VAR_014420	p.Gly435Glu	LP/P	rs756956471	Factor VII deficiency (FA7D) [MIM:227500]
F7	P08709	VAR_015135	p.Phe64Leu	LP/P	-	Factor VII deficiency (FA7D) [MIM:227500]
F7	P08709	VAR_015136	p.Ser120Pro	LP/P	-	Factor VII deficiency (FA7D) [MIM:227500]
F7	P08709	VAR_015137	p.Glu154Lys	LP/P	rs146795869	Factor VII deficiency (FA7D) [MIM:227500]
F7	P08709	VAR_015138	p.Gly216Asp	LP/P	rs1438503836	Factor VII deficiency (FA7D) [MIM:227500]
F7	P08709	VAR_015139	p.Cys254Tyr	LP/P	-	Factor VII deficiency (FA7D) [MIM:227500]

F7	P08709	VAR_015140	p.Ala266Thr	LP/P	rs764807079	Factor VII deficiency (FA7D) [MIM:227500]
F7	P08709	VAR_015141	p.Val312Met	LP/P	rs201991361	Factor VII deficiency (FA7D) [MIM:227500]
F7	P08709	VAR_015142	p.Val341Phe	LP/P	-	Factor VII deficiency (FA7D) [MIM:227500]
F7	P08709	VAR_015143	p.Pro363Arg	LP/P	rs963430078	Factor VII deficiency (FA7D) [MIM:227500]
F7	P08709	VAR_015144	p.Asp403His	LP/P	-	Factor VII deficiency (FA7D) [MIM:227500]
F7	P08709	VAR_018671	p.Thr367Ser	LB/B	rs747673406	-
F7	P08709	VAR_065370	p.Cys82Phe	LP/P	rs1448296564	Factor VII deficiency (FA7D) [MIM:227500]
F7	P08709	VAR_065371	p.Cys82Arg	LP/P	rs745374448	Factor VII deficiency (FA7D) [MIM:227500]
F7	P08709	VAR_065373	p.Glu85Lys	LP/P	rs121964935	Factor VII deficiency (FA7D) [MIM:227500]
F7	P08709	VAR_065374	p.Arg88Gly	LP/P	rs776354144	Factor VII deficiency (FA7D) [MIM:227500]
F7	P08709	VAR_065375	p.Arg88Pro	LP/P	-	Factor VII deficiency (FA7D) [MIM:227500]
F7	P08709	VAR_065376	p.Asn117Asp	LP/P	rs121964932	Factor VII deficiency (FA7D) [MIM:227500]
F7	P08709	VAR_065377	p.Gly138Asp	LP/P	-	Factor VII deficiency (FA7D) [MIM:227500]
F7	P08709	VAR_065378	p.Gly156Ser	LP/P	rs563972504	Factor VII deficiency (FA7D) [MIM:227500]
F7	P08709	VAR_065379	p.Ser171Phe	LP/P	rs143855920	Factor VII deficiency (FA7D) [MIM:227500]
F7	P08709	VAR_065380	p.Gly177Arg	LP/P	-	Factor VII deficiency (FA7D) [MIM:227500]
F7	P08709	VAR_065381	p.Leu181Pro	LP/P	-	Factor VII deficiency (FA7D) [MIM:227500]
F7	P08709	VAR_065382	p.Asp183Asn	LP/P	rs1258691292	Factor VII deficiency (FA7D) [MIM:227500]
F7	P08709	VAR_065383	p.Ser186Phe	LP/P	rs764971156	Factor VII deficiency (FA7D) [MIM:227500]
F7	P08709	VAR_065384	p.Pro189Ser	LP/P	rs1479693459	Factor VII deficiency (FA7D) [MIM:227500]
F7	P08709	VAR_065385	p.Pro194Leu	LP/P	-	Factor VII deficiency (FA7D) [MIM:227500]
F7	P08709	VAR_065386	p.Ile198Thr	LP/P	rs762621913	Factor VII deficiency (FA7D) [MIM:227500]
F7	P08709	VAR_065387	p.Gly240Arg	LP/P	-	Factor VII deficiency (FA7D) [MIM:227500]
F7	P08709	VAR_065388	p.Ser250Phe	LP/P	-	Factor VII deficiency (FA7D) [MIM:227500]
F7	P08709	VAR_065389	p.Ala251Pro	LP/P	-	Factor VII deficiency (FA7D) [MIM:227500]
F7	P08709	VAR_065390	p.Ala251Thr	LP/P	rs1269916662	Factor VII deficiency (FA7D) [MIM:227500]
F7	P08709	VAR_065391	p.Cys254Arg	LP/P	-	Factor VII deficiency (FA7D) [MIM:227500]
F7	P08709	VAR_065392	p.Leu264Pro	LP/P	rs753266903	Factor VII deficiency (FA7D) [MIM:227500]
F7	P08709	VAR_065393	p.Asp272Asn	LP/P	rs751028917	Factor VII deficiency (FA7D) [MIM:227500]
F7	P08709	VAR_065394	p.Asp277Asn	LP/P	rs550074221	Factor VII deficiency (FA7D) [MIM:227500]
F7	P08709	VAR_065395	p.Thr298Ile	LP/P	-	Factor VII deficiency (FA7D) [MIM:227500]
F7	P08709	VAR_065396	p.His301Gln	LP/P	-	Factor VII deficiency (FA7D) [MIM:227500]
F7	P08709	VAR_065397	p.Leu314Val	LP/P	-	Factor VII deficiency (FA7D) [MIM:227500]
F7	P08709	VAR_065398	p.Leu321Phe	LP/P	rs778138366	Factor VII deficiency (FA7D) [MIM:227500]
F7	P08709	VAR_065399	p.Leu323Arg	LP/P	-	Factor VII deficiency (FA7D) [MIM:227500]
F7	P08709	VAR_065400	p.Arg326Gln	LP/P	rs146698837	Factor VII deficiency (FA7D) [MIM:227500]
F7	P08709	VAR_065401	p.Arg337Cys	LP/P	rs139372641	Factor VII deficiency (FA7D) [MIM:227500]
F7	P08709	VAR_065402	p.Gly343Ser	LP/P	rs1250853566	Factor VII deficiency (FA7D) [MIM:227500]
F7	P08709	VAR_065403	p.Trp344Arg	LP/P	-	Factor VII deficiency (FA7D) [MIM:227500]
F7	P08709	VAR_065404	p.Gly345Ser	LP/P	-	Factor VII deficiency (FA7D) [MIM:227500]
F7	P08709	VAR_065405	p.Arg350Cys	LP/P	rs747876824	Factor VII deficiency (FA7D) [MIM:227500]
F7	P08709	VAR_065406	p.Leu360Pro	LP/P	-	Factor VII deficiency (FA7D) [MIM:227500]
F7	P08709	VAR_065407	p.Pro363His	LP/P	-	Factor VII deficiency (FA7D) [MIM:227500]
F7	P08709	VAR_065408	p.Arg364Trp	LP/P	rs750980786	Factor VII deficiency (FA7D) [MIM:227500]
F7	P08709	VAR_065409	p.Arg375Trp	LP/P	rs137919286	Factor VII deficiency (FA7D) [MIM:227500]
F7	P08709	VAR_065410	p.Thr384Met	LP/P	rs531225271	Factor VII deficiency (FA7D) [MIM:227500]
F7	P08709	VAR_065411	p.Met387Thr	LP/P	rs1595080725	Factor VII deficiency (FA7D) [MIM:227500]
F7	P08709	VAR_065412	p.Met387Val	LP/P	rs1215224419	Factor VII deficiency (FA7D) [MIM:227500]
F7	P08709	VAR_065413	p.Phe388Ser	LP/P	rs121964938	Factor VII deficiency (FA7D) [MIM:227500]

F7	P08709	VAR_065414	p.Gly391Cys	LP/P	-	Factor VII deficiency (FA7D) [MIM:227500]
F7	P08709	VAR_065415	p.Asp398Glu	LP/P	-	Factor VII deficiency (FA7D) [MIM:227500]
F7	P08709	VAR_065416	p.Lys401Glu	LP/P	rs748979195	Factor VII deficiency (FA7D) [MIM:227500]
F7	P08709	VAR_065417	p.Ser404Asn	LP/P	-	Factor VII deficiency (FA7D) [MIM:227500]
F7	P08709	VAR_065418	p.His408Gln	LP/P	rs121964936	Factor VII deficiency (FA7D) [MIM:227500]
F7	P08709	VAR_065419	p.His408Arg	LP/P	rs1566910847	Factor VII deficiency (FA7D) [MIM:227500]
F7	P08709	VAR_065420	p.Arg413Gly	LP/P	-	Factor VII deficiency (FA7D) [MIM:227500]
F7	P08709	VAR_065421	p.Gly414Cys	LP/P	rs121964937	Factor VII deficiency (FA7D) [MIM:227500]
F7	P08709	VAR_065422	p.Val422Phe	LP/P	-	Factor VII deficiency (FA7D) [MIM:227500]
F7	P08709	VAR_065423	p.Gly425Ala	LP/P	-	Factor VII deficiency (FA7D) [MIM:227500]
F7	P08709	VAR_065424	p.Gly425Cys	LP/P	-	Factor VII deficiency (FA7D) [MIM:227500]
F7	P08709	VAR_065425	p.Ala429Thr	LP/P	rs755377592	Factor VII deficiency (FA7D) [MIM:227500]
F7	P08709	VAR_065426	p.Gly432Asp	LP/P	rs1450120320	Factor VII deficiency (FA7D) [MIM:227500]
F7	P08709	VAR_065427	p.Tyr437Phe	LP/P	rs758213652	Factor VII deficiency (FA7D) [MIM:227500]
F7	P08709	VAR_076570	p.Trp344Gly	LP/P	-	Factor VII deficiency (FA7D) [MIM:227500]
F8	P00451	VAR_001045	p.Leu26Arg	LP/P	rs137852377	Hemophilia A (HEMA) [MIM:306700]
F8	P00451	VAR_001046	p.Glu30Val	LP/P	rs137852378	Hemophilia A (HEMA) [MIM:306700]
F8	P00451	VAR_001047	p.Gly41Cys	LP/P	rs137852379	Hemophilia A (HEMA) [MIM:306700]
F8	P00451	VAR_001048	p.Arg48Cys	LP/P	-	Hemophilia A (HEMA) [MIM:306700]
F8	P00451	VAR_001049	p.Asp75Val	LB/B	rs1800288	-
F8	P00451	VAR_001050	p.Gly89Asp	LP/P	rs137852380	Hemophilia A (HEMA) [MIM:306700]
F8	P00451	VAR_001051	p.Gly89Val	LP/P	-	Hemophilia A (HEMA) [MIM:306700]
F8	P00451	VAR_001052	p.Val99Asp	LP/P	rs137852382	Hemophilia A (HEMA) [MIM:306700]
F8	P00451	VAR_001053	p.Val104Asp	LP/P	rs137852383	Hemophilia A (HEMA) [MIM:306700]
F8	P00451	VAR_001054	p.Lys108Thr	LP/P	rs137852384	Hemophilia A (HEMA) [MIM:306700]
F8	P00451	VAR_001055	p.Met110Val	LP/P	rs137852385	Hemophilia A (HEMA) [MIM:306700]
F8	P00451	VAR_001056	p.Leu117Arg	LP/P	rs137852386	Hemophilia A (HEMA) [MIM:306700]
F8	P00451	VAR_001057	p.Glu129Val	LP/P	-	Hemophilia A (HEMA) [MIM:306700]
F8	P00451	VAR_001058	p.Gly130Arg	LP/P	rs137852387	Hemophilia A (HEMA) [MIM:306700]
F8	P00451	VAR_001059	p.Glu132Asp	LP/P	rs137852388	Hemophilia A (HEMA) [MIM:306700]
F8	P00451	VAR_001060	p.Tyr133Cys	LP/P	rs137852389	Hemophilia A (HEMA) [MIM:306700]
F8	P00451	VAR_001061	p.Asp135Gly	LP/P	rs137852390	Hemophilia A (HEMA) [MIM:306700]
F8	P00451	VAR_001062	p.Thr137Ile	LP/P	rs137852391	Hemophilia A (HEMA) [MIM:306700]
F8	P00451	VAR_001063	p.Gly164Val	LP/P	rs137852392	Hemophilia A (HEMA) [MIM:306700]
F8	P00451	VAR_001064	p.Pro165Ser	LP/P	rs137852393	Hemophilia A (HEMA) [MIM:306700]
F8	P00451	VAR_001065	p.Val181Met	LP/P	rs137852394	Hemophilia A (HEMA) [MIM:306700]
F8	P00451	VAR_001066	p.Lys185Thr	LP/P	rs137852395	Hemophilia A (HEMA) [MIM:306700]
F8	P00451	VAR_001067	p.Ser189Leu	LP/P	rs137852367	Hemophilia A (HEMA) [MIM:306700]
F8	P00451	VAR_001068	p.Asp222Val	LP/P	rs137852396	Hemophilia A (HEMA) [MIM:306700]
F8	P00451	VAR_001069	p.Gly224Trp	LP/P	rs137852397	Hemophilia A (HEMA) [MIM:306700]
F8	P00451	VAR_001070	p.Val253Phe	LP/P	-	Hemophilia A (HEMA) [MIM:306700]
F8	P00451	VAR_001071	p.Gly266Glu	LP/P	rs137852398	Hemophilia A (HEMA) [MIM:306700]
F8	P00451	VAR_001072	p.Gly278Arg	LP/P	rs137852399	Hemophilia A (HEMA) [MIM:306700]
F8	P00451	VAR_001073	p.Val285Gly	LP/P	rs137852400	Hemophilia A (HEMA) [MIM:306700]
F8	P00451	VAR_001074	p.Glu291Gly	LP/P	rs137852359	Hemophilia A (HEMA) [MIM:306700]
F8	P00451	VAR_001075	p.Thr294Ile	LP/P	rs137852401	Hemophilia A (HEMA) [MIM:306700]
F8	P00451	VAR_001076	p.Asn299Ile	LP/P	rs137852402	Hemophilia A (HEMA) [MIM:306700]
F8	P00451	VAR_001077	p.Arg301His	LP/P	rs137852403	Hemophilia A (HEMA) [MIM:306700]
F8	P00451	VAR_001078	p.Arg301Leu	LP/P	rs137852403	Hemophilia A (HEMA) [MIM:306700]

F8	P00451	VAR_001079	p.Ser308Leu	LP/P	rs137852404	Hemophilia A (HEMA) [MIM:306700]
F8	P00451	VAR_001080	p.Phe312Ser	LP/P	rs137852405	Hemophilia A (HEMA) [MIM:306700]
F8	P00451	VAR_001081	p.Thr314Ala	LP/P	rs137852406	Hemophilia A (HEMA) [MIM:306700]
F8	P00451	VAR_001082	p.Thr314Ile	LP/P	-	Hemophilia A (HEMA) [MIM:306700]
F8	P00451	VAR_001083	p.Leu327Pro	LP/P	rs137852407	Hemophilia A (HEMA) [MIM:306700]
F8	P00451	VAR_001084	p.Ile331Val	LP/P	-	Hemophilia A (HEMA) [MIM:306700]
F8	P00451	VAR_001085	p.Val345Leu	LP/P	rs137852371	Hemophilia A (HEMA) [MIM:306700]
F8	P00451	VAR_001086	p.Cys348Arg	LP/P	rs137852370	Hemophilia A (HEMA) [MIM:306700]
F8	P00451	VAR_001087	p.Cys348Ser	LP/P	rs137852410	Hemophilia A (HEMA) [MIM:306700]
F8	P00451	VAR_001088	p.Cys348Tyr	LP/P	-	Hemophilia A (HEMA) [MIM:306700]
F8	P00451	VAR_001089	p.Arg391Cys	LP/P	rs137852364	Hemophilia A (HEMA) [MIM:306700]
F8	P00451	VAR_001090	p.Arg391His	LP/P	rs28935499	Hemophilia A (HEMA) [MIM:306700]
F8	P00451	VAR_001091	p.Arg391Pro	LP/P	-	Hemophilia A (HEMA) [MIM:306700]
F8	P00451	VAR_001092	p.Ser392Leu	LP/P	rs28933668	Hemophilia A (HEMA) [MIM:306700]
F8	P00451	VAR_001093	p.Ser392Pro	LP/P	rs28933669	Hemophilia A (HEMA) [MIM:306700]
F8	P00451	VAR_001094	p.Ile405Ser	LP/P	rs28933670	Hemophilia A (HEMA) [MIM:306700]
F8	P00451	VAR_001095	p.Glu409Gly	LP/P	rs28933671	Hemophilia A (HEMA) [MIM:306700]
F8	P00451	VAR_001096	p.Leu431Phe	LP/P	rs28933672	Hemophilia A (HEMA) [MIM:306700]
F8	P00451	VAR_001097	p.Gly439Val	LP/P	rs1362305882	Hemophilia A (HEMA) [MIM:306700]
F8	P00451	VAR_001098	p.Lys444Arg	LP/P	rs28937272	Hemophilia A (HEMA) [MIM:306700]
F8	P00451	VAR_001099	p.Tyr450Asn	LP/P	rs111033616	Hemophilia A (HEMA) [MIM:306700]
F8	P00451	VAR_001100	p.Gly474Arg	LP/P	rs1345538633	Hemophilia A (HEMA) [MIM:306700]
F8	P00451	VAR_001101	p.Ala488Gly	LP/P	rs782485864	Hemophilia A (HEMA) [MIM:306700]
F8	P00451	VAR_001102	p.Tyr492His	LP/P	rs137852411	Hemophilia A (HEMA) [MIM:306700]
F8	P00451	VAR_001103	p.Tyr492Cys	LP/P	rs137852412	Hemophilia A (HEMA) [MIM:306700]
F8	P00451	VAR_001104	p.Ile494Thr	LP/P	rs137852413	Hemophilia A (HEMA) [MIM:306700]
F8	P00451	VAR_001105	p.Gly498Arg	LP/P	rs137852414	Hemophilia A (HEMA) [MIM:306700]
F8	P00451	VAR_001106	p.Asp544Asn	LP/P	rs137852415	Hemophilia A (HEMA) [MIM:306700]
F8	P00451	VAR_001107	p.Arg546Trp	LP/P	rs137852416	Hemophilia A (HEMA) [MIM:306700]
F8	P00451	VAR_001108	p.Arg550Cys	LP/P	rs137852417	Hemophilia A (HEMA) [MIM:306700]
F8	P00451	VAR_001109	p.Arg550Gly	LP/P	rs137852417	Hemophilia A (HEMA) [MIM:306700]
F8	P00451	VAR_001110	p.Arg550His	LP/P	rs137852418	Hemophilia A (HEMA) [MIM:306700]
F8	P00451	VAR_001111	p.Ser554Gly	LP/P	rs137852419	Hemophilia A (HEMA) [MIM:306700]
F8	P00451	VAR_001112	p.Val556Asp	LP/P	-	Hemophilia A (HEMA) [MIM:306700]
F8	P00451	VAR_001113	p.Ser577Phe	LP/P	rs28937282	Hemophilia A (HEMA) [MIM:306700]
F8	P00451	VAR_001114	p.Gln584Lys	LP/P	rs137852422	Hemophilia A (HEMA) [MIM:306700]
F8	P00451	VAR_001115	p.Ile585Thr	LP/P	rs137852376	Hemophilia A (HEMA) [MIM:306700]
F8	P00451	VAR_001116	p.Ser596Pro	LP/P	rs137852423	Hemophilia A (HEMA) [MIM:306700]
F8	P00451	VAR_001117	p.Ser603Ile	LP/P	rs137852425	Hemophilia A (HEMA) [MIM:306700]
F8	P00451	VAR_001118	p.Trp604Cys	LP/P	rs137852426	Hemophilia A (HEMA) [MIM:306700]
F8	P00451	VAR_001119	p.Tyr605Ser	LP/P	rs137852427	Hemophilia A (HEMA) [MIM:306700]
F8	P00451	VAR_001120	p.Arg612Cys	LP/P	rs137852428	Hemophilia A (HEMA) [MIM:306700]
F8	P00451	VAR_001121	p.Asn631Lys	LP/P	-	Hemophilia A (HEMA) [MIM:306700]
F8	P00451	VAR_001122	p.Asn631Ser	LP/P	rs137852429	Hemophilia A (HEMA) [MIM:306700]
F8	P00451	VAR_001123	p.Leu644Val	LP/P	-	Hemophilia A (HEMA) [MIM:306700]
F8	P00451	VAR_001124	p.Val653Ala	LP/P	rs137852430	Hemophilia A (HEMA) [MIM:306700]
F8	P00451	VAR_001125	p.Val653Met	LP/P	rs137852431	Hemophilia A (HEMA) [MIM:306700]
F8	P00451	VAR_001126	p.Ala663Val	LP/P	rs137852433	Hemophilia A (HEMA) [MIM:306700]
F8	P00451	VAR_001128	p.Phe677Leu	LP/P	rs137852434	Hemophilia A (HEMA) [MIM:306700]

F8	P00451	VAR_001129	p.Met699Val	LP/P	-	Hemophilia A (HEMA) [MIM:306700]
F8	P00451	VAR_001130	p.Arg717Trp	LP/P	rs137852435	Hemophilia A (HEMA) [MIM:306700]
F8	P00451	VAR_001131	p.Gly720Asp	LP/P	-	Hemophilia A (HEMA) [MIM:306700]
F8	P00451	VAR_001132	p.Ala723Thr	LP/P	rs137852436	Hemophilia A (HEMA) [MIM:306700]
F8	P00451	VAR_001133	p.Val727Phe	LP/P	rs1485277601	Hemophilia A (HEMA) [MIM:306700]
F8	P00451	VAR_001134	p.Glu739Lys	LP/P	rs28937285	Hemophilia A (HEMA) [MIM:306700]
F8	P00451	VAR_001135	p.Glu1057Lys	LP/P	rs28933673	Hemophilia A (HEMA) [MIM:306700]
F8	P00451	VAR_001136	p.Asp1260Glu	LB/B	rs1800291	-
F8	P00451	VAR_001137	p.Leu1481Pro	LB/B	rs1800294	-
F8	P00451	VAR_001138	p.Tyr1699Cys	LP/P	-	Hemophilia A (HEMA) [MIM:306700]
F8	P00451	VAR_001139	p.Tyr1699Phe	LP/P	rs28935203	Hemophilia A (HEMA) [MIM:306700]
F8	P00451	VAR_001140	p.Arg1708Cys	LP/P	rs111033613	Hemophilia A (HEMA) [MIM:306700]
F8	P00451	VAR_001141	p.Arg1708His	LP/P	rs111033614	Hemophilia A (HEMA) [MIM:306700]
F8	P00451	VAR_001142	p.Arg1715Gly	LP/P	rs137852439	Hemophilia A (HEMA) [MIM:306700]
F8	P00451	VAR_001143	p.Glu1723Lys	LP/P	rs137852373	Hemophilia A (HEMA) [MIM:306700]
F8	P00451	VAR_001144	p.Tyr1728Cys	LP/P	rs137852362	Hemophilia A (HEMA) [MIM:306700]
F8	P00451	VAR_001145	p.Gly1769Arg	LP/P	rs137852440	Hemophilia A (HEMA) [MIM:306700]
F8	P00451	VAR_001146	p.Leu1775Val	LP/P	rs28937287	Hemophilia A (HEMA) [MIM:306700]
F8	P00451	VAR_001147	p.Leu1775Phe	LP/P	rs137852441	Hemophilia A (HEMA) [MIM:306700]
F8	P00451	VAR_001148	p.Gly1779Glu	LP/P	rs28937289	Hemophilia A (HEMA) [MIM:306700]
F8	P00451	VAR_001149	p.Met1791Thr	LP/P	rs137852375	Hemophilia A (HEMA) [MIM:306700]
F8	P00451	VAR_001150	p.Arg1800His	LP/P	rs137852442	Hemophilia A (HEMA) [MIM:306700]
F8	P00451	VAR_001151	p.Arg1800Cys	LP/P	rs137852443	Hemophilia A (HEMA) [MIM:306700]
F8	P00451	VAR_001152	p.Arg1800Gly	LP/P	rs137852443	Hemophilia A (HEMA) [MIM:306700]
F8	P00451	VAR_001153	p.Ser1803Tyr	LP/P	rs137852444	Hemophilia A (HEMA) [MIM:306700]
F8	P00451	VAR_001154	p.Leu1808Phe	LP/P	rs137852445	Hemophilia A (HEMA) [MIM:306700]
F8	P00451	VAR_001155	p.Met1842Ile	LP/P	rs28933674	Hemophilia A (HEMA) [MIM:306700]
F8	P00451	VAR_001156	p.Pro1844Ser	LP/P	rs28933675	Hemophilia A (HEMA) [MIM:306700]
F8	P00451	VAR_001157	p.Thr1845Pro	LP/P	rs28933676	Hemophilia A (HEMA) [MIM:306700]
F8	P00451	VAR_001158	p.Ala1853Thr	LP/P	-	Hemophilia A (HEMA) [MIM:306700]
F8	P00451	VAR_001159	p.Ala1853Val	LP/P	rs28933677	Hemophilia A (HEMA) [MIM:306700]
F8	P00451	VAR_001160	p.Asp1865Asn	LP/P	rs28933678	Hemophilia A (HEMA) [MIM:306700]
F8	P00451	VAR_001161	p.Asp1865Tyr	LP/P	rs28933678	Hemophilia A (HEMA) [MIM:306700]
F8	P00451	VAR_001162	p.His1867Arg	LP/P	rs28933679	Hemophilia A (HEMA) [MIM:306700]
F8	P00451	VAR_001163	p.Gly1869Val	LP/P	rs1290383918	Hemophilia A (HEMA) [MIM:306700]
F8	P00451	VAR_001164	p.Pro1873Arg	LP/P	rs28933680	Hemophilia A (HEMA) [MIM:306700]
F8	P00451	VAR_001165	p.Arg1888Ile	LP/P	-	Hemophilia A (HEMA) [MIM:306700]
F8	P00451	VAR_001166	p.Glu1894Gly	LP/P	-	Hemophilia A (HEMA) [MIM:306700]
F8	P00451	VAR_001167	p.Glu1904Lys	LP/P	rs28933681	Hemophilia A (HEMA) [MIM:306700]
F8	P00451	VAR_001168	p.Asn1941Asp	LP/P	rs137852369	Hemophilia A (HEMA) [MIM:306700]
F8	P00451	VAR_001169	p.Asn1941Ser	LP/P	rs28933682	Hemophilia A (HEMA) [MIM:306700]
F8	P00451	VAR_001170	p.Arg1960Gln	LP/P	rs28937294	Hemophilia A (HEMA) [MIM:306700]
F8	P00451	VAR_001171	p.Arg1960Leu	LP/P	rs28937294	Hemophilia A (HEMA) [MIM:306700]
F8	P00451	VAR_001172	p.Gly1967Asp	LP/P	rs111033615	Hemophilia A (HEMA) [MIM:306700]
F8	P00451	VAR_001173	p.Gly1979Val	LP/P	rs137852450	Hemophilia A (HEMA) [MIM:306700]
F8	P00451	VAR_001174	p.His1980Tyr	LP/P	rs137852451	Hemophilia A (HEMA) [MIM:306700]
F8	P00451	VAR_001175	p.Arg2016Trp	LP/P	rs137852453	Hemophilia A (HEMA) [MIM:306700]
F8	P00451	VAR_001176	p.Asn2038Ser	LP/P	rs137852454	Hemophilia A (HEMA) [MIM:306700]
F8	P00451	VAR_001177	p.Trp2065Arg	LP/P	rs137852455	Hemophilia A (HEMA) [MIM:306700]

F8	P00451	VAR_001178	p.Ser2088Phe	LP/P	rs137852456	Hemophilia A (HEMA) [MIM:306700]
F8	P00451	VAR_001179	p.Asp2093Gly	LP/P	rs137852457	Hemophilia A (HEMA) [MIM:306700]
F8	P00451	VAR_001180	p.Thr2105Asn	LP/P	-	Hemophilia A (HEMA) [MIM:306700]
F8	P00451	VAR_001181	p.Gly2107Ser	LP/P	rs1267586059	Hemophilia A (HEMA) [MIM:306700]
F8	P00451	VAR_001182	p.Phe2120Leu	LP/P	rs137852458	Hemophilia A (HEMA) [MIM:306700]
F8	P00451	VAR_001183	p.Tyr2124Cys	LP/P	rs137852459	Hemophilia A (HEMA) [MIM:306700]
F8	P00451	VAR_001184	p.Arg2135Pro	LP/P	rs137852366	Hemophilia A (HEMA) [MIM:306700]
F8	P00451	VAR_001185	p.Ser2138Tyr	LP/P	rs137852460	Hemophilia A (HEMA) [MIM:306700]
F8	P00451	VAR_001186	p.Asn2148Ser	LP/P	rs1321311878	Hemophilia A (HEMA) [MIM:306700]
F8	P00451	VAR_001187	p.Arg2169His	LP/P	rs137852461	Hemophilia A (HEMA) [MIM:306700]
F8	P00451	VAR_001188	p.Pro2172Gln	LP/P	rs137852462	Hemophilia A (HEMA) [MIM:306700]
F8	P00451	VAR_001189	p.Thr2173Ile	LP/P	rs137852463	Hemophilia A (HEMA) [MIM:306700]
F8	P00451	VAR_001190	p.Arg2178Cys	LP/P	rs137852464	Hemophilia A (HEMA) [MIM:306700]
F8	P00451	VAR_001191	p.Arg2178His	LP/P	rs137852465	Hemophilia A (HEMA) [MIM:306700]
F8	P00451	VAR_001192	p.Arg2178Leu	LP/P	rs137852465	Hemophilia A (HEMA) [MIM:306700]
F8	P00451	VAR_001193	p.Arg2182Cys	LP/P	rs137852467	Hemophilia A (HEMA) [MIM:306700]
F8	P00451	VAR_001194	p.Arg2182His	LP/P	rs137852466	Hemophilia A (HEMA) [MIM:306700]
F8	P00451	VAR_001195	p.Met2183Val	LP/P	-	Hemophilia A (HEMA) [MIM:306700]
F8	P00451	VAR_001196	p.Leu2185Ser	LP/P	rs137852365	Hemophilia A (HEMA) [MIM:306700]
F8	P00451	VAR_001197	p.Ile2204Thr	LP/P	-	Hemophilia A (HEMA) [MIM:306700]
F8	P00451	VAR_001198	p.Ile2209Asn	LP/P	-	Hemophilia A (HEMA) [MIM:306700]
F8	P00451	VAR_001199	p.Ala2211Pro	LP/P	rs137852468	Hemophilia A (HEMA) [MIM:306700]
F8	P00451	VAR_001201	p.Arg2228Gly	LP/P	rs137852355	Hemophilia A (HEMA) [MIM:306700]
F8	P00451	VAR_001202	p.Arg2228Leu	LP/P	rs137852358	Hemophilia A (HEMA) [MIM:306700]
F8	P00451	VAR_001203	p.Arg2228Gln	LP/P	rs137852358	Hemophilia A (HEMA) [MIM:306700]
F8	P00451	VAR_001204	p.Val2242Met	LB/B	rs782654096	-
F8	P00451	VAR_001205	p.Trp2248Cys	LP/P	rs137852469	Hemophilia A (HEMA) [MIM:306700]
F8	P00451	VAR_001206	p.Gln2265Arg	LP/P	rs137852470	Hemophilia A (HEMA) [MIM:306700]
F8	P00451	VAR_001207	p.Pro2319Leu	LP/P	rs137852472	Hemophilia A (HEMA) [MIM:306700]
F8	P00451	VAR_001208	p.Pro2319Ser	LP/P	rs137852374	Hemophilia A (HEMA) [MIM:306700]
F8	P00451	VAR_001209	p.Arg2323Cys	LP/P	rs137852473	Hemophilia A (HEMA) [MIM:306700]
F8	P00451	VAR_001210	p.Arg2323His	LP/P	rs137852474	Hemophilia A (HEMA) [MIM:306700]
F8	P00451	VAR_001211	p.Arg2326Leu	LP/P	rs137852360	Hemophilia A (HEMA) [MIM:306700]
F8	P00451	VAR_001212	p.Arg2326Gln	LP/P	rs137852360	Hemophilia A (HEMA) [MIM:306700]
F8	P00451	VAR_008123	p.Ser202Arg	LP/P	-	Hemophilia A (HEMA) [MIM:306700]
F8	P00451	VAR_008967	p.Asp561Tyr	LP/P	-	Hemophilia A (HEMA) [MIM:306700]
F8	P00451	VAR_008968	p.Gly2344Cys	LP/P	-	Hemophilia A (HEMA) [MIM:306700]
F8	P00451	VAR_015127	p.Gly255Val	LP/P	-	Hemophilia A (HEMA) [MIM:306700]
F8	P00451	VAR_015128	p.Gly323Glu	LP/P	-	Hemophilia A (HEMA) [MIM:306700]
F8	P00451	VAR_015129	p.Met586Val	LP/P	-	Hemophilia A (HEMA) [MIM:306700]
F8	P00451	VAR_015130	p.Gly1942Ala	LP/P	-	Hemophilia A (HEMA) [MIM:306700]
F8	P00451	VAR_015131	p.Leu1963Pro	LP/P	-	Hemophilia A (HEMA) [MIM:306700]
F8	P00451	VAR_015132	p.Tyr2036Cys	LP/P	-	Hemophilia A (HEMA) [MIM:306700]
F8	P00451	VAR_015133	p.Pro2172Arg	LP/P	-	Hemophilia A (HEMA) [MIM:306700]
F8	P00451	VAR_015134	p.Asp2307Ala	LP/P	rs1603430929	Hemophilia A (HEMA) [MIM:306700]
F8	P00451	VAR_017330	p.Glu72Lys	LP/P	-	Hemophilia A (HEMA) [MIM:306700]
F8	P00451	VAR_017331	p.Ala97Pro	LP/P	-	Hemophilia A (HEMA) [MIM:306700]
F8	P00451	VAR_017332	p.Tyr155His	LP/P	rs1281943689	Hemophilia A (HEMA) [MIM:306700]
F8	P00451	VAR_017333	p.Val181Glu	LP/P	-	Hemophilia A (HEMA) [MIM:306700]

F8	P00451	VAR_017334	p.Asn254Ile	LP/P	-	Hemophilia A (HEMA) [MIM:306700]
F8	P00451	VAR_017335	p.Gly439Ser	LP/P	-	Hemophilia A (HEMA) [MIM:306700]
F8	P00451	VAR_017336	p.Lys529Glu	LP/P	-	Hemophilia A (HEMA) [MIM:306700]
F8	P00451	VAR_017337	p.Ile567Thr	LP/P	rs782193428	Hemophilia A (HEMA) [MIM:306700]
F8	P00451	VAR_017338	p.Phe1804Ser	LP/P	-	Hemophilia A (HEMA) [MIM:306700]
F8	P00451	VAR_017339	p.Ile2051Ser	LP/P	-	Hemophilia A (HEMA) [MIM:306700]
F8	P00451	VAR_017340	p.Thr2141Asn	LP/P	-	Hemophilia A (HEMA) [MIM:306700]
F8	P00451	VAR_017341	p.Cys2193Gly	LP/P	-	Hemophilia A (HEMA) [MIM:306700]
F8	P00451	VAR_021356	p.Met2257Val	LB/B	rs1800297	-
F8	P00451	VAR_024380	p.Arg795Gly	LB/B	rs2228152	-
F8	P00451	VAR_028447	p.Ser19Arg	LP/P	-	Hemophilia A (HEMA) [MIM:306700]
F8	P00451	VAR_028448	p.Arg22Thr	LP/P	-	Hemophilia A (HEMA) [MIM:306700]
F8	P00451	VAR_028449	p.Tyr24Cys	LP/P	-	Hemophilia A (HEMA) [MIM:306700]
F8	P00451	VAR_028450	p.Tyr25Cys	LP/P	-	Hemophilia A (HEMA) [MIM:306700]
F8	P00451	VAR_028451	p.Leu26Pro	LP/P	-	Hemophilia A (HEMA) [MIM:306700]
F8	P00451	VAR_028452	p.Trp33Gly	LP/P	-	Hemophilia A (HEMA) [MIM:306700]
F8	P00451	VAR_028453	p.Tyr35Cys	LP/P	rs137852476	Hemophilia A (HEMA) [MIM:306700]
F8	P00451	VAR_028454	p.Tyr35His	LP/P	-	Hemophilia A (HEMA) [MIM:306700]
F8	P00451	VAR_028455	p.Arg48Lys	LP/P	rs1261929809	Hemophilia A (HEMA) [MIM:306700]
F8	P00451	VAR_028456	p.Lys67Glu	LP/P	-	Hemophilia A (HEMA) [MIM:306700]
F8	P00451	VAR_028457	p.Lys67Asn	LP/P	-	Hemophilia A (HEMA) [MIM:306700]
F8	P00451	VAR_028458	p.Leu69Pro	LP/P	rs944567323	Hemophilia A (HEMA) [MIM:306700]
F8	P00451	VAR_028459	p.Asp75Glu	LP/P	-	Hemophilia A (HEMA) [MIM:306700]
F8	P00451	VAR_028460	p.Asp75Tyr	LP/P	-	Hemophilia A (HEMA) [MIM:306700]
F8	P00451	VAR_028463	p.Gly92Ala	LP/P	-	Hemophilia A (HEMA) [MIM:306700]
F8	P00451	VAR_028464	p.Gly92Val	LP/P	rs137852381	Hemophilia A (HEMA) [MIM:306700]
F8	P00451	VAR_028465	p.Glu98Lys	LP/P	rs1296842178	Hemophilia A (HEMA) [MIM:306700]
F8	P00451	VAR_028466	p.Asp101Gly	LP/P	rs1312347909	Hemophilia A (HEMA) [MIM:306700]
F8	P00451	VAR_028467	p.Asp101His	LP/P	-	Hemophilia A (HEMA) [MIM:306700]
F8	P00451	VAR_028468	p.Asp101Val	LP/P	-	Hemophilia A (HEMA) [MIM:306700]
F8	P00451	VAR_028469	p.Ala111Thr	LP/P	-	Hemophilia A (HEMA) [MIM:306700]
F8	P00451	VAR_028470	p.Ala111Val	LP/P	-	Hemophilia A (HEMA) [MIM:306700]
F8	P00451	VAR_028471	p.His113Arg	LP/P	-	Hemophilia A (HEMA) [MIM:306700]
F8	P00451	VAR_028472	p.His113Tyr	LP/P	-	Hemophilia A (HEMA) [MIM:306700]
F8	P00451	VAR_028473	p.Leu117Phe	LP/P	rs782481755	Hemophilia A (HEMA) [MIM:306700]
F8	P00451	VAR_028474	p.Gly121Ser	LP/P	-	Hemophilia A (HEMA) [MIM:306700]
F8	P00451	VAR_028475	p.Asp135Tyr	LP/P	-	Hemophilia A (HEMA) [MIM:306700]
F8	P00451	VAR_028476	p.Thr137Ala	LP/P	-	Hemophilia A (HEMA) [MIM:306700]
F8	P00451	VAR_028477	p.Ser138Arg	LP/P	-	Hemophilia A (HEMA) [MIM:306700]
F8	P00451	VAR_028478	p.Glu141Lys	LP/P	rs1388356765	Hemophilia A (HEMA) [MIM:306700]
F8	P00451	VAR_028479	p.Asp145His	LP/P	rs1433420305	Hemophilia A (HEMA) [MIM:306700]
F8	P00451	VAR_028480	p.Val147Asp	LP/P	-	Hemophilia A (HEMA) [MIM:306700]
F8	P00451	VAR_028481	p.Val159Ala	LP/P	-	Hemophilia A (HEMA) [MIM:306700]
F8	P00451	VAR_028482	p.Asn163Lys	LP/P	-	Hemophilia A (HEMA) [MIM:306700]
F8	P00451	VAR_028483	p.Gly164Asp	LP/P	rs137852392	Hemophilia A (HEMA) [MIM:306700]
F8	P00451	VAR_028484	p.Cys172Trp	LP/P	-	Hemophilia A (HEMA) [MIM:306700]
F8	P00451	VAR_028485	p.Ser176Pro	LP/P	-	Hemophilia A (HEMA) [MIM:306700]
F8	P00451	VAR_028486	p.Ser179Pro	LP/P	rs1455943875	Hemophilia A (HEMA) [MIM:306700]
F8	P00451	VAR_028487	p.Asp186Gly	LP/P	-	Hemophilia A (HEMA) [MIM:306700]

F8	P00451	VAR_028488	p.Asp186Tyr	LP/P	-	Hemophilia A (HEMA) [MIM:306700]
F8	P00451	VAR_028489	p.Gly193Arg	LP/P	-	Hemophilia A (HEMA) [MIM:306700]
F8	P00451	VAR_028490	p.Cys198Gly	LP/P	rs137852475	Hemophilia A (HEMA) [MIM:306700]
F8	P00451	VAR_028491	p.Ser202Asn	LP/P	rs1603436218	Hemophilia A (HEMA) [MIM:306700]
F8	P00451	VAR_028492	p.Phe214Val	LP/P	-	Hemophilia A (HEMA) [MIM:306700]
F8	P00451	VAR_028493	p.Leu217His	LP/P	-	Hemophilia A (HEMA) [MIM:306700]
F8	P00451	VAR_028494	p.Ala219Asp	LP/P	-	Hemophilia A (HEMA) [MIM:306700]
F8	P00451	VAR_028495	p.Ala219Thr	LP/P	-	Hemophilia A (HEMA) [MIM:306700]
F8	P00451	VAR_028496	p.Val220Gly	LP/P	-	Hemophilia A (HEMA) [MIM:306700]
F8	P00451	VAR_028497	p.Glu223Lys	LP/P	-	Hemophilia A (HEMA) [MIM:306700]
F8	P00451	VAR_028498	p.Thr252Ile	LP/P	rs1464962436	Hemophilia A (HEMA) [MIM:306700]
F8	P00451	VAR_028499	p.Pro262Leu	LP/P	-	Hemophilia A (HEMA) [MIM:306700]
F8	P00451	VAR_028500	p.Gly263Ser	LP/P	-	Hemophilia A (HEMA) [MIM:306700]
F8	P00451	VAR_028501	p.Cys267Tyr	LP/P	rs1208703993	Hemophilia A (HEMA) [MIM:306700]
F8	P00451	VAR_028502	p.Trp274Cys	LP/P	rs34371500	Hemophilia A (HEMA) [MIM:306700]
F8	P00451	VAR_028503	p.His275Leu	LP/P	-	Hemophilia A (HEMA) [MIM:306700]
F8	P00451	VAR_028504	p.Glu284Lys	LP/P	-	Hemophilia A (HEMA) [MIM:306700]
F8	P00451	VAR_028505	p.Glu291Lys	LP/P	rs868988809	Hemophilia A (HEMA) [MIM:306700]
F8	P00451	VAR_028506	p.Phe295Leu	LP/P	-	Hemophilia A (HEMA) [MIM:306700]
F8	P00451	VAR_028507	p.Val297Ala	LP/P	-	Hemophilia A (HEMA) [MIM:306700]
F8	P00451	VAR_028508	p.Arg301Cys	LP/P	rs1401805753	Hemophilia A (HEMA) [MIM:306700]
F8	P00451	VAR_028510	p.Ala303Glu	LP/P	-	Hemophilia A (HEMA) [MIM:306700]
F8	P00451	VAR_028511	p.Ala303Pro	LP/P	-	Hemophilia A (HEMA) [MIM:306700]
F8	P00451	VAR_028512	p.Ile307Ser	LP/P	-	Hemophilia A (HEMA) [MIM:306700]
F8	P00451	VAR_028513	p.Ala315Val	LP/P	-	Hemophilia A (HEMA) [MIM:306700]
F8	P00451	VAR_028515	p.Leu326Pro	LP/P	-	Hemophilia A (HEMA) [MIM:306700]
F8	P00451	VAR_028516	p.Leu327Val	LP/P	rs1603435395	Hemophilia A (HEMA) [MIM:306700]
F8	P00451	VAR_028517	p.Cys329Phe	LP/P	-	Hemophilia A (HEMA) [MIM:306700]
F8	P00451	VAR_028518	p.Met339Thr	LP/P	-	Hemophilia A (HEMA) [MIM:306700]
F8	P00451	VAR_028519	p.Glu340Lys	LP/P	rs781954986	Hemophilia A (HEMA) [MIM:306700]
F8	P00451	VAR_028520	p.Val345Ala	LP/P	rs1189348665	Hemophilia A (HEMA) [MIM:306700]
F8	P00451	VAR_028521	p.Tyr365Cys	LP/P	rs375241473	Hemophilia A (HEMA) [MIM:306700]
F8	P00451	VAR_028522	p.Trp401Gly	LP/P	-	Hemophilia A (HEMA) [MIM:306700]
F8	P00451	VAR_028523	p.Ile405Phe	LP/P	-	Hemophilia A (HEMA) [MIM:306700]
F8	P00451	VAR_028524	p.Trp412Gly	LP/P	-	Hemophilia A (HEMA) [MIM:306700]
F8	P00451	VAR_028525	p.Trp412Arg	LP/P	rs1234456704	Hemophilia A (HEMA) [MIM:306700]
F8	P00451	VAR_028526	p.Lys427Ile	LP/P	-	Hemophilia A (HEMA) [MIM:306700]
F8	P00451	VAR_028527	p.Leu431Ser	LP/P	-	Hemophilia A (HEMA) [MIM:306700]
F8	P00451	VAR_028528	p.Arg437Pro	LP/P	-	Hemophilia A (HEMA) [MIM:306700]
F8	P00451	VAR_028529	p.Arg437Trp	LP/P	-	Hemophilia A (HEMA) [MIM:306700]
F8	P00451	VAR_028530	p.Ile438Phe	LP/P	rs1258333672	Hemophilia A (HEMA) [MIM:306700]
F8	P00451	VAR_028531	p.Gly439Asp	LP/P	rs1362305882	Hemophilia A (HEMA) [MIM:306700]
F8	P00451	VAR_028532	p.Tyr442Cys	LP/P	rs1441830456	Hemophilia A (HEMA) [MIM:306700]
F8	P00451	VAR_028533	p.Tyr450Asp	LP/P	-	Hemophilia A (HEMA) [MIM:306700]
F8	P00451	VAR_028534	p.Thr454Ile	LP/P	-	Hemophilia A (HEMA) [MIM:306700]
F8	P00451	VAR_028535	p.Phe455Cys	LP/P	rs1603435217	Hemophilia A (HEMA) [MIM:306700]
F8	P00451	VAR_028536	p.Gly466Glu	LP/P	rs1304348198	Hemophilia A (HEMA) [MIM:306700]
F8	P00451	VAR_028537	p.Pro470Leu	LP/P	rs1240470740	Hemophilia A (HEMA) [MIM:306700]
F8	P00451	VAR_028538	p.Pro470Arg	LP/P	rs1240470740	Hemophilia A (HEMA) [MIM:306700]

F8	P00451	VAR_028539	p.Pro470Thr	LP/P	-	Hemophilia A (HEMA) [MIM:306700]
F8	P00451	VAR_028540	p.Gly474Glu	LP/P	-	Hemophilia A (HEMA) [MIM:306700]
F8	P00451	VAR_028541	p.Glu475Lys	LP/P	-	Hemophilia A (HEMA) [MIM:306700]
F8	P00451	VAR_028542	p.Gly477Val	LP/P	-	Hemophilia A (HEMA) [MIM:306700]
F8	P00451	VAR_028543	p.Asp478Asn	LP/P	-	Hemophilia A (HEMA) [MIM:306700]
F8	P00451	VAR_028544	p.Thr479Arg	LP/P	-	Hemophilia A (HEMA) [MIM:306700]
F8	P00451	VAR_028545	p.Phe484Cys	LP/P	-	Hemophilia A (HEMA) [MIM:306700]
F8	P00451	VAR_028546	p.Arg490Gly	LP/P	rs1603435026	Hemophilia A (HEMA) [MIM:306700]
F8	P00451	VAR_028547	p.Arg503His	LP/P	rs35383156	Hemophilia A (HEMA) [MIM:306700]
F8	P00451	VAR_028548	p.Gly513Ser	LP/P	rs1269117966	Hemophilia A (HEMA) [MIM:306700]
F8	P00451	VAR_028549	p.Ile522Tyr	LP/P	-	Hemophilia A (HEMA) [MIM:306700]
F8	P00451	VAR_028550	p.Trp532Gly	LP/P	-	Hemophilia A (HEMA) [MIM:306700]
F8	P00451	VAR_028551	p.Pro540Thr	LP/P	-	Hemophilia A (HEMA) [MIM:306700]
F8	P00451	VAR_028552	p.Thr541Ser	LP/P	rs139526001	Hemophilia A (HEMA) [MIM:306700]
F8	P00451	VAR_028553	p.Ser553Pro	LP/P	-	Hemophilia A (HEMA) [MIM:306700]
F8	P00451	VAR_028554	p.Ser554Cys	LP/P	-	Hemophilia A (HEMA) [MIM:306700]
F8	P00451	VAR_028555	p.Arg560Thr	LP/P	-	Hemophilia A (HEMA) [MIM:306700]
F8	P00451	VAR_028556	p.Asp561Gly	LP/P	rs137852420	Hemophilia A (HEMA) [MIM:306700]
F8	P00451	VAR_028557	p.Asp561His	LP/P	-	Hemophilia A (HEMA) [MIM:306700]
F8	P00451	VAR_028558	p.Val578Ala	LP/P	-	Hemophilia A (HEMA) [MIM:306700]
F8	P00451	VAR_028559	p.Asp579Ala	LP/P	-	Hemophilia A (HEMA) [MIM:306700]
F8	P00451	VAR_028560	p.Asp579His	LP/P	-	Hemophilia A (HEMA) [MIM:306700]
F8	P00451	VAR_028561	p.Asn583Ser	LP/P	rs782657516	Hemophilia A (HEMA) [MIM:306700]
F8	P00451	VAR_028562	p.Gln584His	LP/P	-	Hemophilia A (HEMA) [MIM:306700]
F8	P00451	VAR_028563	p.Gln584Arg	LP/P	rs1354815715	Hemophilia A (HEMA) [MIM:306700]
F8	P00451	VAR_028564	p.Ile585Arg	LP/P	-	Hemophilia A (HEMA) [MIM:306700]
F8	P00451	VAR_028565	p.Asp588Gly	LP/P	-	Hemophilia A (HEMA) [MIM:306700]
F8	P00451	VAR_028566	p.Asp588Tyr	LP/P	-	Hemophilia A (HEMA) [MIM:306700]
F8	P00451	VAR_028567	p.Leu594Gln	LP/P	-	Hemophilia A (HEMA) [MIM:306700]
F8	P00451	VAR_028568	p.Asn601Asp	LP/P	rs1460318222	Hemophilia A (HEMA) [MIM:306700]
F8	P00451	VAR_028569	p.Asn601Lys	LP/P	rs1299810903	Hemophilia A (HEMA) [MIM:306700]
F8	P00451	VAR_028570	p.Arg602Gly	LP/P	rs137852424	Hemophilia A (HEMA) [MIM:306700]
F8	P00451	VAR_028571	p.Ser603Arg	LP/P	-	Hemophilia A (HEMA) [MIM:306700]
F8	P00451	VAR_028572	p.Tyr605His	LP/P	-	Hemophilia A (HEMA) [MIM:306700]
F8	P00451	VAR_028573	p.Asn609Ile	LP/P	rs1253524555	Hemophilia A (HEMA) [MIM:306700]
F8	P00451	VAR_028574	p.Met633Ile	LP/P	-	Hemophilia A (HEMA) [MIM:306700]
F8	P00451	VAR_028575	p.Ser635Asn	LP/P	-	Hemophilia A (HEMA) [MIM:306700]
F8	P00451	VAR_028576	p.Asn637Asp	LP/P	-	Hemophilia A (HEMA) [MIM:306700]
F8	P00451	VAR_028577	p.Asn637Ser	LP/P	-	Hemophilia A (HEMA) [MIM:306700]
F8	P00451	VAR_028578	p.Tyr639Cys	LP/P	-	Hemophilia A (HEMA) [MIM:306700]
F8	P00451	VAR_028579	p.Leu650Phe	LP/P	rs1557280438	Hemophilia A (HEMA) [MIM:306700]
F8	P00451	VAR_028580	p.Leu659Pro	LP/P	-	Hemophilia A (HEMA) [MIM:306700]
F8	P00451	VAR_028581	p.Gln664Pro	LP/P	-	Hemophilia A (HEMA) [MIM:306700]
F8	P00451	VAR_028582	p.Met681Ile	LP/P	rs1603434460	Hemophilia A (HEMA) [MIM:306700]
F8	P00451	VAR_028583	p.Val682Phe	LP/P	rs1569559755	Hemophilia A (HEMA) [MIM:306700]
F8	P00451	VAR_028584	p.Tyr683Cys	LP/P	rs1384374956	Hemophilia A (HEMA) [MIM:306700]
F8	P00451	VAR_028585	p.Tyr683Asn	LP/P	-	Hemophilia A (HEMA) [MIM:306700]
F8	P00451	VAR_028586	p.Thr686Arg	LP/P	-	Hemophilia A (HEMA) [MIM:306700]
F8	P00451	VAR_028587	p.Phe698Leu	LP/P	-	Hemophilia A (HEMA) [MIM:306700]

F8	P00451	VAR_028588	p.Met699Thr	LP/P	-	Hemophilia A (HEMA) [MIM:306700]
F8	P00451	VAR_028589	p.Met701Ile	LP/P	-	Hemophilia A (HEMA) [MIM:306700]
F8	P00451	VAR_028590	p.Gly705Val	LP/P	-	Hemophilia A (HEMA) [MIM:306700]
F8	P00451	VAR_028591	p.Gly710Trp	LP/P	-	Hemophilia A (HEMA) [MIM:306700]
F8	P00451	VAR_028592	p.Asn713Ile	LP/P	-	Hemophilia A (HEMA) [MIM:306700]
F8	P00451	VAR_028593	p.Arg717Leu	LP/P	-	Hemophilia A (HEMA) [MIM:306700]
F8	P00451	VAR_028594	p.Gly720Ser	LP/P	-	Hemophilia A (HEMA) [MIM:306700]
F8	P00451	VAR_028595	p.Met721Ile	LP/P	rs1218576358	Hemophilia A (HEMA) [MIM:306700]
F8	P00451	VAR_028596	p.Met721Leu	LP/P	rs1305924233	Hemophilia A (HEMA) [MIM:306700]
F8	P00451	VAR_028597	p.Leu725Gln	LP/P	-	Hemophilia A (HEMA) [MIM:306700]
F8	P00451	VAR_028598	p.Tyr742Cys	LP/P	-	Hemophilia A (HEMA) [MIM:306700]
F8	P00451	VAR_028599	p.Pro947Arg	LP/P	rs782318401	Hemophilia A (HEMA) [MIM:306700]
F8	P00451	VAR_028600	p.Val1012Leu	LP/P	-	Hemophilia A (HEMA) [MIM:306700]
F8	P00451	VAR_028601	p.His1066Tyr	LP/P	-	Hemophilia A (HEMA) [MIM:306700]
F8	P00451	VAR_028602	p.Gln1336Lys	LP/P	-	Hemophilia A (HEMA) [MIM:306700]
F8	P00451	VAR_028603	p.Asn1460Lys	LP/P	-	Hemophilia A (HEMA) [MIM:306700]
F8	P00451	VAR_028604	p.Ala1610Ser	LP/P	rs782127226	Hemophilia A (HEMA) [MIM:306700]
F8	P00451	VAR_028605	p.Ile1698Thr	LP/P	-	Hemophilia A (HEMA) [MIM:306700]
F8	P00451	VAR_028606	p.Glu1701Lys	LP/P	-	Hemophilia A (HEMA) [MIM:306700]
F8	P00451	VAR_028607	p.Gln1705His	LP/P	-	Hemophilia A (HEMA) [MIM:306700]
F8	P00451	VAR_028608	p.Thr1714Ser	LP/P	-	Hemophilia A (HEMA) [MIM:306700]
F8	P00451	VAR_028609	p.Asp1727Val	LP/P	-	Hemophilia A (HEMA) [MIM:306700]
F8	P00451	VAR_028610	p.Arg1740Gly	LP/P	-	Hemophilia A (HEMA) [MIM:306700]
F8	P00451	VAR_028611	p.Lys1751Gln	LP/P	-	Hemophilia A (HEMA) [MIM:306700]
F8	P00451	VAR_028612	p.Arg1768His	LP/P	rs151202877	Hemophilia A (HEMA) [MIM:306700]
F8	P00451	VAR_028613	p.Leu1771Pro	LP/P	-	Hemophilia A (HEMA) [MIM:306700]
F8	P00451	VAR_028614	p.Leu1777Pro	LP/P	-	Hemophilia A (HEMA) [MIM:306700]
F8	P00451	VAR_028615	p.Gly1779Arg	LP/P	rs1168919288	Hemophilia A (HEMA) [MIM:306700]
F8	P00451	VAR_028616	p.Pro1780Leu	LP/P	-	Hemophilia A (HEMA) [MIM:306700]
F8	P00451	VAR_028617	p.Ile1782Arg	LP/P	rs1466581271	Hemophilia A (HEMA) [MIM:306700]
F8	P00451	VAR_028618	p.Asp1788His	LP/P	-	Hemophilia A (HEMA) [MIM:306700]
F8	P00451	VAR_028619	p.Ala1798Pro	LP/P	rs1263565590	Hemophilia A (HEMA) [MIM:306700]
F8	P00451	VAR_028620	p.Ser1799His	LP/P	-	Hemophilia A (HEMA) [MIM:306700]
F8	P00451	VAR_028621	p.Pro1801Ala	LP/P	-	Hemophilia A (HEMA) [MIM:306700]
F8	P00451	VAR_028622	p.Tyr1802Cys	LP/P	-	Hemophilia A (HEMA) [MIM:306700]
F8	P00451	VAR_028623	p.Glu1848Gly	LP/P	-	Hemophilia A (HEMA) [MIM:306700]
F8	P00451	VAR_028624	p.Ser1858Cys	LP/P	-	Hemophilia A (HEMA) [MIM:306700]
F8	P00451	VAR_028625	p.Lys1864Glu	LP/P	-	Hemophilia A (HEMA) [MIM:306700]
F8	P00451	VAR_028626	p.His1867Pro	LP/P	-	Hemophilia A (HEMA) [MIM:306700]
F8	P00451	VAR_028627	p.Gly1869Asp	LP/P	-	Hemophilia A (HEMA) [MIM:306700]
F8	P00451	VAR_028628	p.Gly1872Glu	LP/P	-	Hemophilia A (HEMA) [MIM:306700]
F8	P00451	VAR_028629	p.Leu1875Pro	LP/P	-	Hemophilia A (HEMA) [MIM:306700]
F8	P00451	VAR_028630	p.Val1876Leu	LP/P	-	Hemophilia A (HEMA) [MIM:306700]
F8	P00451	VAR_028631	p.Cys1877Arg	LP/P	-	Hemophilia A (HEMA) [MIM:306700]
F8	P00451	VAR_028632	p.Leu1882Pro	LP/P	-	Hemophilia A (HEMA) [MIM:306700]
F8	P00451	VAR_028633	p.Ile1901Phe	LP/P	-	Hemophilia A (HEMA) [MIM:306700]
F8	P00451	VAR_028634	p.Glu1904Asp	LP/P	rs1416920499	Hemophilia A (HEMA) [MIM:306700]
F8	P00451	VAR_028635	p.Ser1907Cys	LP/P	rs1160914716	Hemophilia A (HEMA) [MIM:306700]
F8	P00451	VAR_028636	p.Ser1907Arg	LP/P	rs1364158178	Hemophilia A (HEMA) [MIM:306700]

F8	P00451	VAR_028637	p.Trp1908Leu	LP/P	-	Hemophilia A (HEMA) [MIM:306700]
F8	P00451	VAR_028638	p.Tyr1909Cys	LP/P	-	Hemophilia A (HEMA) [MIM:306700]
F8	P00451	VAR_028639	p.Ala1939Thr	LP/P	-	Hemophilia A (HEMA) [MIM:306700]
F8	P00451	VAR_028640	p.Ala1939Val	US	-	Hemophilia A (HEMA) [MIM:306700]
F8	P00451	VAR_028641	p.Met1945Val	LP/P	-	Hemophilia A (HEMA) [MIM:306700]
F8	P00451	VAR_028642	p.Leu1951Phe	LP/P	-	Hemophilia A (HEMA) [MIM:306700]
F8	P00451	VAR_028643	p.Ser1965Ile	LP/P	-	Hemophilia A (HEMA) [MIM:306700]
F8	P00451	VAR_028644	p.Met1966Ile	LP/P	-	Hemophilia A (HEMA) [MIM:306700]
F8	P00451	VAR_028645	p.Met1966Val	LP/P	-	Hemophilia A (HEMA) [MIM:306700]
F8	P00451	VAR_028646	p.Ser1968Arg	LP/P	-	Hemophilia A (HEMA) [MIM:306700]
F8	P00451	VAR_028647	p.Asn1971Thr	LP/P	-	Hemophilia A (HEMA) [MIM:306700]
F8	P00451	VAR_028648	p.His1973Leu	LP/P	rs1273080258	Hemophilia A (HEMA) [MIM:306700]
F8	P00451	VAR_028649	p.His1980Pro	LP/P	-	Hemophilia A (HEMA) [MIM:306700]
F8	P00451	VAR_028650	p.Phe1982Ile	LP/P	-	Hemophilia A (HEMA) [MIM:306700]
F8	P00451	VAR_028651	p.Arg1985Gln	LP/P	rs1490417405	Hemophilia A (HEMA) [MIM:306700]
F8	P00451	VAR_028652	p.Leu1994Pro	LP/P	rs1367630608	Hemophilia A (HEMA) [MIM:306700]
F8	P00451	VAR_028653	p.Tyr1998Cys	LP/P	-	Hemophilia A (HEMA) [MIM:306700]
F8	P00451	VAR_028654	p.Gly2000Ala	LP/P	rs1343859825	Hemophilia A (HEMA) [MIM:306700]
F8	P00451	VAR_028655	p.Thr2004Arg	LP/P	-	Hemophilia A (HEMA) [MIM:306700]
F8	P00451	VAR_028656	p.Met2007Ile	LP/P	-	Hemophilia A (HEMA) [MIM:306700]
F8	P00451	VAR_028657	p.Trp2015Cys	LP/P	rs1190563629	Hemophilia A (HEMA) [MIM:306700]
F8	P00451	VAR_028658	p.Arg2016Pro	LP/P	-	Hemophilia A (HEMA) [MIM:306700]
F8	P00451	VAR_028659	p.Glu2018Gly	LP/P	rs1406262850	Hemophilia A (HEMA) [MIM:306700]
F8	P00451	VAR_028660	p.Gly2022Asp	LP/P	rs1320622042	Hemophilia A (HEMA) [MIM:306700]
F8	P00451	VAR_028661	p.Gly2028Arg	LP/P	rs1603432908	Hemophilia A (HEMA) [MIM:306700]
F8	P00451	VAR_028662	p.Ser2030Asn	LP/P	rs369414658	Hemophilia A (HEMA) [MIM:306700]
F8	P00451	VAR_028663	p.Val2035Ala	LP/P	-	Hemophilia A (HEMA) [MIM:306700]
F8	P00451	VAR_028664	p.Cys2040Tyr	LP/P	-	Hemophilia A (HEMA) [MIM:306700]
F8	P00451	VAR_028665	p.Gly2045Glu	LP/P	-	Hemophilia A (HEMA) [MIM:306700]
F8	P00451	VAR_028666	p.Gly2045Val	LP/P	-	Hemophilia A (HEMA) [MIM:306700]
F8	P00451	VAR_028667	p.Ile2056Asn	LP/P	-	Hemophilia A (HEMA) [MIM:306700]
F8	P00451	VAR_028668	p.Ala2058Pro	LP/P	-	Hemophilia A (HEMA) [MIM:306700]
F8	P00451	VAR_028669	p.Pro2067Leu	LP/P	rs1348849974	Hemophilia A (HEMA) [MIM:306700]
F8	P00451	VAR_028670	p.Ala2070Val	LP/P	-	Hemophilia A (HEMA) [MIM:306700]
F8	P00451	VAR_028671	p.Ser2082Asn	LP/P	rs1569559494	Hemophilia A (HEMA) [MIM:306700]
F8	P00451	VAR_028672	p.Asp2093Tyr	LP/P	-	Hemophilia A (HEMA) [MIM:306700]
F8	P00451	VAR_028673	p.Gln2106Glu	LP/P	-	Hemophilia A (HEMA) [MIM:306700]
F8	P00451	VAR_028674	p.Gln2106Arg	LP/P	-	Hemophilia A (HEMA) [MIM:306700]
F8	P00451	VAR_028675	p.Arg2109Cys	LP/P	rs1475665992	Hemophilia A (HEMA) [MIM:306700]
F8	P00451	VAR_028676	p.Ile2117Phe	LP/P	-	Hemophilia A (HEMA) [MIM:306700]
F8	P00451	VAR_028677	p.Ile2117Ser	LP/P	-	Hemophilia A (HEMA) [MIM:306700]
F8	P00451	VAR_028678	p.Gln2119Arg	LP/P	-	Hemophilia A (HEMA) [MIM:306700]
F8	P00451	VAR_028679	p.Phe2120Cys	LP/P	-	Hemophilia A (HEMA) [MIM:306700]
F8	P00451	VAR_028680	p.Phe2145Cys	LP/P	-	Hemophilia A (HEMA) [MIM:306700]
F8	P00451	VAR_028681	p.Asn2157Asp	LP/P	-	Hemophilia A (HEMA) [MIM:306700]
F8	P00451	VAR_028682	p.Pro2162Leu	LP/P	rs1450770782	Hemophilia A (HEMA) [MIM:306700]
F8	P00451	VAR_028683	p.Arg2169Cys	LP/P	rs782641941	Hemophilia A (HEMA) [MIM:306700]
F8	P00451	VAR_028684	p.Thr2173Ala	LP/P	-	Hemophilia A (HEMA) [MIM:306700]
F8	P00451	VAR_028685	p.His2174Asp	LP/P	-	Hemophilia A (HEMA) [MIM:306700]

F8	P00451	VAR_028686	p.Arg2182Pro	LP/P	-	Hemophilia A (HEMA) [MIM:306700]
F8	P00451	VAR_028687	p.Met2183Arg	LP/P	rs1405473814	Hemophilia A (HEMA) [MIM:306700]
F8	P00451	VAR_028688	p.Leu2185Trp	LP/P	-	Hemophilia A (HEMA) [MIM:306700]
F8	P00451	VAR_028689	p.Ser2192Ile	LP/P	rs782098979	Hemophilia A (HEMA) [MIM:306700]
F8	P00451	VAR_028690	p.Pro2196Arg	LP/P	-	Hemophilia A (HEMA) [MIM:306700]
F8	P00451	VAR_028691	p.Gly2198Val	LP/P	-	Hemophilia A (HEMA) [MIM:306700]
F8	P00451	VAR_028692	p.Glu2200Asp	LP/P	-	Hemophilia A (HEMA) [MIM:306700]
F8	P00451	VAR_028693	p.Ala2220Pro	LP/P	rs782548763	Hemophilia A (HEMA) [MIM:306700]
F8	P00451	VAR_028695	p.Pro2224Leu	LP/P	-	Hemophilia A (HEMA) [MIM:306700]
F8	P00451	VAR_028696	p.Arg2228Pro	LP/P	-	Hemophilia A (HEMA) [MIM:306700]
F8	P00451	VAR_028697	p.Leu2229Phe	LP/P	rs1603431508	Hemophilia A (HEMA) [MIM:306700]
F8	P00451	VAR_028698	p.Trp2248Ser	LP/P	-	Hemophilia A (HEMA) [MIM:306700]
F8	P00451	VAR_028699	p.Val2251Ala	LP/P	rs782479558	Hemophilia A (HEMA) [MIM:306700]
F8	P00451	VAR_028700	p.Val2251Glu	LP/P	rs782479558	Hemophilia A (HEMA) [MIM:306700]
F8	P00451	VAR_028701	p.Thr2264Ala	LP/P	-	Hemophilia A (HEMA) [MIM:306700]
F8	P00451	VAR_028702	p.Phe2279Cys	LP/P	-	Hemophilia A (HEMA) [MIM:306700]
F8	P00451	VAR_028703	p.Phe2279Ile	LP/P	rs782717799	Hemophilia A (HEMA) [MIM:306700]
F8	P00451	VAR_028704	p.Ile2281Thr	LP/P	-	Hemophilia A (HEMA) [MIM:306700]
F8	P00451	VAR_028705	p.Trp2290Leu	LP/P	-	Hemophilia A (HEMA) [MIM:306700]
F8	P00451	VAR_028706	p.Gly2304Val	LP/P	-	Hemophilia A (HEMA) [MIM:306700]
F8	P00451	VAR_028707	p.Arg2323Gly	LP/P	rs137852473	Hemophilia A (HEMA) [MIM:306700]
F8	P00451	VAR_028708	p.Arg2323Leu	LP/P	-	Hemophilia A (HEMA) [MIM:306700]
F8	P00451	VAR_028709	p.Arg2326Gly	LP/P	-	Hemophilia A (HEMA) [MIM:306700]
F8	P00451	VAR_028710	p.Arg2326Pro	LP/P	rs137852360	Hemophilia A (HEMA) [MIM:306700]
F8	P00451	VAR_028711	p.Gln2330Pro	LP/P	-	Hemophilia A (HEMA) [MIM:306700]
F8	P00451	VAR_028712	p.Trp2332Arg	LP/P	-	Hemophilia A (HEMA) [MIM:306700]
F8	P00451	VAR_028713	p.Arg2339Thr	LP/P	-	Hemophilia A (HEMA) [MIM:306700]
F8	P00451	VAR_028714	p.Gly2344Ser	LP/P	-	Hemophilia A (HEMA) [MIM:306700]
F8	P00451	VAR_028715	p.Cys2345Ser	LP/P	-	Hemophilia A (HEMA) [MIM:306700]
F8	P00451	VAR_028716	p.Cys2345Tyr	LP/P	-	Hemophilia A (HEMA) [MIM:306700]
F8	P00451	VAR_048438	p.Lys1289Gln	LB/B	rs1800292	-
F8	P00451	VAR_065303	p.Pro83Arg	LP/P	rs781974394	Hemophilia A (HEMA) [MIM:306700]
F8	P00451	VAR_065304	p.Asp186Asn	LP/P	-	Hemophilia A (HEMA) [MIM:306700]
F8	P00451	VAR_065305	p.Leu191Phe	LP/P	rs1341730743	Hemophilia A (HEMA) [MIM:306700]
F8	P00451	VAR_065306	p.Leu195Pro	LP/P	-	Hemophilia A (HEMA) [MIM:306700]
F8	P00451	VAR_065307	p.Leu261Pro	LP/P	-	Hemophilia A (HEMA) [MIM:306700]
F8	P00451	VAR_065308	p.Gly280Asp	LP/P	-	Hemophilia A (HEMA) [MIM:306700]
F8	P00451	VAR_065309	p.Ala394Ser	LP/P	-	Hemophilia A (HEMA) [MIM:306700]
F8	P00451	VAR_065310	p.Gly474Val	LP/P	-	Hemophilia A (HEMA) [MIM:306700]
F8	P00451	VAR_065311	p.Pro496Arg	LP/P	-	Hemophilia A (HEMA) [MIM:306700]
F8	P00451	VAR_065312	p.Gly513Val	LP/P	-	Hemophilia A (HEMA) [MIM:306700]
F8	P00451	VAR_065313	p.Pro569Arg	LP/P	-	Hemophilia A (HEMA) [MIM:306700]
F8	P00451	VAR_065314	p.Asn637Ile	LP/P	-	Hemophilia A (HEMA) [MIM:306700]
F8	P00451	VAR_065315	p.Ala1720Val	LP/P	-	Hemophilia A (HEMA) [MIM:306700]
F8	P00451	VAR_065316	p.Phe1762Leu	LP/P	-	Hemophilia A (HEMA) [MIM:306700]
F8	P00451	VAR_065317	p.Cys1877Tyr	LP/P	-	Hemophilia A (HEMA) [MIM:306700]
F8	P00451	VAR_065318	p.Gly2013Arg	LP/P	-	Hemophilia A (HEMA) [MIM:306700]
F8	P00451	VAR_065319	p.His2101Asp	LP/P	rs1603432783	Hemophilia A (HEMA) [MIM:306700]
F8	P00451	VAR_065320	p.Gln2106Pro	LP/P	-	Hemophilia A (HEMA) [MIM:306700]

F8	P00451	VAR_065321	p.Met2143Val	LP/P	-	Hemophilia A (HEMA) [MIM:306700]
F8	P00451	VAR_065322	p.Pro2172Leu	LP/P	-	Hemophilia A (HEMA) [MIM:306700]
F8	P00451	VAR_065323	p.Asp2286Gly	LP/P	-	Hemophilia A (HEMA) [MIM:306700]
F8	P00451	VAR_065324	p.Ile2336Phe	LP/P	-	Hemophilia A (HEMA) [MIM:306700]
F8	P00451	VAR_065325	p.Gly2344Asp	LP/P	rs1557271042	Hemophilia A (HEMA) [MIM:306700]
F8	P00451	VAR_075624	p.Pro1828Leu	US	-	Hemophilia A (HEMA) [MIM:306700]
F9	P00740	VAR_006520	p.Ile7Phe	LB/B	rs150190385	-
F9	P00740	VAR_006521	p.Ile17Asn	LP/P	-	Hemophilia B (HEMB) [MIM:306900]
F9	P00740	VAR_006522	p.Cys28Arg	LP/P	rs387906481	Hemophilia B (HEMB) [MIM:306900]
F9	P00740	VAR_006523	p.Val30Ile	LP/P	-	Hemophilia B (HEMB) [MIM:306900]
F9	P00740	VAR_006524	p.Arg43Gln	LP/P	rs1275708479	Hemophilia B (HEMB) [MIM:306900]
F9	P00740	VAR_006525	p.Arg43Leu	LP/P	rs1275708479	Hemophilia B (HEMB) [MIM:306900]
F9	P00740	VAR_006526	p.Arg43Trp	LP/P	rs1603264205	Hemophilia B (HEMB) [MIM:306900]
F9	P00740	VAR_006527	p.Lys45Asn	LP/P	-	Hemophilia B (HEMB) [MIM:306900]
F9	P00740	VAR_006528	p.Arg46Ser	LP/P	-	Hemophilia B (HEMB) [MIM:306900]
F9	P00740	VAR_006529	p.Arg46Thr	LP/P	-	Hemophilia B (HEMB) [MIM:306900]
F9	P00740	VAR_006530	p.Asn48Ile	LP/P	-	Hemophilia B (HEMB) [MIM:306900]
F9	P00740	VAR_006531	p.Ser49Pro	LP/P	-	Hemophilia B (HEMB) [MIM:306900]
F9	P00740	VAR_006532	p.Glu53Ala	LP/P	-	Hemophilia B (HEMB) [MIM:306900]
F9	P00740	VAR_006533	p.Glu54Gly	LP/P	-	Hemophilia B (HEMB) [MIM:306900]
F9	P00740	VAR_006534	p.Phe55Cys	LP/P	-	Hemophilia B (HEMB) [MIM:306900]
F9	P00740	VAR_006535	p.Gly58Ala	LP/P	-	Hemophilia B (HEMB) [MIM:306900]
F9	P00740	VAR_006536	p.Gly58Arg	LP/P	-	Hemophilia B (HEMB) [MIM:306900]
F9	P00740	VAR_006538	p.Glu66Val	LP/P	-	Hemophilia B (HEMB) [MIM:306900]
F9	P00740	VAR_006539	p.Glu67Lys	LP/P	rs1410080079	Hemophilia B (HEMB) [MIM:306900]
F9	P00740	VAR_006540	p.Phe71Ser	LP/P	-	Hemophilia B (HEMB) [MIM:306900]
F9	P00740	VAR_006541	p.Glu73Lys	LP/P	rs137852225	Hemophilia B (HEMB) [MIM:306900]
F9	P00740	VAR_006542	p.Glu73Val	LP/P	rs137852226	Hemophilia B (HEMB) [MIM:306900]
F9	P00740	VAR_006543	p.Tyr91Cys	LP/P	-	Hemophilia B (HEMB) [MIM:306900]
F9	P00740	VAR_006544	p.Asp93Gly	LP/P	rs137852230	Hemophilia B (HEMB) [MIM:306900]
F9	P00740	VAR_006545	p.Gln96Pro	LP/P	rs137852231	Hemophilia B (HEMB) [MIM:306900]
F9	P00740	VAR_006546	p.Cys97Ser	LP/P	-	Hemophilia B (HEMB) [MIM:306900]
F9	P00740	VAR_006547	p.Pro101Arg	LP/P	-	Hemophilia B (HEMB) [MIM:306900]
F9	P00740	VAR_006548	p.Cys102Arg	LP/P	rs1603264719	Hemophilia B (HEMB) [MIM:306900]
F9	P00740	VAR_006549	p.Gly106Ser	LP/P	rs137852233	Hemophilia B (HEMB) [MIM:306900]
F9	P00740	VAR_006550	p.Cys108Ser	LP/P	-	Hemophilia B (HEMB) [MIM:306900]
F9	P00740	VAR_006551	p.Asp110Asn	LP/P	rs137852274	Hemophilia B (HEMB) [MIM:306900]
F9	P00740	VAR_006552	p.Ile112Ser	LP/P	-	Hemophilia B (HEMB) [MIM:306900]
F9	P00740	VAR_006553	p.Asn113Lys	LP/P	-	Hemophilia B (HEMB) [MIM:306900]
F9	P00740	VAR_006554	p.Tyr115Cys	LP/P	rs1603264727	Hemophilia B (HEMB) [MIM:306900]
F9	P00740	VAR_006555	p.Cys119Phe	LP/P	-	Hemophilia B (HEMB) [MIM:306900]
F9	P00740	VAR_006556	p.Cys119Arg	LP/P	-	Hemophilia B (HEMB) [MIM:306900]
F9	P00740	VAR_006557	p.Gly125Glu	LP/P	-	Hemophilia B (HEMB) [MIM:306900]
F9	P00740	VAR_006558	p.Gly125Val	LP/P	-	Hemophilia B (HEMB) [MIM:306900]
F9	P00740	VAR_006560	p.Ile136Thr	LP/P	rs1603265481	Hemophilia B (HEMB) [MIM:306900]
F9	P00740	VAR_006561	p.Gly139Asp	LP/P	rs1216516070	Hemophilia B (HEMB) [MIM:306900]
F9	P00740	VAR_006562	p.Gly139Ser	LP/P	-	Hemophilia B (HEMB) [MIM:306900]
F9	P00740	VAR_006563	p.Cys155Phe	LP/P	rs1330705989	Hemophilia B (HEMB) [MIM:306900]
F9	P00740	VAR_006564	p.Gly160Glu	LP/P	-	Hemophilia B (HEMB) [MIM:306900]

F9	P00740	VAR_006565	p.Gln167His	LP/P	-	Hemophilia B (HEMB) [MIM:306900]
F9	P00740	VAR_006566	p.Cys178Arg	LP/P	-	Hemophilia B (HEMB) [MIM:306900]
F9	P00740	VAR_006567	p.Cys178Trp	LP/P	-	Hemophilia B (HEMB) [MIM:306900]
F9	P00740	VAR_006568	p.Arg191His	LP/P	rs137852238	Hemophilia B (HEMB) [MIM:306900]
F9	P00740	VAR_006569	p.Arg191Cys	LP/P	rs137852237	Hemophilia B (HEMB) [MIM:306900]
F9	P00740	VAR_006570	p.Arg226Trp	LP/P	rs137852240	Hemophilia B (HEMB) [MIM:306900]
F9	P00740	VAR_006571	p.Arg226Gly	LP/P	-	Hemophilia B (HEMB) [MIM:306900]
F9	P00740	VAR_006572	p.Arg226Gln	LP/P	rs137852241	Hemophilia B (HEMB) [MIM:306900]
F9	P00740	VAR_006573	p.Val227Asp	LP/P	-	Hemophilia B (HEMB) [MIM:306900]
F9	P00740	VAR_006574	p.Val228Leu	LP/P	rs137852243	Hemophilia B (HEMB) [MIM:306900]
F9	P00740	VAR_006575	p.Gln241His	LP/P	rs1182648920	Hemophilia B (HEMB) [MIM:306900]
F9	P00740	VAR_006576	p.Gly253Glu	LP/P	-	Hemophilia B (HEMB) [MIM:306900]
F9	P00740	VAR_006577	p.Gly253Arg	LP/P	rs1603267181	Hemophilia B (HEMB) [MIM:306900]
F9	P00740	VAR_006578	p.Ala265Thr	LP/P	-	Hemophilia B (HEMB) [MIM:306900]
F9	P00740	VAR_006579	p.Ala279Thr	LP/P	rs137852247	Hemophilia B (HEMB) [MIM:306900]
F9	P00740	VAR_006580	p.Asn283Asp	LP/P	-	Hemophilia B (HEMB) [MIM:306900]
F9	P00740	VAR_006582	p.Arg294Gly	LP/P	-	Hemophilia B (HEMB) [MIM:306900]
F9	P00740	VAR_006583	p.Arg294Gln	LP/P	rs137852249	Hemophilia B (HEMB) [MIM:306900]
F9	P00740	VAR_006584	p.His302Arg	LP/P	-	Hemophilia B (HEMB) [MIM:306900]
F9	P00740	VAR_006585	p.Ile316Phe	LP/P	-	Hemophilia B (HEMB) [MIM:306900]
F9	P00740	VAR_006586	p.Leu321Gln	LP/P	-	Hemophilia B (HEMB) [MIM:306900]
F9	P00740	VAR_006587	p.Pro333His	LP/P	-	Hemophilia B (HEMB) [MIM:306900]
F9	P00740	VAR_006588	p.Thr342Lys	LP/P	-	Hemophilia B (HEMB) [MIM:306900]
F9	P00740	VAR_006589	p.Thr342Met	LP/P	rs137852254	Hemophilia B (HEMB) [MIM:306900]
F9	P00740	VAR_006590	p.Gly351Asp	LP/P	-	Hemophilia B (HEMB) [MIM:306900]
F9	P00740	VAR_006591	p.Trp356Cys	LP/P	-	Hemophilia B (HEMB) [MIM:306900]
F9	P00740	VAR_006592	p.Gly357Glu	LP/P	rs137852275	Hemophilia B (HEMB) [MIM:306900]
F9	P00740	VAR_006593	p.Lys362Glu	LP/P	-	Hemophilia B (HEMB) [MIM:306900]
F9	P00740	VAR_006594	p.Gly363Trp	LP/P	-	Hemophilia B (HEMB) [MIM:306900]
F9	P00740	VAR_006595	p.Ala366Asp	LP/P	-	Hemophilia B (HEMB) [MIM:306900]
F9	P00740	VAR_006596	p.Arg379Gly	LP/P	rs137852258	Hemophilia B (HEMB) [MIM:306900]
F9	P00740	VAR_006597	p.Arg379Gln	LP/P	rs137852259	Hemophilia B (HEMB) [MIM:306900]
F9	P00740	VAR_006598	p.Cys382Tyr	LP/P	rs1303221289	Hemophilia B (HEMB) [MIM:306900]
F9	P00740	VAR_006599	p.Lys387Glu	LP/P	-	Hemophilia B (HEMB) [MIM:306900]
F9	P00740	VAR_006600	p.Ile390Phe	LP/P	-	Hemophilia B (HEMB) [MIM:306900]
F9	P00740	VAR_006601	p.Met394Lys	LP/P	-	Hemophilia B (HEMB) [MIM:306900]
F9	P00740	VAR_006602	p.Cys396Ser	LP/P	rs137852273	Hemophilia B (HEMB) [MIM:306900]
F9	P00740	VAR_006603	p.Arg404Thr	LP/P	-	Hemophilia B (HEMB) [MIM:306900]
F9	P00740	VAR_006604	p.Cys407Ser	LP/P	-	Hemophilia B (HEMB) [MIM:306900]
F9	P00740	VAR_006605	p.Gly413Arg	LP/P	rs1306658513	Hemophilia B (HEMB) [MIM:306900]
F9	P00740	VAR_006606	p.Val419Glu	LP/P	rs137852280	Hemophilia B (HEMB) [MIM:306900]
F9	P00740	VAR_006607	p.Phe424Val	LP/P	-	Hemophilia B (HEMB) [MIM:306900]
F9	P00740	VAR_006608	p.Thr426Pro	LP/P	-	Hemophilia B (HEMB) [MIM:306900]
F9	P00740	VAR_006609	p.Ser430Thr	LP/P	-	Hemophilia B (HEMB) [MIM:306900]
F9	P00740	VAR_006610	p.Trp431Gly	LP/P	-	Hemophilia B (HEMB) [MIM:306900]
F9	P00740	VAR_006611	p.Trp431Arg	LP/P	-	Hemophilia B (HEMB) [MIM:306900]
F9	P00740	VAR_006612	p.Gly432Ser	LP/P	rs1170838100	Hemophilia B (HEMB) [MIM:306900]
F9	P00740	VAR_006613	p.Gly432Val	LP/P	-	Hemophilia B (HEMB) [MIM:306900]
F9	P00740	VAR_006614	p.Glu433Ala	LP/P	-	Hemophilia B (HEMB) [MIM:306900]

F9	P00740	VAR_006615	p.Glu433Lys	LP/P	rs767828752	Hemophilia B (HEMB) [MIM:306900]
F9	P00740	VAR_006616	p.Ala436Val	LP/P	rs137852266	Hemophilia B (HEMB) [MIM:306900]
F9	P00740	VAR_006618	p.Arg449Gln	LP/P	rs143018900	Hemophilia B (HEMB) [MIM:306900]
F9	P00740	VAR_006619	p.Arg449Trp	LP/P	rs757996262	Hemophilia B (HEMB) [MIM:306900]
F9	P00740	VAR_006620	p.Tyr450Cys	LP/P	rs1243180674	Hemophilia B (HEMB) [MIM:306900]
F9	P00740	VAR_006621	p.Ile454Thr	LP/P	rs1603267486	Hemophilia B (HEMB) [MIM:306900]
F9	P00740	VAR_011773	p.Thr194Ala	LB/B	rs6048	-
F9	P00740	VAR_014308	p.Thr461Pro	LB/B	rs4149751	-
F9	P00740	VAR_017307	p.Ala37Thr	LP/P	rs367569299	Warfarin sensitivity, X-linked (WARFS) [MIM:301052]
F9	P00740	VAR_017308	p.Arg75Gln	LP/P	rs137852228	Hemophilia B (HEMB) [MIM:306900]
F9	P00740	VAR_017309	p.Glu79Asp	LP/P	rs137852229	Hemophilia B (HEMB) [MIM:306900]
F9	P00740	VAR_017310	p.Val227Phe	LP/P	rs137852242	Hemophilia B (HEMB) [MIM:306900]
F9	P00740	VAR_017311	p.Val228Phe	LP/P	rs137852243	Hemophilia B (HEMB) [MIM:306900]
F9	P00740	VAR_017312	p.Cys252Ser	LP/P	rs267606792	Hemophilia B (HEMB) [MIM:306900]
F9	P00740	VAR_017313	p.Cys268Trp	LP/P	rs137852246	Hemophilia B (HEMB) [MIM:306900]
F9	P00740	VAR_017314	p.Glu291Val	LP/P	rs137852279	Hemophilia B (HEMB) [MIM:306900]
F9	P00740	VAR_017315	p.Asn306Ser	LP/P	rs137852251	Hemophilia B (HEMB) [MIM:306900]
F9	P00740	VAR_017316	p.Gly357Arg	LP/P	rs137852257	Hemophilia B (HEMB) [MIM:306900]
F9	P00740	VAR_017317	p.Ala397Pro	LP/P	rs137852281	Hemophilia B (HEMB) [MIM:306900]
F9	P00740	VAR_017318	p.Asp410His	LP/P	rs137852278	Hemophilia B (HEMB) [MIM:306900]
F9	P00740	VAR_017319	p.Ser411Ile	LP/P	rs137852276	Hemophilia B (HEMB) [MIM:306900]
F9	P00740	VAR_017320	p.Ser411Gly	LP/P	rs137852277	Hemophilia B (HEMB) [MIM:306900]
F9	P00740	VAR_017321	p.Pro414Thr	LP/P	rs137852265	Hemophilia B (HEMB) [MIM:306900]
F9	P00740	VAR_017322	p.Gly442Arg	LP/P	rs137852267	Hemophilia B (HEMB) [MIM:306900]
F9	P00740	VAR_017323	p.Ile443Thr	LP/P	rs137852268	Hemophilia B (HEMB) [MIM:306900]
F9	P00740	VAR_017324	p.Trp453Arg	LP/P	rs137852269	Hemophilia B (HEMB) [MIM:306900]
F9	P00740	VAR_017343	p.Cys28Tyr	LP/P	-	Hemophilia B (HEMB) [MIM:306900]
F9	P00740	VAR_017344	p.Leu52Ser	LP/P	-	Hemophilia B (HEMB) [MIM:306900]
F9	P00740	VAR_017345	p.Thr84Arg	LP/P	-	Hemophilia B (HEMB) [MIM:306900]
F9	P00740	VAR_017346	p.Gly106Asp	LP/P	-	Hemophilia B (HEMB) [MIM:306900]
F9	P00740	VAR_017347	p.Glu124Lys	LP/P	-	Hemophilia B (HEMB) [MIM:306900]
F9	P00740	VAR_017348	p.Gly125Arg	LP/P	-	Hemophilia B (HEMB) [MIM:306900]
F9	P00740	VAR_017349	p.Cys134Tyr	LP/P	-	Hemophilia B (HEMB) [MIM:306900]
F9	P00740	VAR_017350	p.Ser169Cys	LP/P	-	Hemophilia B (HEMB) [MIM:306900]
F9	P00740	VAR_017351	p.Cys170Phe	LP/P	-	Hemophilia B (HEMB) [MIM:306900]
F9	P00740	VAR_017352	p.Gln241Lys	LP/P	-	Hemophilia B (HEMB) [MIM:306900]
F9	P00740	VAR_017353	p.Cys252Tyr	LP/P	-	Hemophilia B (HEMB) [MIM:306900]
F9	P00740	VAR_017354	p.Leu318Arg	LP/P	rs1222227572	Hemophilia B (HEMB) [MIM:306900]
F9	P00740	VAR_017355	p.Pro333Thr	LP/P	-	Hemophilia B (HEMB) [MIM:306900]
F9	P00740	VAR_017356	p.Ile344Leu	LP/P	-	Hemophilia B (HEMB) [MIM:306900]
F9	P00740	VAR_017357	p.Leu383Ile	LP/P	-	Hemophilia B (HEMB) [MIM:306900]
F9	P00740	VAR_017358	p.Leu383Phe	LP/P	-	Hemophilia B (HEMB) [MIM:306900]
F9	P00740	VAR_017359	p.Phe395Ile	LP/P	rs1175050951	Hemophilia B (HEMB) [MIM:306900]
F9	P00740	VAR_017360	p.Phe395Leu	LP/P	rs1175050951	Hemophilia B (HEMB) [MIM:306900]
F9	P00740	VAR_017361	p.Cys396Phe	LP/P	-	Hemophilia B (HEMB) [MIM:306900]
F9	P00740	VAR_017362	p.Cys407Arg	LP/P	-	Hemophilia B (HEMB) [MIM:306900]
F9	P00740	VAR_017363	p.Gly412Glu	LP/P	rs1233706534	Hemophilia B (HEMB) [MIM:306900]
F9	P00740	VAR_017364	p.Cys435Tyr	LP/P	rs1385141619	Hemophilia B (HEMB) [MIM:306900]
F9	P00740	VAR_017365	p.Gly442Glu	LP/P	rs1603267474	Hemophilia B (HEMB) [MIM:306900]

F9 P00740 VAR_062999 p.Arg384Leu LP/P rs137852283 Thrombophilia, X-linked, due to factor IX defect (THPH8) [MIM:300807]
F9 P00740 VAR_073975 p.Leu20Ser US - Hemophilia B (HEMB) [MIM:306900]
F9 P00740 VAR_073976 p.Glu54Asp US - Hemophilia B (HEMB) [MIM:306900]
F9 P00740 VAR_073977 p.Gly58Glu US - Hemophilia B (HEMB) [MIM:306900]
F9 P00740 VAR_073978 p.Asn138His US - Hemophilia B (HEMB) [MIM:306900]
F9 P00740 VAR_073980 p.Val296Met US - Hemophilia B (HEMB) [MIM:306900]
F9 P00740 VAR_073981 p.Asn328Lys US - Hemophilia B (HEMB) [MIM:306900]
F9 P00740 VAR_073982 p.Asn328Tyr LP/P - Hemophilia B (HEMB) [MIM:306900]
F9 P00740 VAR_083981 p.Ala37Val LP/P rs1327097914 Warfarin sensitivity, X-linked (WARFS) [MIM:301052]
FA2H Q7L5A8 VAR_037503 p.Pro97Ala LB/B rs35874850 -
FA2H Q7L5A8 VAR_054893 p.Asp35Tyr LP/P rs121918217 Spastic paraplegia 35, autosomal recessive (SPG35) [MIM:612319]
FA2H Q7L5A8 VAR_064621 p.Arg235Cys LP/P rs387907039 Spastic paraplegia 35, autosomal recessive (SPG35) [MIM:612319]
FA2H Q7L5A8 VAR_065245 p.Arg154Cys LP/P rs387907040 Spastic paraplegia 35, autosomal recessive (SPG35) [MIM:612319]
FAAH O00519 VAR_013563 p.Pro129Thr LB/B rs324420 -
FAAH O00519 VAR_035704 p.Ala345Asp US rs772931153 A breast cancer sample
FAAP100 Q0VG06 VAR_032582 p.Pro660Leu LB/B rs11552304 -
FAAP100 Q0VG06 VAR_032583 p.Thr817Ala LB/B rs14422 -
FAAP20 Q6NZ36 VAR_038434 p.Pro126Ser LB/B rs1058411 -
FAAP24 Q9BTP7 VAR_029828 p.Ser158Leu LB/B rs2304103 -
FAAP24 Q9BTP7 VAR_029829 p.Ile192Thr LB/B rs3816032 -
FAAP24 Q9BTP7 VAR_050989 p.Ser126Phe LB/B rs36017455 -
FABP1 P07148 VAR_014662 p.Ala54Thr LB/B rs1801273 -
FABP1 P07148 VAR_022093 p.Thr94Ala LB/B rs2241883 -
FABP2 P12104 VAR_002379 p.Ala55Thr LB/B rs1799883 -
FABP3 P05413 VAR_061165 p.Lys53Arg LB/B rs2228194 -
FABP4 P15090 VAR_036320 p.Glu23Asp US rs1319351179 A breast cancer sample
FABP6 P51161 VAR_039578 p.Arg33His LB/B rs17856662 -
FABP6 P51161 VAR_039579 p.Ser55Tyr LB/B rs17852045 -
FABP6 P51161 VAR_039580 p.Thr79Met LB/B rs1130435 -
FABP7 O15540 VAR_049012 p.Thr61Met LB/B rs2279381 -
FADD Q13158 VAR_065124 p.Cys105Trp LP/P rs387906839 Infections, recurrent, associated with encephalopathy, hepatic dysfunction and cardiovascular malformations (IEHDCM) [MIM:613759]
FADS1 O60427 VAR_035340 p.Pro272Ser LB/B rs17856235 -
FADS3 Q9Y5Q0 VAR_035341 p.Lys192Asn LB/B rs35479241 -
FADS3 Q9Y5Q0 VAR_035342 p.Asn216Lys LB/B rs34511441 -
FAH P16930 VAR_005205 p.Asn16Ile LP/P rs121965073 Tyrosinemia 1 (TYRSN1) [MIM:276700]
FAH P16930 VAR_005206 p.Phe62Cys LP/P - Tyrosinemia 1 (TYRSN1) [MIM:276700]
FAH P16930 VAR_005207 p.Gln64His LP/P rs80338894 Tyrosinemia 1 (TYRSN1) [MIM:276700]
FAH P16930 VAR_005208 p.Ala134Asp LP/P rs121965074 Tyrosinemia 1 (TYRSN1) [MIM:276700]
FAH P16930 VAR_005209 p.Gly158Asp LP/P - Tyrosinemia 1 (TYRSN1) [MIM:276700]
FAH P16930 VAR_005210 p.Val166Gly LP/P rs778387055 Tyrosinemia 1 (TYRSN1) [MIM:276700]
FAH P16930 VAR_005211 p.Cys193Arg LP/P - Tyrosinemia 1 (TYRSN1) [MIM:276700]
FAH P16930 VAR_005212 p.Gly207Asp LP/P rs754196530 Tyrosinemia 1 (TYRSN1) [MIM:276700]
FAH P16930 VAR_005213 p.Asp233Val LP/P rs80338897 Tyrosinemia 1 (TYRSN1) [MIM:276700]

FAH P16930 VAR_005214 p.Trp234Gly LP/P rs1555441595 Tyrosinemia 1 (TYRSN1) [MIM:276700]
FAH P16930 VAR_005215 p.Pro249Thr LP/P - Tyrosinemia 1 (TYRSN1) [MIM:276700]
FAH P16930 VAR_005216 p.Pro261Leu LP/P rs80338898 Tyrosinemia 1 (TYRSN1) [MIM:276700]
FAH P16930 VAR_005217 p.Thr294Pro LP/P rs370634385 Tyrosinemia 1 (TYRSN1) [MIM:276700]
FAH P16930 VAR_005218 p.Gly337Ser LP/P rs80338900 Tyrosinemia 1 (TYRSN1) [MIM:276700]
FAH P16930 VAR_005219 p.Arg341Trp LB/B rs11555096 -
FAH P16930 VAR_005220 p.Pro342Leu LP/P rs779040832 Tyrosinemia 1 (TYRSN1) [MIM:276700]
FAH P16930 VAR_005222 p.Gly369Val LP/P - Tyrosinemia 1 (TYRSN1) [MIM:276700]
FAH P16930 VAR_005223 p.Arg381Gly LP/P rs121965077 Tyrosinemia 1 (TYRSN1) [MIM:276700]
FAH P16930 VAR_005224 p.Phe405His LP/P - Tyrosinemia 1 (TYRSN1) [MIM:276700]
FAH P16930 VAR_065454 p.Ala35Thr LP/P - Tyrosinemia 1 (TYRSN1) [MIM:276700]
FAH P16930 VAR_065455 p.Gln279Arg LP/P rs121965078 Tyrosinemia 1 (TYRSN1) [MIM:276700]
FAHD1 Q6P587 VAR_049014 p.Asp110Asn LB/B rs3743853 -
FAHD2A Q96GK7 VAR_049015 p.Met198Thr LB/B rs1045332 -
FAIM Q9NVQ4 VAR_024314 p.Ala117Thr LB/B rs641320 -
FAIM Q9NVQ4 VAR_024315 p.Leu127Ser LB/B rs13043 -
FAM104B Q5XKR9 VAR_055796 p.Ser60Gly LB/B rs1047037 -
FAM107A O95990 VAR_017238 p.Leu15Met US - Ovarian cancer
FAM107A O95990 VAR_017238 p.Leu15Met US - Renal cell carcinoma cell lines
FAM107A O95990 VAR_017239 p.Pro19Leu US - Renal cell carcinoma cell line
FAM107A O95990 VAR_049016 p.Ala89Ser LB/B rs1043942 -
FAM107A O95990 VAR_049017 p.Glu141Gln LB/B rs11539086 -
FAM110A Q9BQ89 VAR_060150 p.Leu284Ile LB/B rs6055290 -
FAM110B Q8TC76 VAR_036319 p.Ala214Ser US rs150740446 A colorectal cancer sample
FAM110D Q8TAY7 VAR_038860 p.His53Arg LB/B rs3748856 -
FAM111A Q96PZ2 VAR_069513 p.Thr338Ala LP/P rs587777014 Gracile bone dysplasia (GCLEB) [MIM:602361]
FAM111A Q96PZ2 VAR_069515 p.Tyr511His LP/P rs587777012 Kenny-Caffey syndrome 2 (KCS2) [MIM:127000]
FAM111A Q96PZ2 VAR_069516 p.Pro527Thr LP/P rs587777015 Gracile bone dysplasia (GCLEB) [MIM:602361]
FAM111A Q96PZ2 VAR_069517 p.Asp528Gly LP/P rs587777013 Gracile bone dysplasia (GCLEB) [MIM:602361]
FAM111A Q96PZ2 VAR_069518 p.Arg569His LP/P rs587777011 Kenny-Caffey syndrome 2 (KCS2) [MIM:127000]
FAM111B Q6SJ93 VAR_030282 p.Pro731Ala LB/B rs17153376 -
FAM111B Q6SJ93 VAR_053821 p.Gly218Asp LB/B rs1060428 -
FAM111B Q6SJ93 VAR_070953 p.Tyr621Asp LP/P rs587777236 Poikiloderma, hereditary fibrosing, with tendon contractures, myopathy, and pulmonary fibrosis (POIKTMP) [MIM:615704]
FAM111B Q6SJ93 VAR_070954 p.Arg627Gly LP/P rs587777237 Poikiloderma, hereditary fibrosing, with tendon contractures, myopathy, and pulmonary fibrosis (POIKTMP) [MIM:615704]
FAM111B Q6SJ93 VAR_070955 p.Ser628Asn LP/P rs587777238 Poikiloderma, hereditary fibrosing, with tendon contractures, myopathy, and pulmonary fibrosis (POIKTMP) [MIM:615704]
FAM114A1 Q8IWE2 VAR_030320 p.Gly84Arg LB/B rs11096964 -
FAM114A1 Q8IWE2 VAR_030321 p.Leu116Pro LB/B rs11555334 -
FAM114A1 Q8IWE2 VAR_030322 p.Pro201Leu LB/B rs430296 -
FAM114A1 Q8IWE2 VAR_030323 p.Arg367His LB/B rs2306923 -
FAM114A1 Q8IWE2 VAR_030324 p.Val443Ile LB/B rs17429619 -
FAM114A1 Q8IWE2 VAR_053823 p.Ser40Leu LB/B rs34137542 -
FAM114A1 Q8IWE2 VAR_053824 p.Ser446Leu LB/B rs36058104 -
FAM118A Q9NWS6 VAR_022808 p.Val129Leu LB/B rs11556482 -
FAM118A Q9NWS6 VAR_022809 p.Arg239His LB/B rs6007594 -
FAM120A Q9NZB2 VAR_054400 p.Tyr327His LB/B rs11541747 -
FAM120AOS Q5T036 VAR_043677 p.Leu22Phe LB/B rs1055710 -

FAM120AOS	Q5T036	VAR_043678	p.Lys241Glu	LB/B	rs10821128	-
FAM120B	Q96EK7	VAR_043021	p.Asp370Tyr	LB/B	rs6917485	-
FAM120B	Q96EK7	VAR_043022	p.Met379Thr	LB/B	rs6905356	-
FAM120B	Q96EK7	VAR_043023	p.Tyr428Cys	LB/B	rs6900199	-
FAM120B	Q96EK7	VAR_043024	p.Asp430Gly	LB/B	rs6900202	-
FAM120B	Q96EK7	VAR_043025	p.Ser431Pro	LB/B	rs6905610	-
FAM120B	Q96EK7	VAR_043026	p.Pro433Ala	LB/B	rs6934830	-
FAM120B	Q96EK7	VAR_043027	p.Tyr440Cys	LB/B	rs9366138	-
FAM120B	Q96EK7	VAR_043028	p.Cys511Gly	LB/B	rs9348266	-
FAM120C	Q9NX05	VAR_047538	p.Ile82Thr	LB/B	rs2495783	-
FAM120C	Q9NX05	VAR_062001	p.Met934Ile	LB/B	rs41304786	-
FAM124A	Q86V42	VAR_032098	p.Asp181His	LB/B	rs17075482	-
FAM124B	Q9H5Z6	VAR_060151	p.Ile257Thr	LB/B	rs3738954	-
FAM126A	Q9BYI3	VAR_030647	p.Leu53Pro	LP/P	rs72549407	Leukodystrophy, hypomyelinating, 5 (HLD5) [MIM:610532]
FAM126A	Q9BYI3	VAR_075100	p.Cys57Arg	LP/P	-	Leukodystrophy, hypomyelinating, 5 (HLD5) [MIM:610532]
FAM126A	Q9BYI3	VAR_079751	p.Arg217Gln	LB/B	rs192409840	-
FAM131A	Q6UXB0	VAR_036118	p.Ser291Asn	US	-	A breast cancer sample
FAM131B	Q86XD5	VAR_053920	p.Ala307Thr	LB/B	rs17854363	-
FAM131C	Q96AQ9	VAR_031130	p.Lys48Glu	LB/B	rs2863458	-
FAM131C	Q96AQ9	VAR_031131	p.Arg107Gln	LB/B	rs71510977	-
FAM131C	Q96AQ9	VAR_031132	p.Pro123Arg	LB/B	rs11576236	-
FAM131C	Q96AQ9	VAR_031133	p.Ser215Ile	LB/B	rs1832151	-
FAM131C	Q96AQ9	VAR_031134	p.Glu226Asp	LB/B	rs17853749	-
FAM133A	Q8N9E0	VAR_053907	p.Glu67Lys	LB/B	rs34123774	-
FAM135A	Q9P2D6	VAR_037854	p.Ile725Val	LB/B	rs9455142	-
FAM135A	Q9P2D6	VAR_037855	p.Pro954Ser	LB/B	rs16869301	-
FAM135A	Q9P2D6	VAR_037856	p.Asp1242Gly	LB/B	rs2747701	-
FAM135B	Q49AJ0	VAR_053980	p.Ile477Val	LB/B	rs7835830	-
FAM135B	Q49AJ0	VAR_053981	p.Asp846Asn	LB/B	rs2978180	-
FAM135B	Q49AJ0	VAR_062218	p.Ser578Asn	LB/B	rs57534956	-
FAM135B	Q49AJ0	VAR_062219	p.Val1142Ile	LB/B	rs35765793	-
FAM13A	O94988	VAR_049019	p.Val769Ile	LB/B	rs7657817	-
FAM13B	Q9NYF5	VAR_049020	p.Met802Val	LB/B	rs33956817	-
FAM13C	Q8NE31	VAR_047661	p.Pro82His	LB/B	rs17853626	-
FAM149A	A5PLN7	VAR_039050	p.Lys332Glu	LB/B	rs4862650	-
FAM149A	A5PLN7	VAR_039051	p.Lys437Glu	LB/B	rs4862653	-
FAM149A	A5PLN7	VAR_039052	p.His505Arg	LB/B	rs2276924	-
FAM149A	A5PLN7	VAR_039053	p.Pro532Leu	LB/B	rs2276922	-
FAM149A	A5PLN7	VAR_039054	p.Arg722Trp	LB/B	rs9991339	-
FAM149A	A5PLN7	VAR_039055	p.Leu748Val	LB/B	rs6818265	-
FAM149B1	Q96BN6	VAR_060167	p.Gly571Arg	LB/B	rs12573841	-
FAM151A	Q8WW52	VAR_037109	p.Asp51Asn	LB/B	rs17399297	-
FAM151A	Q8WW52	VAR_037110	p.Asn87Asp	LB/B	rs17856620	-
FAM151A	Q8WW52	VAR_037111	p.Ile119Thr	LB/B	rs17851843	-
FAM151A	Q8WW52	VAR_037112	p.Ala416Val	LB/B	rs1368883	-
FAM151A	Q8WW52	VAR_037113	p.Gly526Ala	LB/B	rs11206394	-
FAM151A	Q8WW52	VAR_037114	p.Gly546Asp	LB/B	rs2289015	-

FAM151B	Q6UXP7	VAR_053992	p.Ile155Thr	LB/B	rs369998	-
FAM155B	O75949	VAR_043975	p.Leu172Pro	LB/B	rs1171942	-
FAM161A	Q3B820	VAR_042630	p.Ile236Val	LB/B	rs17513722	-
FAM161A	Q3B820	VAR_042631	p.Glu273Lys	LB/B	rs6733774	-
FAM161A	Q3B820	VAR_060180	p.Ile107Met	LB/B	rs11125895	-
FAM161B	Q96MY7	VAR_027963	p.Gly11Ala	LB/B	rs11848954	-
FAM161B	Q96MY7	VAR_027964	p.Leu622Pro	LB/B	rs17094077	-
FAM161B	Q96MY7	VAR_069168	p.Lys487Arg	LB/B	rs28927675	-
FAM162A	Q96A26	VAR_028849	p.Ala50Val	LB/B	rs17850692	-
FAM162B	Q5T6X4	VAR_053782	p.Gln71His	LB/B	rs654128	-
FAM166C	A6NJV1	VAR_042998	p.Gln66His	LB/B	rs13002673	-
FAM166C	A6NJV1	VAR_054056	p.Gln177Leu	LB/B	rs2272466	-
FAM167A	Q96KS9	VAR_062205	p.His56Gln	LB/B	rs3021513	-
FAM167A	Q96KS9	VAR_062206	p.Thr107Ser	LB/B	rs3021512	-
FAM169B	Q8N8A8	VAR_044345	p.Ser115Pro	LB/B	rs12101356	-
FAM170A	A1A519	VAR_047312	p.Pro173Ser	LB/B	rs328694	-
FAM170B	A6NMN3	VAR_037062	p.Arg69Leu	LB/B	rs17773851	-
FAM171A1	Q5VUB5	VAR_030220	p.Pro465Ser	LB/B	rs3814165	-
FAM171B	Q6P995	VAR_032269	p.Val302Ile	LB/B	rs17855085	-
FAM172A	Q8WUF8	VAR_039312	p.Ser131Asn	LB/B	rs17083426	-
FAM172A	Q8WUF8	VAR_080593	p.Glu228Gln	US	rs767629211	-
FAM174B	Q3ZCQ3	VAR_039989	p.Ser116Leu	LB/B	rs34052034	-
FAM177B	A6PVY3	VAR_039953	p.Ile3Ser	LB/B	rs2378607	-
FAM177B	A6PVY3	VAR_054090	p.Gln143Arg	LB/B	rs6683071	-
FAM178B	Q8IXR5	VAR_039172	p.Arg645Cys	LB/B	rs34013660	-
FAM180A	Q6UWF9	VAR_054081	p.Val96Ile	LB/B	rs3112374	-
FAM180B	Q6P0A1	VAR_046379	p.Ala145Thr	LB/B	rs4486587	-
FAM181A	Q8N9Y4	VAR_022834	p.Ala28Thr	LB/B	rs10141024	-
FAM181A	Q8N9Y4	VAR_057822	p.Leu162Met	LB/B	rs34220325	-
FAM181B	A6NEQ2	VAR_039694	p.Val186Leu	LB/B	rs986097	-
FAM181B	A6NEQ2	VAR_039695	p.Arg367Pro	LB/B	rs6592081	-
FAM184A	Q8NB25	VAR_054101	p.Gln174His	LB/B	rs34681930	-
FAM184A	Q8NB25	VAR_054102	p.Val177Gly	LB/B	rs34977570	-
FAM184A	Q8NB25	VAR_054103	p.Asp599Asn	LB/B	rs17827619	-
FAM184B	Q9ULE4	VAR_039202	p.Asn952Ser	LB/B	rs16895365	-
FAM184B	Q9ULE4	VAR_039203	p.Val1042Ala	LB/B	rs6825562	-
FAM185A	Q8N0U4	VAR_039356	p.Gly60Ala	LB/B	rs28695887	-
FAM185A	Q8N0U4	VAR_084592	p.Leu15Phe	LB/B	rs141352868	-
FAM186A	A6NE01	VAR_054419	p.Lys187Gln	LB/B	rs12303082	-
FAM186A	A6NE01	VAR_054420	p.Arg1204Gly	LB/B	rs10876024	-
FAM186A	A6NE01	VAR_054421	p.Leu1233Pro	LB/B	rs10876023	-
FAM186A	A6NE01	VAR_054422	p.His2166Tyr	LB/B	rs7296291	-
FAM186A	A6NE01	VAR_054423	p.Met2193Ile	LB/B	rs6580742	-
FAM186A	A6NE01	VAR_054424	p.His2228Gln	LB/B	rs6580741	-
FAM186A	A6NE01	VAR_054425	p.Gln2316Glu	LB/B	rs12809349	-
FAM186B	Q8IYM0	VAR_027777	p.Val397Met	LB/B	rs17853450	-
FAM186B	Q8IYM0	VAR_027778	p.Glu553Gln	LB/B	rs12299908	-
FAM187B	Q17R55	VAR_031790	p.Pro3Ser	LB/B	rs34873156	-
FAM187B	Q17R55	VAR_031791	p.Cys160Arg	LB/B	rs565791	-

FAM187B Q17R55 VAR_031792 p.Val216Ile LB/B rs564117 -
FAM189A1 O60320 VAR_039544 p.His375Arg LB/B rs2256277 -
FAM189A1 O60320 VAR_039545 p.Arg393His LB/B rs2256273 -
FAM189A1 O60320 VAR_059333 p.Gly228Ser LB/B rs2292628 -
FAM189A1 O60320 VAR_059334 p.Gly314Asp LB/B rs2306933 -
FAM189A1 O60320 VAR_059335 p.Val431Ala LB/B rs2279482 -
FAM189A2 Q15884 VAR_047364 p.Thr233Ile LB/B rs35386391 -
FAM189A2 Q15884 VAR_050823 p.Arg261Lys LB/B rs11138396 -
FAM189B P81408 VAR_035684 p.Ser358Phe US - A breast cancer sample
FAM189B P81408 VAR_037876 p.Arg549His LB/B rs2072648 -
FAM189B P81408 VAR_056842 p.Arg646His LB/B rs2072648 -
FAM193A P78312 VAR_022863 p.Met192Val LB/B rs17164077 -
FAM193A P78312 VAR_056783 p.Ile1115Val LB/B rs17681870 -
FAM193B Q96PV7 VAR_045619 p.Ser816Pro LB/B rs337382 -
FAM205BP Q63HN1 VAR_036833 p.Asn72Lys LB/B rs521552 -
FAM205BP Q63HN1 VAR_036834 p.Asp203Glu LB/B rs524512 -
FAM207A Q9NSI2 VAR_021949 p.Val212Leu LB/B rs3737075 -
FAM209A Q5JX71 VAR_029621 p.Val66Ala LB/B rs707554 -
FAM209A Q5JX71 VAR_029622 p.Met117Lys LB/B rs1054349 -
FAM209A Q5JX71 VAR_029623 p.Arg135Gly LB/B rs1054358 -
FAM209A Q5JX71 VAR_029624 p.Arg146Lys LB/B rs1054361 -
FAM209A Q5JX71 VAR_033761 p.Leu95Phe LB/B rs1054343 -
FAM209B Q5JX69 VAR_033762 p.Glu129Ala LB/B rs2296129 -
FAM209B Q5JX69 VAR_050918 p.Gln74Lys LB/B rs3209183 -
FAM209B Q5JX69 VAR_061633 p.Glu84Lys LB/B rs61440263 -
FAM20A Q96MK3 VAR_059282 p.Asn332Lys LB/B rs2302234 -
FAM20A Q96MK3 VAR_059283 p.Leu530Ser LB/B rs2907373 -
FAM20A Q96MK3 VAR_072170 p.Leu173Arg LP/P - Amelogenesis imperfecta 1G (AI1G) [MIM:204690]
FAM20A Q96MK3 VAR_072171 p.Gly331Asp LP/P rs981673034 Amelogenesis imperfecta 1G (AI1G) [MIM:204690]
FAM20A Q96MK3 VAR_072172 p.Asp403Asn LP/P rs377432171 Amelogenesis imperfecta 1G (AI1G) [MIM:204690]
FAM20C Q8IXL6 VAR_037530 p.Gly379Glu LP/P rs796051852 Raine syndrome (RNS) [MIM:259775]
FAM20C Q8IXL6 VAR_037531 p.Gly379Arg LP/P - Raine syndrome (RNS) [MIM:259775]
FAM20C Q8IXL6 VAR_037532 p.Leu388Arg LP/P rs796051849 Raine syndrome (RNS) [MIM:259775]
FAM20C Q8IXL6 VAR_037533 p.Arg549Trp LP/P rs796051850 Raine syndrome (RNS) [MIM:259775]
FAM20C Q8IXL6 VAR_073660 p.Ile258Asn LP/P - Raine syndrome (RNS) [MIM:259775]
FAM20C Q8IXL6 VAR_073661 p.Thr268Met LP/P rs778899041 Raine syndrome (RNS) [MIM:259775]
FAM20C Q8IXL6 VAR_073662 p.Gly280Arg LP/P rs779708323 Raine syndrome (RNS) [MIM:259775]
FAM20C Q8IXL6 VAR_073663 p.Pro328Ser LP/P rs797044462 Raine syndrome (RNS) [MIM:259775]
FAM20C Q8IXL6 VAR_073664 p.Asp451Asn LP/P - Raine syndrome (RNS) [MIM:259775]
FAM210A Q96ND0 VAR_035686 p.Arg82His US rs116967198 A breast cancer sample
FAM210A Q96ND0 VAR_035687 p.Val140Glu US - A breast cancer sample
FAM210A Q96ND0 VAR_056844 p.Tyr39Asn LB/B rs35493157 -
FAM210A Q96ND0 VAR_060439 p.Glu167Lys LB/B rs2847146 -
FAM210B Q96KR6 VAR_033763 p.Pro126Ser LB/B rs6099115 -
FAM214A Q32MH5 VAR_038258 p.Gln135Arg LB/B rs8036680 -
FAM214A Q32MH5 VAR_038259 p.Val560Ile LB/B rs12915981 -
FAM216A Q8WUB2 VAR_032513 p.Arg225Gly LB/B rs17188964 -

FAM216B Q8N7L0 VAR_033743 p.Arg134Cys LB/B rs35889214 -
FAM217A Q8IXS0 VAR_023207 p.Val431Ile LB/B rs595413 -
FAM217A Q8IXS0 VAR_056801 p.Ala258Thr LB/B rs639905 -
FAM217A Q8IXS0 VAR_056802 p.Gln309Pro LB/B rs10080405 -
FAM217A Q8IXS0 VAR_056803 p.Cys360Ser LB/B rs17137618 -
FAM217A Q8IXS0 VAR_056804 p.Met442Val LB/B rs10485172 -
FAM217B Q9NTX9 VAR_035691 p.Ser95Gly US rs1180582304 A breast cancer sample
FAM217B Q9NTX9 VAR_050922 p.Tyr380Cys LB/B rs6027210 -
FAM218A Q96MZ4 VAR_044025 p.His54Arg LB/B rs3733418 -
FAM220A Q7Z4H9 VAR_039375 p.Gly71Arg LB/B rs3750041 -
FAM220A Q7Z4H9 VAR_039376 p.Arg127Gln LB/B rs3750040 -
FAM220A Q7Z4H9 VAR_039377 p.Val161Leu LB/B rs3750039 -
FAM220A Q7Z4H9 VAR_039378 p.Val198Met LB/B rs6952125 -
FAM220A Q7Z4H9 VAR_039379 p.Gly237Ser LB/B rs2241445 -
FAM220BP B1ANY3 VAR_050820 p.Trp162Arg LB/B rs1832322 -
FAM220BP B1ANY3 VAR_050821 p.Lys199Asn LB/B rs1832323 -
FAM220BP B1ANY3 VAR_050822 p.Lys203Asn LB/B rs12336220 -
FAM221A A4D161 VAR_033215 p.Tyr20His LB/B rs17855785 -
FAM221A A4D161 VAR_033216 p.Ala90Thr LB/B rs34518648 -
FAM221A A4D161 VAR_033217 p.Cys95Arg LB/B rs35495590 -
FAM221A A4D161 VAR_033218 p.His128Arg LB/B rs17855786 -
FAM221A A4D161 VAR_033219 p.Ser240Gly LB/B rs35928055 -
FAM221B A6H8Z2 VAR_061598 p.His34Arg LB/B rs13294256 -
FAM221B A6H8Z2 VAR_061599 p.Lys41Glu LB/B rs13294245 -
FAM222B Q8WU58 VAR_050900 p.Leu471Ser LB/B rs2043031 -
FAM222B Q8WU58 VAR_050901 p.Gly542Ser LB/B rs36029715 -
FAM228A Q86W67 VAR_046178 p.Tyr140Cys LB/B rs2288073 -
FAM241A Q8N8J7 VAR_032150 p.Pro46Gln LB/B rs17852081 -
FAM24B Q8N5W8 VAR_038485 p.Pro2Leu LB/B rs1891110 -
FAM25A B3EWG3 VAR_054062 p.Val35Leu LB/B rs7079587 -
FAM3A P98173 VAR_011923 p.Trp213Cys LB/B rs1050788 -
FAM3A P98173 VAR_057531 p.Ile21Met LB/B rs35985994 -
FAM3B P58499 VAR_021953 p.Val14Met LB/B rs2838012 -
FAM3D Q96BQ1 VAR_053087 p.Pro60Ser LB/B rs17059569 -
FAM3D Q96BQ1 VAR_053088 p.Ala118Ser LB/B rs33966924 -
FAM47A Q5JRC9 VAR_054412 p.Phe50Leu LB/B rs17856750 -
FAM47A Q5JRC9 VAR_054413 p.Val232Glu LB/B rs17856751 -
FAM47A Q5JRC9 VAR_054414 p.Ala377Ser LB/B rs1811716 -
FAM47A Q5JRC9 VAR_054416 p.Gly531Arg LB/B rs5971820 -
FAM47C Q5HY64 VAR_046358 p.Asn924Thr LB/B rs1995914 -
FAM50A Q14320 VAR_084563 p.Ala137Val LB/B rs149558328 -
FAM50A Q14320 VAR_084564 p.Glu143Lys LB/B rs782017549 -
FAM50A Q14320 VAR_084565 p.Trp206Gly US - Intellectual developmental disorder, X-linked, syndromic, Armfield type (MRXSA) [MIM:300261]
FAM50A Q14320 VAR_084566 p.Glu254Gly LP/P - Intellectual developmental disorder, X-linked, syndromic, Armfield type (MRXSA) [MIM:300261]
FAM50A Q14320 VAR_084567 p.Asp255Gly LP/P - Intellectual developmental disorder, X-linked, syndromic, Armfield type (MRXSA) [MIM:300261]

FAM50A Q14320 VAR_084568 p.Asp255Asn LP/P - Intellectual developmental disorder, X-linked, syndromic, Armfield type (MRXSA) [MIM:300261]
FAM50A Q14320 VAR_084569 p.Arg273Trp LP/P - Intellectual developmental disorder, X-linked, syndromic, Armfield type (MRXSA) [MIM:300261]
FAM53C Q9NYF3 VAR_053089 p.Arg21Cys LB/B rs35360938 -
FAM71A Q8IYT1 VAR_029464 p.Asn22Asp LB/B rs17853363 -
FAM71A Q8IYT1 VAR_029465 p.Asn253Ser LB/B rs3122712 -
FAM71A Q8IYT1 VAR_029466 p.Gly319Asp LB/B rs17853362 -
FAM71A Q8IYT1 VAR_029467 p.Asn551Asp LB/B rs3122713 -
FAM71A Q8IYT1 VAR_029468 p.Thr577Met LB/B rs3795842 -
FAM71B Q8TC56 VAR_032036 p.Ala543Val LB/B rs17852327 -
FAM71B Q8TC56 VAR_032037 p.Met564Thr LB/B rs31208 -
FAM71B Q8TC56 VAR_032038 p.Val599Ile LB/B rs2115480 -
FAM71C Q8NEG0 VAR_029638 p.Arg30Gly LB/B rs11109968 -
FAM71C Q8NEG0 VAR_029639 p.Met71Val LB/B rs11109969 -
FAM71E1 Q6IPT2 VAR_043465 p.Ser206Ile LB/B rs736769 -
FAM71F1 Q96KD3 VAR_037271 p.Ser228Leu LB/B rs6949056 -
FAM71F1 Q96KD3 VAR_037272 p.Glu242Lys LB/B rs6971091 -
FAM71F2 Q6NXP2 VAR_037267 p.Pro112Thr LB/B rs17169357 -
FAM71F2 Q6NXP2 VAR_037268 p.Asp114Val LB/B rs6971819 -
FAM71F2 Q6NXP2 VAR_037269 p.Arg136Trp LB/B rs6467210 -
FAM71F2 Q6NXP2 VAR_037270 p.Ile251Val LB/B rs1109552 -
FAM72B Q86X60 VAR_044011 p.Gly82Val LB/B rs17838150 -
FAM72B Q86X60 VAR_044012 p.Pro94Leu LB/B rs1572701 -
FAM74A7 A6NL05 VAR_045629 p.Asp12Tyr LB/B rs11793234 -
FAM74A7 A6NL05 VAR_045630 p.Arg72Lys LB/B rs2261191 -
FAM74A7 A6NL05 VAR_045631 p.Arg86Thr LB/B rs6423979 -
FAM81B Q96LP2 VAR_057968 p.Arg150Gly LB/B rs10042271 -
FAM81B Q96LP2 VAR_057969 p.Ala187Val LB/B rs11555275 -
FAM81B Q96LP2 VAR_057970 p.Arg239Gln LB/B rs1541797 -
FAM81B Q96LP2 VAR_057971 p.Ser275Pro LB/B rs6878669 -
FAM83A Q86UY5 VAR_032178 p.Ala237Thr LB/B rs7813708 -
FAM83B Q5T0W9 VAR_034638 p.Asn410Ser LB/B rs13211183 -
FAM83B Q5T0W9 VAR_034639 p.Ser435Arg LB/B rs9475076 -
FAM83B Q5T0W9 VAR_034640 p.Lys640Thr LB/B rs239798 -
FAM83B Q5T0W9 VAR_034641 p.Thr907Asn LB/B rs9475077 -
FAM83C Q9BQN1 VAR_021946 p.Arg621Gln LB/B rs2425049 -
FAM83C Q9BQN1 VAR_053900 p.Glu134Lys LB/B rs35162625 -
FAM83C Q9BQN1 VAR_053901 p.Tyr600His LB/B rs35560631 -
FAM83C Q9BQN1 VAR_053902 p.Arg645Cys LB/B rs35518957 -
FAM83E Q2M2I3 VAR_038130 p.Thr91Ala LB/B rs447802 -
FAM83E Q2M2I3 VAR_038131 p.Pro311Leu LB/B rs3745728 -
FAM83E Q2M2I3 VAR_038132 p.Arg372His LB/B rs3745727 -
FAM83F Q8NEG4 VAR_038134 p.Arg245Ser LB/B rs12330063 -
FAM83F Q8NEG4 VAR_038135 p.Gly353Ser LB/B rs35823589 -
FAM83F Q8NEG4 VAR_038136 p.Ala418Val US - A breast cancer sample
FAM83F Q8NEG4 VAR_038137 p.Arg436Gly LB/B rs5995794 -
FAM83G A6ND36 VAR_053903 p.Ile109Thr LB/B rs2074283 -
FAM83G A6ND36 VAR_075713 p.Leu64Phe US rs371100508 -

FAM83G A6ND36 VAR_075714 p.Arg630Trp US rs201046878 -
FAM83H Q6ZRV2 VAR_062189 p.Gln201His LB/B rs9969600 -
FAM83H Q6ZRV2 VAR_073954 p.Gly557Cys LP/P rs312262803 Amelogenesis imperfecta 3A (AI3A) [MIM:130900]
FAM86B2 P0C5J1 VAR_036617 p.Asp43Tyr LB/B rs2684093 -
FAM86B2 P0C5J1 VAR_036618 p.Arg285Ser LB/B rs7817085 -
FAM86C1P Q9NVL1 VAR_036619 p.Ala7Ser LB/B rs12283300 -
FAM86C1P Q9NVL1 VAR_036620 p.Arg30Pro LB/B rs12283346 -
FAM86C1P Q9NVL1 VAR_036621 p.Val70Ala LB/B rs3935309 -
FAM86C1P Q9NVL1 VAR_062211 p.Pro135Leu LB/B rs57679800 -
FAM90A1 Q86YD7 VAR_057778 p.Ala234Glu LB/B rs9668582 -
FAM90A1 Q86YD7 VAR_060154 p.Arg343Ser LB/B rs9668475 -
FAM90A1 Q86YD7 VAR_060155 p.Thr410Ala LB/B rs11044098 -
FAM90A1 Q86YD7 VAR_060293 p.Arg123Gly LB/B rs9738115 -
FAM90A1 Q86YD7 VAR_060294 p.Pro334Leu LB/B rs17855656 -
[truncated: 493,784 more chars]
